# Macrophage–Derived Ferritin Exacerbates Silica‐Induced Pulmonary Fibrosis via PIK3R2‐Mediated Fibroblast Differentiation

**DOI:** 10.1002/advs.202519191

**Published:** 2026-01-21

**Authors:** Liqun Wang, Xuxi Chen, Hongying Quan, Rui Qian, Shuyu Gong, Qiurong He, Ying Gao, Ajia Axi, Manyu Zhao, Qin Zhang, Ling Zhang, Lijun Peng, Xin Sun, Ben Zhang, Yuqin Yao

**Affiliations:** ^1^ West China School of Public Health and West China Fourth Hospital Sichuan University Chengdu China; ^2^ Department of Respiratory and Critical Care Medicine, West China Hospital Sichuan University Chengdu China; ^3^ West China Occupational Pneumoconiosis Cohort Study (WCOPCS) working group, Research Center for Prevention and Therapy of Occupational Disease, West China School of Public Health and West China Fourth Hospital Sichuan University Chengdu China

**Keywords:** ferritin, fibroblast, macrophages, phosphoinositide‐3‐kinase regulatory subunit 2, silicosis

## Abstract

Silicosis is a progressive and life‐threatening fibrotic lung disease caused by crystalline silica. However, targeted therapies remain unavailable due to its incompletely understood pathogenic mechanisms. Here, we identify ferritin as a pivotal mediator of silica‐induced pulmonary fibrosis by integrating clinical exploration with experimental validation. We detected persistently elevated ferritin levels in lung tissues and serum from silicosis patients and silica‐exposed mice, and demonstrated that exogenous ferritin administration exacerbates fibrosis in vivo. Multi‐omics profiling and co‐culture experiments revealed that macrophage–secreted ferritin promotes fibroblast‐to‐myofibroblast differentiation and pathological extracellular matrix (ECM) deposition via the PIK3R2/SMAD signaling axis. Importantly, genetic knockdown of ferritin in macrophages significantly suppressed myofibroblast differentiation and collagen accumulation both in vivo and in vitro. These findings underscore that ferritin functions not only as a potential clinical biomarker for silicosis surveillance but also as a pathogenic driver through macrophage‐fibroblast crosstalk, and provide a theoretical foundation for developing integrated diagnostic and therapeutic strategies against silicosis.

## Graphical abstract

This study identifies ferritin as a pivotal mediator of silica‐induced pulmonary fibrosis. Macrophage‐derived ferritin drives fibroblast‐to‐myofibroblast differentiation via the PIK3R2/SMAD pathway, while ferritin knockdown alleviates fibrosis. These findings define ferritin as both a biomarker and pathogenic driver, highlighting ferritin‐PIK3R2 signaling axis as a promising cross‐cellular therapeutic target for silicosis.

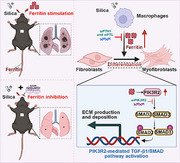

## 1 Introduction

Silica is the most abundant mineral in the Earth's crust and is extensively utilized in industrial applications. However, the free crystalline silica dust generated during its processing poses a serious occupational health risk, and silicosis caused by silica exposure has emerged as a global public health crisis [1]. Currently, tens of millions of workers remain occupationally exposed to hazardous silica concentrations worldwide, and the number of silicosis cases is expected to rise [2]. Indeed, a resurgence of silicosis has been reported in the United States, Australia, and Spain [3]. Silicosis is characterized by progressive lung injury and irreversible fibrosis, and the disease can continue to progress even after cessation of exposure. Clinically, silicosis still faces two major challenges about delayed diagnosis and limited therapeutic options. At present, only tetrandrine and Nintedanib are approved for anti‐fibrotic treatments [4, 5]. However, advanced fibrosis remains largely irreversible, resulting in high disability and mortality rates [6]. Fundamentally, the poorly defined molecular pathogenesis of silicosis hampers the discovery of early diagnostic biomarkers and mechanism‐based anti‐fibrotic drugs. Therefore, elucidating the molecular mechanisms underlying silica‐induced pulmonary fibrosis is essential for improving diagnosis, monitoring disease progression, and developing precision therapeutics for silicosis.

Progressive pulmonary fibrosis caused by silica particle deposition in lung tissue drives a gradual decline in lung function. Silica particles that enter the lung parenchyma trigger innate immune responses through mechanical damage or injury induced by their surface properties [7]. Macrophages then phagocytose silica particles and release diverse signaling molecules to regulate tissue repair [3]. However, owing to the non‐biodegradable nature of silica, macrophages cannot effectively degrade these silica particles, resulting in macrophage dysfunction and death, accompanied by excessive release of pro‐inflammatory and pro‐fibrotic cytokines [8]. This pathological cycle of phagocytosis, cell death, and silica release disrupts pulmonary immune homeostasis and impairs repair processes. In this context, cytokines secreted by activated macrophages and other immune cells persistently stimulate lung fibroblasts, driving their proliferation and differentiation into myofibroblasts, which leads to fibrotic scar formation and excessive extracellular matrix (ECM) deposition, ultimately resulting in irreversible pulmonary fibrosis [9]. Thus, silicosis arises from abnormal cellular functions and complex intercellular interactions under persistent silica exposure. Among these, the sustained activation and injury of macrophages in the early phase, together with the subsequent excessive activation and differentiation of fibroblasts, constitute the central cellular axis driving fibrotic progression. Therefore, elucidating macrophage‐fibroblast crosstalk and their underlying molecular interactions is critical for identifying early risk prediction biomarkers and developing effective targeted therapies for advanced disease.

Ferritin is encoded by two independent genes, the ferritin heavy chain 1 (*FTH1*) and ferritin light chain 1 (*FTL*). After transcription and translation, FTH1 and FTL polypeptides self‐assemble to form ferritin under physiological conditions [10]. Traditionally, ferritin is known for its essential role in maintaining iron homeostasis [11]. However, emerging evidence indicates that ferritin is highly expressed in inflammatory, tumor, and fibrotic diseases, in both iron‐dependent and iron‐independent contexts, and is often associated with poor disease prognosis. Clinically, ferritin has been used as a biomarker for the diagnosis and prognosis of malignant tumors and metabolic dysfunction‐associated fatty liver disease [12, 13]. More recently, ferritin has been shown to aggravate systemic inflammation and activate hepatic stellate cells [14, 15]. These findings emphasize that ferritin not only plays a surface role as a biomarker but may also act as a pathogenic molecule actively involved in disease progression. Previously, elevated ferritin levels have been observed in the serum and bronchoalveolar lavage fluid (BALF) of dust‐exposed workers [16, 17]. Nevertheless, whether aberrant ferritin expression contributes to silicosis pathogenesis remains unclear. Investigating the toxicological effects and molecular mechanisms of elevated ferritin in silicosis may provide critical insights for both diagnosis and therapy. Moreover, owing to its iron‐loading capacity, ferritin has been proposed as a potential agent for imaging‐based diagnosis and iron‐targeted therapies [18], as well as a promising nanocarrier platform due to its unique hollow spherical structure and favorable biocompatibility [19]. Hence, evaluating the pathophysiological role of ferritin is essential for guiding the safe and effective translation of ferritin‐based applications, thereby minimizing potential risks associated with its use in biomedical contexts.

Here, we show that ferritin expression increases with disease severity both in patients with silicosis and in silica‐exposed mice. Mechanistically, macrophage‐derived ferritin exacerbates fibrosis by activating the Phosphoinositide‐3‐kinase regulatory subunit 2 (PIK3R2)/SMADs pathway in fibroblasts. Selective knockdown of ferritin in macrophages markedly attenuated fibroblast differentiation and extracellular matrix (ECM) deposition in vivo and in vitro, underscoring the critical role of ferritin in silicosis pathogenesis. Our findings uncover a novel ferritin–PIK3R2 axis orchestrating macrophage–fibroblast crosstalk, highlight ferritin as a measurable biomarker for silicosis monitoring and early diagnosis, and identify PIK3R2 as a viable therapeutic target. This dual framework connects mechanistic insight with translational potential, offering new avenues for precision medicine in silicosis.

## 2 Results

### 2.1 Elevated Ferritin Expression in Silicosis Patients and Silica‐Induced Pulmonary Fibrosis Mice

**FIGURE 1:**
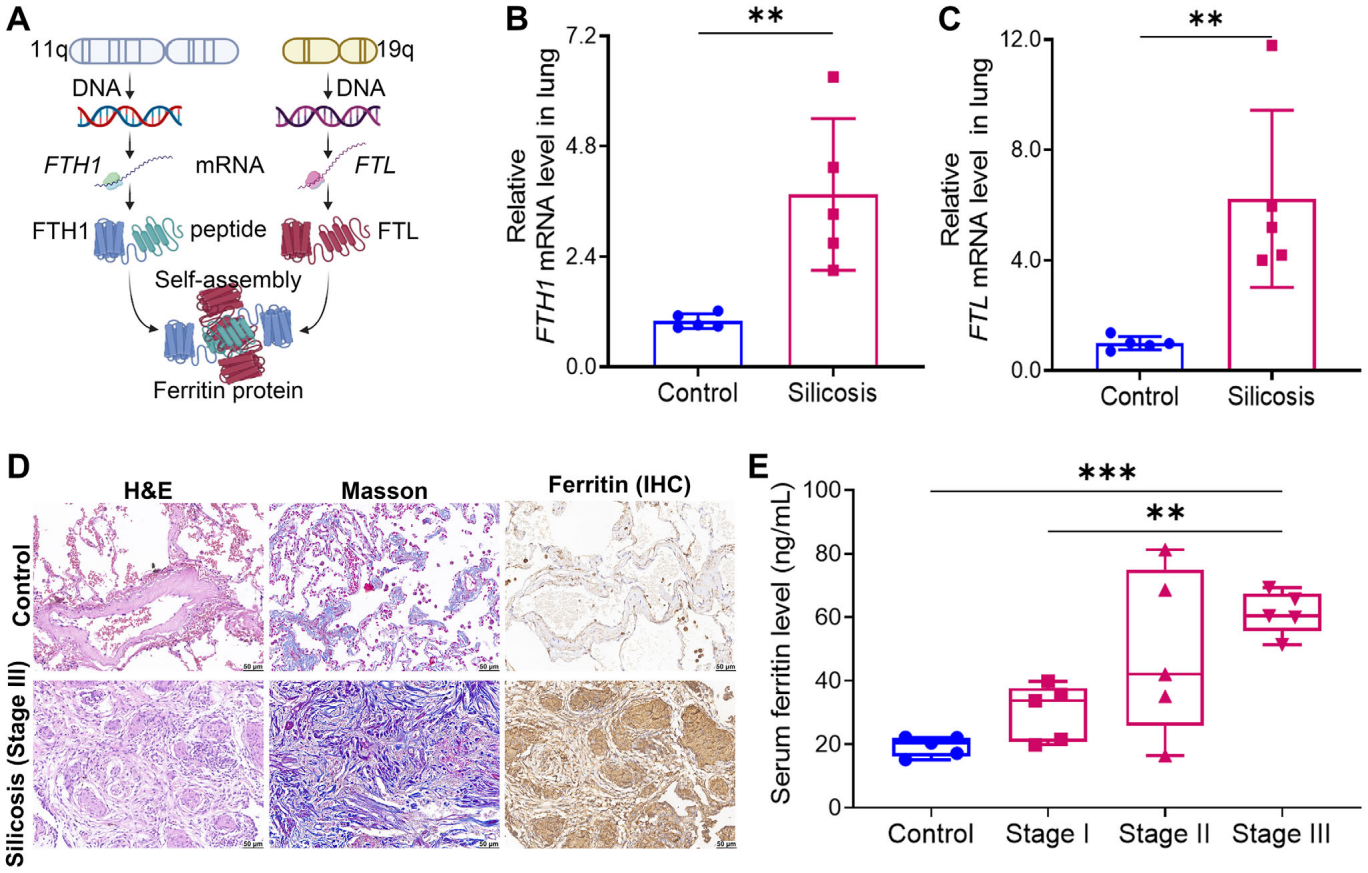
Elevated ferritin expression in lung tissue and serum of patients with silicosis. A) Schematic of ferritin biosynthesis: ferritin is encoded by two independent genes, *FTH1* and *FTL*. After transcription and translation, they self‐assemble into complete ferritin (Created in BioRender.com, with permission). B) *FTH1* mRNA expression levels in lung tissue from patients with stage III silicosis (n = 5 per group). C) *FTL* mRNA expression levels in lung tissue from patients with stage III silicosis (n = 5 per group). D) Representative H&E, Masson's trichrome staining, and ferritin immunohistochemical staining in patients with stage III silicosis (magnification, 20×). E) Serum ferritin levels measured by ELISA in patients with stage I, II, and III silicosis (n = 5 per group). Data are presented as mean ± SD, ^**^ *p* < 0.01, ^***^ *p* < 0.001. Statistical analysis was performed using two‐tailed Student's *t*‐test (B and C), and one‐way ANOVA followed by Dunnett's T3 test (E).

To investigate ferritin expression in lung tissue following silica exposure, we collected lung nodule samples from patients with stage III silicosis. Ferritin is encoded by two independent genes, *FTH1* and *FTL* (Figure 1A). At the transcriptional level, the mRNA levels of *FTH1* and *FTL* were significantly elevated in lung tissues from silicosis patients compared to controls (Figure 1B, C). We then performed immunohistochemical staining to evaluate ferritin protein expression and distribution in the lung tissues. H&E and Masson's trichrome staining revealed severe fibrosis and collagen deposition in the lung tissue of patients with stage III silicosis (Figure 1D). In control lungs, ferritin was primarily localized to alveolar interstitial cells, with only minor expression in vascular endothelium and alveolar epithelial cells. In contrast, lungs from silicosis patients displayed diffuse ferritin distribution throughout the parenchyma, with prominent accumulation within fibrotic lesions. To evaluate the relationship between ferritin levels and disease progression, we measured serum ferritin levels in patients with stage I, II, and III silicosis. ELISA results showed a progressive increase in serum ferritin levels across silicosis stages (Figure 1E), suggesting that ferritin may serve as a potential biomarker associated with silicosis severity.

**FIGURE 2:**
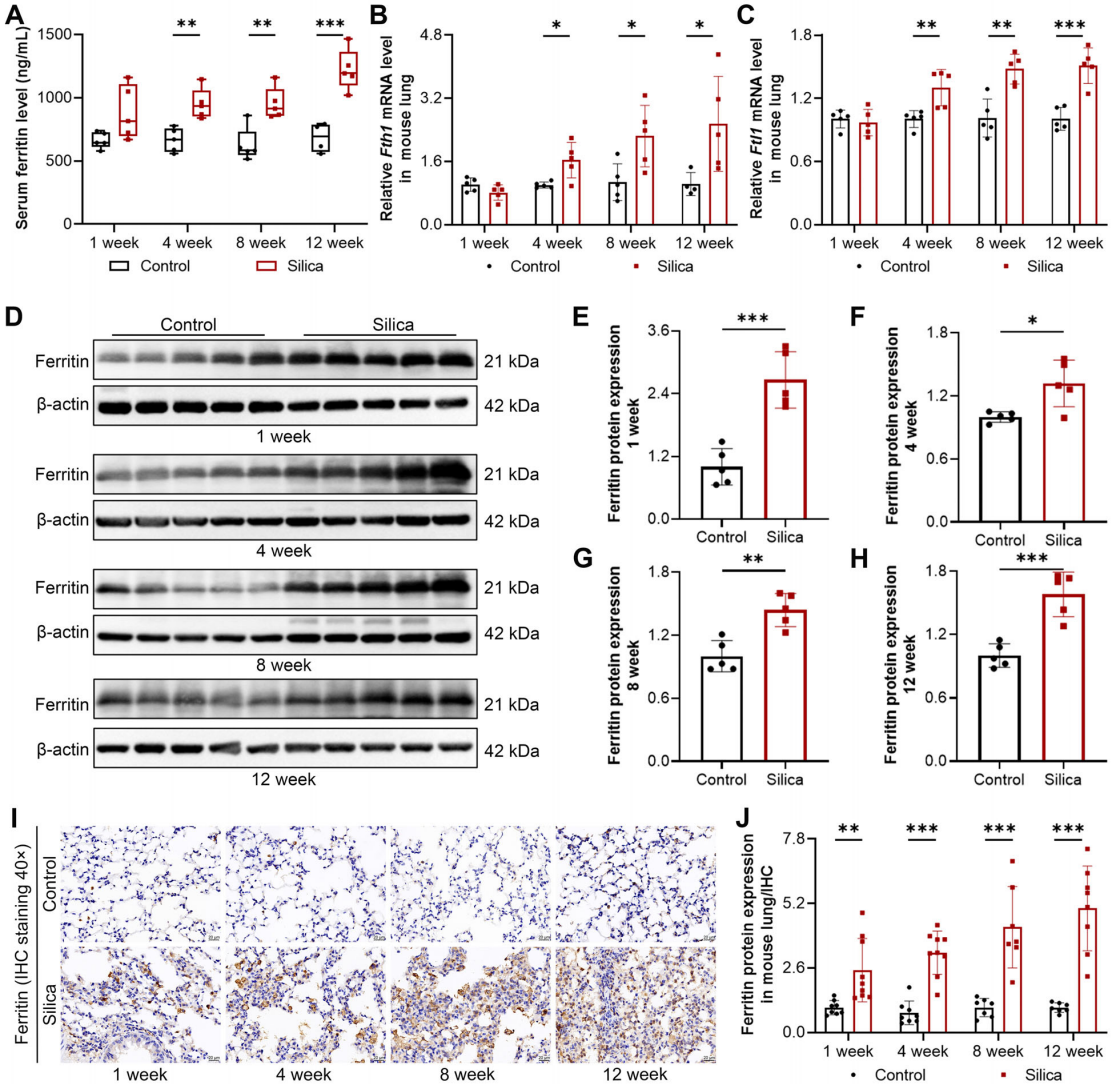
Elevated ferritin expression in the serum and lung tissue of silica‐exposed mice. A) Serum ferritin levels measured by ELISA after 1, 4, 8, and 12 weeks of silica exposure (n = 5 per group). B, C) *Fth1* and *Ftl1* mRNA expression levels in lung tissue were determined by RT‐qPCR after exposure to silica for different durations (n = 5 per group). D–H) Ferritin protein expression levels in lung tissues were assessed by Western blot after exposure to silica for different durations (n = 5 per group). I) Representative immunohistochemical staining of ferritin in lung tissue after exposure to silica for different durations (magnification, 40×). J) Quantitative analysis of ferritin expression in mouse lung tissue based on immunohistochemical staining (n = 9 per group). Data are presented as mean ± SD, ^*^ *p* < 0.05, ^**^ *p* < 0.01, ^***^ *p* < 0.001. Statistical analysis was performed using a two‐tailed Student's *t*‐test (A–C, E–H, J).

To determine whether aberrant ferritin expression is a consequence of silica exposure, we established a silica‐induced mouse model. In response to silica injury, inflammatory cell infiltration, fibroblast proliferation, and ECM secretion contributed to an increase in lung mass. Compared to the controls, the lung weight coefficient was significantly higher in silica‐treated mice (Figure S1A). Histological analysis revealed that lung lesion severity and collagen deposition progressively increased with prolonged silica exposure (Figure S1B). Concurrently, protein levels of ECM components, including Collagen I, Collagen III, and fibronectin, were upregulated (Figure S1C–F), suggesting that continuous silica exposure induced progressive fibrotic‐like lesions in the mouse lung. Subsequently, serum and lung tissues were used to measure ferritin expression. Consistent with human data, serum ferritin levels were significantly increased after 1, 4, 8, and 12 weeks of silica exposure (Figure 2A). In lung tissue, silica challenge progressively upregulated *Fth1* and *Ftl1* mRNA levels (Figure 2B, C), along with elevated ferritin protein levels (Figure 2D–H). Immunohistochemistry staining revealed that the expression of ferritin was mainly confined within alveolar interstitial cells after 1 and 4 weeks of silica exposure (Figure 2I). With exposure time extension, ferritin‐expressing cells released ferritin into the lung interstitium, and overall ferritin expression was obviously increased in silica‐induced mice (Figure 2J). In addition, ferritin expression was examined in major organs of silica‐exposed mice. Immunohistochemical staining revealed that prolonged silica exposure did not induce detectable changes in ferritin levels in the heart, liver, spleen, and kidney (Figure S2), indicating that the elevated ferritin in the circulation system primarily originated from the injured lung tissue. Collectively, ferritin expression was markedly increased in the lung and was released into the bloodstream, and serum ferritin levels increased alongside disease severity. These results suggest that ferritin may contribute to the progression of silicosis and may serve as a potential biomarker for disease assessment.

### 2.2 Exogenous Ferritin Treatment Exacerbates Silica‐Induced Pulmonary Fibrosis in Mice

**FIGURE 3:**
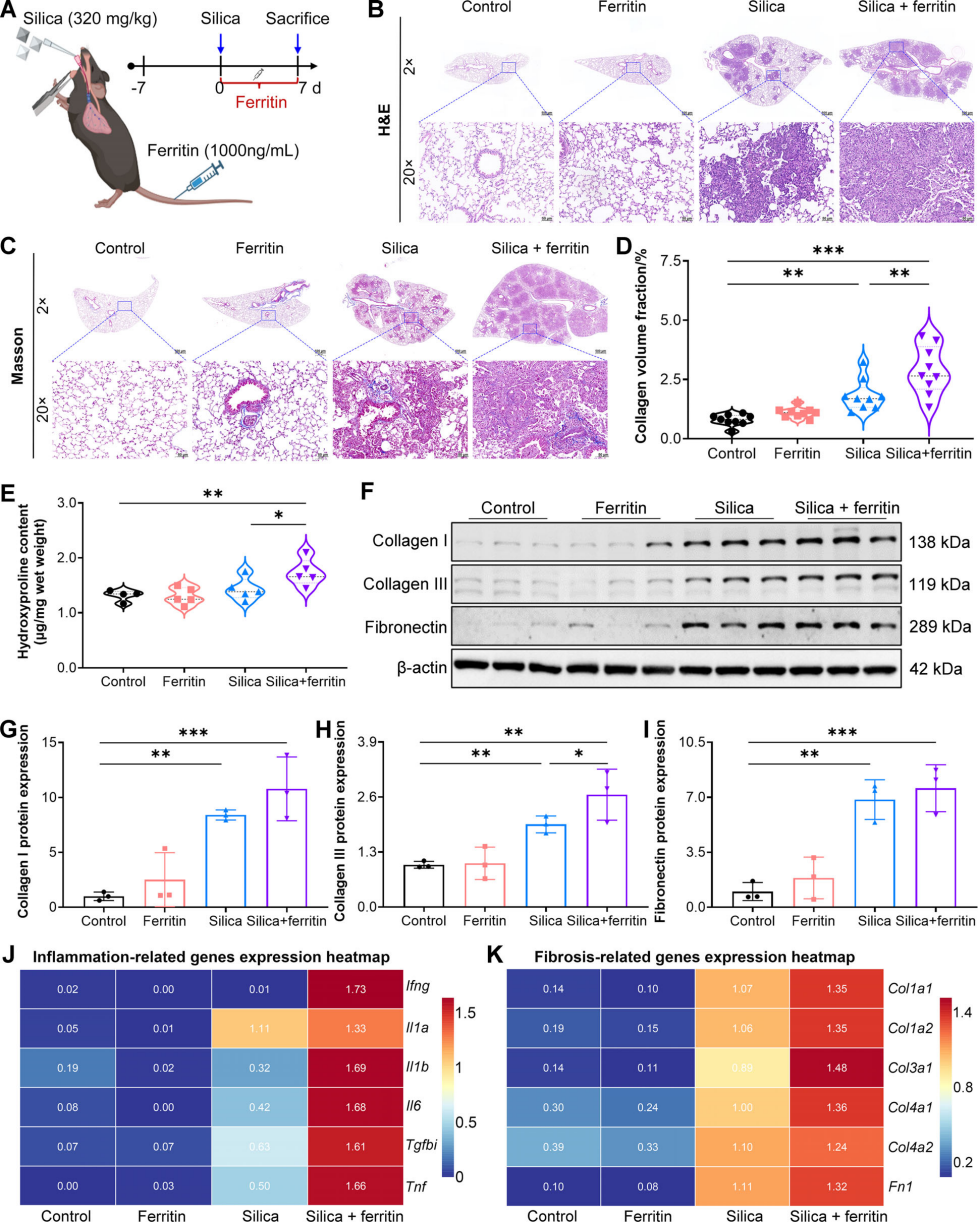
Exogenous ferritin aggravates silica‐induced pulmonary fibrosis in mice. A) Schematic of in vivo study design. Mice were given an intratracheal infusion of silica, followed by daily intravenous ferritin for 7 days. B) Representative H&E staining of lung tissue from mice co‐exposed to silica and ferritin (magnification, 2×, 20×). C) Representative Masson's trichrome staining of lung tissue from co‐exposed mice (magnification, 2×, 20×). D) Quantification of collagen volume fraction based on Masson's trichrome staining (n = 9 per group). E) Hydroxyproline content in lung tissues from silica and ferritin co‐exposed mice (n = 4 or 5 per group). F) Protein expression of Collagen I, Collagen III, and fibronectin detected by Western blot. G–I) Quantitative analysis of Collagen I, Collagen III, and fibronectin protein levels (n = 3 per group). J, K) Heatmaps of inflammation‐ and fibrosis‐related genes in lung tissue from silica and ferritin co‐exposed mice. Data are presented as mean ± SD, ^*^ *p* < 0.05, ^**^ *p* < 0.01, ^***^ *p* < 0.001. Statistical analysis was performed using one‐way ANOVA followed by Dunnett's test (G, I) and LSD (D, E, H).

According to the immunohistochemistry staining results of silicosis patients and silica‐exposed mice (Figures 1D and 2I), ferritin was highly expressed and subsequently released into the pulmonary interstitium, generating a high‐ferritin load microenvironment for pulmonary cells, which may contribute to the progression of advanced silicosis. To address this hypothesis, we constructed a combined‐exposure mouse model involving tracheal instillation of silica together with tail vein injection of ferritin, using the mean serum ferritin concentration in silica‐exposed mice as the exposure dose reference (Figure 3A). Blood biochemical analyses revealed that systemic ferritin administration did not impair liver or kidney function in healthy mice (Figure S3A, B). Consistently, no overt histopathological abnormalities were observed in major organs, including the heart, liver, spleen, and kidney (Figure S3C). These findings indicate that high ferritin loading does not cause detectable organ toxicity under normal physiological conditions.

However, co‐exposure to silica and ferritin significantly exacerbated pulmonary fibrosis compared with silica exposure alone, as evidenced by expanded fibrotic areas (Figure 3B). Masson's trichrome staining further revealed increased collagen deposition and a higher collagen volume fraction in the co‐exposed group (Figure 3C, D). Hydroxyproline content serves as a key biochemical indicator of collagen accumulation. Compared with silica exposure alone, co‐exposure to silica and ferritin resulted in a marked elevated in hydroxyproline levels(Figure 3E), and Western blot analysis determined that ECM components, including Collagen I, Collagen III, and fibronectin, were further upregulated upon additional ferritin stimulation (Figure 3F–I). Moreover, we utilized RNA sequencing to analyze the underlying transcriptional changes of lung tissue after silica and ferritin co‐treatment. Relative to silica exposed alone, combined exposure substantially increased the expressions of inflammation‐related genes (*Ifng*, *Il1a*, *Il1b*, *Il6*, *Tgfbi*, *Tnf*) and fibrosis‐related genes (*Col1a1*, *Col1a2, Col3a1*, *Col4a1*, *Col4a2, Fn1*) in lung tissue (Figure 3J, K). Collectively, these results provide compelling evidence that ferritin exacerbates fibrosis in silica‐induced pulmonary fibrosis.

Ferritin is an important iron‐regulatory protein, and ferroptosis driven by excessive iron deposition plays a critical role in the initiation and progression of fibrotic diseases [20]. To investigate whether silica‐induced ferritin upregulation and its pro‐fibrotic role were iron‐dependent, we assessed iron levels in lung tissue. Prussian blue staining revealed that, despite severe fibrotic lesions forming after 12 weeks of silica exposure, no distinct blue deposits indicative of iron accumulation were observed in lung tissue, even within fibrotic regions (Figure S4A). Consistently, total iron levels in lung homogenates were comparable to those of controls (Figure S4C). Similarly, combined silica and ferritin exposure did not induce evident iron deposition or alter total iron levels (Figure S4B, D). Together, these results demonstrate that silica‐induced ferritin upregulation and its fibrosis‐aggravating effects occur independently of iron concentration, suggesting that ferritin itself may function as a direct regulatory mediator of fibrosis progression.

### 2.3 Exogenous Ferritin Facilitates Fibroblast‐to‐Myofibroblast Differentiation

**FIGURE 4:**
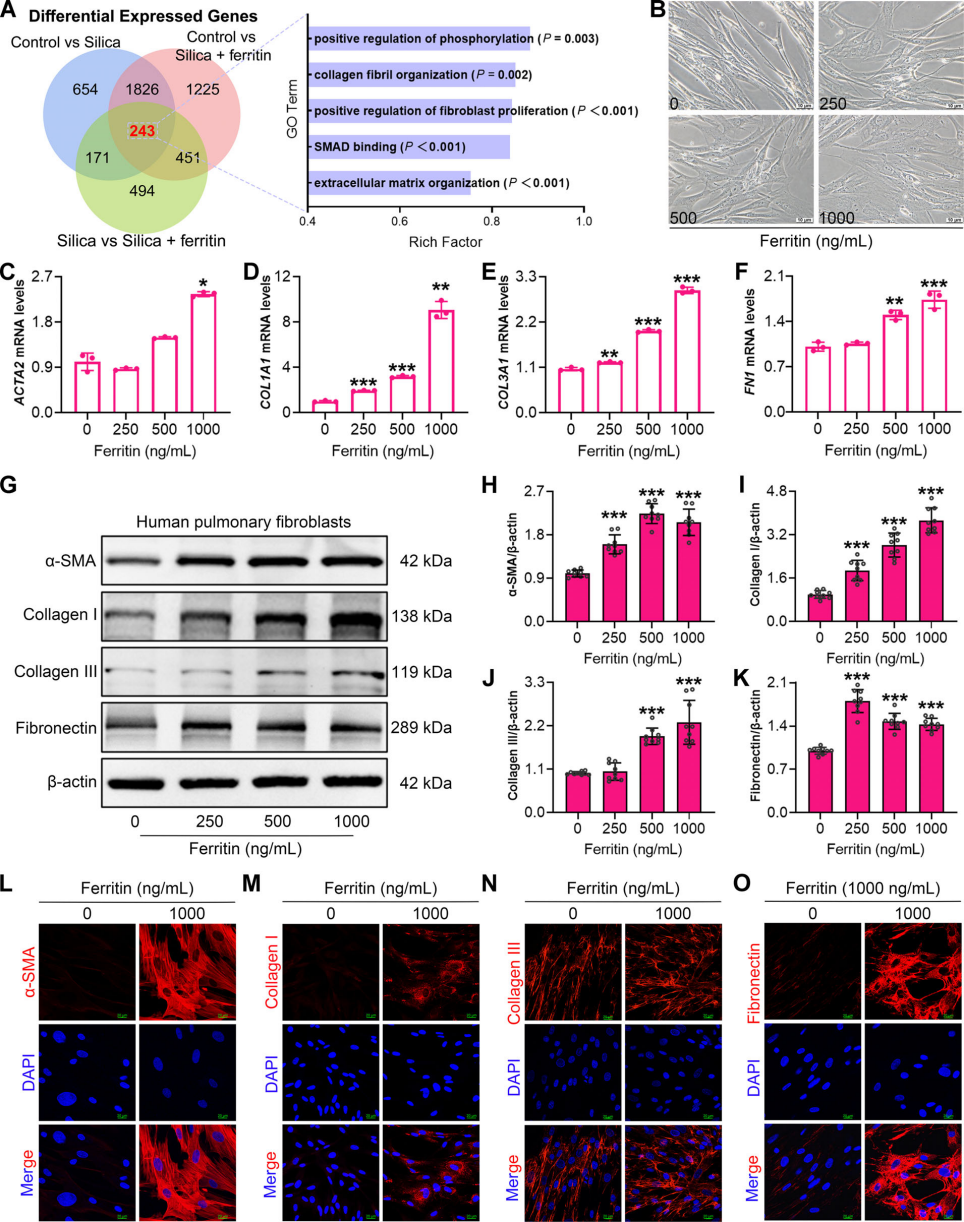
Exogenous ferritin stimulation promotes fibroblast differentiation into a profibrotic phenotype. A) Transcriptomic analysis of lung tissue from silica and ferritin co‐exposed mice. B) Morphological changes in HPFs after 0, 250, 500, 1000 ng/mL ferritin stimulation (magnification, 20×). C‐F) mRNA expression levels of *ACTA2*, *COL1A1*, *COL3A1*, and *FN1* in HPFs treated with increasing concentrations of ferritin (n = 3 per group). G) Protein expression levels of α‐SMA, Collagen I, Collagen III, and fibronectin in HPFs evaluated by Western blot. H–K) Quantitative analysis of α‐SMA, Collagen I, Collagen III, and fibronectin protein expression in HPFs (n = 3 per group). L‐O) Immunofluorescence staining of α‐SMA, Collagen I, Collagen III, and fibronectin in HPFs treated with 0 or 1000 ng/mL ferritin (magnification, 60×). Data are presented as mean ± SD, ^*^ *p* < 0.05, ^**^ *p* < 0.01, ^***^ *p* < 0.001. Statistical analysis was performed using a hypergeometric test (A) and one‐way ANOVA followed by Dunnett's test (E, F, H, K) and Dunnett's T3 test (C, D, I, J).

The above findings establish ferritin as an iron‐independent pro‐fibrotic factor in silicosis. To elucidate the mechanisms by which ferritin accelerates fibrosis under silica exposure, we performed transcriptomic profiling of lung tissues from mice co‐exposed to silica and ferritin. Pairwise comparisons among the control, silica‐exposed alone, and silica plus ferritin co‐exposure group identified 243 overlapping differentially expressed genes (DEGs) (Figure 4A). Gene Ontology (GO) enrichment analysis of the DEGs indicated that ferritin predominantly affected biological processes associated with fibroblast proliferation and migration, SMAD binding and phosphorylation, and extracellular matrix organization. Moreover, spatial transcriptomic analysis further showed that the ferritin‐encoding gene *Fth1* was co‐localized with the myofibroblast marker *Acta2*, as well as with *Col1a1*, *Col3a1*, and *Fn1*, all of which were aggregated in the lesion areas (Figure S5A–F). These observations imply that ferritin may promote pulmonary fibrosis through regulating fibroblast differentiation. To test this possibility, we treated human lung fibroblasts (HPFs) with ferritin at concentrations ranging from 0 to 1000 ng/mL. After 48 h of stimulation, fibroblasts displayed myofibroblast‐like morphological changes, characterized by cell elongation, swelling, and increased pseudopodia formation (Figure 4B). RT‐qPCR analysis revealed robust upregulation of the myofibroblast marker *ACTA2* and elevated expression of ECM‐related genes *COL1A1*, *COL3A1*, and *FN1* in fibroblasts at 1000 ng/mL ferritin stimulation (Figure 4C–F). Consistently, Western blot assays further confirmed increased protein levels of α‐SMA, Collagen I, Collagen III, and fibronectin in ferritin‐treated fibroblasts (Figure 4G–K). Moreover, immunofluorescence staining further confirmed that 1000 ng/mL ferritin stimulation markedly enhanced α‐SMA, Collagen I, Collagen III, and fibronectin expression (Figure 4L–O, Figure S5G–J). Collectively, these results demonstrate that ferritin promotes fibroblasts‐to‐myofibroblast differentiation and ECM production, functioning as an independent pro‐fibrotic mediator.

### 2.4 Ferritin Drives Fibroblast Differentiation and Collagen Production Though PIK3R2/SMADs Signaling Pathway

**FIGURE 5:**
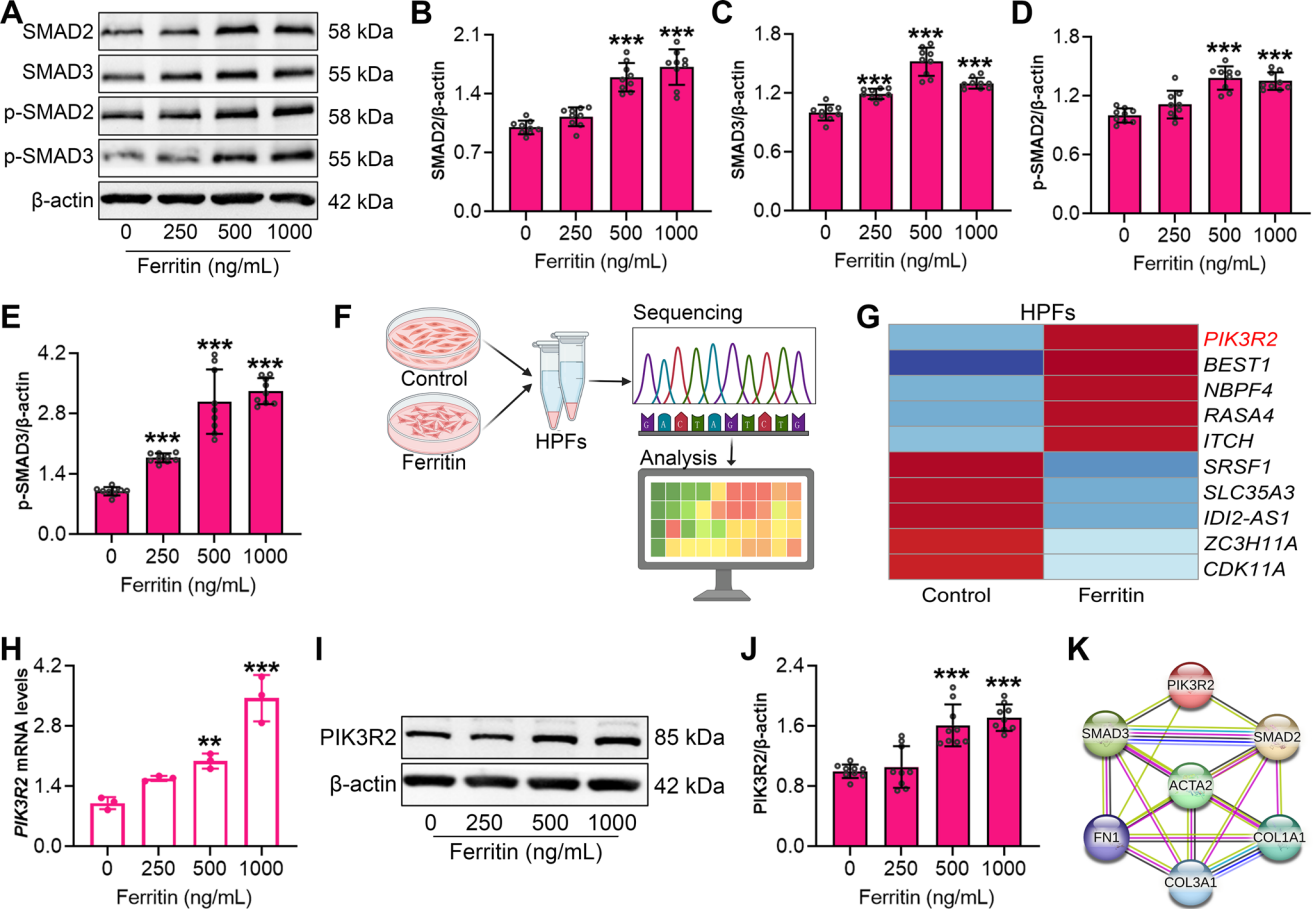
Ferritin activates the SMADs signaling pathway in fibroblasts via PIK3R2. A) Protein expression levels of SMADs pathway components in ferritin‐stimulated HPFs, as detected by Western blot. B‐E) Quantitative analysis of SMAD2, SMAD3, p‐SMAD2, p‐SMAD3 in HPFs treated with ferritin (n = 3 per group). F) Schematic diagram of RNA sequencing (Created in BioRender.com, with permission). G) Heatmap of the top five upregulated and downregulated genes in HPFs after 1000 ng/mL ferritin stimulation. H) *PIK3R2* mRNA expression in HPFs treated with 0, 250, 500, 1000 ng/mL ferritin for 48 h (n = 3 per group). I) PIK3R2 protein expression levels in ferritin‐stimulated HPFs, as detected by Western blot. J) Quantitative analysis of PIK3R2 protein in HPFs treated with ferritin (n = 3 per group). K) Interaction analysis of PIK3R2 between SMADs pathway and ECM‐related molecules. Data are presented as mean ± SD, ^*^ *p* < 0.05, ^**^ *p* < 0.01, ^***^ *p* < 0.001. Statistical analysis was performed using one‐way ANOVA followed by Dunnett's test (B, D, H) and Dunnett's T3 test (C, E, J).

Based on the transcriptomic results of lung tissues from silica and ferritin co‐exposed mice (Figure 4A), ferritin exposure significantly perturbed pathways involved in SMAD protein binding and phosphorylation. We therefore postulated that ferritin stimulation facilitates SMADs phosphorylation, a central event in pro‐fibrotic signaling, to drive fibroblast phenotypic transition and ECM production. To test this hypothesis, we treated HPFs with exogenous ferritin for 48 h and analyzed key proteins in the SMAD pathway. Ferritin stimulation markedly upregulated SMAD2 and SMAD3 protein expression (Figure 5A–C). Consistently, ferritin promoted the phosphorylation of both SMAD2 and SMAD3 (Figure 5D, E), confirming activation of the SMADs cascade. To further delineate how ferritin regulates SMADs signaling, we performed RNA sequencing on fibroblasts stimulated with 1000 ng/mL ferritin (Figure 5F). Differentially expressed genes analysis identified *PIK3R2* as one of the most upregulated genes in ferritin‐treated HPFs (Figure 5G). RT‐qPCR and Western blot analyses further confirmed that ferritin substantially increased PIK3R2 expression at both mRNA and protein levels (Figure 5H–J). Furthermore, we employed the STRING database to explore potential interactions between PIK3R2, the SMADs signaling pathway, and ECM‐related molecules (Figure 5K). The analysis revealed that PIK3R2 was functionally associated with the expression of SMAD2 and SMAD3, and then SMAD2 and SMAD3 directly linked to the myofibroblast marker α‐SMA (*ACTA2*) as well as ECM proteins, including Collagen I (*COL1A1*), Collagen III (*COL3A1*), and fibronectin (*FN1*), thereby forming a coherent signaling cascade. The above results suggest that PIKR32 may serve as a critical mediator through which ferritin activates SMADs pathway, thereby driving fibroblast differentiation and ECM production.

**FIGURE 6:**
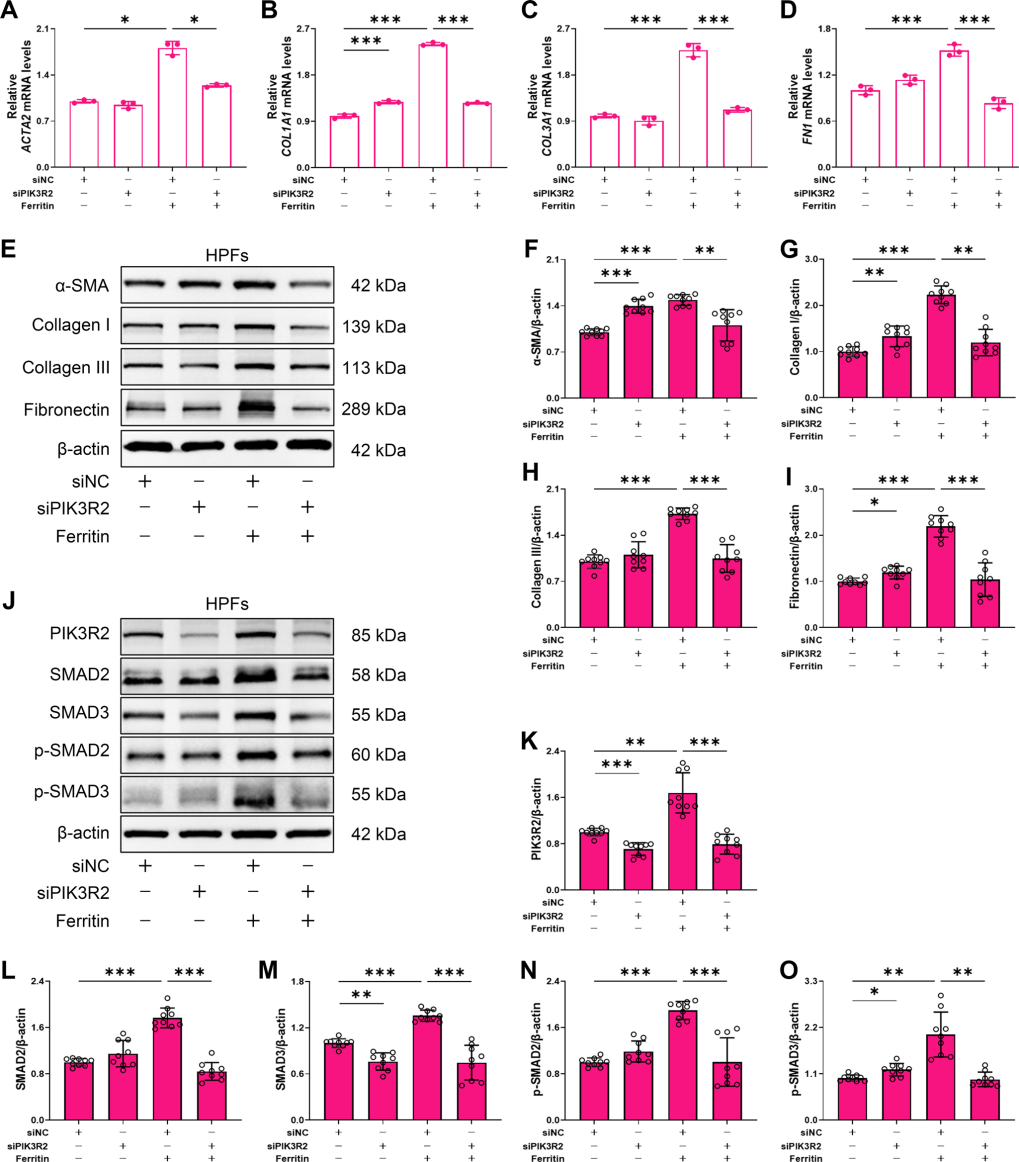
PIK3R2 knockdown inhibits ferritin‐induced ECM production and SMADs signaling activation in fibroblasts. A‐D) mRNA expression levels of *ACTA2*, *COL1A1*, *COL3A1*, and *FN1* in *PIK3R2*‐knockdown HPFs treated with 1000 ng/mL ferritin, as detected by RT‐qPCR (n = 3 per group). E) Protein expression of α‐SMA, Collagen I, Collagen III, and fibronectin was analyzed by Western blot under the same conditions. F‐I) Quantitative analysis of α‐SMA, Collagen I, Collagen III, and fibronectin proteins in *PIK3R2*‐knockdown HPFs (n = 3 per group). J) Expressions of SMADs signaling pathway proteins in *PIK3R2*‐knockdown HPFs after 1000 ng/mL ferritin treatment. K‐O) Quantitative analysis of PIK3R2, SMAD2, SMAD3, p‐SMAD2 and p‐SMAD3 proteins expression (n = 3 per group). Data are presented as mean ± SD, ^*^ *p* < 0.05, ^**^ *p* < 0.01, ^***^ *p* < 0.001. Statistical analysis was performed using one‐way ANOVA followed by LSD (B, C, D, G, H) and Dunnett's T3 test (A, F, I, K–O).

To further validate the role of PIK3R2 in ferritin‐regulated fibroblast differentiation, we generated *PIK3R2‐*knockdown HPFs using specific siRNA (Figure S6A). Since PIK3R2 is a well‐known regulator of PI3K signaling, we assessed PI3K and AKT protein levels by Western blot. The results showed no significant change in PI3K or p‐AKT expression (Figure S6B,C), suggesting that the canonical PI3K/AKT pathway was not perturbed under *PIK3R2* knockdown. Subsequently, *PIK3R2*‐knockdown HPFs were treated with 1000 ng/mL ferritin. RT‑qPCR analysis showed that PIK3R2 inhibition markedly attenuated ferritin‐induced *ACTA2* expression, accompanied by decreased mRNA levels of *COL1A1*, *COL3A1*, and *FN1* in HPFs (Figure 6A–D). Consistently, Western blot results showed decreased protein levels of α‐SMA, Collagen I, Collagen III, and fibronectin following *PIK3R2*‐knockdown (Figure 6E–I), indicating that PIK3R2 inhibition suppresses ferritin‐driven fibroblast differentiation and ECM production. As expected, PIK3R2 depletion significantly inhibited both the expression and phosphorylation of SMAD2 and SMAD3 (Figure 6J–O). To further investigate whether PIK3R2 mediates ferritin‐induced SMADs signaling, we performed a rescue experiment by re‐activating PIK3R2 in knockdown cells. Following rescue, the expression of SMAD2 and p‐SMAD2 was partially restored. For SMAD3, although its basal expression did not change significantly after PIK3R2 activation, the level of phosphorylated SMAD3 was markedly upregulated (Figure S6D–I). These findings demonstrated that PIK3R2 mediates ferritin‐activated SMADs signaling in fibroblasts and that PIK3R2 inhibition reverses fibroblast differentiation and suppresses ECM production.

### 2.5 Ferritin Mainly Originates From Macrophages During Silica‐Exposure

**FIGURE 7:**
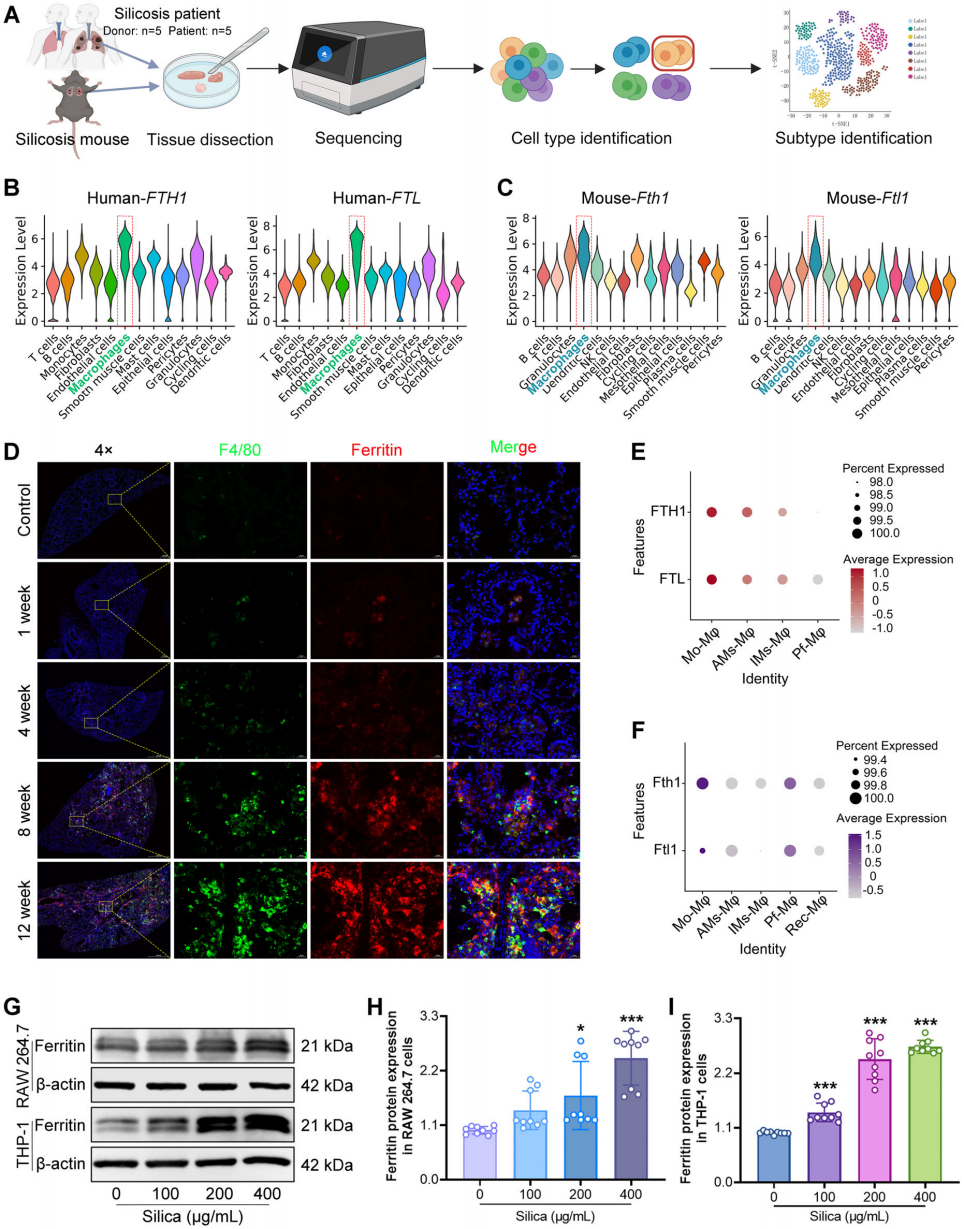
Ferritin is predominantly derived from macrophages following silica exposure. A) Single‐cell RNA‐sequencing workflow. Lung tissues from silicosis patients and silica‐challenged mice were dissociated into single‐cell suspensions and analyzed using the 10x Genomics Chromium. Cell populations were defined according to molecular signatures, and macrophage subtypes were further re‐clustered for downstream analysis (Created in BioRender.com, with permission). B) Single‐cell RNA sequencing analysis of *FTH1* and *FTL* expression across cell types in human lung tissue (n = 5 per group). C) Single‐cell RNA sequencing analysis of *Fth1* and *Ftl1* expression across cell types in mouse lung tissue (n = 3 per group). D) Multiplex immunofluorescence staining demonstrating co‐localization of ferritin with the macrophage marker F4/80 in lung tissue (magnification, 4×, 40×). E, F) Expression of ferritin‐encoding genes in different macrophage subtypes in human and mouse lungs. G) Ferritin protein expression in RAW 264.7 and THP‐1 stimulated with 0, 100, 200, 400 µg/mL silica for 48 h, assessed by Western blot. H, I) Quantitative analysis of ferritin protein levels in RAW 264.7 cells and THP‐1 cells after silica treatment (n = 3 per group). Data are presented as mean ± SD, ^*^ *p* < 0.05, ^***^ *p* < 0.001. Statistical analysis was performed using one‐way ANOVA followed by Dunnett's T3 test (H and I).

Given the profibrotic role of ferritin, targeting ferritin may represent a potential strategy for silicosis treatment. To precisely target ferritin for silicosis treatment, we performed single‐cell RNA sequencing to delineate its cellular source following silica exposure (Figure 7A). Human lung tissue samples were obtained from five healthy donors and five patients with stage III silicosis, and all patients were confirmed as simple silicosis characteristics by histopathological staining before sequencing. Our analysis revealed that ferritin‐coding genes *FTH1* and *FTL* were predominantly expressed in macrophages in both humans and mice (Figure 7B, C). To further validate whether ferritin predominantly originates from macrophages, we carried out multiplex immunofluorescence staining on silica‐injured lungs at different durations. We observed co‐localization of ferritin with the macrophage marker F4/80 (Figure 7D), and consistent with mRNA and protein expression data, the ferritin fluorescence intensity of ferritin increased progressively with prolonged silica exposure time (Figure S7A, B). Moreover, we established a macrophage‐depletion silicosis model to determine whether macrophage ablation modulates ferritin expression in the lung and circulation. Administration of clodronate liposomes (CTL), a chemical macrophage‐depleting reagent, markedly reduced the protein abundance of F4/80 and Cd11b in lung tissue, confirming efficient macrophage depletion (Figure S7C, E, F). Notably, macrophage depletion substantially suppressed ferritin accumulation in lung tissue compared with silica‐exposed mice (Figure S7D). Consistently, serum ferritin concentrations were also significantly diminished following macrophage depletion (Figure S7G). These data indicate that macrophages are the dominant cellular source of ferritin in the lung microenvironment and serum following silica exposure.

Given that macrophage function is closely linked to their phenotypic heterogeneity under disease conditions, we next investigated which macrophage subpopulation contributes more to ferritin production after silica exposure. Subcluster analysis demonstrated that monocyte‐derived macrophages (Mo‐Mφ) exhibited the highest ferritin expression in both human and murine lungs (Figure 7E, F). Subsequently, to test whether silica stimulation directly induces ferritin expression in this subpopulation, we treated mouse mononuclear‐derived macrophages (RAW 264.7) and human mononuclear‐derived macrophages (THP‐1) with increasing concentrations of silica. The results demonstrated that silica stimulation markedly increased ferritin expression in both human and mouse mononuclear‐derived macrophages (Figure 7G–I). Collectively, these findings indicate that ferritin is predominantly derived from monocyte‐derived macrophages in the lung and can be directly upregulated by silica exposure.

### 2.6 Macrophage‐Target Ferritin Knockdown Ameliorates Silica‐Induced Pulmonary Fibrosis in Mice

Given that ferritin regulates SMADs signaling activation in fibroblasts via PIK3R2 and promotes collagen deposition (Figure 6), and that ferritin is predominantly expressed in macrophages in lung tissues after silica exposure, especially pathogenic monocyte‐derived macrophages (Figure 7). We hypothesized that macrophage‐specific inhibition of ferritin could alleviate silica‐induced pulmonary fibrosis. Since ferritin plays an essential role in iron homeostasis, complete inhibition of ferritin expression in vivo may disrupt systemic iron balance. Previous studies have shown that lung *FTL* expression increases rapidly under pathological conditions, while *FTH1* levels remain largely unchanged [21]. Moreover, *Fth1* can be compensatorily upregulated to maintain iron balance under *Ftl1* deficiency in mice [22]. Therefore, to minimize the potential iron‐related metabolic disturbances while investigating the therapeutic relevance of ferritin in silicosis, we selectively targeted *Ftl1* to alternatively assess whether ferritin inhibition ameliorates silica‐induced pulmonary fibrosis in vivo. To achieve macrophage‐specific ferritin knockdown, we synthesized short hairpin RNA (shRNA) targeting the mouse *Ftl1* gene and packaged it into recombinant adeno‐associated virus 6 (AAV6) under the control of the F4/80 promoter (AAV6‐F4/80‐*shFtl1*). Two weeks after intratracheal infusion of the virus, ZsGreen fluorescence, which indicates AAV6 infection, was visible in the lung tissue (Figure S8A). Western blot analysis confirmed a marked reduction of ferritin protein expression in lung tissues compared with control mice (Figure S8B, C), demonstrating the successful establishment of a macrophage‐specific ferritin knockdown mouse model.

**FIGURE 8:**
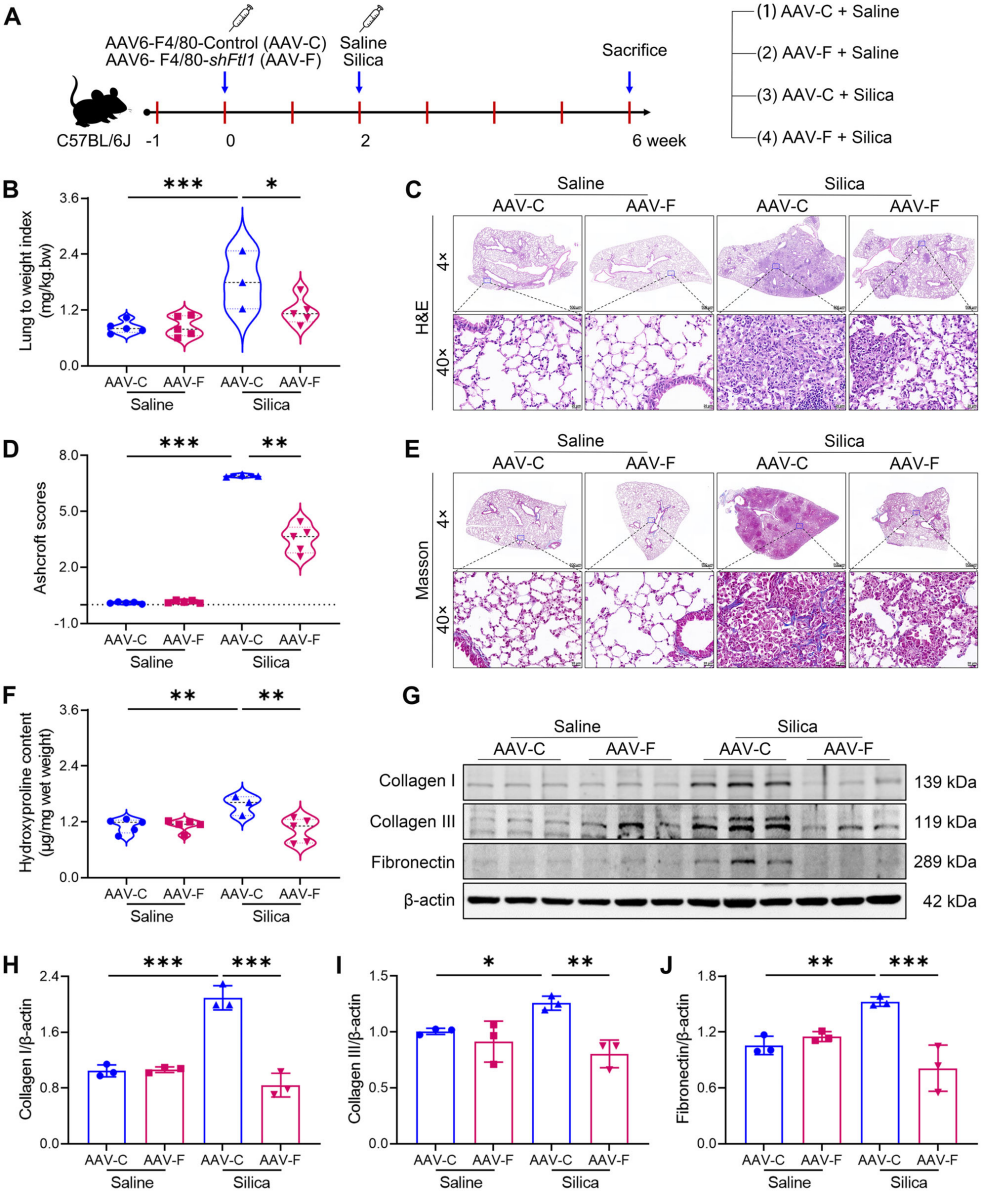
Reduced ferritin expression alleviates silica‐induced pulmonary fibrosis in mice. A) Schematic of in vivo study design. Mice were given an intratracheal infusion of AAV6 carrying *Ftl1*‐specific shRNA. After two weeks of infection, silica was administered intratracheally, and lung tissues were collected after four weeks of silica exposure. B) Lung weight coefficients in silica‐induced pulmonary fibrosis mice with or without ferritin knockdown (n = 3 or 5 per group). C) Representative H&E staining results of lung tissues (magnification, 4×, 40×). D) Ashcroft scores of silica‐induced pulmonary fibrosis mice with or without ferritin knockdown (n = 3 or 5 per group). E) Representative Masson's trichrome staining of lung tissues (magnification, 4×, 40×). F) Hydroxyproline content of lung tissue (n = 4 or 5 per group). G) Expression of ECM proteins (Collagen I, Collagen III, and fibronectin) in lung tissue evaluated by Western blot. H–J) Quantitative analysis of Collagen I, Collagen III, and fibronectin protein levels (n = 3 per group). Data are presented as mean ± SD, ^*^ *p* < 0.05, ^**^ *p* < 0.01, ^***^ *p* < 0.001. Statistical analysis was performed using one‐way ANOVA followed by LSD (B, F, H‐J) and Dunnett's T3 test (D).

Subsequently, mice were received intratracheal silica instillation to induce pulmonary fibrosis, and lung tissues were harvested after 4 weeks of silica exposure (Figure 8A). Relative to AAV control mice (AAV‐C + saline), the lung coefficient was dramatically increased in silica‐exposed mice (AAV‐C + silica), which was significantly attenuated by macrophage‐targeted ferritin knockdown (Figure 8B). Histopathological analysis revealed that silica‐exposed lungs exhibited large areas of confluent cellular nodules and parenchymal injury, whereas ferritin knockdown resulted in smaller, localized nodules (Figure 8C). Correspondingly, ferritin knockdown significantly decreased Ashcroft scores, indicating attenuated fibrosis severity (Figure 8D). Collagen deposition is a hallmark of pulmonary fibrosis. Silica exposure induced classical cord‐like collagen deposition in lungs, whereas ferritin knockdown markedly limited collagen accumulation, even at lesion sites, accompanied by reduced hydroxyproline levels (Figure 8E, F). Furthermore, ferritin inhibition significantly downregulated ECM proteins, including Collagen I, Collagen III, and fibronectin (Figure 8G–J), verifying that ferritin knockdown alleviates silica‐induced fibrotic lesions and collagen deposition.

**FIGURE 9:**
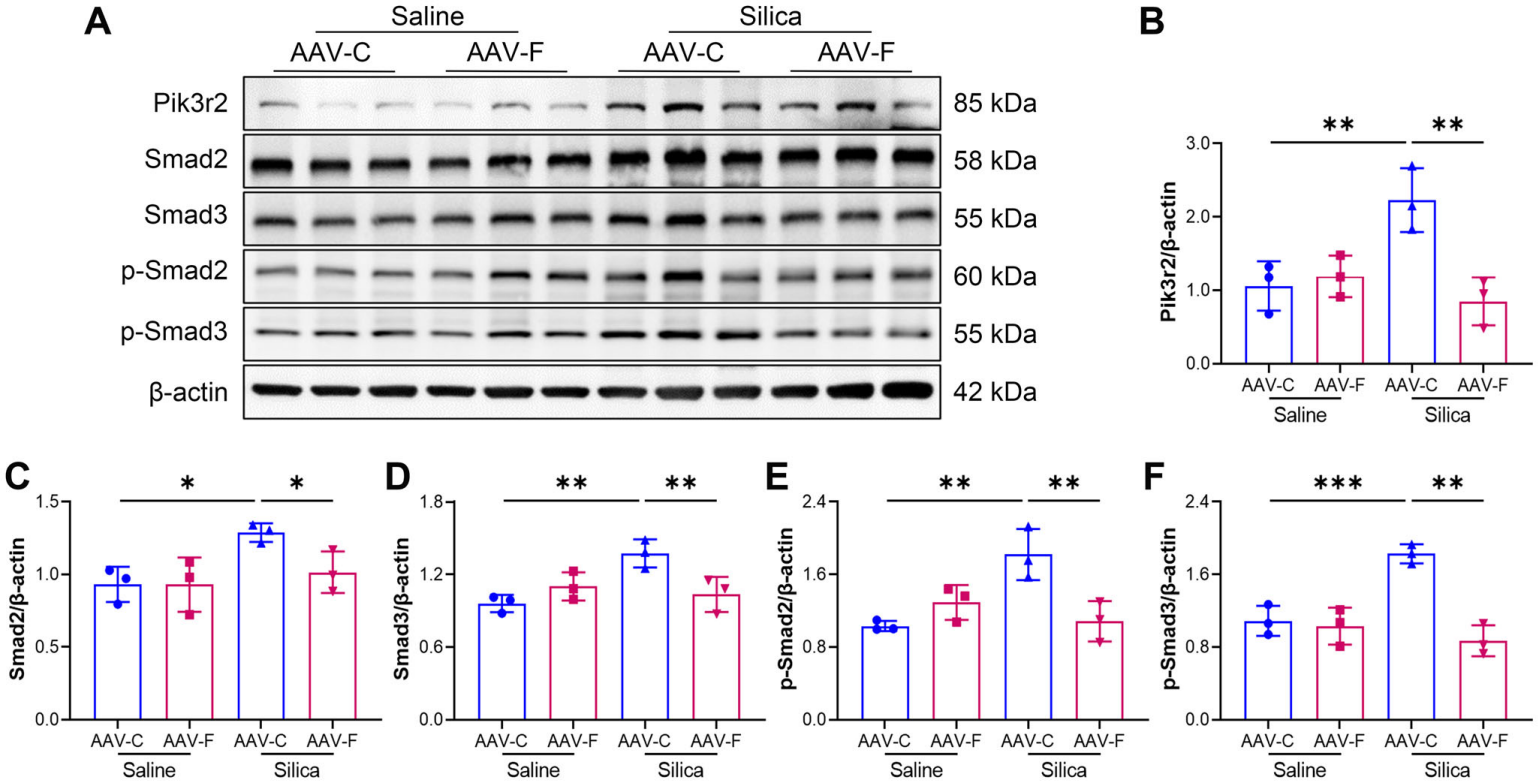
Reduced ferritin expression in macrophages inhibits Pik3r2/Smads pathway activation in mice. A) Expression of Pik3r2/Smads signaling pathway proteins in lung tissues from silica‐induced mice with or without ferritin knockdown, as detected by Western blot. B) Quantitative analysis of Pik3r2 protein expression (n = 3 per group). C–F) Quantitative analysis of Smad2, Smad3, p‐Smad2, and p‐Smad3 protein expression (n = 3 per group). Data are presented as mean ± SD, ^*^ *p* < 0.05, ^**^ *p* < 0.01, ^***^ *p* < 0.001. Statistical analysis was performed using one‐way ANOVA followed by LSD (B‐F).

Given that ferritin is critical for iron homeostasis, we investigated the impact of ferritin modulation on iron levels in lung tissue. We found that ferritin inhibition in healthy mice did not affect serum ferritin concentrations, but in silica‐exposed mice, ferritin knockdown reduced circulating ferritin levels (Figure S8D). Importantly, under both normal and pathological conditions, ferritin inhibition did not result in significant iron deposition or changes in total iron levels in lung tissue (Figure S8E, F). These findings further support the notion that ferritin‐mediated fibrosis is not influenced by iron concentration. We then asked whether ferritin knockdown hinders silica‐induced Pik3r2/Smads pathway activation in silica‐exposed mice. Western blot analysis showed that silica exposure upregulated Pik3r2 expression and activated the Smads pathway, as evidenced by elevated Smad2, Smad3, p‐Smad2, and p‐Smad3 levels, and ferritin knockdown suppressed these molecular changes (Figure 9). Collectively, these findings demonstrate that targeting ferritin in macrophages effectively alleviates silica‐induced pulmonary fibrosis by inhibiting Pik3r2/Smads pathway activation.

### 2.7 Macrophage‐Specific Ferritin Knockdown Suppresses Silica‐Induced Fibroblast Differentiation and ECM Production

To investigate whether ferritin drives silicosis via macrophage‐fibroblast crosstalk, we established a macrophage‐fibroblast co‐culture model to mimic intercellular crosstalk under silica challenge. A key prerequisite for establishing this co‐culture system is the extracellular release of ferritin by silica‐stimulated macrophages. Therefore, we extracted proteins from the culture supernatant of silica‐stimulated macrophages using the chloroform‐methanol method. Western blot results indicated that ferritin levels in the supernatant were markedly upregulated with increasing silica stimulation concentration (Figure S9A, B), demonstrating that macrophages release ferritin into the extracellular space upon silica exposure. We next established a co‐culture system with THP‐1‐derived macrophages in the upper chamber and human pulmonary fibroblasts in the lower chamber. Macrophages were treated with 0, 100, 200, or 400 µg/mL silica for 48 h, and mRNA was subsequently extracted from fibroblasts to assess whether silica‐stimulated macrophages induce fibroblast differentiation. Consistently, RT‐qPCR results showed that the expression levels of *ACTA2*, *COL1A1*, *COL3A1*, and *FN1* mRNA were all upregulated in fibroblasts (Figure S9C–F), with significant increases observed at 200 and 400 µg/mL of silica treatment compared with control. These findings confirm the successful establishment of a macrophage‐driven fibroblast differentiation co‐culture model under silica exposure, and a silica concentration of 200 µg/mL was selected for subsequent experiments.

**FIGURE 10:**
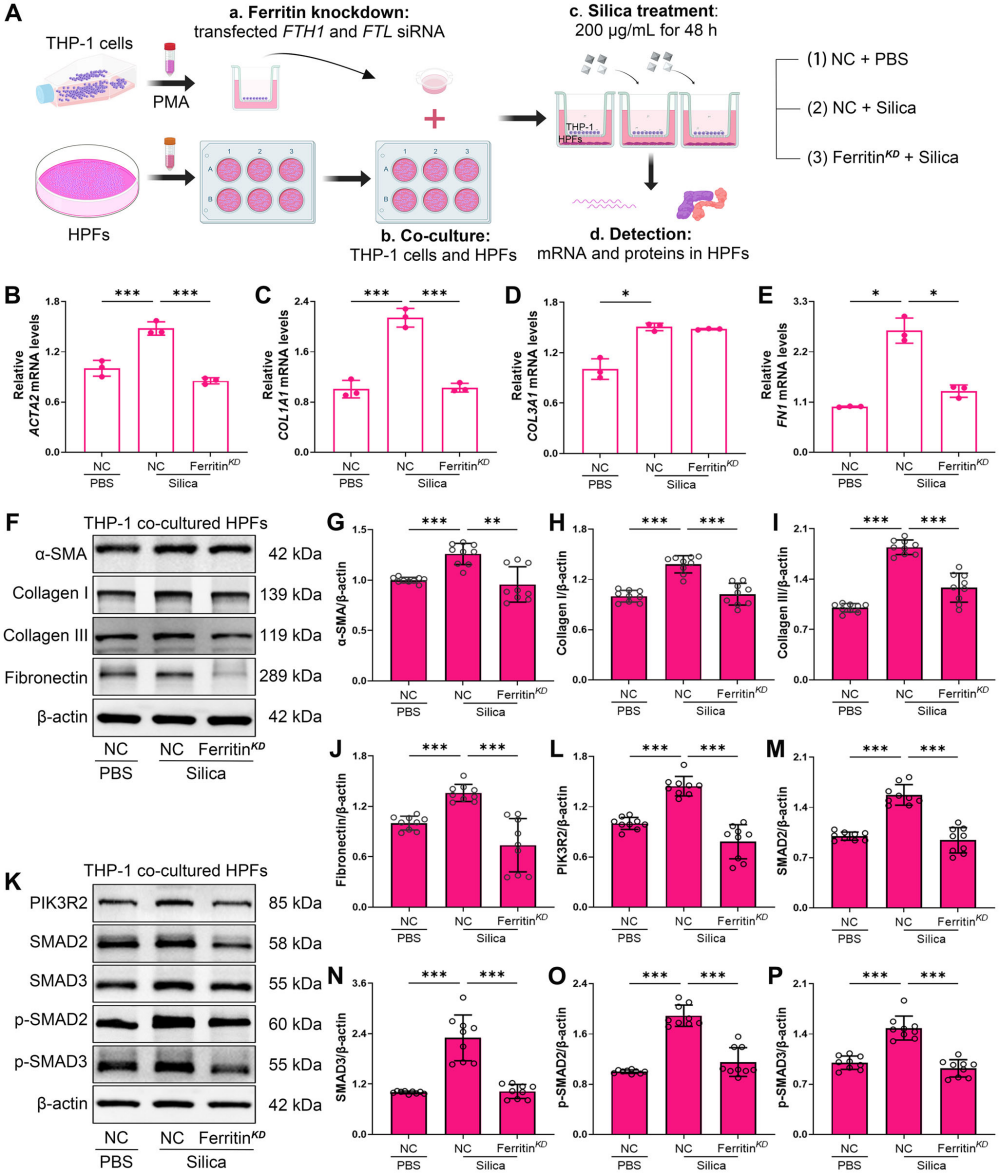
Ferritin‐knockdown in macrophages attenuates silica‐induced fibroblast differentiation and ECM production. A) Schematic diagram of the macrophages and fibroblasts co‐culture system. THP‐1 cells were induced into macrophages and transfected with *FTH1* and *FTL* siRNA for 24 h, then transferred to 6‐well plates containing pre‐seeded HPFs. Macrophages were stimulated with 200 µg/mL silica, and after 48 h of co‐culture, fibrosis‐related markers in HPFs were detected. B‐E) mRNA expression levels of *ACTA2*, *COL1A1*, *COL3A1*, and *FN1* in HPFs after co‐culture with ferritin‐knockdown macrophages and silica stimulation, as determined by RT‐qPCR (n = 3 per group). F) Protein expression of α‐SMA, Collagen I, Collagen III, and fibronectin in co‐cultured HPFs evaluated by Western blot. G‐J) Quantitative analysis of α‐SMA, Collagen I, Collagen III, and fibronectin protein expression in co‐cultured HPFs (n = 3 per group). K) Expression of PIK3R2/SMADs signaling pathway proteins in co‐cultured HPFs. L‐P) Quantitative analysis of PIK3R2/SMADs signaling pathway proteins (n = 3 per group). Data are presented as mean ± SD, ^*^ *p* < 0.05, ^**^ *p* < 0.01, ^***^ *p* < 0.001. Statistical analysis was performed using one‐way ANOVA followed by Dunnett's test (B, C, H, P) and Dunnett's T3 test (D, E, G, I–M, N, O).

To further investigate the therapeutic potential of targeting ferritin in silicosis, we silenced ferritin expression in macrophages by co‐transfecting THP‐1 cells with *FTH1* and *FTL* siRNA (Figure S9G–I). These transfected macrophages were then co‐cultured with HPFs and stimulated with 200 µg/mL silica (Figure 10A). After 48 h of co‐culture, the expression of fibrotic and SMAD signaling components in fibroblasts was analyzed by RT‐qPCR and Western blot. Ferritin knockdown in macrophages markedly attenuated silica‐induced fibrotic genes expression in fibroblasts (Figure 10B–E). Silica‐stimulated macrophages robustly induced α‐SMA expression in co‐cultured fibroblasts (Figure 10F, G), along with upregulation of classic ECM proteins, including Collagen I, Collagen III, and fibronectin (Figure 10H–J). Notably, ferritin knockdown in macrophages reduced the expression of α‐SMA and ECM‐related proteins in co‐cultured fibroblasts. Consistent with ferritin knockdown in vivo, ferritin knockdown in macrophages reduced PIK3R2 expression and impaired SMADs pathway activation, as evidenced by decreased expression and phosphorylation of SMAD2 and SMAD3 in fibroblasts (Figure 10K–P). Collectively, these findings identify ferritin as a critical signaling mediator of macrophage‐fibroblast crosstalk in silicosis progression. Following silica exposure, macrophage‐secreted ferritin facilitates fibroblast‐to‐myofibroblast differentiation and collagen deposition though PIK3R2/SMADs signaling axis.

## 3 Discussion

Silicosis is a progressive and ultimately fatal fibrotic lung disease that remains irreversible. Thus, early detection and continuous surveillance after diagnosis are essential to enable timely clinical interventions. In this study, we demonstrated that ferritin is markedly upregulated in lung tissues and serum from both silicosis patients and silica‐exposed mice, and its expression correlates with disease progression in an iron‐independent manner. Notably, we found that excess ferritin exacerbates silica‐induced pulmonary fibrosis. Mechanistically, silica‐induced ferritin secretion from macrophages facilitates fibroblast differentiation and ECM production via PIK3R2/SMADs signaling axis, thereby accelerating pulmonary fibrosis. These findings provide novel mechanistic insights into the pathogenesis of silica‐induced pulmonary fibrosis. Furthermore, our study highlights a potential translational framework in which ferritin could serve as a biomarker for early screening, while ferritin itself or its downstream effector PIK3R2 may represent actionable therapeutic targets. Together, these results propose a three‐tiered management strategy integrating biomarker‐based surveillance with targeted interventions to mitigate silicosis progression.

Elevated ferritin levels are frequently observed in inflammatory and infectious conditions such as sepsis and septic shock, where it serves as an auxiliary diagnostic indicator [23, 24]. Serum ferritin often rises earlier than C‐reactive protein (CRP) during acute inflammation and shows higher sensitivity in infectious diseases [25]. In our study, we found that serum ferritin levels increased with disease progression in silicosis patients, and this abnormality was further validated in a silica‐exposed mouse model. Consistent with our results, ferritin elevations have been reported in dust‐ or smoke‐exposed lungs and patients with acute exacerbation of idiopathic pulmonary fibrosis [16, 26, 27]. Notably, serum ferritin levels correlate with disease severity. Higher ferritin levels are associated with increased risk of concurrent pulmonary tuberculosis and higher mortality in pneumoconiosis, and are associated with severe disease and mortality in COVID‐19 [28, 29]. Although ferritin is traditionally recognized as a marker of iron metabolism, studies in liver fibrosis have demonstrated that serum ferritin, rather than hepatic iron concentration, more accurately predicts fibrosis severity and histological damage [30, 31]. Together, these clinical data establish a robust association between ferritin elevation, fibrosis severity, and poor prognosis. Consistently, our work found that iron concentration in lung tissue did not change despite marked increases of ferritin in serum and lung parenchyma, reinforcing that silica‐induced ferritin elevation occurs independently of iron overload. Given that ferritin assays are widely standardized and clinically available, our findings, together with past reports, underscore its considerable potential as a noninvasive biomarker for early screening and risk stratification in silicosis.

Previous studies have reported that iron overload accelerates fibrosis through ferroptosis by inducing ROS accumulation, GSH depletion, and lipid peroxidation [32]. However, in our mouse models, silica exposure did not result in increased iron deposition or total iron concentration in lung tissues, suggesting that ferritin‐driven fibrosis occurs independently of iron regulation. This is consistent with reports showing that exogenous ferritin promotes cancer cell proliferation in an iron‐independent manner [33]. Mechanistically, recent evidence demonstrates that ferritin can induce neutrophil extracellular trap formation, activate B‐cell immunity, and amplify cytokine storm responses by engaging macrophage scavenger receptor 1 [14]. In line with these observations, systemic administration of ferritin in our study markedly aggravated silica‐induced pulmonary fibrosis, also consistent with prior observations in sepsis [34]. Given previous evidence that serum ferritin correlates with disease severity and prognosis, mechanistic studies further revealed that ferritin activates PI3K/PKC/MAPK signaling and promotes nuclear translocation of NF‐κB in hepatic stellate cells independent of iron levels, thereby promoting cytokine release and liver fibrosis [35, 36]. Conversely, silencing *Fth1* to reduce ferritin expression alleviates rhabdomyolysis‐induced renal fibrosis [15]. Together, these findings indicate that ferritin is not merely a passive marker of iron metabolism but also functions as a direct pro‐fibrotic effector, underscoring its pivotal role in silica‐induced pulmonary fibrosis.

SMADs signaling represents a canonical pathway governing the initiation and progression of fibrosis, particularly through macrophage polarization and fibroblast differentiation [37]. Studies have revealed that macrophage‐derived ferritin contributes to cell proliferation and differentiation via the PI3K/AKT signaling pathway [38, 39], implicating its function in tissue remodeling. Consistently, we observed that exogenous ferritin promoted fibroblast‐to‐myofibroblast transition and enhanced ECM production, whereas ferritin knockdown in macrophages suppressed SMADs protein upregulation in both silica‐exposed mice and co‐cultured fibroblasts. A similar regulatory pattern has been reported in renal fibrosis, where *Fth1* deficiency impeded S100A8‐TLR‐SMAD signaling and attenuated fibrotic progression [15]. These data collectively establish ferritin as an active upstream regulator of SMAD‐mediated fibrotic signaling. We further identified PIK3R2 as a key intermediary that links ferritin to SMAD activation. Indeed, PIK3R2 mutations have been associated with tumor overgrowth by driving epithelial‐mesenchymal transition and activating the GSK3β/β‐catenin pathway [40, 41]. In line with this mechanistic framework, PIK3R2 was significantly upregulated in our omics analysis, and rescue experiments confirmed its pivotal role as an intermediary mediator of ferritin‐induced SMAD activation and fibroblast differentiation. Although PIK3R2 is a classically regulatory subunit of PI3K [42], no activation of the PI3K/AKT signaling axis was observed in ferritin‐treated fibroblasts. This may be explained by the fact that PIK3R2 is not the dominant regulatory subunit for PI3K, whereas PIK3R1 plays the dominant role, and PIK3R2 can act independently of canonical PI3K/AKT signaling in regulating cell growth [43, 44, 45]. Together, these findings reveal that ferritin drives fibroblast differentiation and ECM deposition through a PIK3R2‐mediated but PI3K/AKT‐independent mechanism, revealing a previously unrecognized signaling axis underlying silica‐induced pulmonary fibrosis.

Aberrant macrophage‐fibroblast crosstalk under sustained silica exposure constitutes a key driver of silicosis pathogenesis. Macrophages activate fibroblasts through direct contact or cytokine secretion, while activated fibroblasts in turn amplify macrophage‐mediated inflammation, forming a feed‐forward loop that accelerates pulmonary fibrosis [46, 47]. As the primary cells exposed to inhaled particulates, macrophages clear silica through phagocytosis, and their numbers, subtypes, and functions shift dynamically throughout both the inflammatory and fibrotic stages of silicosis [48, 49]. Based on single‐cell RNA sequencing results, we identified macrophages as the predominant source of ferritin, consistent with findings in cigarette smoke‐induced fibrosis and autoimmune lung disease [26, 50]. Particularly, ferritin was highly expressed in monocyte‐derived macrophages and could be secreted into extracellularly. Fibroblasts serve as central effectors of tissue repair and fibrotic progression in response to cytokine and mechanical stimuli, thereby producing excessive collagen fibers through complex signaling pathway changes and metabolic reprogramming, ultimately leading to ECM deposition [51]. Although fibroblasts do not directly contact inhaled particles, they are activated by microenvironmental changes such as TGF‐β1 and PDGF stimulation [52]. Furthermore, spatial epitranscriptomic analyses have shown that monocyte‐derived macrophages drive fibroblasts into a pro‐fibrotic phenotype, whereas their depletion attenuates pulmonary fibrosis [9, 53]. In our study, we observed that ferritin localized within myofibroblast foci in fibrotic lung, and ferritin secreted by macrophages engaged fibroblasts to promote myofibroblast differentiation and ECM deposition. Collectively, these findings identify ferritin as a critical pro‐fibrotic mediator of macrophage‐fibroblast crosstalk, underscoring its central role in amplifying silica‐induced pulmonary fibrosis.

Ferritin is composed of two subunits, FTH1 and FTL, whose relative abundance varies across tissues and correlates with functional heterogeneity [25]. FTH1‐enriched ferritin predominates in the heart, kidney, and brain, whereas FTL‐rich ferritin is mainly distributed in the liver, spleen, and bone marrow [54]. Notably, the FTH1/FTL ratio also changes under pathological microenvironments. In the lung, physiological ferritin exhibits comparable levels of both subunits, but pathogenic stimuli such as silicosis and hypoxia preferentially induce FTL expression in bronchoalveolar lavage fluid and serum, functionally promote epithelial–mesenchymal transition [16, 21], indicating that FTL may be the dominant ferritin component involved in pulmonary pathology. This subunit preference may reflect their distinct biological functions. FTH1 provides essential ferroxidase activity for iron sequestration, whereas FTL deficiency can be compensated by FTH1 to maintain iron homeostasis [22], implying that FTL may exert broader signaling functions beyond iron storage. Indeed, FTL has been shown to regulate PI3K/AKT, GADD45/JNK, and TGF‐β1 pathways and activate hepatic stellate cells via NF‐κB signaling [55, 56]. These findings support the hypothesis that in diseases where ferritin elevation occurs independently of iron overload, FTL serves as the principal driver of pathological remodeling. Consistent with these observations, our study demonstrates that selective targeting of *Ftl1* to reduce ferritin expression attenuated silica‐induced pulmonary fibrosis without altering iron levels, indicating that FTL contributes to fibrotic progression through iron‐independent mechanisms. These findings suggest that FTL may represent the major pathogenic effector subunit of ferritin in silicosis, future work incorporating subunit‐specific manipulation and detection strategies will be essential to definitively determine the respective roles of FTH1 and FTL in silicosis and guide the development of subunit‐targeted therapeutic interventions.

Early diagnosis and continuous surveillance are critical medical strategies for improving survival outcomes and reducing late‐stage disability and mortality in silicosis. Although we utilized experimental silica dust free of metal ions as the inducer and observed iron‐independent ferritin overexpression in vivo, iron remains a common component adsorbed on silica particles in real‐world occupational settings. Accordingly, elevated ferritin levels in serum and lung samples from silicosis patients may also partially result from iron exposure. Moreover, due to the limited sample size, we were unable to perform an accurate correlation analysis between serum ferritin levels and silicosis severity in the current study. Thus, large‐scale cohort studies are required to account for both occupational environment related iron exposure and systemic iron status, and to clarify the relative contributions of silica dust versus occupational iron exposure to ferritin elevation. Importantly, ferritin consists of FTH1 and FTL subunits that self‐assemble into a functional heteropolymer with distinct expression patterns and biological activities, yet we treated ferritin as a single entity in the current study, which limits mechanistic resolution. While we reduced total ferritin expression by targeting the *Ftl1* gene in vivo, the present data do not determine whether the pro‐fibrotic activity of ferritin is predominantly mediated by FTH1, FTL, or their specific FTH1/FTL stoichiometry. This unresolved question represents a major limitation of our findings. Future studies should dissect the differential roles of FTH1 versus FTL in macrophage‐fibroblast crosstalk and fibrosis, and define their distinct toxicological profiles and therapeutic utilities, including the possibility of selectively repurposing or modifying ferritin‐derived subunits for safe nanodelivery.

In summary, our work demonstrates that ferritin functions as an iron‐independent pathogenic signaling mediator in silica‐induced pulmonary fibrosis through macrophage‐fibroblast crosstalk. Ferritin secreted by macrophages upregulates PIK3R2 in fibroblasts, thereby activating SMADs signaling, driving fibroblast‐to‐myofibroblast differentiation, and facilitating ECM deposition. Collectively, these findings highlight ferritin as a promising clinical biomarker for early diagnosis and underscore PIK3R2 as a potential therapeutic target for silicosis intervention.

## 4 Experimental Section

### 4.1 Human Lung and Serum Specimen Harvest

Lung tissues and serum samples from patients with silicosis were obtained from the West China Occupational Pneumoconiosis Cohort Study (WCOPCS). Lung tissue samples from patients (n = 5) were collected via CT‐guided percutaneous lung biopsy and were used for single‐cell RNA sequencing and ferritin immunohistochemical staining after being confirmed as simple silicosis by pathological staining. Control lung tissue samples (n = 5) were obtained from non‐diseased margins of surgically resected lungs. Serum samples (n = 20) were collected from the established occupational pneumoconiosis cohort for quantification of ferritin levels. The study was approved by the Medical Ethics Committee of West China Fourth University Hospital, Sichuan University (Approval Numbers: EC‐2022040, EC‐2022105), and conducted in accordance with the ethical standards outlined in the 1964 Declaration of Helsinki and its later amendments, or comparable ethical standards.

### 4.2 Experimental Animals and Treatment

Male C57BL/6J mice aged 6‐8 weeks were purchased from Beijing Charles River Laboratories (Beijing, China), license number: SCXK (Jing) 2021‐0011. Mice were housed in a specific pathogen‐free (SPF) facility with a temperature of 25 ± 2°C and a humidity of 40%–70%, and were kept under a 12/12 h light‐dark cycle. The animal experiment scheme was approved by the Ethics Committee of West China School of Public Health/West China Fourth Hospital, Sichuan University (Approval Numbers: Gwll2023128, Gwll2023233), and was performed in accordance with the ethical standards as laid down in the 1964 Declaration of Helsinki and its later amendments or comparable ethical standards. After quarantine, mice were randomly divided into control and model groups based on body weight. Subsequently, mice were anesthetized via intraperitoneal injection of 50 mg/kg sodium pentobarbital (dissolved in saline), followed by intratracheal instillation of silica (Forsman, 1407207‐A, sterilized at 200°C for 2 h and suspended in saline at 40 mg/mL, 100 µL per mouse). Mice were gently agitated to ensure uniform distribution of silica. Mice were sacrificed after 1, 4, 8, and 12 weeks of silica exposure, respectively. Serum was collected for ELISA assays, and lung tissues were harvested for histological staining, single‐cell RNA sequencing, and molecular analyses.

For experiments involving concurrent silica and ferritin exposure, given the short duration of the experimental period, we increased the silica exposure dose to rapidly induce lung tissue lesions, and a pulmonary fibrosis model was established by intratracheal instillation of silica (80 mg/mL, 100 µL per mouse) as described above, followed by daily via tail vein injection of ferritin (Sigma–Aldrich, F4503) at 1000 ng/(mL.blood), corresponding to the average serum ferritin concentration observed in silica‐induced mice, to establish a systemic high ferritin environment. Animals were split into control, silica, and silica + ferritin co‐exposure groups (n = 5 per group). In addition, a separate ferritin‐only control group was included to assess potential physiological effects of high ferritin levels. After 7 days, mice were sacrificed, and lung tissues were collected for histological and molecular analyses.

For in vivo macrophage depletion experiments, mice were randomly assigned into four groups: control (Saline), silica, silica + liposome control (Silica + Lipo‐PBS), and silica + Clodronate liposomes (Silica + Lipo‐CTL), with 6–8 mice per group. Mice received tail‐vein injections of liposomes with or without clodronate (YEASEN, 40337ES10) at a concentration of 5 mg/mL (200 µL per mouse). On the day following the first liposome injection, pulmonary fibrosis was induced by intratracheal instillation of silica as described above. Thereafter, mice continued to receive tail‐vein injections of clodronate liposomes every other day, whereas mice in the Silica + Lipo‐PBS group were injected with liposomes without clodronate. After 7 days of treatment, mice were euthanized, and lung tissues were collected for subsequent analyses.

For confirmatory experiments, a recombinant adeno‐associated virus serotype 6 (AAV6) vector carrying shRNA against the mouse *Ftl1* gene (5’‐CTCTGGGCGAGTATCTCTTTG‐3’) under the control of the F4/80 promoter (AAV6‐F4/80‐*shFtl1*, Hanbio, Shanghai, China) was established. Then, 2.5 ×10^11^ vg 100 µL of virus were intratracheal instillation into the lung to generate macrophage‐specific ferritin knockdown mice. Two weeks post‐infection, lung tissues were collected to assess transduction efficiency and ferritin expression. Subsequently, mice were randomly assigned to control and treatment groups and received silica intratracheal instillation (80 mg/mL, 100 µL per mouse). After 4 weeks of exposure, lung tissues were harvested for downstream analyses.

### 4.3 Histopathological Staining

Lung tissues were fixed in 4% paraformaldehyde for at least 24 h, then placed in tissue cassettes, rinsed under tap water overnight, and processed through graded dehydration using an automatic tissue processor (Leica, ASP6025). Subsequently, tissues were then sectioned at 3–5 µm, floated in a 45°C water bath, and mounted on adhesive glass slides. After dewaxing and rehydration, sections were stained as required. Subsequently, Hematoxylin‐Eosin (H&E) staining was performed using a kit (MCE, HY‐K0315) to evaluate lung histopathology. Masson's trichrome staining (MCE, HY‐K0315) was used to assess collagen deposition, and collagen volume fraction was quantified using ImageJ software. For immunohistochemical staining, dewaxed and rehydrated sections underwent antigen retrieval, and endogenous peroxidase activity was blocked. After blocking with goat serum, sections were incubated with ferritin primary antibody (1:2000, HUABIO, R1601‐9) overnight at 4°C. Next, sections were incubated with a corresponding biotin‐labeled secondary antibody after rewarming, washed, and differentiated with DAB chromogen, followed by hematoxylin counterstaining of nuclei. Finally, slides were scanned using a whole‐slide imaging system (3DHISTECH, Pannoramic MIDI), and ferritin expression was quantified using the Image J IHC profiler plugin.

### 4.4 Hydroxyproline Content Detection

Approximately 10 mg of lung tissue was weighed and hydrolyzed in 6 mol/L hydrochloric acid at 95°C for 6 h. Hydroxyproline levels were subsequently quantified using a commercial hydroxyproline assay kit (Elabscience, E‐BC‐K062‐M) following the manufacturer's protocol.

### 4.5 Serum Ferritin Measurement

The concentration of ferritin in human serum was determined using the QuicKey Pro Human FE(Ferritin) ELISA Kit (Elabscience, E‐OSEL‐H0003). Ferritin levels in mouse serum were assessed using the Mouse FE(Ferritin) ELISA Kit (Elabscience, E‐EL‐M0491c), and the detection procedures were all carried out in accordance with the instructions of the manufacturer.

### 4.6 Spatial Transcriptomics Sequencing

Lung tissues from mice after 12 weeks of silica exposure were collected, and immediately embedded in OCT frozen compound and rapidly frozen on dry ice. Then, H&E staining and spatial transcriptomics sequencing of frozen sections were performed by LC‐Biotechnology (Hangzhou, China). Visualization of Spatial transcriptomics sequencing data was performed using the OmicStudio platform (https://www.omicstudio.cn/).

### 4.7 Cell Culture and Treatment

Human monocytic leukemia cells (THP‐1, RRID: CVCL_0006) and mouse mononuclear macrophage leukemia cells (RAW 264.7, RRID: CVCL_0493) were purchased from ATCC (TIB‐71, TIB‐202), and human pulmonary fibroblasts (HPFs, RRID: CVCL_6885) were purchased from Shanghai Zhong Qiao Xin Zhou Biotechnology Co., Ltd (PRI‐H‐00016). THP‐1 cells were cultured in RPMI‐1640 complete medium (Procell, CM‐023), RAW 264.7 cells were cultured in DMEM medium (Gibco, 11965092) containing 10% FBS, and HPFs were cultured in DMEM medium with 2% FBS, 1% penicillin‐streptomycin, and 1% GlutaMAX (Thermo Fisher, 35050061). Prior to experiments, all cell lines were tested for mycoplasma contamination using the MycoBlue Mycoplasma Detector (Vazyme, D101‐01), and the results were all negative (data not shown).

For molecular analyses, THP‐1 cells were seeded into 6‐well plates at a density of 1 × 10^6^ cells per well and differentiated into macrophages with 20 nM phorbol 12 myristate 13 acetate (PMA, Sigma–Aldrich, p8139) for 24 h. RAW 264.7 were seeded at the density of 5 × 10^5^ cells per well and cultured overnight to allow complete adherence. Subsequently, both THP‐1‐derived macrophages and RAW 264.7 cells were then treated with 0, 100, 200, 400 µg/mL silica for 48 h, after which proteins were extracted. HPFs were seeded in 6‐well plates at 2 × 10^5^ cells per well, and then stimulated with 0, 250, 500, 1000 ng/mL ferritin (Sigma, F6754). After 48 h of treatment, RNA and protein were extracted for detection. For co‐culture experiments, a non‐contact co‐culture chamber (particle size 1 µm) was used. THP‐1 cells were placed in the upper chamber and differentiated into macrophages, which were subsequently treated with silica. The chambers were then transferred onto 6‐well plates seeded with HPFs. After 48 h of co‐culture, RNA and protein were extracted from HPFs.

### 4.8 Transcriptomic Sequencing

RNA sequencing was performed on lung tissues from mice co‐exposed to silica and ferritin, as well as on HPFs treated with 1000 ng/mL ferritin. Total RNA was extracted using Trizol reagent (Invitrogen, 15596018CN). Then. RNA sequencing was performed by LC‐Biotechnology. For data analysis, StringTe was used to quantify gene expression, while GO and KEGG databases were employed for enrichment analysis. Data visualization was carried out on the OmicStudio platform (https://www.omicstudio.cn/).

### 4.9 Immunofluorescence Staining

Sterile glass coverslips were pre‐placed into 24‐well plates, and HPFs were seeded at a density of 1 × 10^5^ cells per well and cultured overnight to allow adhesion. Cells were then treated with 0 or 1000 ng/mL ferritin for 48 h. After treatment, HPFs were washed with PBS and fixed with acetone and methanol (1:1 mixed) at room temperature for 15 min. Next, HPFs were blocked with 2% BSA for 2 h and incubated overnight at 4°C with primary antibodies against α‐SMA (1:600, Abcam, ab5694), Collagen I (1:400, Abcam, ab270993), Collagen III (1:400, Abcam, ab184993), and fibronectin (1:600, Abcam, ab45688), respectively. On the following day, cells were washed with PBST (PBS + 0.1% Tween‐20) and incubated with the corresponding fluorescent secondary antibody at room temperature for 1.5 h in the dark. Afterward, nuclei were counterstained with DAPI (Solarbio, C0060), and coverslips were mounted using an anti‐fade mounting medium. Finally, images were acquired using a laser confocal microscope (Nikon, A1+).

### 4.10 Single‐Cell RNA Sequencing

Lung tissues from silicosis patients and silica‐exposed mice were dissected into 1–2 mm^3^ pieces. A mixture of collagenase I, collagenase II, and neutral protease (1:1:1 ratio) was added for enzymatic digestion in a 37 °C water bath for 5 min, and the reaction was terminated by adding complete medium. The resulting suspension was filtered through a 70 µm cell strainer, and residual tissue was further dissociated with a fresh enzyme mixture until no additional cells were released. Subsequently, dead cells were removed using a Dead Cell Removal kit (Miltenyi Biotec company, 130‐090‐101), and cells were collected by centrifugation. Cell count, viability, aggregation rate, and nuclear ratio were assessed with an automated cell counter, and samples with cell viability >85%, aggregation rate <5%, and nuclear ratio >70% were considered qualified. The cell suspension was then adjusted to 700‐1200 cells/mL. Single‐cell suspension, gel beads containing barcodes, unique molecular identifiers (UMI), and poly‐dT primers were loaded into separate channels of a Chromium next Gem Chip g to generate microdroplets system containing single cells, barcodes, and reaction reagents via the 10× Genomics system. Transcripts from individual cells were reverse‐transcribed and amplified within the microdroplets to construct single‐cell cDNA libraries, which were sequenced on a Novaseq 6000 sequencing platform after passing quality control. Next, raw sequencing data were processed using Cell Ranger software for quality control and alignment to generate a gene expression matrix for each cell, followed by Seurat software for cell filtering, normalization, and clustering. Cell populations were annotated based on gene expression profiles using the CellMarker database (http://bio‐bigdata.hrbmu.edu.cn/CellMarker/), the lungmap database (https://www.lungmap.net/), the Mouse cell atlas database (https://bis.zju.edu.cn/MCA/), and relevant published literature. Cell atlas maps of silicosis patients and silica‐exposed mice were generated based on annotated clusters. Visualization of single‐cell RNA‐seq data was performed using the OmicStudio platform (https://www.omicstudio.cn/).

### 4.11 Multiplex Immunofluorescence Staining

Sections underwent antigen retrieval using EDTA and were incubated with 3% H_2_O_2_ at room temperature to block endogenous peroxidase activity. Then, the staining areas were outlined with a Pap Pen and blocked with 10% goat serum for 30 min. Subsequently, sections were incubated with an F4/80 primary antibody (1:800, Cell Signaling Technology, 70076) overnight at 4°C. The following day, sections were washed three times using TBST and incubated with a secondary antibody at room temperature. Next, tyramide signal amplification (TSA) solution was applied and stained in the dark for 10 min at room temperature. After three times washes with TBST, primary antibodies were eluted at 42°C for 20 min. Subsequently, sections were incubated with ferritin primary antibody (1:1000) and corresponding secondary antibody following the same procedures. Finally, nuclei were counterstained with DAPI, and sections were mounted with anti‐fade mounting medium. Ferritin and F4/80 expression and localization were visualized using a section scanning system.

### 4.12 Cell Transfection

Cell transfection was performed using Lipofectamine RNAiMAX (Invitrogen, 13778030) according to the manufacturer's protocol. In detail, 5 µL of RNAiMAX and 100 nM siRNA were each diluted in 250 µL of Opti‐MEM medium (Gibco, 31985062). After incubation for 5 min, the two solutions were mixed and then incubated for 10 min. Subsequently, replaced the culture medium with serum‐free medium in the cells to be transfected, and the prepared transfection mixture was added dropwise. Cells were then incubated in a humidified 5% CO_2_ incubator at 37°C for 6–8 h. Afterward, the culture medium was changed to fresh medium, and the cells were harvested for downstream analyses 48 h post‐transfection. The siRNA sequences used in this study are listed in Table S1.

### 4.13 PIK3R2 Knockdown and Rescue Experiments

HPFs were seeded at 2 × 10^5^ cells per well in 6‐well plates and allowed to attach overnight. Cells were transfected with *PIK3R2*‐targeting siRNA using RNAiMAX and stimulated with ferritin for 48 h. mRNA and protein were subsequently collected for analysis. For rescue experiments, *PIK3R2*‐knockdown cells were treated with 3 µm 740 Y‐P (MCE, HY‐P0175) to upregulate PIK3R2, concurrently stimulated with 1000 ng/mL ferritin for 48 h, and proteins were collected for detection.

### 4.14 Quantitative Reverse Transcription Polymerase Chain Reaction (RT‐qPCR)

Total RNA was extracted from mouse lung tissues using the Animal Total RNA Isolation Kit (FOREGENE, RE‐03011) and from cultured cells using the Cell Total RNA Isolation Kit (FOREGENE, RE‐03113), following the manufacturer's instructions. RNA concentration and purity were measured using a nucleic acid spectrophotometer (Theromo, nanodrop 2000). Subsequently, applying ABScript III RT Master Mix for qPCR with gDNA Remover (ABclonal Technology, RK20429) to the synthesized complementary DNA (cDNA). And then, the qPCR reaction was prepared using PCR‐AceQ qPCR SYBR Green Master Mix (Vazyme, Q712‐1) on a real‐time fluorescent quantitative PCR system (Thermo Fisher, Quantstudio3). Primer sequences used in this study are listed in Table S2. Finally, Relative mRNA expression levels were normalized to *β‐actin* as the internal reference gene and calculated using the 2^‐△△CT^ method.

### 4.15 Western Blot

Protein extraction and detection from tissues and cells were performed as our previously described [57]. Protein concentration was determined using BSA as a standard. Then, equal amounts of protein were mixed with 1×SDS loading buffer and boiled at 100°C for 5 min. Subsequently, proteins were separated on 10% SDS‐PAGE gels and transferred to PVDF membranes. Next, membranes were blocked with sealing fluid (Epizyme, PS108P) for 30 min and incubated overnight at 4°C with the following primary antibodies: rabbit anti‐Ferritin (1:1000), rabbit anti‐α‐SMA (1:5000), rabbit anti‐Collagen I (1:1000), rabbit anti‐Collagen III (1:1000), rabbit anti‐Fibronectin (1:1000), rabbit anti‐PIK3R2 (1:500, Invitrogen, PA5‐84807), rabbit anti‐SMAD2 (1:1000, HUBIO, ET1604‐22), rabbit anti‐SMAD3 (1:1000, HUBIO, ET1607‐41), rabbit anti‐p‐SMAD2 (1:1000, HUBIO, ET1702‐34), rabbit anti‐p‐SMAD3 (1:1000, HUBIO, ET1609‐41), PI3K (HUBIO, HA601206), AKT (HUBIO, ET1609‐47), p‐AKT (HUBIO, ET1607‐73). On the second day, membranes were washed with TBST (TBS + 0.1% Tween‐20) and incubated with HRP‐Conjugated Goat anti‐Rabbit IgG (1:25000, HUBIO, HA1001) or HRP‐Conjugated Goat anti‐Mouse IgG (1:25000, HUBIO, HA1006) at room temperature for 1 h. Finally, Protein bands were visualized using an optical imager (Thermo Fisher, iBright CL750), and relative protein expression levels were quantified using iBright analysis software, with β‐actin as the internal reference control.

### 4.16 Statistical Analysis

Experimental results were presented as mean ± standard deviation (SD). GraphPad Prism 9.5.0 software and Adobe Photoshop 2021 software were used for figure preparation. The number of biologically independent samples (n) was indicated in each figure legend. Statistical analyses were performed with SPSS Statistics 26.0 software, and Student's *t*‐test was used for comparison between two groups, and one‐way analysis of variance (ANOVA) was utilized for comparison among multiple groups. For data with homogeneous variance, Dunnett's test and the least significant difference (LSD) test were used for multiple comparisons. For data with heterogeneous variance, Dunnett's T3 test was employed for multiple comparisons. The two‐sided confidence level was set at 0.05. A *p*‐value < 0.05 was considered statistically significant, with significance levels further categorized as follows: ^*^ *p* < 0.05, ^**^ *p* < 0.01, and ^***^ *p* < 0.001.

## Author Contributions

L.W. did conceptualization, investigation, funding acquisition, and Writing – original draft. X.C. did the investigation, methodology, and Writing – original draft. H.Q. and Q.R. did data curation and methodology. S.G., Y.G., and A.A. did formal analysis, visualization, and investigation. Q. H., M.Z., Q.Z., and L.P. did resources. L.Z. did funding acquisition. X.S. and B.Z. did supervision. Y.Y. did project administration, Writing – review & editing, and funding acquisition.

## Ethics Statement

All experimental protocols met the requirements of the National Institutes of Health guidelines. The use of human lung tissues and serum samples was reviewed and approved by the Medical Ethics Committee of West China Fourth University Hospital, Sichuan University (Approval Numbers: EC‐2022040, EC‐2022105). Animal experiments were approved by the Ethics Committee of West China School of Public Health/West China Fourth Hospital, Sichuan University (Approval Numbers: Gwll2023128, Gwll2023233).

## Conflicts of Interest

The authors declare no conflict of interest.

## Supporting information




**Supporting File 1**: advs73867‐sup‐0001‐SuppMat.docx.

**Supporting File 2**: advs73867‐sup‐0002‐CellLines.docx.

**Supporting File 3**: advs73867‐sup‐0003‐SupportingFiguresData.zip.

**Supporting File 4**: advs73867‐sup‐0001‐FiguresData.zip.

## Data Availability

The data that support this study were present in the article and supplementary information, and from the corresponding author on reasonable request.
