# Supplementary material for: Macrophage–Derived Ferritin Exacerbates Silica‐Induced Pulmonary Fibrosis via PIK3R2‐Mediated Fibroblast Differentiation
Source: Adv Sci (Weinh). 2026 Jan 21;13(17):e19191. doi: 10.1002/advs.202519191 (PMC13042690; doi:10.1002/advs.202519191)
Supplement: Supplementary file 1 — Supporting File 1: advs73867‐sup‐0001‐SuppMat.docx. [file ADVS-13-e19191-s002.docx]

Supporting Information

**Macrophage-Derived Ferritin Exacerbates Silica-Induced Pulmonary Fibrosis via PIK3R2-Mediated Fibroblast Differentiation**

*Liqun Wang, Xuxi Chen, Hongying Quan, Rui Qian, Shuyu Gong, Qiurong He, Ying Gao, Ajia Axi, Manyu Zhao, Qin Zhao, Ling Zhang, Lijun Peng, Xin Sun, Ben Zhang, Yuqin Yao**


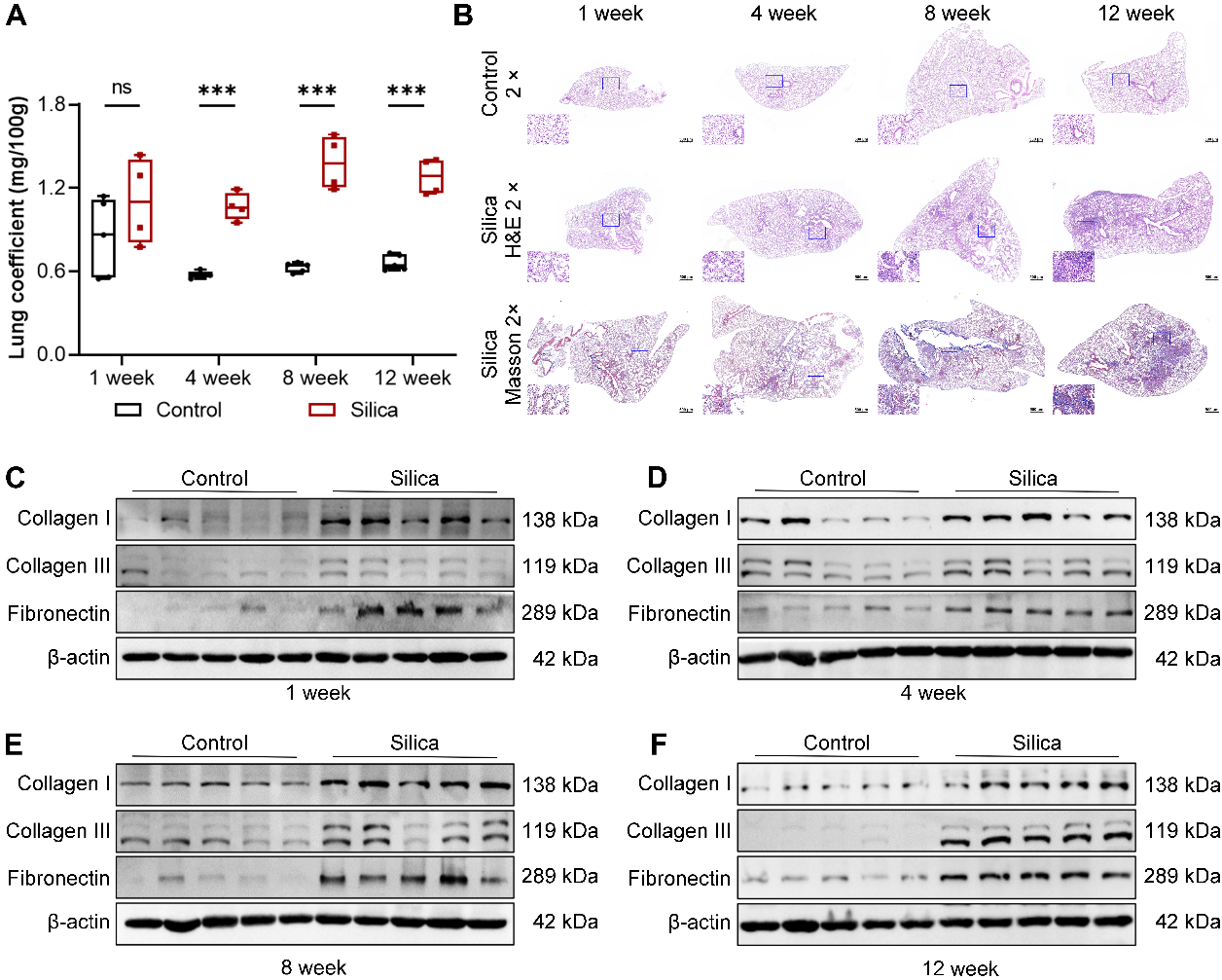
**Supplementary Figures S1 to S9**

**FIGURE S1. Silica exposure induces pulmonary fibrosis and ECM deposition in mice.** A) Lung coefficients in mice at 1, 4, 8, and 12 weeks after silica exposure (n=5 per group). B) Representative H&E and Masson’s trichrome staining of lung tissue from silica-exposed mice at different time points (magnification, 2×, 20×). C-F) Protein expression levels of Collagen I, Collagen III, and fibronectin in lung tissue from silica-exposed mice at indicated time points detected by Western blot. Data are presented as mean ± SD, ****p*＜0.001. Statistical analysis was performed using a two-tailed Student’s *t*-test (A).


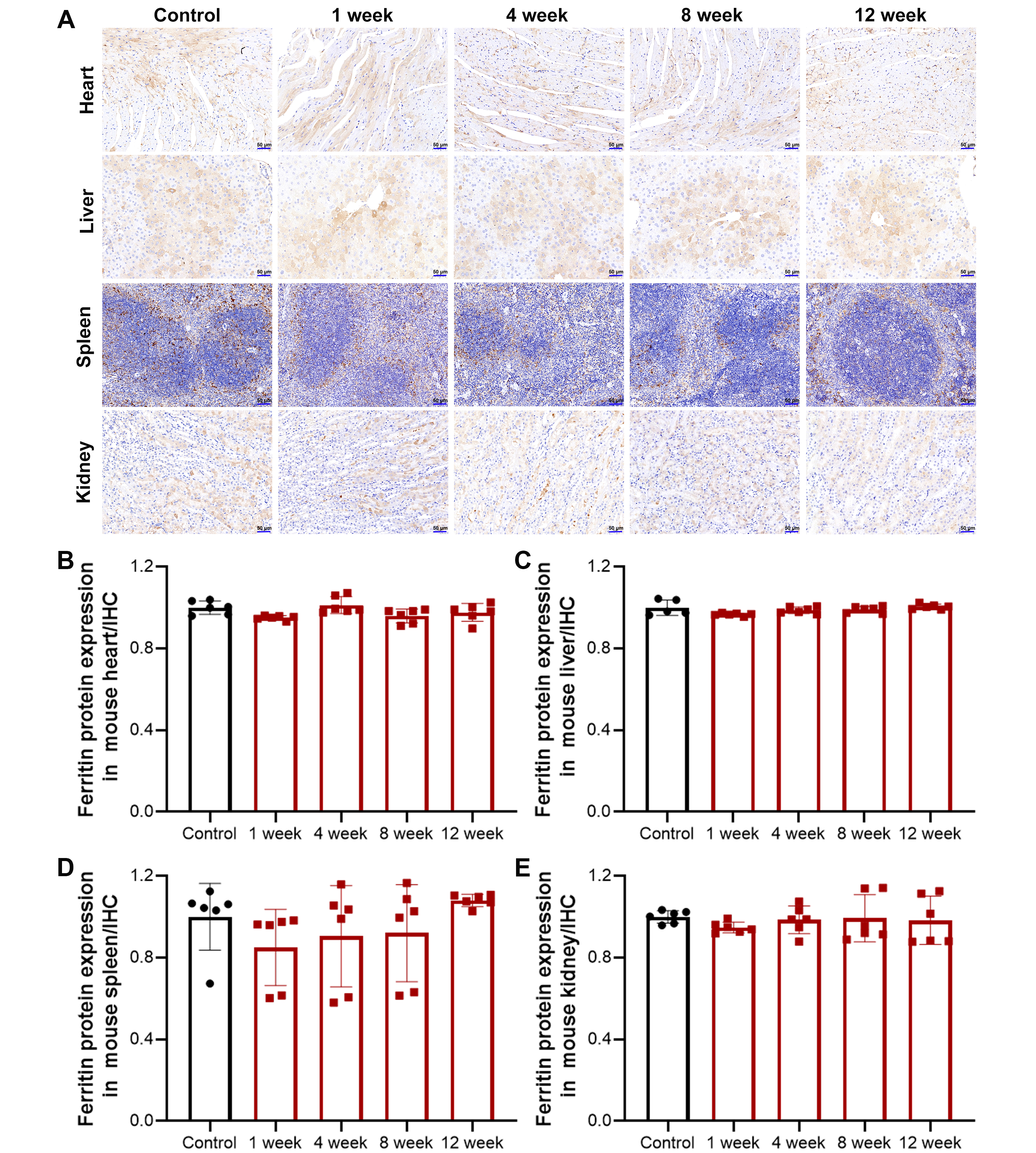
**FIGURE S2. Ferritin expression in major organs of silica-exposed mice.** A) Representative immunohistochemical staining of heart, liver, spleen, and kidney from silica-exposed mice (magnification, 20×). B) Quantitative analysis of ferritin expression in mouse heart tissue based on immunohistochemical staining (n=5 or 6 per group). C) Quantitative analysis of ferritin expression in mouse liver tissue based on immunohistochemical staining (n=6 per group). D) Quantitative analysis of ferritin expression in mouse spleen tissue based on immunohistochemical staining (n=6 per group). E) Quantitative analysis of ferritin expression in mouse kidney tissue based on immunohistochemical staining (n=6 per group). Data are presented as mean ± SD. Statistical analysis was performed using one-way ANOVA followed by Dunnett's T3 test (B-E).

**
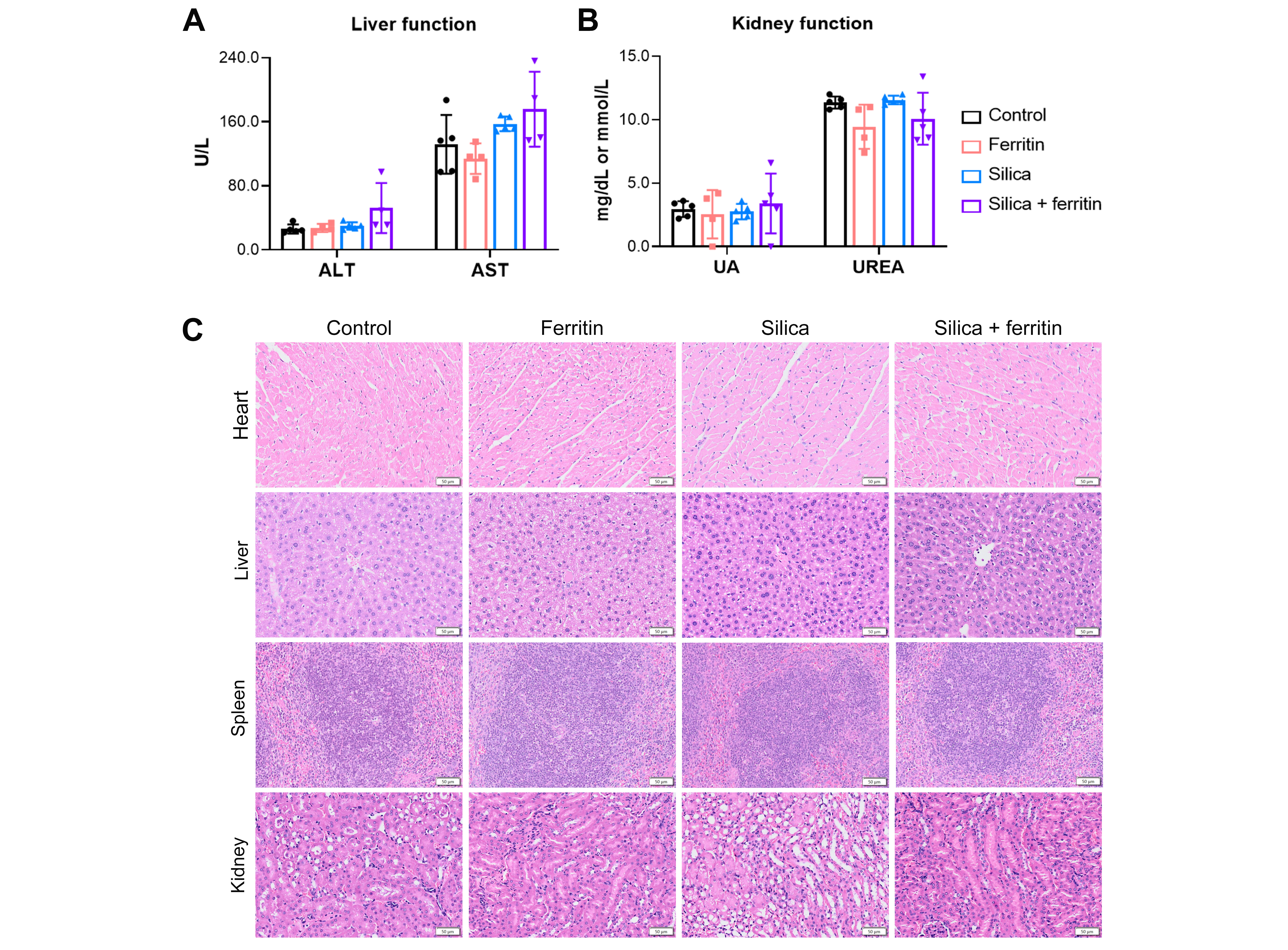
FIGURE S3. Ferritin administration does not impair physiological functions in normal mice.** A) Serum aspartate aminotransferase (AST) and alanine aminotransferase (ALT) levels in mice co-exposed to silica and ferritin (n=4 or 5 per group). B) Serum uric acid (UA) and urea (UREA) levels in co-exposed mice (n=4 or 5 per group). C) Representative H&E staining of heart, liver, spleen, and kidney tissues from mice treated with silica and ferritin (magnification, 10×). Data are presented as mean ± SD. Statistical analysis was performed using one-way ANOVA followed by Dunnett's T3 test (A and B).

**
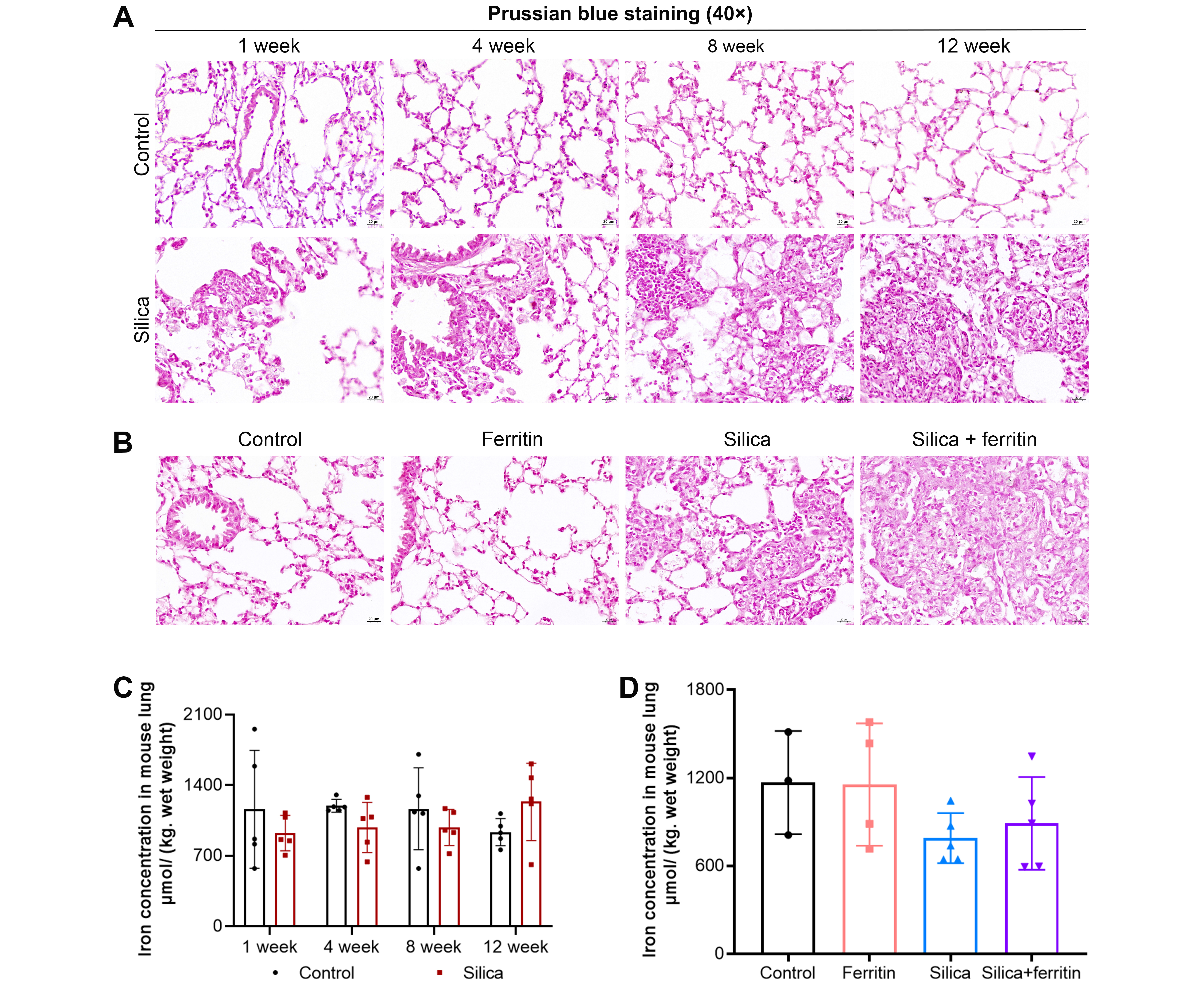
FIGURE S4. Silica and/or ferritin exposure does not disrupt pulmonary iron homeostasis in mice.** A) Representative Prussian blue staining of lung tissue after 1, 4, 8, and 12 weeks of silica exposure (magnification, 40×). B) Representative Prussian blue staining of lung tissue from silica and ferritin co-exposed mice (magnification, 40×). C) Total iron concentrations in lung tissues after silica exposure for 1, 4, 8, and 12 weeks, measured by ELISA (n=5 per group). D) Total iron concentrations in lung tissues from silica and ferritin co-exposed mice (n=3~5 per group). Data are presented as mean ± SD. Statistical analysis was performed using two-tailed Student’s *t*-test (C) and one-way ANOVA followed by Dunnett's test (D).

**
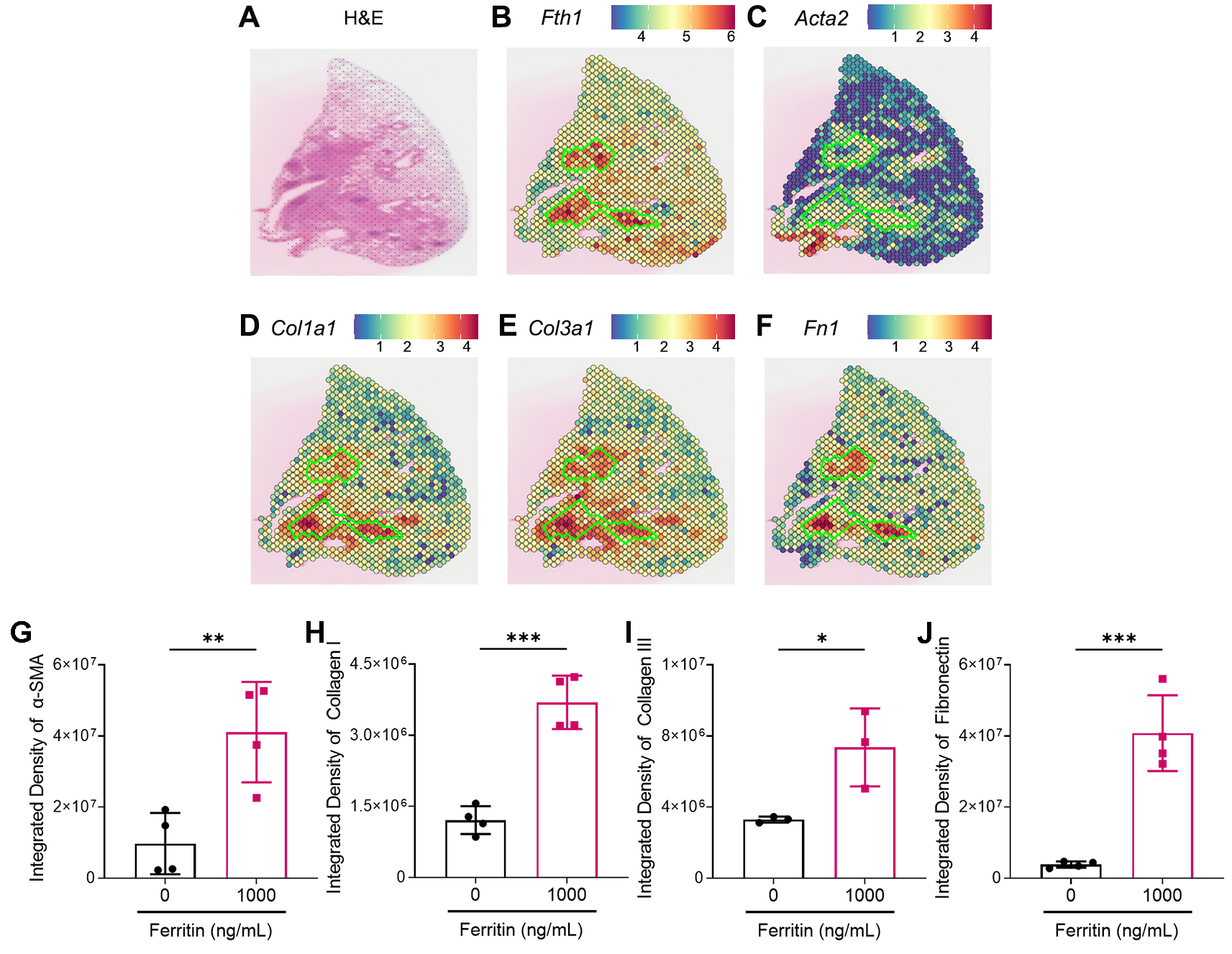
FIGURE S5.** **Ferritin promoted fibroblast differentiation.** A) Representative H&E staining of mouse lung tissue after 12 weeks of silica exposure. B) Spatial localization of *Fth1* in fibrotic lung tissue. C) Spatial localization of the myofibroblast marker *Acta2* in fibrotic lung tissue. (D-F) Spatial localization of ECM-associated components *Col1a1*, *Col3a1*, and *Fn1* in fibrotic lung tissue. G) Quantitative analysis of α-SMA fluorescence intensity in ferritin-stimulated HPFs (n=4 per group). H) Quantitative analysis of Collagen I fluorescence intensity in ferritin-stimulated HPFs (n=4 per group). I) Quantitative analysis of Collagen III fluorescence intensity in ferritin-stimulated HPFs (n=3 per group). J) Quantitative analysis of fibronectin fluorescence intensity in ferritin-stimulated HPFs (n=4 per group). Data are presented as mean ± SD, **p*＜0.05, ***p*＜0.01, ****p*＜0.001. Statistical analysis was performed using two-tailed Student’s *t*-test (G-J).

**
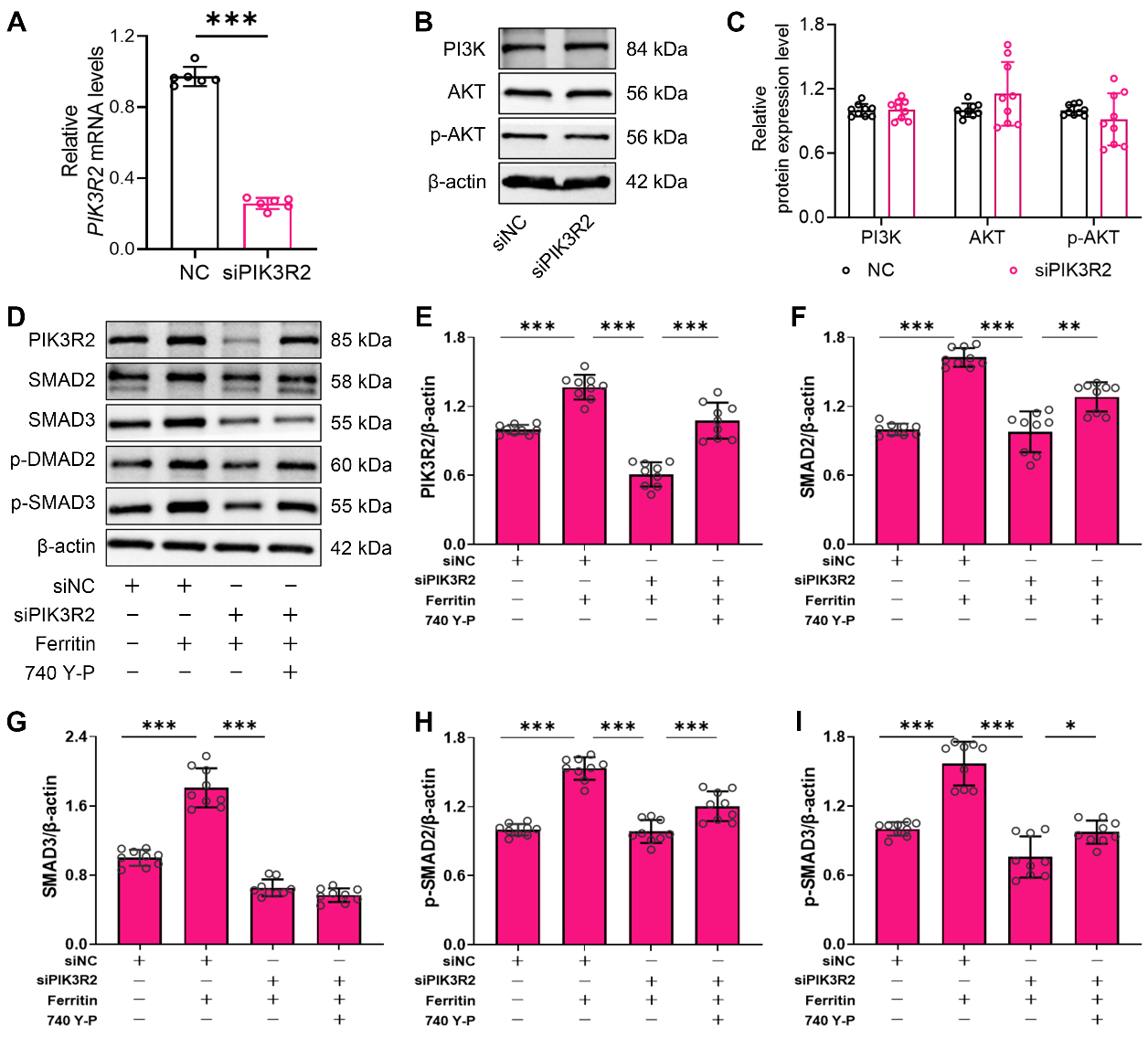
FIGURE S6. PIK3R2 overexpression restores SMADs signaling in ferritin-induced fibroblasts.** A) *PIK3R2* mRNA expression in HPFs after *siPIK3R2* transfection for 48 h, as determined by RT-qPCR (n=3 per group). B) Protein expression levels of PI3K/AKT pathway components in *PIK3R2*-knockdowned HPFs. C) Quantitative analysis of PI3K, AKT, p-AKT proteins in *PIK3R2*-knockdowned HPFs (n=3 per group). D) Protein expression levels of SMADs pathway components in *PIK3R2*-knockdowned HPFs stimulated with the PIK3R2 agonist 740 Y-P. E-I) Quantitative analysis of SMADs pathway proteins in *PIK3R2*-knockdowned HPFs stimulated with the PIK3R2 agonist 740 Y-P (n=3 per group). Data are presented as mean ± SD, ***p*＜0.01, ****p*＜0.001. Statistical analysis was performed using two-tailed Student’s *t*-test (A and C) and one-way ANOVA followed by Dunnett’s test, LSD (H) and Dunnett's T3 test (E, F, G, I).

**
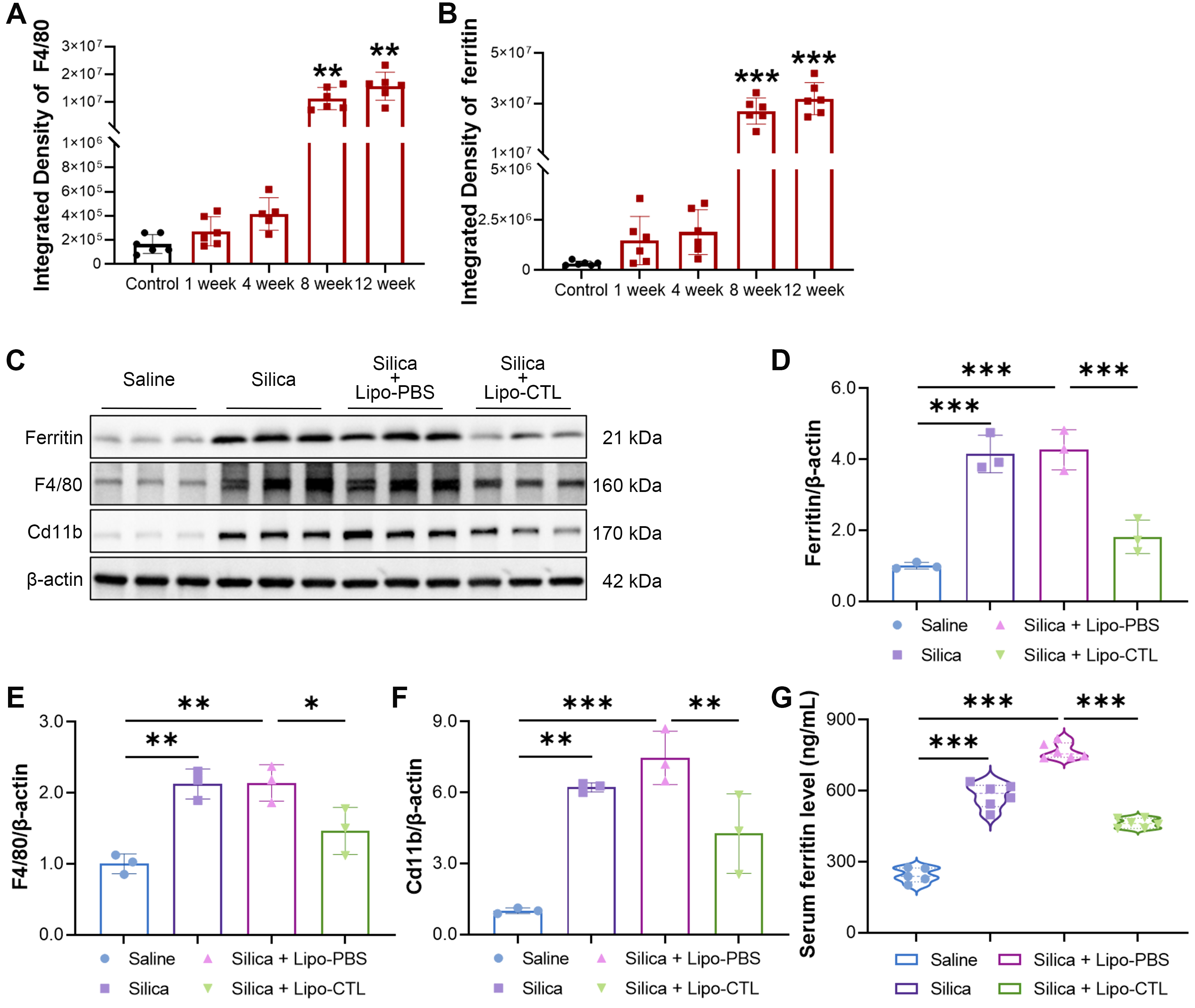
FIGURE S7. Macrophage depletion reduces ferritin accumulation in the lung and circulation.** A) Quantitative analysis of F4/80 fluorescence intensity in lung of silica-indued mice (n=5 or 6 per group). B) Quantitative analysis of F4/80 fluorescence intensity in lung of silica-indued mice (n= 6 per group). C) Expression of ferritin and macrophage markers in macrophage-depletion mice lung tissue evaluated by Western blot. D-G) Quantitative analysis of ferritin, F4/80 and Cd11b protein in lung tissue of macrophage-depletion silicosis mice (n=3 per group). G) Serum ferritin concentrations in macrophage-depleted silicosis mice (n=5 or 6 per group). Data are presented as mean ± SD, **p*＜0.05, ****p*＜0.001. Statistical analysis was performed using one-way ANOVA followed by LSD (D-G) and Dunnett's T3 test (A, B).

**
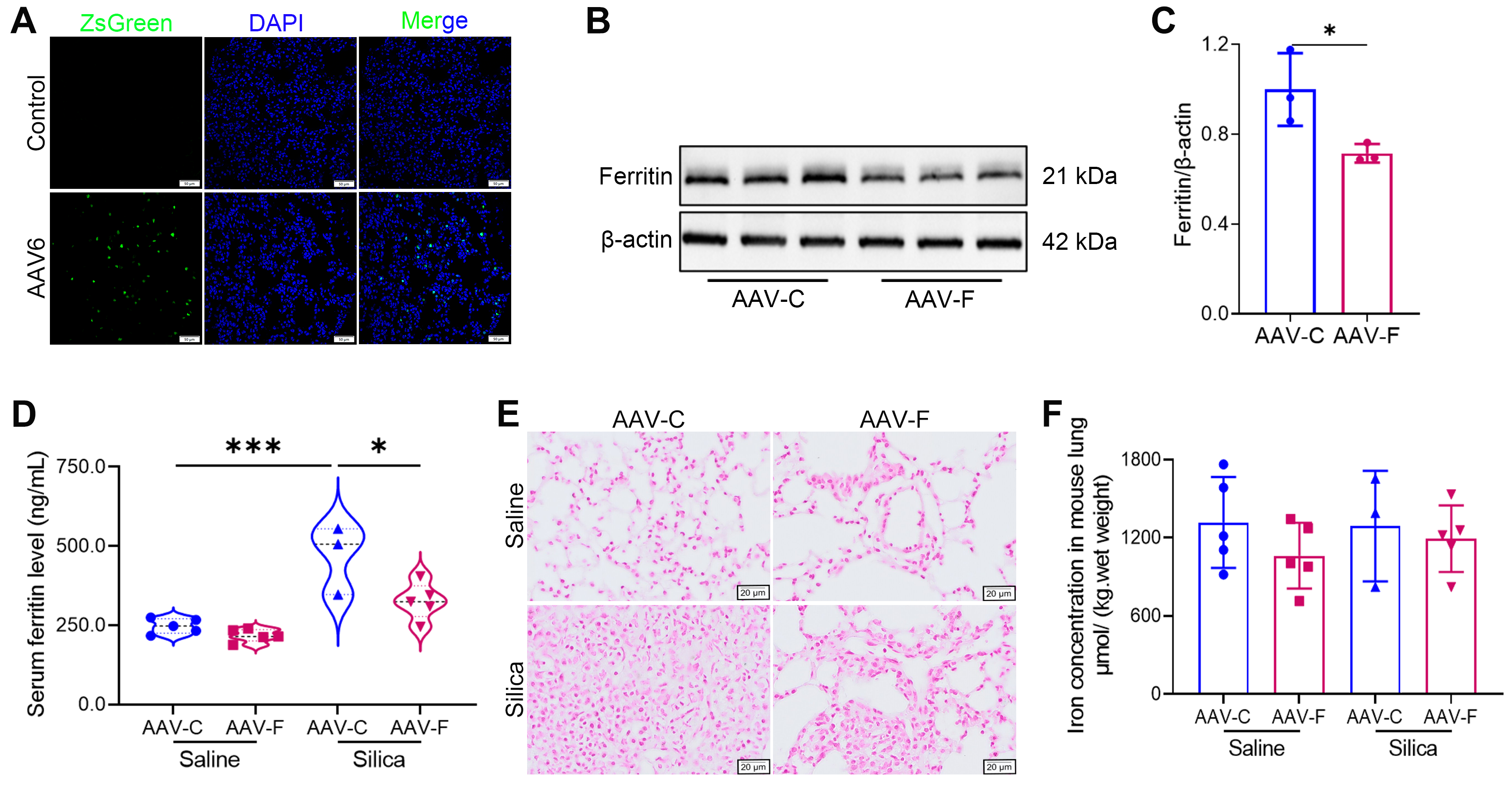
FIGURE S8. Construction of mice with low pulmonary ferritin expression.** A) Representative ZsGreen fluorescence in lung tissues from mice infected with AAV6-F4/80-control or AAV6-F4/80-*shFtl1* for two weeks, evaluating viral infection efficiency (magnification, 10×). B) Ferritin protein expression levels in lung tissues after 2 weeks AAV6 infection detecting by Western blot. C) Quantitative analysis of ferritin protein expression levels (n=3 per group). D) Serum ferritin levels measured by ELISA in silica-induced pulmonary fibrosis mice with or without ferritin knockdown (n=3 or 5 per group). E) Representative Prussian blue staining of lung tissues in silica-induced pulmonary fibrosis mice with or without ferritin knockdown (magnification, 40×). F) Total iron contents in lung tissues from silica and ferritin co-exposed mice (n=3 or 5 per group). Data are presented as mean ± SD, **p*＜0.05, ****p*＜0.001. Statistical analysis was performed using two-tailed Student’s *t*-test (C) and one-way ANOVA followed by LSD (D, F).

**
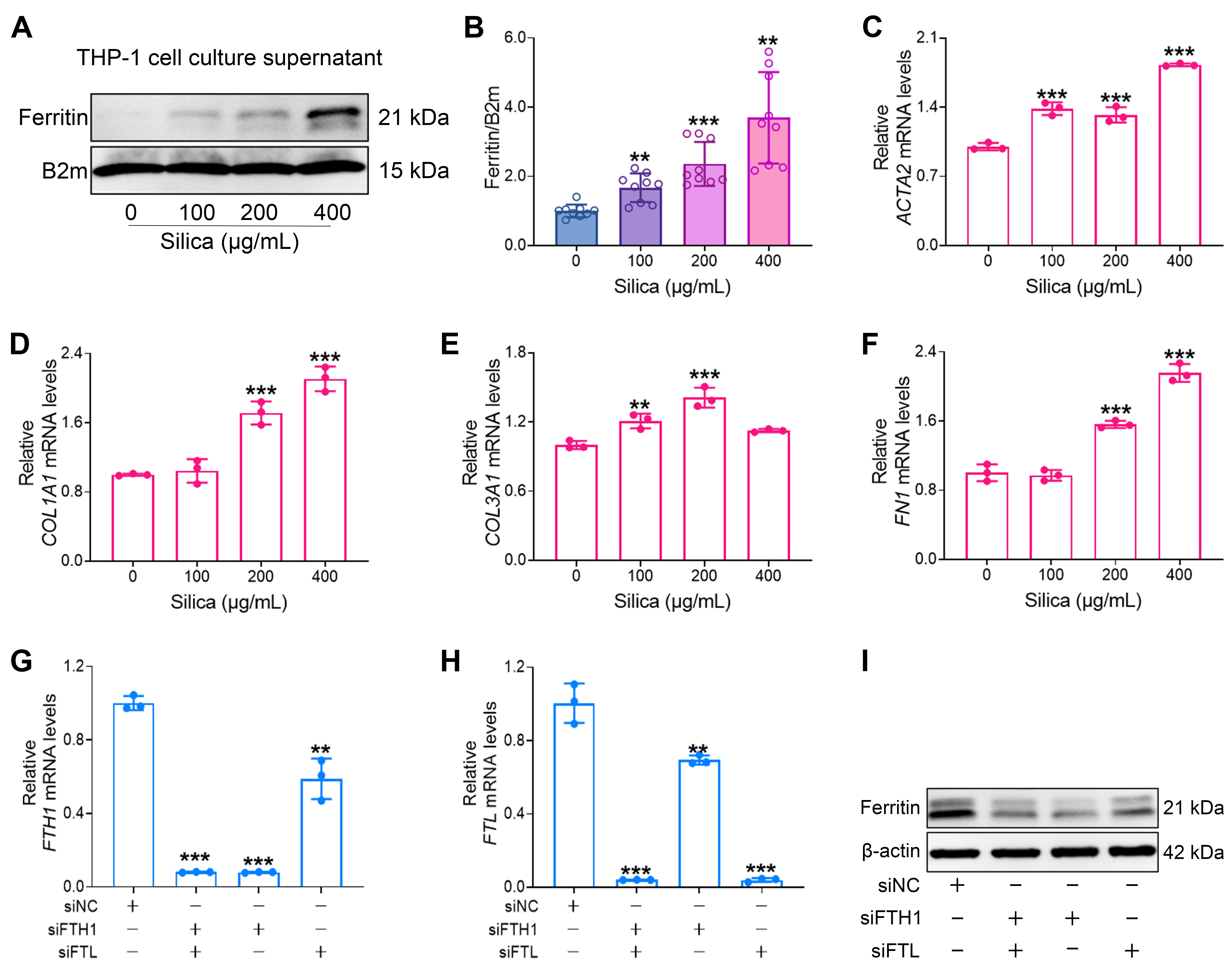
FIGURE S9. Co-culture with silica-treated macrophages facilitates ECM production in fibroblasts.** A) Ferritin protein expression in the supernatant of THP-1-derived macrophages after 48 h of stimulation with 0, 100, 200, and 400 μg/mL silica. B) Quantitative analysis of ferritin protein levels in the supernatant (n=3 per group). C-F) mRNA expression levels of *ACTA2*, *COL1A1*, *COL3A1*, and *FN1* in fibroblasts co-cultured with silica-treated macrophages (n=3 per group). G) *FTH1* mRNA expression in THP-1 cells after *FTH1* and *FTL* siRNA transfection, measured by RT-qPCR (n=3 per group). H) *FTL* mRNA expression in THP-1 cells under the same conditions (n=3 per group). I) Ferritin protein expression in THP-1 cells after *FTH1* and *FTL* siRNA transfection, detected by Western blot. Data are presented as mean ± SD, ***p*＜0.01, ****p*＜0.001. Statistical analysis was performed using one-way ANOVA followed by Dunnett’s test, LSD (C-F), or Dunnett's T3 test (B, G, H).

**Supplementary Tables S1 and S2**

**Table S1. siRNA sequences used in this study**

| **Target gene** | **5' to 3'** |
| --- | --- |
| Homo-*NC* | UUCUCCGAACGUGUCACGUTT |
|  | ACGUGACACGUUCGGAGAATT |
| Homo-*PIK3R2*  (Gene ID:5296) | GGCCAGACUCAAGAGAAAUTT |
|  | AUUUCUCUUGAGUCUGGCCTT |
| Homo-*FTH1*  (Gene ID:2495) | AGAUCAACCUGGAGCUCUATT |
|  | UAGAGCUCCAGGUUGAUCUTT |
| Homo-*FTL*  (Gene ID:2512) | GGCGAGUAUCUCUUCGAAATT |
|  | UUUCGAAGAGAUACUCGCCTT |

**Table S2. Primer sequences used in this study**

| **Gene name** | **5' to 3'** |
| --- | --- |
| Homo-*ACTA2*  (Gene ID: 59) | GATGGTGGGAATGGGACAAA |
|  | GCCATGTTCTATCGGGTACTTC |
| Homo-*COL1A1*  (Gene ID: 1277) | AGTGGTTTGGATGGTGCCAA |
|  | GCACCATCATTTCCACGAGC |
| Homo-*COL3A1*  (Gene ID: 1281) | CCTCATTAGTCCTGATGGTTCTC |
|  | GGTTAGGGTCAACCCAGTATTC |
| Homo-*FN1*  (Gene ID: 2335) | CTATCACCTGTACCCACACGG |
|  | CCAGGAACCCTGAACTGTAAGG |
| Homo-*PIK3R2*  (Gene ID: 5296) | CTAGCAAGATCCAGGGCGAG |
|  | ACAACGGAGCAGAAGGTGAG |
| Homo-*FTH1*  (Gene ID: 2495) | CAACGAGGTGGCCGAATCTT |
|  | AAGTCACACAAATGGGGGTC |
| Homo-*FTL*  (Gene ID: 2512) | ATGAGCTCCCAGATTCGTCAG |
|  | GCCCAGAGAGAGGTAGGTGT |
| mus-*Fth1*  (Gene ID: 14319) | AAGTGCGCCAGAACTACCAC |
|  | TCAGAGCCACATCATCTCGG |
| mus-*Ftl1*  (Gene ID: 14325) | TACACCTACCTCTCTCTGGGC |
|  | CCCGCGATCGTTCTGAAACT |
