# Supplementary material for: Macrophage–Derived Ferritin Exacerbates Silica‐Induced Pulmonary Fibrosis via PIK3R2‐Mediated Fibroblast Differentiation
Source: Adv Sci (Weinh). 2026 Jan 21;13(17):e19191. doi: 10.1002/advs.202519191 (PMC13042690; doi:10.1002/advs.202519191)
Supplement: Supplementary file 2 — Supporting File 2: advs73867‐sup‐0002‐CellLines.docx. [file ADVS-13-e19191-s004.docx]

**Supporting Information:
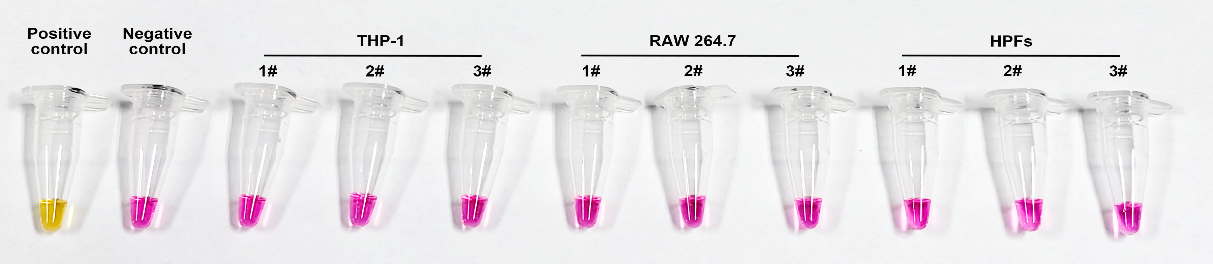
Cell lines mycoplasma test results**

**Assessment of mycoplasma contamination in cell lines.** Detection was performed using the MycoBlue Mycoplasma Detector (Vazyme, D101-01), with yellow indicating a positive result and pink indicating a negative result. All tested cell lines (THP-1, RAW 264.7, and HPFs) were confirmed to be free of mycoplasma contamination.
