# Supplementary material for: Macrophage–Derived Ferritin Exacerbates Silica‐Induced Pulmonary Fibrosis via PIK3R2‐Mediated Fibroblast Differentiation
Source: Adv Sci (Weinh). 2026 Jan 21;13(17):e19191. doi: 10.1002/advs.202519191 (PMC13042690; doi:10.1002/advs.202519191)
Supplement: Supplementary file 3 — Supporting File 3: advs73867‐sup‐0003‐SupportingFiguresData.zip. [file ADVS-13-e19191-s003.zip › Supporting information Figure S1-S9/S6/Figure S6D-I.pdf]

Figure S6D-I

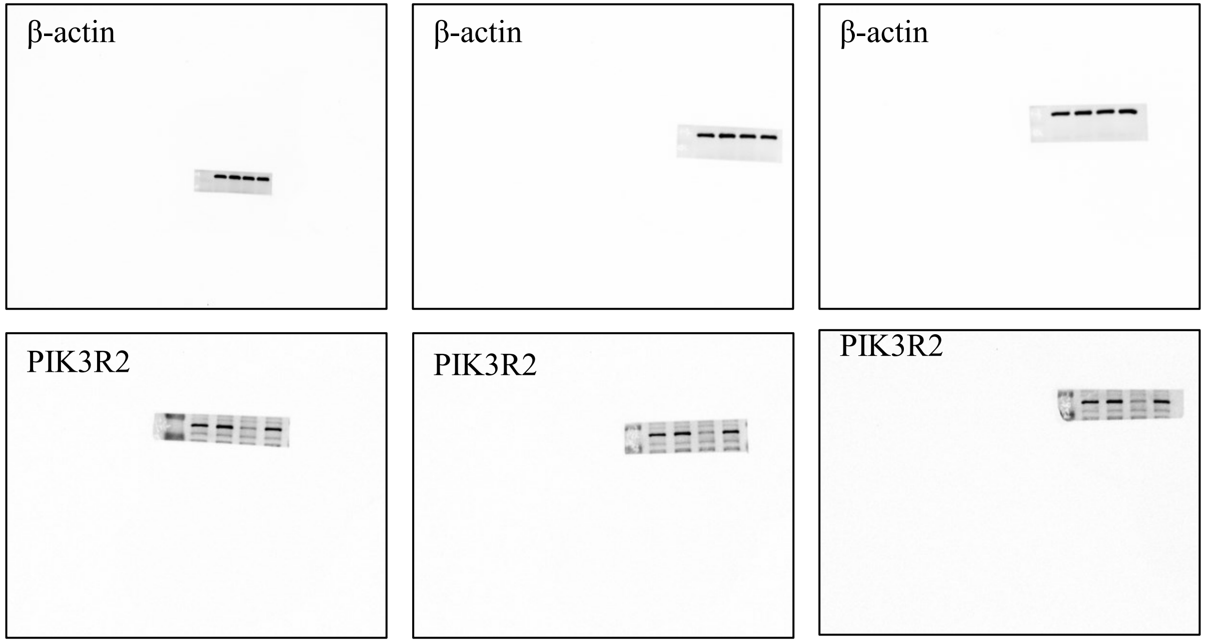

		actin	PIK3R2			PIK3R2/actin			Control me	relative expression		
1	**siNC**	6929200	1511539	1482222	1573678	0.21814	0.21391	0.227108	0.219719	0.992814	0.973558	1.033628
	**siNC+Ferritin**	7939218	2406526	2457619	2490317	0.303119	0.309554	0.313673	0.219719	1.379572	1.408862	1.427606
	**siPIK3R2+Ferritin**	7322214	810370	696649	821205	0.110673	0.095142	0.112153	0.219719	0.503701	0.433015	0.510435
	**siPIK3R2+Ferritin+740 YP**	7032902	1766237	1383156	1423955	0.251139	0.196669	0.20247	0.219719	1.142999	0.895093	0.921496
		actin	PIK3R2			PIK3R2/actin			Control me	relative expression		
2	**siNC**	6406203	1366422	1302948	1232862	0.213297	0.203388	0.192448	0.203044	1.050493	1.001694	0.947813
	**siNC+Ferritin**	7628987	1824397	1957047	2036875	0.23914	0.256528	0.266992	0.203044	1.177772	1.263407	1.314941
	**siPIK3R2+Ferritin**	6307040	846718	923367	923367	0.13425	0.146403	0.146403	0.203044	0.661184	0.721037	0.721037
	**siPIK3R2+Ferritin+740 YP**	5833102	1567331	1470580	1398807	0.268696	0.252109	0.239805	0.203044	1.323336	1.241646	1.181047
		actin	PIK3R2			PIK3R2/actin			Control me	relative expression		
3	**siNC**	7684516	983101	1045750	940129	0.127933	0.136085	0.122341	0.128786	0.993372	1.056676	0.949951
	**siNC+Ferritin**	8057858	1408166	1476346	1609435	0.174757	0.183218	0.199735	0.128786	1.356953	1.422653	1.550902
	**siPIK3R2+Ferritin**	8071602	613031	665437	732158	0.075949	0.082442	0.090708	0.128786	0.58973	0.640144	0.704329
	**siPIK3R2+Ferritin+740YP**	8739151	1210263	1126916	1022045	0.138487	0.12895	0.11695	0.128786	1.075328	1.001274	0.908095

Figure S6D-I

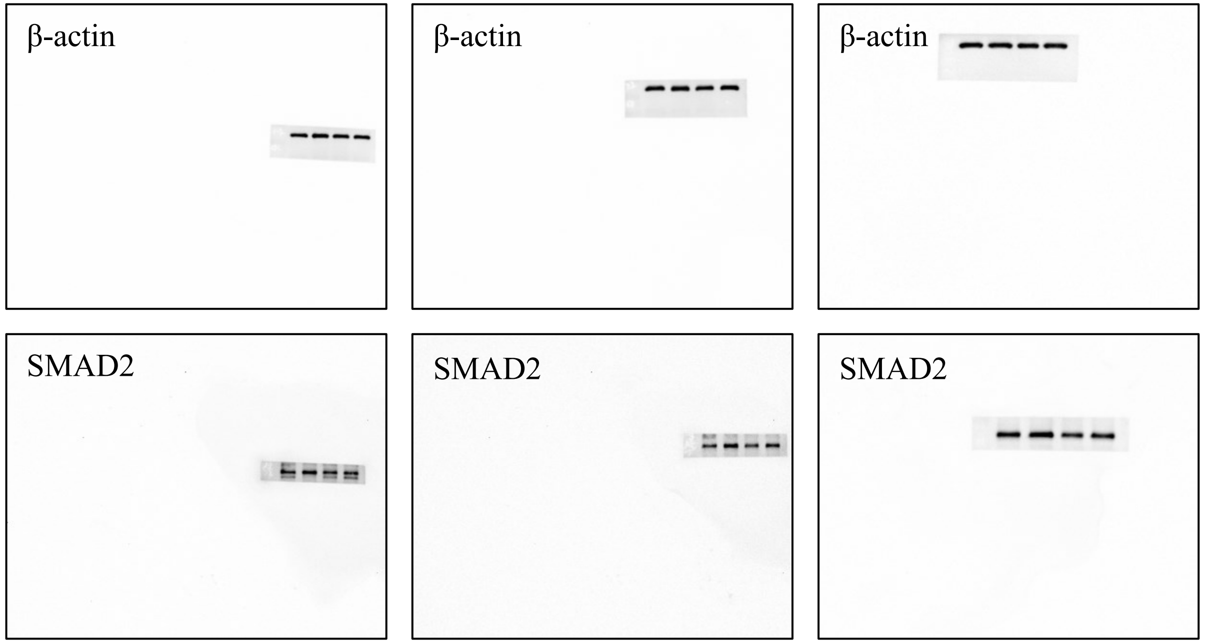

		actin	SMAD2			SMAD2/actin			Control mean	lative expression		
	**siNC**	6406203		1181924			0.184496807		0.168624743		1.094126545	
	**siNC+Ferritin**	7628987	2030776	2065238	2082422	0.266192091	0.270709335	0.272961797	0.168624743	1.578606356	1.605395097	1.618752932
1	**siPIK3R2+Ferritin**	6307040	1154008	1127736	1127736	0.18297141	0.178805906	0.178805906	0.168624743	1.085080438	1.060377634	1.060377634
	**siPIK3R2+Ferritin+740 YP**	5833102	1339304	1380946	1339304	0.229604077	0.236742989	0.229604077	0.168624743	1.36162744	1.403963526	1.36162744
		actin	SMAD2			SMAD2/actin			Control mean	lative expression		
	**siNC**	5486295		645356			0.117630569		0.113358286		1.037688309	
	**siNC+Ferritin**	5644070	1102186	1098541	1114751	0.195282128	0.194636317	0.197508358	0.113358286	1.722698306	1.717001232	1.742337191
2	**siPIK3R2+Ferritin**	4786759	620151	634857	565189	0.129555509	0.132627734	0.118073419	0.113358286	1.142885213	1.169987112	1.041594951
	**siPIK3R2+Ferritin+740 YP**	5607869	844021	866725	878056	0.150506547	0.154555144	0.156575697	0.113358286	1.327706618	1.363421667	1.381246157
		actin		SMAD2			SMAD2/actin		Control mean		relative expression	
	**siNC**	2476855	3378263	3378249	3142261	1.363932487	1.363926835	1.268649558	1.332169626	1.023842955	1.023838712	0.952318333
3	**siNC+Ferritin**	2011284	4108150	4227011	4110707	2.042550928	2.101648002	2.043822255	1.332169626	1.533251387	1.577612911	1.534205715
	**siPIK3R2+Ferritin**	2287688	2444945	2331561	2096009	1.06874058	1.019177877	0.916212788	1.332169626	0.802255628	0.76505113	0.687759854
	**siPIK3R2+Ferritin+740 YP**	2092656	3073789	3186505	3073789	1.468845811	1.522708462	1.468845811	1.332169626	1.102596683	1.14302896	1.102596683

Figure S6D-I

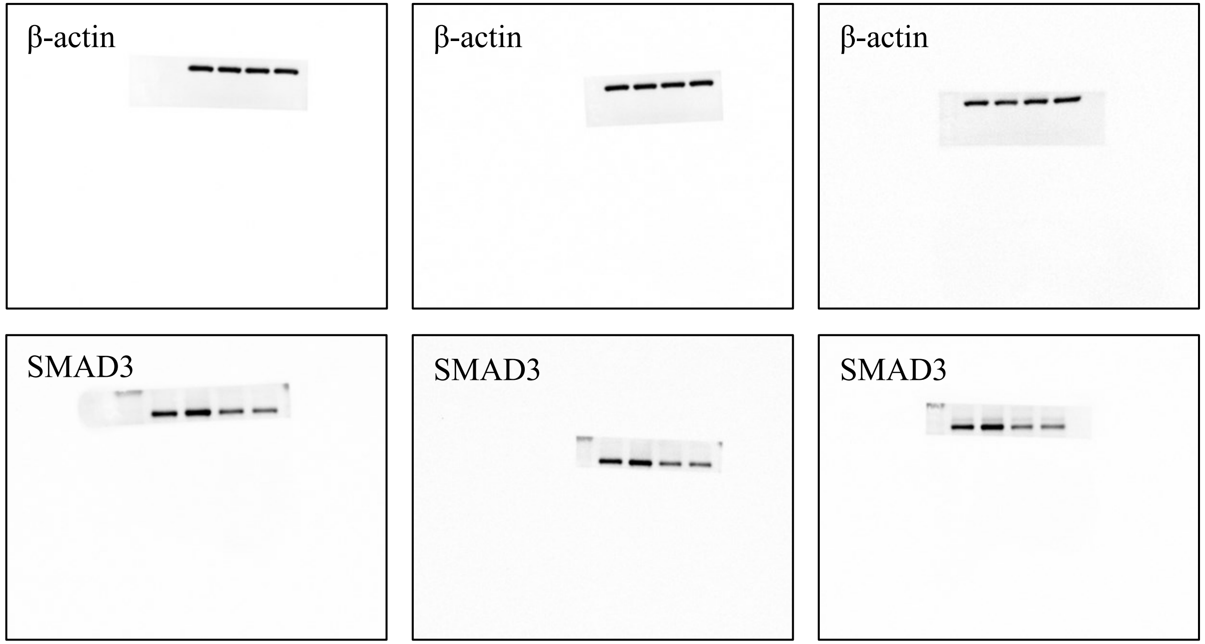

		actin	SMAD3			SMAD3/actin			Control me	relative expression		
1	**siNC**	5920124	3276030	3931049	4235693	0.553372	0.664015	0.715474	0.644287	0.858891	1.03062	1.11049
	**siNC+Ferritin**	5290346	5391109	5868993	6272089	1.019047	1.109378	1.185573	0.644287	1.581666	1.72187	1.840132
	**siPIK3R2+Ferritin**	5253599	2112888	2725861	2766704	0.402179	0.518856	0.52663	0.644287	0.624224	0.805318	0.817385
	**siPIK3R2+Ferritin+740 YP**	5376978	2222496	2195740	2368419	0.413336	0.408359	0.440474	0.644287	0.64154	0.633816	0.683661
		actin	SMAD3			SMAD3/actin			Control me	relative expression		
2	**siNC**	3008589	1531682	1433050	1329628	0.509103	0.47632	0.441944	0.475789	1.070019	1.001115	0.928866
	**siNC+Ferritin**	2804268	2208771	2228330	2076110	0.787646	0.794621	0.740339	0.475789	1.655453	1.670112	1.556025
	**siPIK3R2+Ferritin**	2840761	861102	806072	902701	0.303124	0.283752	0.317767	0.475789	0.637097	0.596382	0.667875
	**siPIK3R2+Ferritin+740 YP**	2981526	814466	787493	833162	0.273171	0.264124	0.279441	0.475789	0.574143	0.555129	0.587322
		actin	SMAD3			SMAD3/actin			Control me	relative expression		
3	**siNC**	909357	3531356	3253436	2810876	3.883355	3.577732	3.091059	3.517382	1.104047	1.017158	0.878795
	**siNC+Ferritin**	680292	4812603	5205895	4945367	7.07432	7.652442	7.269477	3.517382	2.011246	2.175607	2.066729
	**siPIK3R2+Ferritin**	892985	1874612	1691381	1854332	2.099265	1.894075	2.076554	3.517382	0.596826	0.53849	0.590369
	**siPIK3R2+Ferritin+740 YP**	1101040	1815880	1742012	2022227	1.649241	1.582151	1.836652	3.517382	0.468883	0.449809	0.522164

Figure S6D-I

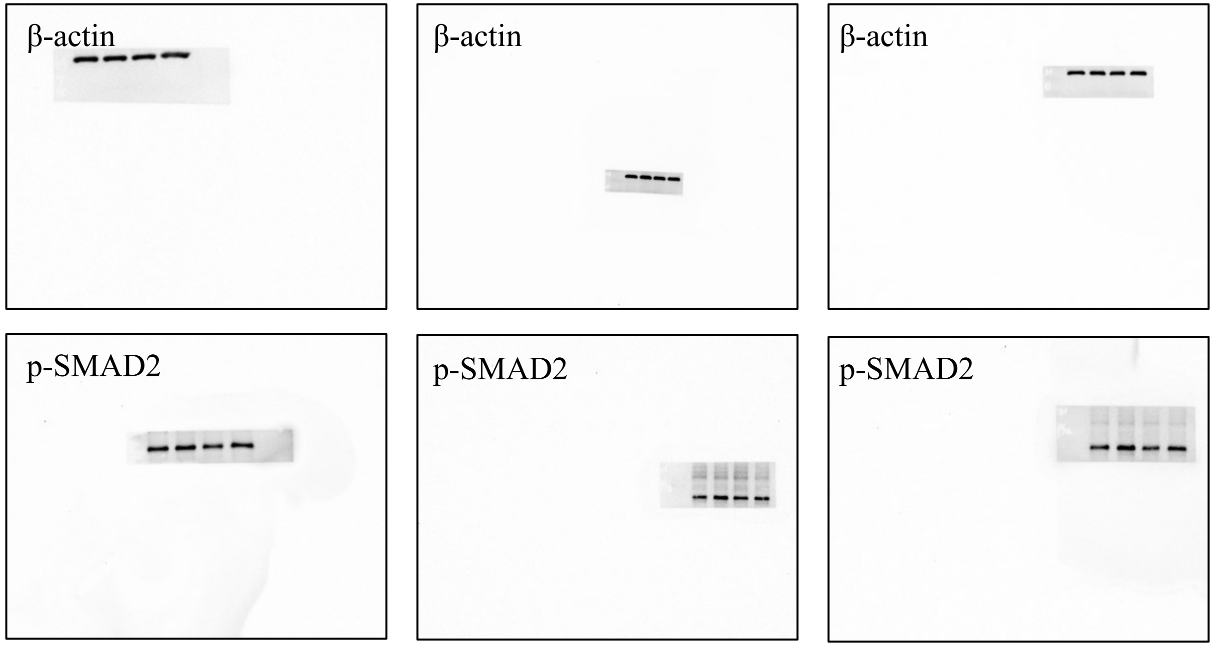

		actin	p-SMAD2			p-SMAD2/actin			Control me	relative expression		
1	**siNC**	1598470	2235334	2393838	2042041	1.398421	1.497581	1.277497	1.391166	1.005215	1.076493	0.918292
	**siNC+Ferritin**	1436956	2674592	3013772	2852881	1.86129	2.097331	1.985364	1.391166	1.337935	1.507606	1.427122
	**siPIK3R2+Ferritin**	1338715	1805109	1789281	1723531	1.348389	1.336566	1.287452	1.391166	0.969251	0.960752	0.925448
	**siPIK3R2+Ferritin+740 YP**	1431195	2161504	2632172	2733747	1.510279	1.839143	1.910115	1.391166	1.085621	1.322015	1.373031
		actin	p-SMAD2			p-SMAD2/actin			Control me	relative expression		
2	**siNC**	6929200	755477	721248	756488	0.109028	0.104088	0.109174	0.10743	1.014875	0.968893	1.016233
	**siNC+Ferritin**	7939218	1310974	1370742	1376097	0.165126	0.172655	0.173329	0.10743	1.537059	1.607134	1.613413
	**siPIK3R2+Ferritin**	7322214	871484	880873	853906	0.119019	0.120301	0.116619	0.10743	1.107876	1.119812	1.08553
	**siPIK3R2+Ferritin+740 YP**	7032902	922140	928810	1030579	0.131118	0.132066	0.146537	0.10743	1.220496	1.229324	1.364021
		actin	p-SMAD2			p-SMAD2/actin			Control me	relative expression		
3	**siNC**	4188700	1638043	1471975	1532228	0.391062	0.351416	0.3658	0.369426	1.058567	0.951248	0.990185
	**siNC+Ferritin**	4138746	2527136	2395755	2348337	0.610604	0.57886	0.567403	0.369426	1.652845	1.566917	1.535904
	**siPIK3R2+Ferritin**	3922484	1376781	1319363	1198200	0.350997	0.336359	0.30547	0.369426	0.950115	0.910491	0.826876
	**siPIK3R2+Ferritin+740 YP**	4359561	1820274	1701613	1696841	0.417536	0.390318	0.389223	0.369426	1.130229	1.056551	1.053588

Figure S6D-I

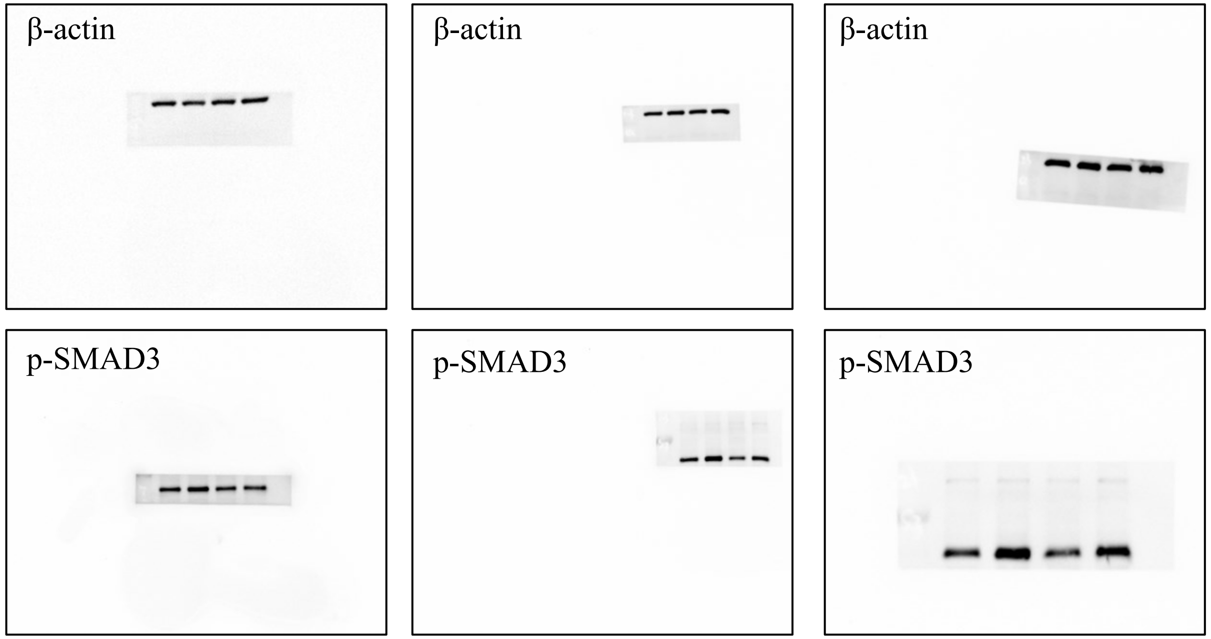

		actin	p-SMAD3			p-SMAD3/actin			Control me	relative expression		
1	siNC	1100984	3405791	3764145	3780144	3.093406	3.418892	3.433423	3.31524	0.933087	1.031265	1.035648
	siNC+Ferritin	903393	4541395	5092093	5082041	5.027042	5.636631	5.625504	3.31524	1.516343	1.700218	1.696862
	siPIK3R2+Ferritin	1281986	3086615	3039600	2894130	2.407682	2.371009	2.257536	3.31524	0.726247	0.715185	0.680957
	siPIK3R2+Ferritin+740 YP	1107083	3693781	3335440	3471053	3.336499	3.012818	3.135314	3.31524	1.006412	0.908778	0.945728
		actin	p-SMAD3			p-SMAD3/actin			Control me	relative expression		
2	siNC	7684516	5738896	6081978	5879837	0.746813	0.791459	0.765154	0.767809	0.972655	1.030802	0.996543
	siNC+Ferritin	8057858	8199047	8213309	8276813	1.017522	1.019292	1.027173	0.767809	1.325229	1.327534	1.337798
	siPIK3R2+Ferritin	8071602	3406370	3667468	3723304	0.422019	0.454367	0.461284	0.767809	0.549641	0.591771	0.60078
	siPIK3R2+Ferritin+740 YP	8739151	6054313	6254364	5380031	0.69278	0.715672	0.615624	0.767809	0.902283	0.932097	0.801794
		actin	p-SMAD3			p-SMAD3/actin			Control me	relative expression		
3	siNC	9511405	5859268	5928812	4960562	0.616025	0.623337	0.521538	0.586967	1.049506	1.061963	0.888531
	siNC+Ferritin	9303006	9335392	9456904	9569239	1.003481	1.016543	1.028618	0.586967	1.709604	1.731857	1.752429
	siPIK3R2+Ferritin	8982202	5088542	5184489	5264301	0.566514	0.577196	0.586081	0.586967	0.965155	0.983353	0.998491
	siPIK3R2+Ferritin+740 YP	11619746	7518379	7150746	7518379	0.647035	0.615396	0.647035	0.586967	1.102336	1.048434	1.102336
