# Supplementary material for: Macrophage–Derived Ferritin Exacerbates Silica‐Induced Pulmonary Fibrosis via PIK3R2‐Mediated Fibroblast Differentiation
Source: Adv Sci (Weinh). 2026 Jan 21;13(17):e19191. doi: 10.1002/advs.202519191 (PMC13042690; doi:10.1002/advs.202519191)
Supplement: Supplementary file 3 — Supporting File 3: advs73867‐sup‐0003‐SupportingFiguresData.zip. [file ADVS-13-e19191-s003.zip › Supporting information Figure S1-S9/S7/Figure S7C-F.pdf]

Figure S7C-F

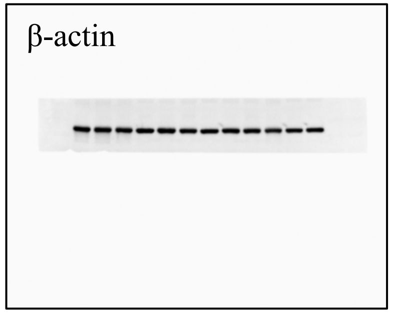

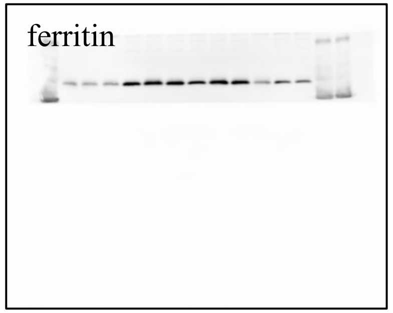

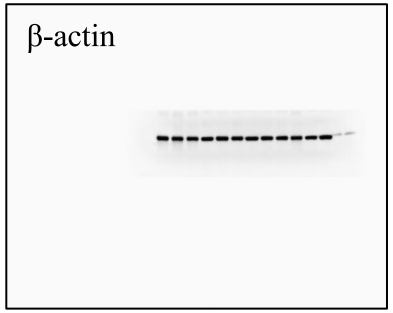

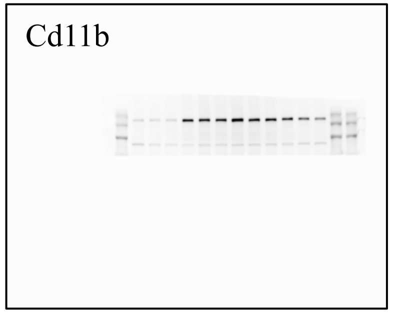

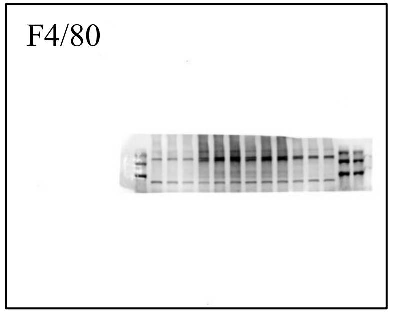

		actin	ferritin	ferritin/actin	Control mean	relative expression
Control	1#	4778599	1613889	0.3377327	0.363372368	0.929439606
	2#	5005890	1762920	0.3521691	0.363372368	0.969168754
	3#	4521384	1809527	0.4002153	0.363372368	1.10139164
Silica	1#	4646802	6376968	1.3723348	0.363372368	3.77666246
	2#	4964843	7068446	1.4236998	0.363372368	3.918018896
	3#	4103270	7089112	1.7276738	0.363372368	4.754554655
Silica+PBS	1#	3780894	5069413	1.3407974	0.363372368	3.689871769
	2#	4123480	7218667	1.750625	0.363372368	4.817716242
	3#	4121239	6423798	1.5587055	0.363372368	4.289554363
Silica+CTL	1#	3522866	1787846	0.5074976	0.363372368	1.396632308
	2#	3492733	2956262	0.8464037	0.363372368	2.329301116
	3#	3848185	2402328	0.6242756	0.363372368	1.718005153

		actin	F4/80	F4/80/actin	Control mean	relative expression
Control	1#	4110831	3021249	0.7349485	0.864372499	0.850268237
	2#	3256700	2889492	0.8872454	0.864372499	1.026461823
	3#	3152940	3061264	0.9709236	0.864372499	1.12326994
Silica	1#	3274453	5358903	1.6365796	0.864372499	1.893373062
	2#	3286511	6188636	1.8830413	0.864372499	2.178506741
	3#	3383106	6734783	1.99071	0.864372499	2.303069588
Silica+PBS	1#	3617796	5827899	1.6108976	0.864372499	1.863661362
	2#	3345634	6315546	1.8876978	0.864372499	2.183893887
	3#	2948159	6046369	2.0508965	0.864372499	2.37269986
Silica+CTL	1#	3043906	4674869	1.5358125	0.864372499	1.776794772
	2#	3135419	4070925	1.2983671	0.864372499	1.502092143
	3#	4344710	4188451	0.9640347	0.864372499	1.115300006

		actin	Cd11b	Cd11b/actin	Control mean	relative expression
Control	1#	4110831	470289	0.1144024	0.130259264	0.878267024
	2#	3256700	425575	0.1306768	0.130259264	1.003205103
	3#	3152940	459379	0.1456986	0.130259264	1.118527873
Silica	1#	3274453	2720202	0.8307348	0.130259264	6.37754855
	2#	3286511	2686085	0.817306	0.130259264	6.274455455
	3#	3383106	2645784	0.7820577	0.130259264	6.003854508
Silica+PBS	1#	3617796	4099618	1.1331811	0.130259264	8.69942796
	2#	3345634	2827909	0.8452535	0.130259264	6.489009053
	3#	2948159	2762972	0.9371855	0.130259264	7.194770754
Silica+CTL	1#	3043906	2337349	0.7678782	0.130259264	5.894998596
	2#	3135419	1783367	0.5687811	0.130259264	4.366530714
	3#	4344710	1436244	0.330573	0.130259264	2.537808297
