# Supplementary figures and images for: Macrophage–Derived Ferritin Exacerbates Silica‐Induced Pulmonary Fibrosis via PIK3R2‐Mediated Fibroblast Differentiation (part 2 of 4)
Source: Adv Sci (Weinh). 2026 Jan 21;13(17):e19191. doi: 10.1002/advs.202519191 (PMC13042690; doi:10.1002/advs.202519191)

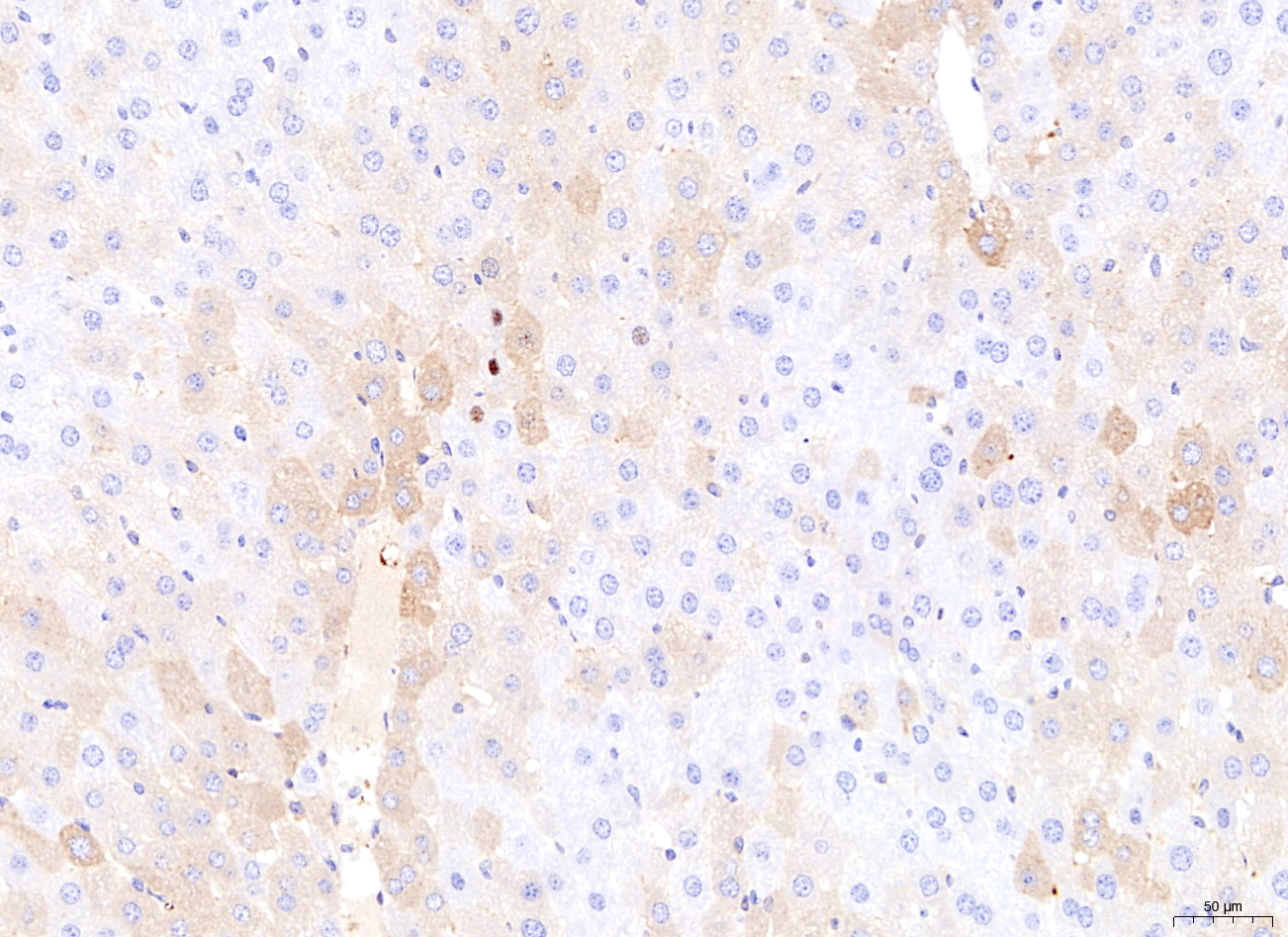

Supplement: Supplementary file 3 — Supporting File 3: advs73867‐sup‐0003‐SupportingFiguresData.zip. [file ADVS-13-e19191-s003.zip › Supporting information Figure S1-S9/S2/Liver/SCRS 4week Model 744_20.0x-3.jpg]

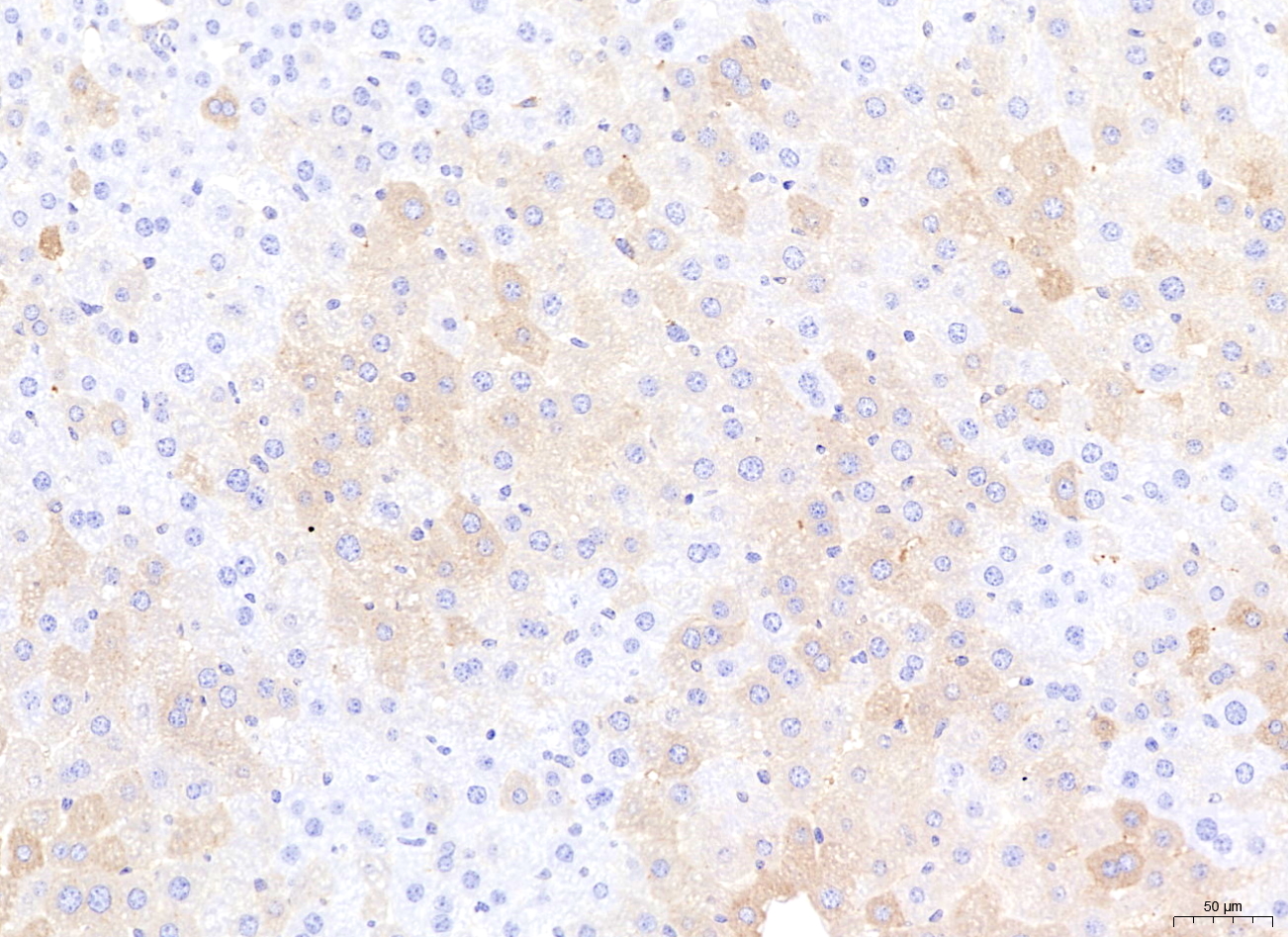

Supplement: Supplementary file 3 — Supporting File 3: advs73867‐sup‐0003‐SupportingFiguresData.zip. [file ADVS-13-e19191-s003.zip › Supporting information Figure S1-S9/S2/Liver/SCRS 4week Model 744_20.0x-4.jpg]

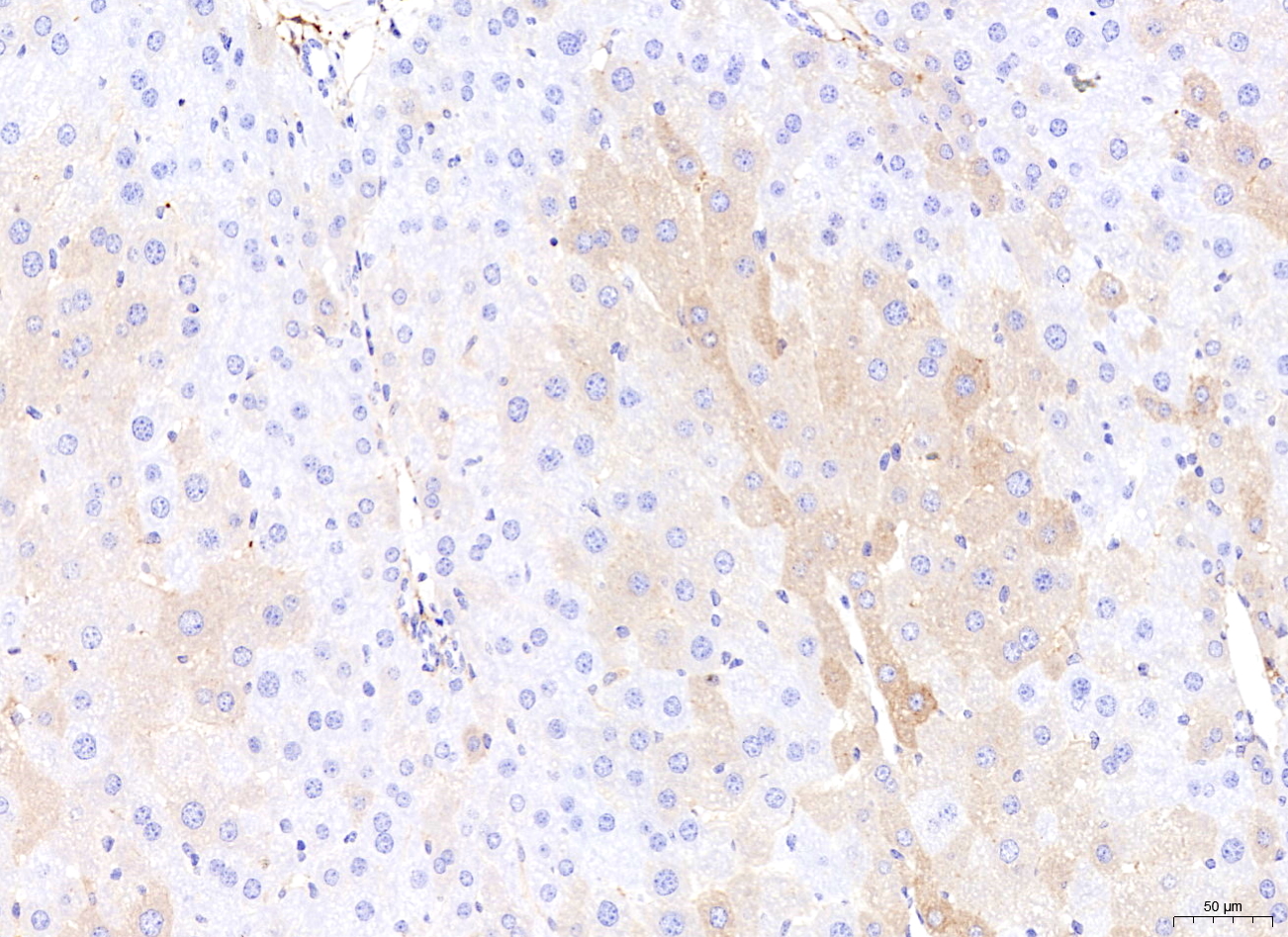

Supplement: Supplementary file 3 — Supporting File 3: advs73867‐sup‐0003‐SupportingFiguresData.zip. [file ADVS-13-e19191-s003.zip › Supporting information Figure S1-S9/S2/Liver/SCRS 4week Model 744_20.0x-5.jpg]

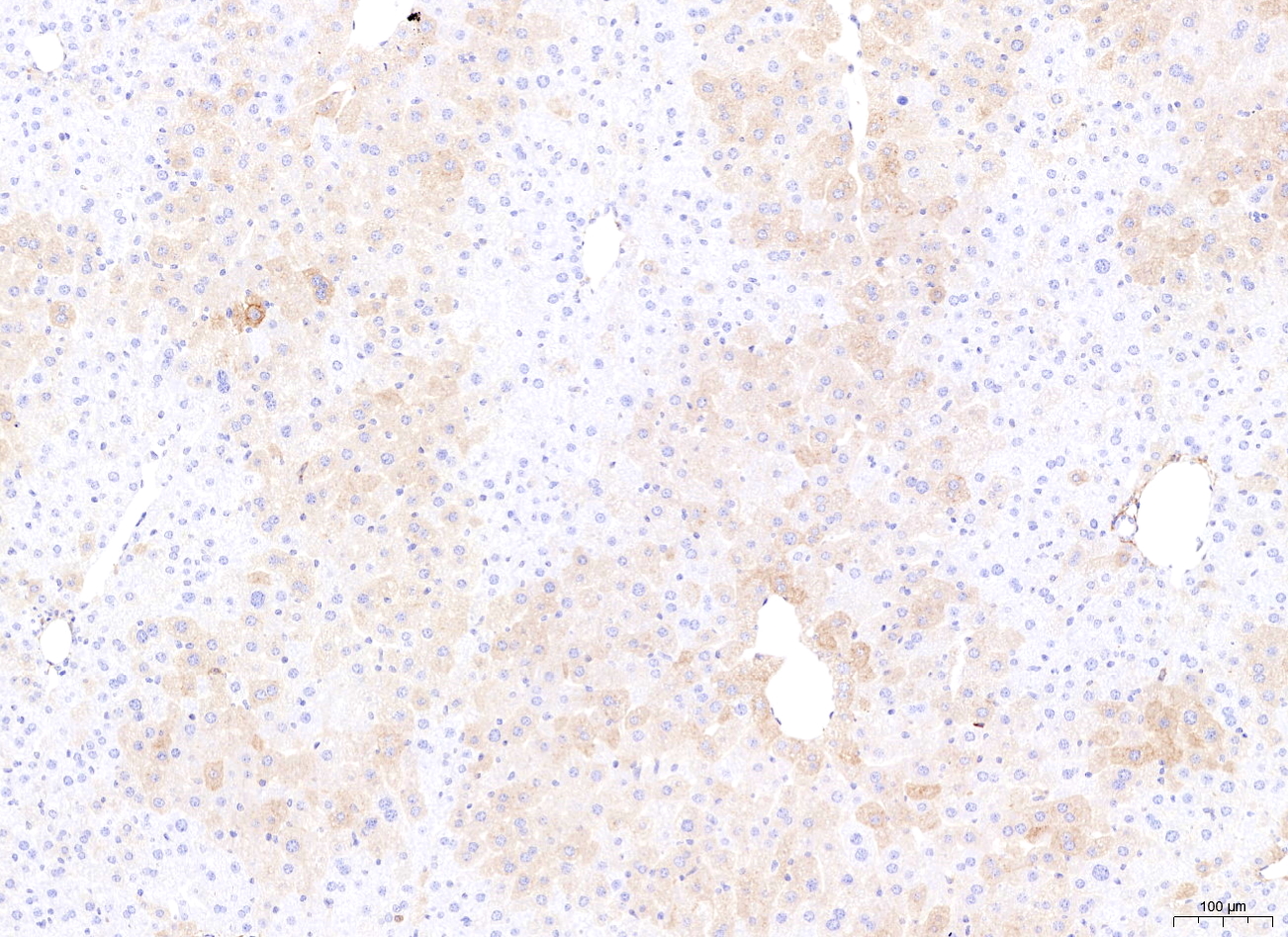

Supplement: Supplementary file 3 — Supporting File 3: advs73867‐sup‐0003‐SupportingFiguresData.zip. [file ADVS-13-e19191-s003.zip › Supporting information Figure S1-S9/S2/Liver/SCRS 8week Model 763_10.0x.jpg]

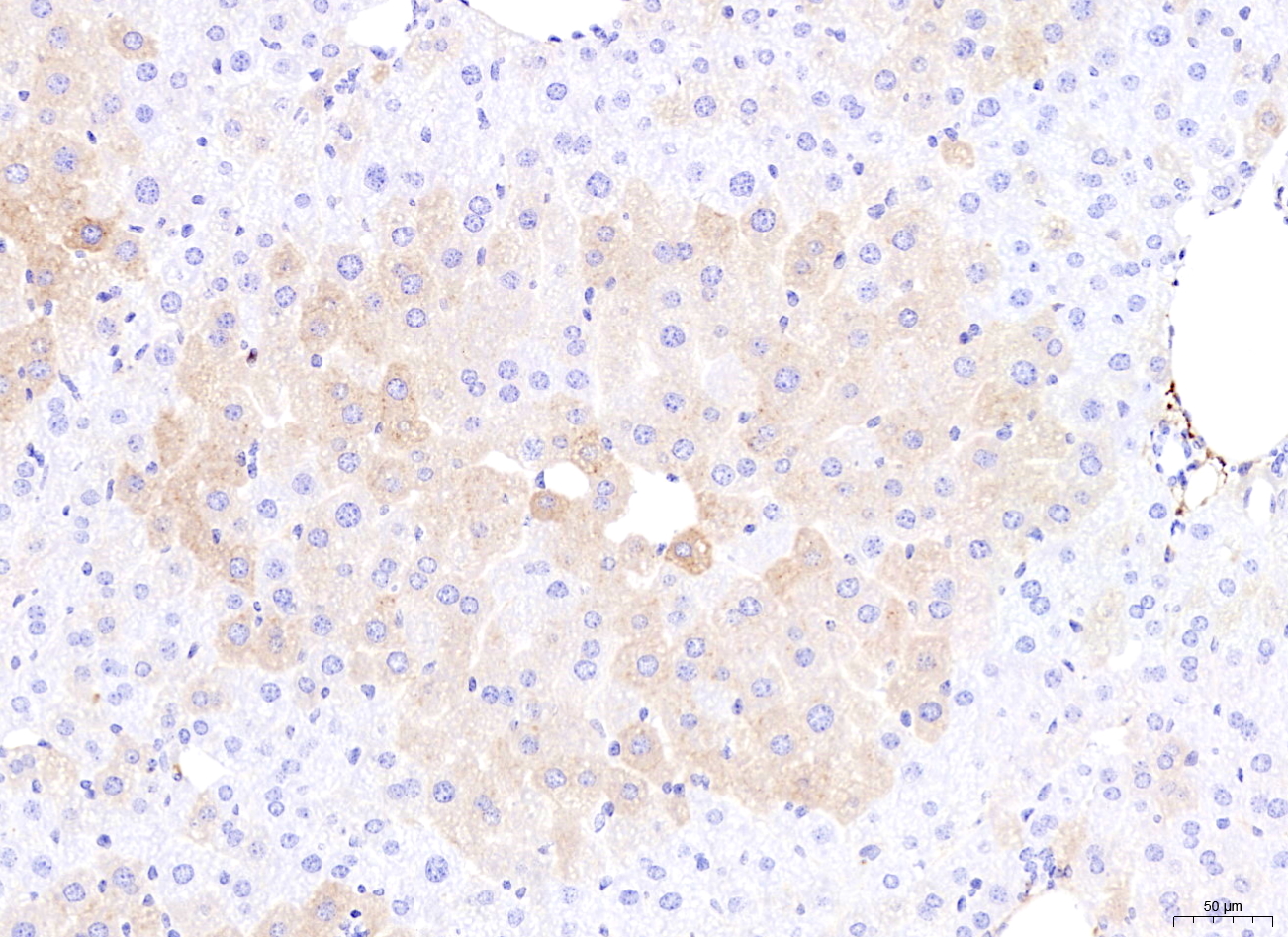

Supplement: Supplementary file 3 — Supporting File 3: advs73867‐sup‐0003‐SupportingFiguresData.zip. [file ADVS-13-e19191-s003.zip › Supporting information Figure S1-S9/S2/Liver/SCRS 8week Model 763_20.0x-1.jpg]

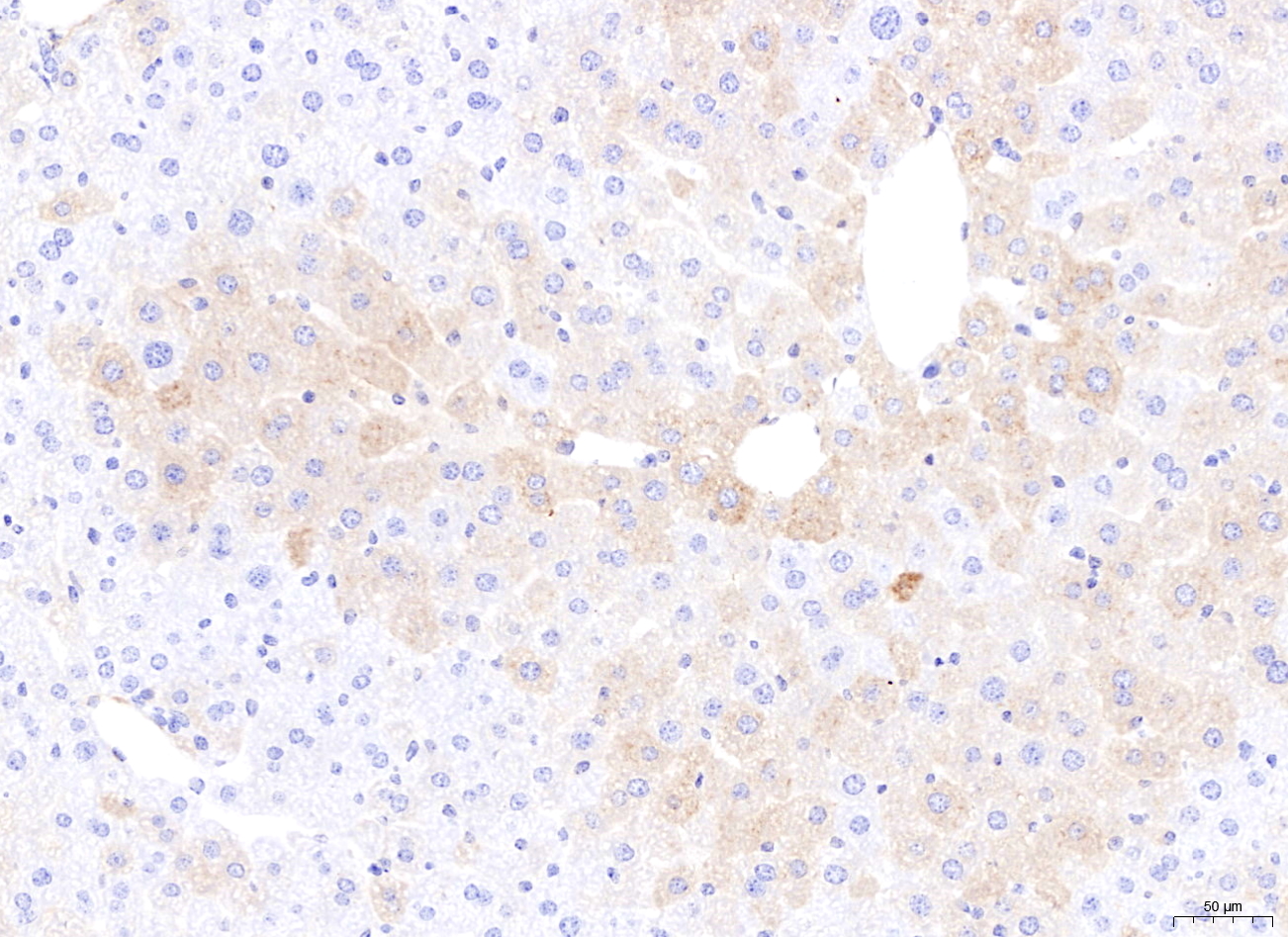

Supplement: Supplementary file 3 — Supporting File 3: advs73867‐sup‐0003‐SupportingFiguresData.zip. [file ADVS-13-e19191-s003.zip › Supporting information Figure S1-S9/S2/Liver/SCRS 8week Model 763_20.0x-2.jpg]

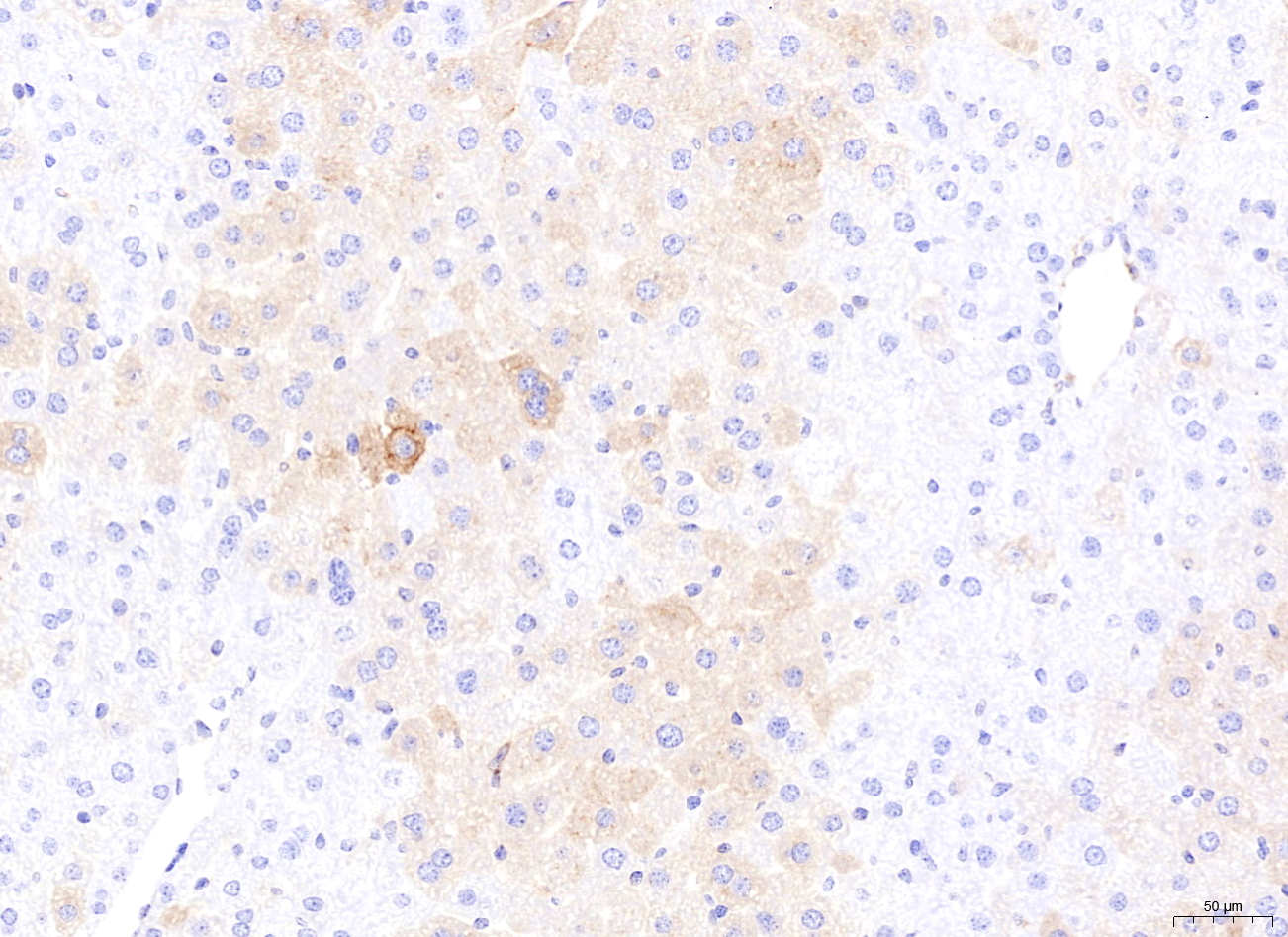

Supplement: Supplementary file 3 — Supporting File 3: advs73867‐sup‐0003‐SupportingFiguresData.zip. [file ADVS-13-e19191-s003.zip › Supporting information Figure S1-S9/S2/Liver/SCRS 8week Model 763_20.0x-3.jpg]

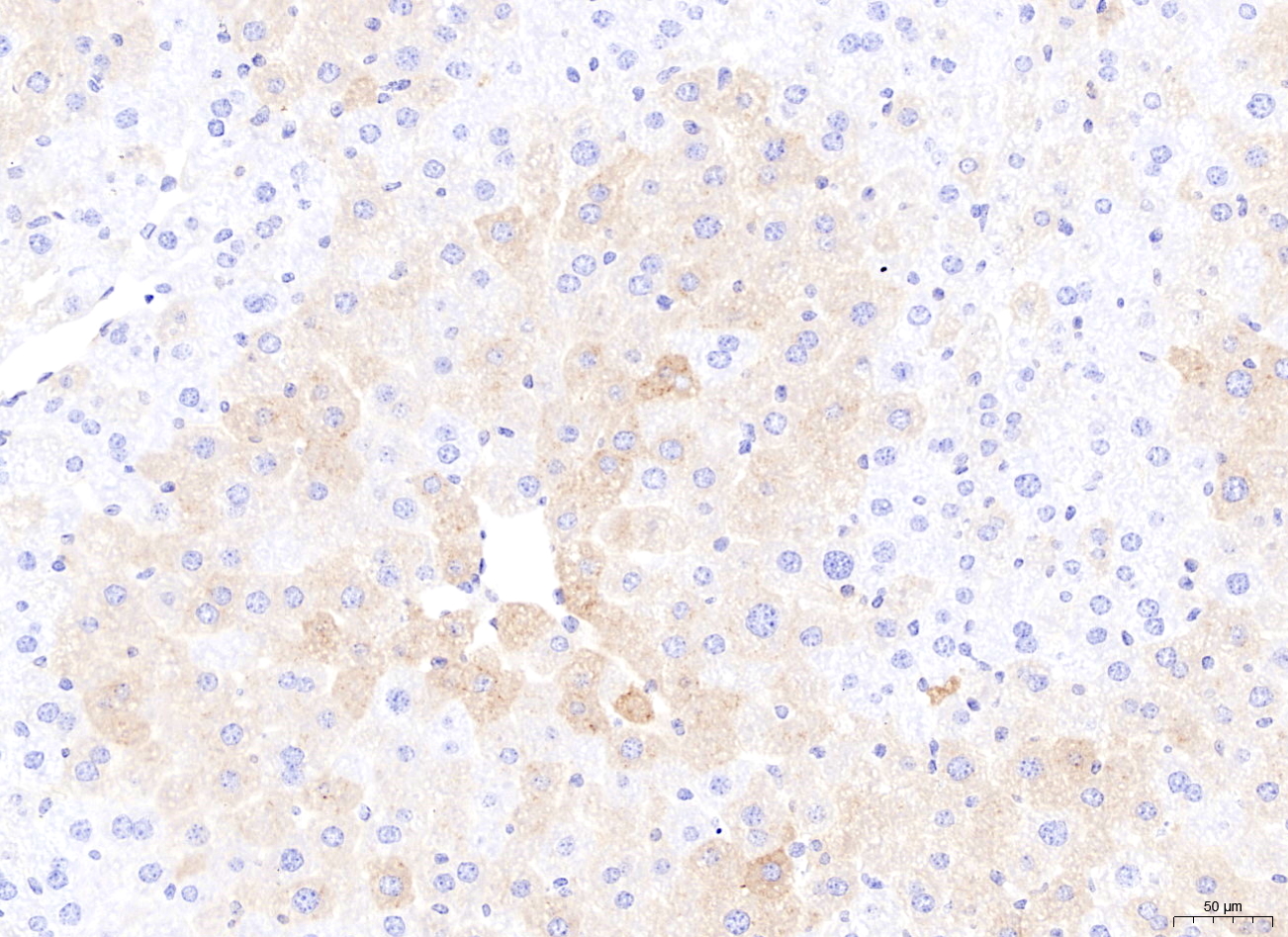

Supplement: Supplementary file 3 — Supporting File 3: advs73867‐sup‐0003‐SupportingFiguresData.zip. [file ADVS-13-e19191-s003.zip › Supporting information Figure S1-S9/S2/Liver/SCRS 8week Model 763_20.0x-4.jpg]

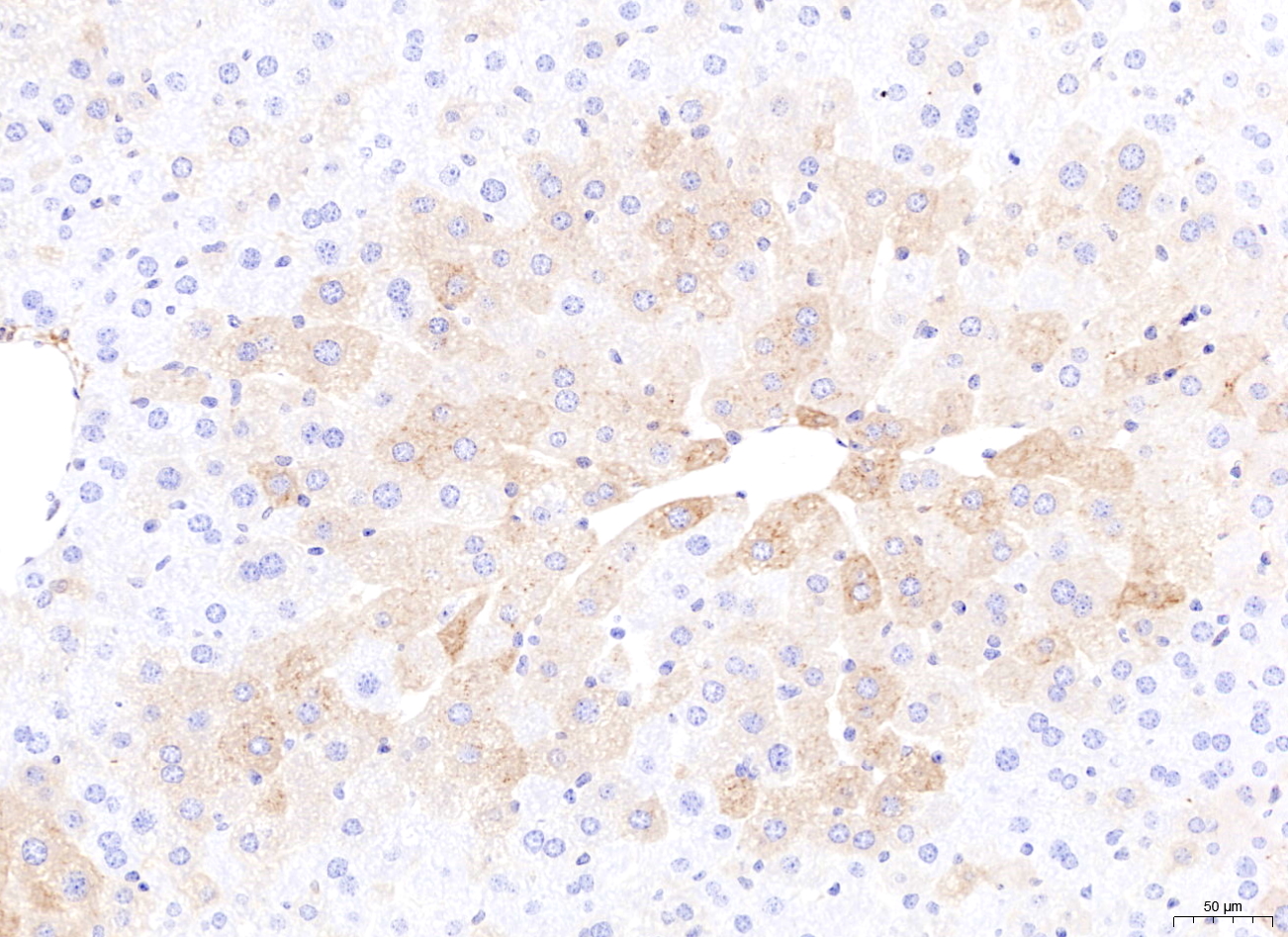

Supplement: Supplementary file 3 — Supporting File 3: advs73867‐sup‐0003‐SupportingFiguresData.zip. [file ADVS-13-e19191-s003.zip › Supporting information Figure S1-S9/S2/Liver/SCRS 8week Model 763_20.0x-5.jpg]

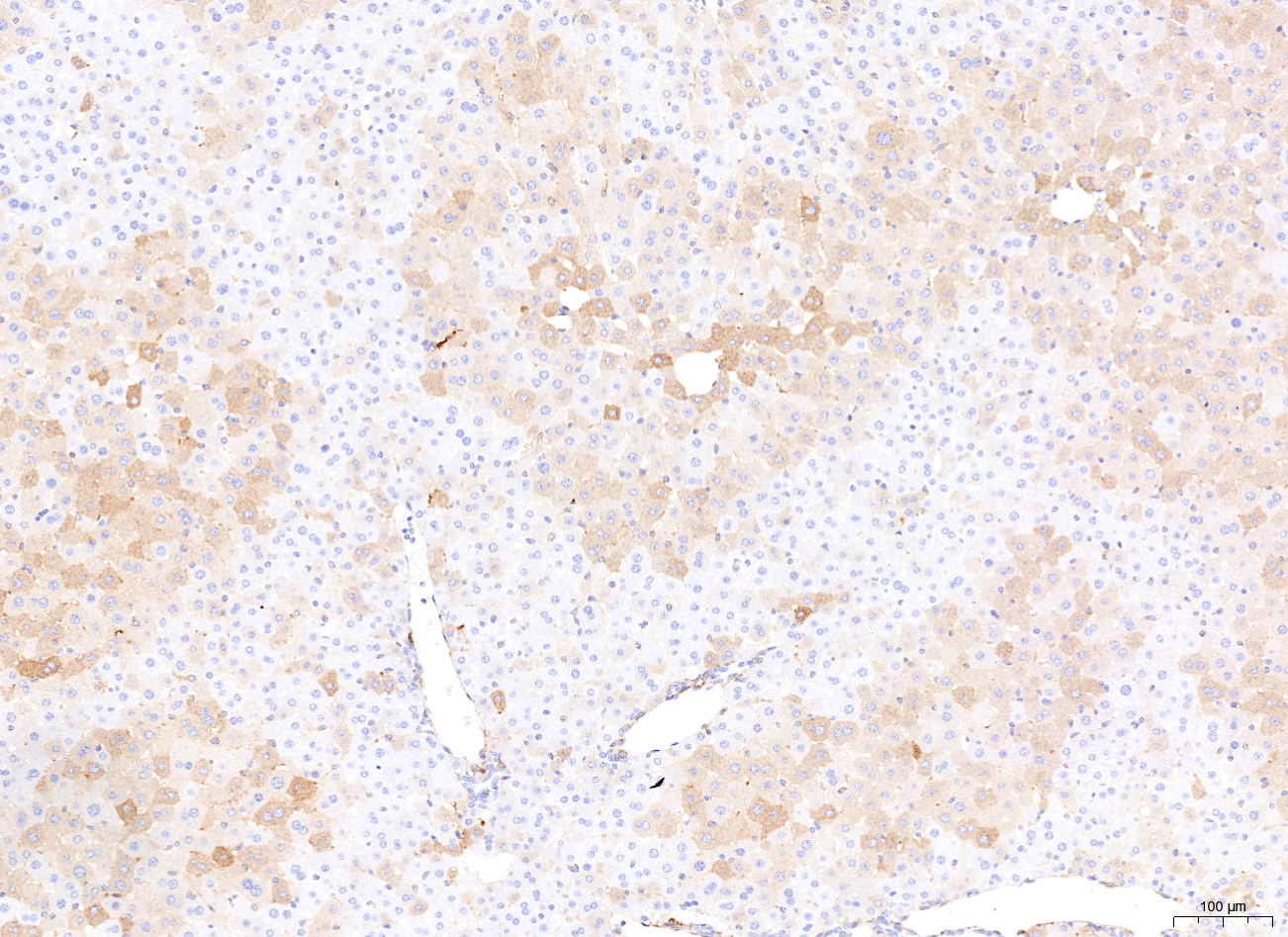

Supplement: Supplementary file 3 — Supporting File 3: advs73867‐sup‐0003‐SupportingFiguresData.zip. [file ADVS-13-e19191-s003.zip › Supporting information Figure S1-S9/S2/Liver/SCRS Control 782_10.0x.jpg]

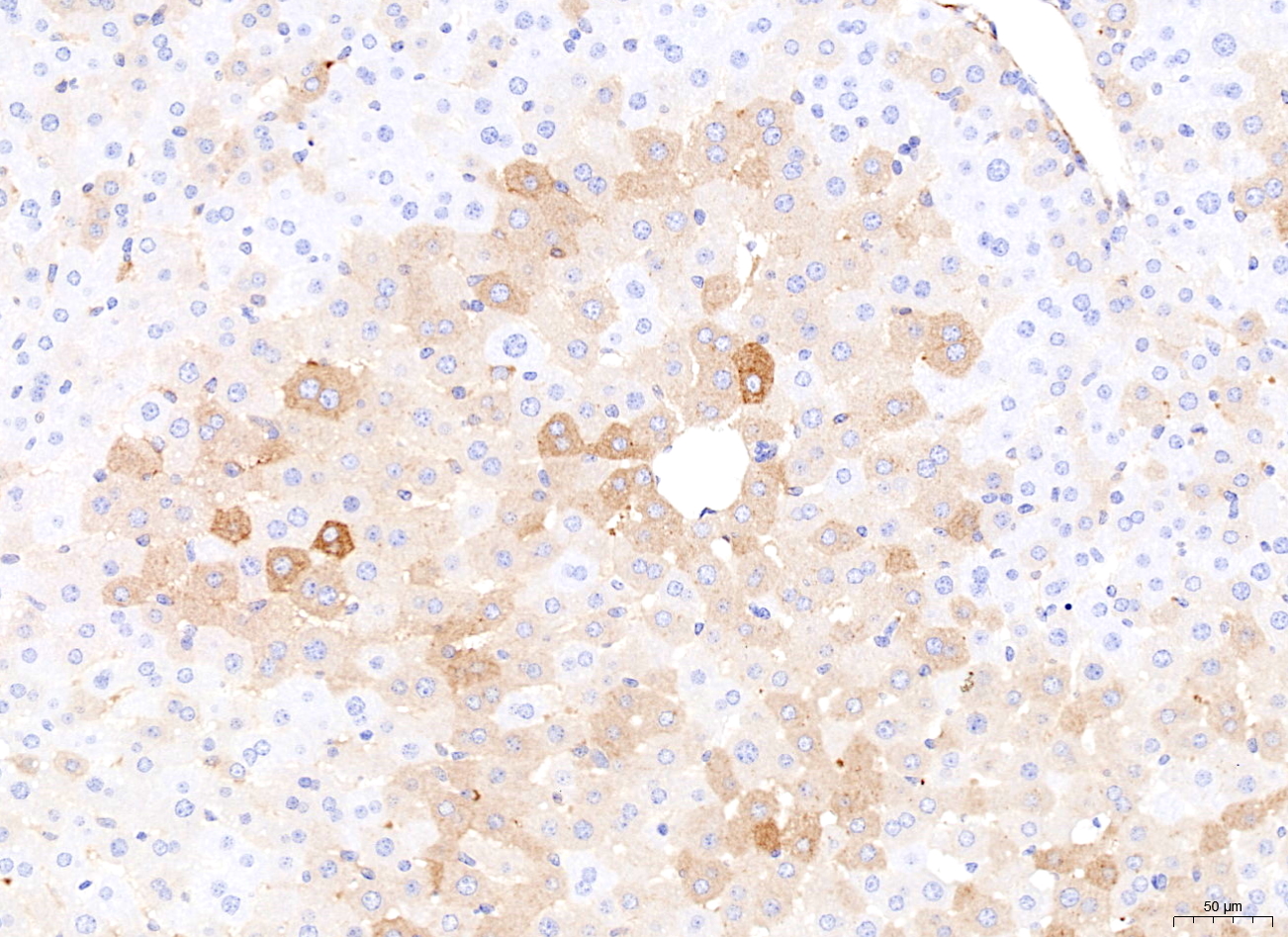

Supplement: Supplementary file 3 — Supporting File 3: advs73867‐sup‐0003‐SupportingFiguresData.zip. [file ADVS-13-e19191-s003.zip › Supporting information Figure S1-S9/S2/Liver/SCRS Control 782_20.0x-1.jpg]

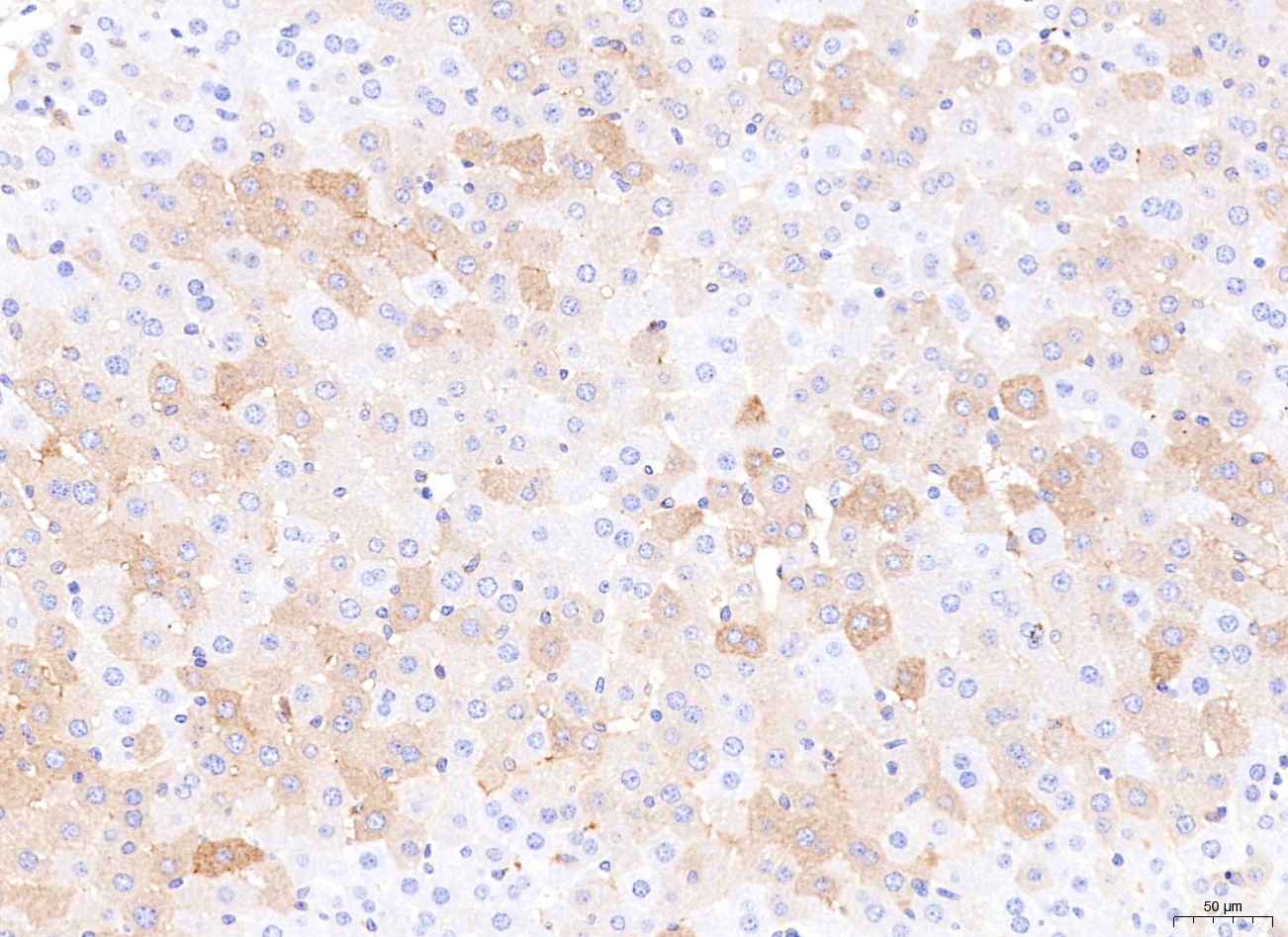

Supplement: Supplementary file 3 — Supporting File 3: advs73867‐sup‐0003‐SupportingFiguresData.zip. [file ADVS-13-e19191-s003.zip › Supporting information Figure S1-S9/S2/Liver/SCRS Control 782_20.0x-2.jpg]

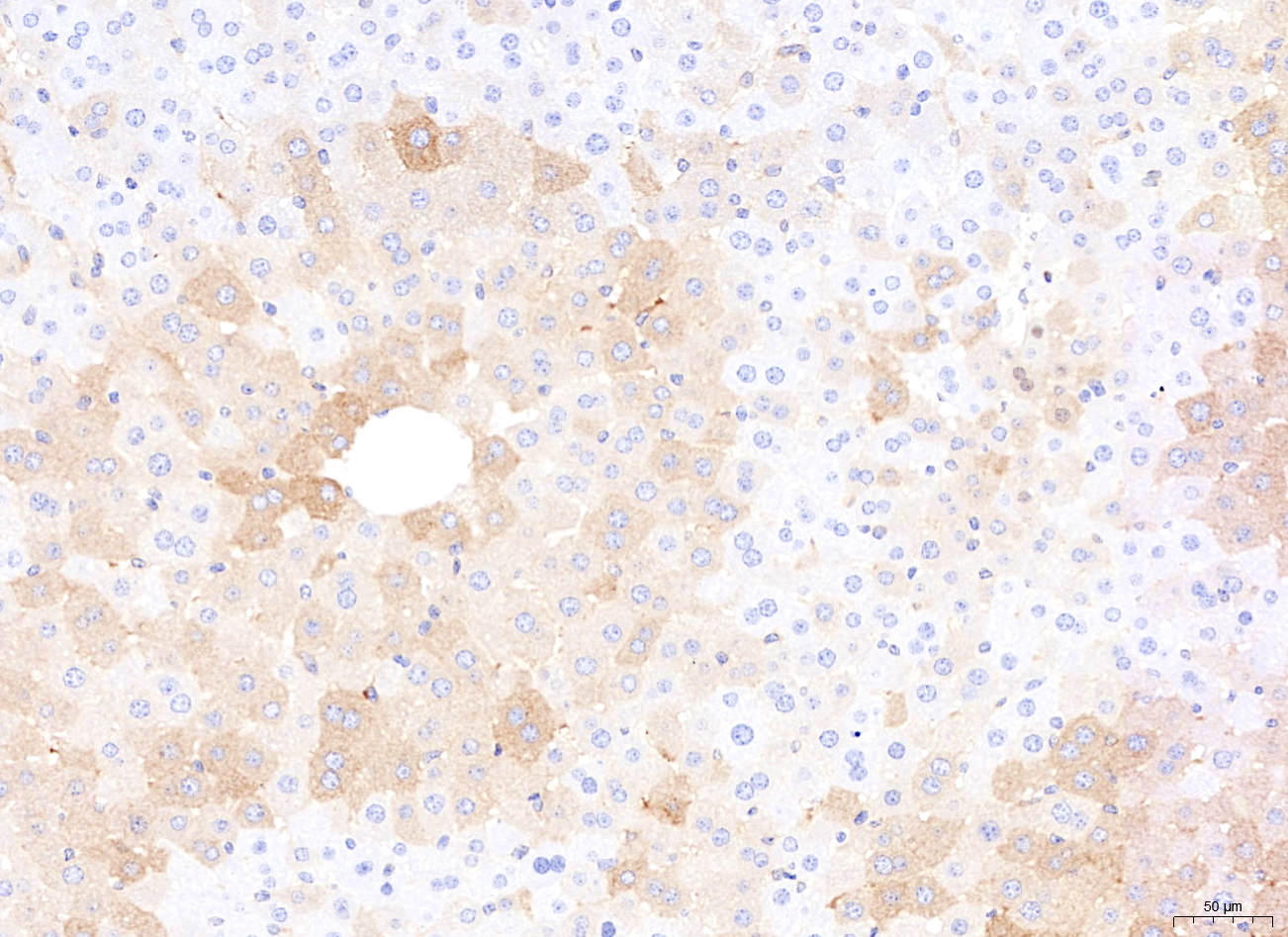

Supplement: Supplementary file 3 — Supporting File 3: advs73867‐sup‐0003‐SupportingFiguresData.zip. [file ADVS-13-e19191-s003.zip › Supporting information Figure S1-S9/S2/Liver/SCRS Control 782_20.0x-3.jpg]

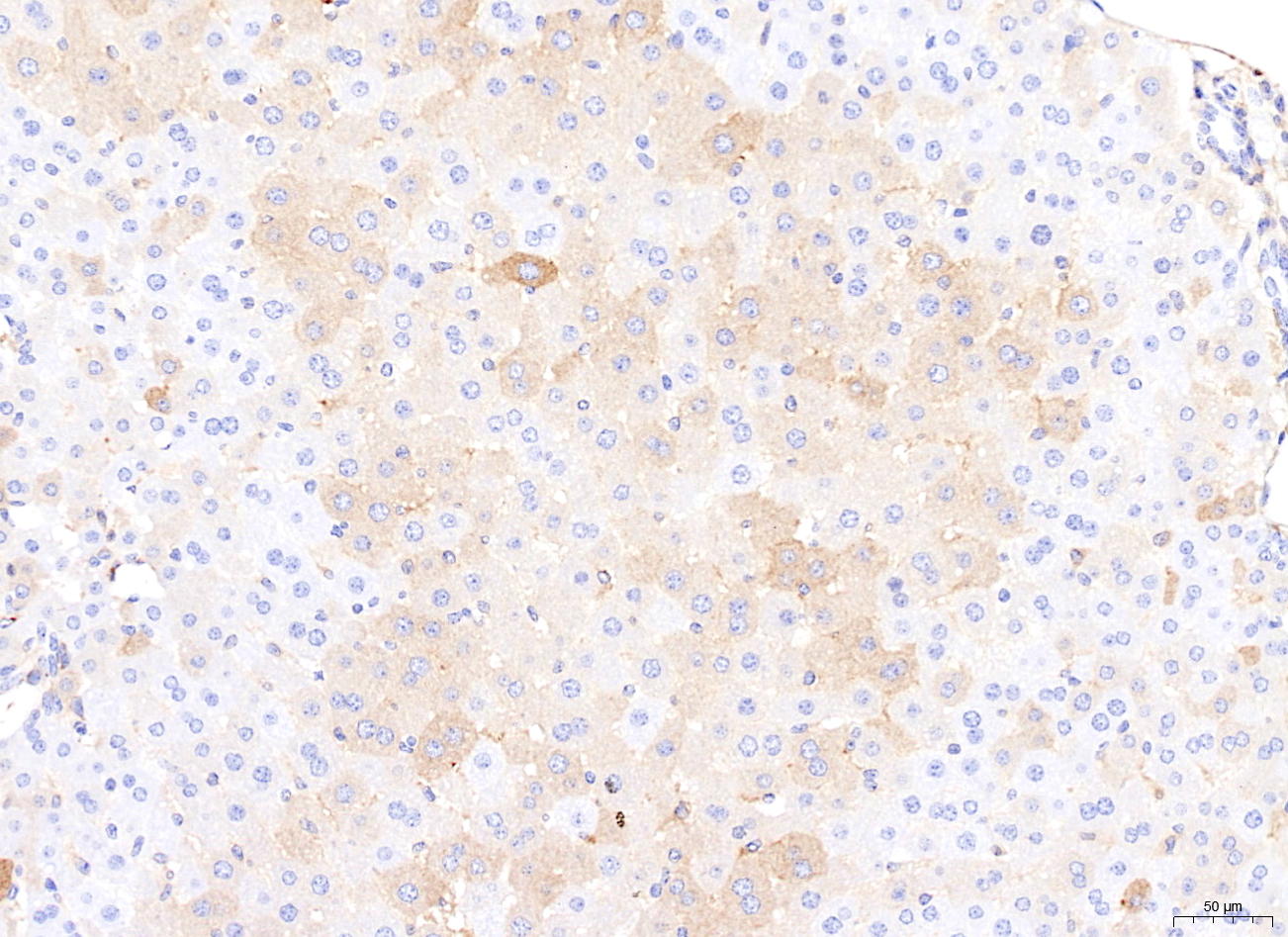

Supplement: Supplementary file 3 — Supporting File 3: advs73867‐sup‐0003‐SupportingFiguresData.zip. [file ADVS-13-e19191-s003.zip › Supporting information Figure S1-S9/S2/Liver/SCRS Control 782_20.0x-4.jpg]

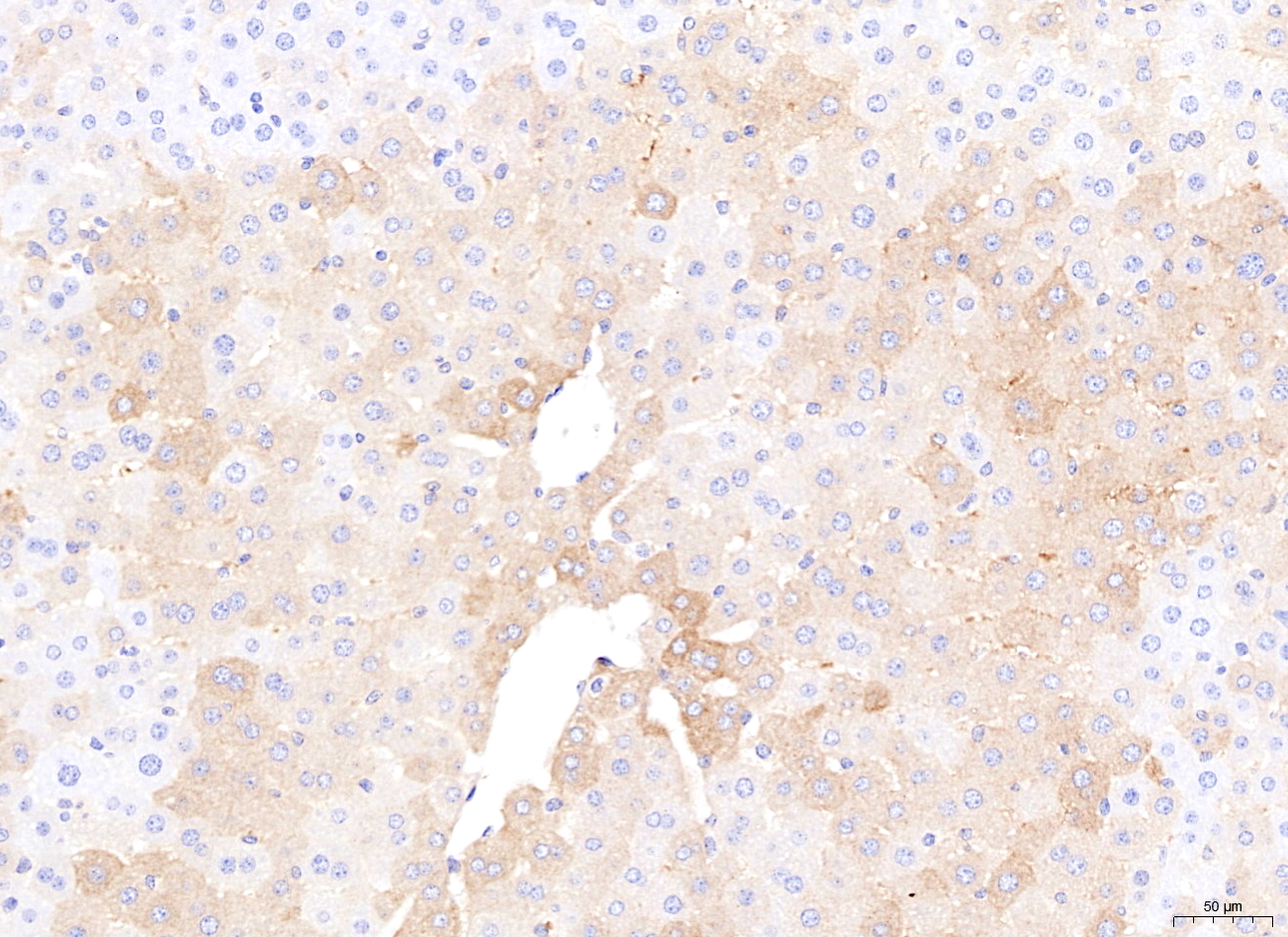

Supplement: Supplementary file 3 — Supporting File 3: advs73867‐sup‐0003‐SupportingFiguresData.zip. [file ADVS-13-e19191-s003.zip › Supporting information Figure S1-S9/S2/Liver/SCRS Control 782_20.0x-5.jpg]

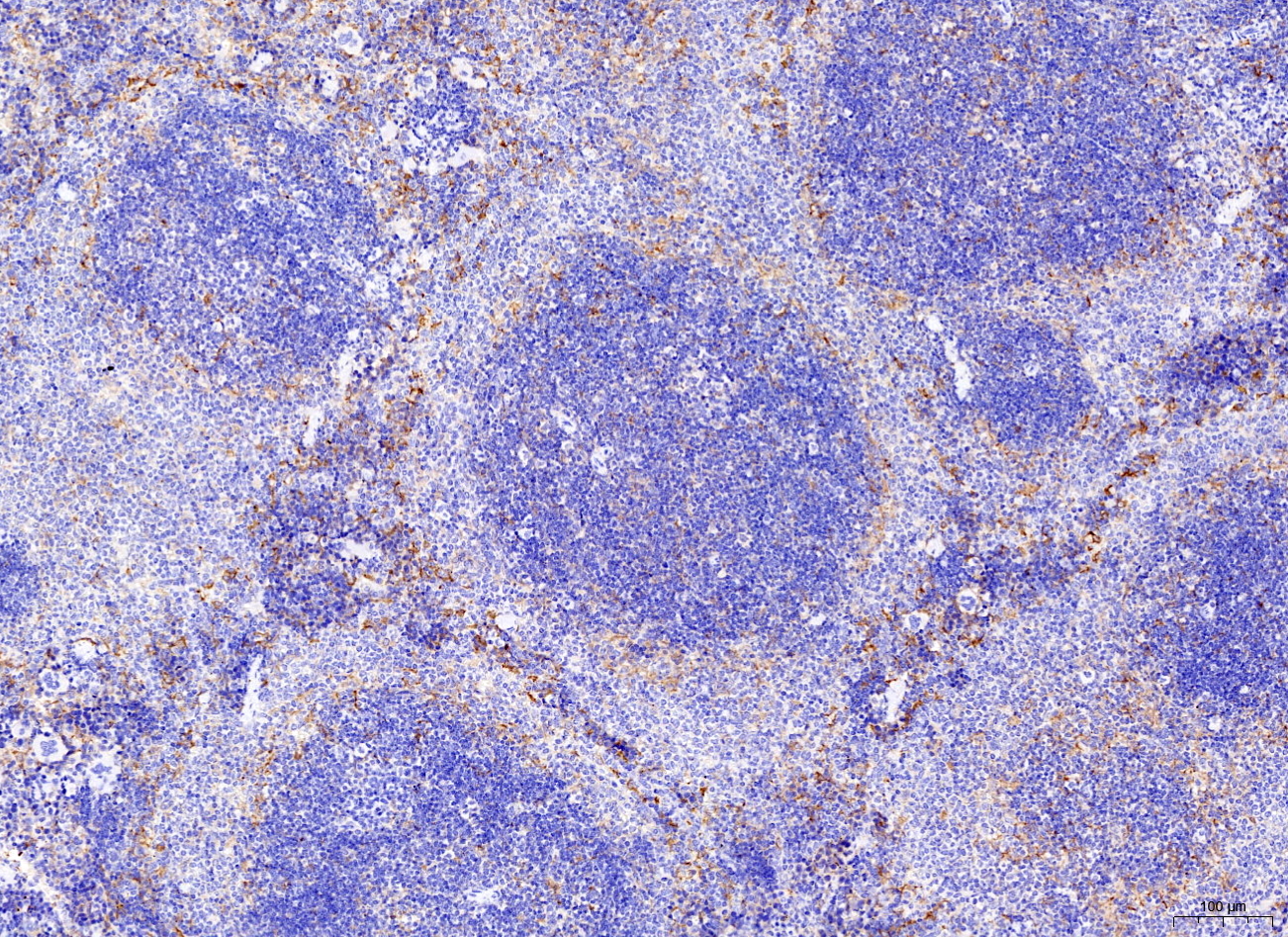

Supplement: Supplementary file 3 — Supporting File 3: advs73867‐sup‐0003‐SupportingFiguresData.zip. [file ADVS-13-e19191-s003.zip › Supporting information Figure S1-S9/S2/Spleen/SCRS 12week Model 784_10.0x.jpg]

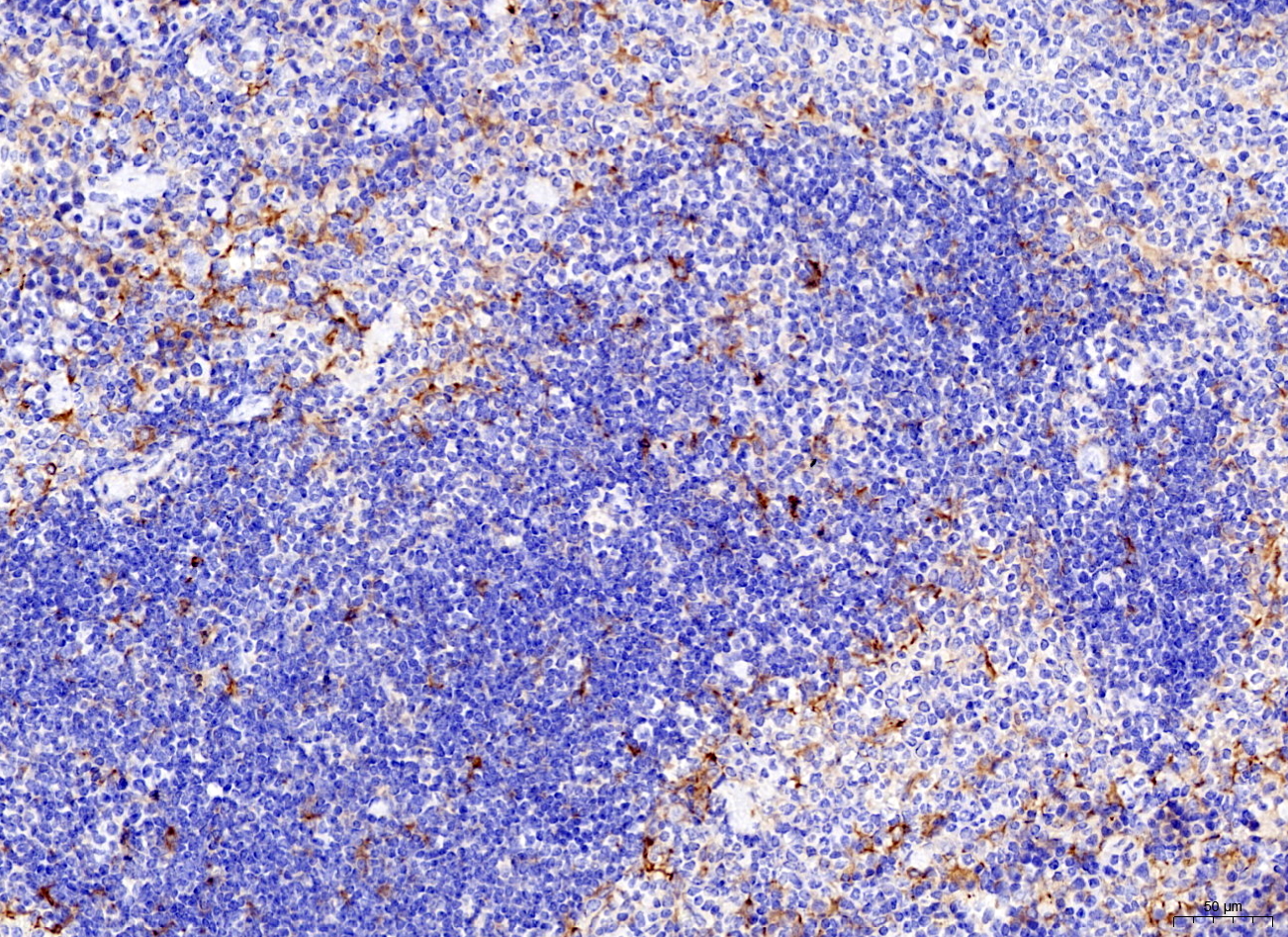

Supplement: Supplementary file 3 — Supporting File 3: advs73867‐sup‐0003‐SupportingFiguresData.zip. [file ADVS-13-e19191-s003.zip › Supporting information Figure S1-S9/S2/Spleen/SCRS 12week Model 784_20.0x-1.jpg]

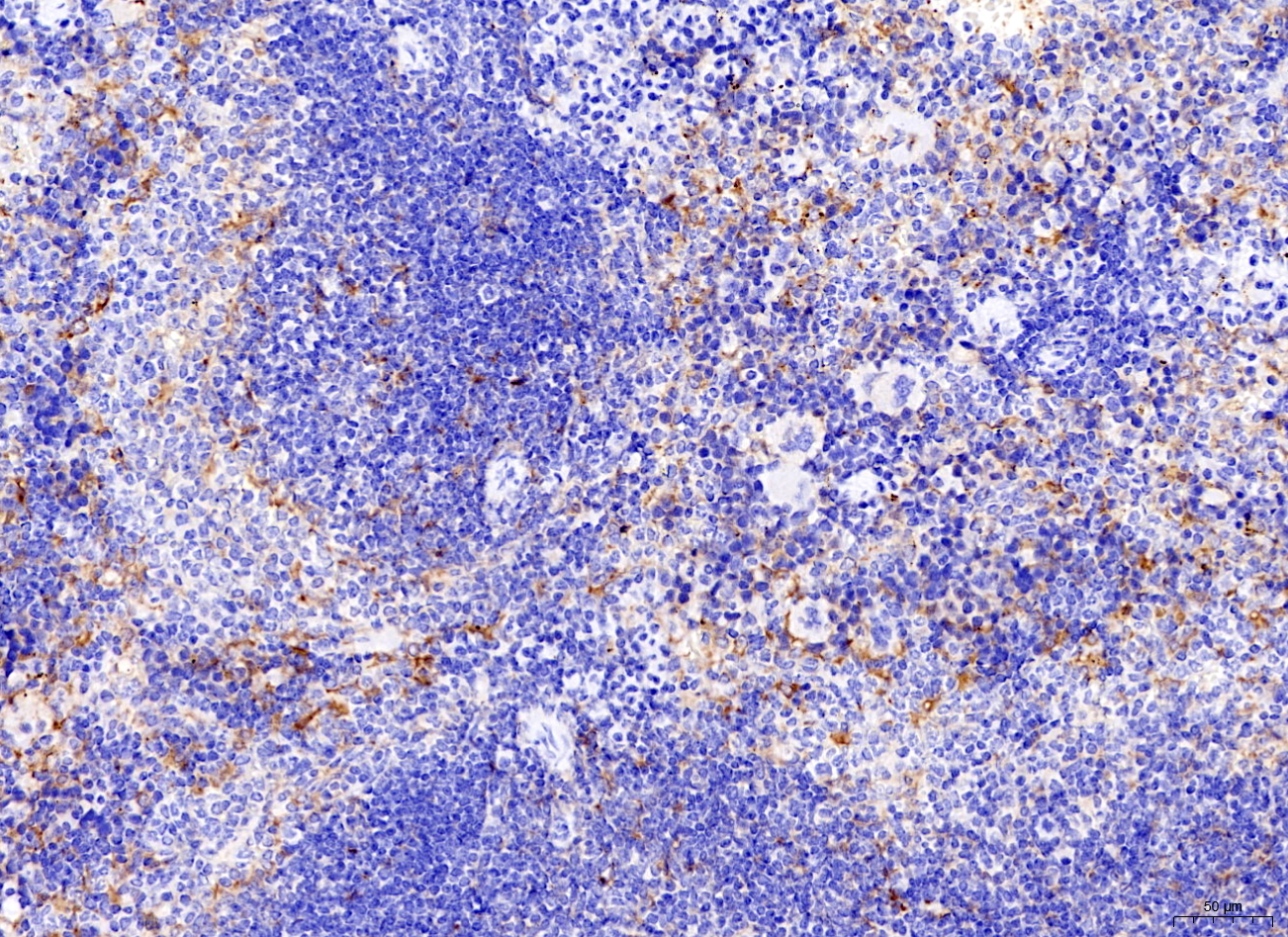

Supplement: Supplementary file 3 — Supporting File 3: advs73867‐sup‐0003‐SupportingFiguresData.zip. [file ADVS-13-e19191-s003.zip › Supporting information Figure S1-S9/S2/Spleen/SCRS 12week Model 784_20.0x-2.jpg]

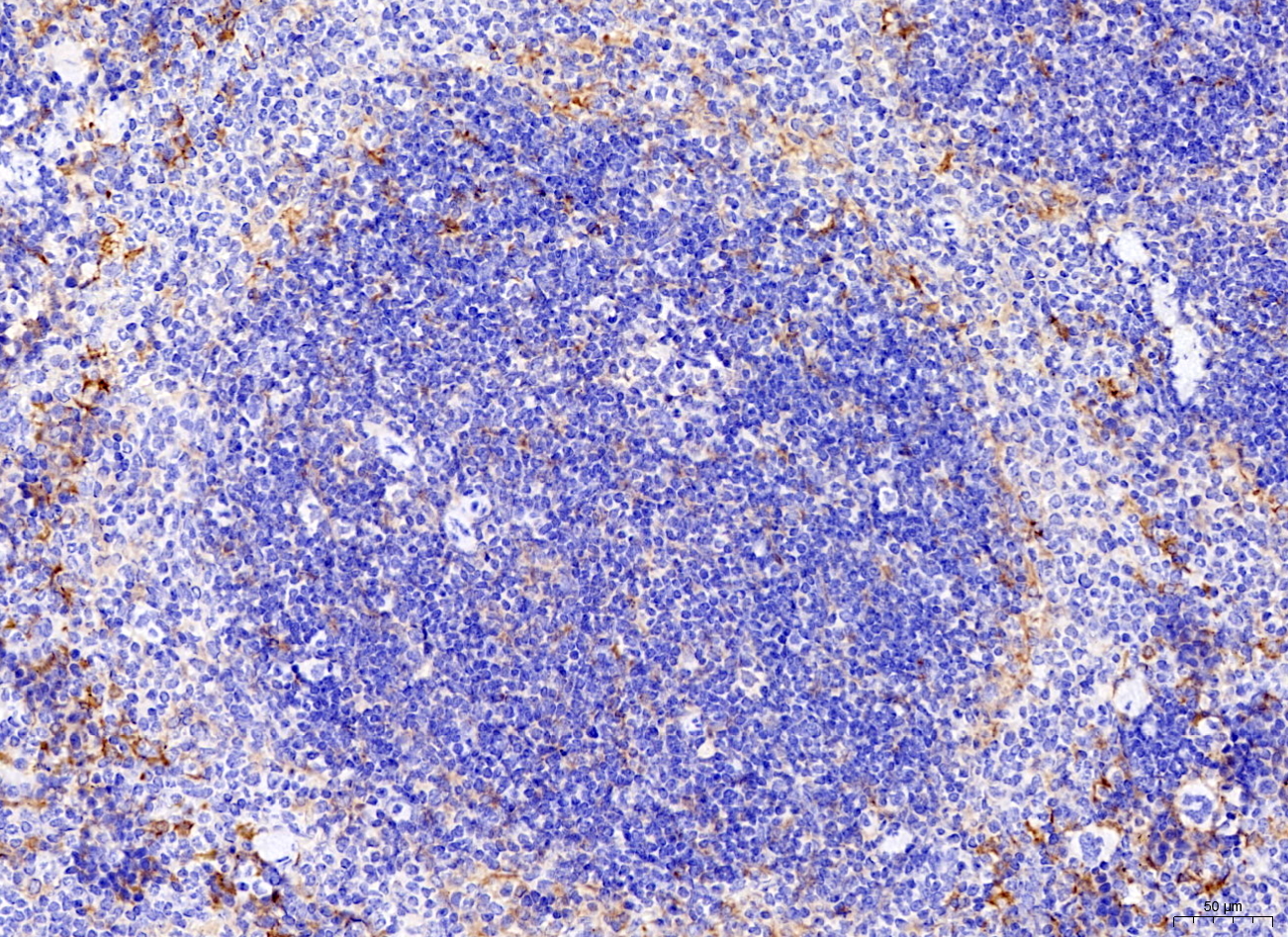

Supplement: Supplementary file 3 — Supporting File 3: advs73867‐sup‐0003‐SupportingFiguresData.zip. [file ADVS-13-e19191-s003.zip › Supporting information Figure S1-S9/S2/Spleen/SCRS 12week Model 784_20.0x-3.jpg]

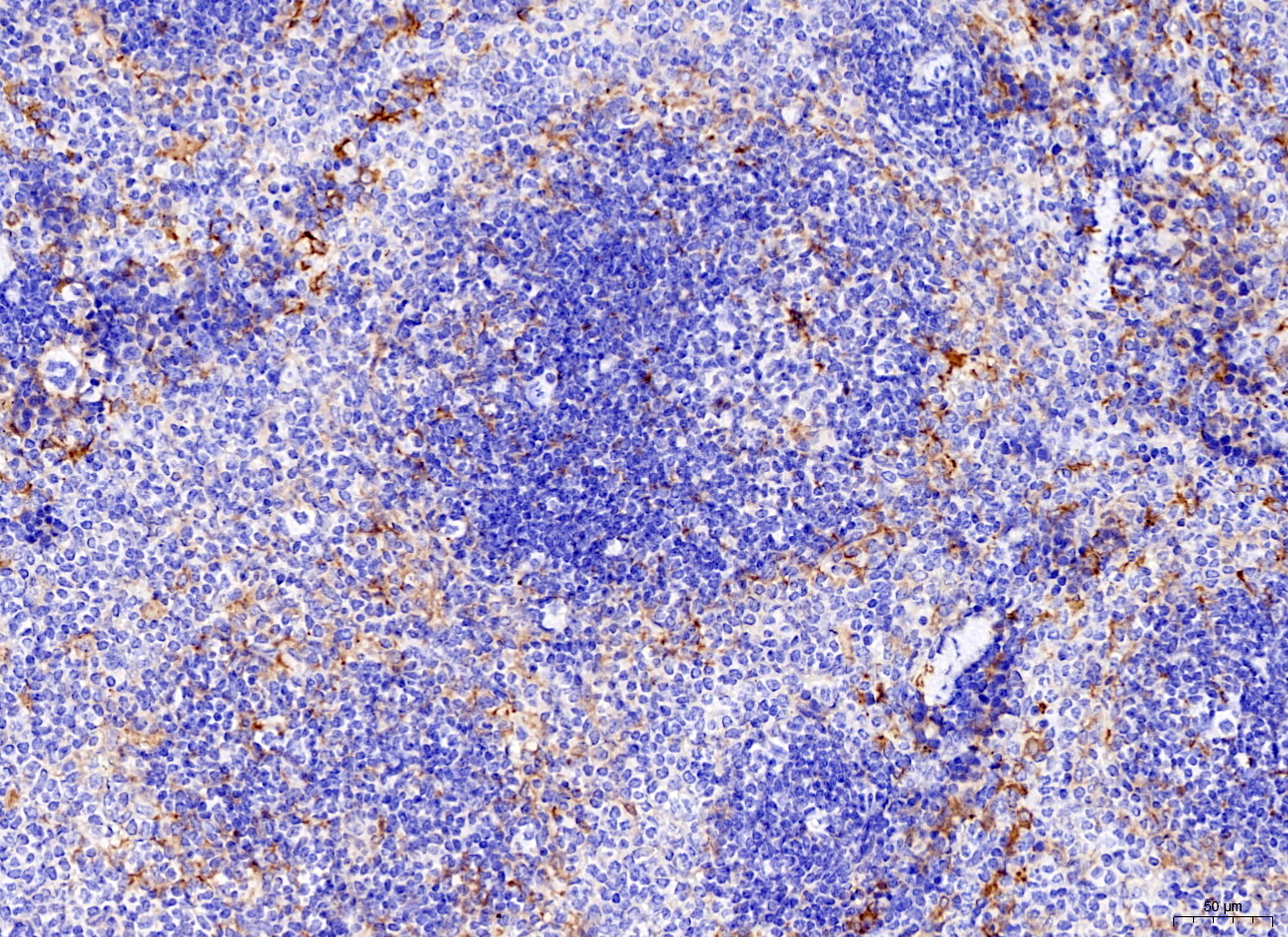

Supplement: Supplementary file 3 — Supporting File 3: advs73867‐sup‐0003‐SupportingFiguresData.zip. [file ADVS-13-e19191-s003.zip › Supporting information Figure S1-S9/S2/Spleen/SCRS 12week Model 784_20.0x-4.jpg]

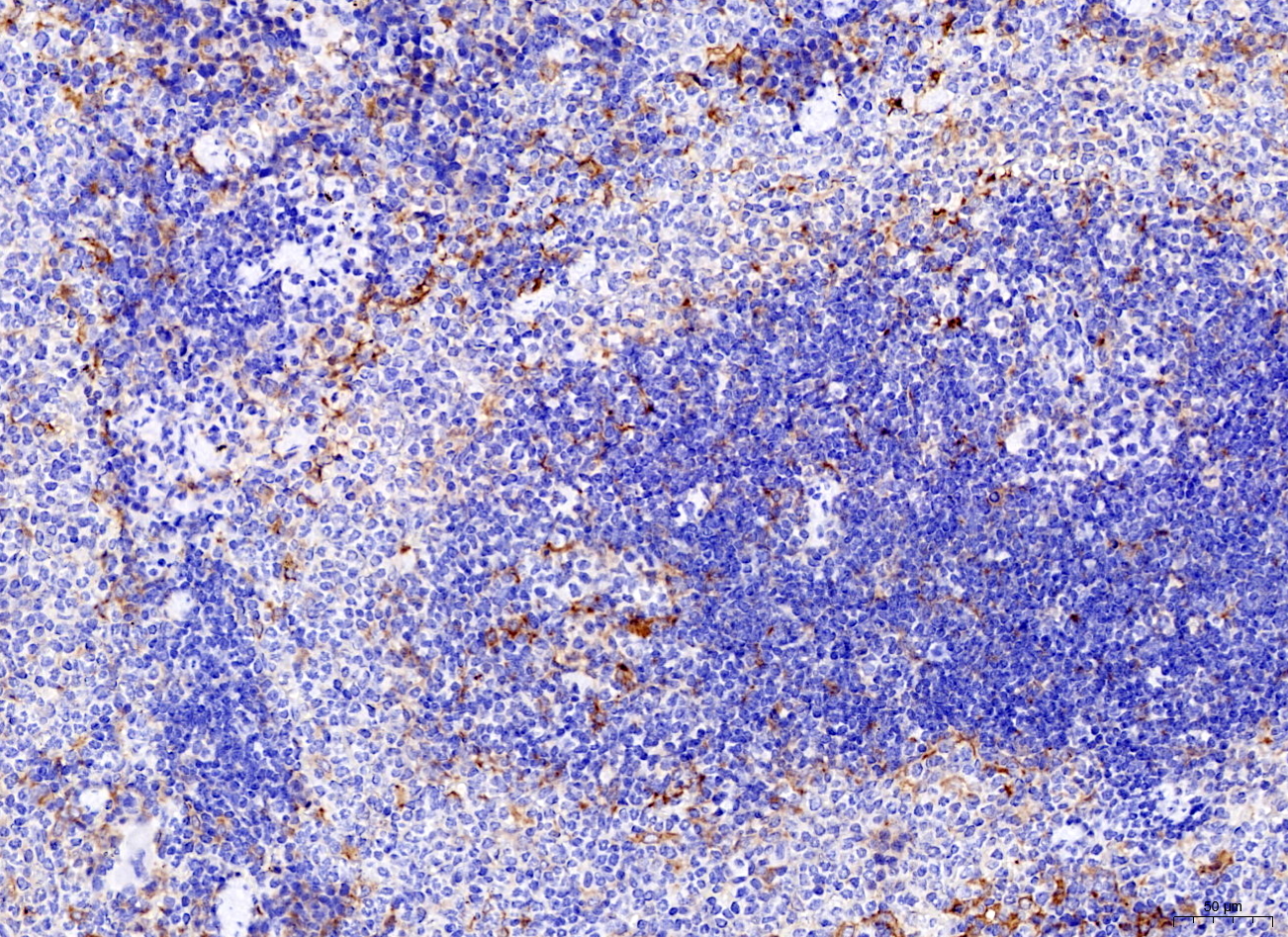

Supplement: Supplementary file 3 — Supporting File 3: advs73867‐sup‐0003‐SupportingFiguresData.zip. [file ADVS-13-e19191-s003.zip › Supporting information Figure S1-S9/S2/Spleen/SCRS 12week Model 784_20.0x-5.jpg]

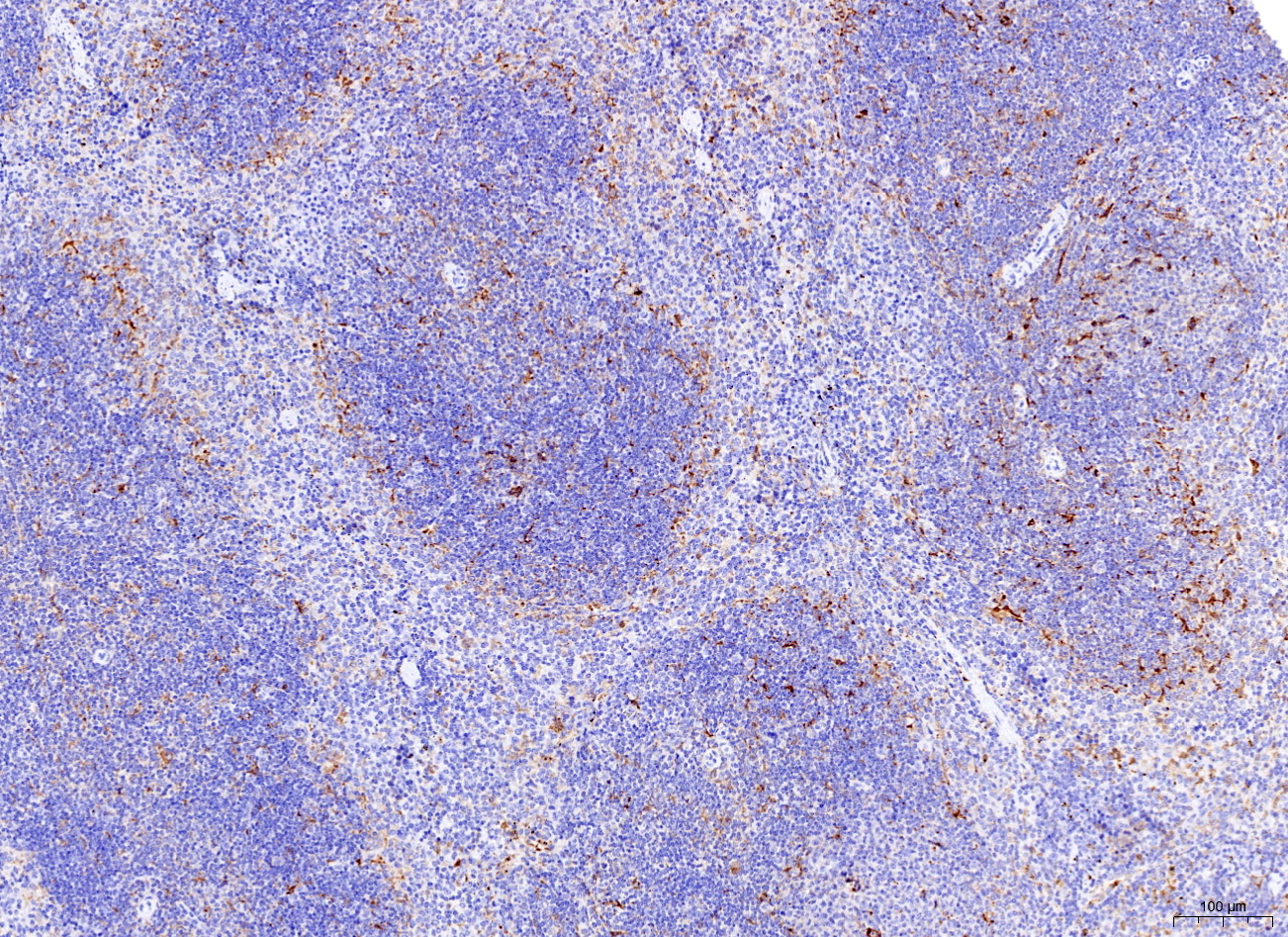

Supplement: Supplementary file 3 — Supporting File 3: advs73867‐sup‐0003‐SupportingFiguresData.zip. [file ADVS-13-e19191-s003.zip › Supporting information Figure S1-S9/S2/Spleen/SCRS 1week Model 757_10.0x.jpg]

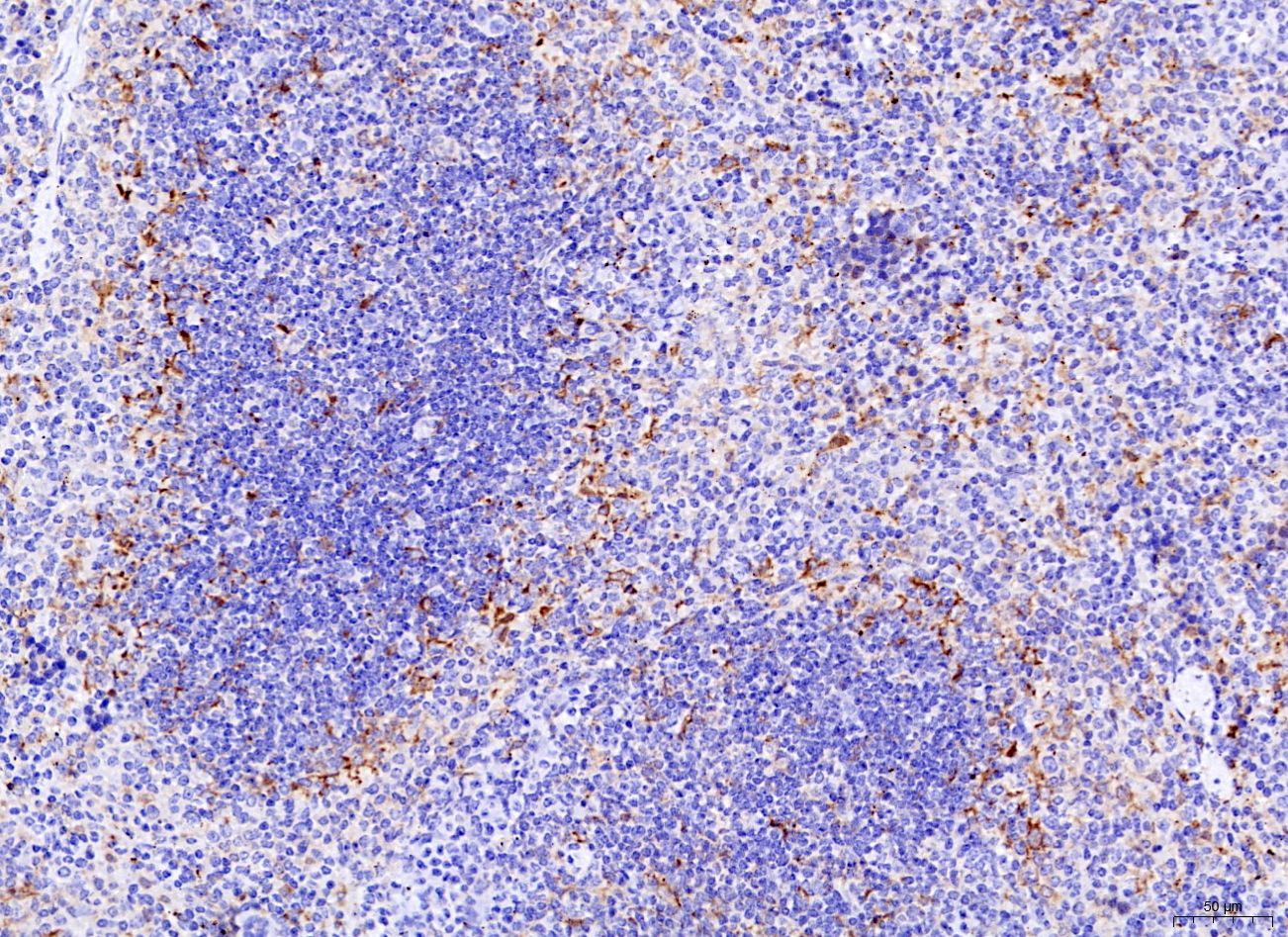

Supplement: Supplementary file 3 — Supporting File 3: advs73867‐sup‐0003‐SupportingFiguresData.zip. [file ADVS-13-e19191-s003.zip › Supporting information Figure S1-S9/S2/Spleen/SCRS 1week Model 757_20.0x-1.jpg]

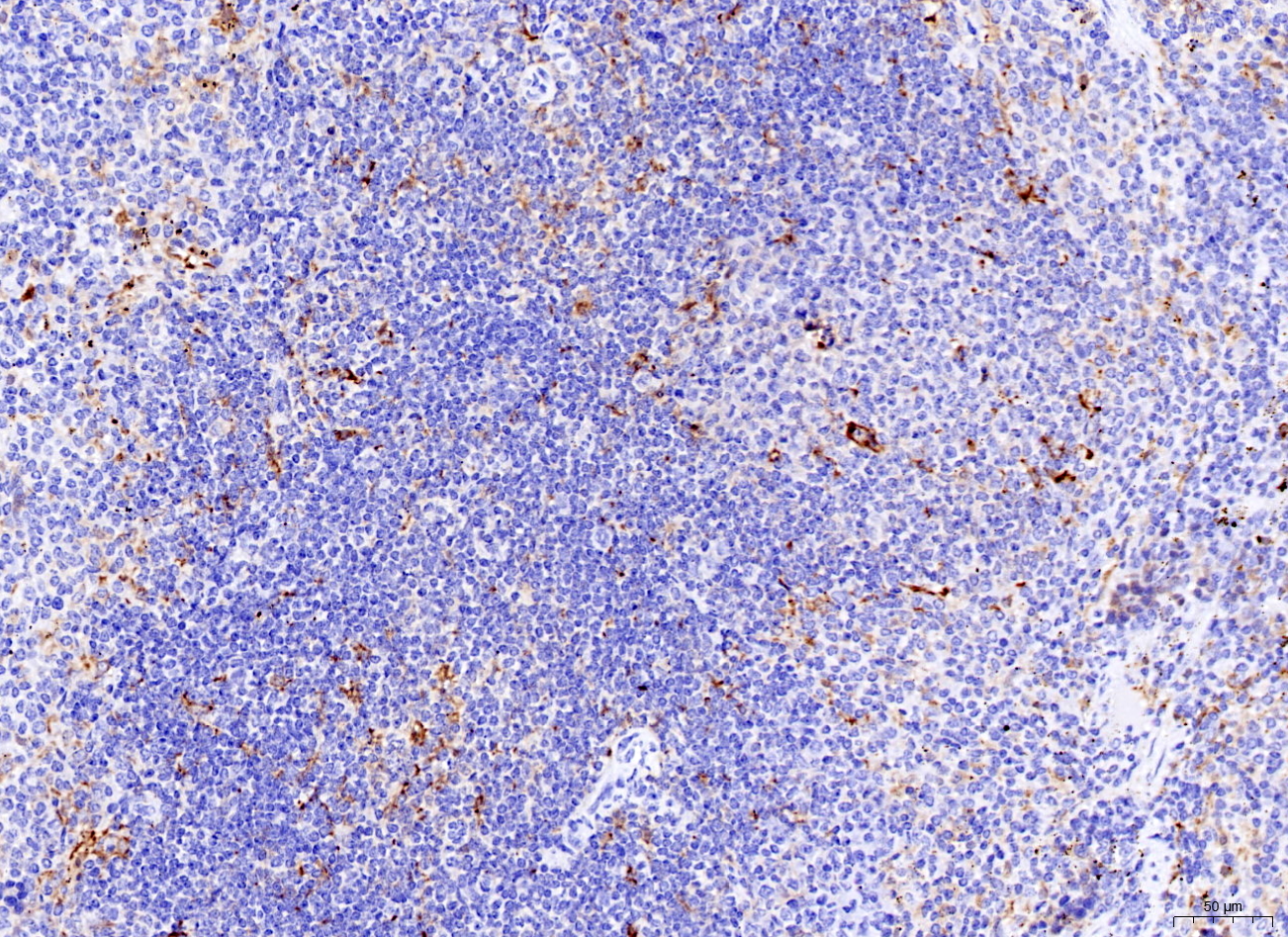

Supplement: Supplementary file 3 — Supporting File 3: advs73867‐sup‐0003‐SupportingFiguresData.zip. [file ADVS-13-e19191-s003.zip › Supporting information Figure S1-S9/S2/Spleen/SCRS 1week Model 757_20.0x-2.jpg]

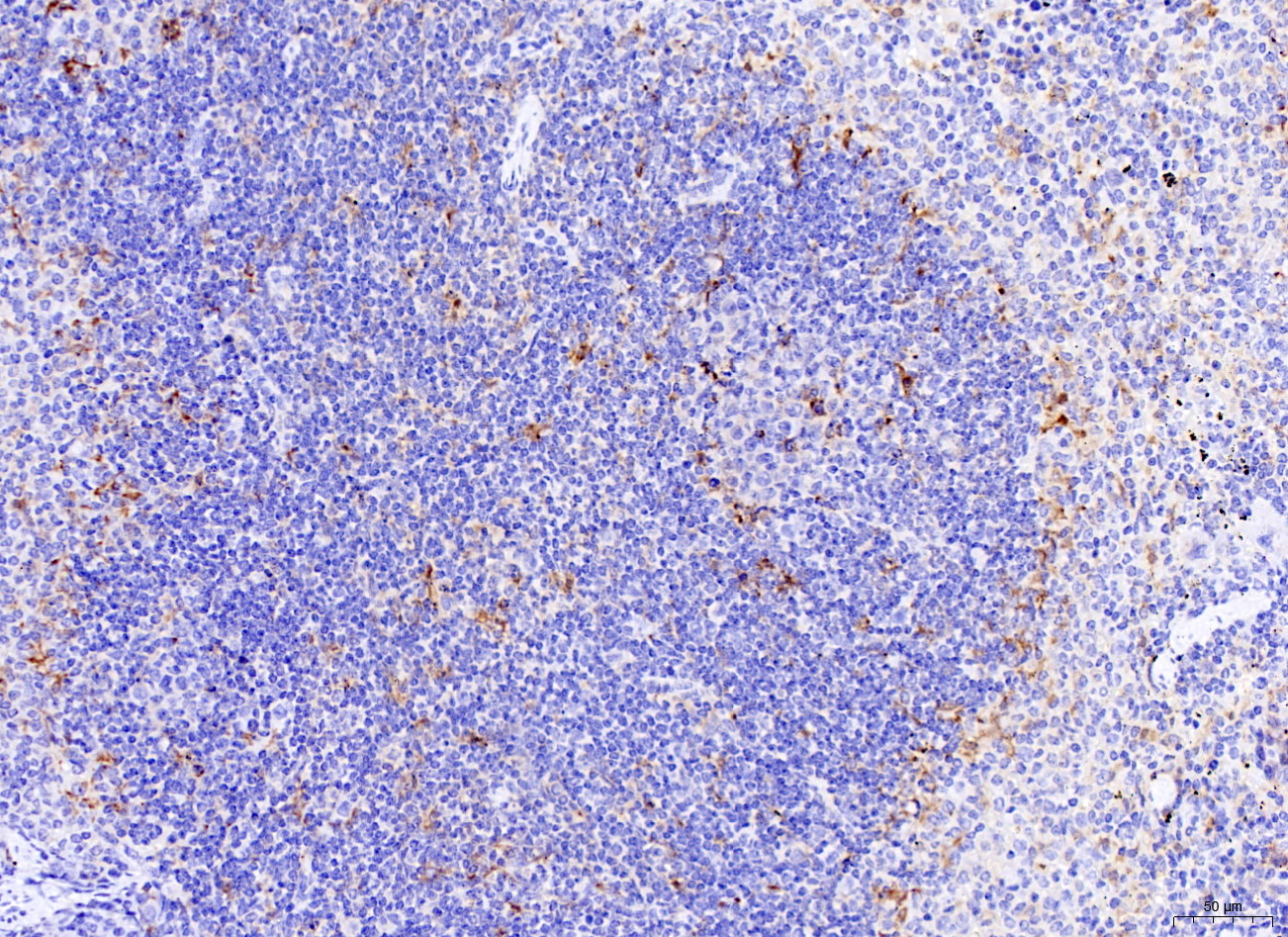

Supplement: Supplementary file 3 — Supporting File 3: advs73867‐sup‐0003‐SupportingFiguresData.zip. [file ADVS-13-e19191-s003.zip › Supporting information Figure S1-S9/S2/Spleen/SCRS 1week Model 757_20.0x-3.jpg]

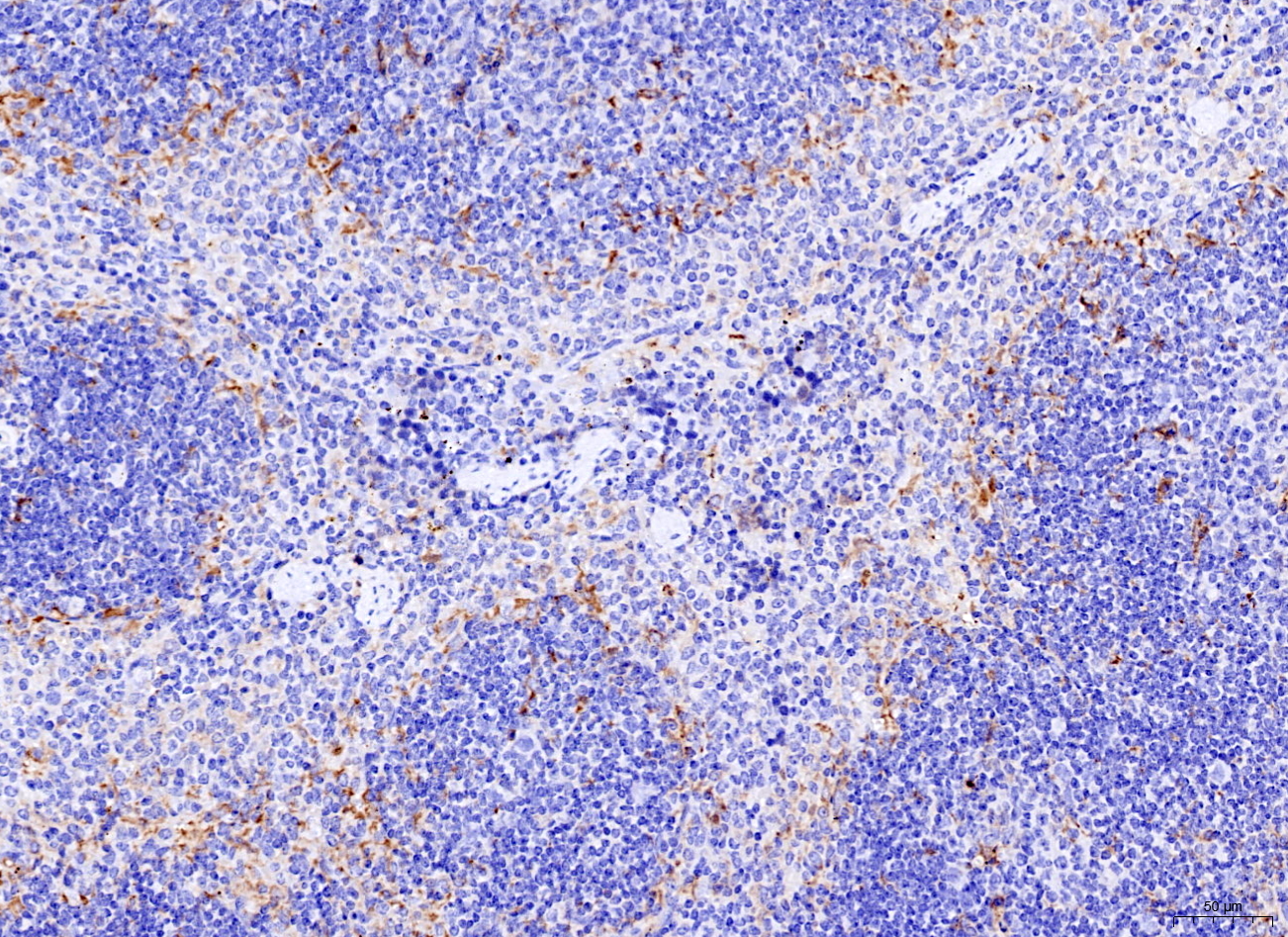

Supplement: Supplementary file 3 — Supporting File 3: advs73867‐sup‐0003‐SupportingFiguresData.zip. [file ADVS-13-e19191-s003.zip › Supporting information Figure S1-S9/S2/Spleen/SCRS 1week Model 757_20.0x-4.jpg]

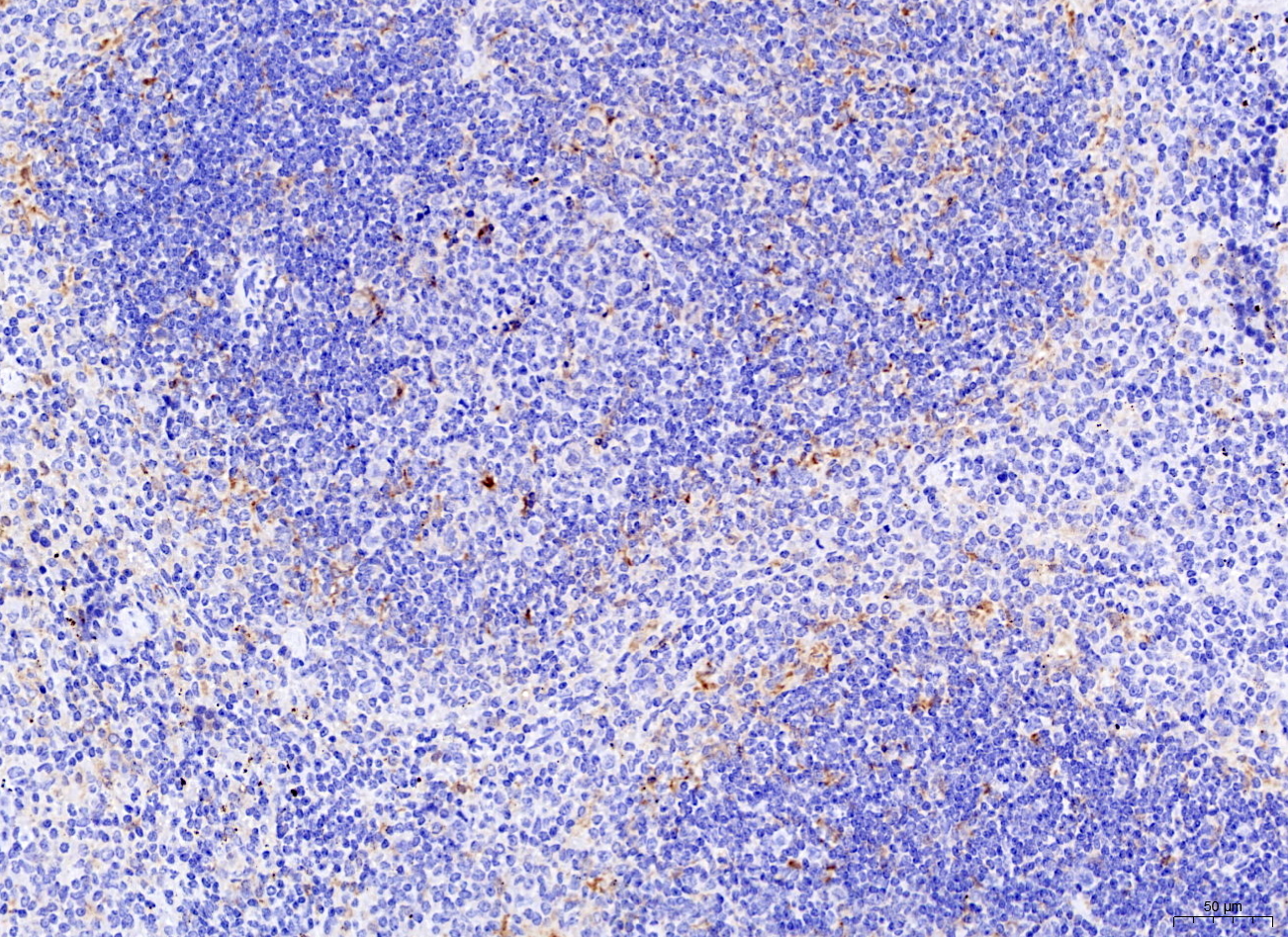

Supplement: Supplementary file 3 — Supporting File 3: advs73867‐sup‐0003‐SupportingFiguresData.zip. [file ADVS-13-e19191-s003.zip › Supporting information Figure S1-S9/S2/Spleen/SCRS 1week Model 757_20.0x-5.jpg]

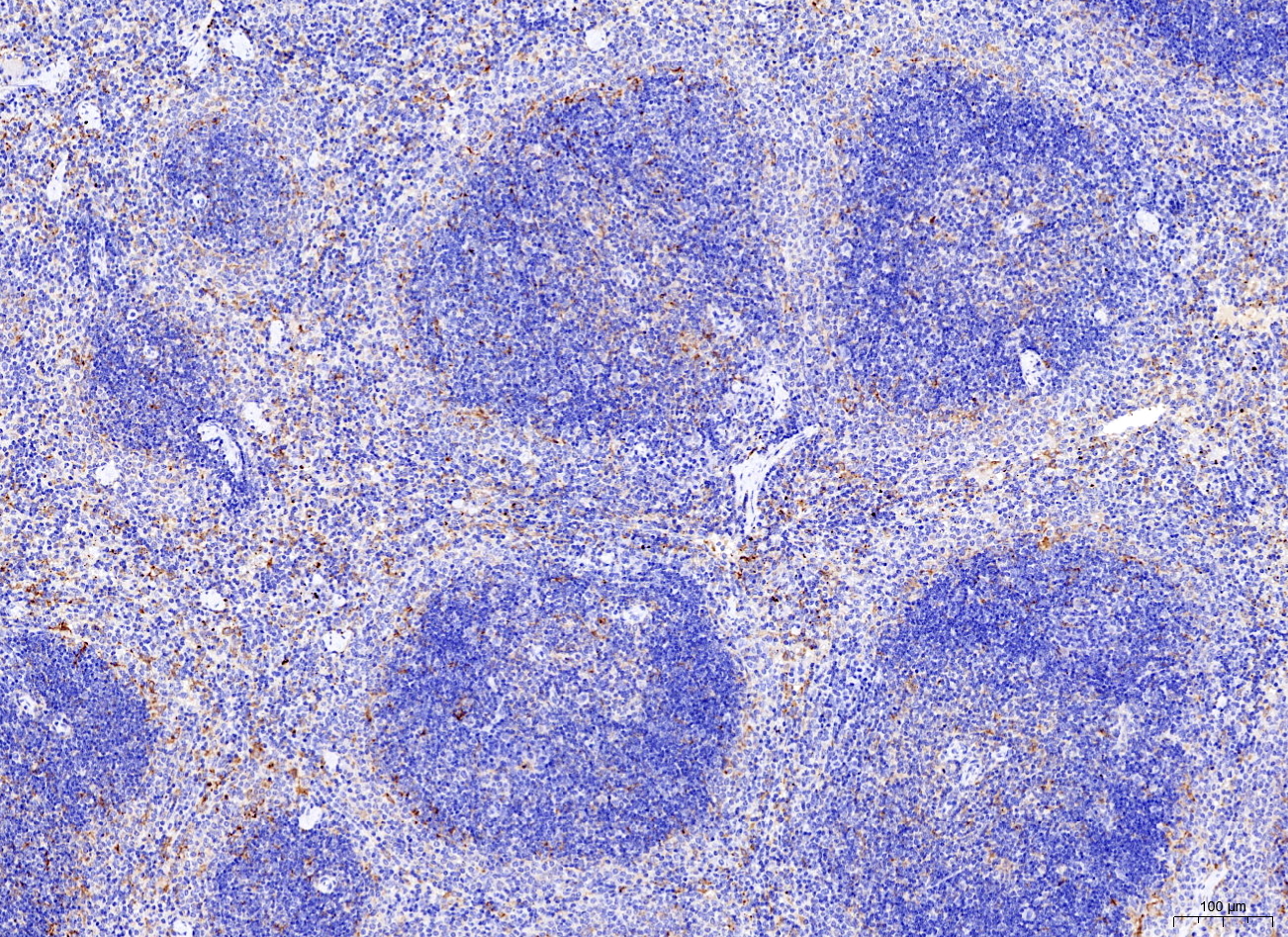

Supplement: Supplementary file 3 — Supporting File 3: advs73867‐sup‐0003‐SupportingFiguresData.zip. [file ADVS-13-e19191-s003.zip › Supporting information Figure S1-S9/S2/Spleen/SCRS 4week Model 744_10.0x.jpg]

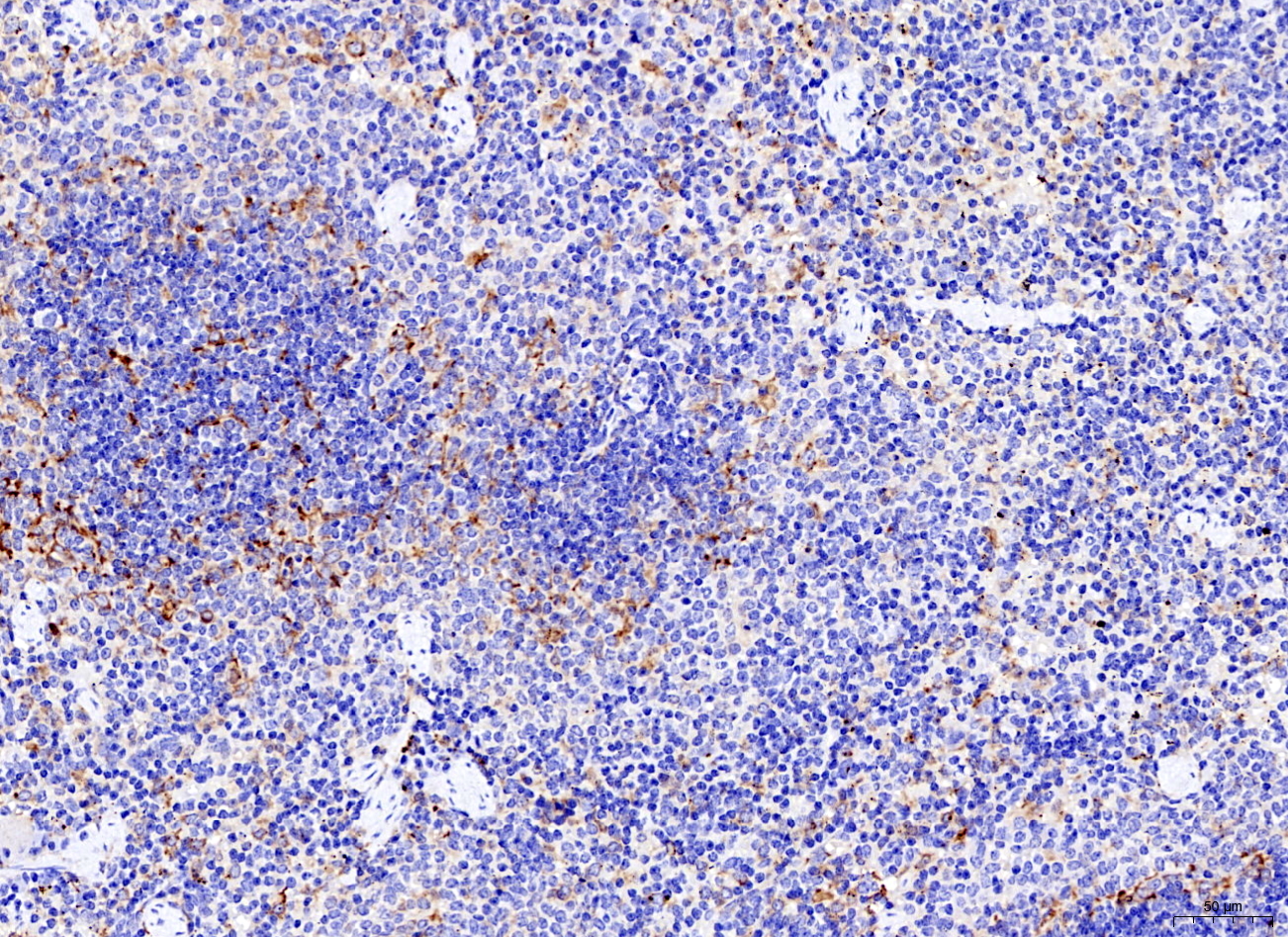

Supplement: Supplementary file 3 — Supporting File 3: advs73867‐sup‐0003‐SupportingFiguresData.zip. [file ADVS-13-e19191-s003.zip › Supporting information Figure S1-S9/S2/Spleen/SCRS 4week Model 744_20.0x-1.jpg]

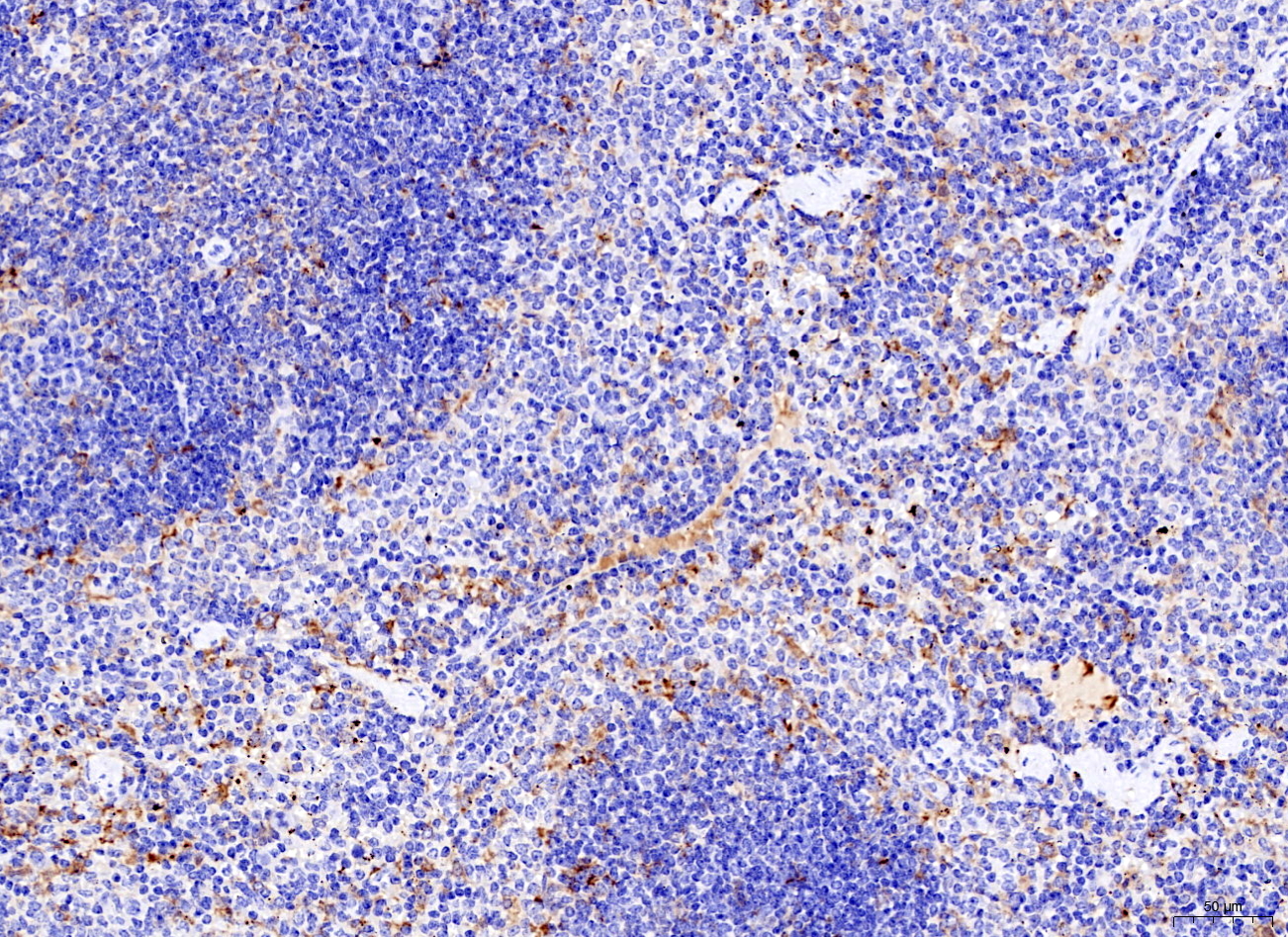

Supplement: Supplementary file 3 — Supporting File 3: advs73867‐sup‐0003‐SupportingFiguresData.zip. [file ADVS-13-e19191-s003.zip › Supporting information Figure S1-S9/S2/Spleen/SCRS 4week Model 744_20.0x-2.jpg]

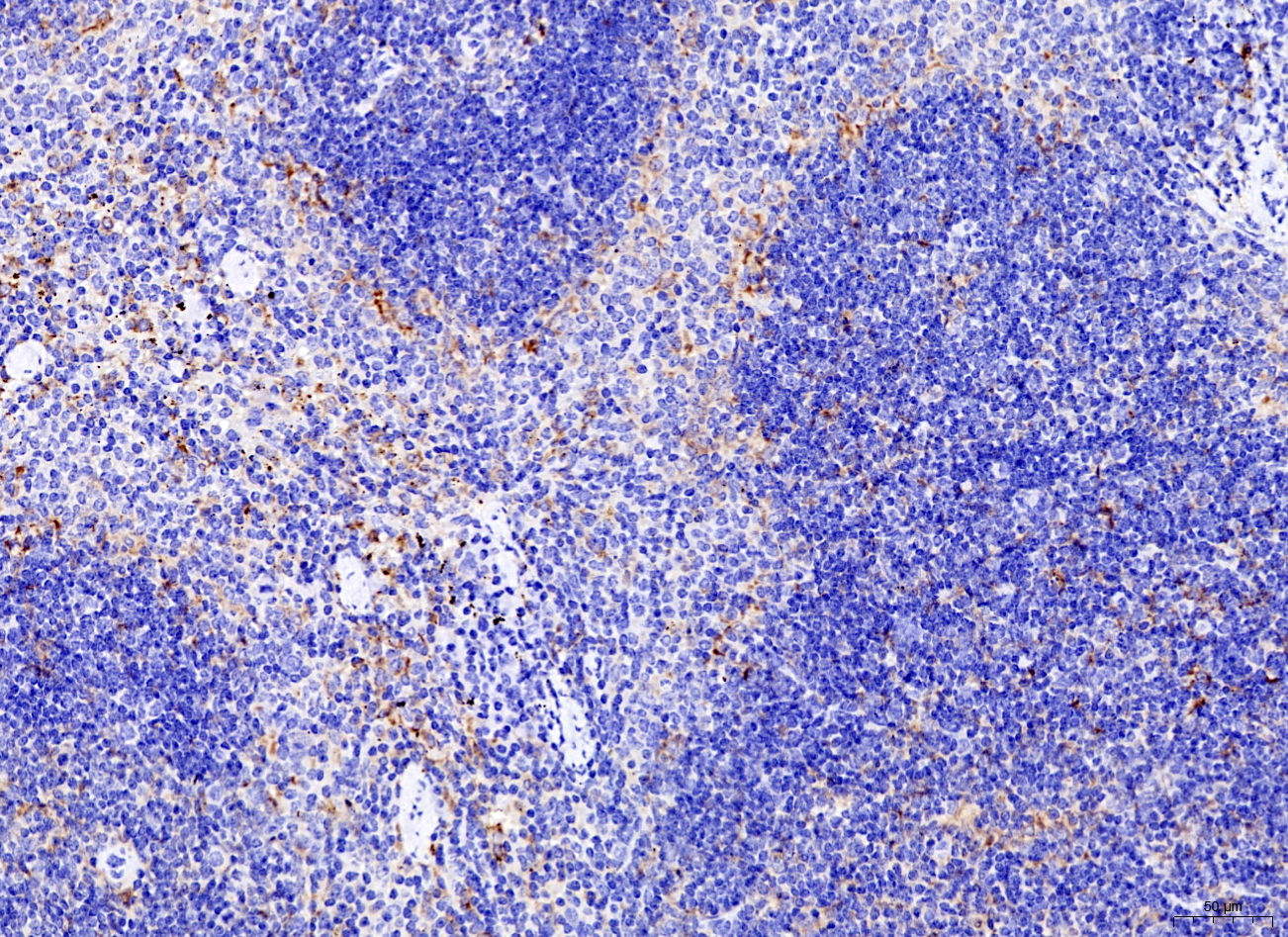

Supplement: Supplementary file 3 — Supporting File 3: advs73867‐sup‐0003‐SupportingFiguresData.zip. [file ADVS-13-e19191-s003.zip › Supporting information Figure S1-S9/S2/Spleen/SCRS 4week Model 744_20.0x-3.jpg]

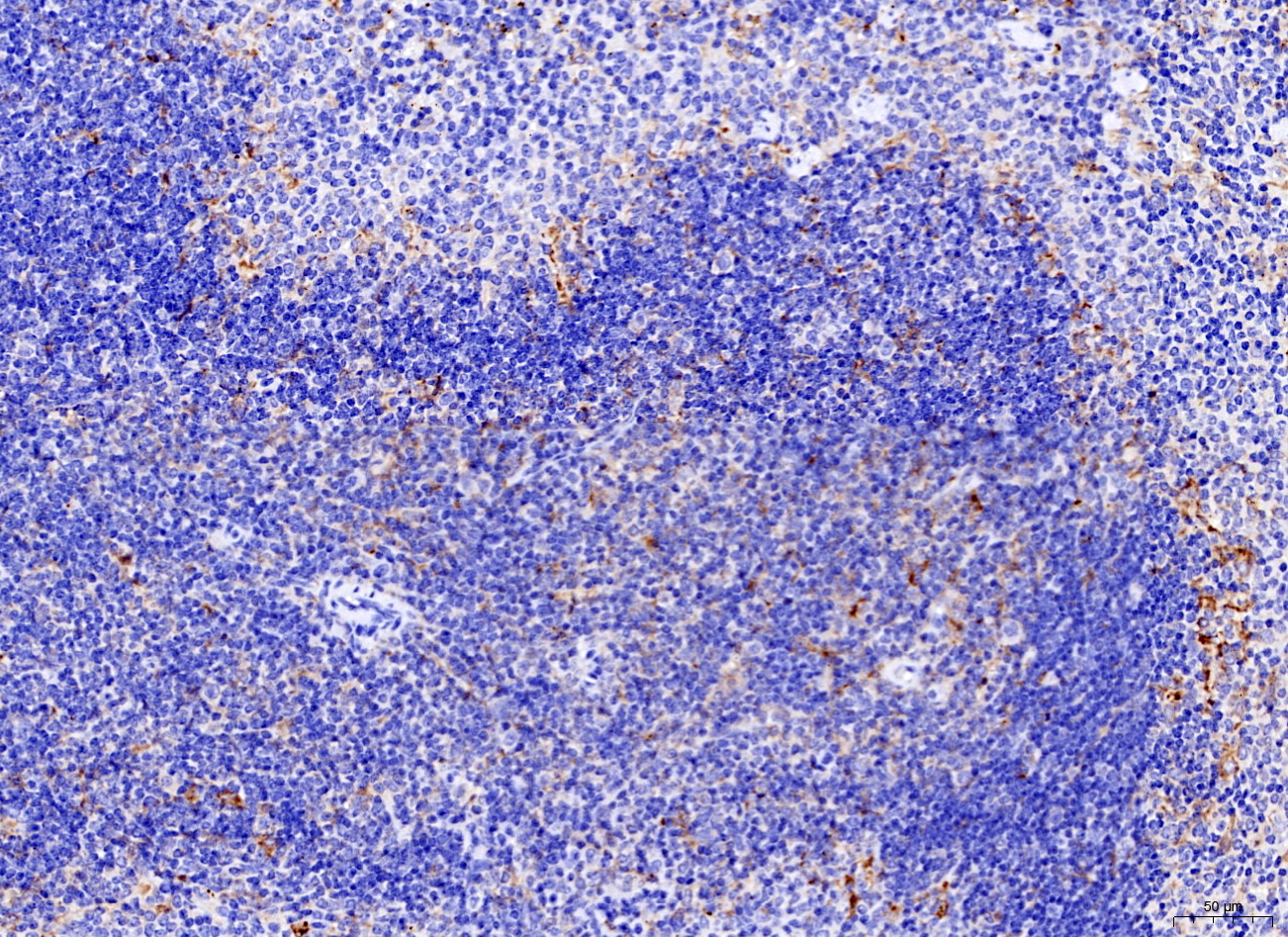

Supplement: Supplementary file 3 — Supporting File 3: advs73867‐sup‐0003‐SupportingFiguresData.zip. [file ADVS-13-e19191-s003.zip › Supporting information Figure S1-S9/S2/Spleen/SCRS 4week Model 744_20.0x-4.jpg]

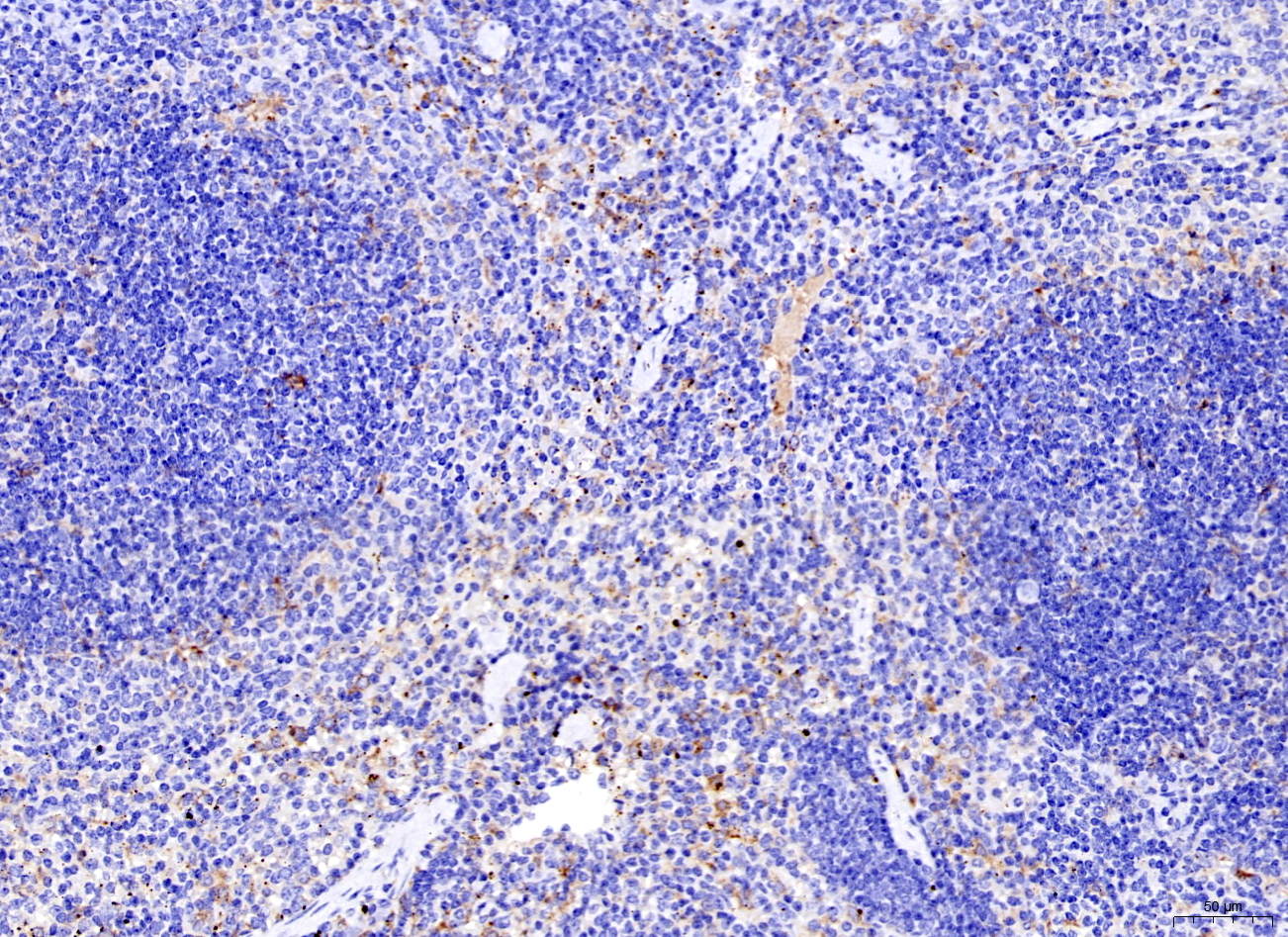

Supplement: Supplementary file 3 — Supporting File 3: advs73867‐sup‐0003‐SupportingFiguresData.zip. [file ADVS-13-e19191-s003.zip › Supporting information Figure S1-S9/S2/Spleen/SCRS 4week Model 744_20.0x-5.jpg]

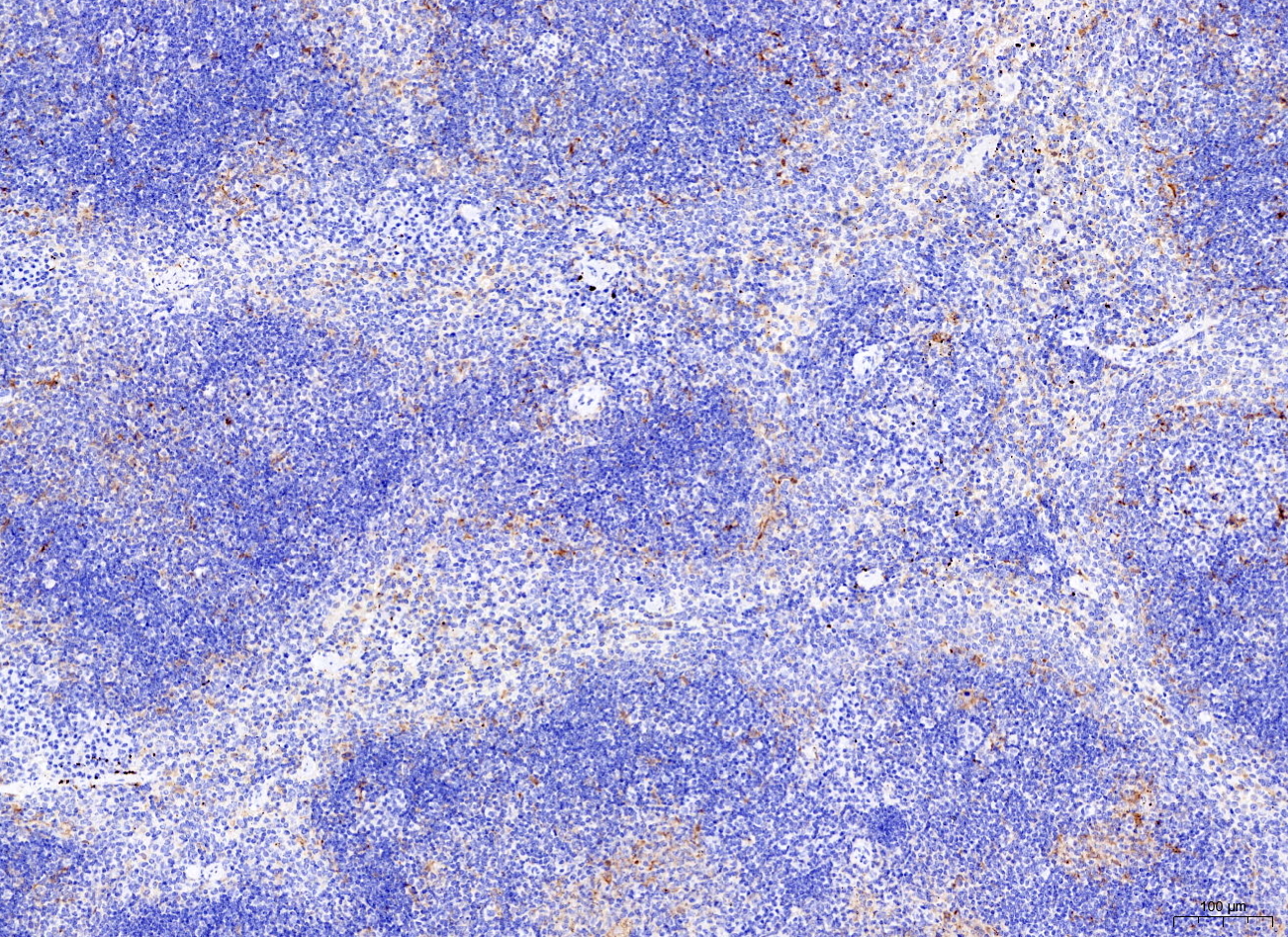

Supplement: Supplementary file 3 — Supporting File 3: advs73867‐sup‐0003‐SupportingFiguresData.zip. [file ADVS-13-e19191-s003.zip › Supporting information Figure S1-S9/S2/Spleen/SCRS 8week Model 763_10.0x.jpg]

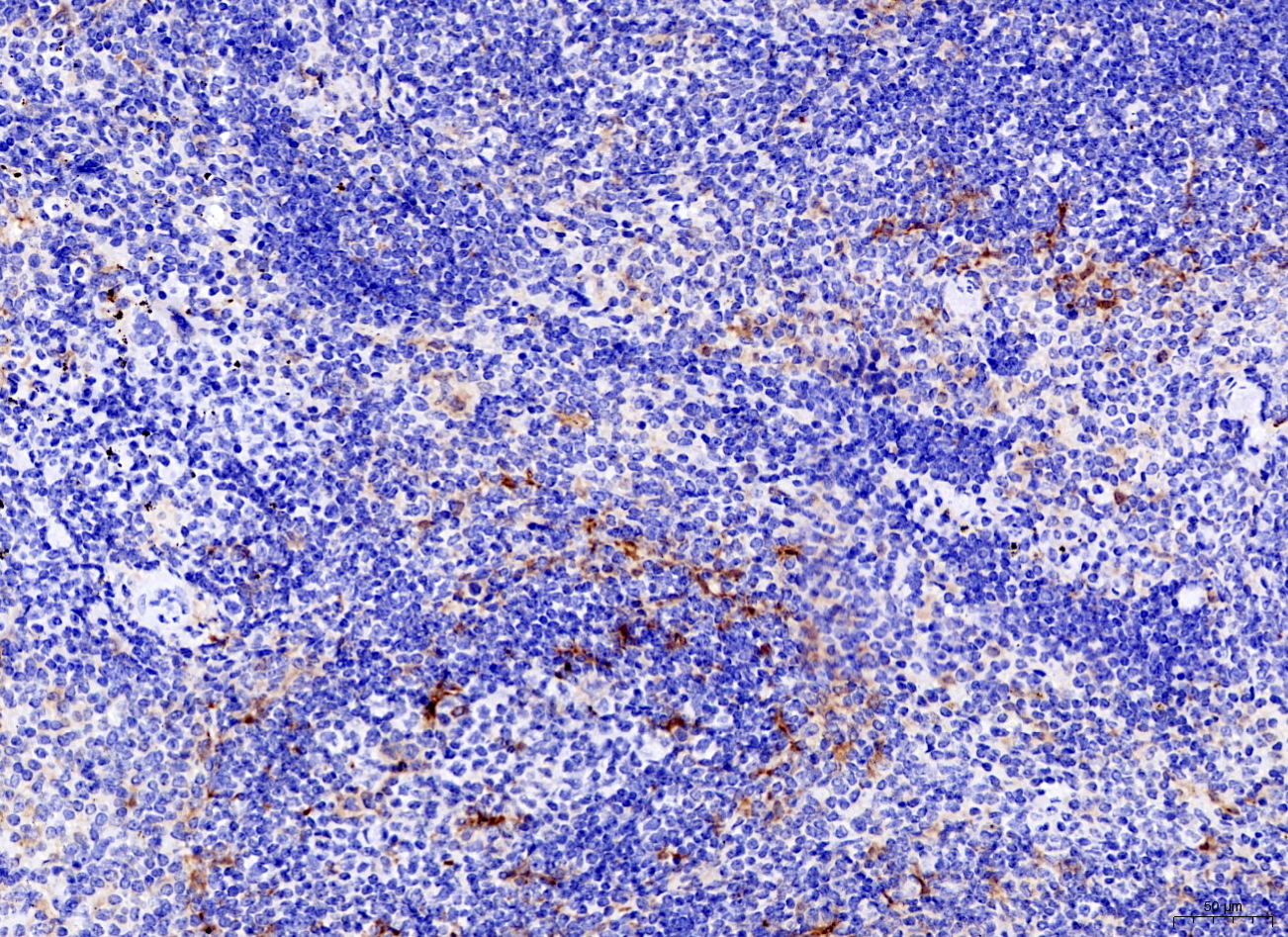

Supplement: Supplementary file 3 — Supporting File 3: advs73867‐sup‐0003‐SupportingFiguresData.zip. [file ADVS-13-e19191-s003.zip › Supporting information Figure S1-S9/S2/Spleen/SCRS 8week Model 763_20.0x-1.jpg]

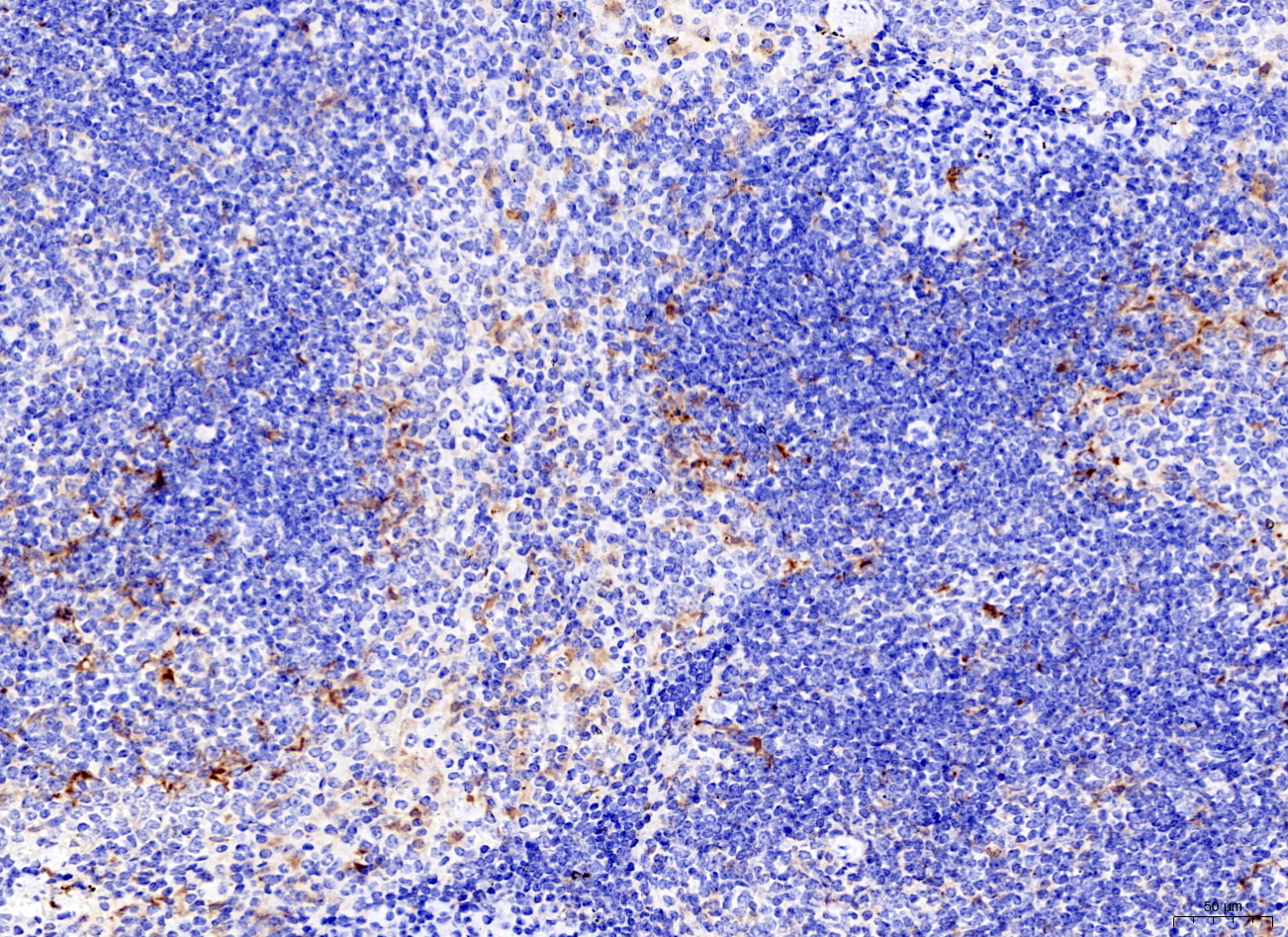

Supplement: Supplementary file 3 — Supporting File 3: advs73867‐sup‐0003‐SupportingFiguresData.zip. [file ADVS-13-e19191-s003.zip › Supporting information Figure S1-S9/S2/Spleen/SCRS 8week Model 763_20.0x-2.jpg]

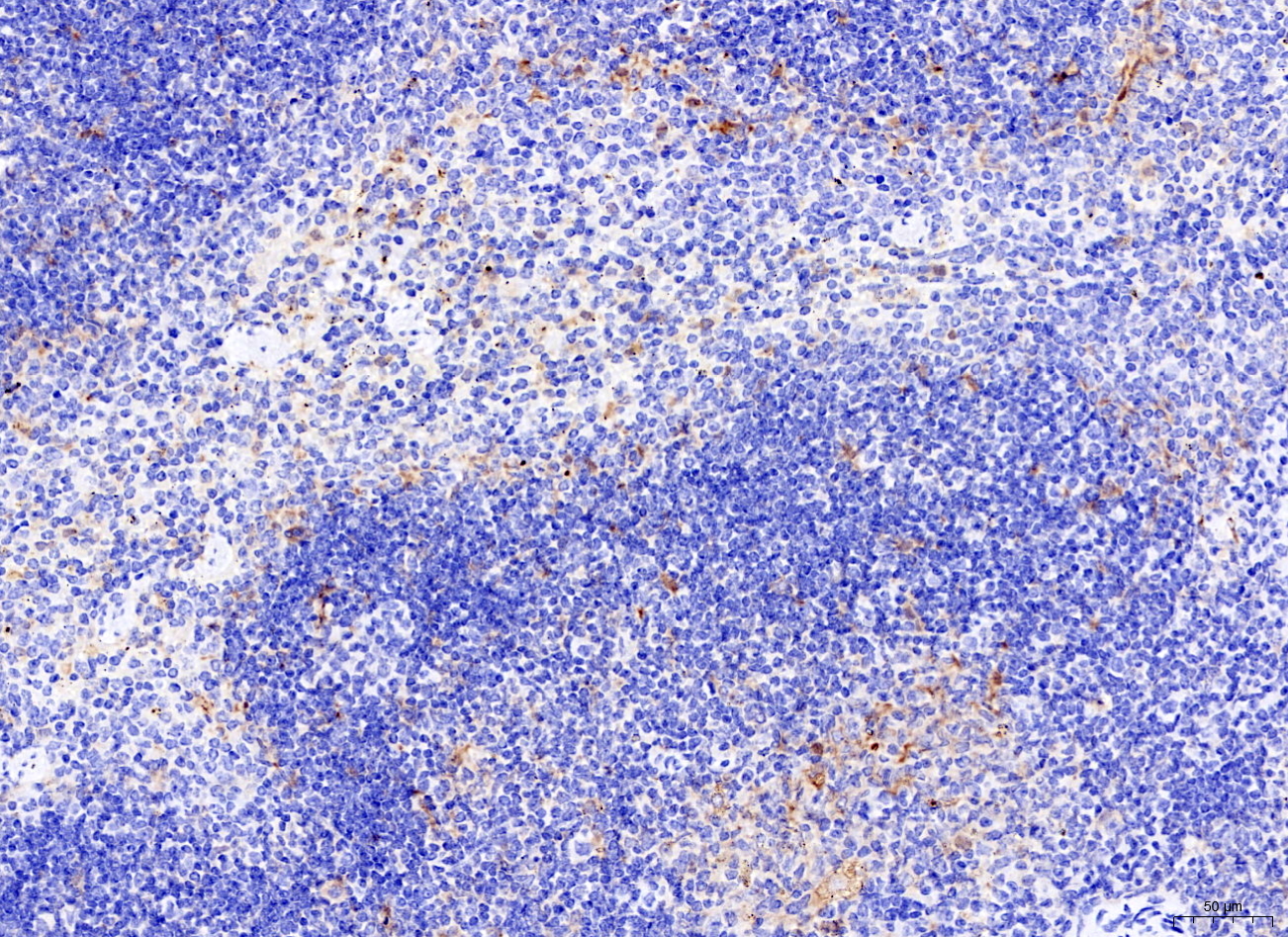

Supplement: Supplementary file 3 — Supporting File 3: advs73867‐sup‐0003‐SupportingFiguresData.zip. [file ADVS-13-e19191-s003.zip › Supporting information Figure S1-S9/S2/Spleen/SCRS 8week Model 763_20.0x-3.jpg]

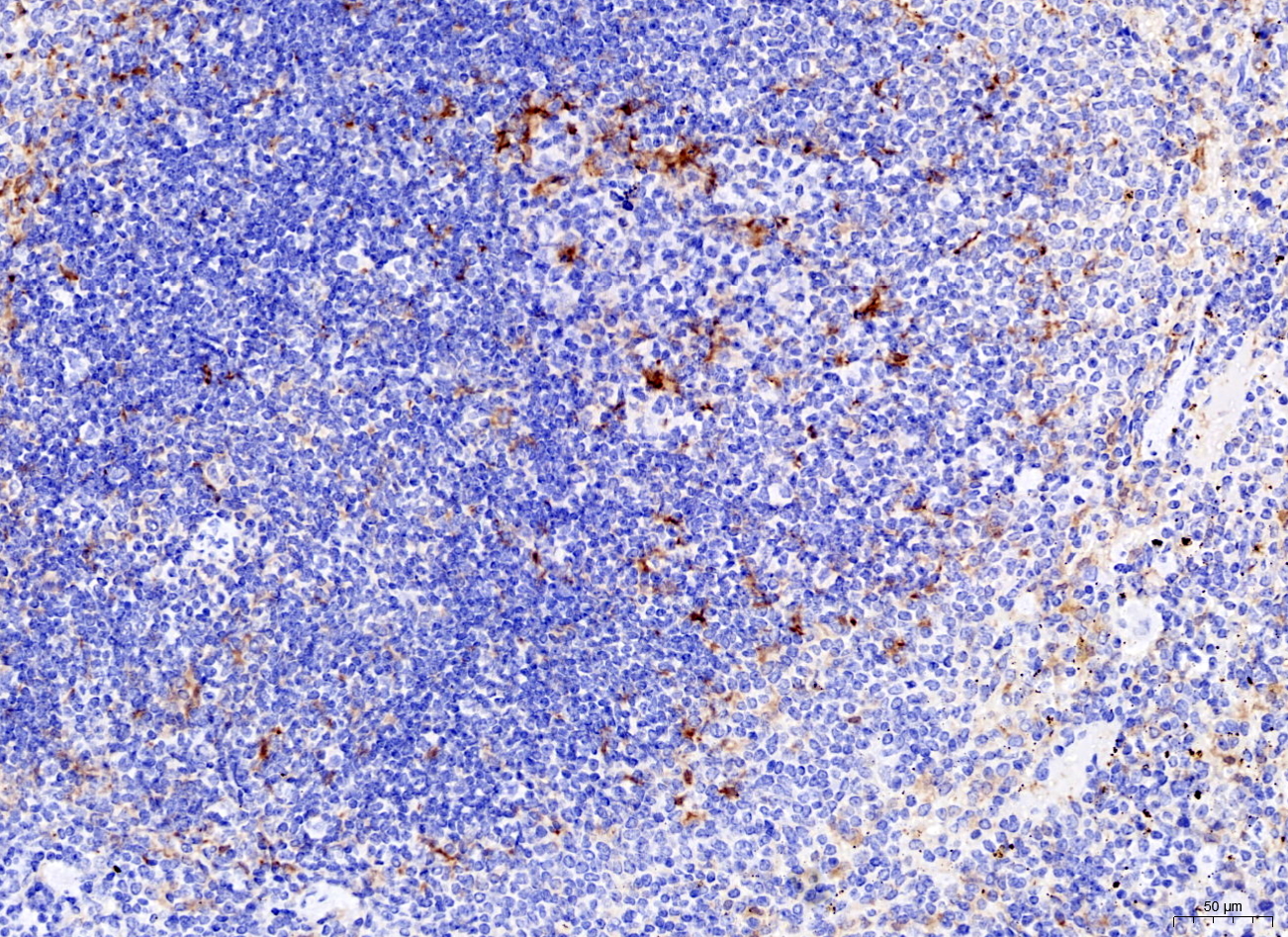

Supplement: Supplementary file 3 — Supporting File 3: advs73867‐sup‐0003‐SupportingFiguresData.zip. [file ADVS-13-e19191-s003.zip › Supporting information Figure S1-S9/S2/Spleen/SCRS 8week Model 763_20.0x-4.jpg]

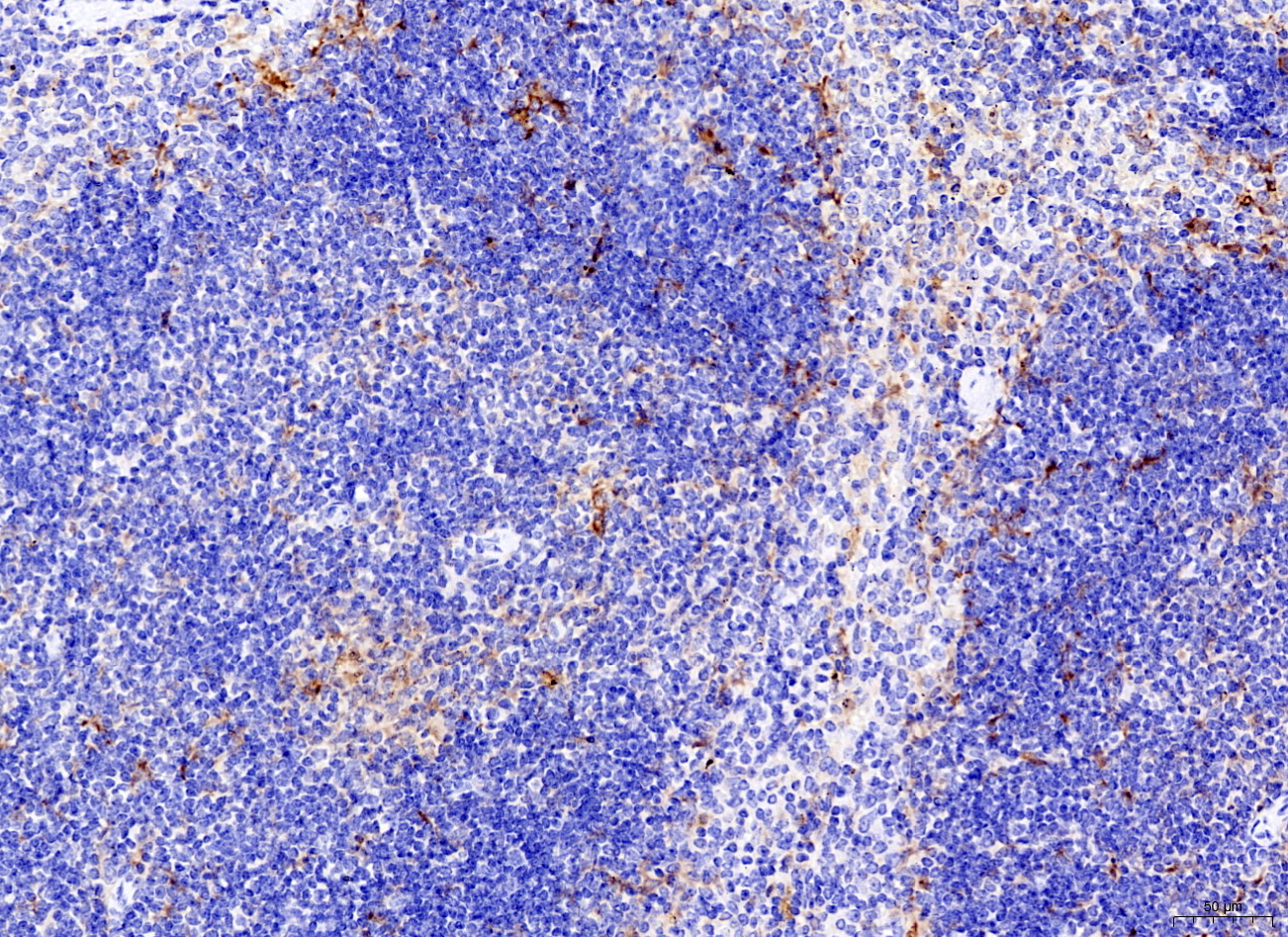

Supplement: Supplementary file 3 — Supporting File 3: advs73867‐sup‐0003‐SupportingFiguresData.zip. [file ADVS-13-e19191-s003.zip › Supporting information Figure S1-S9/S2/Spleen/SCRS 8week Model 763_20.0x-5.jpg]

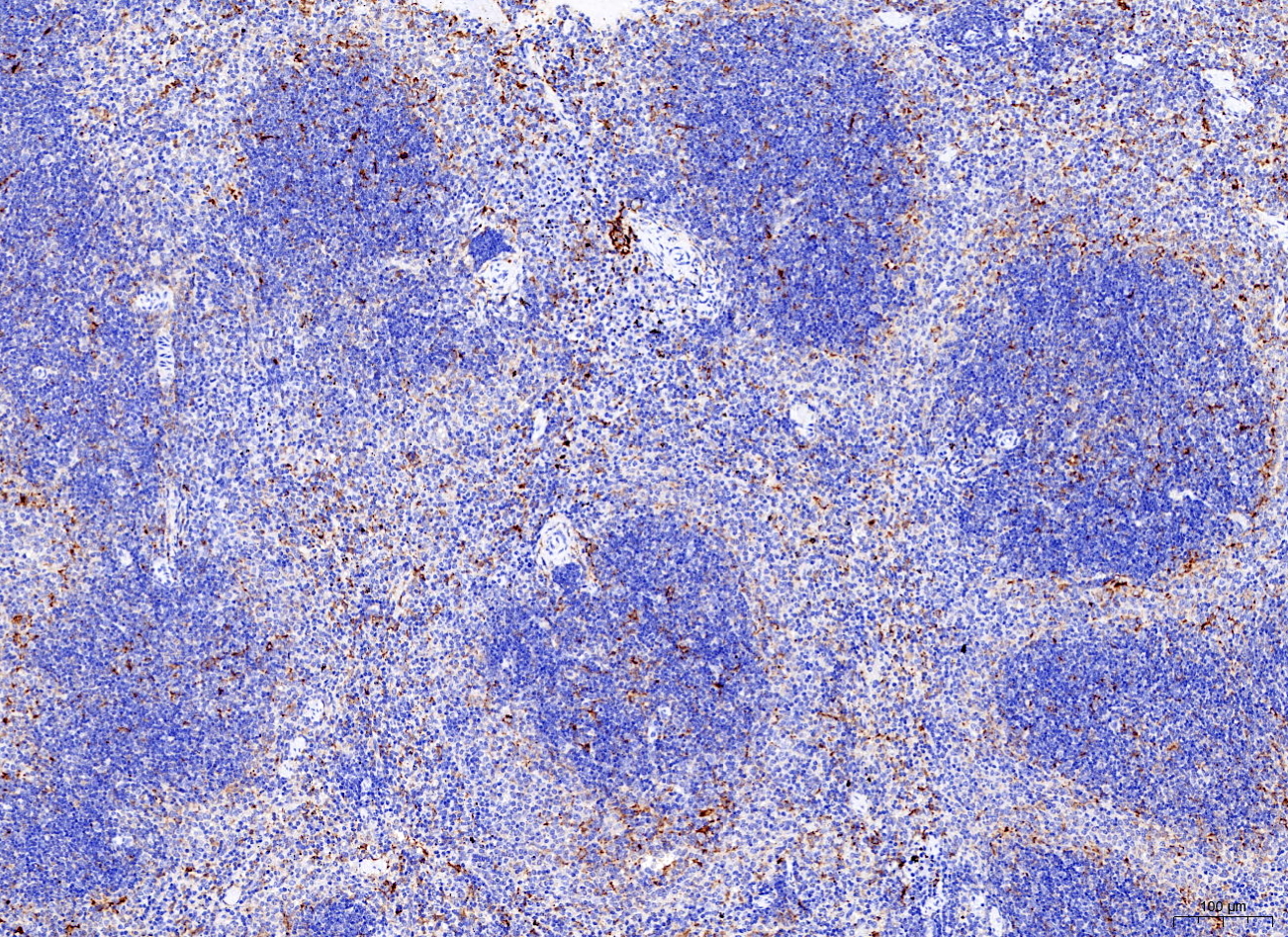

Supplement: Supplementary file 3 — Supporting File 3: advs73867‐sup‐0003‐SupportingFiguresData.zip. [file ADVS-13-e19191-s003.zip › Supporting information Figure S1-S9/S2/Spleen/SCRS Control 782_10.0x.jpg]

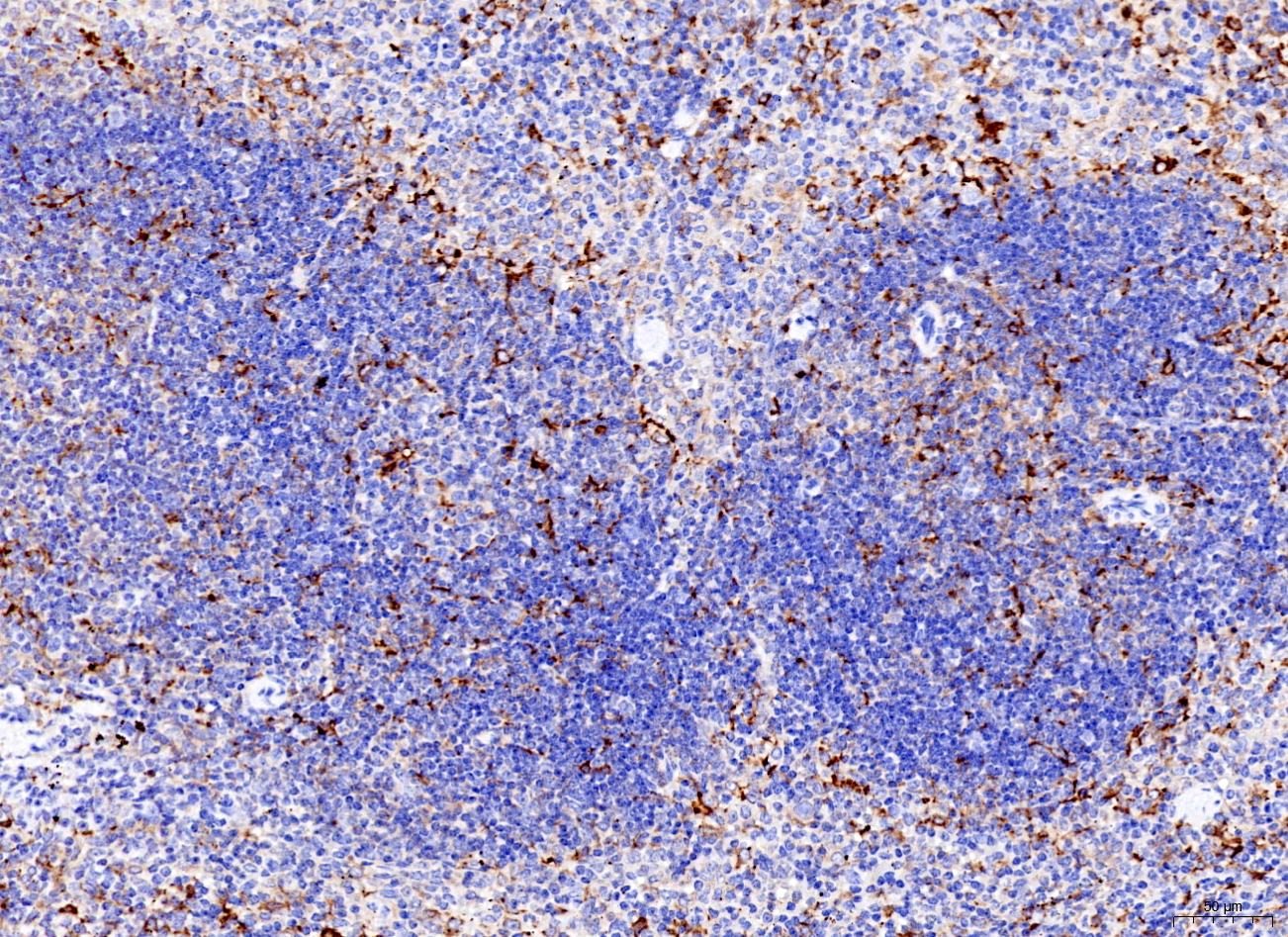

Supplement: Supplementary file 3 — Supporting File 3: advs73867‐sup‐0003‐SupportingFiguresData.zip. [file ADVS-13-e19191-s003.zip › Supporting information Figure S1-S9/S2/Spleen/SCRS Control 782_20.0x-1.jpg]

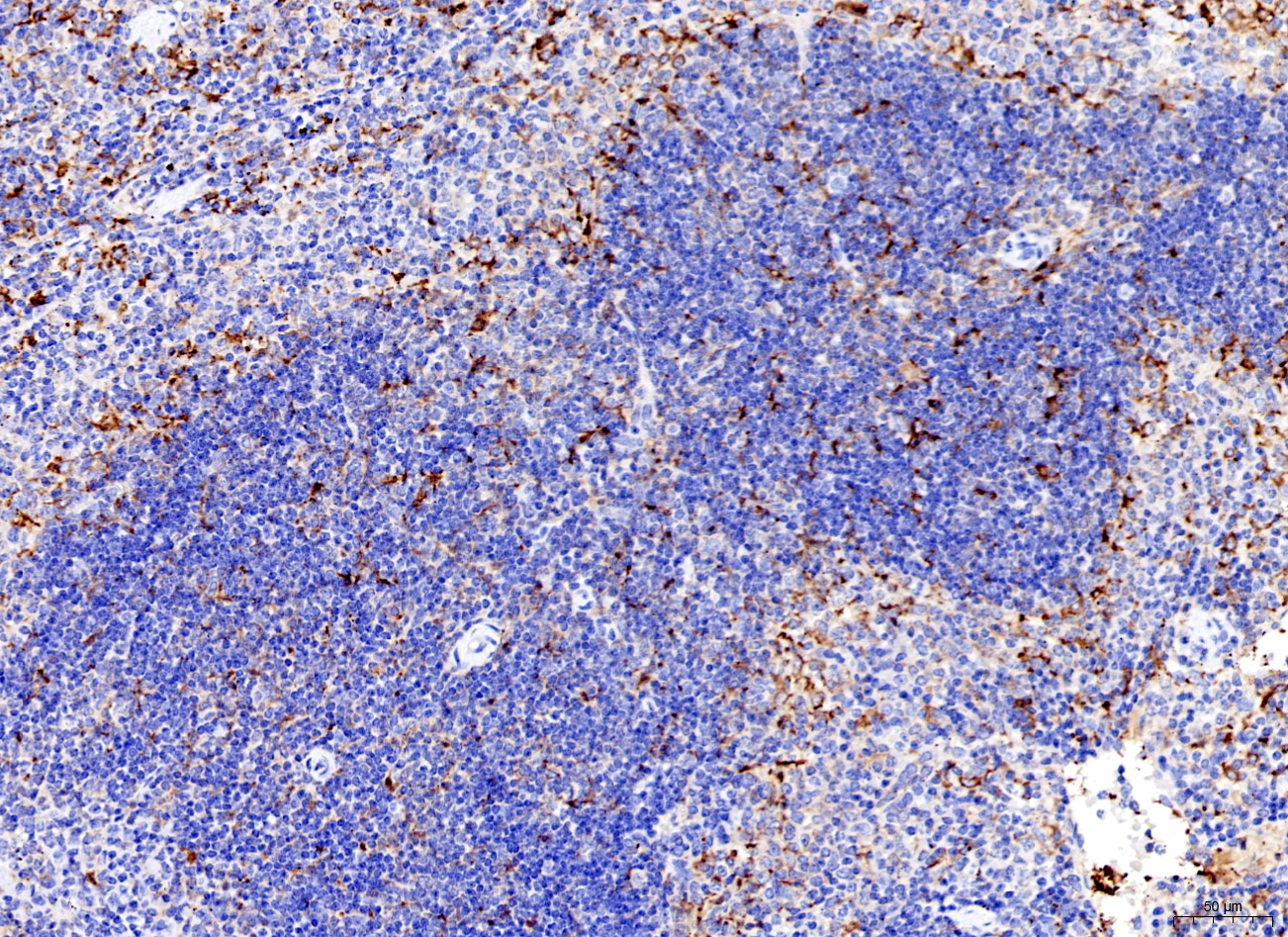

Supplement: Supplementary file 3 — Supporting File 3: advs73867‐sup‐0003‐SupportingFiguresData.zip. [file ADVS-13-e19191-s003.zip › Supporting information Figure S1-S9/S2/Spleen/SCRS Control 782_20.0x-2.jpg]

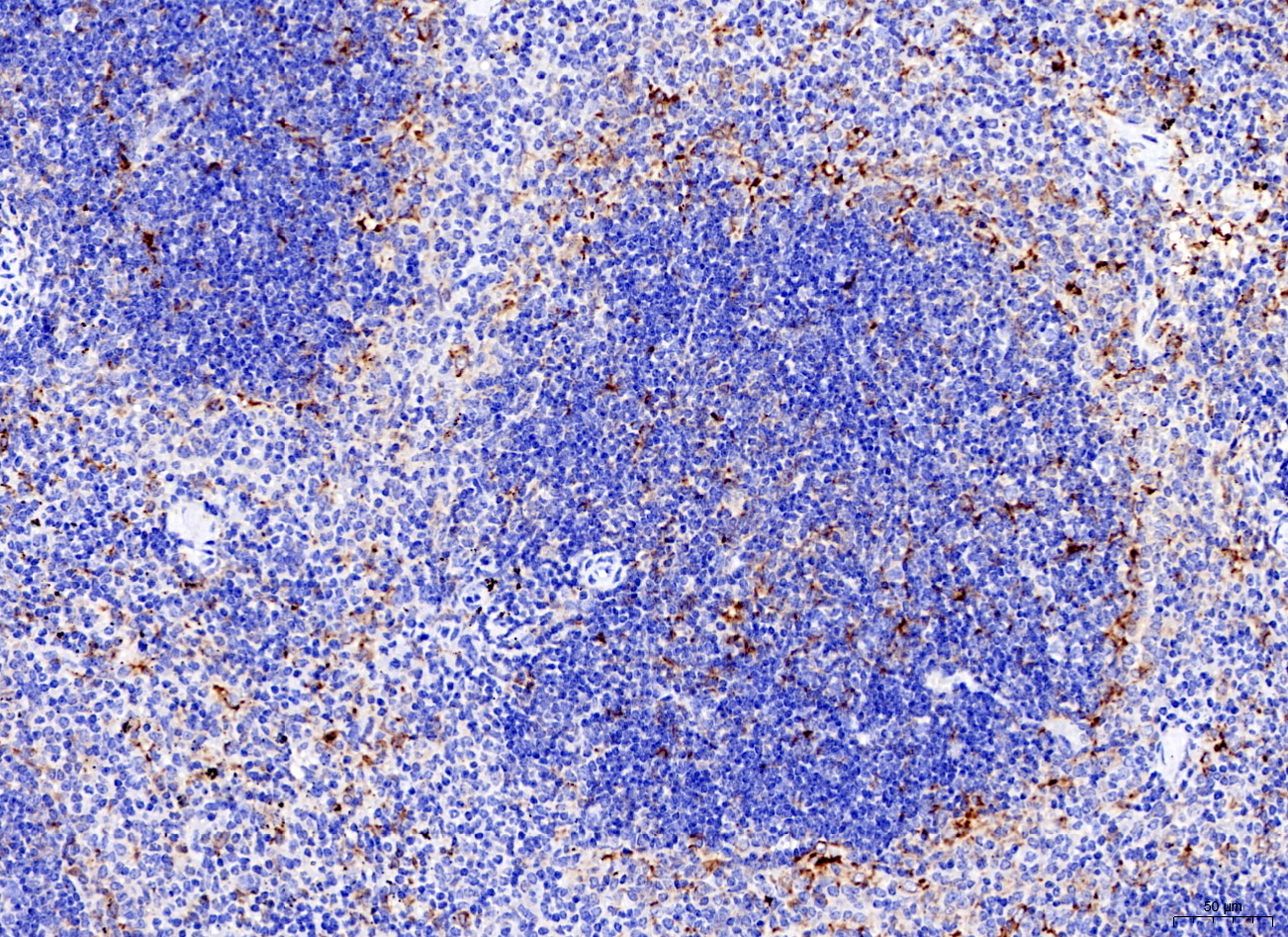

Supplement: Supplementary file 3 — Supporting File 3: advs73867‐sup‐0003‐SupportingFiguresData.zip. [file ADVS-13-e19191-s003.zip › Supporting information Figure S1-S9/S2/Spleen/SCRS Control 782_20.0x-3.jpg]

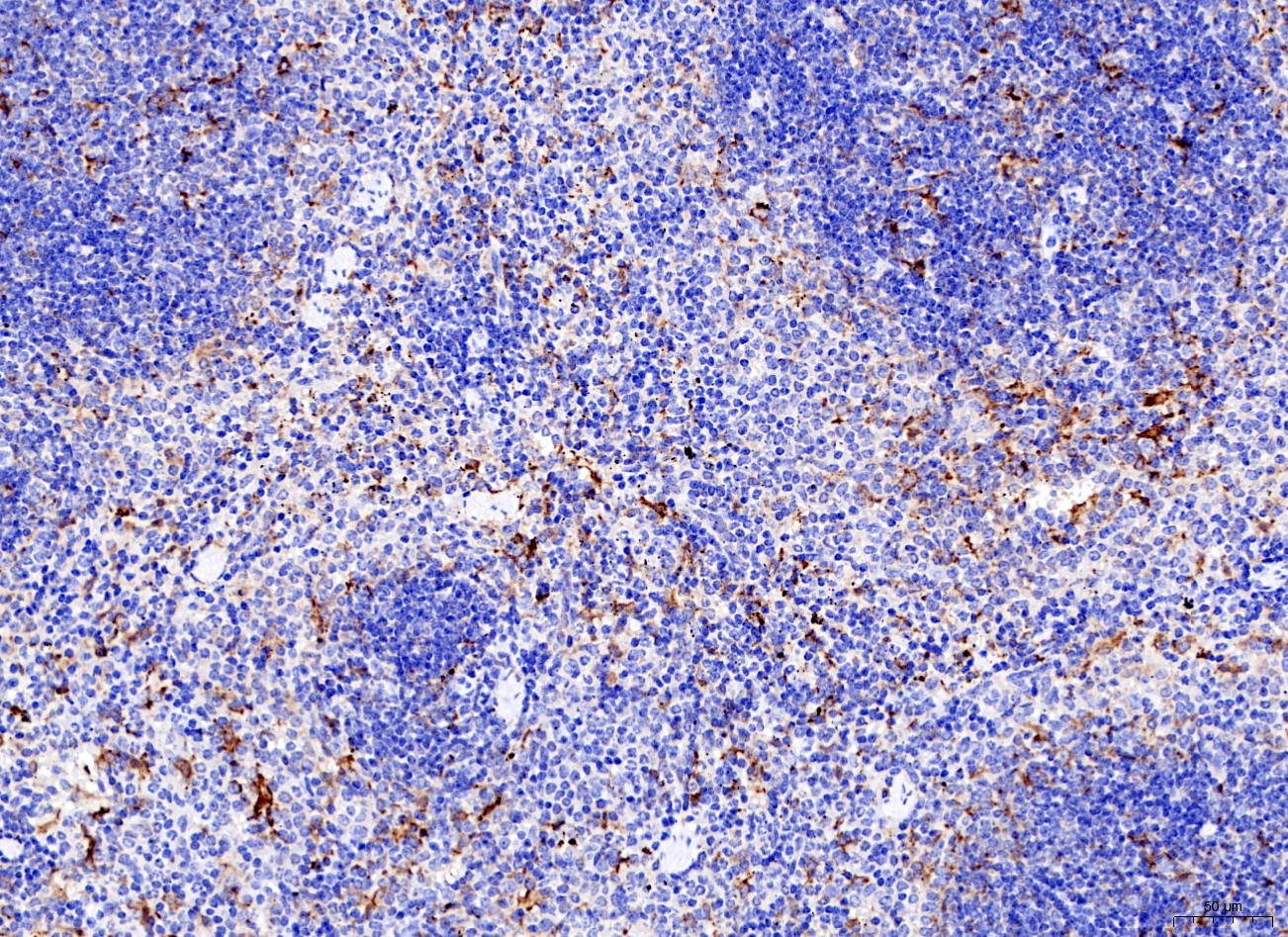

Supplement: Supplementary file 3 — Supporting File 3: advs73867‐sup‐0003‐SupportingFiguresData.zip. [file ADVS-13-e19191-s003.zip › Supporting information Figure S1-S9/S2/Spleen/SCRS Control 782_20.0x-4.jpg]

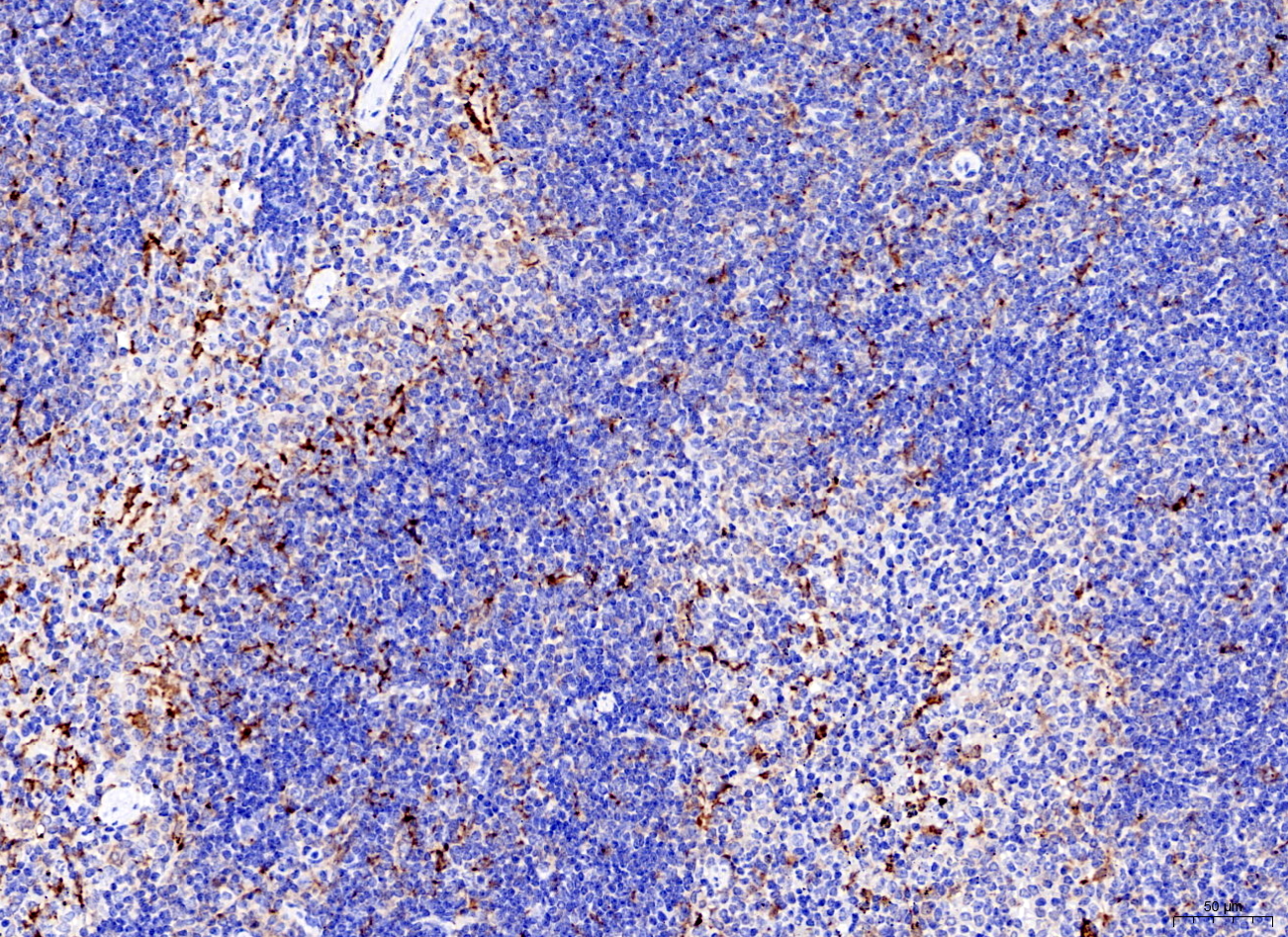

Supplement: Supplementary file 3 — Supporting File 3: advs73867‐sup‐0003‐SupportingFiguresData.zip. [file ADVS-13-e19191-s003.zip › Supporting information Figure S1-S9/S2/Spleen/SCRS Control 782_20.0x-5.jpg]

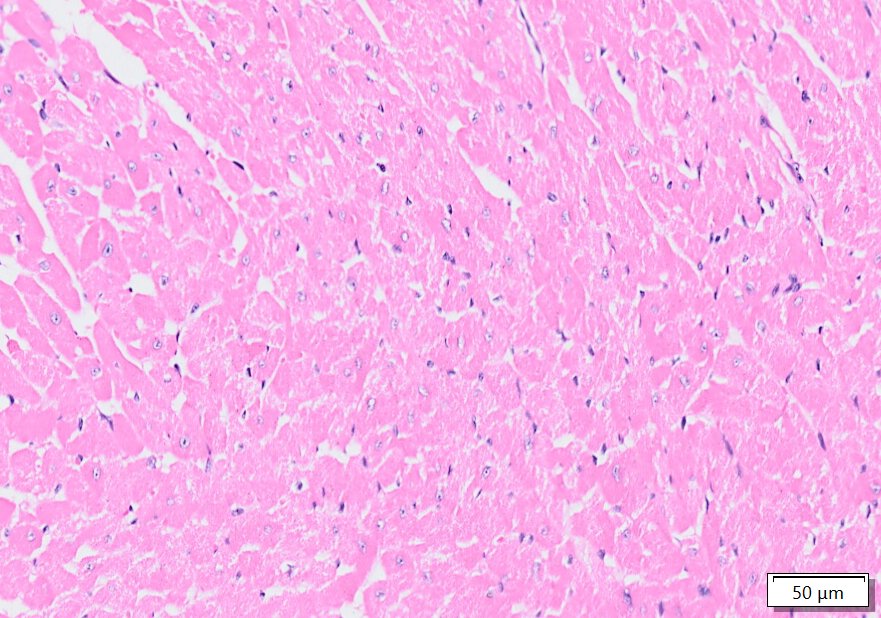

Supplement: Supplementary file 3 — Supporting File 3: advs73867‐sup‐0003‐SupportingFiguresData.zip. [file ADVS-13-e19191-s003.zip › Supporting information Figure S1-S9/S3/Figure S3C/Control-126-Heart-10í┴.jpg]

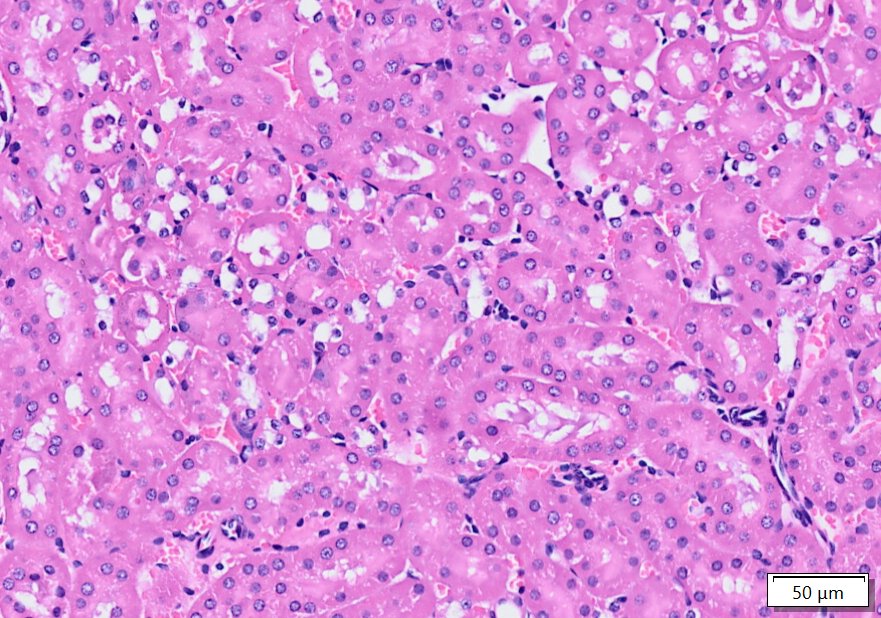

Supplement: Supplementary file 3 — Supporting File 3: advs73867‐sup‐0003‐SupportingFiguresData.zip. [file ADVS-13-e19191-s003.zip › Supporting information Figure S1-S9/S3/Figure S3C/Control-126-Kidney-10í┴.jpg]

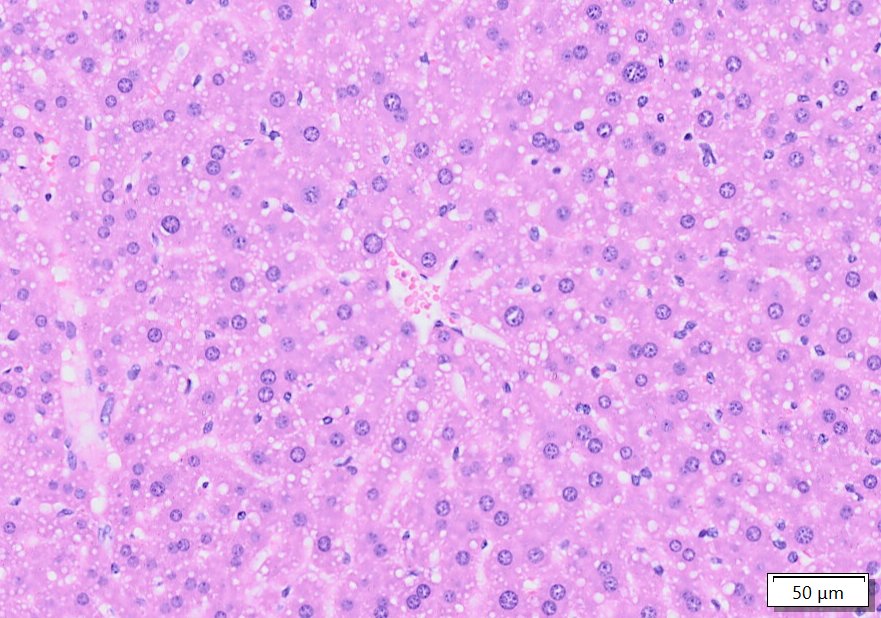

Supplement: Supplementary file 3 — Supporting File 3: advs73867‐sup‐0003‐SupportingFiguresData.zip. [file ADVS-13-e19191-s003.zip › Supporting information Figure S1-S9/S3/Figure S3C/Control-126-Liver-10í┴.jpg]

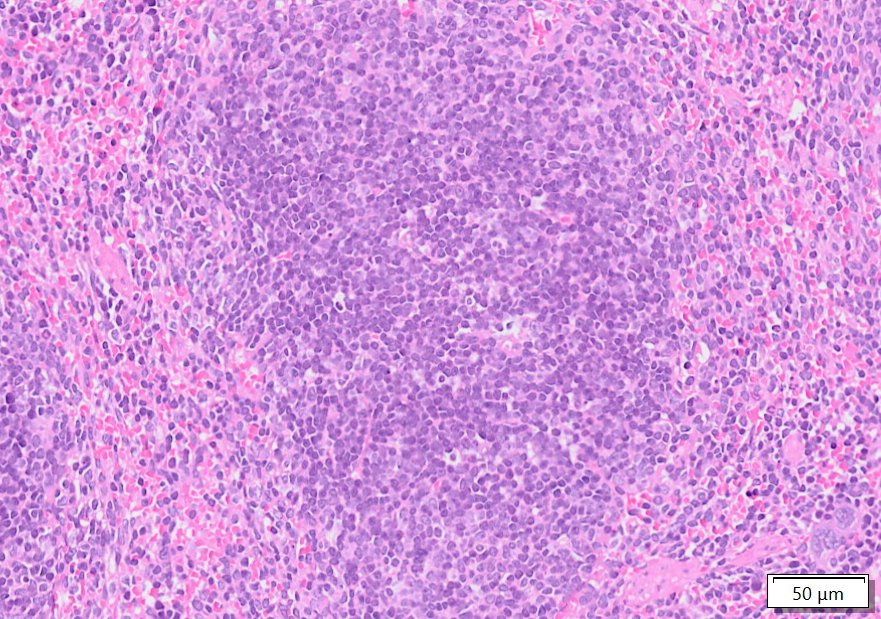

Supplement: Supplementary file 3 — Supporting File 3: advs73867‐sup‐0003‐SupportingFiguresData.zip. [file ADVS-13-e19191-s003.zip › Supporting information Figure S1-S9/S3/Figure S3C/Control-126-Spleen-10í┴.jpg]

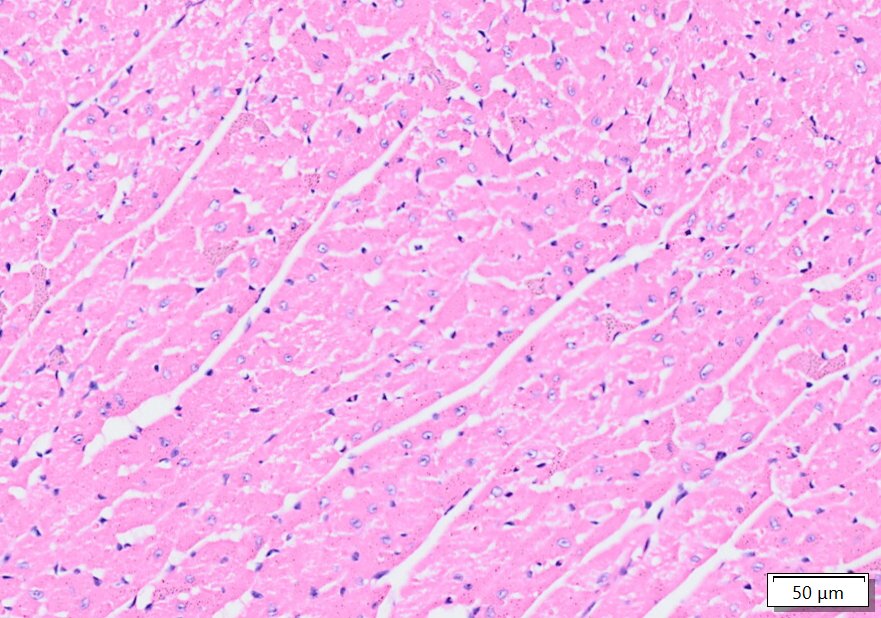

Supplement: Supplementary file 3 — Supporting File 3: advs73867‐sup‐0003‐SupportingFiguresData.zip. [file ADVS-13-e19191-s003.zip › Supporting information Figure S1-S9/S3/Figure S3C/Ferritin-109-Heart-10í┴.jpg]

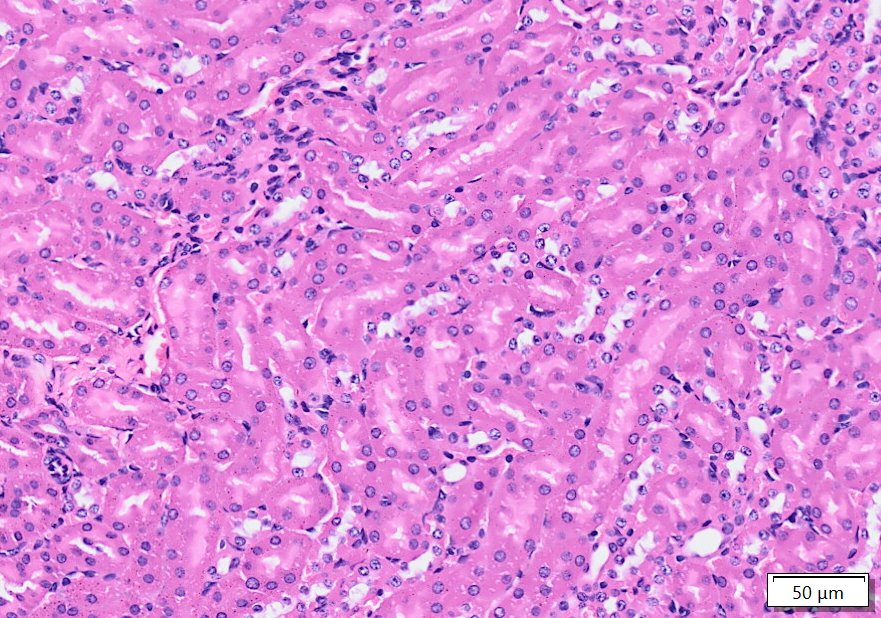

Supplement: Supplementary file 3 — Supporting File 3: advs73867‐sup‐0003‐SupportingFiguresData.zip. [file ADVS-13-e19191-s003.zip › Supporting information Figure S1-S9/S3/Figure S3C/Ferritin-109-Kidney-10í┴.jpg]

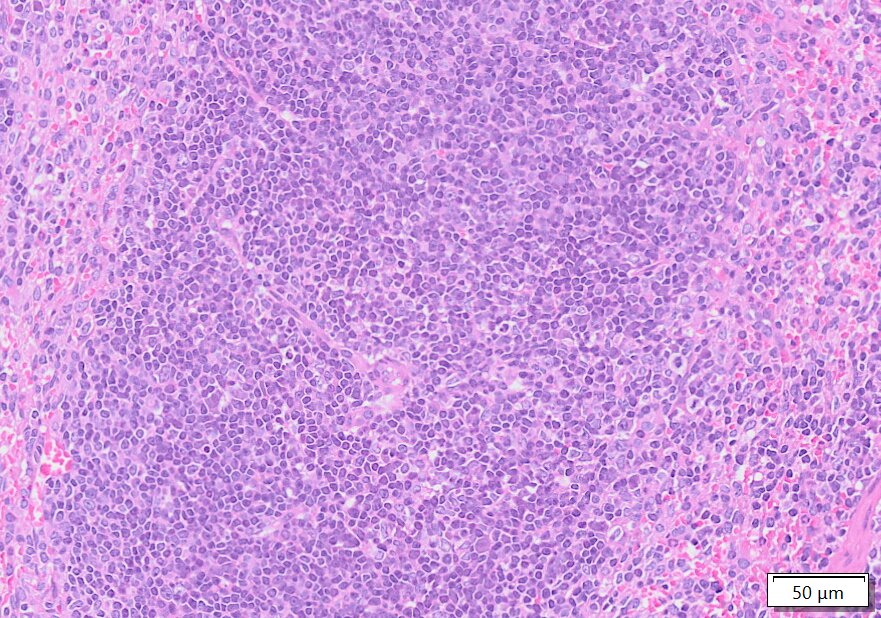

Supplement: Supplementary file 3 — Supporting File 3: advs73867‐sup‐0003‐SupportingFiguresData.zip. [file ADVS-13-e19191-s003.zip › Supporting information Figure S1-S9/S3/Figure S3C/Ferritin-109-Spleen-10í┴.jpg]

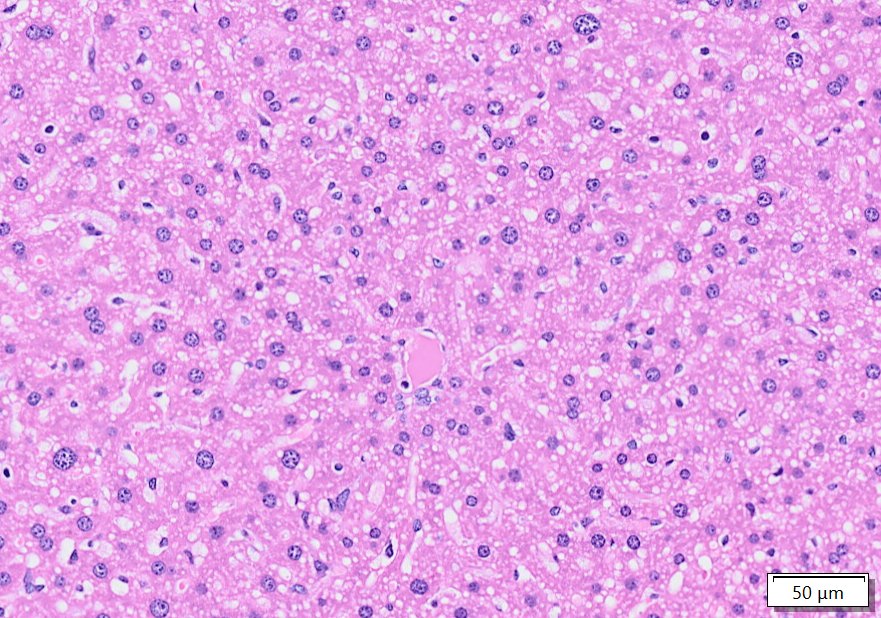

Supplement: Supplementary file 3 — Supporting File 3: advs73867‐sup‐0003‐SupportingFiguresData.zip. [file ADVS-13-e19191-s003.zip › Supporting information Figure S1-S9/S3/Figure S3C/Ferritin-123-Liver-10í┴.jpg]

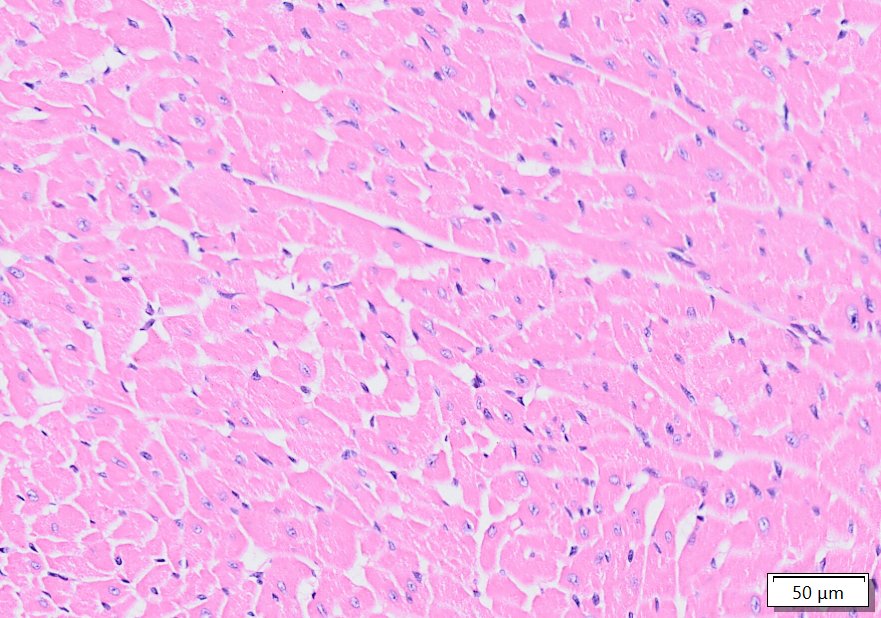

Supplement: Supplementary file 3 — Supporting File 3: advs73867‐sup‐0003‐SupportingFiguresData.zip. [file ADVS-13-e19191-s003.zip › Supporting information Figure S1-S9/S3/Figure S3C/Silica+Ferritin-116-Heart-10í┴.jpg]

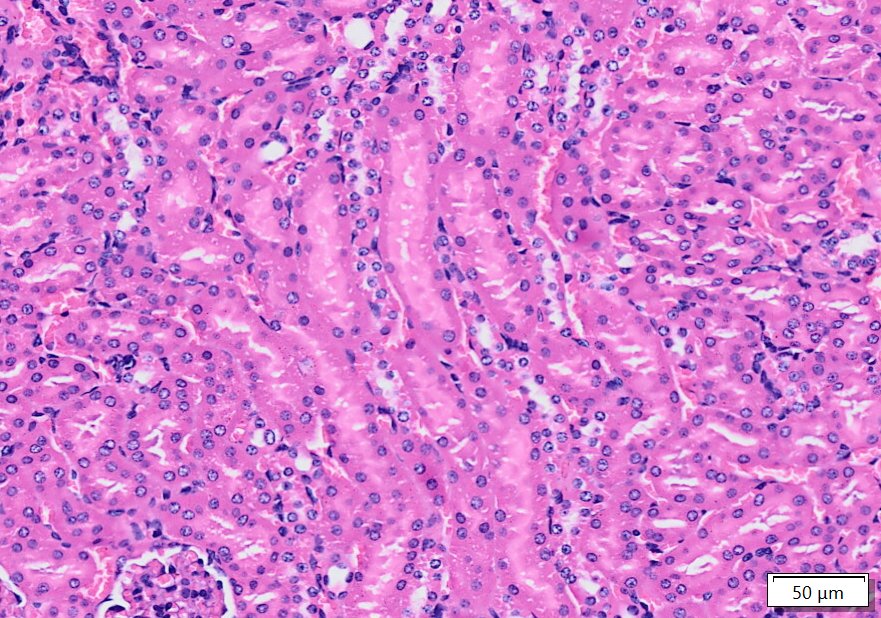

Supplement: Supplementary file 3 — Supporting File 3: advs73867‐sup‐0003‐SupportingFiguresData.zip. [file ADVS-13-e19191-s003.zip › Supporting information Figure S1-S9/S3/Figure S3C/Silica+Ferritin-116-Kidney-10í┴.jpg]

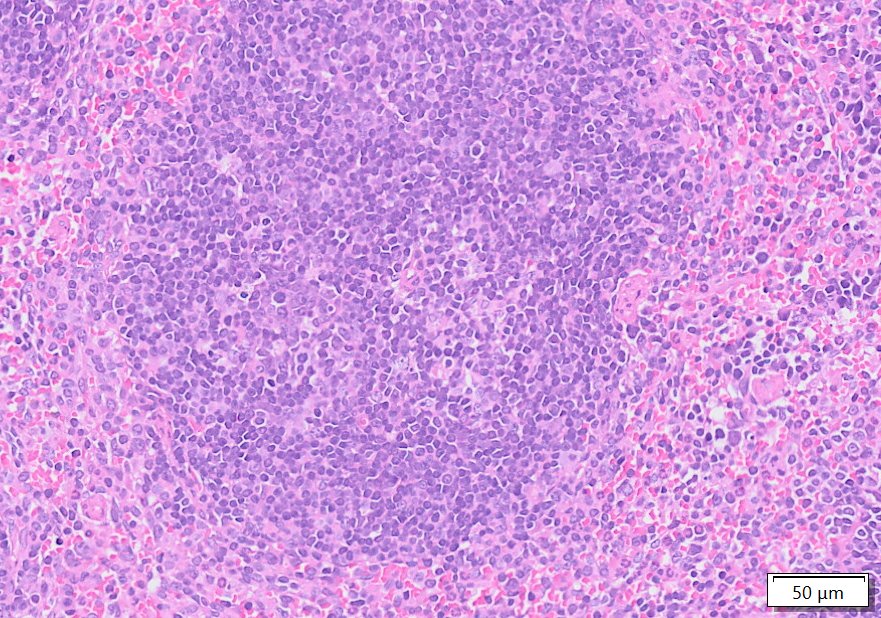

Supplement: Supplementary file 3 — Supporting File 3: advs73867‐sup‐0003‐SupportingFiguresData.zip. [file ADVS-13-e19191-s003.zip › Supporting information Figure S1-S9/S3/Figure S3C/Silica+Ferritin-116-Spleen-10í┴.jpg]

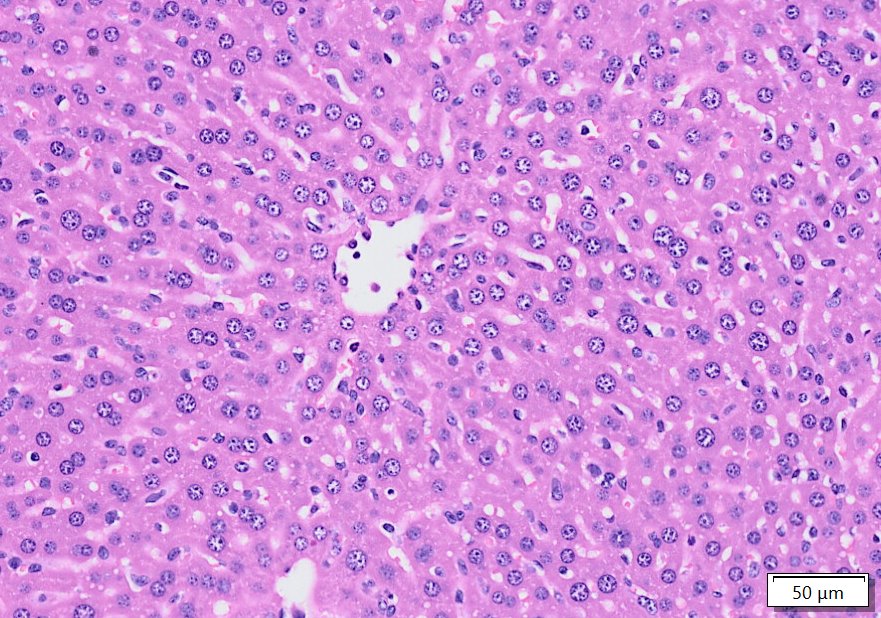

Supplement: Supplementary file 3 — Supporting File 3: advs73867‐sup‐0003‐SupportingFiguresData.zip. [file ADVS-13-e19191-s003.zip › Supporting information Figure S1-S9/S3/Figure S3C/Silica+Ferritin-117-Liver-10í┴.jpg]

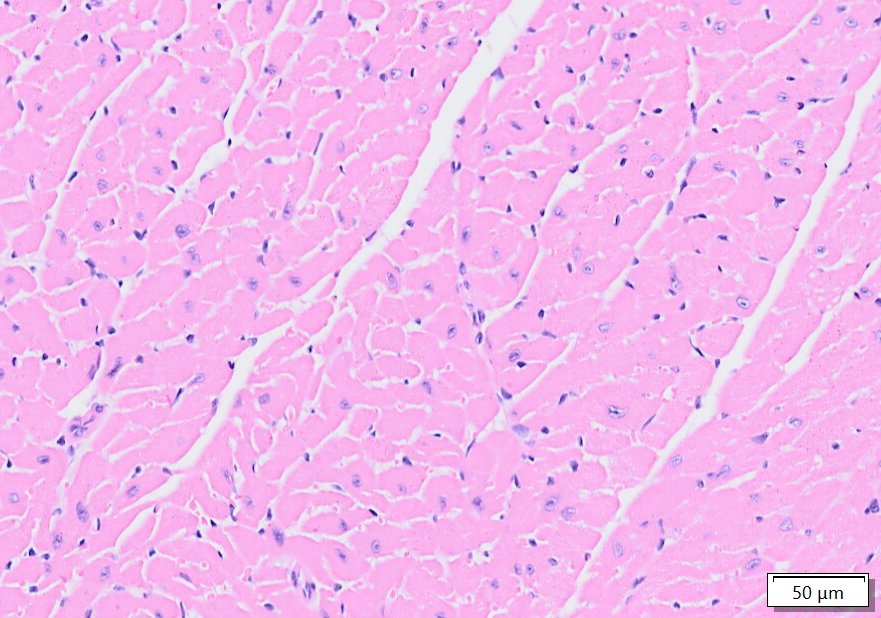

Supplement: Supplementary file 3 — Supporting File 3: advs73867‐sup‐0003‐SupportingFiguresData.zip. [file ADVS-13-e19191-s003.zip › Supporting information Figure S1-S9/S3/Figure S3C/Silica-157-Heart-10í┴.jpg]

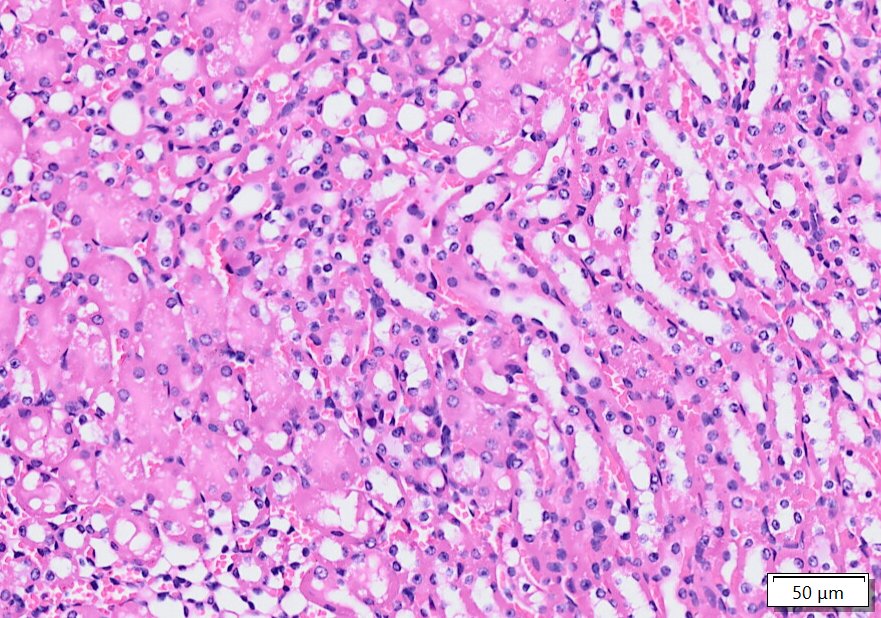

Supplement: Supplementary file 3 — Supporting File 3: advs73867‐sup‐0003‐SupportingFiguresData.zip. [file ADVS-13-e19191-s003.zip › Supporting information Figure S1-S9/S3/Figure S3C/Silica-157-Kidney-10í┴.jpg]

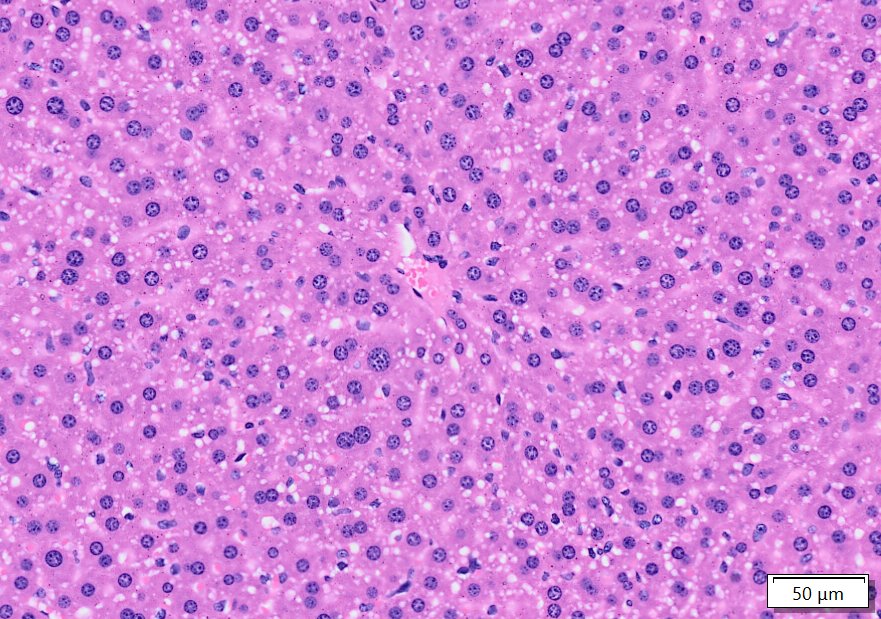

Supplement: Supplementary file 3 — Supporting File 3: advs73867‐sup‐0003‐SupportingFiguresData.zip. [file ADVS-13-e19191-s003.zip › Supporting information Figure S1-S9/S3/Figure S3C/Silica-157-Liver-10í┴.jpg]

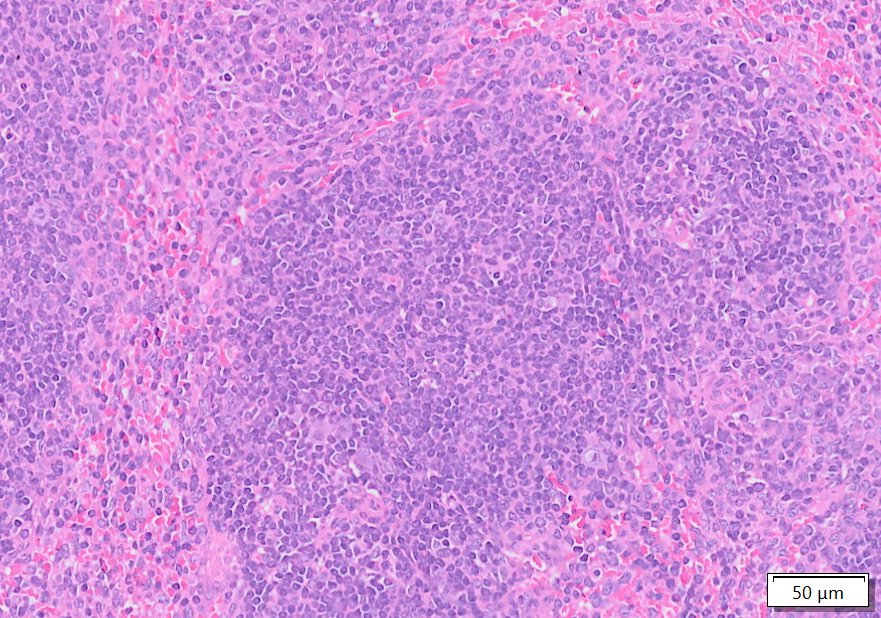

Supplement: Supplementary file 3 — Supporting File 3: advs73867‐sup‐0003‐SupportingFiguresData.zip. [file ADVS-13-e19191-s003.zip › Supporting information Figure S1-S9/S3/Figure S3C/Silica-157-Spleen-10í┴.jpg]

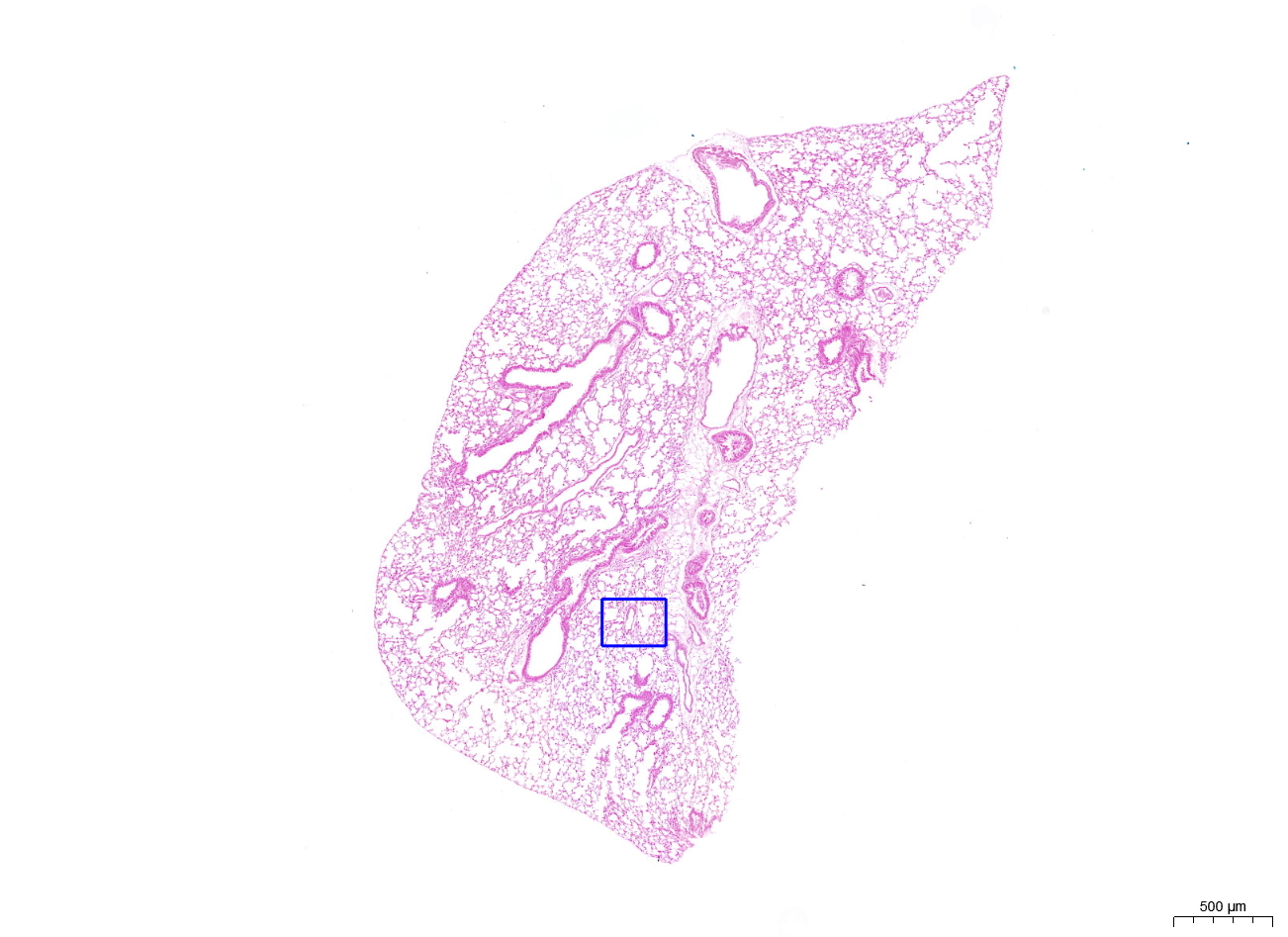

Supplement: Supplementary file 3 — Supporting File 3: advs73867‐sup‐0003‐SupportingFiguresData.zip. [file ADVS-13-e19191-s003.zip › Supporting information Figure S1-S9/S4/Figure S4A/SCRS-1 week Control 786_2.0x.jpg]

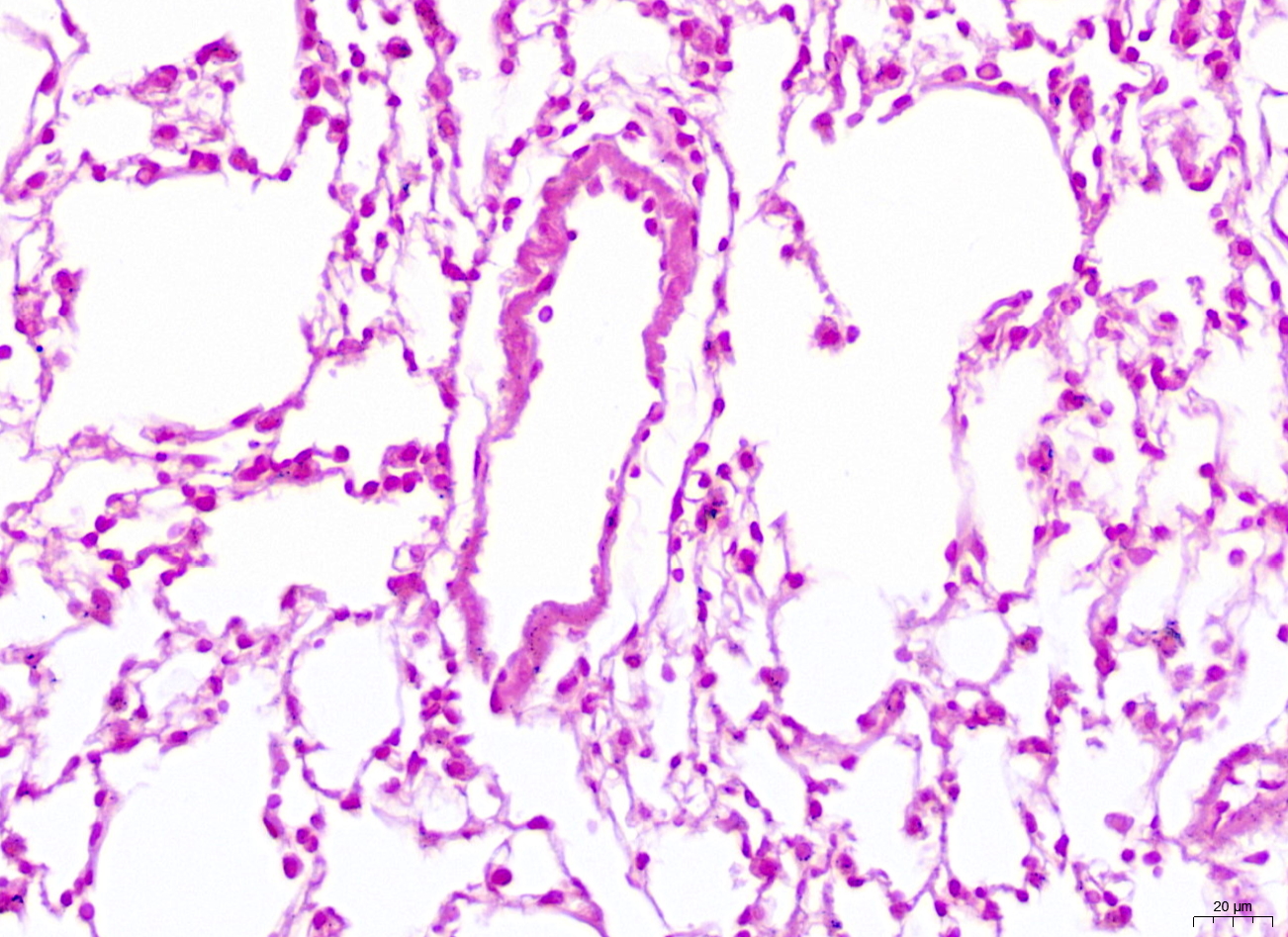

Supplement: Supplementary file 3 — Supporting File 3: advs73867‐sup‐0003‐SupportingFiguresData.zip. [file ADVS-13-e19191-s003.zip › Supporting information Figure S1-S9/S4/Figure S4A/SCRS-1 week Control 786_40.0x.jpg]

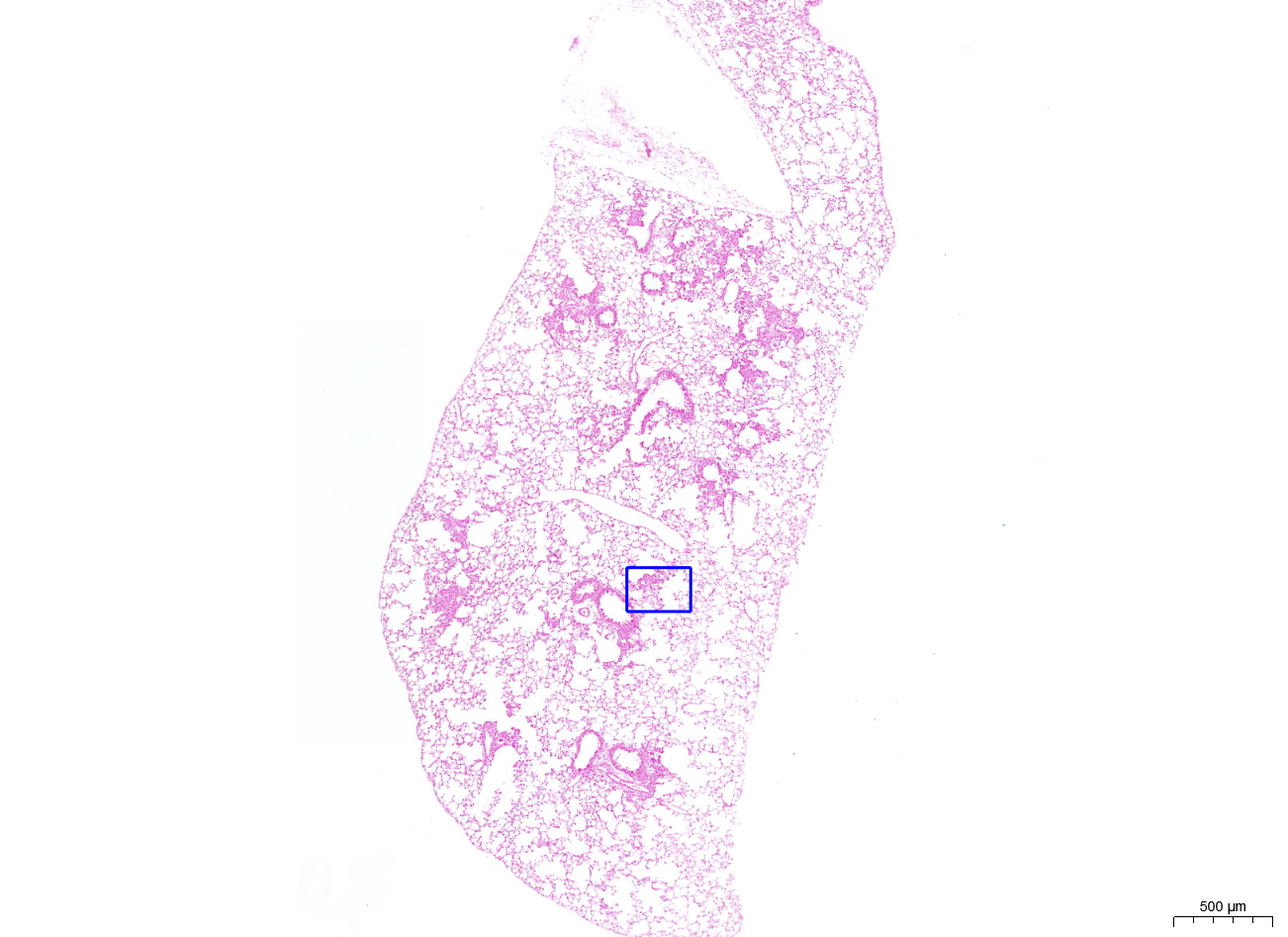

Supplement: Supplementary file 3 — Supporting File 3: advs73867‐sup‐0003‐SupportingFiguresData.zip. [file ADVS-13-e19191-s003.zip › Supporting information Figure S1-S9/S4/Figure S4A/SCRS-1 week Modell 769_2.0x.jpg]

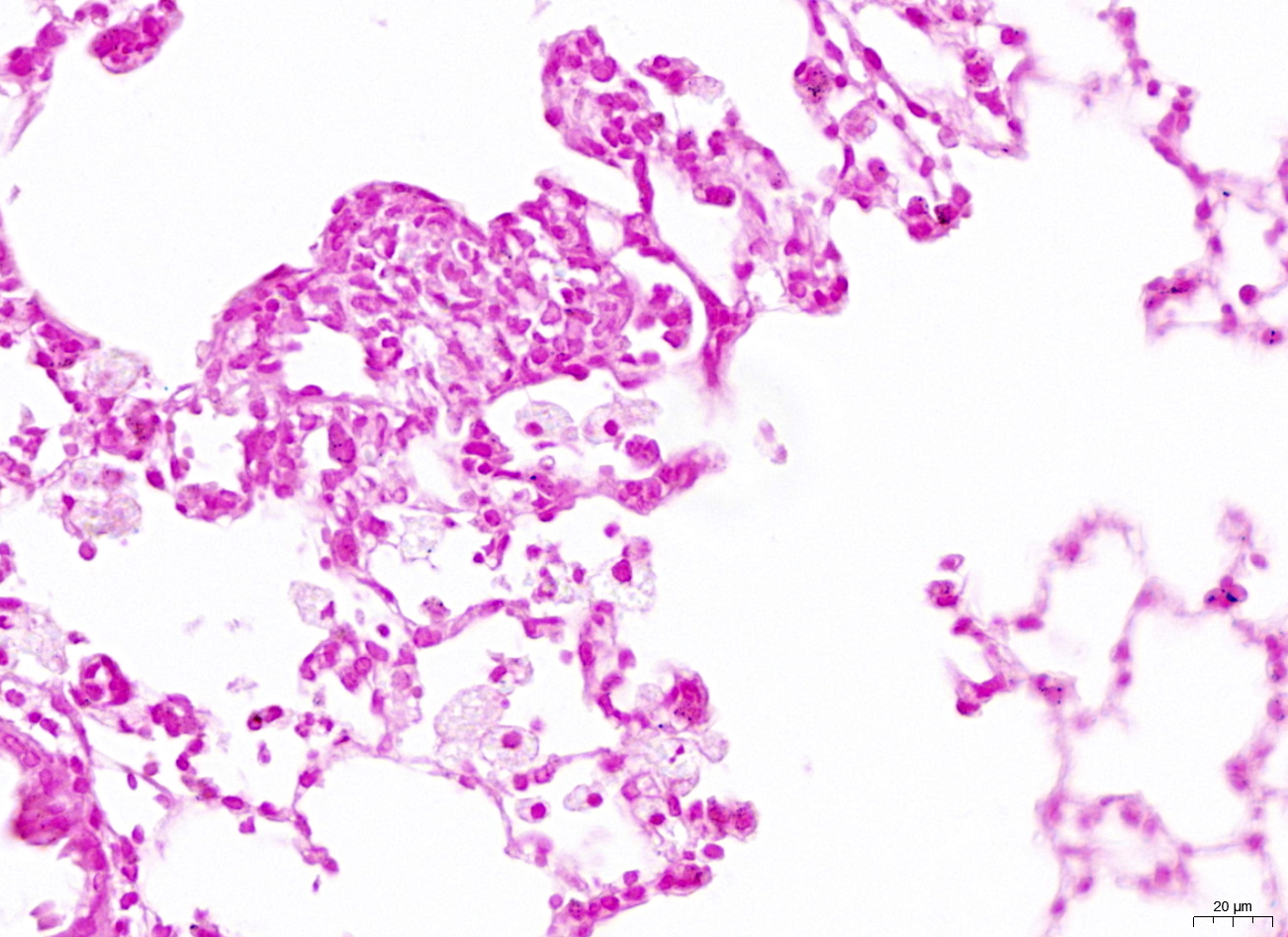

Supplement: Supplementary file 3 — Supporting File 3: advs73867‐sup‐0003‐SupportingFiguresData.zip. [file ADVS-13-e19191-s003.zip › Supporting information Figure S1-S9/S4/Figure S4A/SCRS-1 week Modell 769_40.0x.jpg]

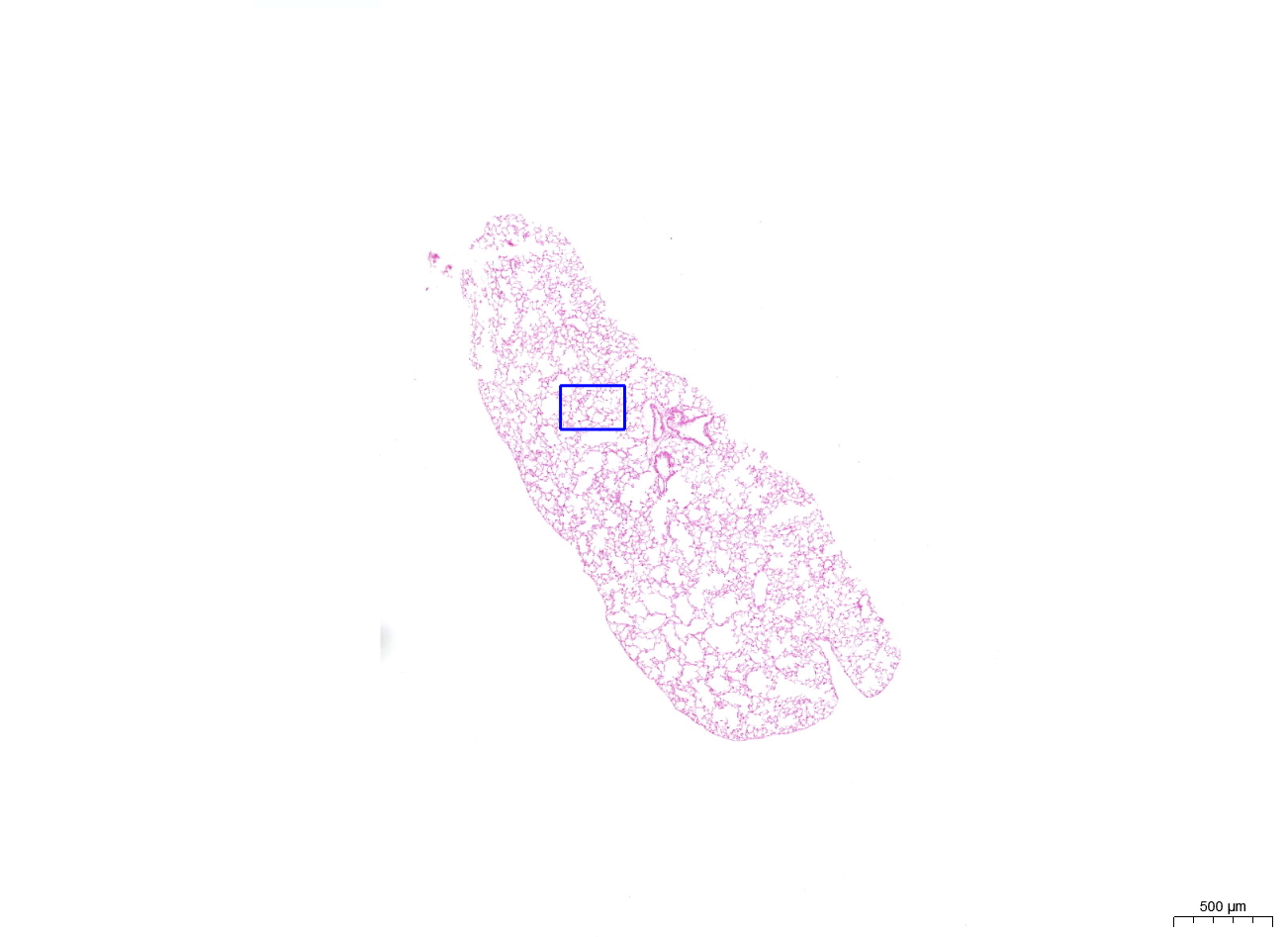

Supplement: Supplementary file 3 — Supporting File 3: advs73867‐sup‐0003‐SupportingFiguresData.zip. [file ADVS-13-e19191-s003.zip › Supporting information Figure S1-S9/S4/Figure S4A/SCRS-12 week Control 781_2.0x.jpg]

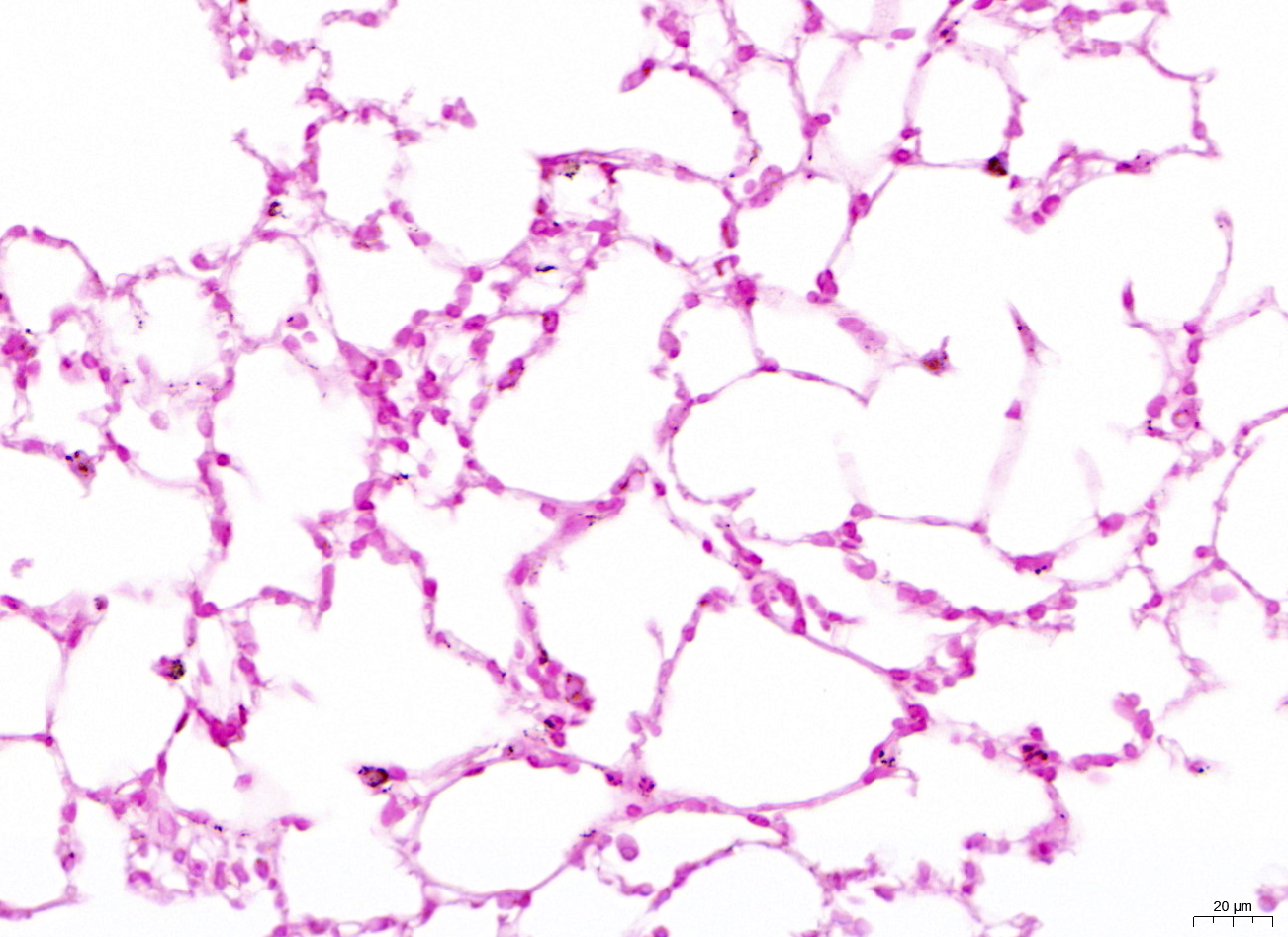

Supplement: Supplementary file 3 — Supporting File 3: advs73867‐sup‐0003‐SupportingFiguresData.zip. [file ADVS-13-e19191-s003.zip › Supporting information Figure S1-S9/S4/Figure S4A/SCRS-12 week Control 781_40.0x.jpg]

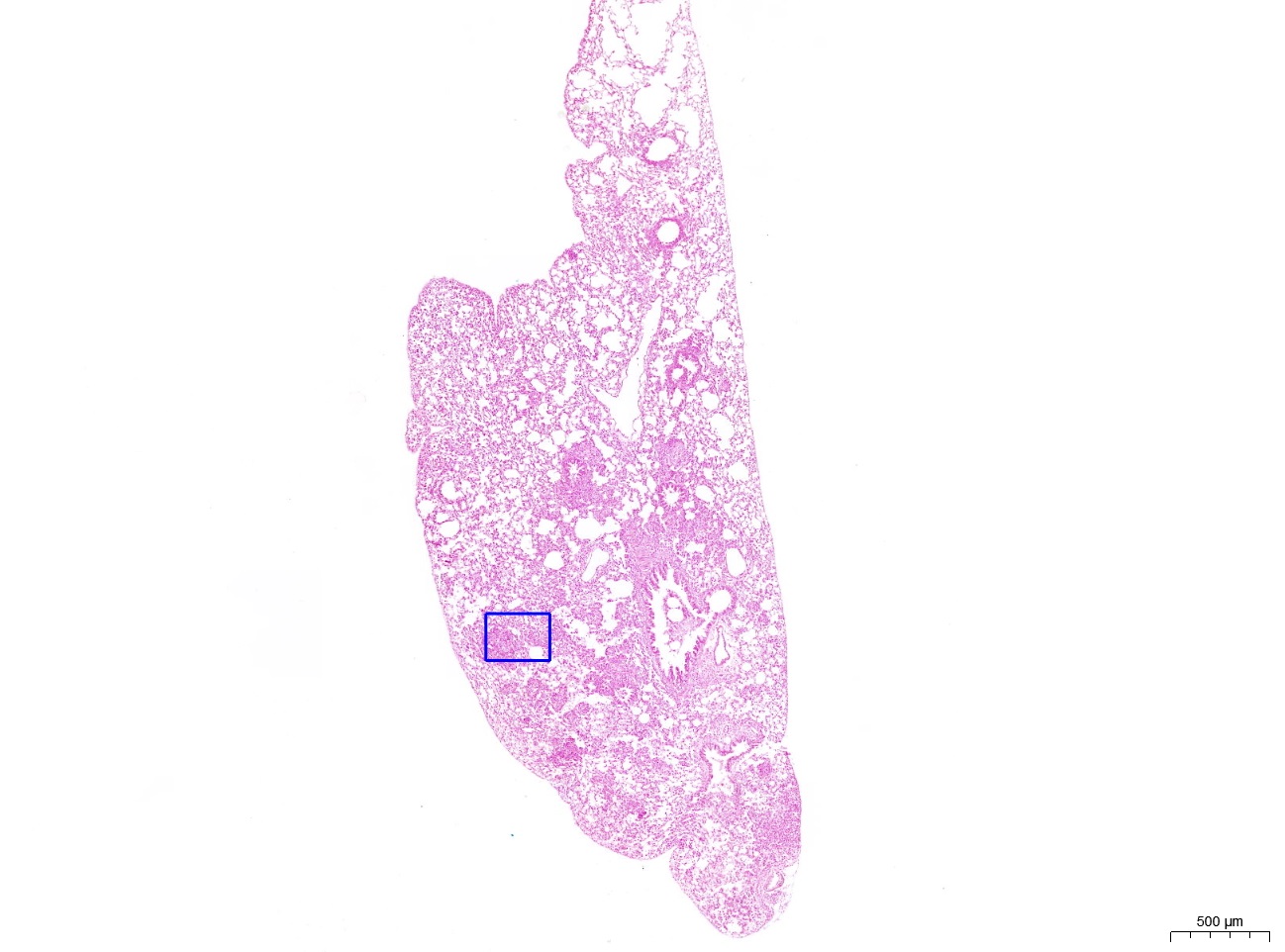

Supplement: Supplementary file 3 — Supporting File 3: advs73867‐sup‐0003‐SupportingFiguresData.zip. [file ADVS-13-e19191-s003.zip › Supporting information Figure S1-S9/S4/Figure S4A/SCRS-12 week Model-751_2.0x.jpg]

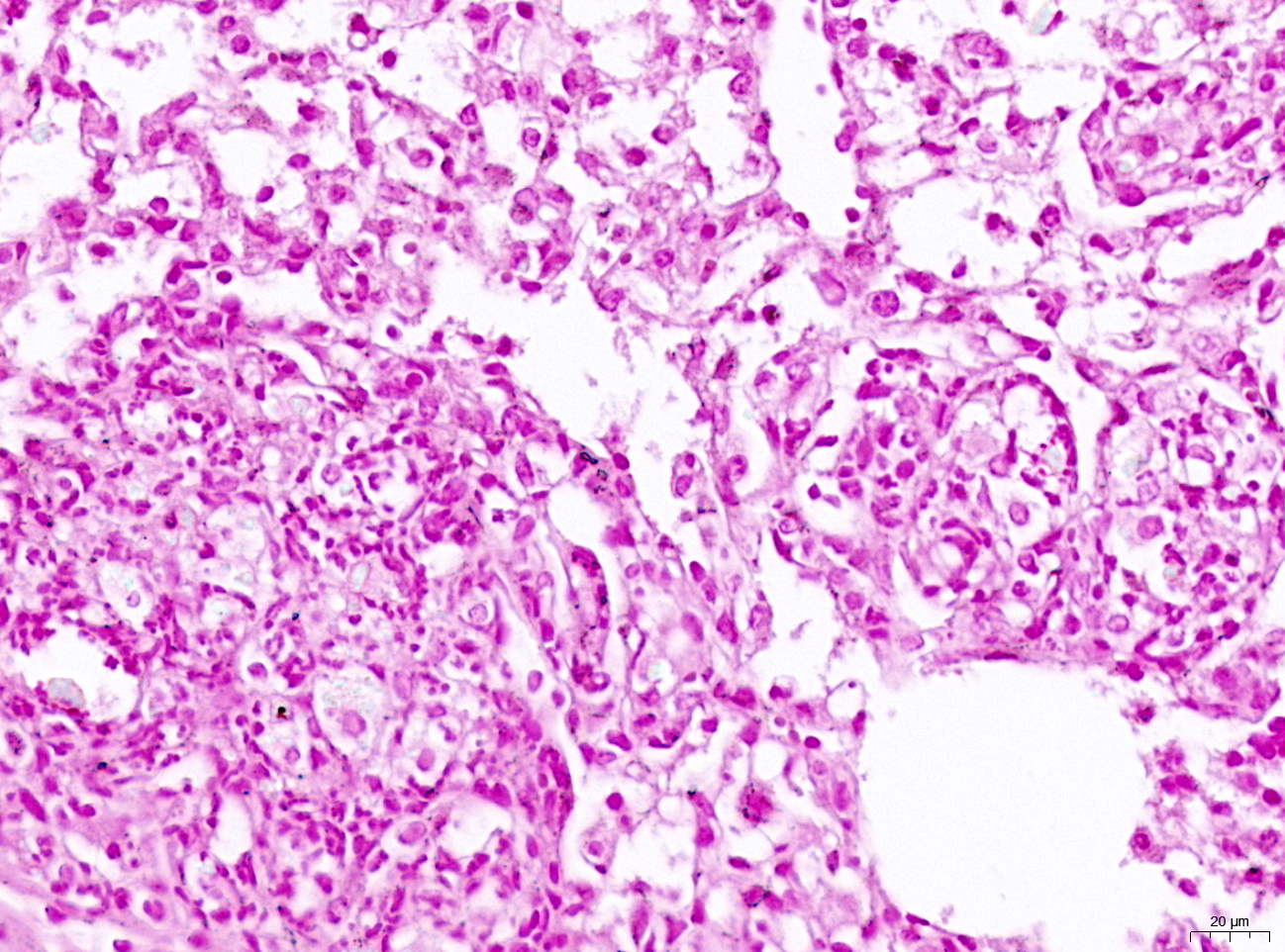

Supplement: Supplementary file 3 — Supporting File 3: advs73867‐sup‐0003‐SupportingFiguresData.zip. [file ADVS-13-e19191-s003.zip › Supporting information Figure S1-S9/S4/Figure S4A/SCRS-12 week Model-751_40.0x.jpg]

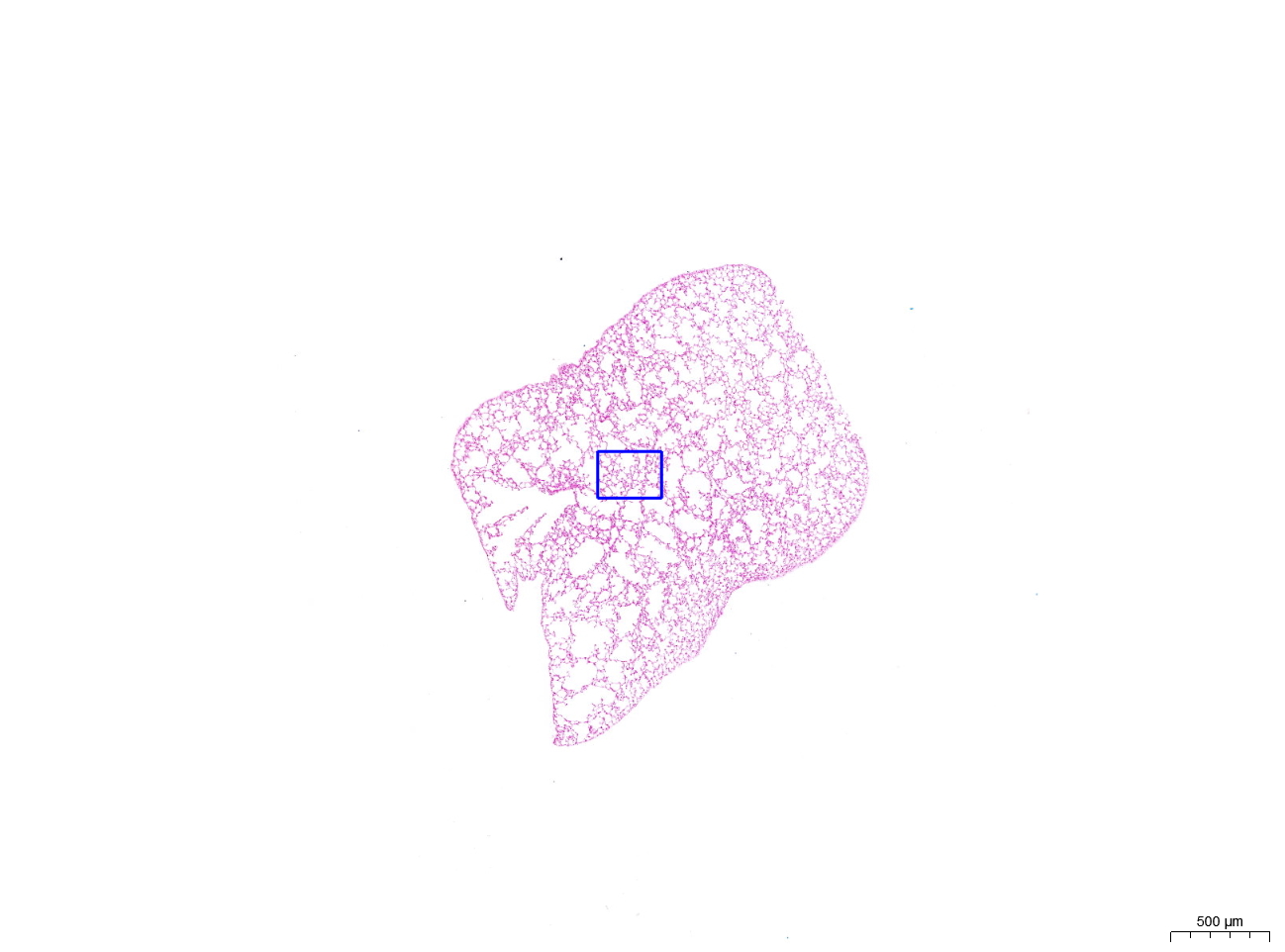

Supplement: Supplementary file 3 — Supporting File 3: advs73867‐sup‐0003‐SupportingFiguresData.zip. [file ADVS-13-e19191-s003.zip › Supporting information Figure S1-S9/S4/Figure S4A/SCRS-4 week Control 778_2.0x.jpg]

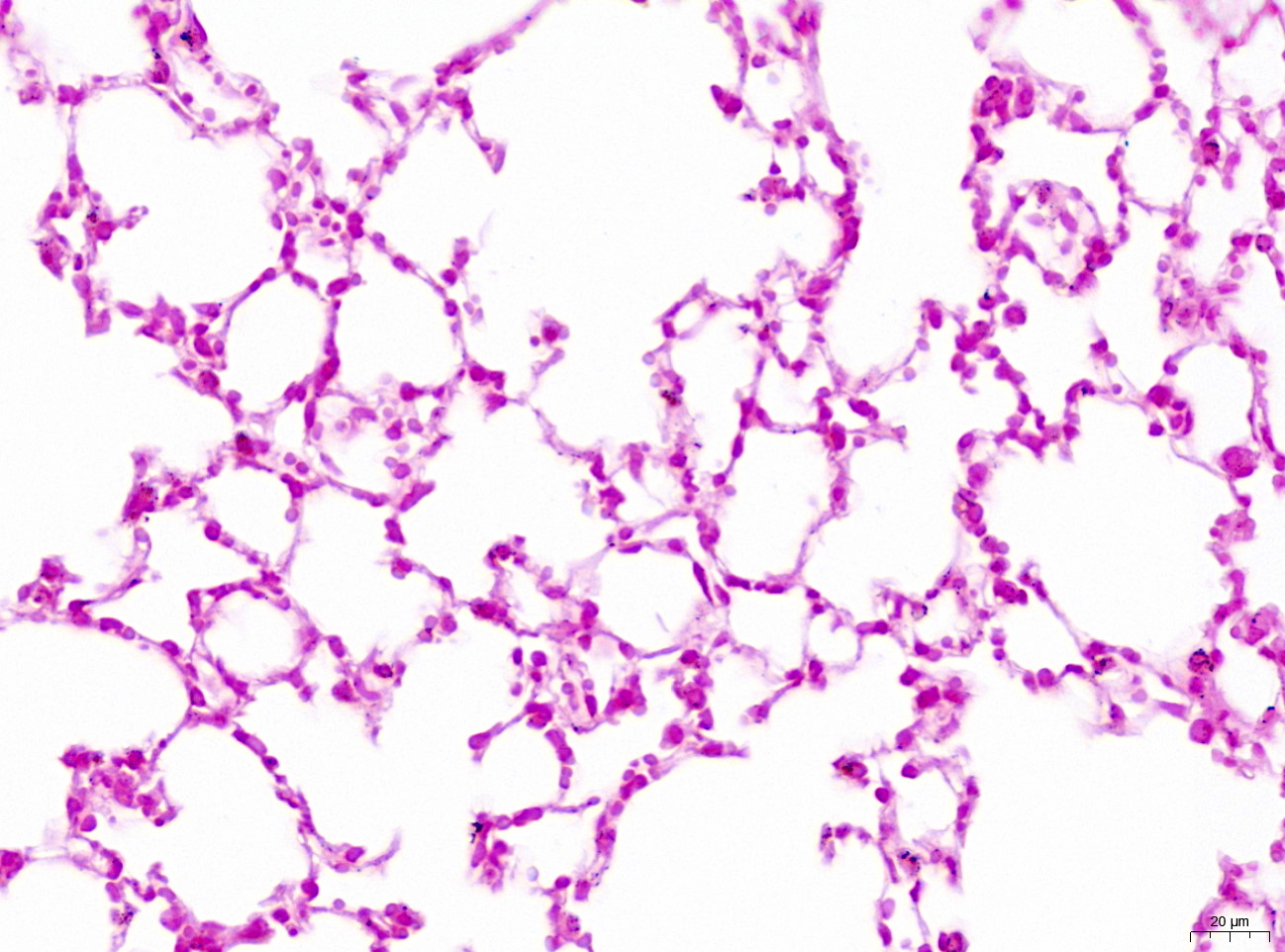

Supplement: Supplementary file 3 — Supporting File 3: advs73867‐sup‐0003‐SupportingFiguresData.zip. [file ADVS-13-e19191-s003.zip › Supporting information Figure S1-S9/S4/Figure S4A/SCRS-4 week Control 778_40.0x.jpg]

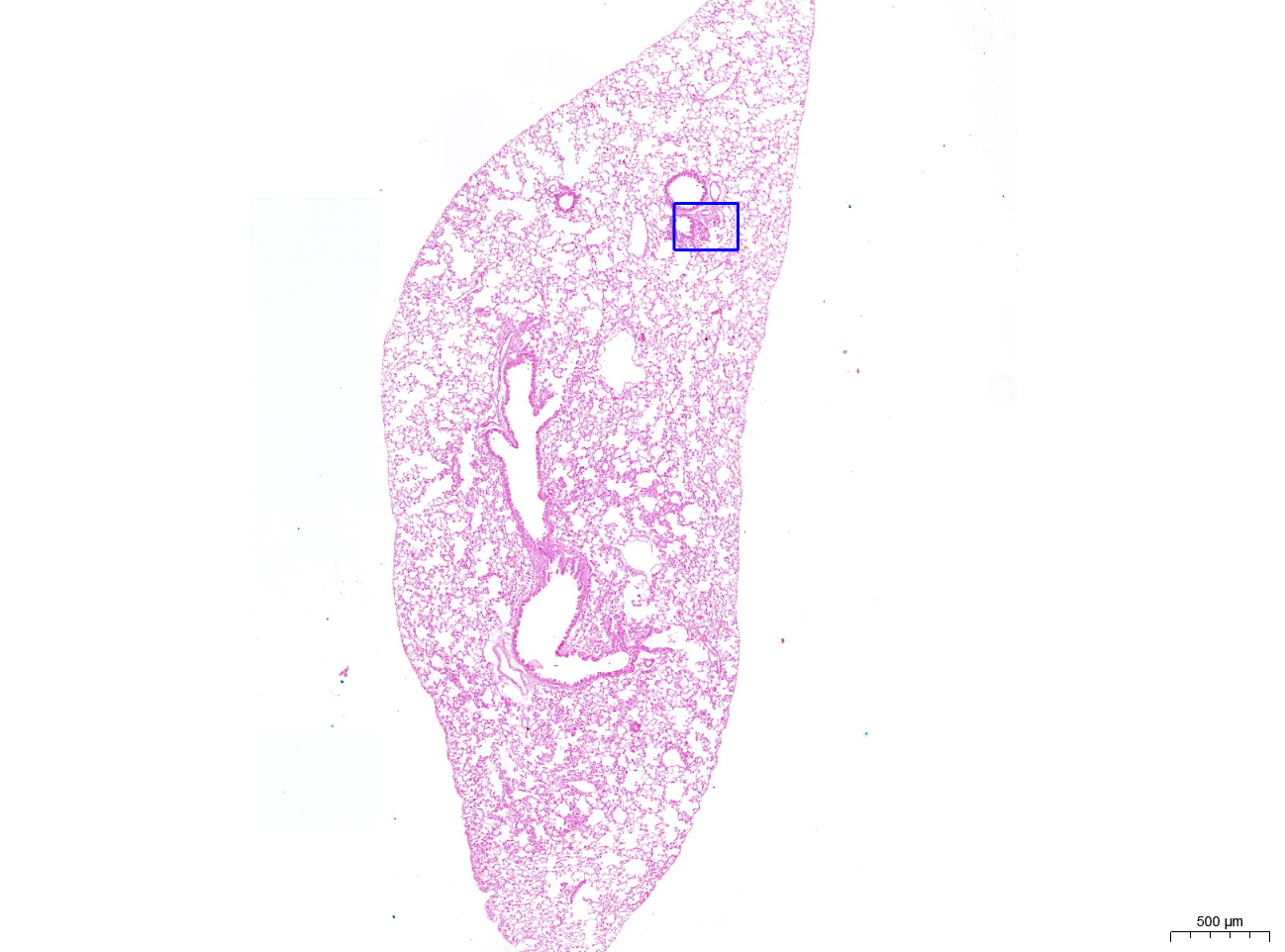

Supplement: Supplementary file 3 — Supporting File 3: advs73867‐sup‐0003‐SupportingFiguresData.zip. [file ADVS-13-e19191-s003.zip › Supporting information Figure S1-S9/S4/Figure S4A/SCRS-4 week Model 760_2.0x.jpg]

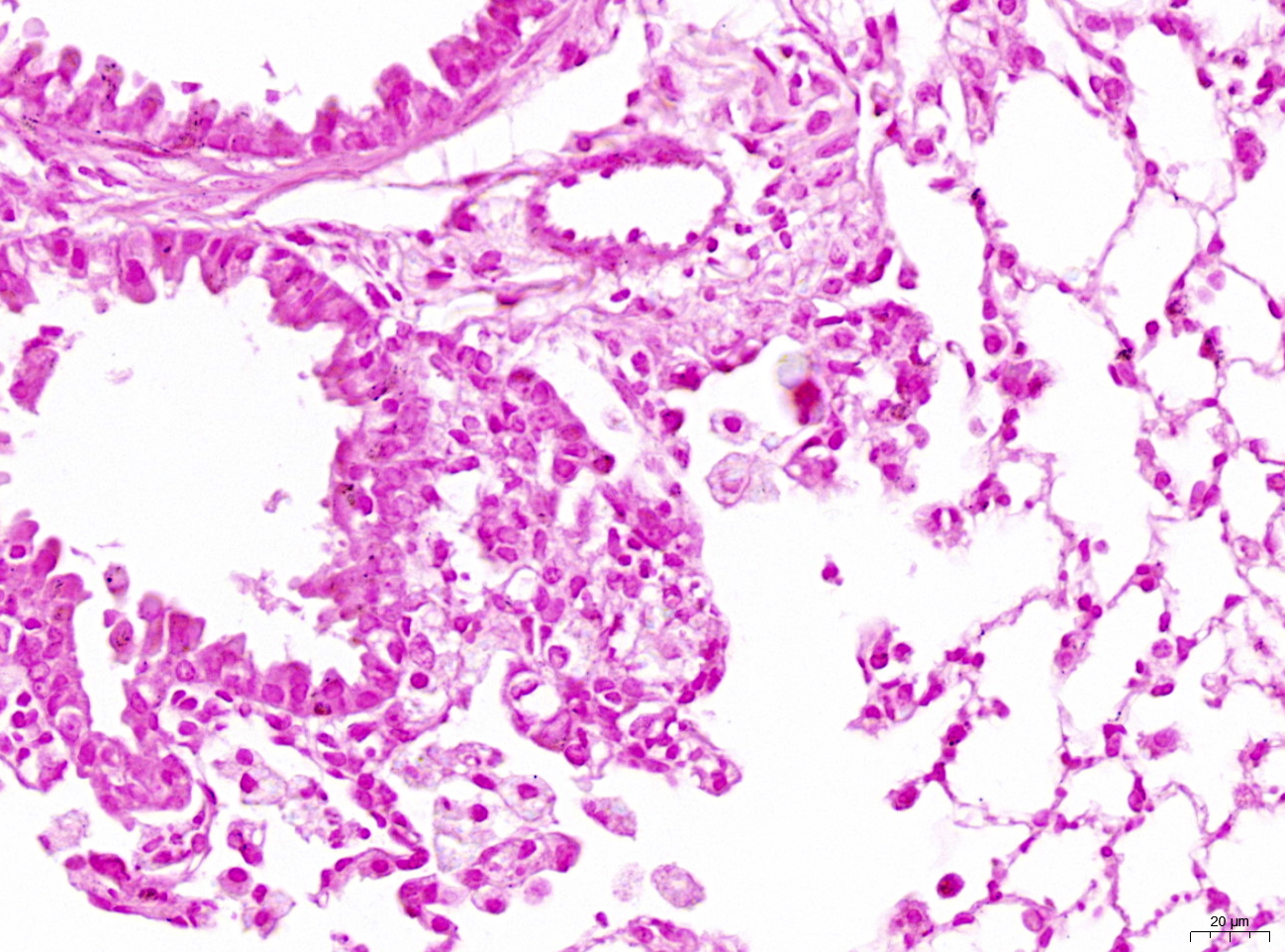

Supplement: Supplementary file 3 — Supporting File 3: advs73867‐sup‐0003‐SupportingFiguresData.zip. [file ADVS-13-e19191-s003.zip › Supporting information Figure S1-S9/S4/Figure S4A/SCRS-4 week Model 760_40.0x.jpg]

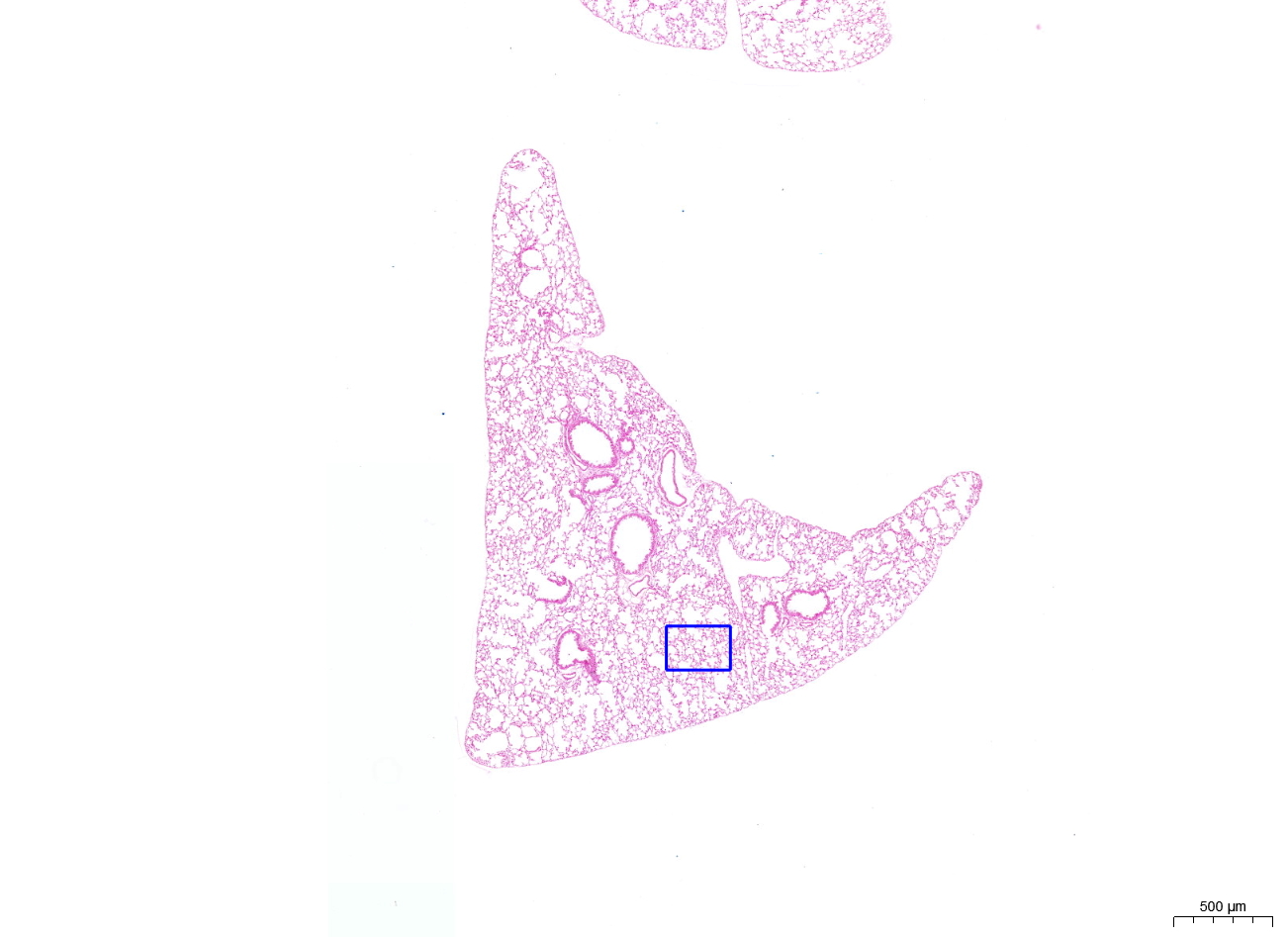

Supplement: Supplementary file 3 — Supporting File 3: advs73867‐sup‐0003‐SupportingFiguresData.zip. [file ADVS-13-e19191-s003.zip › Supporting information Figure S1-S9/S4/Figure S4A/SCRS-8 week Control 785_2.0x.jpg]

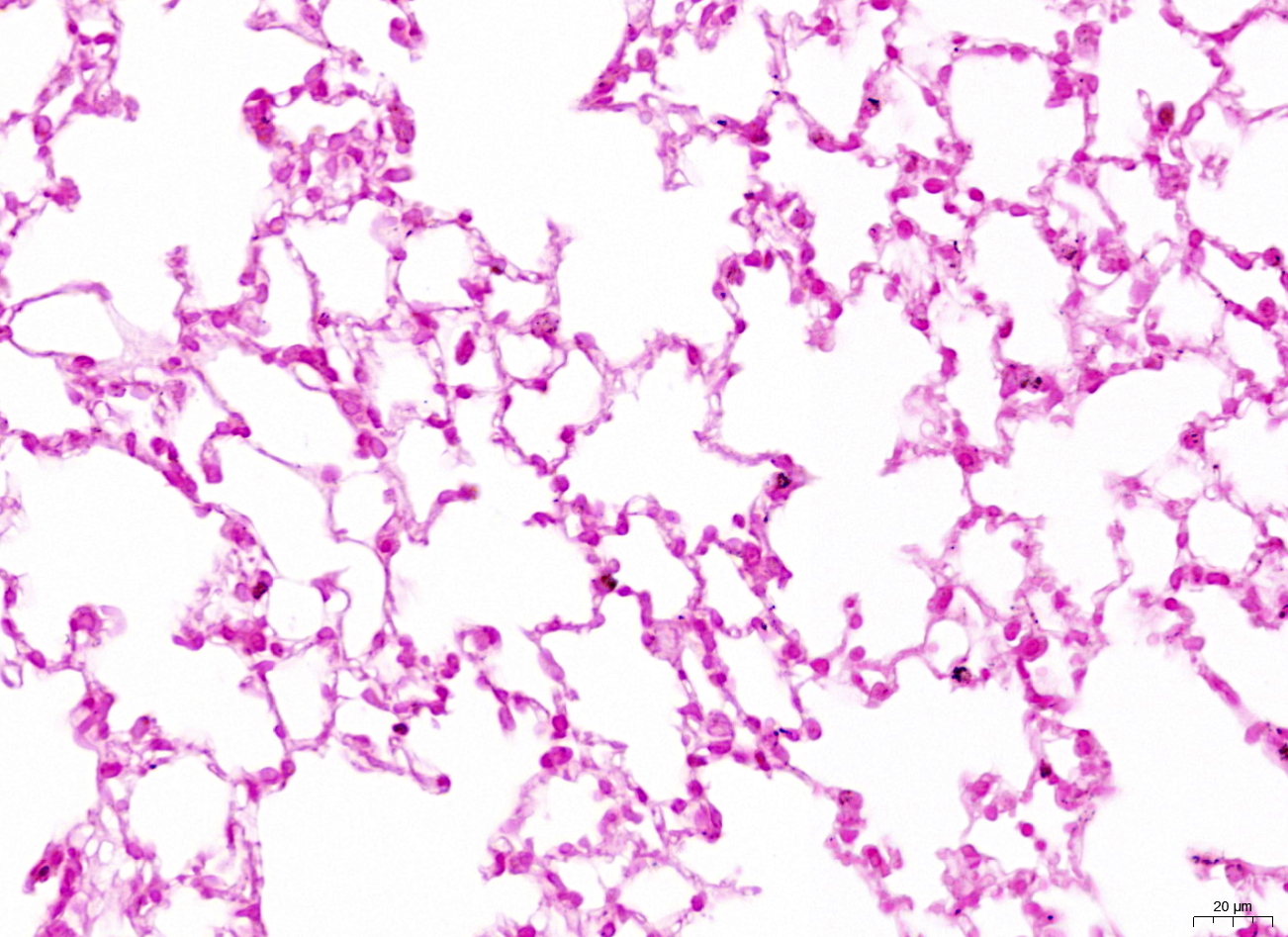

Supplement: Supplementary file 3 — Supporting File 3: advs73867‐sup‐0003‐SupportingFiguresData.zip. [file ADVS-13-e19191-s003.zip › Supporting information Figure S1-S9/S4/Figure S4A/SCRS-8 week Control 785_40.0x.jpg]

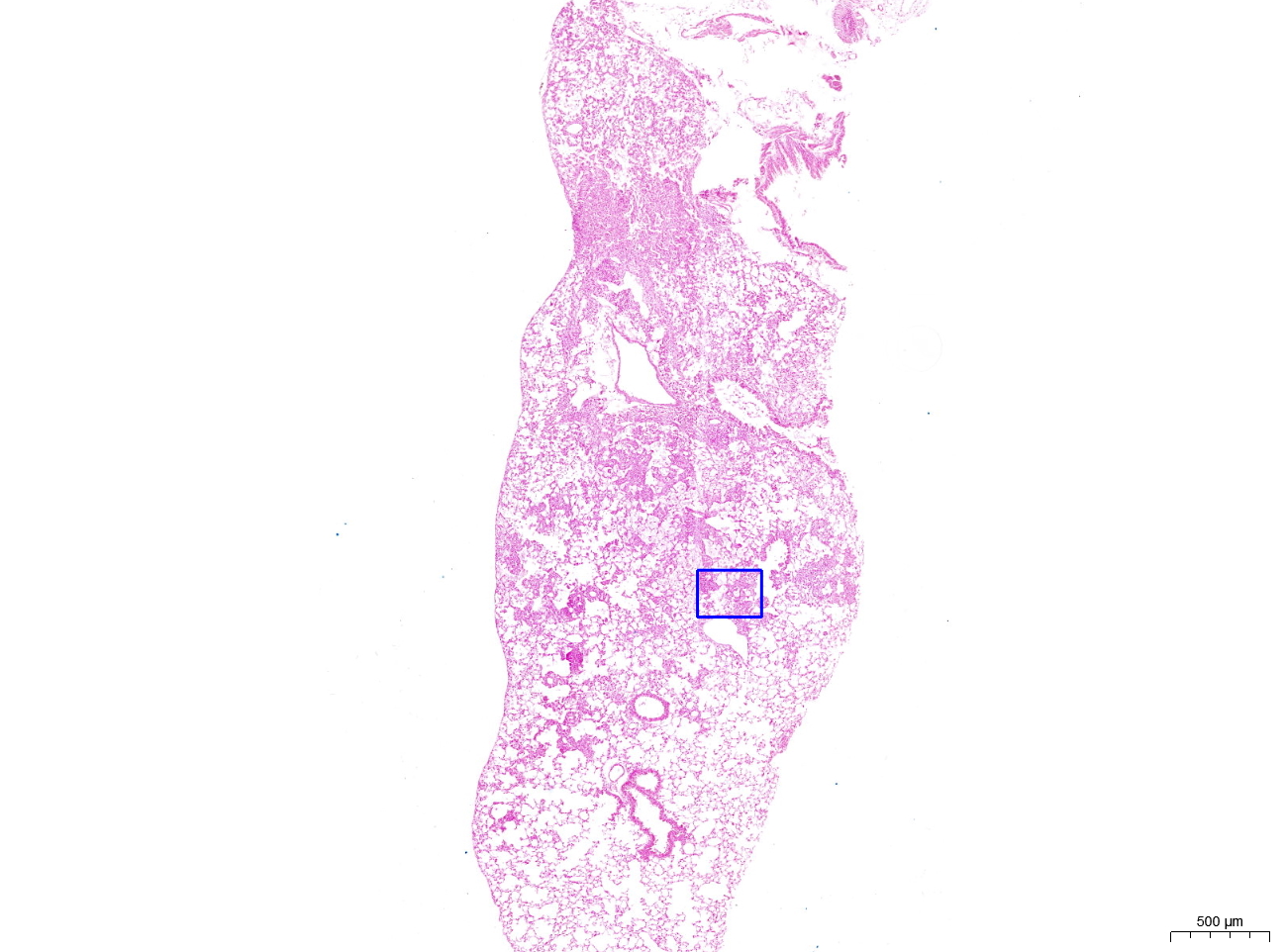

Supplement: Supplementary file 3 — Supporting File 3: advs73867‐sup‐0003‐SupportingFiguresData.zip. [file ADVS-13-e19191-s003.zip › Supporting information Figure S1-S9/S4/Figure S4A/SCRS-8 week Model 766_2.0x.jpg]

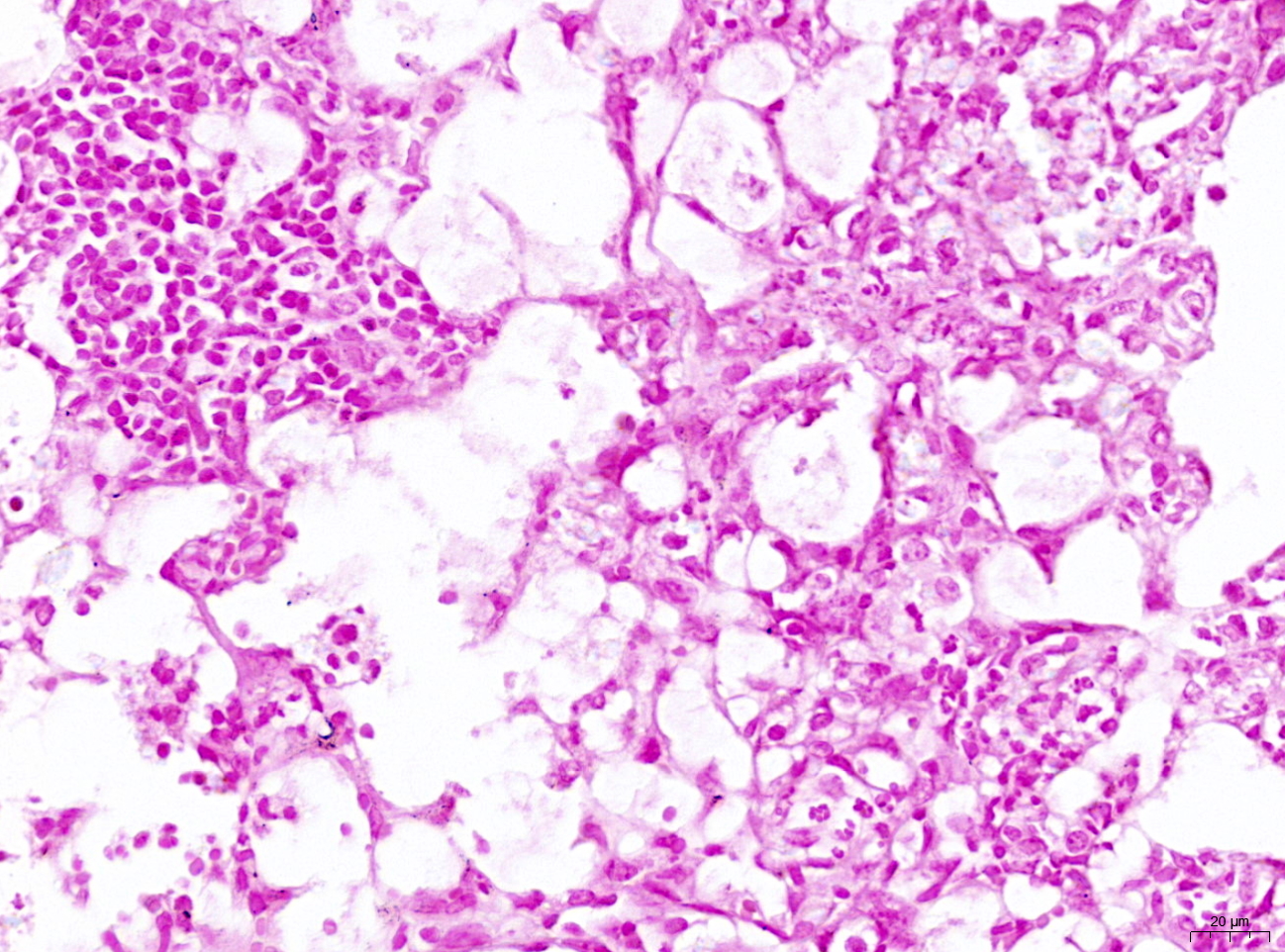

Supplement: Supplementary file 3 — Supporting File 3: advs73867‐sup‐0003‐SupportingFiguresData.zip. [file ADVS-13-e19191-s003.zip › Supporting information Figure S1-S9/S4/Figure S4A/SCRS-8 week Model 766_40.0x.jpg]

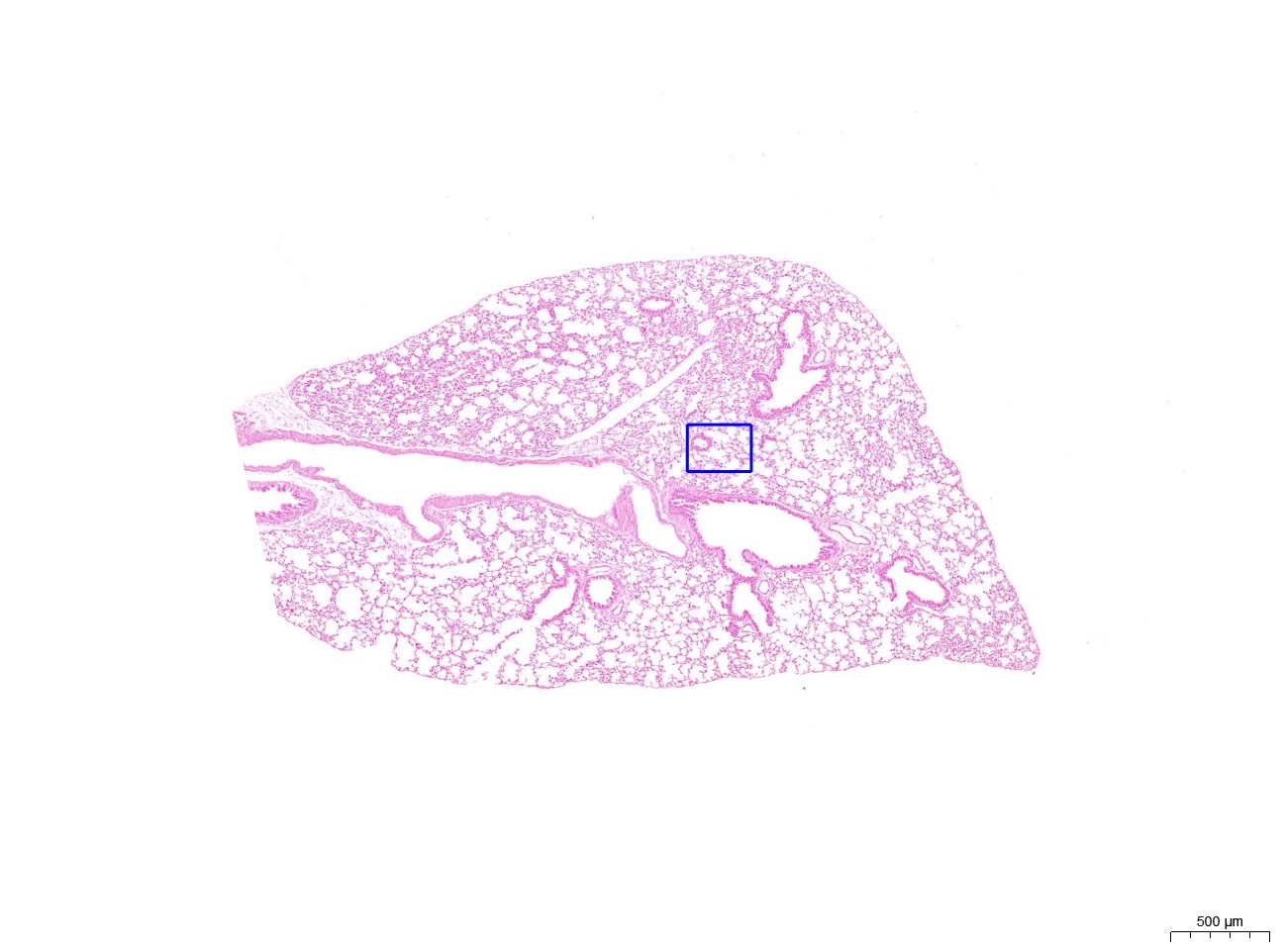

Supplement: Supplementary file 3 — Supporting File 3: advs73867‐sup‐0003‐SupportingFiguresData.zip. [file ADVS-13-e19191-s003.zip › Supporting information Figure S1-S9/S4/Figure S4B/CONTROL-104_2.0x.jpg]

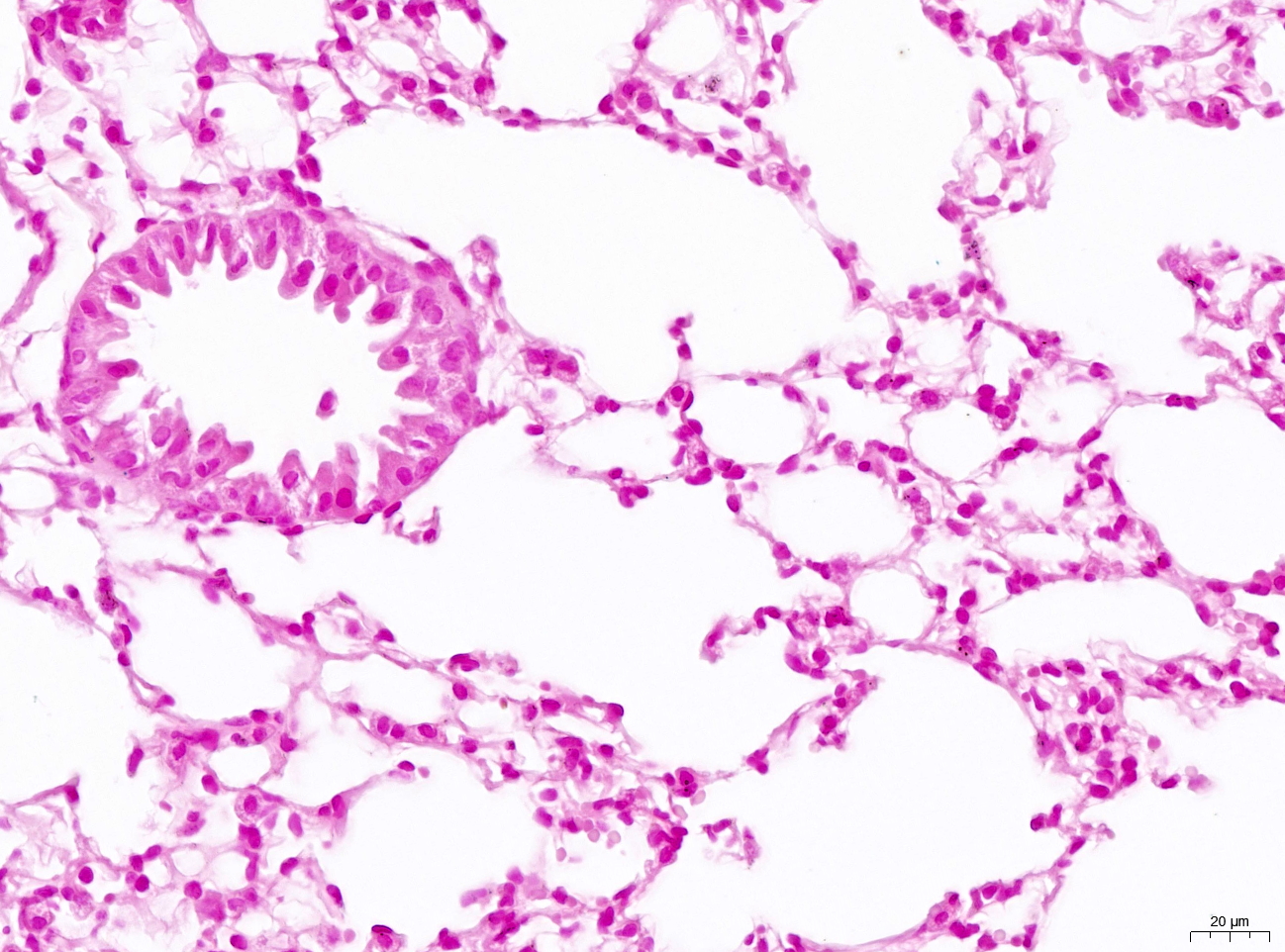

Supplement: Supplementary file 3 — Supporting File 3: advs73867‐sup‐0003‐SupportingFiguresData.zip. [file ADVS-13-e19191-s003.zip › Supporting information Figure S1-S9/S4/Figure S4B/CONTROL-104_40.0x.jpg]

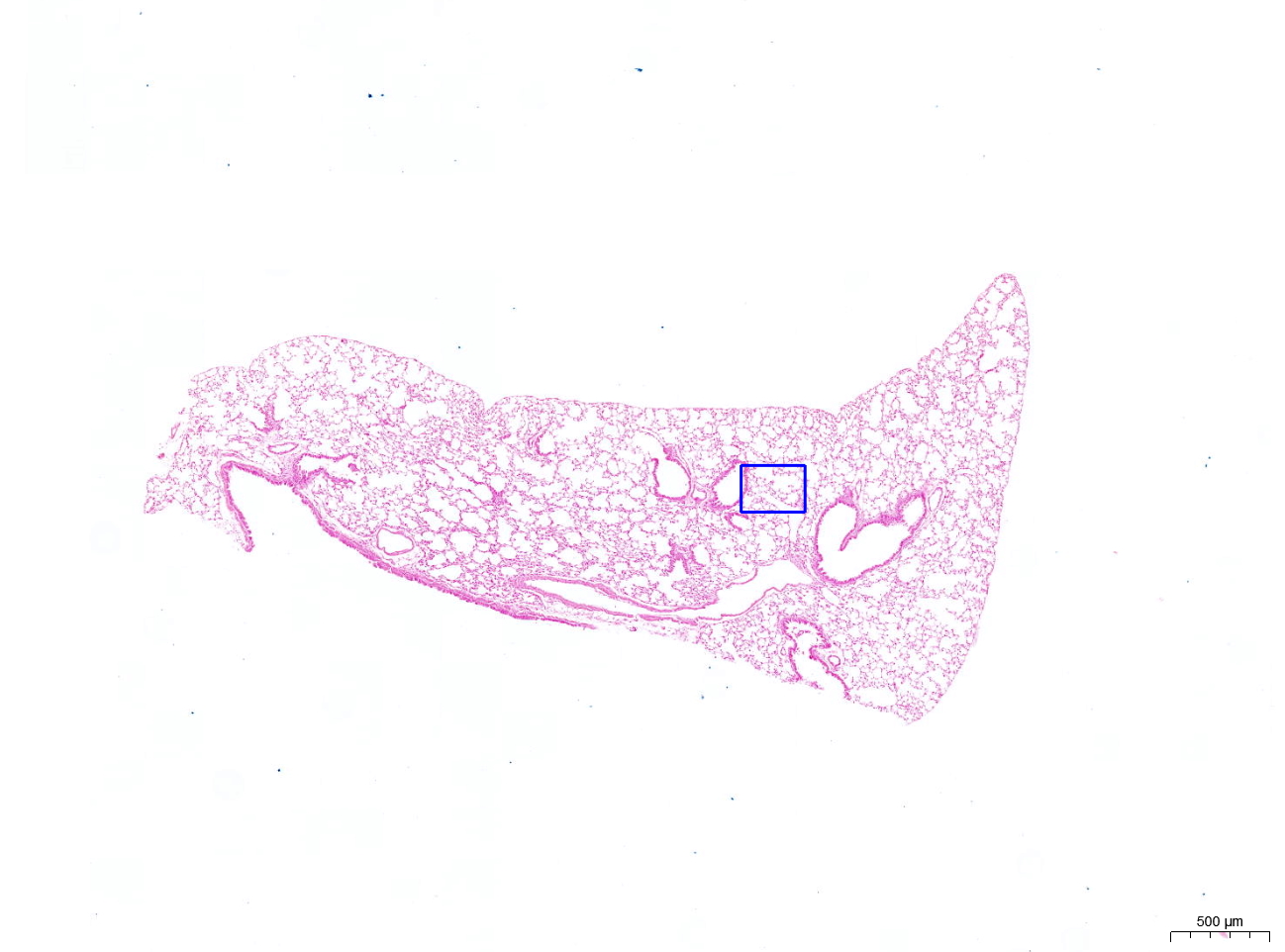

Supplement: Supplementary file 3 — Supporting File 3: advs73867‐sup‐0003‐SupportingFiguresData.zip. [file ADVS-13-e19191-s003.zip › Supporting information Figure S1-S9/S4/Figure S4B/F125-2_2.0x.jpg]

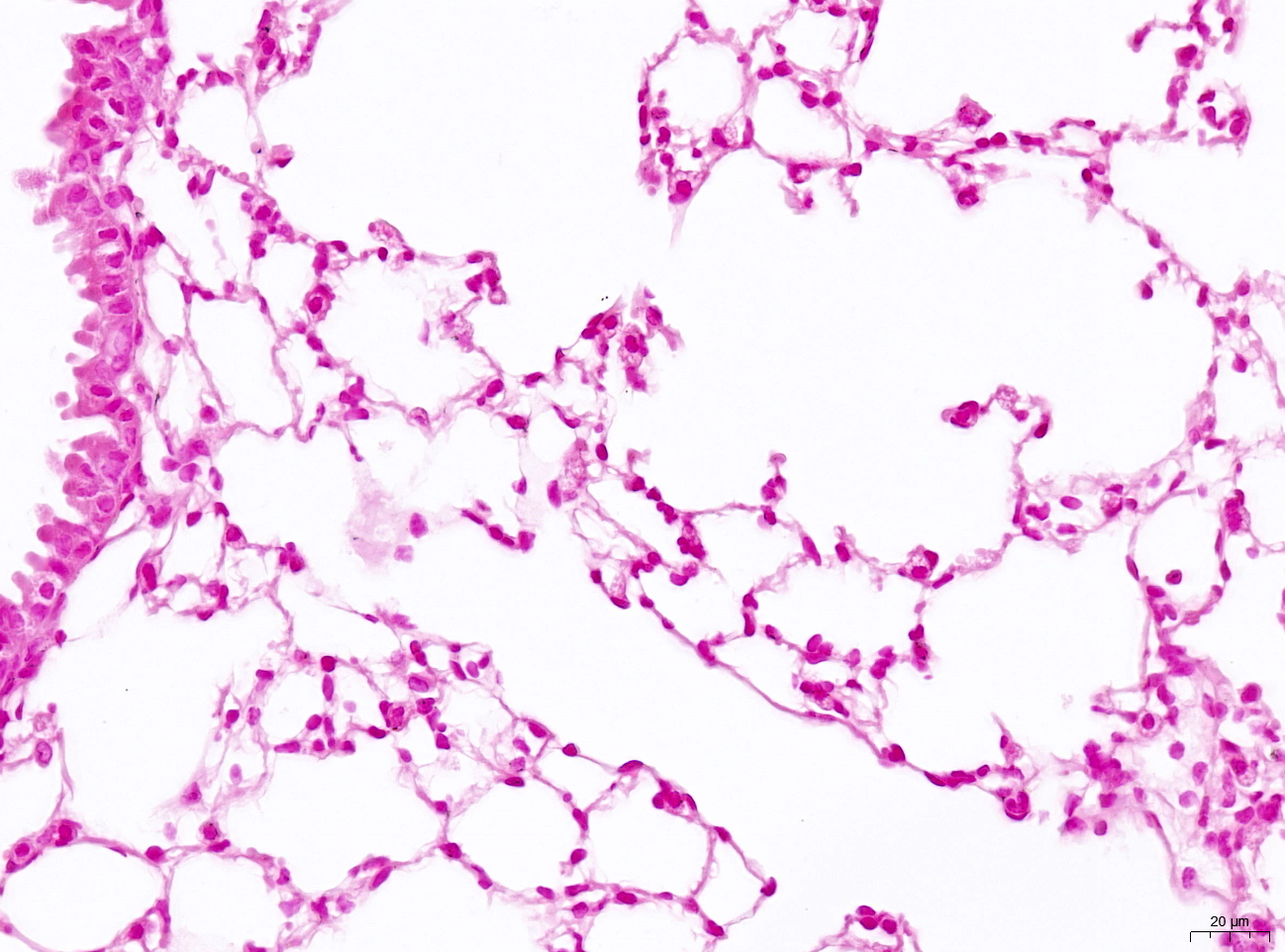

Supplement: Supplementary file 3 — Supporting File 3: advs73867‐sup‐0003‐SupportingFiguresData.zip. [file ADVS-13-e19191-s003.zip › Supporting information Figure S1-S9/S4/Figure S4B/F125-2_40.0x.jpg]

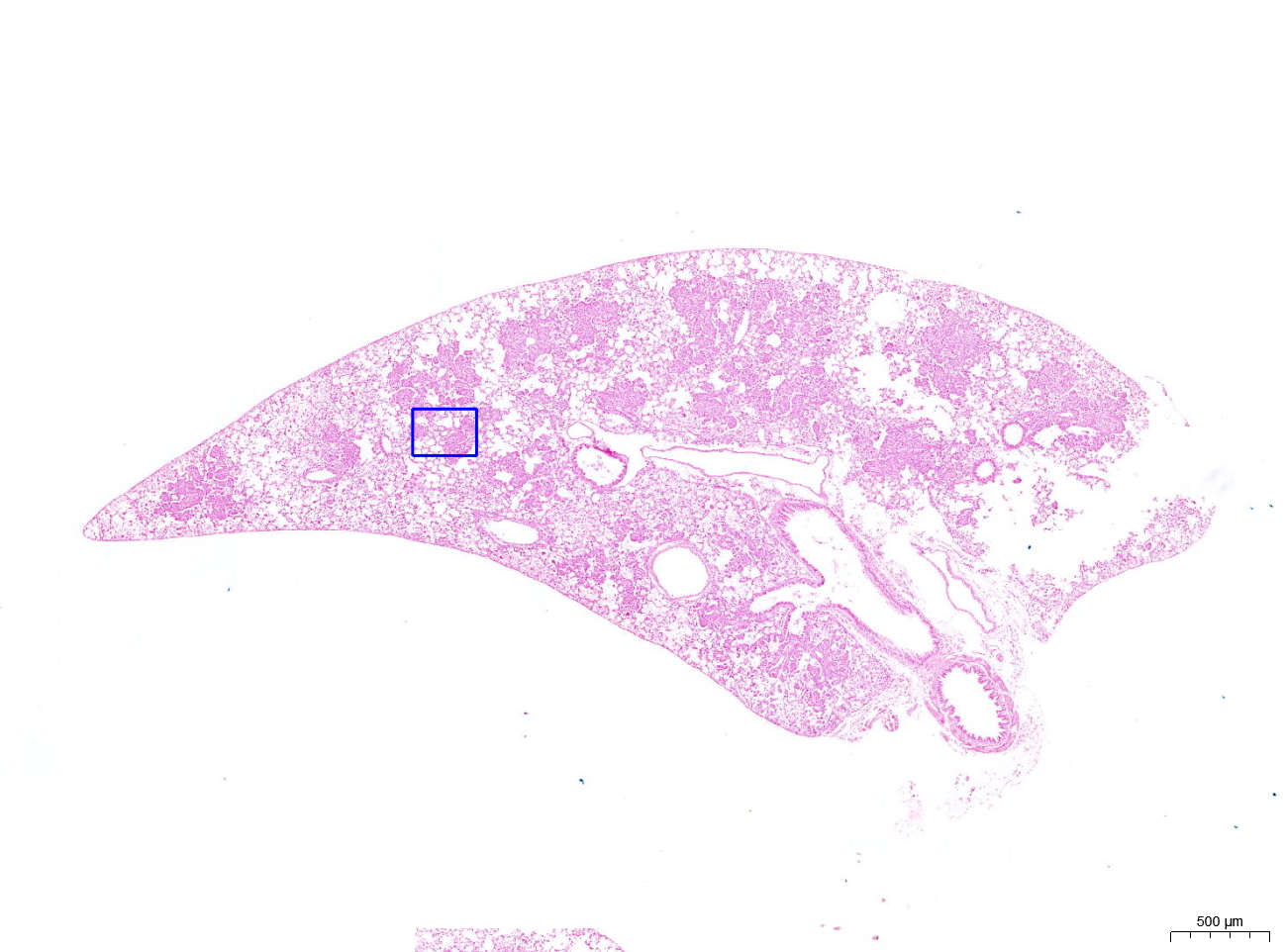

Supplement: Supplementary file 3 — Supporting File 3: advs73867‐sup‐0003‐SupportingFiguresData.zip. [file ADVS-13-e19191-s003.zip › Supporting information Figure S1-S9/S4/Figure S4B/S112_2.0x.jpg]

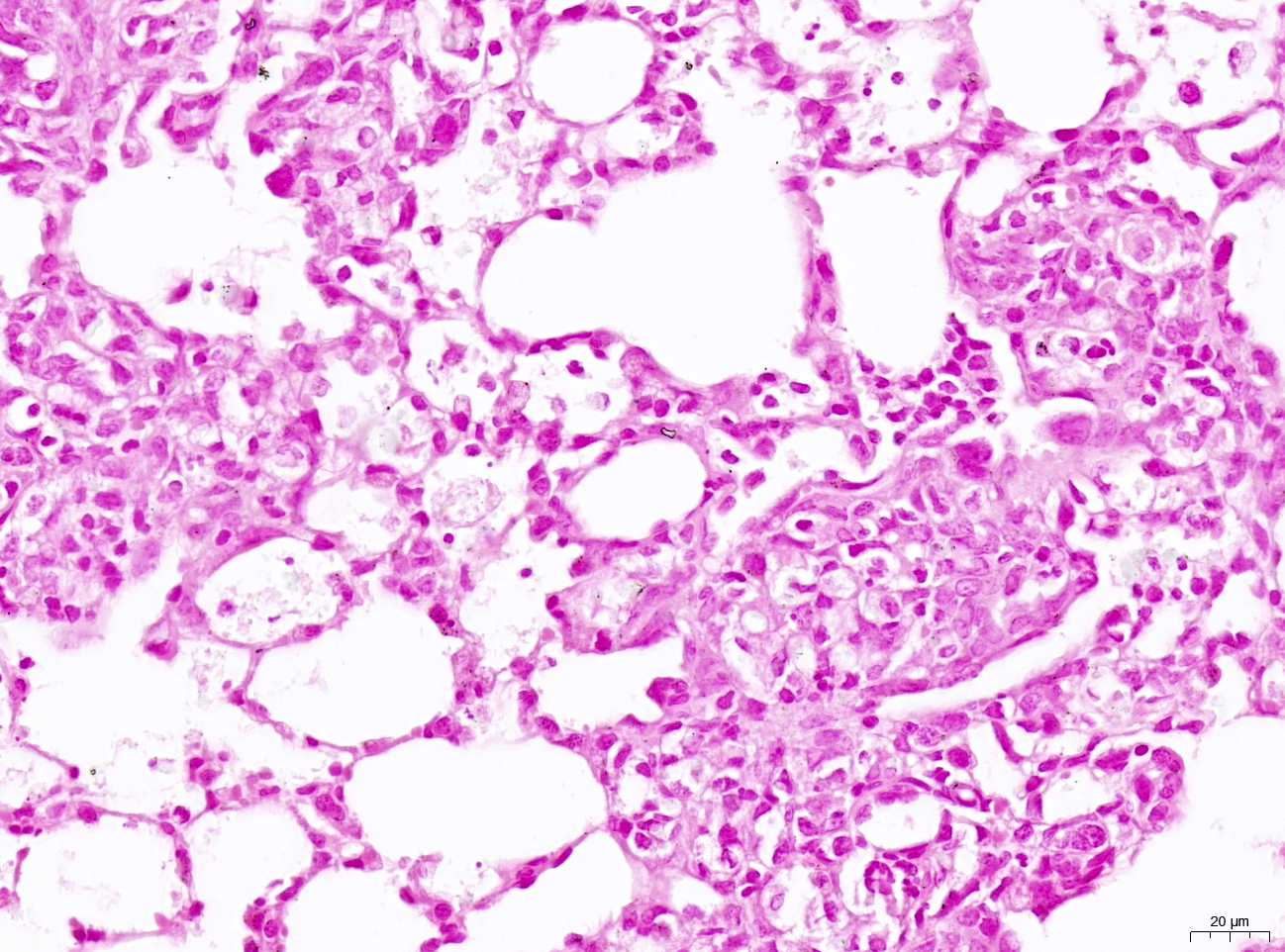

Supplement: Supplementary file 3 — Supporting File 3: advs73867‐sup‐0003‐SupportingFiguresData.zip. [file ADVS-13-e19191-s003.zip › Supporting information Figure S1-S9/S4/Figure S4B/S112_40.0x.jpg]

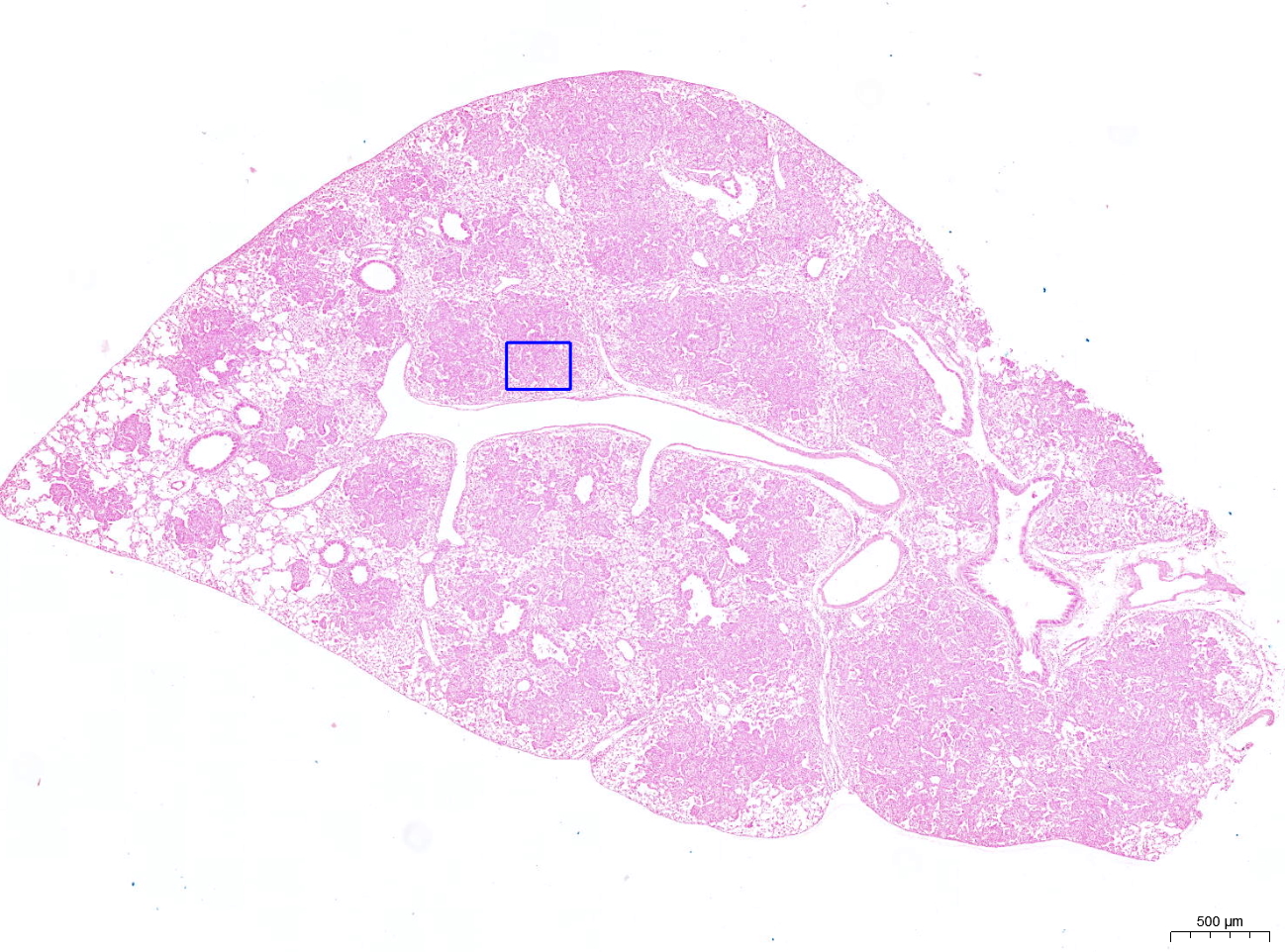

Supplement: Supplementary file 3 — Supporting File 3: advs73867‐sup‐0003‐SupportingFiguresData.zip. [file ADVS-13-e19191-s003.zip › Supporting information Figure S1-S9/S4/Figure S4B/SF-120-1_2.0x.jpg]

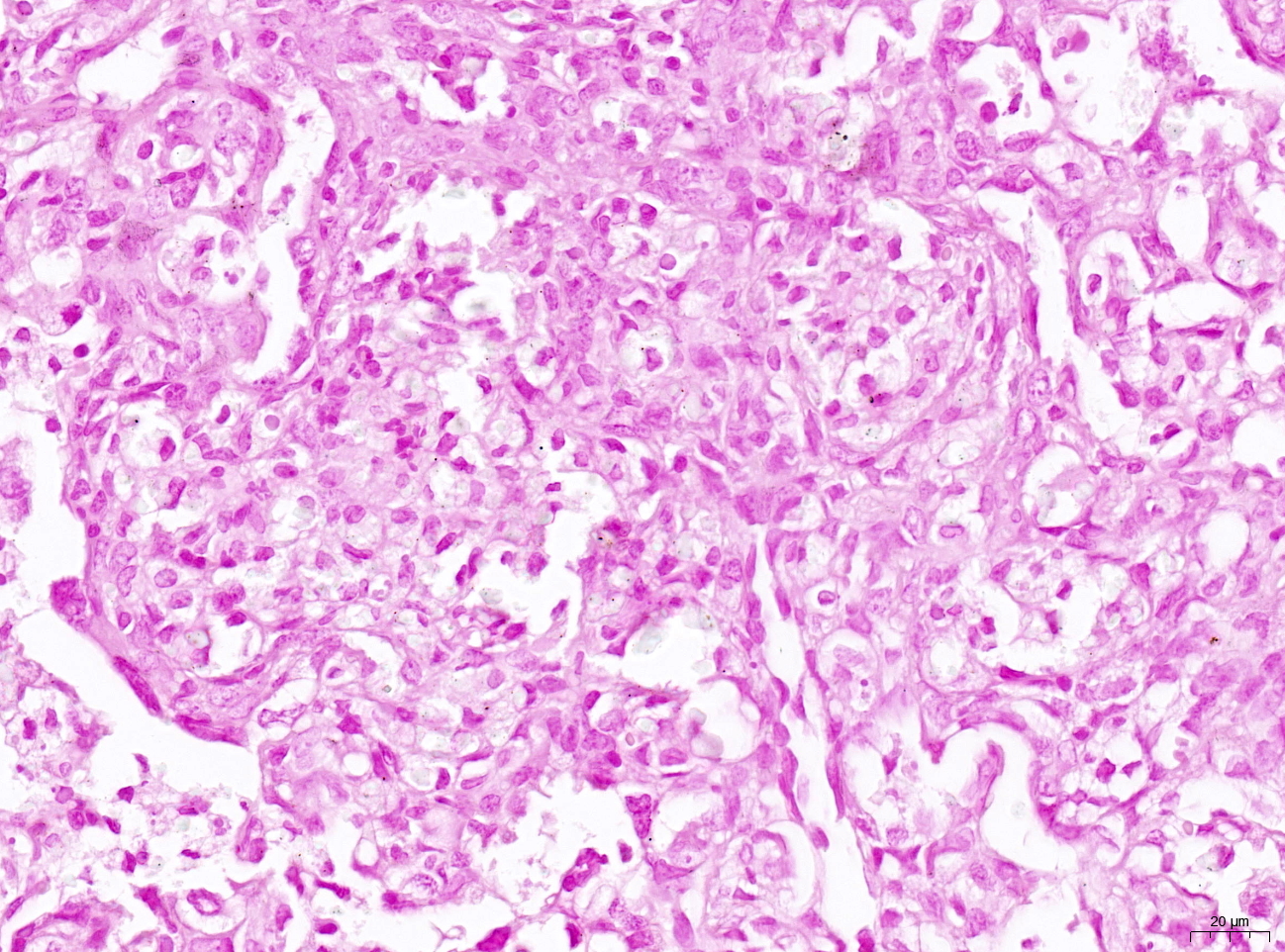

Supplement: Supplementary file 3 — Supporting File 3: advs73867‐sup‐0003‐SupportingFiguresData.zip. [file ADVS-13-e19191-s003.zip › Supporting information Figure S1-S9/S4/Figure S4B/SF-120-1_40.0x.jpg]

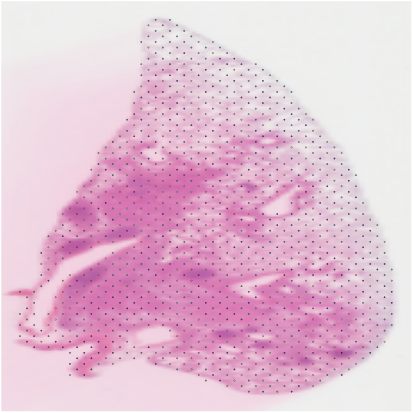

Supplement: Supplementary file 3 — Supporting File 3: advs73867‐sup‐0003‐SupportingFiguresData.zip. [file ADVS-13-e19191-s003.zip › Supporting information Figure S1-S9/S5/Figure S5A.png]

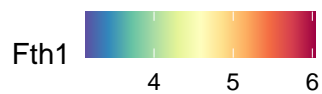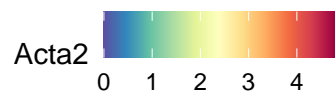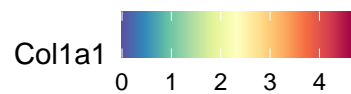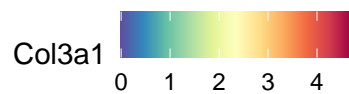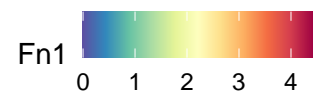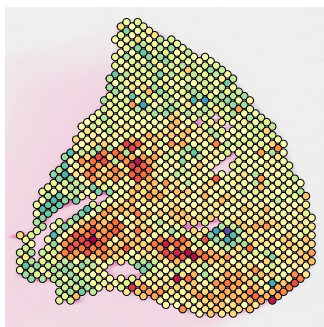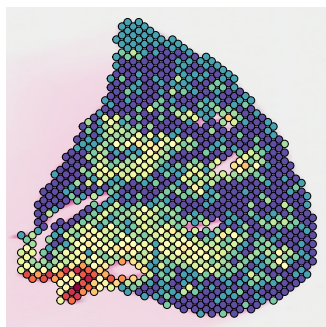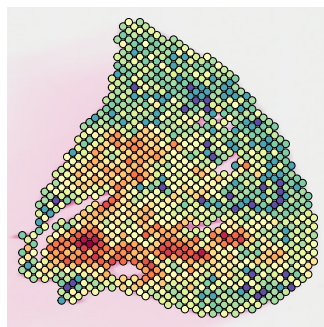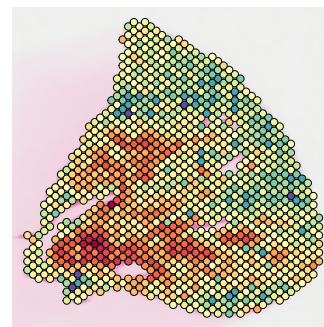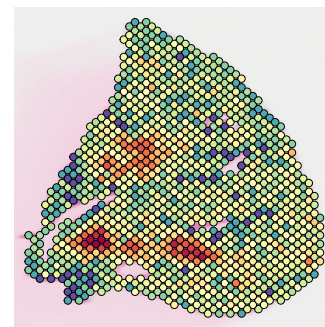

Supplement: Supplementary file 3 — Supporting File 3: advs73867‐sup‐0003‐SupportingFiguresData.zip. [file ADVS-13-e19191-s003.zip › Supporting information Figure S1-S9/S5/Figure S5B-F.pdf]

### Figure S6B, C

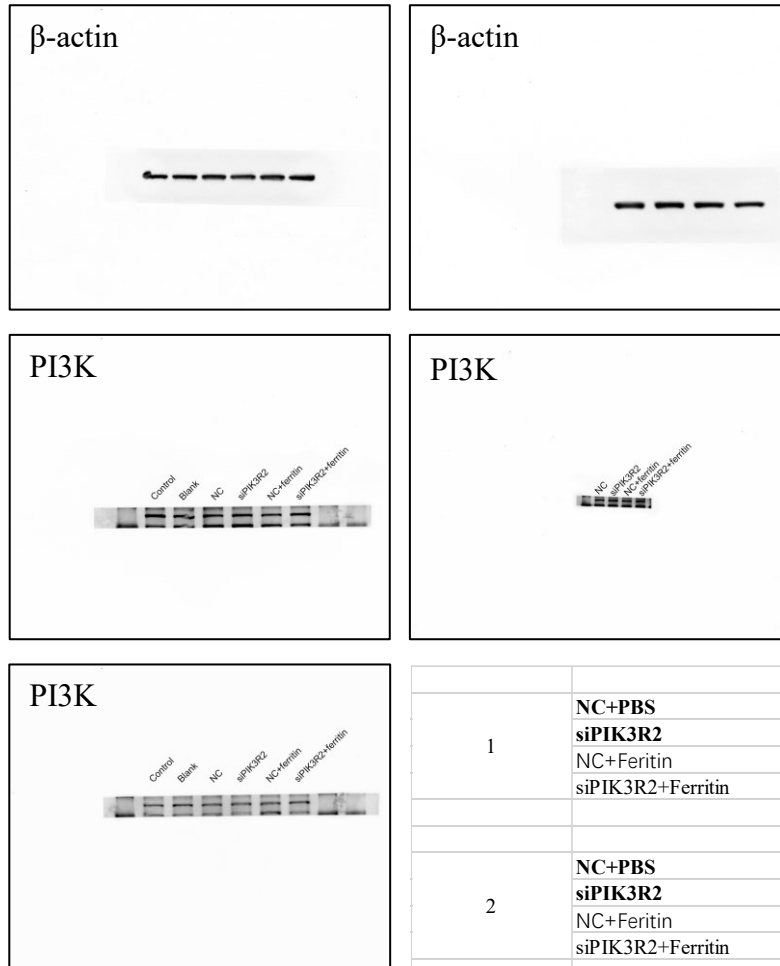[illegible]

### Figure S6B, C

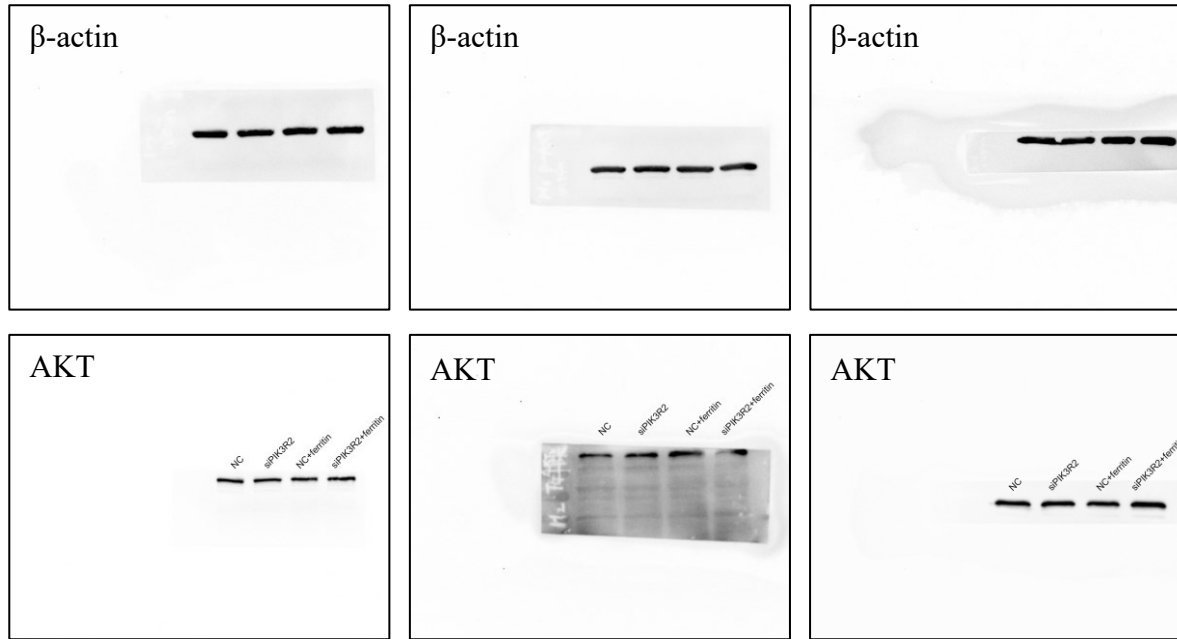[illegible]

### Figure S6B, C

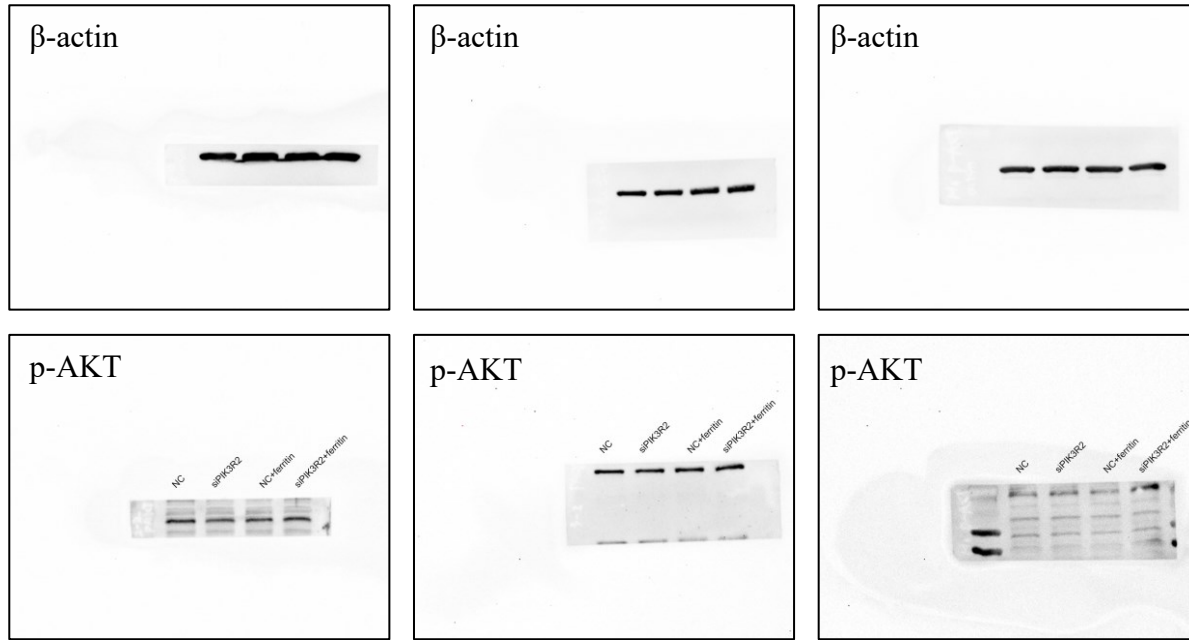[illegible]

Supplement: Supplementary file 3 — Supporting File 3: advs73867‐sup‐0003‐SupportingFiguresData.zip. [file ADVS-13-e19191-s003.zip › Supporting information Figure S1-S9/S6/Figure S6B,C.pdf]

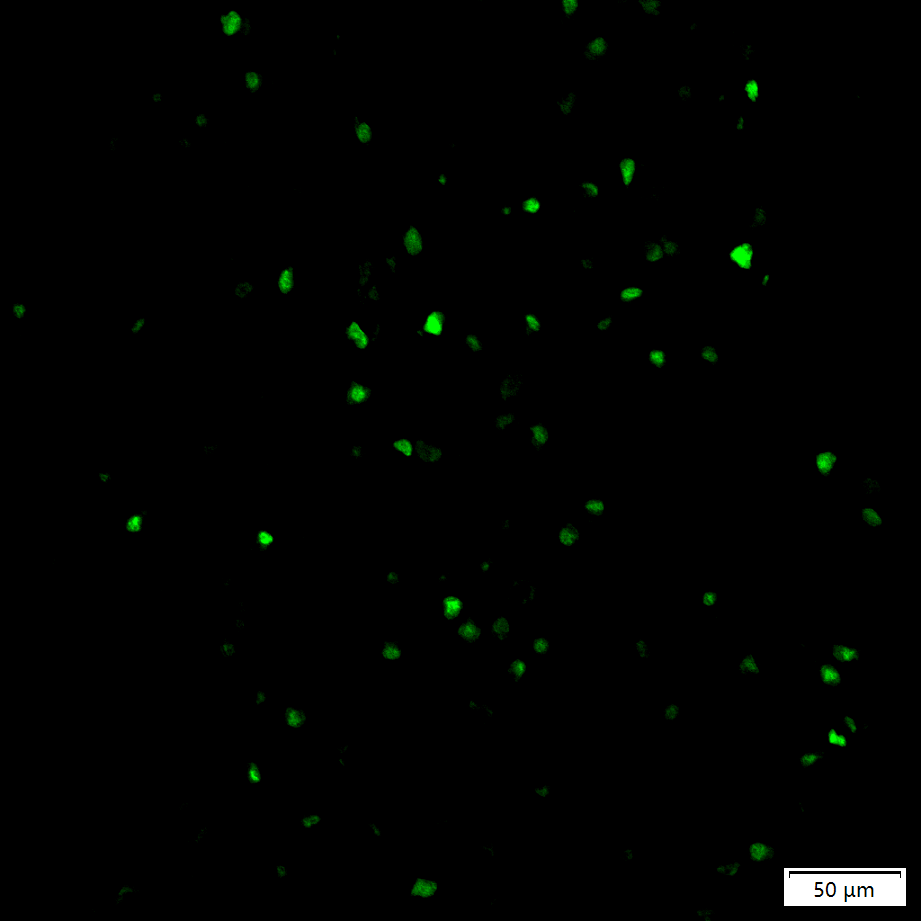

Supplement: Supplementary file 3 — Supporting File 3: advs73867‐sup‐0003‐SupportingFiguresData.zip. [file ADVS-13-e19191-s003.zip › Supporting information Figure S1-S9/S8/Figure S8A/AAV/AAV injection (1).tif]

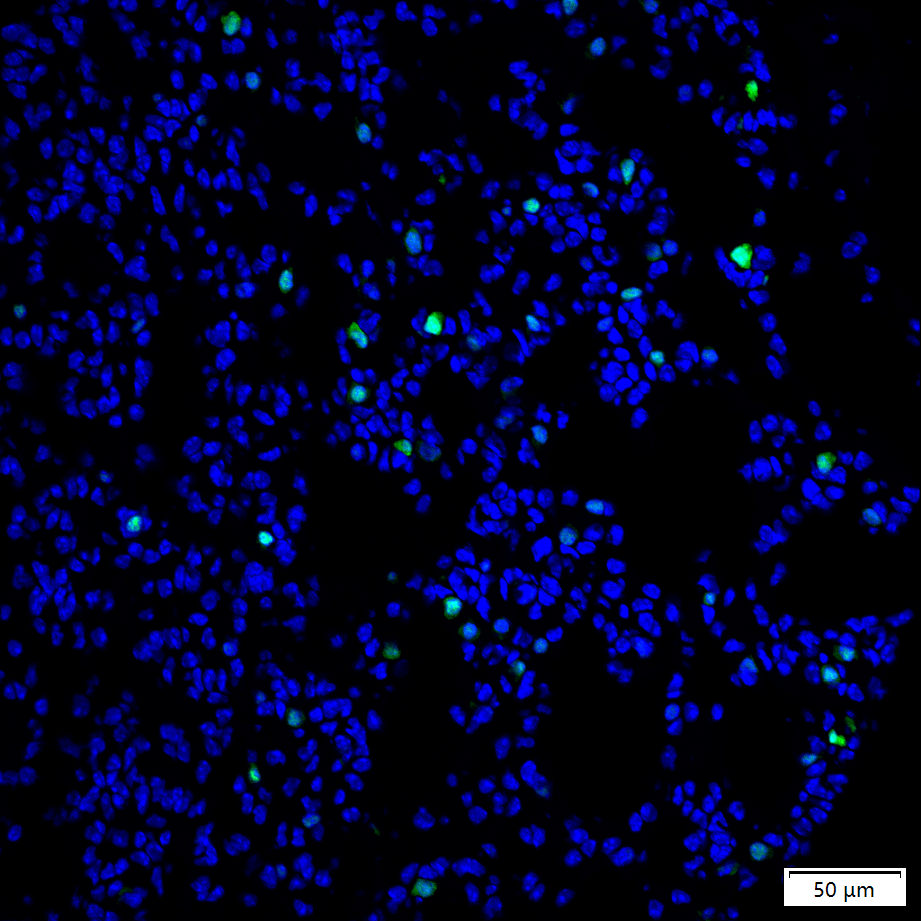

Supplement: Supplementary file 3 — Supporting File 3: advs73867‐sup‐0003‐SupportingFiguresData.zip. [file ADVS-13-e19191-s003.zip › Supporting information Figure S1-S9/S8/Figure S8A/AAV/AAV injection (2).tif]

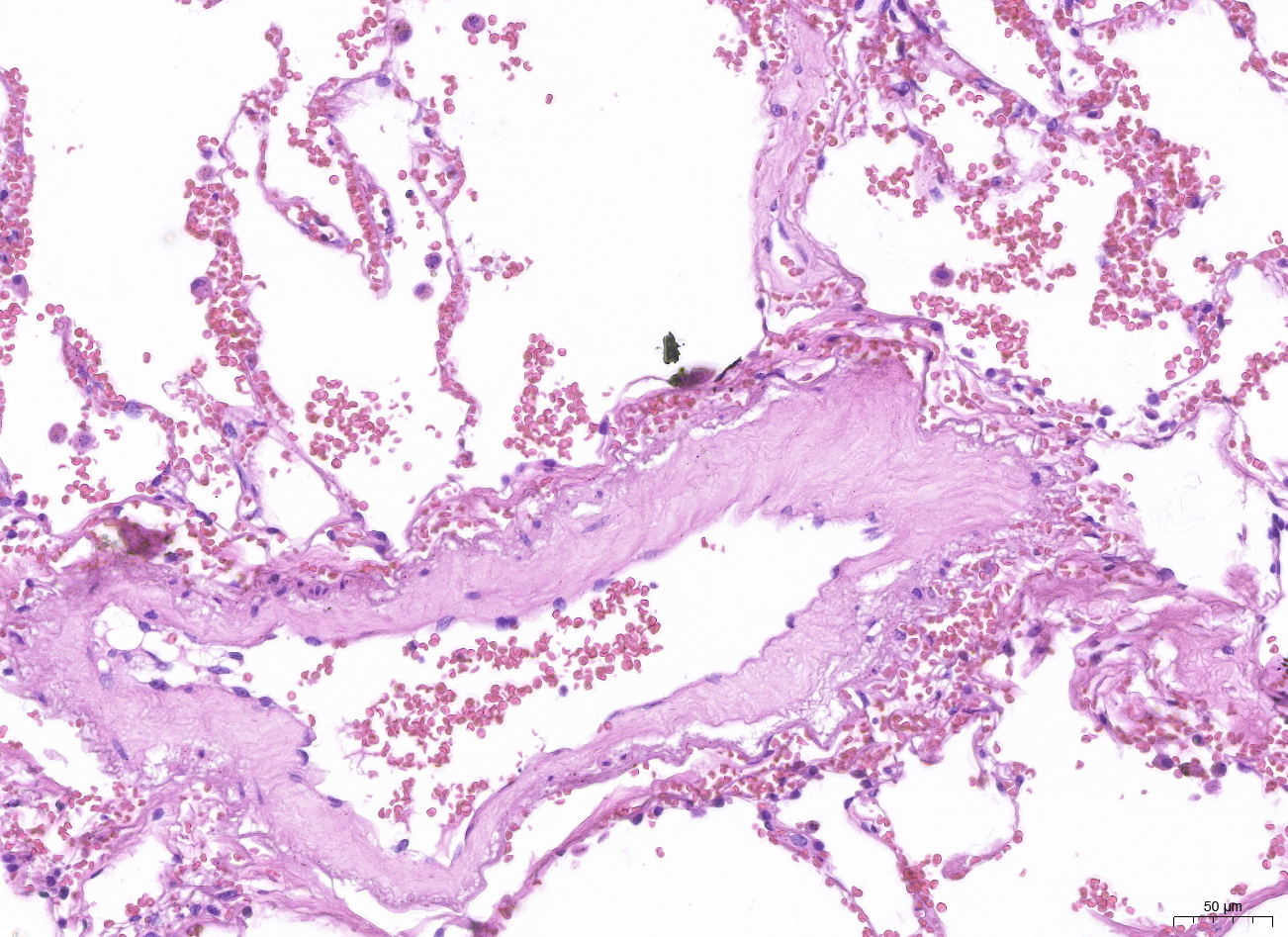

Supplement: Supplementary file 4 — Supporting File 4: advs73867‐sup‐0001‐FiguresData.zip. [file ADVS-13-e19191-s001.zip › Supporting information Figure1-10/Figure 1/Figure 1D/H&E-Control.jpg]

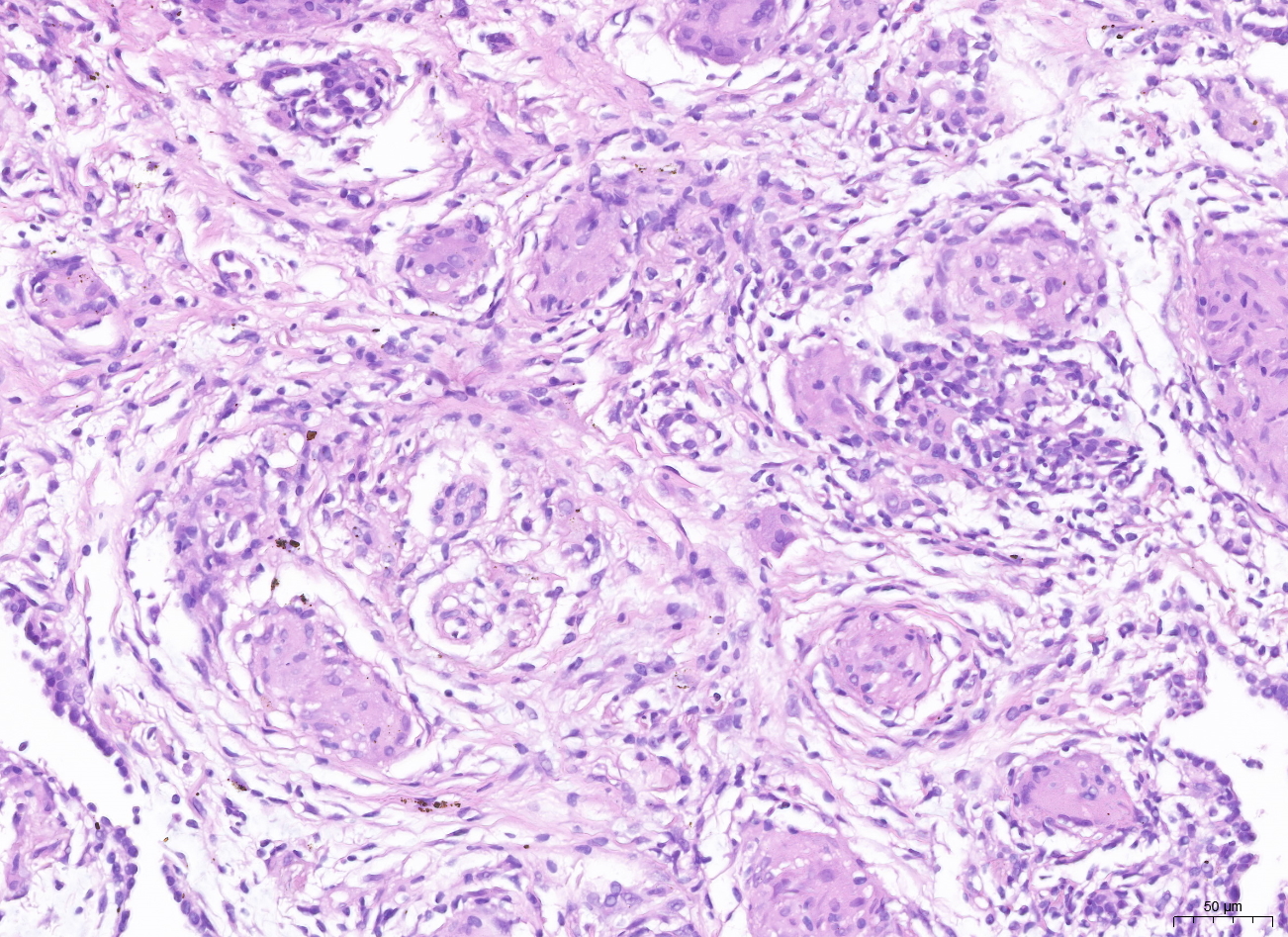

Supplement: Supplementary file 4 — Supporting File 4: advs73867‐sup‐0001‐FiguresData.zip. [file ADVS-13-e19191-s001.zip › Supporting information Figure1-10/Figure 1/Figure 1D/H&E-Silicosis-.jpg]

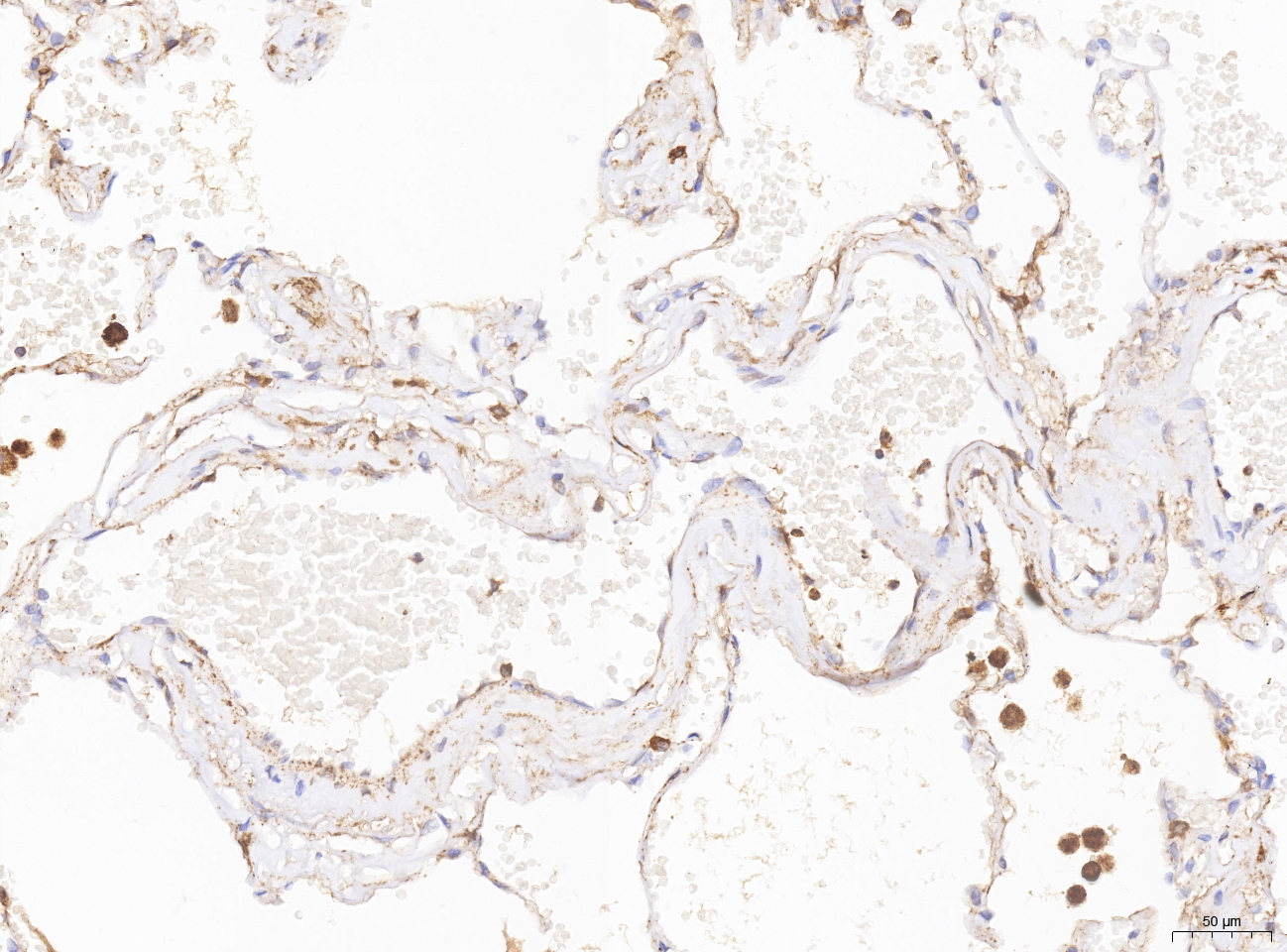

Supplement: Supplementary file 4 — Supporting File 4: advs73867‐sup‐0001‐FiguresData.zip. [file ADVS-13-e19191-s001.zip › Supporting information Figure1-10/Figure 1/Figure 1D/IHC-Control.jpg]

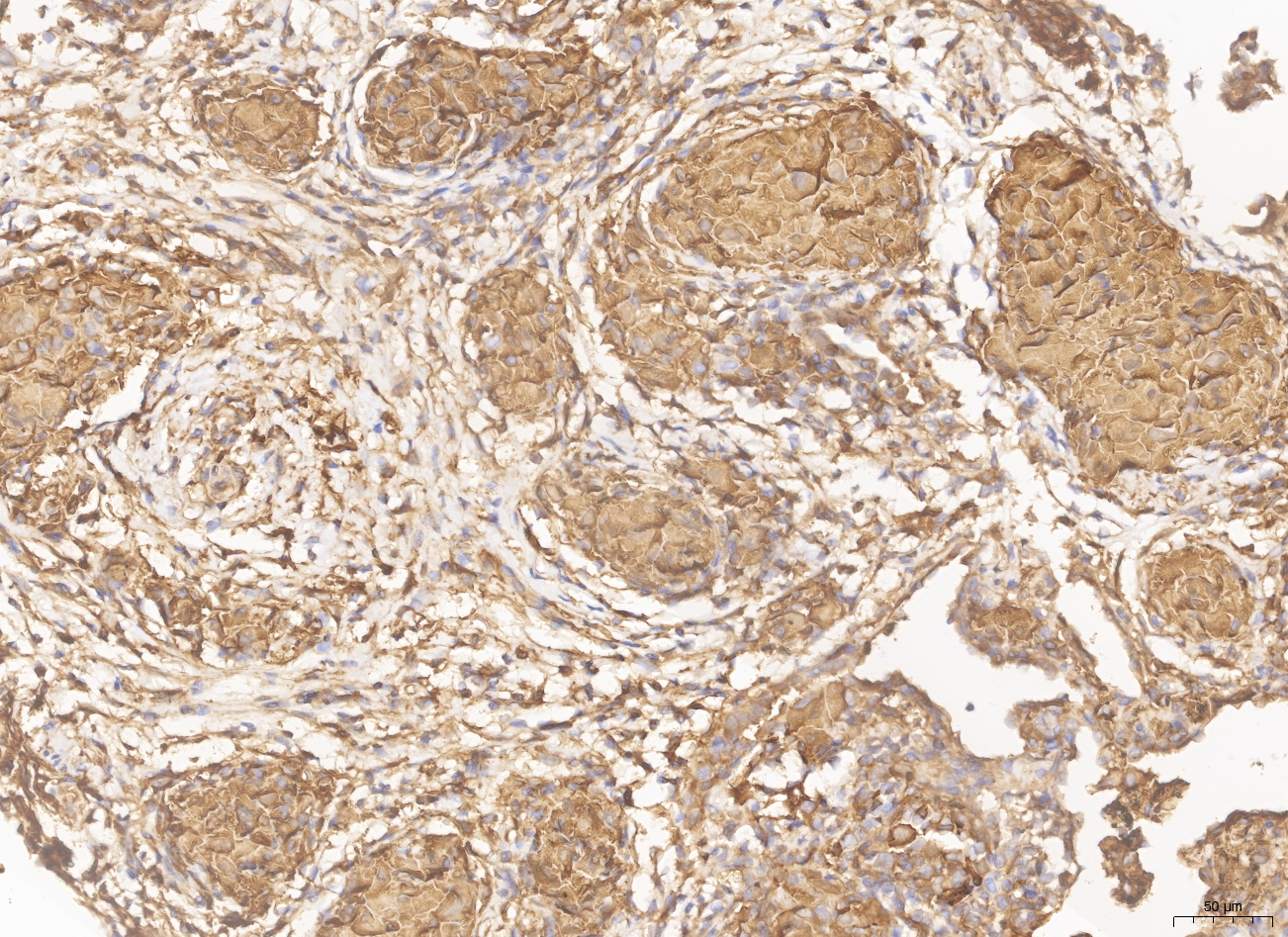

Supplement: Supplementary file 4 — Supporting File 4: advs73867‐sup‐0001‐FiguresData.zip. [file ADVS-13-e19191-s001.zip › Supporting information Figure1-10/Figure 1/Figure 1D/IHC-Silicosis.jpg]

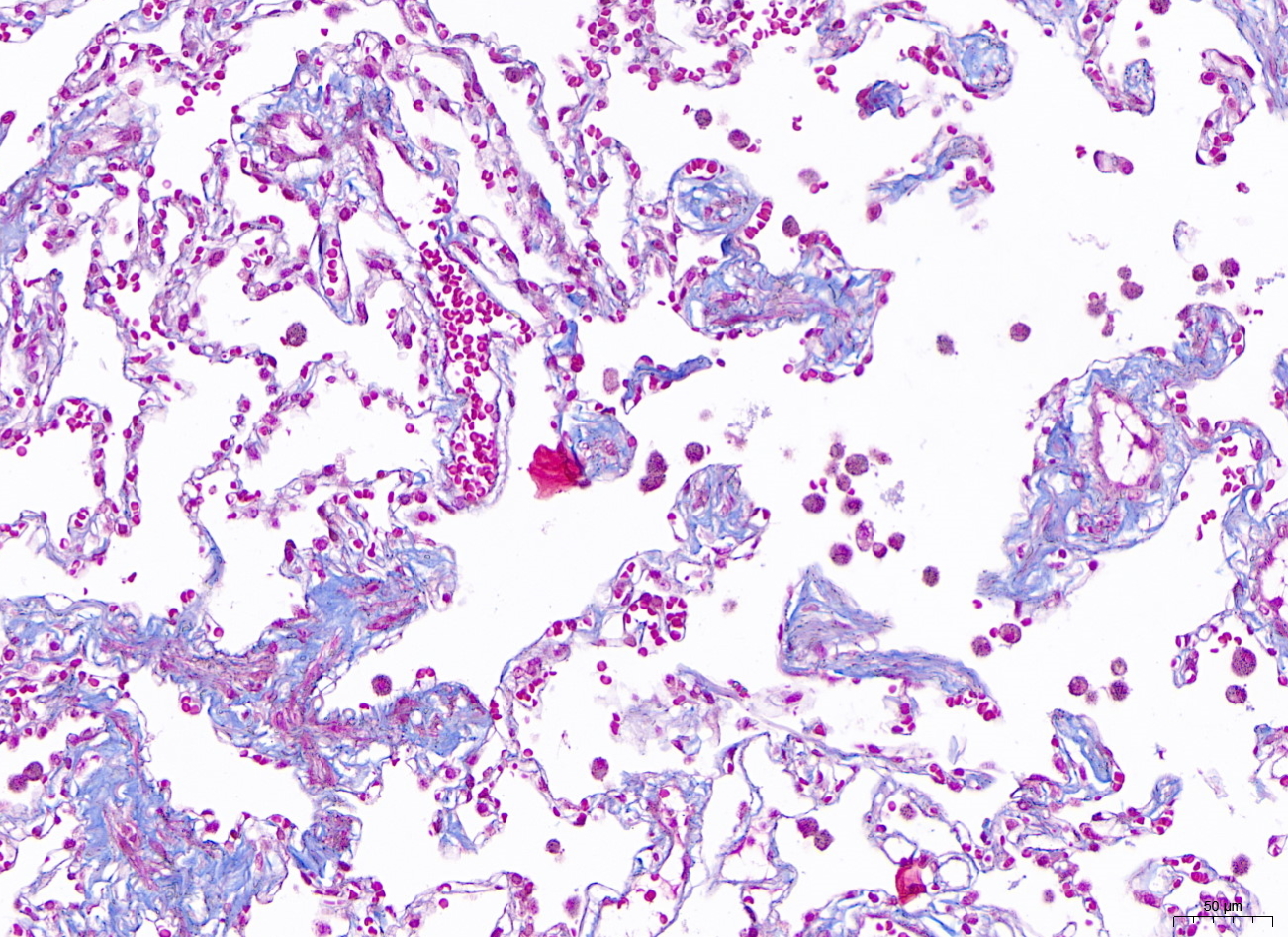

Supplement: Supplementary file 4 — Supporting File 4: advs73867‐sup‐0001‐FiguresData.zip. [file ADVS-13-e19191-s001.zip › Supporting information Figure1-10/Figure 1/Figure 1D/Masson-Control.jpg]

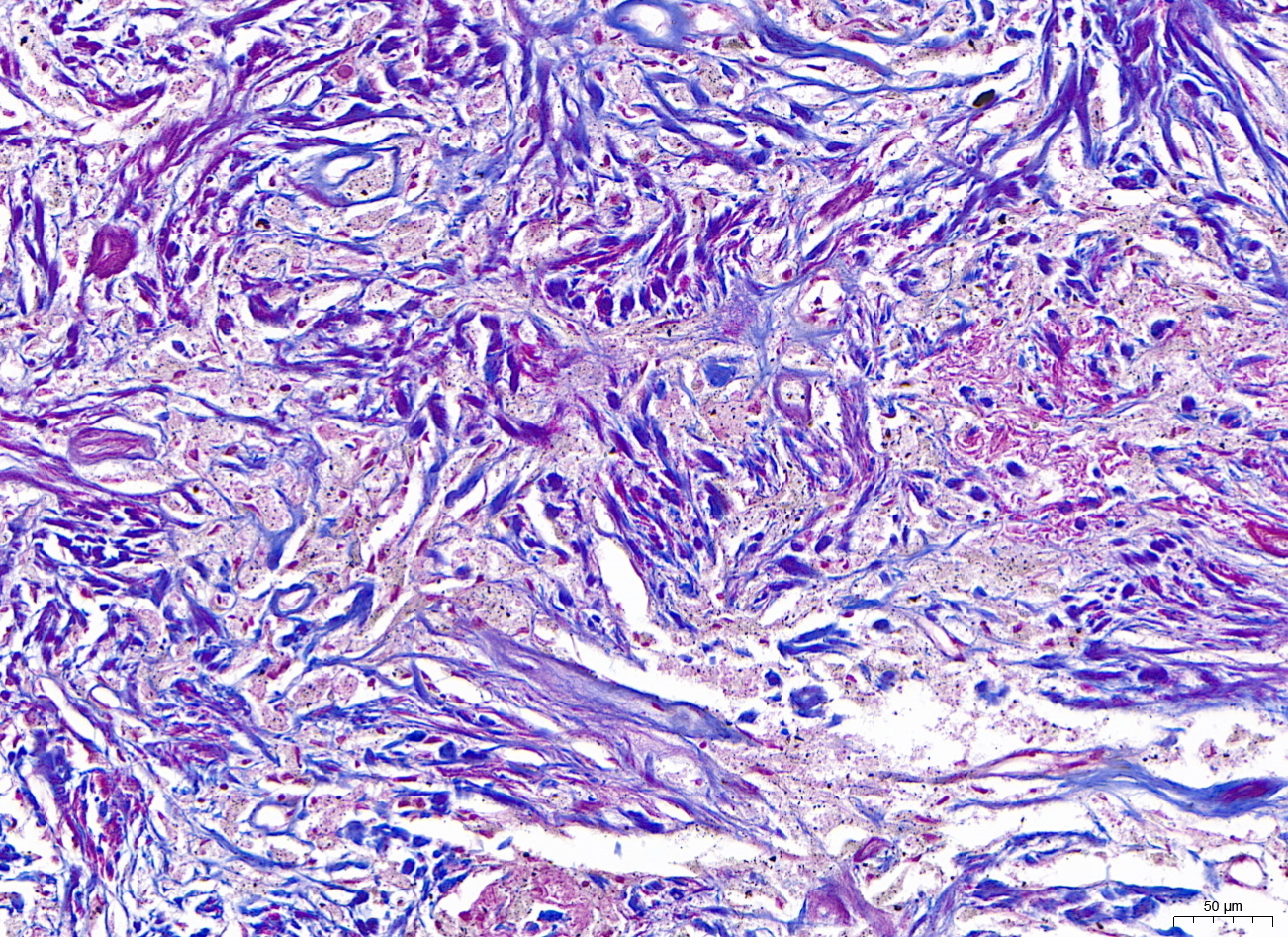

Supplement: Supplementary file 4 — Supporting File 4: advs73867‐sup‐0001‐FiguresData.zip. [file ADVS-13-e19191-s001.zip › Supporting information Figure1-10/Figure 1/Figure 1D/Masson-Silicosis.jpg]

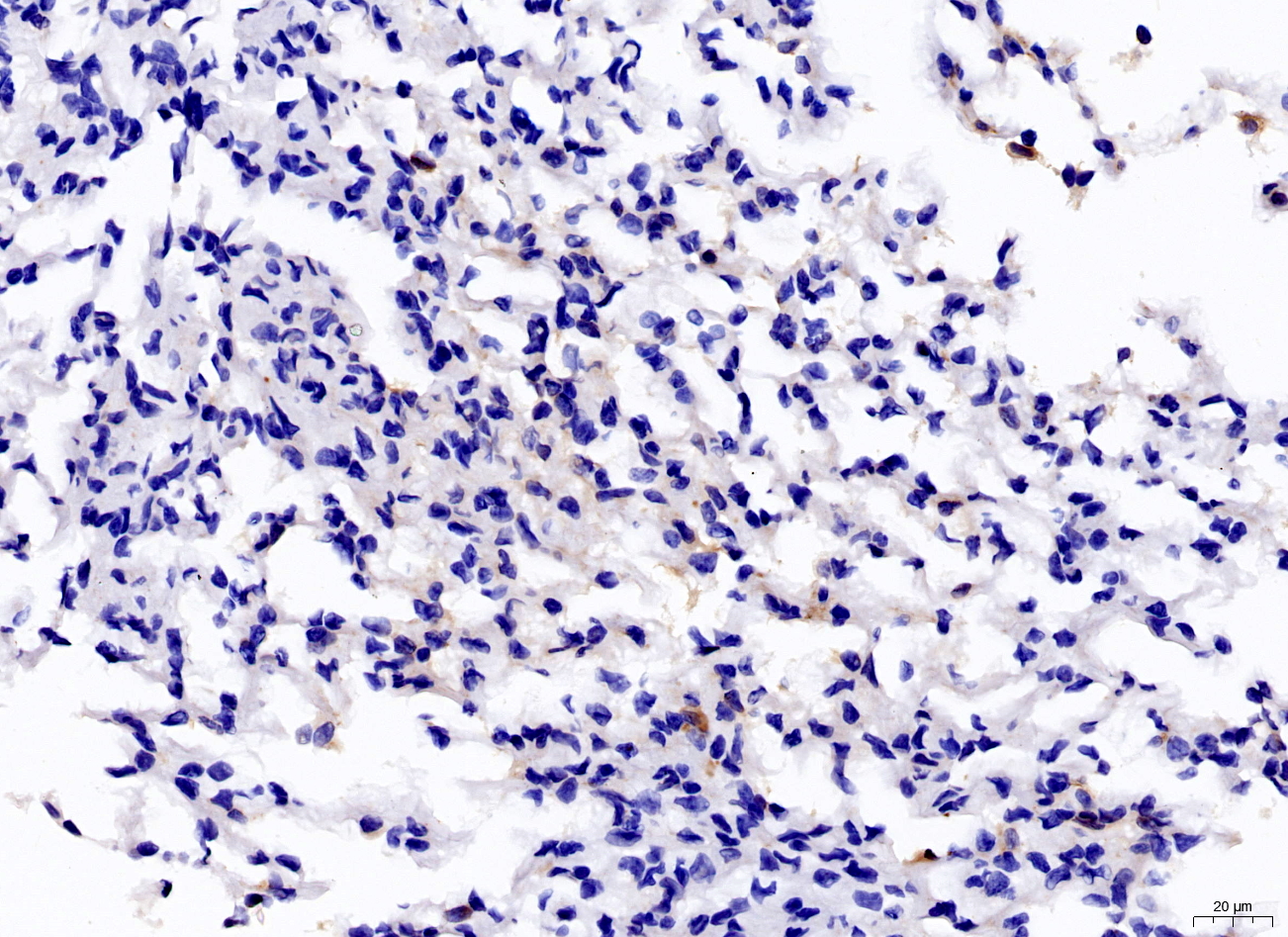

Supplement: Supplementary file 4 — Supporting File 4: advs73867‐sup‐0001‐FiguresData.zip. [file ADVS-13-e19191-s001.zip › Supporting information Figure1-10/Figure 2/Figure 2I/SCRS-1 week-Control-779-1_40.0x.jpg]

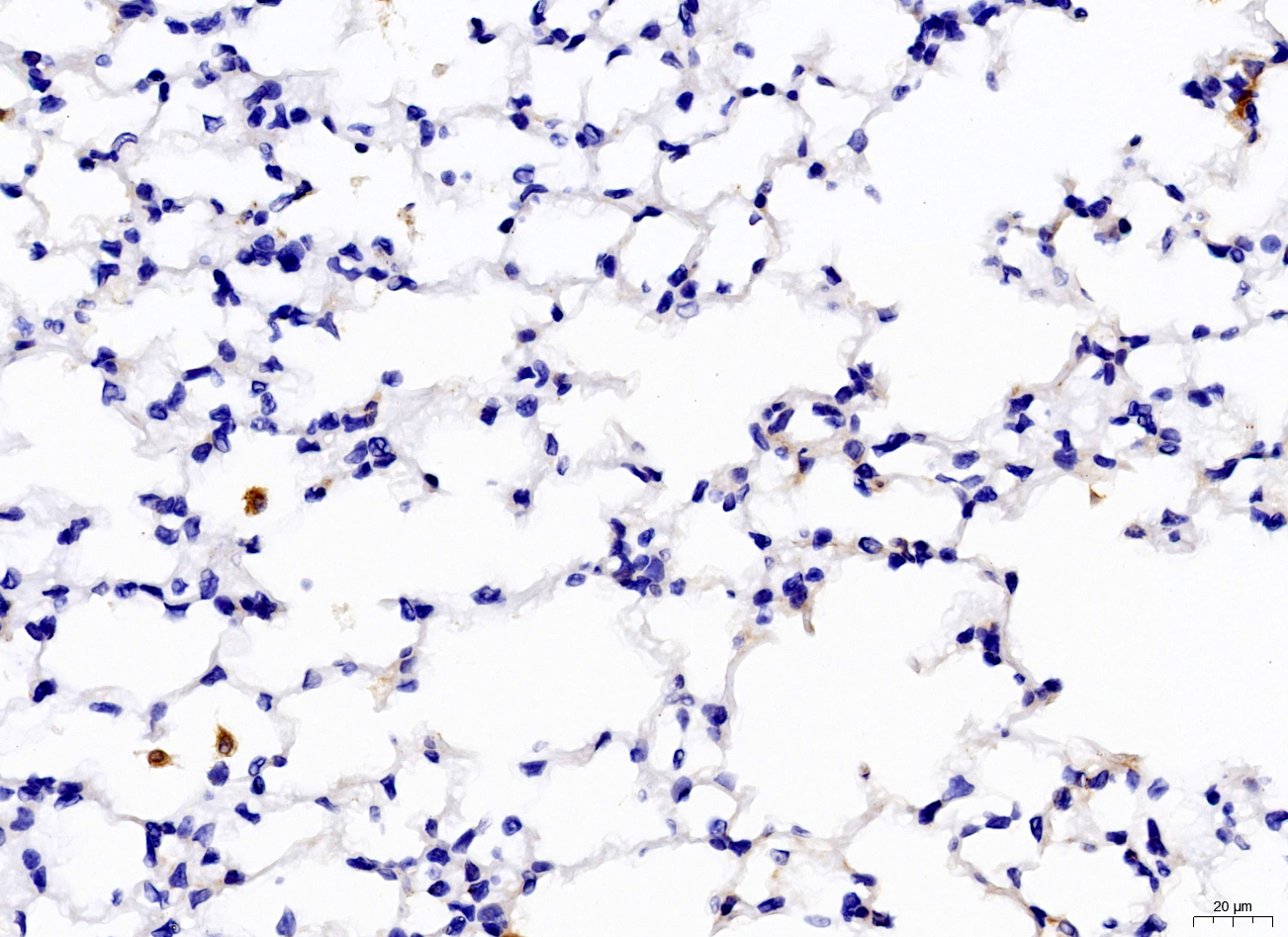

Supplement: Supplementary file 4 — Supporting File 4: advs73867‐sup‐0001‐FiguresData.zip. [file ADVS-13-e19191-s001.zip › Supporting information Figure1-10/Figure 2/Figure 2I/SCRS-1 week-Control-792_40.0x.jpg]

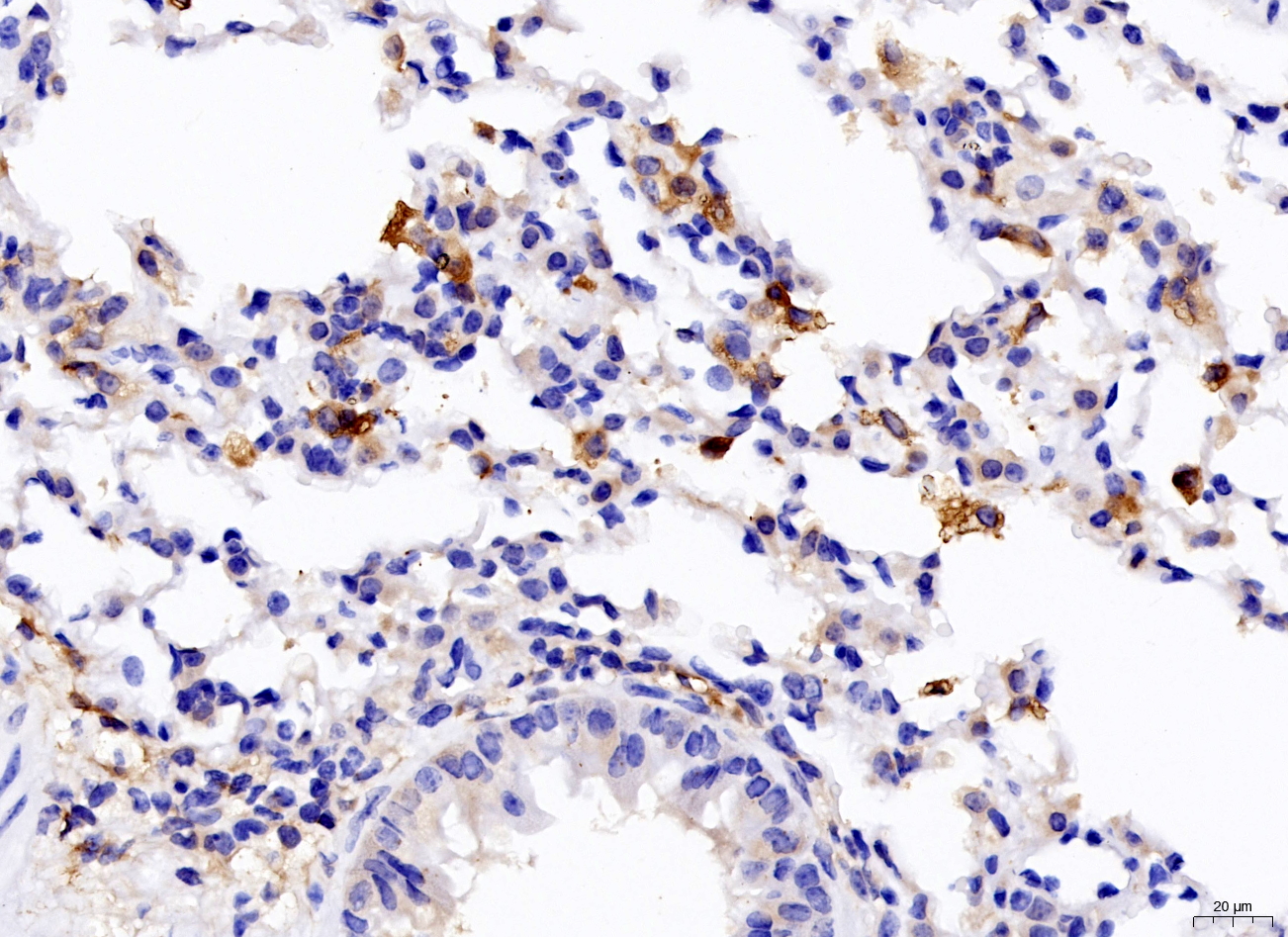

Supplement: Supplementary file 4 — Supporting File 4: advs73867‐sup‐0001‐FiguresData.zip. [file ADVS-13-e19191-s001.zip › Supporting information Figure1-10/Figure 2/Figure 2I/SCRS-1 week-Model-762_40.0x.jpg]

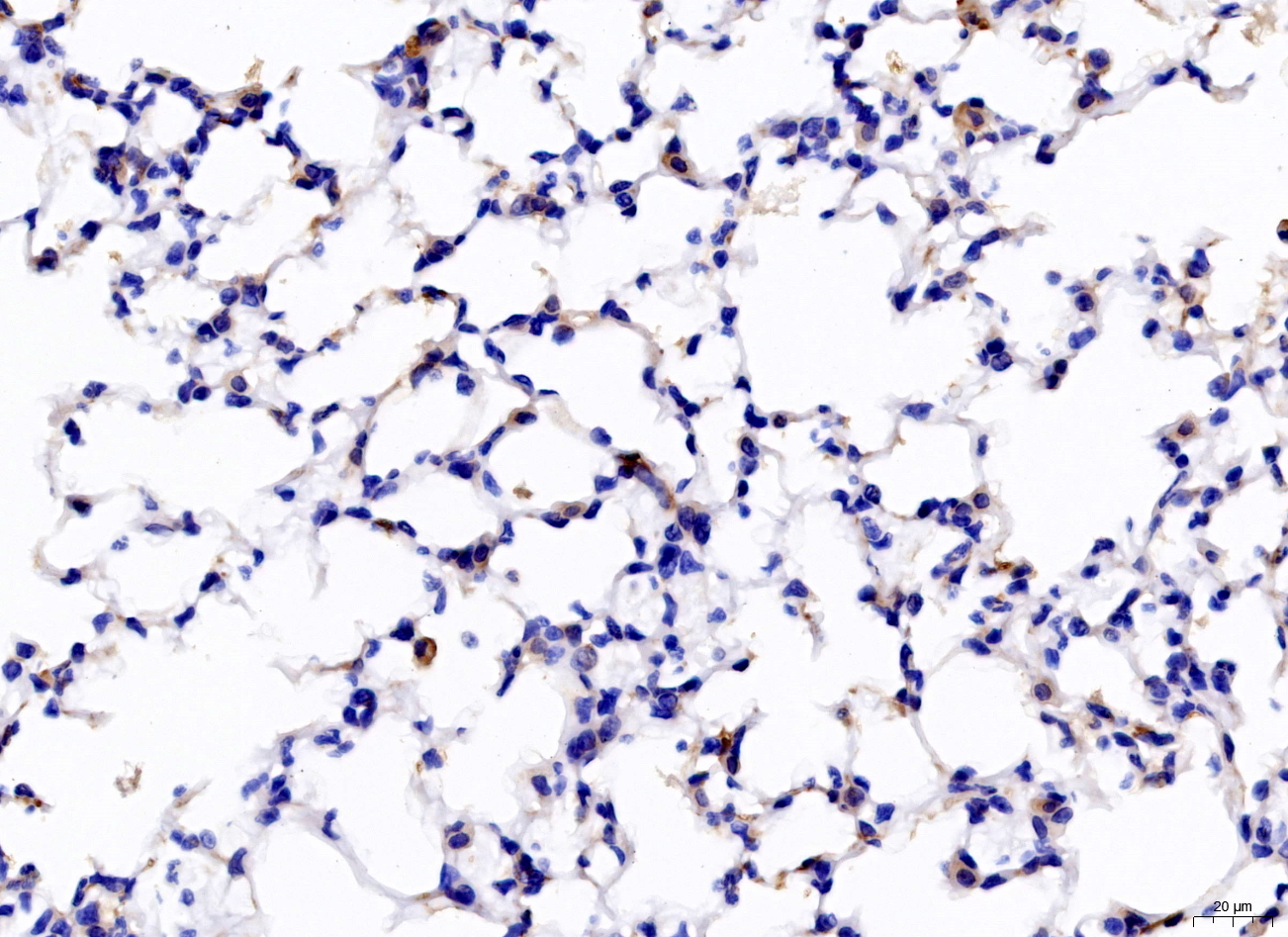

Supplement: Supplementary file 4 — Supporting File 4: advs73867‐sup‐0001‐FiguresData.zip. [file ADVS-13-e19191-s001.zip › Supporting information Figure1-10/Figure 2/Figure 2I/SCRS-Ferritin-12w-Control-788_40.0x.jpg]
