# Supplementary figures and images for: Macrophage–Derived Ferritin Exacerbates Silica‐Induced Pulmonary Fibrosis via PIK3R2‐Mediated Fibroblast Differentiation (part 1 of 4)
Source: Adv Sci (Weinh). 2026 Jan 21;13(17):e19191. doi: 10.1002/advs.202519191 (PMC13042690; doi:10.1002/advs.202519191)

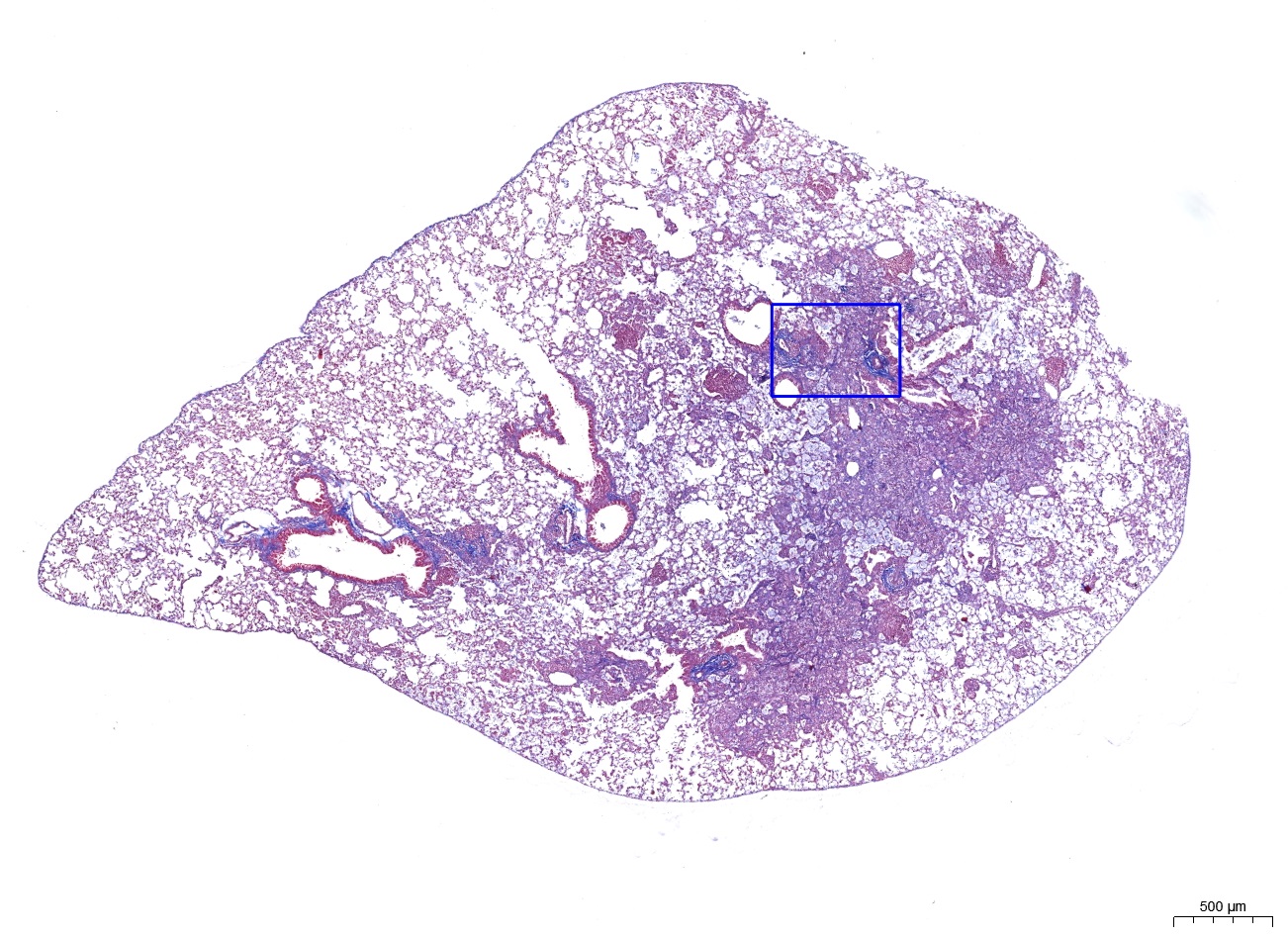

Supplement: Supplementary file 3 — Supporting File 3: advs73867‐sup‐0003‐SupportingFiguresData.zip. [file ADVS-13-e19191-s003.zip › Supporting information Figure S1-S9/S1/Figure S1B/12W-Model-1775_2.0x.jpg]

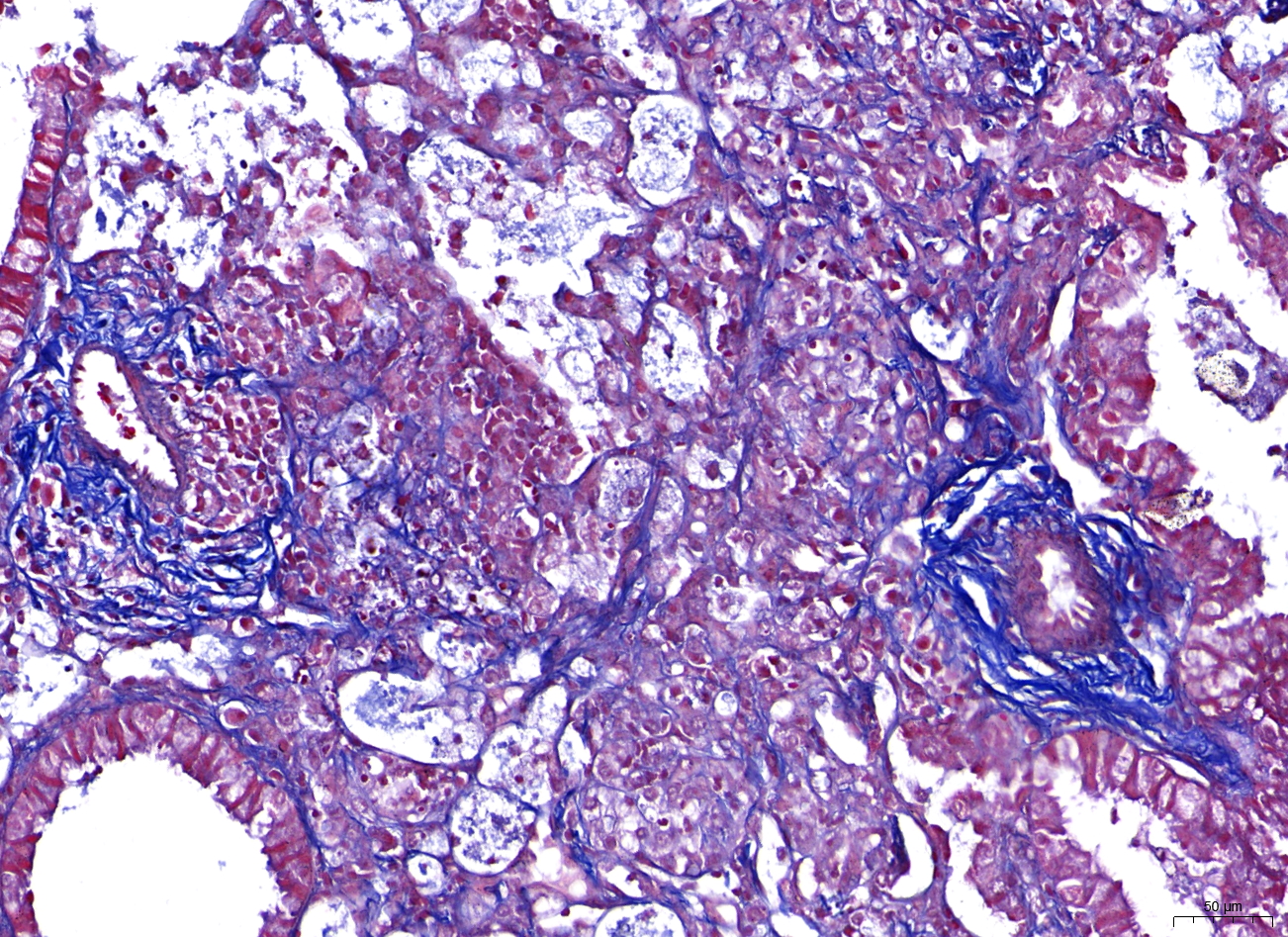

Supplement: Supplementary file 3 — Supporting File 3: advs73867‐sup‐0003‐SupportingFiguresData.zip. [file ADVS-13-e19191-s003.zip › Supporting information Figure S1-S9/S1/Figure S1B/12W-Model-1775_20.0x.jpg]

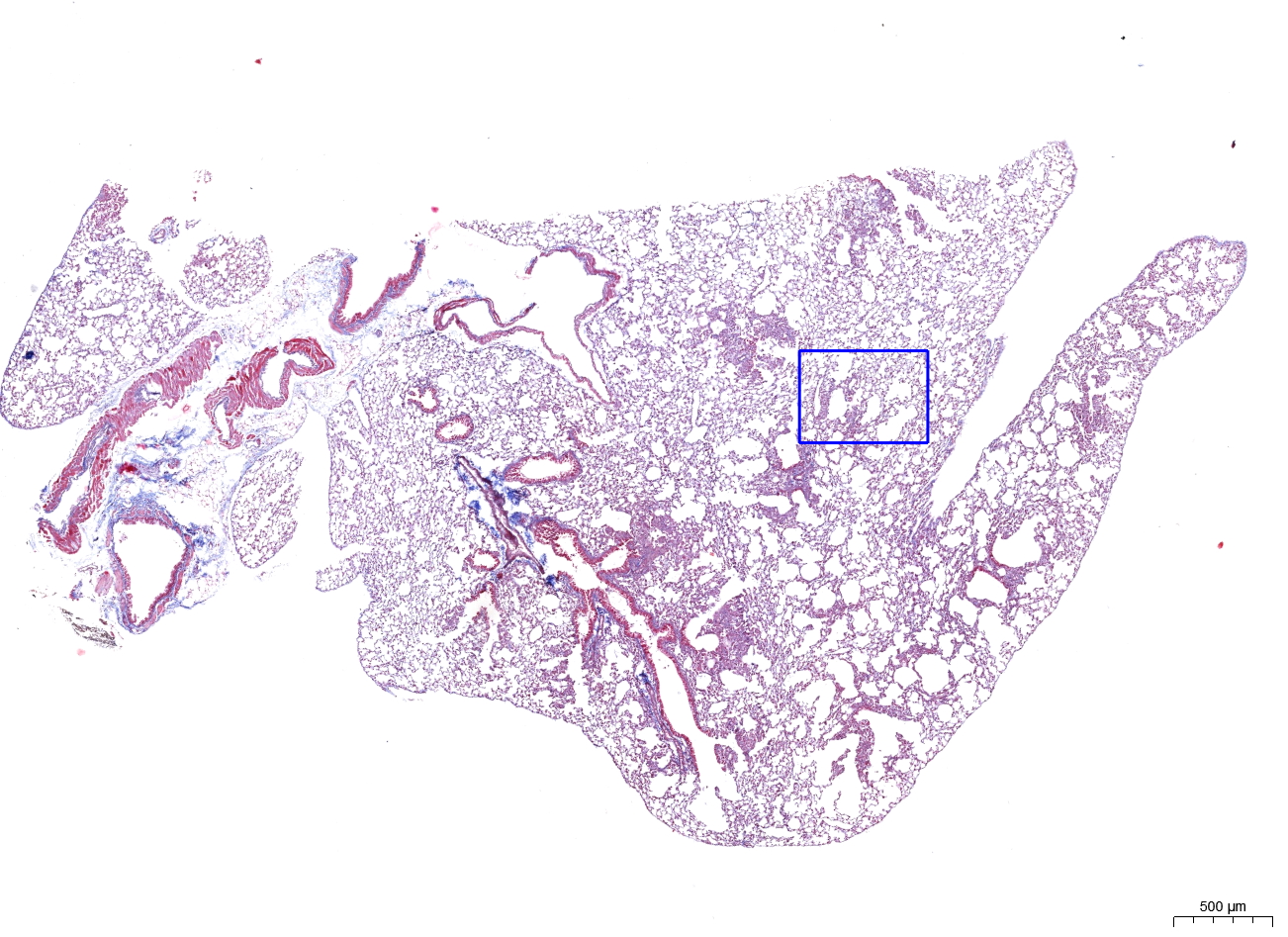

Supplement: Supplementary file 3 — Supporting File 3: advs73867‐sup‐0003‐SupportingFiguresData.zip. [file ADVS-13-e19191-s003.zip › Supporting information Figure S1-S9/S1/Figure S1B/1W-Model-769_2.0x.jpg]

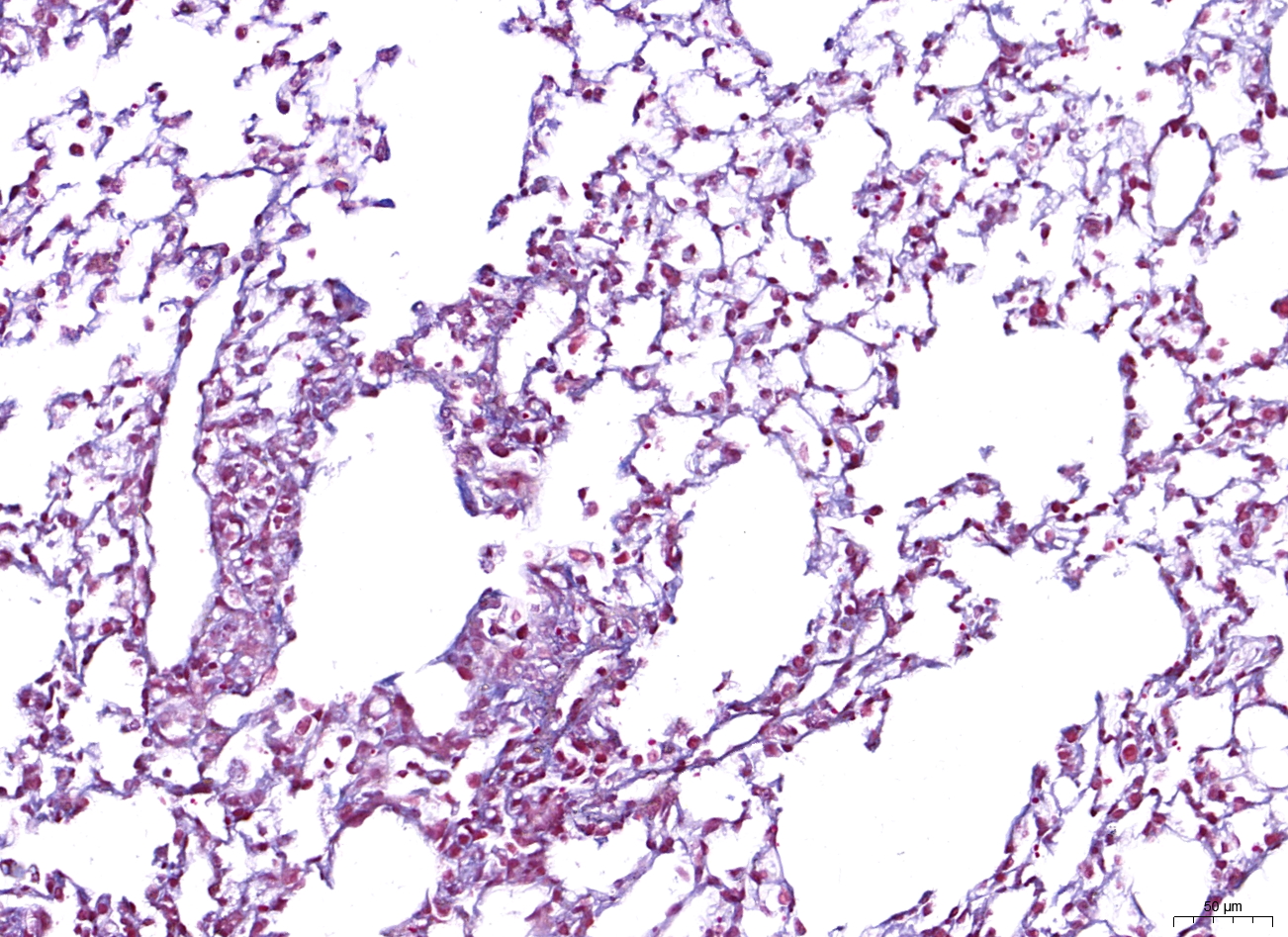

Supplement: Supplementary file 3 — Supporting File 3: advs73867‐sup‐0003‐SupportingFiguresData.zip. [file ADVS-13-e19191-s003.zip › Supporting information Figure S1-S9/S1/Figure S1B/1W-Model-769_20.0x.jpg]

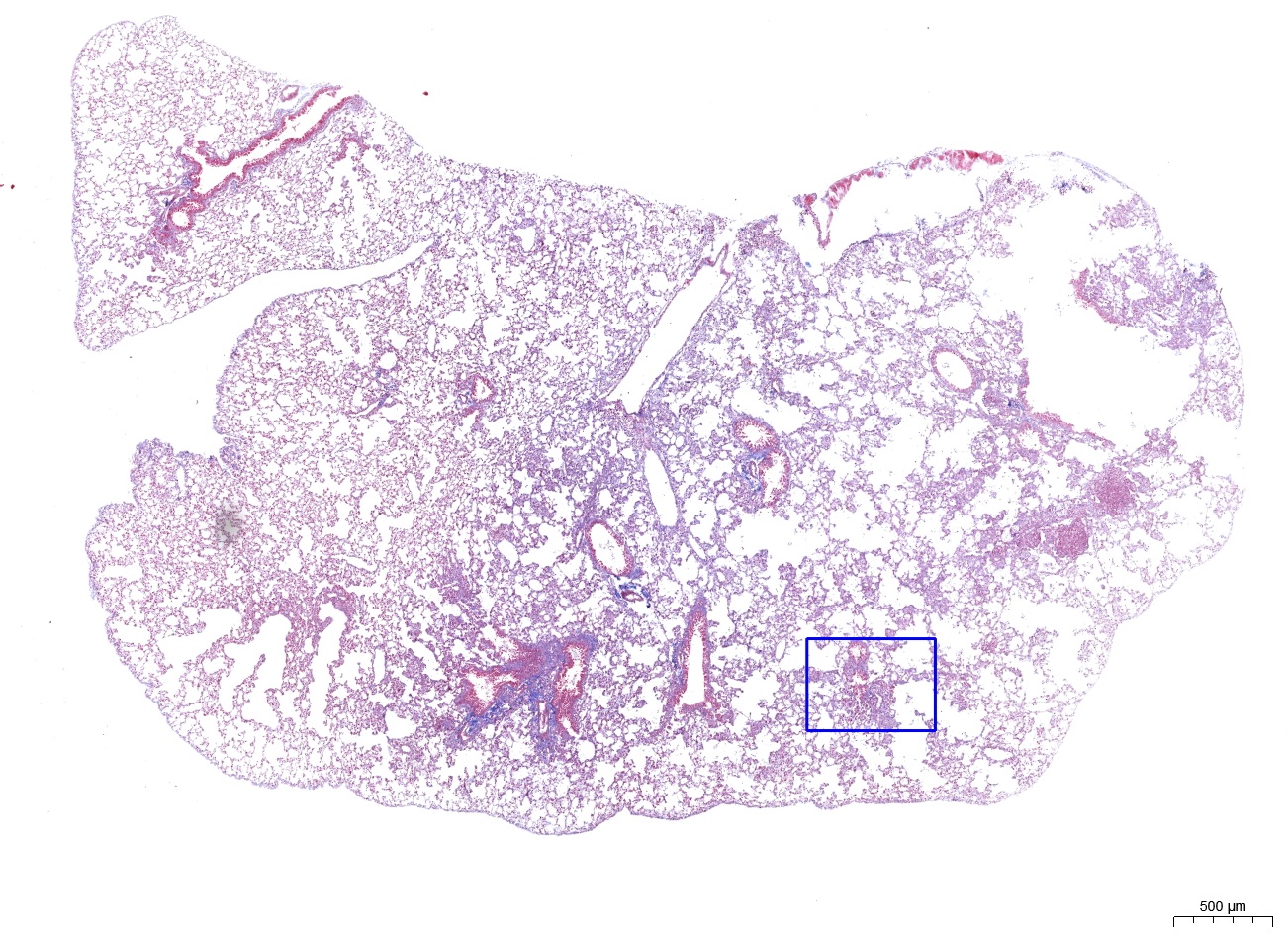

Supplement: Supplementary file 3 — Supporting File 3: advs73867‐sup‐0003‐SupportingFiguresData.zip. [file ADVS-13-e19191-s003.zip › Supporting information Figure S1-S9/S1/Figure S1B/4W-Model-764_2.0x.jpg]

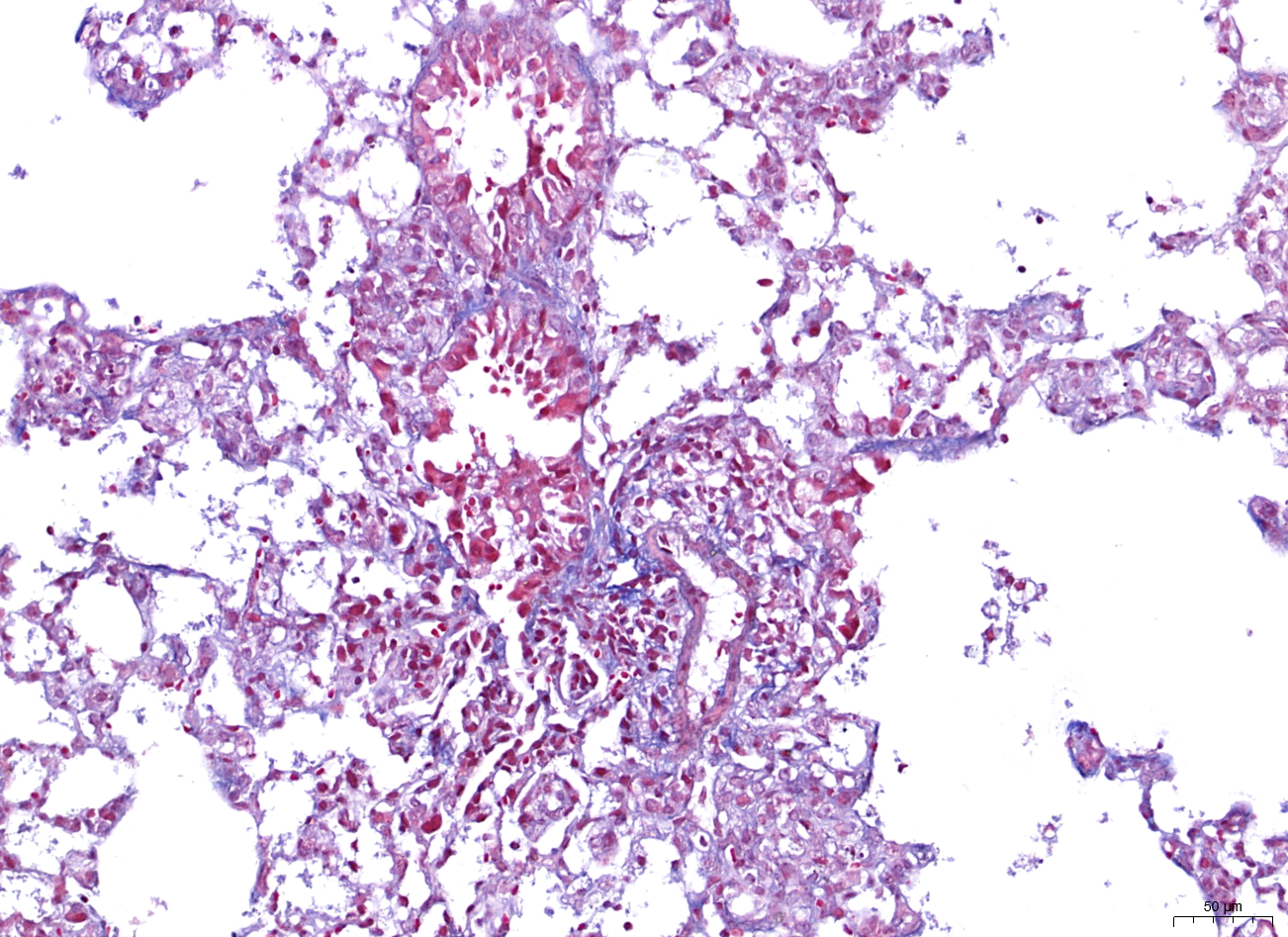

Supplement: Supplementary file 3 — Supporting File 3: advs73867‐sup‐0003‐SupportingFiguresData.zip. [file ADVS-13-e19191-s003.zip › Supporting information Figure S1-S9/S1/Figure S1B/4W-Model-764_20.0x.jpg]

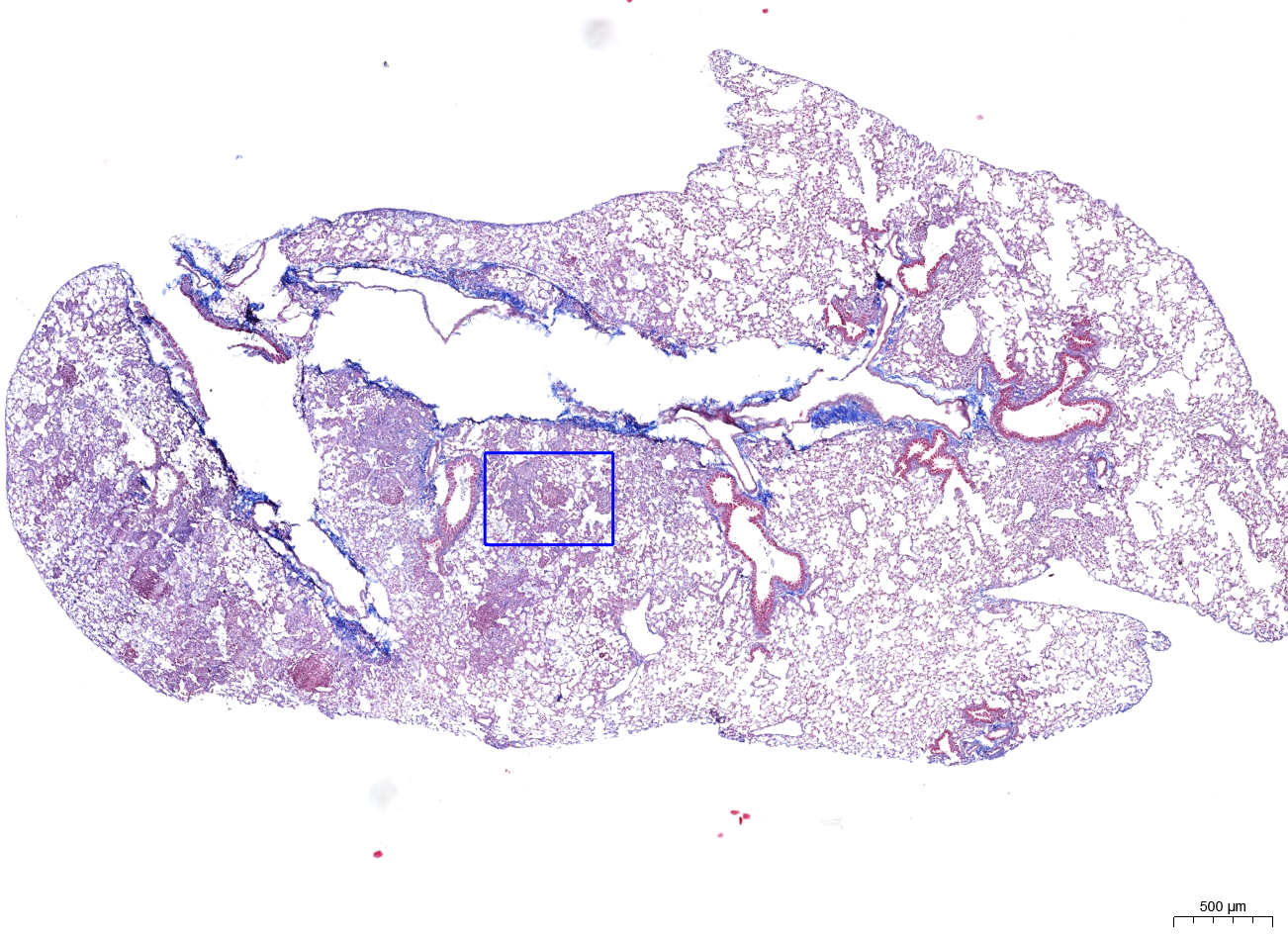

Supplement: Supplementary file 3 — Supporting File 3: advs73867‐sup‐0003‐SupportingFiguresData.zip. [file ADVS-13-e19191-s003.zip › Supporting information Figure S1-S9/S1/Figure S1B/8W-Model-763_2.0x.jpg]

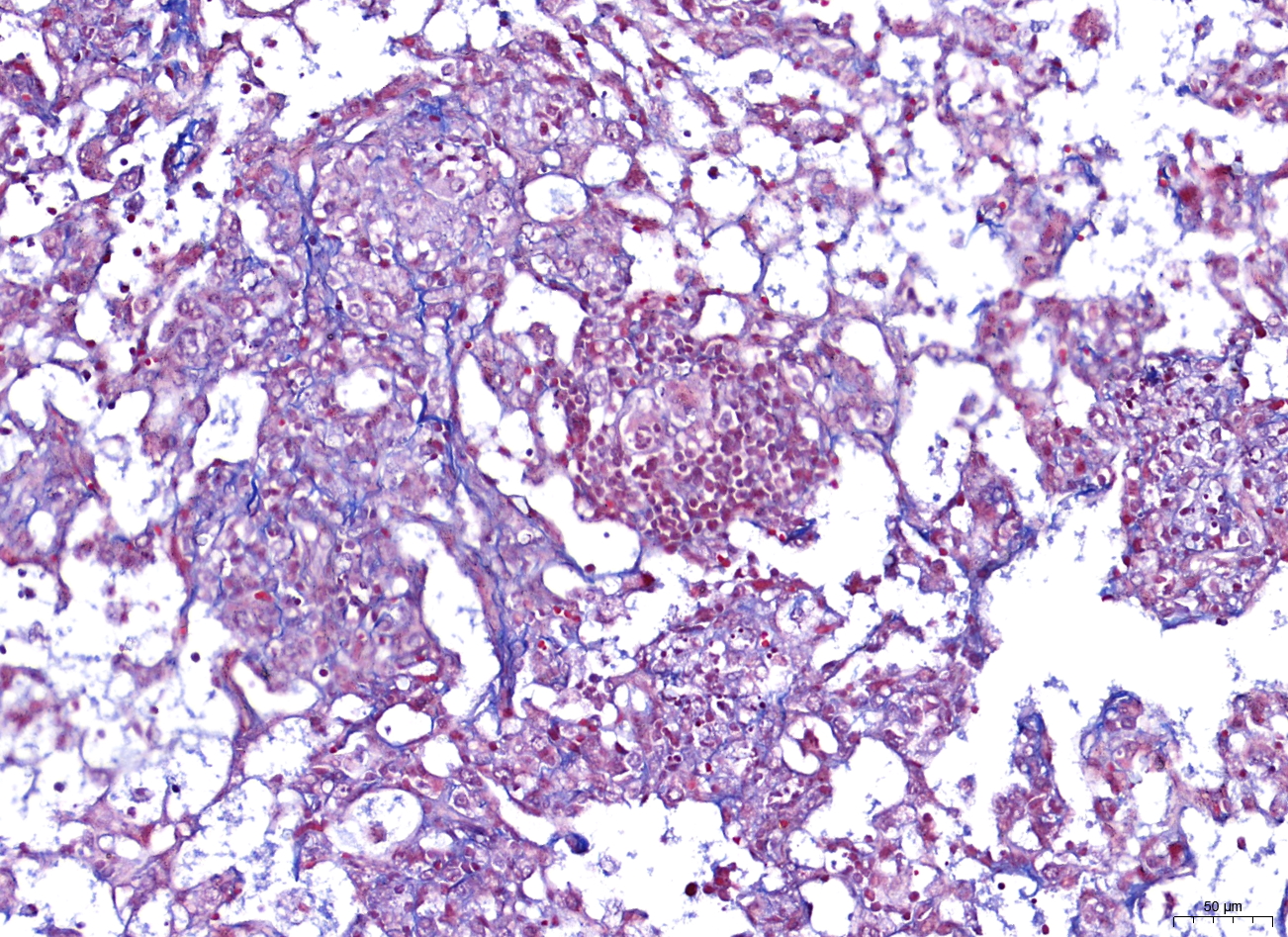

Supplement: Supplementary file 3 — Supporting File 3: advs73867‐sup‐0003‐SupportingFiguresData.zip. [file ADVS-13-e19191-s003.zip › Supporting information Figure S1-S9/S1/Figure S1B/8W-Model-763_20.0x.jpg]

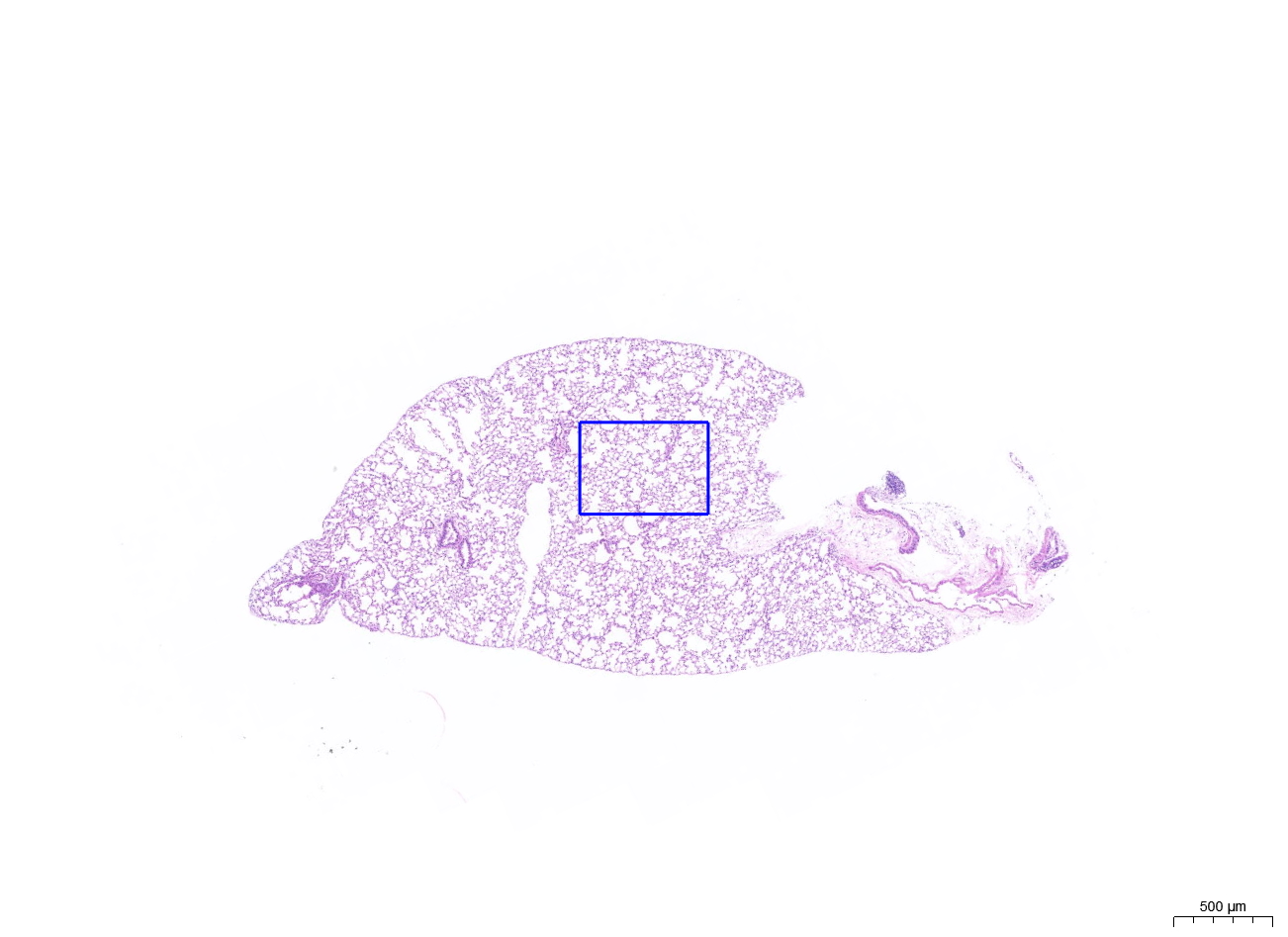

Supplement: Supplementary file 3 — Supporting File 3: advs73867‐sup‐0003‐SupportingFiguresData.zip. [file ADVS-13-e19191-s003.zip › Supporting information Figure S1-S9/S1/Figure S1B/SCRS-1 week-Control-792_2.0x.jpg]

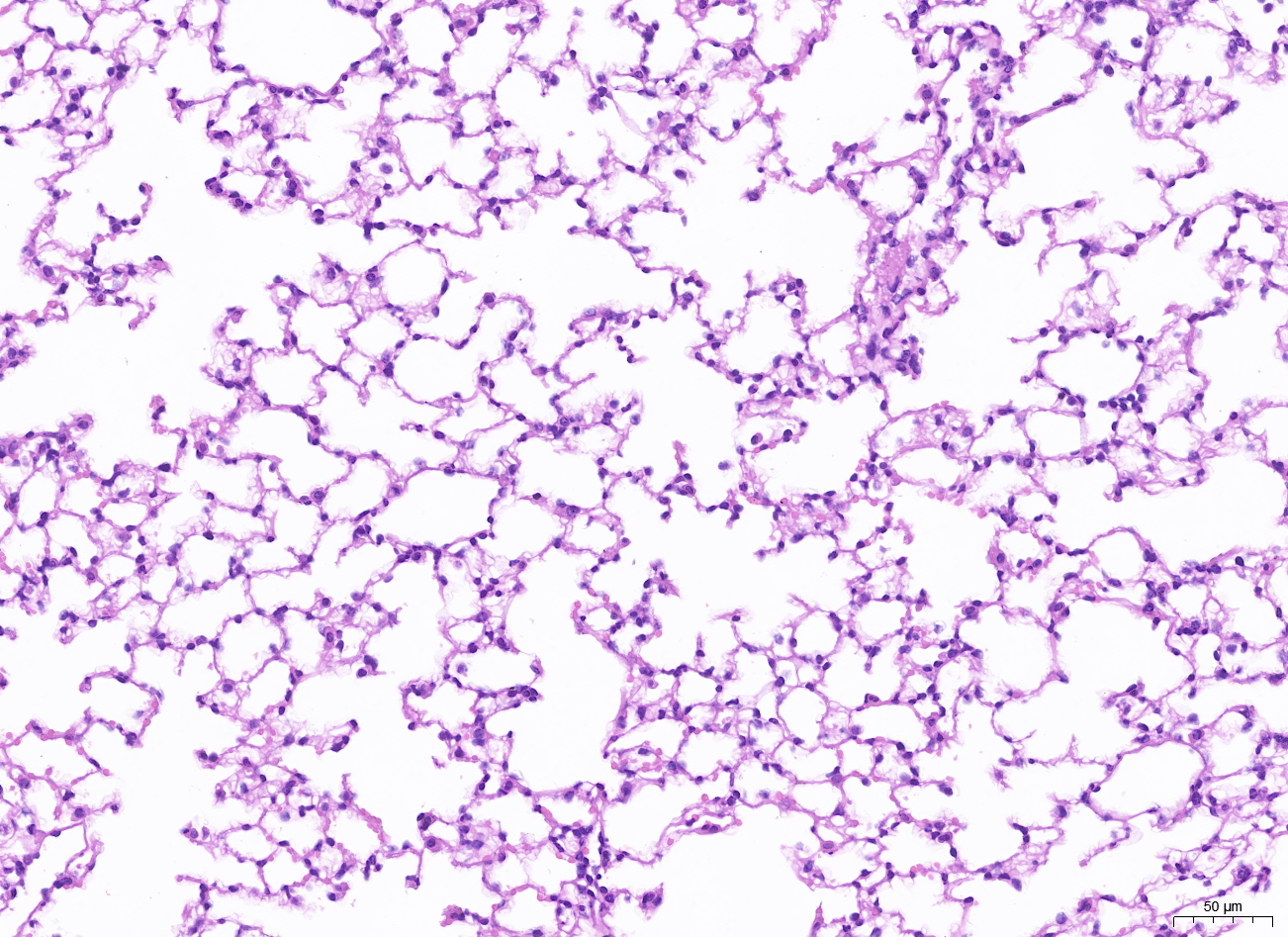

Supplement: Supplementary file 3 — Supporting File 3: advs73867‐sup‐0003‐SupportingFiguresData.zip. [file ADVS-13-e19191-s003.zip › Supporting information Figure S1-S9/S1/Figure S1B/SCRS-1 week-Control-792_20.0x.jpg]

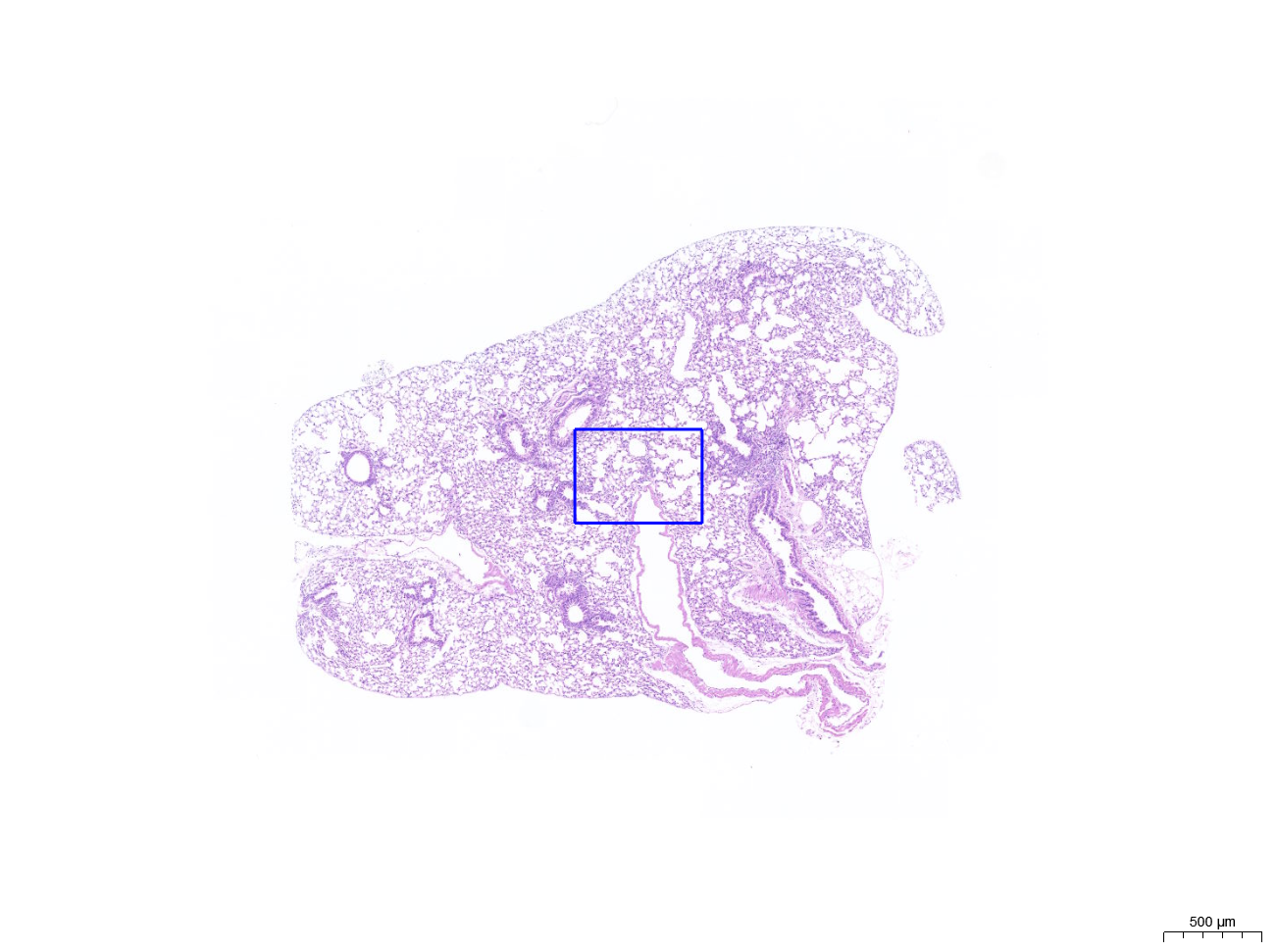

Supplement: Supplementary file 3 — Supporting File 3: advs73867‐sup‐0003‐SupportingFiguresData.zip. [file ADVS-13-e19191-s003.zip › Supporting information Figure S1-S9/S1/Figure S1B/SCRS-1 week-Model-792_2.0x.tif]

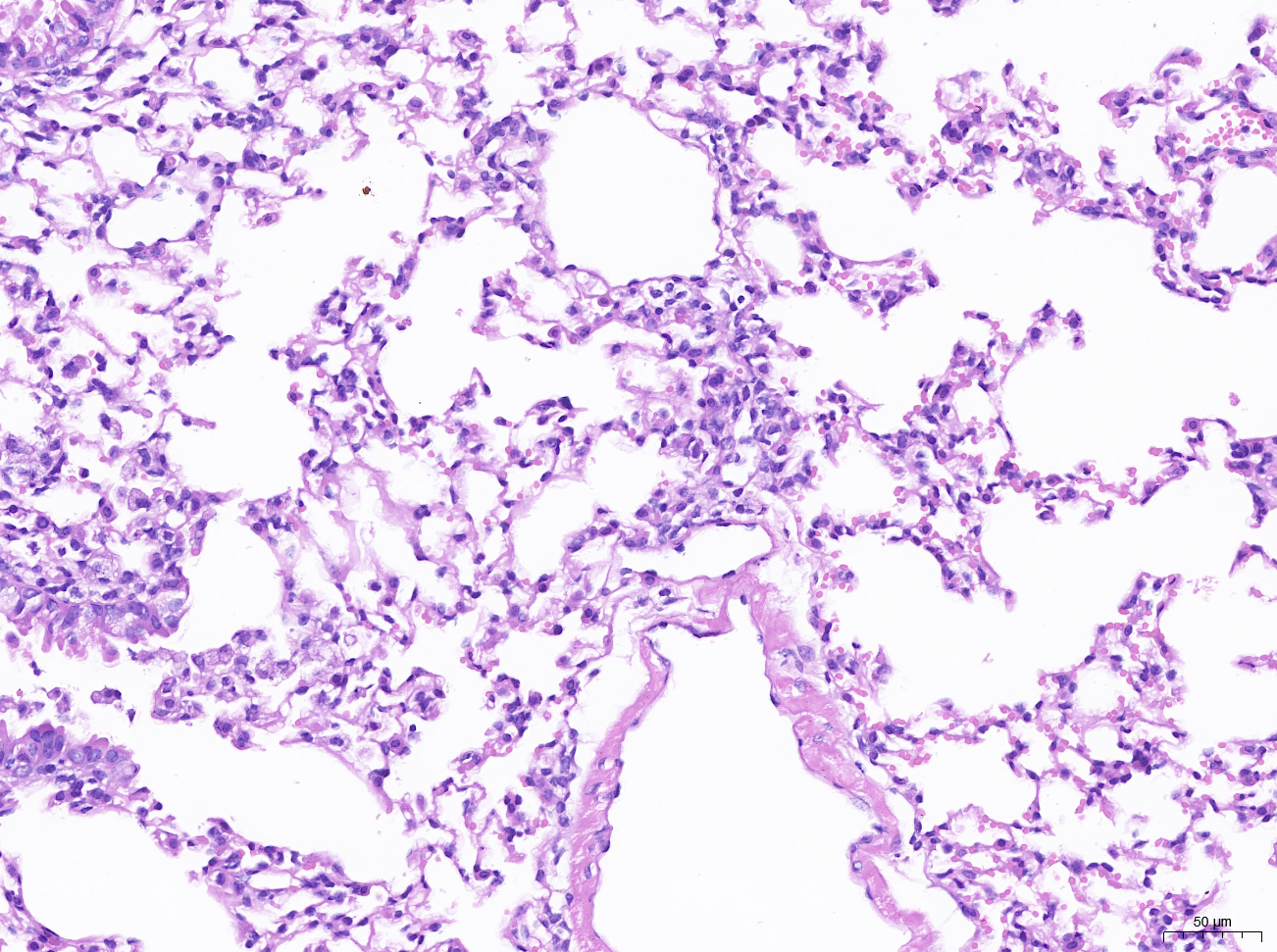

Supplement: Supplementary file 3 — Supporting File 3: advs73867‐sup‐0003‐SupportingFiguresData.zip. [file ADVS-13-e19191-s003.zip › Supporting information Figure S1-S9/S1/Figure S1B/SCRS-1 week-Model-792_20.0x.tif]

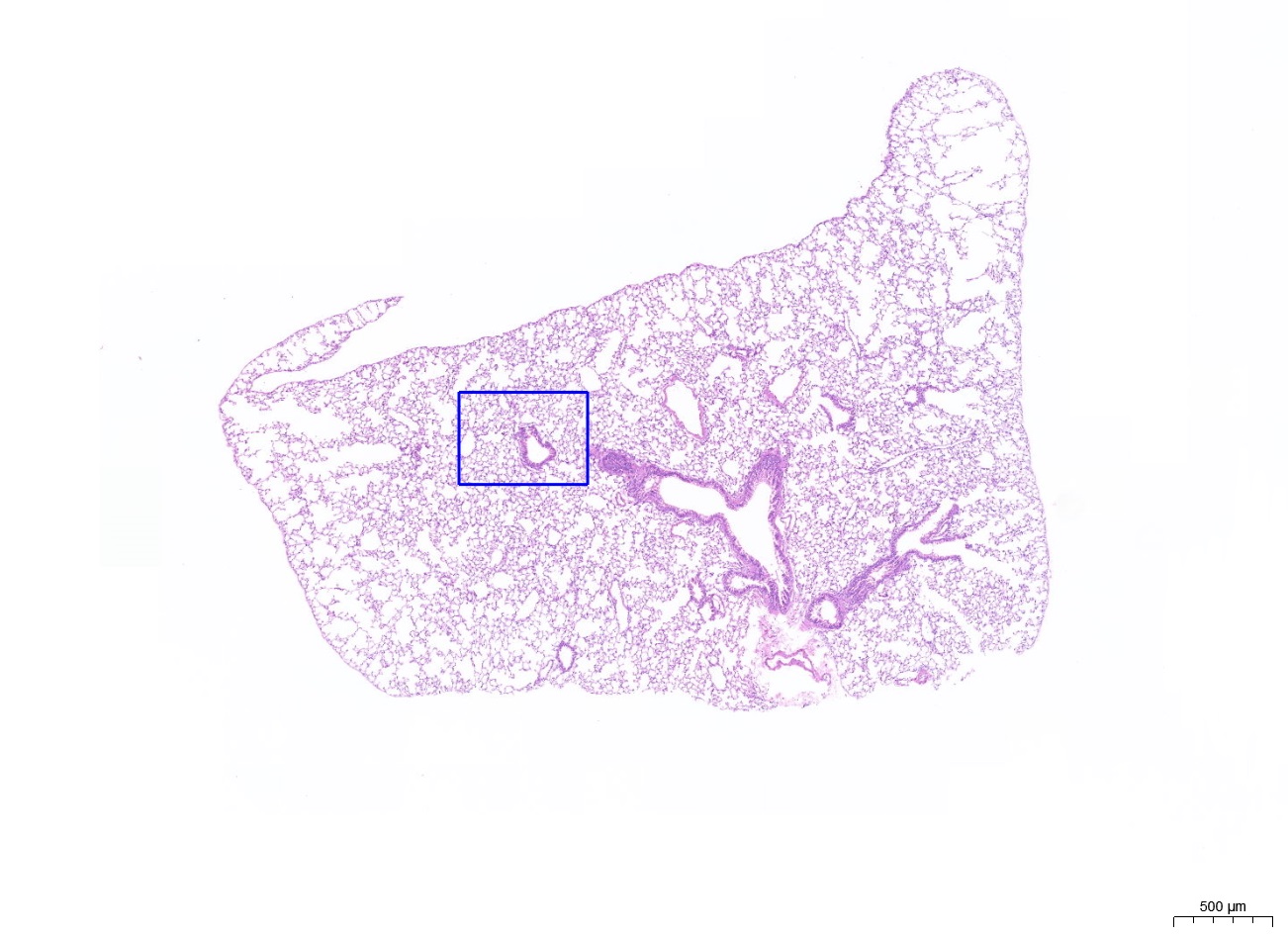

Supplement: Supplementary file 3 — Supporting File 3: advs73867‐sup‐0003‐SupportingFiguresData.zip. [file ADVS-13-e19191-s003.zip › Supporting information Figure S1-S9/S1/Figure S1B/SCRS-12 week-Control-781_2.0x.jpg]

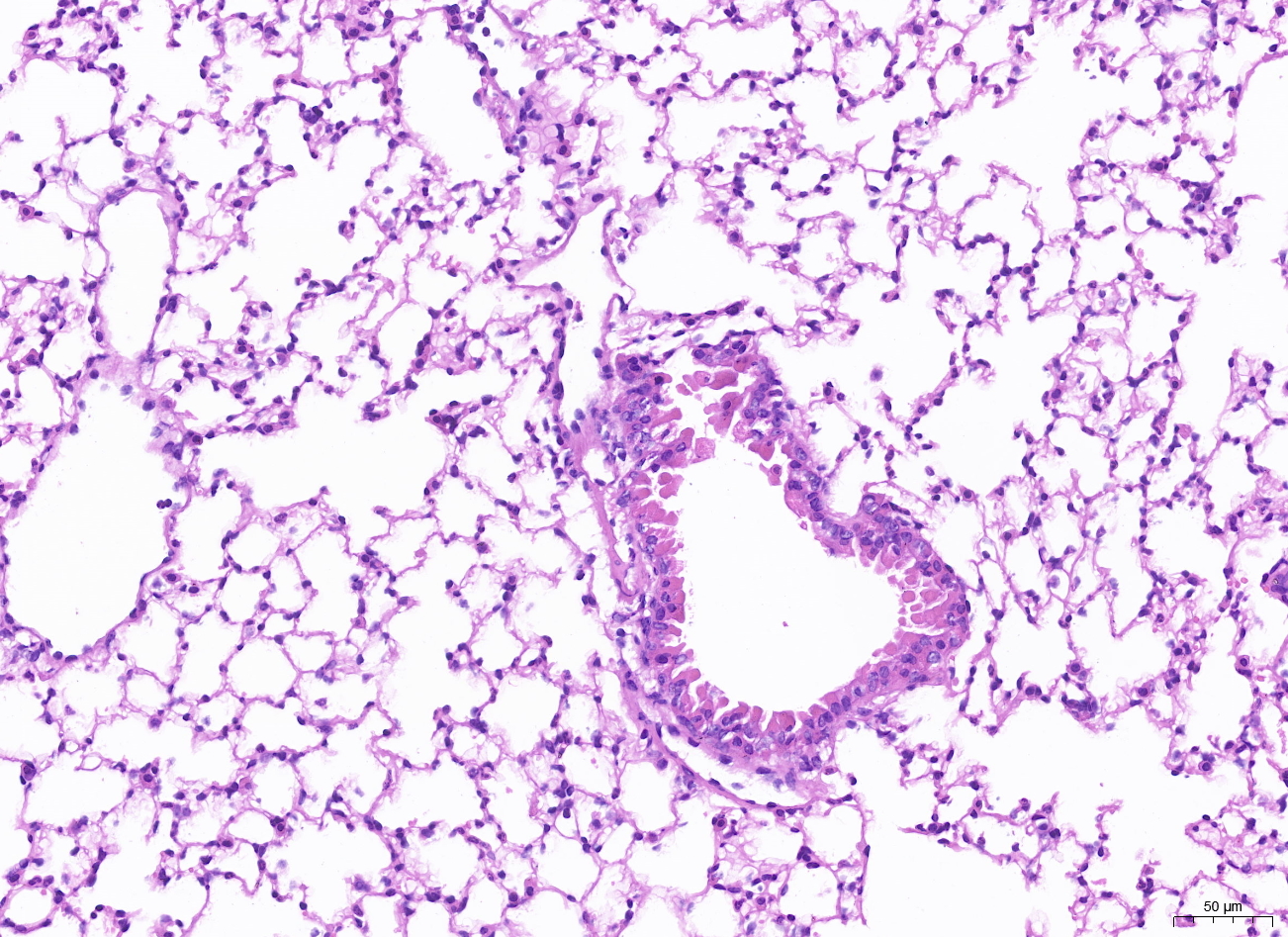

Supplement: Supplementary file 3 — Supporting File 3: advs73867‐sup‐0003‐SupportingFiguresData.zip. [file ADVS-13-e19191-s003.zip › Supporting information Figure S1-S9/S1/Figure S1B/SCRS-12 week-Control-781_20.0x.jpg]

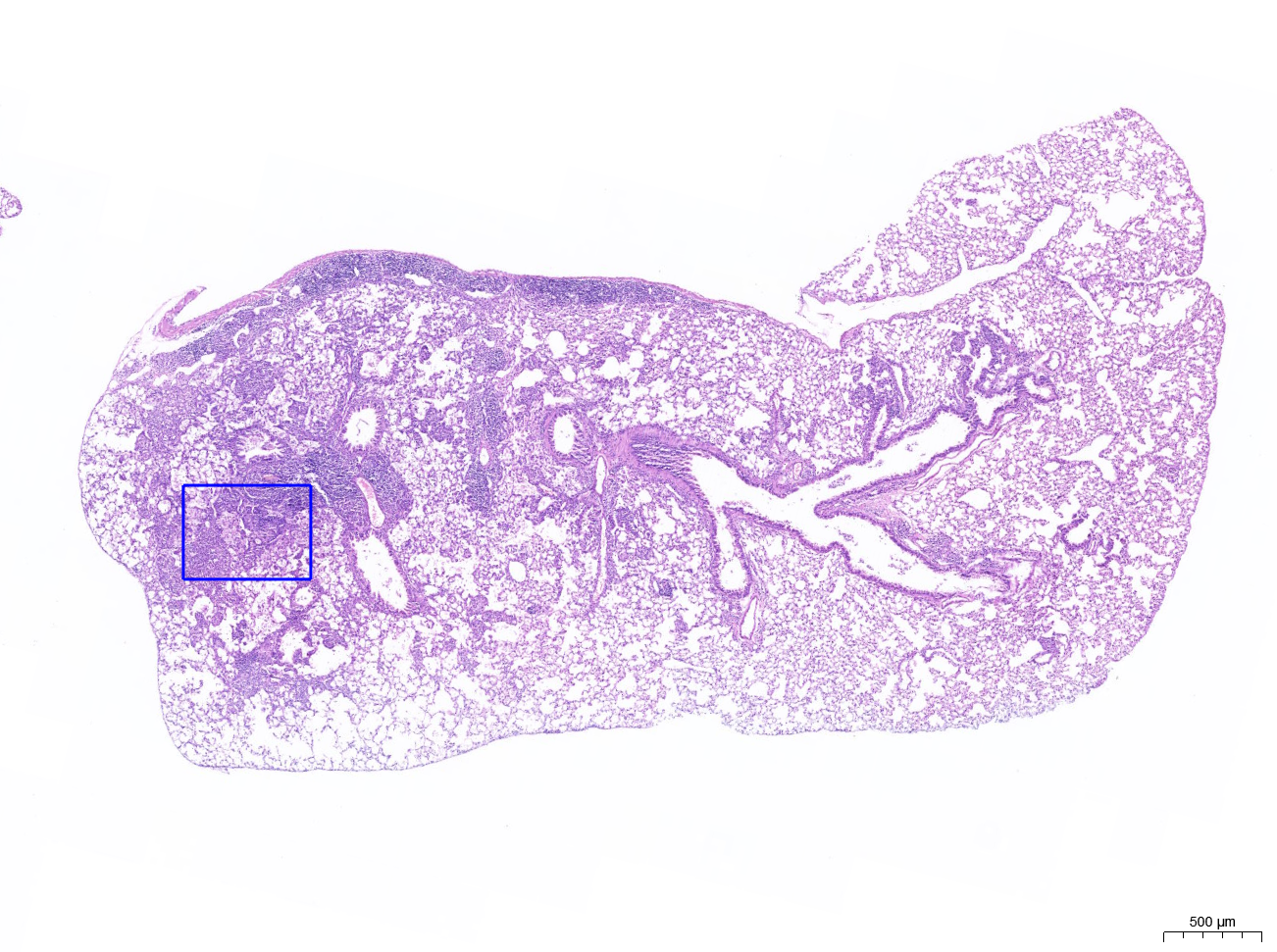

Supplement: Supplementary file 3 — Supporting File 3: advs73867‐sup‐0003‐SupportingFiguresData.zip. [file ADVS-13-e19191-s003.zip › Supporting information Figure S1-S9/S1/Figure S1B/SCRS-12 week-Model-767_2.0x.tif]

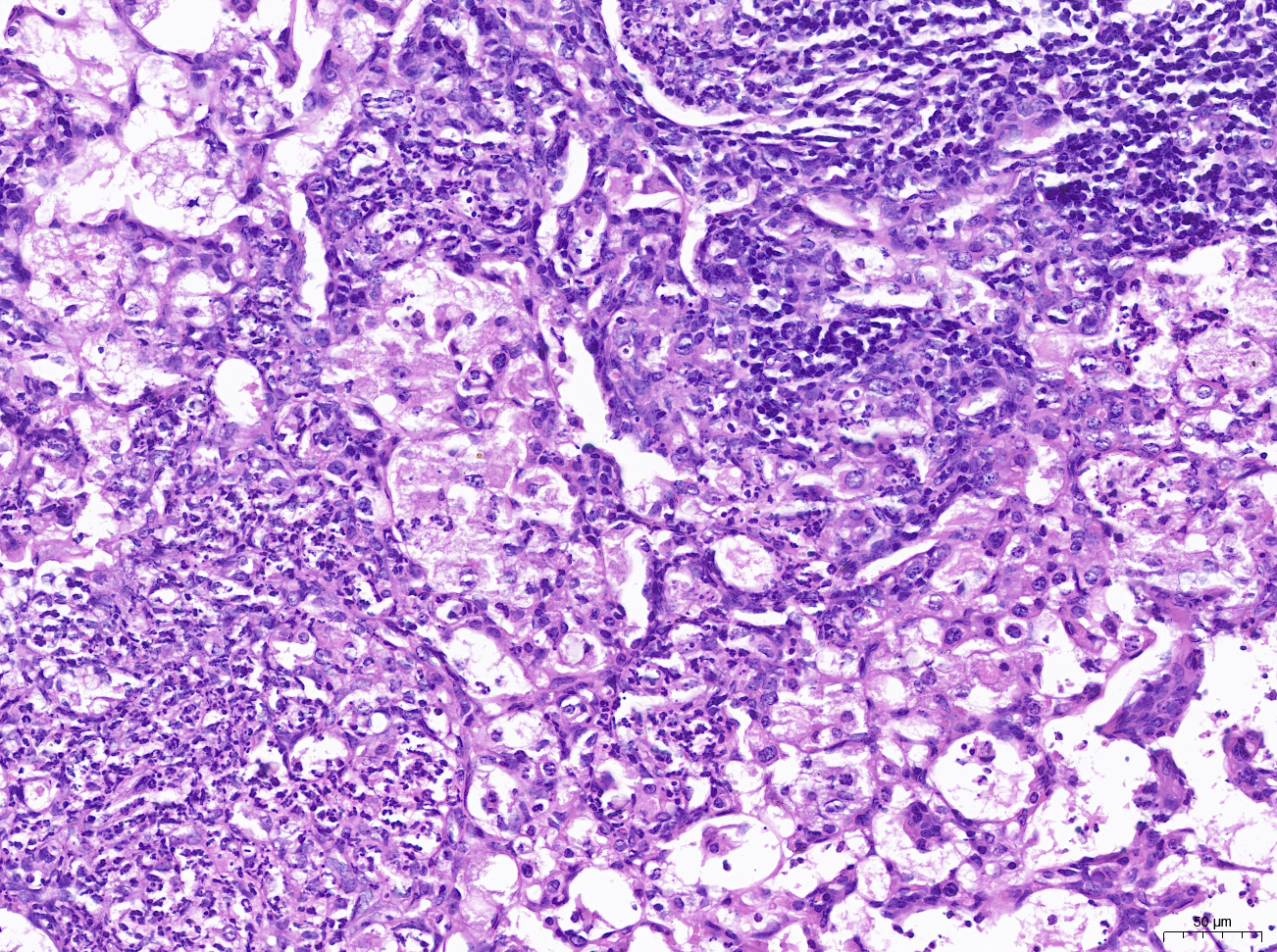

Supplement: Supplementary file 3 — Supporting File 3: advs73867‐sup‐0003‐SupportingFiguresData.zip. [file ADVS-13-e19191-s003.zip › Supporting information Figure S1-S9/S1/Figure S1B/SCRS-12 week-Model-767_20.0x.tif]

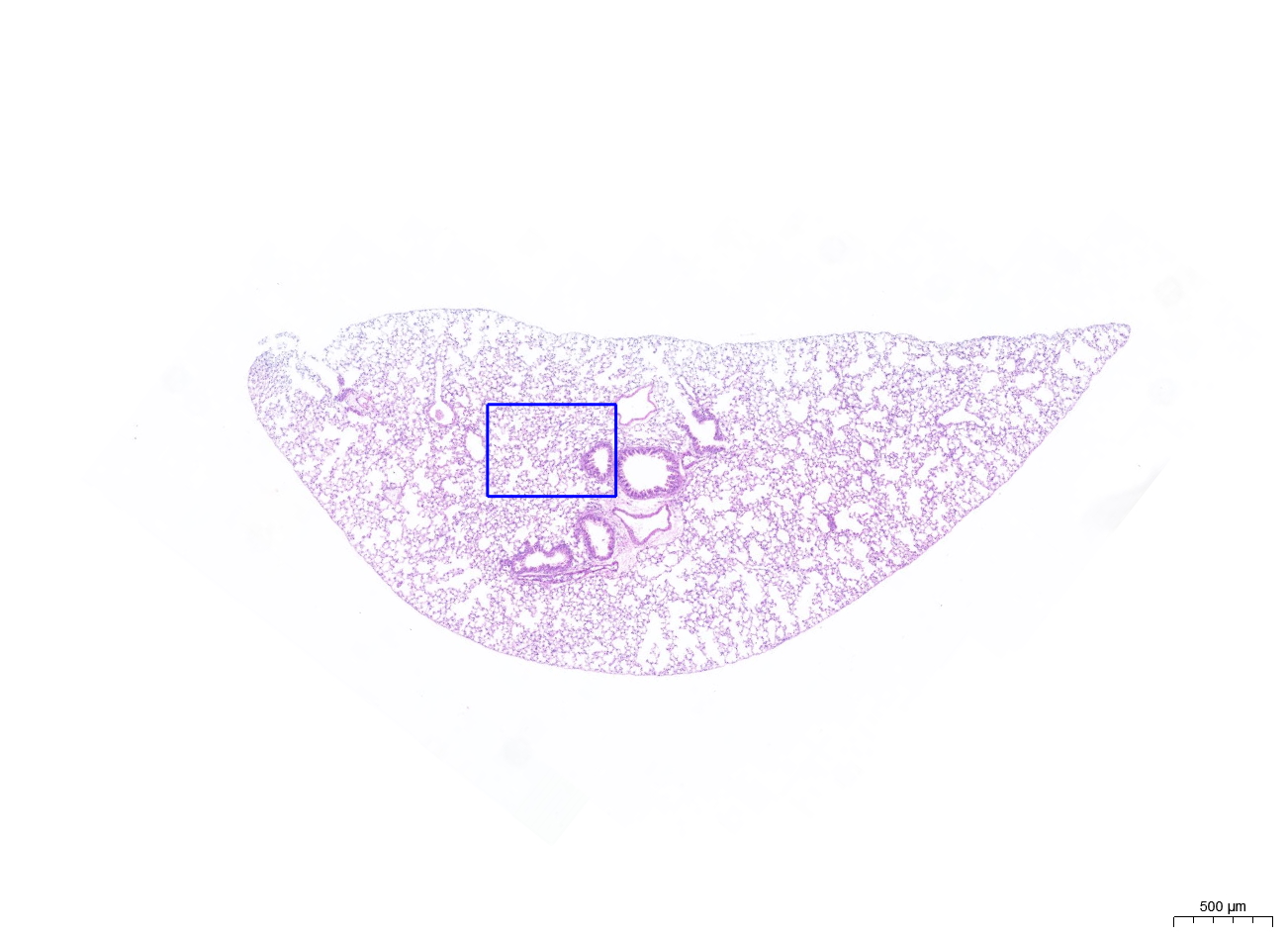

Supplement: Supplementary file 3 — Supporting File 3: advs73867‐sup‐0003‐SupportingFiguresData.zip. [file ADVS-13-e19191-s003.zip › Supporting information Figure S1-S9/S1/Figure S1B/SCRS-4 week-Control-774_2.0x.jpg]

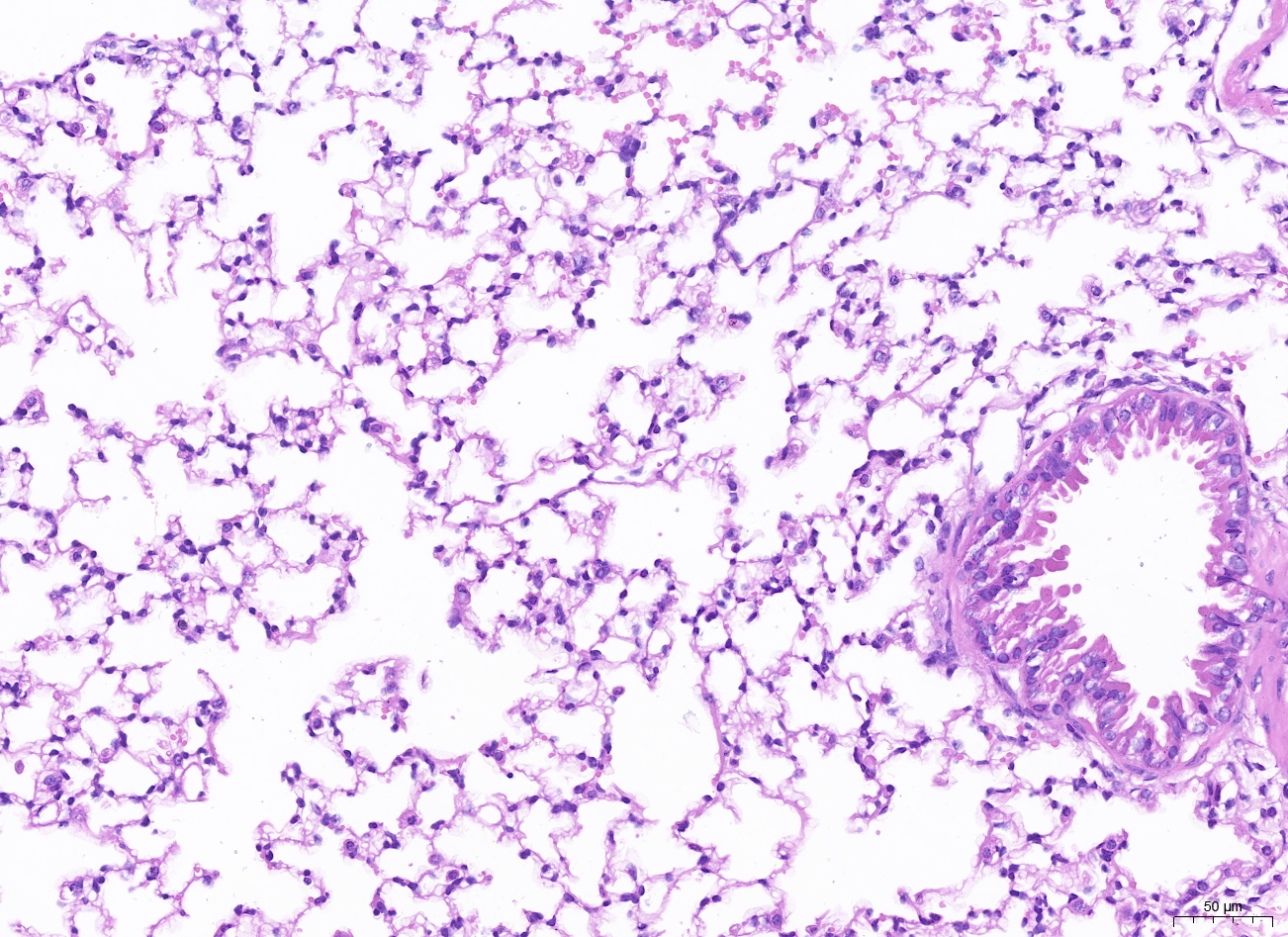

Supplement: Supplementary file 3 — Supporting File 3: advs73867‐sup‐0003‐SupportingFiguresData.zip. [file ADVS-13-e19191-s003.zip › Supporting information Figure S1-S9/S1/Figure S1B/SCRS-4 week-Control-774_20.0x.jpg]

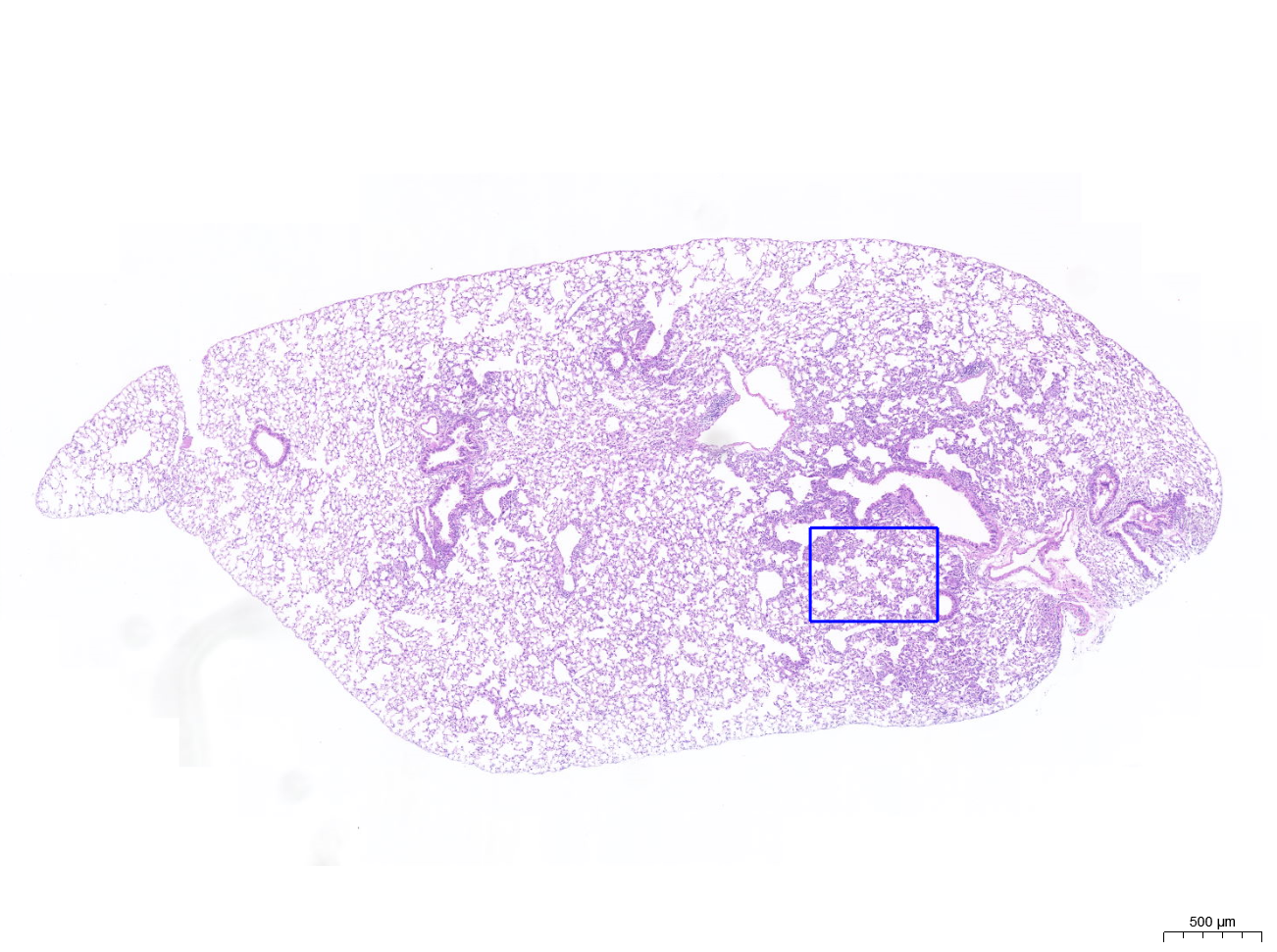

Supplement: Supplementary file 3 — Supporting File 3: advs73867‐sup‐0003‐SupportingFiguresData.zip. [file ADVS-13-e19191-s003.zip › Supporting information Figure S1-S9/S1/Figure S1B/SCRS-4 week-Model-760_2.0x.tif]

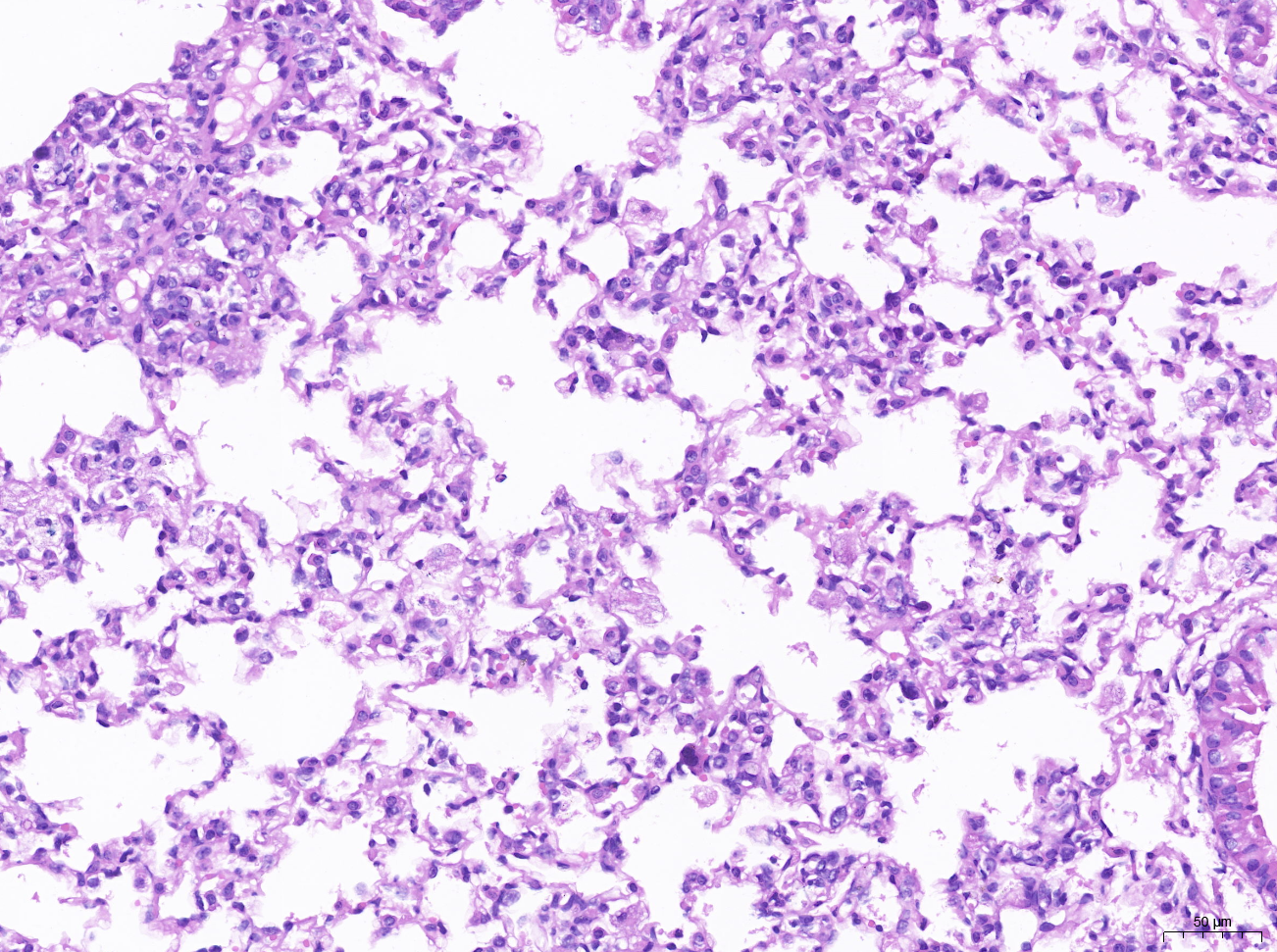

Supplement: Supplementary file 3 — Supporting File 3: advs73867‐sup‐0003‐SupportingFiguresData.zip. [file ADVS-13-e19191-s003.zip › Supporting information Figure S1-S9/S1/Figure S1B/SCRS-4 week-Model-760_20.0x.tif]

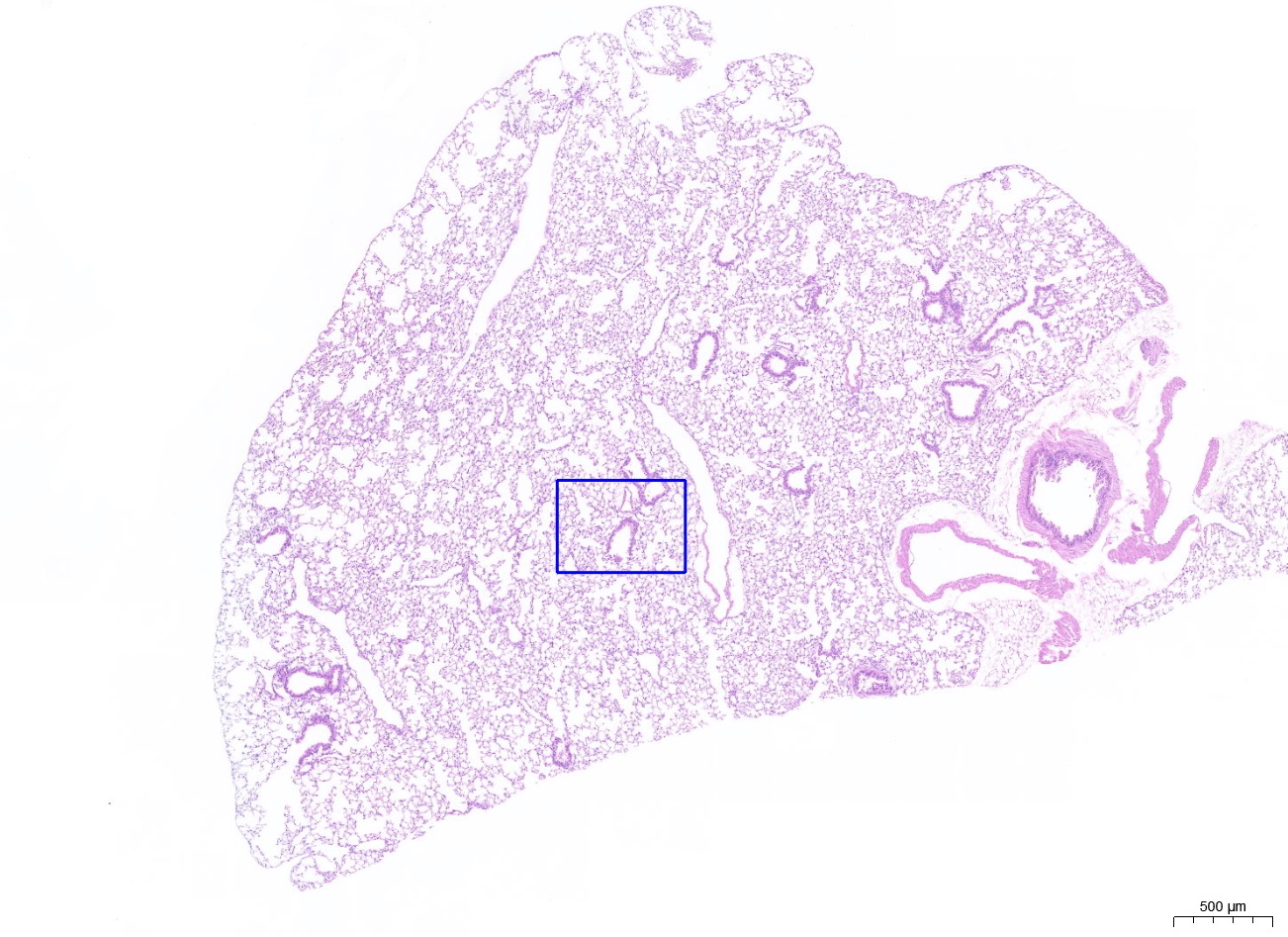

Supplement: Supplementary file 3 — Supporting File 3: advs73867‐sup‐0003‐SupportingFiguresData.zip. [file ADVS-13-e19191-s003.zip › Supporting information Figure S1-S9/S1/Figure S1B/SCRS-8 week-Control-790_2.0x.jpg]

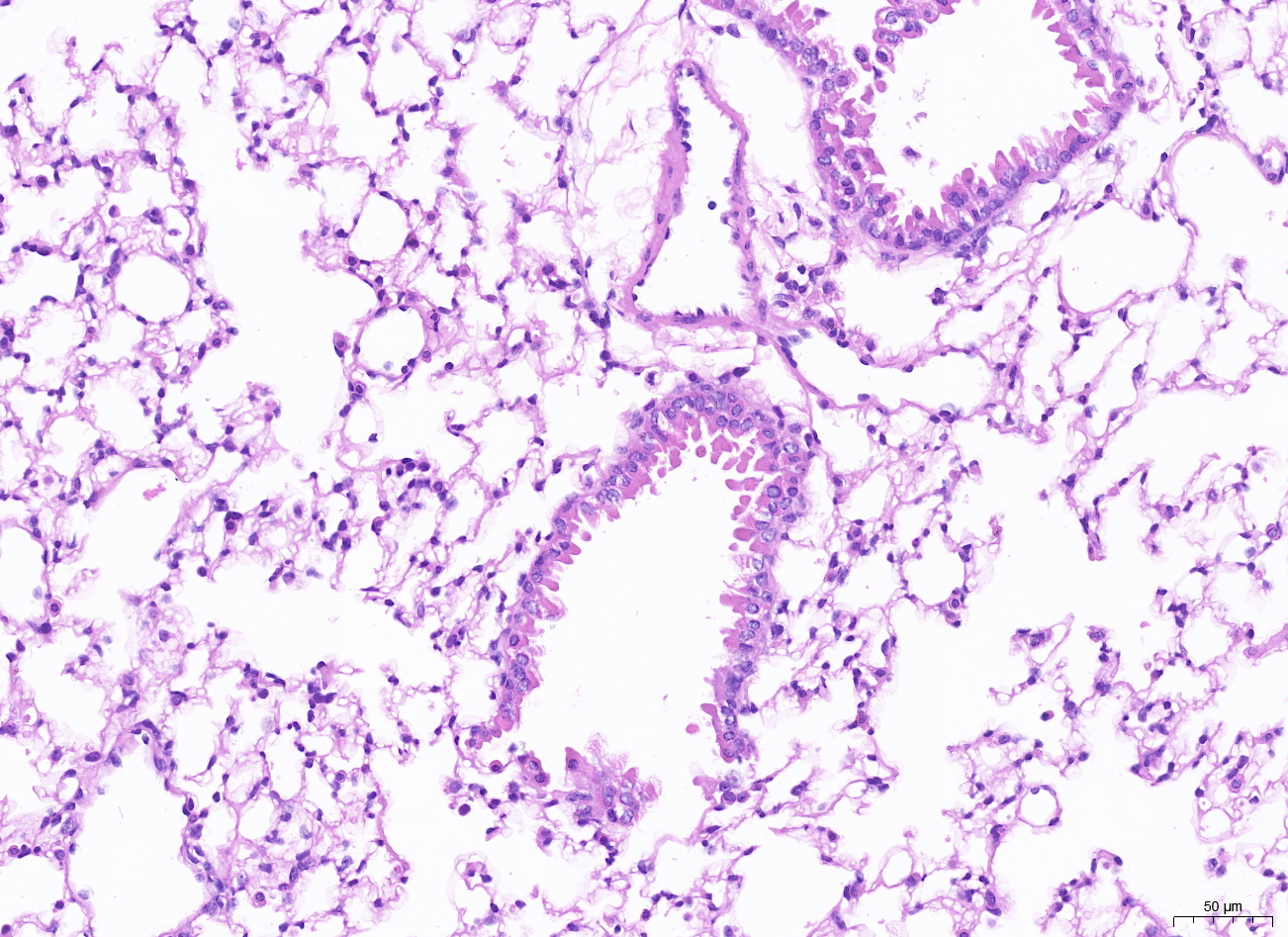

Supplement: Supplementary file 3 — Supporting File 3: advs73867‐sup‐0003‐SupportingFiguresData.zip. [file ADVS-13-e19191-s003.zip › Supporting information Figure S1-S9/S1/Figure S1B/SCRS-8 week-Control-790_20.0x.jpg]

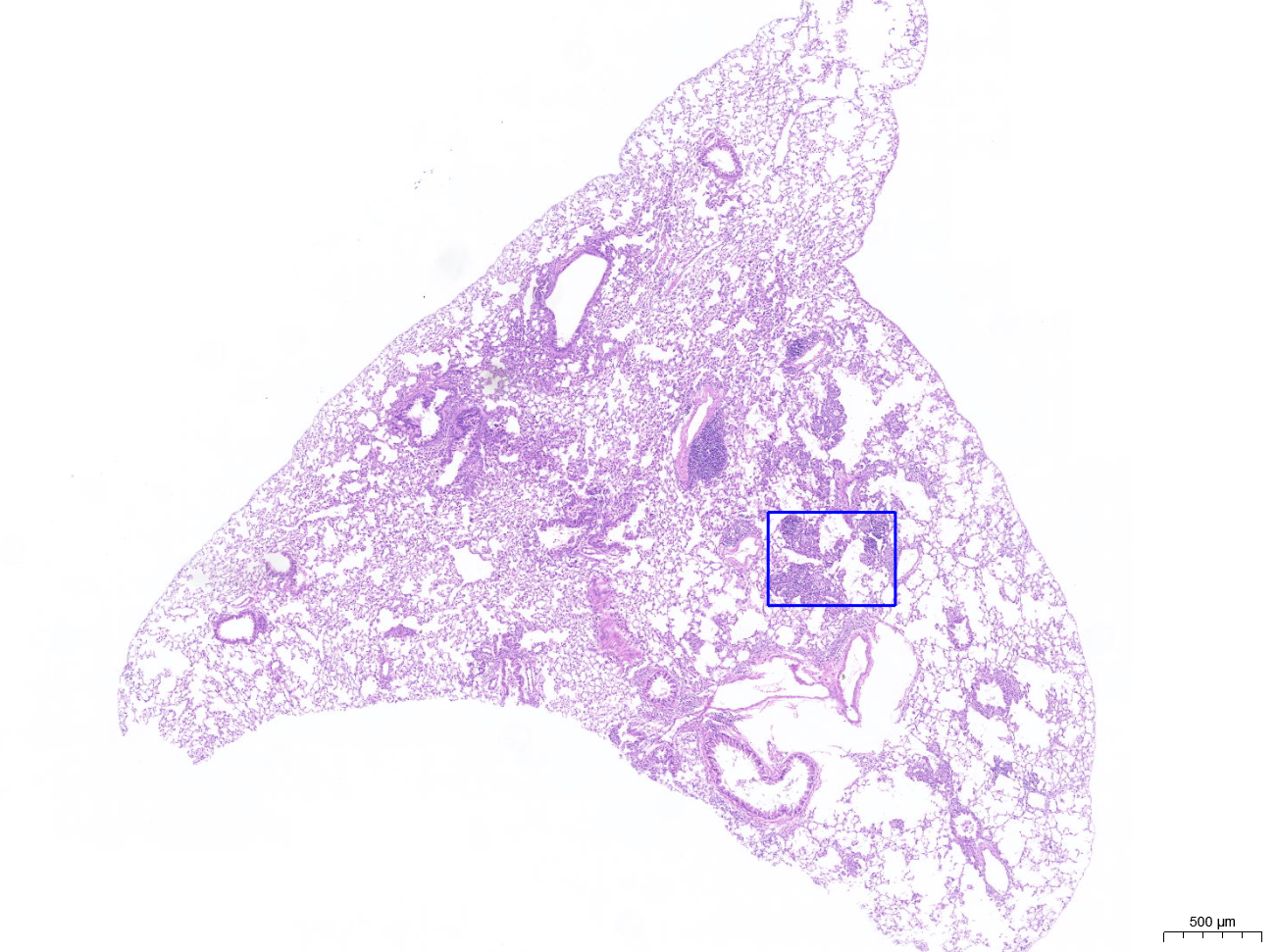

Supplement: Supplementary file 3 — Supporting File 3: advs73867‐sup‐0003‐SupportingFiguresData.zip. [file ADVS-13-e19191-s003.zip › Supporting information Figure S1-S9/S1/Figure S1B/SCRS-8 week-Model-750_2.0x.tif]

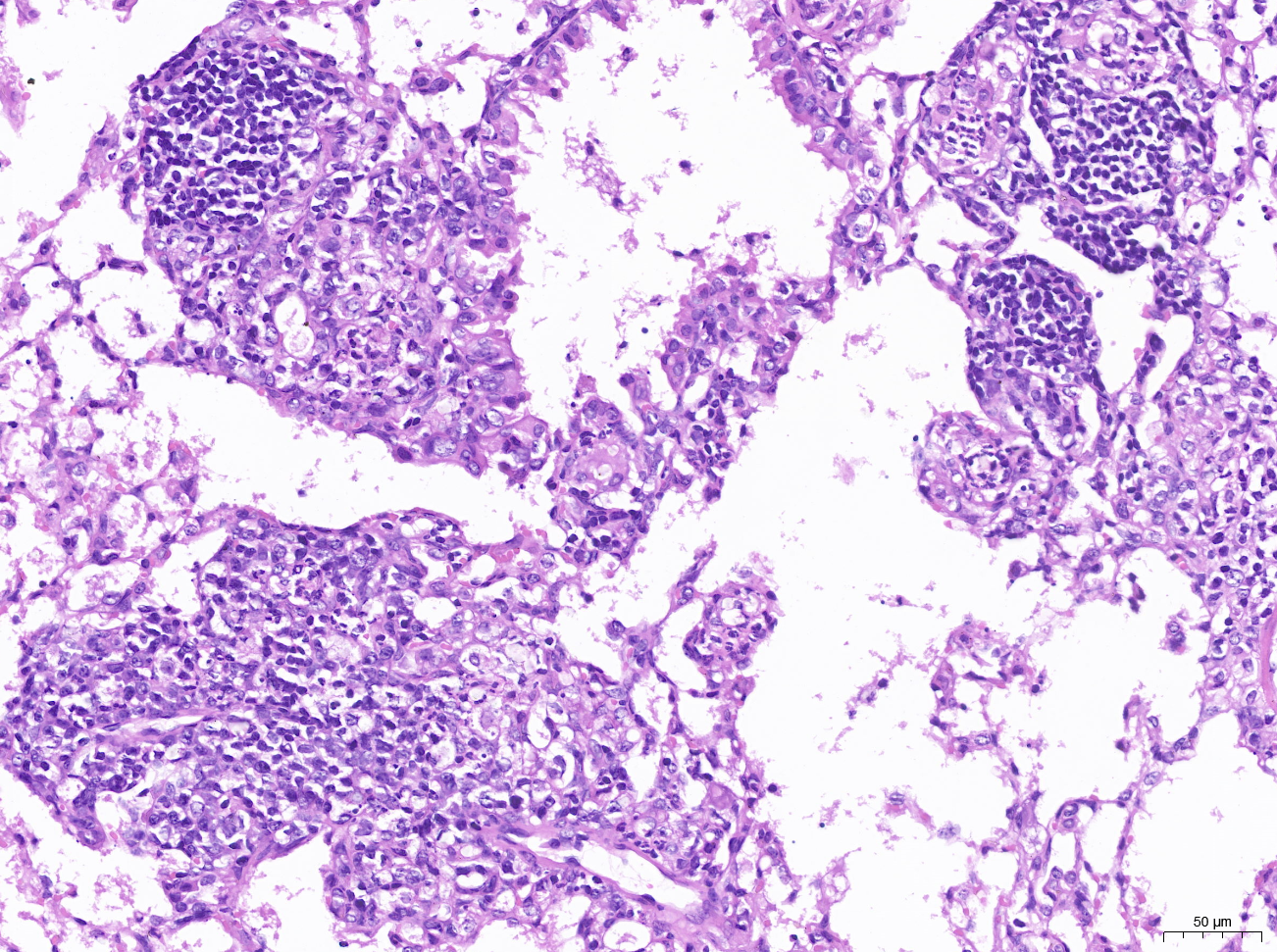

Supplement: Supplementary file 3 — Supporting File 3: advs73867‐sup‐0003‐SupportingFiguresData.zip. [file ADVS-13-e19191-s003.zip › Supporting information Figure S1-S9/S1/Figure S1B/SCRS-8 week-Model-750_20.0x.tif]

Figure S1C

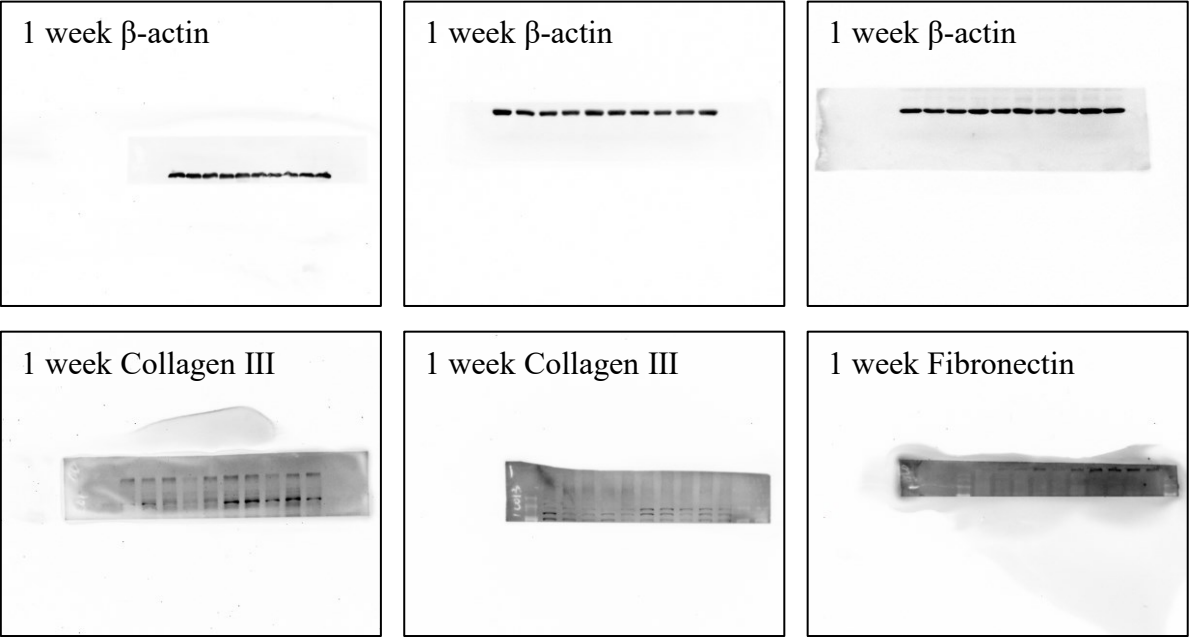

Figure S1D

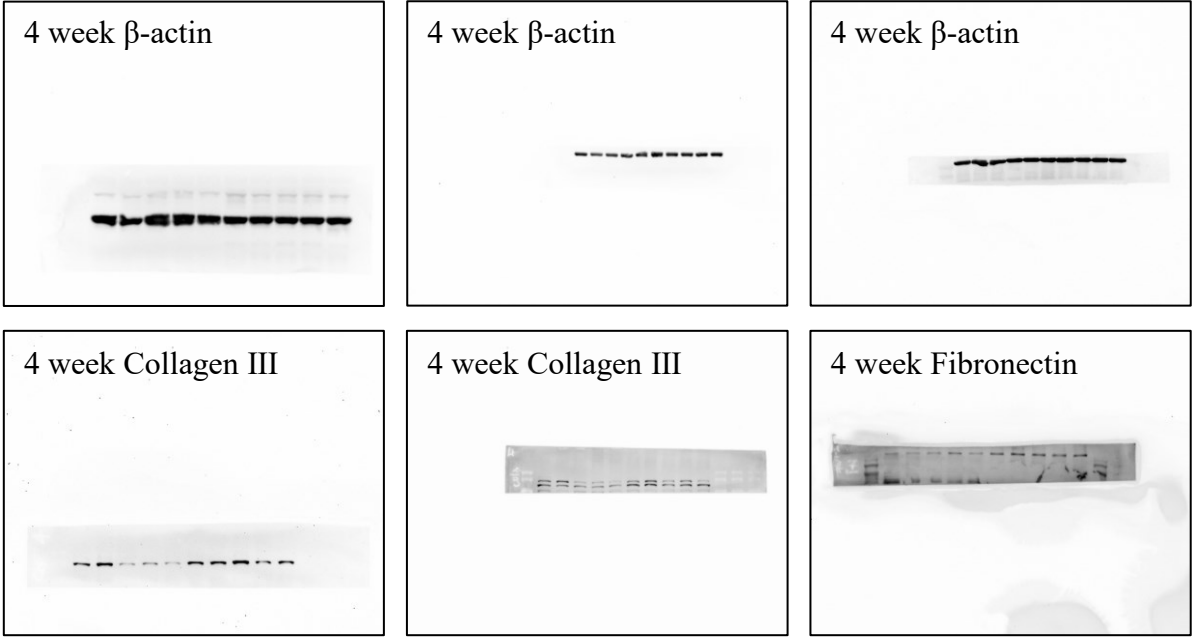

Figure S1E

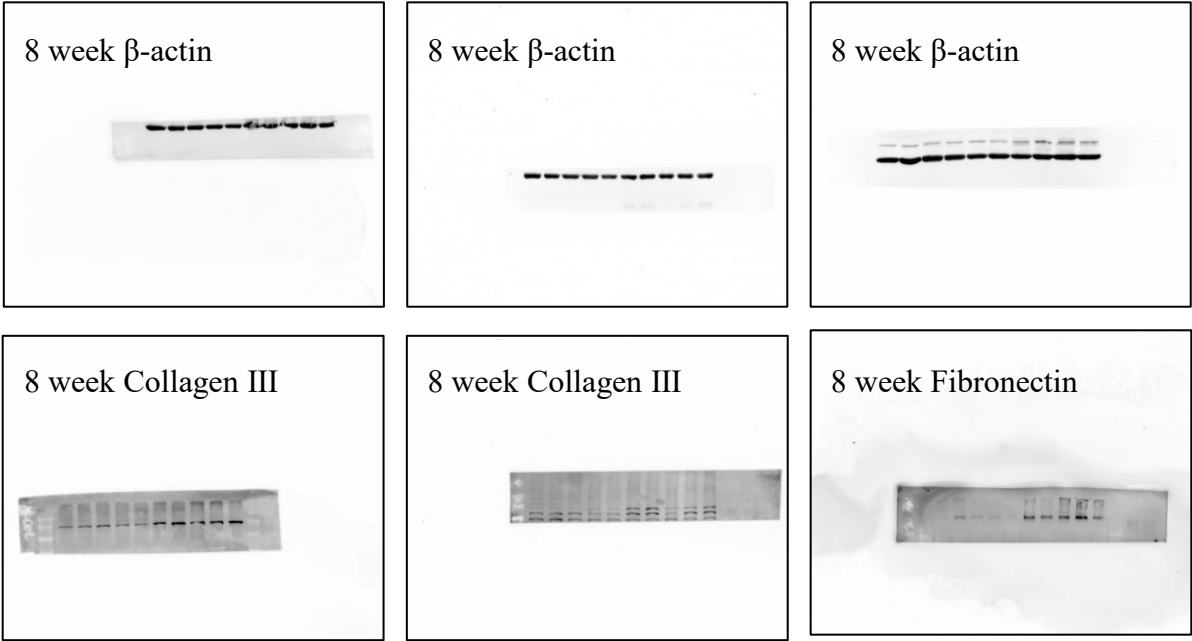

Figure S1F

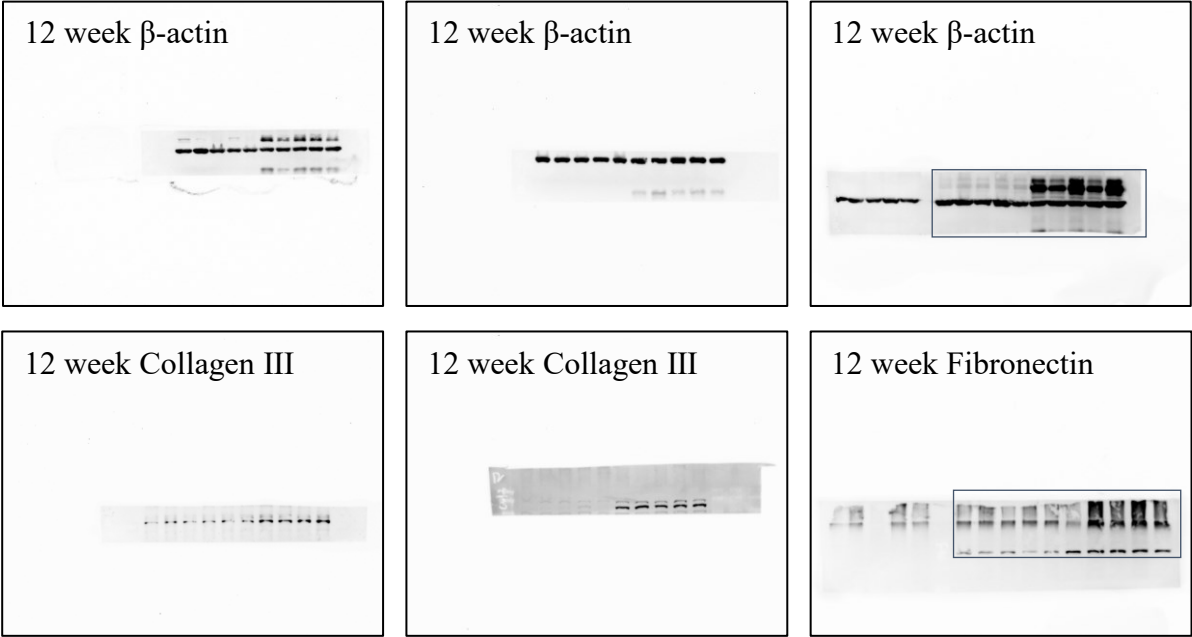

Supplement: Supplementary file 3 — Supporting File 3: advs73867‐sup‐0003‐SupportingFiguresData.zip. [file ADVS-13-e19191-s003.zip › Supporting information Figure S1-S9/S1/Figure S1C-F.pdf]

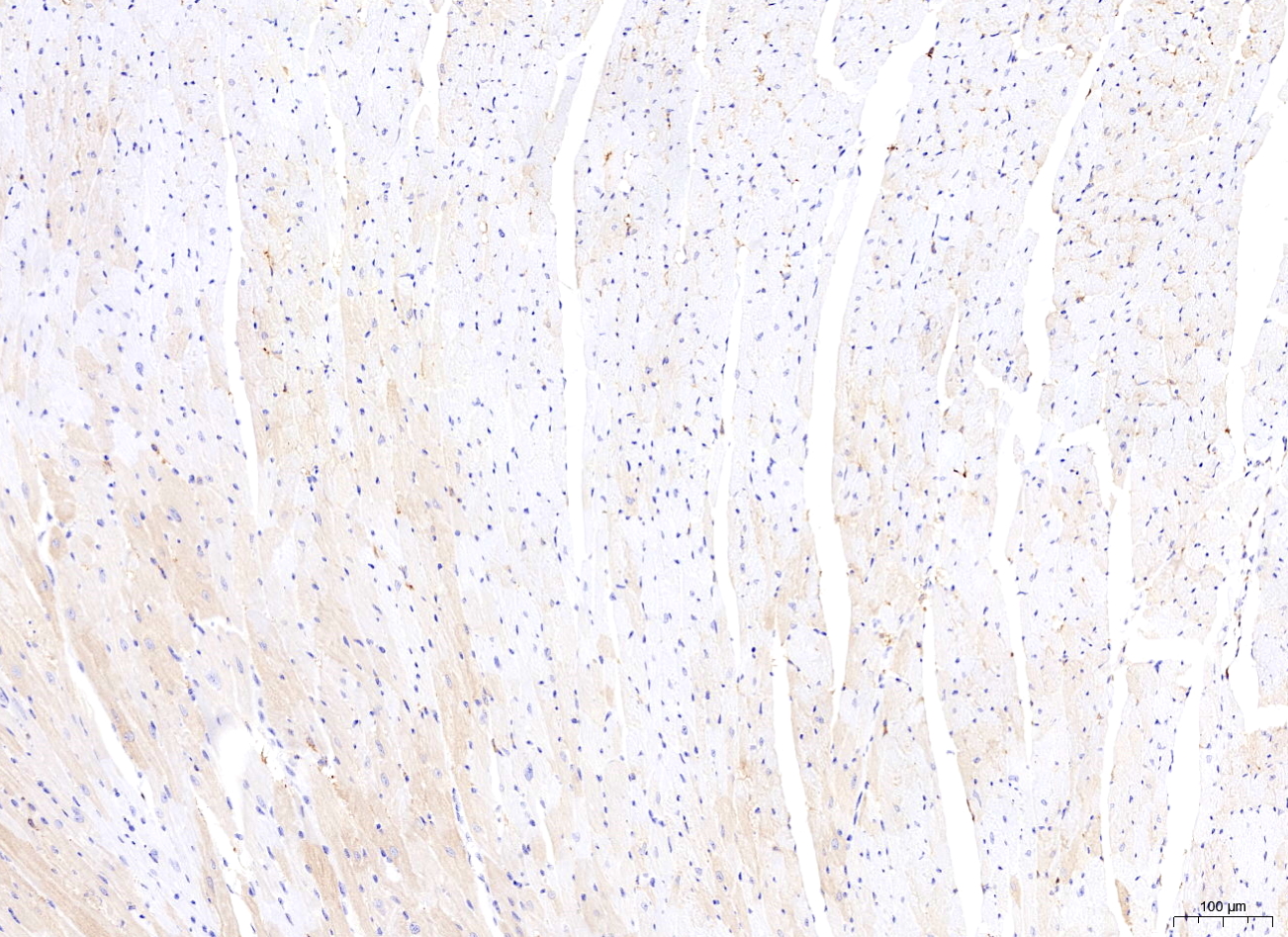

Supplement: Supplementary file 3 — Supporting File 3: advs73867‐sup‐0003‐SupportingFiguresData.zip. [file ADVS-13-e19191-s003.zip › Supporting information Figure S1-S9/S2/Heart/SCRS 12week Model 784_10.0x.jpg]

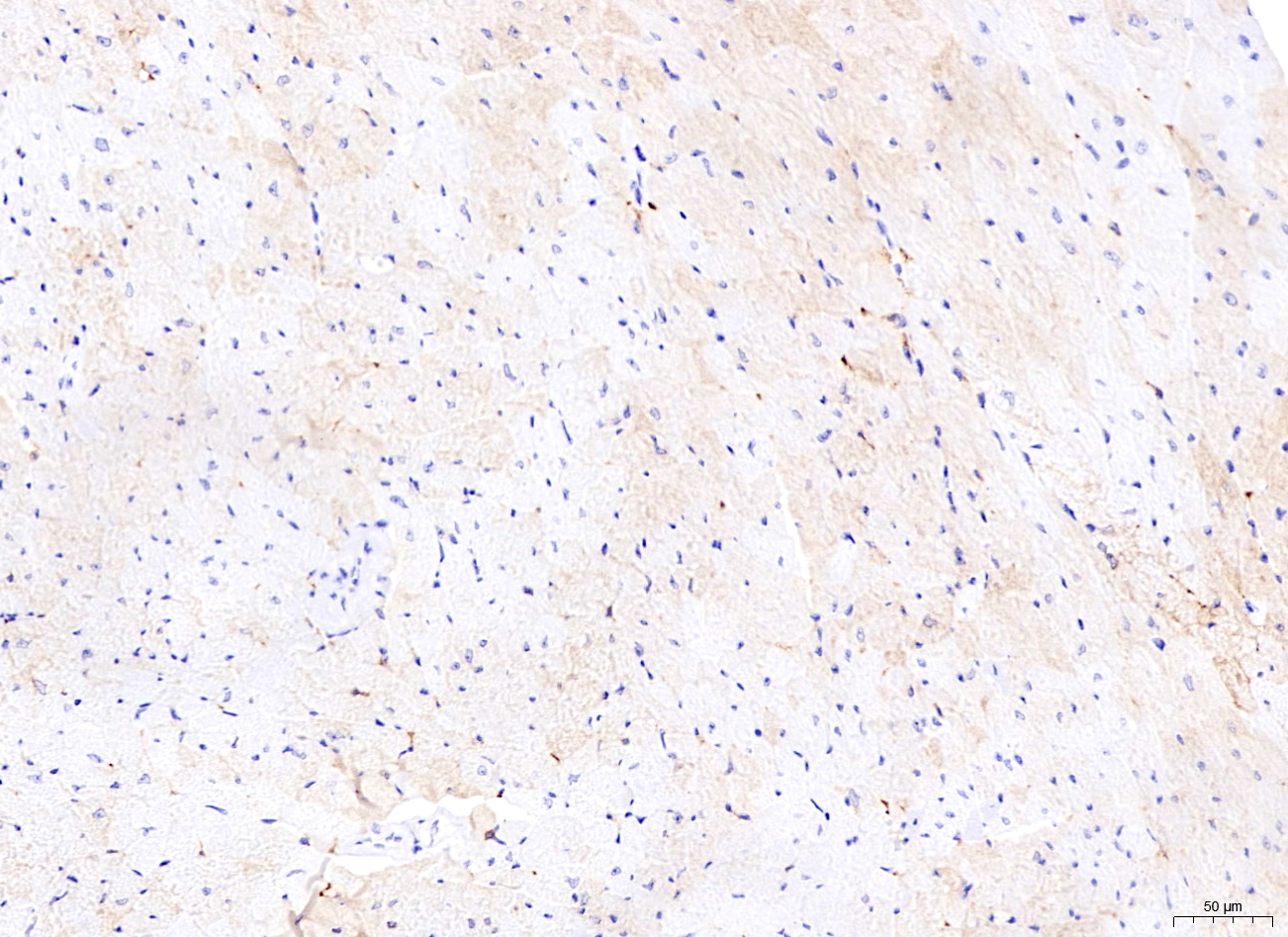

Supplement: Supplementary file 3 — Supporting File 3: advs73867‐sup‐0003‐SupportingFiguresData.zip. [file ADVS-13-e19191-s003.zip › Supporting information Figure S1-S9/S2/Heart/SCRS 12week Model 784_20.0x-1.jpg]

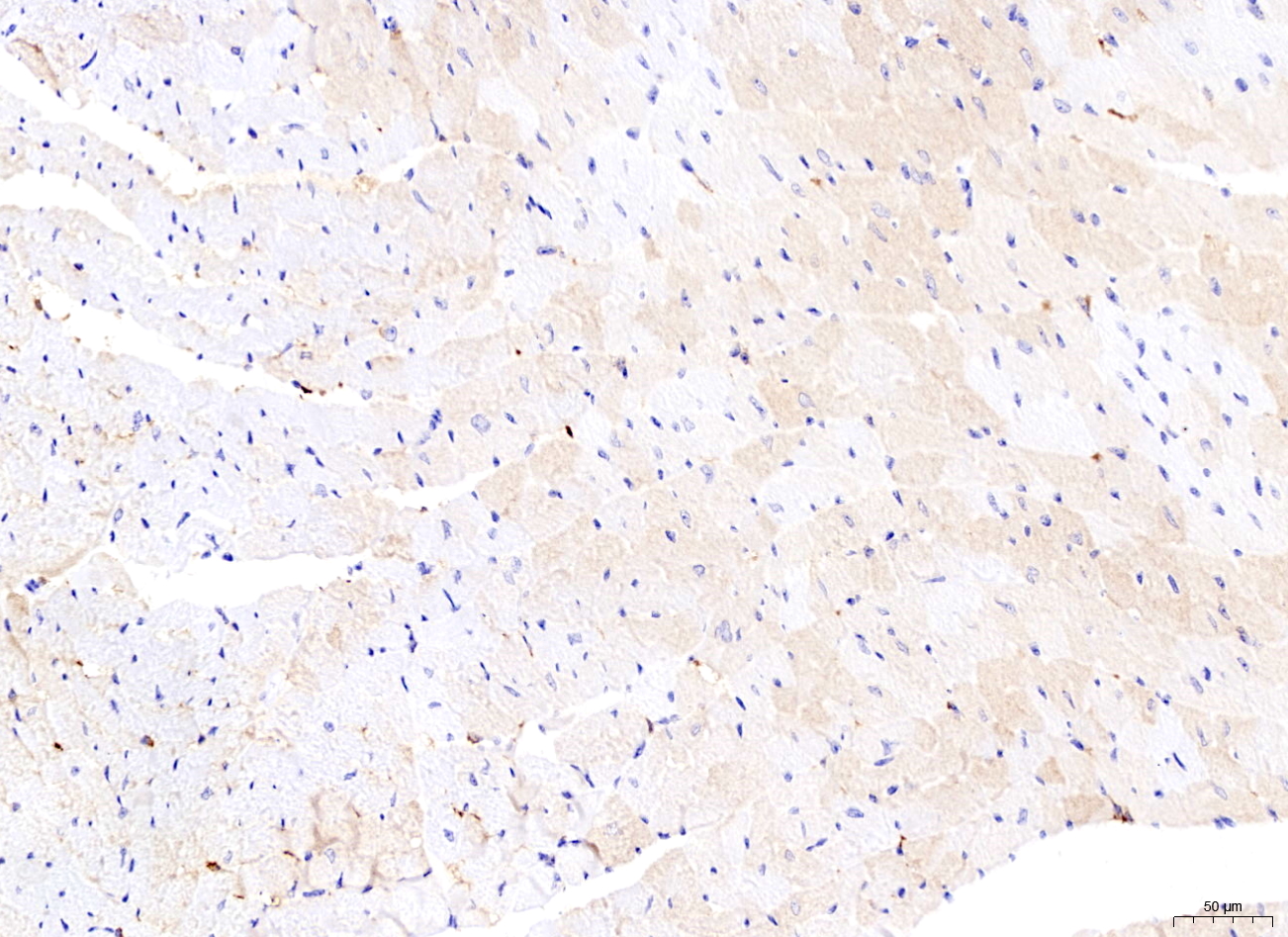

Supplement: Supplementary file 3 — Supporting File 3: advs73867‐sup‐0003‐SupportingFiguresData.zip. [file ADVS-13-e19191-s003.zip › Supporting information Figure S1-S9/S2/Heart/SCRS 12week Model 784_20.0x-2.jpg]

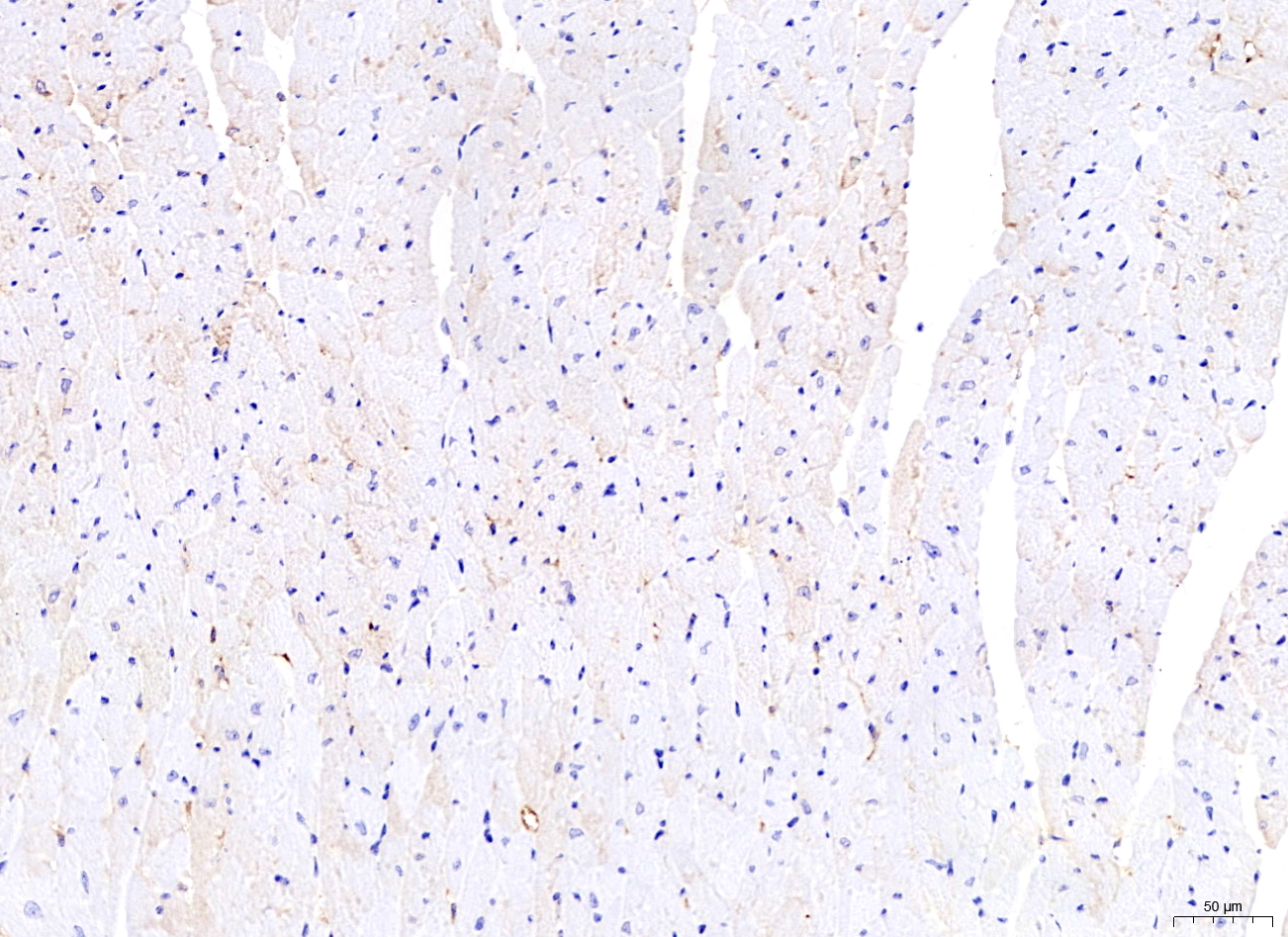

Supplement: Supplementary file 3 — Supporting File 3: advs73867‐sup‐0003‐SupportingFiguresData.zip. [file ADVS-13-e19191-s003.zip › Supporting information Figure S1-S9/S2/Heart/SCRS 12week Model 784_20.0x-3.jpg]

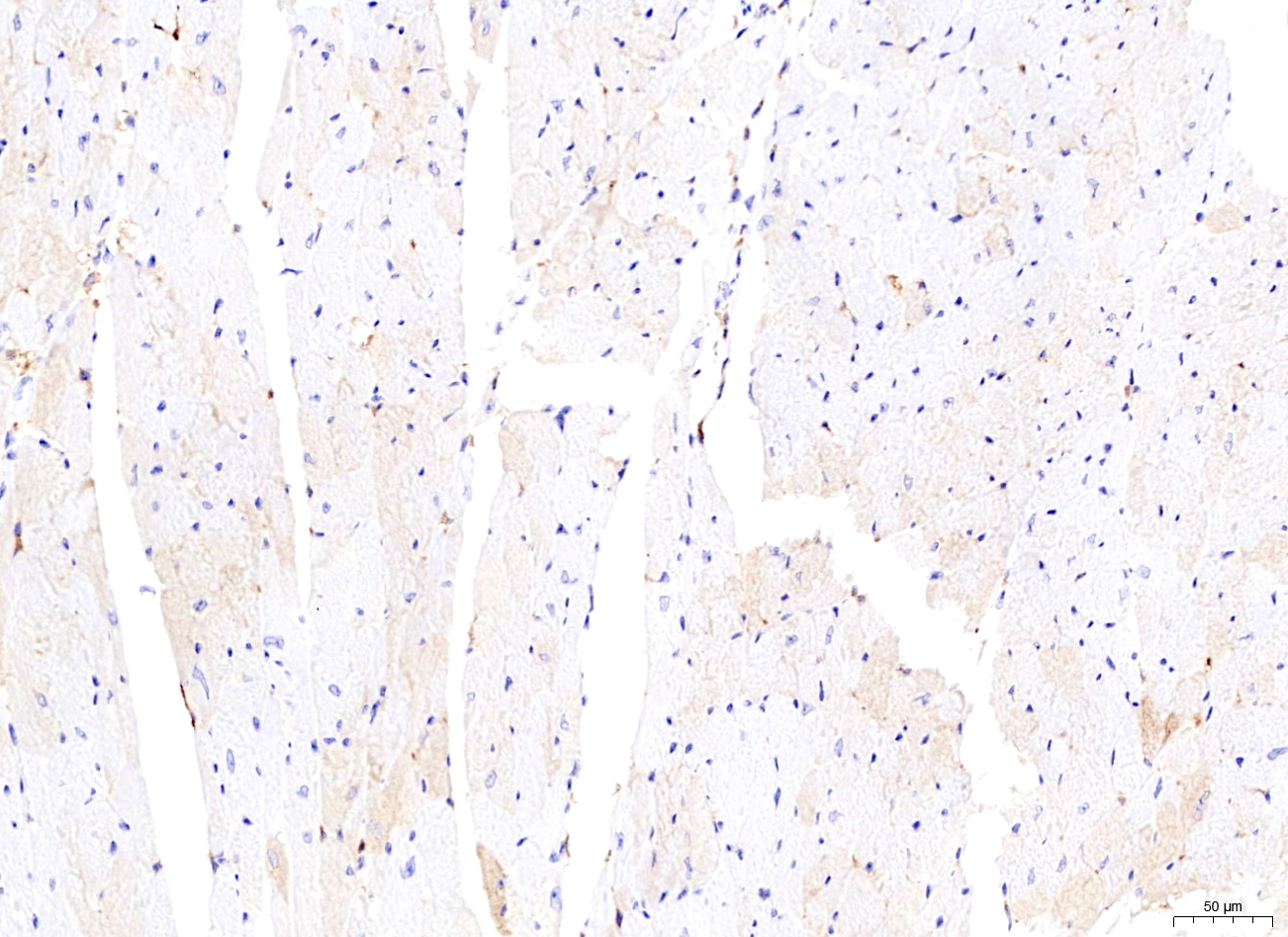

Supplement: Supplementary file 3 — Supporting File 3: advs73867‐sup‐0003‐SupportingFiguresData.zip. [file ADVS-13-e19191-s003.zip › Supporting information Figure S1-S9/S2/Heart/SCRS 12week Model 784_20.0x-4.jpg]

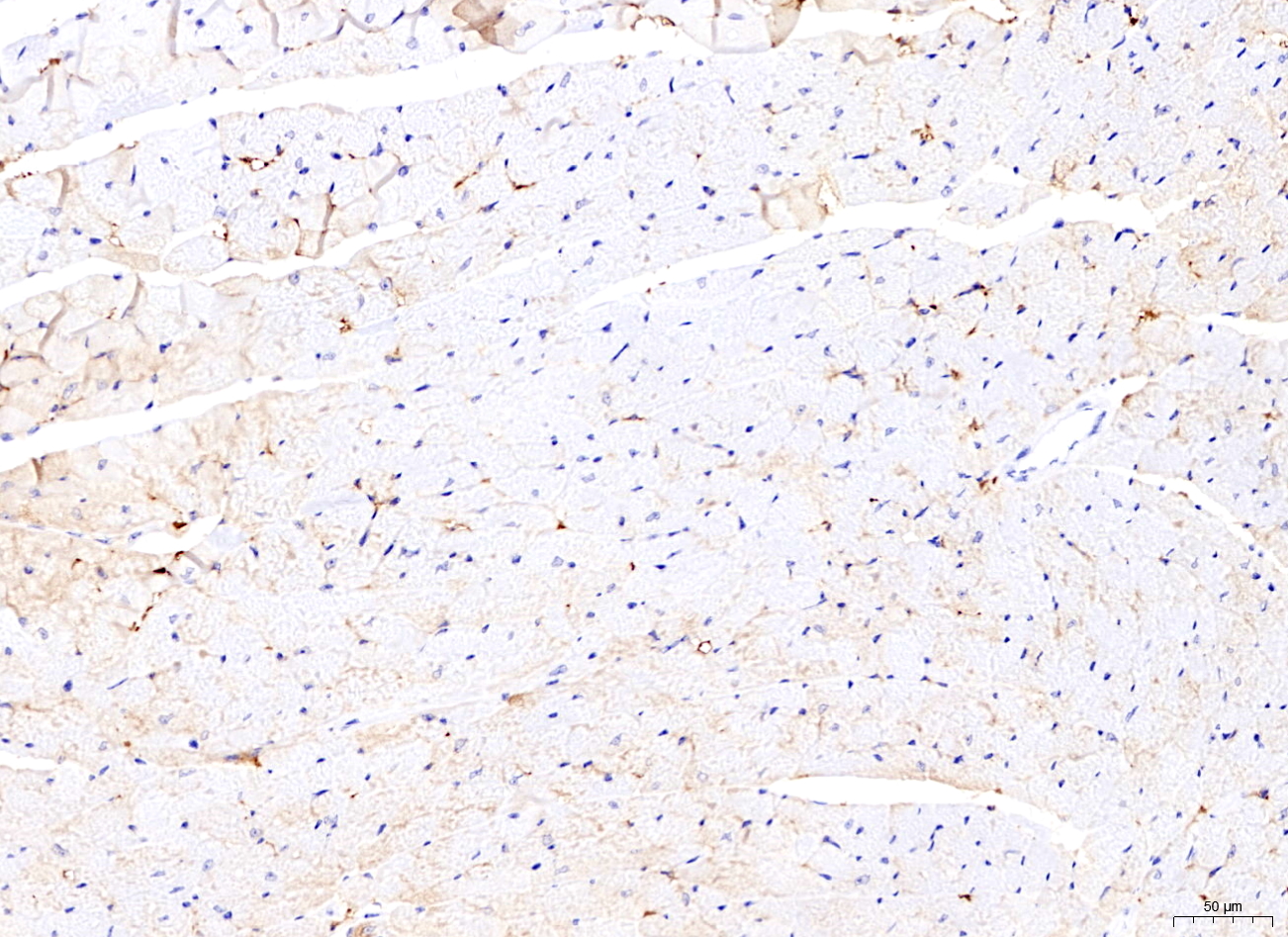

Supplement: Supplementary file 3 — Supporting File 3: advs73867‐sup‐0003‐SupportingFiguresData.zip. [file ADVS-13-e19191-s003.zip › Supporting information Figure S1-S9/S2/Heart/SCRS 12week Model 784_20.0x-5.jpg]

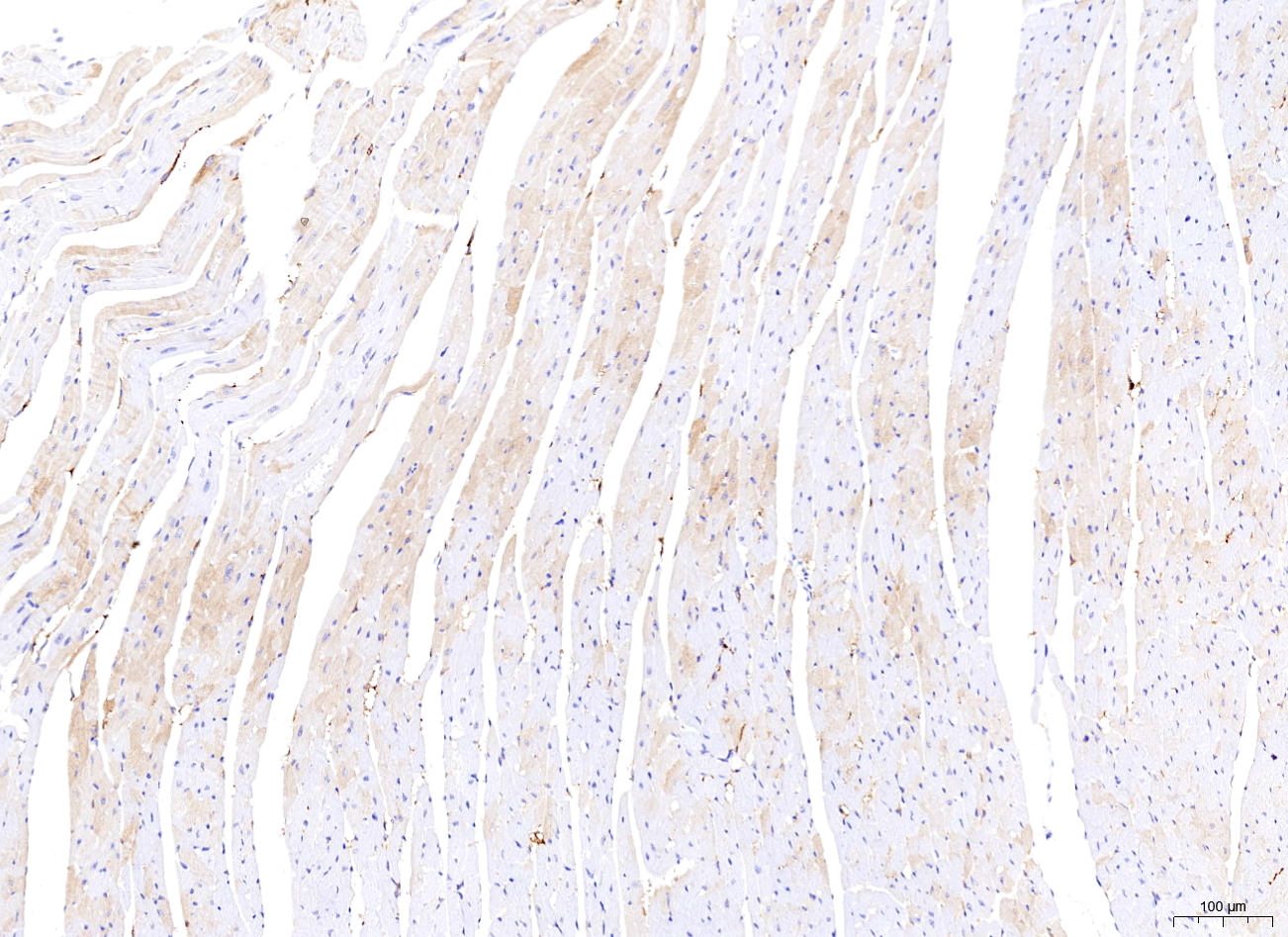

Supplement: Supplementary file 3 — Supporting File 3: advs73867‐sup‐0003‐SupportingFiguresData.zip. [file ADVS-13-e19191-s003.zip › Supporting information Figure S1-S9/S2/Heart/SCRS 1week Model 757_10.0x.jpg]

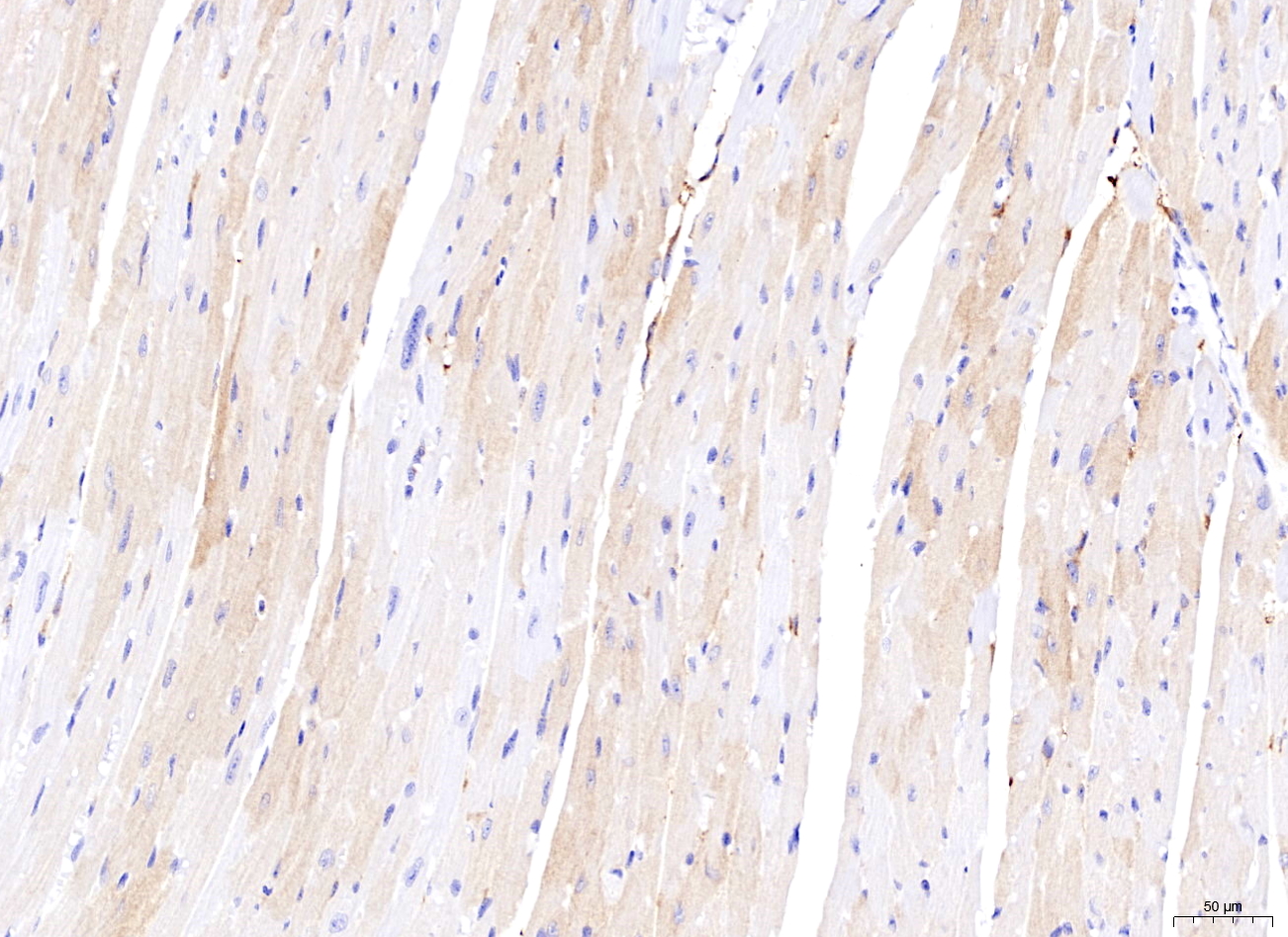

Supplement: Supplementary file 3 — Supporting File 3: advs73867‐sup‐0003‐SupportingFiguresData.zip. [file ADVS-13-e19191-s003.zip › Supporting information Figure S1-S9/S2/Heart/SCRS 1week Model 757_20.0x-1.jpg]

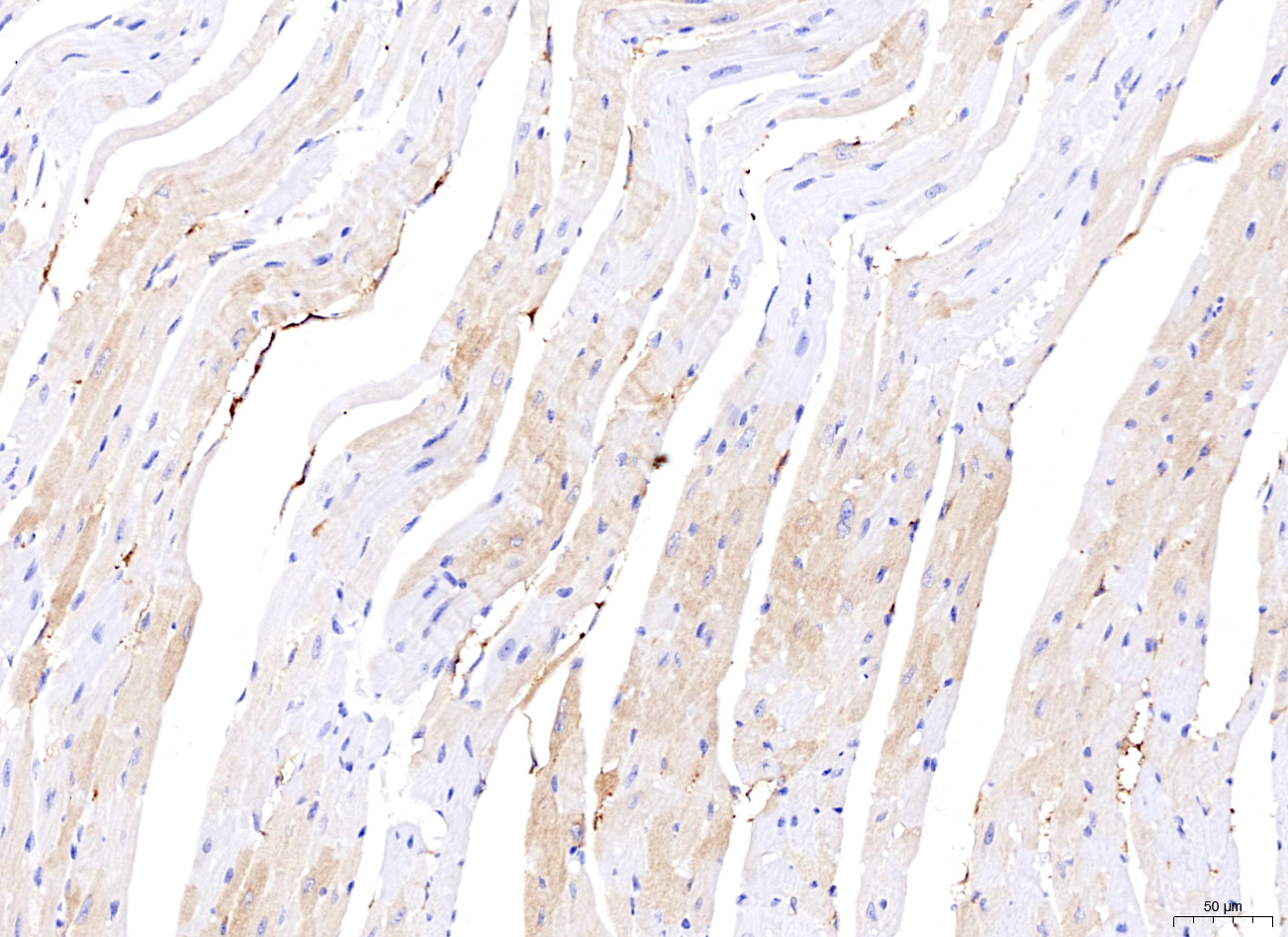

Supplement: Supplementary file 3 — Supporting File 3: advs73867‐sup‐0003‐SupportingFiguresData.zip. [file ADVS-13-e19191-s003.zip › Supporting information Figure S1-S9/S2/Heart/SCRS 1week Model 757_20.0x-2.jpg]

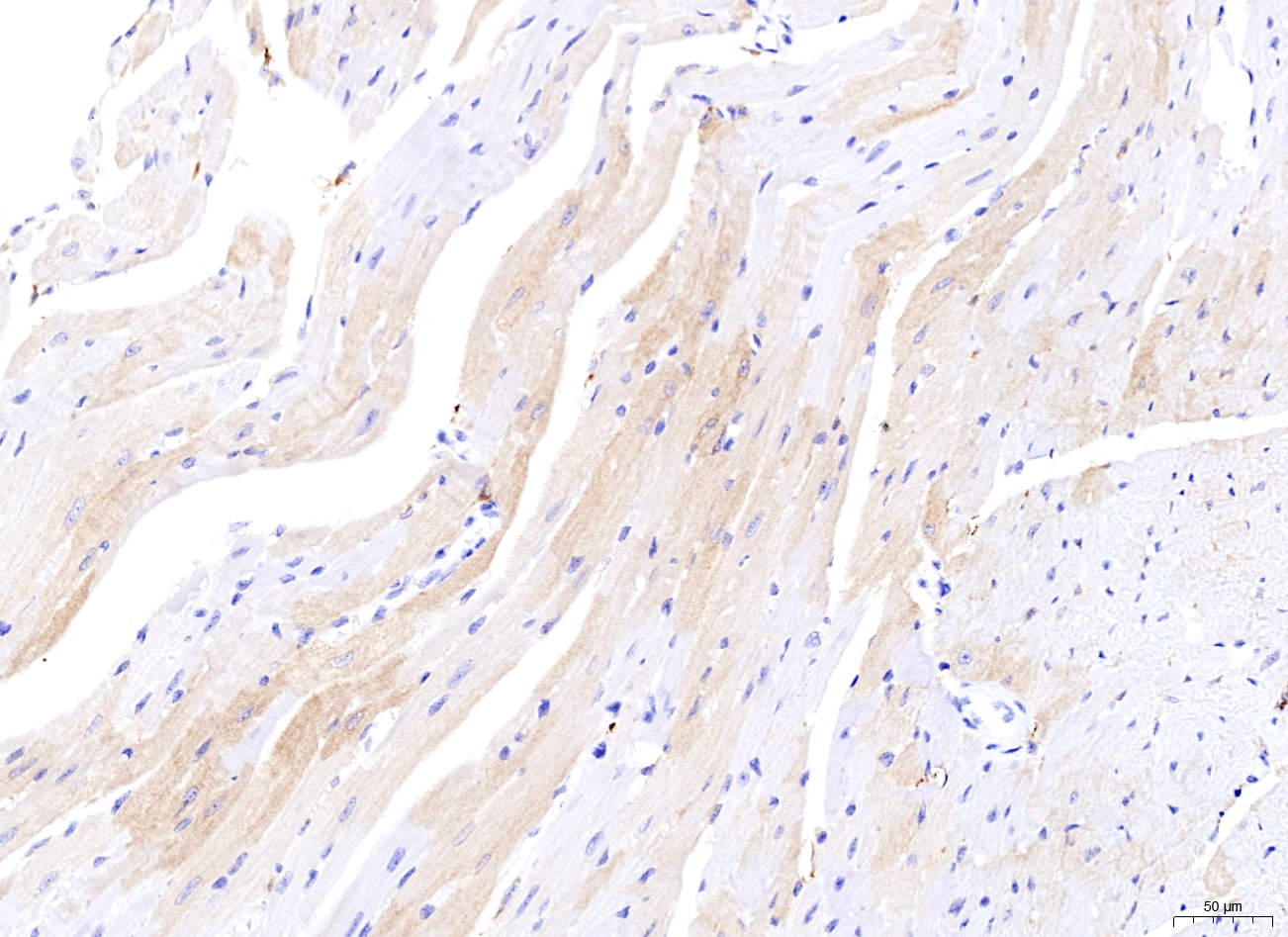

Supplement: Supplementary file 3 — Supporting File 3: advs73867‐sup‐0003‐SupportingFiguresData.zip. [file ADVS-13-e19191-s003.zip › Supporting information Figure S1-S9/S2/Heart/SCRS 1week Model 757_20.0x-3.jpg]

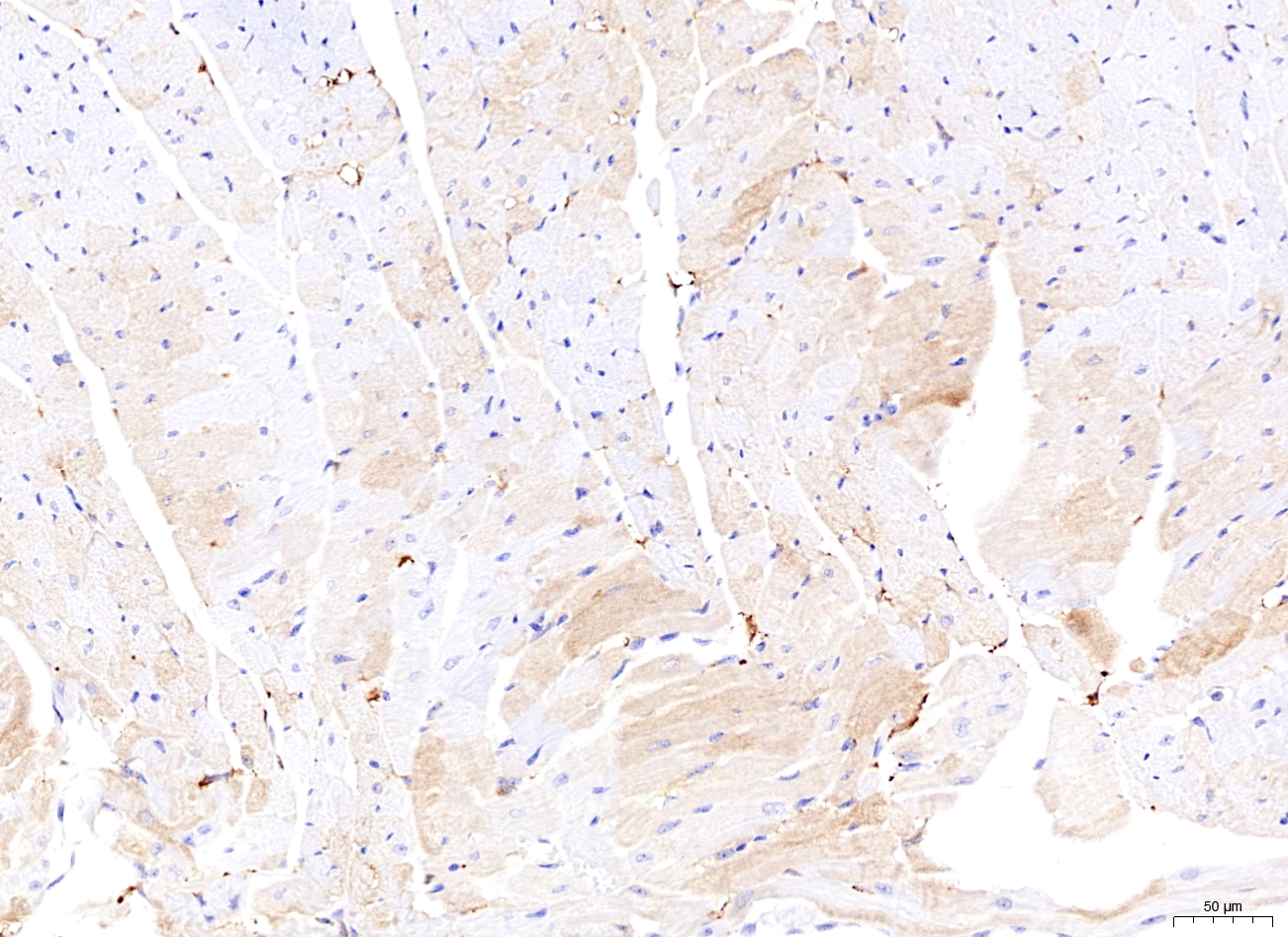

Supplement: Supplementary file 3 — Supporting File 3: advs73867‐sup‐0003‐SupportingFiguresData.zip. [file ADVS-13-e19191-s003.zip › Supporting information Figure S1-S9/S2/Heart/SCRS 1week Model 757_20.0x-4.jpg]

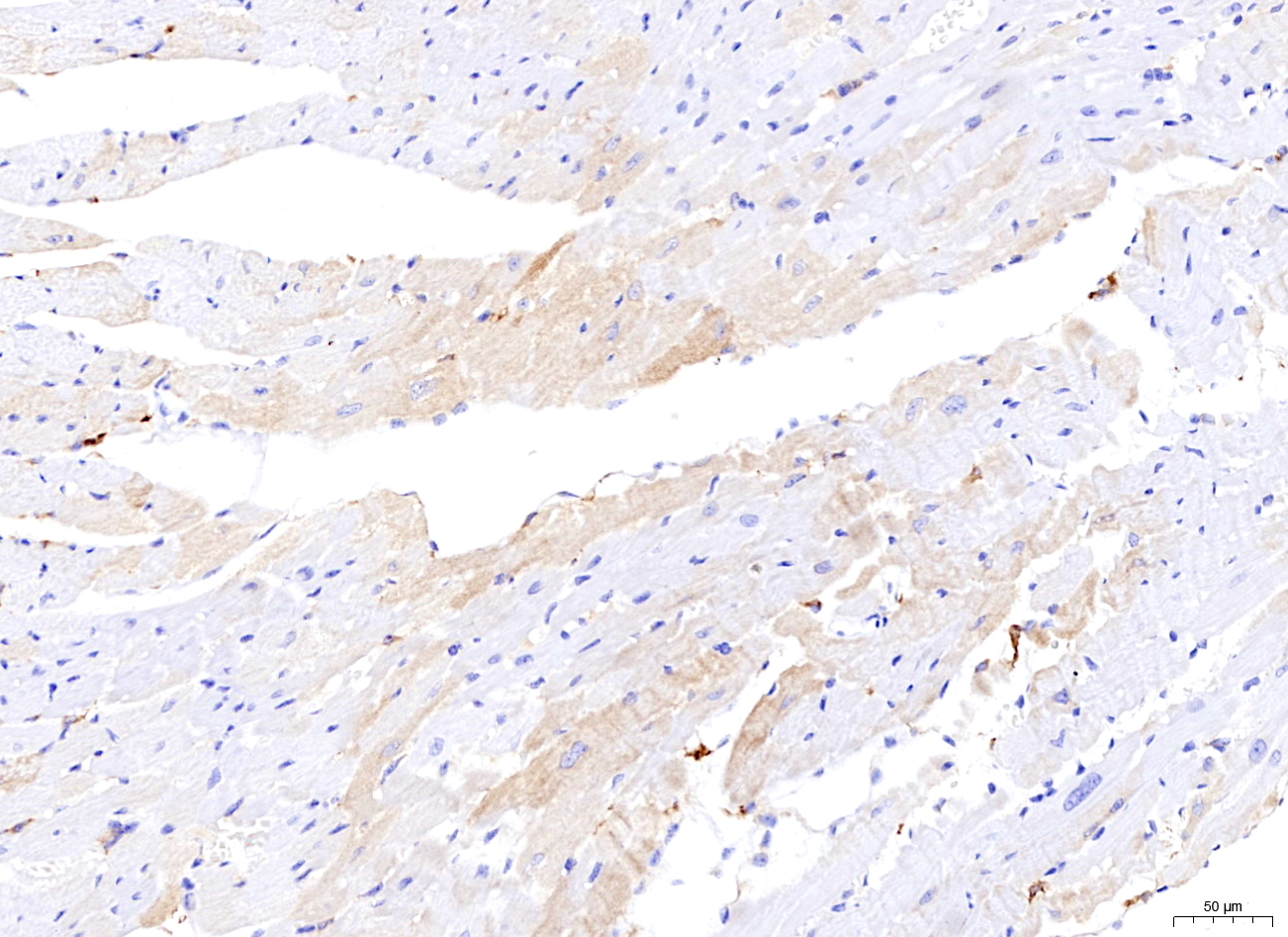

Supplement: Supplementary file 3 — Supporting File 3: advs73867‐sup‐0003‐SupportingFiguresData.zip. [file ADVS-13-e19191-s003.zip › Supporting information Figure S1-S9/S2/Heart/SCRS 1week Model 757_20.0x-5.jpg]

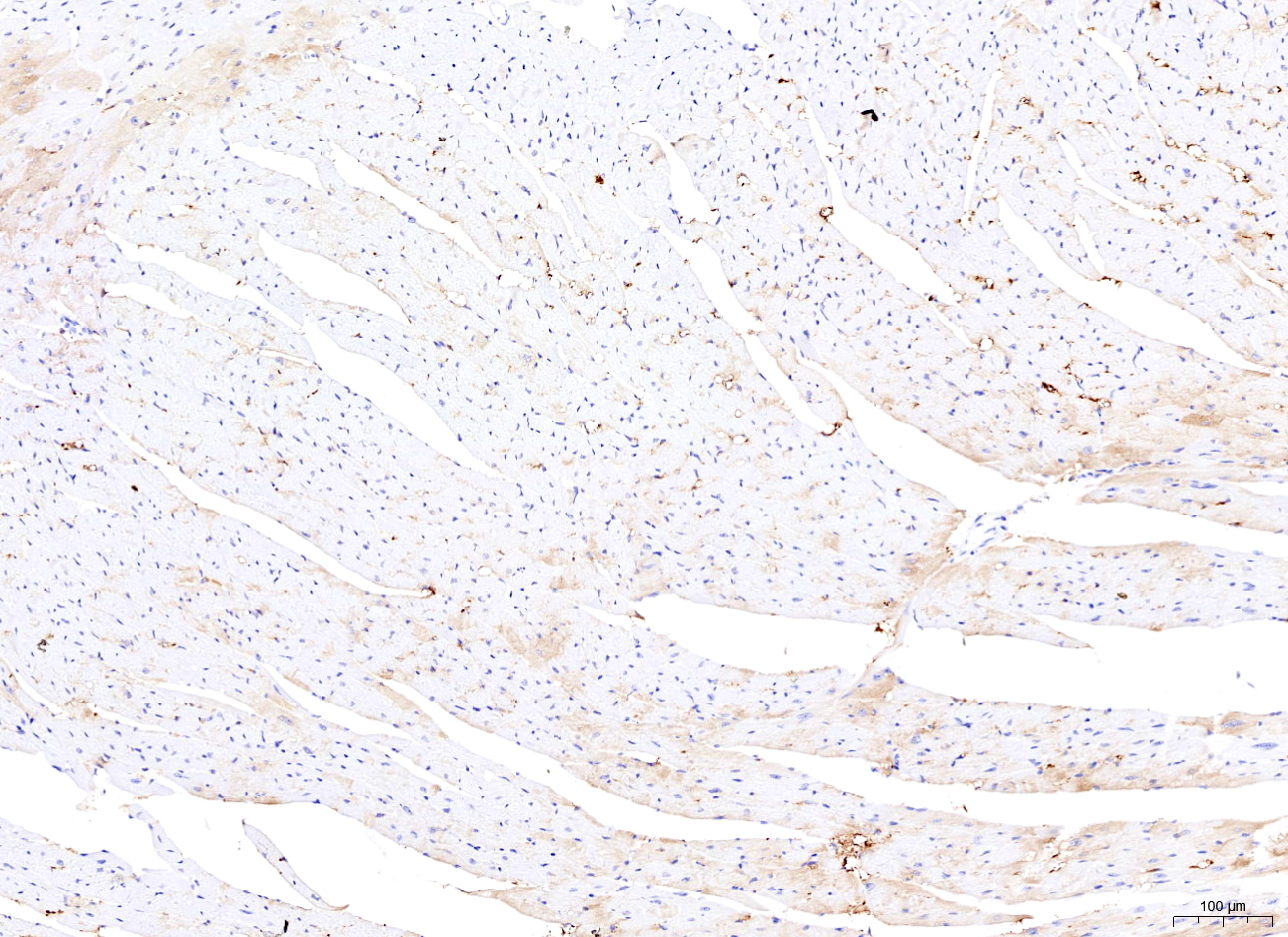

Supplement: Supplementary file 3 — Supporting File 3: advs73867‐sup‐0003‐SupportingFiguresData.zip. [file ADVS-13-e19191-s003.zip › Supporting information Figure S1-S9/S2/Heart/SCRS 4week Model 744_10.0x.jpg]

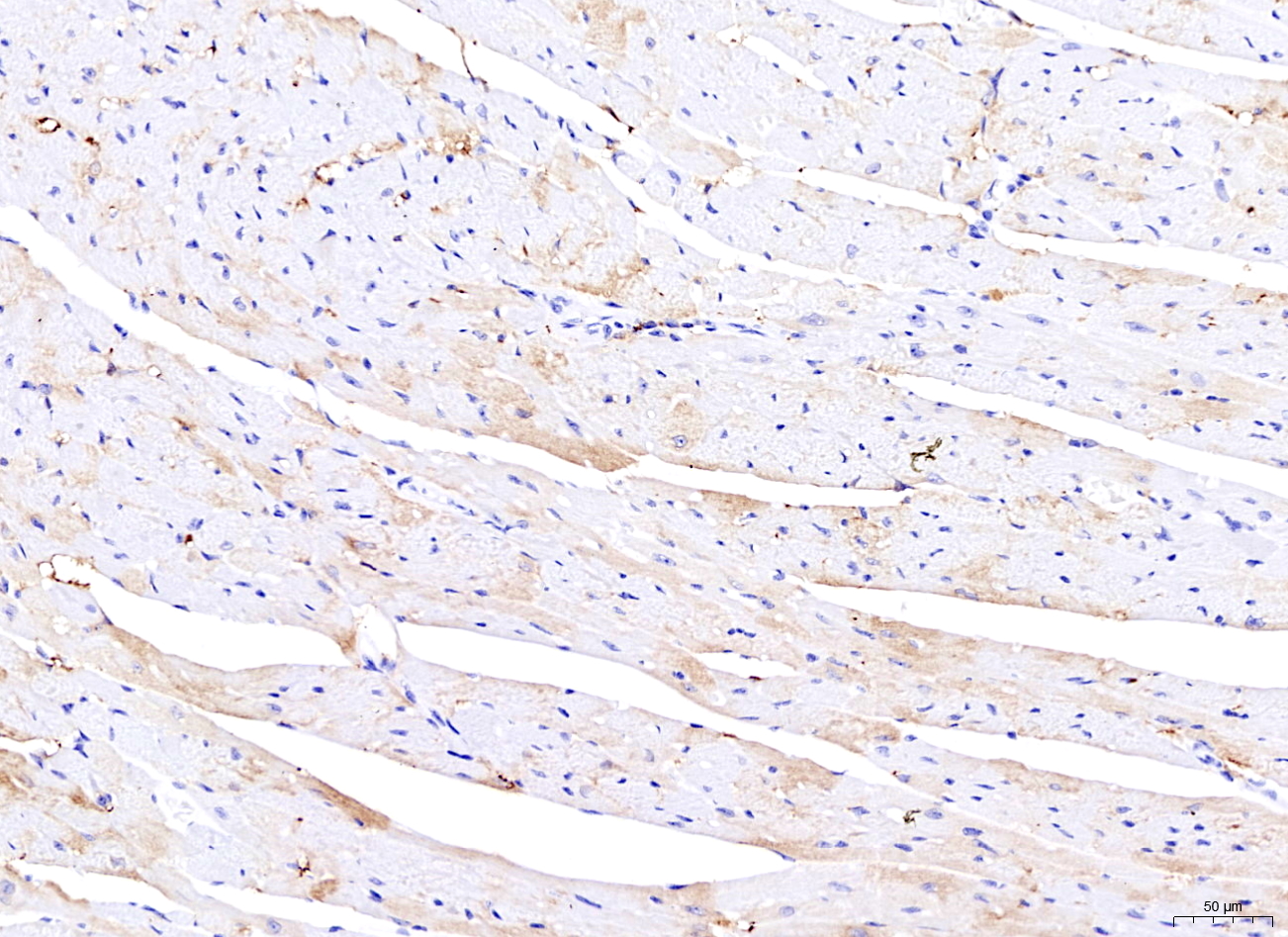

Supplement: Supplementary file 3 — Supporting File 3: advs73867‐sup‐0003‐SupportingFiguresData.zip. [file ADVS-13-e19191-s003.zip › Supporting information Figure S1-S9/S2/Heart/SCRS 4week Model 744_20.0x-1.jpg]

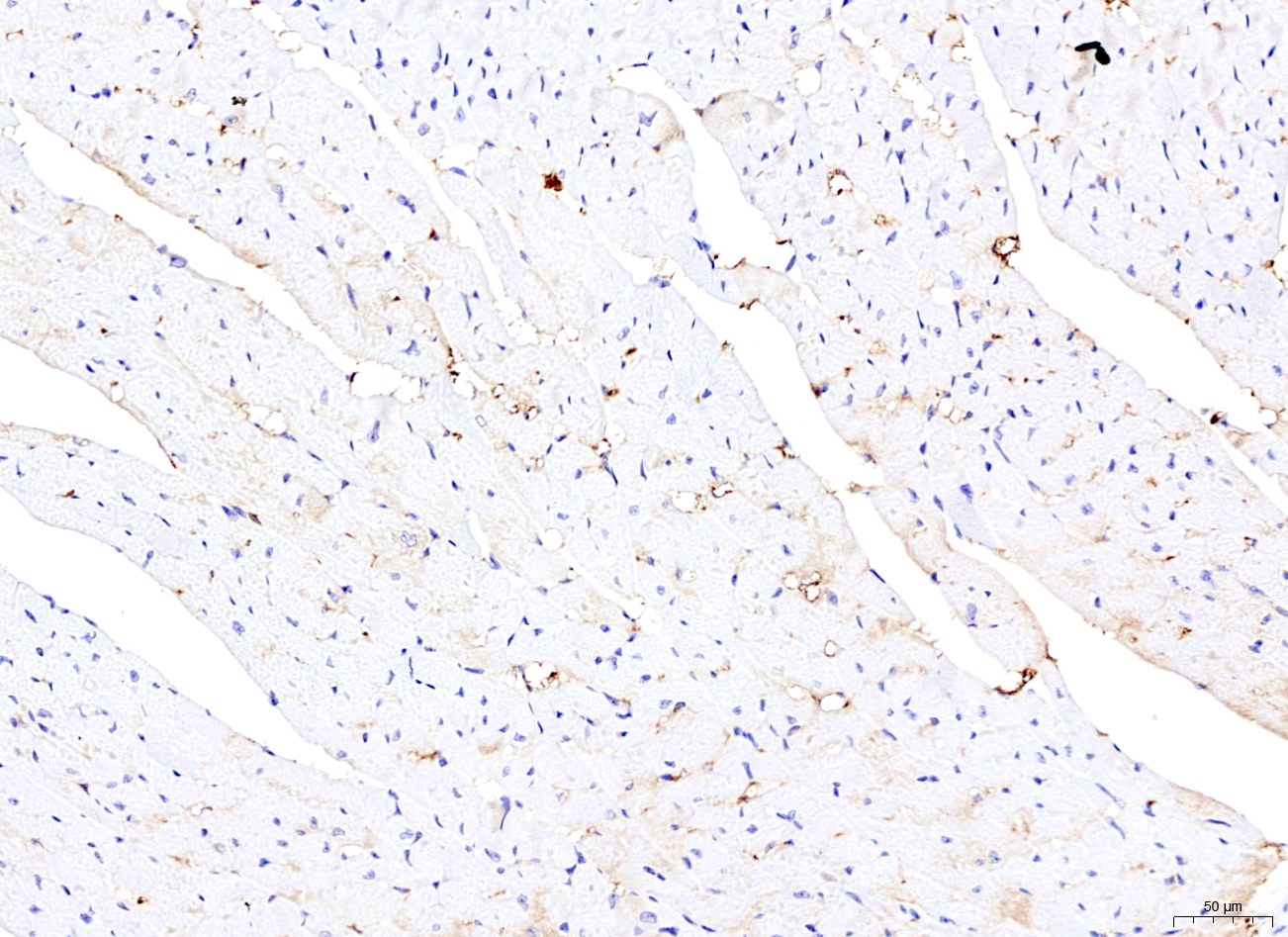

Supplement: Supplementary file 3 — Supporting File 3: advs73867‐sup‐0003‐SupportingFiguresData.zip. [file ADVS-13-e19191-s003.zip › Supporting information Figure S1-S9/S2/Heart/SCRS 4week Model 744_20.0x-2.jpg]

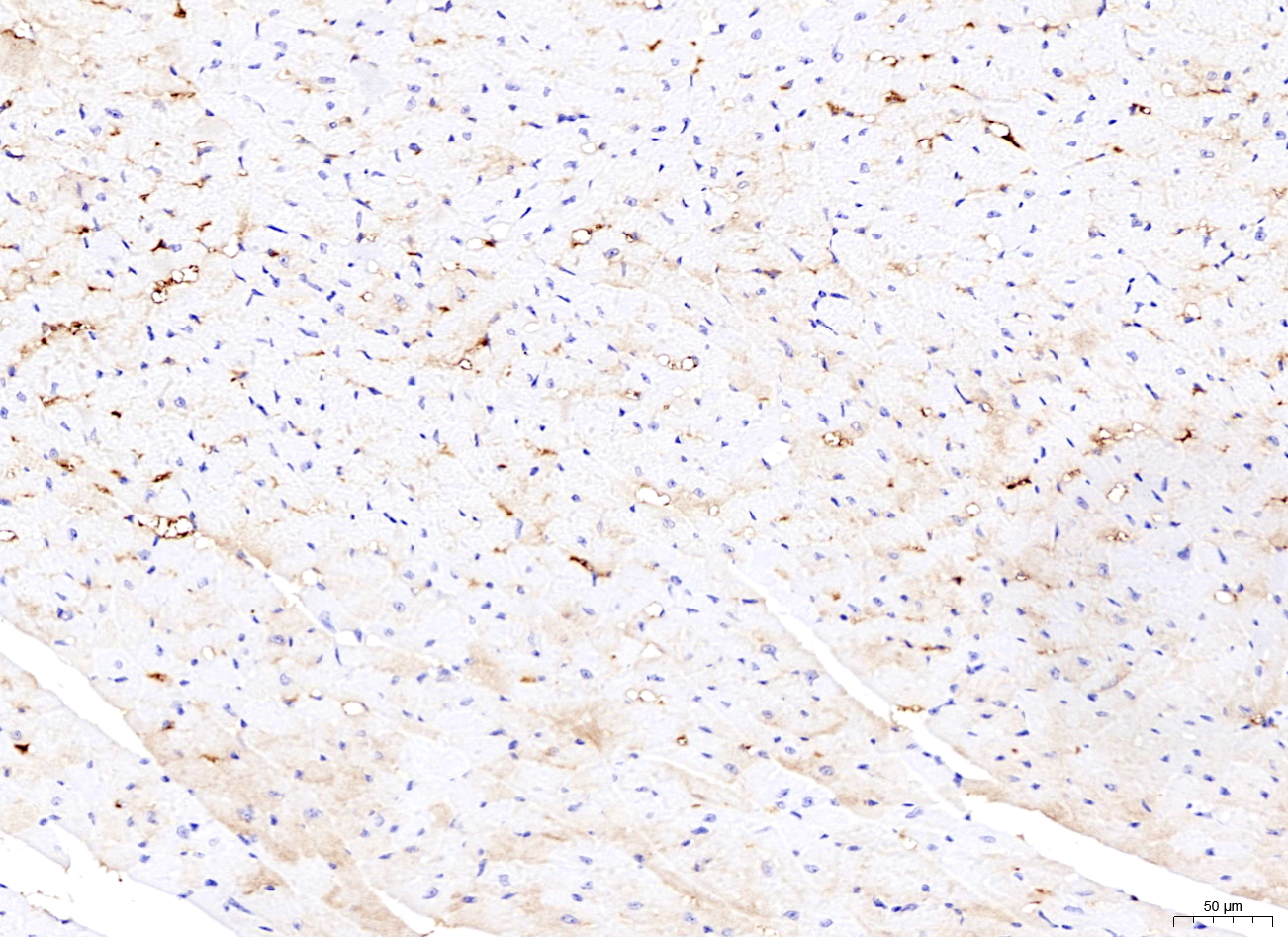

Supplement: Supplementary file 3 — Supporting File 3: advs73867‐sup‐0003‐SupportingFiguresData.zip. [file ADVS-13-e19191-s003.zip › Supporting information Figure S1-S9/S2/Heart/SCRS 4week Model 744_20.0x-3.jpg]

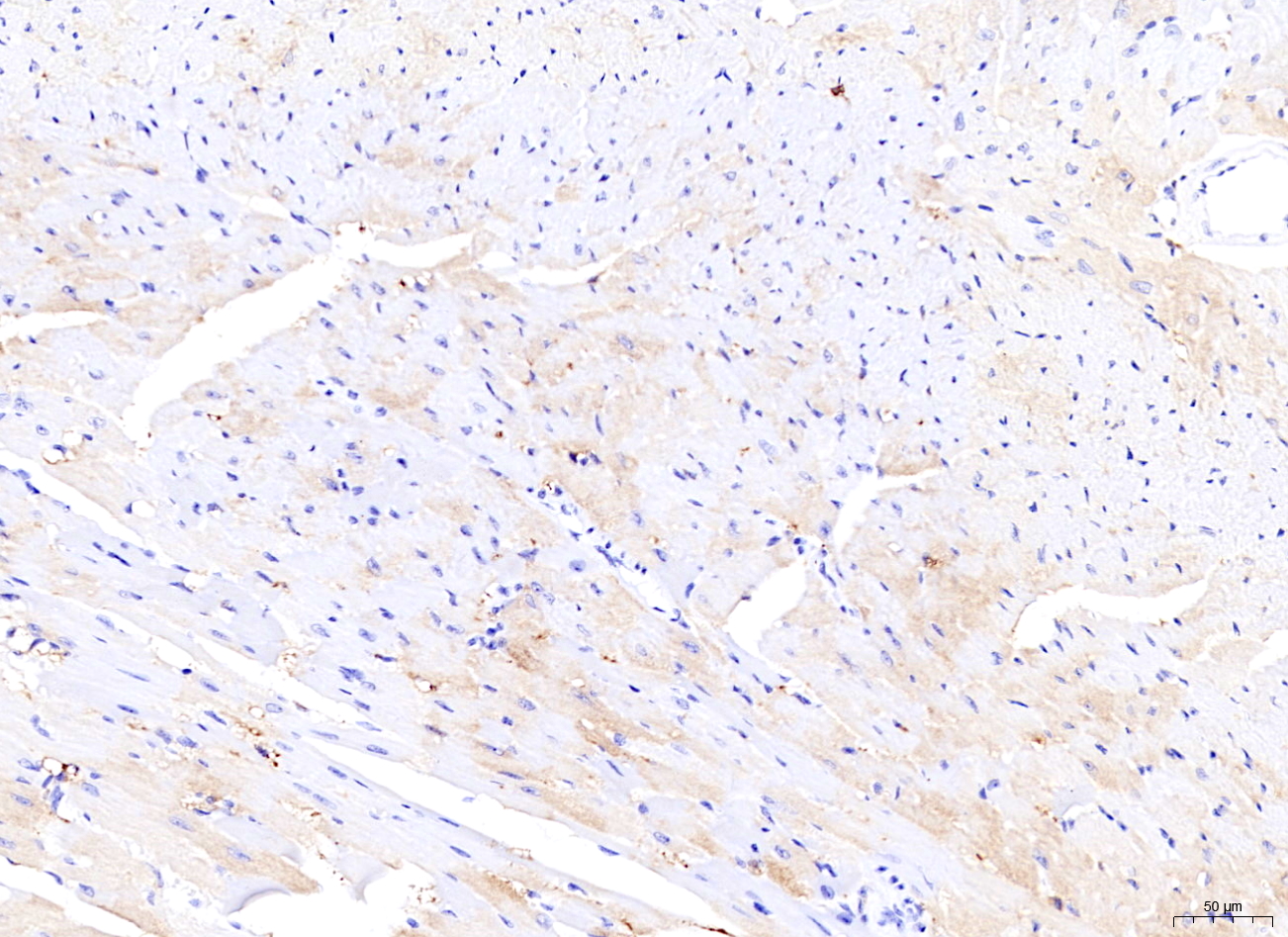

Supplement: Supplementary file 3 — Supporting File 3: advs73867‐sup‐0003‐SupportingFiguresData.zip. [file ADVS-13-e19191-s003.zip › Supporting information Figure S1-S9/S2/Heart/SCRS 4week Model 744_20.0x-4.jpg]

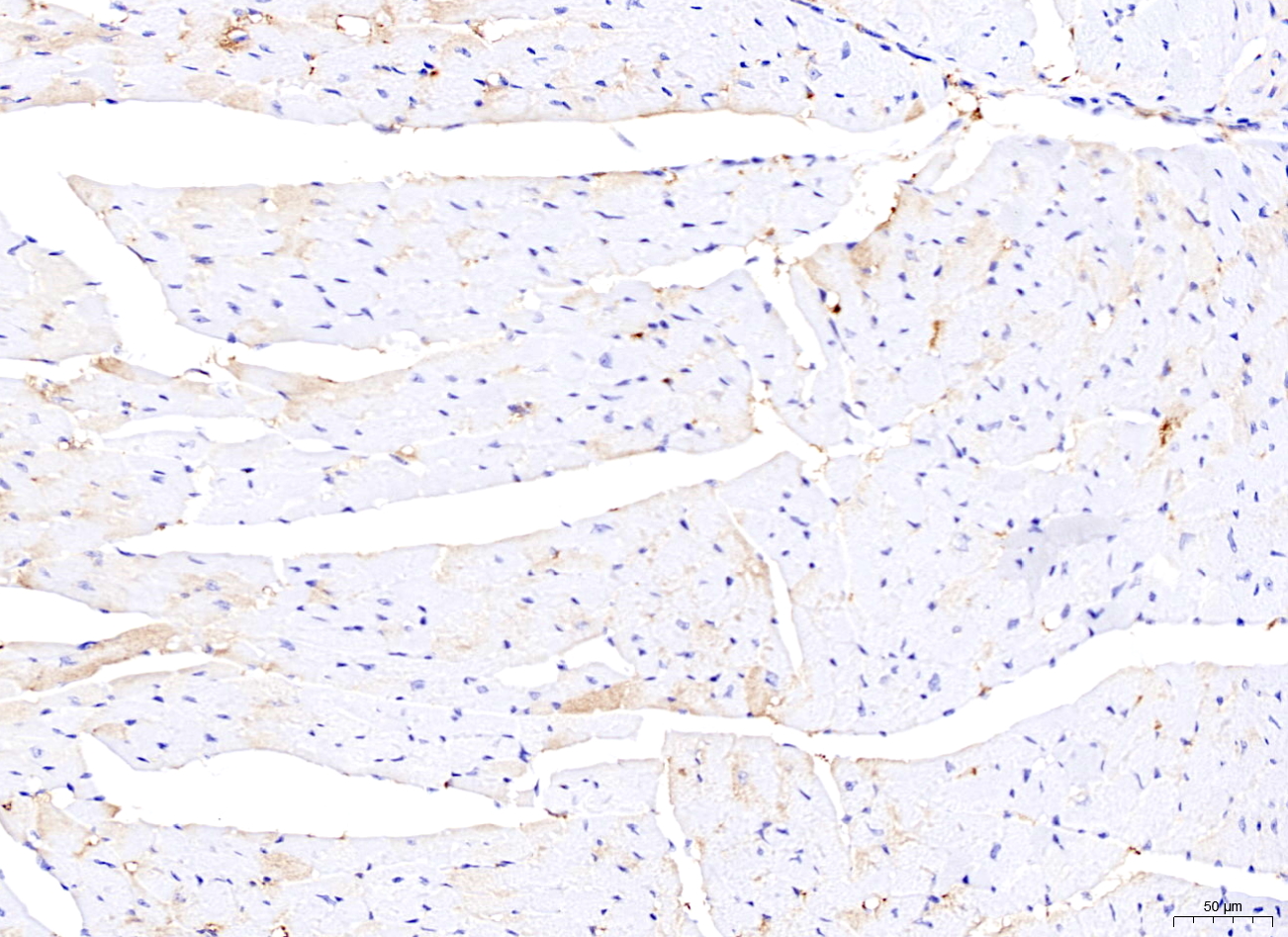

Supplement: Supplementary file 3 — Supporting File 3: advs73867‐sup‐0003‐SupportingFiguresData.zip. [file ADVS-13-e19191-s003.zip › Supporting information Figure S1-S9/S2/Heart/SCRS 4week Model 744_20.0x-5.jpg]

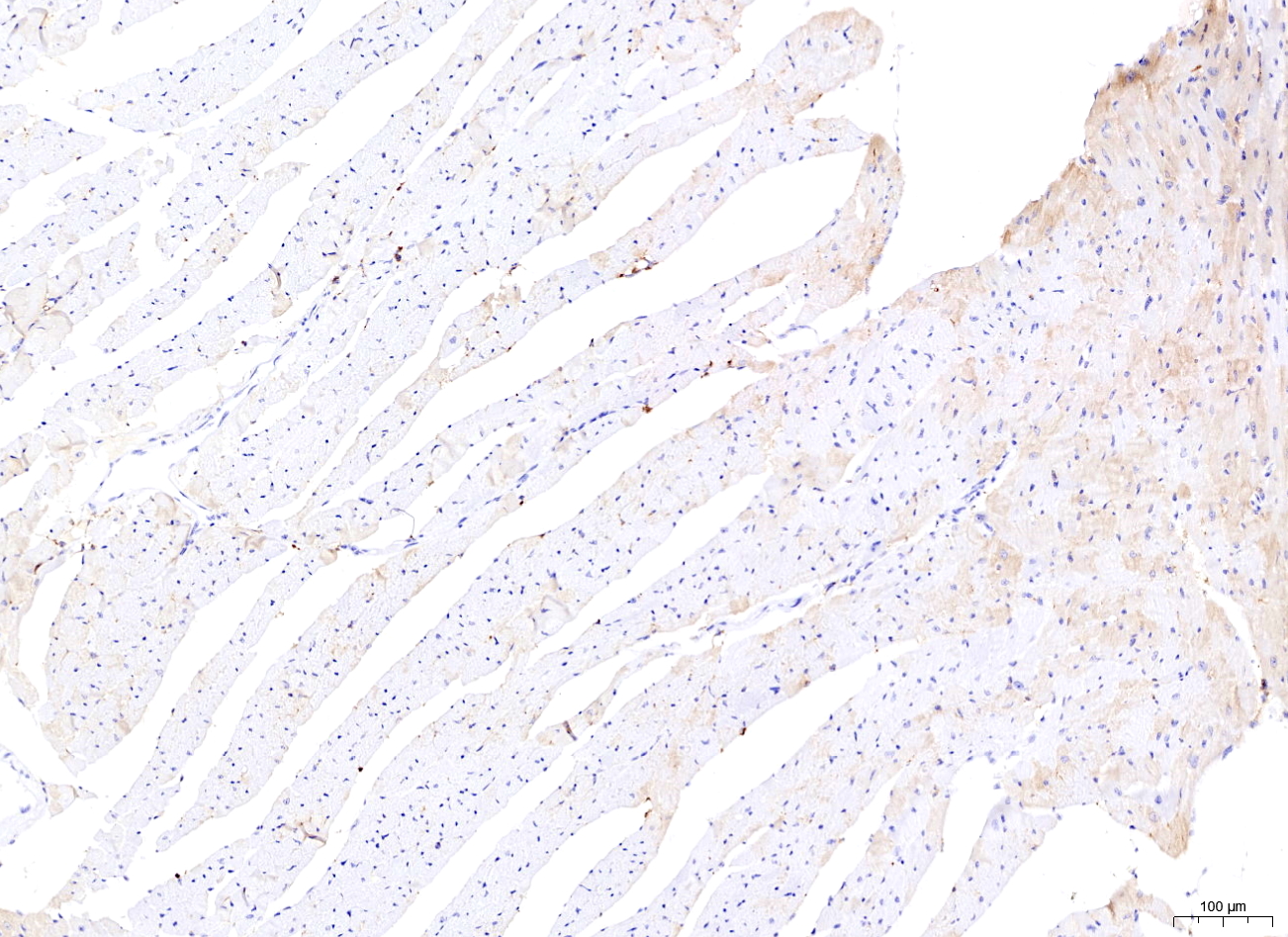

Supplement: Supplementary file 3 — Supporting File 3: advs73867‐sup‐0003‐SupportingFiguresData.zip. [file ADVS-13-e19191-s003.zip › Supporting information Figure S1-S9/S2/Heart/SCRS 8week Model 763_10.0x.jpg]

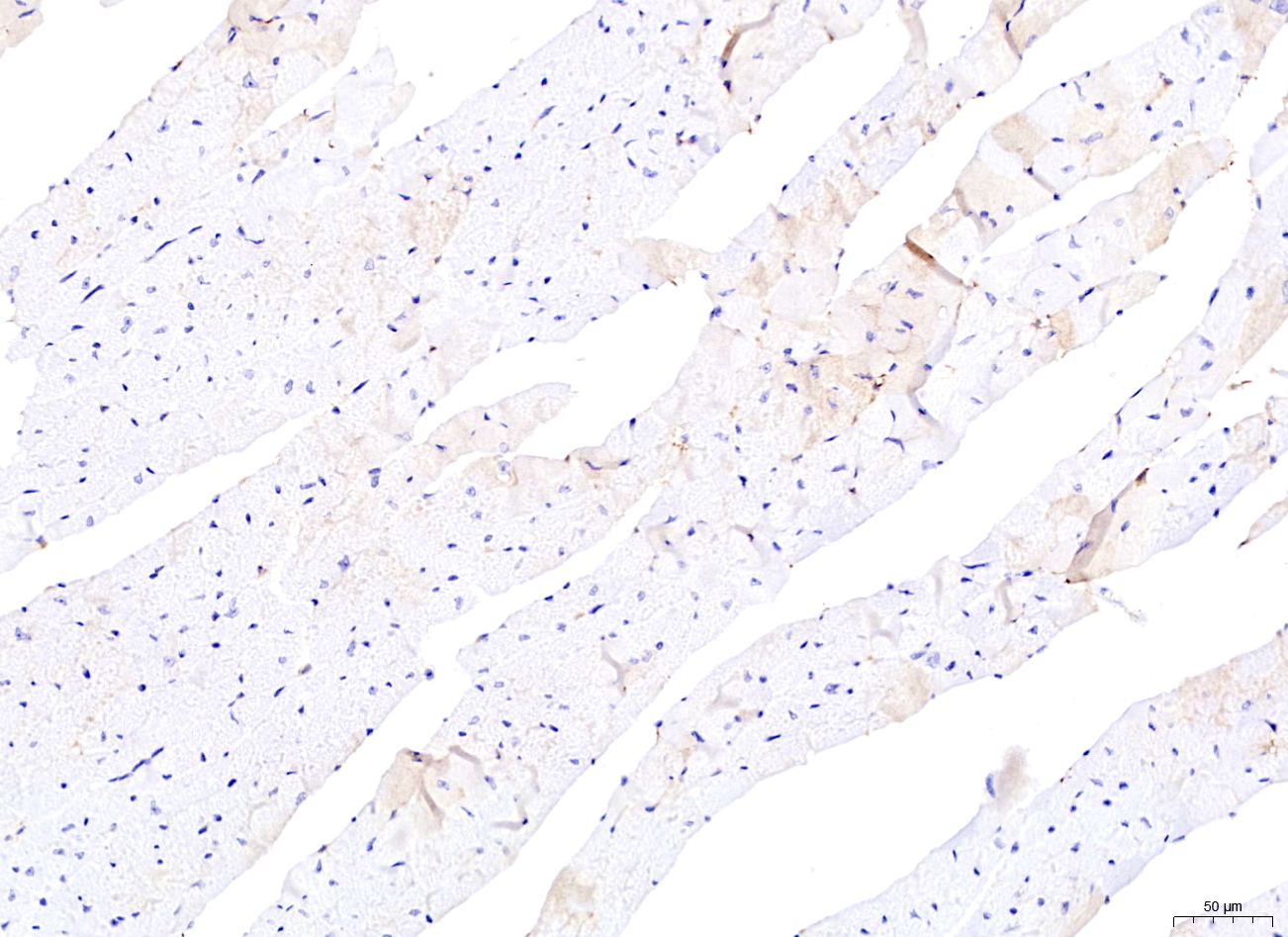

Supplement: Supplementary file 3 — Supporting File 3: advs73867‐sup‐0003‐SupportingFiguresData.zip. [file ADVS-13-e19191-s003.zip › Supporting information Figure S1-S9/S2/Heart/SCRS 8week Model 763_20.0x-1.jpg]

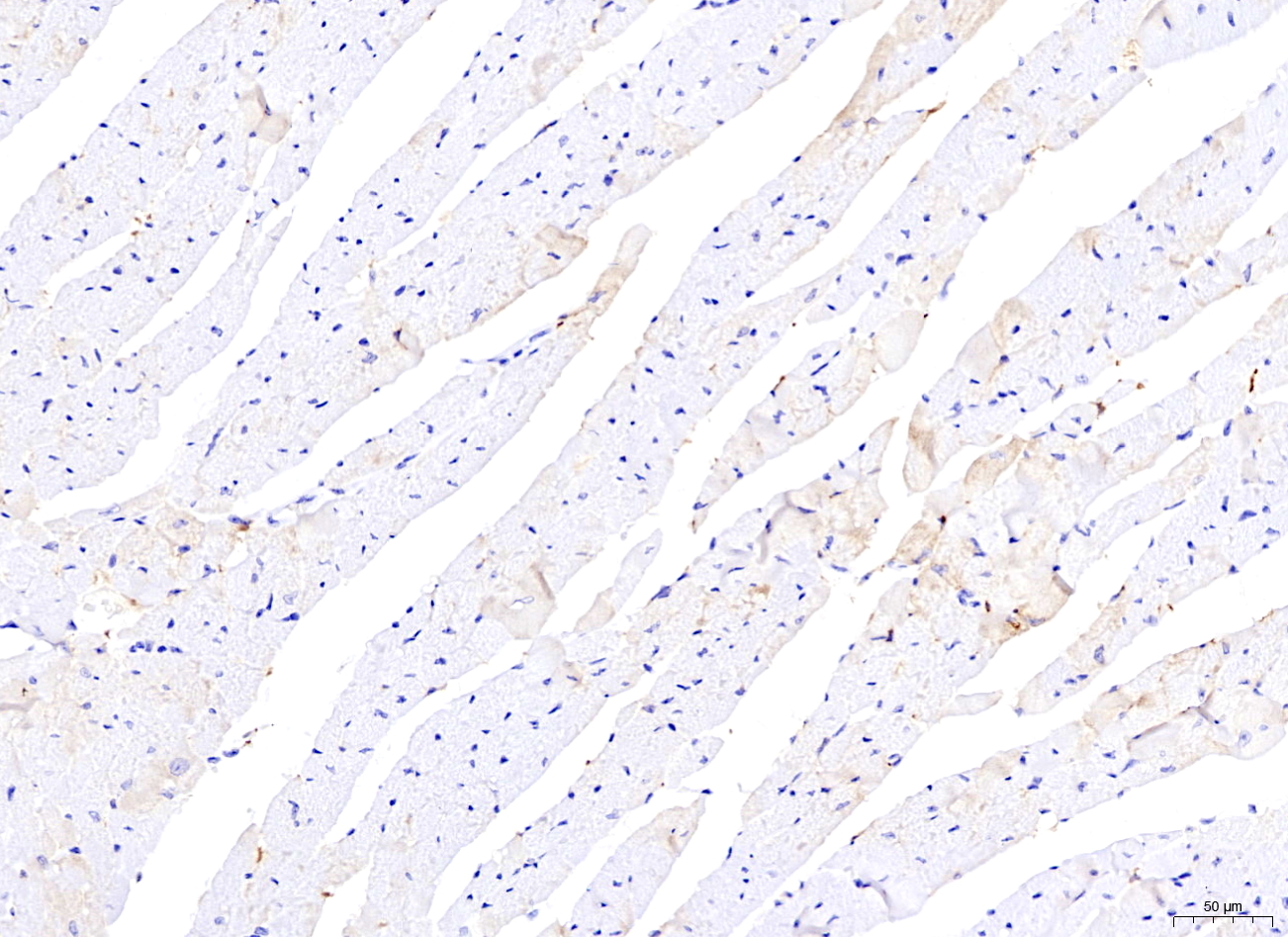

Supplement: Supplementary file 3 — Supporting File 3: advs73867‐sup‐0003‐SupportingFiguresData.zip. [file ADVS-13-e19191-s003.zip › Supporting information Figure S1-S9/S2/Heart/SCRS 8week Model 763_20.0x-2.jpg]

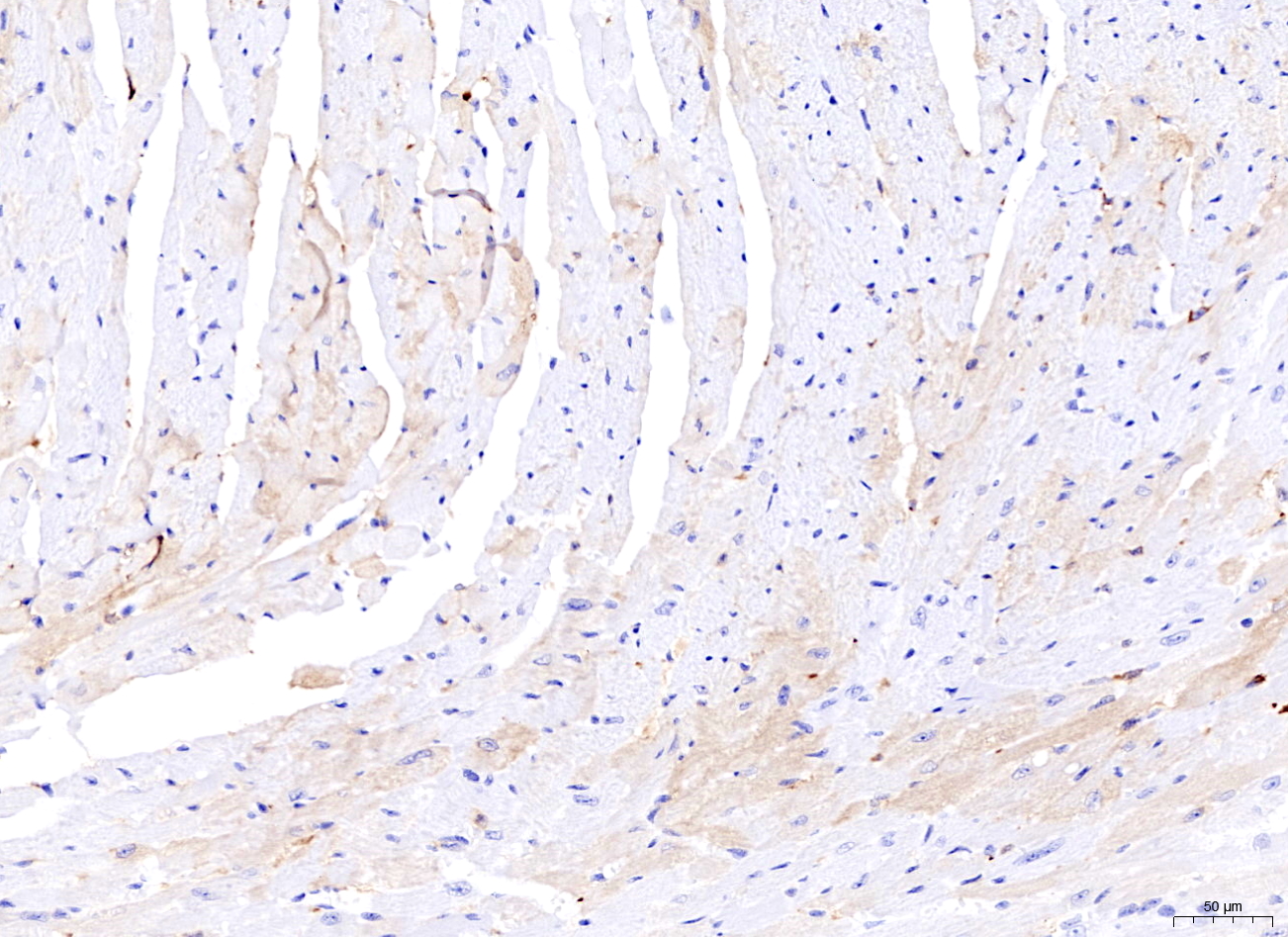

Supplement: Supplementary file 3 — Supporting File 3: advs73867‐sup‐0003‐SupportingFiguresData.zip. [file ADVS-13-e19191-s003.zip › Supporting information Figure S1-S9/S2/Heart/SCRS 8week Model 763_20.0x-3.jpg]

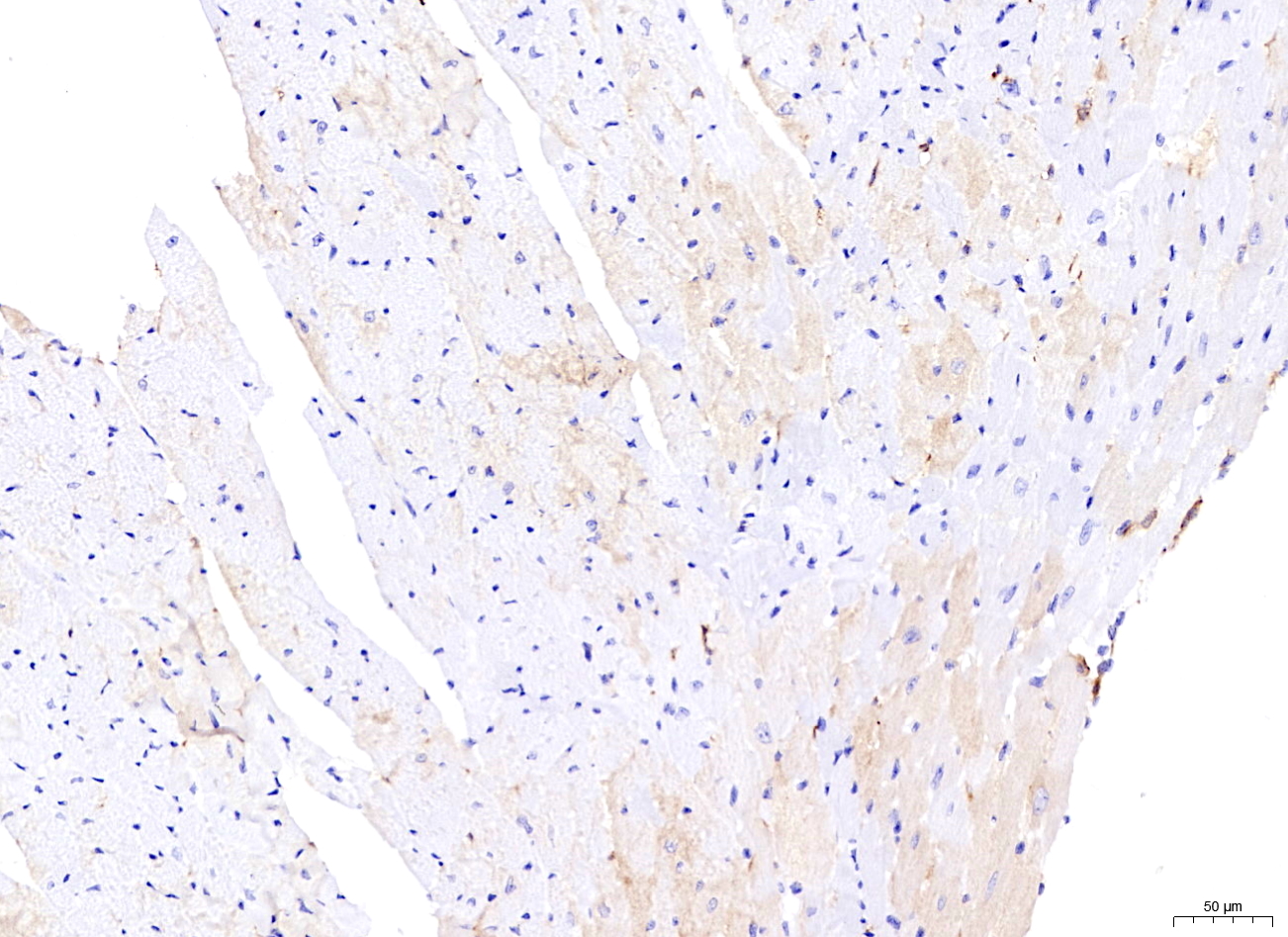

Supplement: Supplementary file 3 — Supporting File 3: advs73867‐sup‐0003‐SupportingFiguresData.zip. [file ADVS-13-e19191-s003.zip › Supporting information Figure S1-S9/S2/Heart/SCRS 8week Model 763_20.0x-4.jpg]

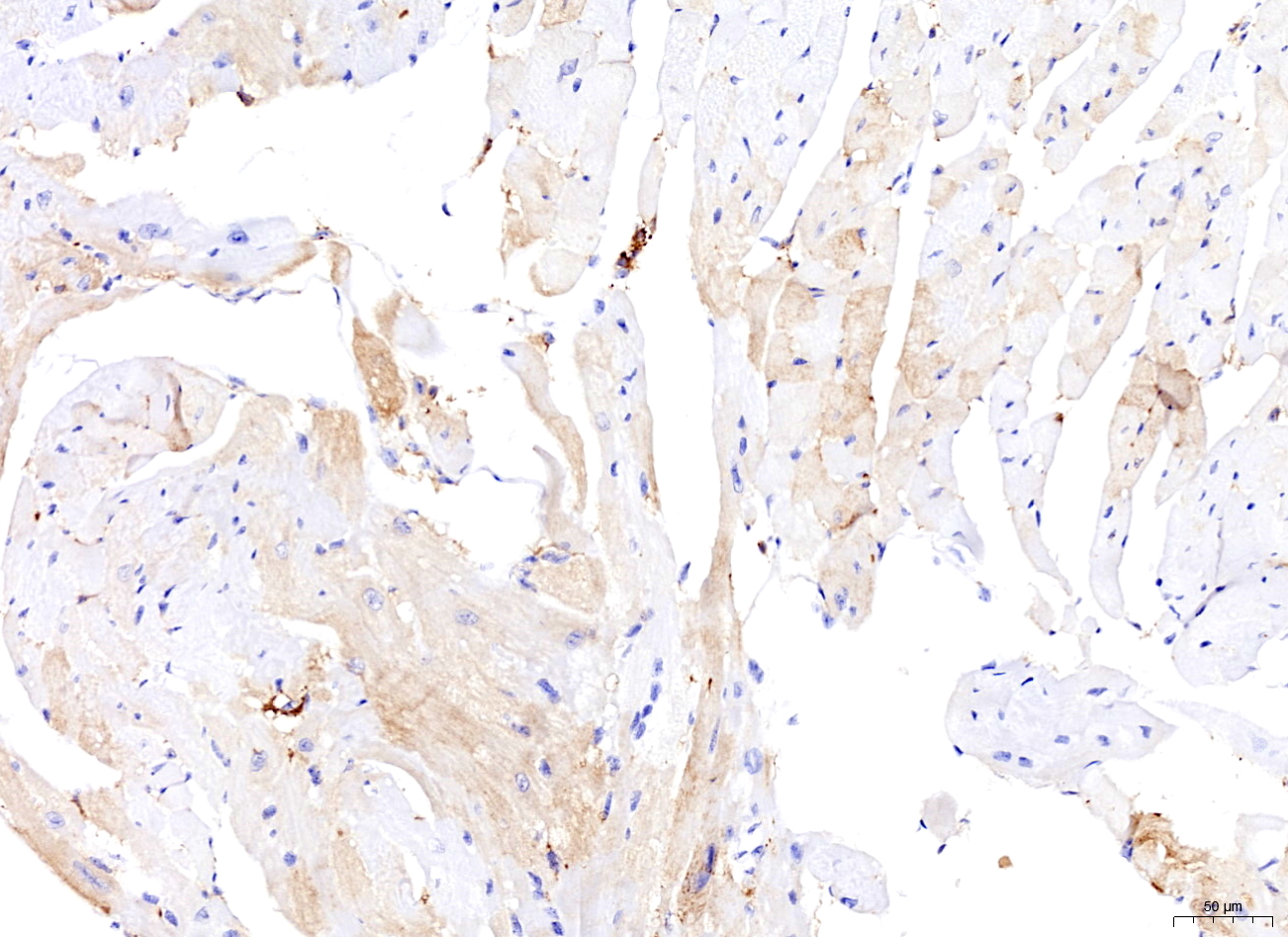

Supplement: Supplementary file 3 — Supporting File 3: advs73867‐sup‐0003‐SupportingFiguresData.zip. [file ADVS-13-e19191-s003.zip › Supporting information Figure S1-S9/S2/Heart/SCRS 8week Model 763_20.0x-5.jpg]

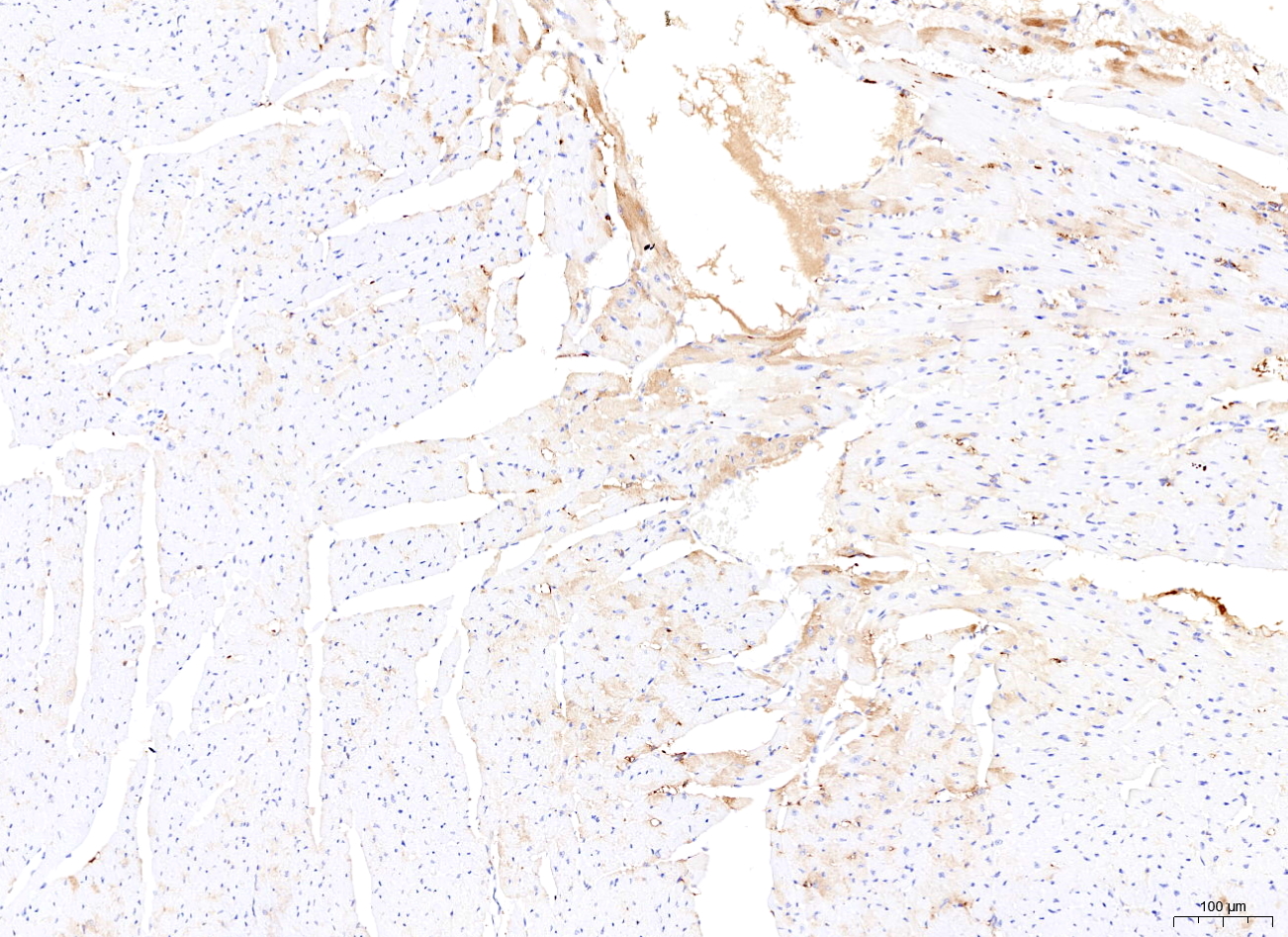

Supplement: Supplementary file 3 — Supporting File 3: advs73867‐sup‐0003‐SupportingFiguresData.zip. [file ADVS-13-e19191-s003.zip › Supporting information Figure S1-S9/S2/Heart/SCRS Control 782_10.0x.jpg]

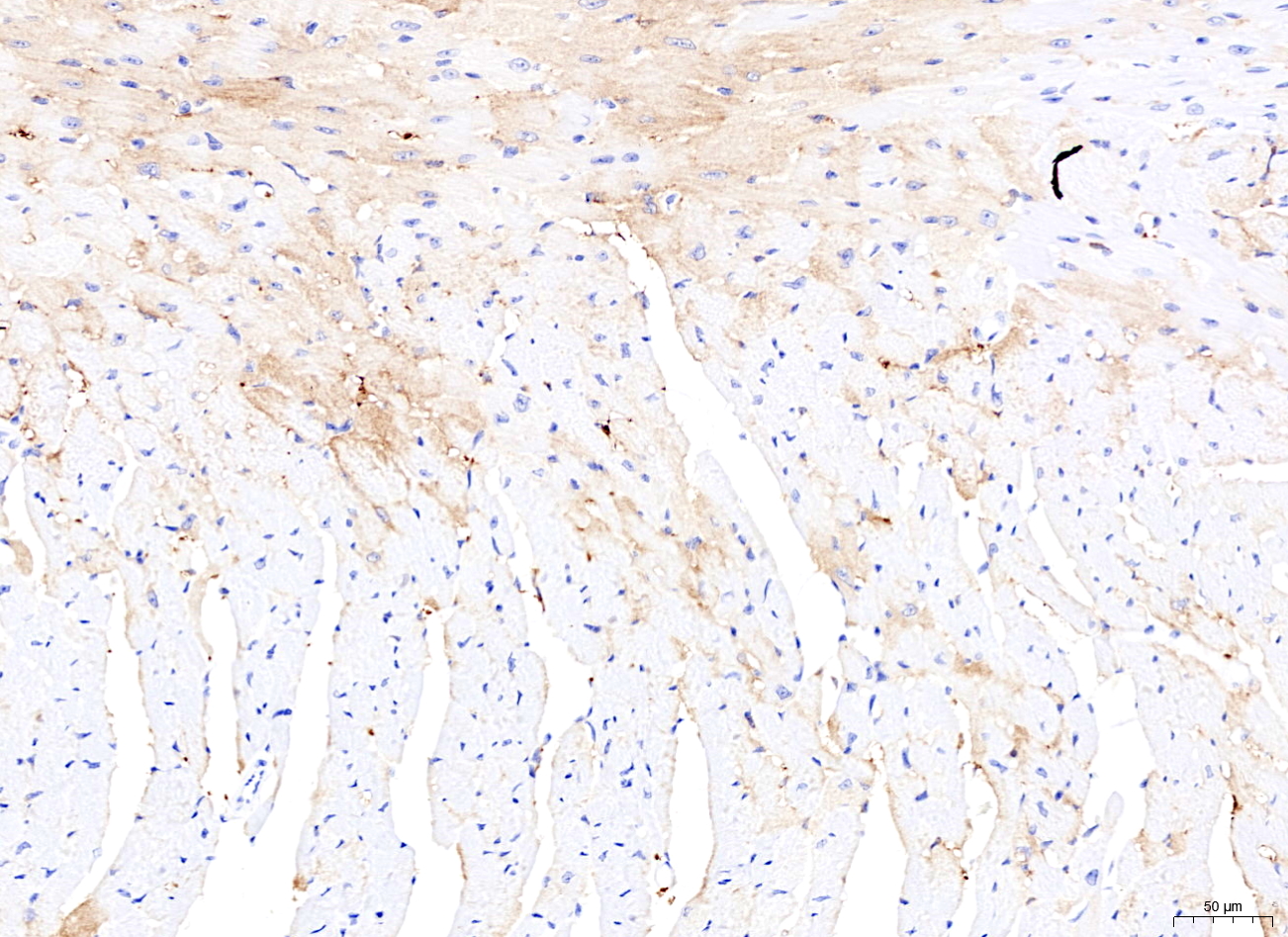

Supplement: Supplementary file 3 — Supporting File 3: advs73867‐sup‐0003‐SupportingFiguresData.zip. [file ADVS-13-e19191-s003.zip › Supporting information Figure S1-S9/S2/Heart/SCRS Control 782_20.0x-1.jpg]

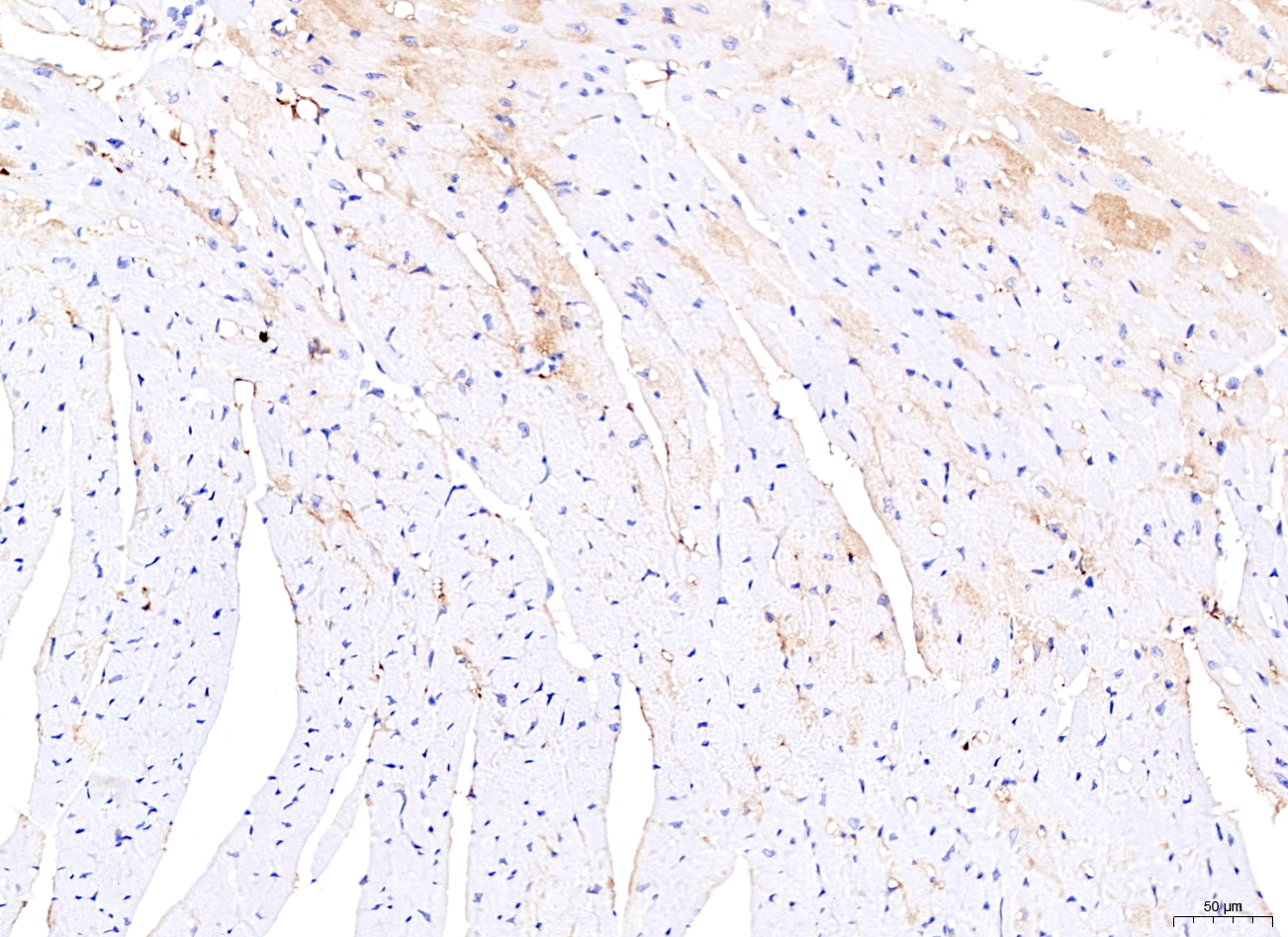

Supplement: Supplementary file 3 — Supporting File 3: advs73867‐sup‐0003‐SupportingFiguresData.zip. [file ADVS-13-e19191-s003.zip › Supporting information Figure S1-S9/S2/Heart/SCRS Control 782_20.0x-2.jpg]

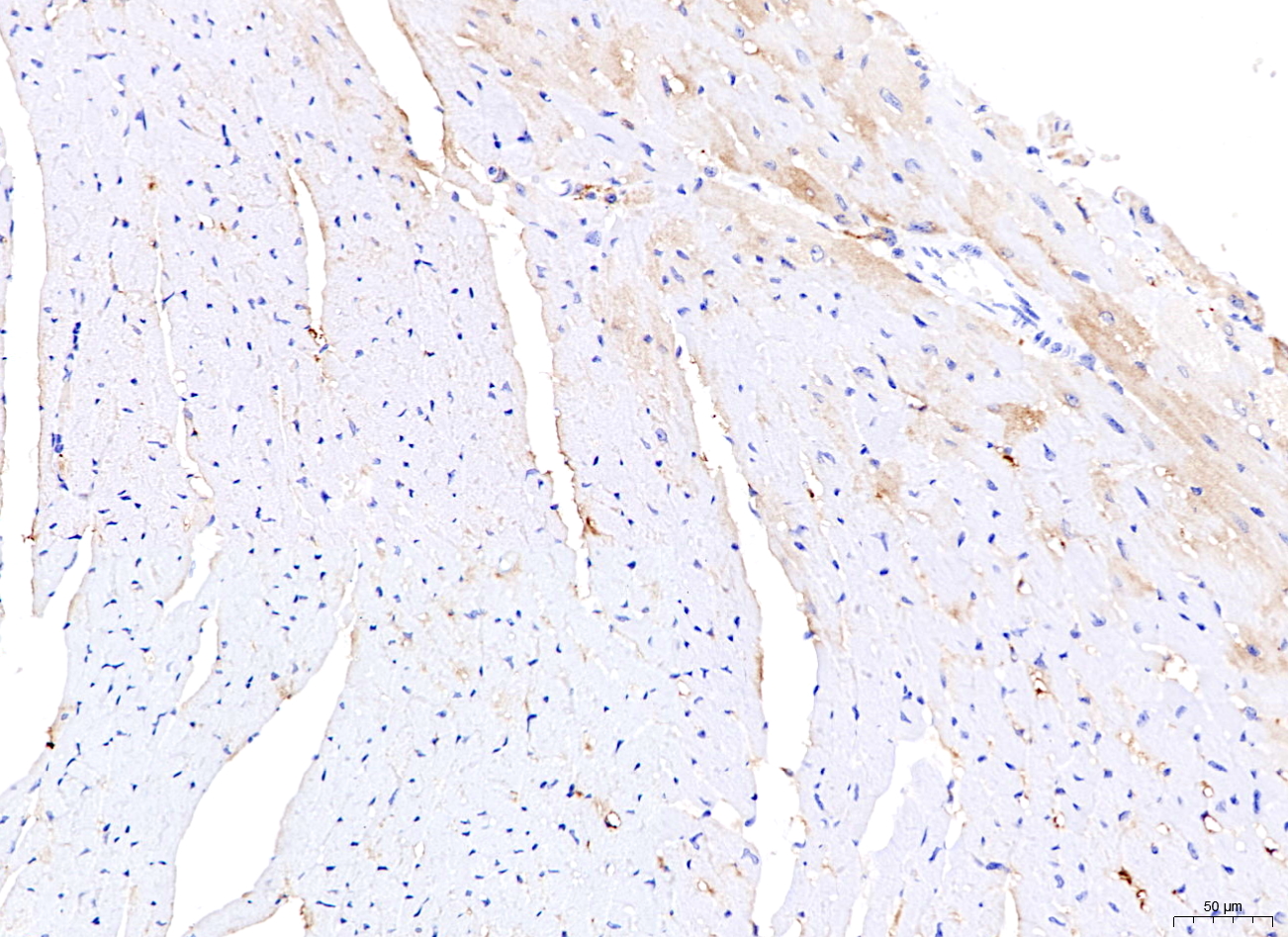

Supplement: Supplementary file 3 — Supporting File 3: advs73867‐sup‐0003‐SupportingFiguresData.zip. [file ADVS-13-e19191-s003.zip › Supporting information Figure S1-S9/S2/Heart/SCRS Control 782_20.0x-3.jpg]

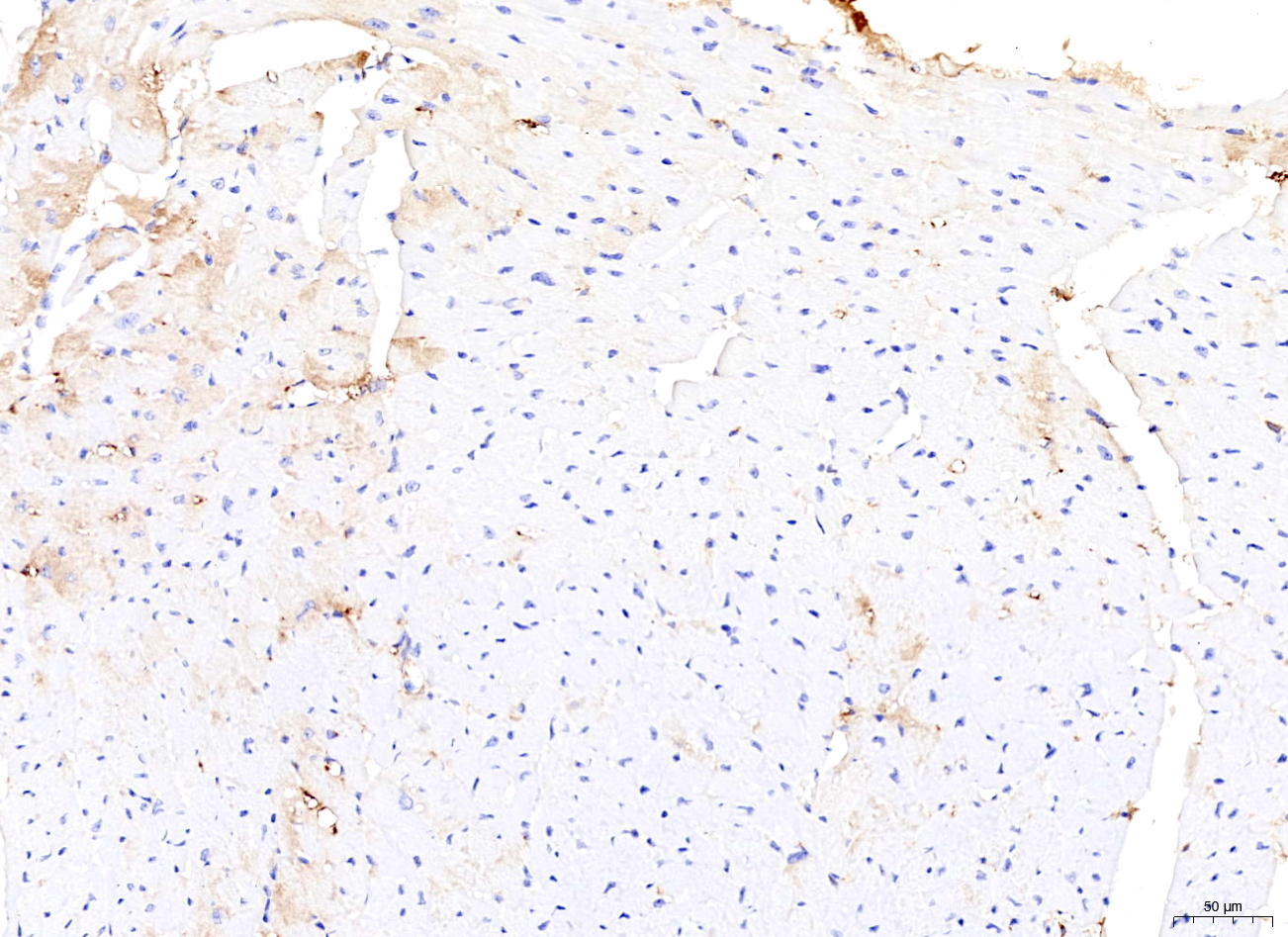

Supplement: Supplementary file 3 — Supporting File 3: advs73867‐sup‐0003‐SupportingFiguresData.zip. [file ADVS-13-e19191-s003.zip › Supporting information Figure S1-S9/S2/Heart/SCRS Control 782_20.0x-4-2.jpg]

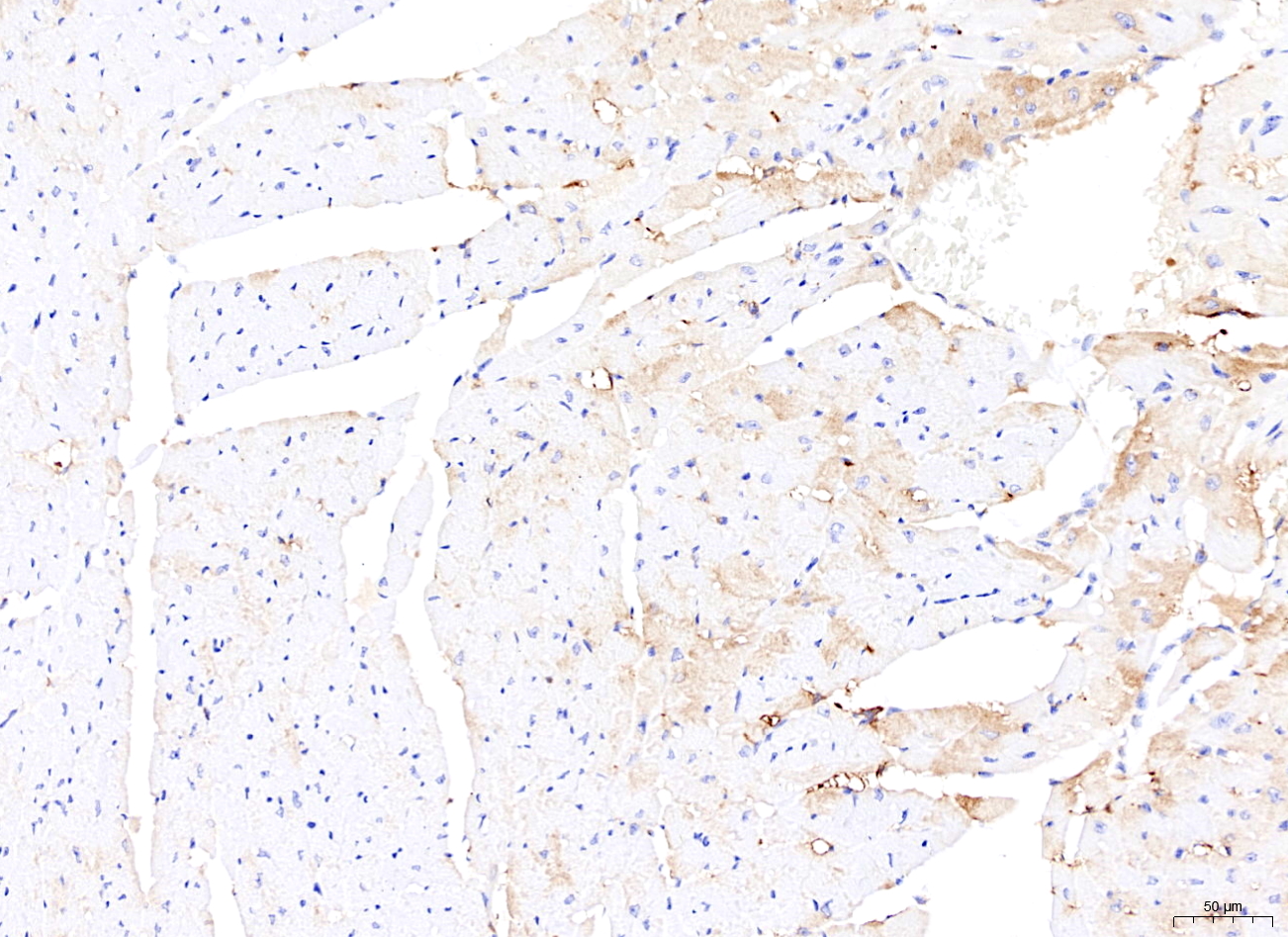

Supplement: Supplementary file 3 — Supporting File 3: advs73867‐sup‐0003‐SupportingFiguresData.zip. [file ADVS-13-e19191-s003.zip › Supporting information Figure S1-S9/S2/Heart/SCRS Control 782_20.0x-5.jpg]

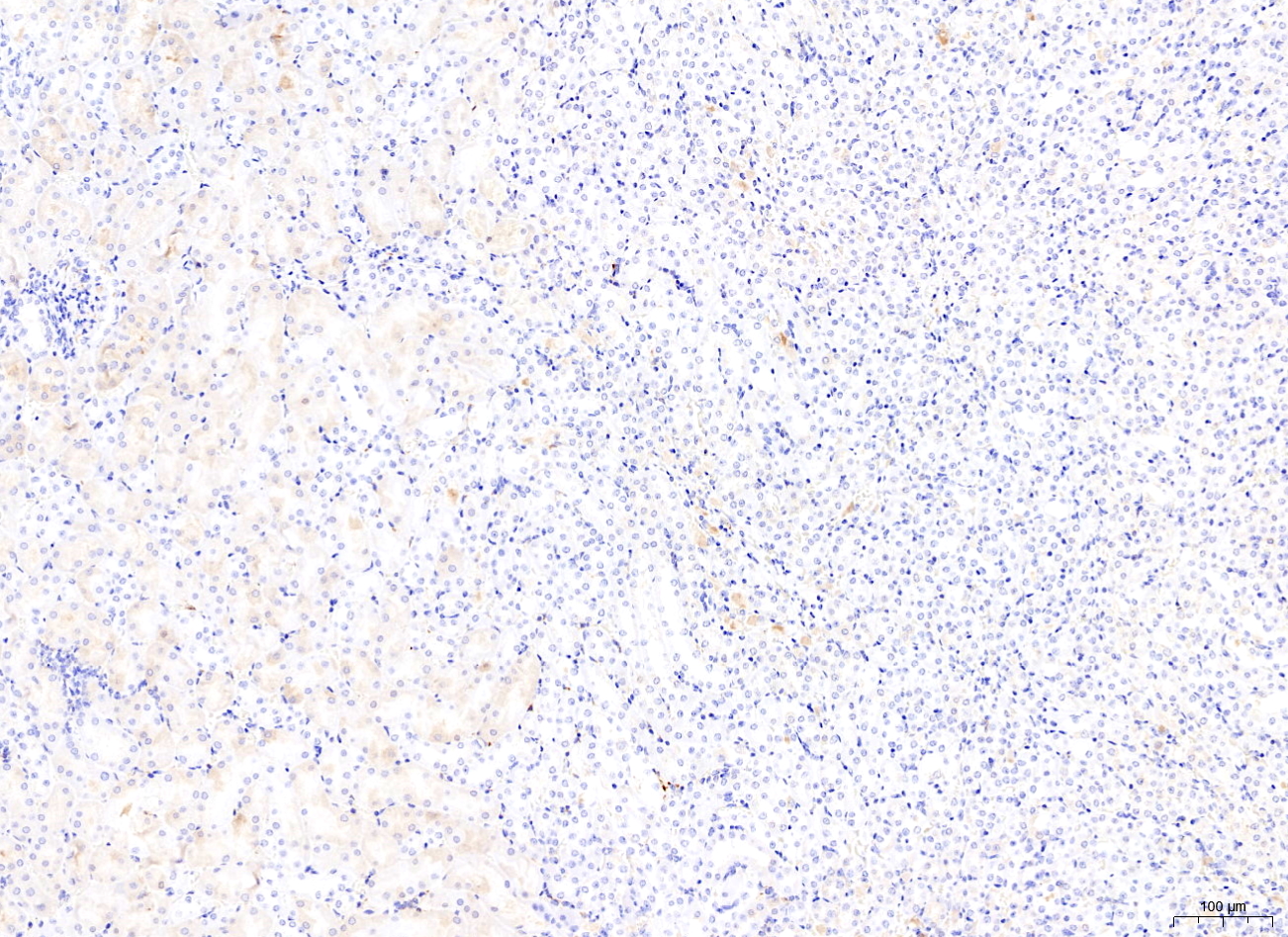

Supplement: Supplementary file 3 — Supporting File 3: advs73867‐sup‐0003‐SupportingFiguresData.zip. [file ADVS-13-e19191-s003.zip › Supporting information Figure S1-S9/S2/Kidney/SCRS 12week Model 784_10.0x.jpg]

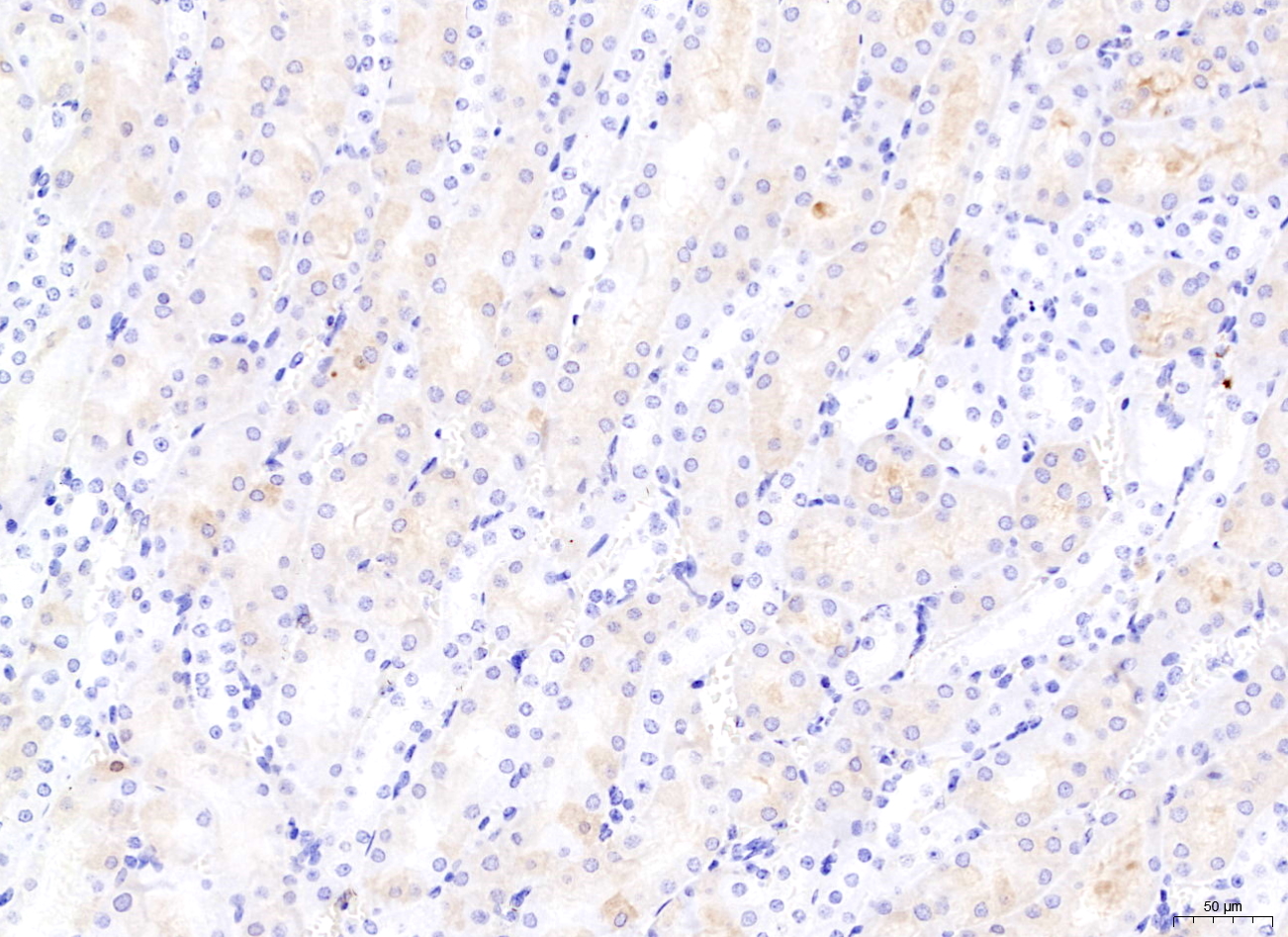

Supplement: Supplementary file 3 — Supporting File 3: advs73867‐sup‐0003‐SupportingFiguresData.zip. [file ADVS-13-e19191-s003.zip › Supporting information Figure S1-S9/S2/Kidney/SCRS 12week Model 784_20.0x-1.jpg]

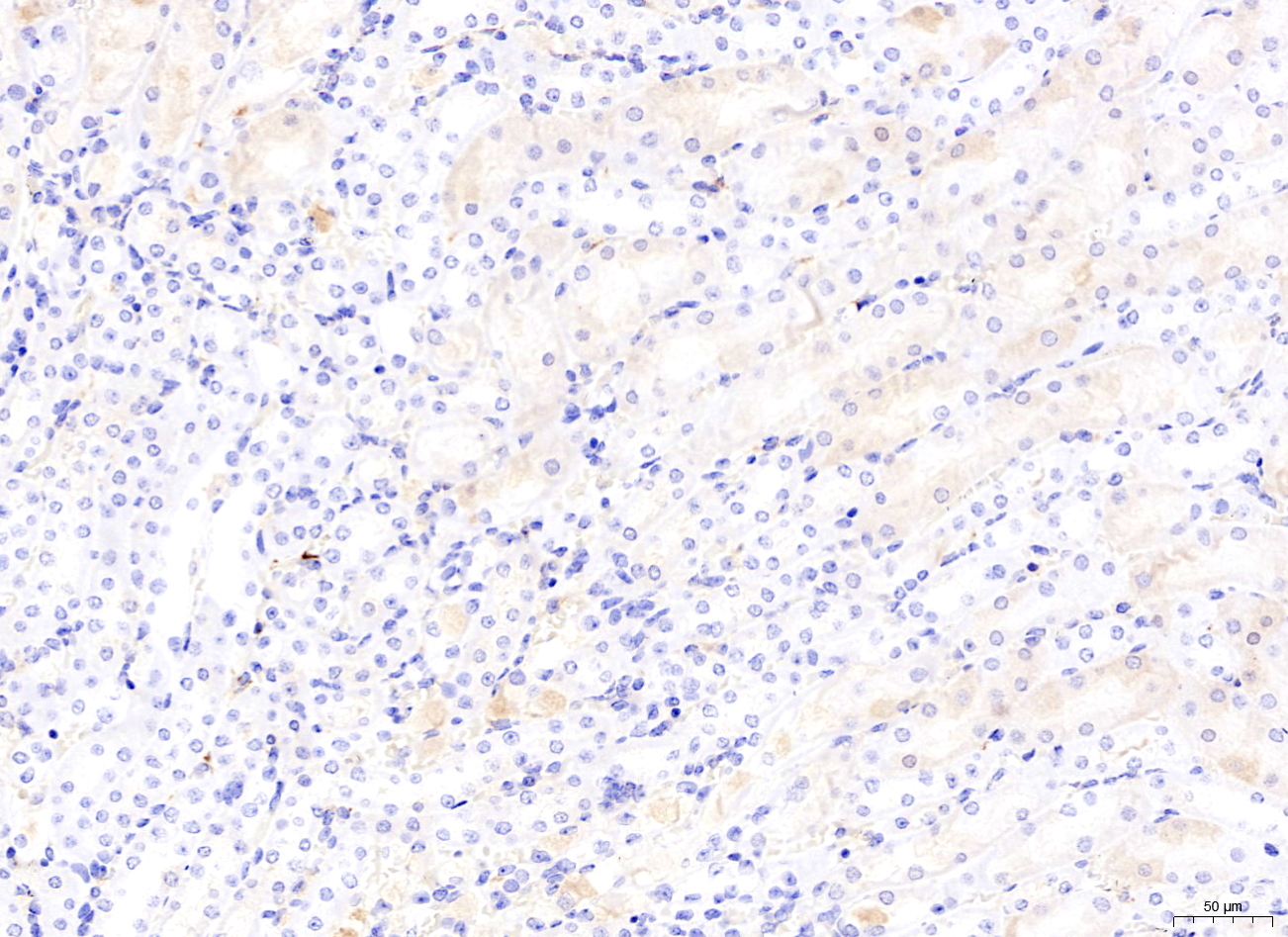

Supplement: Supplementary file 3 — Supporting File 3: advs73867‐sup‐0003‐SupportingFiguresData.zip. [file ADVS-13-e19191-s003.zip › Supporting information Figure S1-S9/S2/Kidney/SCRS 12week Model 784_20.0x-2.jpg]

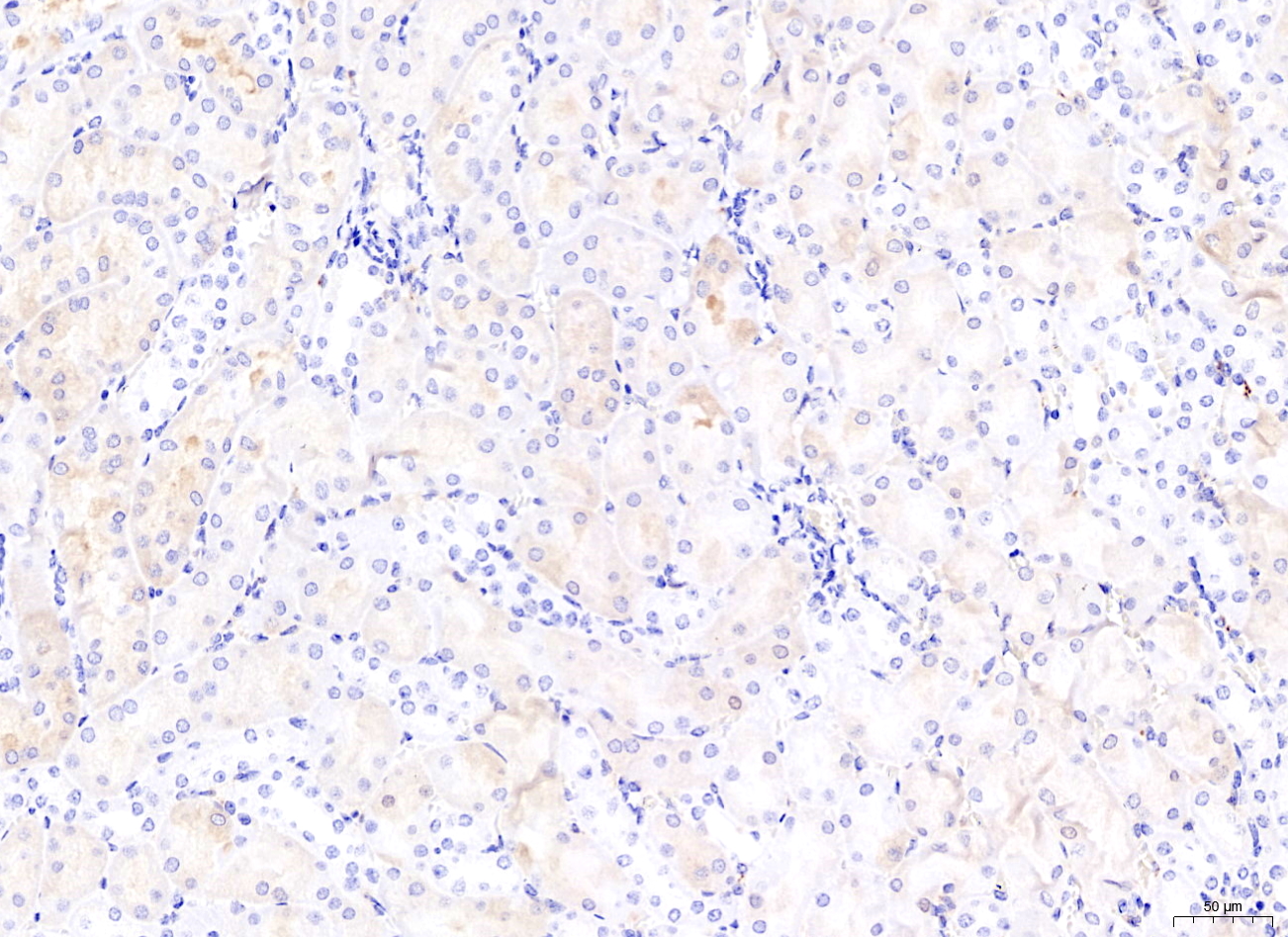

Supplement: Supplementary file 3 — Supporting File 3: advs73867‐sup‐0003‐SupportingFiguresData.zip. [file ADVS-13-e19191-s003.zip › Supporting information Figure S1-S9/S2/Kidney/SCRS 12week Model 784_20.0x-3.jpg]

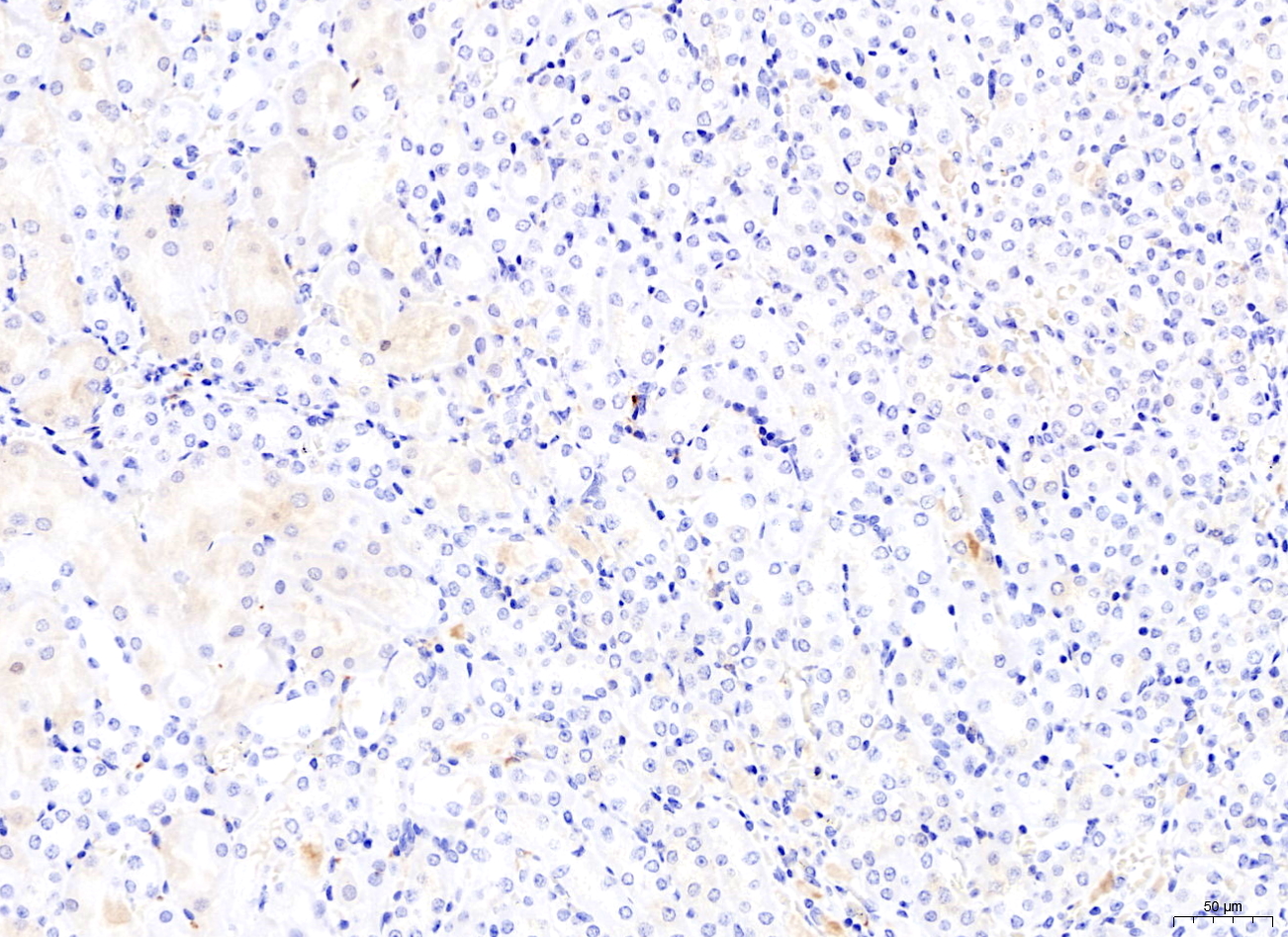

Supplement: Supplementary file 3 — Supporting File 3: advs73867‐sup‐0003‐SupportingFiguresData.zip. [file ADVS-13-e19191-s003.zip › Supporting information Figure S1-S9/S2/Kidney/SCRS 12week Model 784_20.0x-4.jpg]

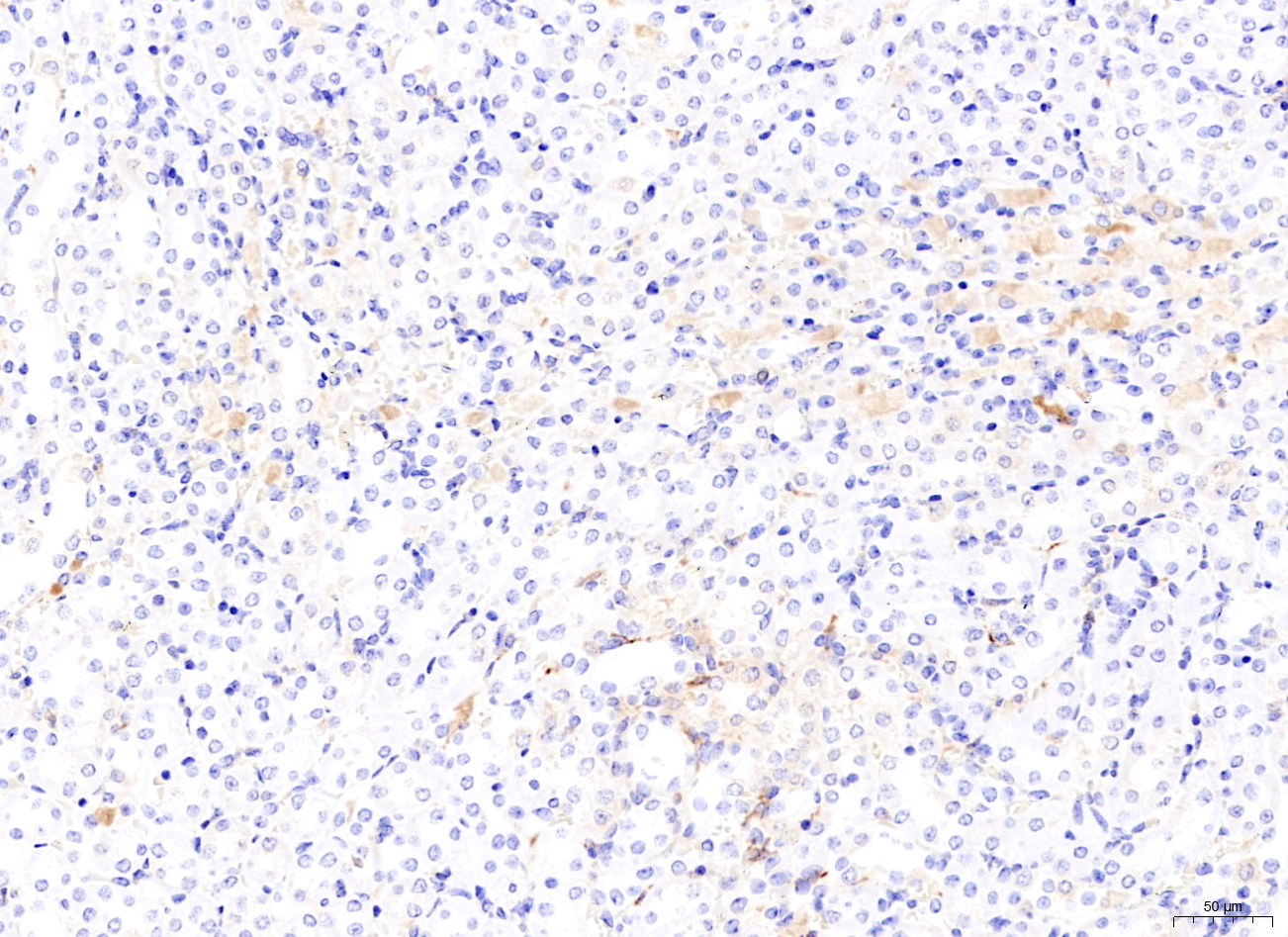

Supplement: Supplementary file 3 — Supporting File 3: advs73867‐sup‐0003‐SupportingFiguresData.zip. [file ADVS-13-e19191-s003.zip › Supporting information Figure S1-S9/S2/Kidney/SCRS 12week Model 784_20.0x-5.jpg]

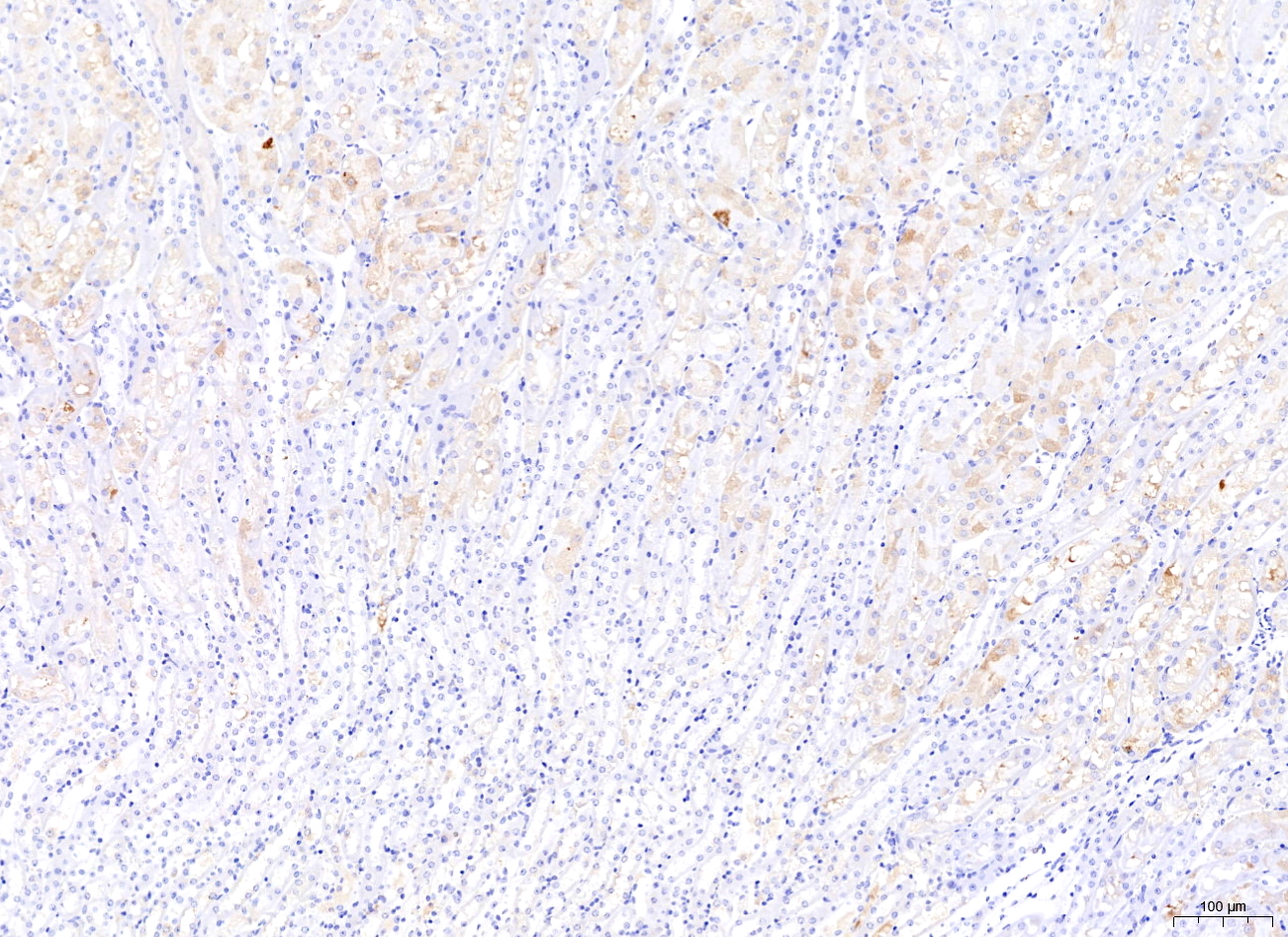

Supplement: Supplementary file 3 — Supporting File 3: advs73867‐sup‐0003‐SupportingFiguresData.zip. [file ADVS-13-e19191-s003.zip › Supporting information Figure S1-S9/S2/Kidney/SCRS 1week Model 757_10.0x.jpg]

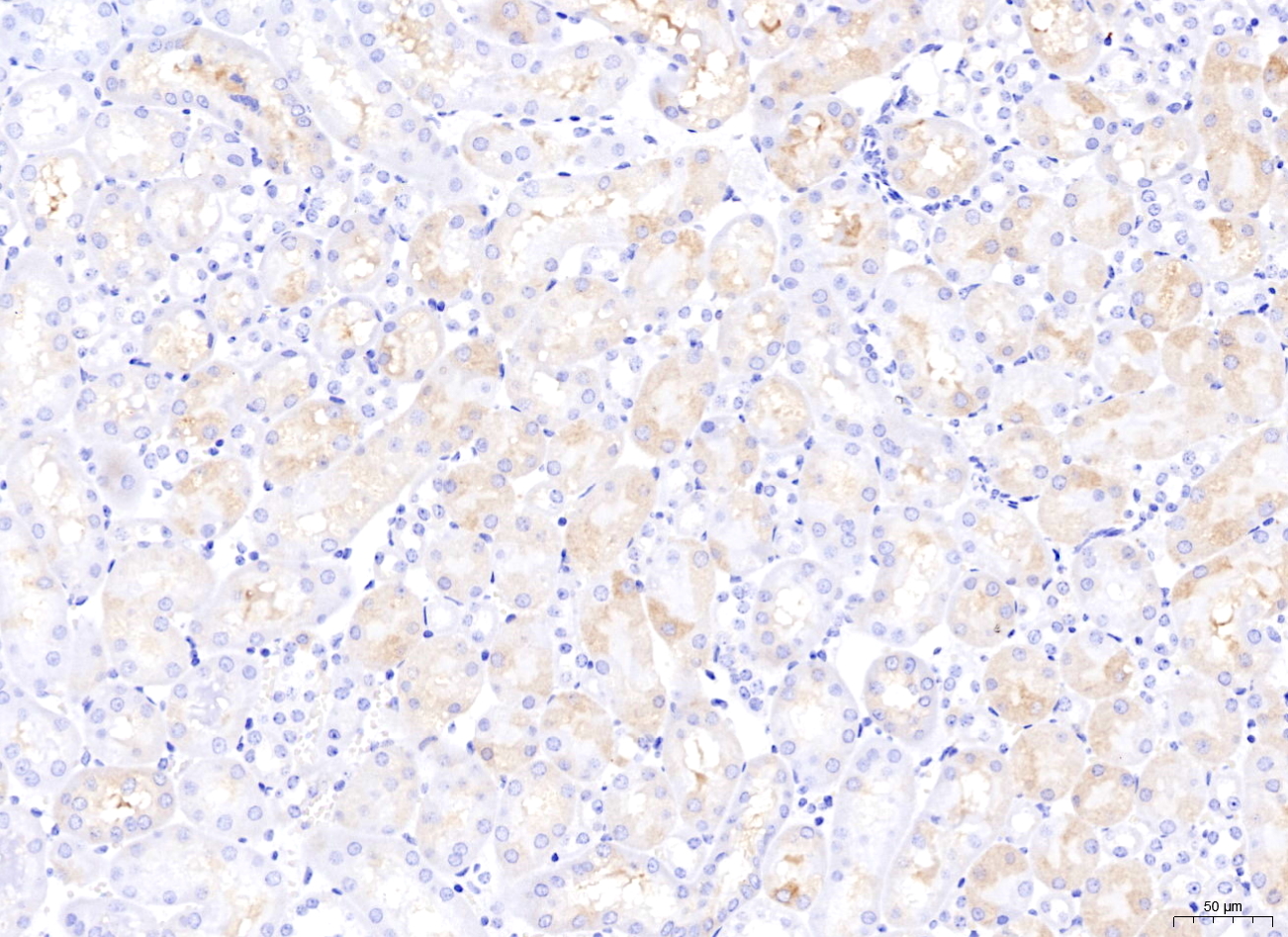

Supplement: Supplementary file 3 — Supporting File 3: advs73867‐sup‐0003‐SupportingFiguresData.zip. [file ADVS-13-e19191-s003.zip › Supporting information Figure S1-S9/S2/Kidney/SCRS 1week Model 757_20.0x-1.jpg]

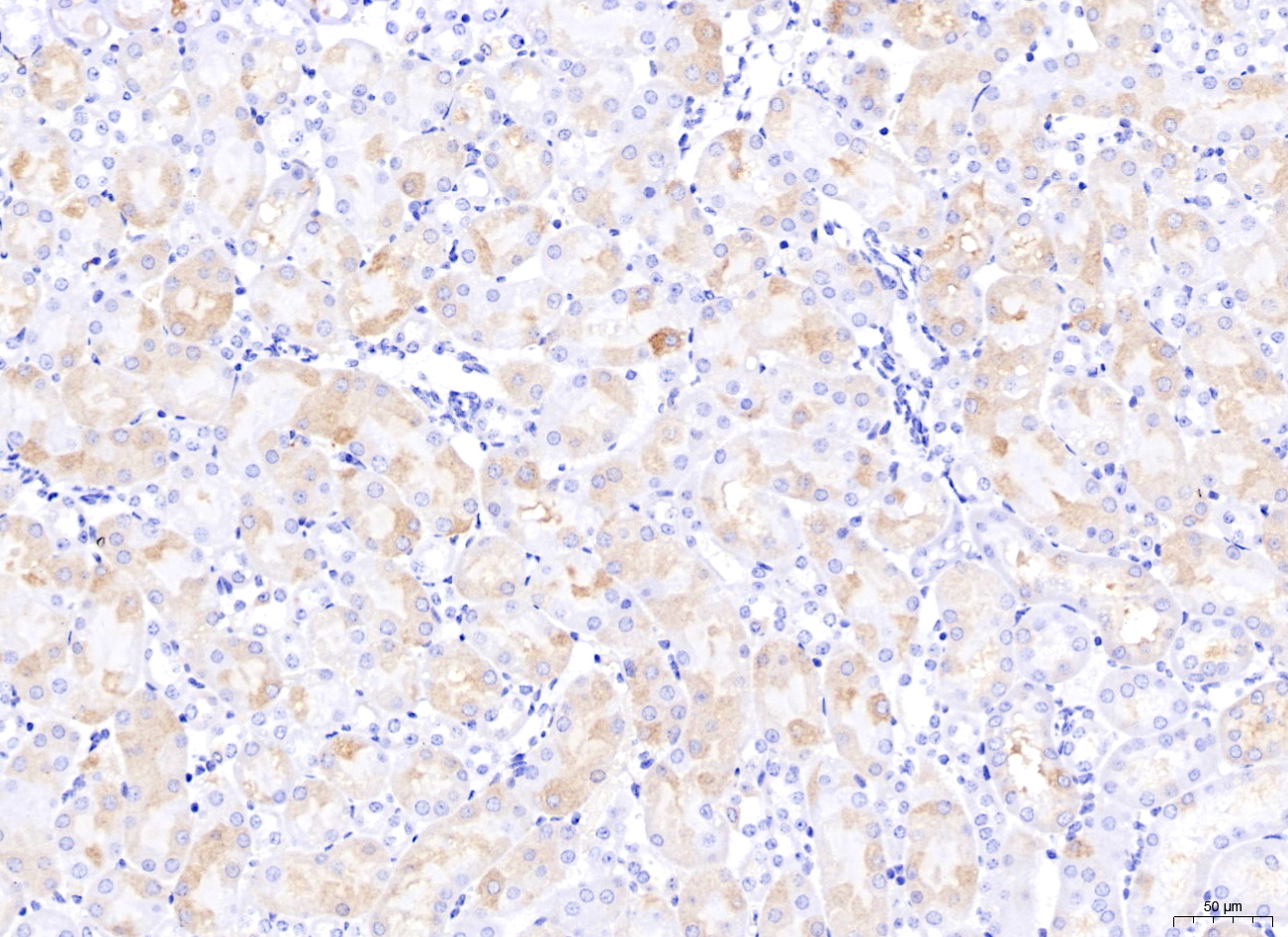

Supplement: Supplementary file 3 — Supporting File 3: advs73867‐sup‐0003‐SupportingFiguresData.zip. [file ADVS-13-e19191-s003.zip › Supporting information Figure S1-S9/S2/Kidney/SCRS 1week Model 757_20.0x-2.jpg]

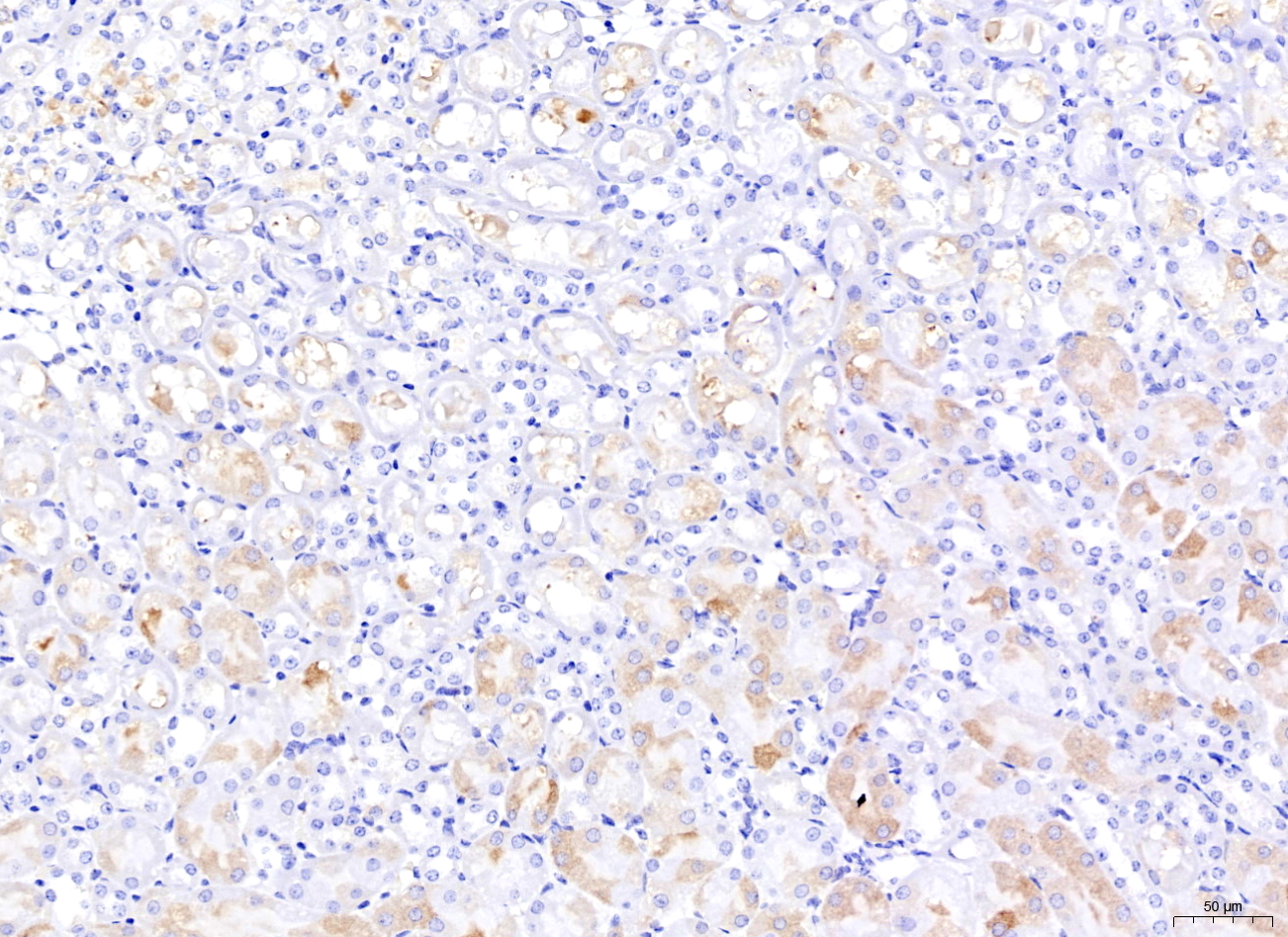

Supplement: Supplementary file 3 — Supporting File 3: advs73867‐sup‐0003‐SupportingFiguresData.zip. [file ADVS-13-e19191-s003.zip › Supporting information Figure S1-S9/S2/Kidney/SCRS 1week Model 757_20.0x-3.jpg]

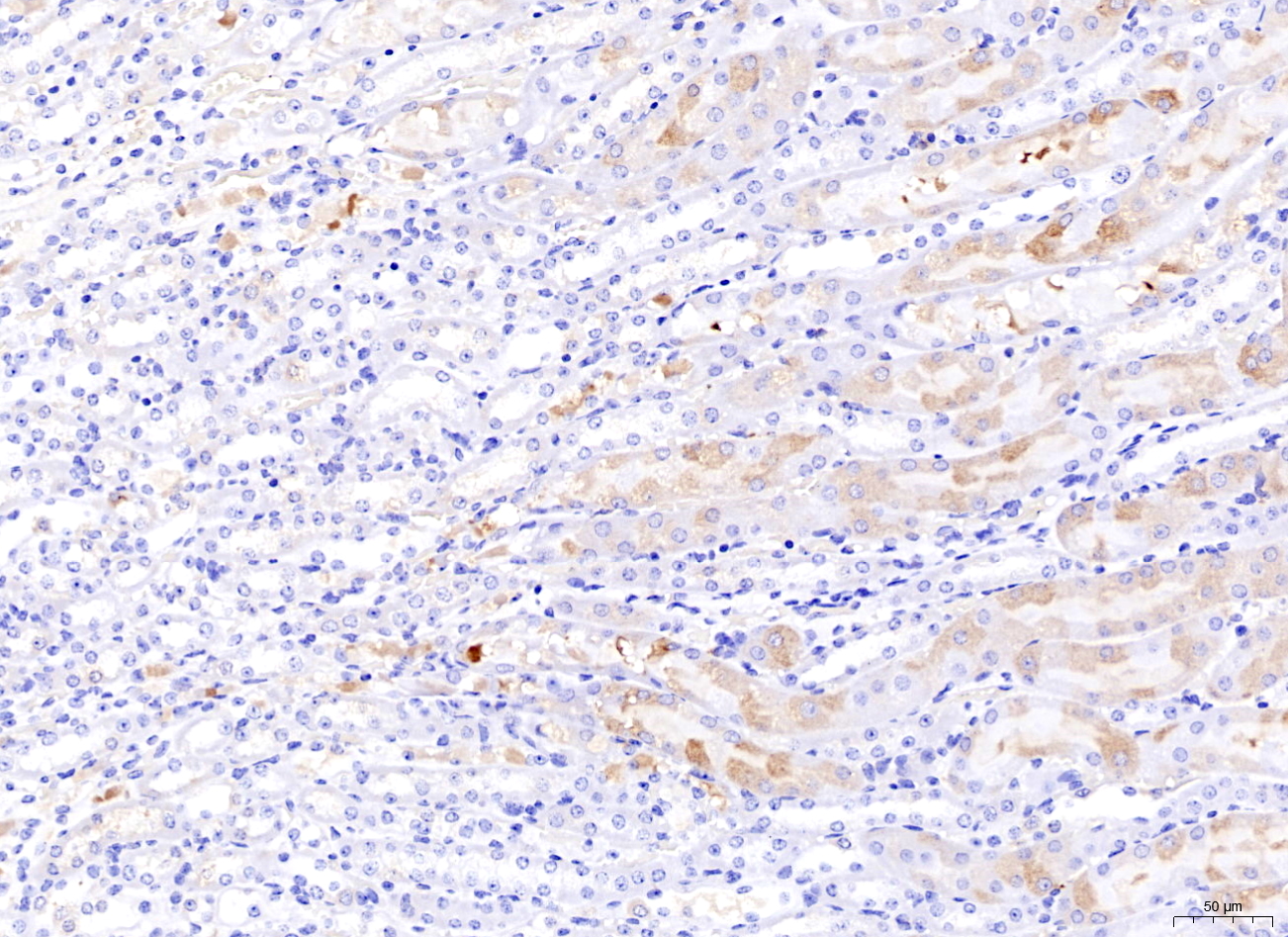

Supplement: Supplementary file 3 — Supporting File 3: advs73867‐sup‐0003‐SupportingFiguresData.zip. [file ADVS-13-e19191-s003.zip › Supporting information Figure S1-S9/S2/Kidney/SCRS 1week Model 757_20.0x-4.jpg]

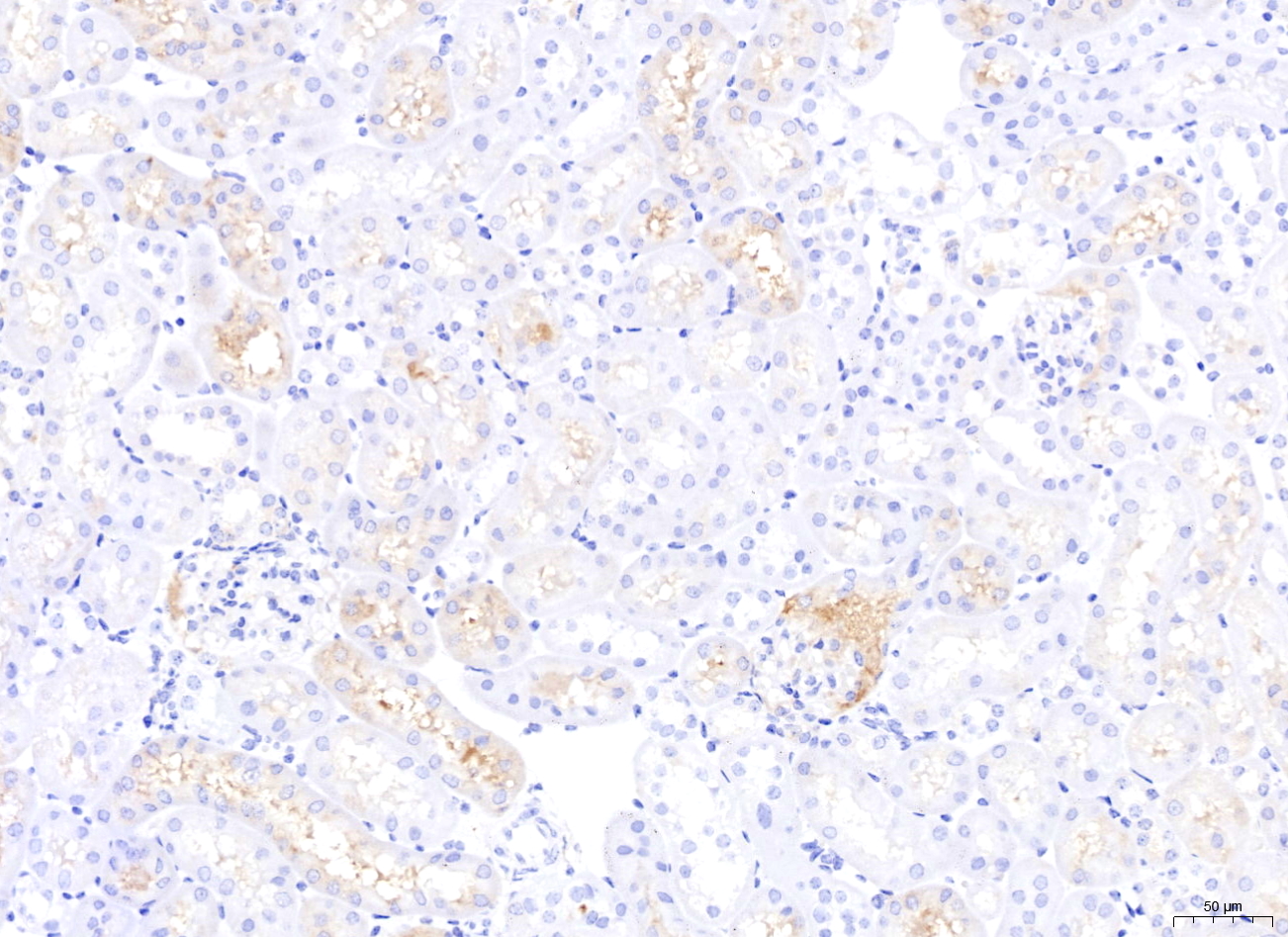

Supplement: Supplementary file 3 — Supporting File 3: advs73867‐sup‐0003‐SupportingFiguresData.zip. [file ADVS-13-e19191-s003.zip › Supporting information Figure S1-S9/S2/Kidney/SCRS 1week Model 757_20.0x-5.jpg]

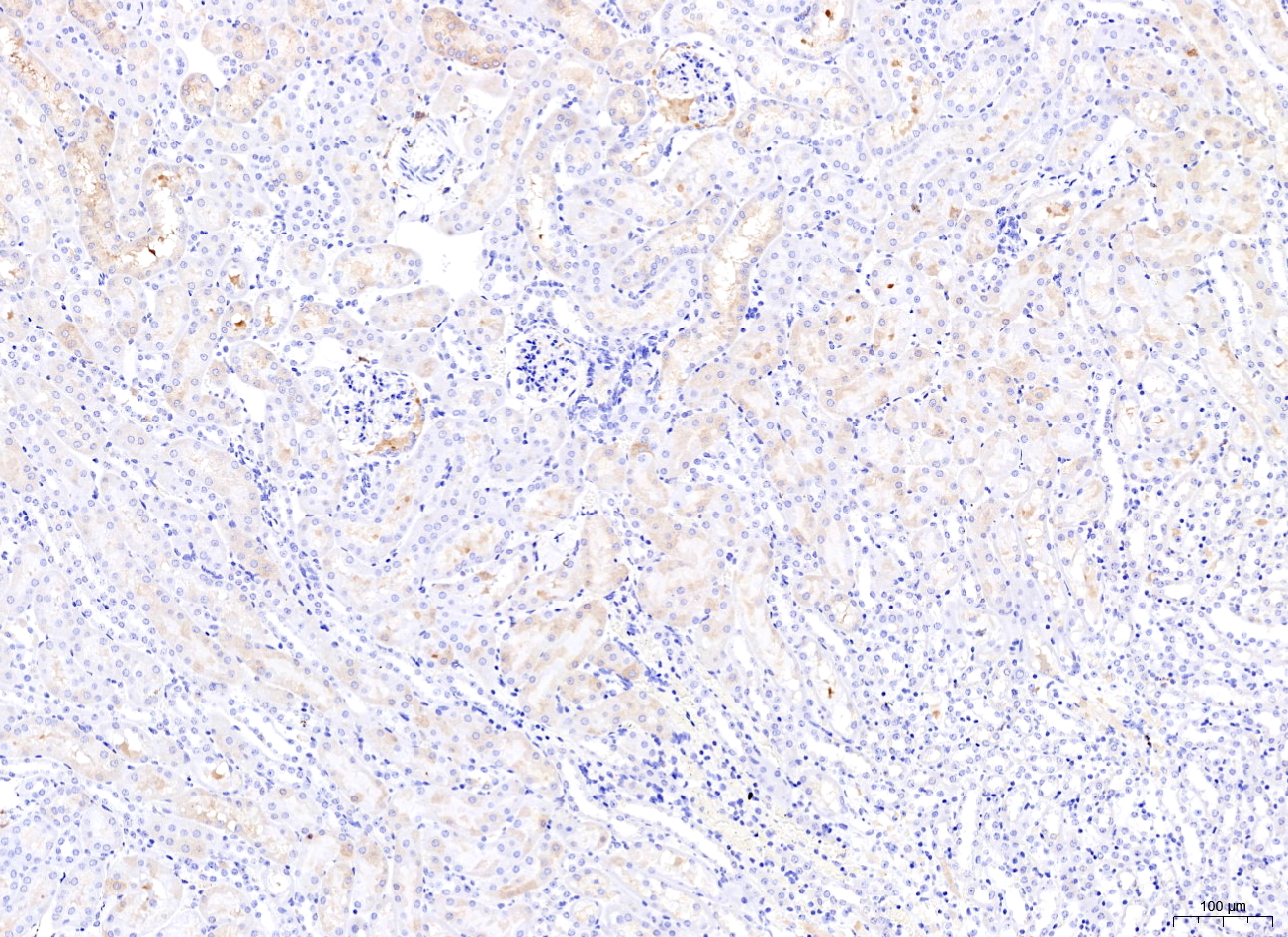

Supplement: Supplementary file 3 — Supporting File 3: advs73867‐sup‐0003‐SupportingFiguresData.zip. [file ADVS-13-e19191-s003.zip › Supporting information Figure S1-S9/S2/Kidney/SCRS 4week Model 744_10.0x.jpg]

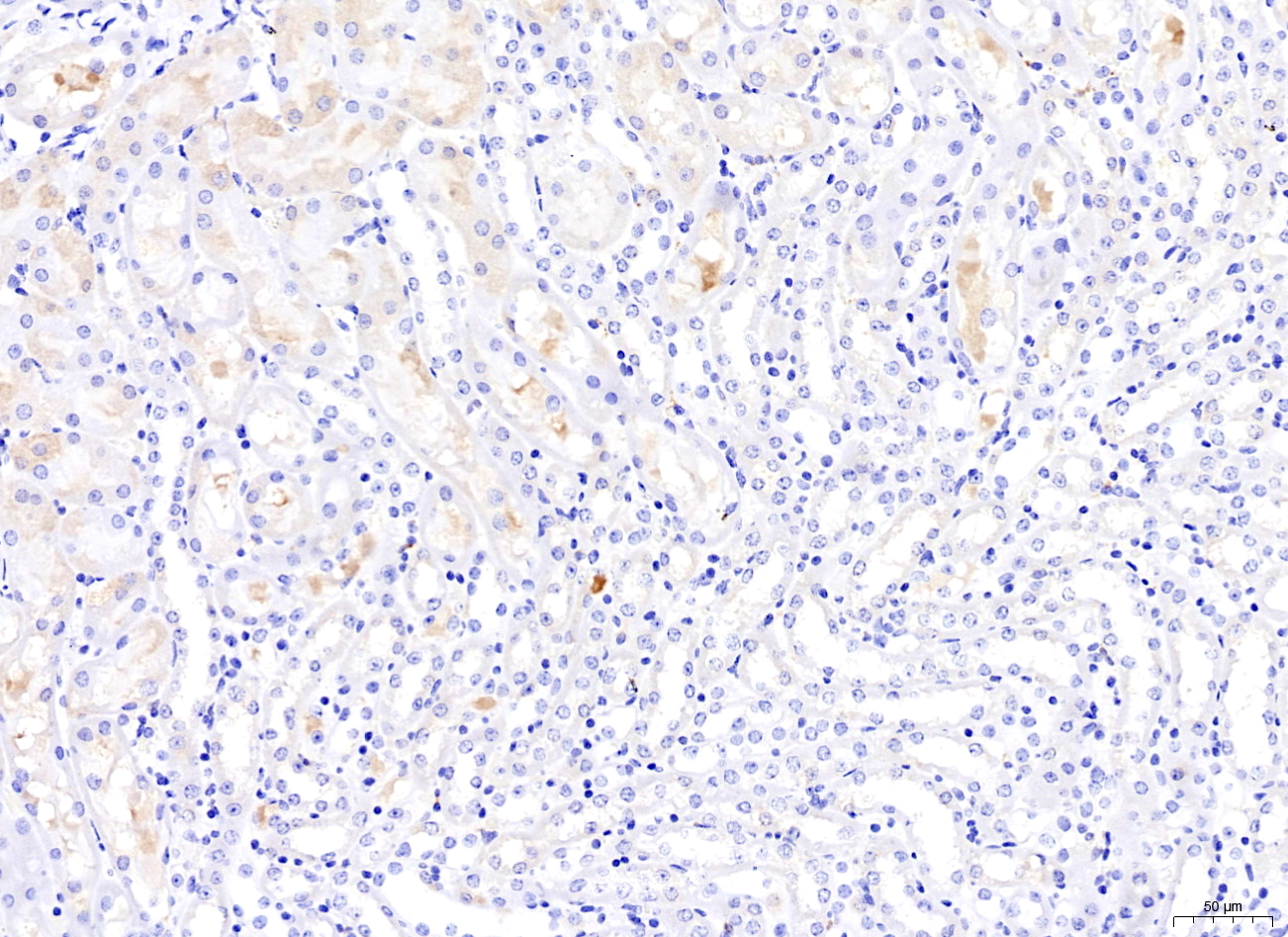

Supplement: Supplementary file 3 — Supporting File 3: advs73867‐sup‐0003‐SupportingFiguresData.zip. [file ADVS-13-e19191-s003.zip › Supporting information Figure S1-S9/S2/Kidney/SCRS 4week Model 744_20.0x-1.jpg]

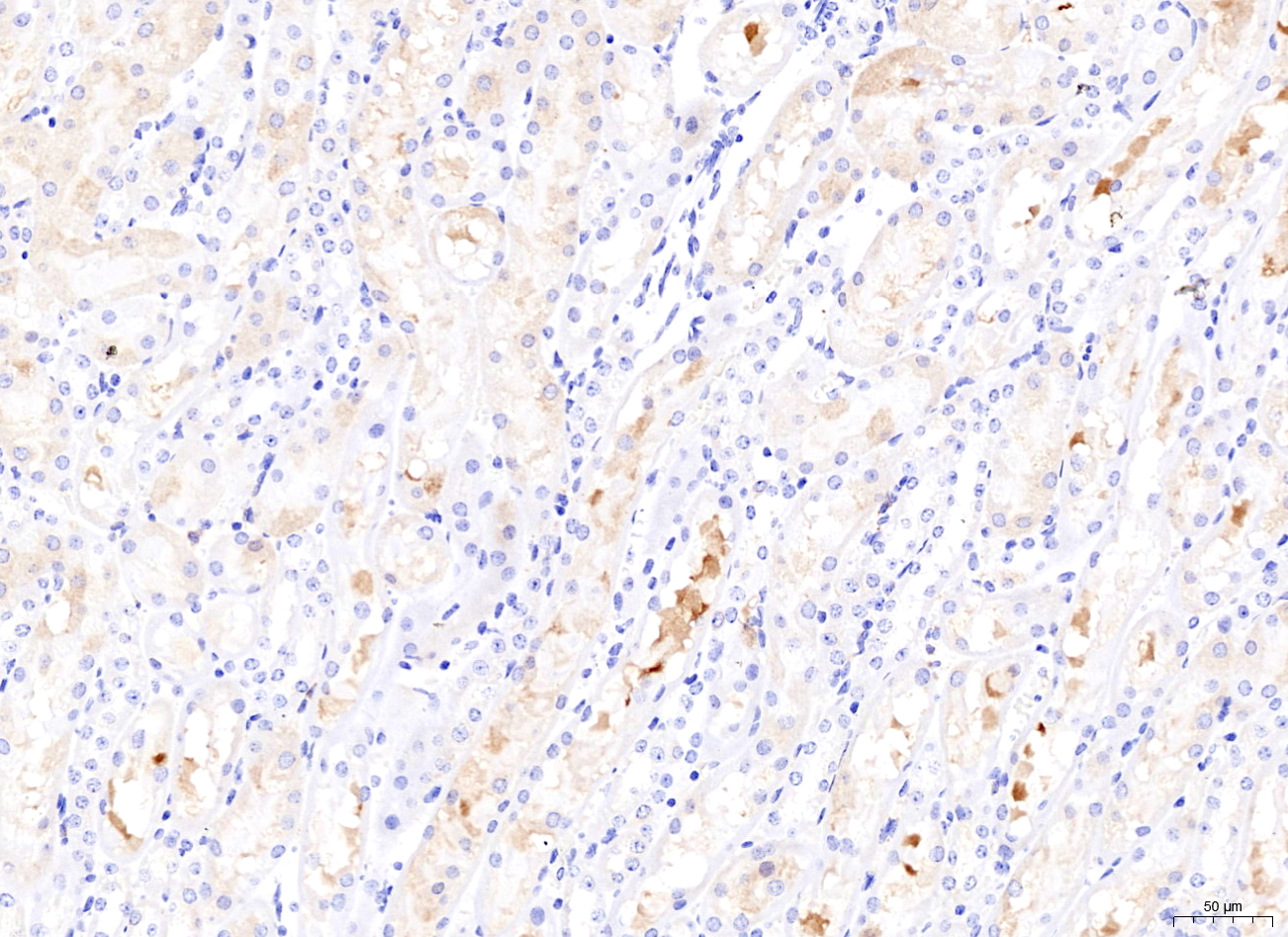

Supplement: Supplementary file 3 — Supporting File 3: advs73867‐sup‐0003‐SupportingFiguresData.zip. [file ADVS-13-e19191-s003.zip › Supporting information Figure S1-S9/S2/Kidney/SCRS 4week Model 744_20.0x-2.jpg]

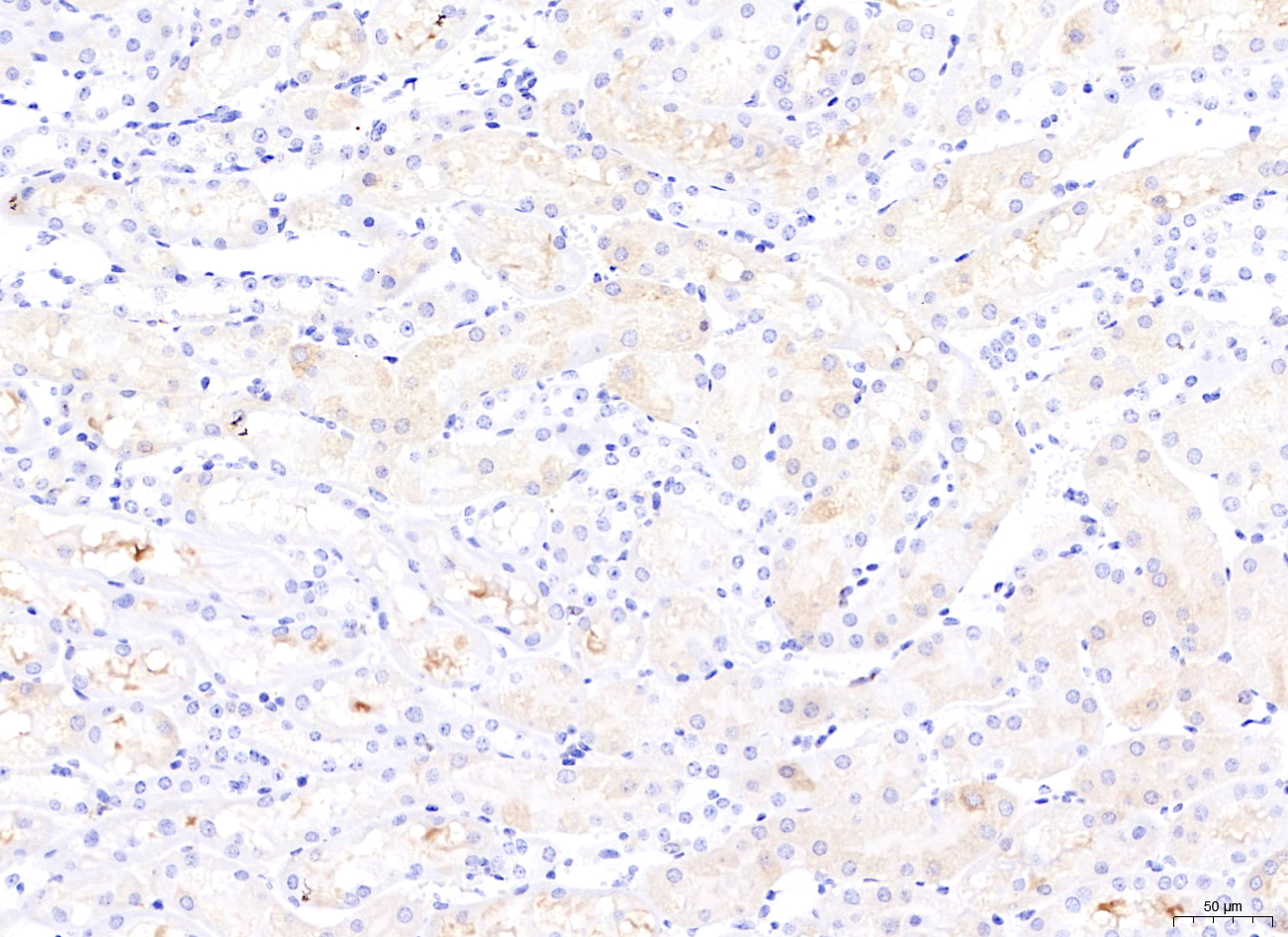

Supplement: Supplementary file 3 — Supporting File 3: advs73867‐sup‐0003‐SupportingFiguresData.zip. [file ADVS-13-e19191-s003.zip › Supporting information Figure S1-S9/S2/Kidney/SCRS 4week Model 744_20.0x-3.jpg]

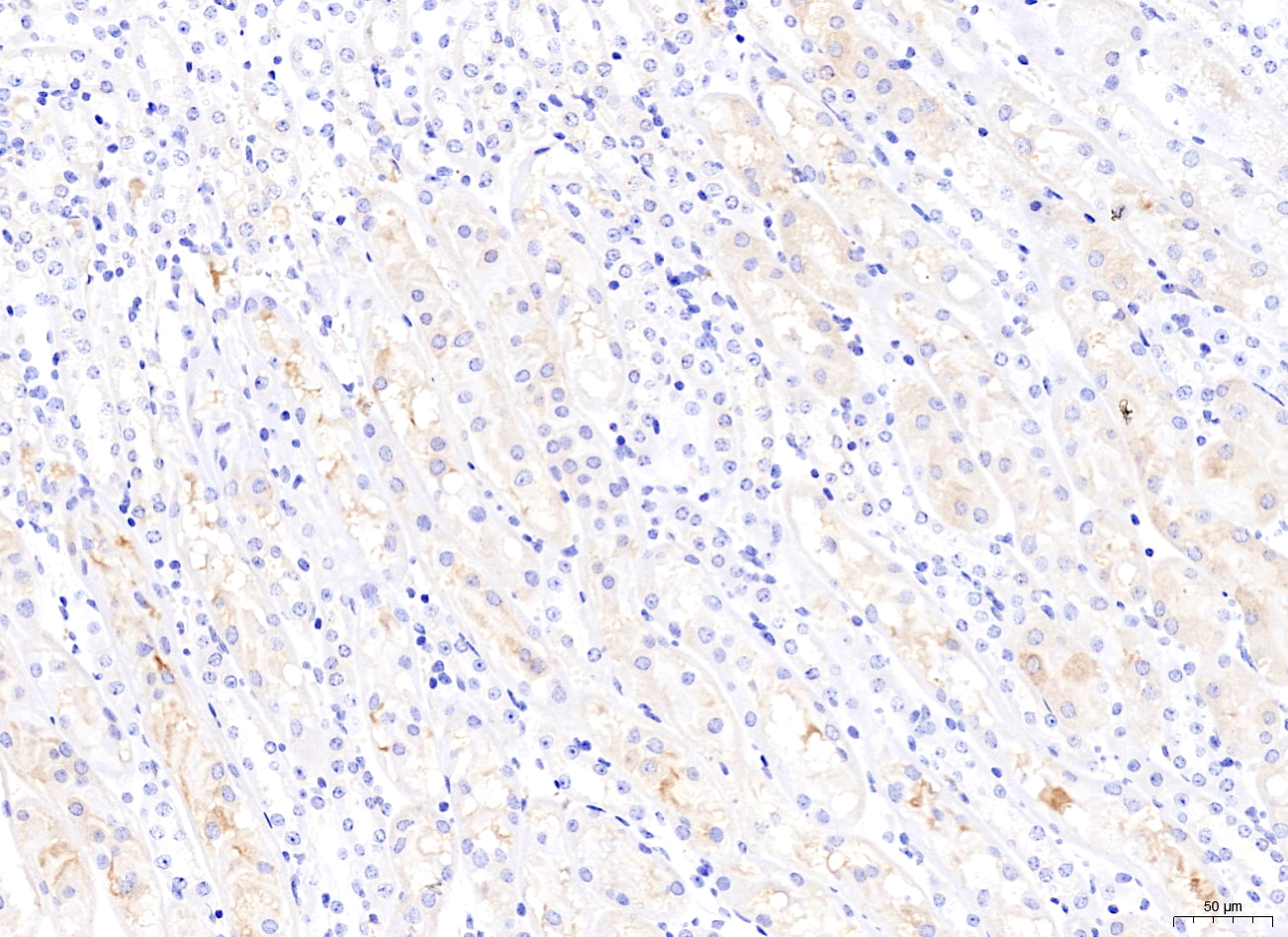

Supplement: Supplementary file 3 — Supporting File 3: advs73867‐sup‐0003‐SupportingFiguresData.zip. [file ADVS-13-e19191-s003.zip › Supporting information Figure S1-S9/S2/Kidney/SCRS 4week Model 744_20.0x-4.jpg]

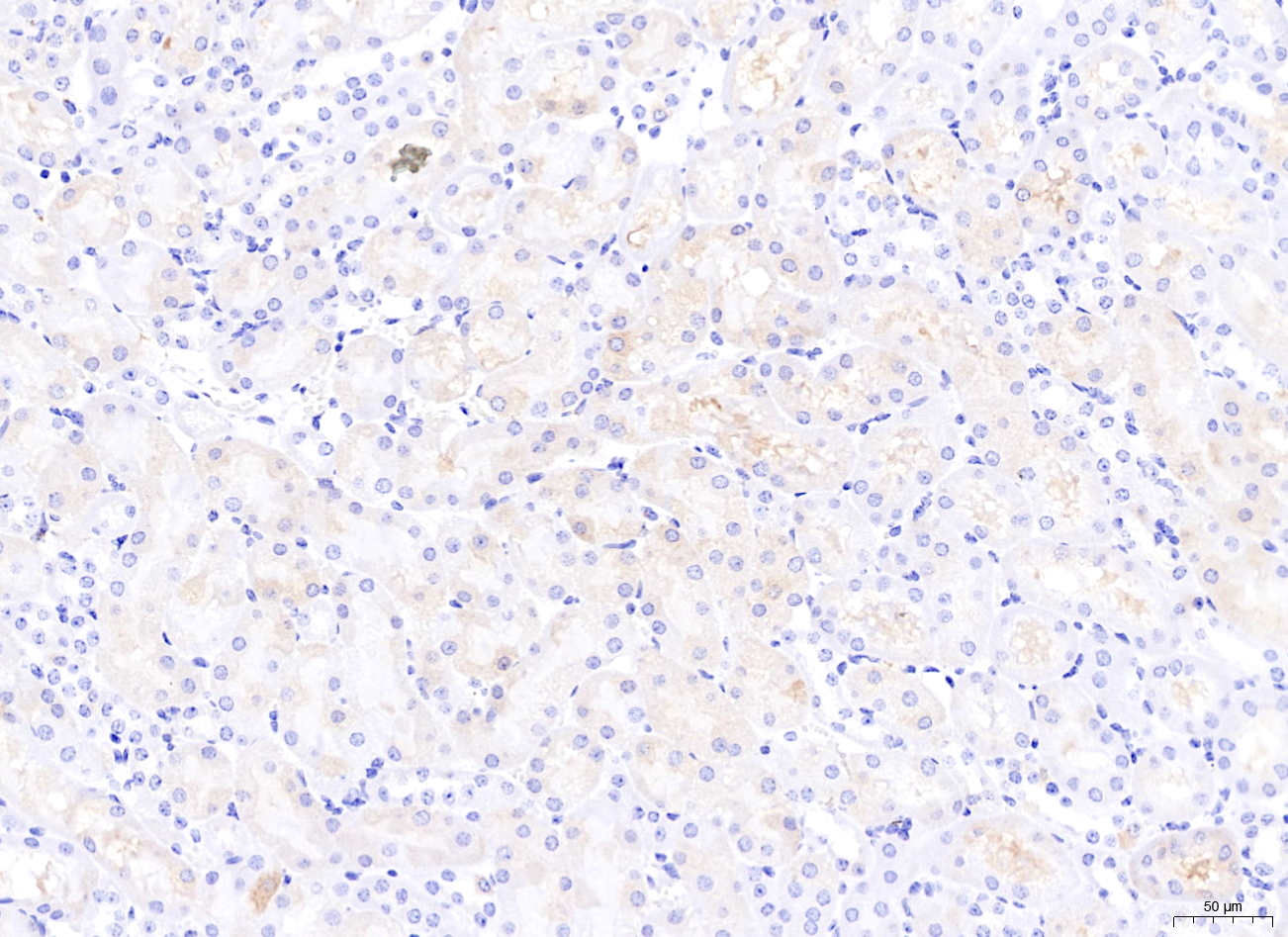

Supplement: Supplementary file 3 — Supporting File 3: advs73867‐sup‐0003‐SupportingFiguresData.zip. [file ADVS-13-e19191-s003.zip › Supporting information Figure S1-S9/S2/Kidney/SCRS 4week Model 744_20.0x-5.jpg]

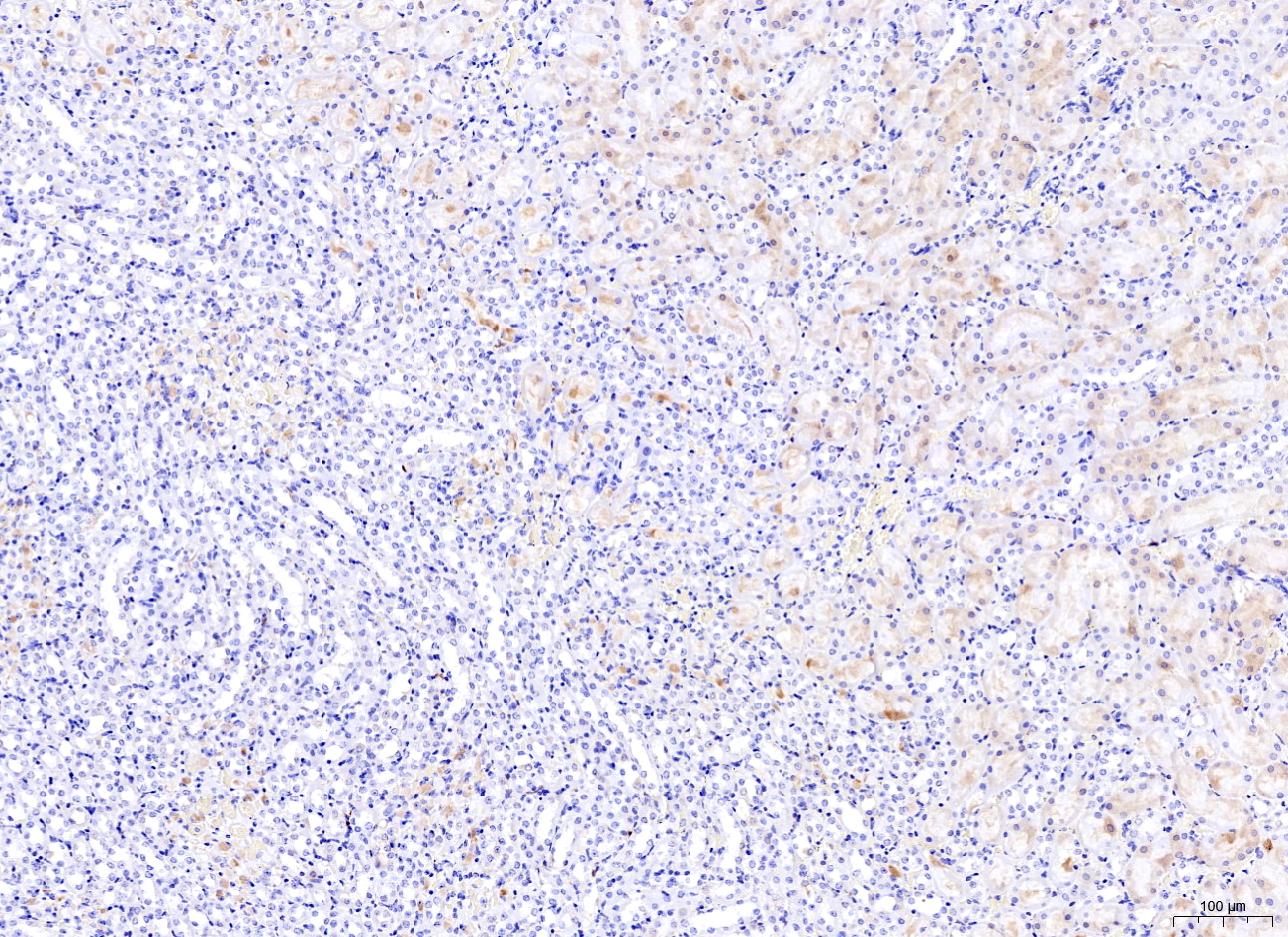

Supplement: Supplementary file 3 — Supporting File 3: advs73867‐sup‐0003‐SupportingFiguresData.zip. [file ADVS-13-e19191-s003.zip › Supporting information Figure S1-S9/S2/Kidney/SCRS 8week Model 763_10.0x.jpg]

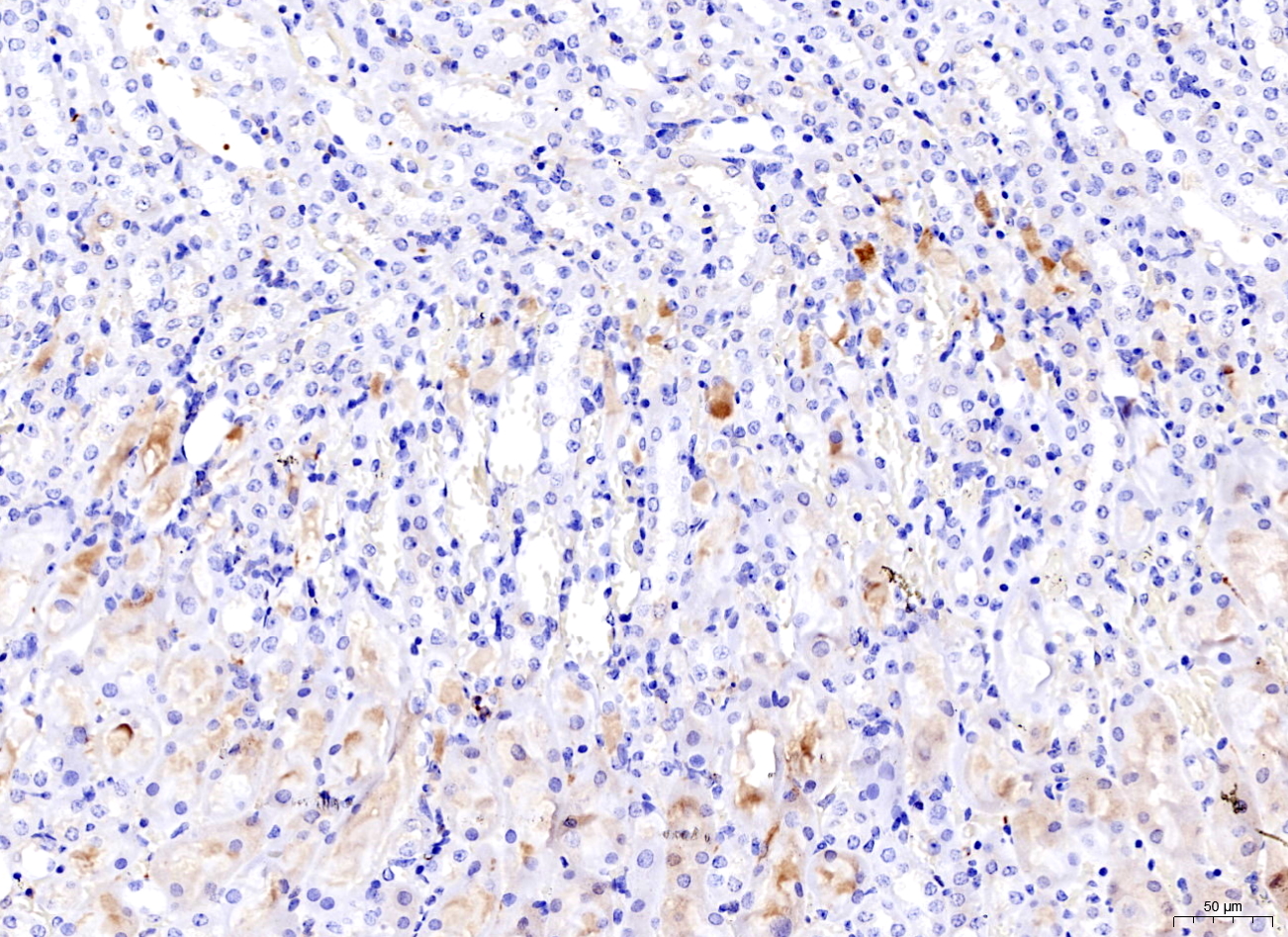

Supplement: Supplementary file 3 — Supporting File 3: advs73867‐sup‐0003‐SupportingFiguresData.zip. [file ADVS-13-e19191-s003.zip › Supporting information Figure S1-S9/S2/Kidney/SCRS 8week Model 763_20.0x-1.jpg]

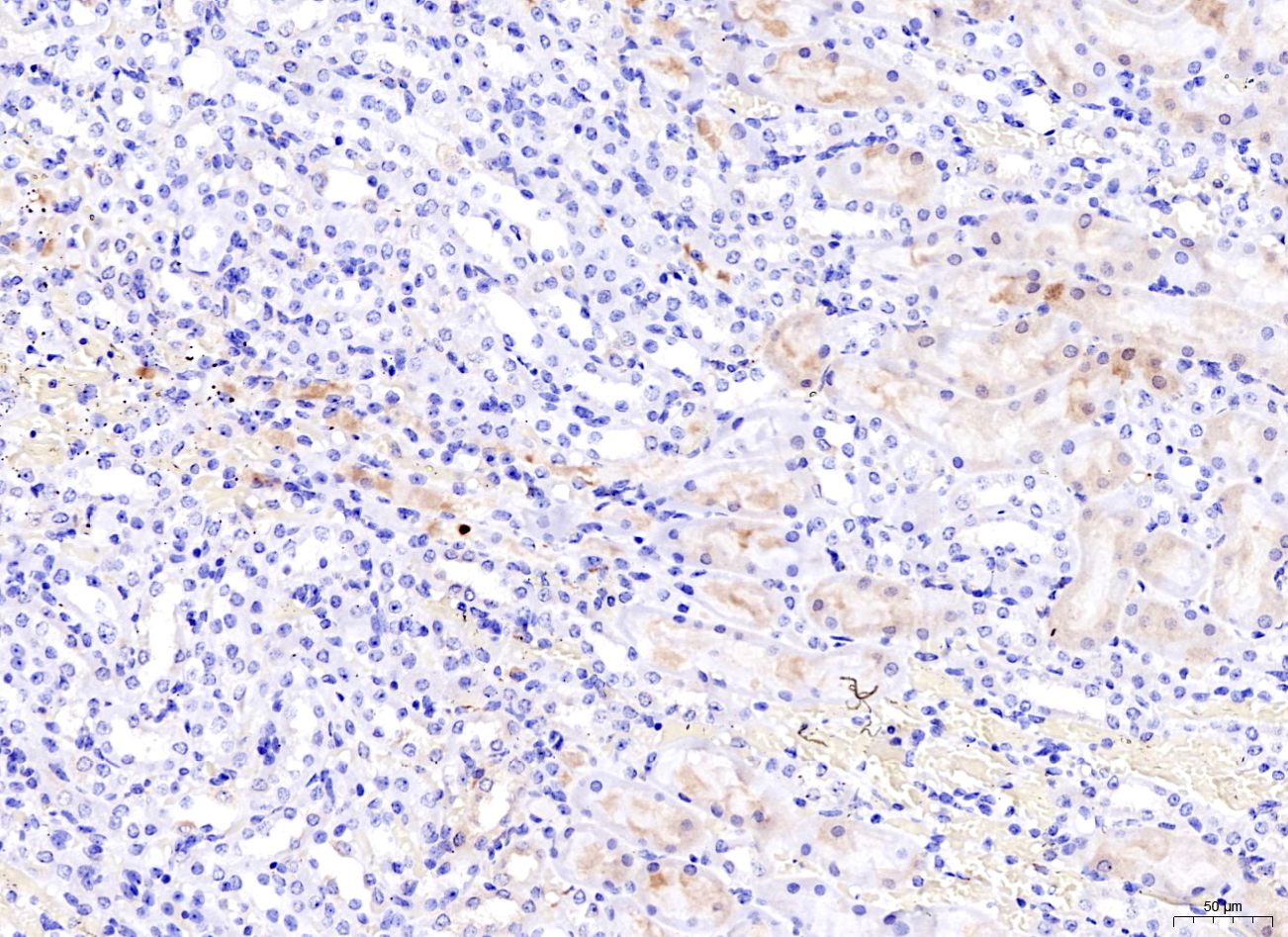

Supplement: Supplementary file 3 — Supporting File 3: advs73867‐sup‐0003‐SupportingFiguresData.zip. [file ADVS-13-e19191-s003.zip › Supporting information Figure S1-S9/S2/Kidney/SCRS 8week Model 763_20.0x-2.jpg]

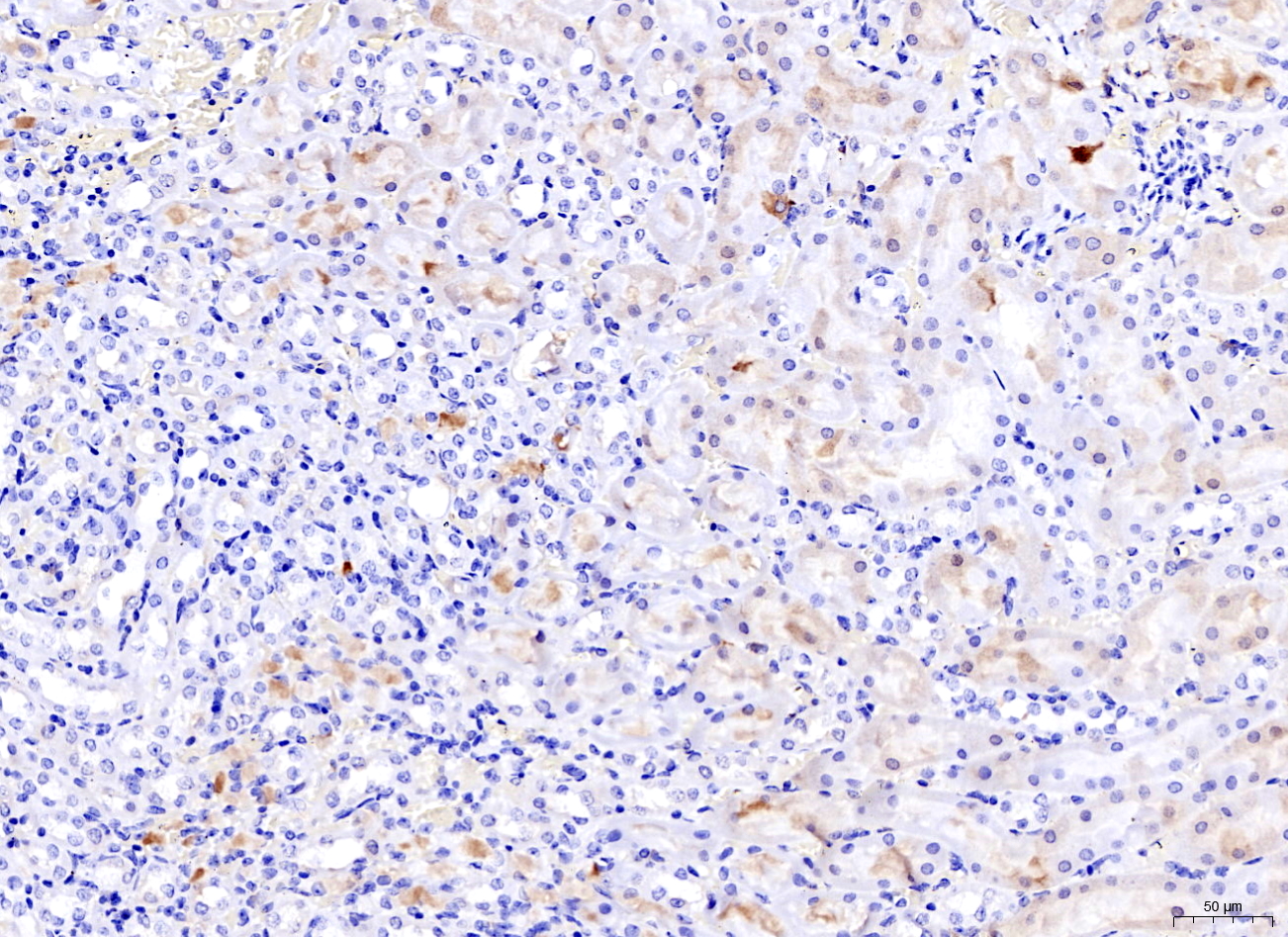

Supplement: Supplementary file 3 — Supporting File 3: advs73867‐sup‐0003‐SupportingFiguresData.zip. [file ADVS-13-e19191-s003.zip › Supporting information Figure S1-S9/S2/Kidney/SCRS 8week Model 763_20.0x-3.jpg]

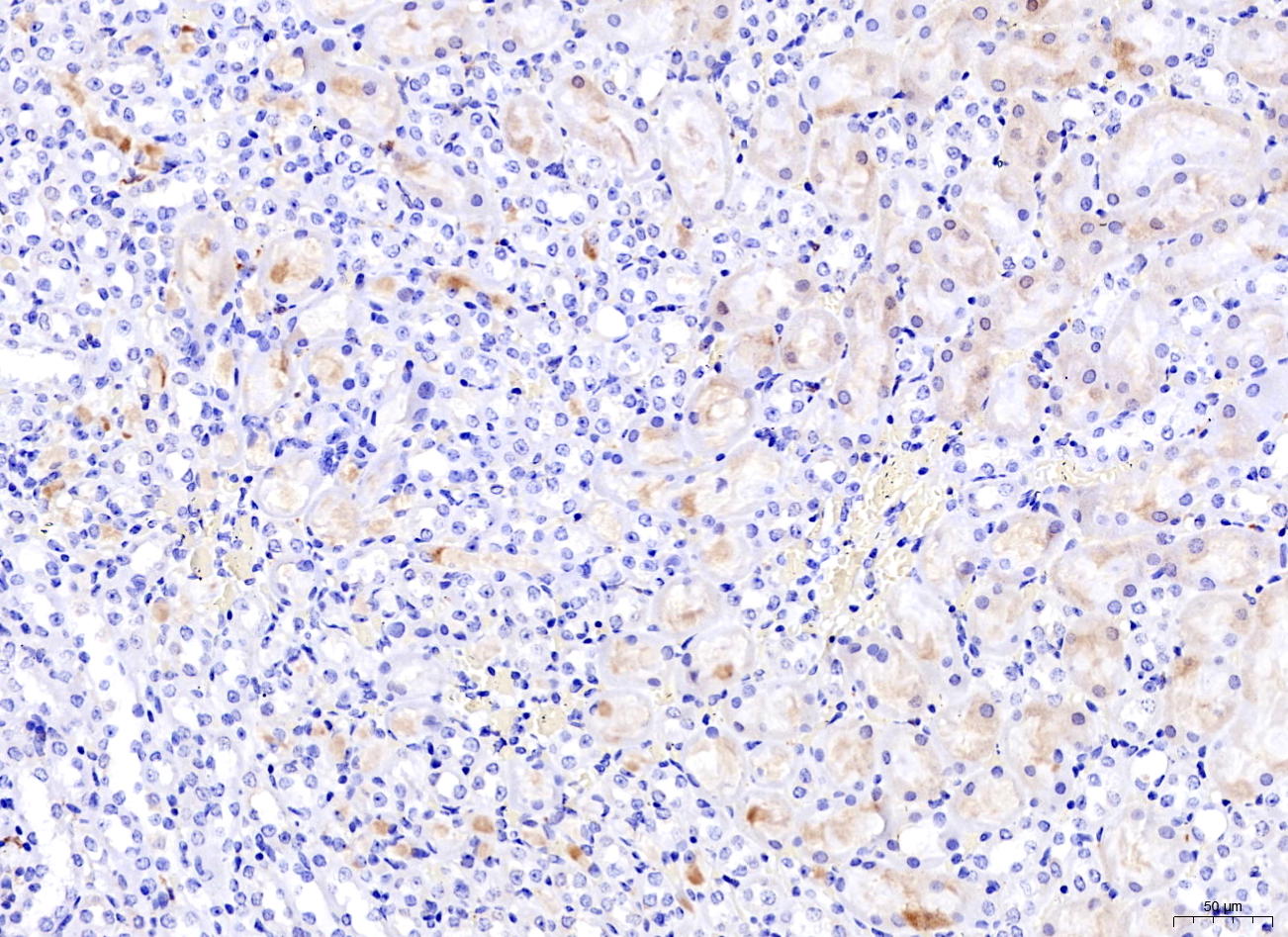

Supplement: Supplementary file 3 — Supporting File 3: advs73867‐sup‐0003‐SupportingFiguresData.zip. [file ADVS-13-e19191-s003.zip › Supporting information Figure S1-S9/S2/Kidney/SCRS 8week Model 763_20.0x-4.jpg]

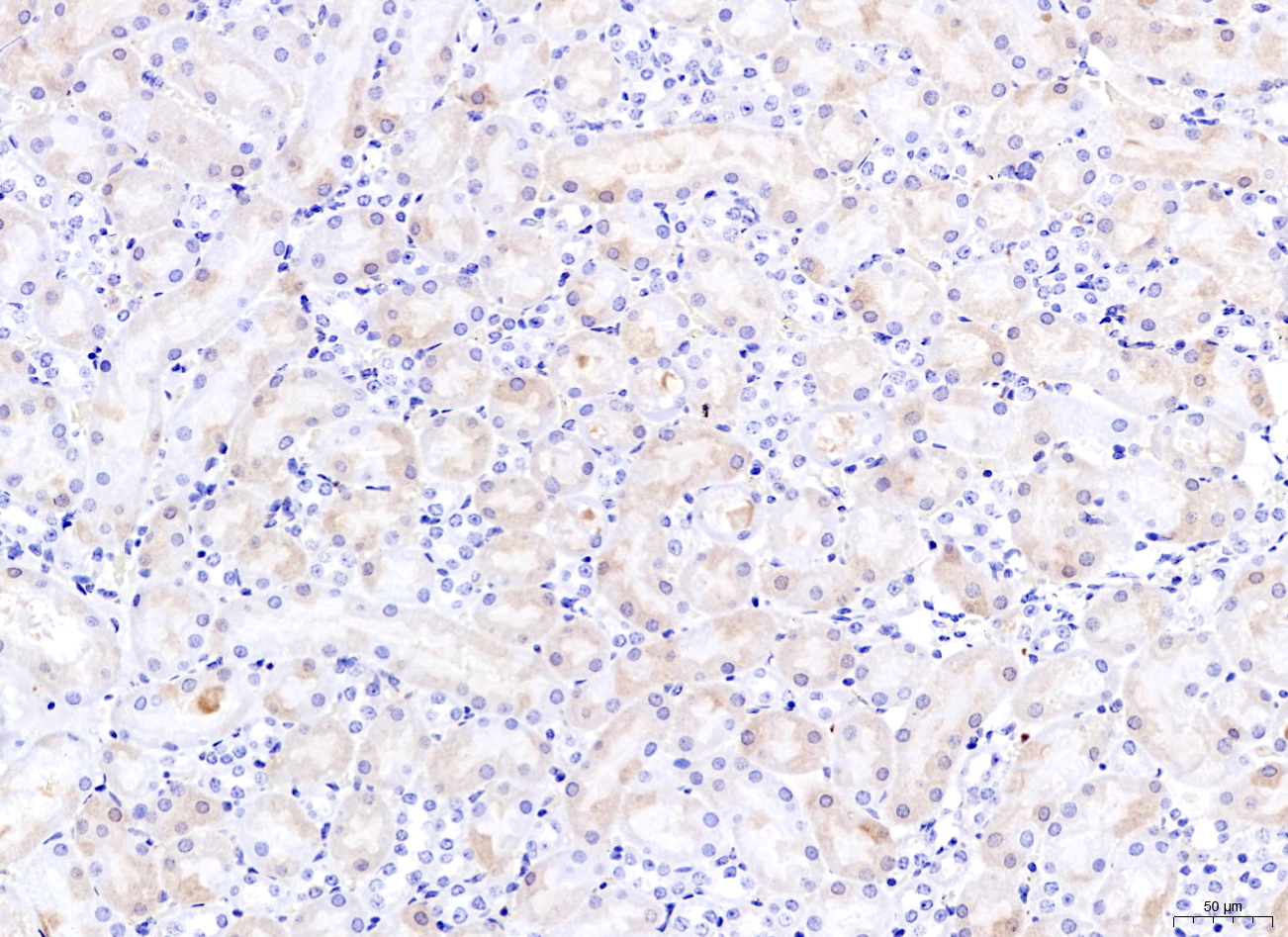

Supplement: Supplementary file 3 — Supporting File 3: advs73867‐sup‐0003‐SupportingFiguresData.zip. [file ADVS-13-e19191-s003.zip › Supporting information Figure S1-S9/S2/Kidney/SCRS 8week Model 763_20.0x-5.jpg]

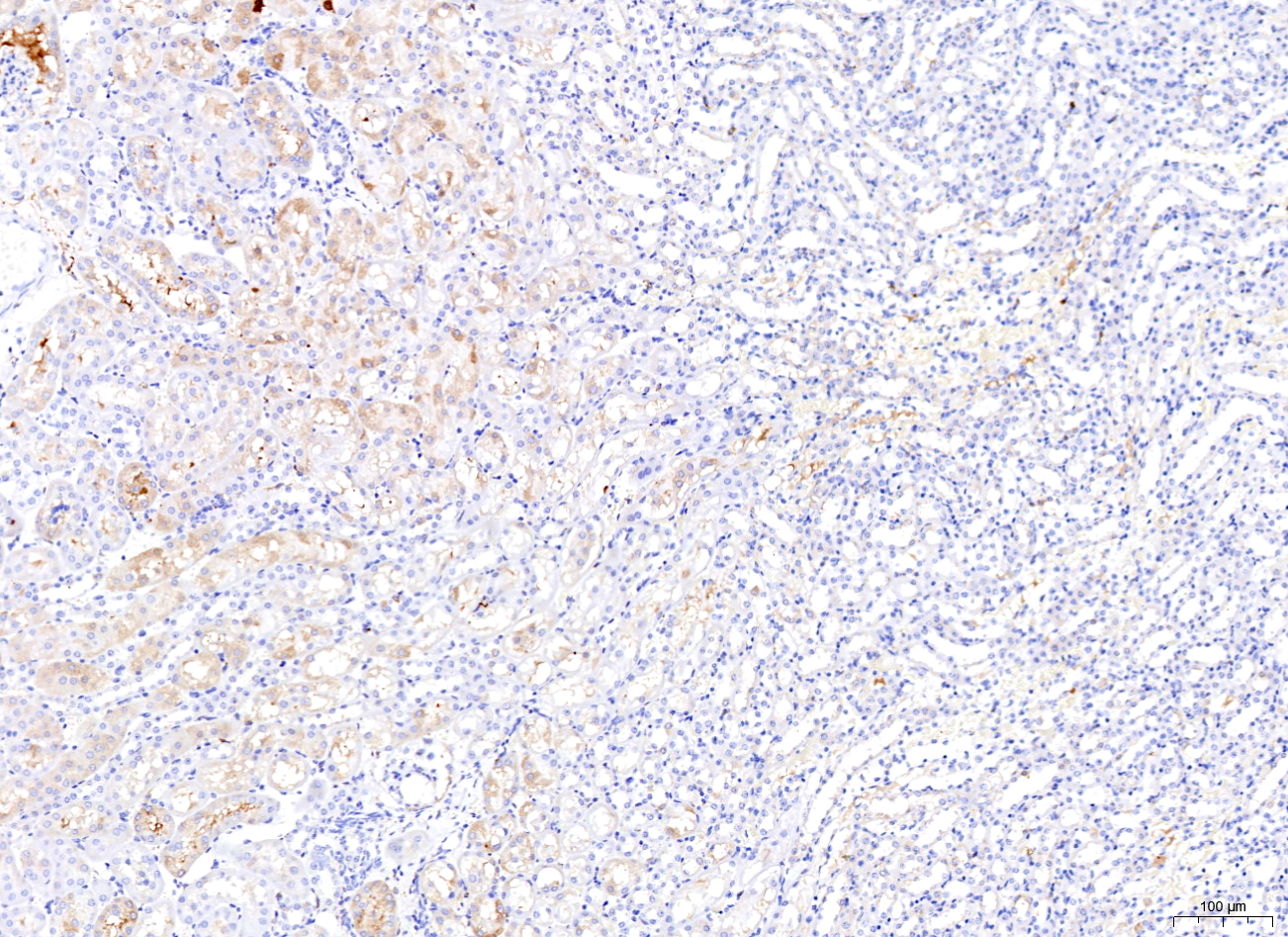

Supplement: Supplementary file 3 — Supporting File 3: advs73867‐sup‐0003‐SupportingFiguresData.zip. [file ADVS-13-e19191-s003.zip › Supporting information Figure S1-S9/S2/Kidney/SCRS Control 782_10.0x.jpg]

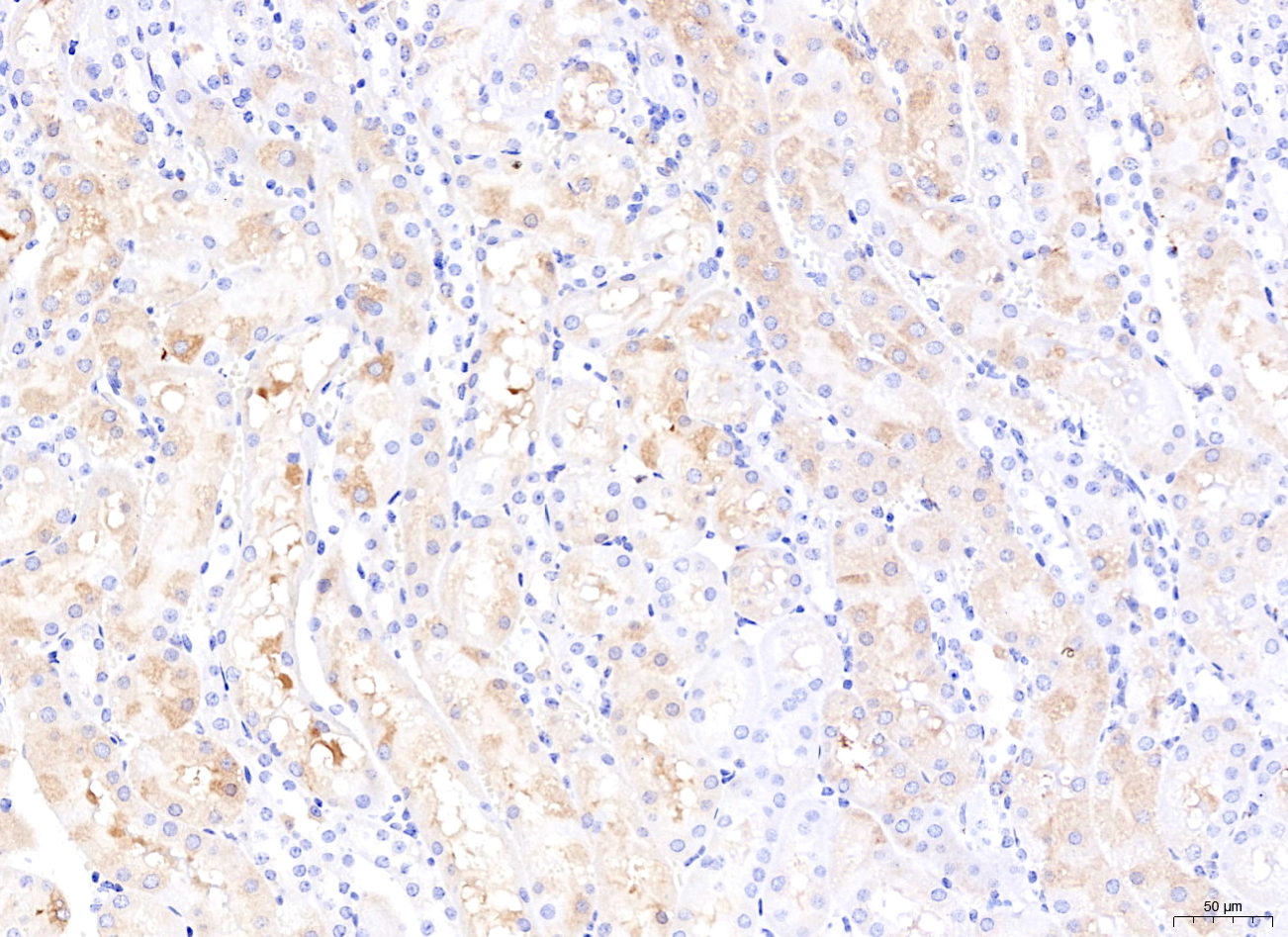

Supplement: Supplementary file 3 — Supporting File 3: advs73867‐sup‐0003‐SupportingFiguresData.zip. [file ADVS-13-e19191-s003.zip › Supporting information Figure S1-S9/S2/Kidney/SCRS Control 782_20.0x-1.jpg]

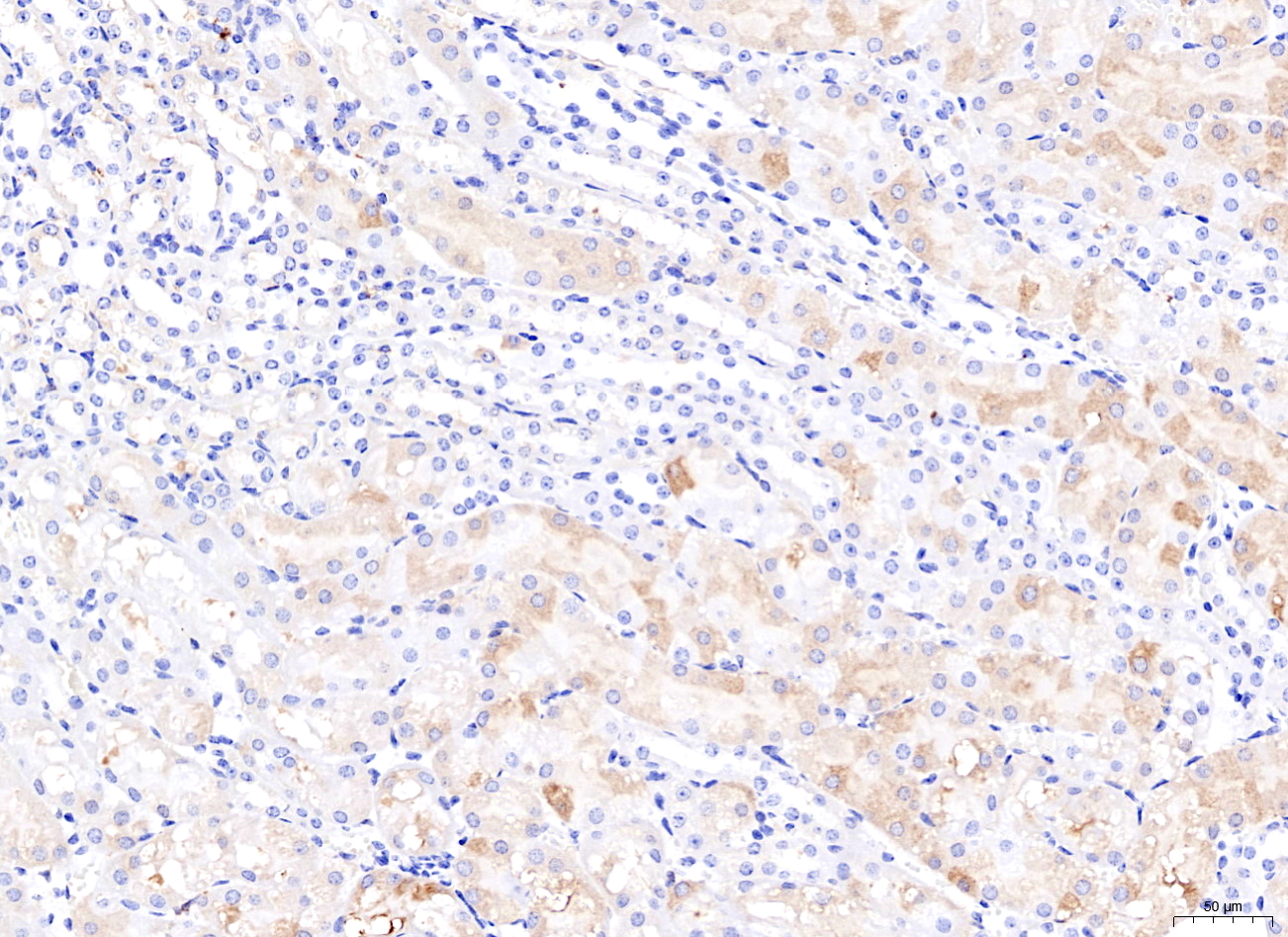

Supplement: Supplementary file 3 — Supporting File 3: advs73867‐sup‐0003‐SupportingFiguresData.zip. [file ADVS-13-e19191-s003.zip › Supporting information Figure S1-S9/S2/Kidney/SCRS Control 782_20.0x-2.jpg]

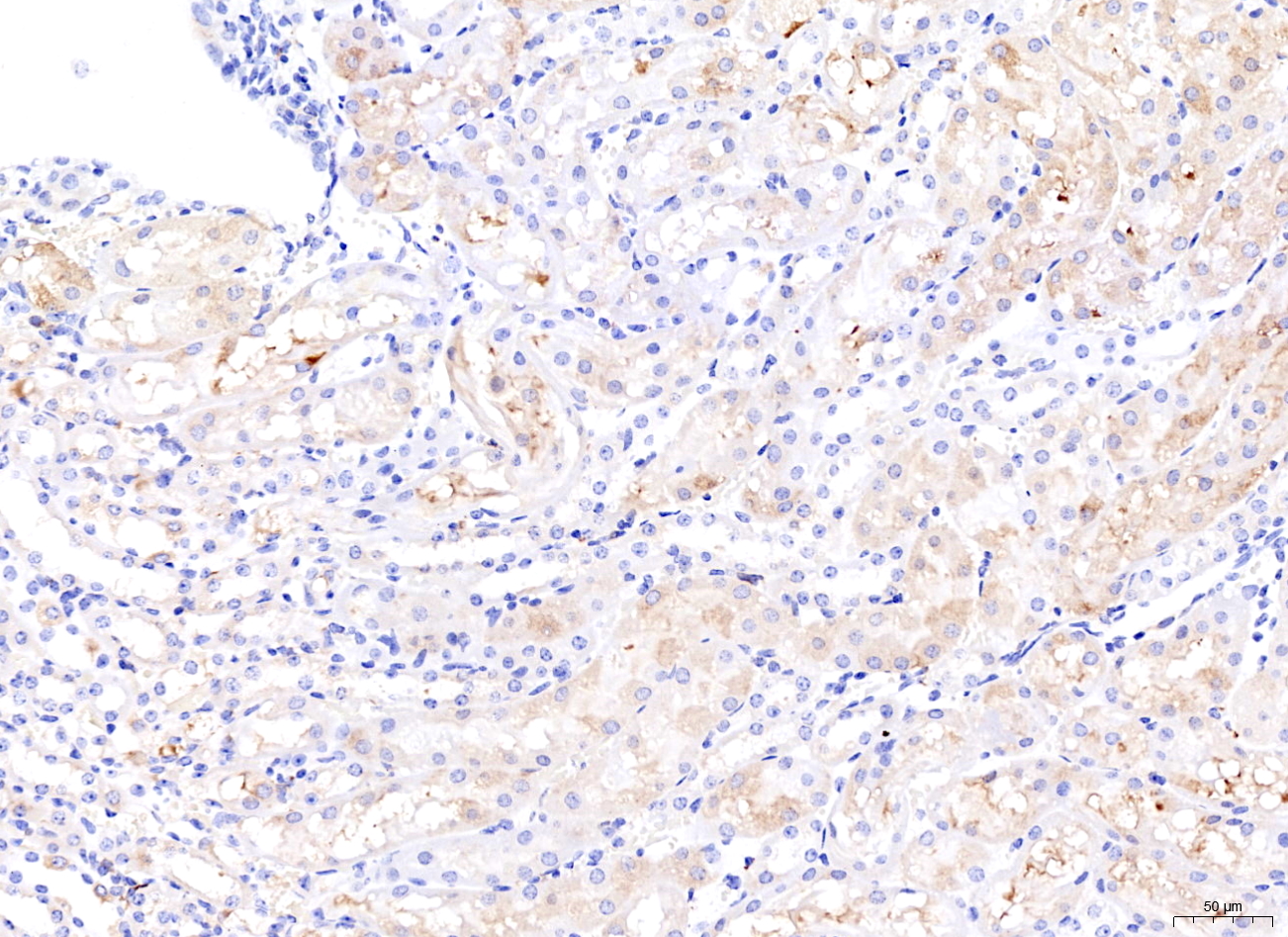

Supplement: Supplementary file 3 — Supporting File 3: advs73867‐sup‐0003‐SupportingFiguresData.zip. [file ADVS-13-e19191-s003.zip › Supporting information Figure S1-S9/S2/Kidney/SCRS Control 782_20.0x-3.jpg]

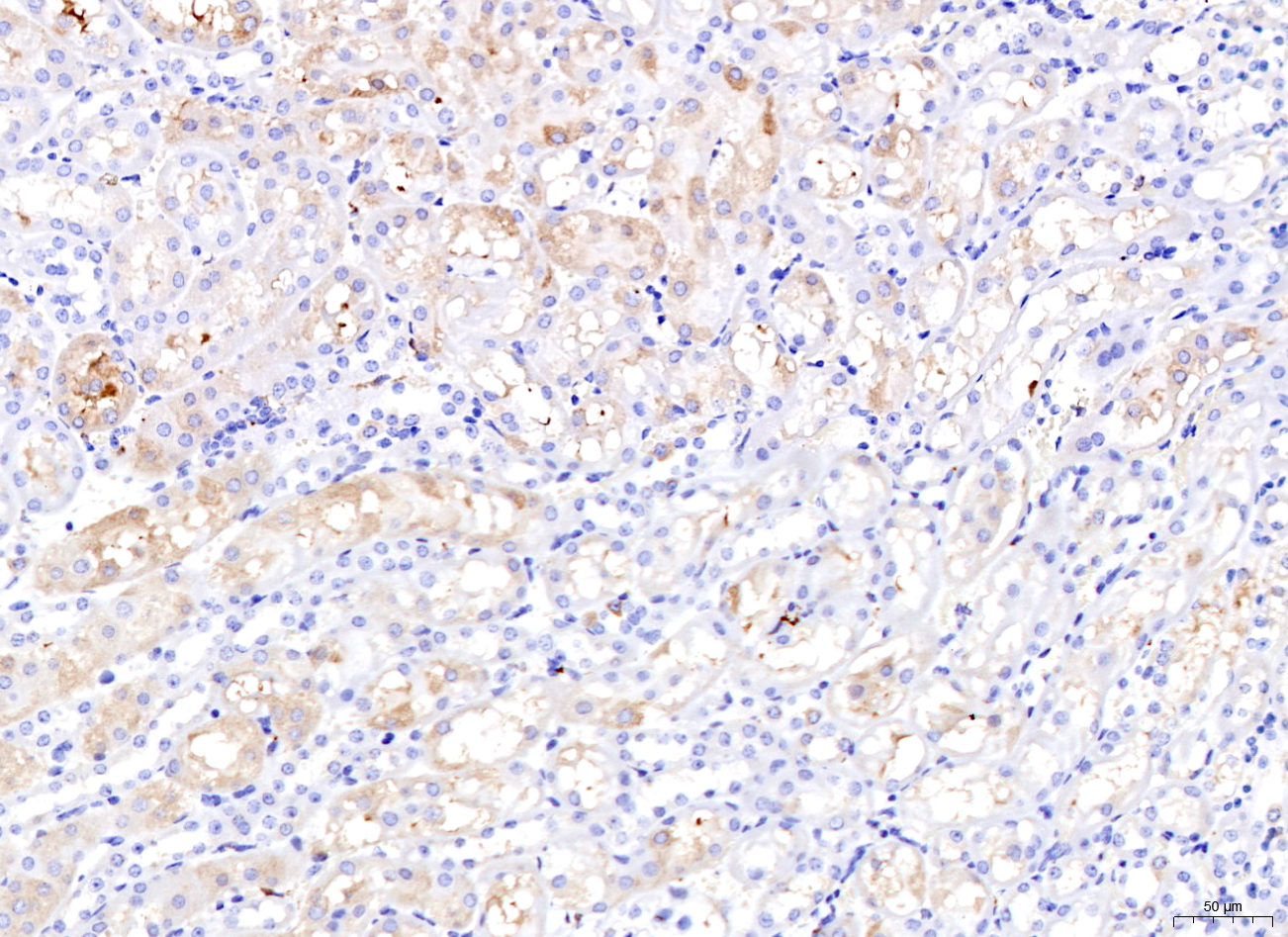

Supplement: Supplementary file 3 — Supporting File 3: advs73867‐sup‐0003‐SupportingFiguresData.zip. [file ADVS-13-e19191-s003.zip › Supporting information Figure S1-S9/S2/Kidney/SCRS Control 782_20.0x-4.jpg]

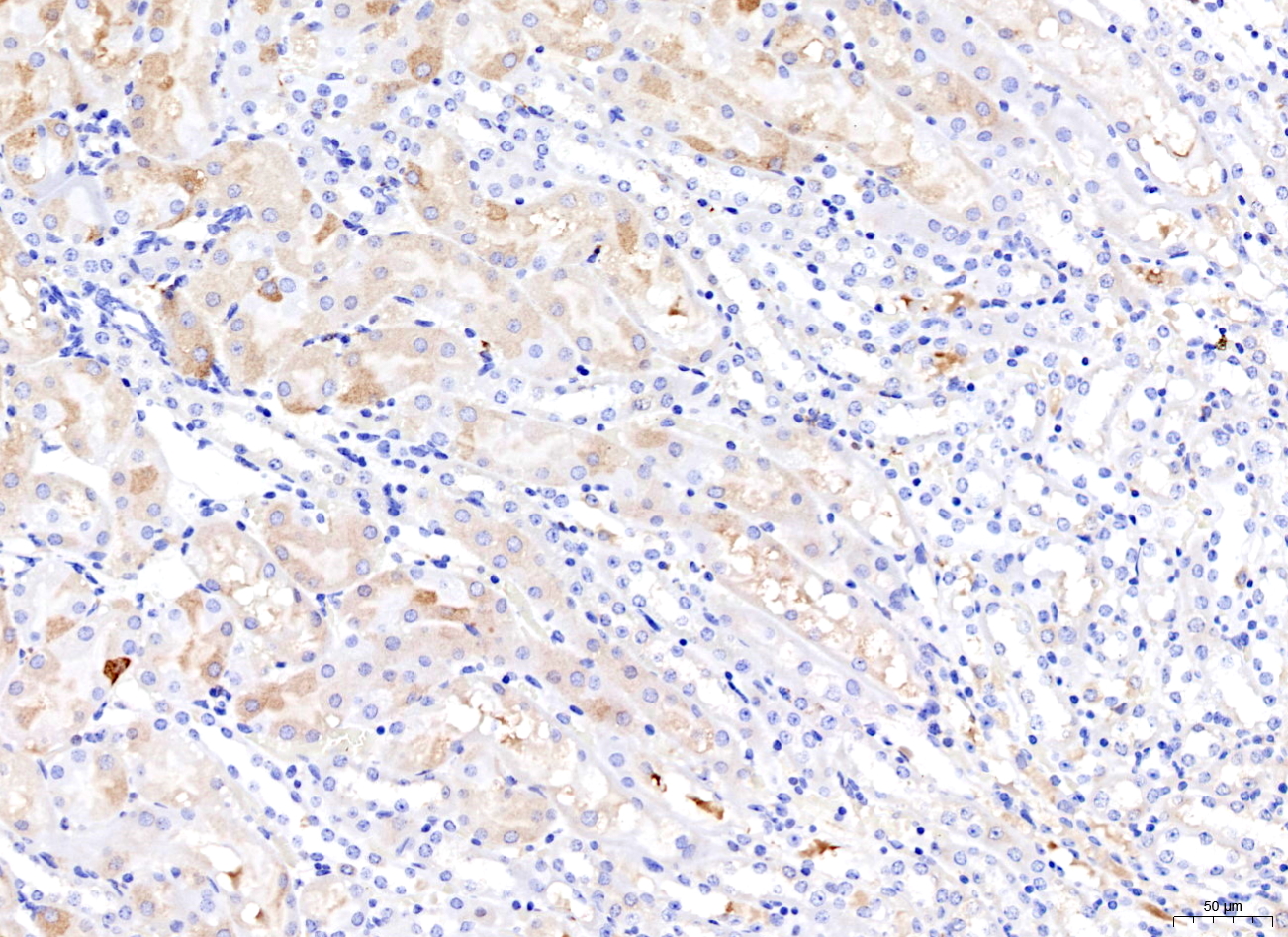

Supplement: Supplementary file 3 — Supporting File 3: advs73867‐sup‐0003‐SupportingFiguresData.zip. [file ADVS-13-e19191-s003.zip › Supporting information Figure S1-S9/S2/Kidney/SCRS Control 782_20.0x-5.jpg]

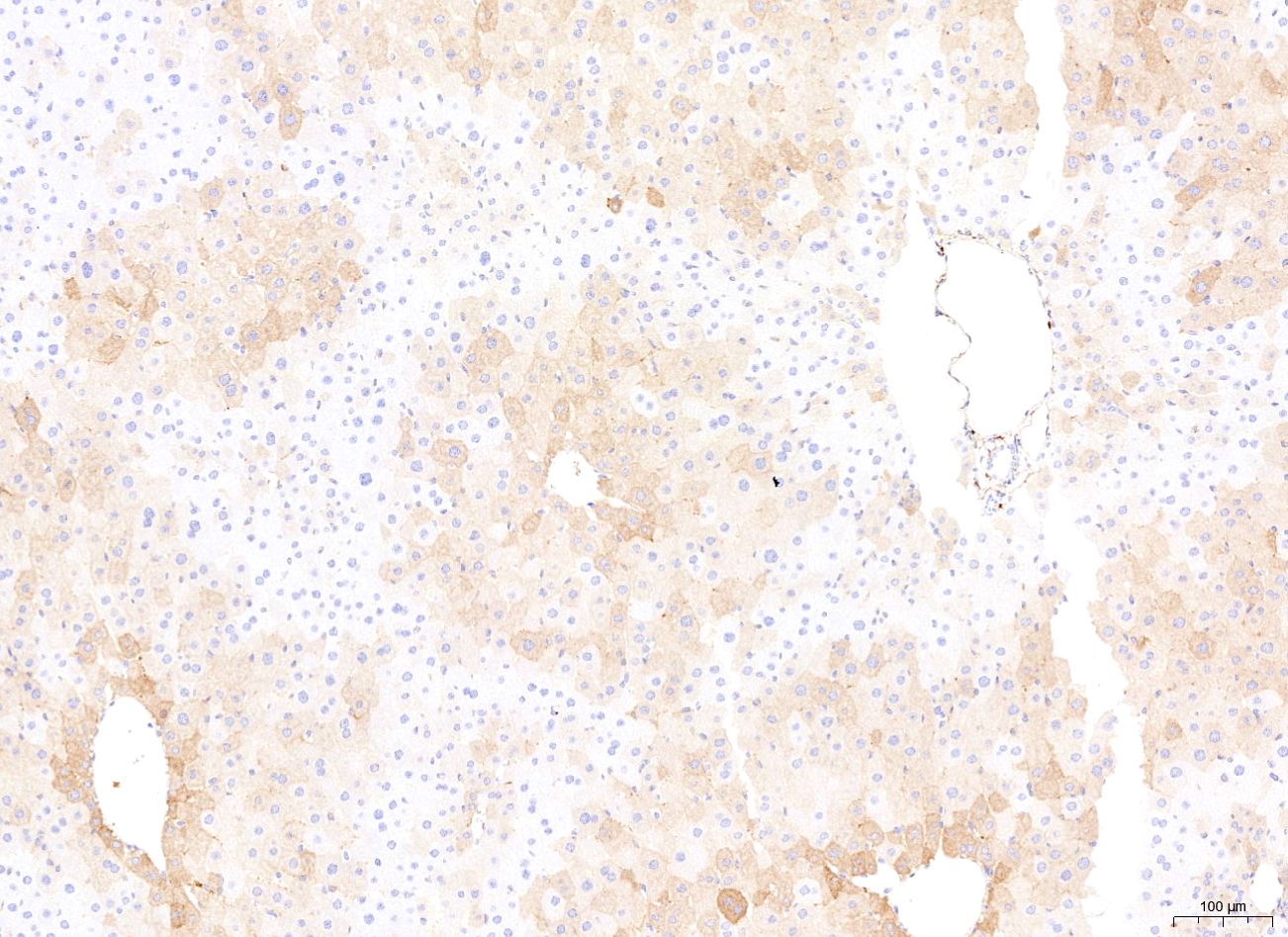

Supplement: Supplementary file 3 — Supporting File 3: advs73867‐sup‐0003‐SupportingFiguresData.zip. [file ADVS-13-e19191-s003.zip › Supporting information Figure S1-S9/S2/Liver/SCRS 12week Model 784_10.0x.jpg]

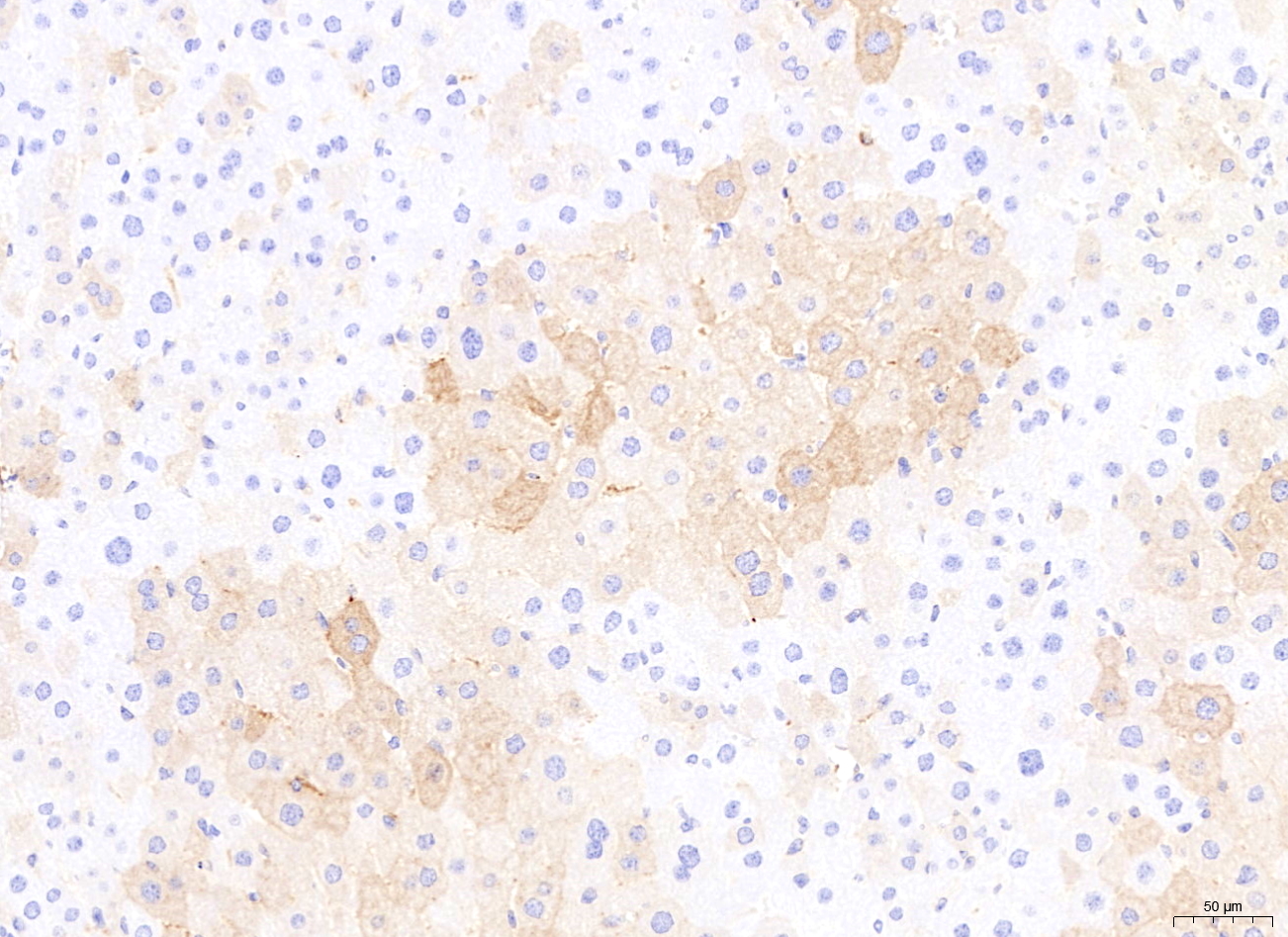

Supplement: Supplementary file 3 — Supporting File 3: advs73867‐sup‐0003‐SupportingFiguresData.zip. [file ADVS-13-e19191-s003.zip › Supporting information Figure S1-S9/S2/Liver/SCRS 12week Model 784_20.0x-1.jpg]

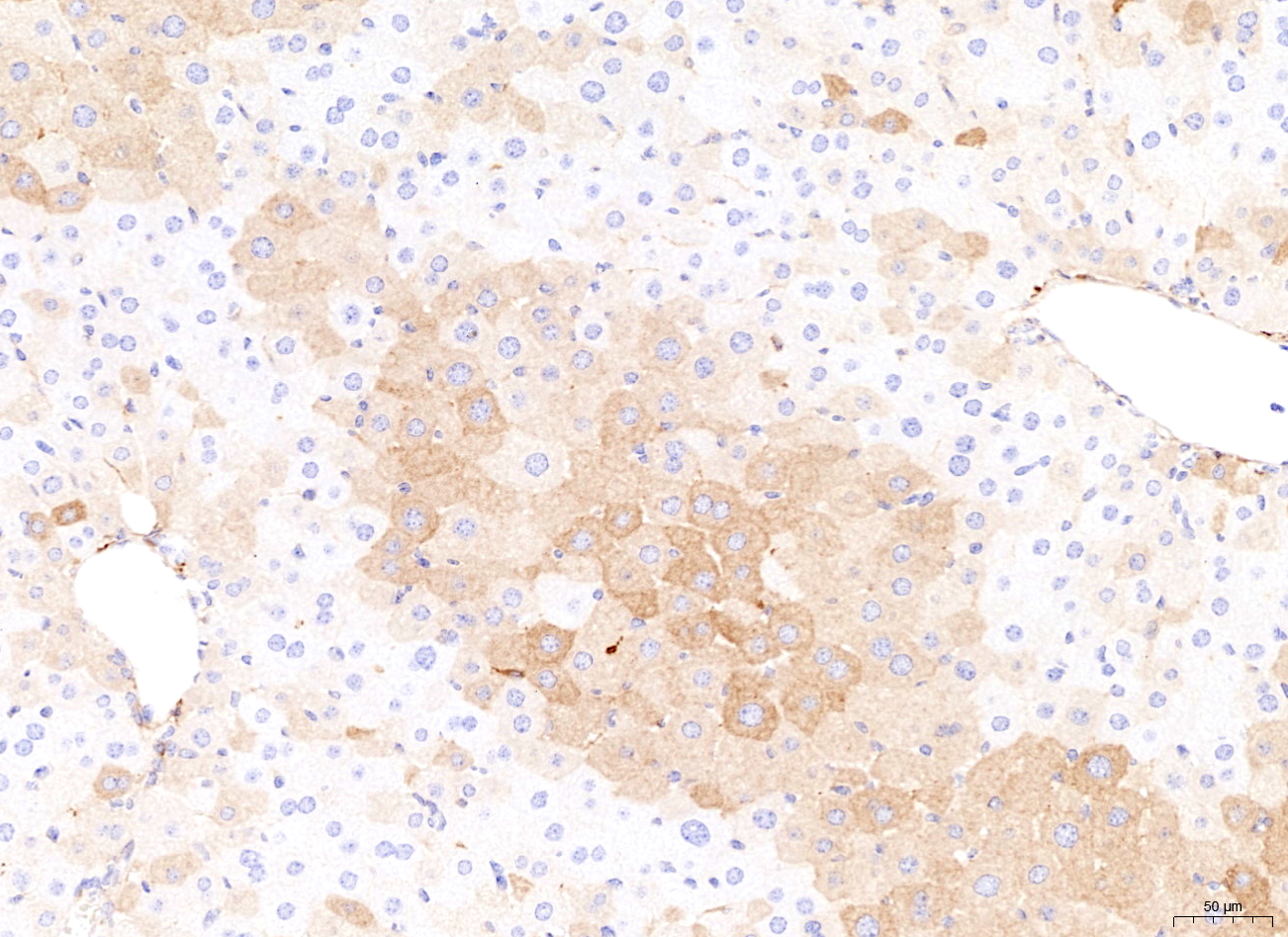

Supplement: Supplementary file 3 — Supporting File 3: advs73867‐sup‐0003‐SupportingFiguresData.zip. [file ADVS-13-e19191-s003.zip › Supporting information Figure S1-S9/S2/Liver/SCRS 12week Model 784_20.0x-2.jpg]

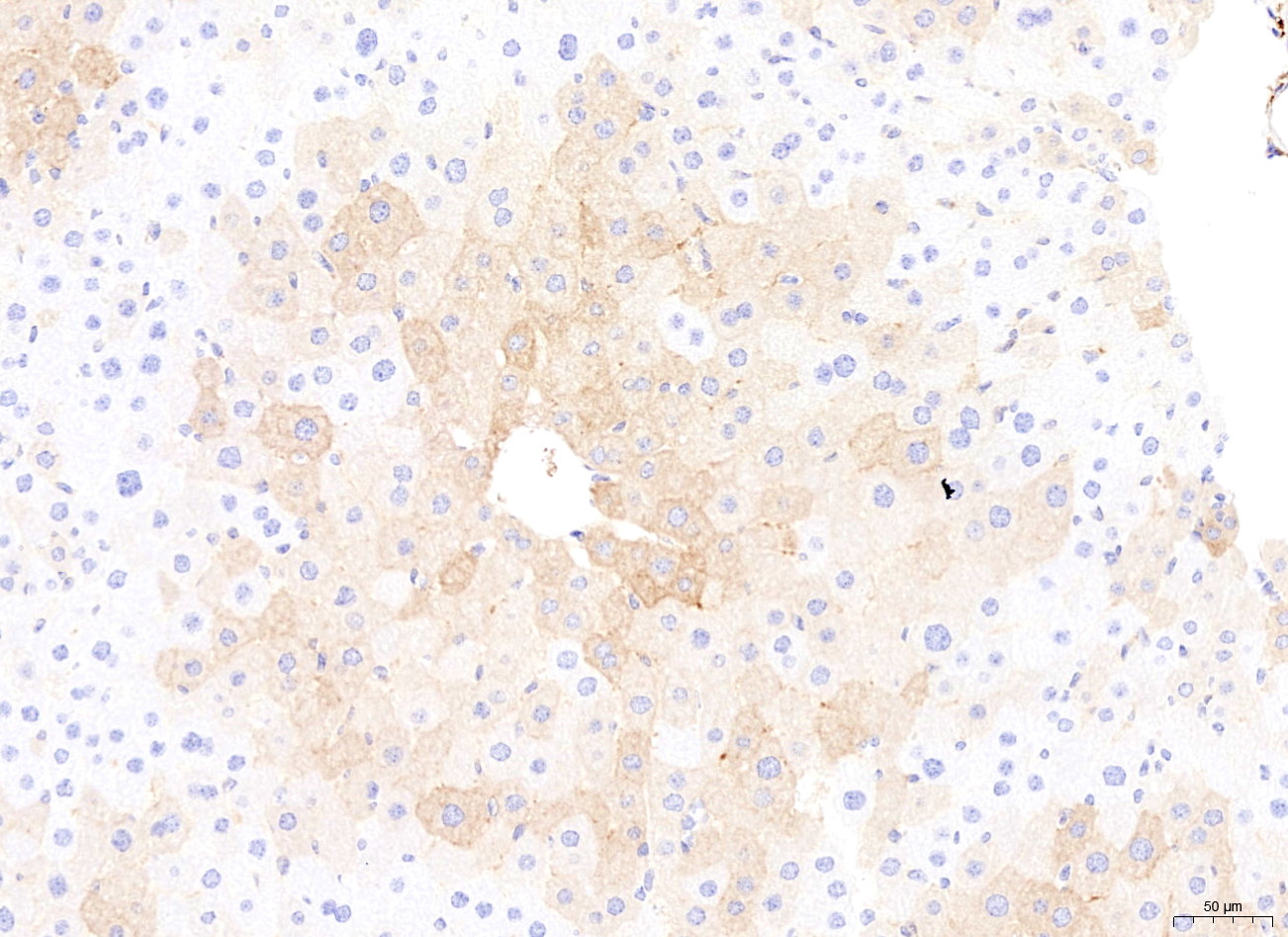

Supplement: Supplementary file 3 — Supporting File 3: advs73867‐sup‐0003‐SupportingFiguresData.zip. [file ADVS-13-e19191-s003.zip › Supporting information Figure S1-S9/S2/Liver/SCRS 12week Model 784_20.0x-4.jpg]

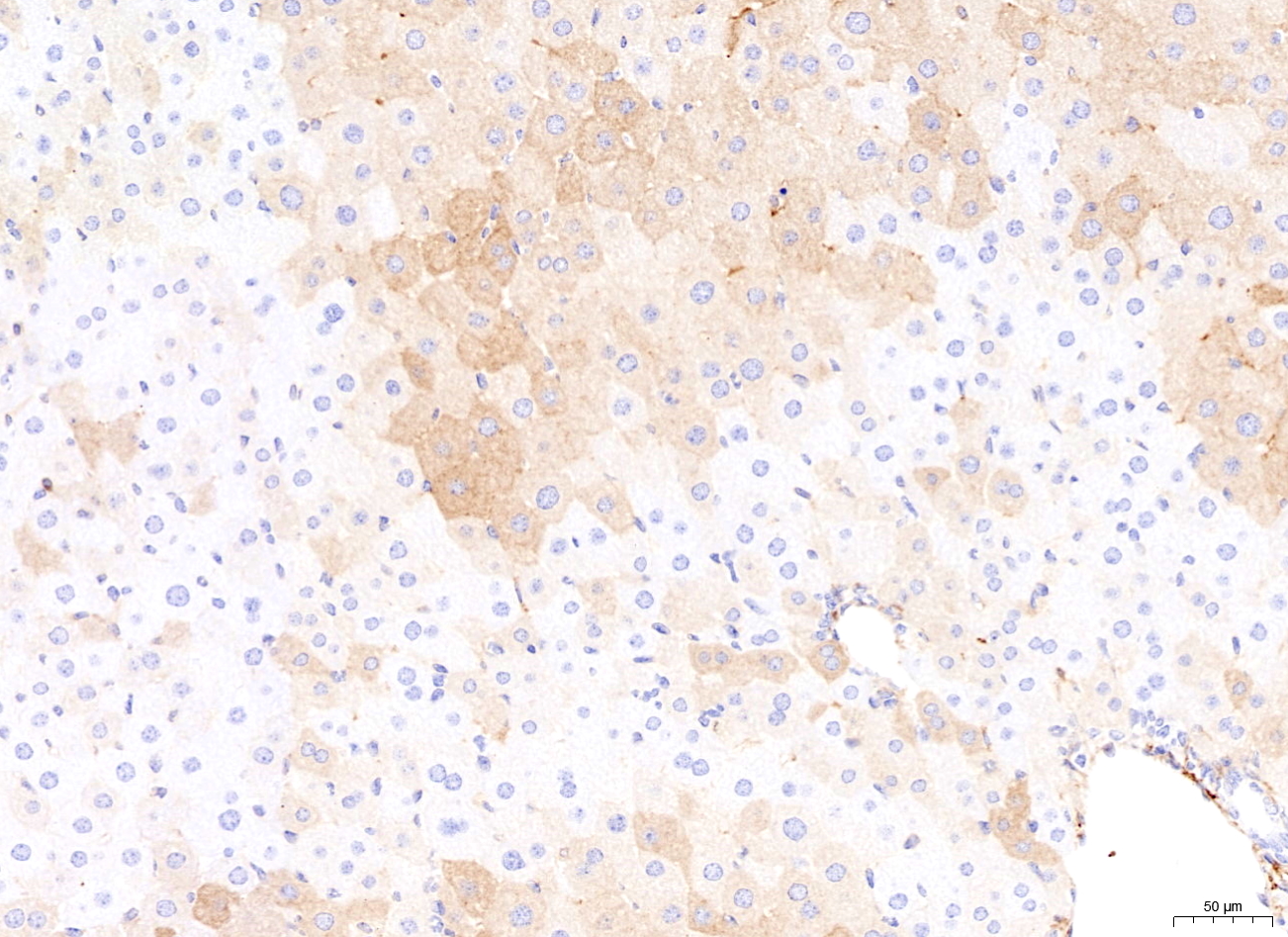

Supplement: Supplementary file 3 — Supporting File 3: advs73867‐sup‐0003‐SupportingFiguresData.zip. [file ADVS-13-e19191-s003.zip › Supporting information Figure S1-S9/S2/Liver/SCRS 12week Model 784_20.0x-5.jpg]

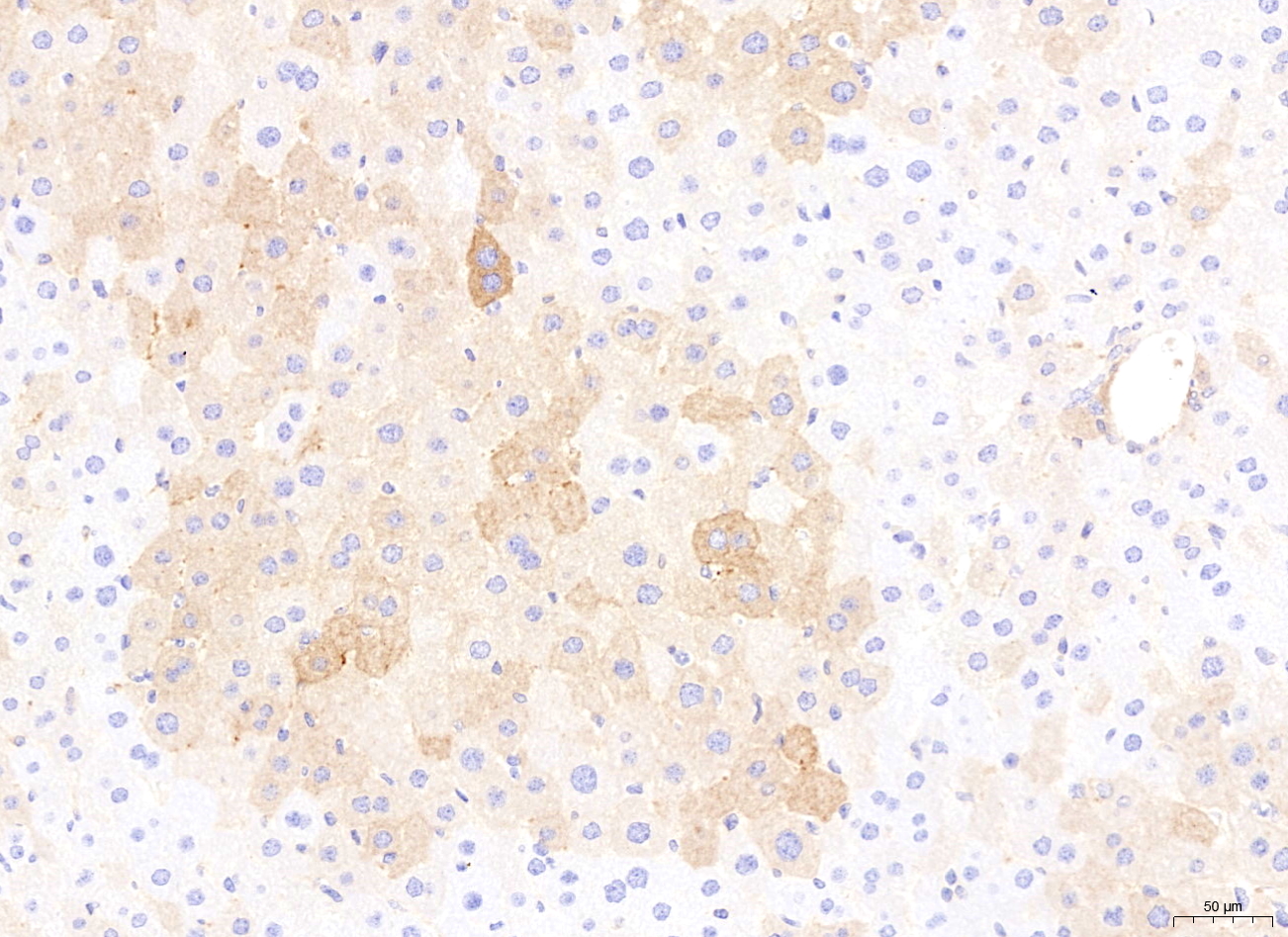

Supplement: Supplementary file 3 — Supporting File 3: advs73867‐sup‐0003‐SupportingFiguresData.zip. [file ADVS-13-e19191-s003.zip › Supporting information Figure S1-S9/S2/Liver/SCRS 12week Model 784_20.0x-6.jpg]

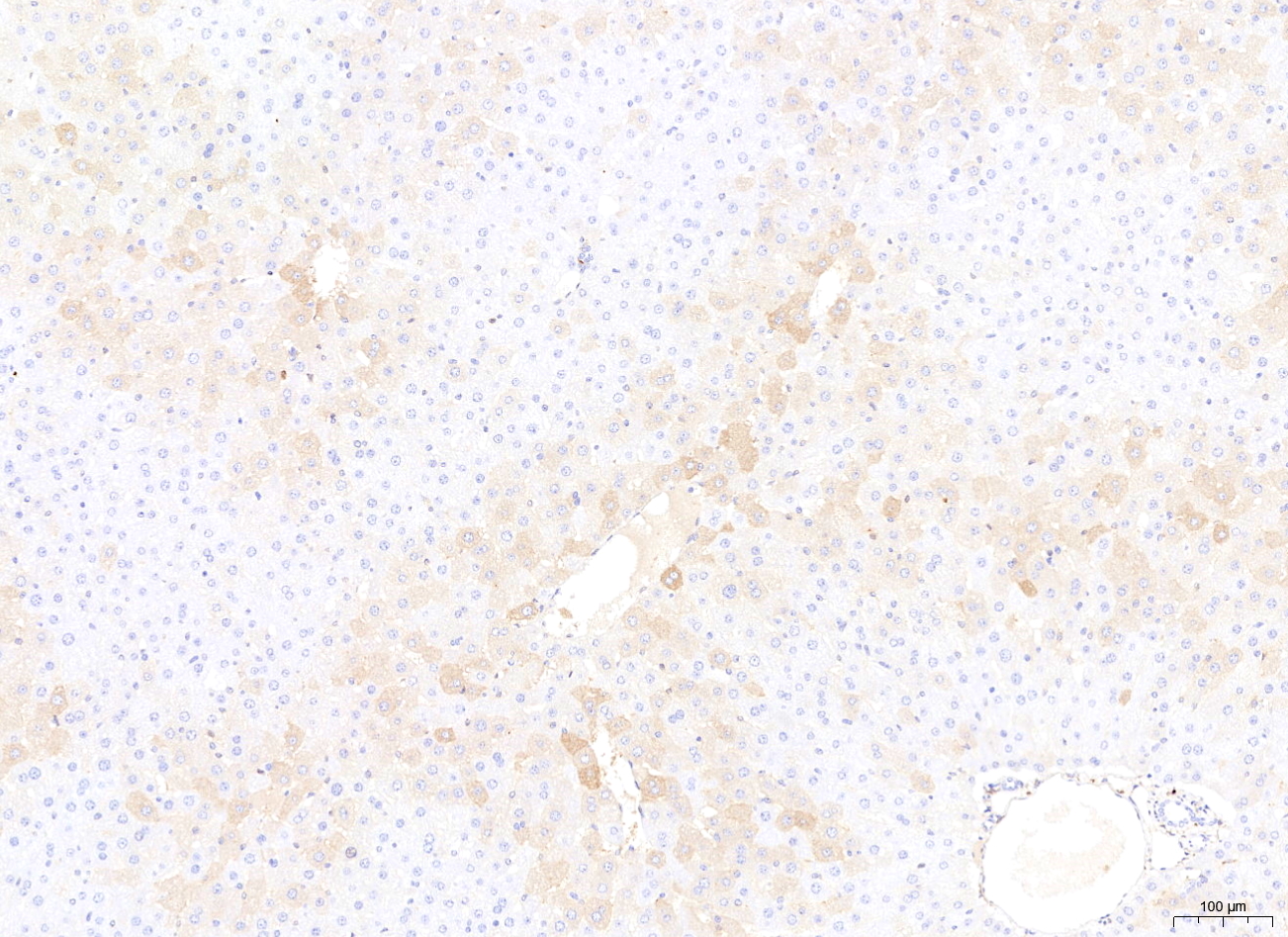

Supplement: Supplementary file 3 — Supporting File 3: advs73867‐sup‐0003‐SupportingFiguresData.zip. [file ADVS-13-e19191-s003.zip › Supporting information Figure S1-S9/S2/Liver/SCRS 1week Model 757_10.0x.jpg]

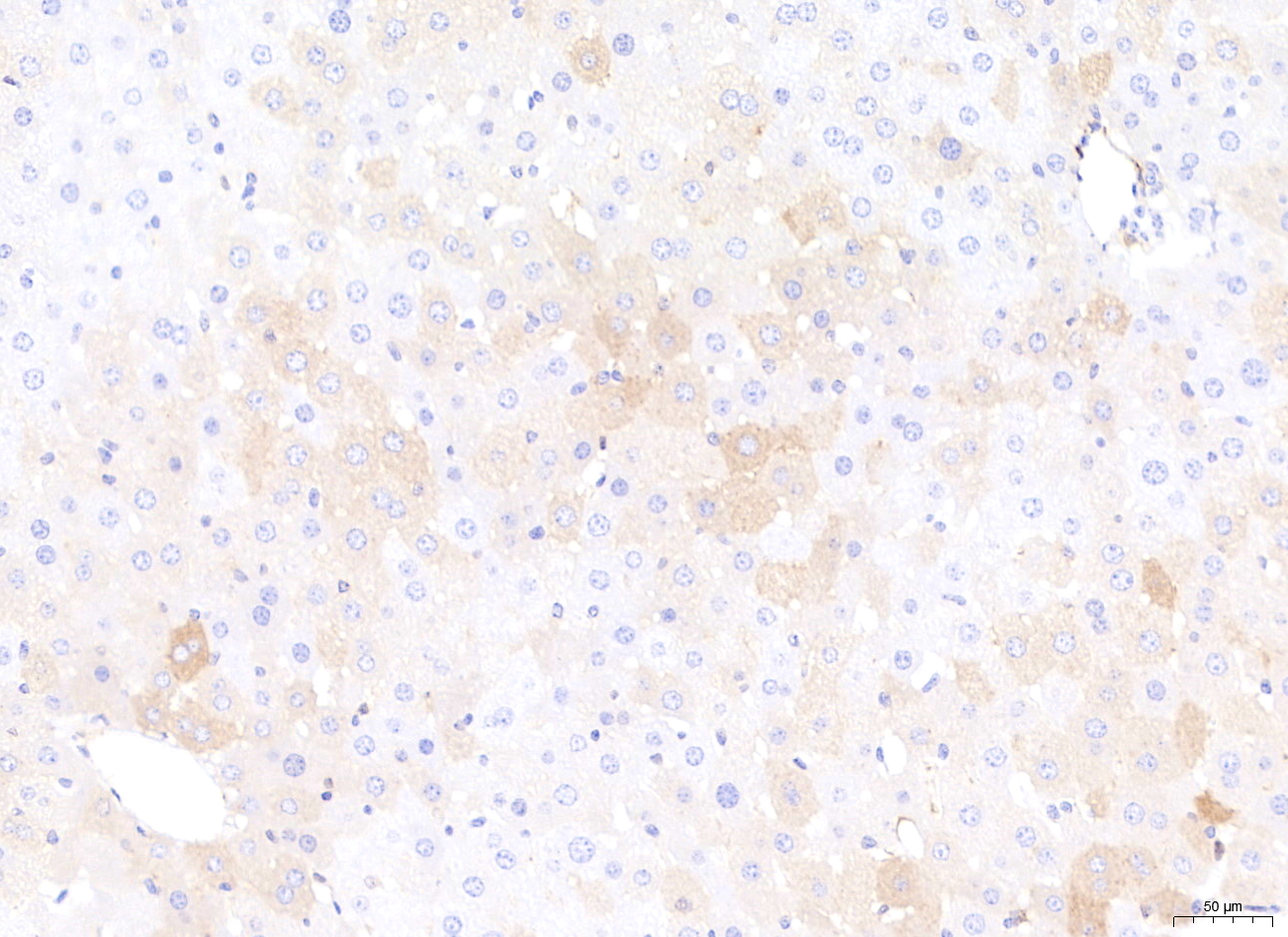

Supplement: Supplementary file 3 — Supporting File 3: advs73867‐sup‐0003‐SupportingFiguresData.zip. [file ADVS-13-e19191-s003.zip › Supporting information Figure S1-S9/S2/Liver/SCRS 1week Model 757_20.0x-1.jpg]

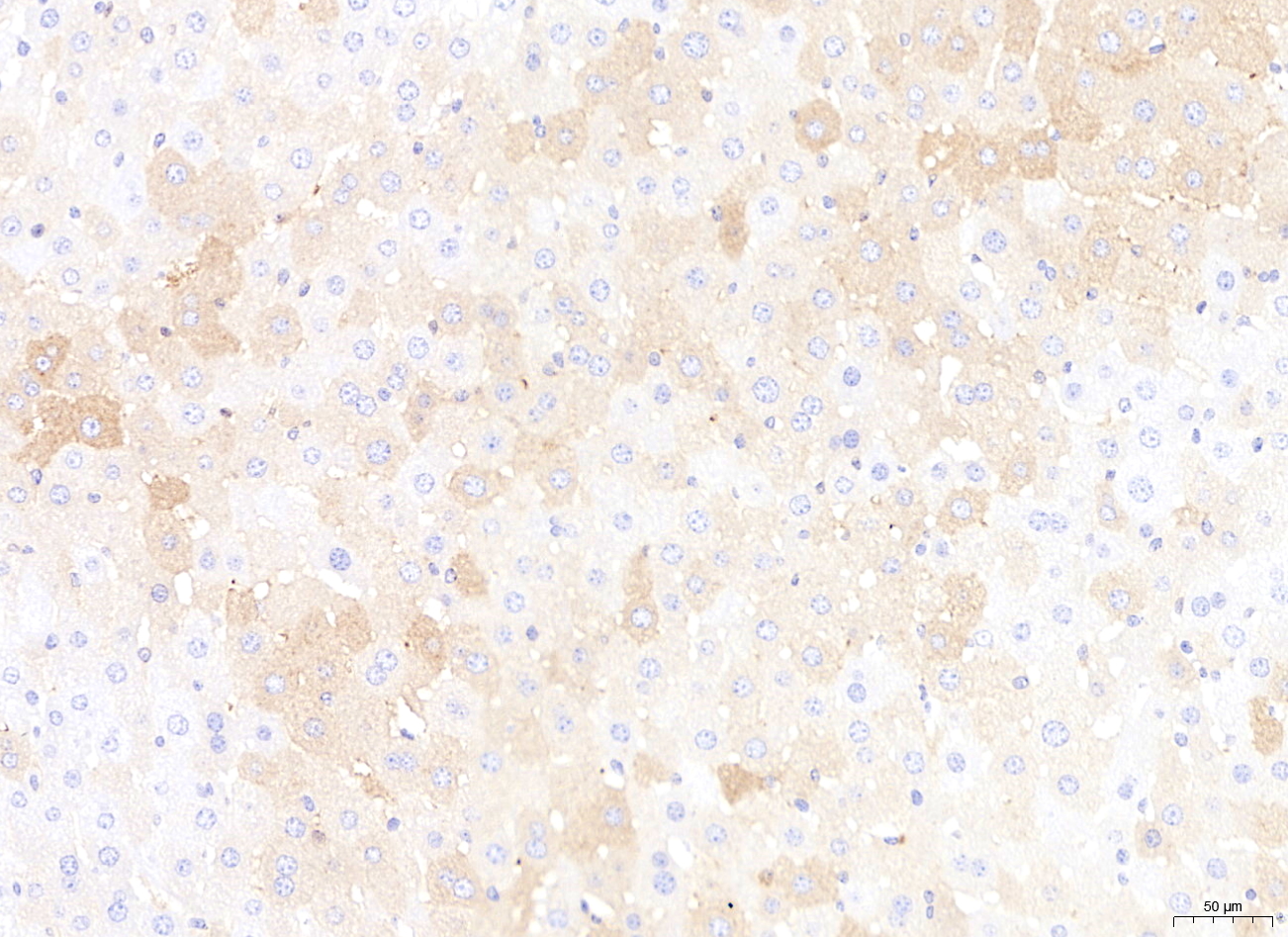

Supplement: Supplementary file 3 — Supporting File 3: advs73867‐sup‐0003‐SupportingFiguresData.zip. [file ADVS-13-e19191-s003.zip › Supporting information Figure S1-S9/S2/Liver/SCRS 1week Model 757_20.0x-2.jpg]

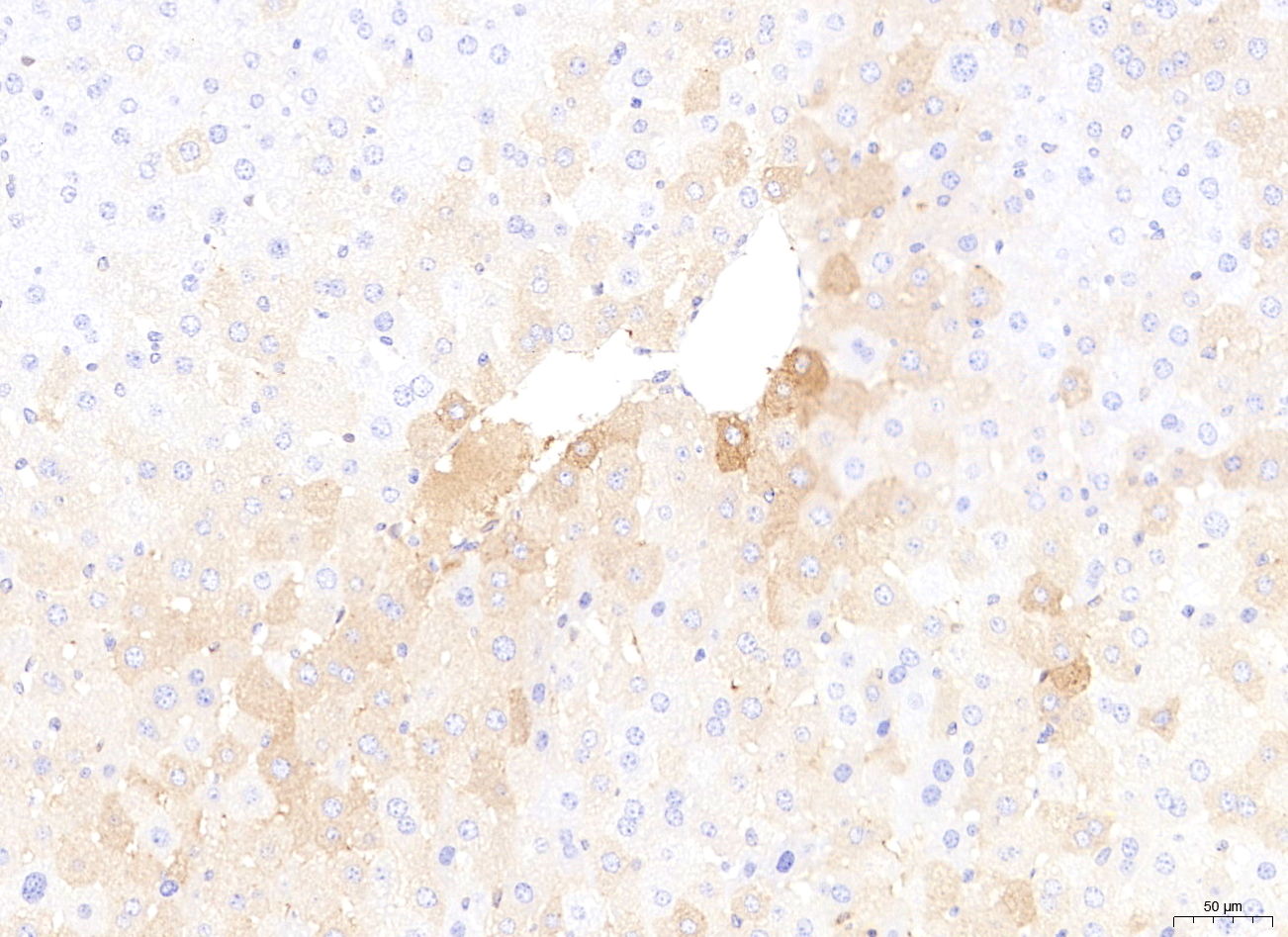

Supplement: Supplementary file 3 — Supporting File 3: advs73867‐sup‐0003‐SupportingFiguresData.zip. [file ADVS-13-e19191-s003.zip › Supporting information Figure S1-S9/S2/Liver/SCRS 1week Model 757_20.0x-3.jpg]

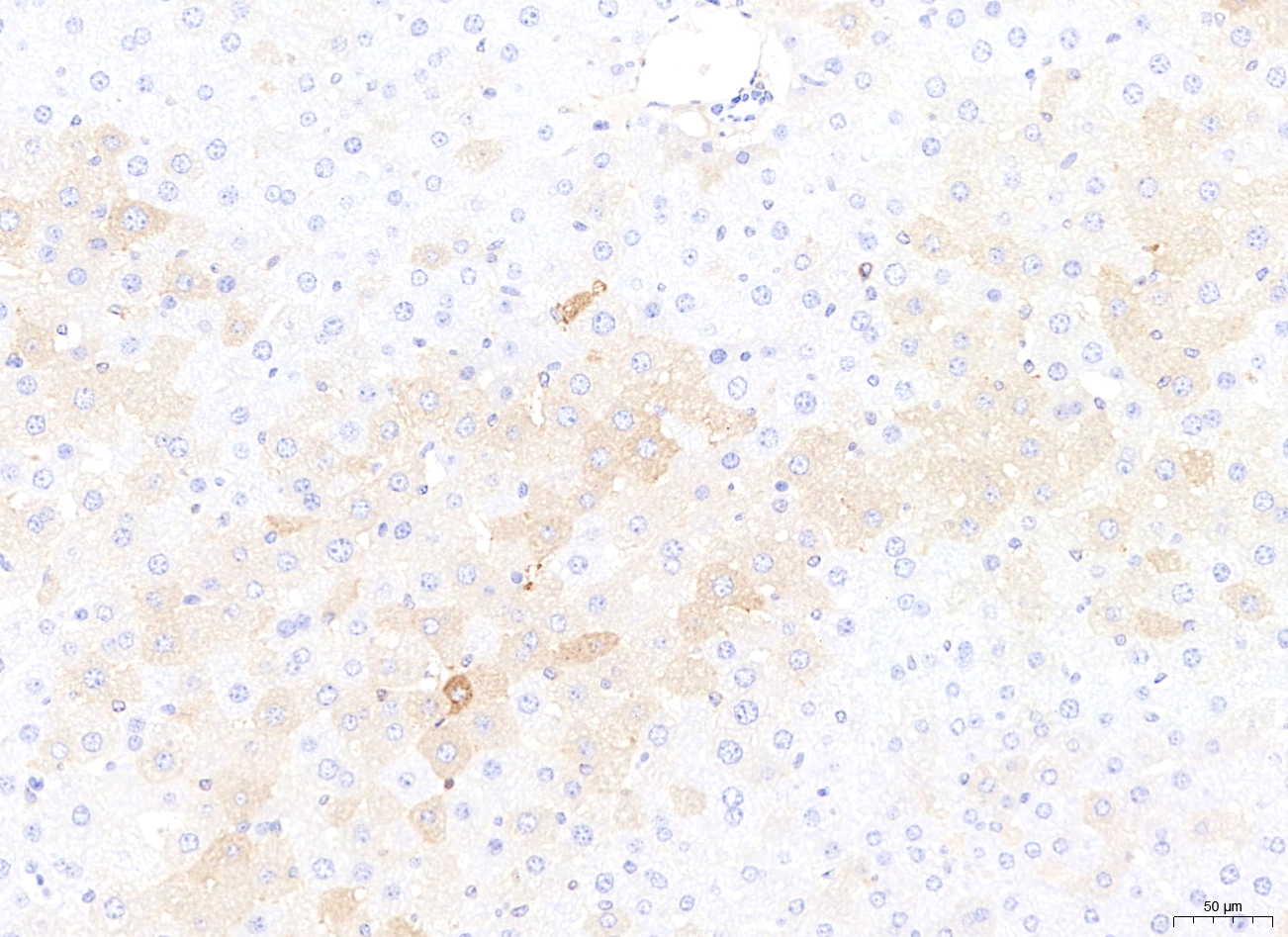

Supplement: Supplementary file 3 — Supporting File 3: advs73867‐sup‐0003‐SupportingFiguresData.zip. [file ADVS-13-e19191-s003.zip › Supporting information Figure S1-S9/S2/Liver/SCRS 1week Model 757_20.0x-4.jpg]

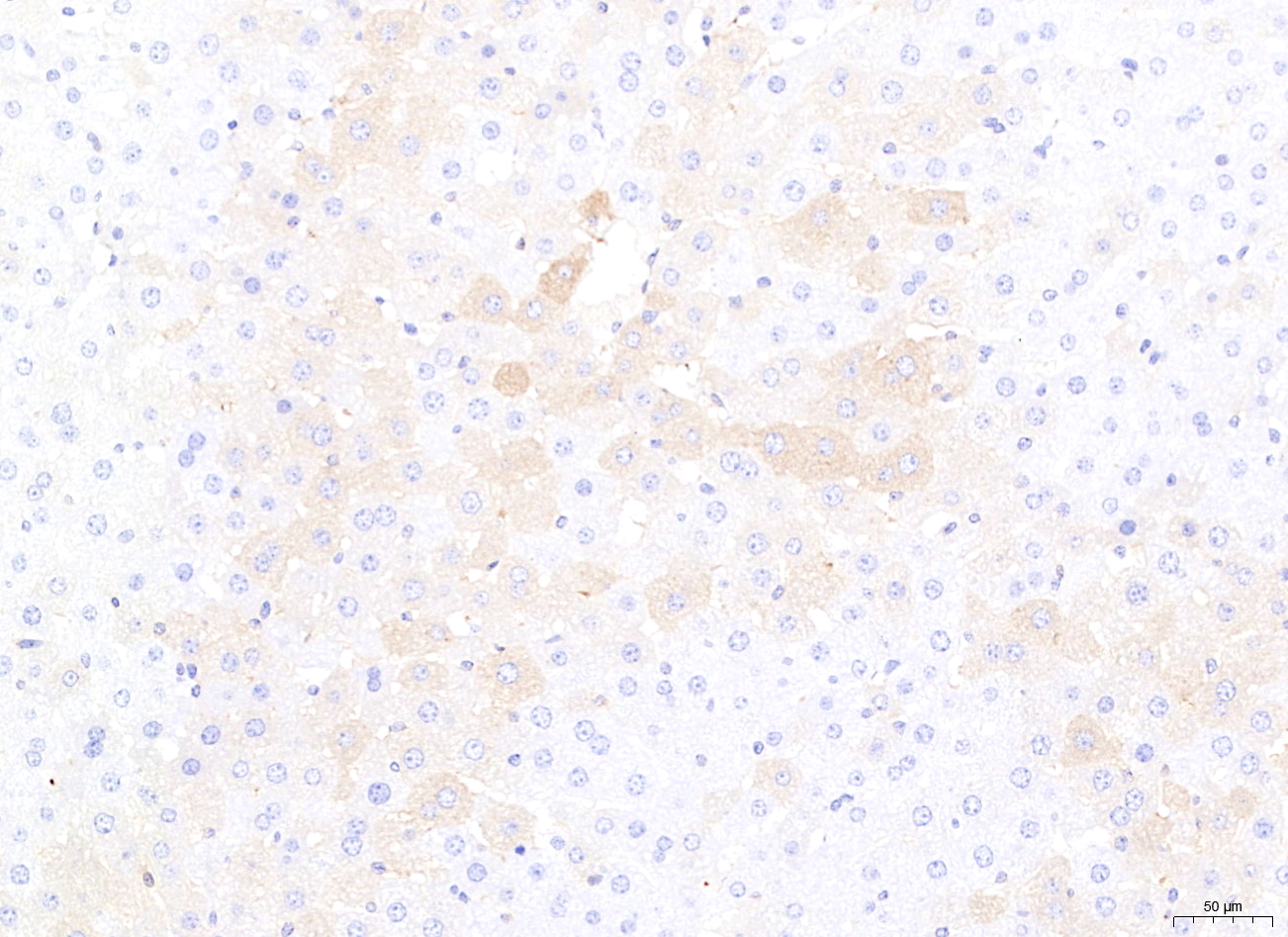

Supplement: Supplementary file 3 — Supporting File 3: advs73867‐sup‐0003‐SupportingFiguresData.zip. [file ADVS-13-e19191-s003.zip › Supporting information Figure S1-S9/S2/Liver/SCRS 1week Model 757_20.0x-5.jpg]

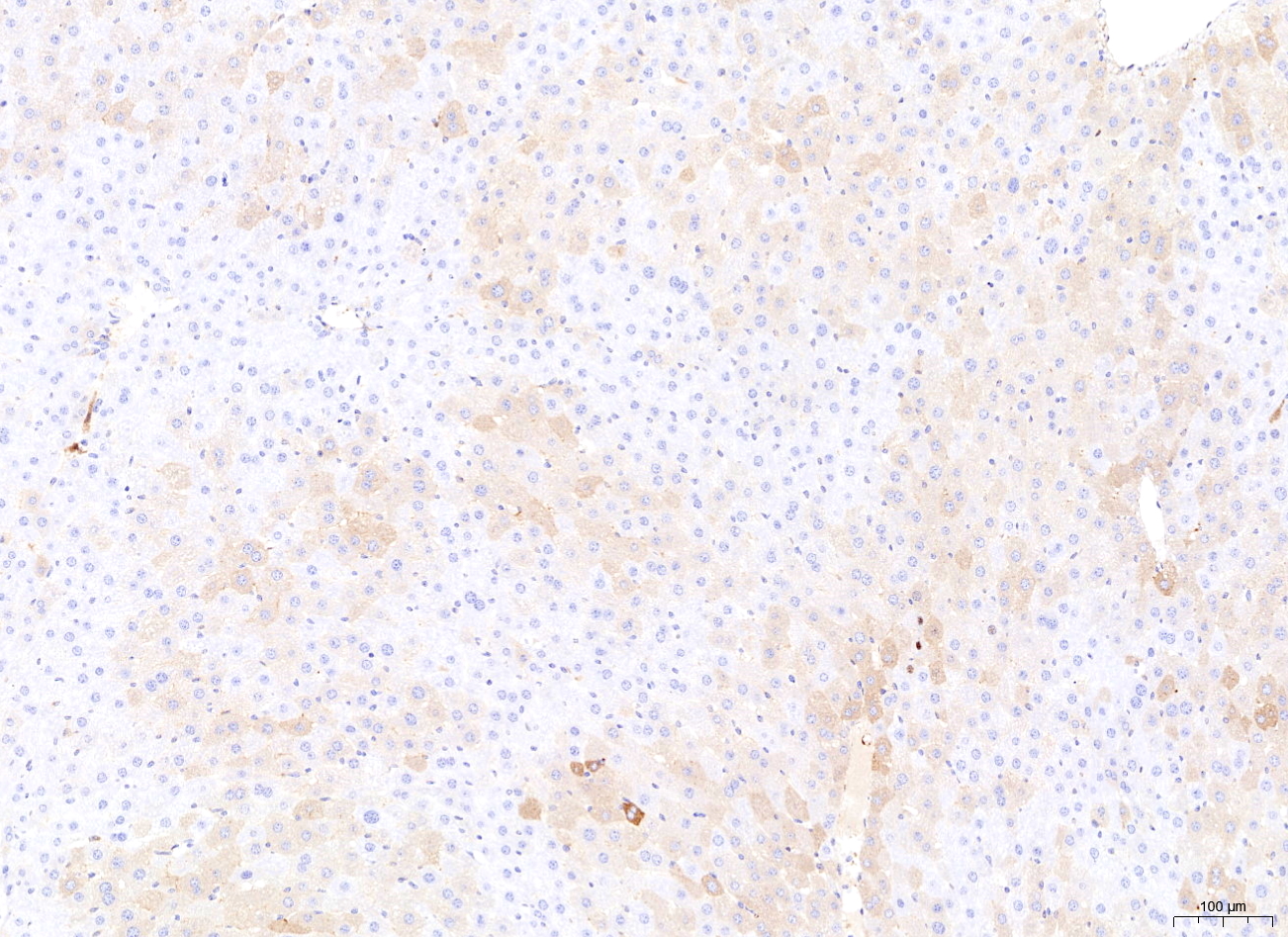

Supplement: Supplementary file 3 — Supporting File 3: advs73867‐sup‐0003‐SupportingFiguresData.zip. [file ADVS-13-e19191-s003.zip › Supporting information Figure S1-S9/S2/Liver/SCRS 4week Model 744_10.0x.jpg]

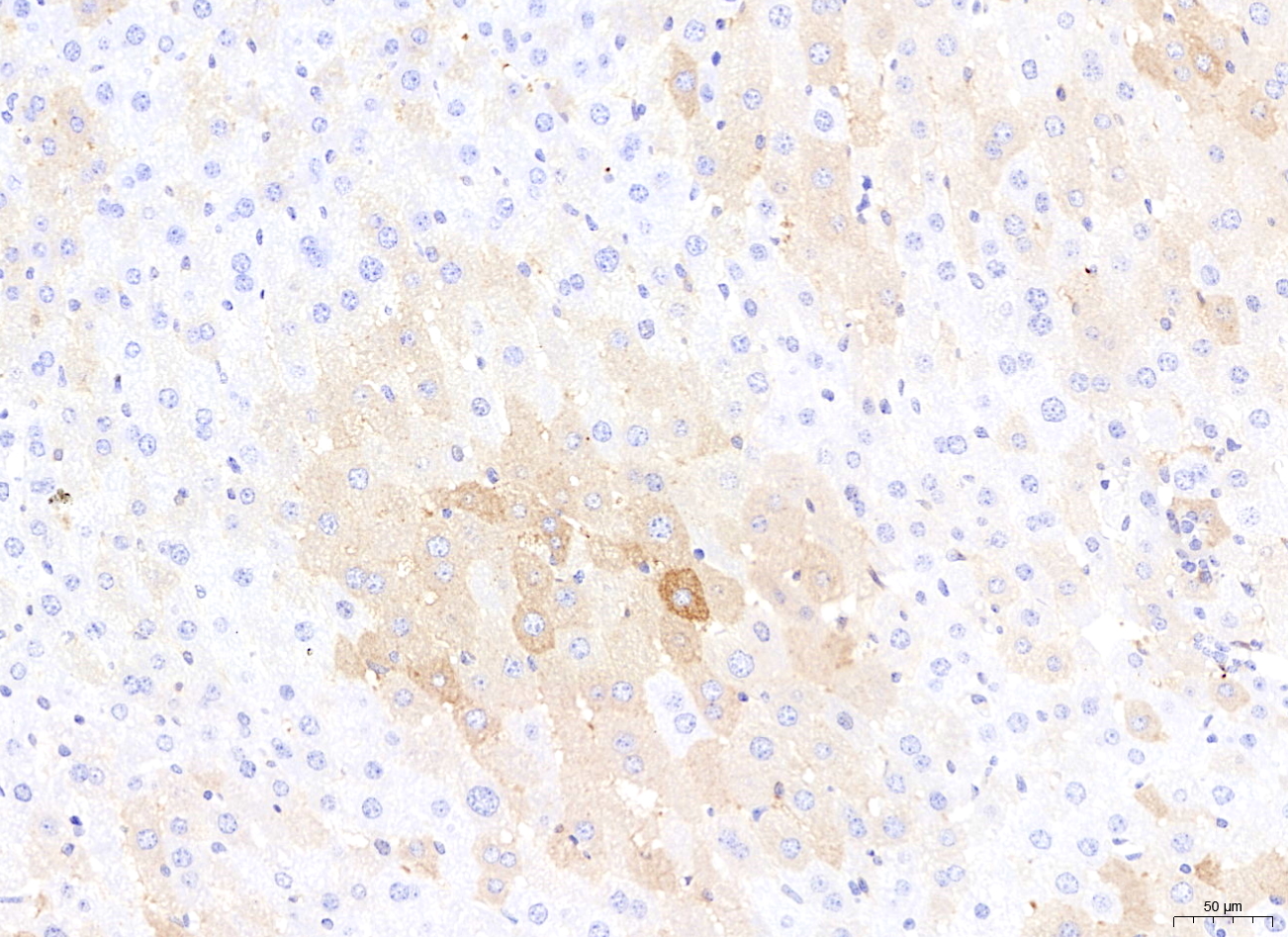

Supplement: Supplementary file 3 — Supporting File 3: advs73867‐sup‐0003‐SupportingFiguresData.zip. [file ADVS-13-e19191-s003.zip › Supporting information Figure S1-S9/S2/Liver/SCRS 4week Model 744_20.0x-1.jpg]

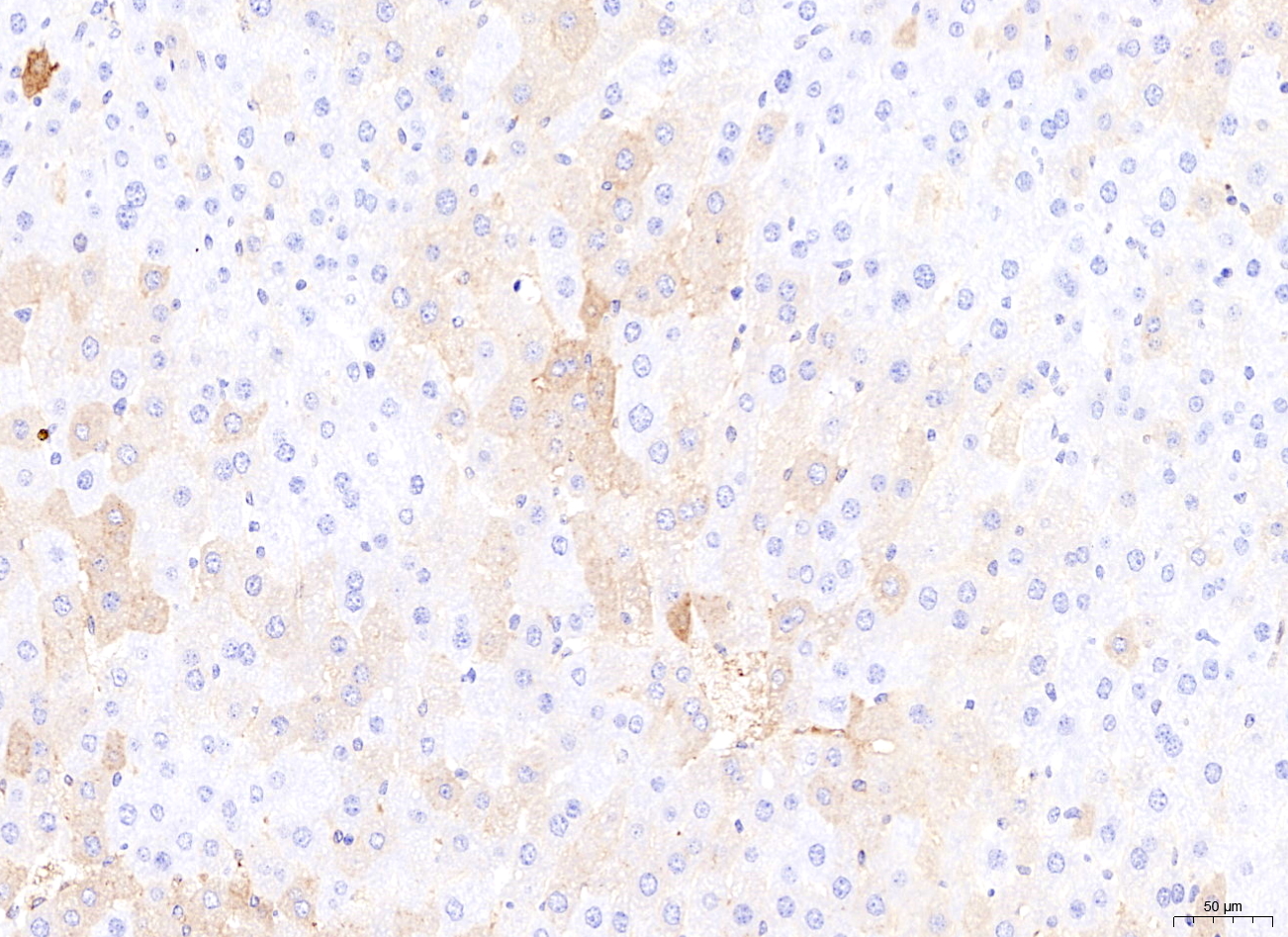

Supplement: Supplementary file 3 — Supporting File 3: advs73867‐sup‐0003‐SupportingFiguresData.zip. [file ADVS-13-e19191-s003.zip › Supporting information Figure S1-S9/S2/Liver/SCRS 4week Model 744_20.0x-2.jpg]
