# Supplementary material for: Macrophage–Derived Ferritin Exacerbates Silica‐Induced Pulmonary Fibrosis via PIK3R2‐Mediated Fibroblast Differentiation
Source: Adv Sci (Weinh). 2026 Jan 21;13(17):e19191. doi: 10.1002/advs.202519191 (PMC13042690; doi:10.1002/advs.202519191)
Supplement: Supplementary file 4 — Supporting File 4: advs73867‐sup‐0001‐FiguresData.zip. [file ADVS-13-e19191-s001.zip › Supporting information Figure1-10/Figure 10/Figure 10F-P.pdf]

Figure 10F-P

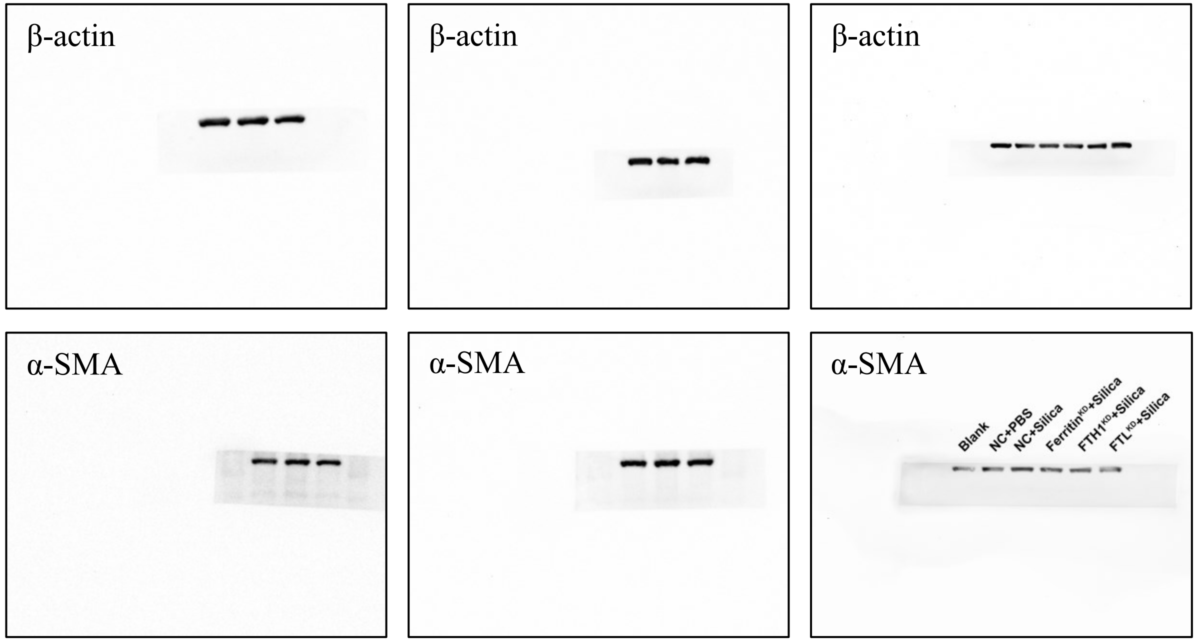

Explanation: In the initial experiments, to investigate the potential influence of transfection reagents on the results, we included a blank control (Blank) and a vector control (NC+PBS) in some biological replicates. Additionally, to examine whether co-transfecting two different siRNAs might lead to knockdown failure, we also separately set up FTH1 knockdown and FTL knockdown groups. Therefore, the immunoblotting results may show either 5 or 6 lanes, and the corresponding groups have been labeled accordingly in the original figures.

	1	actin	α-SMA			α-SMA/actin			Control mean	relative expression		
1	NC+PBS	4635386	922624	914467	948489	0.199039303	0.1972796	0.204619205	0.200312696	0.993642976	0.984858091	1.021498934
	NC+Silica	4356420	1201037	1134901	1185231	0.275693574	0.2605123	0.272065366	0.200312696	1.376316028	1.300528157	1.358203304
	Ferritin^{KD}+Silica	4187942	983028	986625	1010454	0.234728179	0.2355871	0.24127698	0.200312696	1.171808796	1.176096564	1.204501688
	1	actin	α-SMA			α-SMA/actin			Control mean	relative expression		
2	NC+PBS	4805806	4296887	4160832	3912035	0.894103299	0.8657928	0.814022663	0.857972905	1.042111347	1.009114328	0.948774325
	NC+Silica	3885009	4497458	4270499	4270113	1.157644165	1.099225	1.099125639	0.857972905	1.349278233	1.281188473	1.281072669
	Ferritin^{KD}+Silica	5363259	3979730	3817089	3544069	0.742035766	0.7117107	0.660805119	0.857972905	0.864870862	0.829525886	0.770193458
	1	actin	α-SMA			α-SMA/actin			Control mean	relative expression		
3	Blank											
	**NC+PBS**	16269664	3848942	3946668	3812145	0.236571696	0.2425783	0.234310002	0.237820011	0.994751009	1.020008089	0.985240901
	**NC+Silica**	15969234	4393460	4416883	4075034	0.275120272	0.276587	0.255180305	0.237820011	1.1568424	1.163009912	1.072997617
	**Ferritin^{KD}+Silica**	15259125	3210821	3263168	2929965	0.210419732	0.2138503	0.192013959	0.237820011	0.884785649	0.899210581	0.807391936
	FTH1^{KD}+Silica											
	FTL^{KD}+Silica											

Figure 10F-P

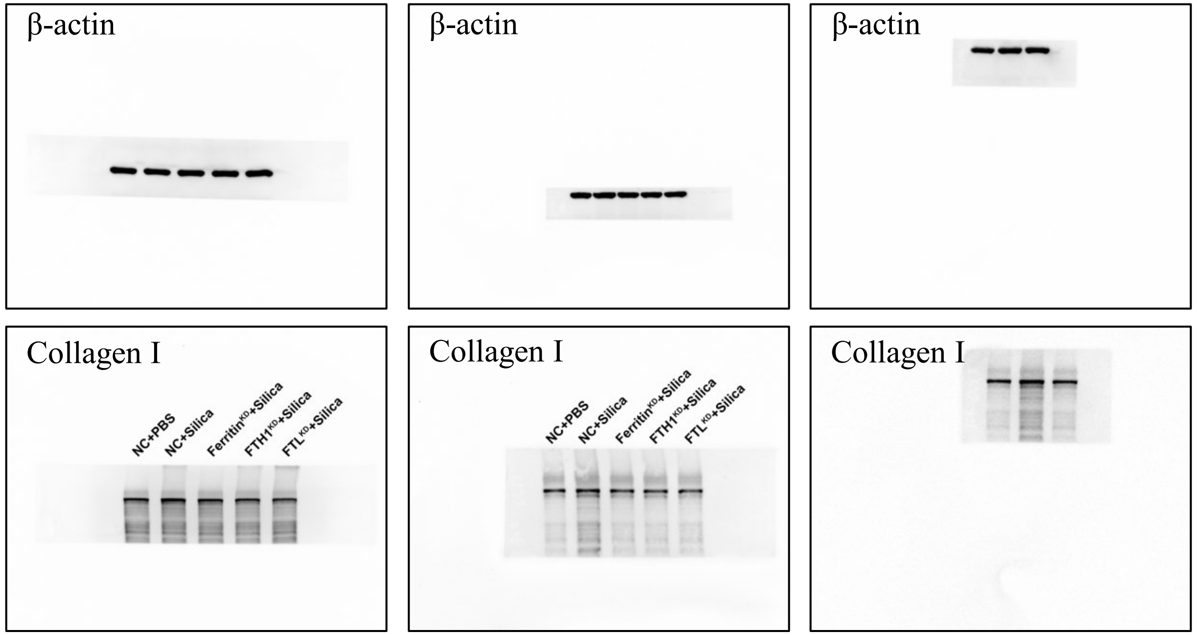

		actin	Collagen I			Collagen I/actin			Control mean	relative expression		
1	Blank											
	**NC+PBS**	19072722	4291400	4168492	3683126	0.225002	0.2185578	0.1931096	0.212223125	1.0602142	1.0298491	0.9099367
	**NC+Silica**	18320056	5496772	4896540	4572170	0.3000412	0.2672776	0.2495718	0.212223125	1.4138008	1.2594177	1.1759879
	**Ferritin^{KD}+Silica**	18860796	4323944	4170510	3798477	0.2292556	0.2211206	0.2013954	0.212223125	1.0802576	1.041925	0.9489794
	FTH1^{KD}+Silica											
	FTL^{KD}+Silica											
		actin	Collagen I			Collagen I/actin			Control mean	relative expression		
2	Blank											
	**NC+PBS**	13708218	3197576	2655490	2932219	0.2332598	0.1937152	0.2139023	0.213625749	1.0919086	0.906797	1.0012944
	**NC+Silica**	13627714	4291733	3894940	4268216	0.3149268	0.2858102	0.3132012	0.213625749	1.4741989	1.3379016	1.4661209
	**Ferritin^{KD}+Silica**	12279328	2939079	3108401	3123046	0.2393518	0.253141	0.2543336	0.213625749	1.1204257	1.1849741	1.190557
	FTH1^{KD}+Silica											
	FTL^{KD}+Silica											
		actin	Collagen I			Collagen I/actin			Control mean	relative expression		
3	**NC+PBS**	9789016	2225693	2054639	1941090	0.2273664	0.2098923	0.1982927	0.211850439	1.07324	0.9907569	0.9360031
	**NC+Silica**	10659062	3152046	3258120	3290967	0.2957151	0.3056667	0.3087483	0.211850439	1.3958675	1.4428418	1.4573879
	**Ferritin^{KD}+Silica**	11710321	2274301	2232609	2066791	0.1942134	0.1906531	0.1764931	0.211850439	0.9167476	0.899942	0.8331024

Figure 10F-P

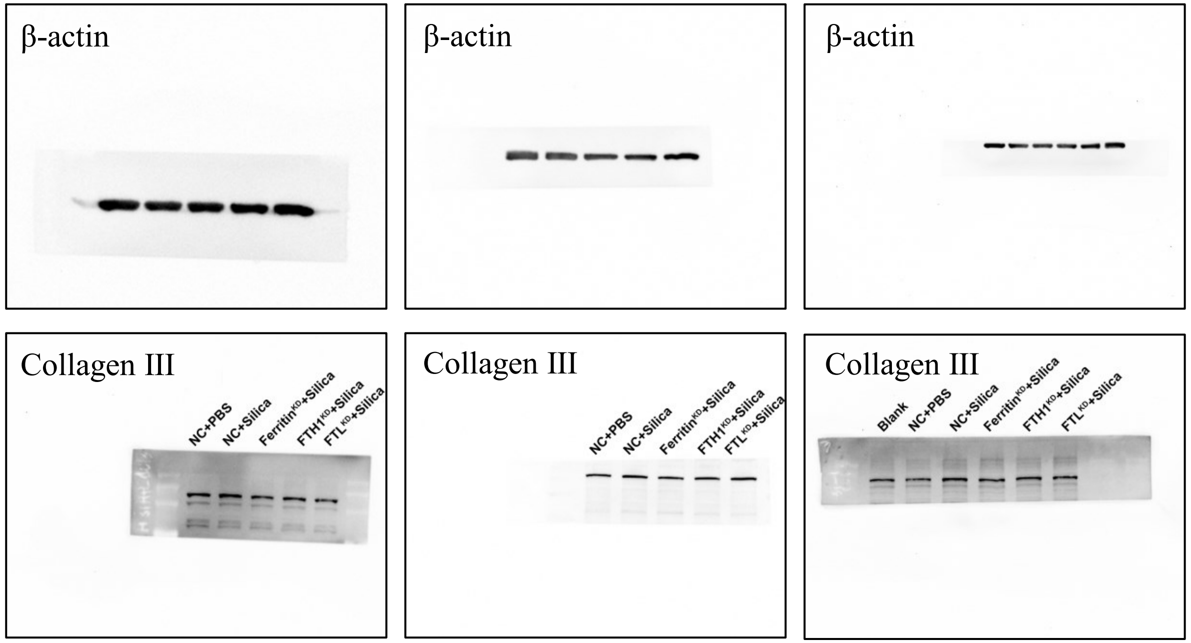

		actin	Collagen III			Collagen III/actin			Control mean	relative expression		
1	Blank											
	**NC+PBS**	3594453	2768524	2307634	2696388	0.770221227	0.641998657	0.750152527	0.720790804	1.068578044	0.890686527	1.04073543
	**NC+Silica**	2431424	3365741	3200177	3223918	1.384267409	1.316173979	1.325938216	0.720790804	1.920484282	1.826013834	1.8395604
	**Ferritin^{KD}+Silica**	2826150	2308440	1976730	2125054	0.816814394	0.699442705	0.751925411	0.720790804	1.133219777	0.970382393	1.04319507
	FTH1^{KD}+Silica											
	FTL^{KD}+Silica											
		actin	Collagen III			Collagen III/actin			Control mean	relative expression		
2	Blank											
	**NC+PBS**	8681601	2902913	2724713	2692838	0.334375307	0.313849139	0.310177581	0.319467342	1.046665066	0.982413841	0.97092109
	**NC+Silica**	7179132	4005348	3884184	3991421	0.557915358	0.541038109	0.55597543	0.319467342	1.74639246	1.693563118	1.74032008
	**Ferritin^{KD}+Silica**	6355118	2732123	2606513	2565777	0.429909091	0.410143919	0.403733967	0.319467342	1.345705909	1.283836762	1.26377226
	FTH1^{KD}+Silica											
	FTL^{KD}+Silica											
		actin (230	Collagen III			Collagen III/actin			Control mean	relative expression		
3	Blank											
	**NC+PBS**	7761187	2698106	2457029	2730572	0.347640896	0.316579023	0.351824019	0.338681313	1.026454318	0.934740157	1.03880552
	**NC+Silica**	7124218	4663714	4524253	4824856	0.654628199	0.635052577	0.677247103	0.338681313	1.932873693	1.875074158	1.99965891
	**Ferritin^{KD}+Silica**	7014468	3467197	3439807	3725672	0.494292226	0.490387439	0.531141064	0.338681313	1.459461173	1.44793179	1.56826209
	FTH1^{KD}+Silica											
	FTL^{KD}+Silica											

Figure 10F-P

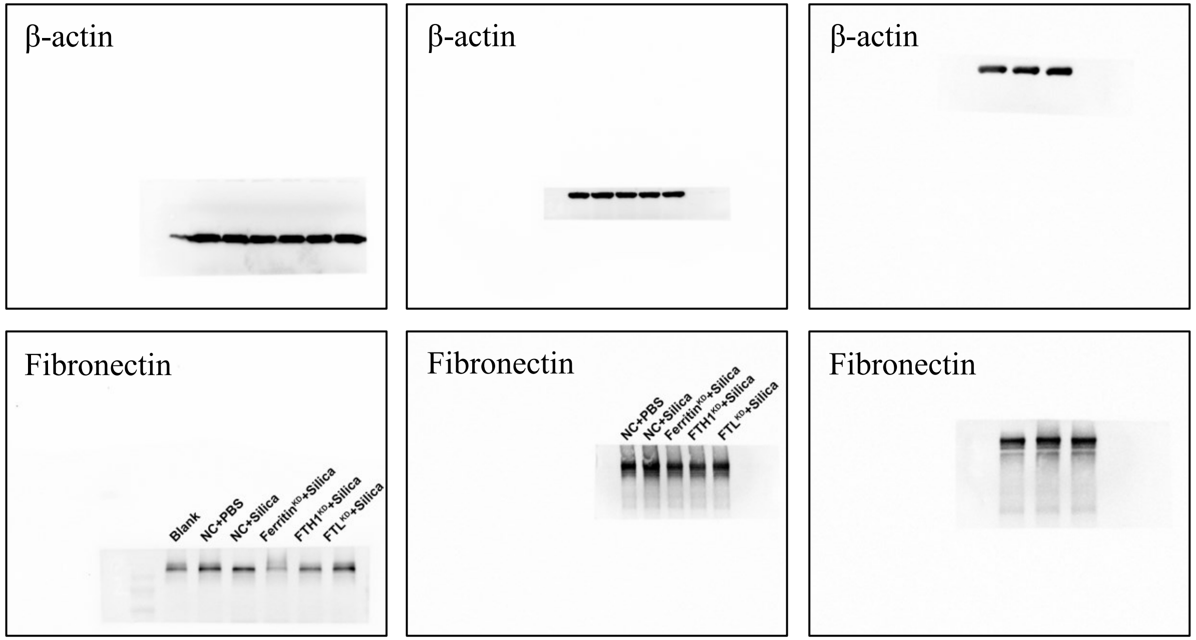

		actin	Fibronectin			Fibronectin/actin			Control mean	relative expression		
1	Blank											
	**NC+PBS**	14227849	3507914	3452756	2952899	0.246552659	0.242675896	0.2075436	0.232257385	1.061549277	1.044857609	0.893593115
	**NC+Silica**	12909671	4176865	3943078	3760094	0.323545426	0.305435979	0.2912618	0.232257385	1.393046882	1.315075425	1.254047527
	**Ferritin^{KD}+Silica**	13201942	1156691	1088757	1103144	0.087615216	0.082469458	0.08355922	0.232257385	0.377233284	0.355077872	0.359769925
	FTH1^{KD}+Silica											
	FTL^{KD}+Silica											
		actin	Fibronectin			Fibronectin/actin			Control mean	relative expression		
2	Blank											
	**NC+PBS**	13708218	4406025	5029145	5539693	0.321414862	0.366870807	0.40411474	0.364133471	0.882684202	1.007517398	1.1097984
	**NC+Silica**	13627714	7766999	6627587	7096241	0.569941444	0.48633153	0.5207213	0.364133471	1.565199274	1.335585902	1.430028672
	**Ferritin^{KD}+Silica**	12279328	4662358	4923797	4958502	0.379691625	0.400982611	0.40380891	0.364133471	1.042726514	1.101196794	1.108958494
	FTH1^{KD}+Silica											
	FTL^{KD}+Silica											
		actin	Fibronectin			Fibronectin/actin			Control mean	relative expression		
3	**NC+PBS**	3344065	3127435	2704157	2958651	0.935219561	0.808643672	0.88474686	0.876203363	1.067354452	0.922894964	1.009750584
	**NC+Silica**	3568416	3894006	4398193	4026399	1.091242165	1.232533707	1.1283435	0.876203363	1.245421109	1.406675389	1.287764402
	**Ferritin^{KD}+Silica**	4537197	3484520	2672872	2949086	0.767989576	0.589102038	0.64997971	0.876203363	0.876496951	0.672334829	0.741813761

Figure 10F-P

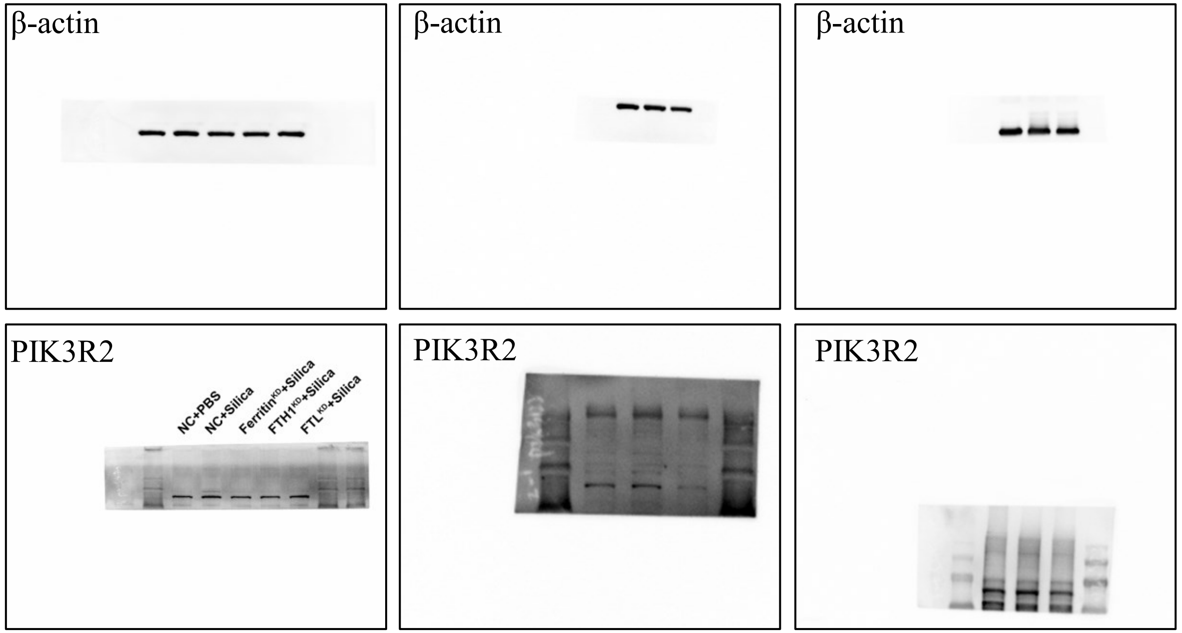

		actin	PIK3R2			PIK3R2/actin			Control mean	relative expression		
1	Blank											
	**NC+PBS**	13861068	1640363	1408147	1494147	0.11834319	0.10159008	0.10779451	0.10924259	1.08330631	0.92994937	0.98674432
	**NC+Silica**	14516204	2165129	2049882	2138979	0.14915256	0.14121336	0.14735113	0.10924259	1.36533341	1.29265849	1.34884318
	**Ferritin^{KD}+Silica**	13360833	1482055	1266926	1320504	0.11092534	0.09482388	0.09883396	0.10924259	1.01540381	0.86801197	0.90471999
	FTH1^{KD}+Silica											
	FTL^{KD}+Silica											
		actin	PIK3R2			PIK3R2/actin			Control mean	relative expression		
2	**NC+PBS**	4854192	539336	539336	558446	0.11110727	0.11110727	0.11504407	0.11241953	0.98832705	0.98832705	1.0233459
	**NC+Silica**	4089754	668248	686900	668248	0.16339565	0.16795631	0.16339565	0.11241953	1.45344534	1.4940136	1.45344534
	**Ferritin^{KD}+Silica**	3360980	176918	220530	194566	0.05263881	0.06561479	0.05788966	0.11241953	0.46823548	0.58366005	0.5149431
		actin	PIK3R2			PIK3R2/actin			Control mean	relative expression		
3	**NC+PBS**	10938785	3130118	3374171	2656402	0.2861486	0.30845939	0.24284251	0.27915017	1.02507049	1.10499448	0.86993503
	**NC+Silica**	9826130	4050818	4663322	3891235	0.41224958	0.47458379	0.39600891	0.27915017	1.47680221	1.70010211	1.41862321
	**Ferritin^{KD}+Silica**	9951255	2686785	2321454	2425013	0.26999459	0.23328254	0.24368916	0.27915017	0.96720196	0.83568832	0.872968

Figure 10F-P

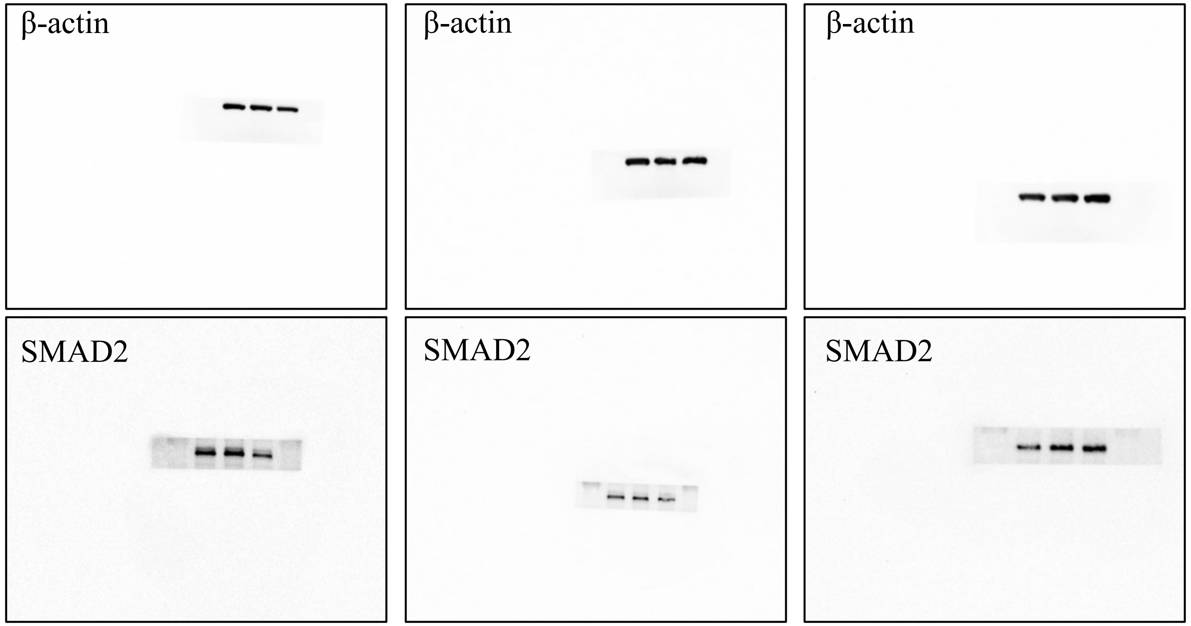

		actin	SMAD2			SMAD2/actin			Control mean	relative expression		
1	**NC+PBS**	4854192	598929	616775	695324	0.12338387	0.12706028	0.14324196	0.13122871	0.94022013	0.96823542	1.09154445
	**NC+Silica**	4089754	802562	954097	977021	0.19623723	0.23328958	0.23889481	0.13122871	1.49538342	1.77773286	1.82044628
	**Ferritin^{KD}+Silica**	3360980	365840	443555	487924	0.1088492	0.13197192	0.14517313	0.13122871	0.82946185	1.00566354	1.1062605
		actin	SMAD2			SMAD2/actin			Control mean	relative expression		
2	**NC+PBS**	4805806	1021044	1006990	912232	0.21246051	0.20953613	0.18981873	0.20393846	1.04178738	1.02744786	0.93076477
	**NC+Silica**	3885009	1130953	1213194	1128418	0.29110692	0.31227572	0.29045441	0.20393846	1.42742532	1.53122529	1.42422578
	**Ferritin^{KD}+Silica**	5363259	765189	871694	822314	0.14267239	0.16253066	0.15332357	0.20393846	0.69958553	0.79695932	0.75181292
		actin	SMAD2			SMAD2/actin			Control mean	relative expression		
3	**NC+PBS**	7611742	605371	676750	649036	0.0795312	0.08890869	0.08526774	0.08456921	0.94042742	1.05131276	1.00825982
	**NC+Silica**	8703734	1098991	1151148	1196257	0.12626661	0.1322591	0.13744182	0.08456921	1.49305655	1.5639155	1.62519925
	**Ferritin^{KD}+Silica**	11433917	1151074	965896	1058628	0.10067189	0.08447639	0.09258664	0.08456921	1.19040828	0.99890242	1.09480324

Figure 10F-P

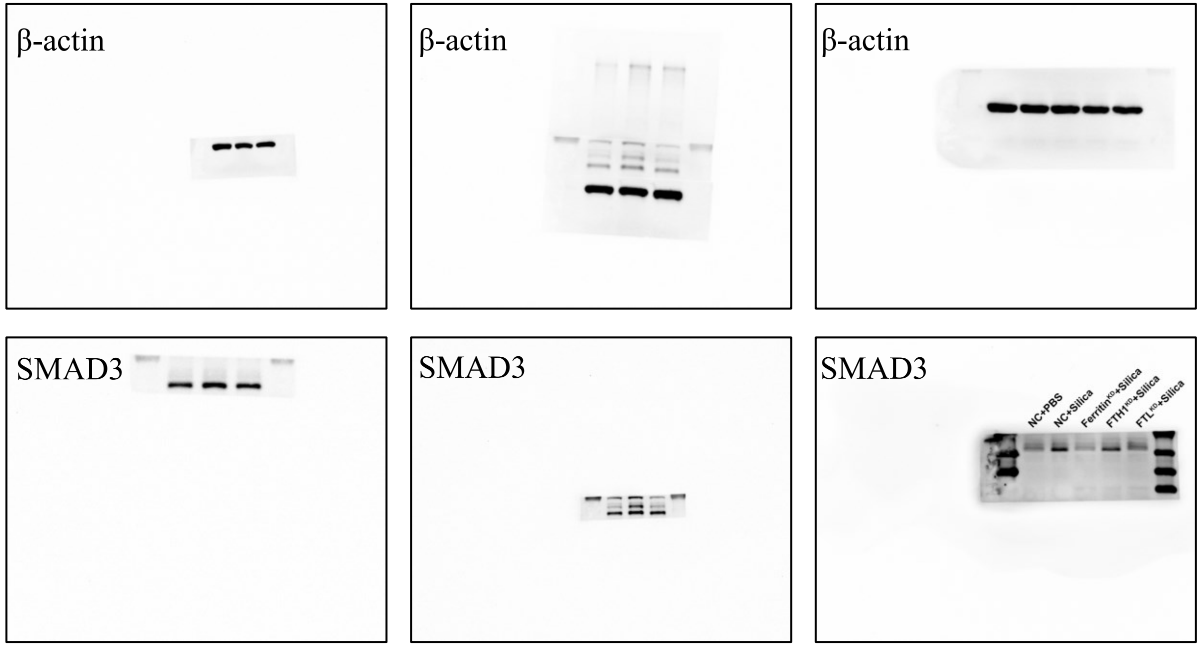

		actin	SMAD3			SMAD3/actin			Control mean	relative expression		
1	**NC+PBS**	8848307	2222828	2033897	2194396	0.25121506	0.22986284	0.2480018	0.24302657	1.0336938	0.94583422	1.02047195
	**NC+Silica**	5018692	3087663	2981982	3157363	0.61523261	0.59417514	0.6291207	0.24302657	2.5315447	2.44489788	2.58869104
	**Ferritin^{KD}+Silica**	8469293	2516003	2336139	2485989	0.29707356	0.27583637	0.2935297	0.24302657	1.2223913	1.13500499	1.20780909
		actin	SMAD3			SMAD3/actin			Control mean	relative expression		
2	**NC+PBS**	11753335	1210077	1143021	1164649	0.10295605	0.09725078	0.0990909	0.09976592	1.0319761	0.97478955	0.99323431
	**NC+Silica**	11936959	1986695	2046343	2012047	0.16643225	0.17142917	0.1685561	0.09976592	1.6682275	1.71831387	1.68951553
	**Ferritin^{KD}+Silica**	14885609	1494879	1533408	1695799	0.10042444	0.10301278	0.113922	0.09976592	1.0066006	1.03254476	1.14189333
		actin	SMAD3			SMAD3/actin			Control mean	relative expression		
3	Blank											
	**NC+PBS**	14932431	2155329	2155329	2054833	0.14433879	0.14433879	0.1376087	0.14209544	1.0157876	1.01578763	0.96842475
	**NC+Silica**	12555503	3735073	4806906	5816009	0.29748494	0.38285252	0.4632239	0.14209544	2.0935572	2.69433366	3.25994908
	**Ferritin^{KD}+Silica**	12257522	1466012	1412706	1416677	0.11960101	0.11525217	0.1155761	0.14209544	0.8416949	0.81108986	0.81336977
	FTH1^{KD}+Silica											
	FTL^{KD}+Silica											

Figure 10F-P

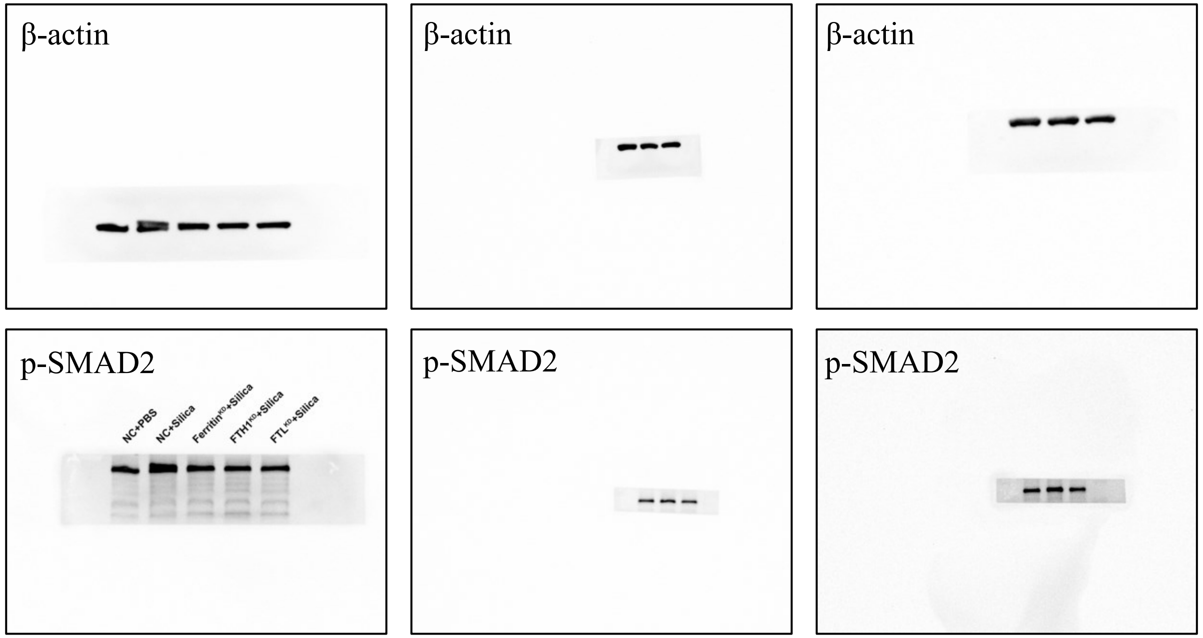

		actin	p-SMAD2			p-SMAD2/actin			Control mean	relative expression		
1	Blank											
	**NC+PBS**	10907569	6024681	6568804	6304978	0.5523395	0.6022244	0.57803696	0.57753361	0.9563763	1.0427521	1.000872
	**NC+Silica**	10046942	10317038	10547120	10724329	1.0268834	1.0497841	1.06742221	0.57753361	1.7780496	1.81770219	1.848243
	**Ferritin^{KD}+Silica**	9142326	7353631	8222385	7353631	0.8043501	0.8993756	0.80435012	0.57753361	1.392733	1.55726974	1.392733
	FTH1^{KD}+Silica											
	FTL^{KD}+Silica											
		actin	p-SMAD2			p-SMAD2/actin			Control mean	relative expression		
2	**NC+PBS**	8848307	2308645	2261415	2189040	0.2609138	0.255576	0.24739648	0.25462875	1.024683	1.00372017	0.971597
	**NC+Silica**	5018692	2689220	2600578	2786523	0.5358408	0.5181784	0.55522893	0.25462875	2.1044003	2.03503511	2.180543
	**Ferritin^{KD}+Silica**	8469293	1948135	2183033	2216483	0.2300233	0.2577586	0.26170815	0.25462875	0.9033675	1.01229178	1.027803
		actin	p-SMAD2			p-SMAD2/actin			Control mean	relative expression		
3	**NC+PBS**	4635386	971709	961662	932136	0.2096285	0.207461	0.20109134	0.20606029	1.0173163	1.00679775	0.975886
	**NC+Silica**	4356420	1539942	1580716	1592269	0.353488	0.3628475	0.36549942	0.20606029	1.7154589	1.76088013	1.77375
	**Ferritin^{KD}+Silica**	4187942	903820	873359	891767	0.2158148	0.2085413	0.21293681	0.20606029	1.0473383	1.01204035	1.033371

Figure 10F-P

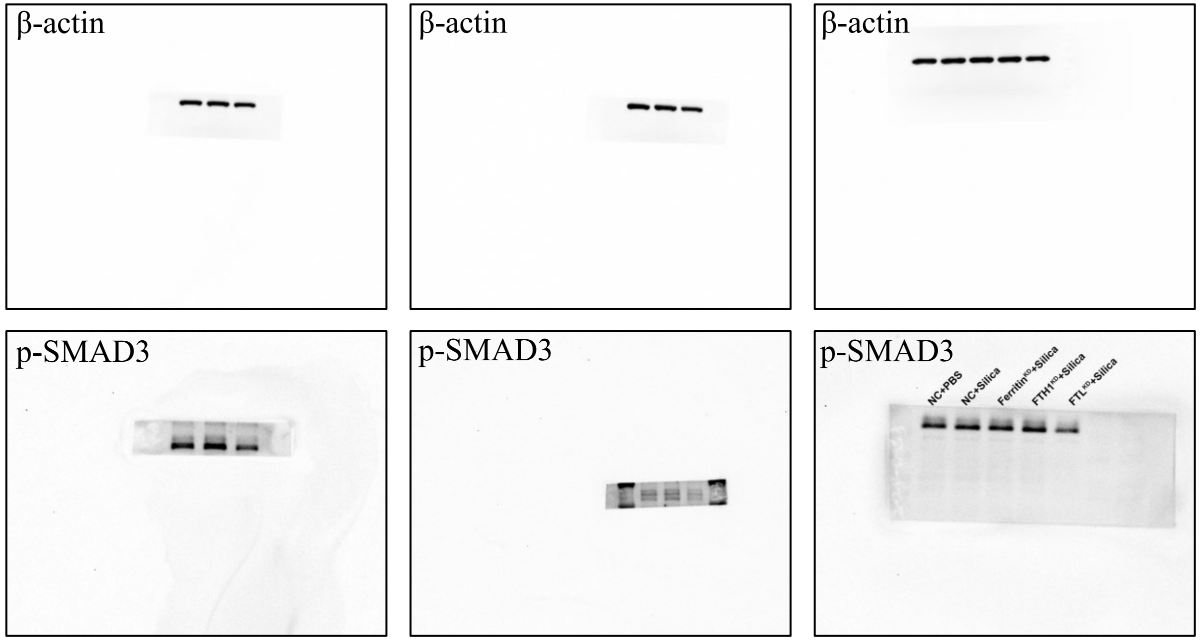

		actin	p-SMAD3			p-SMAD3/actin			Control mean	relative expression		
1	**NC+PBS**	4244200	1211810	1021855	1063590	0.285521	0.24076504	0.25059846	0.25896164	1.10256259	0.92973246	0.967705
	**NC+Silica**	4113183	1523342	1674968	1987676	0.370356	0.40721942	0.48324521	0.25896164	1.43015775	1.57250865	1.866088
	**Ferritin^{KD}+Silica**	3641747	923078	853651	951203	0.253471	0.234407	0.26119415	0.25896164	0.97879826	0.9051804	1.008621
		actin	p-SMAD3			p-SMAD3/actin			Control mean	relative expression		
2	**NC+PBS**	4854192	888271	1007091	1076525	0.18299	0.20746831	0.22177223	0.20407701	0.89667373	1.01661772	1.086709
	**NC+Silica**	4089754	1113394	1221051	1229731	0.27224	0.29856343	0.30068581	0.20407701	1.33400545	1.46299395	1.473394
	**Ferritin^{KD}+Silica**	3360980	520947	545427	537486	0.154999	0.16228213	0.15991943	0.20407701	0.75951005	0.79520045	0.783623
		actin	p-SMAD3			p-SMAD3/actin			Control mean	relative expression		
3	Blank											
	**NC+PBS**	9146679	1153014	1027955	881083	0.126058	0.1123856	0.09632819	0.11159067	1.12964835	1.00712365	0.863228
	**NC+Silica**	9317924	1351024	1449222	1565877	0.144992	0.15553057	0.16804999	0.11159067	1.29931972	1.39375964	1.50595
	**Ferritin^{KD}+Silica**	9395309	1170583	1022600	1016125	0.124592	0.10884155	0.10815238	0.11159067	1.11651169	0.97536429	0.969188
	FTH1^{KD}+Silica											
	FTL^{KD}+Silica											
