# Supplementary material for: Macrophage–Derived Ferritin Exacerbates Silica‐Induced Pulmonary Fibrosis via PIK3R2‐Mediated Fibroblast Differentiation
Source: Adv Sci (Weinh). 2026 Jan 21;13(17):e19191. doi: 10.1002/advs.202519191 (PMC13042690; doi:10.1002/advs.202519191)
Supplement: Supplementary file 4 — Supporting File 4: advs73867‐sup‐0001‐FiguresData.zip. [file ADVS-13-e19191-s001.zip › Supporting information Figure1-10/Figure 2/Figure D-H.pdf]

Figure 2D

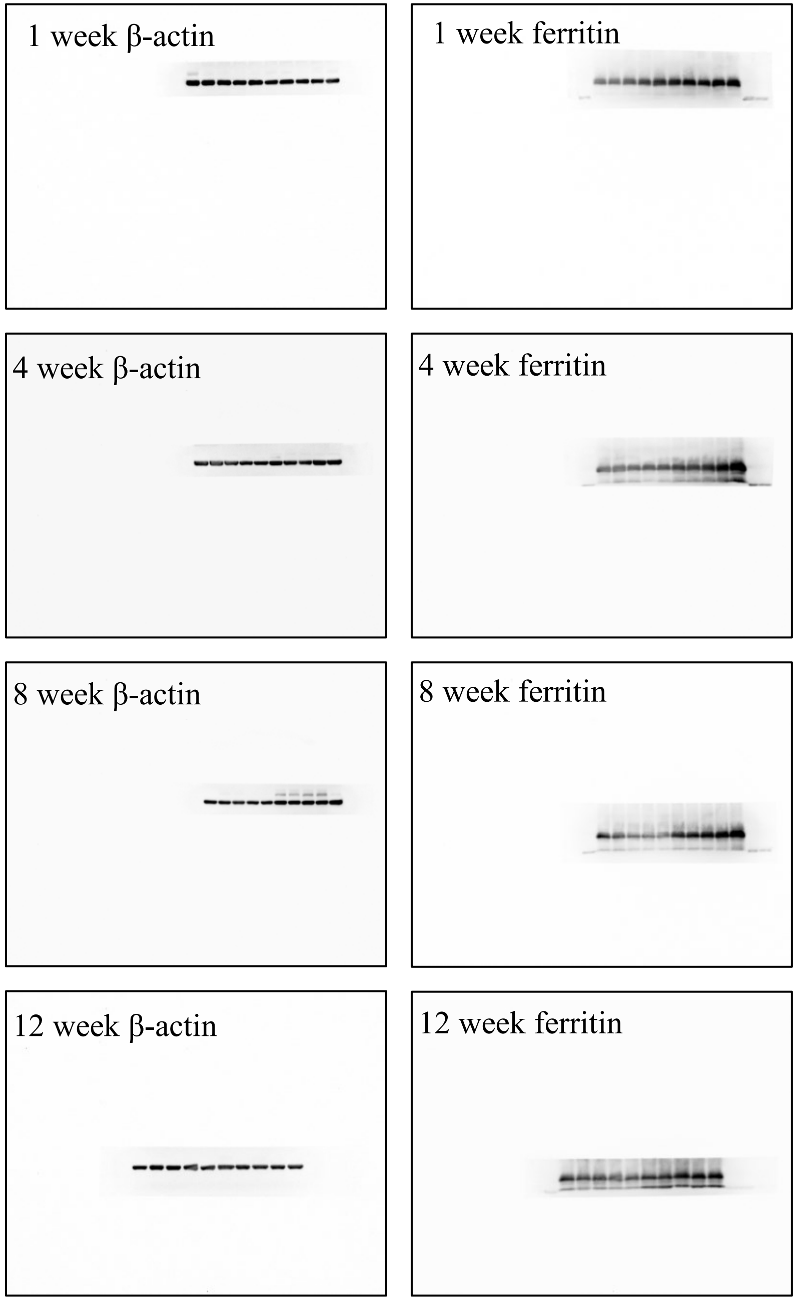

		β-actin	Ferritin	Ferritin/β-actin	Control mean	Relative expression	Student *t*-Test
1 week Control	1#	6948171	6483716	0.933154351	1.422777653	0.655868012	0.000411
	2#	6720194	6762603	1.006310681	1.422777653	0.707285976	
	3#	6437016	8544667	1.327426714	1.422777653	0.932982544	
	4#	5682157	9881609	1.73905948	1.422777653	1.222298844	
	5#	5893825	12423812	2.107937036	1.422777653	1.481564623	
1 week Silica	1#	4064710	13129296	3.23006955	1.422777653	2.270256033	
	2#	4740173	14508757	3.060807485	1.422777653	2.151290105	
	3#	3942541	13485692	3.420558467	1.422777653	2.404141266	
	4#	3504135	15840387	4.520484228	1.422777653	3.177224649	
	5#	3123760	14682296	4.700199759	1.422777653	3.303537802	

		β-actin	Ferritin	Ferritin/β-actin	Control mean	Relative expression	Student *t*-Test
4 week Control	1#	3081358	5813752	1.886749933	2.035453154	0.926943432	0.013866
	2#	2789985	5868832	2.103535324	2.035453154	1.033448163	
	3#	2755071	5894061	2.139349948	2.035453154	1.051043569	
	4#	3109787	6392896	2.055734364	2.035453154	1.009963978	
	5#	3431354	6834901	1.991896202	2.035453154	0.978600857	
4 week Silica	1#	4113746	8291097	2.015461577	2.035453154	0.990178316	
	2#	3273431	8649751	2.64241128	2.035453154	1.29819312	
	3#	3260813	9931425	3.045689833	2.035453154	1.49632028	
	4#	4648657	11922674	2.56475666	2.035453154	1.260042097	
	5#	4750586	15001066	3.157729594	2.035453154	1.551364416	

		β-actin	Ferritin	Ferritin/β-actin	Control mean	Relative expression	Student *t*-Test
8 week Control	1#	475680	631459	1.327486966	1.098027755	1.208973963	0.001893
	2#	443407	537001	1.211079212	1.098027755	1.102958651	
	3#	408744	417102	1.020448007	1.098027755	0.929346277	
	4#	427578	413211	0.966399113	1.098027755	0.880122664	
	5#	446101	430365	0.964725477	1.098027755	0.878598444	
8 week Silica	1#	573390	772266	1.346842463	1.098027755	1.226601475	
	2#	556194	820647	1.475468991	1.098027755	1.343744714	
	3#	593265	946309	1.595086513	1.098027755	1.452683237	
	4#	589591	1035899	1.756978991	1.098027755	1.600122568	
	5#	670351	1160227	1.73077537	1.098027755	1.576258307	

		β-actin	Ferritin	Ferritin/β-actin	Control mean	Relative expression	Student *t*-Test
12 week Control	1#	2780463	12769260	4.592494128	4.004209105	1.146916659	0.00063
	2#	3371434	11859014	3.517498489	4.004209105	0.87845025	
	3#	3380398	12489142	3.694577384	4.004209105	0.922673439	
	4#	2804967	10927131	3.895636205	4.004209105	0.972885307	
	5#	2480441	10717587	4.320839318	4.004209105	1.079074345	
12 week Silica	1#	2689726	13776594	5.121932122	4.004209105	1.279137025	
	2#	2625530	15071305	5.740290532	4.004209105	1.433564128	
	3#	2481389	17408550	7.015647285	4.004209105	1.752068161	
	4#	2473015	16799833	6.793259645	4.004209105	1.696529693	
	5#	2337546	16229263	6.942863584	4.004209105	1.733891363	
