# Supplementary material for: Macrophage–Derived Ferritin Exacerbates Silica‐Induced Pulmonary Fibrosis via PIK3R2‐Mediated Fibroblast Differentiation
Source: Adv Sci (Weinh). 2026 Jan 21;13(17):e19191. doi: 10.1002/advs.202519191 (PMC13042690; doi:10.1002/advs.202519191)
Supplement: Supplementary file 4 — Supporting File 4: advs73867‐sup‐0001‐FiguresData.zip. [file ADVS-13-e19191-s001.zip › Supporting information Figure1-10/Figure 3/Figure 3F-I.pdf]

# Figure 3F-I

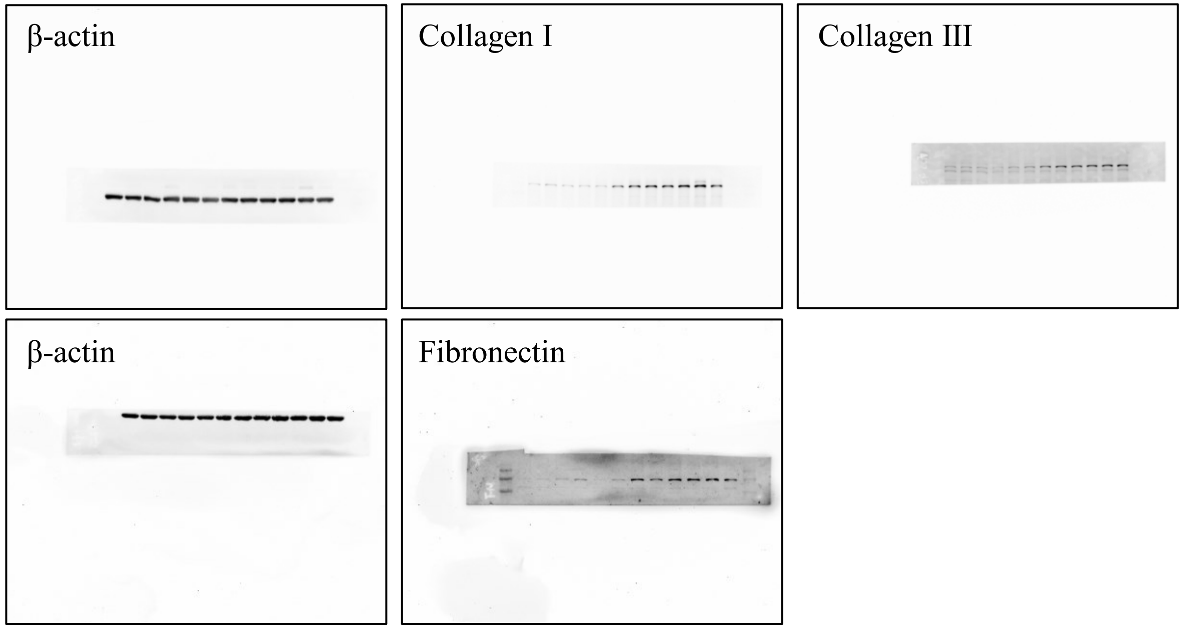


| | | actin | Collagen I | Collagen I/actin | Control mean | relative expression |
|---|---|---|---|---|---|---|
| Control | 1# | 7791498 | 258468 | 0.033173082 | 0.053550702 | 0.619470528 |
| | 2# | 5995601 | 446720 | 0.07450796 | 0.053550702 | 1.391353559 |
| | 3# | 6019475 | 318858 | 0.052971065 | 0.053550702 | 0.989175913 |
| Ferritin | 1# | 5770714 | 343678 | 0.059555542 | 0.053550702 | 1.112133727 |
| | 2# | 6253198 | 362370 | 0.057949548 | 0.053550702 | 1.082143576 |
| | 3# | 4845662 | 1386877 | 0.286210016 | 0.053550702 | 5.344654777 |
| Silica | 1# | 5506486 | 2342061 | 0.425327695 | 0.053550702 | 7.942523216 |
| | 2# | 6053467 | 2874961 | 0.474928004 | 0.053550702 | 8.868753996 |
| | 3# | 5803465 | 2607038 | 0.44922094 | 0.053550702 | 8.388703064 |
| Silica+Ferritin | 1# | 6311697 | 3510098 | 0.556125872 | 0.053550702 | 10.38503417 |
| | 2# | 5267184 | 3908306 | 0.742010532 | 0.053550702 | 13.85622413 |
| | 3# | 5596506 | 2424554 | 0.433226374 | 0.053550702 | 8.090022278 |

| | | actin | Fibronectin | Fibronectin/actin | Control mean | relative expression |
|---|---|---|---|---|---|---|
| Control | 1# | 6358427 | 169403 | 0.026642281 | 0.039227766 | 0.679168965 |
| | 2# | 5687877 | 146134 | 0.025692187 | 0.039227766 | 0.654949026 |
| | 3# | 4911381 | 320953 | 0.06534883 | 0.039227766 | 1.66588201 |
| Ferritin | 1# | 5008497 | 618947 | 0.123579389 | 0.039227766 | 3.150304031 |
| | 2# | 4768333 | 93532 | 0.019615241 | 0.039227766 | 0.500034614 |
| | 3# | 5333016 | 404532 | 0.075854263 | 0.039227766 | 1.933688078 |
| Silica | 1# | 6475361 | 1887473 | 0.29148537 | 0.039227766 | 7.43058809 |
| | 2# | 5900389 | 1250267 | 0.21189569 | 0.039227766 | 5.401676214 |
| | 3# | 6979666 | 2112447 | 0.302657319 | 0.039227766 | 7.715385054 |
| Silica+Ferritin | 1# | 6323724 | 2163941 | 0.342194093 | 0.039227766 | 8.723262336 |
| | 2# | 7195731 | 2288903 | 0.318091796 | 0.039227766 | 8.108843026 |
| | 3# | 6544556 | 1510737 | 0.230838731 | 0.039227766 | 5.884574997 |

| | | actin | Collagen III | | | |
|---|---|---|---|---|---|---|
| Control | 1# | 7791498 | 1399280 | 0.179590626 | 0.189435701 | 0.948029461 |
| | 2# | 5995601 | 1241893 | 0.20713403 | 0.189435701 | 1.093426579 |
| | 3# | 6019475 | 1093031 | 0.181582447 | 0.189435701 | 0.95854396 |
| Ferritin | 1# | 5770714 | 732973 | 0.127015998 | 0.189435701 | 0.670496626 |
| | 2# | 6253198 | 1178535 | 0.188469164 | 0.189435701 | 0.99489781 |
| | 3# | 4845662 | 1316923 | 0.271773599 | 0.189435701 | 1.43464826 |
| Silica | 1# | 5506486 | 1826108 | 0.331628556 | 0.189435701 | 1.750612763 |
| | 2# | 6053467 | 2462365 | 0.406769377 | 0.189435701 | 2.147268835 |
| | 3# | 5803465 | 2157041 | 0.371681573 | 0.189435701 | 1.962046072 |
| Silica+Ferritin | 1# | 6311697 | 2387210 | 0.378219994 | 0.189435701 | 1.996561323 |
| | 2# | 5267184 | 2792019 | 0.530078121 | 0.189435701 | 2.798195476 |
| | 3# | 5596506 | 3369167 | 0.602012577 | 0.189435701 | 3.177925671 |
