# Supplementary material for: Macrophage–Derived Ferritin Exacerbates Silica‐Induced Pulmonary Fibrosis via PIK3R2‐Mediated Fibroblast Differentiation
Source: Adv Sci (Weinh). 2026 Jan 21;13(17):e19191. doi: 10.1002/advs.202519191 (PMC13042690; doi:10.1002/advs.202519191)
Supplement: Supplementary file 4 — Supporting File 4: advs73867‐sup‐0001‐FiguresData.zip. [file ADVS-13-e19191-s001.zip › Supporting information Figure1-10/Figure 4/Figure 4G-K.pdf]

Figure 4G-K

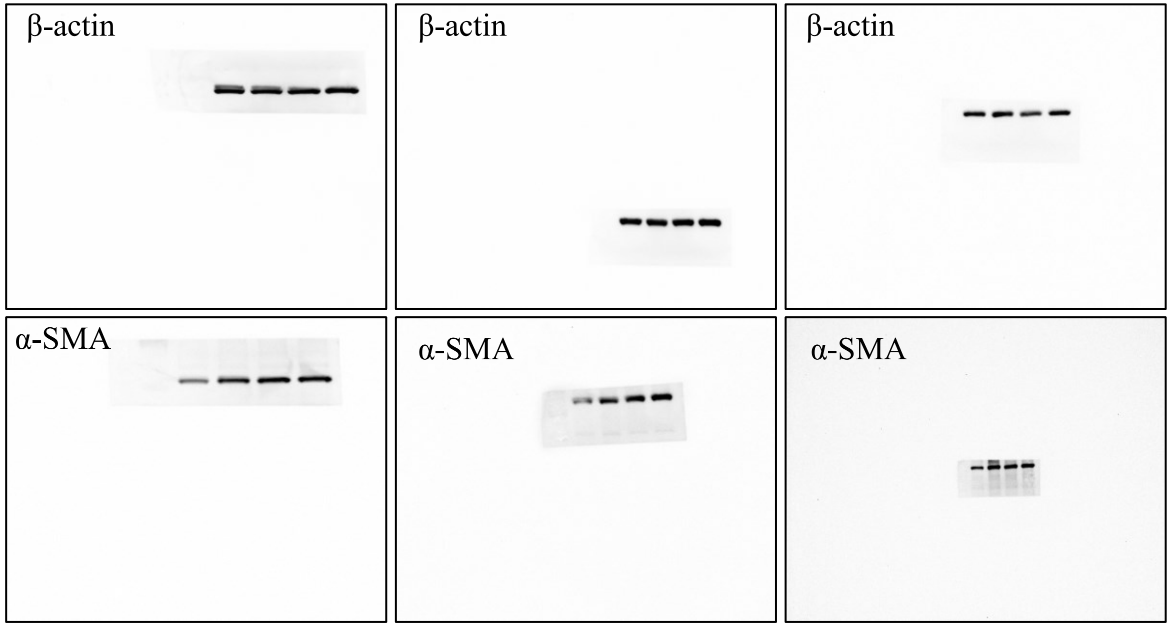

1		actin	α-SMA			α-SMA/actin			Control mean	relative expression		
Ferritin	0	20307461	8482409	7899540	8736647	0.41769914	0.388996931	0.430218578	0.412304883	1.013083176	0.943469133	1.043447691
	250	18520921	14619069	11515269	14727863	0.789327323	0.621743865	0.795201437	0.412304883	1.914426328	1.50797114	1.928673343
	500	17687924	16377293	13546034	15841168	0.925902497	0.765835154	0.89559227	0.412304883	2.245674342	1.857448663	2.17216023
	1000	19042964	16258346	12855787	16261879	0.853771818	0.675093804	0.853957346	0.412304883	2.07072934	1.637365531	2.071179317
2		actin	α-SMA			α-SMA/actin			Control mean	relative expression		
Ferritin	0	10932956	1089102	1105845	1295970	0.099616426	0.101147851	0.118537932	0.10643407	0.935944911	0.950333394	1.113721695
	250	10054283	1671902	1462804	1745152	0.166287541	0.145490633	0.173572994	0.105716148	1.572962539	1.376238496	1.641877766
	500	10800696	2876579	2848195	2719499	0.266332744	0.263704765	0.251789237	0.105716148	2.519319399	2.494460579	2.381748107
	1000	12116970	3141531	3152432	2754686	0.259267044	0.260166692	0.227341159	0.105716148	2.452482881	2.460992909	2.150486581
3		actin	α-SMA			α-SMA/actin			Control mean	relative expression		
Ferritin	0	2891008	839985	795929	703707	0.290550908	0.275311933	0.243412332	0.269758391	1.077078296	1.020587095	0.902334609
	250	3245792	1275109	1384407	1272941	0.392849881	0.426523634	0.392181939	0.269758391	1.456302728	1.581132037	1.453826654
	500	2454668	1414665	1468230	1414665	0.576316227	0.598137915	0.576316227	0.269758391	2.136416312	2.217309767	2.136416312
	1000	3440464	1674186	1860490	1747268	0.48661634	0.540767176	0.507858242	0.269758391	1.803896955	2.004635235	1.882641131

Figure 4G-K

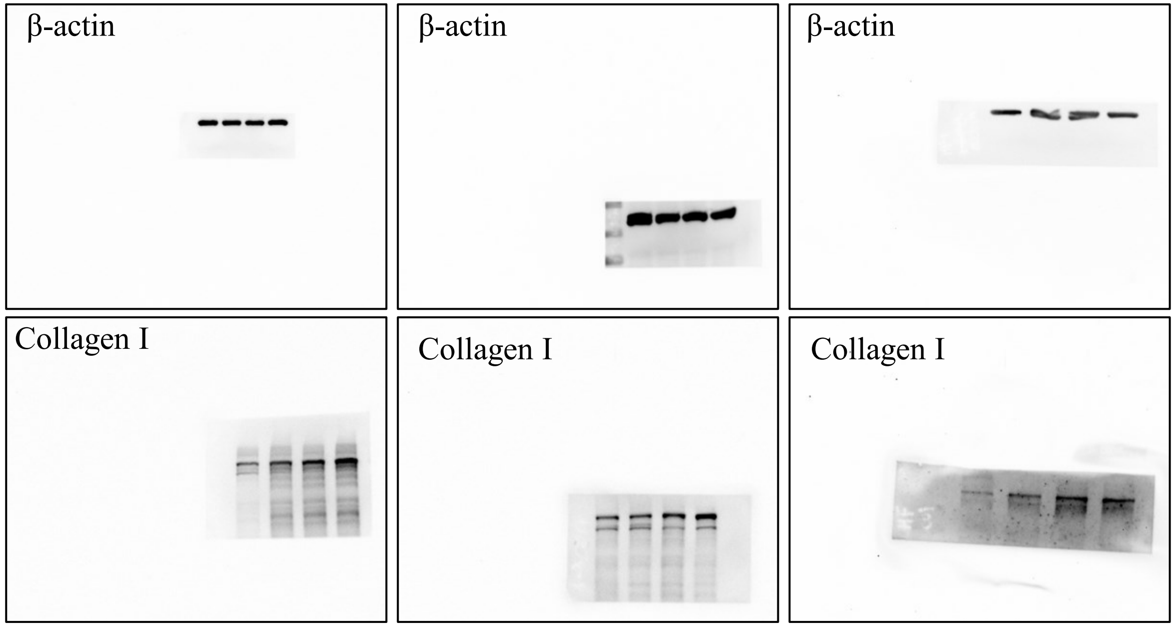

1		actin	Collagen I			Collagen I/actin			Control mean	relative expression		
Ferritin	0	7792681	970521	742145	706099	0.124543	0.095236	0.090611	0.103463109	1.20374	0.920484	0.875776
	250	6059997	1552182	1361516	1329904	0.256136	0.224673	0.219456	0.103463109	2.475624	2.171525	2.121106
	500	5809231	2063574	1800311	1947196	0.355223	0.309905	0.33519	0.103463109	3.433333	2.995321	3.239705
	1000	8307538	3592129	2829613	3567015	0.432394	0.340608	0.429371	0.103463109	4.179209	3.292071	4.14999
2		actin	Collagen I			Collagen I/actin			Control mean	relative expression		
Ferritin	0	18610397	1169964	1057321	1044605	0.062866	0.056813	0.05613	0.058603264	1.072741	0.969459	0.9578
	250	13136176	1177076	1182371	1047404	0.089606	0.090009	0.079734	0.058603264	1.529022	1.5359	1.360578
	500	13668230	2043970	1838429	1728223	0.149542	0.134504	0.126441	0.058603264	2.551764	2.295159	2.157574
	1000	15828023	3431987	3145289	3002804	0.21683	0.198716	0.189714	0.058603264	3.699961	3.390877	3.237267
3		actin	Collagen I			Collagen I/actin			Control mean	relative expression		
Ferritin	0	8315467	947801	845563	604839	0.11398	0.101686	0.072737	0.096134228	1.185639	1.057746	0.756615
	250	9852166	2016966	1852663	1566888	0.204723	0.188046	0.15904	0.096134228	2.129555	1.95608	1.654353
	500	9824997	2999001	2774772	2451277	0.305242	0.28242	0.249494	0.096134228	3.175164	2.937764	2.595266
	1000	7507420	3284535	2483094	2626578	0.437505	0.330752	0.349864	0.096134228	4.550982	3.440523	3.639331

Figure 4G-K

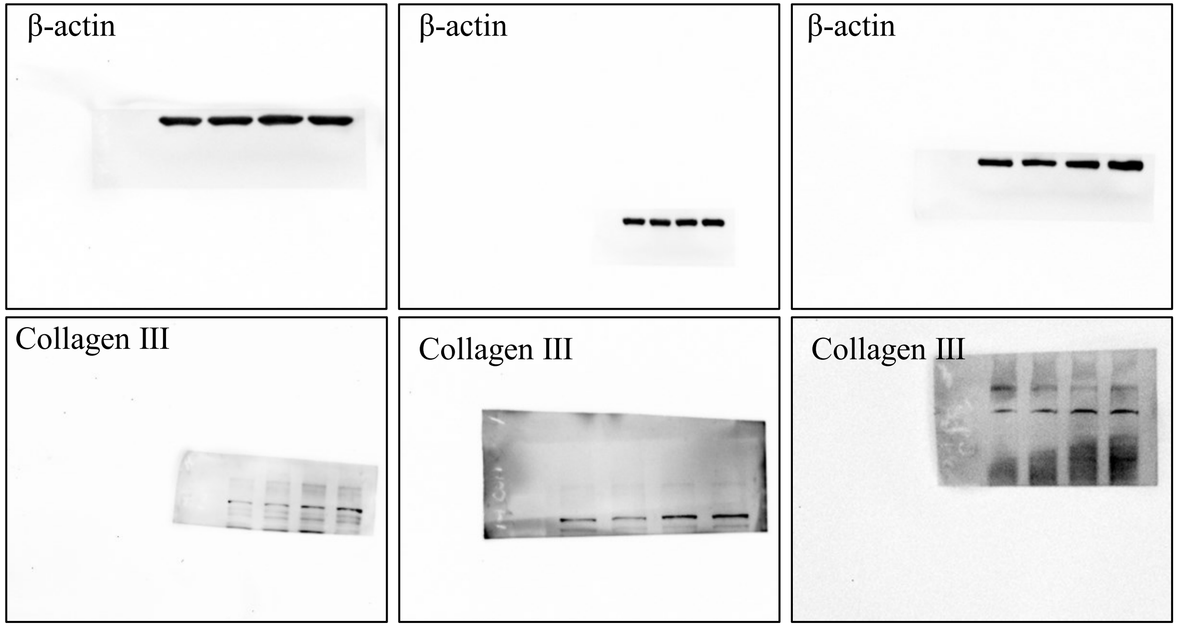

1		actin	Collagen III			Collagen III/actin			Control mean	tive expression		
Ferritin	0	9691406	1009974	996082	1112223	0.104213	0.10278	0.114764	0.107252377	0.971665	0.9583	1.070035
	250	10424699	1089630	962113	1233978	0.104524	0.092292	0.118371	0.107252377	0.97456	0.860509	1.103664
	500	10985527	2319528	2040630	2464174	0.211144	0.185756	0.224311	0.107252377	1.968665	1.731954	2.091431
	1000	12236489	4095124	3582728	4059059	0.334665	0.292791	0.331718	0.107252377	3.12035	2.729921	3.09287
2		actin	Collagen III			Collagen III/actin			Control mean	tive expression		
Ferritin	0	6712882	1386932	1357238	1419767	0.206608	0.202184	0.211499	0.206763503	0.999246	0.977852	1.022902
	250	5789754	1050068	948758	990906	0.181367	0.163868	0.171148	0.206763503	0.877169	0.79254	0.827749
	500	7428277	2778481	2842120	2831931	0.374041	0.382608	0.381237	0.206763503	1.809029	1.850463	1.843829
	1000	8922563	3336850	3375976	3085989	0.373979	0.378364	0.345864	0.206763503	1.808728	1.829936	1.672749
3		actin	Collagen III			Collagen III/actin			Control mean	tive expression		
Ferritin	0	12616057	536053	572986	518632	0.04249	0.045417	0.041109	0.043005275	0.988012	1.056084	0.955903
	250	12272119	663636	716870	687156	0.054077	0.058415	0.055993	0.043005275	1.257444	1.358311	1.302009
	500	13171549	1363301	1002361	1091474	0.103503	0.0761	0.082866	0.043005275	2.406762	1.769561	1.926881
	1000	13643035	1278362	1163964	1278362	0.093701	0.085316	0.093701	0.043005275	2.178819	1.983841	2.178819

Figure 4G-K

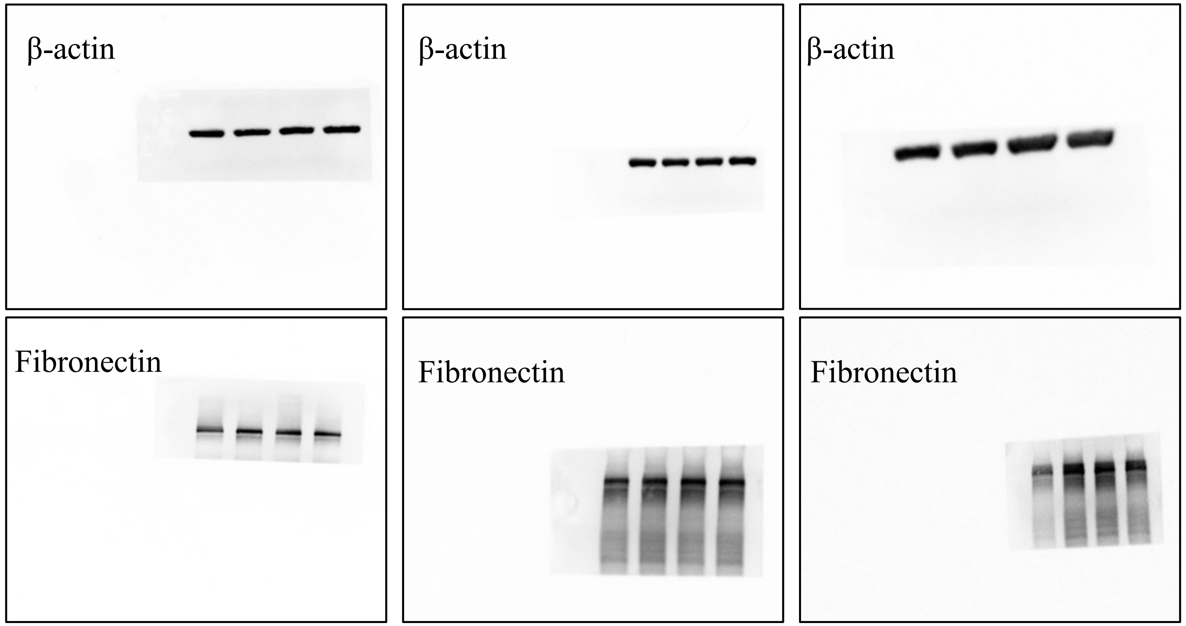

1		actin	Fibronectin			Fibronectin/actin			Control mean	tive expression		
Ferritin	0	8352367	5955412	5932433	5932433	0.713021	0.71027	0.71027	0.711186741	1.002579	0.998711	0.998711
	250	8590870	10994956	8909984	9961851	1.279842	1.037146	1.159586	0.711186741	1.799586	1.458331	1.630494
	500	8607860	10657481	8962257	9582187	1.23811	1.041171	1.11319	0.711186741	1.740908	1.463991	1.565258
	1000	9068180	10125090	9227615	9227615	1.116552	1.017582	1.017582	0.711186741	1.569984	1.430822	1.430822
2		actin	Fibronectin			Fibronectin/actin			Control mean	tive expression		
Ferritin	0	10354744	3970089	3681675	3256848	0.383408	0.355554	0.314527	0.3511631	1.091822	1.012505	0.895673
	250	9466815	6548624	6863414	6501646	0.691745	0.724997	0.686783	0.3511631	1.969869	2.06456	1.955737
	500	9914578	5038792	5167596	4391945	0.508221	0.521212	0.442979	0.3511631	1.447249	1.484244	1.261461
	1000	11084779	5160936	4948230	5446883	0.465588	0.446399	0.491384	0.3511631	1.325844	1.2712	1.399304
3		actin	Fibronectin			Fibronectin/actin			Control mean	tive expression		
Ferritin	0	9071536	2254080	2257127	2064649	0.248478	0.248814	0.227596	0.241629642	1.028344	1.029734	0.941923
	250	8791719	3668572	3907008	3796943	0.417276	0.444396	0.431877	0.241629642	1.726923	1.839163	1.787352
	500	10373364	3473922	3720002	3588798	0.334889	0.358611	0.345963	0.241629642	1.385959	1.484135	1.43179
	1000	9761294	3251603	3674686	3464778	0.333112	0.376455	0.354951	0.241629642	1.378605	1.557983	1.468987
