# Supplementary material for: Macrophage–Derived Ferritin Exacerbates Silica‐Induced Pulmonary Fibrosis via PIK3R2‐Mediated Fibroblast Differentiation
Source: Adv Sci (Weinh). 2026 Jan 21;13(17):e19191. doi: 10.1002/advs.202519191 (PMC13042690; doi:10.1002/advs.202519191)
Supplement: Supplementary file 4 — Supporting File 4: advs73867‐sup‐0001‐FiguresData.zip. [file ADVS-13-e19191-s001.zip › Supporting information Figure1-10/Figure 5/Figure 5A-E.pdf]

Figure 5A-E

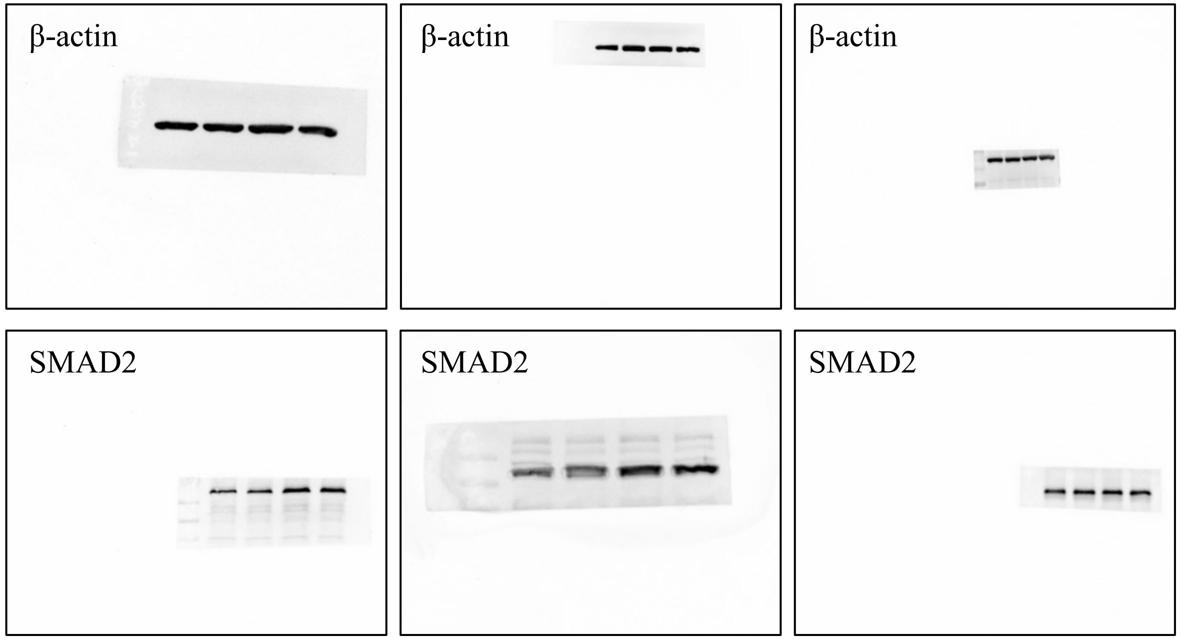

1		actin	SMAD2			SMAD2/actin			Control mean	relative expression		
Ferritin	0	5365462	5203092	5666627	5765651	0.969737928	1.056130302	1.074586121	1.033484783	0.938318535	1.021911806	1.039769659
	250	4904448	5361063	5788372	6227913	1.093102221	1.180229049	1.26984994	1.033484783	1.057685839	1.141989769	1.228706954
	500	5539463	9656109	10270195	10841406	1.74314893	1.854005524	1.957122198	1.033484783	1.686671113	1.793935966	1.893711672
	1000	4593788	8388910	8964458	9574820	1.826142173	1.951430497	2.084297316	1.033484783	1.766975385	1.888204382	2.016766332
2		actin	SMAD2			SMAD2/actin			Control mean	relative expression		
Ferritin	0	19679103	5640363	5206973	4974427	0.286616875	0.264594021	0.252777121	0.267996006	1.06948189	0.987305839	0.94321227
	250	23989469	7354553	6618163	5779711	0.306574231	0.275877845	0.240927008	0.267996006	1.143950746	1.029410285	0.898994773
	500	23552677	10192151	9901610	8792986	0.432738538	0.420402742	0.373332764	0.267996006	1.614720101	1.568690329	1.393053463
	1000	21293571	10274775	10102129	9930023	0.482529445	0.474421552	0.466339018	0.267996006	1.800509839	1.770256055	1.740096899
3		actin	SMAD2			SMAD2/actin			Control mean	relative expression		
Ferritin	0	8551777	3219531	3776820	2894743	0.376475088	0.441641544	0.338496081	0.385537571	0.976493905	1.145521416	0.87798468
	250	8630900	3975116	4117992	3988186	0.460567959	0.477121969	0.462082286	0.385537571	1.19461239	1.237549864	1.198540221
	500	7663874	4183042	4351781	4491837	0.545812992	0.567830447	0.586105278	0.385537571	1.415719331	1.472827786	1.520228693
	1000	7325492	3813230	4007066	4780603	0.520542511	0.547002986	0.652598215	0.385537571	1.350173239	1.418805915	1.692696804

Figure 5A-E

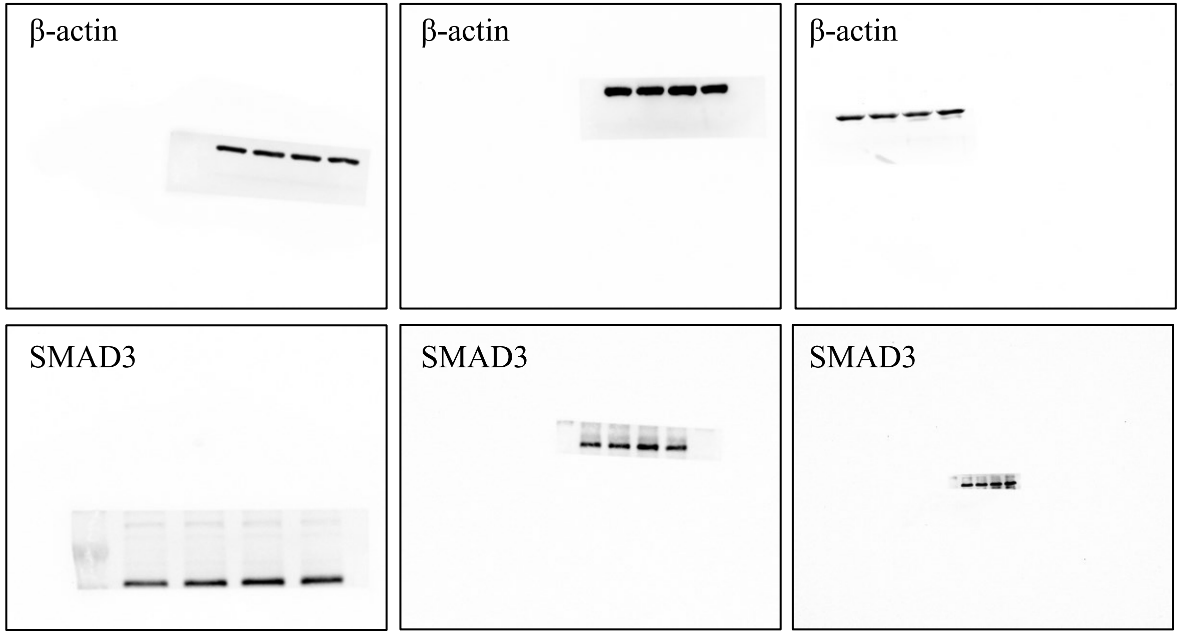

1		actin	SMAD3			SMAD3/actin			Control mean	relative expression		
Ferritin	0	10031527	5156984	4717895	5216537	0.5140777	0.4703068	0.5200143	0.5014662	1.0251491	0.9378633	1.0369876
	250	10078518	6238897	5791822	6198842	0.6190292	0.57467	0.6150549	0.5014662	1.2344385	1.1459795	1.2265131
	500	10397395	6938185	6837625	7930963	0.6673003	0.6576287	0.7627837	0.5014662	1.3306984	1.3114117	1.5211067
	1000	9849492	6431778	6366571	6921001	0.6530061	0.6463857	0.7026759	0.5014662	1.3021935	1.2889915	1.4012428
2		actin	SMAD3			SMAD3/actin			Control mean	relative expression		
Ferritin	0	16846493	2050053	1821074	1534503	0.1216902	0.1080981	0.0910874	0.1069586	1.1377321	1.0106541	0.8516138
	250	17191152	2296843	2296843	2180143	0.1336061	0.1336061	0.1268177	0.1069586	1.249139	1.249139	1.1856717
	500	19229896	3309460	2869181	3500796	0.1720997	0.1492042	0.1820497	0.1069586	1.6090319	1.3949719	1.7020578
	1000	16198912	2145665	2157190	2168057	0.1324574	0.1331688	0.1338397	0.1069586	1.2383988	1.2450506	1.2513226
3		actin	SMAD3			SMAD3/actin			Control mean	relative expression		
Ferritin	0	7966362	2909340	2729580	2681595	0.3652031	0.3426382	0.3366148	0.348152	1.0489759	0.9841626	0.9668614
	250	7219254	2971425	2881196	2757832	0.4115972	0.3990989	0.3820107	0.348152	1.1822342	1.146335	1.0972525
	500	6361356	3407042	3538355	3695228	0.5355842	0.5562265	0.5808868	0.348152	1.5383632	1.5976542	1.6684862
	1000	8816713	4205927	3978673	3977954	0.4770403	0.4512649	0.4511833	0.348152	1.3702068	1.296172	1.2959377

Figure 5A-E

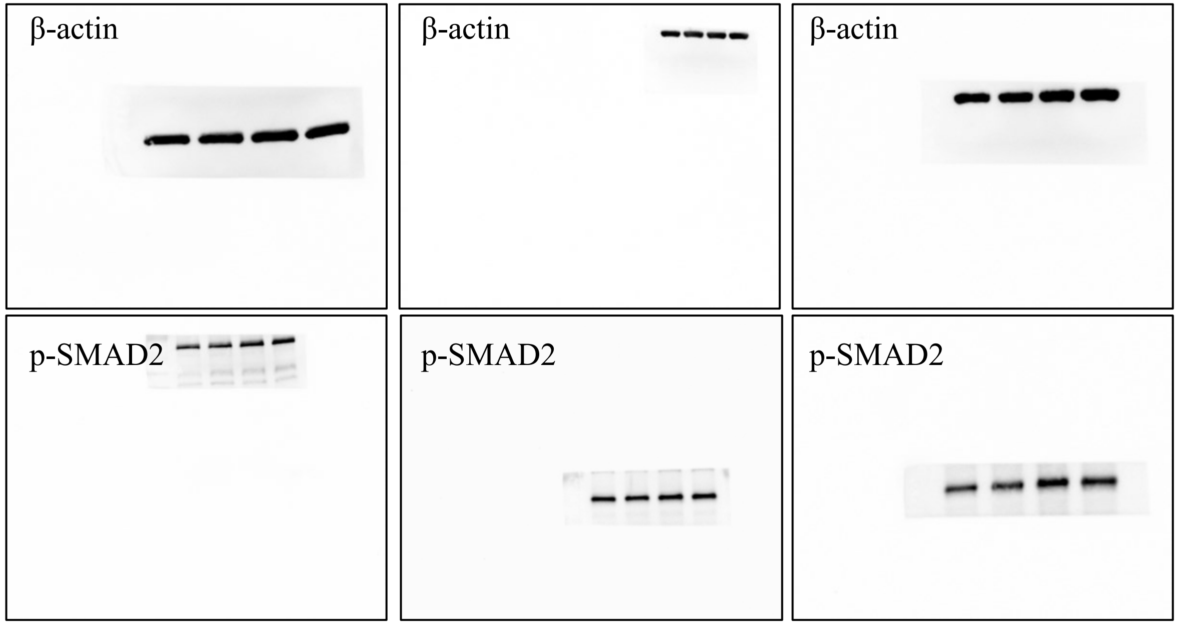

1		actin	p-SMAD2			p-SMAD2/actin			Control mean	relative expression		
Ferritin	0	22840322	7481680	7291042	6718816	0.327564559	0.319218004	0.29416468	0.3136491	1.0443664	1.017755	0.9378783
	250	25186347	8078763	7291713	7725240	0.320759616	0.289510543	0.30672332	0.3136491	1.0226704	0.92304	0.9779188
	500	27799804	11257227	10411426	10671739	0.404939078	0.374514367	0.38387821	0.3136491	1.2910578	1.194055	1.2239099
	1000	26301628	11823192	12076861	12317963	0.449523201	0.459167813	0.46833462	0.3136491	1.4332043	1.463954	1.4931803
2		actin	p-SMAD2			p-SMAD2/actin			Control mean	relative expression		
Ferritin	0	6962397	3496675	3347028	2894837	0.50222287	0.480729266	0.41578166	0.4662446	1.0771661	1.031067	0.8917672
	250	6251158	3468895	3082649	3229711	0.554920384	0.493132472	0.51665803	0.4662446	1.1901916	1.057669	1.1081266
	500	6809654	4544825	4576294	4576294	0.667409093	0.672030326	0.67203033	0.4662446	1.431457	1.441369	1.4413686
	1000	7643941	4629628	4481838	4587602	0.605659829	0.586325562	0.60016188	0.4662446	1.2990174	1.257549	1.2872254
3		actin	p-SMAD2			p-SMAD2/actin			Control mean	relative expression		
Ferritin	0	11898068	3517724	3638199	4170437	0.295655059	0.305780653	0.3505138	0.3173165	0.9317355	0.963646	1.1046189
	250	10730144	3765532	4164688	4714944	0.350930239	0.38812974	0.43941106	0.3173165	1.1059313	1.223163	1.3847722
	500	11689139	5170534	5383716	5789972	0.4423366	0.460574213	0.49532921	0.3173165	1.3939918	1.451466	1.5609942
	1000	15061053	6029000	6378720	6266019	0.400304016	0.423524172	0.41604123	0.3173165	1.2615291	1.334706	1.3111238

Figure 5A-E

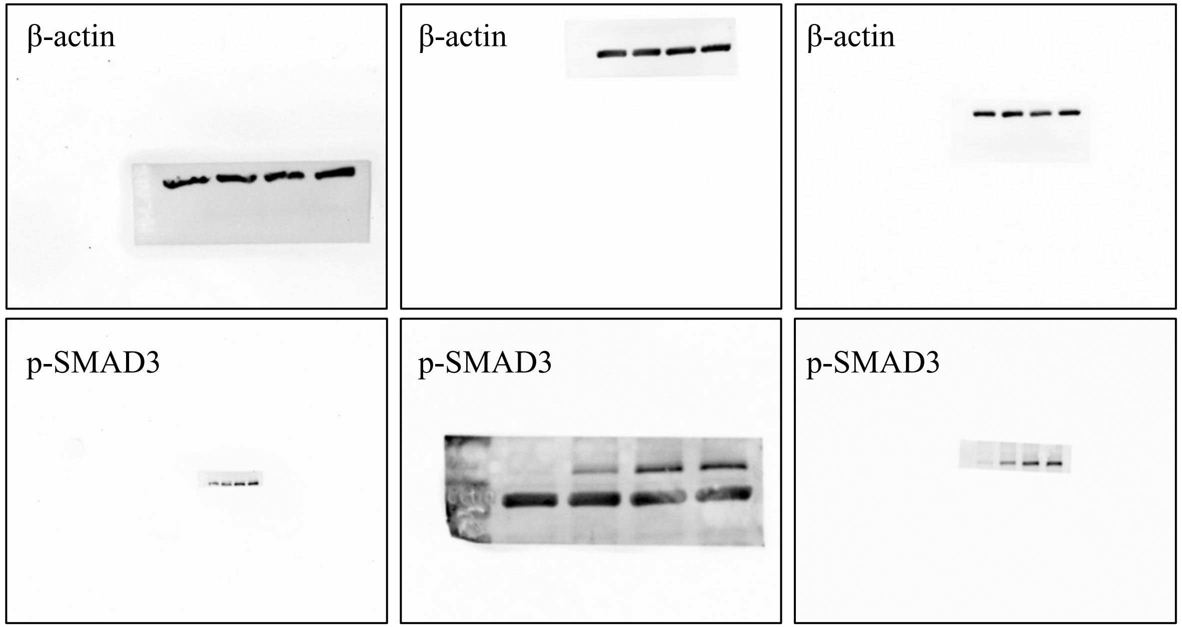

1		actin	p-SMAD3			p-SMAD3/actin			Control mean	relative expression		
Ferritin	0	7640886	1794250	1755232	1593531	0.234822244	0.229715769	0.20855317	0.22436373	1.046614115	1.023854305	0.92953158
	250	5634495	2398525	2220058	2013886	0.425685887	0.394011886	0.357420851	0.22436373	1.897302611	1.756130055	1.593042043
	500	5501737	3549328	3744271	3331753	0.64512862	0.680561612	0.605582019	0.22436373	2.875369502	3.033296061	2.699108385
	1000	5355923	4154620	4438172	3738397	0.7757057	0.828647462	0.697993044	0.22436373	3.45735787	3.693321866	3.1109888
2		actin	p-SMAD3			p-SMAD3/actin			Control mean	relative expression		
Ferritin	0	16538780	2628269	1828213	2383925	0.158915531	0.110540983	0.144141527	0.13786601	1.152680974	0.801800098	1.045518929
	250	16693201	3957044	3919780	4320474	0.23704525	0.234812964	0.258816389	0.13786601	1.719388584	1.703196877	1.877303784
	500	17783544	6171327	5718811	5362280	0.347024586	0.321578815	0.301530449	0.13786601	2.517114824	2.332545974	2.187126769
	1000	18806210	7883057	7855345	7650917	0.419173082	0.417699526	0.406829287	0.13786601	3.040438111	3.029749793	2.950903391
3		actin	p-SMAD3			p-SMAD3/actin			Control mean	relative expression		
Ferritin	0	3279167	1140096	1114424	1130676	0.347678542	0.339849724	0.344805861	0.34411138	1.010366313	0.987615488	1.002018199
	250	3655879	2120241	2357840	2269206	0.579953822	0.644944759	0.620700521	0.34411138	1.68536661	1.874232603	1.803777978
	500	2782118	3647657	3988590	3853799	1.31110794	1.433652347	1.385203288	0.34411138	3.810126702	4.166245144	4.02545044
	1000	3720887	4603992	4746137	4180682	1.237337226	1.275539139	1.123571342	0.34411138	3.595746362	3.70676249	3.265138621
