# Supplementary material for: Macrophage–Derived Ferritin Exacerbates Silica‐Induced Pulmonary Fibrosis via PIK3R2‐Mediated Fibroblast Differentiation
Source: Adv Sci (Weinh). 2026 Jan 21;13(17):e19191. doi: 10.1002/advs.202519191 (PMC13042690; doi:10.1002/advs.202519191)
Supplement: Supplementary file 4 — Supporting File 4: advs73867‐sup‐0001‐FiguresData.zip. [file ADVS-13-e19191-s001.zip › Supporting information Figure1-10/Figure 5/Figure 5I, J.pdf]

Figure 5I, J

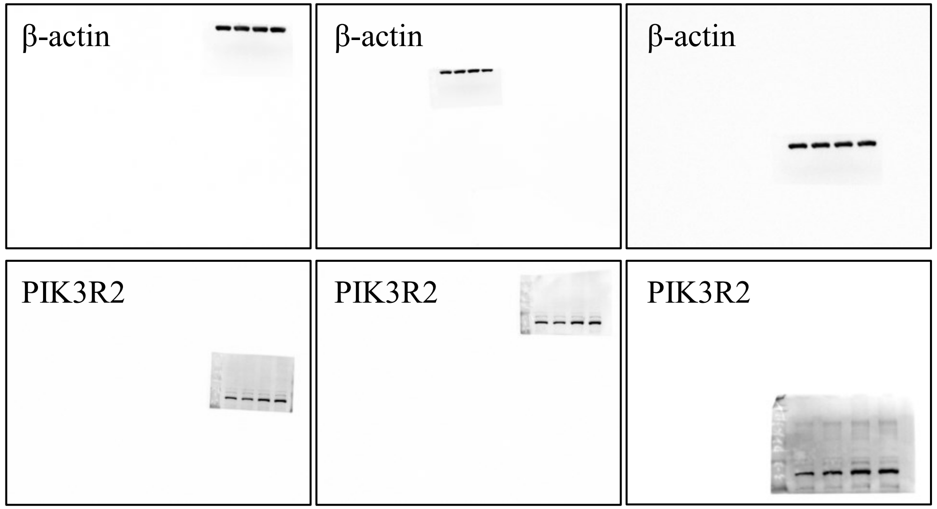

1		actin	PIK3R2			PIK3R2/actin			Control mean	relative expression		
Ferritin	0	6962397	1378268	1384247	1262615	0.197959	0.198818	0.181348	0.192708057	1.027247	1.031704	0.941049
	250	6251158	1192045	1191126	1177610	0.190692	0.190545	0.188383	0.192708057	0.989538	0.988775	0.977555
	500	6809654	2012853	2033289	1819418	0.295588	0.298589	0.267182	0.192708057	1.533865	1.549438	1.386461
	1000	7643941	2322405	2284892	2178683	0.303823	0.298915	0.285021	0.192708057	1.576597	1.551131	1.47903
2		actin	PIK3R2			PIK3R2/actin			Control mean	relative expression		
Ferritin	0	6930942	1546261	1120281	1399949	0.223095	0.161635	0.201985	0.195571828	1.140734	0.826473	1.032794
	250	7803177	1280120	1166773	1130979	0.164051	0.149525	0.144938	0.195571828	0.838828	0.764555	0.7411
	500	8600690	2357774	2348160	2304334	0.274138	0.27302	0.267924	0.195571828	1.401724	1.396009	1.369954
	1000	6902598	2437895	2270268	2411607	0.353185	0.328901	0.349377	0.195571828	1.80591	1.681738	1.786437
3		actin	PIK3R2			PIK3R2/actin			Control mean	relative expression		
Ferritin	0	1972282	2141996	2183396	2398813	1.08605	1.10704	1.216263	1.136450906	0.95565	0.974121	1.070229
	250	1786416	2701903	2979493	2844435	1.512471	1.667861	1.592258	1.136450906	1.330873	1.467605	1.401079
	500	1806921	4094827	3547752	4340801	2.26619	1.963424	2.402319	1.136450906	1.994094	1.72768	2.113879
	1000	2024540	4400175	3696481	4602796	2.17342	1.825837	2.273502	1.136450906	1.912462	1.606614	2.000528
