# Supplementary material for: Macrophage–Derived Ferritin Exacerbates Silica‐Induced Pulmonary Fibrosis via PIK3R2‐Mediated Fibroblast Differentiation
Source: Adv Sci (Weinh). 2026 Jan 21;13(17):e19191. doi: 10.1002/advs.202519191 (PMC13042690; doi:10.1002/advs.202519191)
Supplement: Supplementary file 4 — Supporting File 4: advs73867‐sup‐0001‐FiguresData.zip. [file ADVS-13-e19191-s001.zip › Supporting information Figure1-10/Figure 6/Figure S6E-O.pdf]

# Figure 6E-O

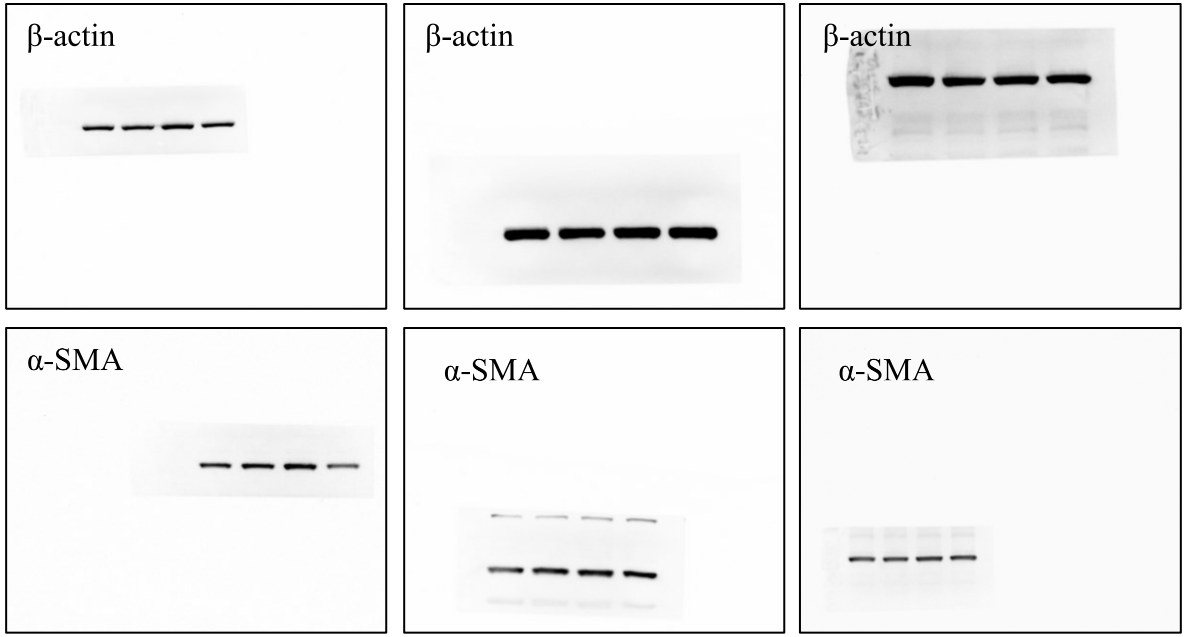


| | | actin | α-SMA | | | α-SMA/actin | | | Control mean | relative expression | | |
|---|---|---|---|---|---|---|---|---|---|---|---|---|
| 1 | NC | 11345268 | 2554358 | 2915809 | 2731261 | 0.225147 | 0.257007 | 0.24074 | 0.240964721 | 0.934359 | 1.066574 | 0.999068 |
| | siPIK3R2 | 11410239 | 3517177 | 3627123 | 3562992 | 0.308247 | 0.317883 | 0.312263 | 0.240964721 | 1.279222 | 1.31921 | 1.295886 |
| | NC+Ferritir | 13922946 | 4594693 | 4671940 | 4655720 | 0.330009 | 0.335557 | 0.334392 | 0.240964721 | 1.369531 | 1.392556 | 1.387721 |
| | K3R2+Fer | 11700644 | 2110071 | 2316416 | 2311834 | 0.180338 | 0.197973 | 0.197582 | 0.240964721 | 0.7484 | 0.821587 | 0.819961 |
| | | | | | | | | | | | | |
| | | actin | α-SMA | | | α-SMA/actin | | | Control mean | relative expression | | |
| 2 | NC | 20263997 | 8560676 | 8157454 | 7625313 | 0.422457 | 0.402559 | 0.376299 | 0.400438324 | 1.054987 | 1.005296 | 0.939717 |
| | siPIK3R2 | 19840135 | 12433090 | 11878864 | 12005185 | 0.626664 | 0.598729 | 0.605096 | 0.400438324 | 1.564944 | 1.495184 | 1.511084 |
| | NC+Ferritir | 21818009 | 13177071 | 13692721 | 13786079 | 0.603954 | 0.627588 | 0.631867 | 0.400438324 | 1.508232 | 1.567253 | 1.577938 |
| | K3R2+Fer | 23763683 | 12336288 | 11986183 | 10990247 | 0.519124 | 0.504391 | 0.462481 | 0.400438324 | 1.296388 | 1.259597 | 1.154936 |
| | | | | | | | | | | | | |
| | | actin | α-SMA | | | α-SMA/actin | | | Control mean | relative expression | | |
| 3 | NC | 19142106 | 3631902 | 3514595 | 3259933 | 0.189734 | 0.183605 | 0.170302 | 0.181213603 | 1.047017 | 1.013199 | 0.939784 |
| | siPIK3R2 | 14007545 | 3488672 | 3338860 | 3500422 | 0.249057 | 0.238362 | 0.249895 | 0.181213603 | 1.374382 | 1.315362 | 1.379011 |
| | NC+Ferritir | 14918335 | 4018812 | 4171600 | 4171600 | 0.269387 | 0.279629 | 0.279629 | 0.181213603 | 1.486574 | 1.543091 | 1.543091 |
| | K3R2+Fer | 15710131 | 3647651 | 3503274 | 3766818 | 0.232185 | 0.222995 | 0.23977 | 0.181213603 | 1.281276 | 1.230562 | 1.323135 |

# Figure 6E-O

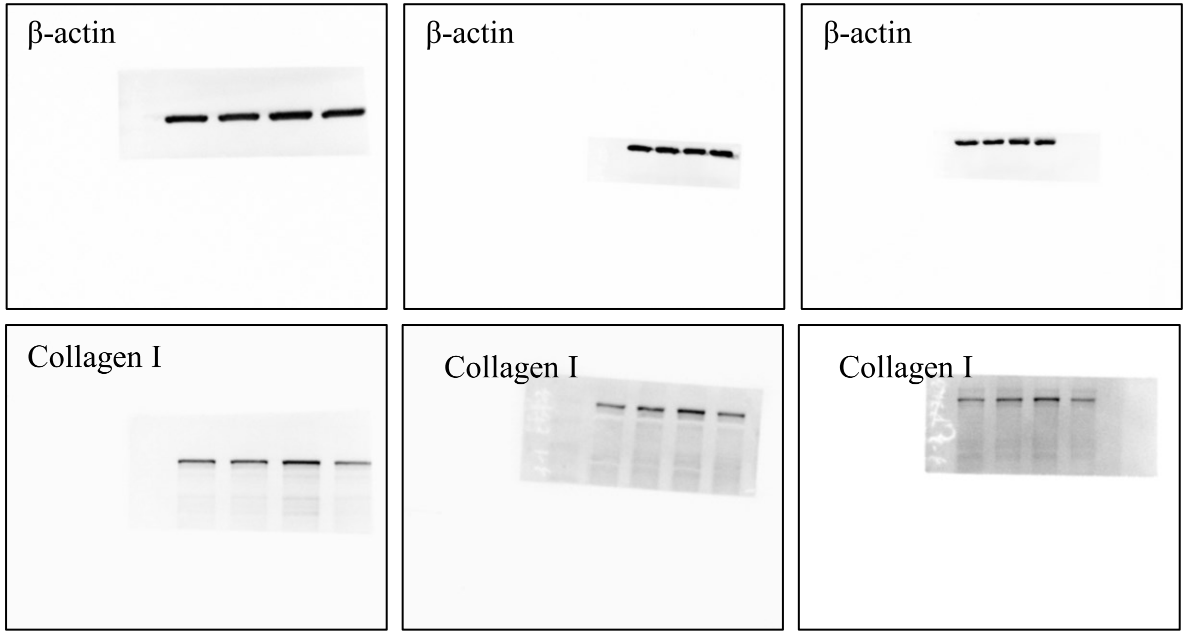


| | | actin | Collagen I | | | Collagen I/actin | | | Control mean | relative expression | | |
|---|---|---|---|---|---|---|---|---|---|---|---|---|
| 1 | NC | 16283620 | 2937527 | 3643846 | 2858119 | 0.180398 | 0.223774 | 0.175521 | 0.193230825 | 0.933586 | 1.158064 | 0.908349 |
| | siPIK3R2 | 15057859 | 2674303 | 3360481 | 3130622 | 0.177602 | 0.223171 | 0.207906 | 0.193230825 | 0.919117 | 1.154946 | 1.075947 |
| | NC+Ferritir | 17148031 | 8365397 | 7594386 | 6756144 | 0.487834 | 0.442872 | 0.393989 | 0.193230825 | 2.524619 | 2.291933 | 2.038958 |
| | siPIK3R2+ | 18154376 | 4624368 | 6121147 | 5375824 | 0.254725 | 0.337172 | 0.296117 | 0.193230825 | 1.318241 | 1.744918 | 1.532454 |
| | | | | | | | | | | | | |
| | | actin | Collagen I | | | Collagen I/actin | | | Control mean | relative expression | | |
| 2 | NC | 11361498 | 773583 | 750646 | 830745 | 0.068088 | 0.066069 | 0.073119 | 0.069092239 | 0.985467 | 0.956247 | 1.058286 |
| | siPIK3R2 | 10967409 | 1175449 | 1044393 | 1068435 | 0.107177 | 0.095227 | 0.097419 | 0.069092239 | 1.55121 | 1.378258 | 1.409986 |
| | NC+Ferritir | 10038674 | 1559516 | 1592912 | 1701671 | 0.155351 | 0.158678 | 0.169512 | 0.069092239 | 2.248455 | 2.296604 | 2.453409 |
| | siPIK3R2+ | 12665825 | 945092 | 881377 | 1014457 | 0.074617 | 0.069587 | 0.080094 | 0.069092239 | 1.079969 | 1.007161 | 1.159233 |
| | | | | | | | | | | | | |
| | | actin | Collagen I | | | Collagen I/actin | | | Control mean | relative expression | | |
| 3 | NC | 10782413 | 400906 | 295234 | 373683 | 0.037181 | 0.027381 | 0.034657 | 0.033073085 | 1.124221 | 0.827896 | 1.047883 |
| | siPIK3R2 | 9874330 | 495811 | 466637 | 495811 | 0.050212 | 0.047258 | 0.050212 | 0.033073085 | 1.518217 | 1.428883 | 1.518217 |
| | NC+Ferriti | 10308456 | 659644 | 743386 | 707781 | 0.063991 | 0.072114 | 0.06866 | 0.033073085 | 1.934823 | 2.18045 | 2.076015 |
| | siPIK3R2+ | 10843655 | 313771 | 383075 | 348688 | 0.028936 | 0.035327 | 0.032156 | 0.033073085 | 0.874908 | 1.068153 | 0.972269 |

# Figure 6E-O

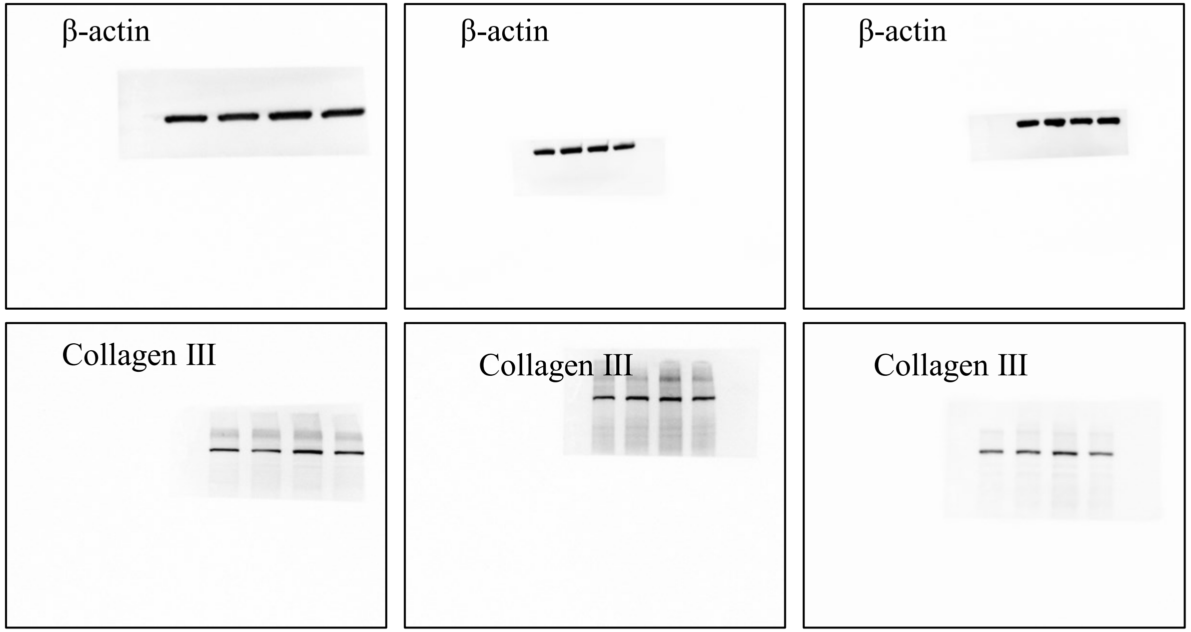


| | | actin | Collagen III | | | Collagen III/actin | | | Control mean | relative expression | | |
|---|---|---|---|---|---|---|---|---|---|---|---|---|
| 1 | NC | 8902861 | 2295767 | 1553289 | 2108107 | 0.257868 | 0.174471 | 0.23679 | 0.22304302 | 1.156138 | 0.782229 | 1.061633 |
| | siPIK3R2 | 8113918 | 1806735 | 1601315 | 1609181 | 0.222671 | 0.197354 | 0.198324 | 0.22304302 | 0.998332 | 0.884825 | 0.889172 |
| | NC+Ferritir | 10449587 | 4003658 | 3752637 | 3646655 | 0.38314 | 0.359118 | 0.348976 | 0.22304302 | 1.717787 | 1.610085 | 1.564613 |
| | K3R2+Fer | 9350597 | 2379894 | 2240491 | 2157092 | 0.254518 | 0.239609 | 0.23069 | 0.22304302 | 1.141116 | 1.074274 | 1.034286 |
| | | | | | | | | | | | | |
| | | actin | Collagen III | | | Collagen III/actin | | | Control mean | relative expression | | |
| 2 | NC | 10768215 | 1227585 | 1211346 | 1190919 | 0.114001 | 0.112493 | 0.110596 | 0.112363098 | 1.014575 | 1.001154 | 0.984271 |
| | siPIK3R2 | 12644711 | 2026701 | 1987514 | 1432563 | 0.160281 | 0.157181 | 0.113293 | 0.112363098 | 1.426452 | 1.398871 | 1.00828 |
| | NC+Ferritir | 11757252 | 2331517 | 2372672 | 2292023 | 0.198305 | 0.201805 | 0.194945 | 0.112363098 | 1.764855 | 1.796008 | 1.73496 |
| | K3R2+Fer | 10379345 | 1587536 | 1550626 | 1193183 | 0.152951 | 0.149395 | 0.114957 | 0.112363098 | 1.361225 | 1.329577 | 1.023089 |
| | | | | | | | | | | | | |
| | | actin | Collagen III | | | Collagen III/actin | | | Control mean | relative expression | | |
| 3 | NC | 11866349 | 1843371 | 1663818 | 1857124 | 0.155344 | 0.140213 | 0.156503 | 0.150686983 | 1.030908 | 0.930493 | 1.038599 |
| | siPIK3R2 | 12428083 | 2039029 | 2022612 | 2167740 | 0.164066 | 0.162745 | 0.174423 | 0.150686983 | 1.088788 | 1.080022 | 1.157517 |
| | NC+Ferritir | 11126568 | 2866444 | 2990321 | 3071448 | 0.257622 | 0.268755 | 0.276046 | 0.150686983 | 1.709647 | 1.783532 | 1.831919 |
| | K3R2+Fer | 12752926 | 1651342 | 1455314 | 1616222 | 0.129487 | 0.114116 | 0.126733 | 0.150686983 | 0.859313 | 0.757306 | 0.841038 |

# Figure 6E-O

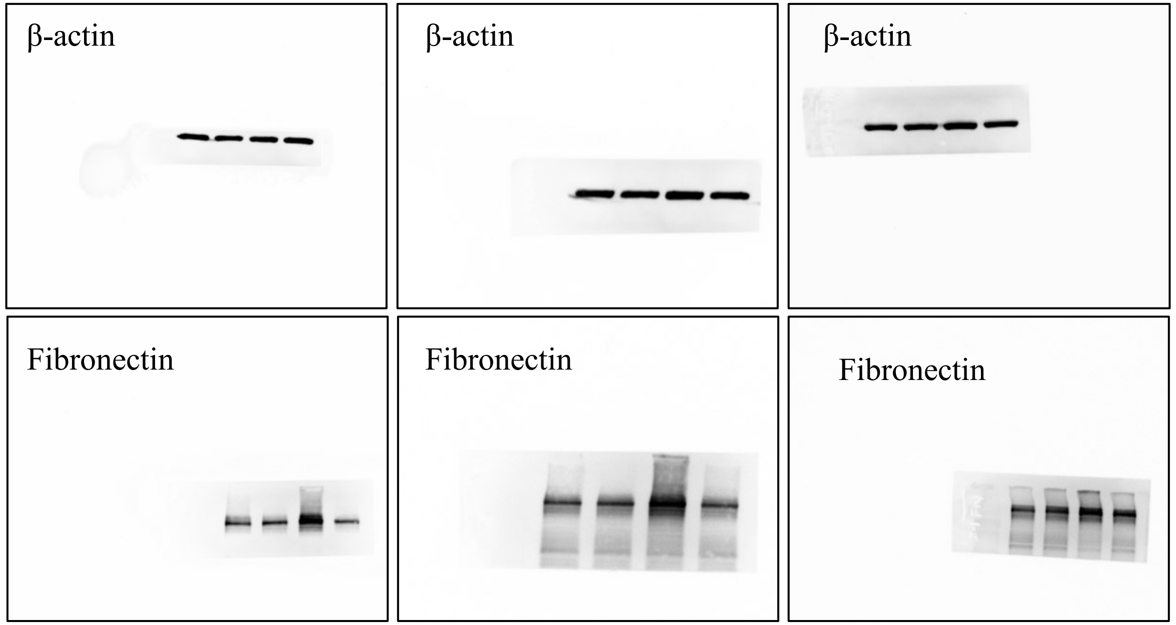


| | | actin | Fibronectin | | | Fibronectin/actin | | | Control mean | relative expression | | |
|---|---|---|---|---|---|---|---|---|---|---|---|---|
| | NC | 20866661 | 7935446 | 8982319 | 8317853 | 0.380293 | 0.430463 | 0.398619 | 0.403124998 | 0.943363 | 1.067814 | 0.988823 |
| 1 | siPIK3R2 | 16401457 | 5894577 | 8270448 | 7201532 | 0.359393 | 0.504251 | 0.439079 | 0.403124998 | 0.891519 | 1.250855 | 1.089188 |
| | NC+Ferritin | 17529336 | 15806621 | 14808288 | 14984975 | 0.901724 | 0.844772 | 0.854851 | 0.403124998 | 2.236834 | 2.095558 | 2.120561 |
| | siPIK3R2+Ferritin | 20602906 | 4646985 | 5791399 | 5418629 | 0.22555 | 0.281096 | 0.263003 | 0.403124998 | 0.559504 | 0.697293 | 0.652411 |
| | | | | | | | | | | | | |
| | | | | | | | | | | | | |
| | | actin | Fibronectin | | | Fibronectin/actin | | | Control mean | relative expression | | |
| 2 | NC | 19123035 | 11132524 | 9940267 | 9250222 | 0.582153 | 0.519806 | 0.483721 | 0.52855998 | 1.101394 | 0.983438 | 0.915168 |
| | siPIK3R2 | 17107856 | 11895807 | 10534367 | 11373964 | 0.695342 | 0.615762 | 0.664839 | 0.52855998 | 1.31554 | 1.16498 | 1.25783 |
| | NC+Ferritin | 19957294 | 19080149 | 20267419 | 23866921 | 0.956049 | 1.015539 | 1.1959 | 0.52855998 | 1.80878 | 1.921332 | 2.262562 |
| | siPIK3R2+Ferritin | 18306453 | 10524834 | 9425425 | 10224008 | 0.574925 | 0.514869 | 0.558492 | 0.52855998 | 1.087719 | 0.974098 | 1.056629 |
| | | | | | | | | | | | | |
| | | actin | Fibronectin | | | Fibronectin/actin | | | Control mean | relative expression | | |
| 3 | NC | 11345268 | 3225597 | 3779313 | 3340499 | 0.284312 | 0.333118 | 0.29444 | 0.303956651 | 0.935371 | 1.095939 | 0.96869 |
| | siPIK3R2 | 11410239 | 4659110 | 4026277 | 4307195 | 0.408327 | 0.352865 | 0.377485 | 0.303956651 | 1.343373 | 1.160907 | 1.241904 |
| | NC+Ferritin | 13922946 | 10116882 | 10848901 | 9801295 | 0.726634 | 0.77921 | 0.703967 | 0.303956651 | 2.390583 | 2.563557 | 2.316011 |
| | siPIK3R2+Ferritin | 11700644 | 5774281 | 5109412 | 4462766 | 0.493501 | 0.436678 | 0.381412 | 0.303956651 | 1.62359 | 1.436645 | 1.254824 |

# Figure 6E-O

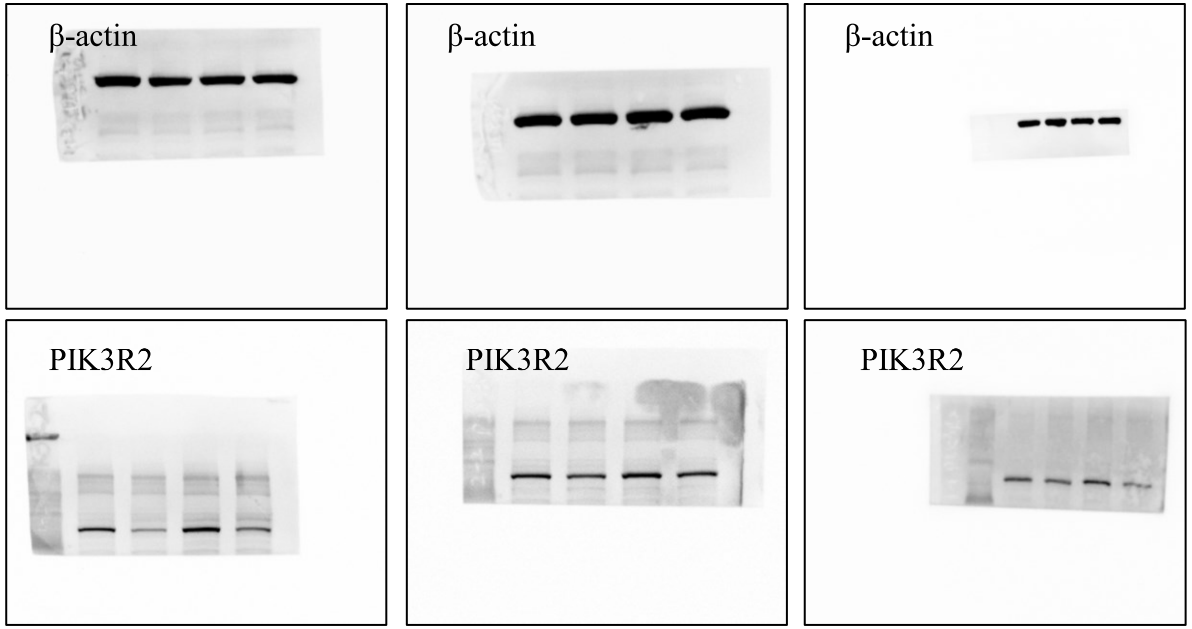


| | | actin | PIK3R2 | | | PIK3R2/actin | | | Control mean | relative expression | | |
|---|---|---|---|---|---|---|---|---|---|---|---|---|
| 1 | NC | 18801990 | 3848522 | 3980633 | 3848522 | 0.204687 | 0.211713 | 0.204687 | 0.207029096 | 0.988687 | 1.022626 | 0.988687 |
| | siPIK3R2 | 13523201 | 1467921 | 1689948 | 1590124 | 0.108548 | 0.124967 | 0.117585 | 0.207029096 | 0.524314 | 0.603618 | 0.567963 |
| | NC+Ferritin | 13844288 | 5957068 | 6294800 | 6027513 | 0.430291 | 0.454686 | 0.435379 | 0.207029096 | 2.078407 | 2.196241 | 2.102985 |
| | siPIK3R2+Ferritin | 15217838 | 2638170 | 2741858 | 2464312 | 0.17336 | 0.180174 | 0.161936 | 0.207029096 | 0.837372 | 0.870283 | 0.782188 |
| | | | | | | | | | | | | |
| | | | | | | | | | | | | |
| 2 | | actin | PIK3R2 | | | PIK3R2/actin | | | Control mean | relative expression | | |
| | NC | 19110132 | 1742885 | 1677781 | 1695226 | 0.091202 | 0.087795 | 0.088708 | 0.089235246 | 1.022042 | 0.983864 | 0.994094 |
| | siPIK3R2 | 18942427 | 1343734 | 1275681 | 1283355 | 0.070938 | 0.067345 | 0.06775 | 0.089235246 | 0.794953 | 0.754693 | 0.759232 |
| | NC+Ferritin | 19504281 | 2212695 | 2521680 | 2523466 | 0.113447 | 0.129289 | 0.12938 | 0.089235246 | 1.271321 | 1.448851 | 1.449877 |
| | siPIK3R2+Ferritin | 19785634 | 1567919 | 1824922 | 1682452 | 0.079245 | 0.092235 | 0.085034 | 0.089235246 | 0.88805 | 1.033613 | 0.95292 |
| | | | | | | | | | | | | |
| 3 | | actin | PIK3R2 | | | PIK3R2/actin | | | Control mean | relative expression | | |
| | NC | 11866349 | 2168711 | 2168711 | 1716102 | 0.182761 | 0.182761 | 0.144619 | 0.170047361 | 1.074768 | 1.074768 | 0.850464 |
| | siPIK3R2 | 12428083 | 1648802 | 1642673 | 1714637 | 0.132667 | 0.132174 | 0.137965 | 0.170047361 | 0.780179 | 0.777279 | 0.811331 |
| | NC+Ferritin | 11126568 | 2742432 | 2873771 | 3012940 | 0.246476 | 0.25828 | 0.270788 | 0.170047361 | 1.449455 | 1.518872 | 1.592426 |
| | siPIK3R2+Ferritin | 12752926 | 1121808 | 1266137 | 1439318 | 0.087965 | 0.099282 | 0.112862 | 0.170047361 | 0.517296 | 0.58385 | 0.663708 |

# Figure 6E-O

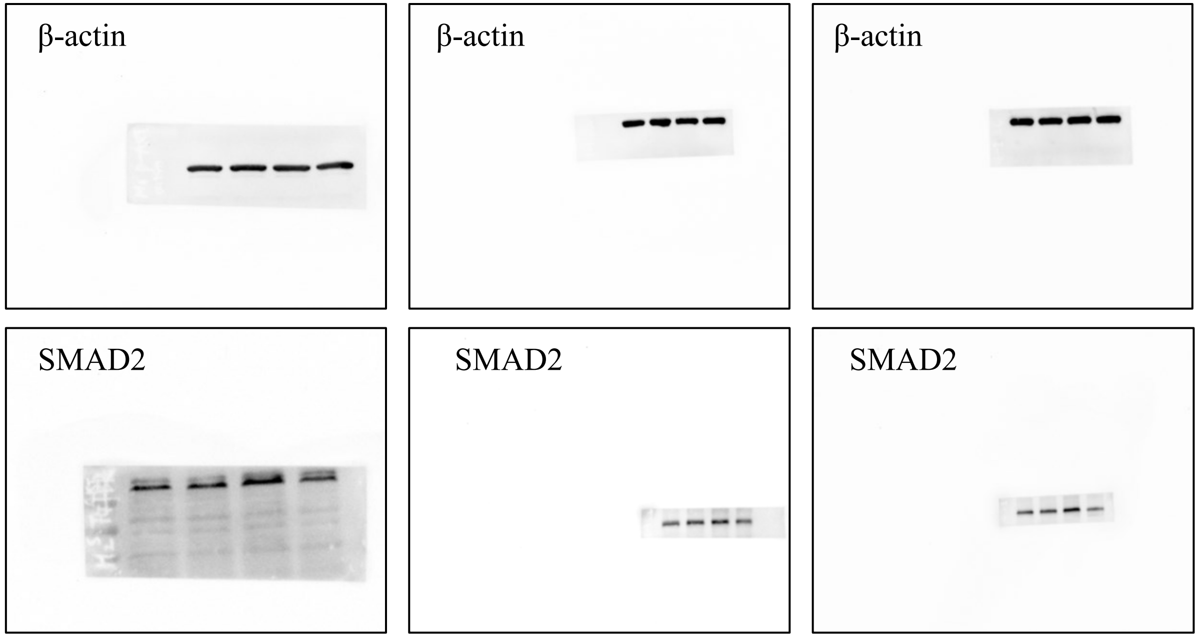


| | | actin | SMAD2 | | | SMAD2/actin | | | Control mean | relative expression | | |
|---|---|---|---|---|---|---|---|---|---|---|---|---|
| 1 | NC | 7595240 | 2519280 | 2227948 | 2287849 | 0.331692 | 0.293335 | 0.301221 | 0.308749383 | 1.074308 | 0.950074 | 0.975618 |
| | siPIK3R2 | 8635564 | 2687781 | 2295024 | 2535894 | 0.311246 | 0.265764 | 0.293657 | 0.308749383 | 1.008085 | 0.860777 | 0.951118 |
| | NC+Ferritin | 8174935 | 4860899 | 5316027 | 4263112 | 0.59461 | 0.650284 | 0.521486 | 0.308749383 | 1.925867 | 2.106186 | 1.689026 |
| | K3R2+Fer | 9186933 | 3296216 | 1806388 | 2524579 | 0.358794 | 0.196626 | 0.274801 | 0.308749383 | 1.162088 | 0.636846 | 0.890046 |
| | | | | | | | | | | | | |
| | | actin | SMAD2 | | | SMAD2/actin | | | Control mean | relative expression | | |
| 2 | NC | 11866349 | 2317397 | 1957211 | 2317397 | 0.195291 | 0.164938 | 0.195291 | 0.185173637 | 1.05464 | 0.89072 | 1.05464 |
| | siPIK3R2 | 12428083 | 2704413 | 2552000 | 2210810 | 0.217605 | 0.205341 | 0.177888 | 0.185173637 | 1.17514 | 1.108913 | 0.960656 |
| | NC+Ferritin | 11126568 | 3237955 | 3398999 | 3333326 | 0.291011 | 0.305485 | 0.299583 | 0.185173637 | 1.571558 | 1.649722 | 1.617847 |
| | K3R2+Fer | 12752926 | 1816397 | 1869326 | 2251552 | 0.14243 | 0.14658 | 0.176552 | 0.185173637 | 0.769169 | 0.791582 | 0.953439 |
| | | | | | | | | | | | | |
| | | actin | SMAD2 | | | SMAD2/actin | | | Control mean | relative expression | | |
| 3 | NC | 11000588 | 3003616 | 3003616 | 2735113 | 0.273041 | 0.273041 | 0.248633 | 0.264905385 | 1.030713 | 1.030713 | 0.938574 |
| | siPIK3R2 | 9409614 | 3464280 | 3464280 | 3730585 | 0.368164 | 0.368164 | 0.396465 | 0.264905385 | 1.389794 | 1.389794 | 1.49663 |
| | NC+Ferritin | 11069393 | 4930335 | 5361448 | 5320539 | 0.445402 | 0.484349 | 0.480653 | 0.264905385 | 1.681364 | 1.828384 | 1.814433 |
| | K3R2+Fer | 12366198 | 2280132 | 2692557 | 2791219 | 0.184384 | 0.217735 | 0.225714 | 0.264905385 | 0.696038 | 0.821936 | 0.852054 |

# Figure 6E-O

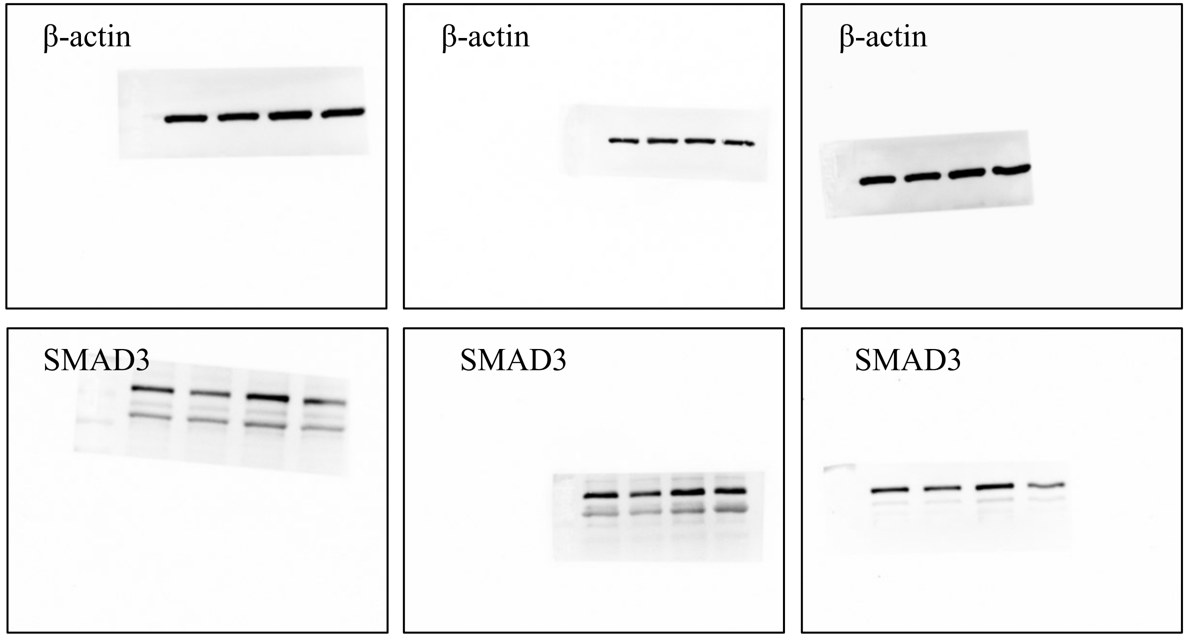


| | | actin | SMAD3 | | | SMAD3/actin | | | Control mean | relative expression | | |
|---|---|---|---|---|---|---|---|---|---|---|---|---|
| 1 | NC | 8902861 | 5437409 | 5170037 | 5431722 | 0.610748 | 0.580716 | 0.61011 | 0.600524857 | 1.017025 | 0.967015 | 1.015961 |
| | siPIK3R2 | 8113918 | 4059850 | 3642886 | 3907731 | 0.500356 | 0.448968 | 0.481608 | 0.600524857 | 0.833198 | 0.747625 | 0.801979 |
| | NC+Ferritin | 10449587 | 8484801 | 7978039 | 8337861 | 0.811975 | 0.763479 | 0.797913 | 0.600524857 | 1.352109 | 1.271353 | 1.328693 |
| | K3R2+Fer | 9350597 | 4658812 | 3957239 | 4403484 | 0.498237 | 0.423207 | 0.470931 | 0.600524857 | 0.829669 | 0.704729 | 0.784199 |
| | | | | | | | | | | | | |
| | | actin | SMAD3 | | | SMAD3/actin | | | Control mean | relative expression | | |
| 2 | NC | 5938023 | 7252895 | 6617506 | 5871691 | 1.221433 | 1.114429 | 0.988829 | 1.108230354 | 1.102147 | 1.005593 | 0.89226 |
| | siPIK3R2 | 6311904 | 4722290 | 4497195 | 3947073 | 0.748156 | 0.712494 | 0.625338 | 1.108230354 | 0.675091 | 0.642912 | 0.564267 |
| | NC+Ferritin | 5691163 | 9309173 | 9075028 | 8939581 | 1.635724 | 1.594582 | 1.570783 | 1.108230354 | 1.475979 | 1.438855 | 1.417379 |
| | K3R2+Fer | 6103277 | 7123642 | 6660432 | 6245574 | 1.167183 | 1.091288 | 1.023315 | 1.108230354 | 1.053195 | 0.984712 | 0.923377 |
| | | | | | | | | | | | | |
| | | actin | SMAD3 | | | SMAD3/actin | | | Control mean | relative expression | | |
| 3 | NC | 16283620 | 6260339 | 6333776 | 6019199 | 0.384456 | 0.388966 | 0.369647 | 0.381023261 | 1.00901 | 1.020846 | 0.970144 |
| | siPIK3R2 | 15057859 | 5108951 | 5039112 | 4685723 | 0.339288 | 0.33465 | 0.311181 | 0.381023261 | 0.890465 | 0.878293 | 0.816699 |
| | NC+Ferritin | 17148031 | 8692897 | 8532504 | 8330344 | 0.506933 | 0.497579 | 0.48579 | 0.381023261 | 1.330451 | 1.305902 | 1.274962 |
| | K3R2+Fer | 18154376 | 3504388 | 3351696 | 3108188 | 0.193033 | 0.184622 | 0.171209 | 0.381023261 | 0.506617 | 0.484542 | 0.449339 |

# Figure 6E-O

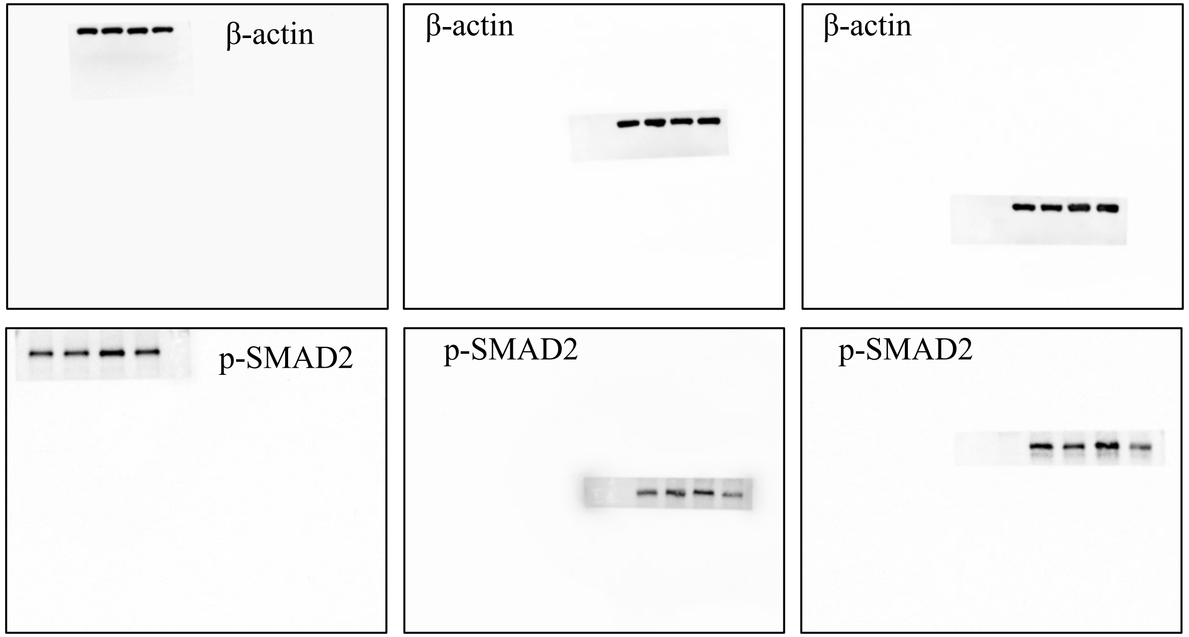


| | | actin | p-SMAD2 | | | p-SMAD2/actin | | | Control mean | relative expression | | |
|---|---|---|---|---|---|---|---|---|---|---|---|---|
| 1 | NC | 5055292 | 1283152 | 1244659 | 1078423 | 0.253824 | 0.246209 | 0.213326 | 0.237786067 | 1.067445 | 1.035423 | 0.897132 |
| | siPIK3R2 | 4939530 | 1280669 | 1373348 | 1211394 | 0.259269 | 0.278032 | 0.245245 | 0.237786067 | 1.090347 | 1.169253 | 1.031367 |
| | NC+Ferritir | 5103507 | 2430073 | 2477734 | 2419068 | 0.476157 | 0.485496 | 0.474001 | 0.237786067 | 2.002462 | 2.041736 | 1.993393 |
| | K3R2+Fer | 3890543 | 1462223 | 1403004 | 1409066 | 0.37584 | 0.360619 | 0.362177 | 0.237786067 | 1.580582 | 1.516569 | 1.523122 |
| | | | | | | | | | | | | |
| | | actin | p-SMAD2 | | | p-SMAD2/actin | | | Control mean | relative expression | | |
| 2 | NC | 11866349 | 3532017 | 2813383 | 2983878 | 0.29765 | 0.237089 | 0.251457 | 0.262065386 | 1.135785 | 0.904695 | 0.959521 |
| | siPIK3R2 | 12428083 | 4327334 | 4640829 | 4828201 | 0.34819 | 0.373415 | 0.388491 | 0.262065386 | 1.328638 | 1.424891 | 1.482421 |
| | NC+Ferritir | 11126568 | 5467418 | 5711005 | 6025598 | 0.491384 | 0.513276 | 0.54155 | 0.262065386 | 1.875044 | 1.958582 | 2.066471 |
| | K3R2+Fer | 12752926 | 2534648 | 2989219 | 3087929 | 0.19875 | 0.234395 | 0.242135 | 0.262065386 | 0.7584 | 0.894413 | 0.923949 |
| | | | | | | | | | | | | |
| | | actin | p-SMAD2 | | | p-SMAD2/actin | | | Control mean | relative expression | | |
| 3 | NC | 6593687 | 1563160 | 1573293 | 1538338 | 0.237069 | 0.238606 | 0.233305 | 0.236326606 | 1.003142 | 1.009645 | 0.987213 |
| | siPIK3R2 | 5155772 | 1224095 | 1231746 | 1384495 | 0.237422 | 0.238906 | 0.268533 | 0.236326606 | 1.004636 | 1.010915 | 1.136279 |
| | NC+Ferritir | 6179181 | 2409486 | 2487331 | 2569770 | 0.389936 | 0.402534 | 0.415876 | 0.236326606 | 1.649988 | 1.703296 | 1.759749 |
| | K3R2+Fer | 8230771 | 1146406 | 1194904 | 1247885 | 0.139283 | 0.145175 | 0.151612 | 0.236326606 | 0.589366 | 0.614299 | 0.641537 |

# Figure 6E-O

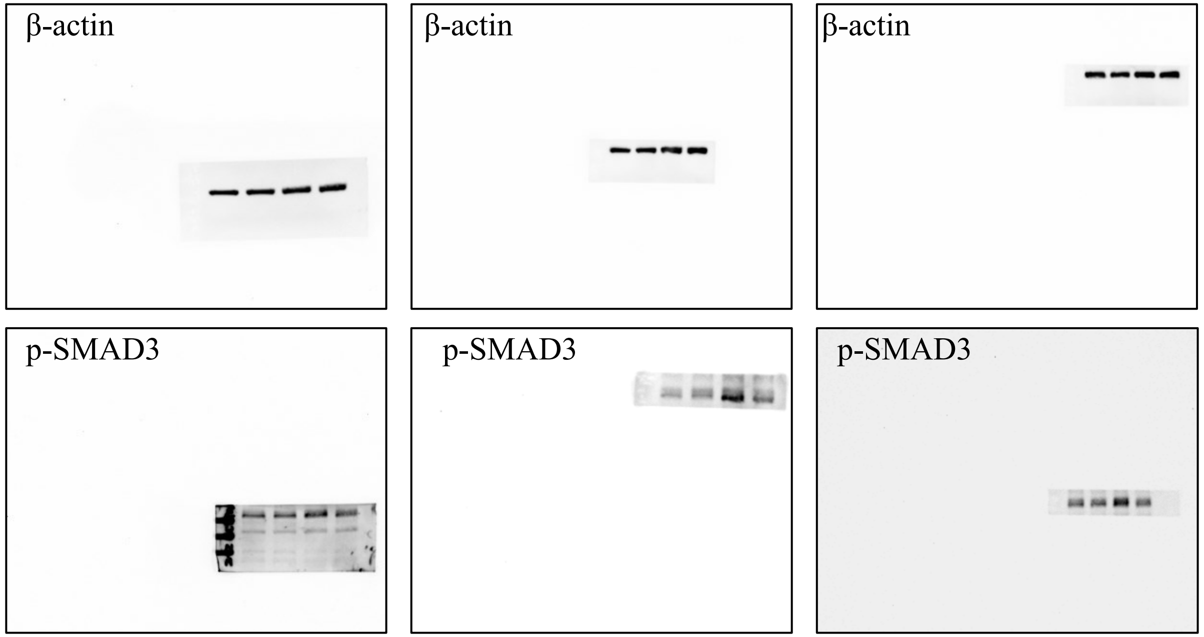


| | | actin | p-SMAD3 | | | p-SMAD3/actin | | | Control mean | relative expression | | |
|---|---|---|---|---|---|---|---|---|---|---|---|---|
| 1 | NC | 8915753 | 1783489 | 1748231 | 2172578 | 0.200038 | 0.196083 | 0.243679 | 0.213266638 | 0.937971 | 0.919428 | 1.142601 |
| | siPIK3R2 | 8763860 | 2146729 | 1814414 | 1882962 | 0.244952 | 0.207034 | 0.214855 | 0.213266638 | 1.148574 | 0.970774 | 1.007449 |
| | NC+Ferritin | 9328798 | 3642318 | 3960879 | 4306489 | 0.390438 | 0.424586 | 0.461634 | 0.213266638 | 1.830751 | 1.99087 | 2.164585 |
| | siPIK3R2+Ferritin | 10306119 | 1833988 | 2183115 | 2604100 | 0.177951 | 0.211827 | 0.252675 | 0.213266638 | 0.834408 | 0.99325 | 1.184785 |
| | | | | | | | | | | | | |
| | | actin | p-SMAD3 | | | p-SMAD3/actin | | | Control mean | relative expression | | |
| 2 | NC | 9932074 | 837857 | 917356 | 965276 | 0.084359 | 0.092363 | 0.097188 | 0.091303152 | 0.923941 | 1.011608 | 1.064451 |
| | siPIK3R2 | 9596483 | 1028540 | 1132302 | 1280721 | 0.107179 | 0.117991 | 0.133457 | 0.091303152 | 1.173879 | 1.292303 | 1.461695 |
| | NC+Ferritin | 11354641 | 2236215 | 2810257 | 3066333 | 0.196943 | 0.247499 | 0.270051 | 0.091303152 | 2.157021 | 2.710734 | 2.957741 |
| | siPIK3R2+Ferritin | 12888798 | 1145052 | 1078556 | 1536609 | 0.088841 | 0.083682 | 0.119221 | 0.091303152 | 0.973032 | 0.916525 | 1.305765 |
| | | | | | | | | | | | | |
| | | actin | p-SMAD3 | | | p-SMAD3/actin | | | Control mean | relative expression | | |
| 3 | NC | 10934617 | 1761920 | 1673391 | 1759175 | 0.161132 | 0.153036 | 0.160881 | 0.158349884 | 1.017571 | 0.966443 | 1.015986 |
| | siPIK3R2 | 8880241 | 1811105 | 1709978 | 1707807 | 0.203948 | 0.19256 | 0.192315 | 0.158349884 | 1.287956 | 1.21604 | 1.214497 |
| | NC+Ferritin | 11833396 | 2682872 | 2893422 | 2813514 | 0.22672 | 0.244513 | 0.23776 | 0.158349884 | 1.431769 | 1.544133 | 1.501488 |
| | siPIK3R2+Ferritin | 12557214 | 1667477 | 1606880 | 1678903 | 0.13279 | 0.127965 | 0.1337 | 0.158349884 | 0.838588 | 0.808114 | 0.844335 |
