# Supplementary material for: Macrophage–Derived Ferritin Exacerbates Silica‐Induced Pulmonary Fibrosis via PIK3R2‐Mediated Fibroblast Differentiation
Source: Adv Sci (Weinh). 2026 Jan 21;13(17):e19191. doi: 10.1002/advs.202519191 (PMC13042690; doi:10.1002/advs.202519191)
Supplement: Supplementary file 4 — Supporting File 4: advs73867‐sup‐0001‐FiguresData.zip. [file ADVS-13-e19191-s001.zip › Supporting information Figure1-10/Figure 7/Figure 7G-I.pdf]

Figure 7G-I

Raw264.7

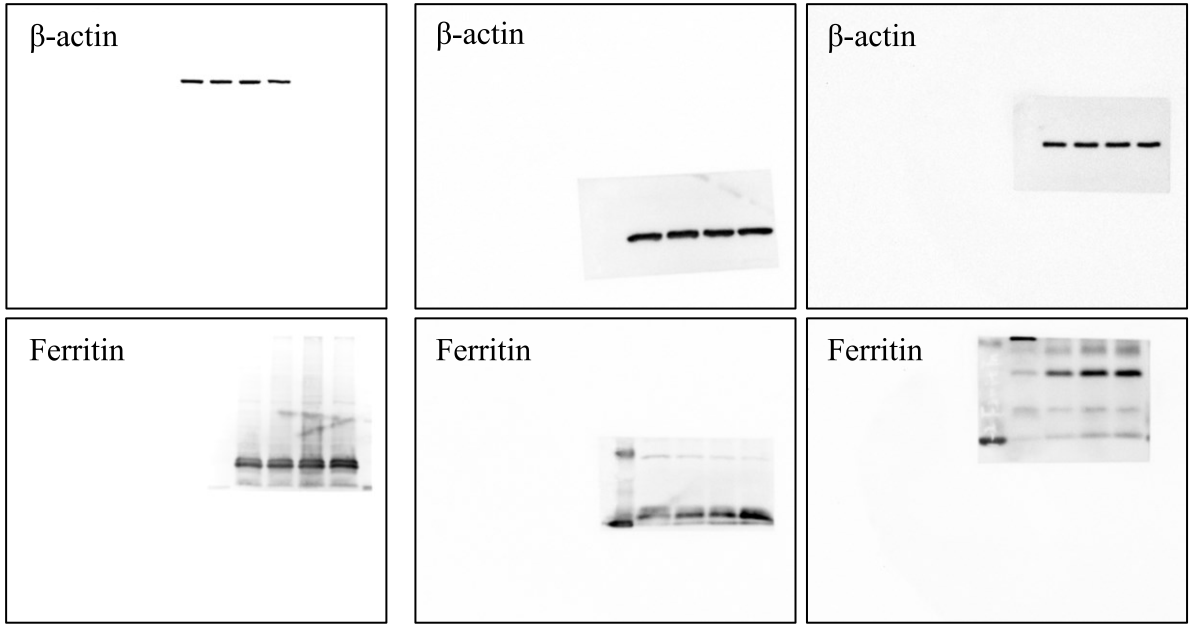

	silica (μg/mL)	actin	Ferritin			Ferritin/actin			Control mean	relative expression		
1	0	8980135	27683849	26096980	29645289	3.082788	2.906079	3.301207	3.096691308	0.99551	0.938446	1.066043
	100	8726195	28157447	30011829	32459316	3.226773	3.43928	3.719756	3.096691308	1.042007	1.110631	1.201203
	200	9385437	35662356	35157762	38431321	3.799754	3.745991	4.094782	3.096691308	1.227037	1.209675	1.322309
	400	7570415	40422969	39329638	42894944	5.339597	5.195176	5.666128	3.096691308	1.724291	1.677654	1.829736
	silica (μg/mL)	actin	Ferritin			Ferritin/actin			Control mean	relative expression		
2	0	4363543	3821161	3131812	3616254	0.875701	0.717722	0.828743	0.807388782	1.084609	0.888943	1.026448
	100	3852067	3641388	3671329	3671329	0.945308	0.95308	0.95308	0.807388782	1.170821	1.180448	1.180448
	200	3464205	3379655	3374698	3667928	0.975593	0.974162	1.058808	0.807388782	1.208331	1.206559	1.311398
	400	4603530	10309197	10179829	10412732	2.239411	2.211309	2.261902	0.807388782	2.773647	2.738841	2.801502
	silica (μg/mL)	actin	Ferritin			Ferritin/actin			Control mean	relative expression		
3	0	919409	2169898	1800269	1948513	2.360101	1.958072	2.11931	2.145827736	1.099856	0.912502	0.987642
	100	940751	4085496	3644048	3862004	4.342803	3.873552	4.105235	2.145827736	2.023836	1.805155	1.913124
	200	988839	5942791	5352562	5301571	6.009867	5.412976	5.36141	2.145827736	2.800722	2.522559	2.498528
	400	993874	6405797	6026840	5804533	6.445281	6.063988	5.840311	2.145827736	3.003634	2.825944	2.721705

Figure 7G-I

THP-1

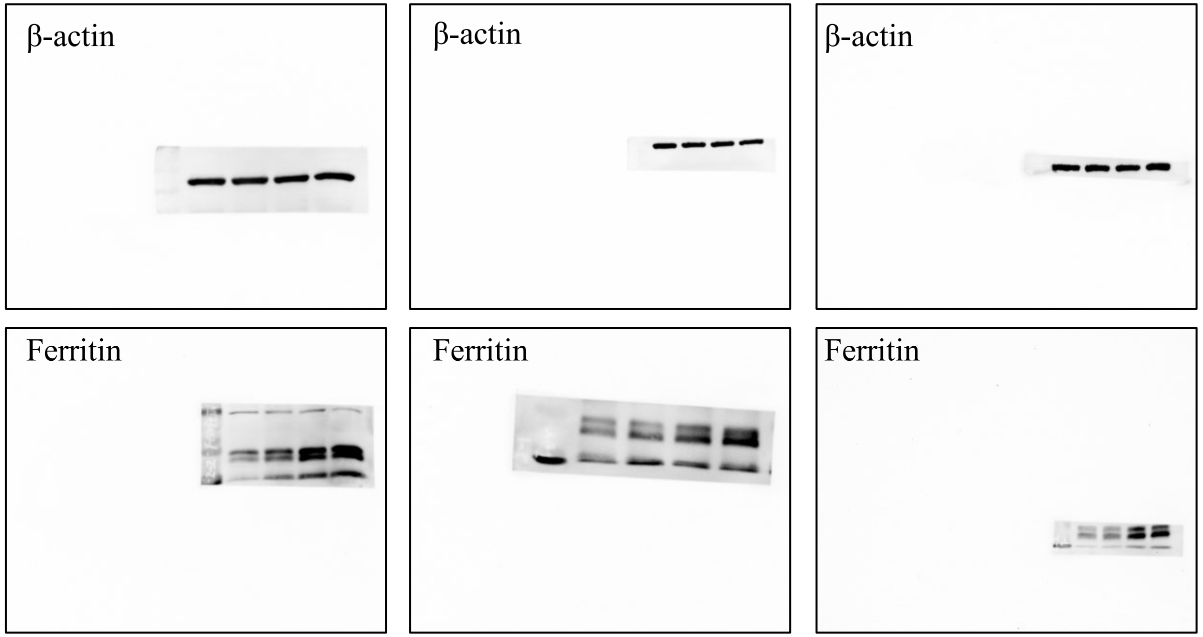

	silica (μg/mL)	actin	Ferritin			Ferritin/actin			Control mean	relative expression		
1	0	16471647	13150081	13508194	13089641	0.798346	0.820088	0.794677	0.804370403	0.992511	1.01954	0.987949
	100	15851062	20798889	22122302	20035898	1.312145	1.395635	1.26401	0.804370403	1.631269	1.735065	1.571428
	200	15461521	32408809	31752760	28874598	2.096094	2.053663	1.867513	0.804370403	2.605882	2.553132	2.321708
	400	18587971	40216100	39682072	41844673	2.163555	2.134825	2.251169	0.804370403	2.68975	2.654033	2.798673
	silica (μg/mL)	actin	Ferritin			Ferritin/actin			Control mean	relative expression		
2	0	20726439	8063237	7761165	7209862	0.389031	0.374457	0.347858	0.370448971	1.050162	1.01082	0.939018
	100	19214056	9735844	9230823	8613459	0.506704	0.48042	0.448289	0.370448971	1.367811	1.29686	1.210125
	200	18745785	15085616	14181148	12912824	0.804747	0.756498	0.688839	0.370448971	2.172356	2.042111	1.85947
	400	18311642	20454154	19236449	18324589	1.117003	1.050504	1.000707	0.370448971	3.015267	2.835758	2.701336
	silica (μg/mL)	actin	Ferritin			Ferritin/actin			Control mean	relative expression		
3	0	1922184	6451029	6577176	6451029	3.356093	3.42172	3.356093	3.377969019	0.993524	1.012952	0.993524
	100	1805964	7980563	7980563	7666185	4.419004	4.419004	4.244927	3.377969019	1.308184	1.308184	1.256651
	200	1709564	16868091	17370223	16494916	9.866896	10.16062	9.64861	3.377969019	2.920955	3.007907	2.856335
	400	1892008	16634620	16715586	16947559	8.792045	8.834839	8.957446	3.377969019	2.602761	2.615429	2.651725
