# Supplementary figures and images for: Macrophage–Derived Ferritin Exacerbates Silica‐Induced Pulmonary Fibrosis via PIK3R2‐Mediated Fibroblast Differentiation (part 4 of 4)
Source: Adv Sci (Weinh). 2026 Jan 21;13(17):e19191. doi: 10.1002/advs.202519191 (PMC13042690; doi:10.1002/advs.202519191)

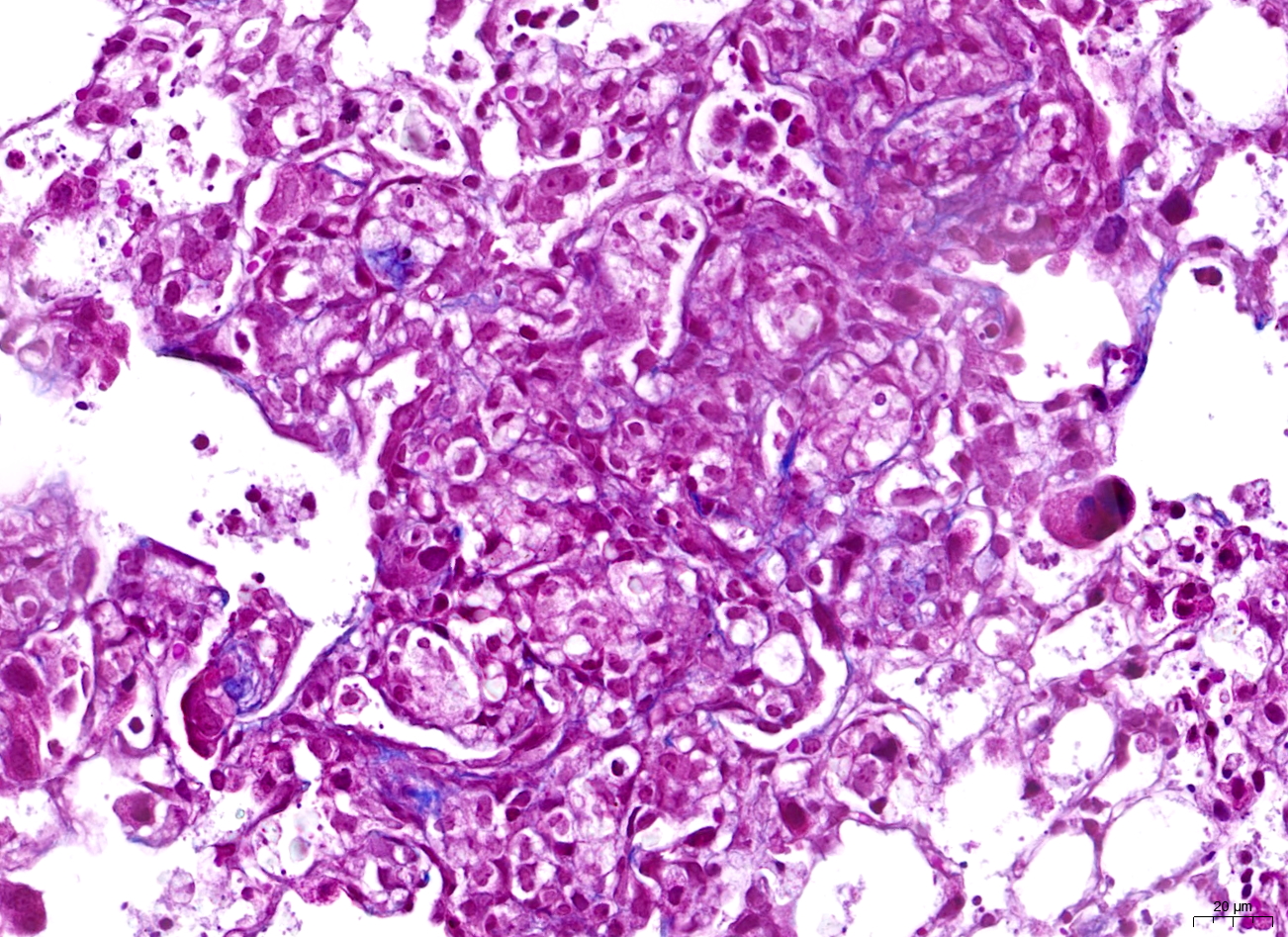

Supplement: Supplementary file 4 — Supporting File 4: advs73867‐sup‐0001‐FiguresData.zip. [file ADVS-13-e19191-s001.zip › Supporting information Figure1-10/Figure 3/Figure 3D/Silica/silica-112_40.0x-2.jpg]

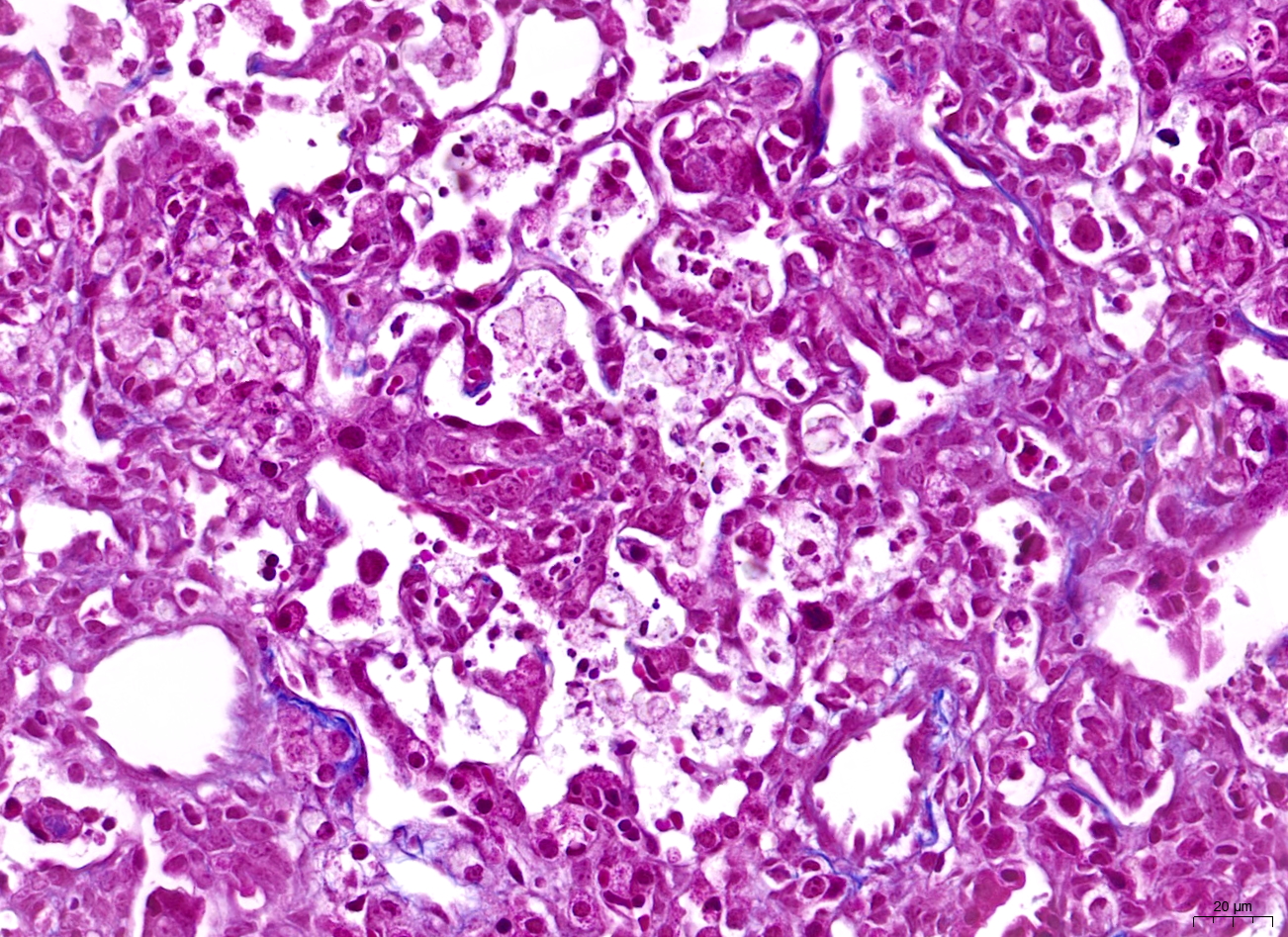

Supplement: Supplementary file 4 — Supporting File 4: advs73867‐sup‐0001‐FiguresData.zip. [file ADVS-13-e19191-s001.zip › Supporting information Figure1-10/Figure 3/Figure 3D/Silica/silica-112_40.0x-3.jpg]

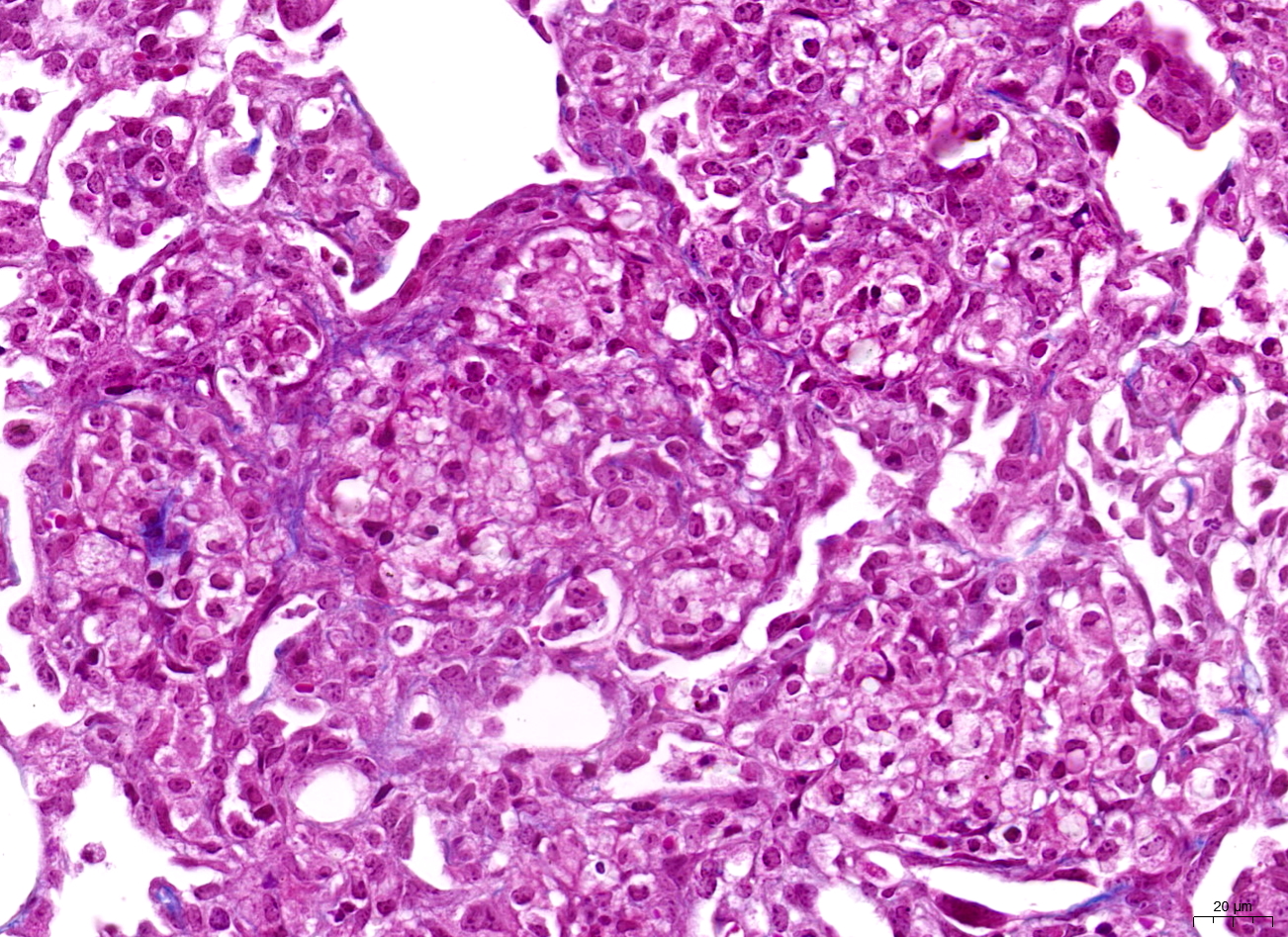

Supplement: Supplementary file 4 — Supporting File 4: advs73867‐sup‐0001‐FiguresData.zip. [file ADVS-13-e19191-s001.zip › Supporting information Figure1-10/Figure 3/Figure 3D/Silica/silica-113_40.0x.jpg]

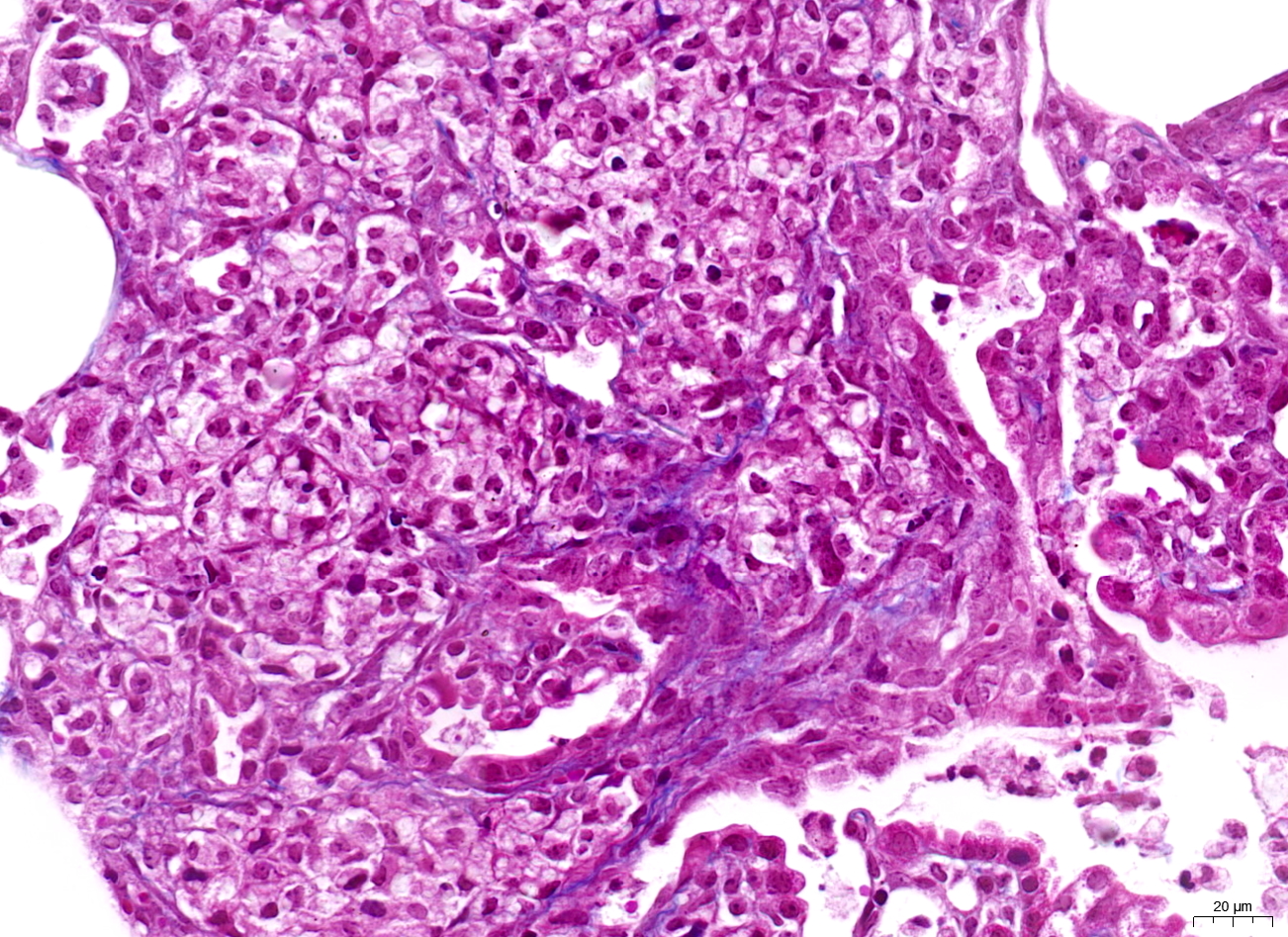

Supplement: Supplementary file 4 — Supporting File 4: advs73867‐sup‐0001‐FiguresData.zip. [file ADVS-13-e19191-s001.zip › Supporting information Figure1-10/Figure 3/Figure 3D/Silica/silica-113_40.0x-2.jpg]

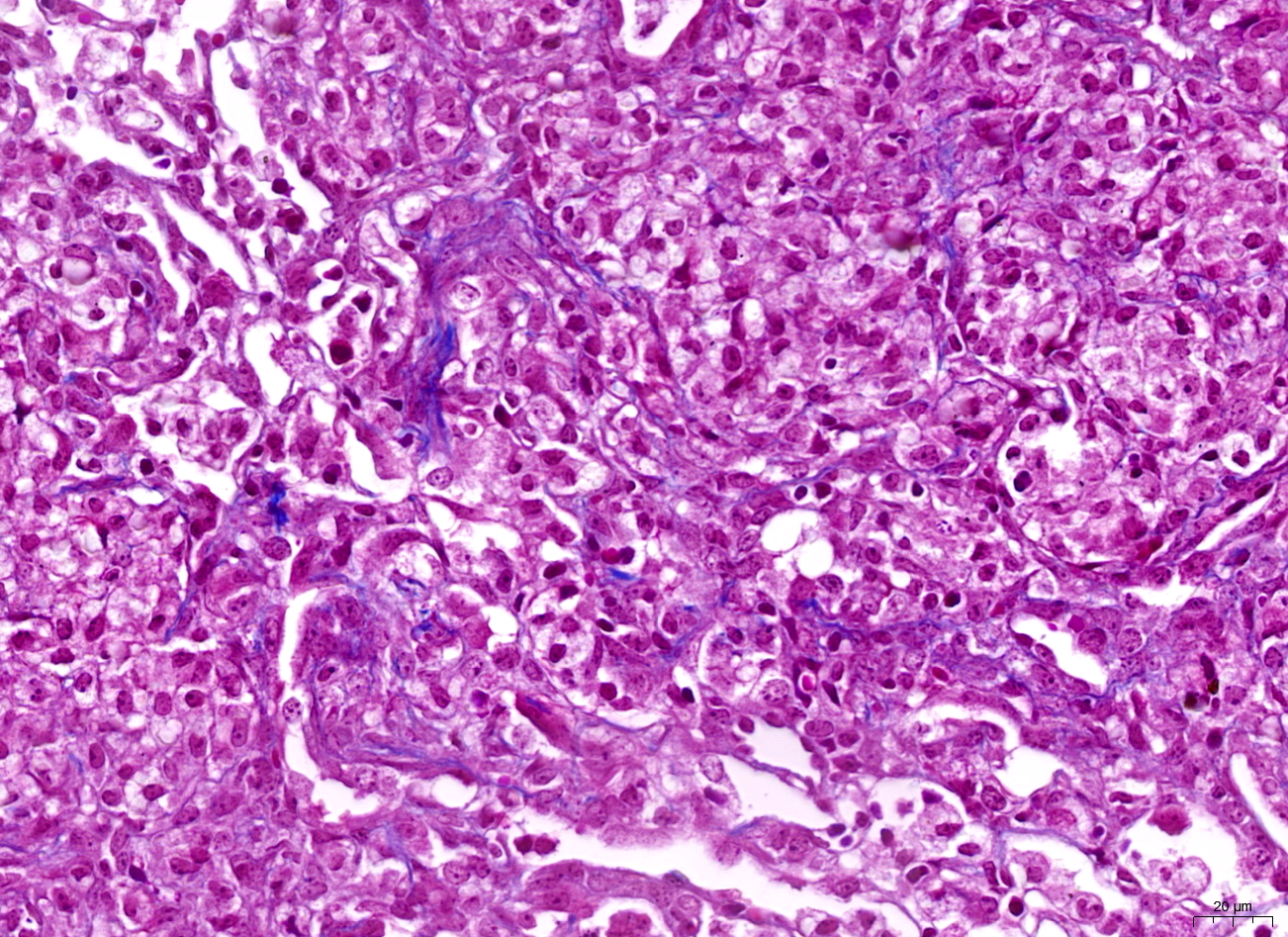

Supplement: Supplementary file 4 — Supporting File 4: advs73867‐sup‐0001‐FiguresData.zip. [file ADVS-13-e19191-s001.zip › Supporting information Figure1-10/Figure 3/Figure 3D/Silica/silica-113_40.0x-3.jpg]

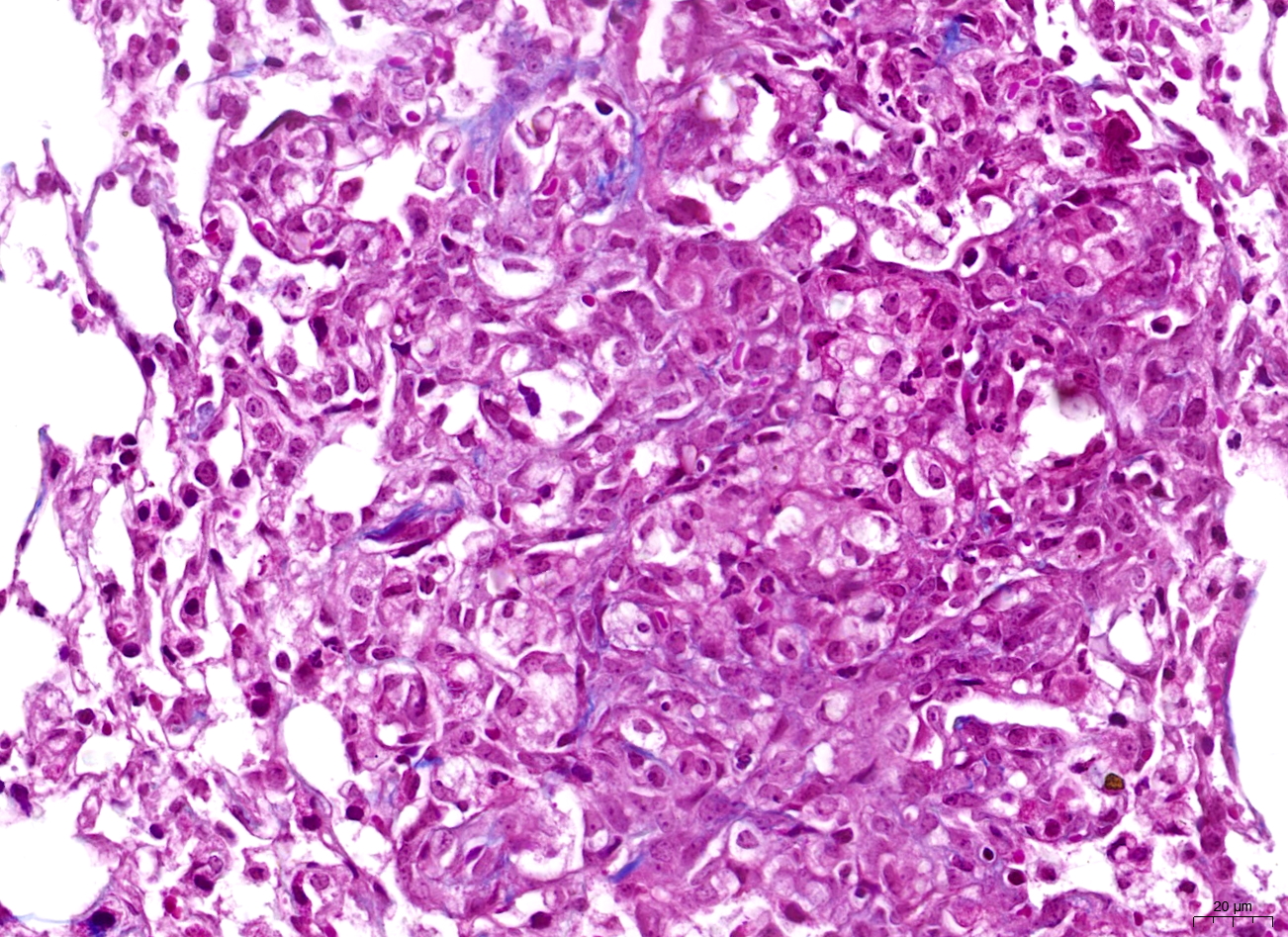

Supplement: Supplementary file 4 — Supporting File 4: advs73867‐sup‐0001‐FiguresData.zip. [file ADVS-13-e19191-s001.zip › Supporting information Figure1-10/Figure 3/Figure 3D/Silica/silica-114_40.0x.jpg]

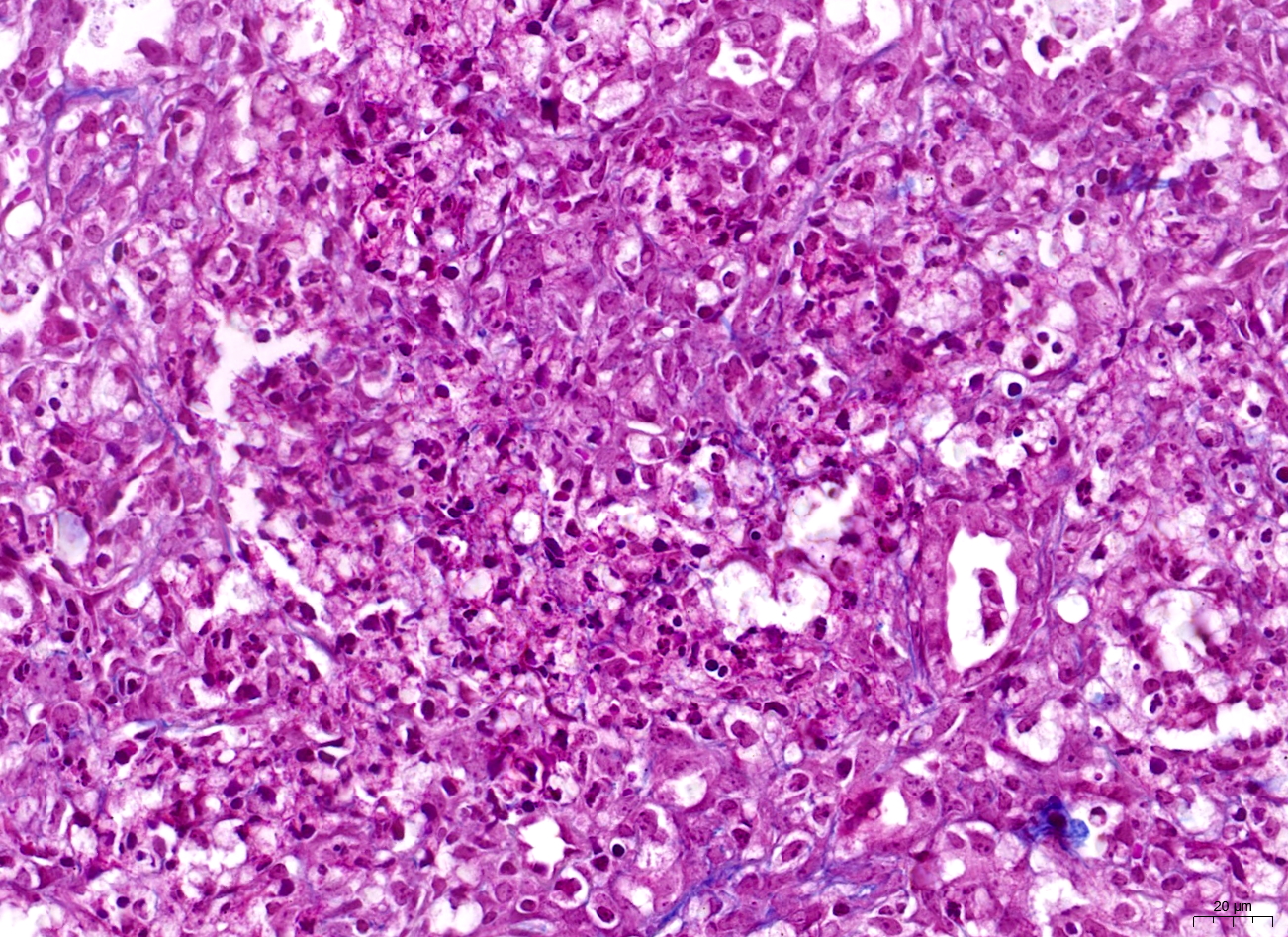

Supplement: Supplementary file 4 — Supporting File 4: advs73867‐sup‐0001‐FiguresData.zip. [file ADVS-13-e19191-s001.zip › Supporting information Figure1-10/Figure 3/Figure 3D/Silica/silica-114_40.0x-2.jpg]

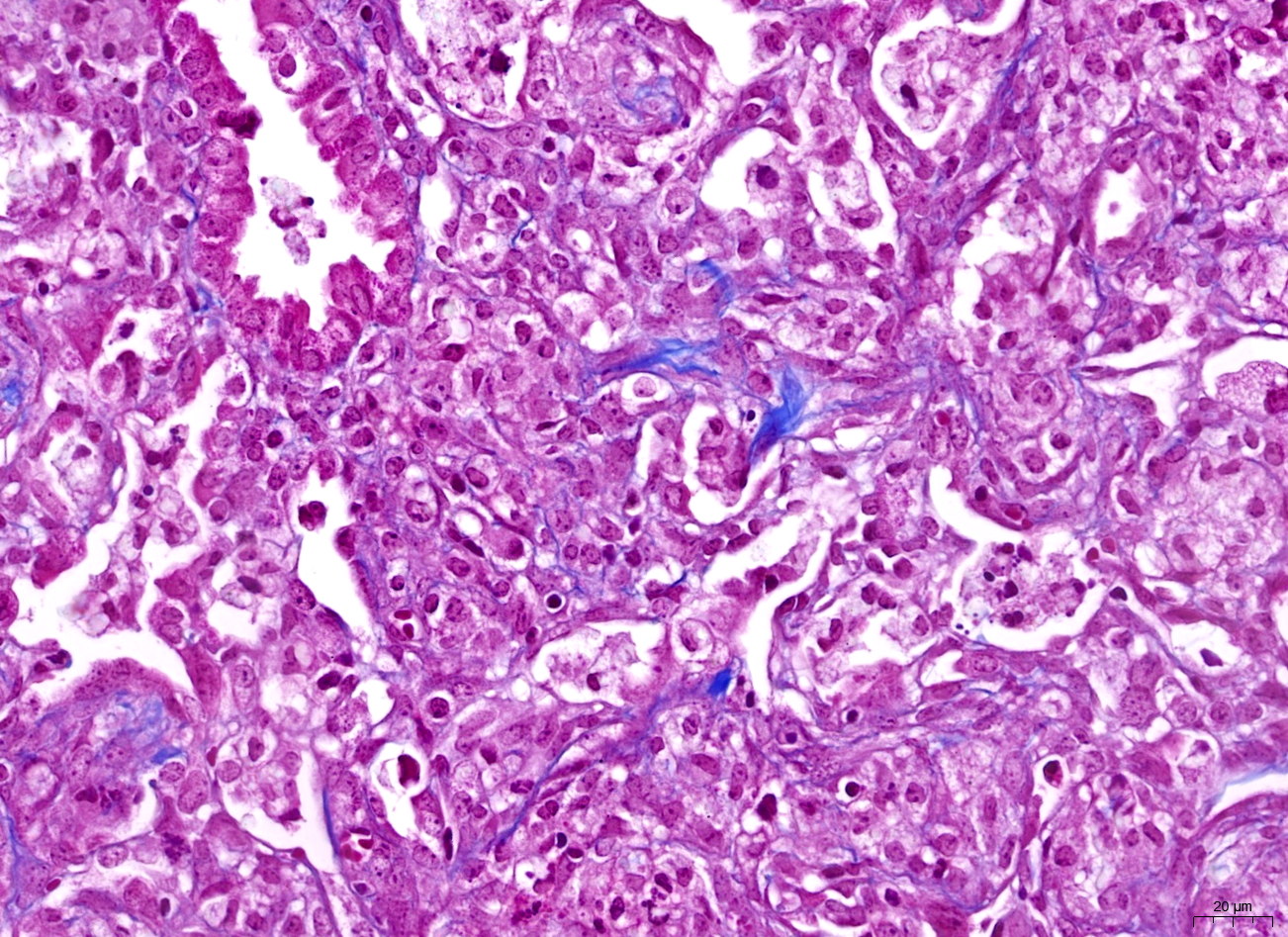

Supplement: Supplementary file 4 — Supporting File 4: advs73867‐sup‐0001‐FiguresData.zip. [file ADVS-13-e19191-s001.zip › Supporting information Figure1-10/Figure 3/Figure 3D/Silica/silica-114_40.0x-3.jpg]

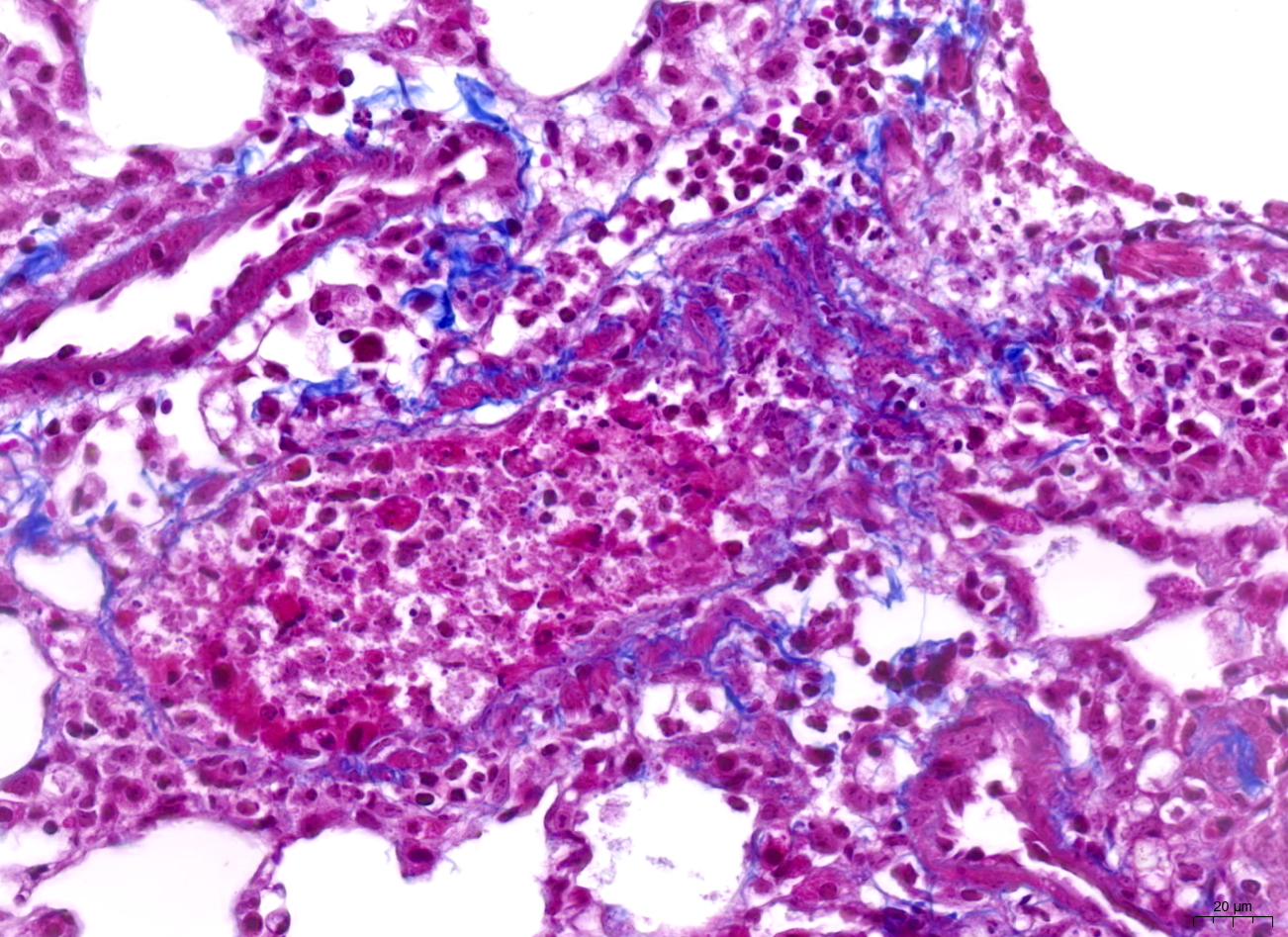

Supplement: Supplementary file 4 — Supporting File 4: advs73867‐sup‐0001‐FiguresData.zip. [file ADVS-13-e19191-s001.zip › Supporting information Figure1-10/Figure 3/Figure 3D/Silica+Ferritin/Silica+Ferritin-117_40.0x.jpg]

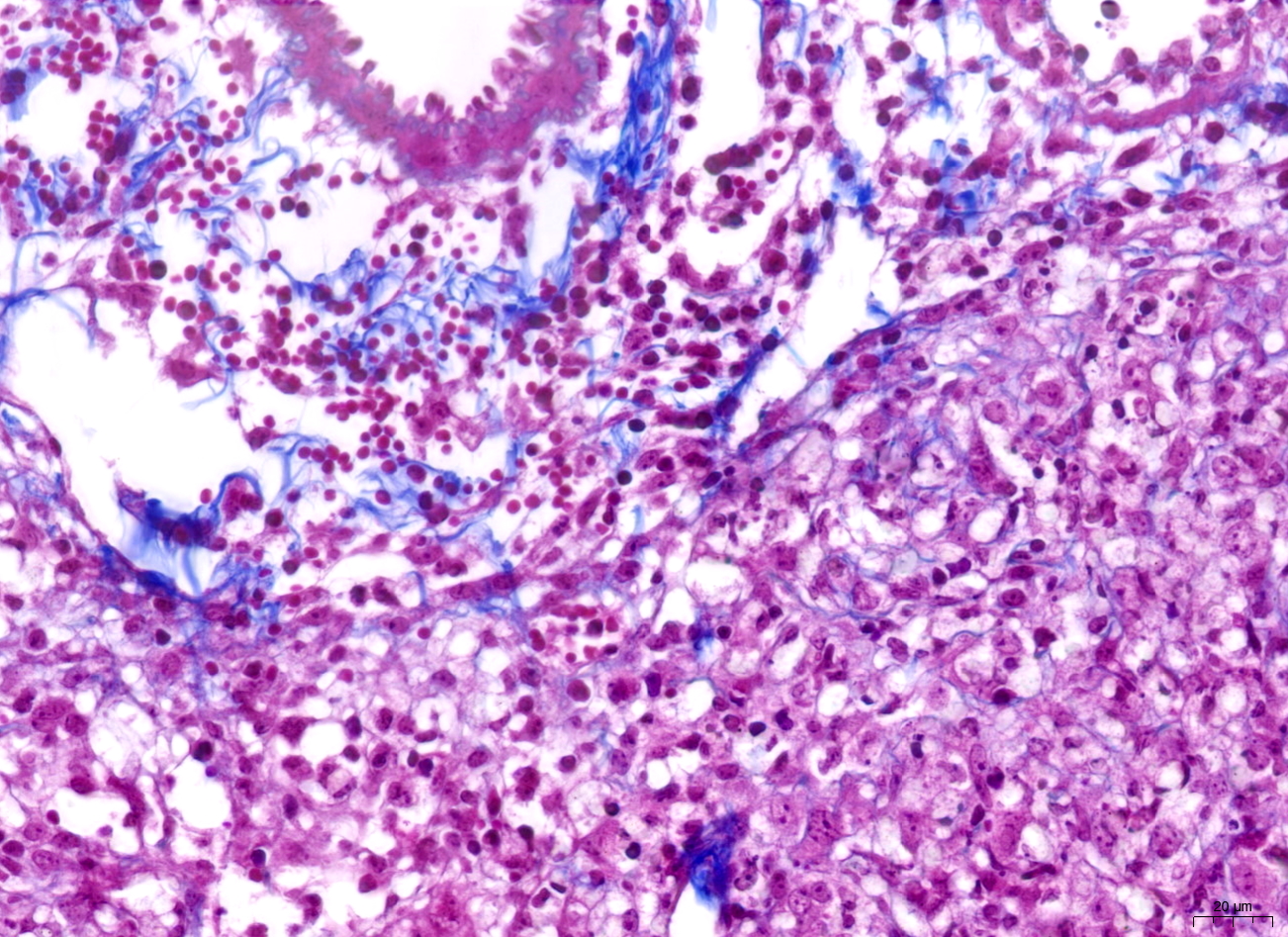

Supplement: Supplementary file 4 — Supporting File 4: advs73867‐sup‐0001‐FiguresData.zip. [file ADVS-13-e19191-s001.zip › Supporting information Figure1-10/Figure 3/Figure 3D/Silica+Ferritin/Silica+Ferritin-117_40.0x-2.jpg]

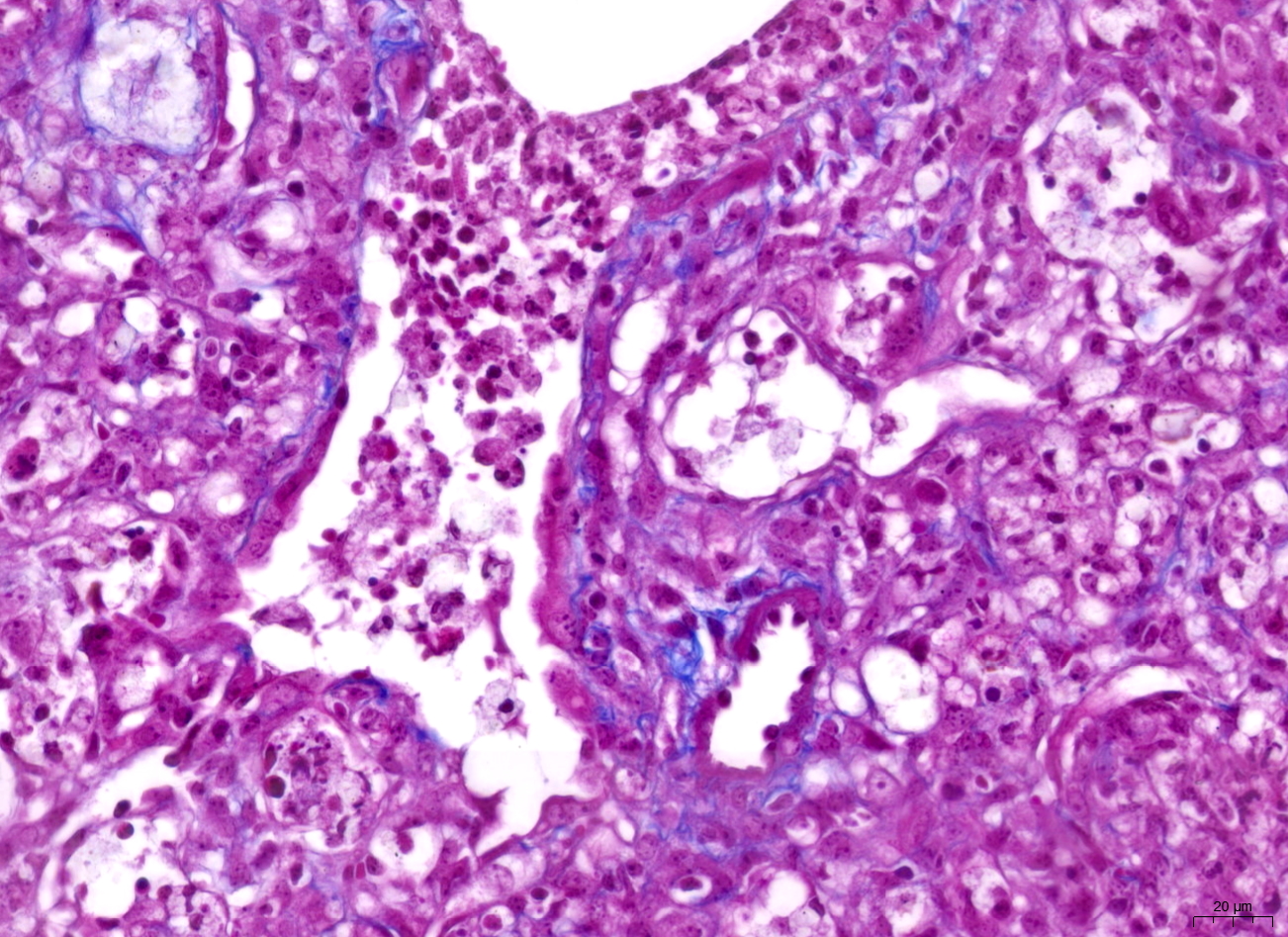

Supplement: Supplementary file 4 — Supporting File 4: advs73867‐sup‐0001‐FiguresData.zip. [file ADVS-13-e19191-s001.zip › Supporting information Figure1-10/Figure 3/Figure 3D/Silica+Ferritin/Silica+Ferritin-117_40.0x-3.jpg]

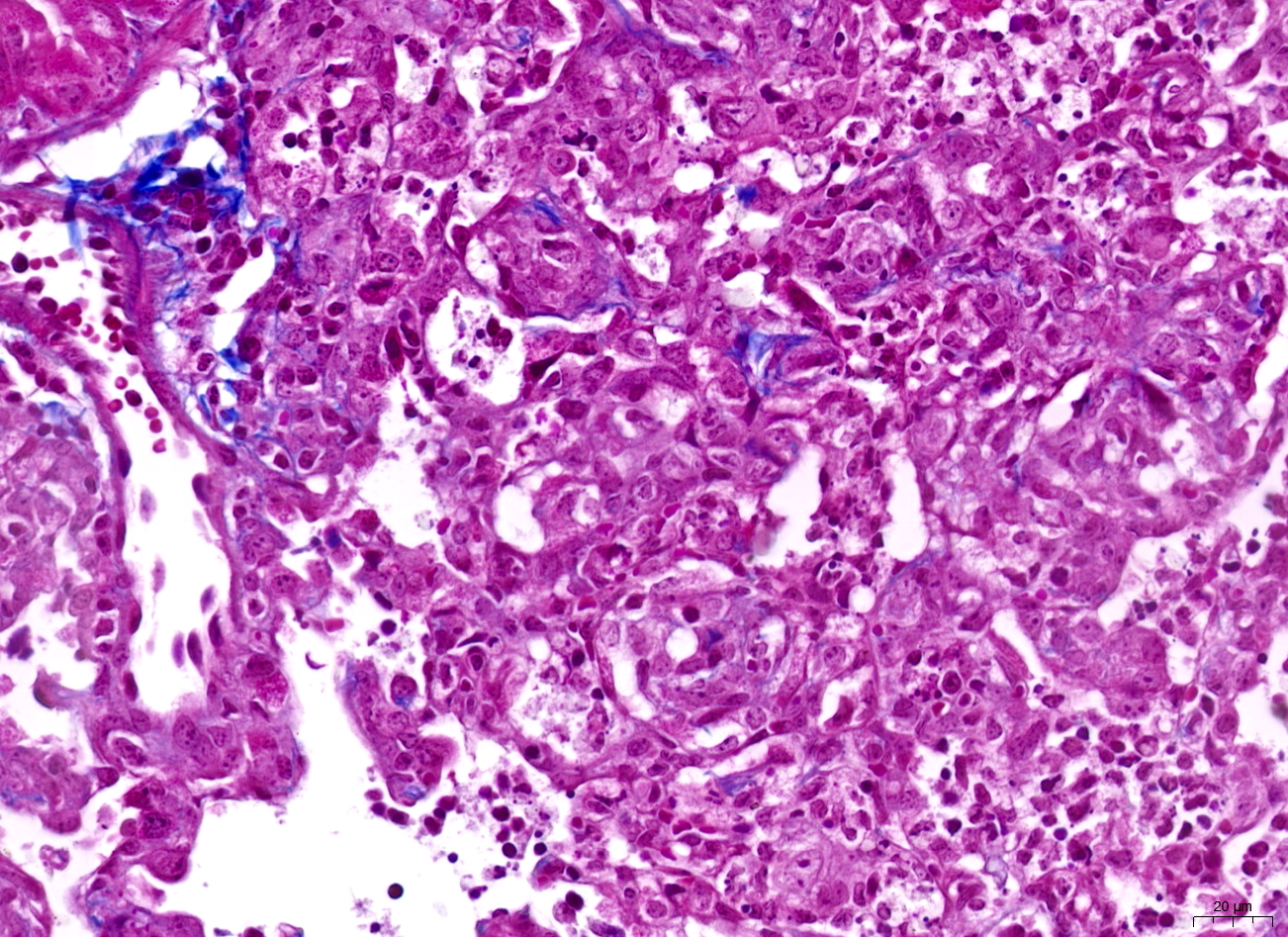

Supplement: Supplementary file 4 — Supporting File 4: advs73867‐sup‐0001‐FiguresData.zip. [file ADVS-13-e19191-s001.zip › Supporting information Figure1-10/Figure 3/Figure 3D/Silica+Ferritin/Silica+Ferritin-118_40.0x.jpg]

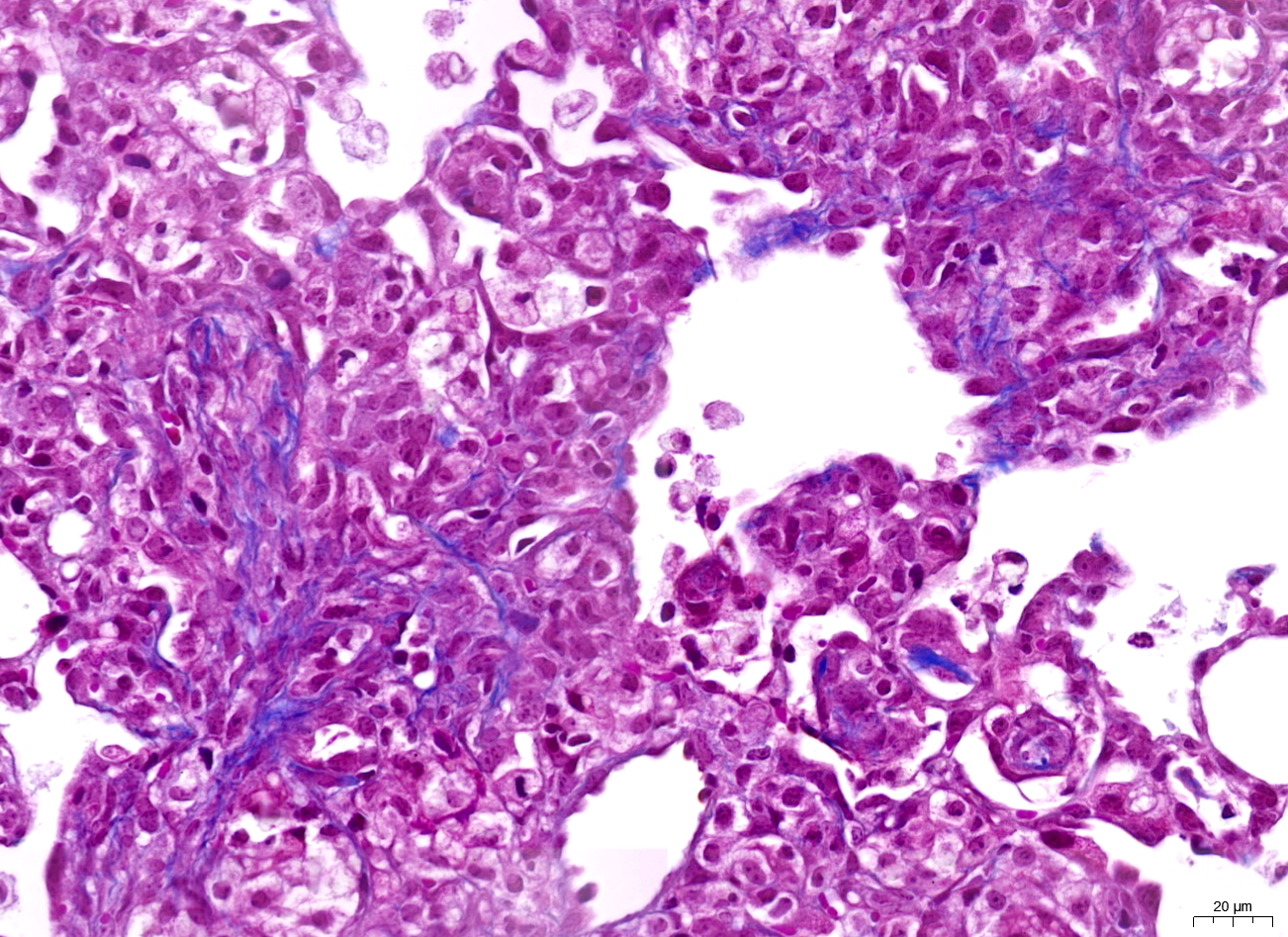

Supplement: Supplementary file 4 — Supporting File 4: advs73867‐sup‐0001‐FiguresData.zip. [file ADVS-13-e19191-s001.zip › Supporting information Figure1-10/Figure 3/Figure 3D/Silica+Ferritin/Silica+Ferritin-118_40.0x-2.jpg]

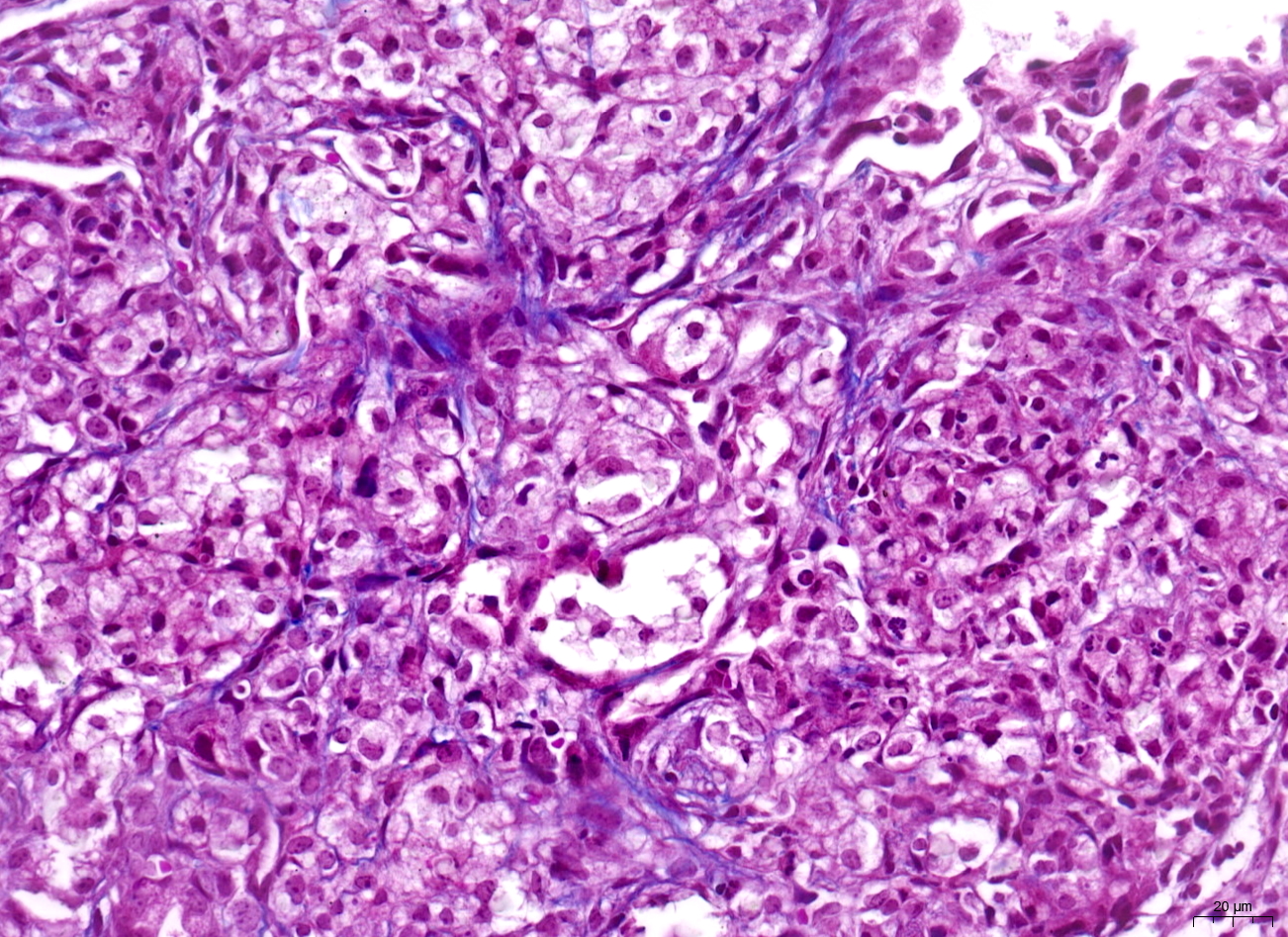

Supplement: Supplementary file 4 — Supporting File 4: advs73867‐sup‐0001‐FiguresData.zip. [file ADVS-13-e19191-s001.zip › Supporting information Figure1-10/Figure 3/Figure 3D/Silica+Ferritin/Silica+Ferritin-118_40.0x-3.jpg]

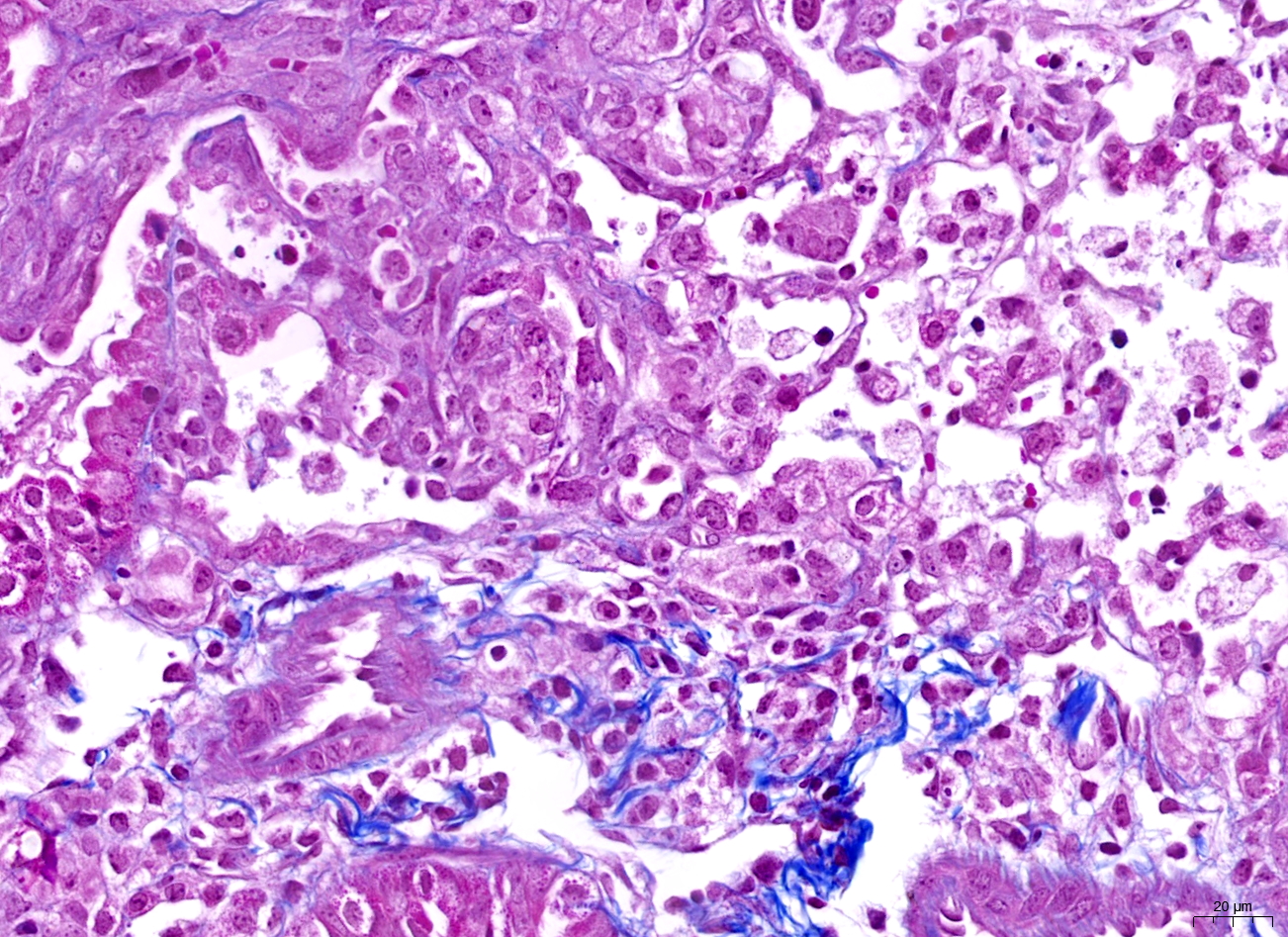

Supplement: Supplementary file 4 — Supporting File 4: advs73867‐sup‐0001‐FiguresData.zip. [file ADVS-13-e19191-s001.zip › Supporting information Figure1-10/Figure 3/Figure 3D/Silica+Ferritin/Silica+Ferritin-120_40.0x.jpg]

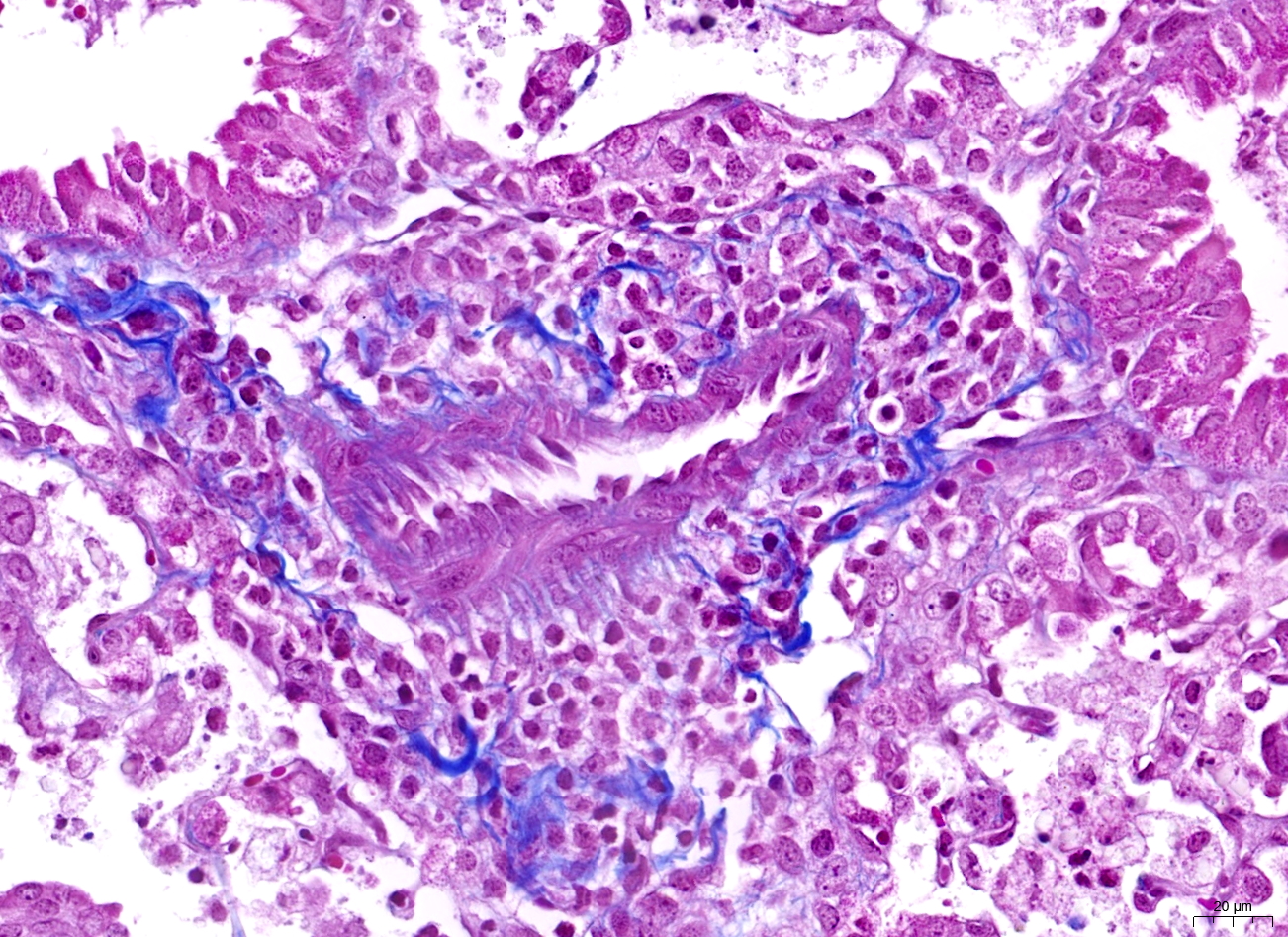

Supplement: Supplementary file 4 — Supporting File 4: advs73867‐sup‐0001‐FiguresData.zip. [file ADVS-13-e19191-s001.zip › Supporting information Figure1-10/Figure 3/Figure 3D/Silica+Ferritin/Silica+Ferritin-120_40.0x-2.jpg]

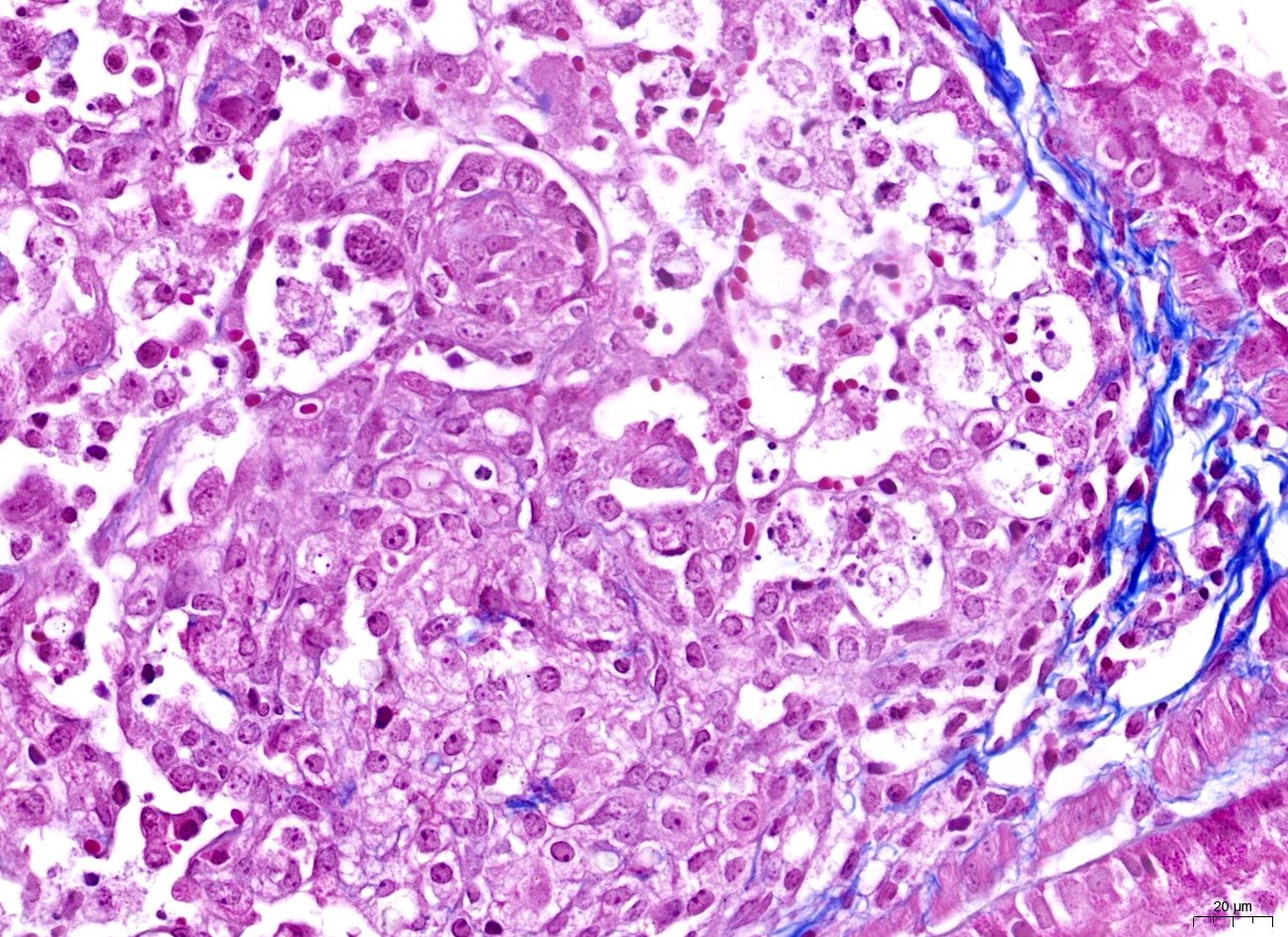

Supplement: Supplementary file 4 — Supporting File 4: advs73867‐sup‐0001‐FiguresData.zip. [file ADVS-13-e19191-s001.zip › Supporting information Figure1-10/Figure 3/Figure 3D/Silica+Ferritin/Silica+Ferritin-120_40.0x-3.jpg]

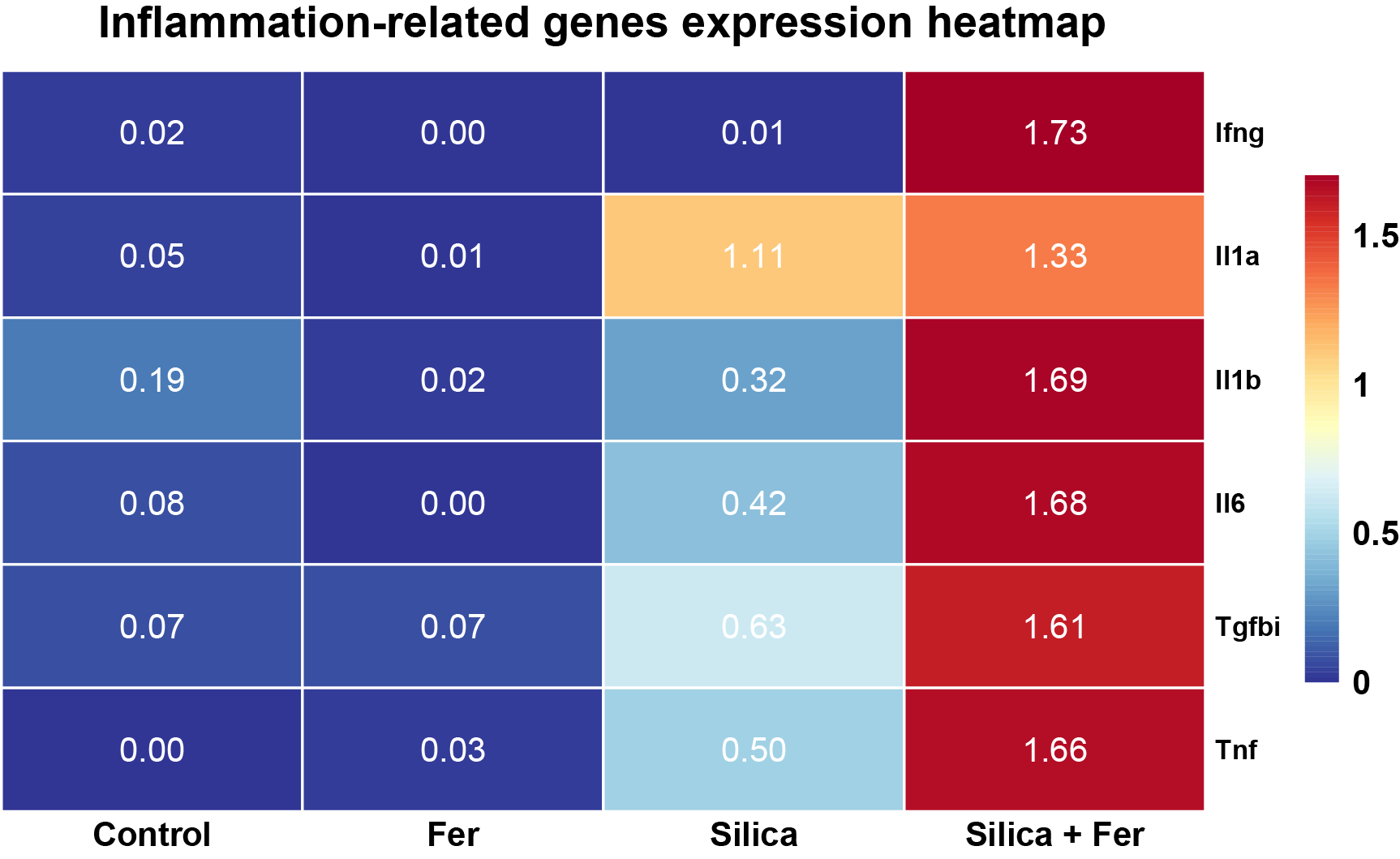

Supplement: Supplementary file 4 — Supporting File 4: advs73867‐sup‐0001‐FiguresData.zip. [file ADVS-13-e19191-s001.zip › Supporting information Figure1-10/Figure 3/Figure 3J/Figure 3J.png]

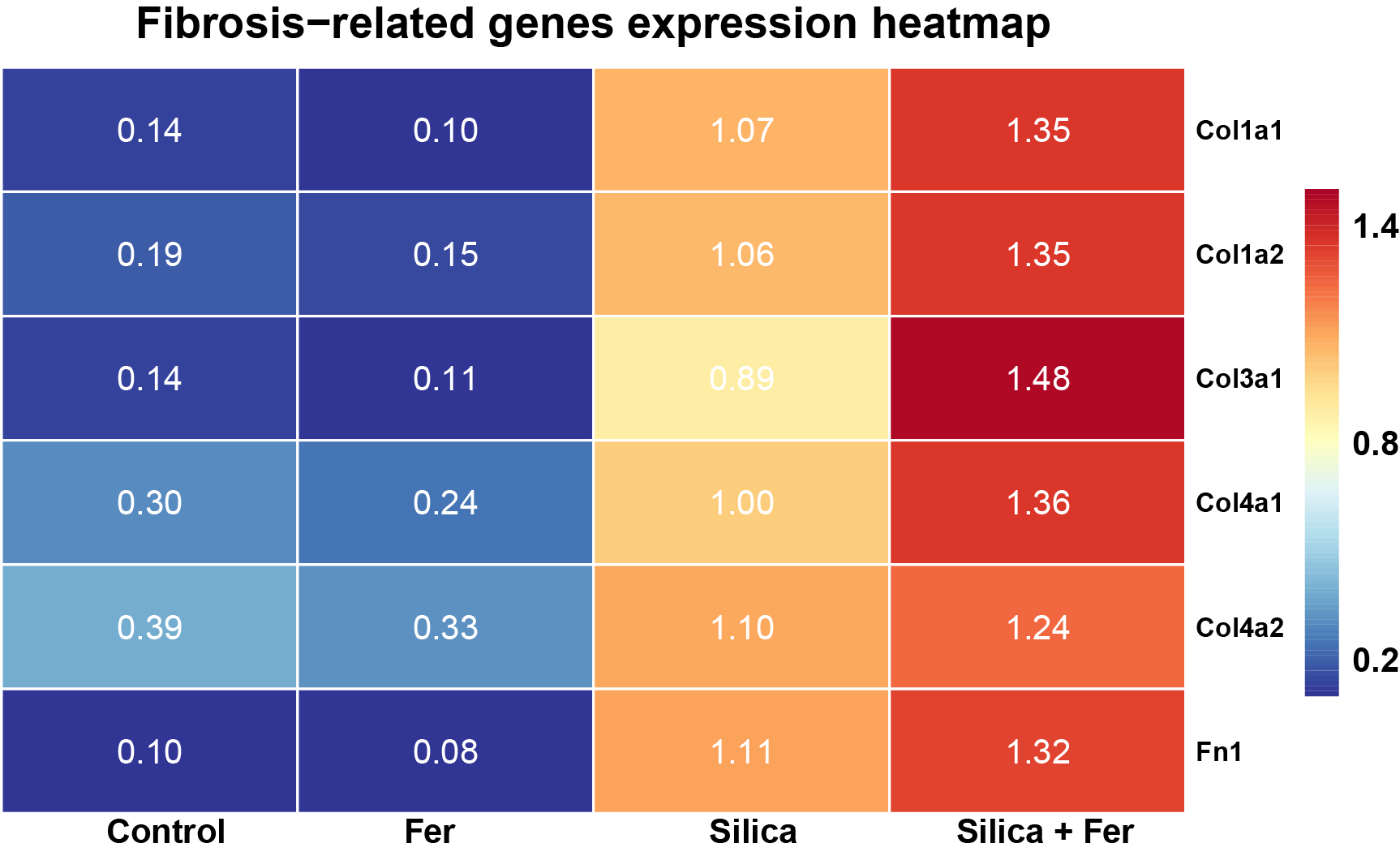

Supplement: Supplementary file 4 — Supporting File 4: advs73867‐sup‐0001‐FiguresData.zip. [file ADVS-13-e19191-s001.zip › Supporting information Figure1-10/Figure 3/Figure 3K/Figure 3K.png]

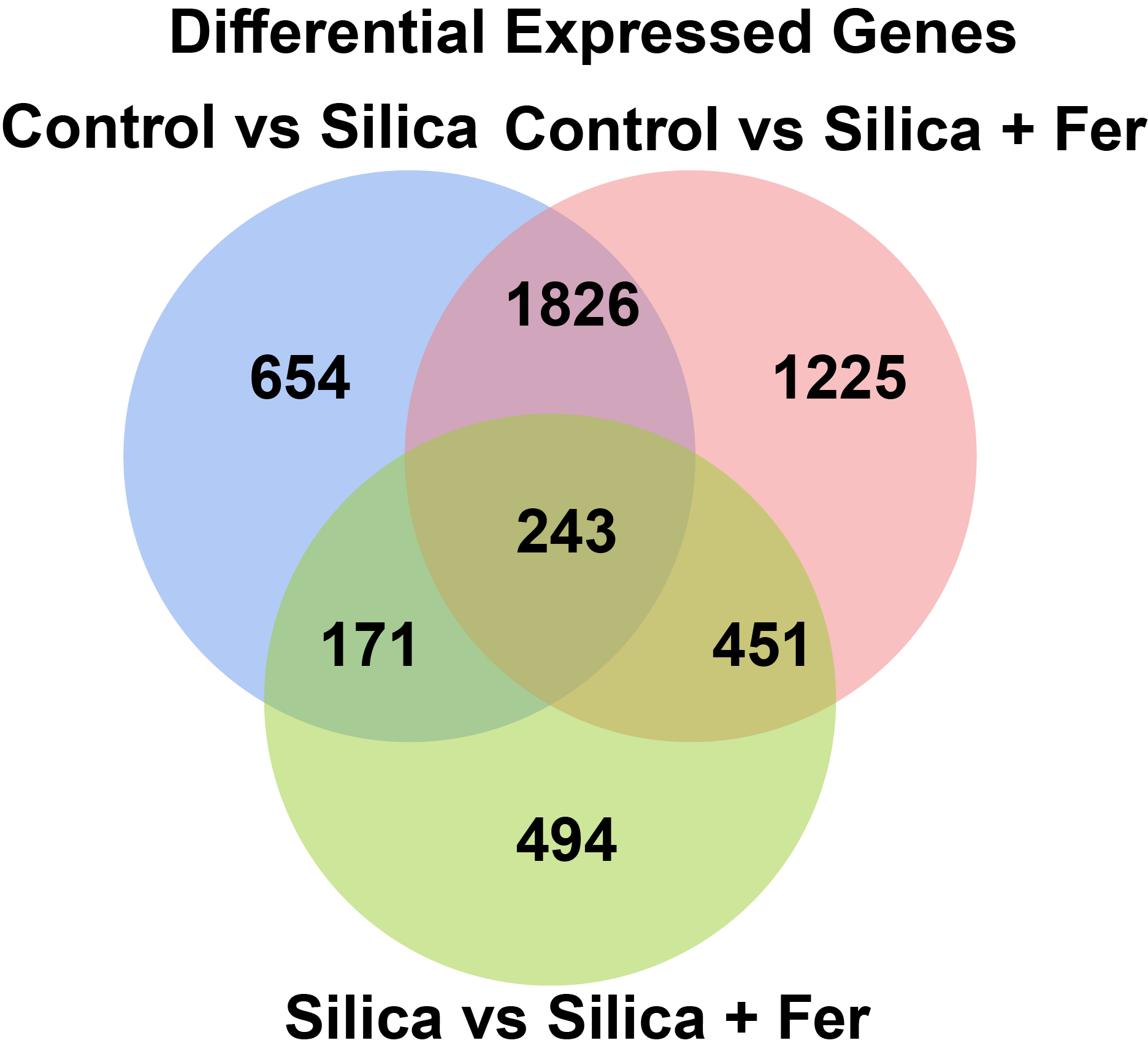

Supplement: Supplementary file 4 — Supporting File 4: advs73867‐sup‐0001‐FiguresData.zip. [file ADVS-13-e19191-s001.zip › Supporting information Figure1-10/Figure 4/Figure 4A/Venn Analysis/Venn analysis.png]

**Figure 4B**

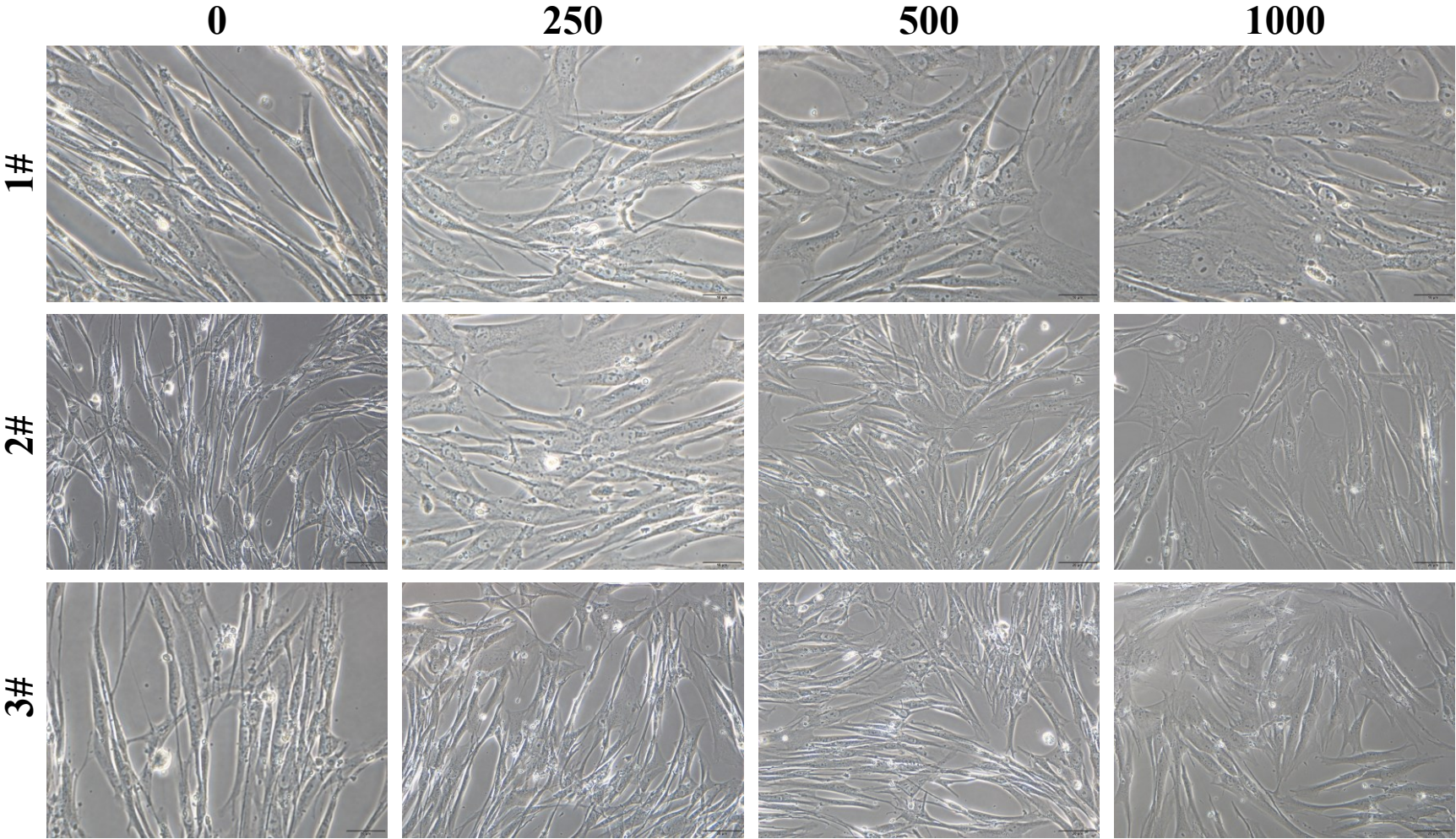

Supplement: Supplementary file 4 — Supporting File 4: advs73867‐sup‐0001‐FiguresData.zip. [file ADVS-13-e19191-s001.zip › Supporting information Figure1-10/Figure 4/Figure 4B.pdf]

Figure 4L-O

$\alpha$ -SMA

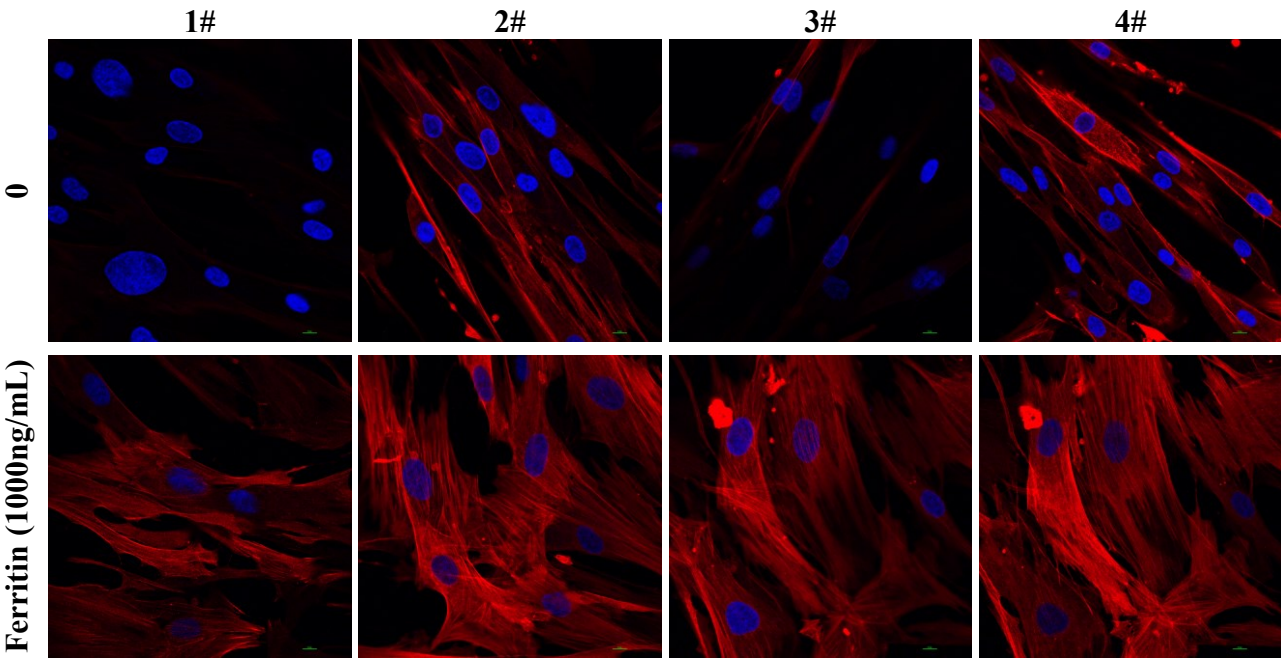

Collagen I

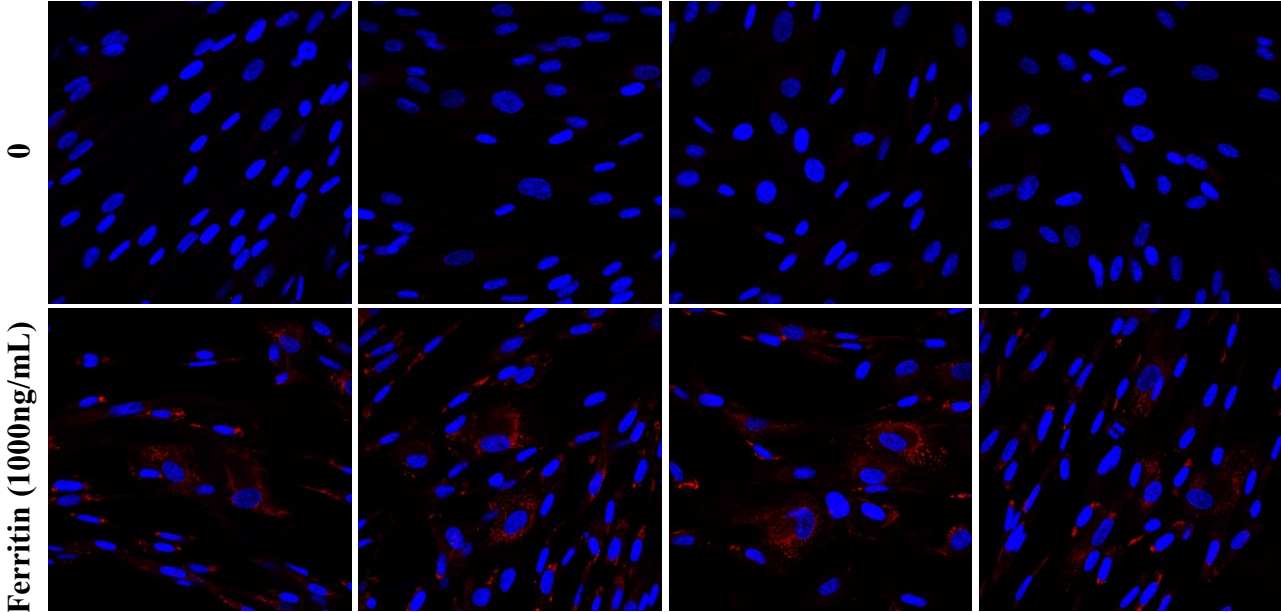

Figure 4L-O

Collagen III

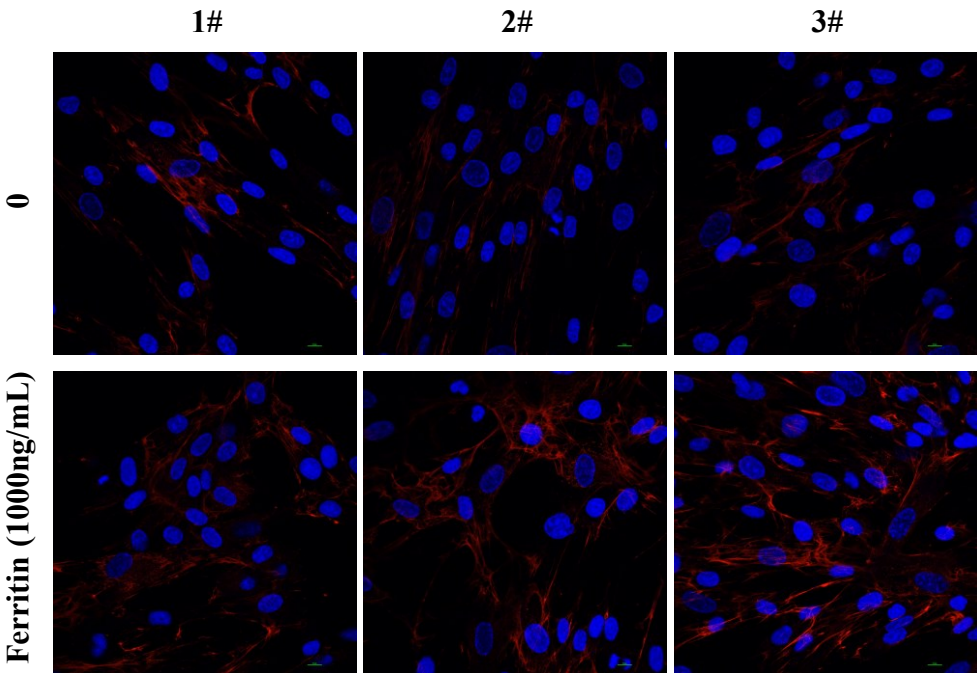

Fibronectin

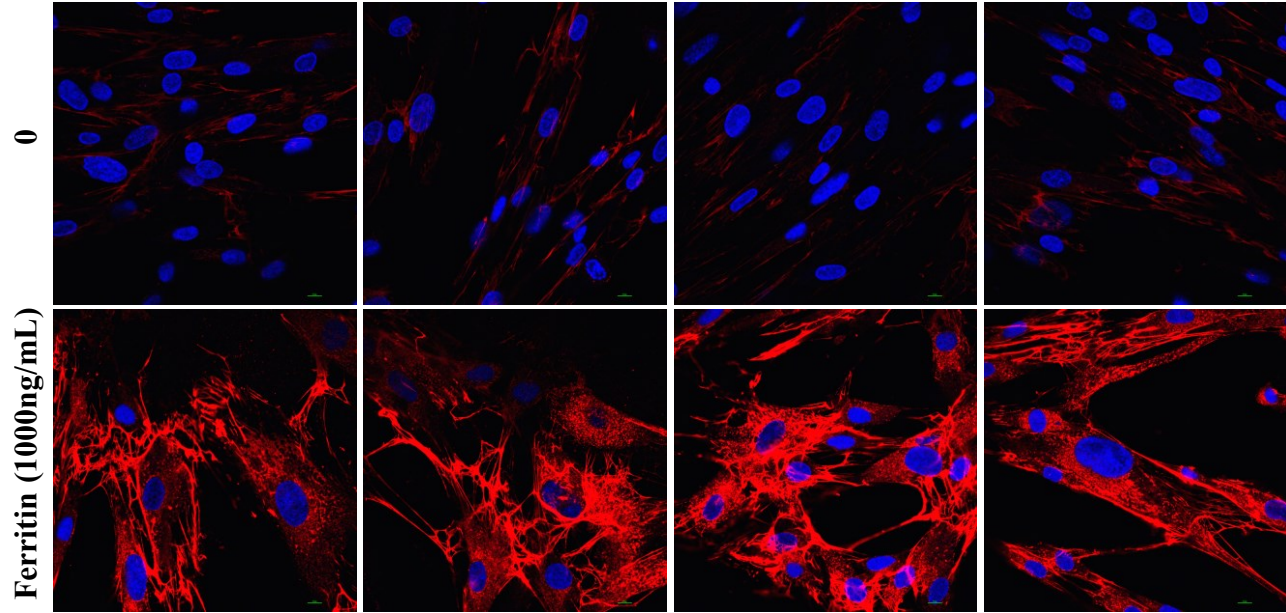

Supplement: Supplementary file 4 — Supporting File 4: advs73867‐sup‐0001‐FiguresData.zip. [file ADVS-13-e19191-s001.zip › Supporting information Figure1-10/Figure 4/Figure 4L-O.pdf]

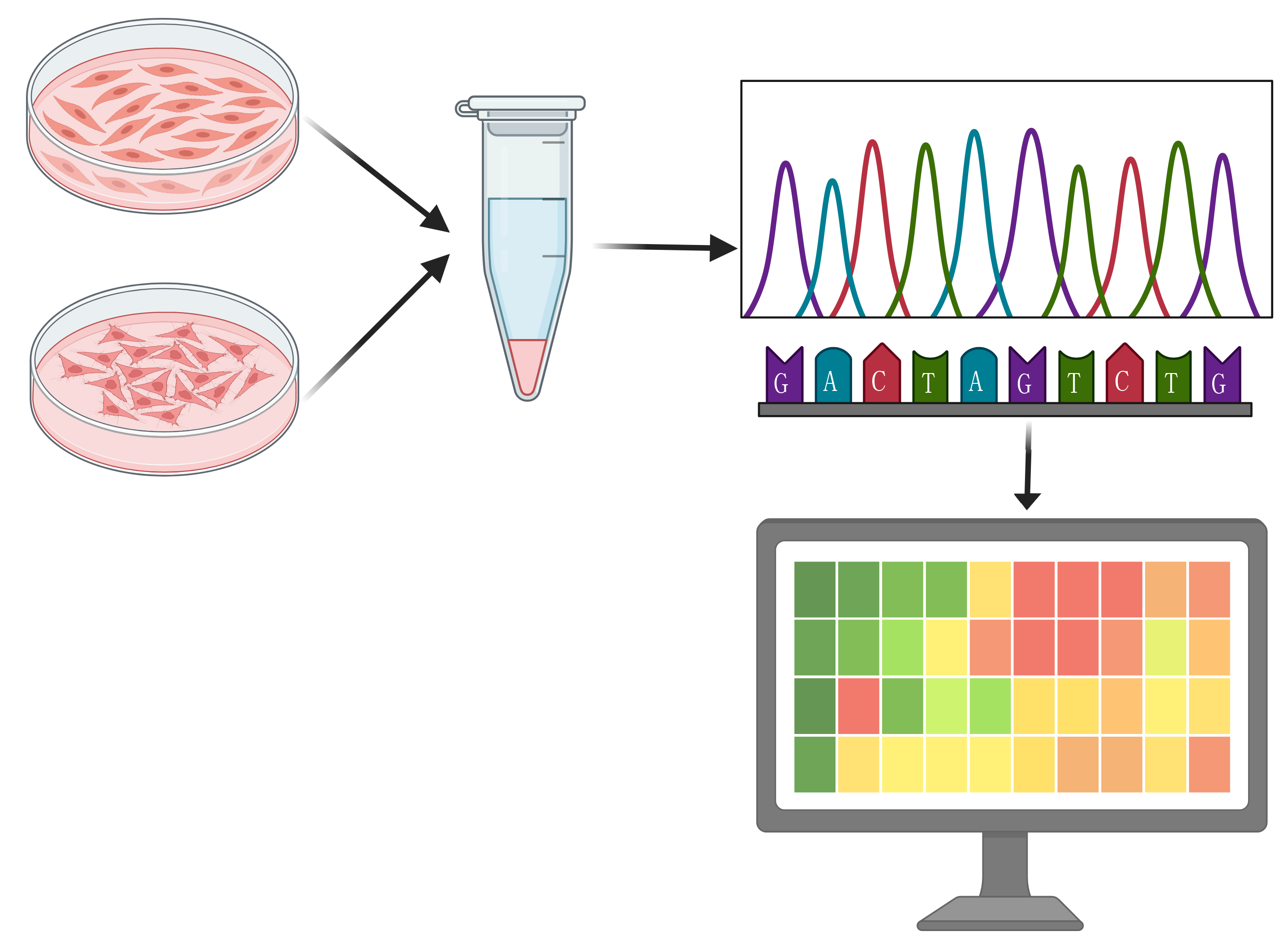

Supplement: Supplementary file 4 — Supporting File 4: advs73867‐sup‐0001‐FiguresData.zip. [file ADVS-13-e19191-s001.zip › Supporting information Figure1-10/Figure 5/Figure 5F.png]

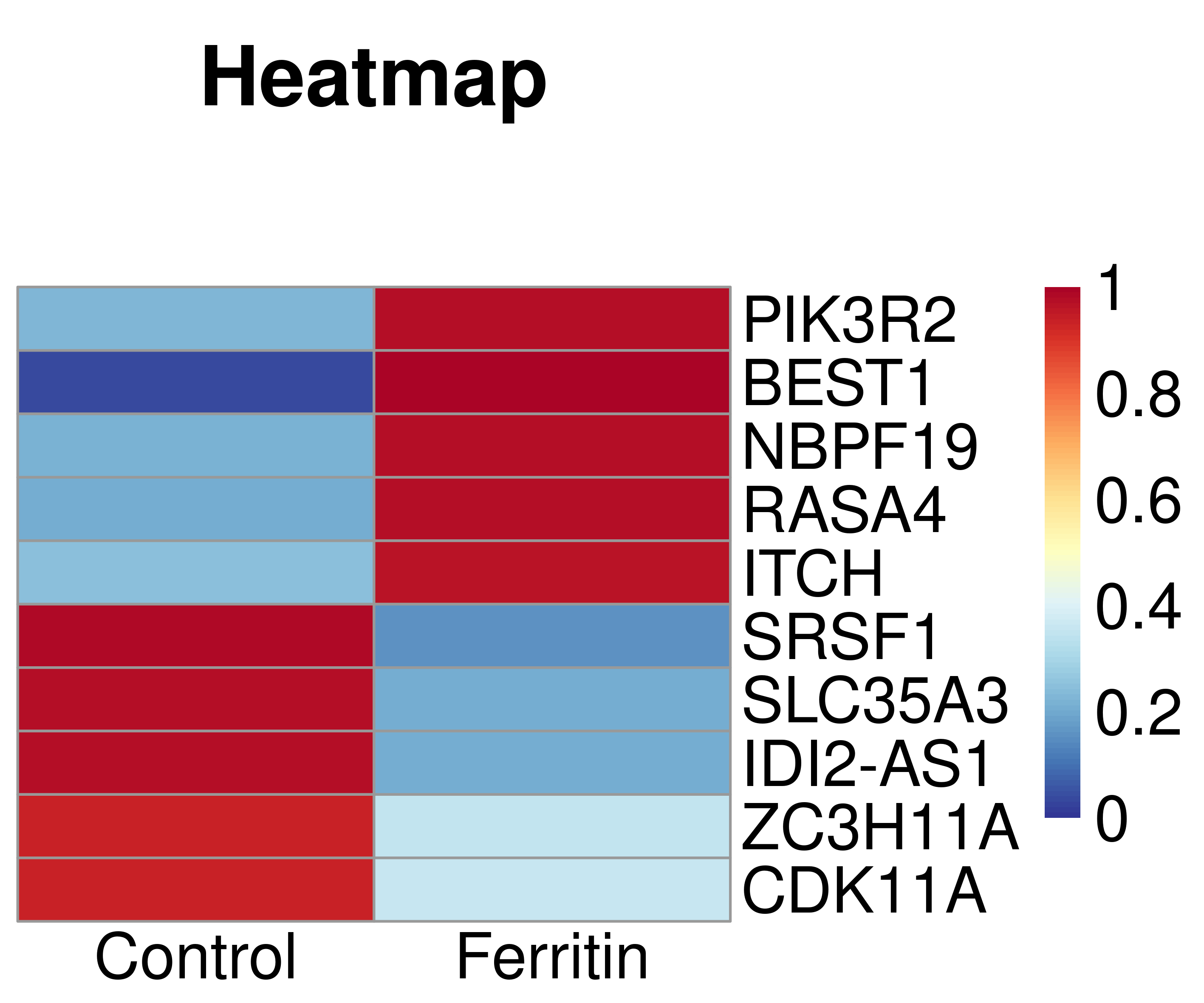

Supplement: Supplementary file 4 — Supporting File 4: advs73867‐sup‐0001‐FiguresData.zip. [file ADVS-13-e19191-s001.zip › Supporting information Figure1-10/Figure 5/Figure 5G/TOP10 DEGs in HPF after ferritin stimulation.png]

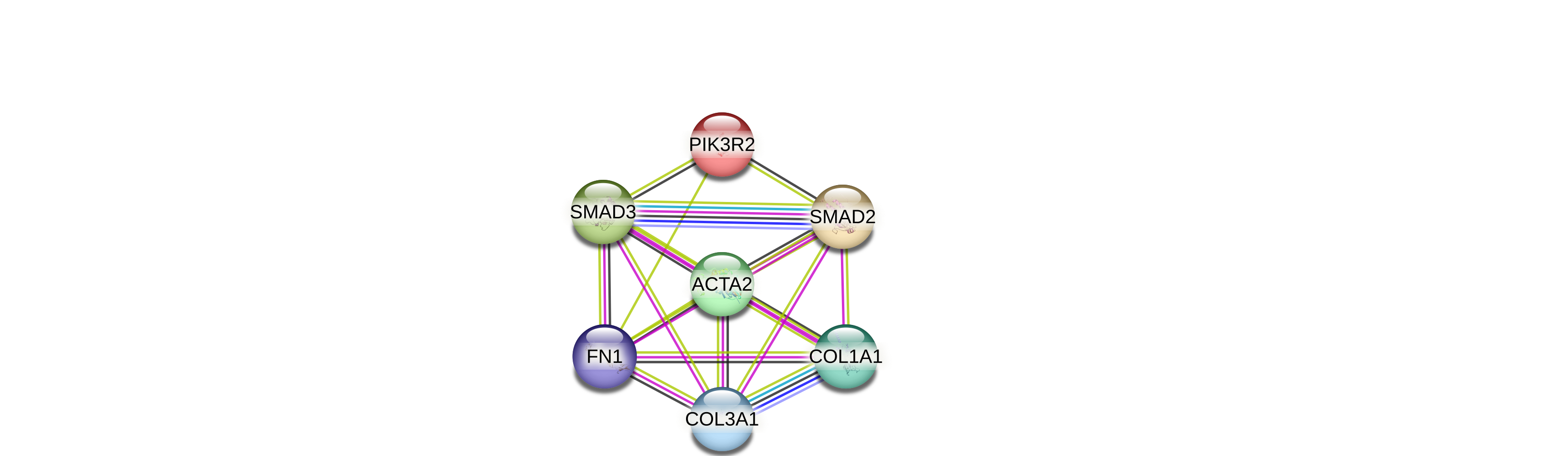

Supplement: Supplementary file 4 — Supporting File 4: advs73867‐sup‐0001‐FiguresData.zip. [file ADVS-13-e19191-s001.zip › Supporting information Figure1-10/Figure 5/Figure 5K.png]

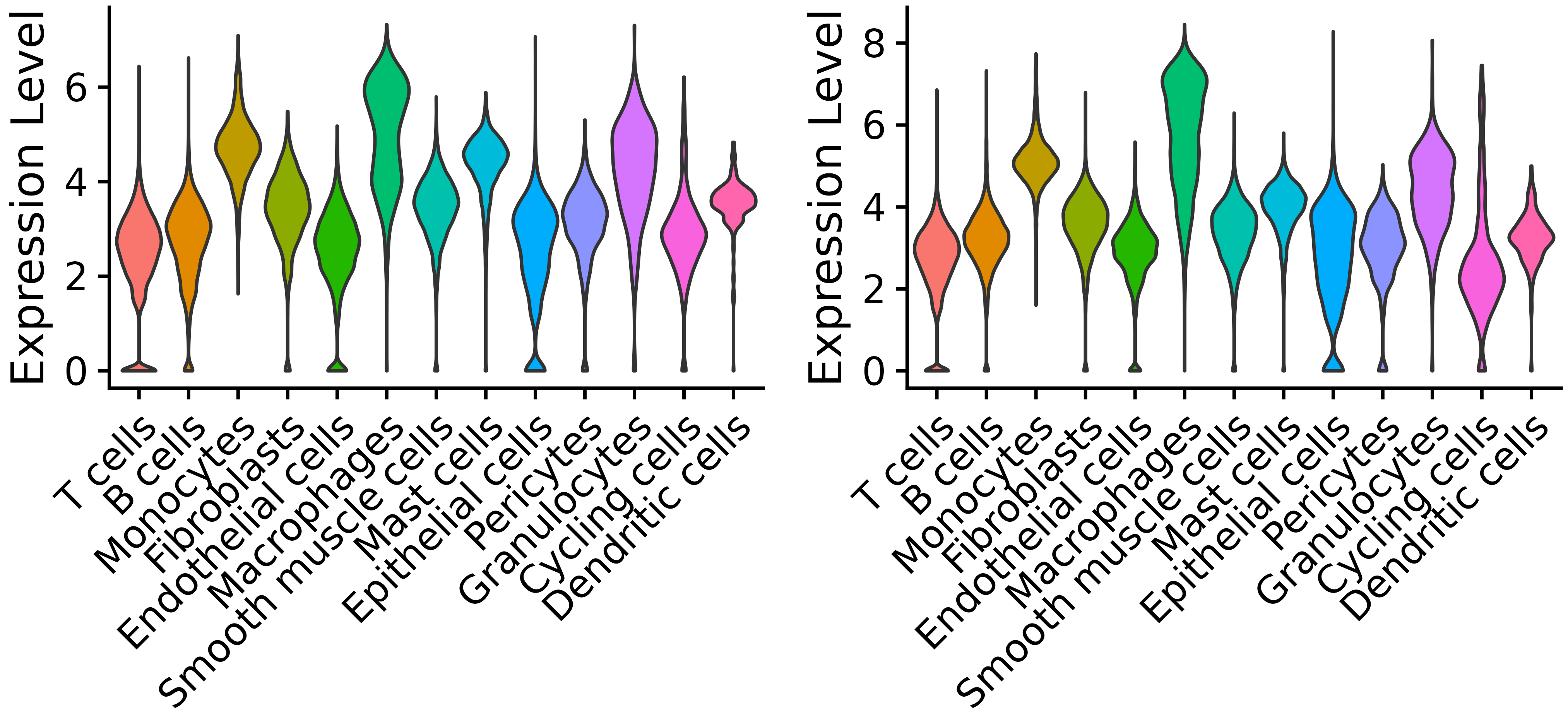

Supplement: Supplementary file 4 — Supporting File 4: advs73867‐sup‐0001‐FiguresData.zip. [file ADVS-13-e19191-s001.zip › Supporting information Figure1-10/Figure 7/Figure 7B.png]

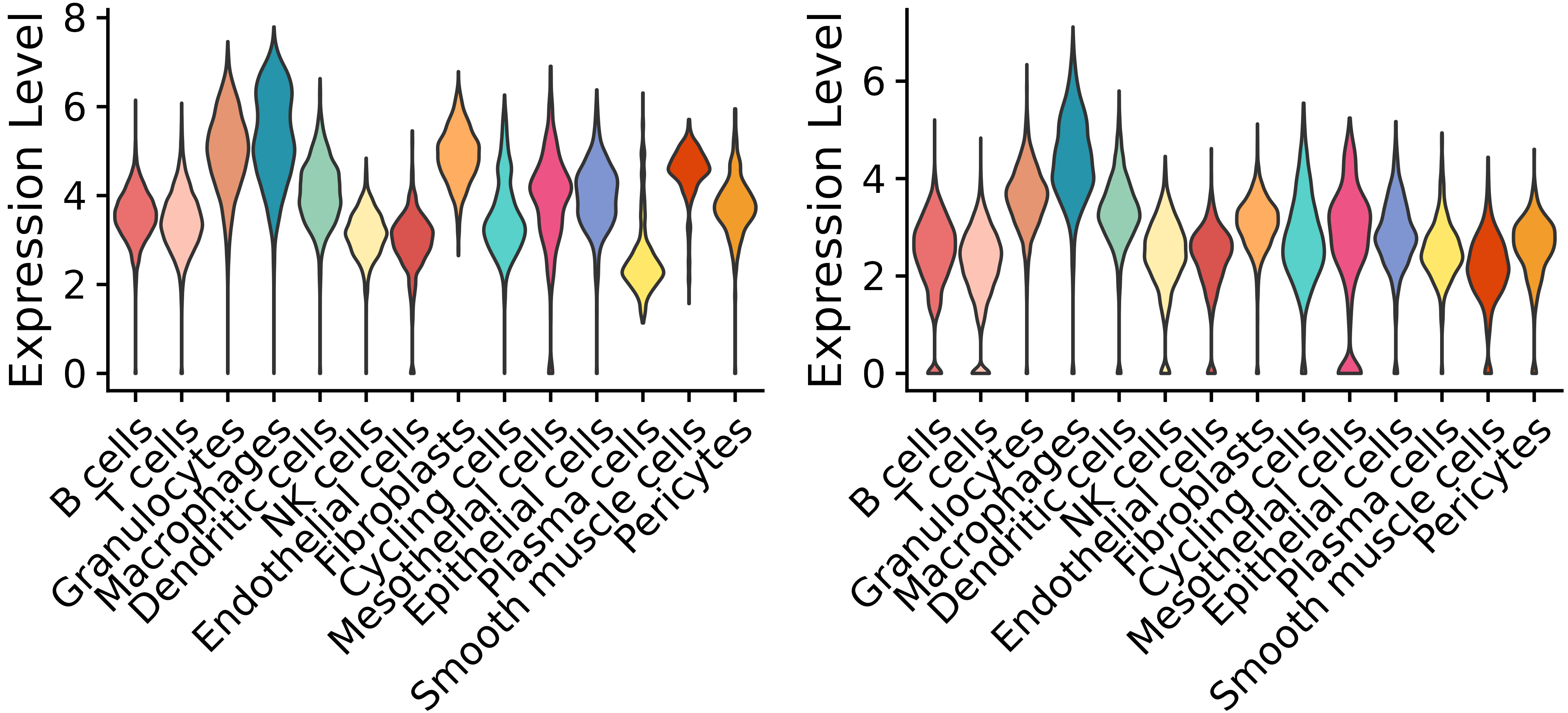

Supplement: Supplementary file 4 — Supporting File 4: advs73867‐sup‐0001‐FiguresData.zip. [file ADVS-13-e19191-s001.zip › Supporting information Figure1-10/Figure 7/Figure 7C.png]

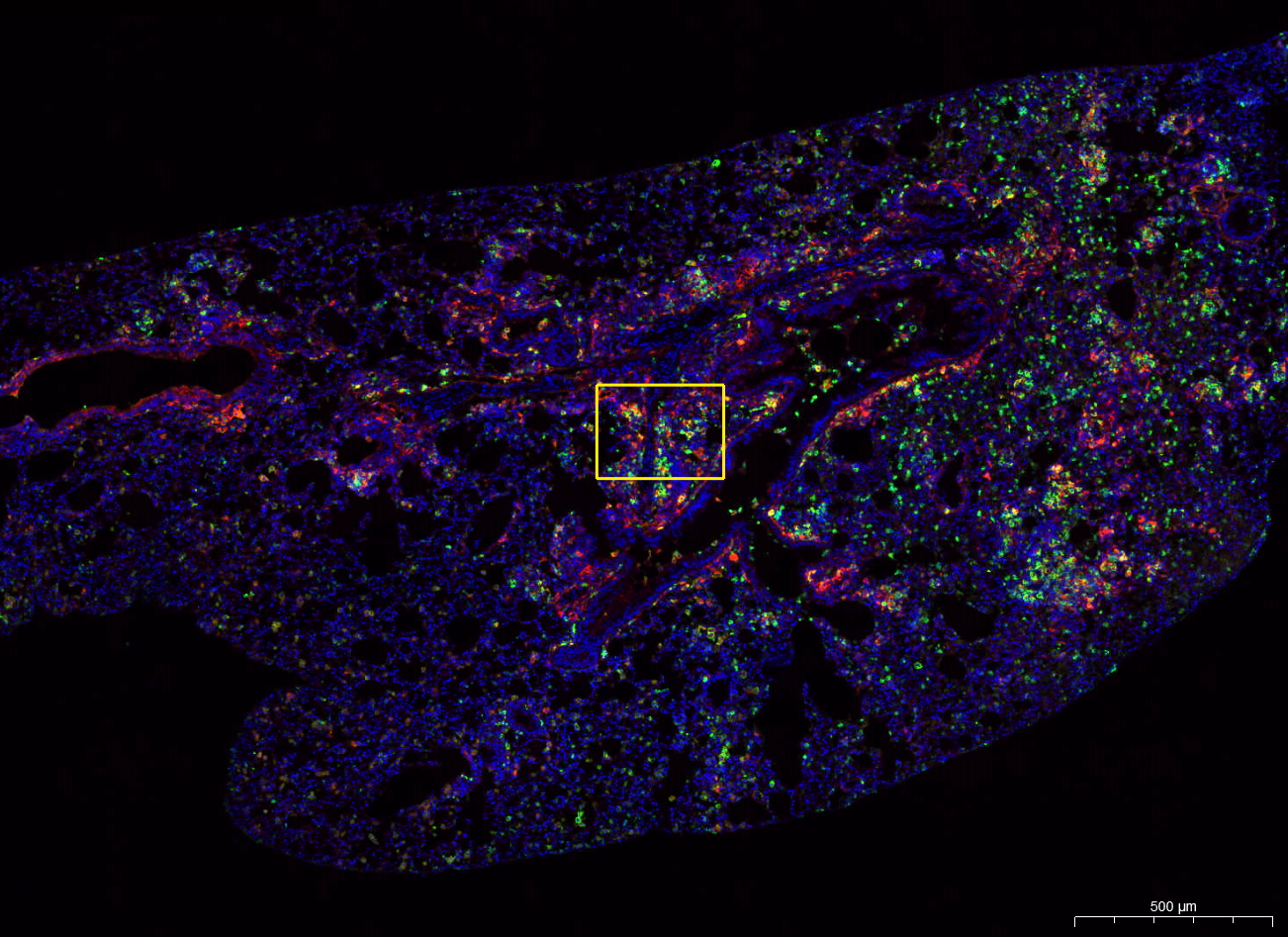

Supplement: Supplementary file 4 — Supporting File 4: advs73867‐sup‐0001‐FiguresData.zip. [file ADVS-13-e19191-s001.zip › Supporting information Figure1-10/Figure 7/Figure 7D/12W Model 751_ IF_0240117144731065_4.0x.jpg]

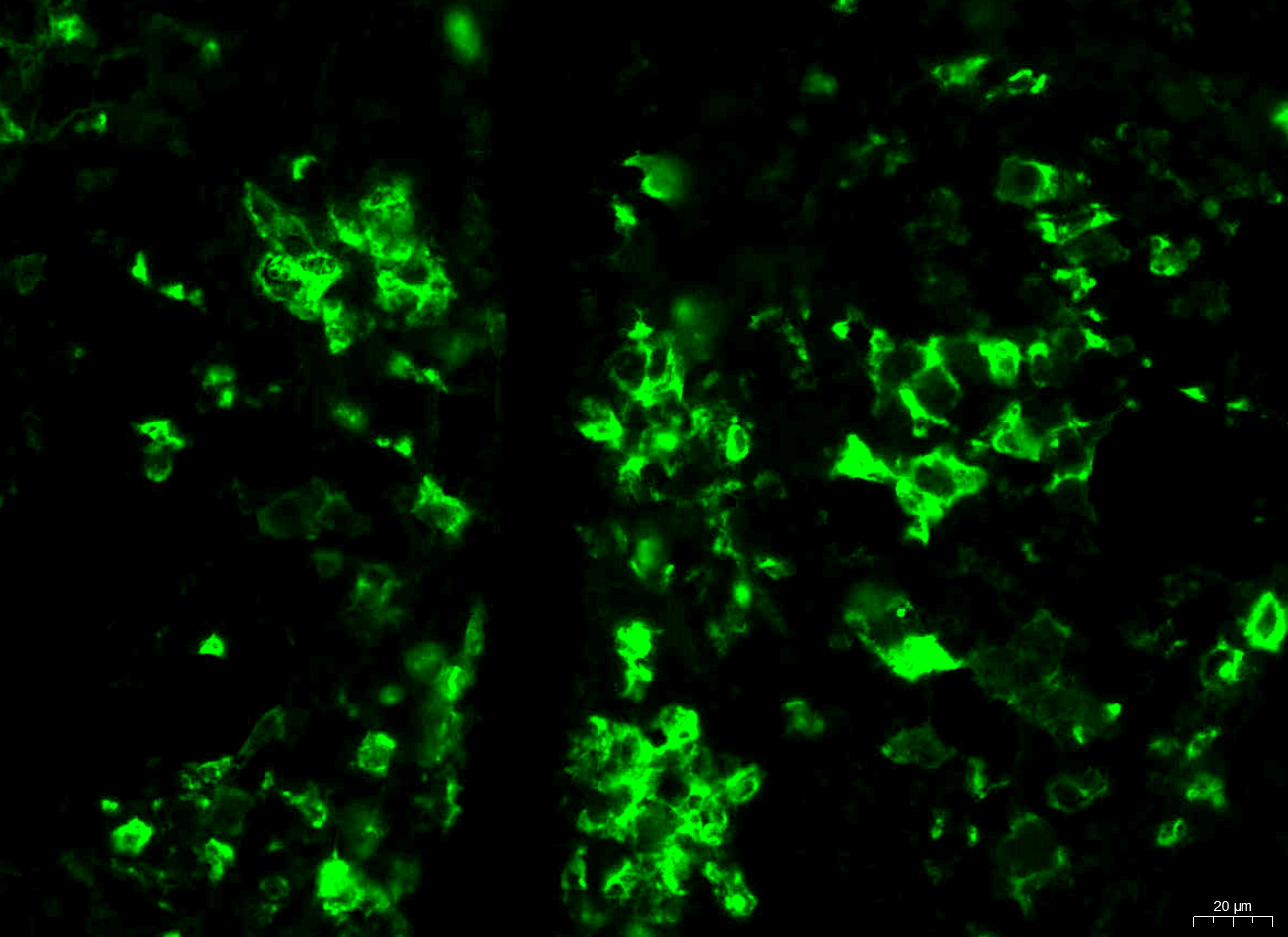

Supplement: Supplementary file 4 — Supporting File 4: advs73867‐sup‐0001‐FiguresData.zip. [file ADVS-13-e19191-s001.zip › Supporting information Figure1-10/Figure 7/Figure 7D/12W Model 751_ IF_0240117144731065_40.0x-F480-.jpg]

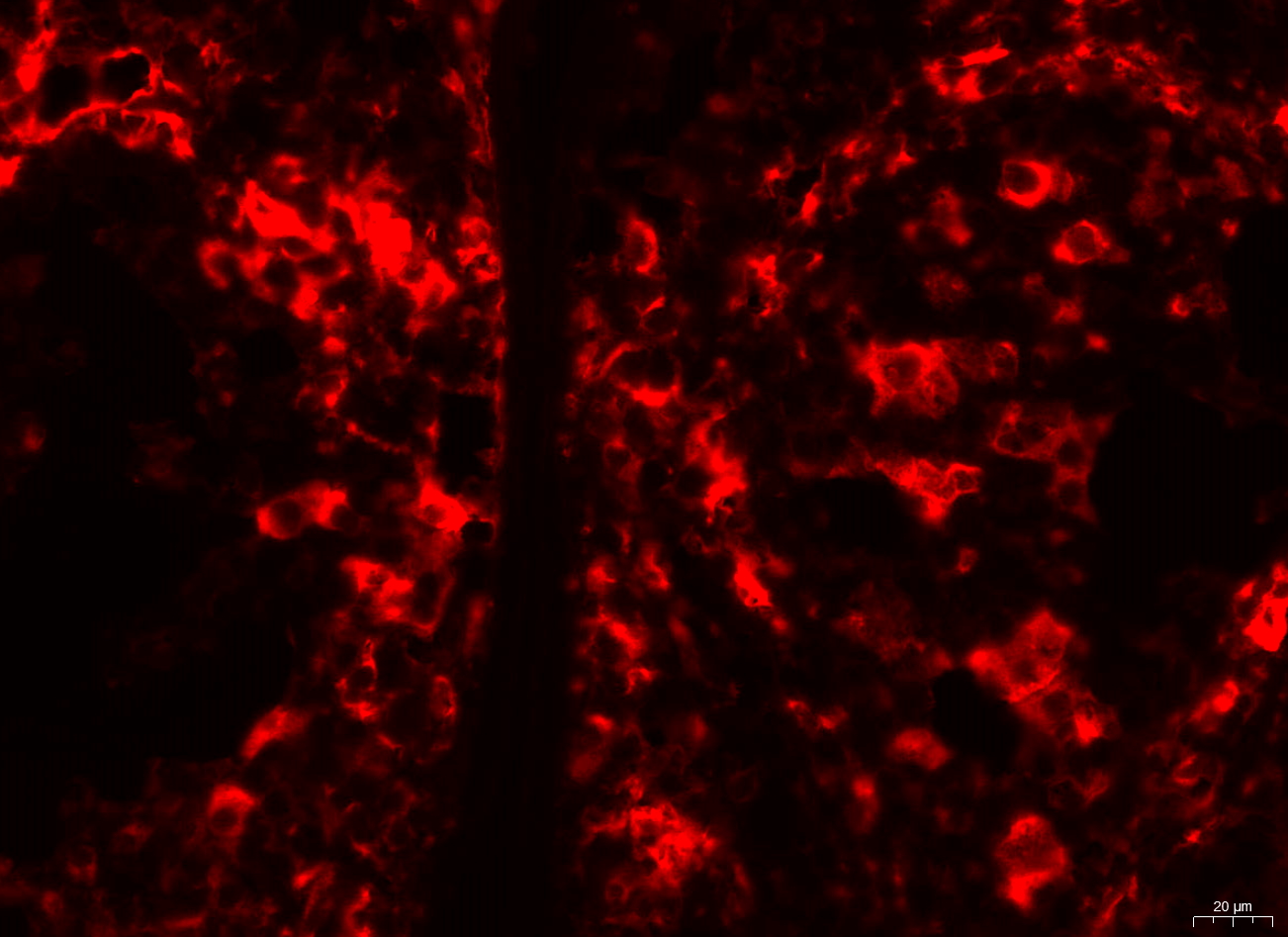

Supplement: Supplementary file 4 — Supporting File 4: advs73867‐sup‐0001‐FiguresData.zip. [file ADVS-13-e19191-s001.zip › Supporting information Figure1-10/Figure 7/Figure 7D/12W Model 751_ IF_20240117144731065_40.0x-Ferritin-.jpg]

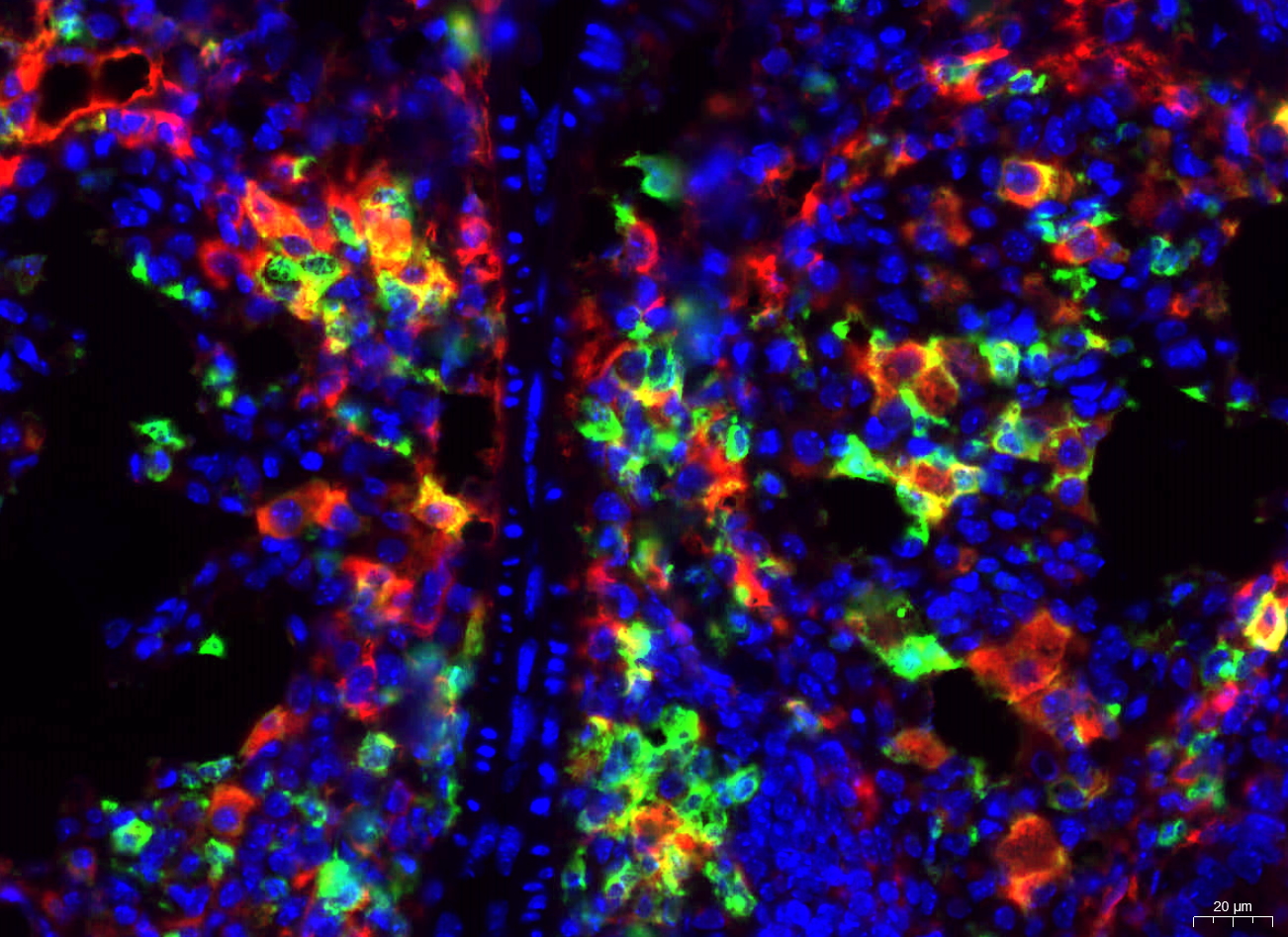

Supplement: Supplementary file 4 — Supporting File 4: advs73867‐sup‐0001‐FiguresData.zip. [file ADVS-13-e19191-s001.zip › Supporting information Figure1-10/Figure 7/Figure 7D/12W Model 751_ IF_20240117144731065_40.0x-Merge-.jpg]

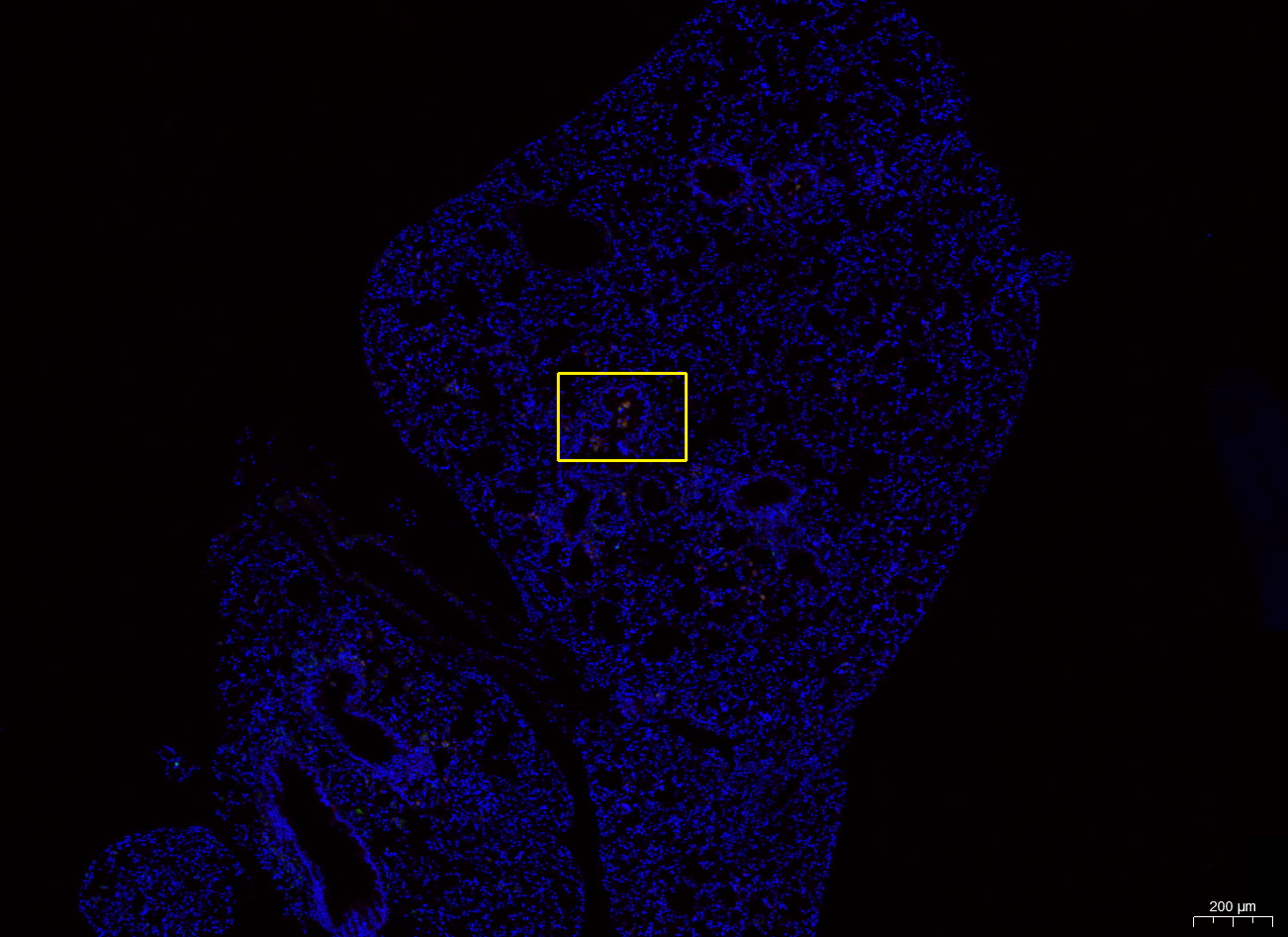

Supplement: Supplementary file 4 — Supporting File 4: advs73867‐sup‐0001‐FiguresData.zip. [file ADVS-13-e19191-s001.zip › Supporting information Figure1-10/Figure 7/Figure 7D/1week Model 762_ IF_20240117145516852_4.0x.jpg]

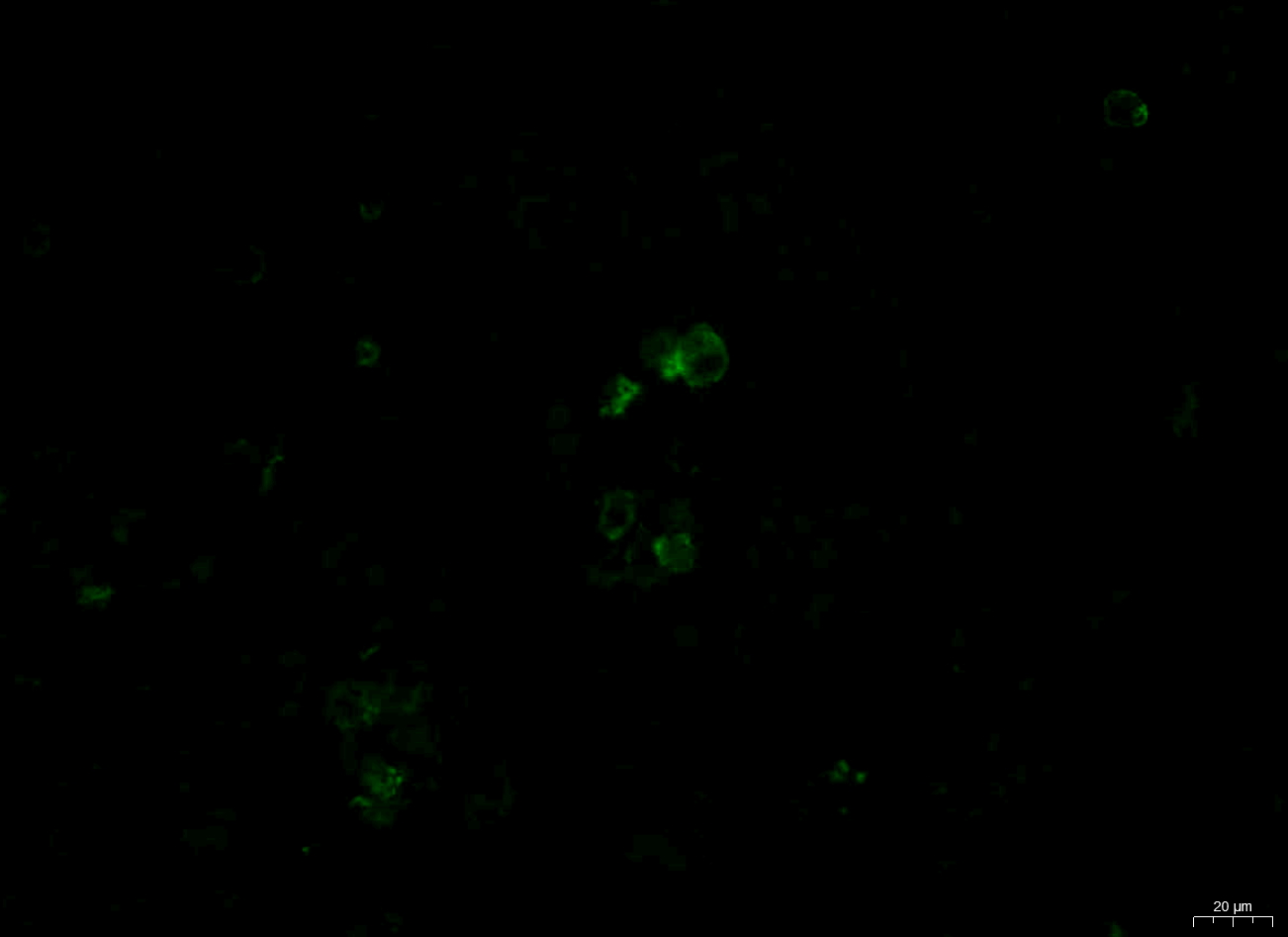

Supplement: Supplementary file 4 — Supporting File 4: advs73867‐sup‐0001‐FiguresData.zip. [file ADVS-13-e19191-s001.zip › Supporting information Figure1-10/Figure 7/Figure 7D/1week Model 762_ IF_20240117145516852_40.0x-F480-.jpg]

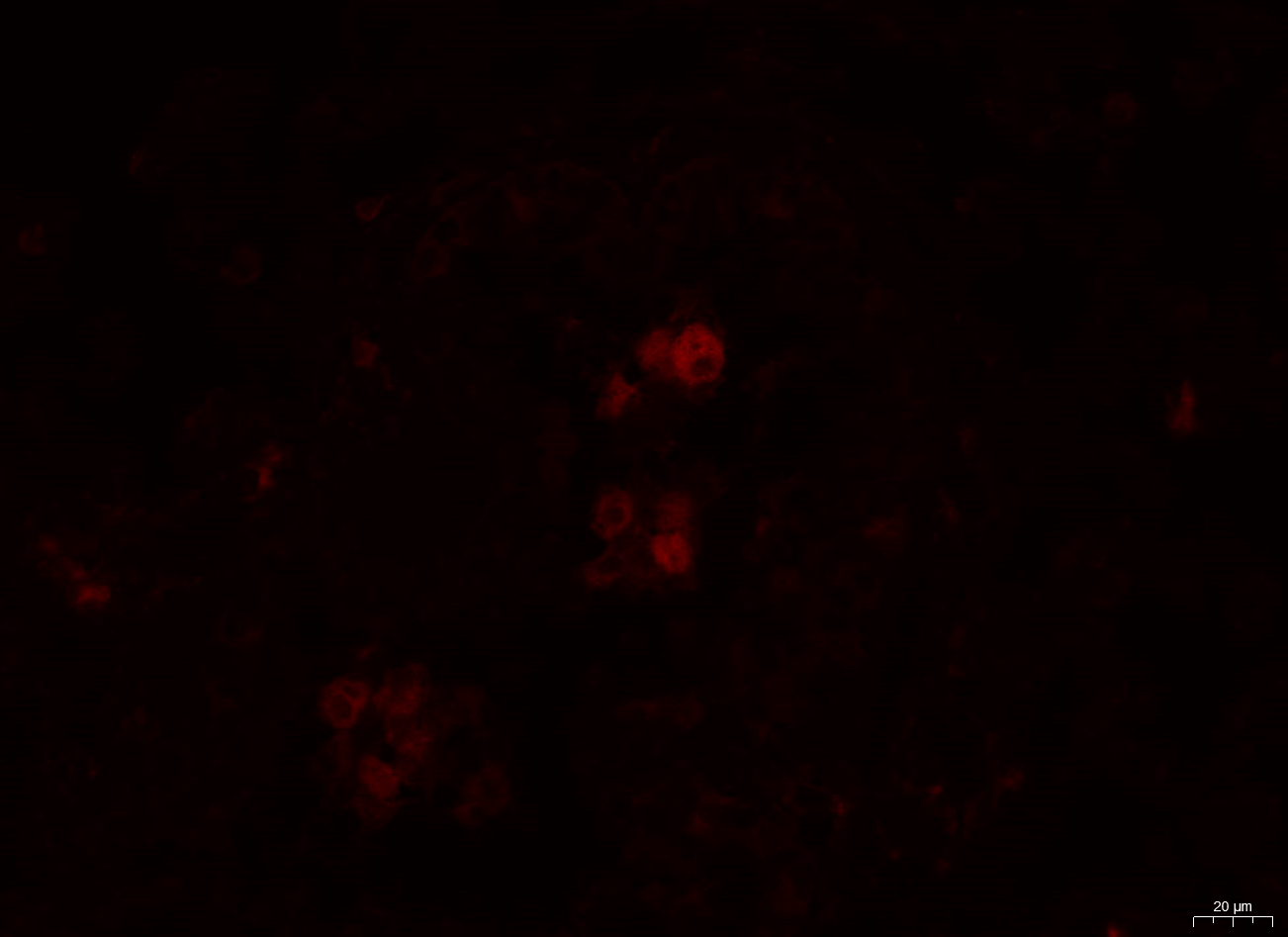

Supplement: Supplementary file 4 — Supporting File 4: advs73867‐sup‐0001‐FiguresData.zip. [file ADVS-13-e19191-s001.zip › Supporting information Figure1-10/Figure 7/Figure 7D/1week Model 762_ IF_20240117145516852_40.0x-Ferritin-.jpg]

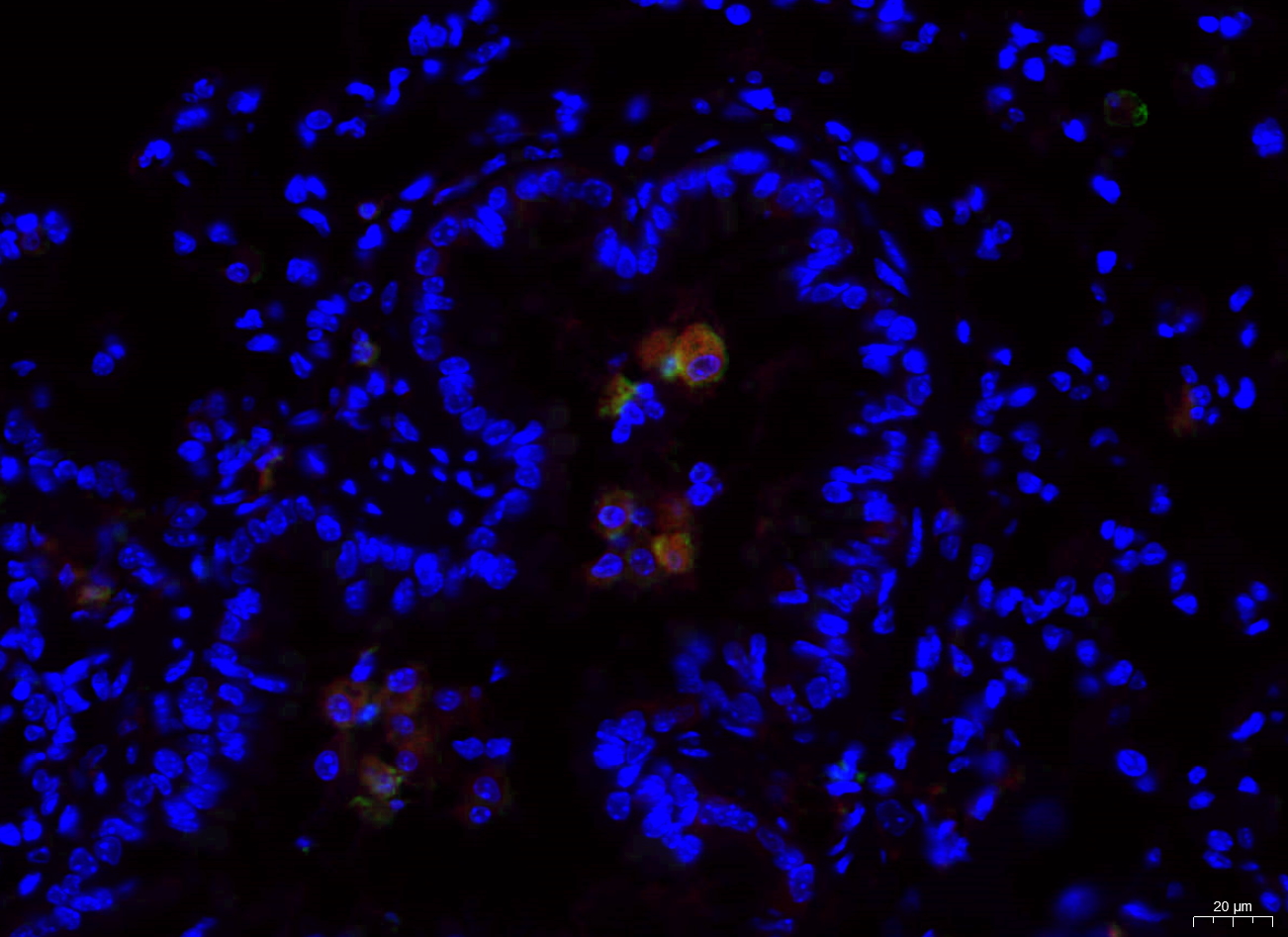

Supplement: Supplementary file 4 — Supporting File 4: advs73867‐sup‐0001‐FiguresData.zip. [file ADVS-13-e19191-s001.zip › Supporting information Figure1-10/Figure 7/Figure 7D/1week Model 762_ IF_20240117145516852_40.0x-Merge-.jpg]

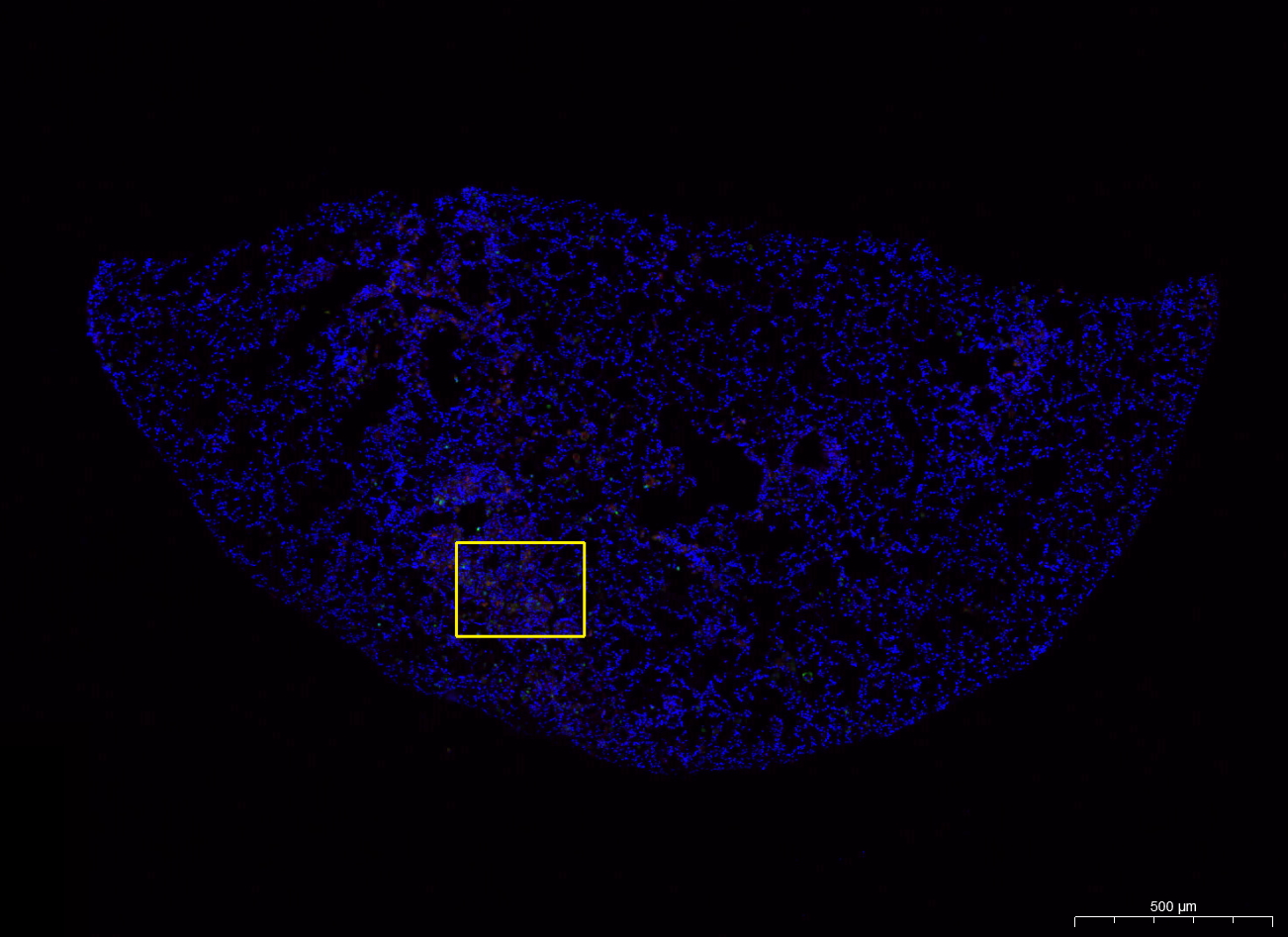

Supplement: Supplementary file 4 — Supporting File 4: advs73867‐sup‐0001‐FiguresData.zip. [file ADVS-13-e19191-s001.zip › Supporting information Figure1-10/Figure 7/Figure 7D/4W Model 756_ IF_20240117145200180_4.0x.jpg]

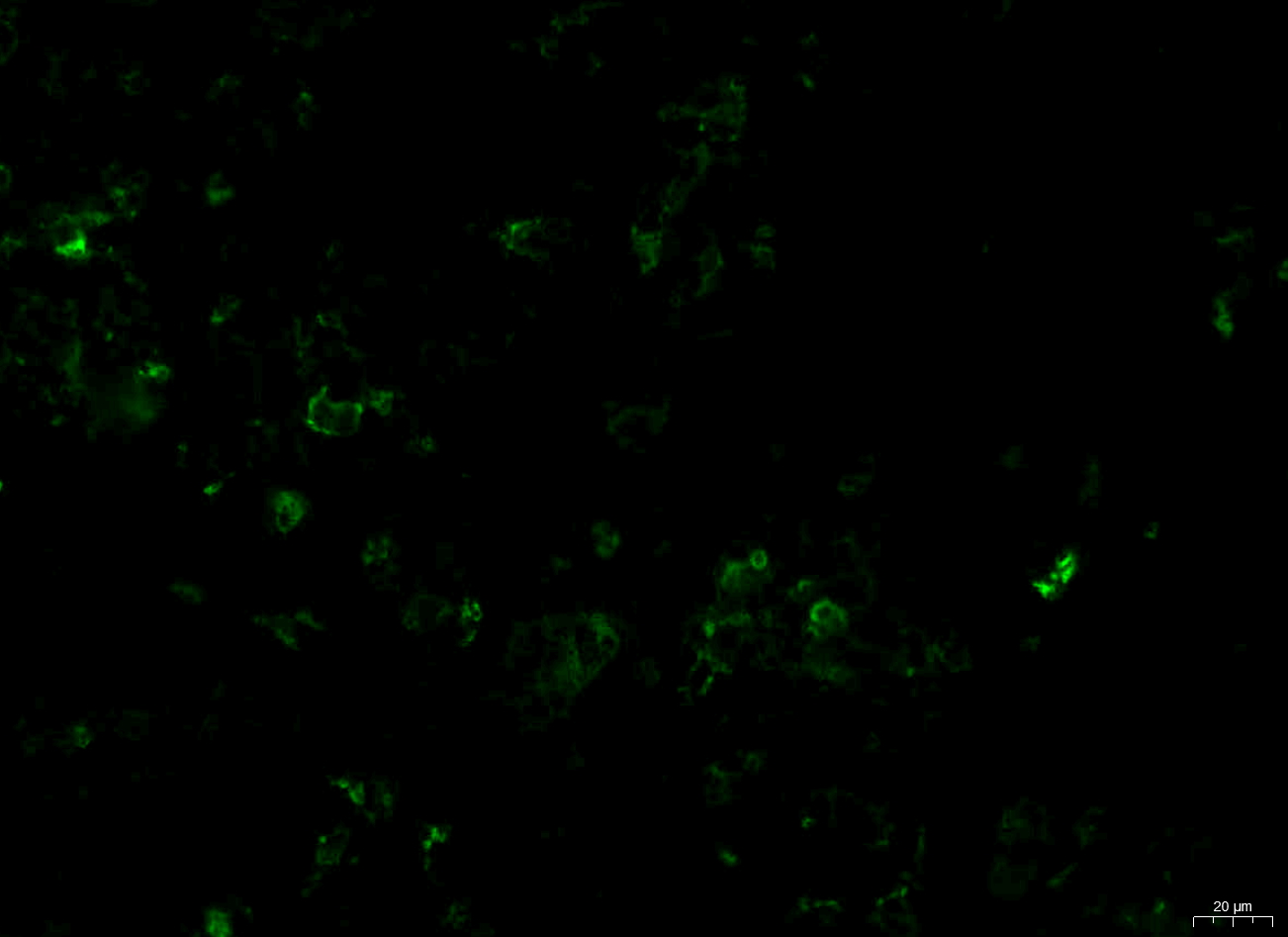

Supplement: Supplementary file 4 — Supporting File 4: advs73867‐sup‐0001‐FiguresData.zip. [file ADVS-13-e19191-s001.zip › Supporting information Figure1-10/Figure 7/Figure 7D/4W Model 756_ IF_20240117145200180_40.0x-F480-.jpg]

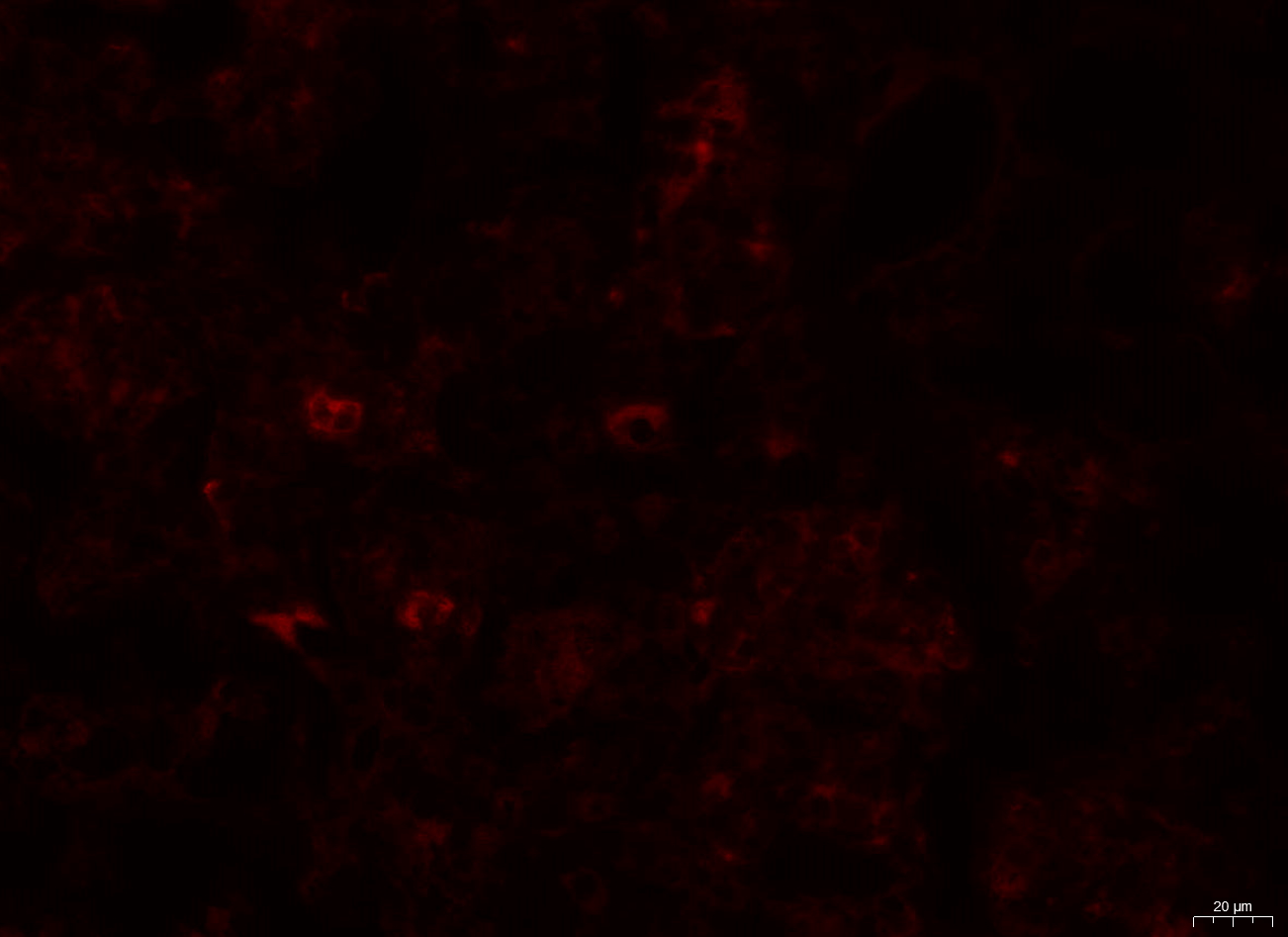

Supplement: Supplementary file 4 — Supporting File 4: advs73867‐sup‐0001‐FiguresData.zip. [file ADVS-13-e19191-s001.zip › Supporting information Figure1-10/Figure 7/Figure 7D/4W Model 756_ IF_20240117145200180_40.0x-Ferritin-.jpg]

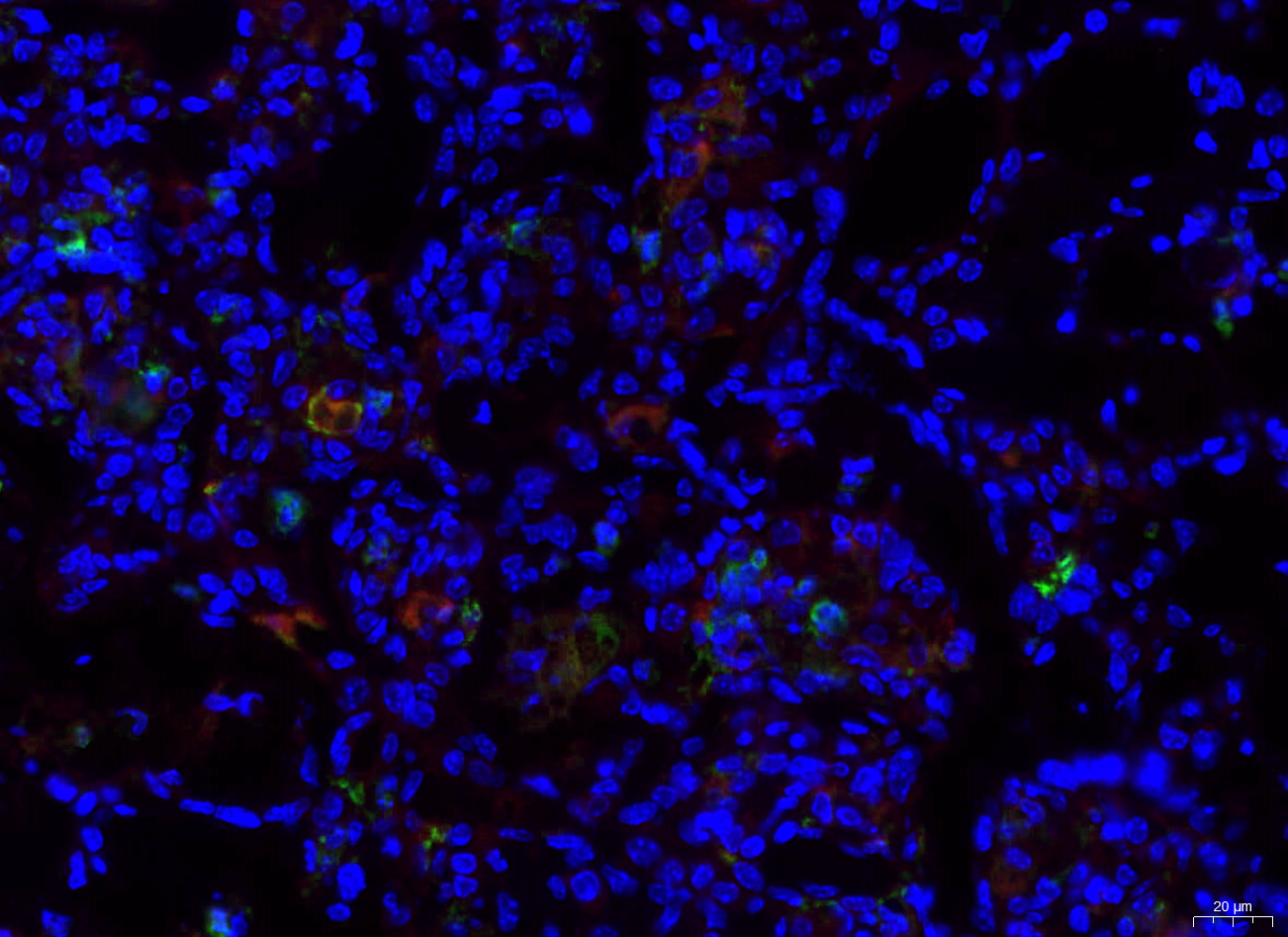

Supplement: Supplementary file 4 — Supporting File 4: advs73867‐sup‐0001‐FiguresData.zip. [file ADVS-13-e19191-s001.zip › Supporting information Figure1-10/Figure 7/Figure 7D/4W Model 756_ IF_20240117145200180_40.0x-Merge-.jpg]

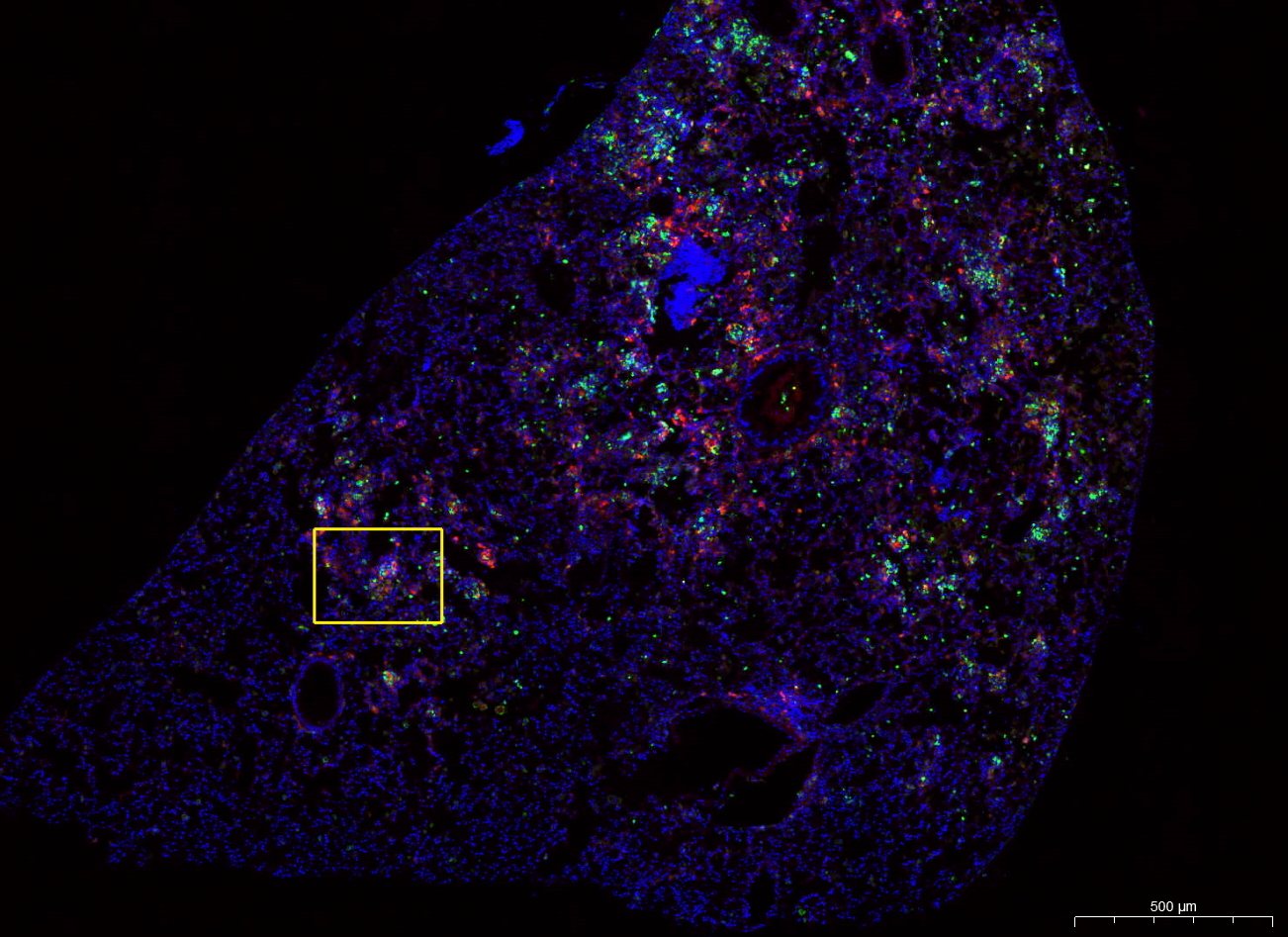

Supplement: Supplementary file 4 — Supporting File 4: advs73867‐sup‐0001‐FiguresData.zip. [file ADVS-13-e19191-s001.zip › Supporting information Figure1-10/Figure 7/Figure 7D/8W Model 766_ IF_20240117145825200_4.0x.jpg]

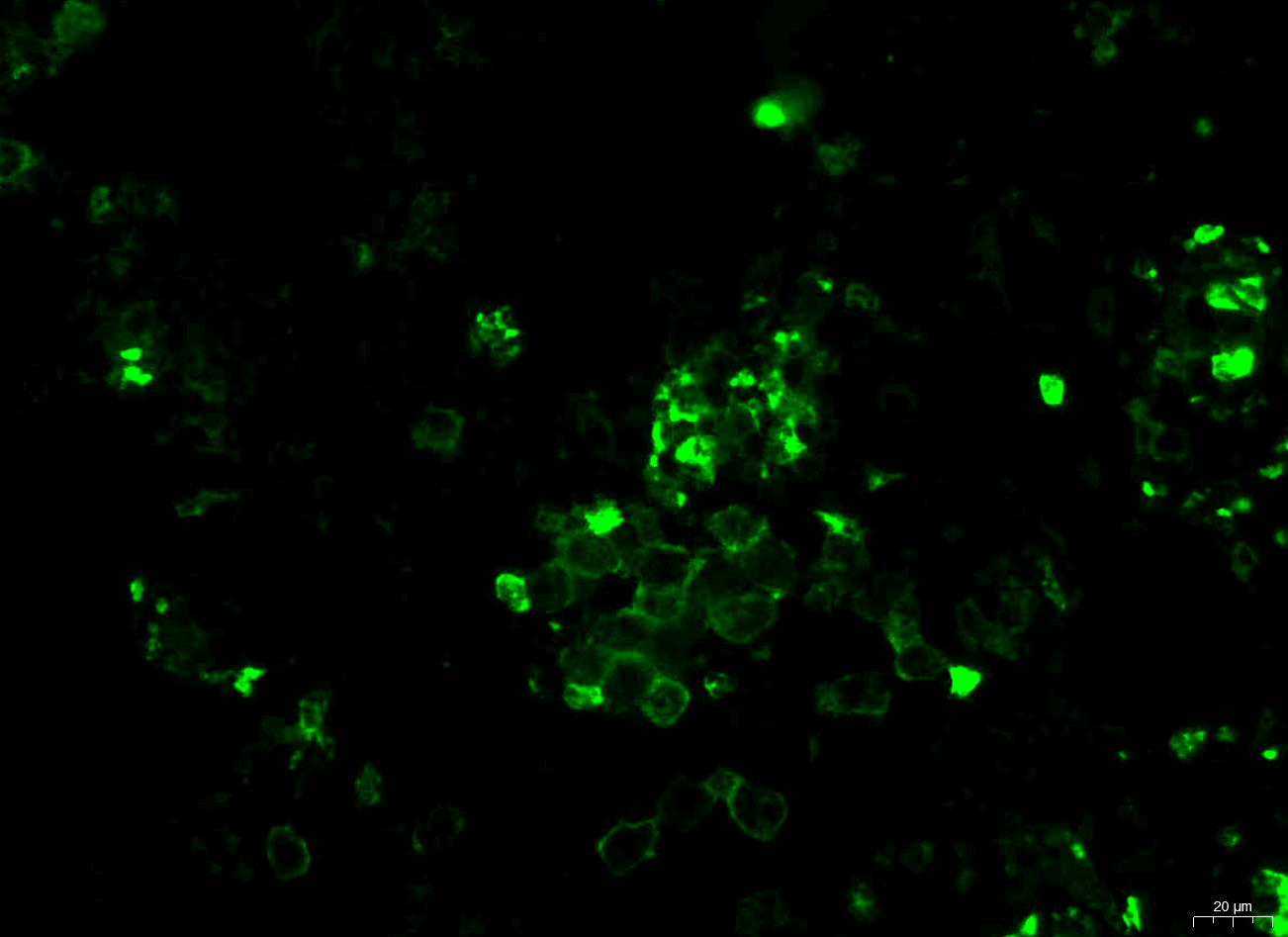

Supplement: Supplementary file 4 — Supporting File 4: advs73867‐sup‐0001‐FiguresData.zip. [file ADVS-13-e19191-s001.zip › Supporting information Figure1-10/Figure 7/Figure 7D/8W Model 766_ IF_20240117145825200_40.0x-F480-.jpg]

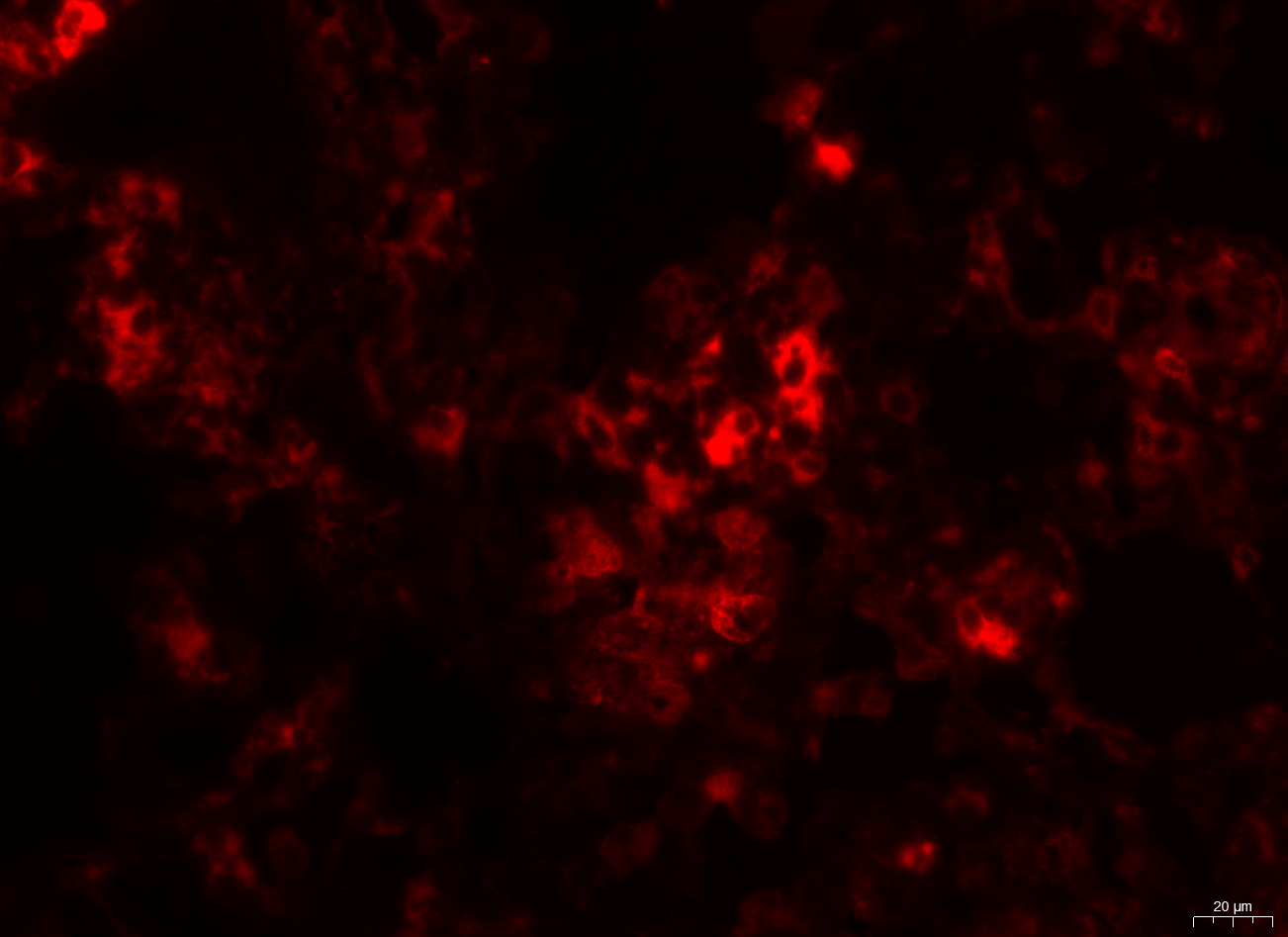

Supplement: Supplementary file 4 — Supporting File 4: advs73867‐sup‐0001‐FiguresData.zip. [file ADVS-13-e19191-s001.zip › Supporting information Figure1-10/Figure 7/Figure 7D/8W Model 766_ IF_20240117145825200_40.0x-Ferritin-.jpg]

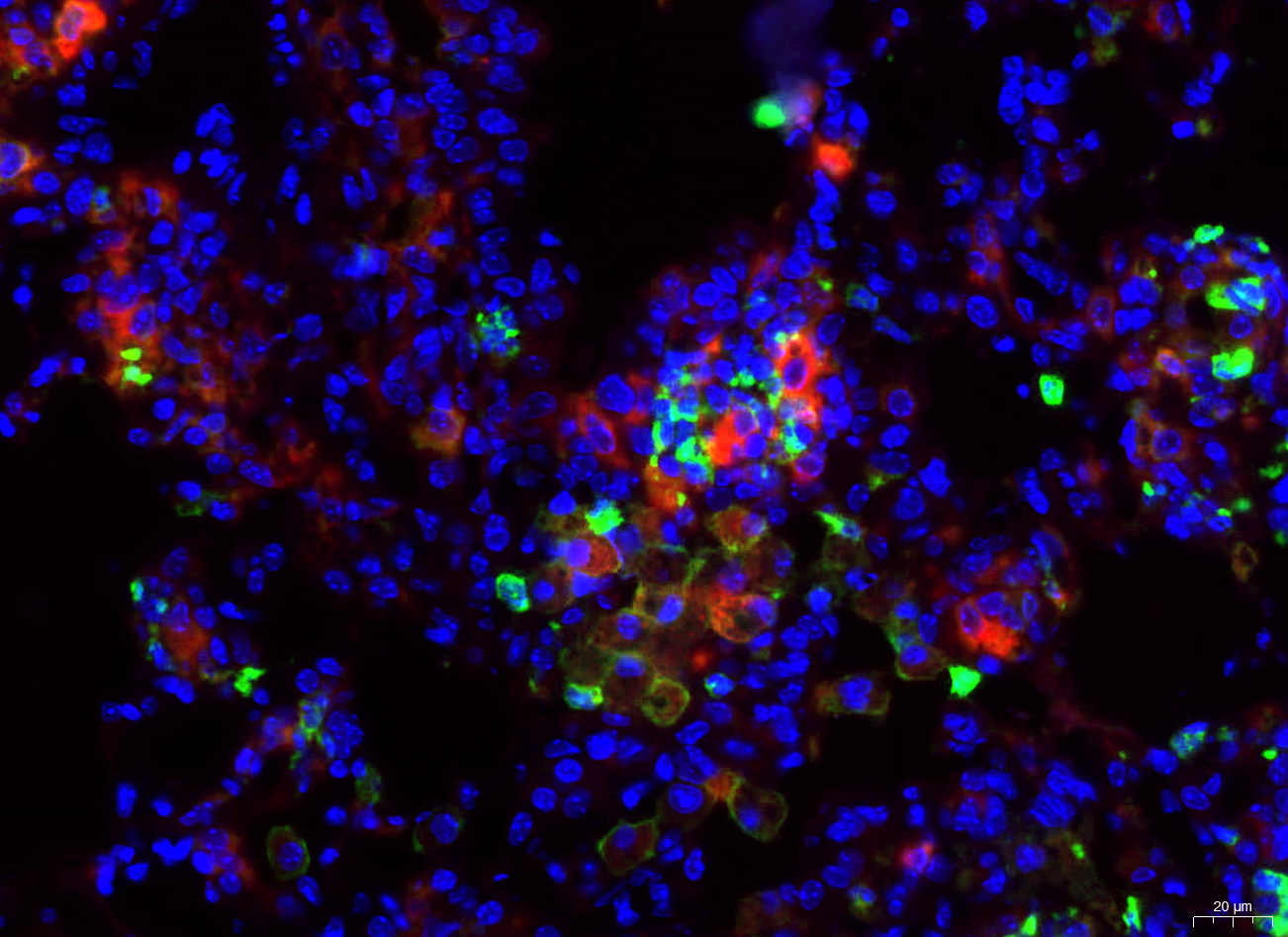

Supplement: Supplementary file 4 — Supporting File 4: advs73867‐sup‐0001‐FiguresData.zip. [file ADVS-13-e19191-s001.zip › Supporting information Figure1-10/Figure 7/Figure 7D/8W Model 766_ IF_20240117145825200_40.0x-Merge-.jpg]

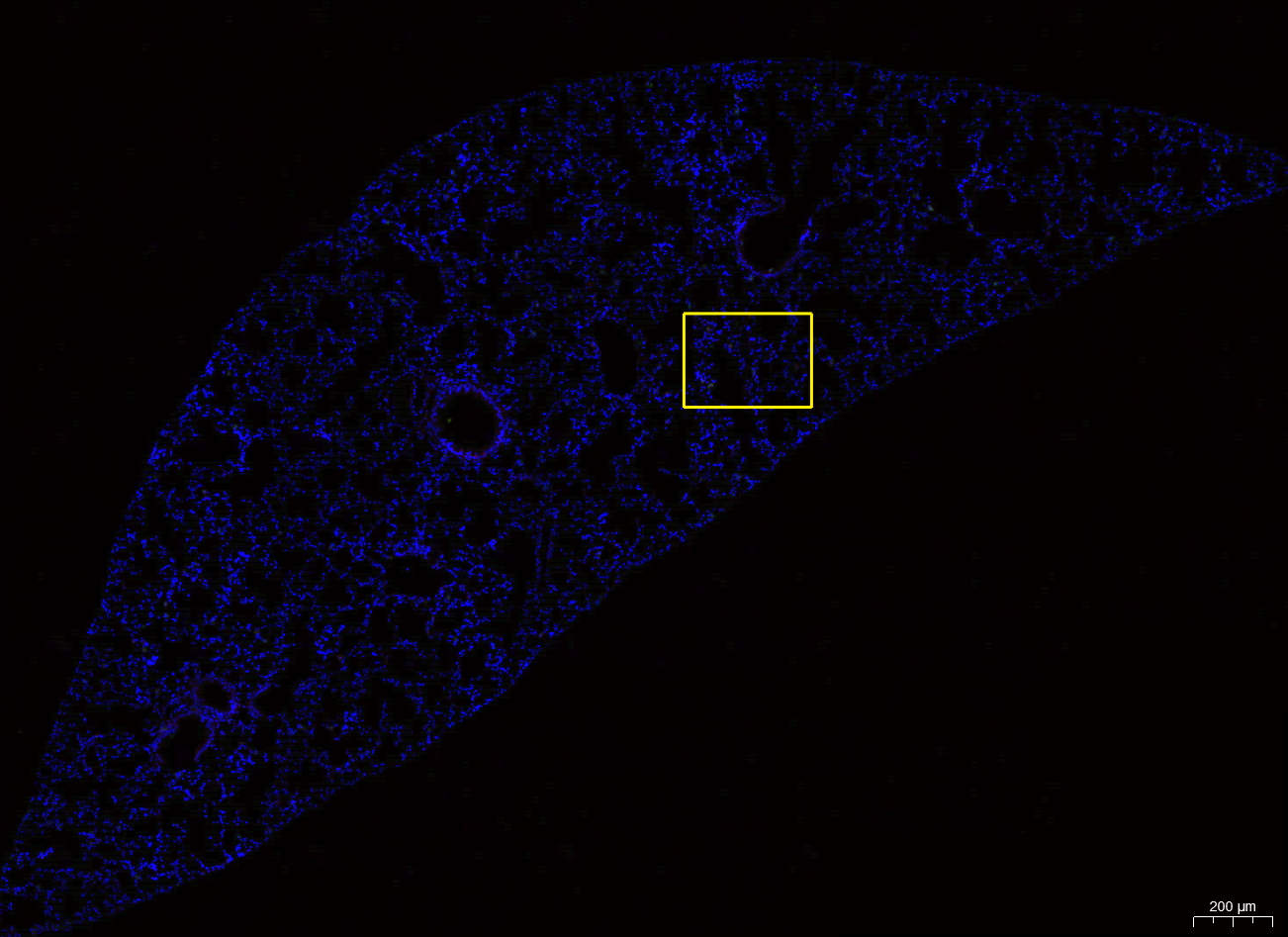

Supplement: Supplementary file 4 — Supporting File 4: advs73867‐sup‐0001‐FiguresData.zip. [file ADVS-13-e19191-s001.zip › Supporting information Figure1-10/Figure 7/Figure 7D/Control 774_ IF_20240117150104370_4.0x.jpg]

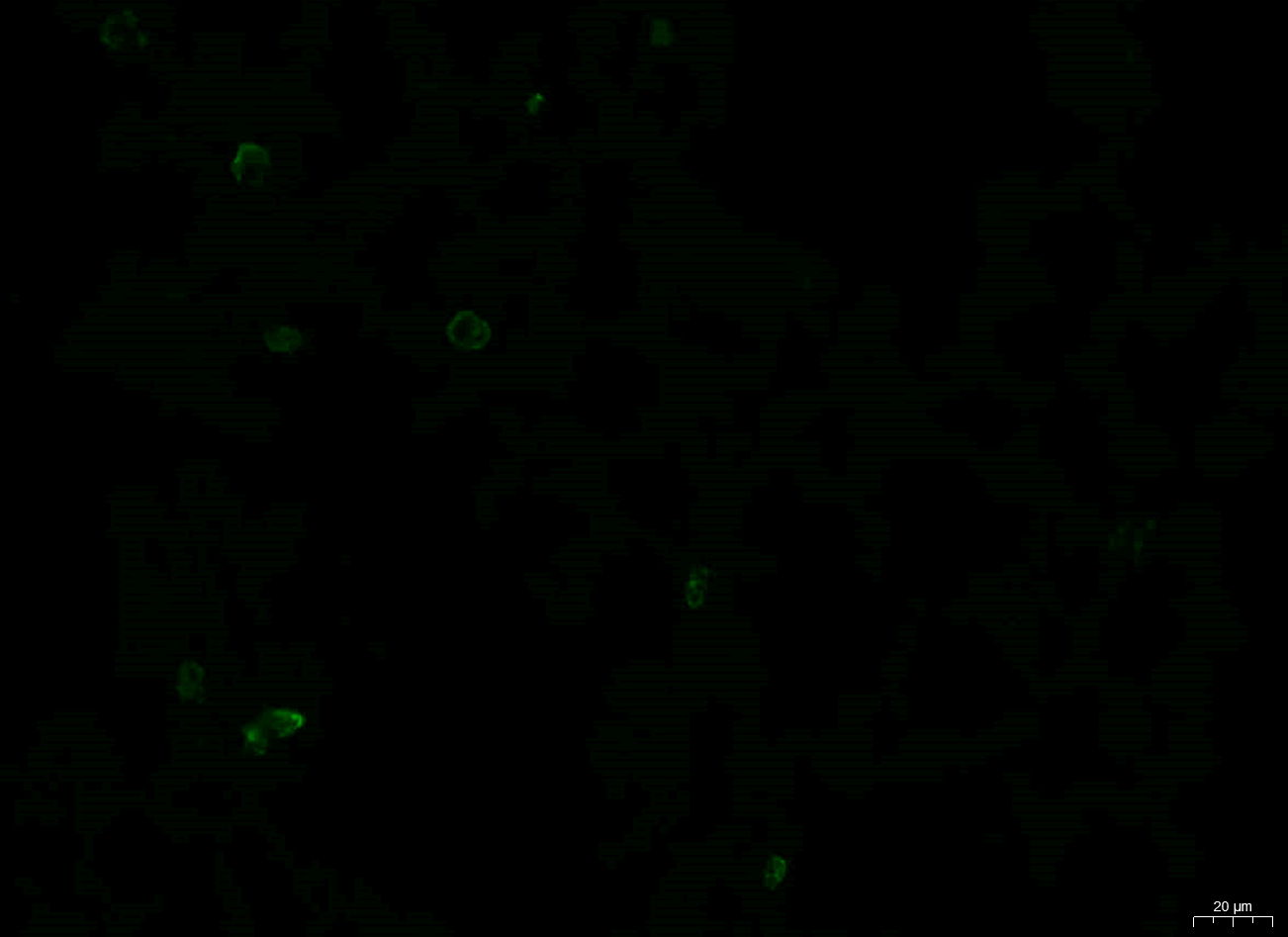

Supplement: Supplementary file 4 — Supporting File 4: advs73867‐sup‐0001‐FiguresData.zip. [file ADVS-13-e19191-s001.zip › Supporting information Figure1-10/Figure 7/Figure 7D/Control 774_ IF_20240117150104370_40.0x-F480-.jpg]

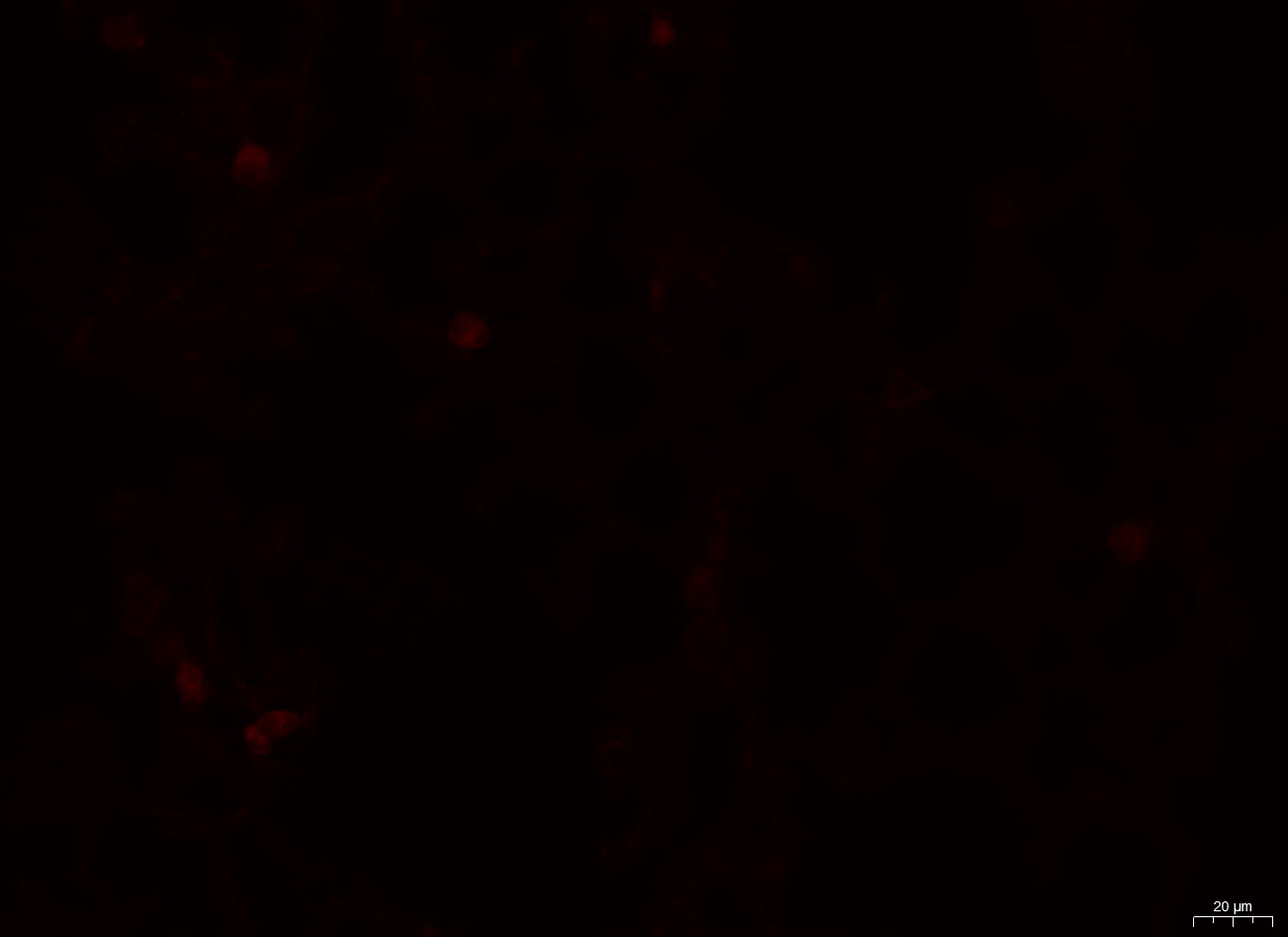

Supplement: Supplementary file 4 — Supporting File 4: advs73867‐sup‐0001‐FiguresData.zip. [file ADVS-13-e19191-s001.zip › Supporting information Figure1-10/Figure 7/Figure 7D/Control 774_ IF_20240117150104370_40.0x-Ferritin-.jpg]

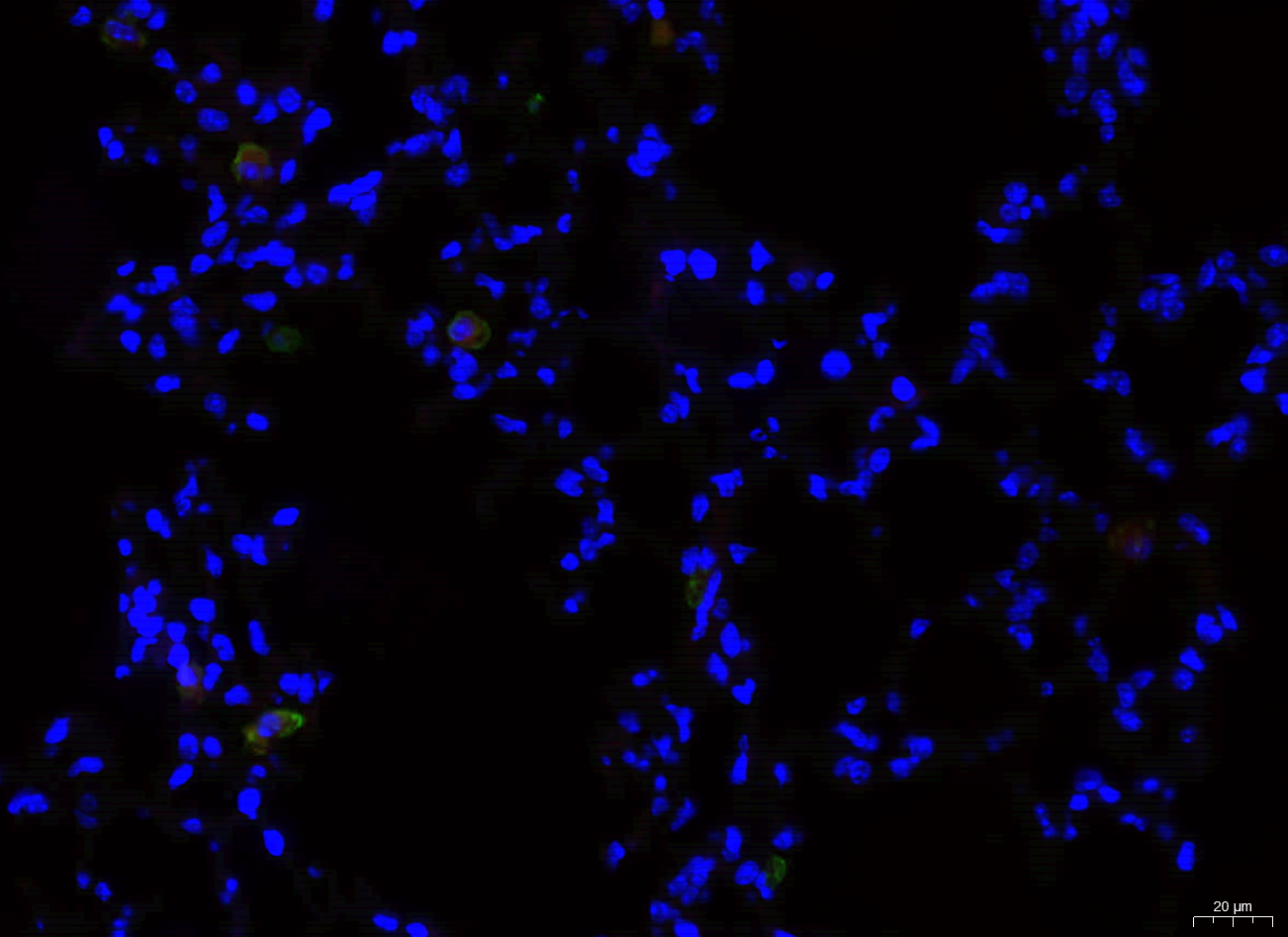

Supplement: Supplementary file 4 — Supporting File 4: advs73867‐sup‐0001‐FiguresData.zip. [file ADVS-13-e19191-s001.zip › Supporting information Figure1-10/Figure 7/Figure 7D/Control 774_ IF_20240117150104370_40.0x-Merge-.jpg]

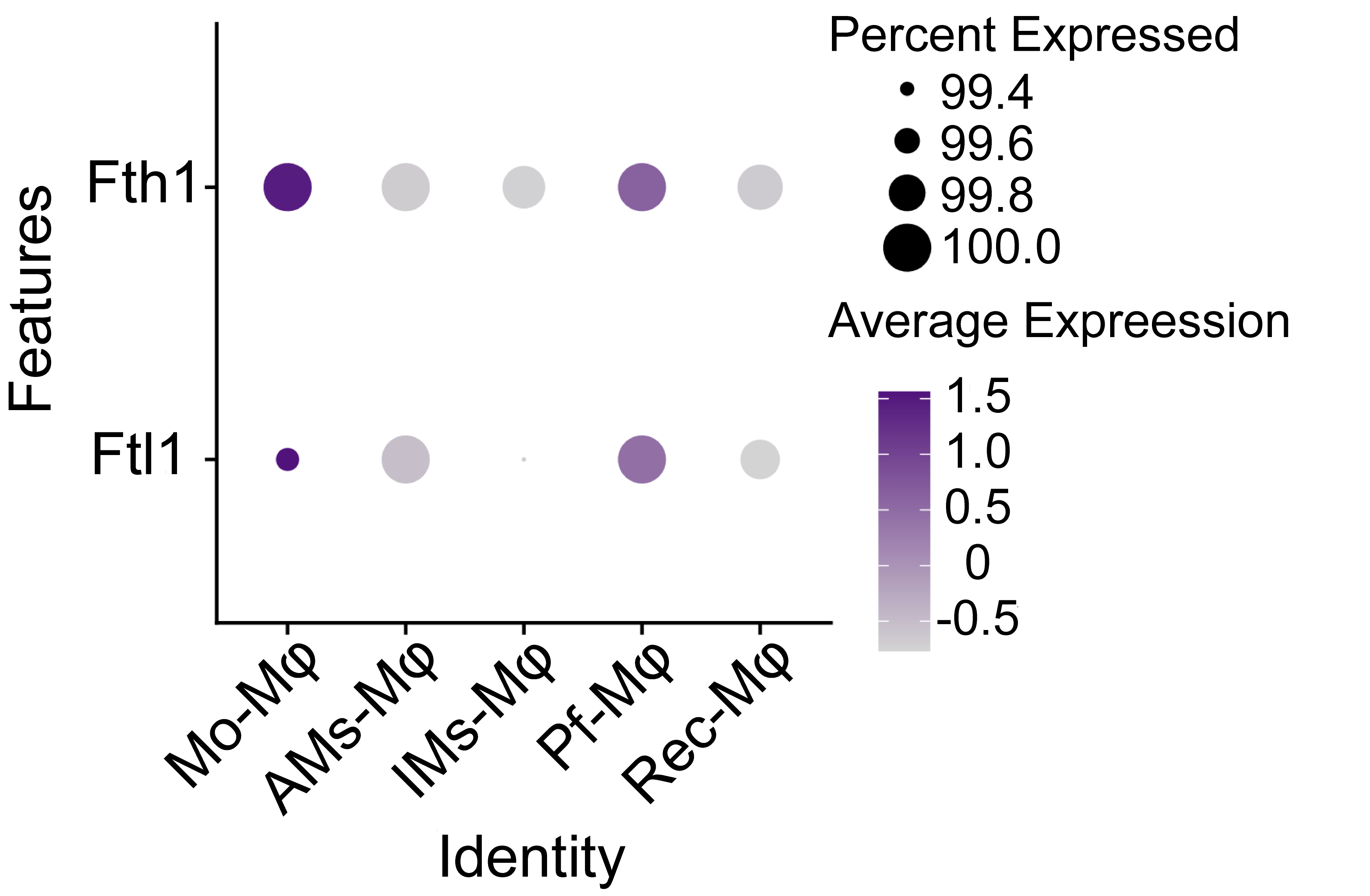

Supplement: Supplementary file 4 — Supporting File 4: advs73867‐sup‐0001‐FiguresData.zip. [file ADVS-13-e19191-s001.zip › Supporting information Figure1-10/Figure 7/Figure 7E.tif]

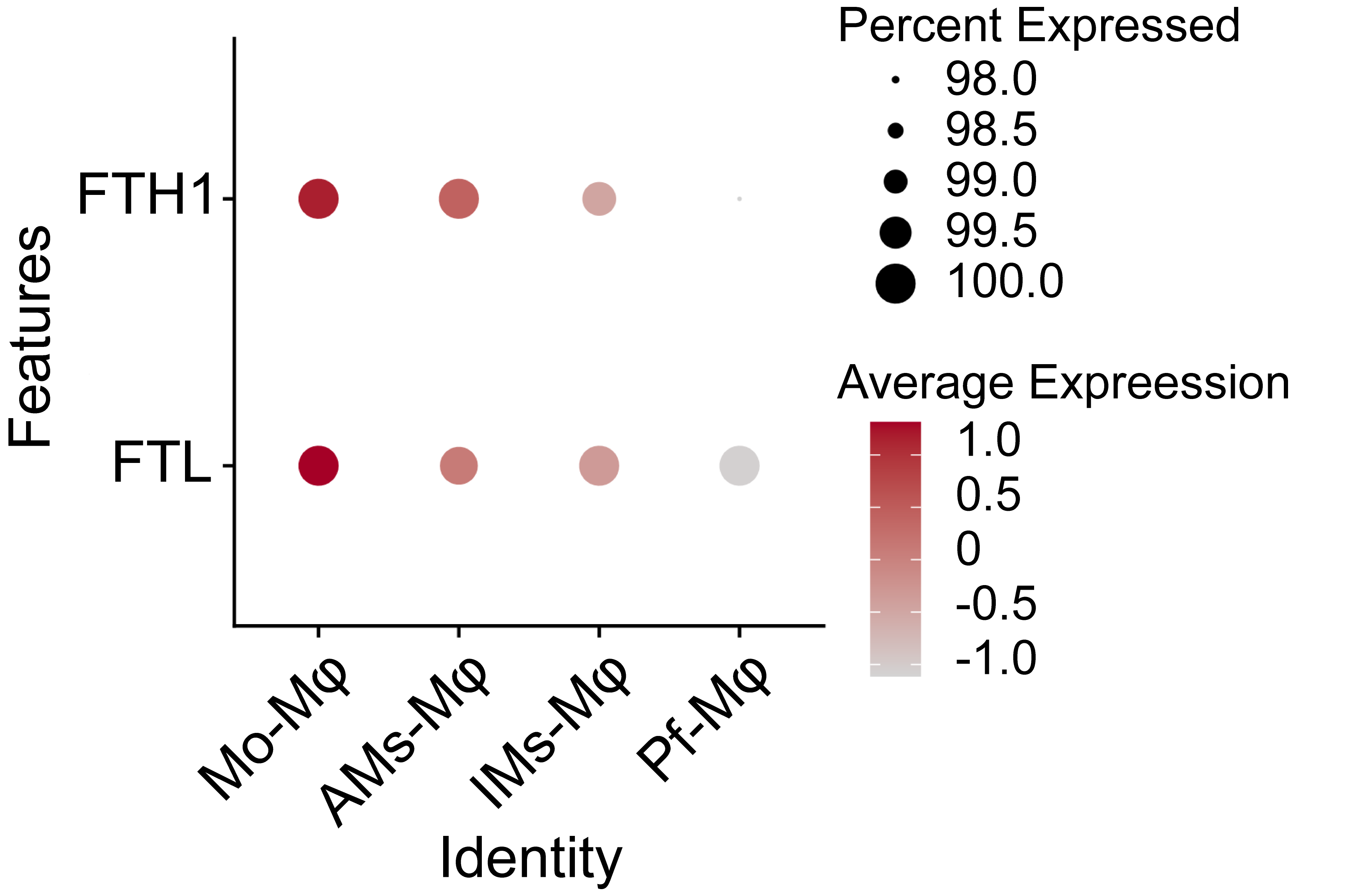

Supplement: Supplementary file 4 — Supporting File 4: advs73867‐sup‐0001‐FiguresData.zip. [file ADVS-13-e19191-s001.zip › Supporting information Figure1-10/Figure 7/Figure 7F.tif]

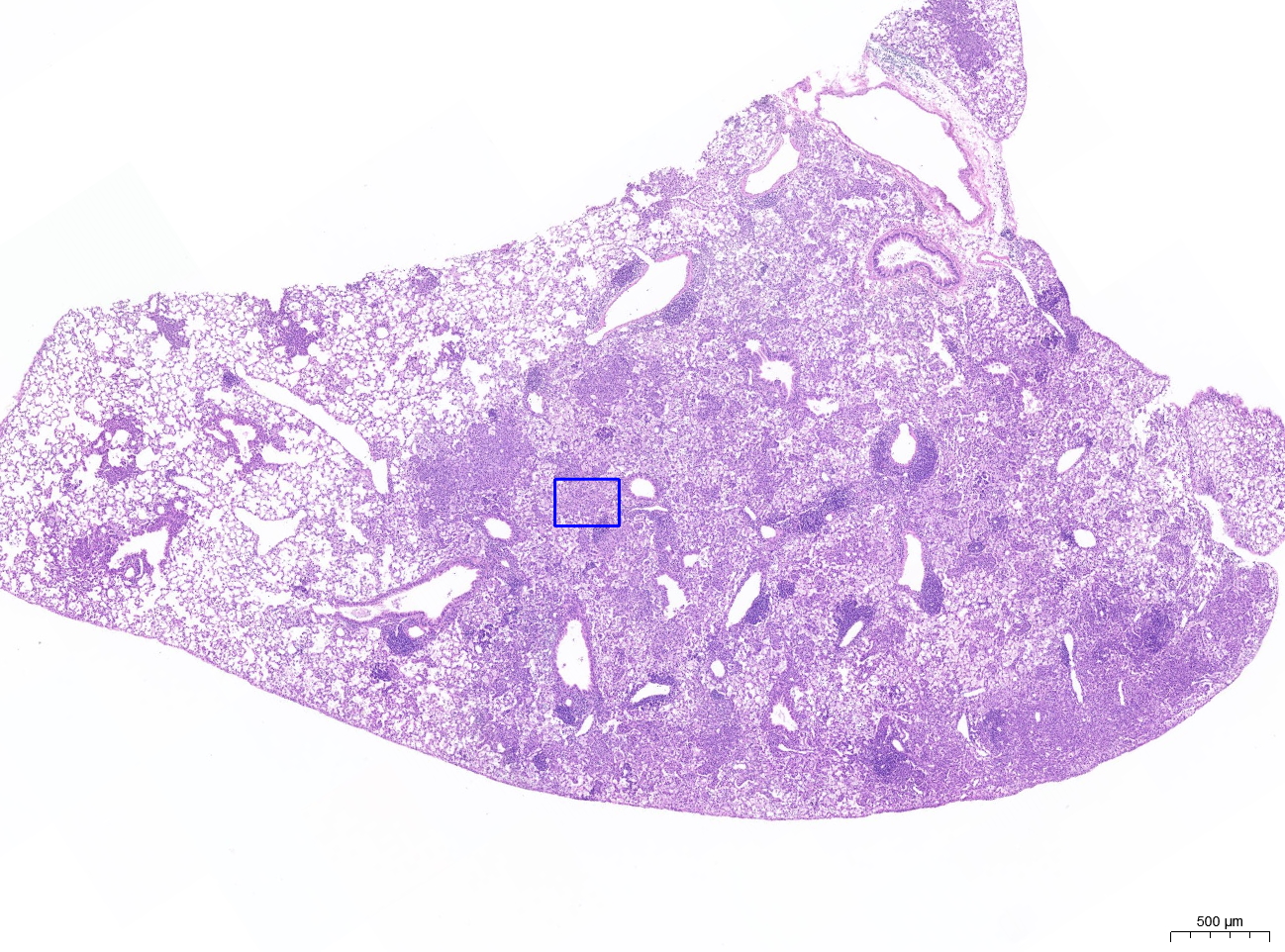

Supplement: Supplementary file 4 — Supporting File 4: advs73867‐sup‐0001‐FiguresData.zip. [file ADVS-13-e19191-s001.zip › Supporting information Figure1-10/Figure 8/Figure 8C/aav-c+Silica-B25_2.0x.jpg]

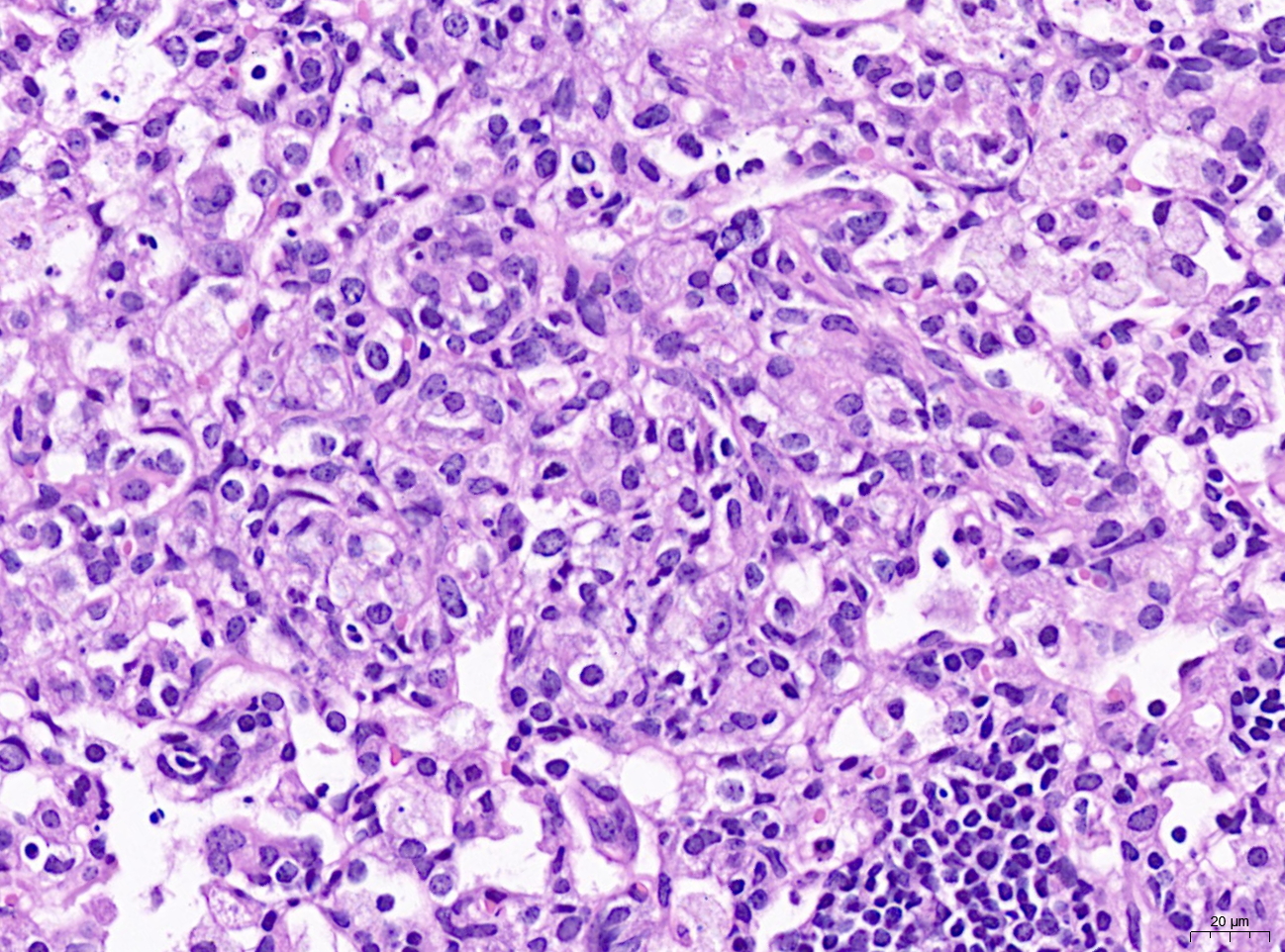

Supplement: Supplementary file 4 — Supporting File 4: advs73867‐sup‐0001‐FiguresData.zip. [file ADVS-13-e19191-s001.zip › Supporting information Figure1-10/Figure 8/Figure 8C/aav-c+Silica-B25_40.0x.jpg]

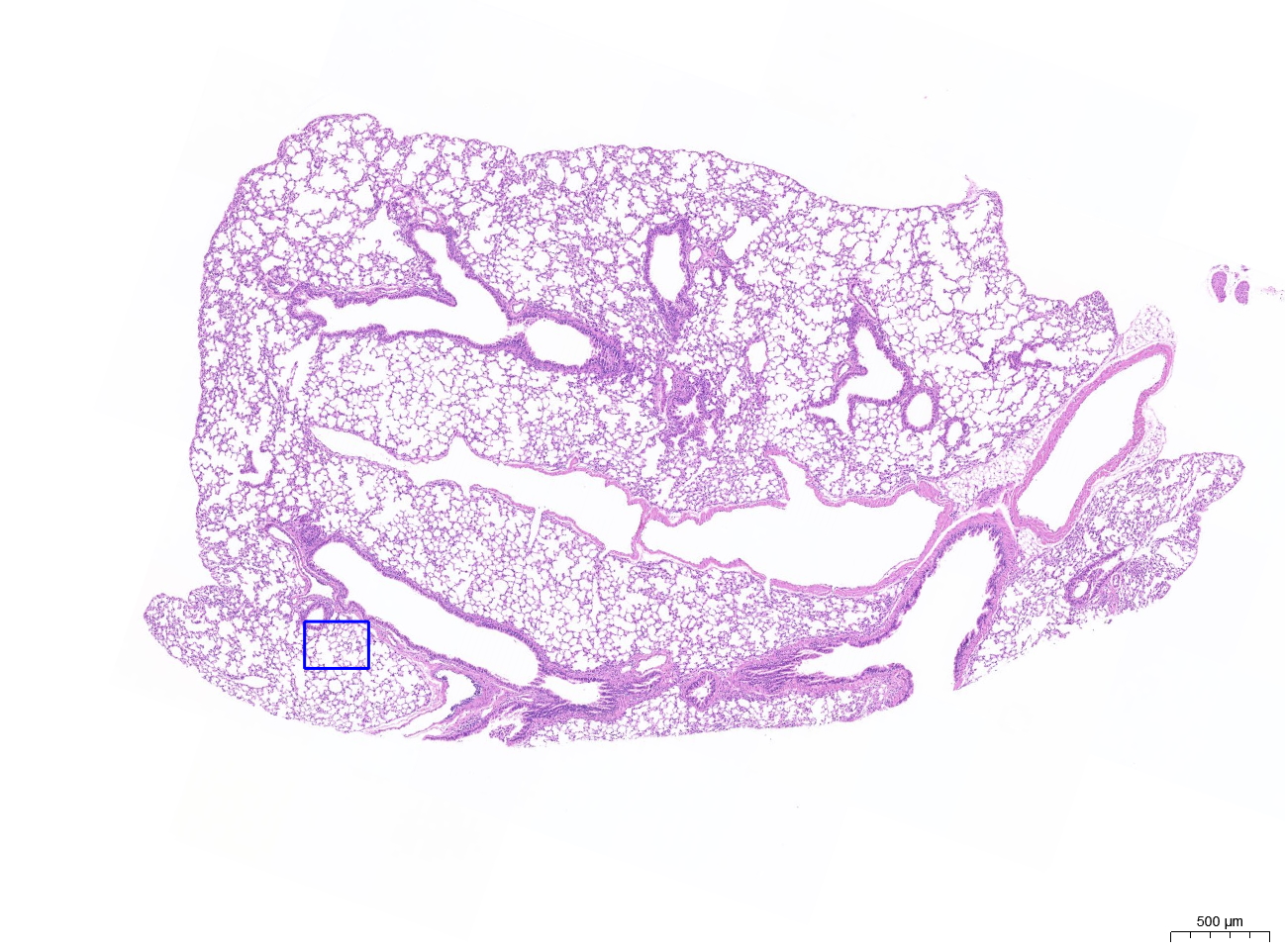

Supplement: Supplementary file 4 — Supporting File 4: advs73867‐sup‐0001‐FiguresData.zip. [file ADVS-13-e19191-s001.zip › Supporting information Figure1-10/Figure 8/Figure 8C/aav-c-b6_2.0x.jpg]

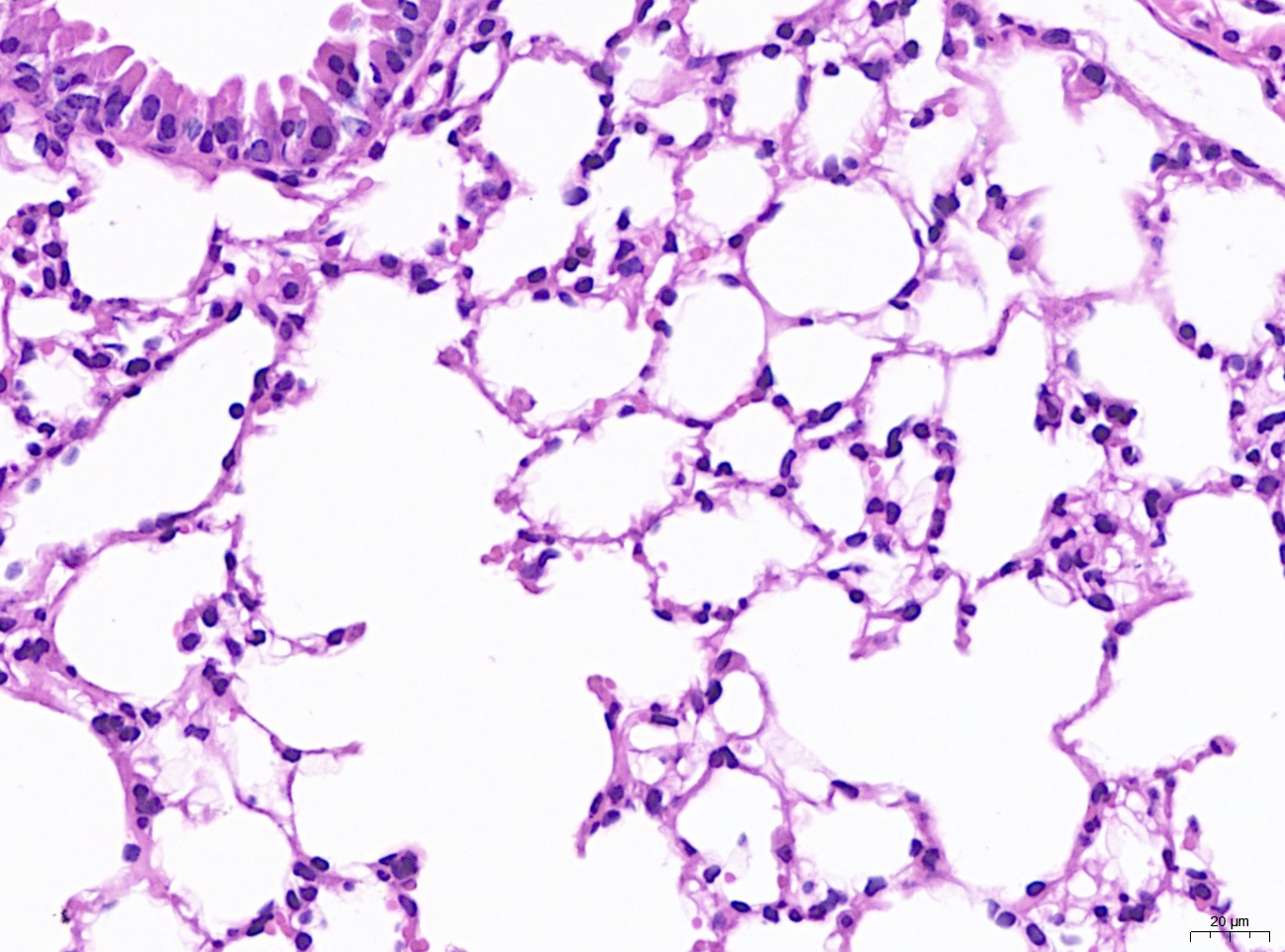

Supplement: Supplementary file 4 — Supporting File 4: advs73867‐sup‐0001‐FiguresData.zip. [file ADVS-13-e19191-s001.zip › Supporting information Figure1-10/Figure 8/Figure 8C/AAV-C-B6-40.0x.jpg]

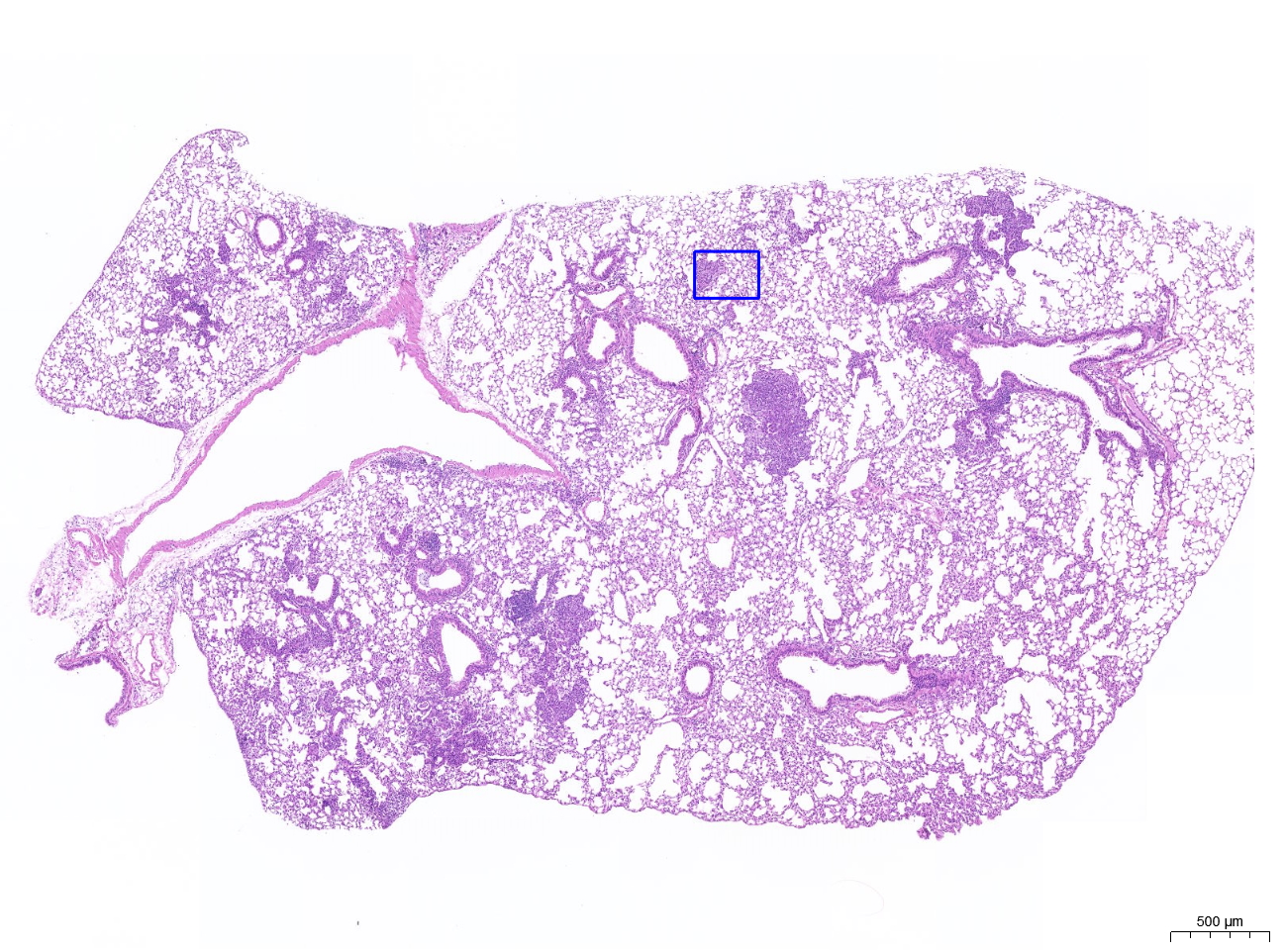

Supplement: Supplementary file 4 — Supporting File 4: advs73867‐sup‐0001‐FiguresData.zip. [file ADVS-13-e19191-s001.zip › Supporting information Figure1-10/Figure 8/Figure 8C/aav-f+Silica-b20_2.0x.jpg]

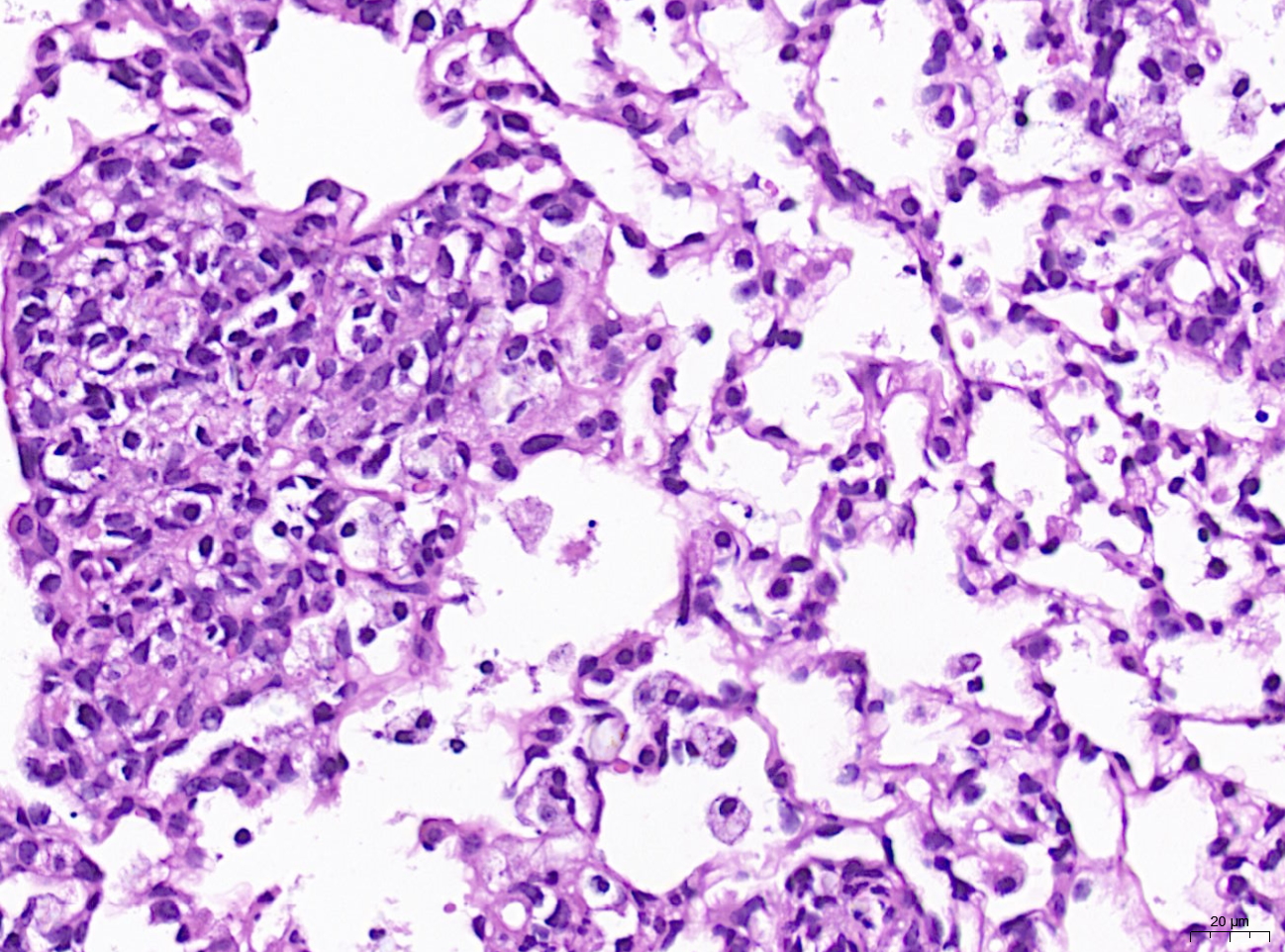

Supplement: Supplementary file 4 — Supporting File 4: advs73867‐sup‐0001‐FiguresData.zip. [file ADVS-13-e19191-s001.zip › Supporting information Figure1-10/Figure 8/Figure 8C/aav-f+Silica-b20_40.0x.jpg]

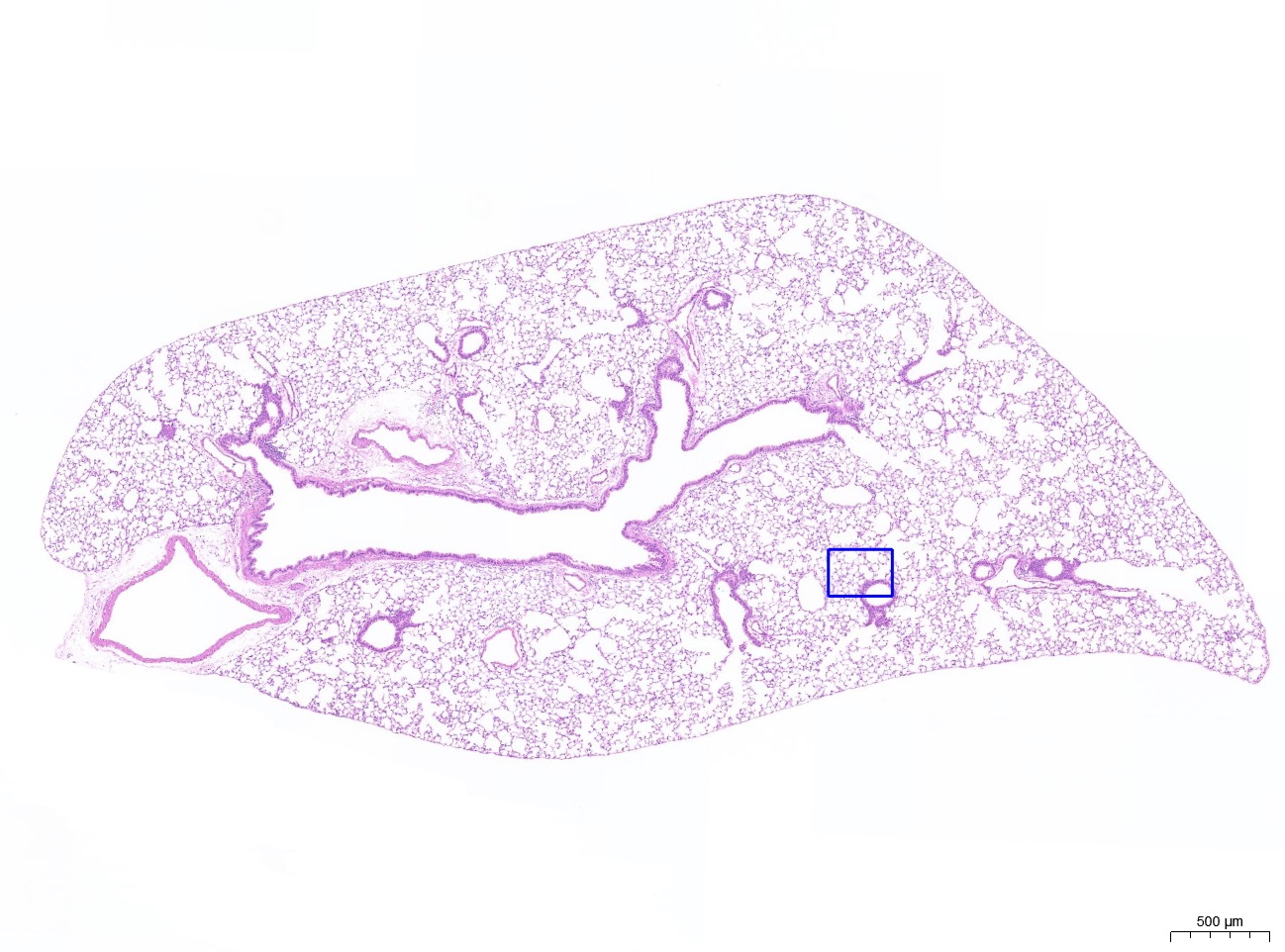

Supplement: Supplementary file 4 — Supporting File 4: advs73867‐sup‐0001‐FiguresData.zip. [file ADVS-13-e19191-s001.zip › Supporting information Figure1-10/Figure 8/Figure 8C/AAV-F-B14_2.0x.jpg]
