# Supplementary figures and images for: Macrophage–Derived Ferritin Exacerbates Silica‐Induced Pulmonary Fibrosis via PIK3R2‐Mediated Fibroblast Differentiation (part 3 of 4)
Source: Adv Sci (Weinh). 2026 Jan 21;13(17):e19191. doi: 10.1002/advs.202519191 (PMC13042690; doi:10.1002/advs.202519191)

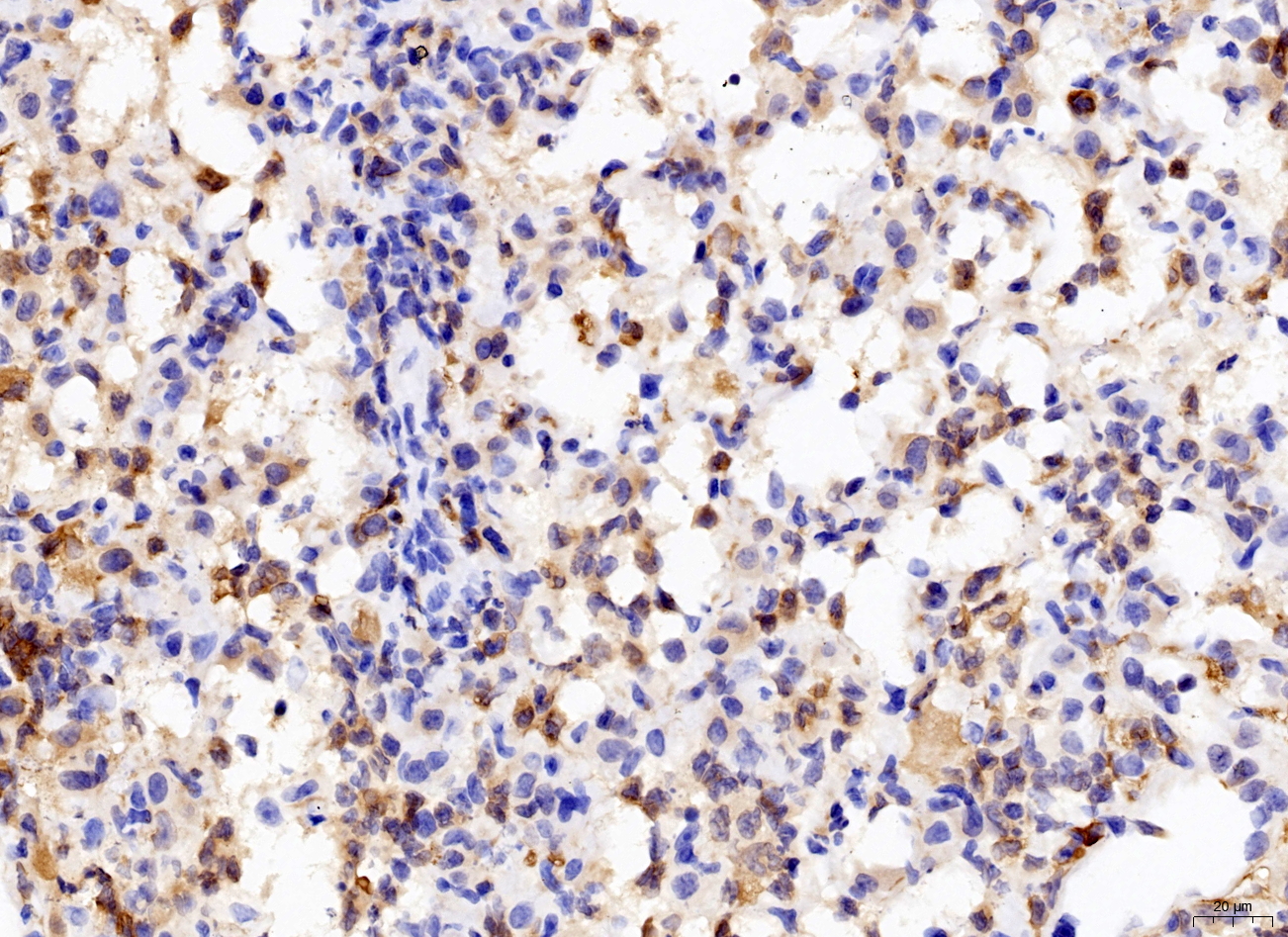

Supplement: Supplementary file 4 — Supporting File 4: advs73867‐sup‐0001‐FiguresData.zip. [file ADVS-13-e19191-s001.zip › Supporting information Figure1-10/Figure 2/Figure 2I/SCRS-Ferritin-12w-Model-765_40.0x.jpg]

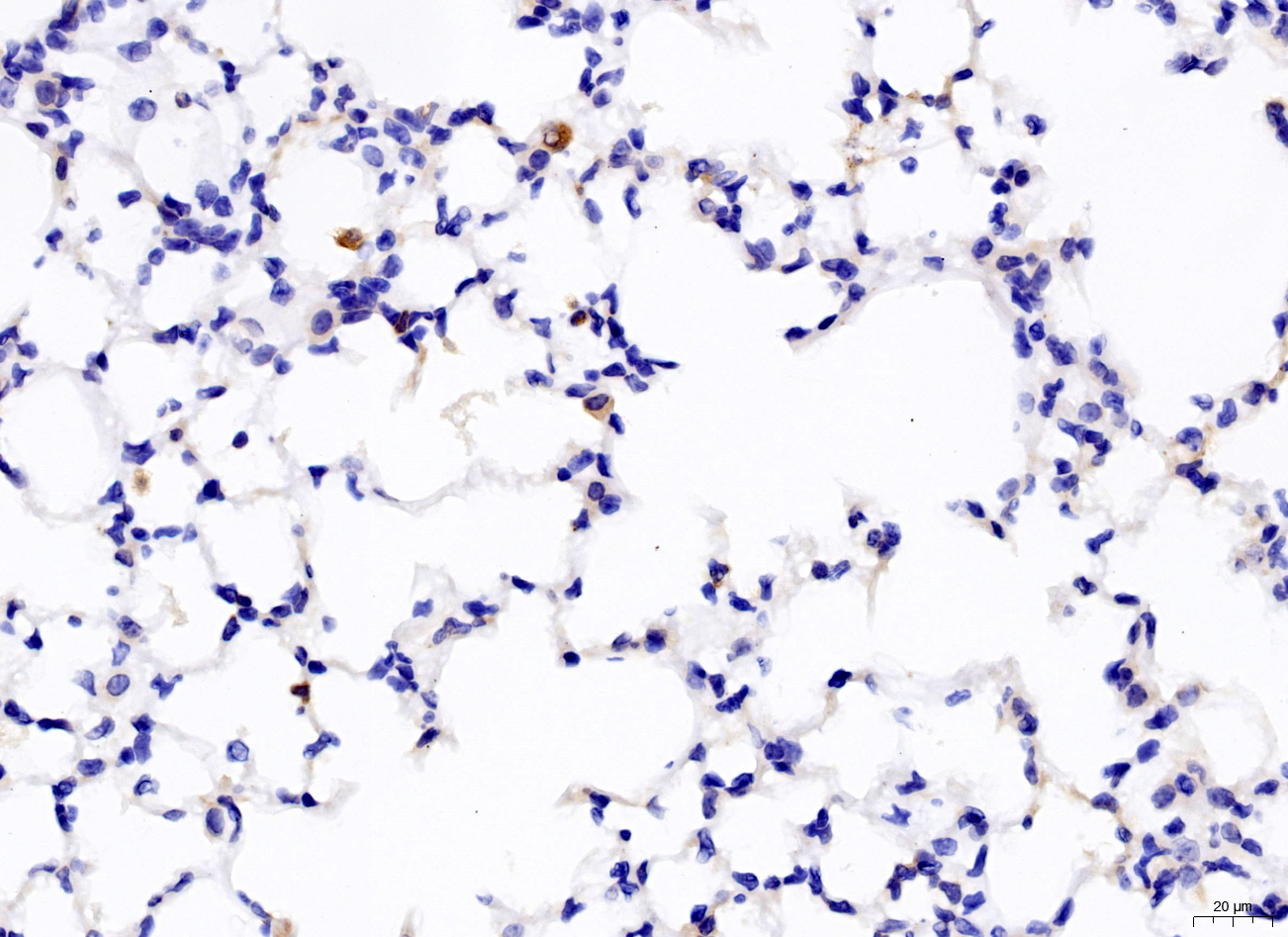

Supplement: Supplementary file 4 — Supporting File 4: advs73867‐sup‐0001‐FiguresData.zip. [file ADVS-13-e19191-s001.zip › Supporting information Figure1-10/Figure 2/Figure 2I/SCRS-Ferritin-4w-Control-774_40.0x.jpg]

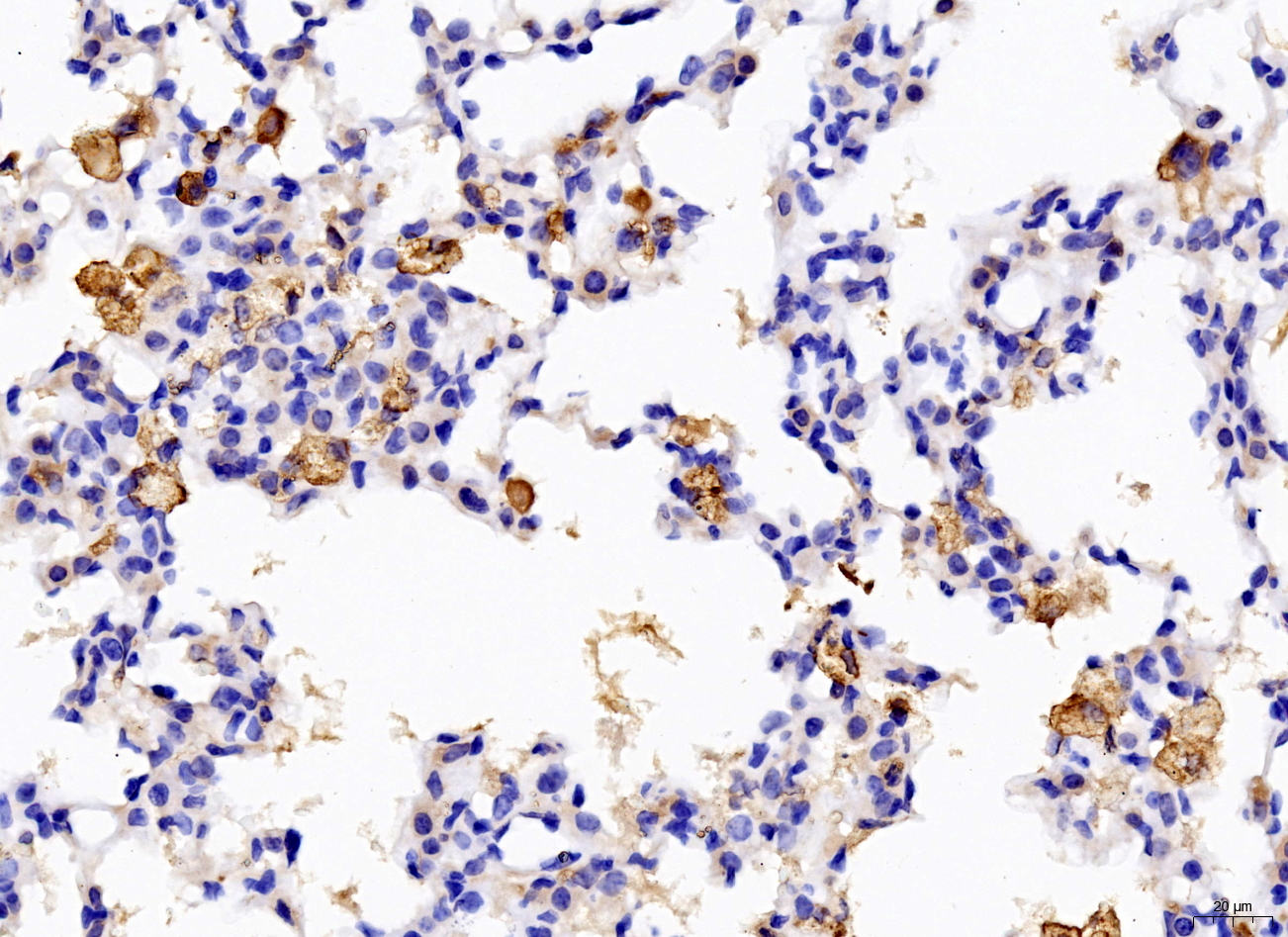

Supplement: Supplementary file 4 — Supporting File 4: advs73867‐sup‐0001‐FiguresData.zip. [file ADVS-13-e19191-s001.zip › Supporting information Figure1-10/Figure 2/Figure 2I/SCRS-Ferritin-4w-Model-744_40.0x.jpg]

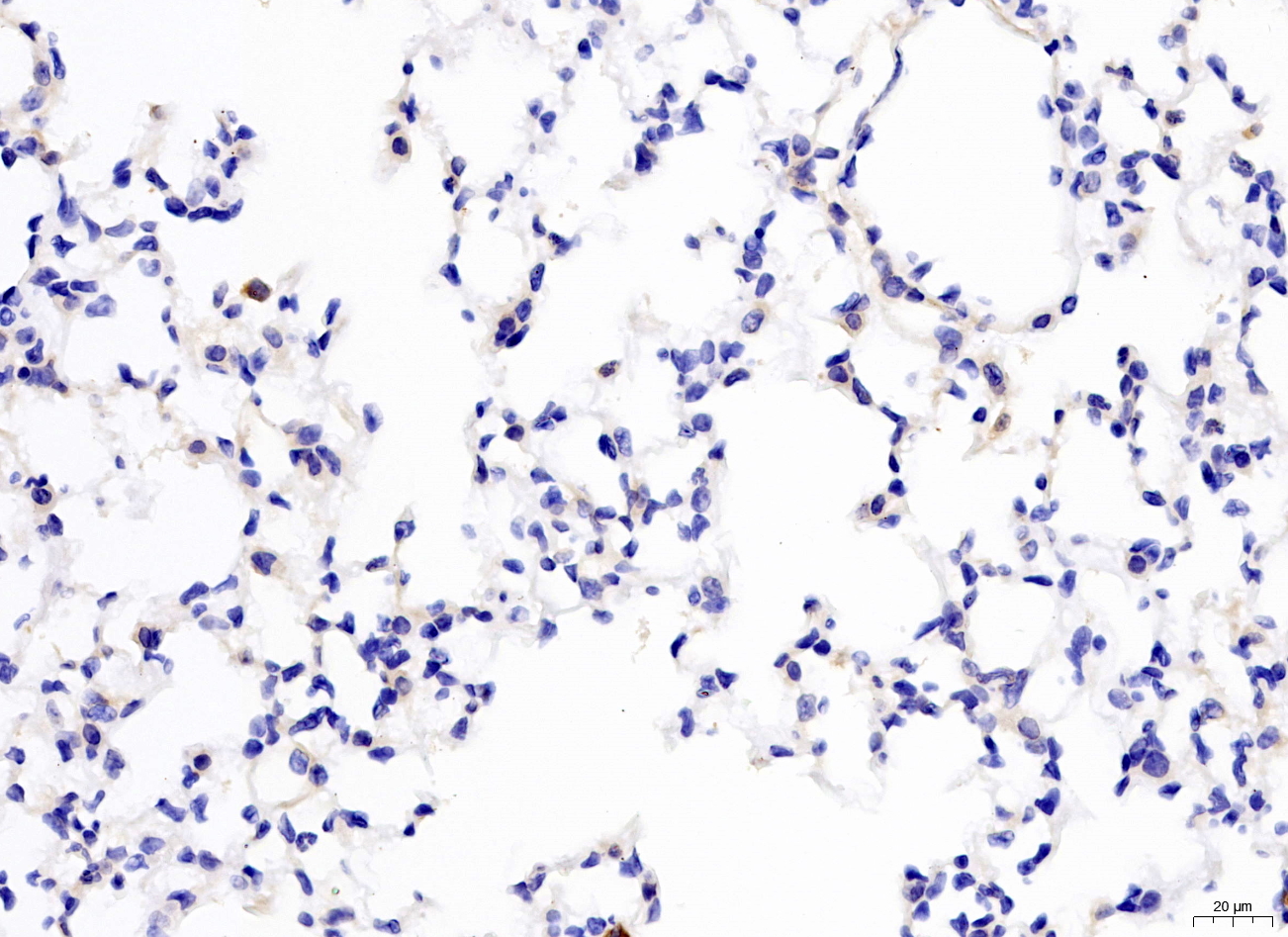

Supplement: Supplementary file 4 — Supporting File 4: advs73867‐sup‐0001‐FiguresData.zip. [file ADVS-13-e19191-s001.zip › Supporting information Figure1-10/Figure 2/Figure 2I/SCRS-Ferritin-8w-Control-785_40.0x.jpg]

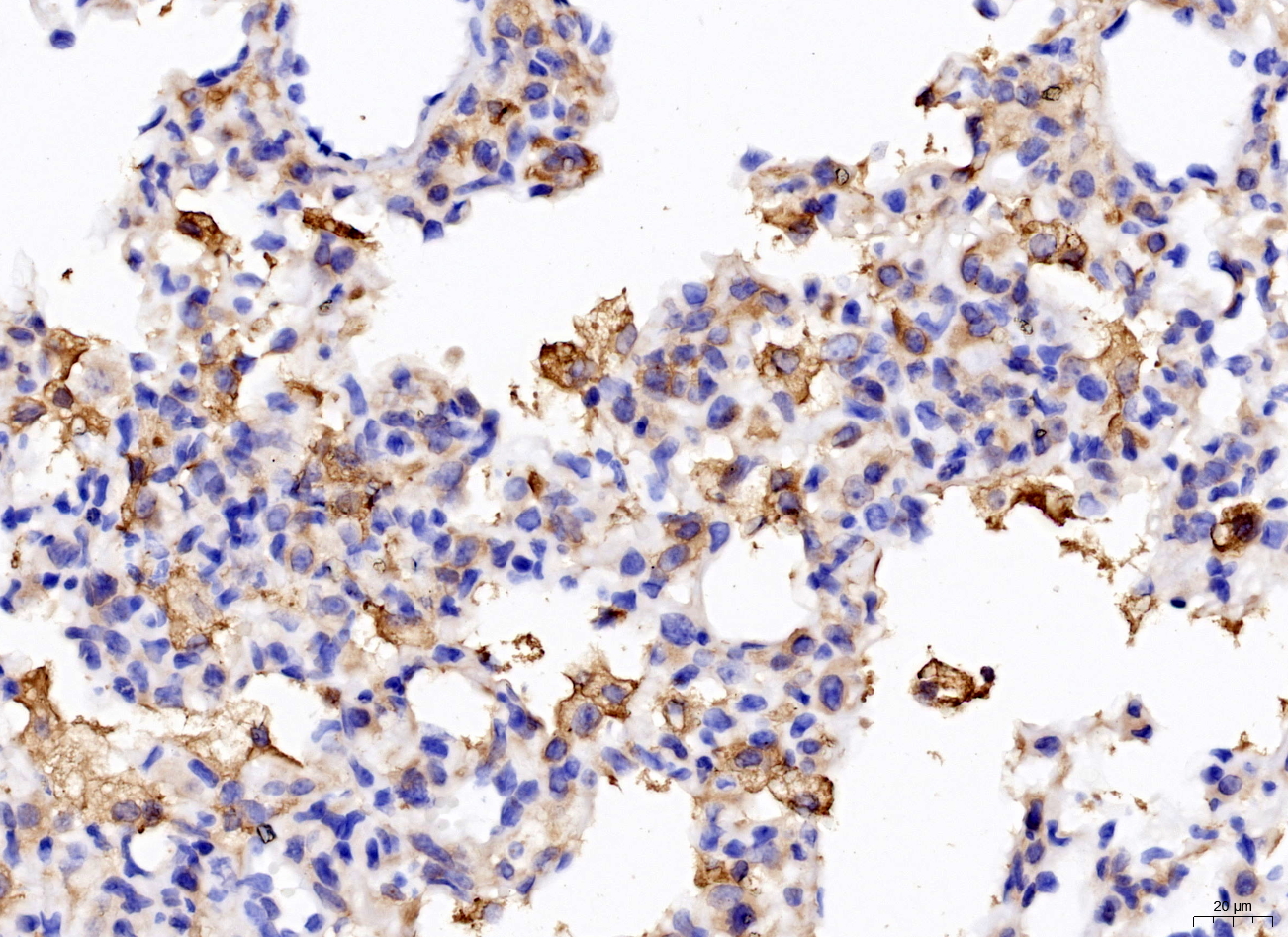

Supplement: Supplementary file 4 — Supporting File 4: advs73867‐sup‐0001‐FiguresData.zip. [file ADVS-13-e19191-s001.zip › Supporting information Figure1-10/Figure 2/Figure 2I/SCRS-Ferritin-8w-Model-750_40.0x.jpg]

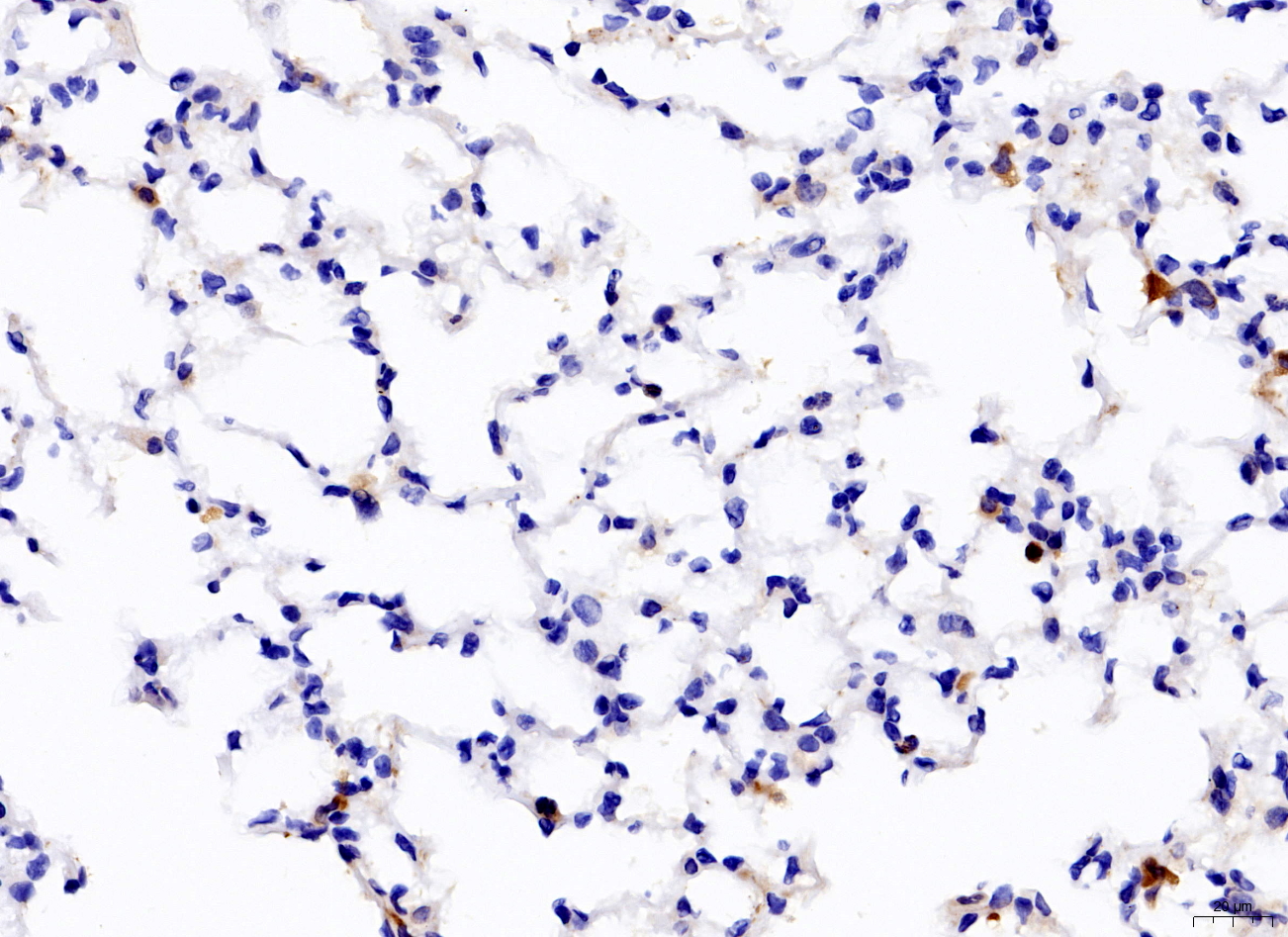

Supplement: Supplementary file 4 — Supporting File 4: advs73867‐sup‐0001‐FiguresData.zip. [file ADVS-13-e19191-s001.zip › Supporting information Figure1-10/Figure 2/Figure 2J/1 week Control/SCRS-1 week-Control-779-2_40.0x.jpg]

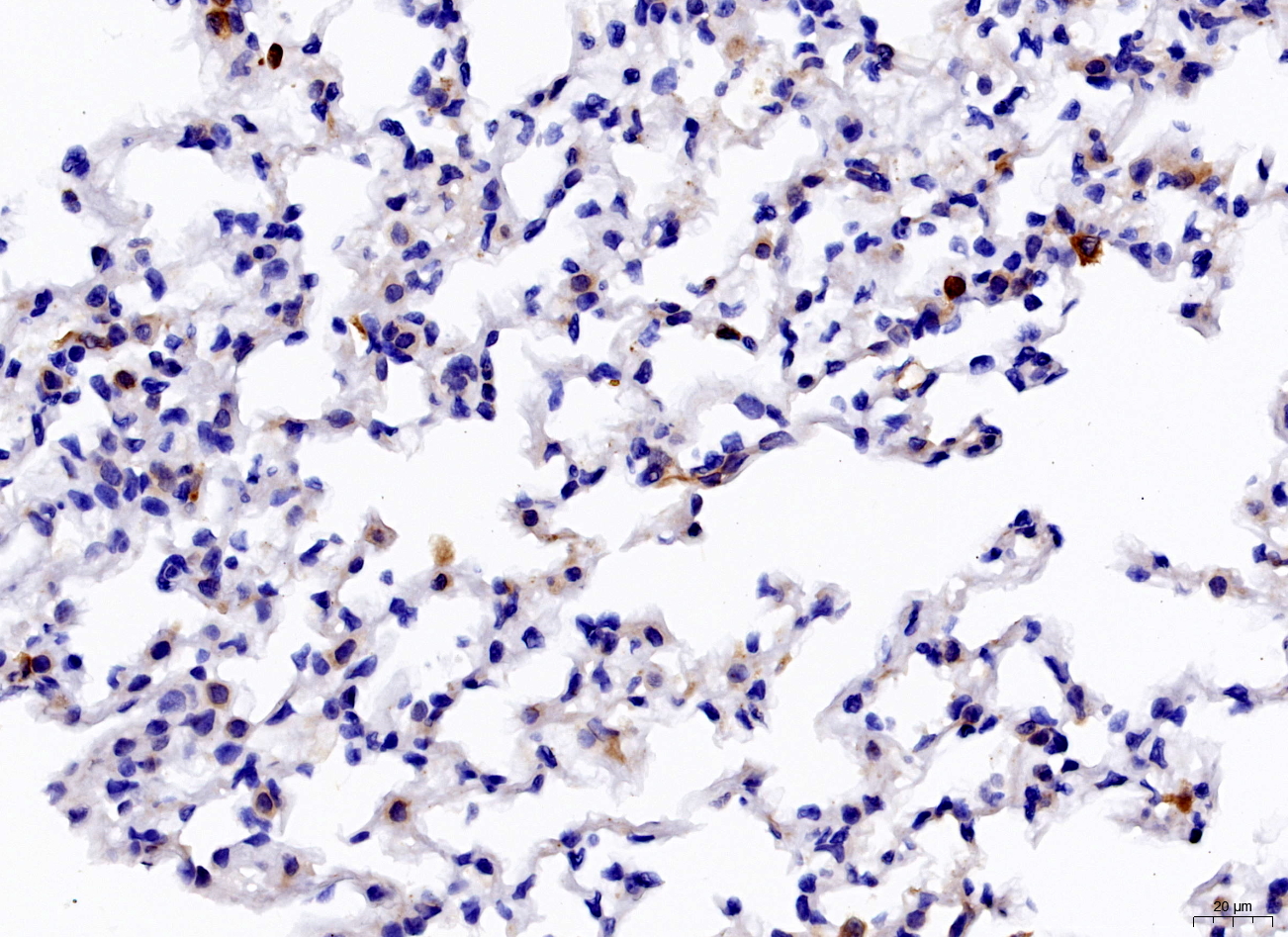

Supplement: Supplementary file 4 — Supporting File 4: advs73867‐sup‐0001‐FiguresData.zip. [file ADVS-13-e19191-s001.zip › Supporting information Figure1-10/Figure 2/Figure 2J/1 week Control/SCRS-1 week-Control-786_40.0x-3.jpg]

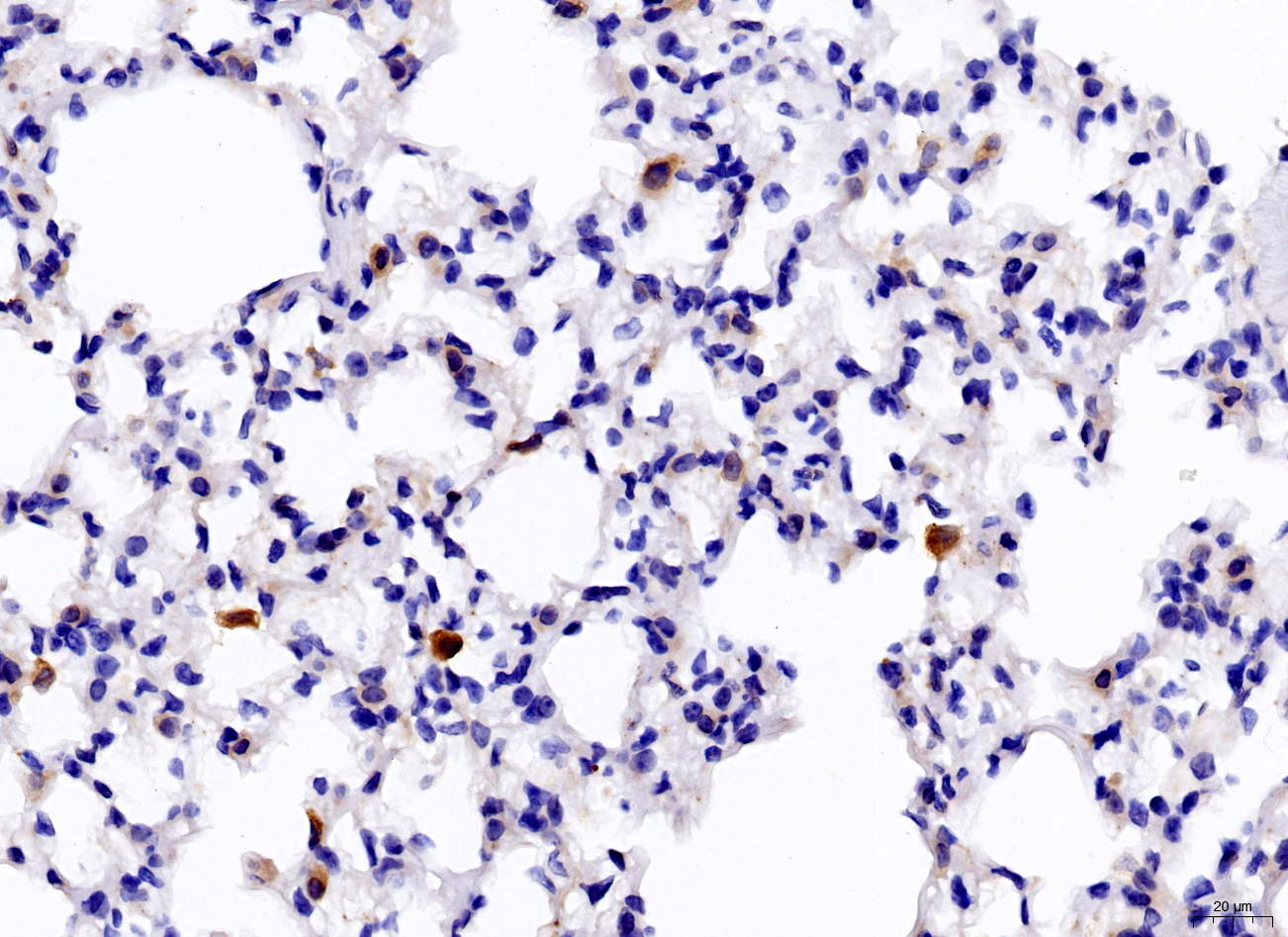

Supplement: Supplementary file 4 — Supporting File 4: advs73867‐sup‐0001‐FiguresData.zip. [file ADVS-13-e19191-s001.zip › Supporting information Figure1-10/Figure 2/Figure 2J/1 week Control/SCRS-1 week-Control-786_40.0x-4.jpg]

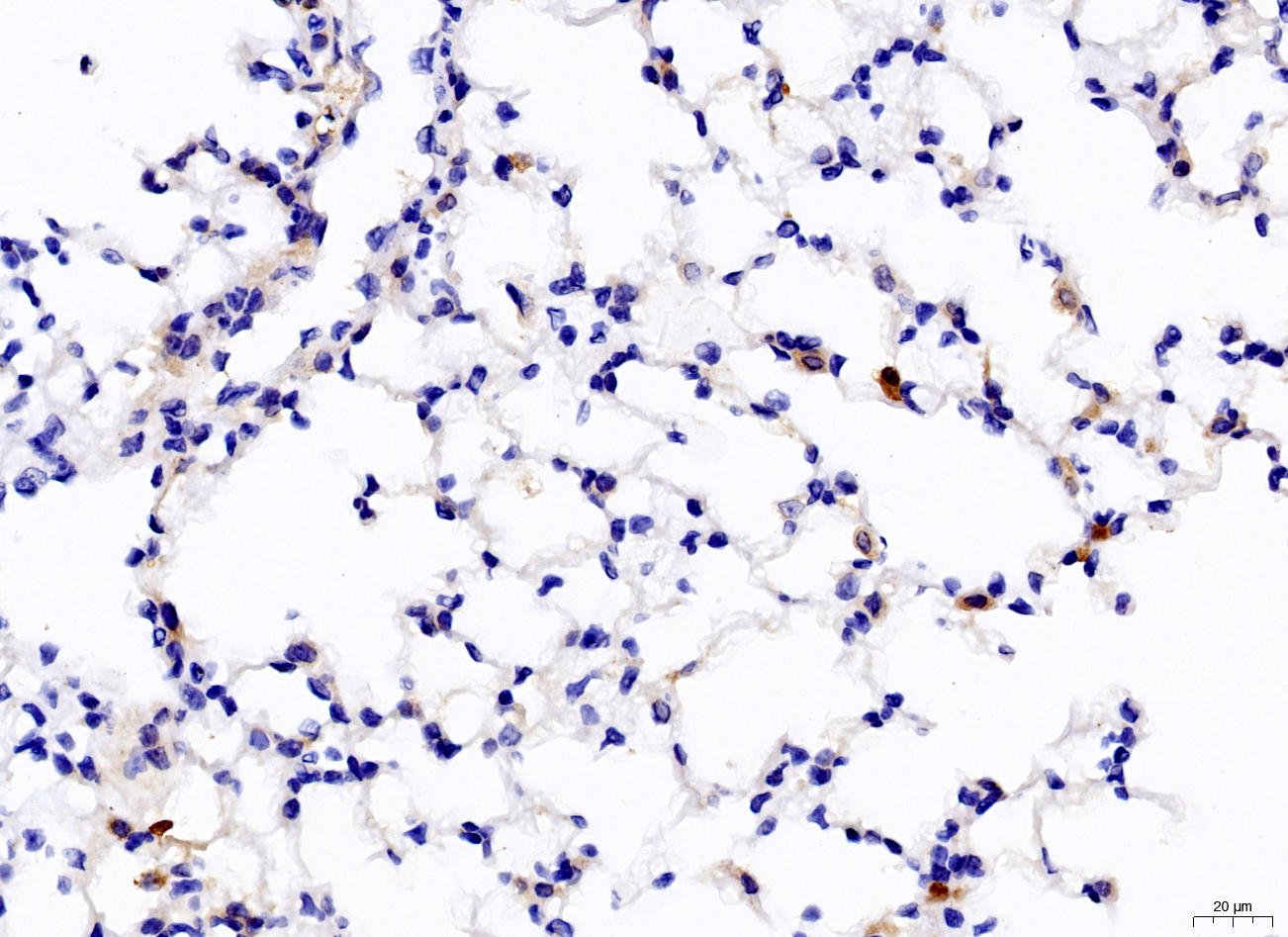

Supplement: Supplementary file 4 — Supporting File 4: advs73867‐sup‐0001‐FiguresData.zip. [file ADVS-13-e19191-s001.zip › Supporting information Figure1-10/Figure 2/Figure 2J/1 week Control/SCRS-1 week-Control-792_40.0x-5.jpg]

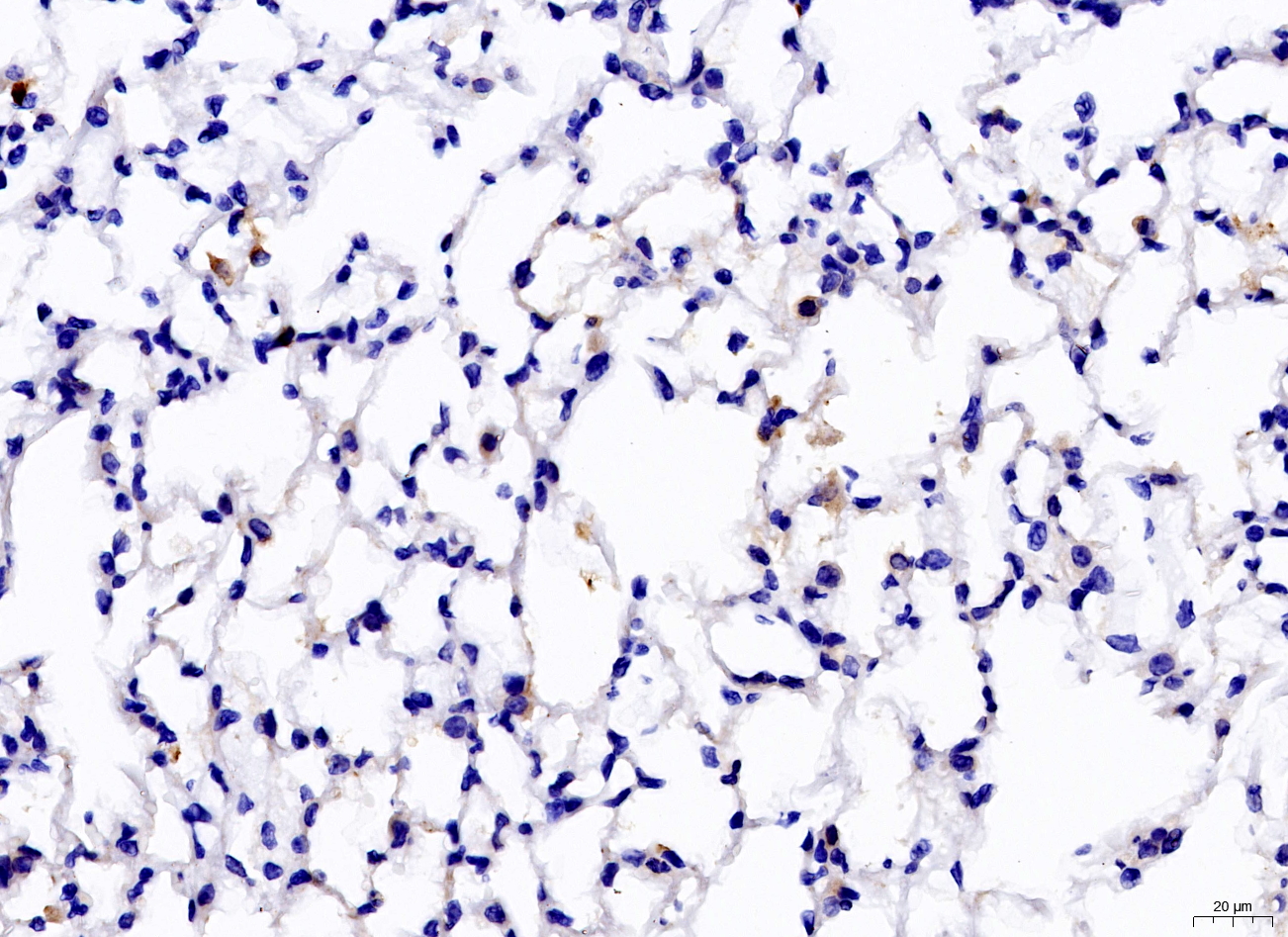

Supplement: Supplementary file 4 — Supporting File 4: advs73867‐sup‐0001‐FiguresData.zip. [file ADVS-13-e19191-s001.zip › Supporting information Figure1-10/Figure 2/Figure 2J/1 week Control/SCRS-1 week-Control-792_40.0x-6.jpg]

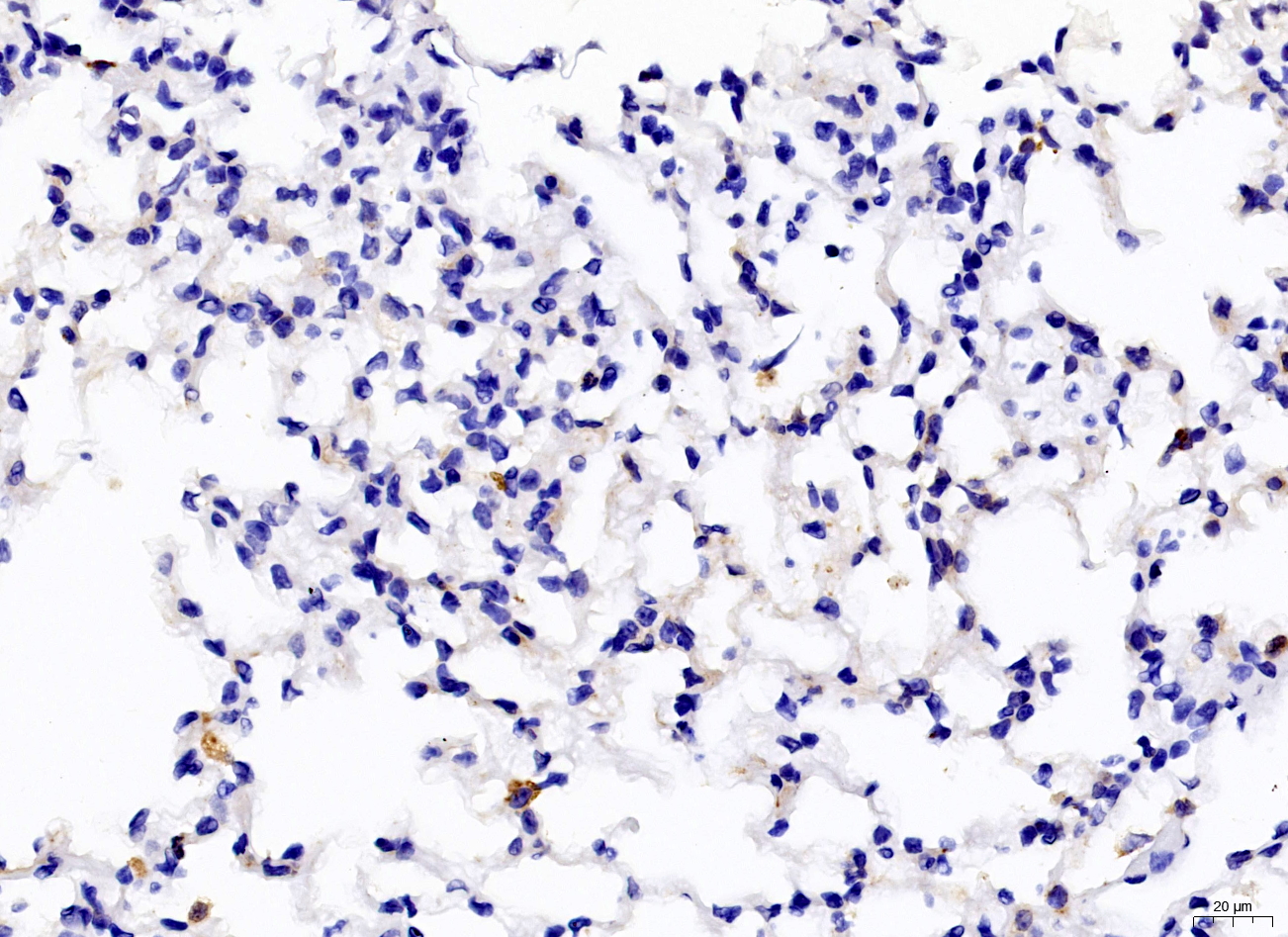

Supplement: Supplementary file 4 — Supporting File 4: advs73867‐sup‐0001‐FiguresData.zip. [file ADVS-13-e19191-s001.zip › Supporting information Figure1-10/Figure 2/Figure 2J/1 week Control/SCRS-1 week-Control-792_40.0x-7.jpg]

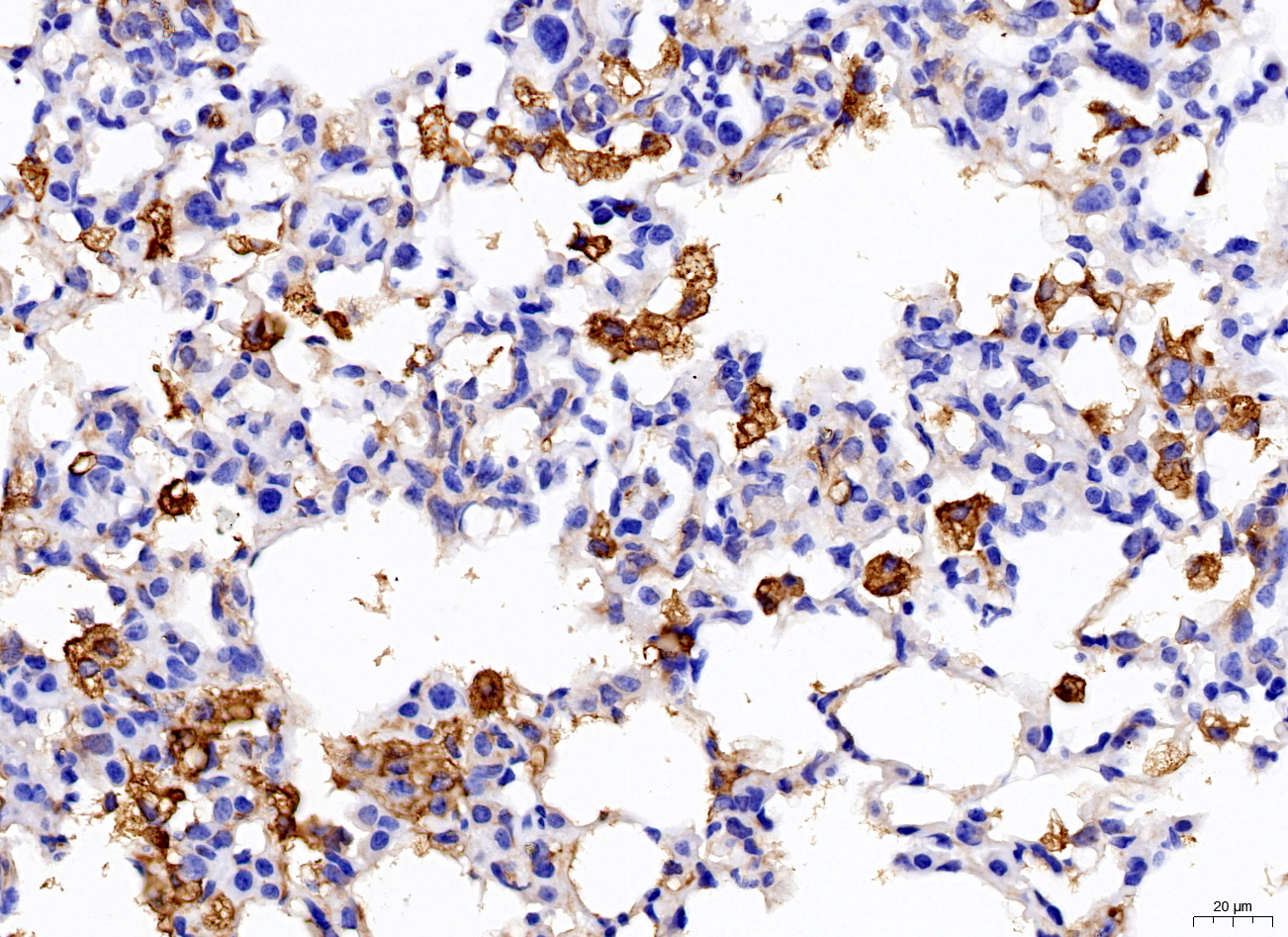

Supplement: Supplementary file 4 — Supporting File 4: advs73867‐sup‐0001‐FiguresData.zip. [file ADVS-13-e19191-s001.zip › Supporting information Figure1-10/Figure 2/Figure 2J/1 week Silica/SCRS-1 week-Model-736_40.0x-1.jpg]

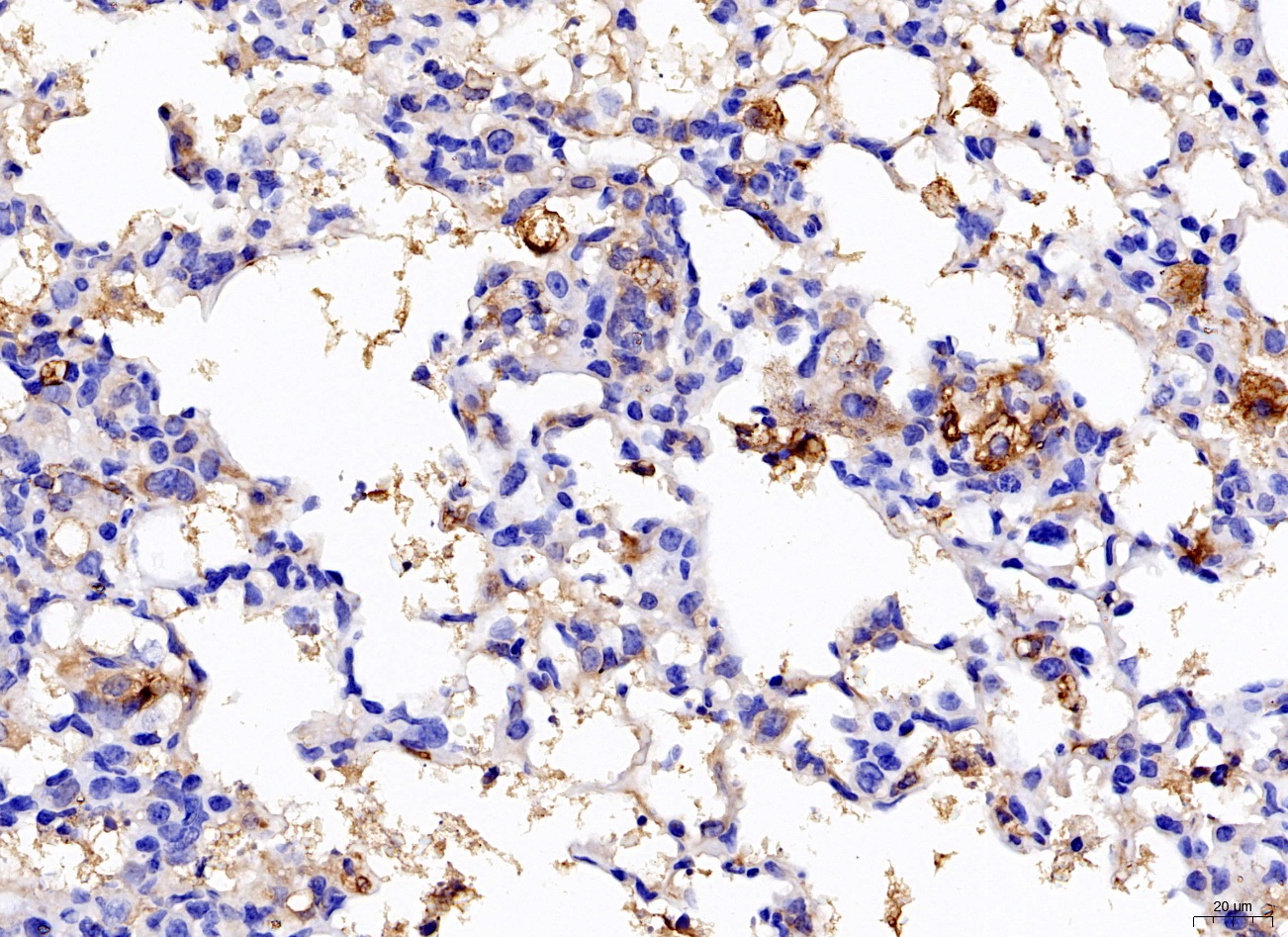

Supplement: Supplementary file 4 — Supporting File 4: advs73867‐sup‐0001‐FiguresData.zip. [file ADVS-13-e19191-s001.zip › Supporting information Figure1-10/Figure 2/Figure 2J/1 week Silica/SCRS-1 week-Model-736_40.0x-2.jpg]

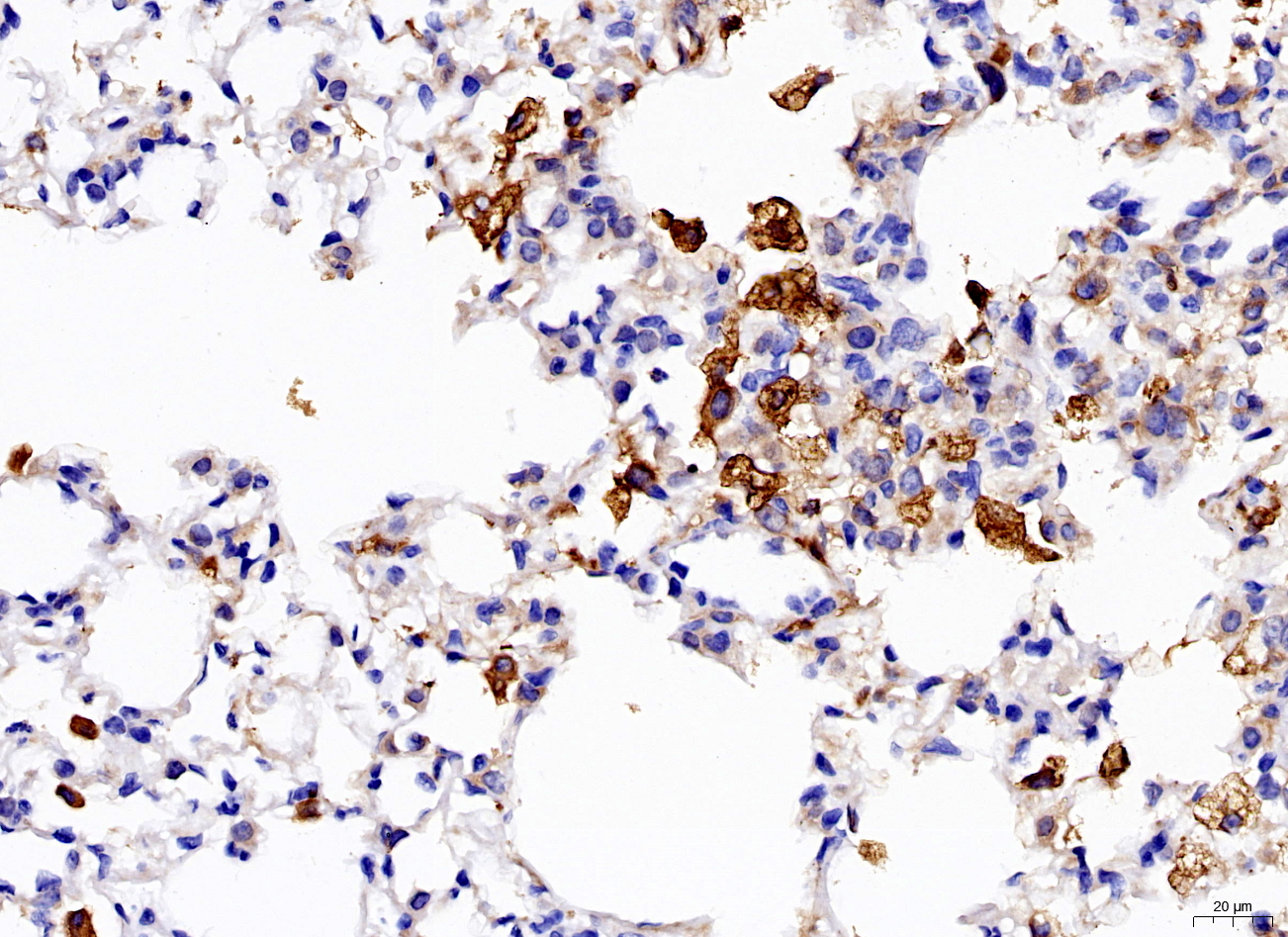

Supplement: Supplementary file 4 — Supporting File 4: advs73867‐sup‐0001‐FiguresData.zip. [file ADVS-13-e19191-s001.zip › Supporting information Figure1-10/Figure 2/Figure 2J/1 week Silica/SCRS-1 week-Model-736_40.0x-3.jpg]

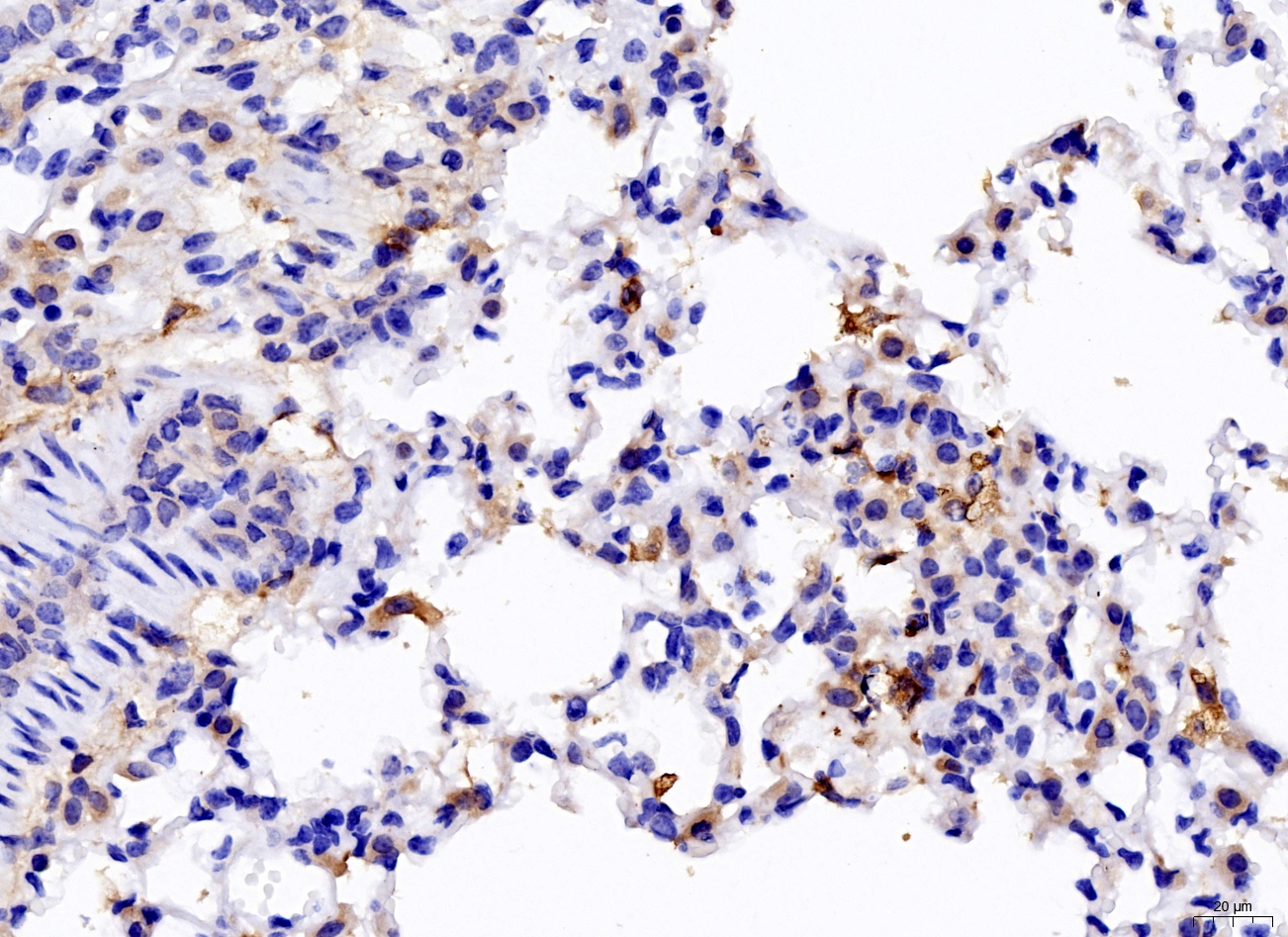

Supplement: Supplementary file 4 — Supporting File 4: advs73867‐sup‐0001‐FiguresData.zip. [file ADVS-13-e19191-s001.zip › Supporting information Figure1-10/Figure 2/Figure 2J/1 week Silica/SCRS-1 week-Model-762_40.0x-4.jpg]

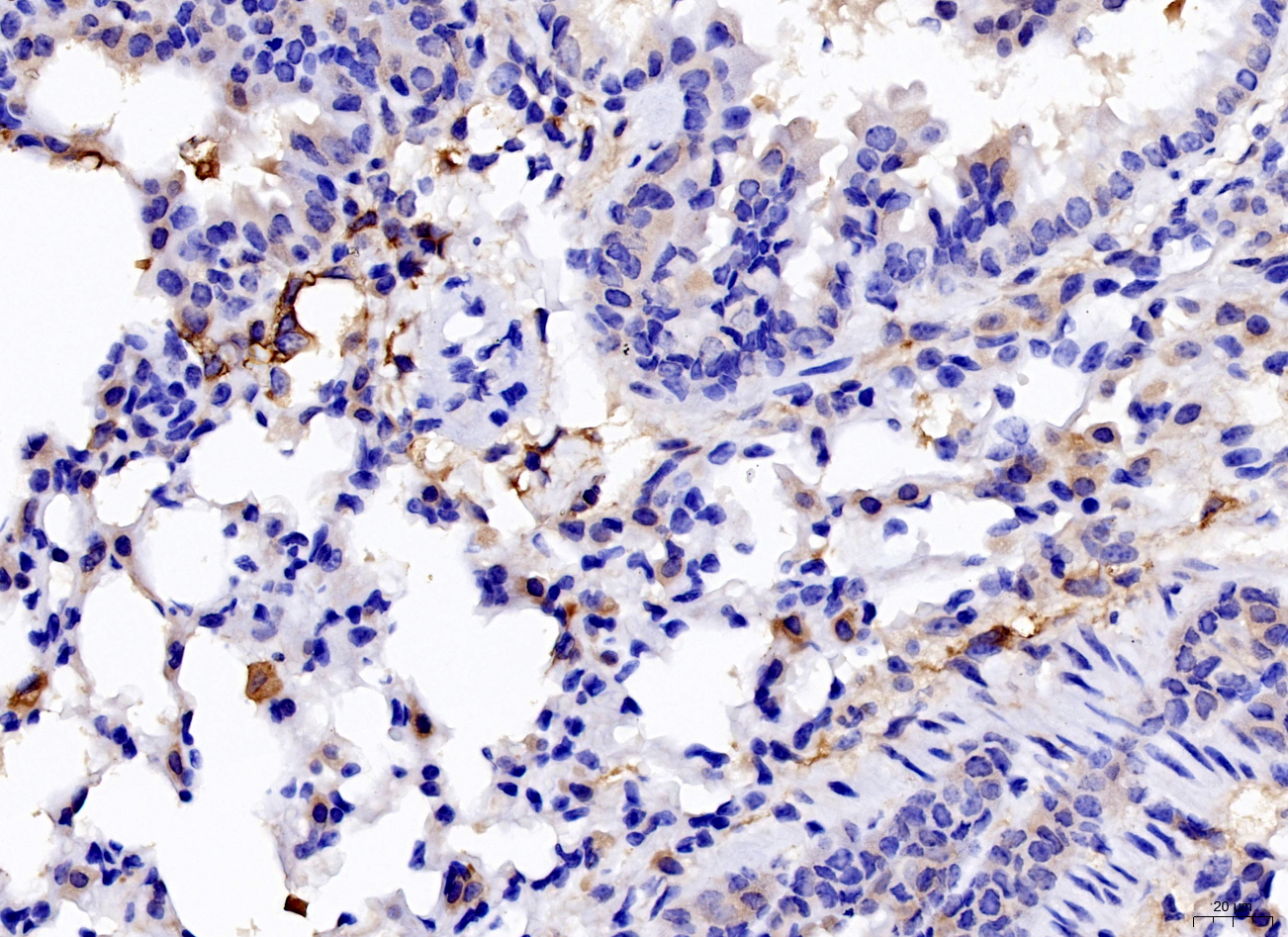

Supplement: Supplementary file 4 — Supporting File 4: advs73867‐sup‐0001‐FiguresData.zip. [file ADVS-13-e19191-s001.zip › Supporting information Figure1-10/Figure 2/Figure 2J/1 week Silica/SCRS-1 week-Model-762_40.0x-5.jpg]

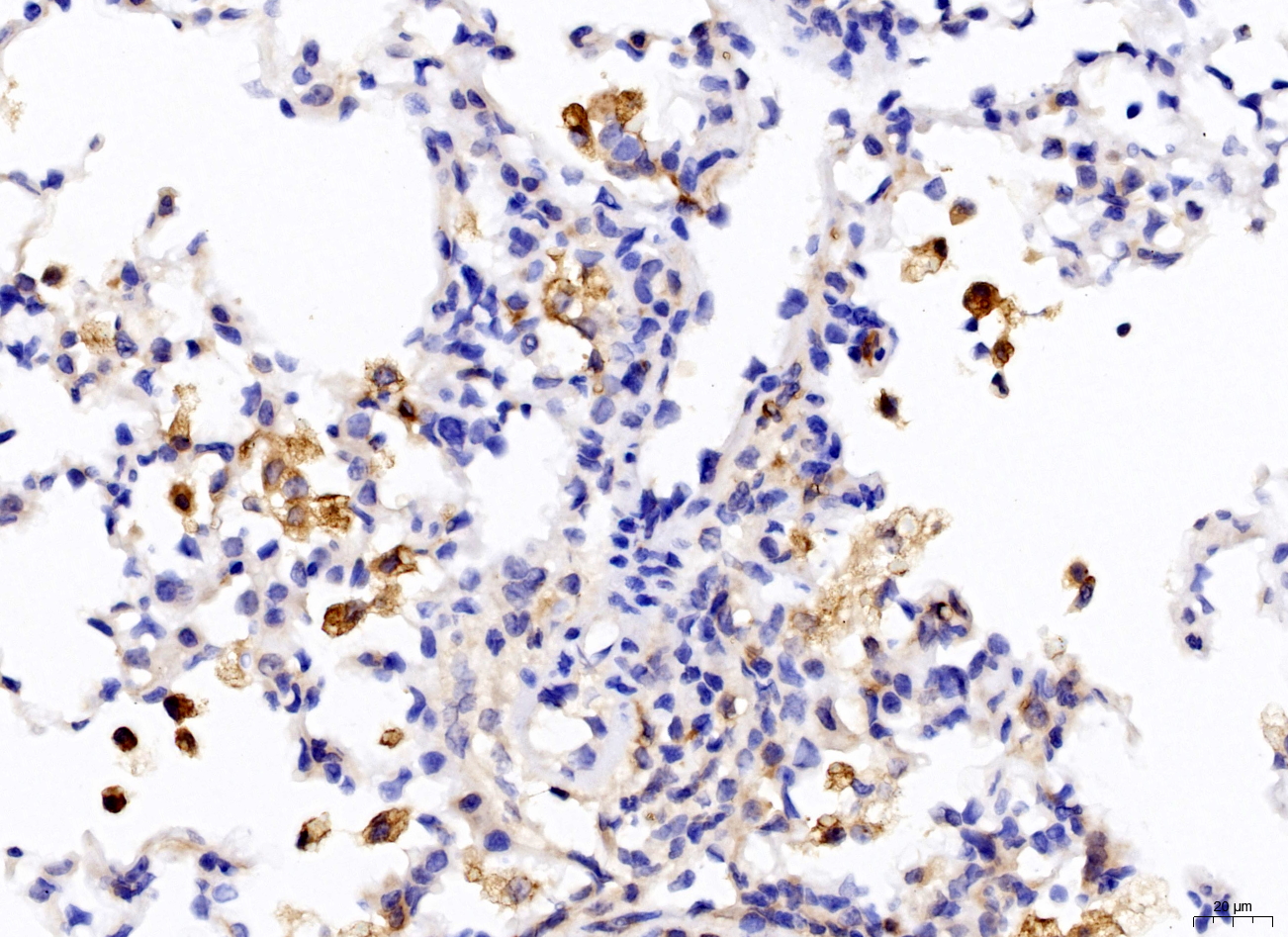

Supplement: Supplementary file 4 — Supporting File 4: advs73867‐sup‐0001‐FiguresData.zip. [file ADVS-13-e19191-s001.zip › Supporting information Figure1-10/Figure 2/Figure 2J/1 week Silica/SCRS-Ferritin-1w-Model-769_40.0x-6.jpg]

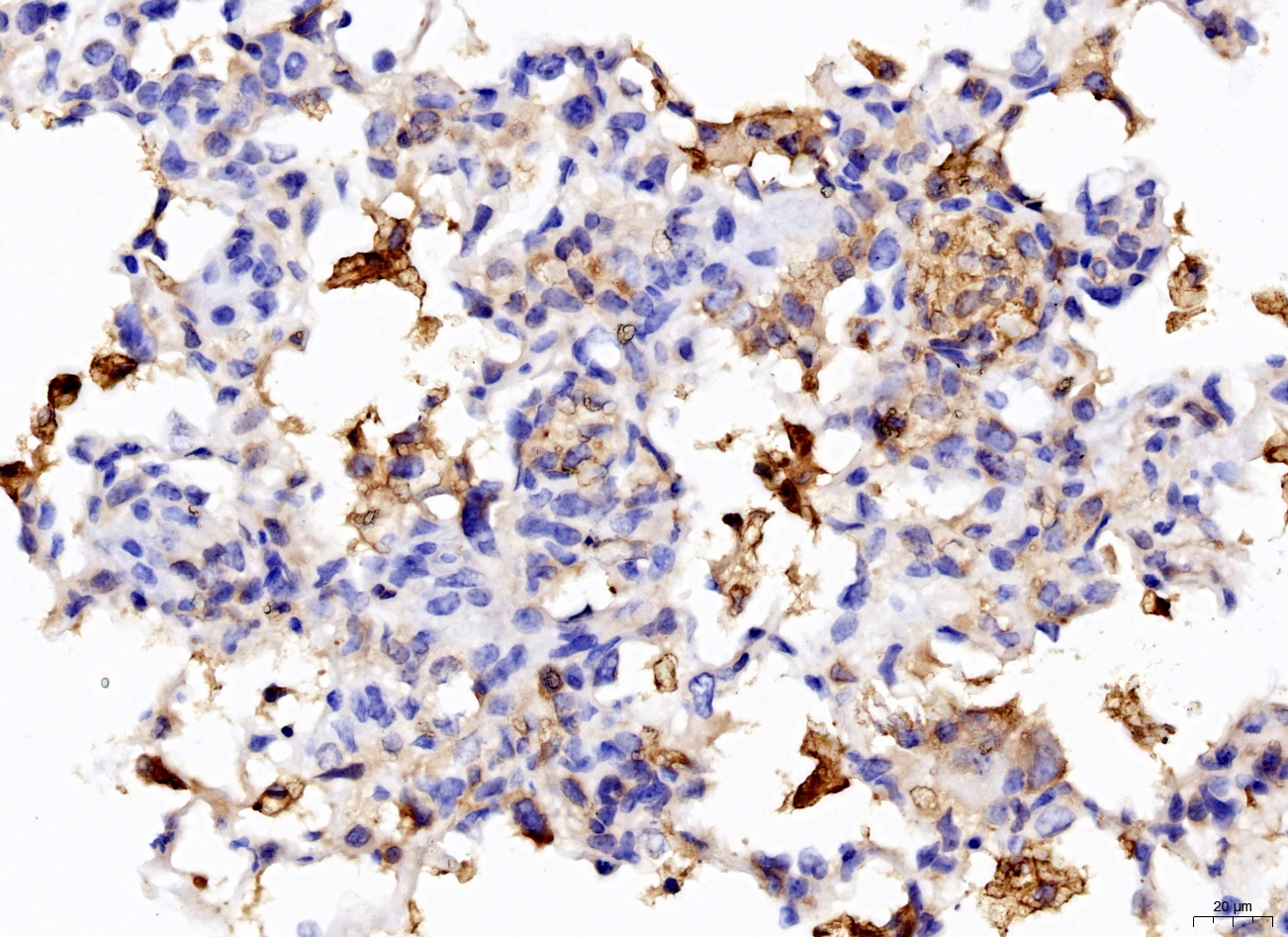

Supplement: Supplementary file 4 — Supporting File 4: advs73867‐sup‐0001‐FiguresData.zip. [file ADVS-13-e19191-s001.zip › Supporting information Figure1-10/Figure 2/Figure 2J/1 week Silica/SCRS-Ferritin-1w-Model-769_40.0x-7.jpg]

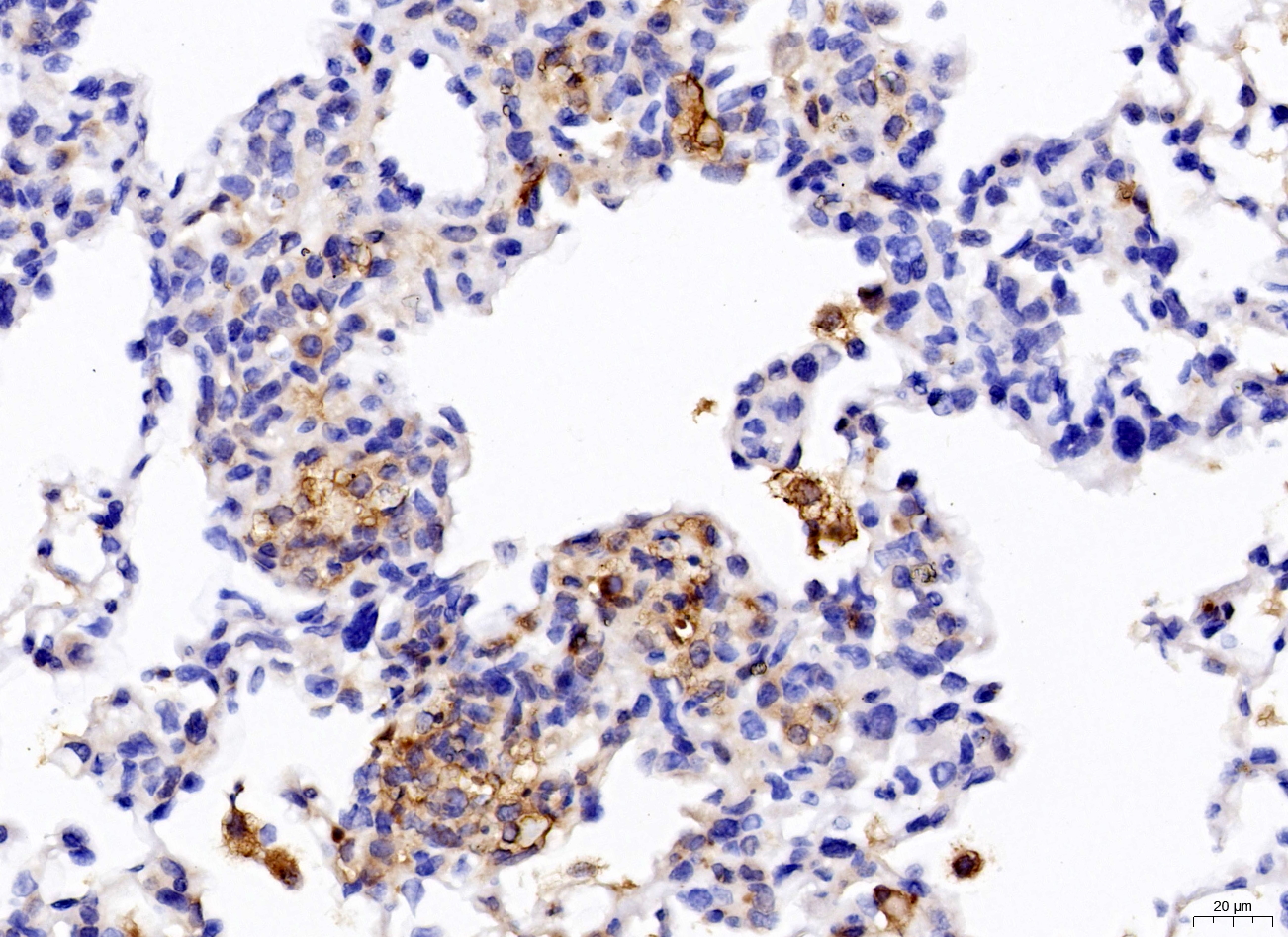

Supplement: Supplementary file 4 — Supporting File 4: advs73867‐sup‐0001‐FiguresData.zip. [file ADVS-13-e19191-s001.zip › Supporting information Figure1-10/Figure 2/Figure 2J/1 week Silica/SCRS-Ferritin-1w-Model-769_40.0x-8.jpg]

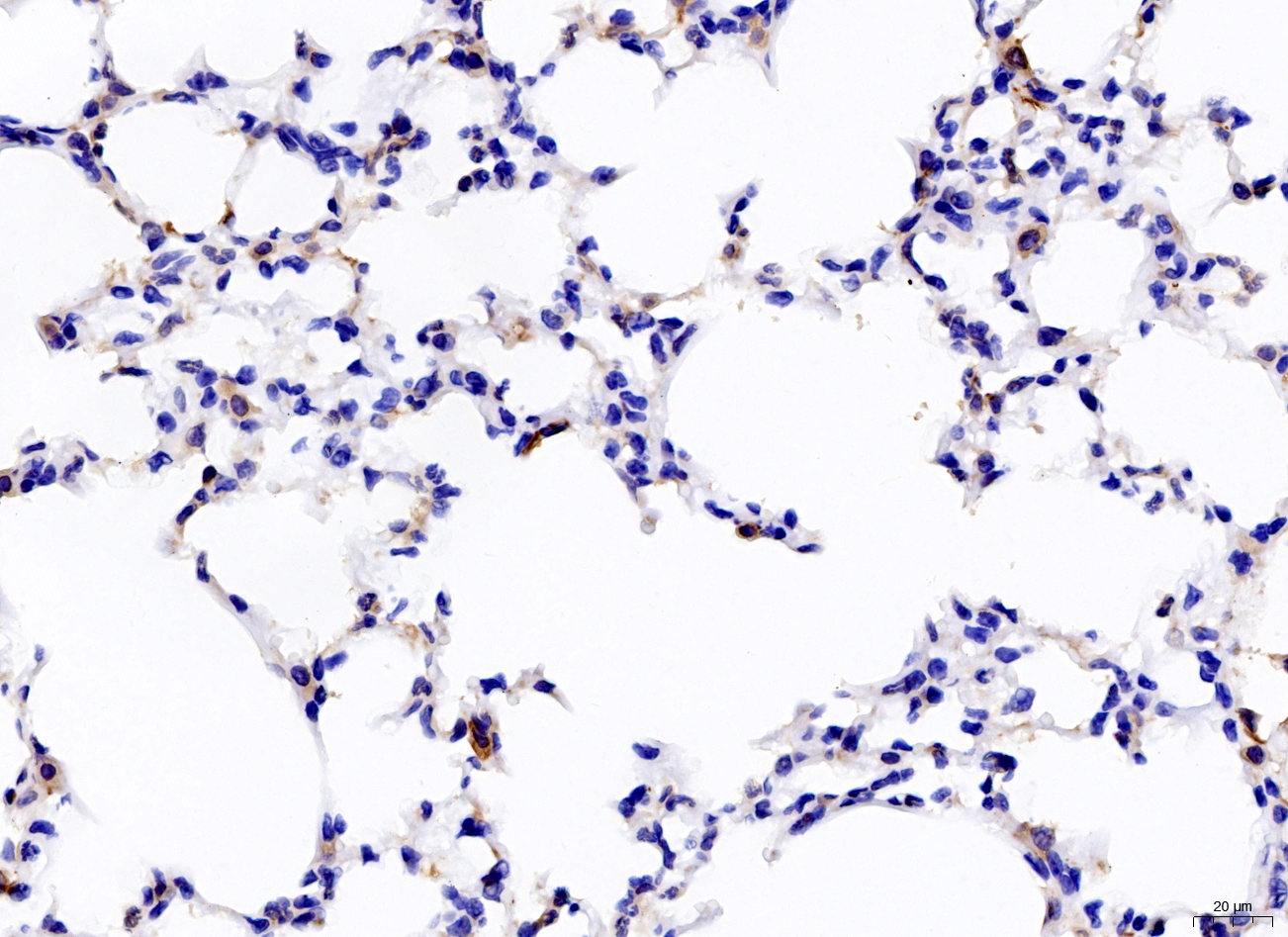

Supplement: Supplementary file 4 — Supporting File 4: advs73867‐sup‐0001‐FiguresData.zip. [file ADVS-13-e19191-s001.zip › Supporting information Figure1-10/Figure 2/Figure 2J/12 week Control/SCRS-Ferritin-12w-Control-781_40.0x-1.jpg]

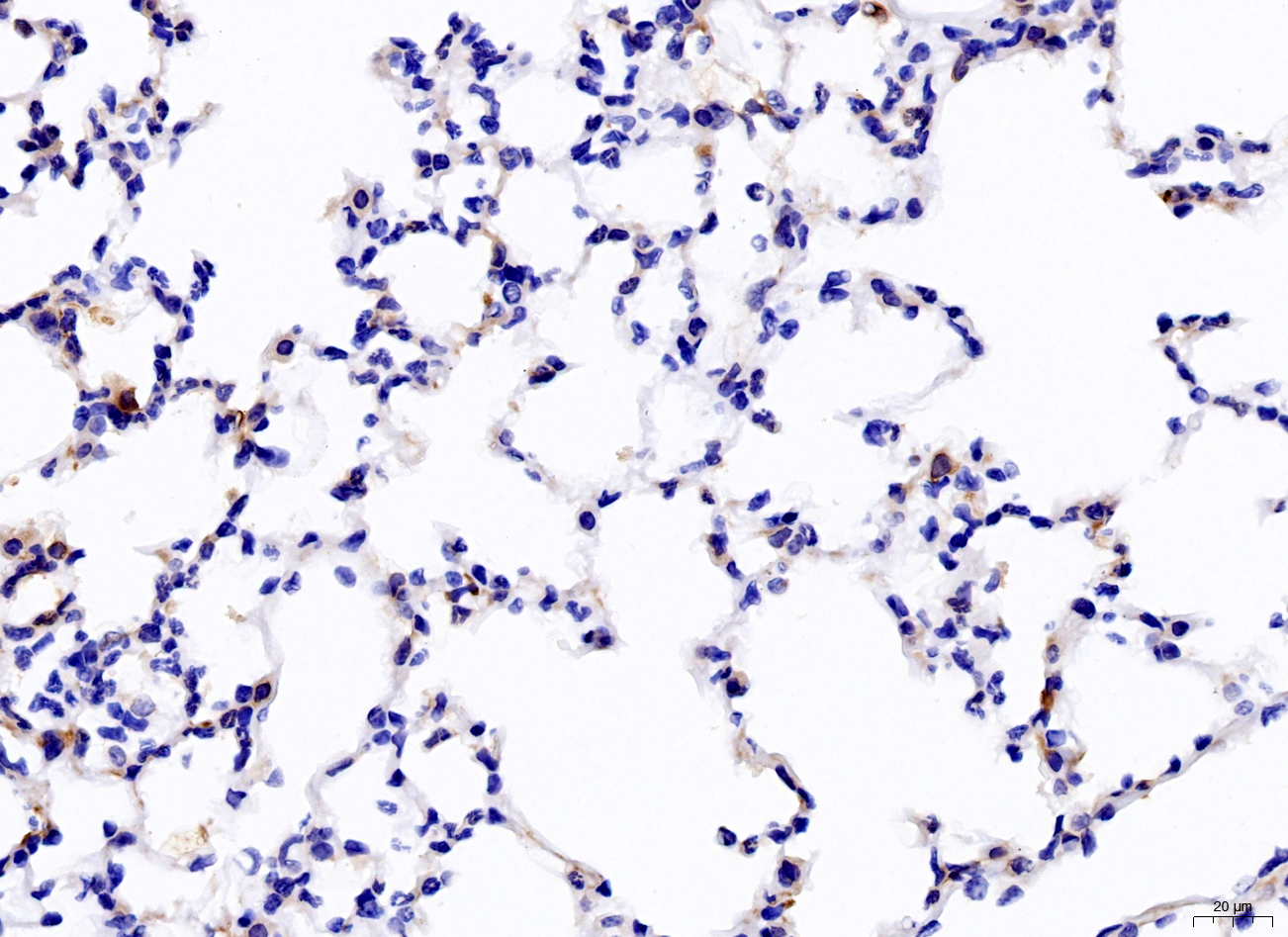

Supplement: Supplementary file 4 — Supporting File 4: advs73867‐sup‐0001‐FiguresData.zip. [file ADVS-13-e19191-s001.zip › Supporting information Figure1-10/Figure 2/Figure 2J/12 week Control/SCRS-Ferritin-12w-Control-781_40.0x-2.jpg]

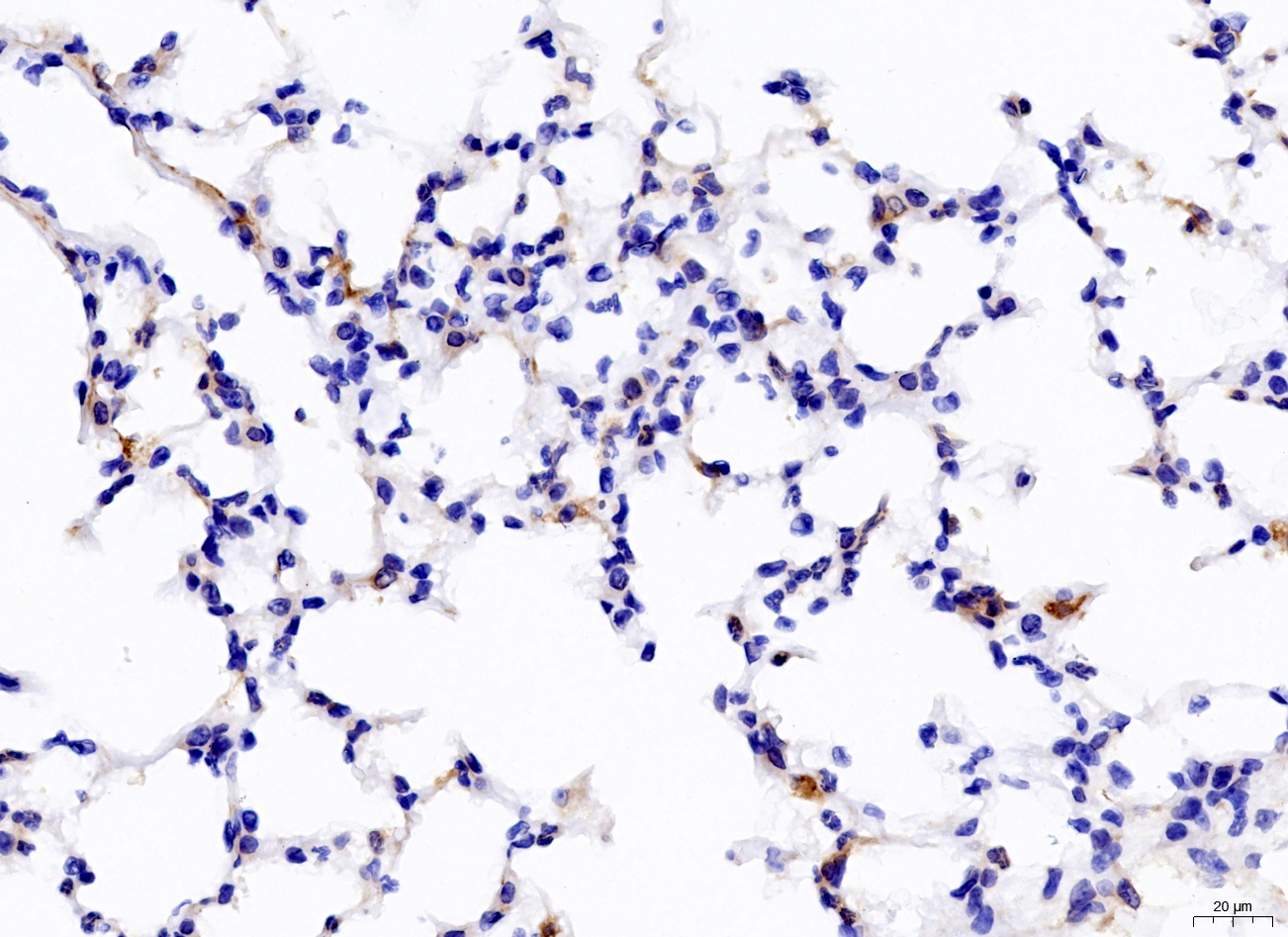

Supplement: Supplementary file 4 — Supporting File 4: advs73867‐sup‐0001‐FiguresData.zip. [file ADVS-13-e19191-s001.zip › Supporting information Figure1-10/Figure 2/Figure 2J/12 week Control/SCRS-Ferritin-12w-Control-781_40.0x-3.jpg]

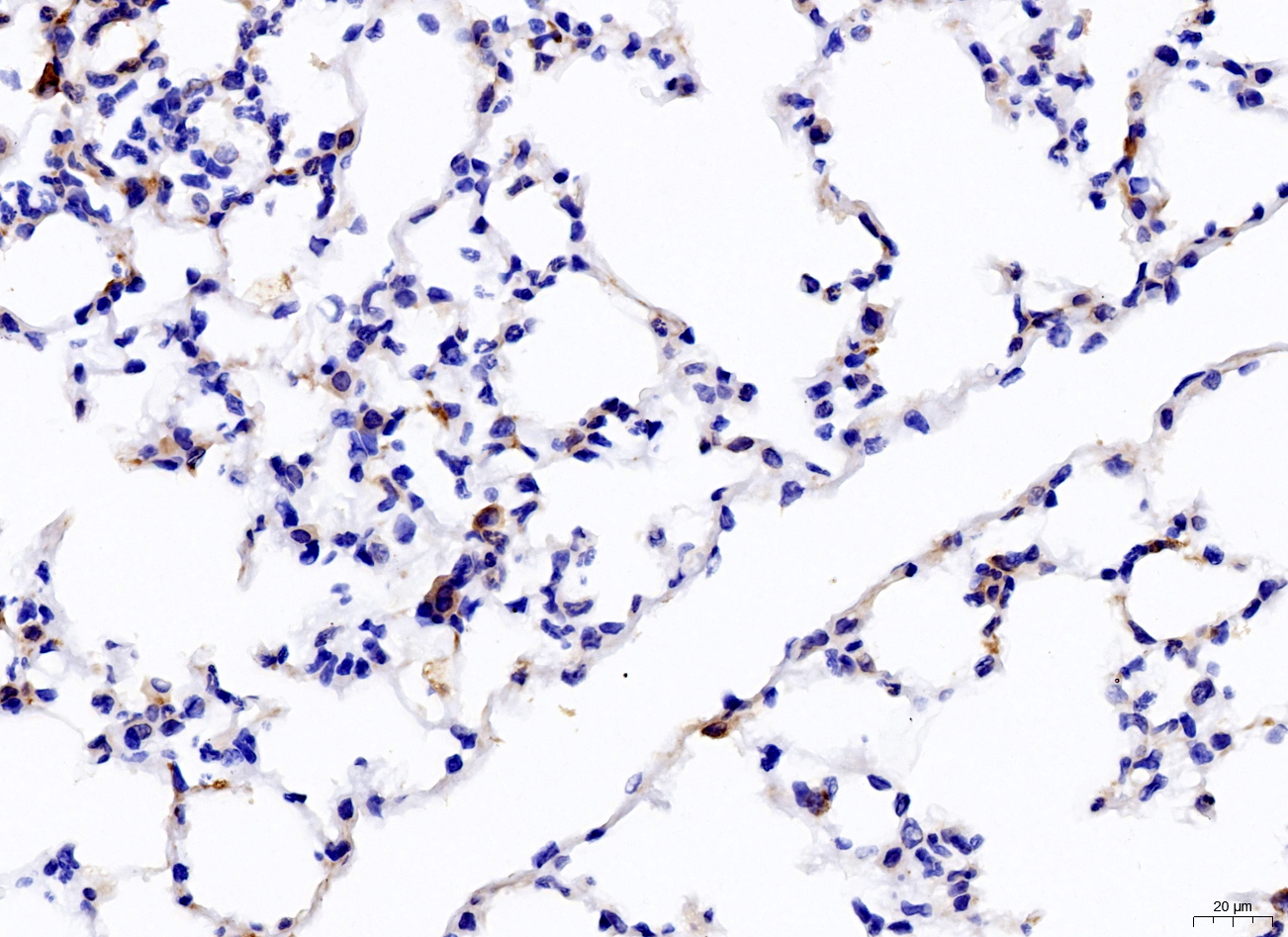

Supplement: Supplementary file 4 — Supporting File 4: advs73867‐sup‐0001‐FiguresData.zip. [file ADVS-13-e19191-s001.zip › Supporting information Figure1-10/Figure 2/Figure 2J/12 week Control/SCRS-Ferritin-12w-Control-781_40.0x-4.jpg]

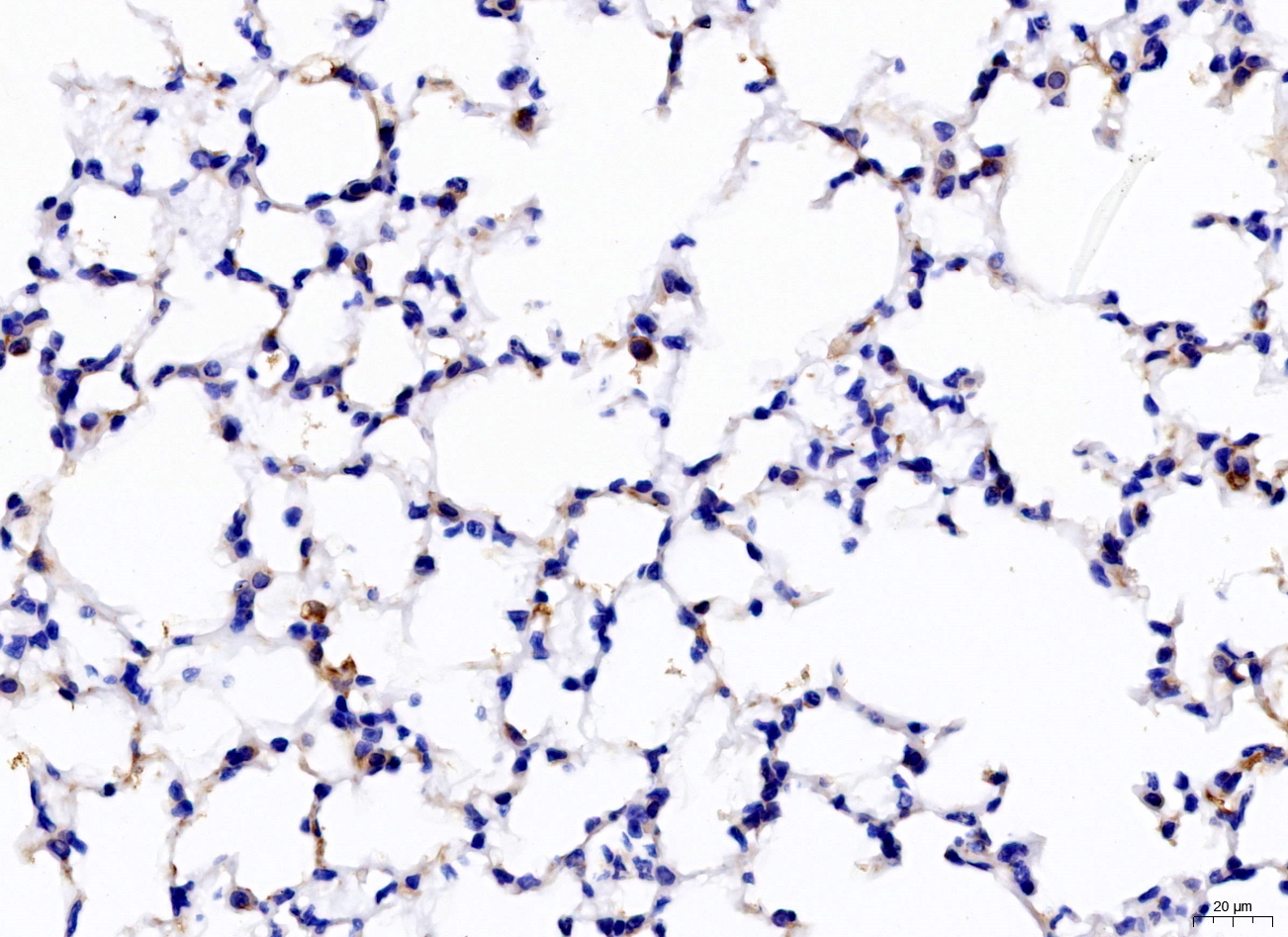

Supplement: Supplementary file 4 — Supporting File 4: advs73867‐sup‐0001‐FiguresData.zip. [file ADVS-13-e19191-s001.zip › Supporting information Figure1-10/Figure 2/Figure 2J/12 week Control/SCRS-Ferritin-12w-Control-788_40.0x-6.jpg]

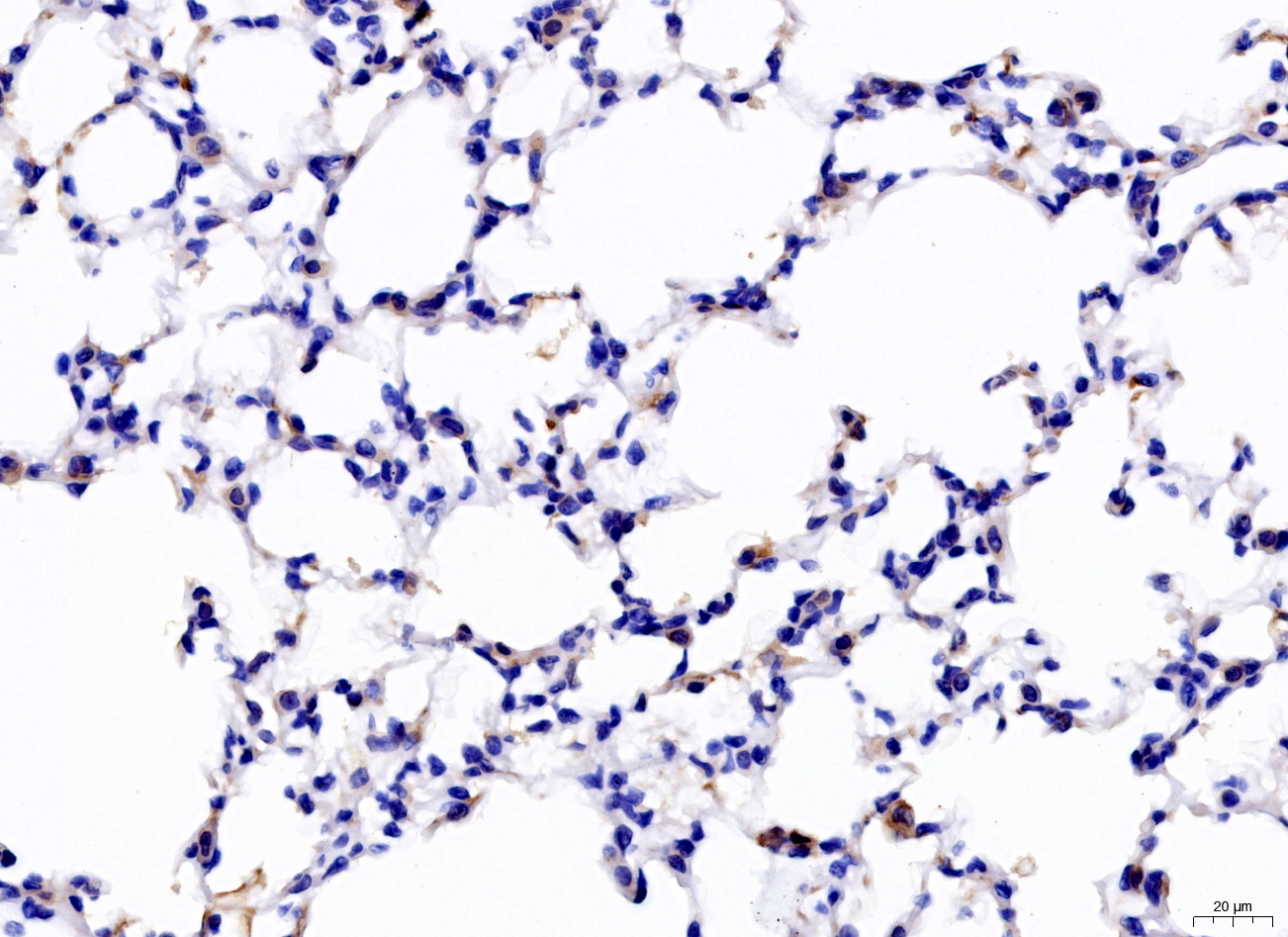

Supplement: Supplementary file 4 — Supporting File 4: advs73867‐sup‐0001‐FiguresData.zip. [file ADVS-13-e19191-s001.zip › Supporting information Figure1-10/Figure 2/Figure 2J/12 week Control/SCRS-Ferritin-12w-Control-788_40.0x-7.jpg]

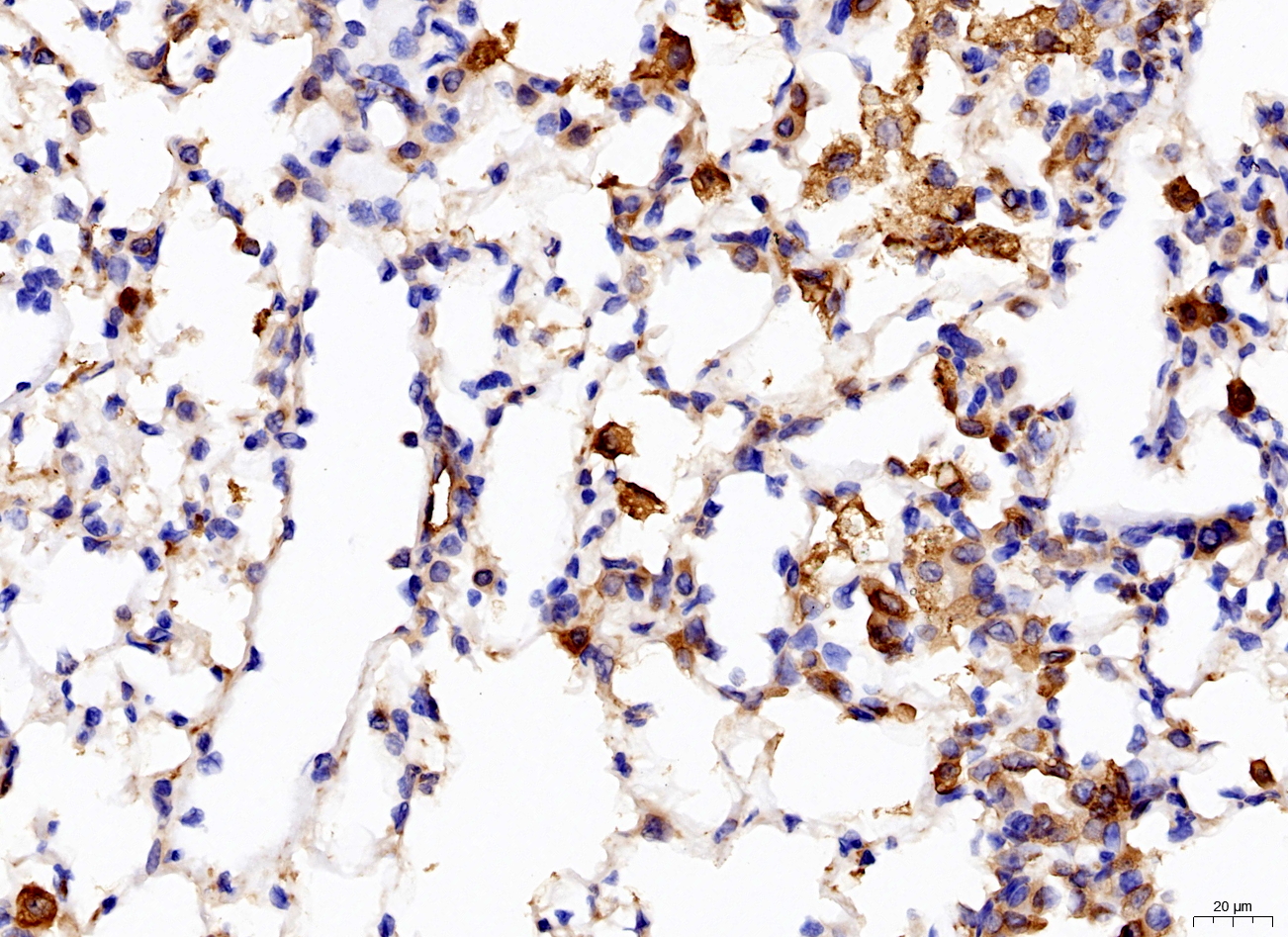

Supplement: Supplementary file 4 — Supporting File 4: advs73867‐sup‐0001‐FiguresData.zip. [file ADVS-13-e19191-s001.zip › Supporting information Figure1-10/Figure 2/Figure 2J/12 week Silica/SCRS-Ferritin-12w-Model-765_40.0x-1.jpg]

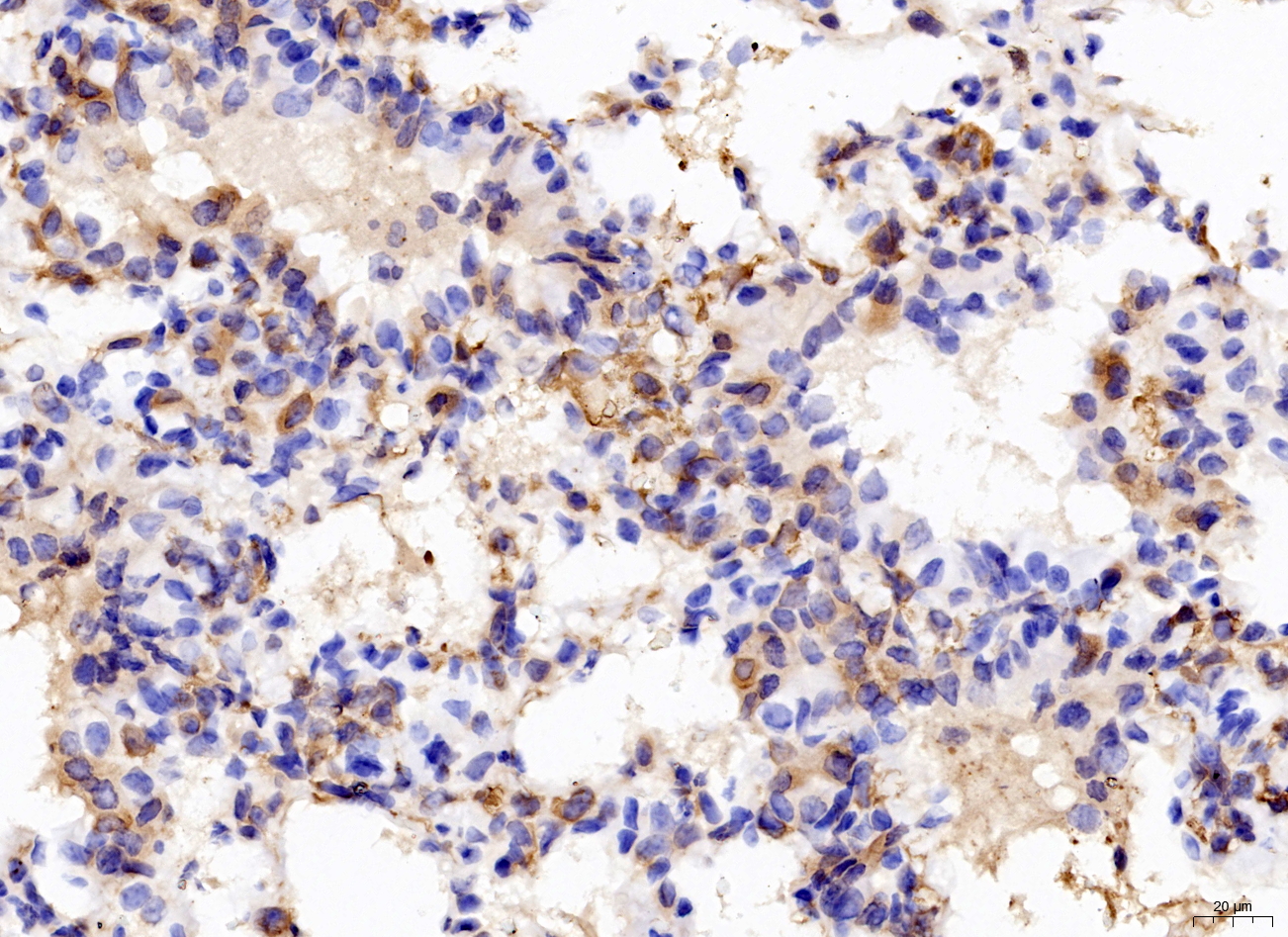

Supplement: Supplementary file 4 — Supporting File 4: advs73867‐sup‐0001‐FiguresData.zip. [file ADVS-13-e19191-s001.zip › Supporting information Figure1-10/Figure 2/Figure 2J/12 week Silica/SCRS-Ferritin-12w-Model-765_40.0x-2.jpg]

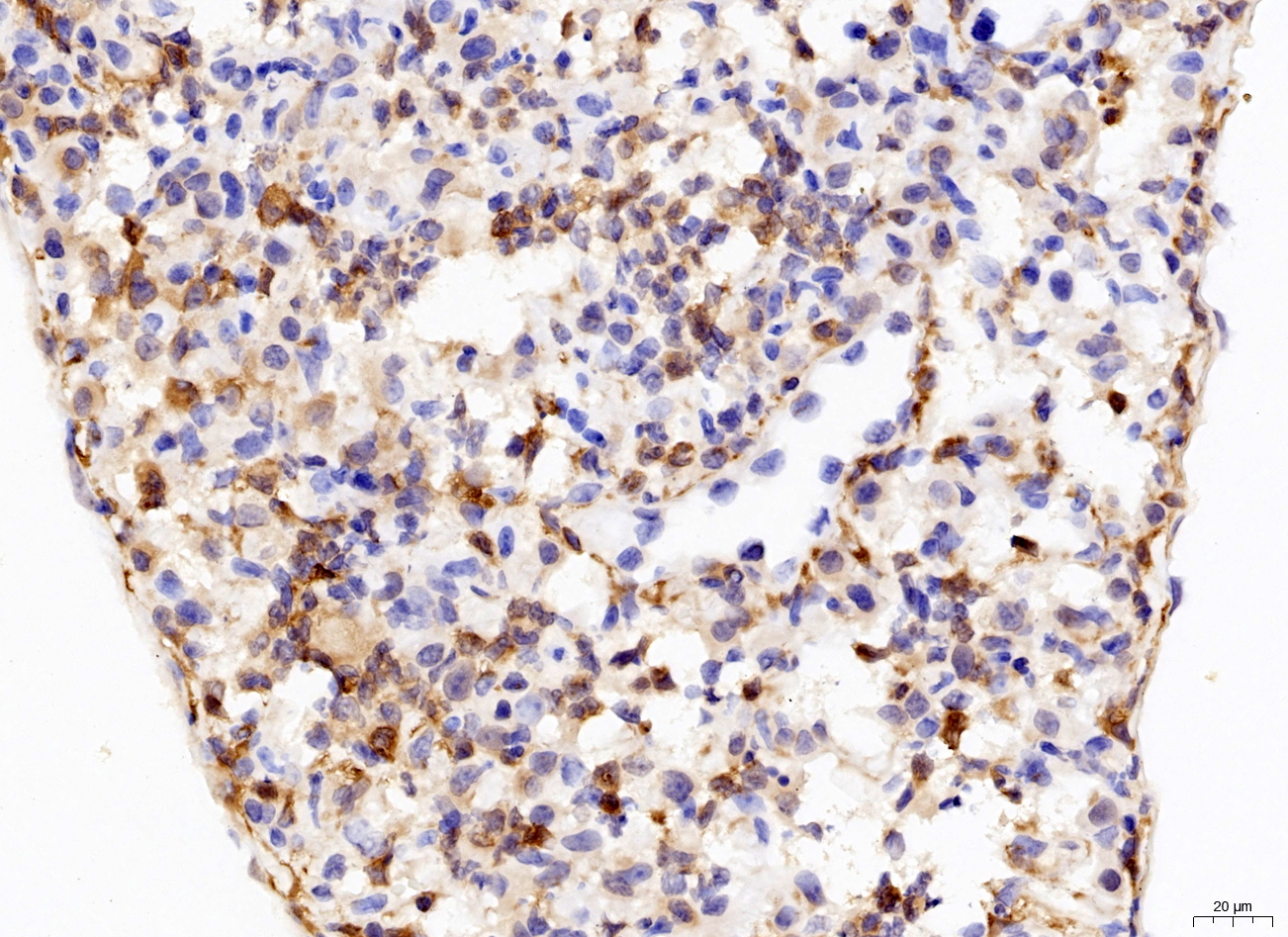

Supplement: Supplementary file 4 — Supporting File 4: advs73867‐sup‐0001‐FiguresData.zip. [file ADVS-13-e19191-s001.zip › Supporting information Figure1-10/Figure 2/Figure 2J/12 week Silica/SCRS-Ferritin-12w-Model-765_40.0x-3.jpg]

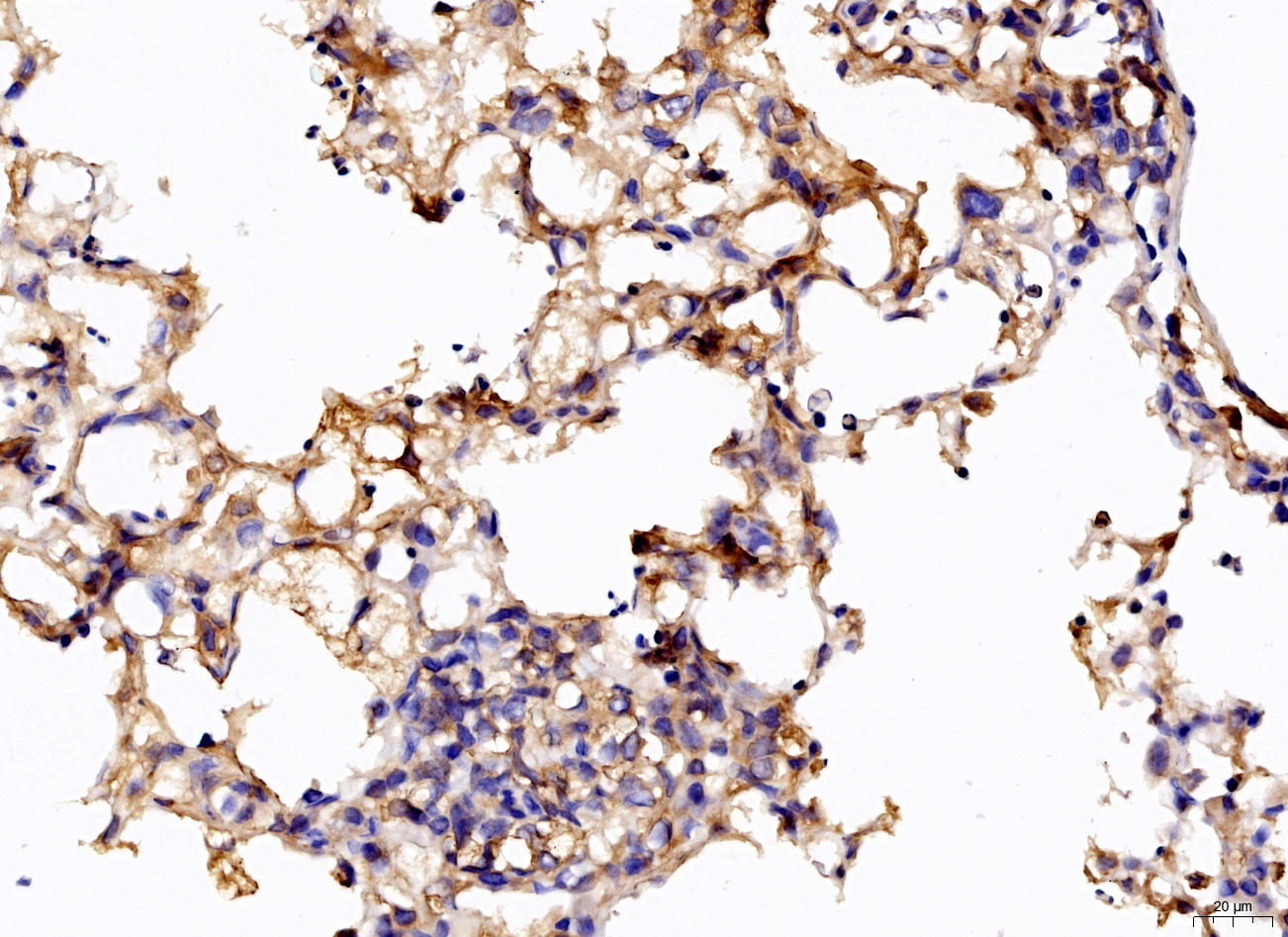

Supplement: Supplementary file 4 — Supporting File 4: advs73867‐sup‐0001‐FiguresData.zip. [file ADVS-13-e19191-s001.zip › Supporting information Figure1-10/Figure 2/Figure 2J/12 week Silica/SCRS-Ferritin-12w-Model-767_40.0x-4.jpg]

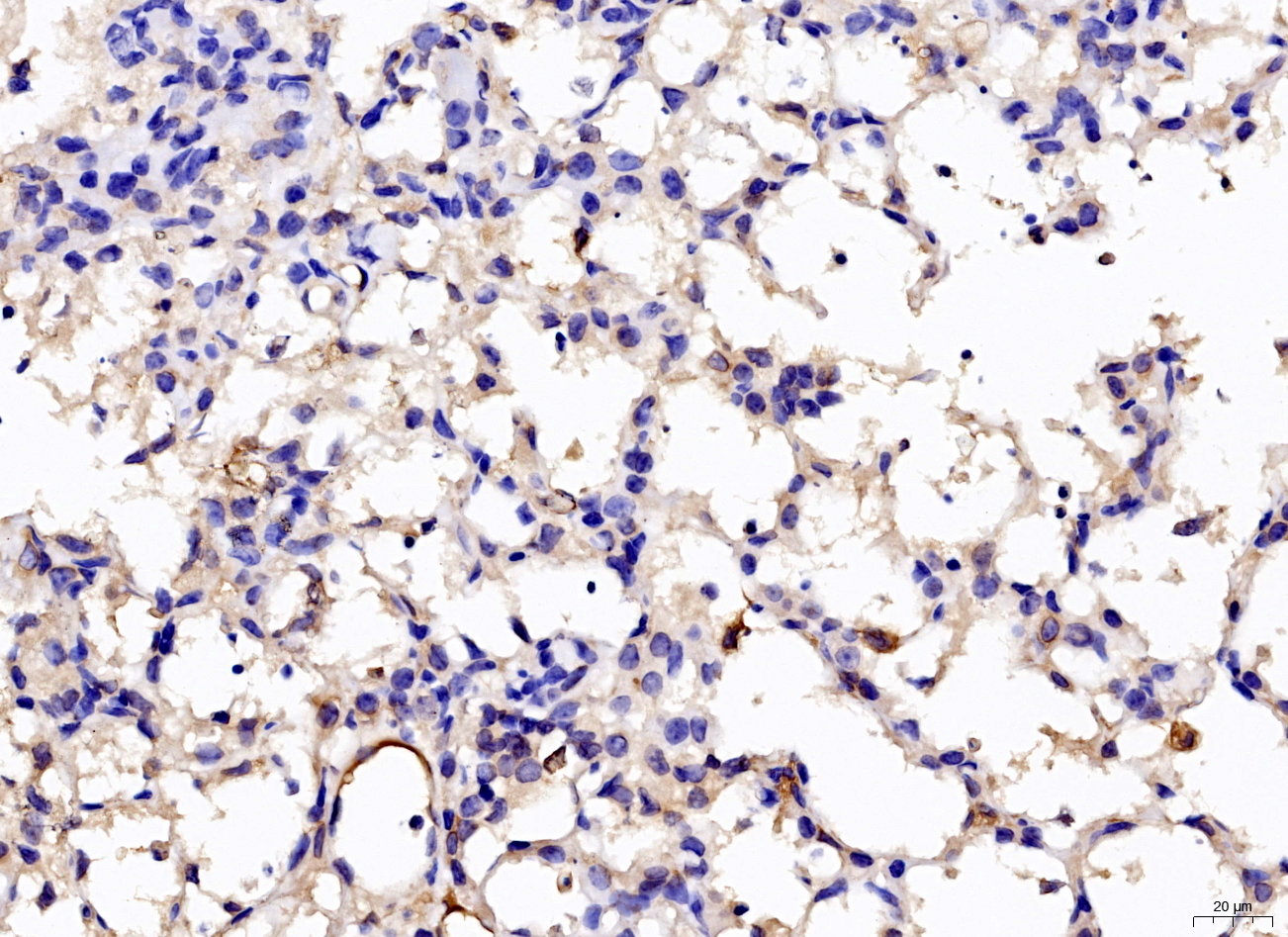

Supplement: Supplementary file 4 — Supporting File 4: advs73867‐sup‐0001‐FiguresData.zip. [file ADVS-13-e19191-s001.zip › Supporting information Figure1-10/Figure 2/Figure 2J/12 week Silica/SCRS-Ferritin-12w-Model-767_40.0x-5.jpg]

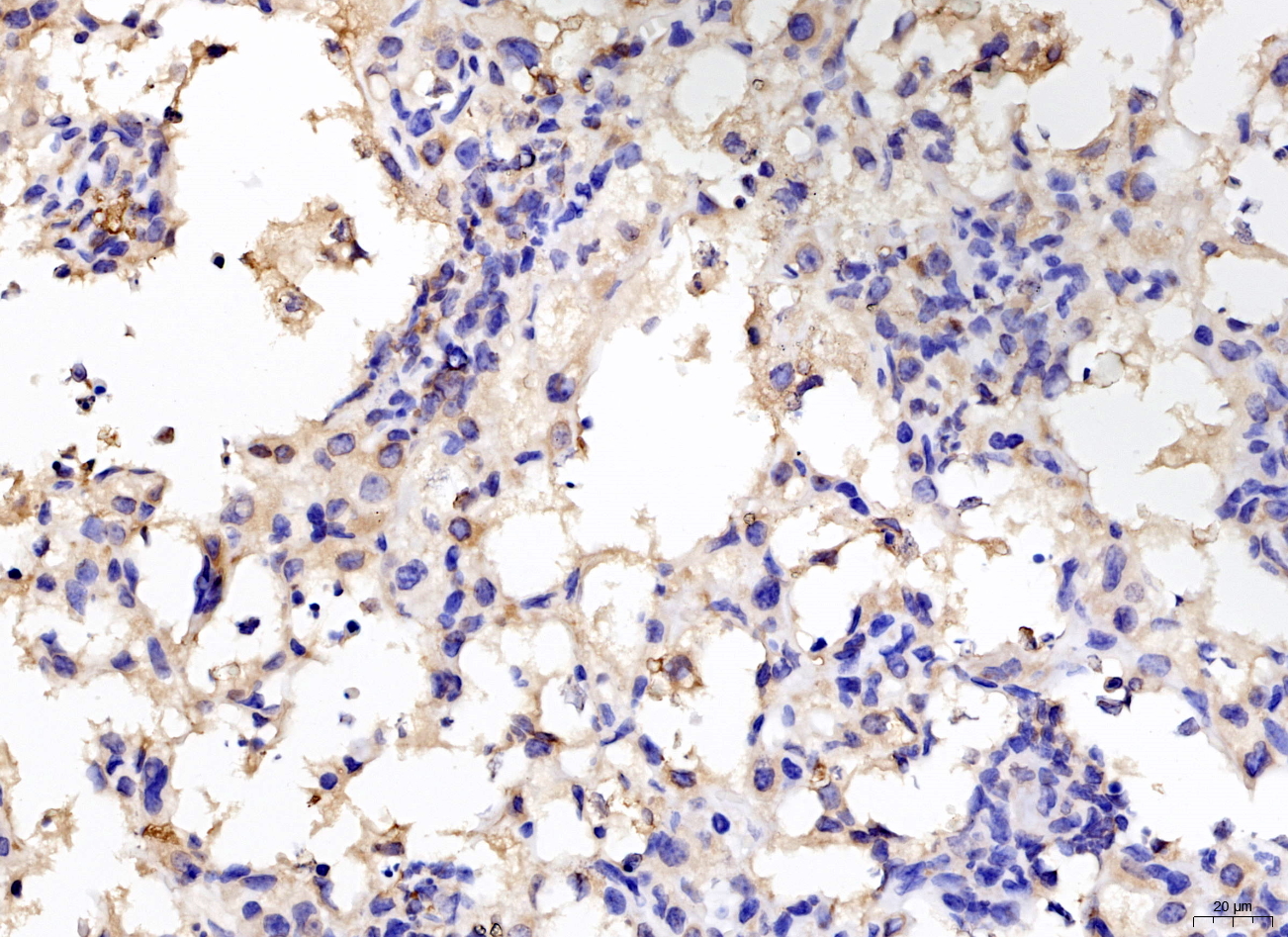

Supplement: Supplementary file 4 — Supporting File 4: advs73867‐sup‐0001‐FiguresData.zip. [file ADVS-13-e19191-s001.zip › Supporting information Figure1-10/Figure 2/Figure 2J/12 week Silica/SCRS-Ferritin-12w-Model-767_40.0x-6.jpg]

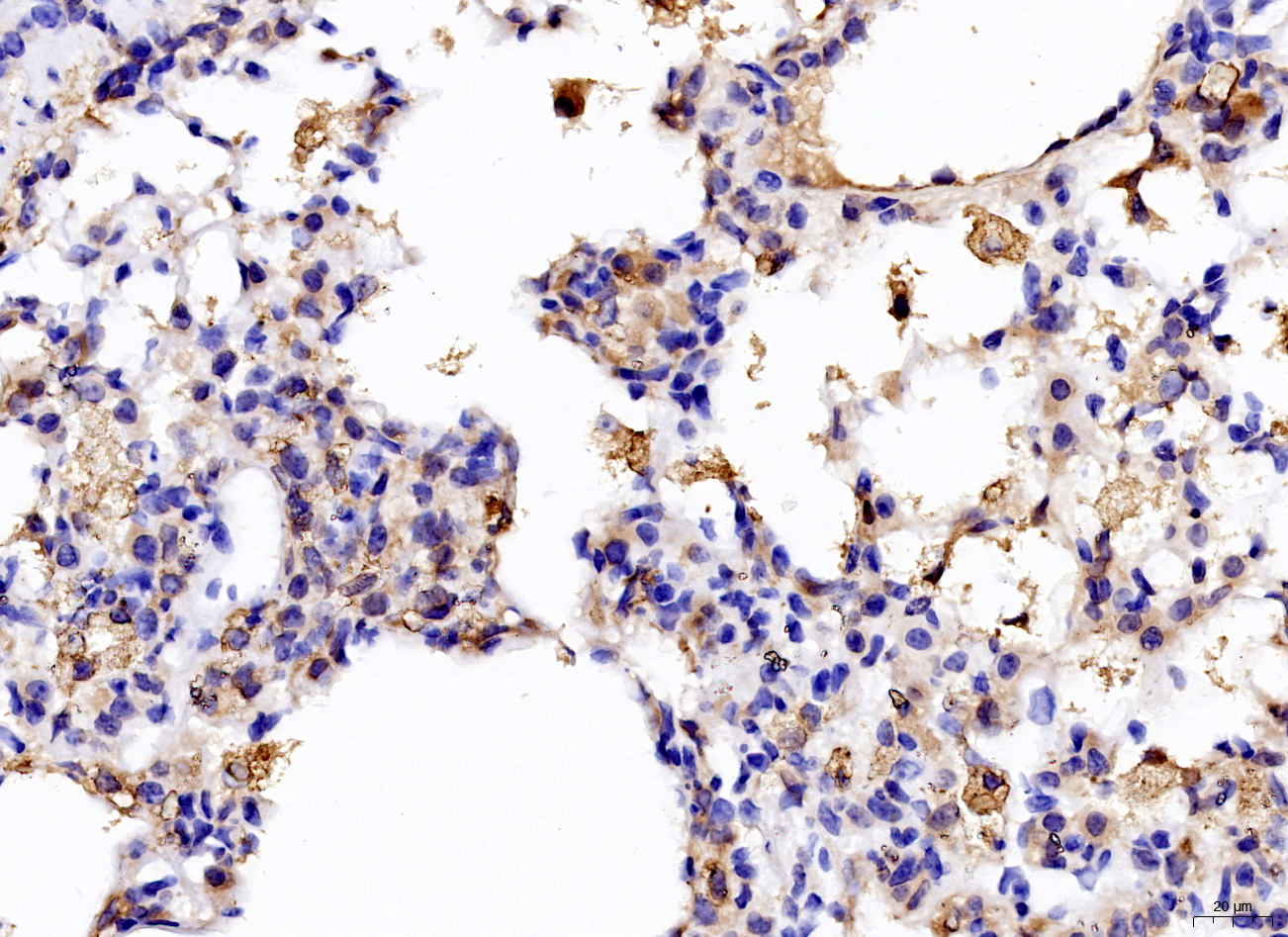

Supplement: Supplementary file 4 — Supporting File 4: advs73867‐sup‐0001‐FiguresData.zip. [file ADVS-13-e19191-s001.zip › Supporting information Figure1-10/Figure 2/Figure 2J/12 week Silica/SCRS-Ferritin-12w-Model-768_40.0x-7.jpg]

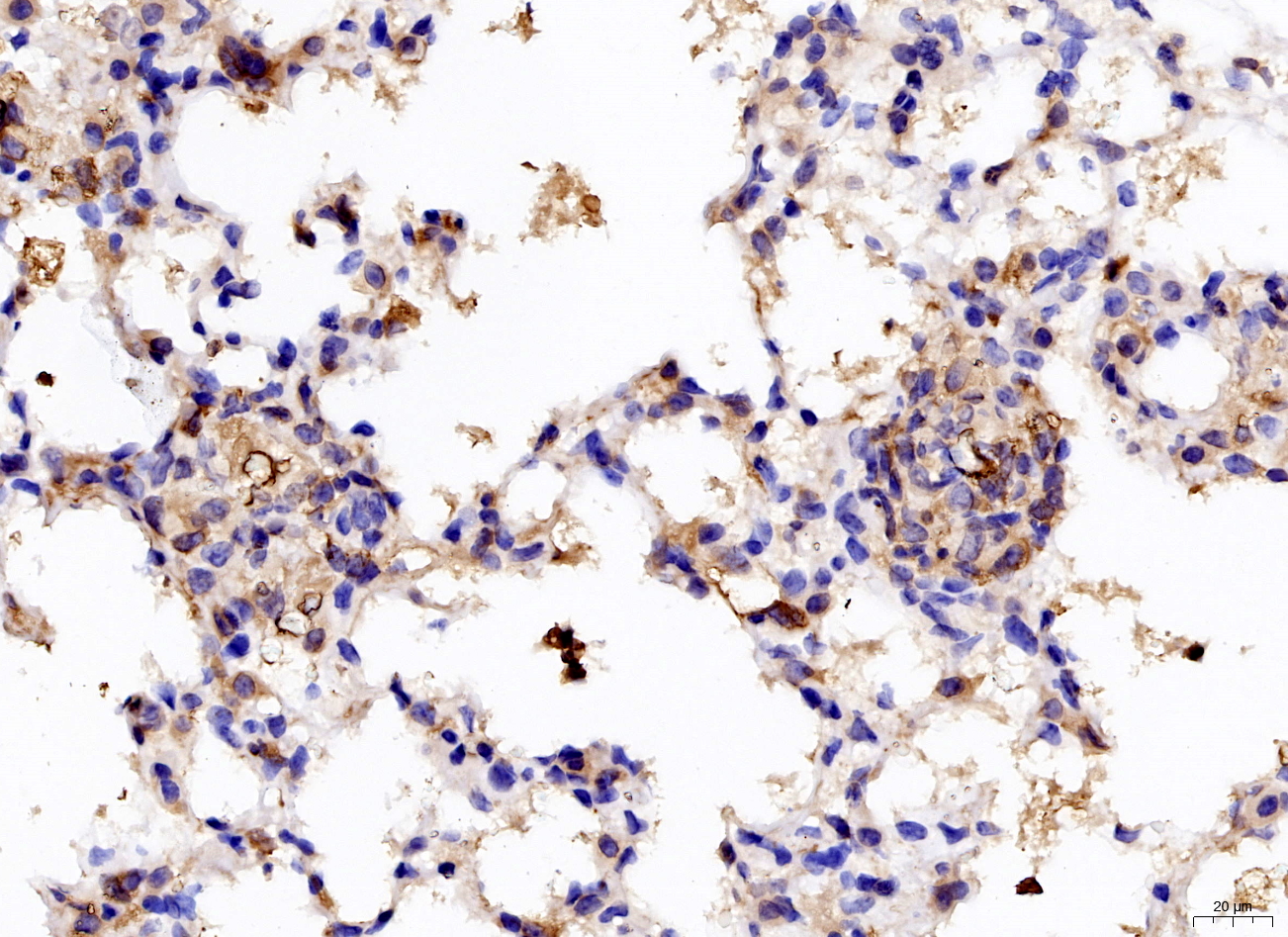

Supplement: Supplementary file 4 — Supporting File 4: advs73867‐sup‐0001‐FiguresData.zip. [file ADVS-13-e19191-s001.zip › Supporting information Figure1-10/Figure 2/Figure 2J/12 week Silica/SCRS-Ferritin-12w-Model-768_40.0x-8.jpg]

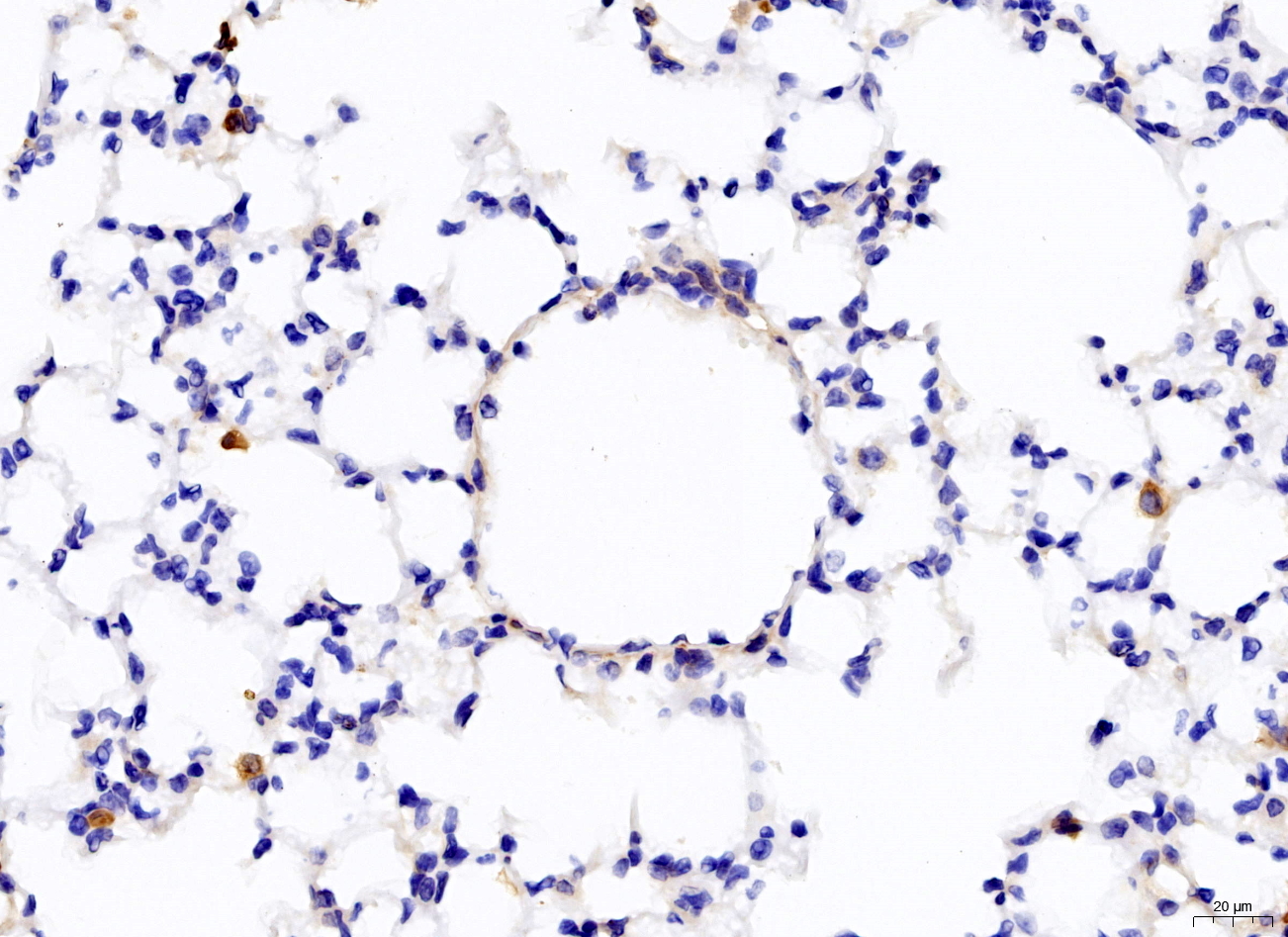

Supplement: Supplementary file 4 — Supporting File 4: advs73867‐sup‐0001‐FiguresData.zip. [file ADVS-13-e19191-s001.zip › Supporting information Figure1-10/Figure 2/Figure 2J/4 week Control/SCRS-Ferritin-4w-Control-774_40.0x-1.jpg]

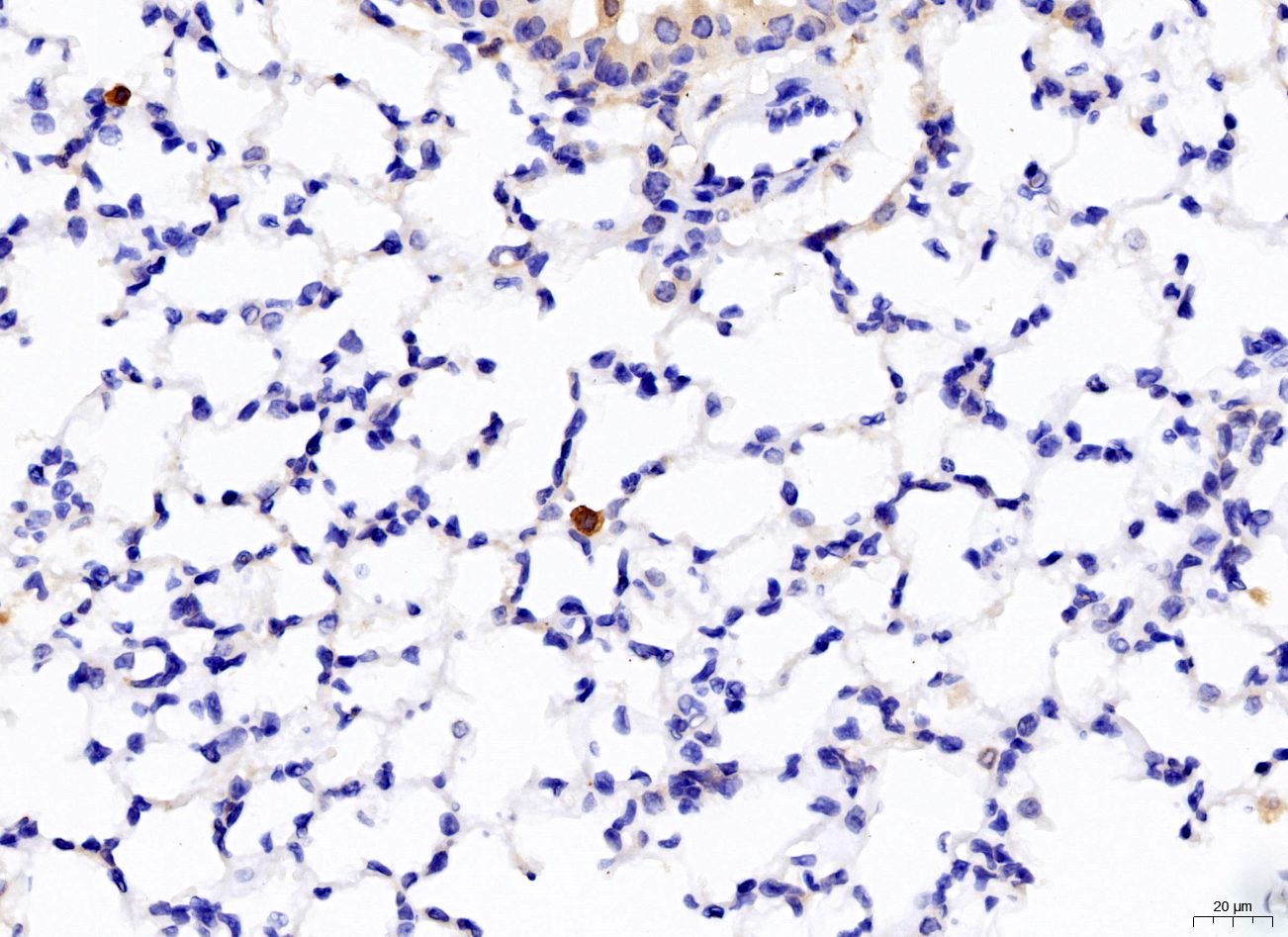

Supplement: Supplementary file 4 — Supporting File 4: advs73867‐sup‐0001‐FiguresData.zip. [file ADVS-13-e19191-s001.zip › Supporting information Figure1-10/Figure 2/Figure 2J/4 week Control/SCRS-Ferritin-4w-Control-774_40.0x-2.jpg]

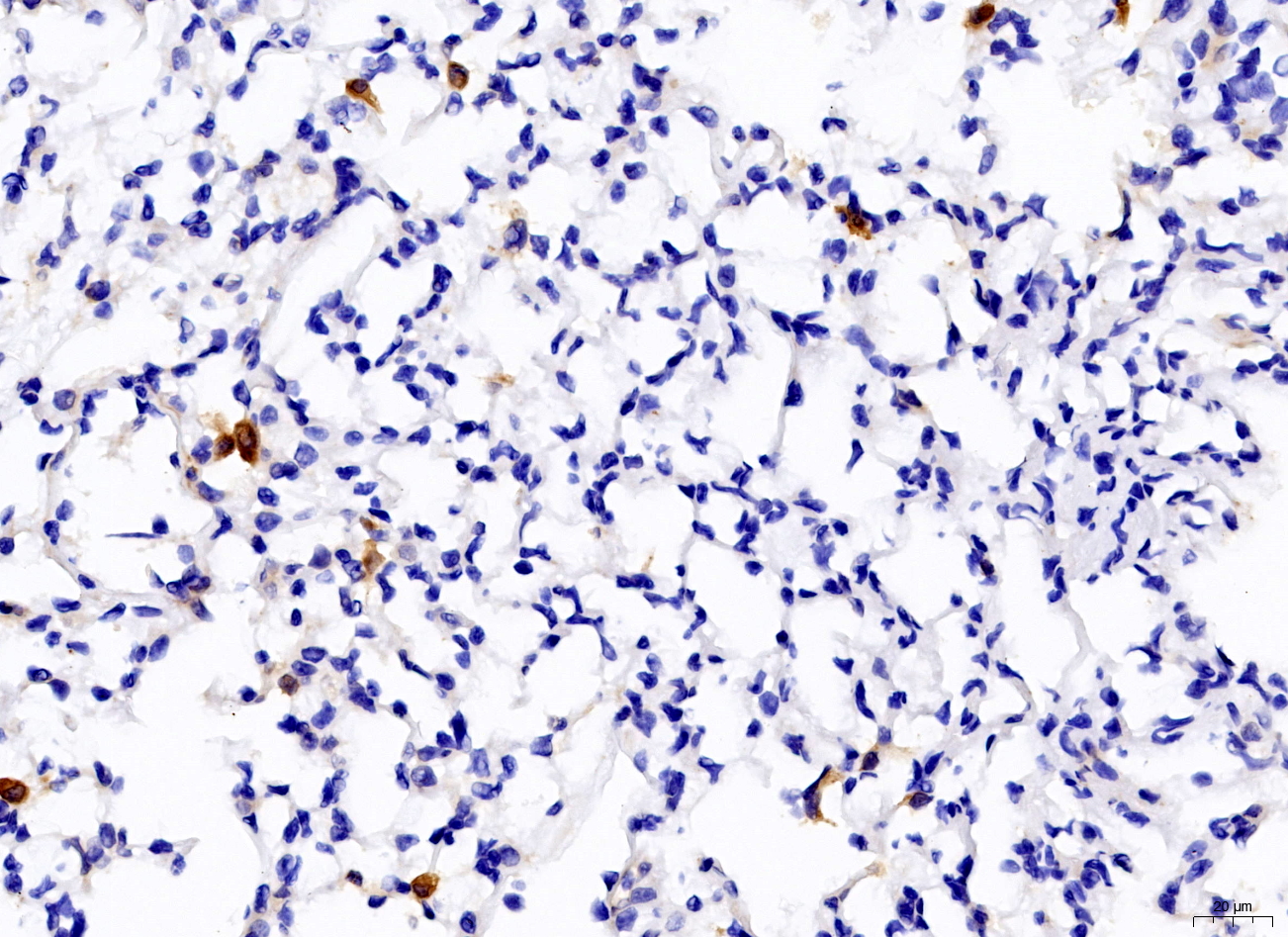

Supplement: Supplementary file 4 — Supporting File 4: advs73867‐sup‐0001‐FiguresData.zip. [file ADVS-13-e19191-s001.zip › Supporting information Figure1-10/Figure 2/Figure 2J/4 week Control/SCRS-Ferritin-4w-Control-774_40.0x-3.jpg]

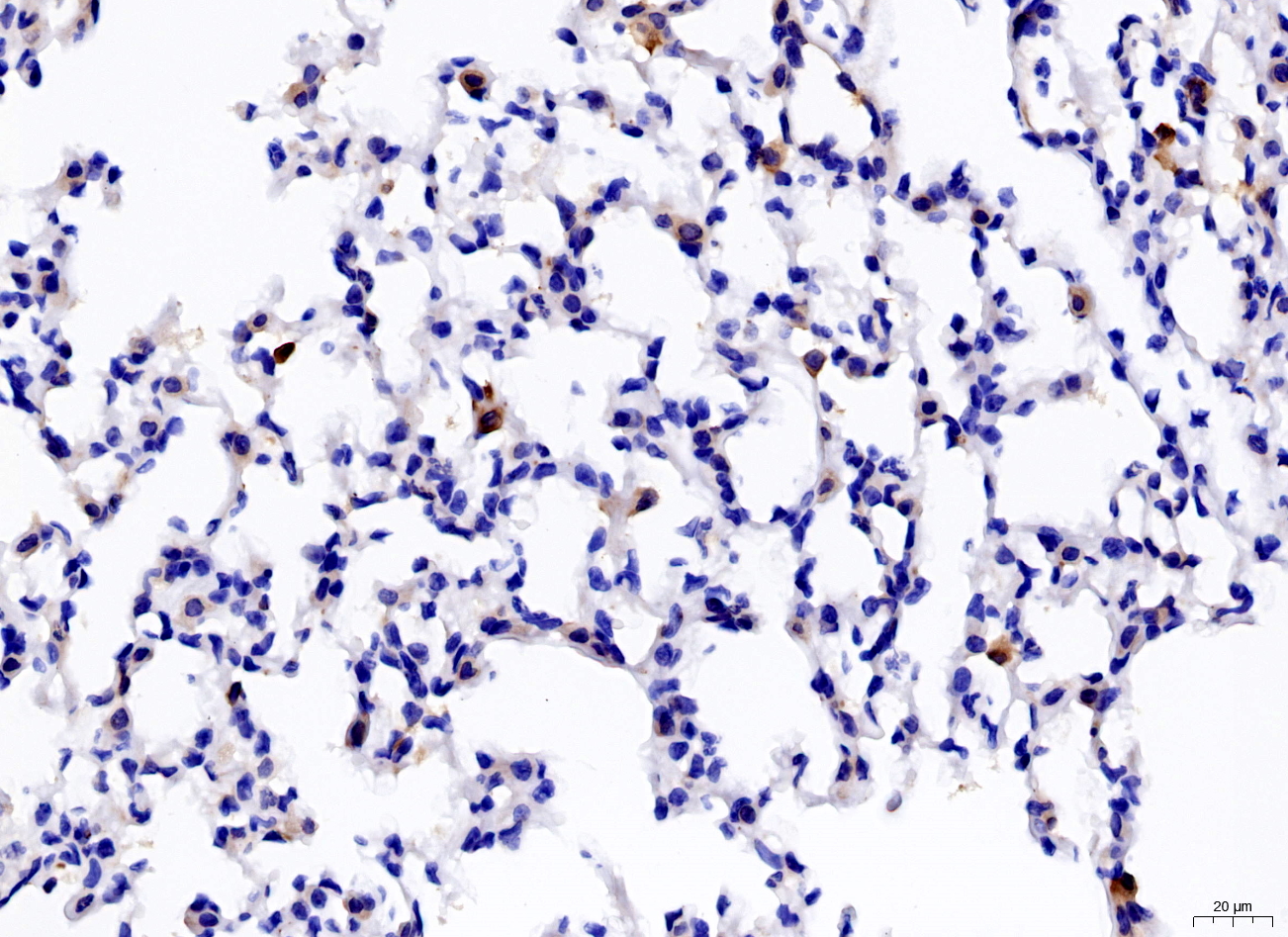

Supplement: Supplementary file 4 — Supporting File 4: advs73867‐sup‐0001‐FiguresData.zip. [file ADVS-13-e19191-s001.zip › Supporting information Figure1-10/Figure 2/Figure 2J/4 week Control/SCRS-Ferritin-4w-Control-777_40.0x-4.jpg]

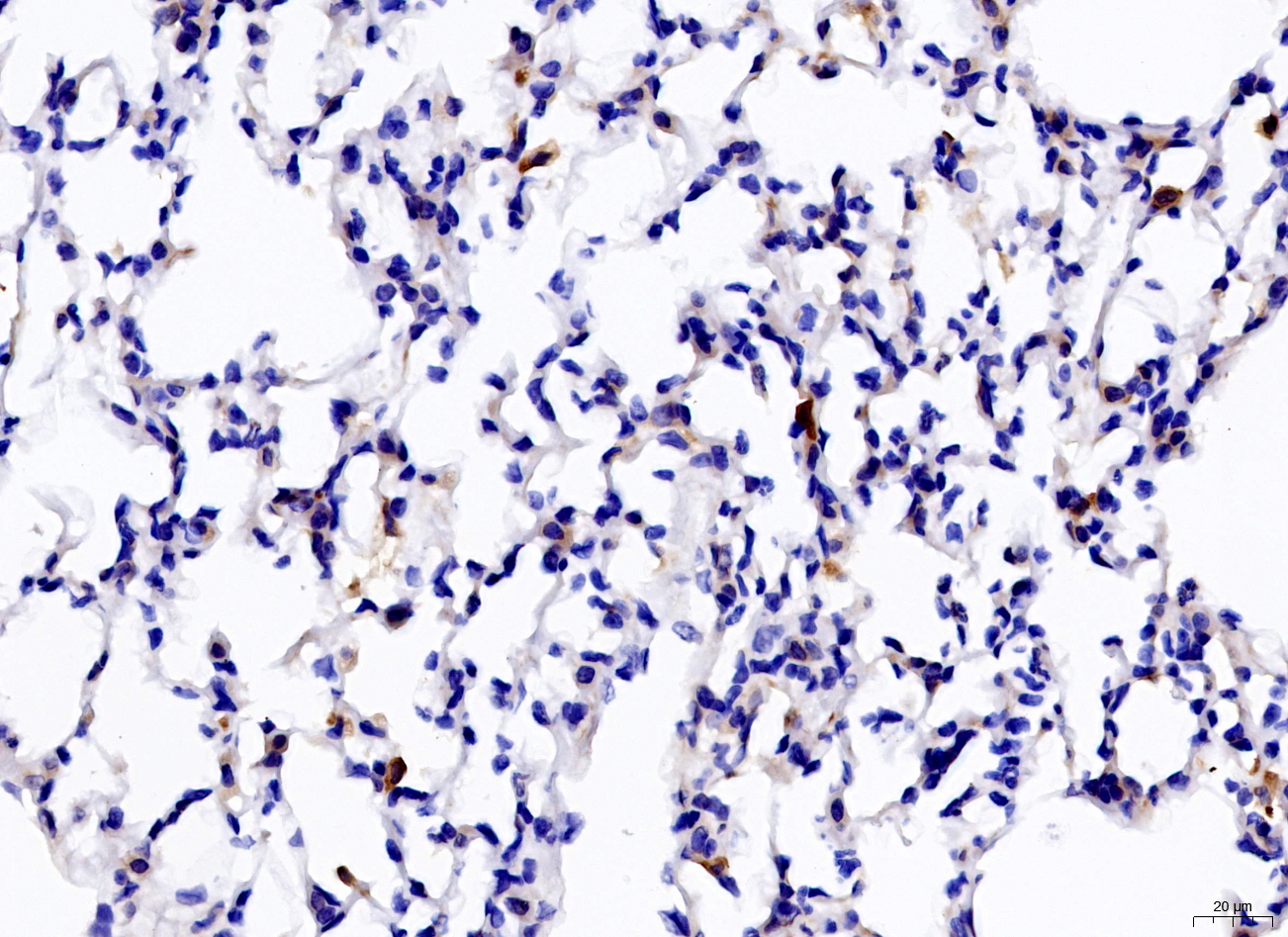

Supplement: Supplementary file 4 — Supporting File 4: advs73867‐sup‐0001‐FiguresData.zip. [file ADVS-13-e19191-s001.zip › Supporting information Figure1-10/Figure 2/Figure 2J/4 week Control/SCRS-Ferritin-4w-Control-777_40.0x-5.jpg]

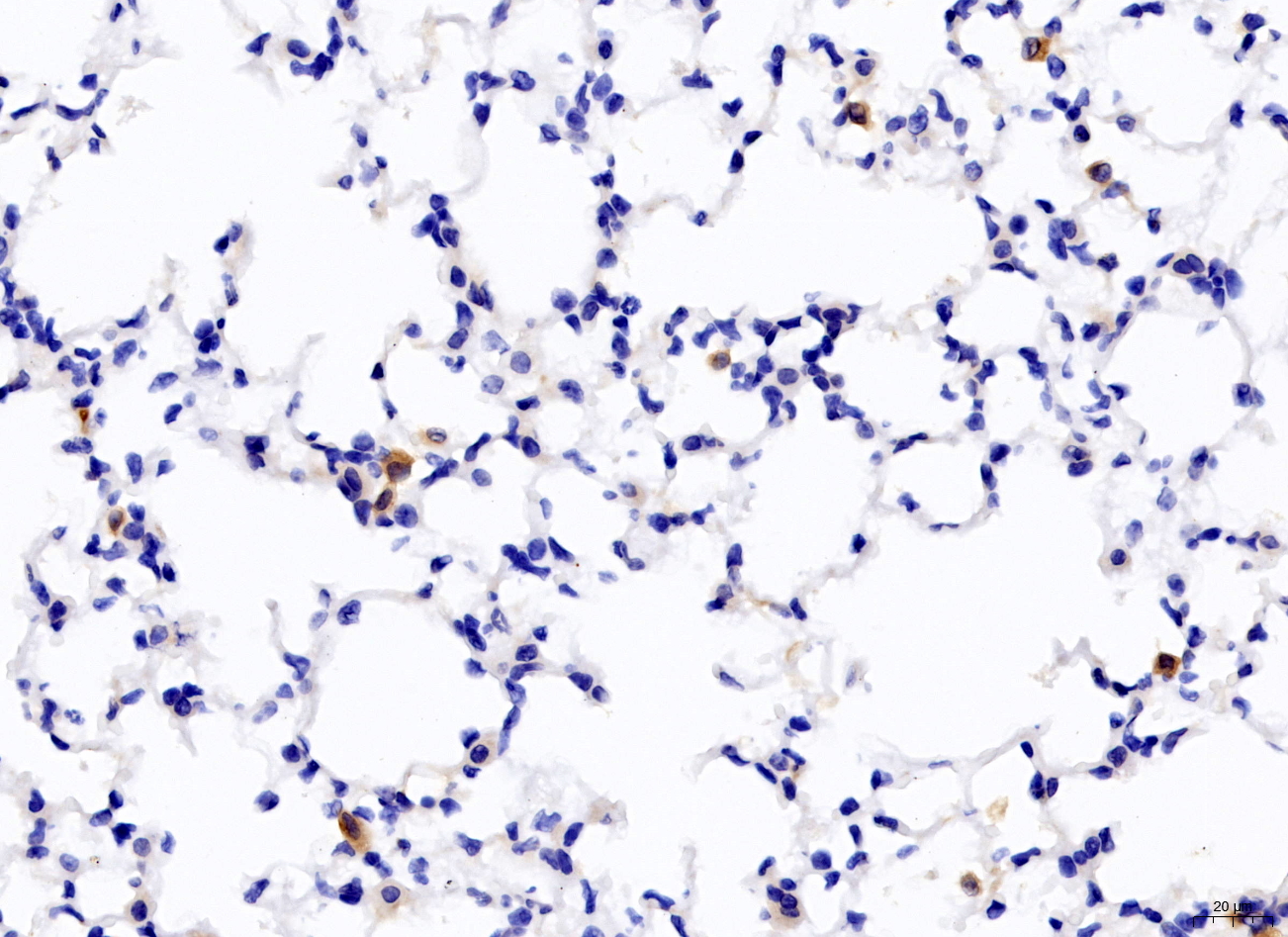

Supplement: Supplementary file 4 — Supporting File 4: advs73867‐sup‐0001‐FiguresData.zip. [file ADVS-13-e19191-s001.zip › Supporting information Figure1-10/Figure 2/Figure 2J/4 week Control/SCRS-Ferritin-4w-Control-778_40.0x-6.jpg]

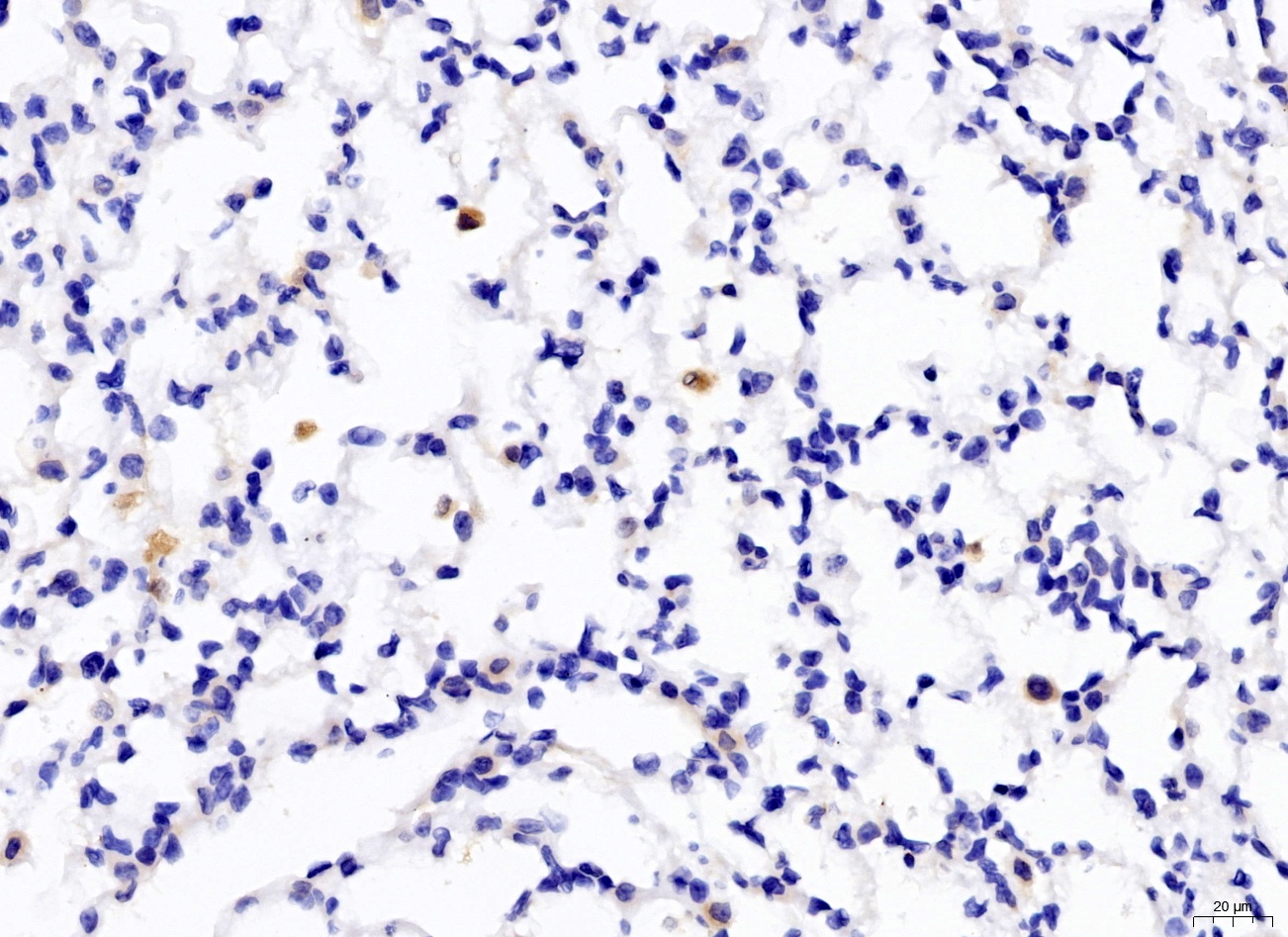

Supplement: Supplementary file 4 — Supporting File 4: advs73867‐sup‐0001‐FiguresData.zip. [file ADVS-13-e19191-s001.zip › Supporting information Figure1-10/Figure 2/Figure 2J/4 week Control/SCRS-Ferritin-4w-Control-778_40.0x-7.jpg]

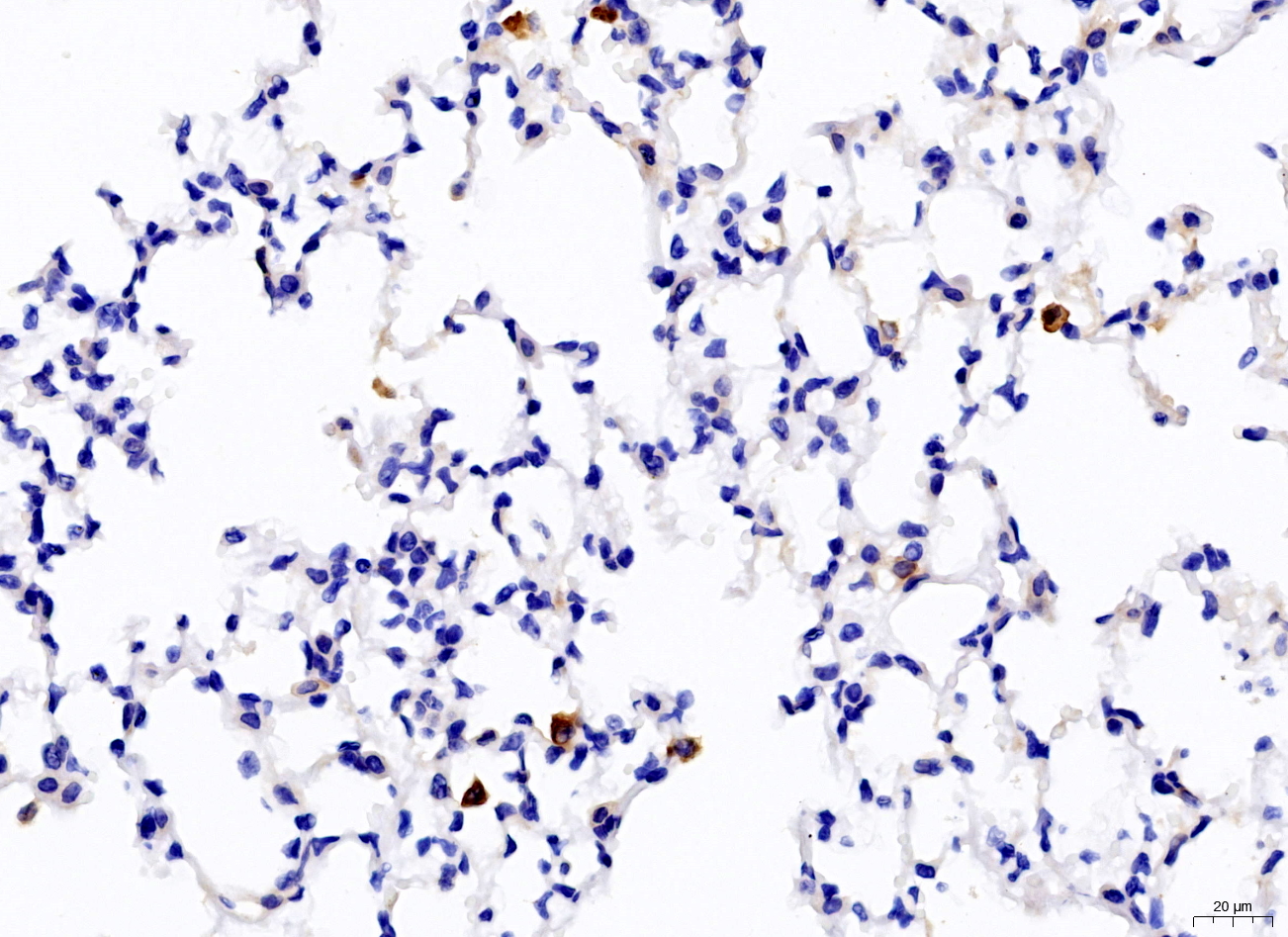

Supplement: Supplementary file 4 — Supporting File 4: advs73867‐sup‐0001‐FiguresData.zip. [file ADVS-13-e19191-s001.zip › Supporting information Figure1-10/Figure 2/Figure 2J/4 week Control/SCRS-Ferritin-4w-Control-778_40.0x-8.jpg]

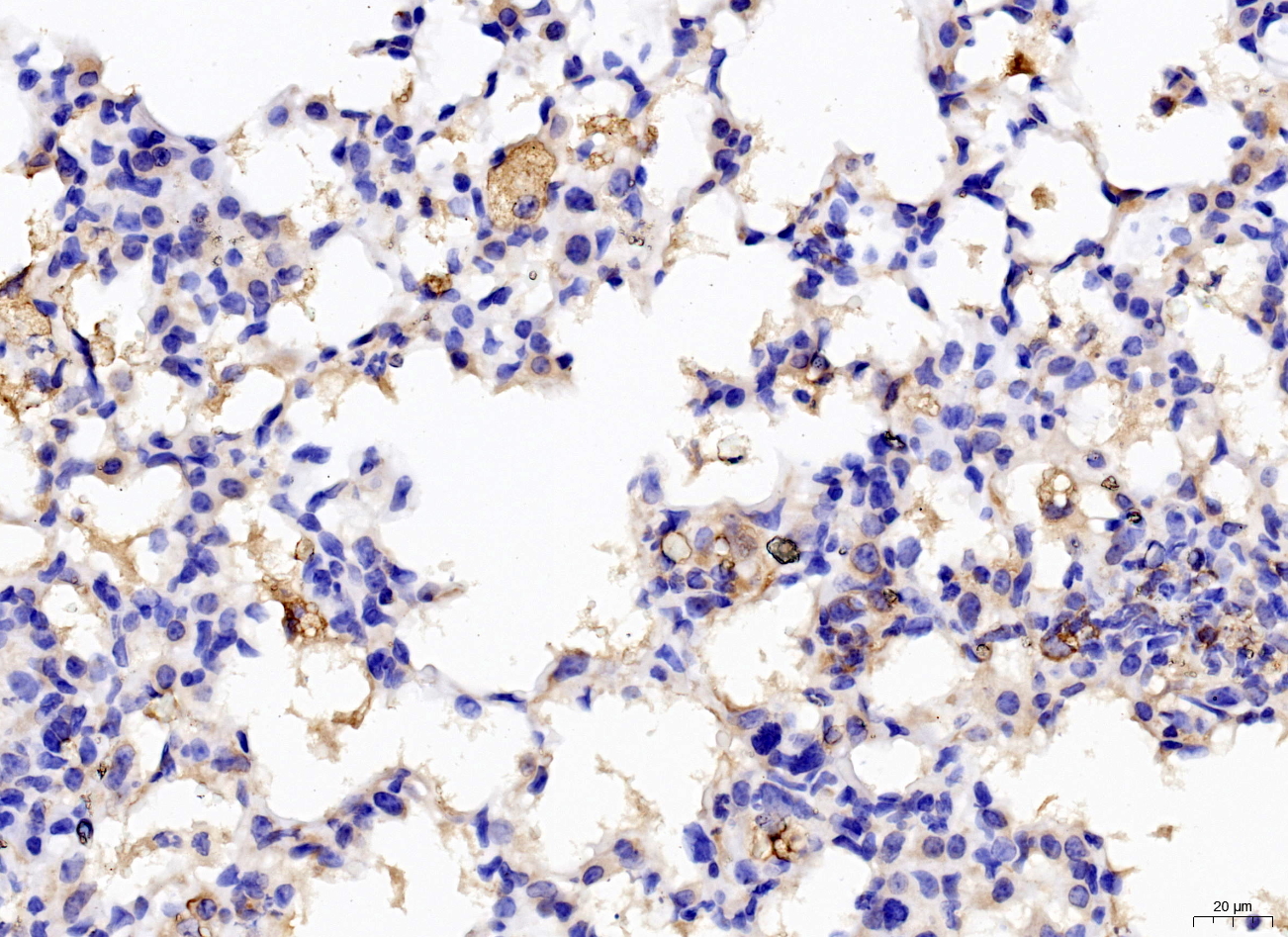

Supplement: Supplementary file 4 — Supporting File 4: advs73867‐sup‐0001‐FiguresData.zip. [file ADVS-13-e19191-s001.zip › Supporting information Figure1-10/Figure 2/Figure 2J/4 week Silica/SCRS-Ferritin-4w-Model-744_40.0x-1.jpg]

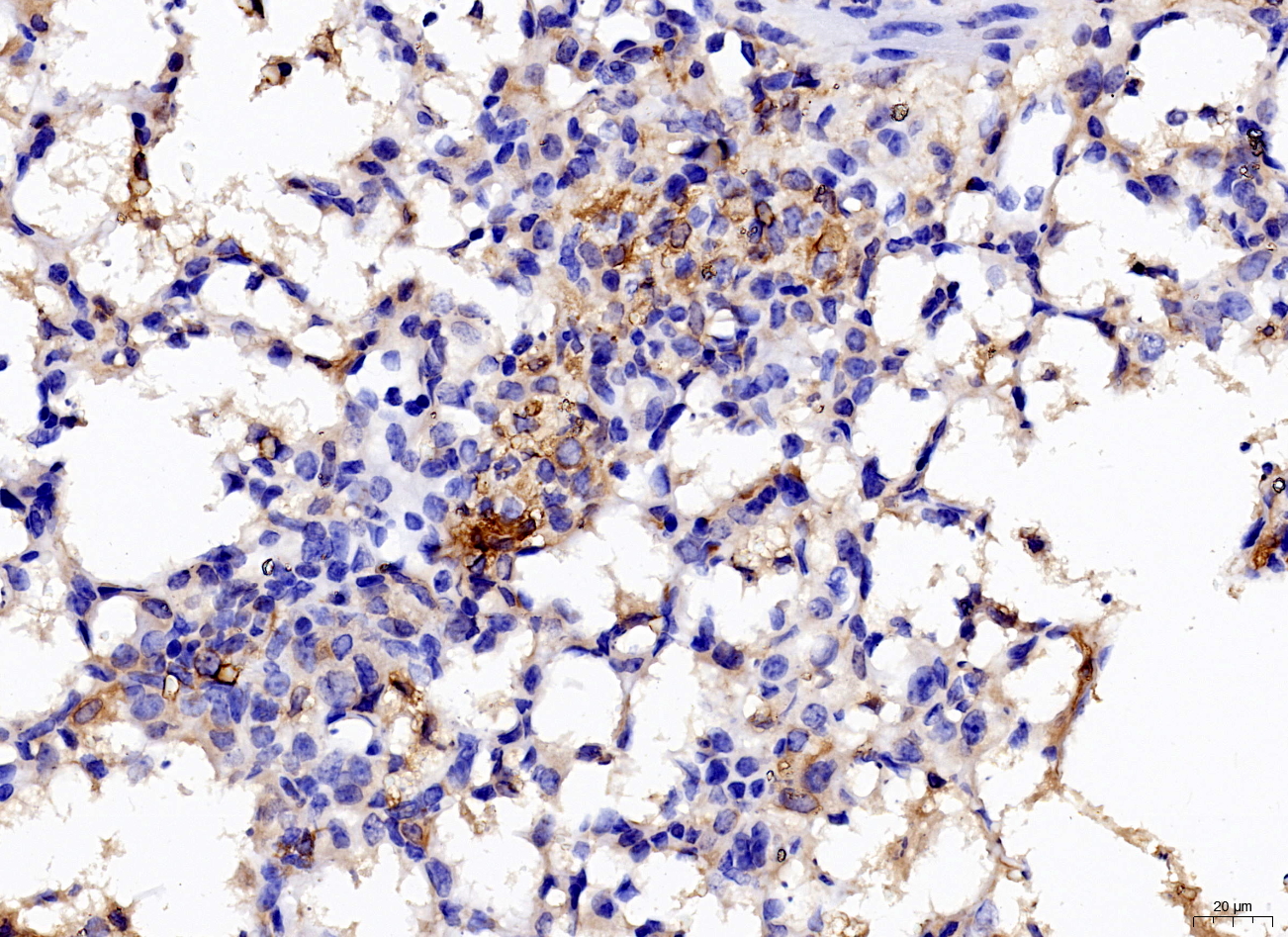

Supplement: Supplementary file 4 — Supporting File 4: advs73867‐sup‐0001‐FiguresData.zip. [file ADVS-13-e19191-s001.zip › Supporting information Figure1-10/Figure 2/Figure 2J/4 week Silica/SCRS-Ferritin-4w-Model-744_40.0x-2.jpg]

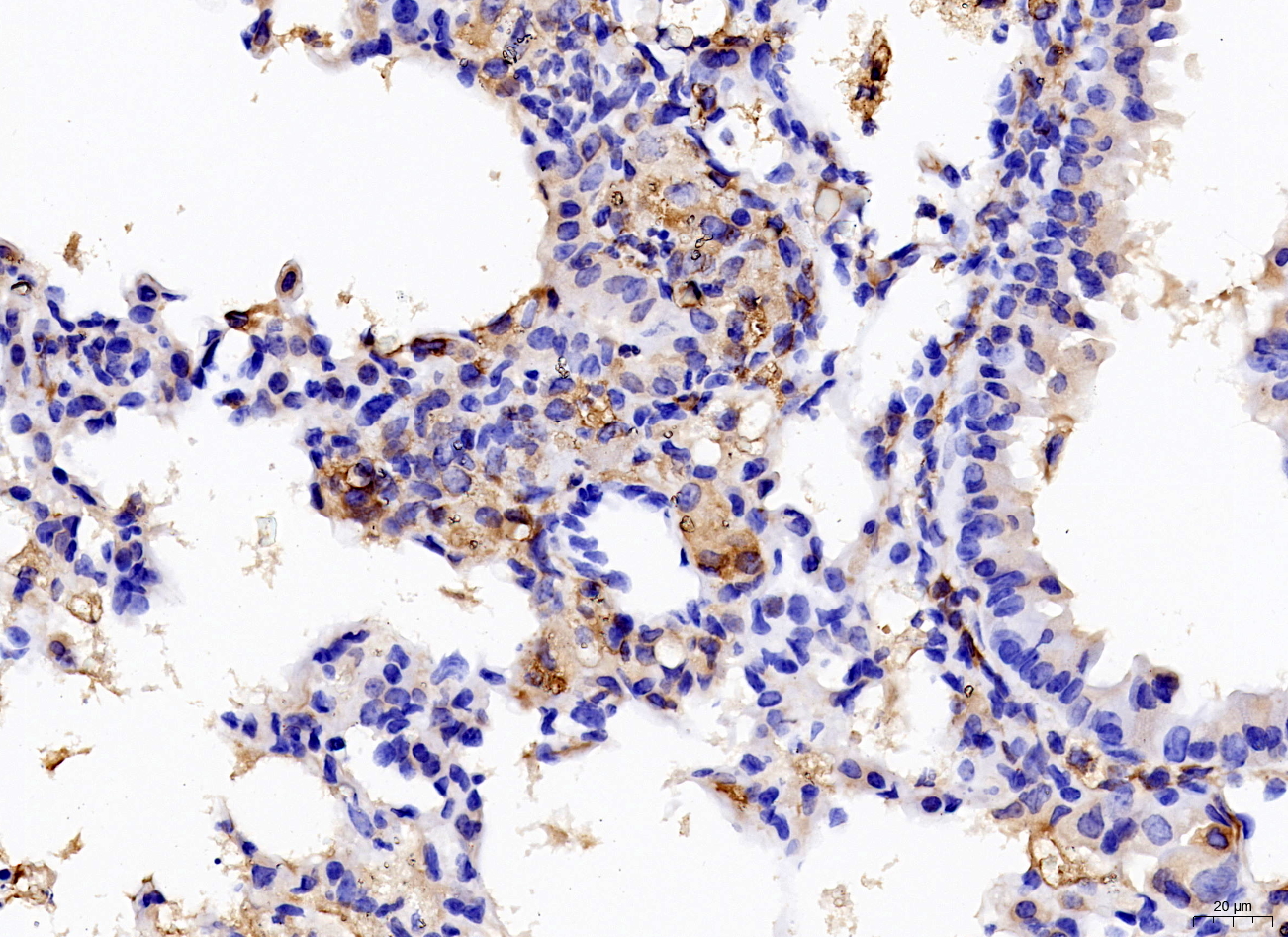

Supplement: Supplementary file 4 — Supporting File 4: advs73867‐sup‐0001‐FiguresData.zip. [file ADVS-13-e19191-s001.zip › Supporting information Figure1-10/Figure 2/Figure 2J/4 week Silica/SCRS-Ferritin-4w-Model-744_40.0x-4.jpg]

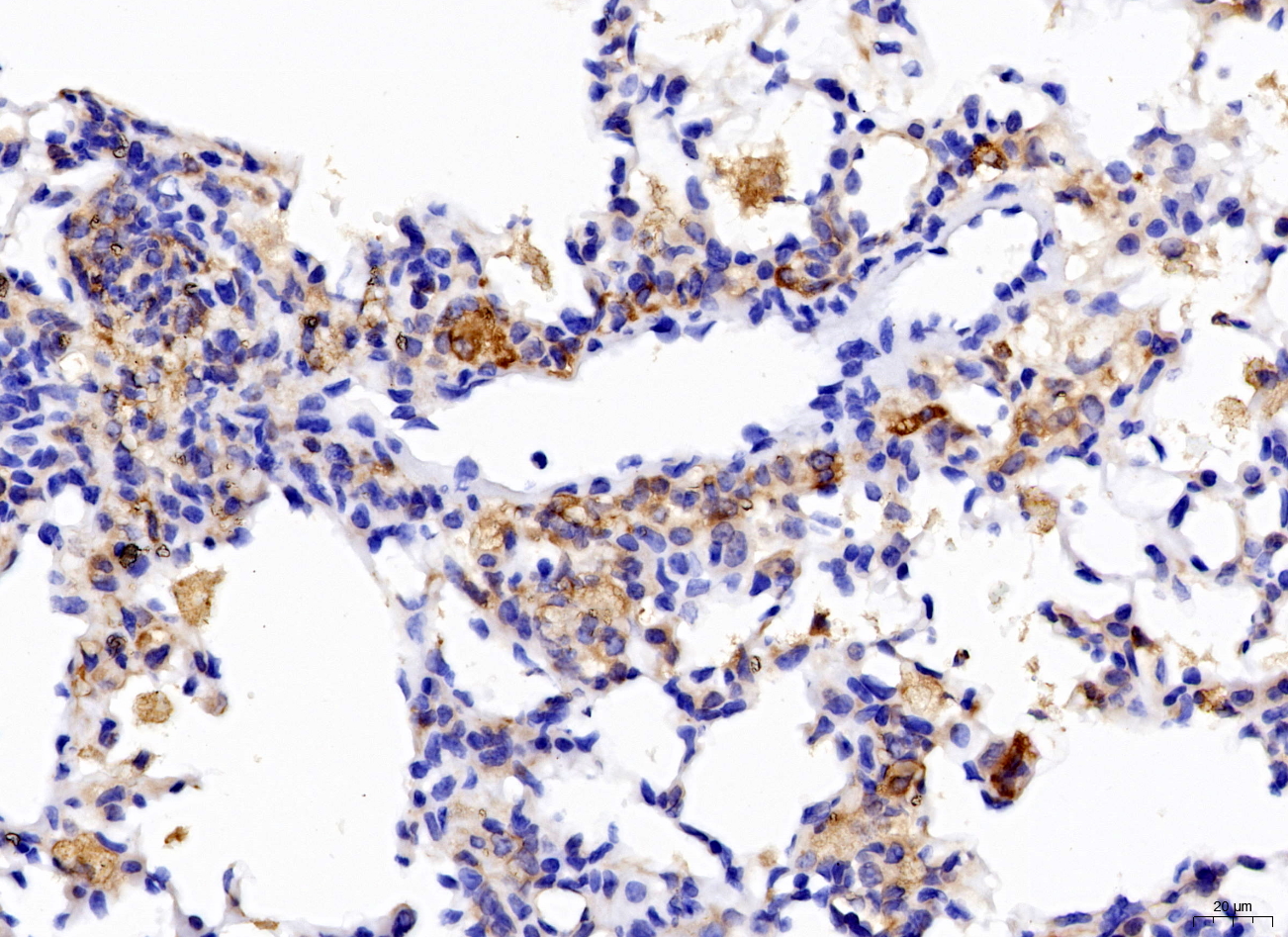

Supplement: Supplementary file 4 — Supporting File 4: advs73867‐sup‐0001‐FiguresData.zip. [file ADVS-13-e19191-s001.zip › Supporting information Figure1-10/Figure 2/Figure 2J/4 week Silica/SCRS-Ferritin-4w-Model-756_40.0x-3.jpg]

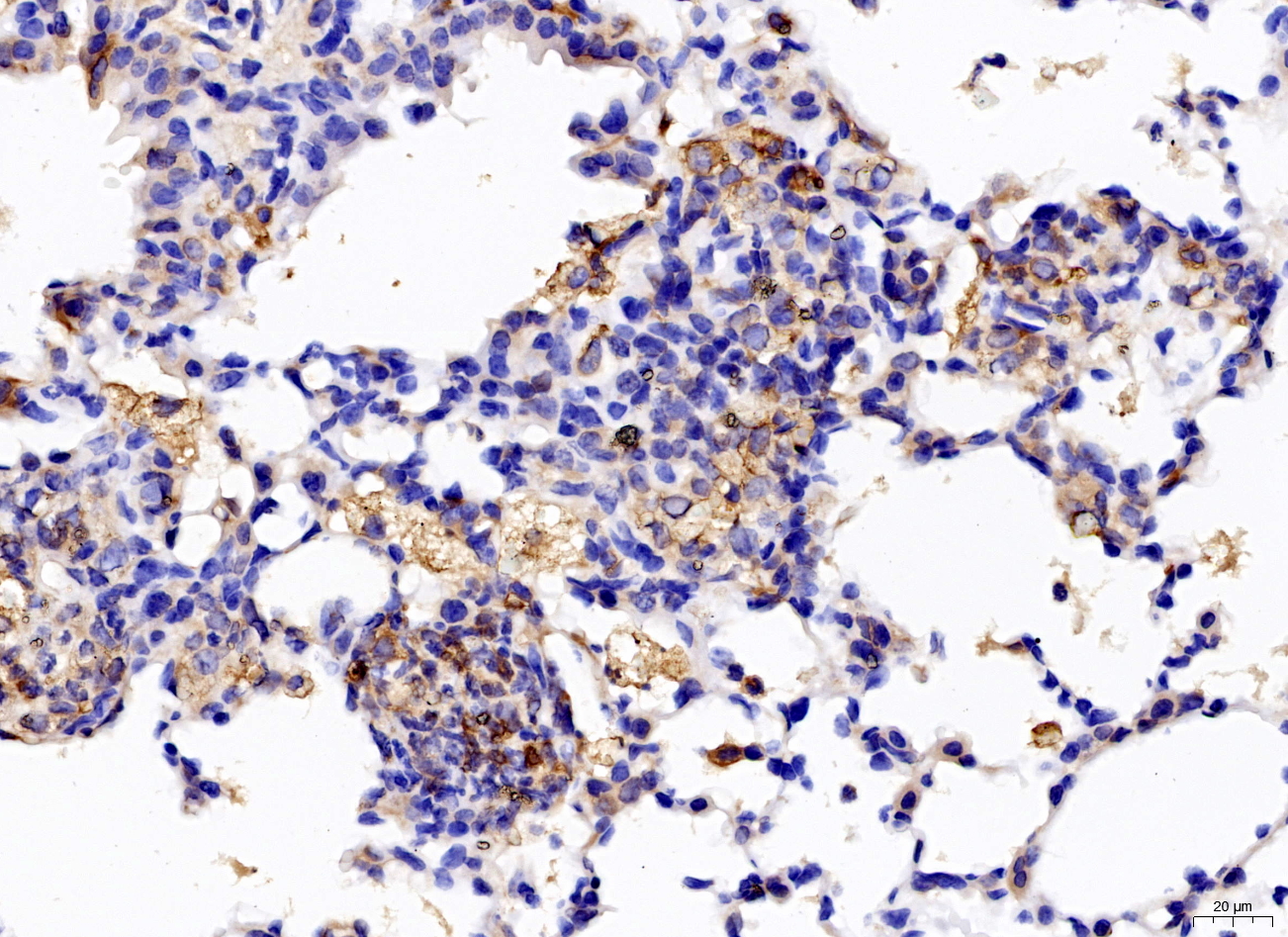

Supplement: Supplementary file 4 — Supporting File 4: advs73867‐sup‐0001‐FiguresData.zip. [file ADVS-13-e19191-s001.zip › Supporting information Figure1-10/Figure 2/Figure 2J/4 week Silica/SCRS-Ferritin-4w-Model-756_40.0x-5.jpg]

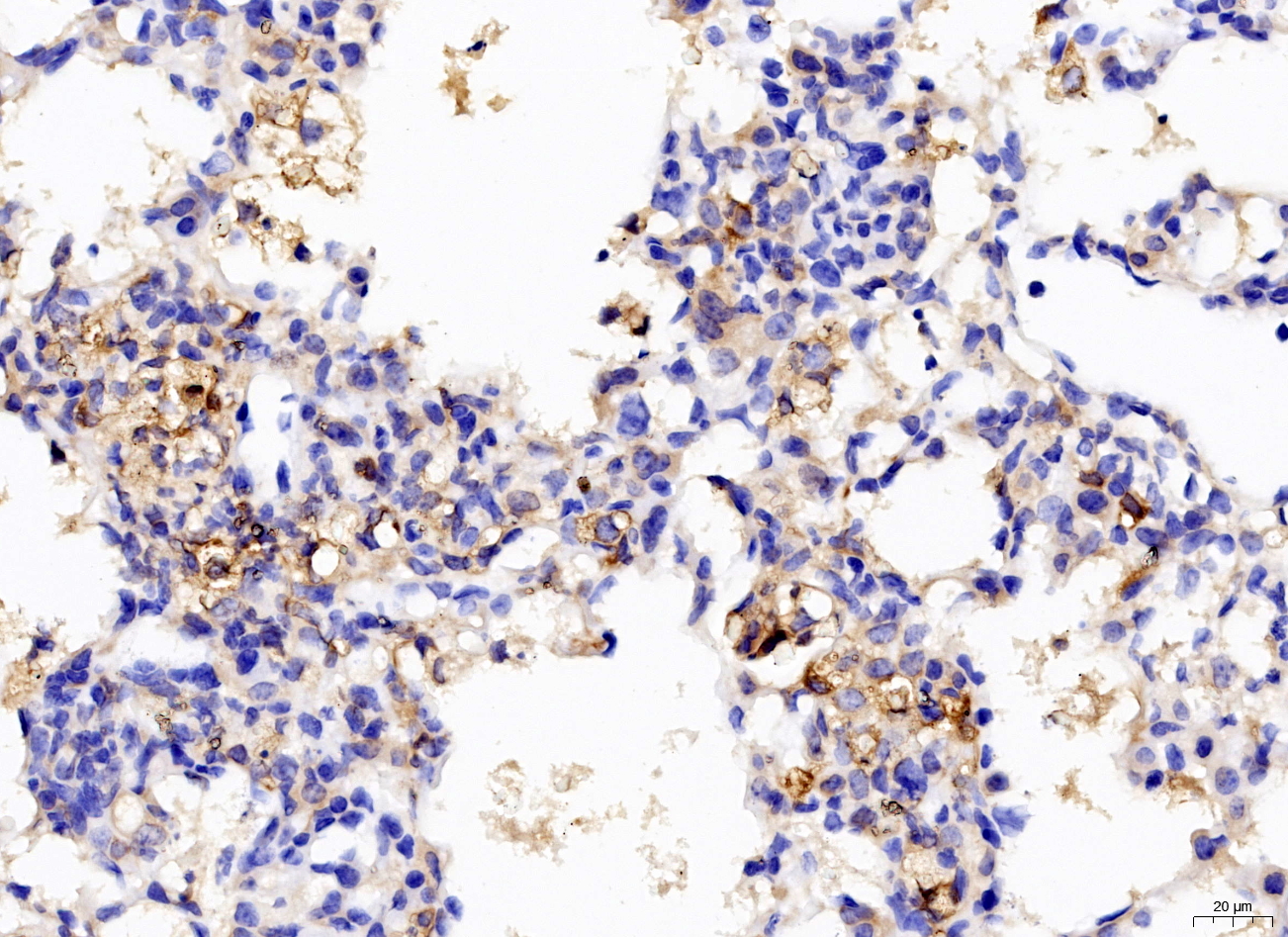

Supplement: Supplementary file 4 — Supporting File 4: advs73867‐sup‐0001‐FiguresData.zip. [file ADVS-13-e19191-s001.zip › Supporting information Figure1-10/Figure 2/Figure 2J/4 week Silica/SCRS-Ferritin-4w-Model-756_40.0x-7.jpg]

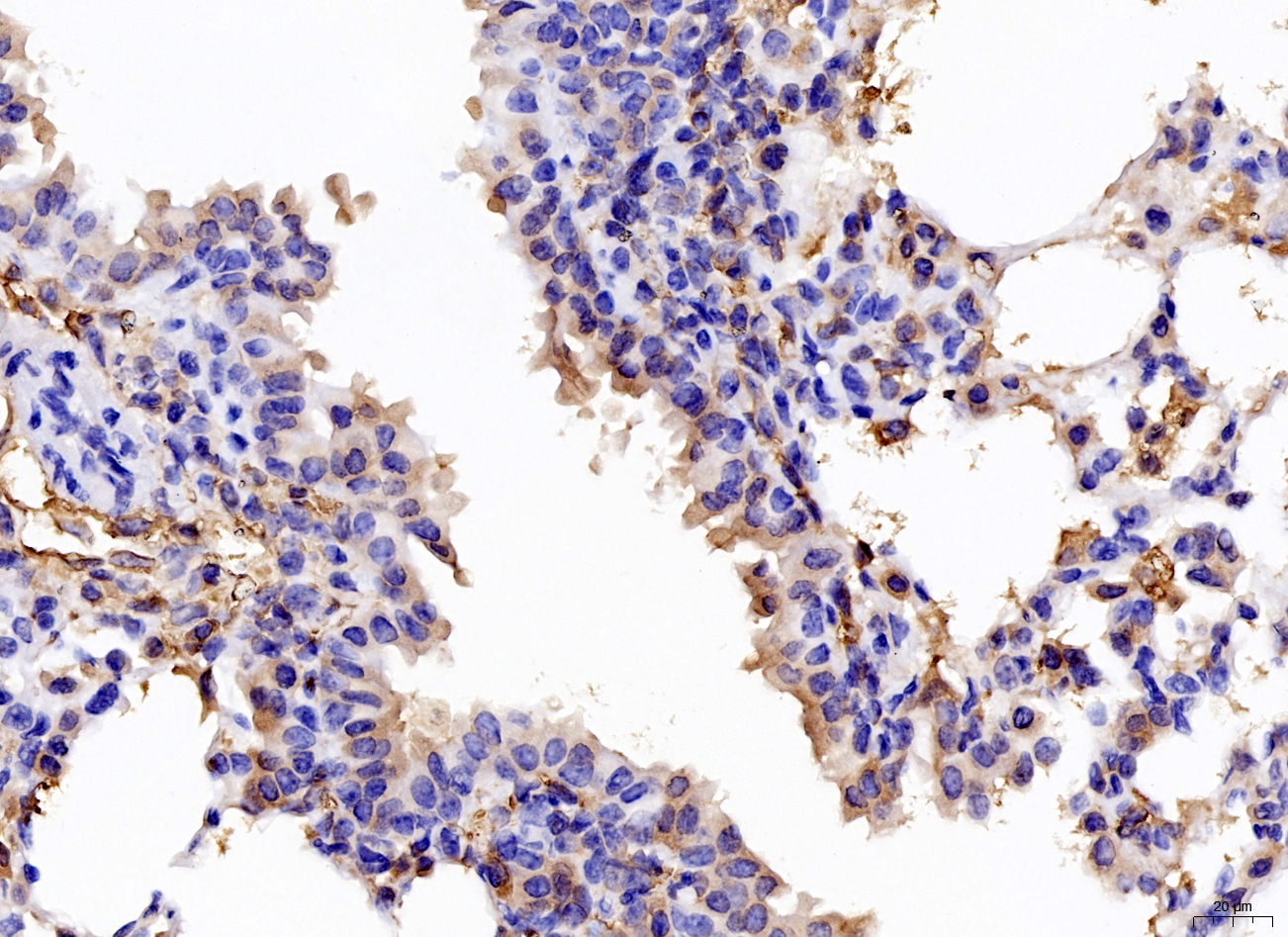

Supplement: Supplementary file 4 — Supporting File 4: advs73867‐sup‐0001‐FiguresData.zip. [file ADVS-13-e19191-s001.zip › Supporting information Figure1-10/Figure 2/Figure 2J/4 week Silica/SCRS-Ferritin-4w-Model-760_40.0x-6.jpg]

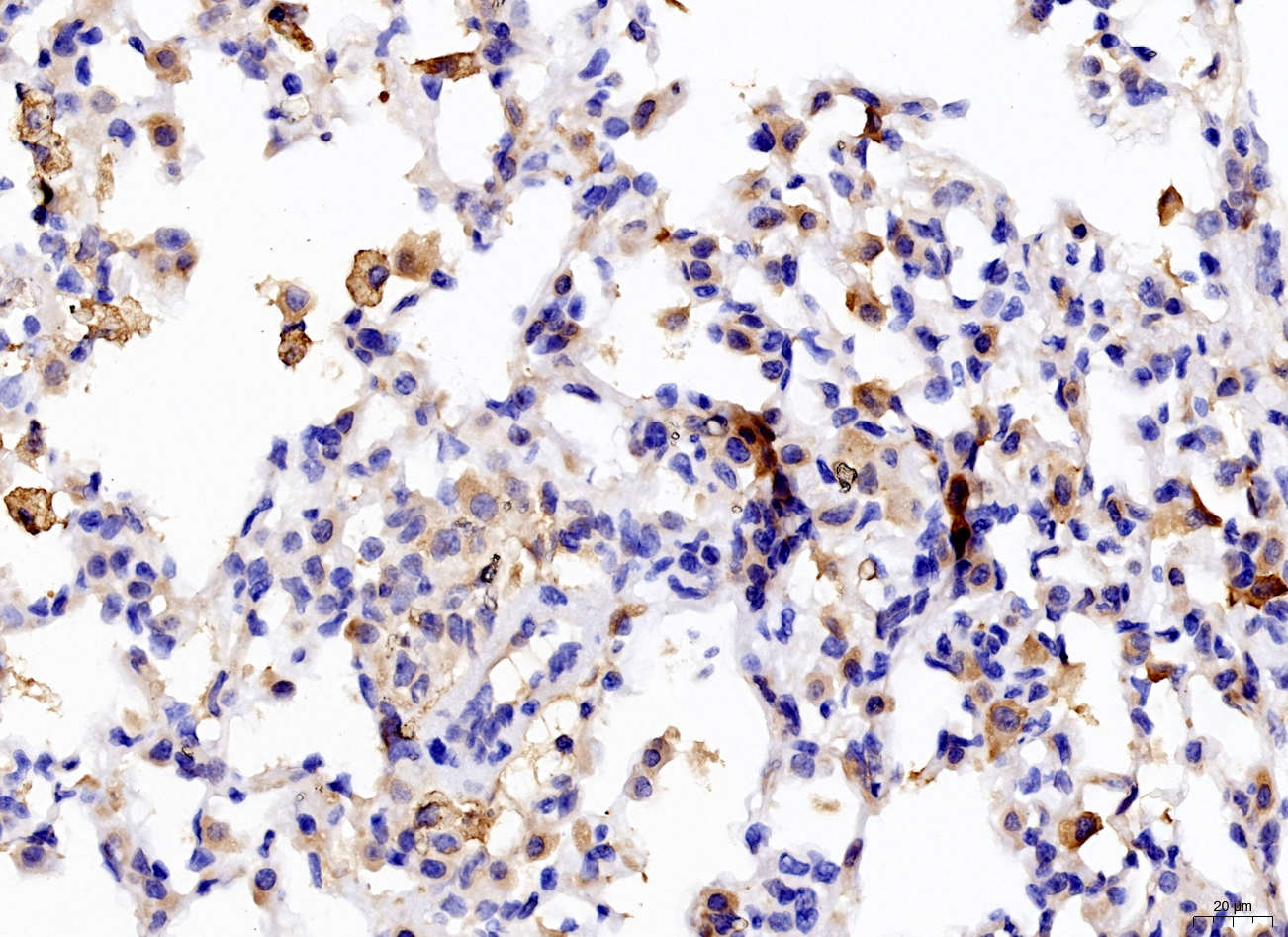

Supplement: Supplementary file 4 — Supporting File 4: advs73867‐sup‐0001‐FiguresData.zip. [file ADVS-13-e19191-s001.zip › Supporting information Figure1-10/Figure 2/Figure 2J/4 week Silica/SCRS-Ferritin-4w-Model-760_40.0x-8.jpg]

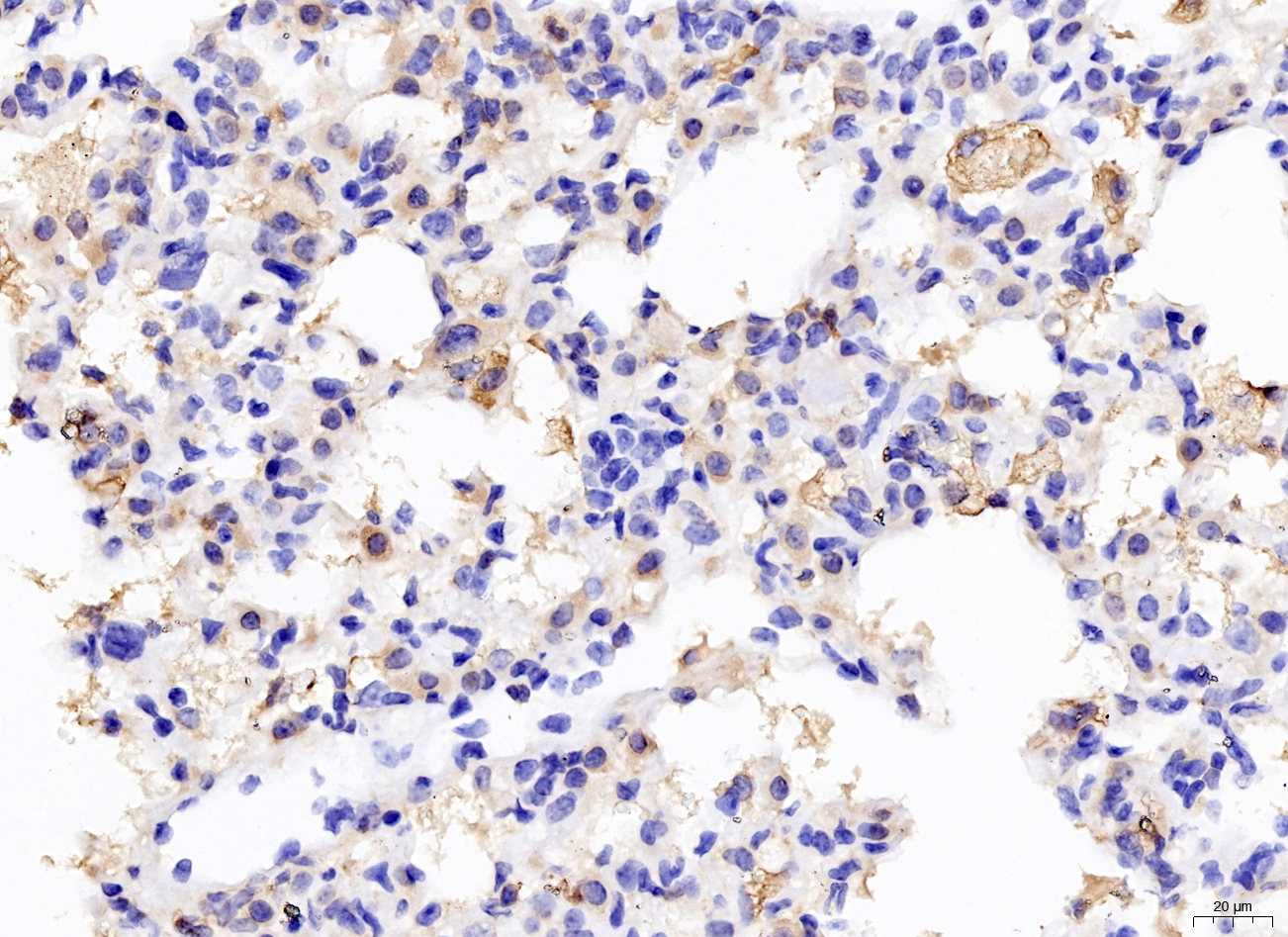

Supplement: Supplementary file 4 — Supporting File 4: advs73867‐sup‐0001‐FiguresData.zip. [file ADVS-13-e19191-s001.zip › Supporting information Figure1-10/Figure 2/Figure 2J/4 week Silica/SCRS-Ferritin-4w-Model-760_40.0x-9.jpg]

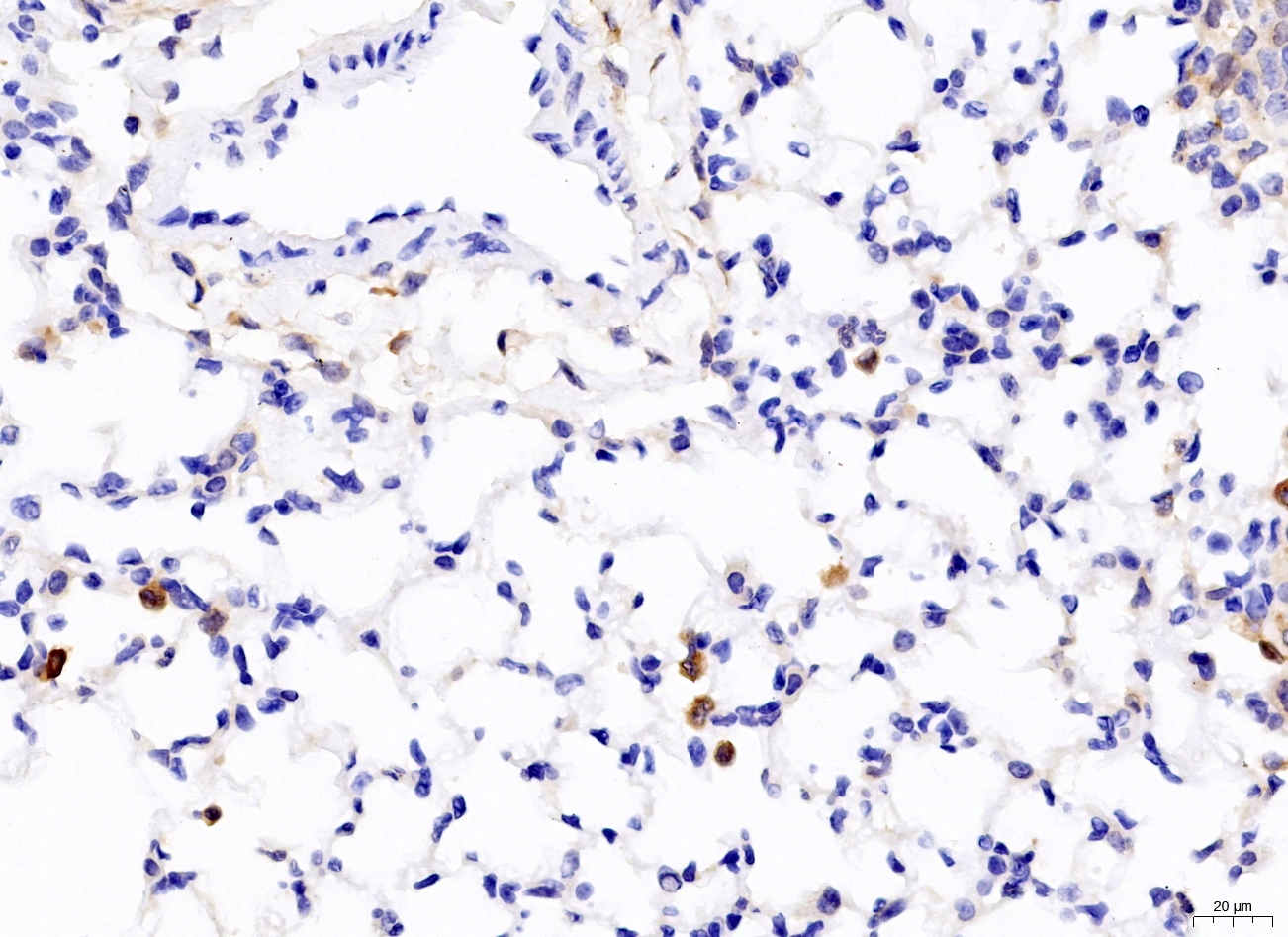

Supplement: Supplementary file 4 — Supporting File 4: advs73867‐sup‐0001‐FiguresData.zip. [file ADVS-13-e19191-s001.zip › Supporting information Figure1-10/Figure 2/Figure 2J/8 week Control/SCRS-Ferritin-8w-Control-785_40.0x-1.jpg]

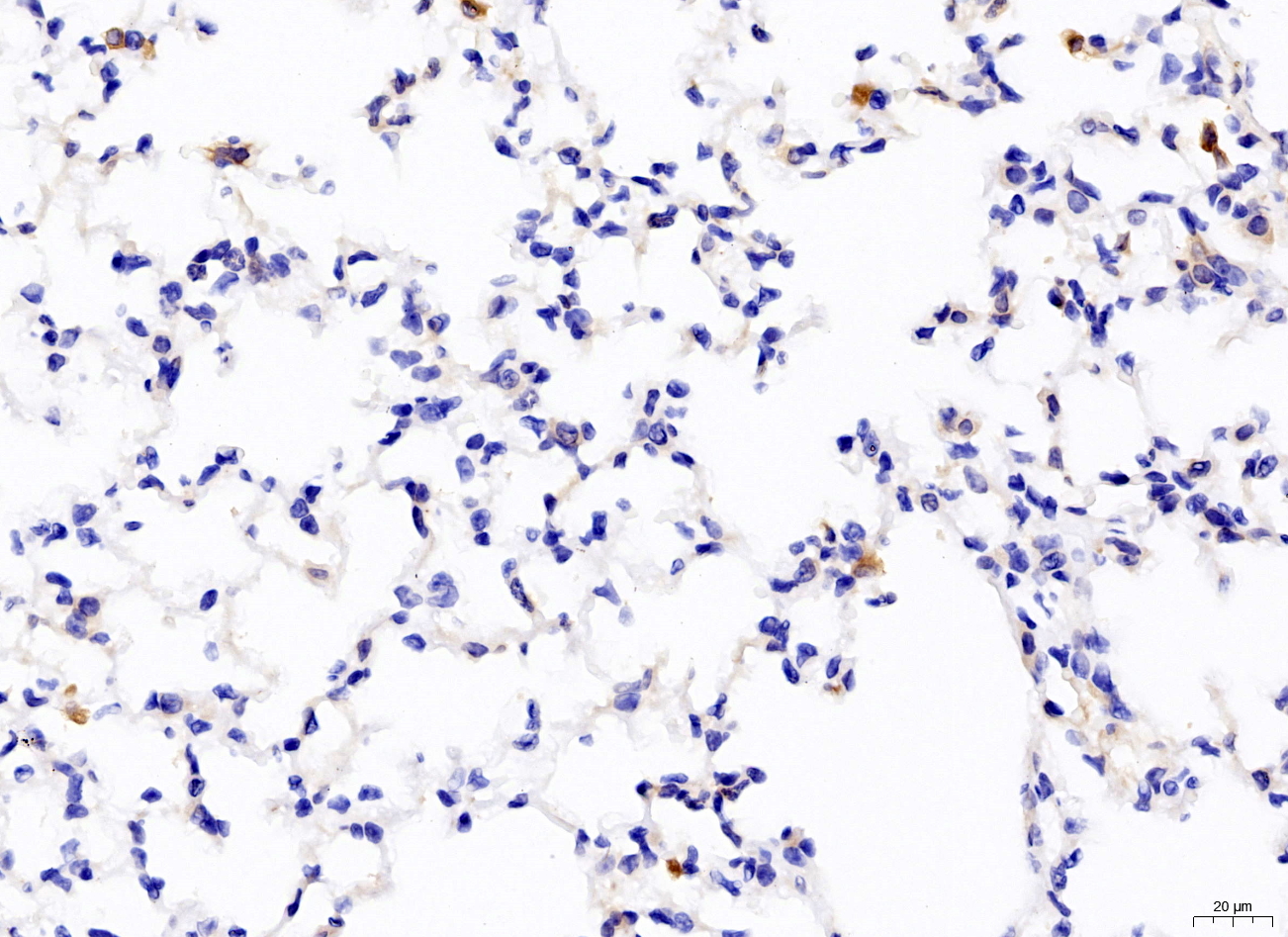

Supplement: Supplementary file 4 — Supporting File 4: advs73867‐sup‐0001‐FiguresData.zip. [file ADVS-13-e19191-s001.zip › Supporting information Figure1-10/Figure 2/Figure 2J/8 week Control/SCRS-Ferritin-8w-Control-785_40.0x-2.jpg]

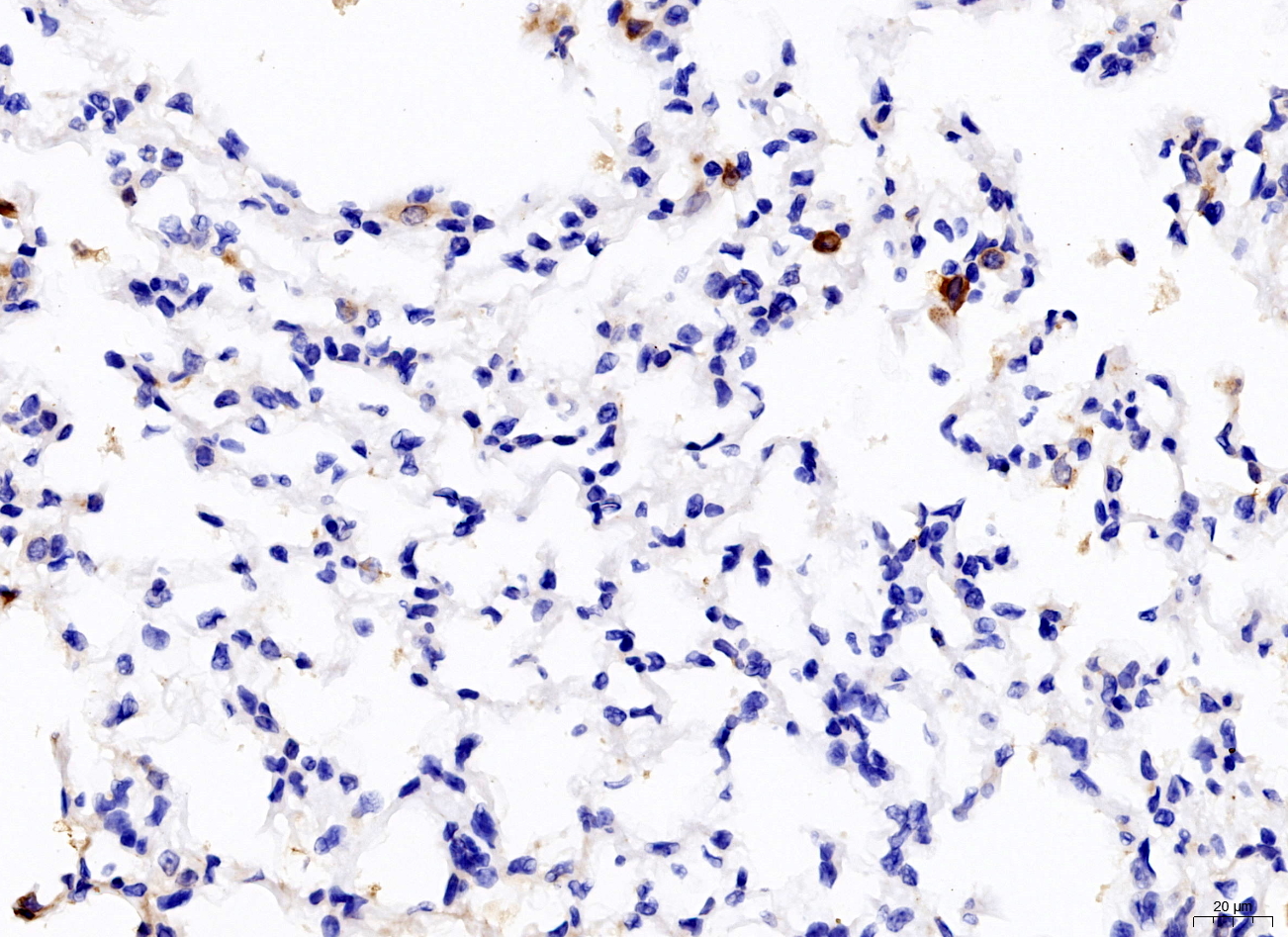

Supplement: Supplementary file 4 — Supporting File 4: advs73867‐sup‐0001‐FiguresData.zip. [file ADVS-13-e19191-s001.zip › Supporting information Figure1-10/Figure 2/Figure 2J/8 week Control/SCRS-Ferritin-8w-Control-785_40.0x-3.jpg]

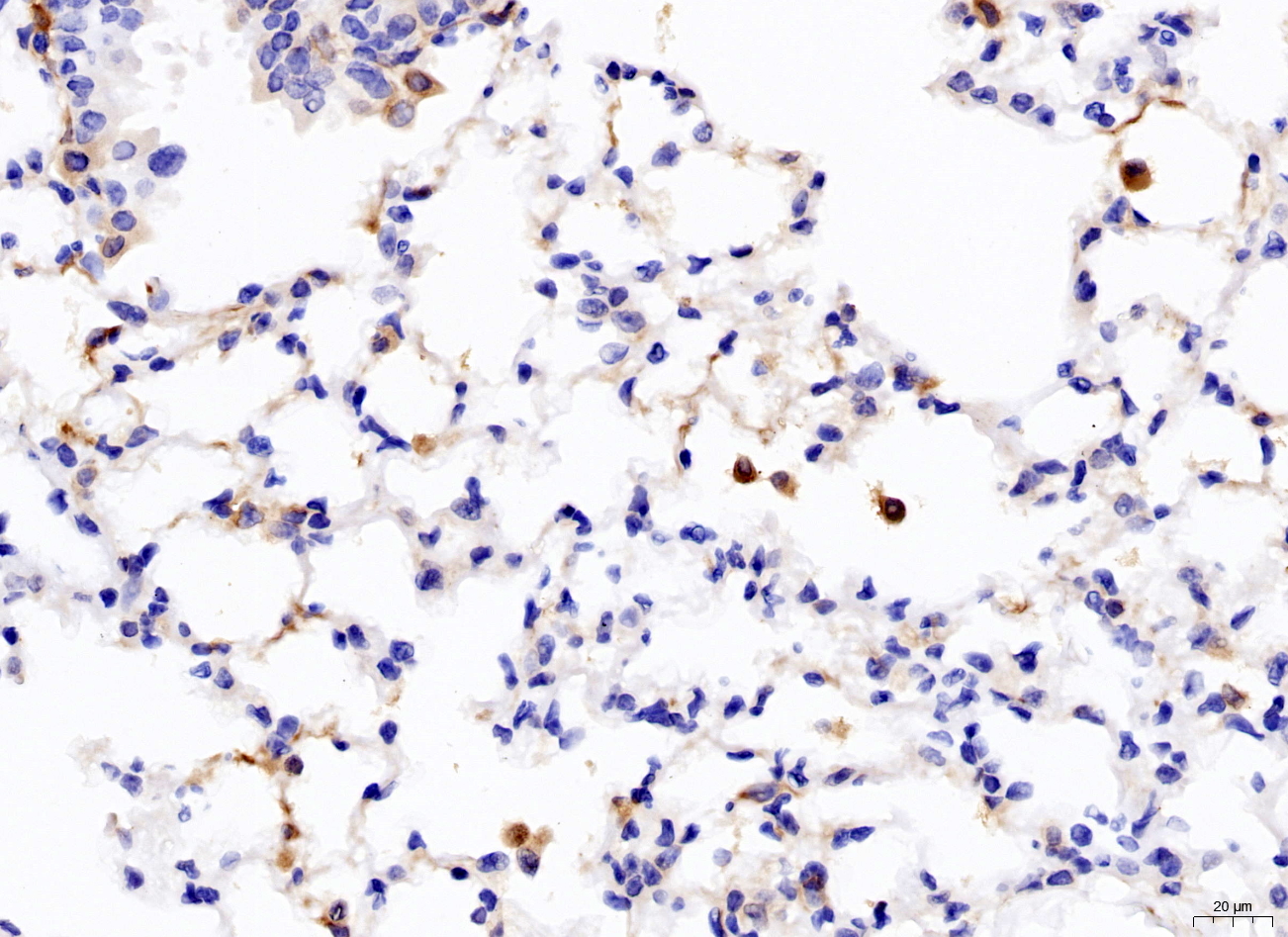

Supplement: Supplementary file 4 — Supporting File 4: advs73867‐sup‐0001‐FiguresData.zip. [file ADVS-13-e19191-s001.zip › Supporting information Figure1-10/Figure 2/Figure 2J/8 week Control/SCRS-Ferritin-8w-Control-790_40.0x-5.jpg]

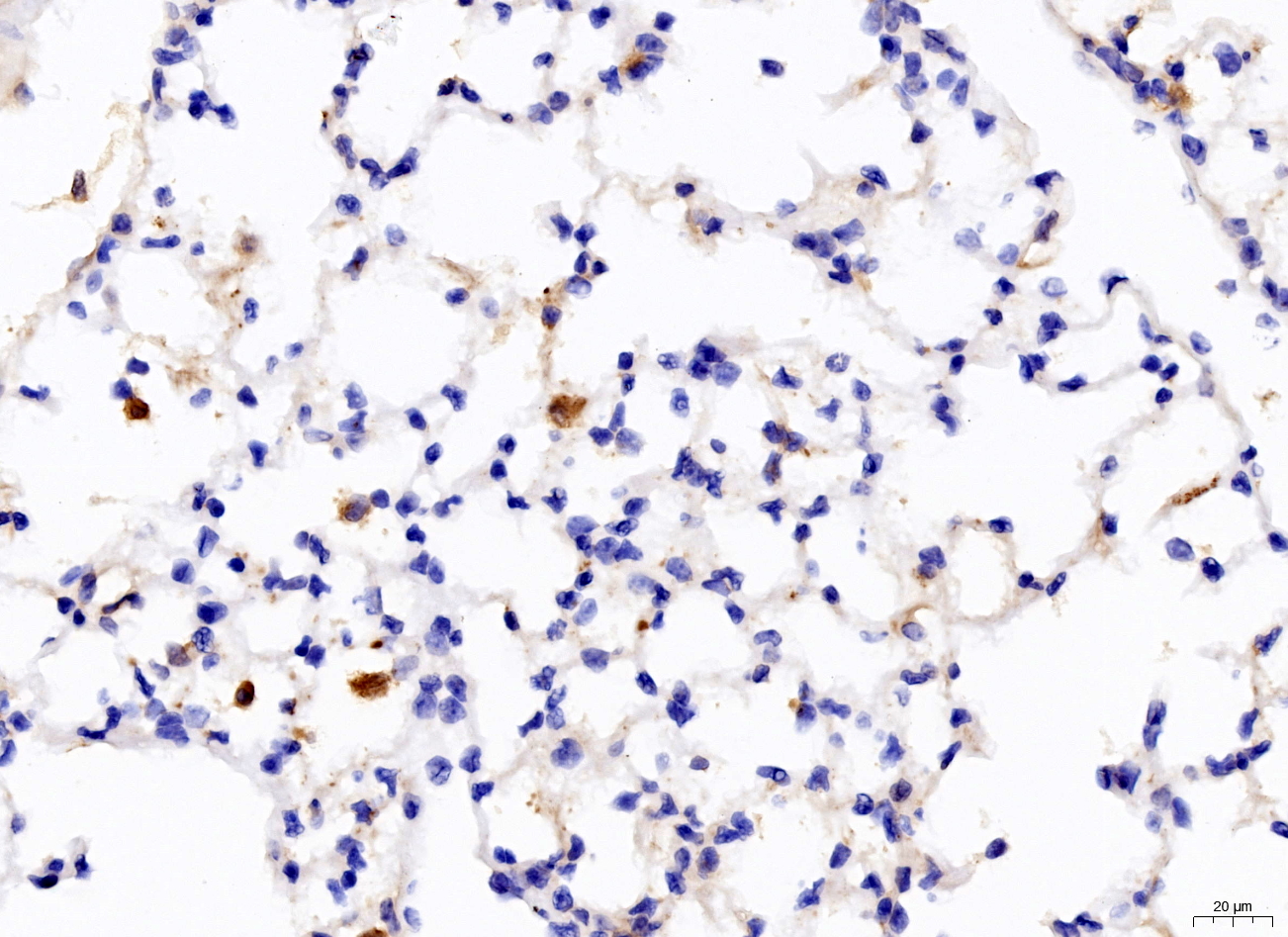

Supplement: Supplementary file 4 — Supporting File 4: advs73867‐sup‐0001‐FiguresData.zip. [file ADVS-13-e19191-s001.zip › Supporting information Figure1-10/Figure 2/Figure 2J/8 week Control/SCRS-Ferritin-8w-Control-794_40.0x-4.jpg]

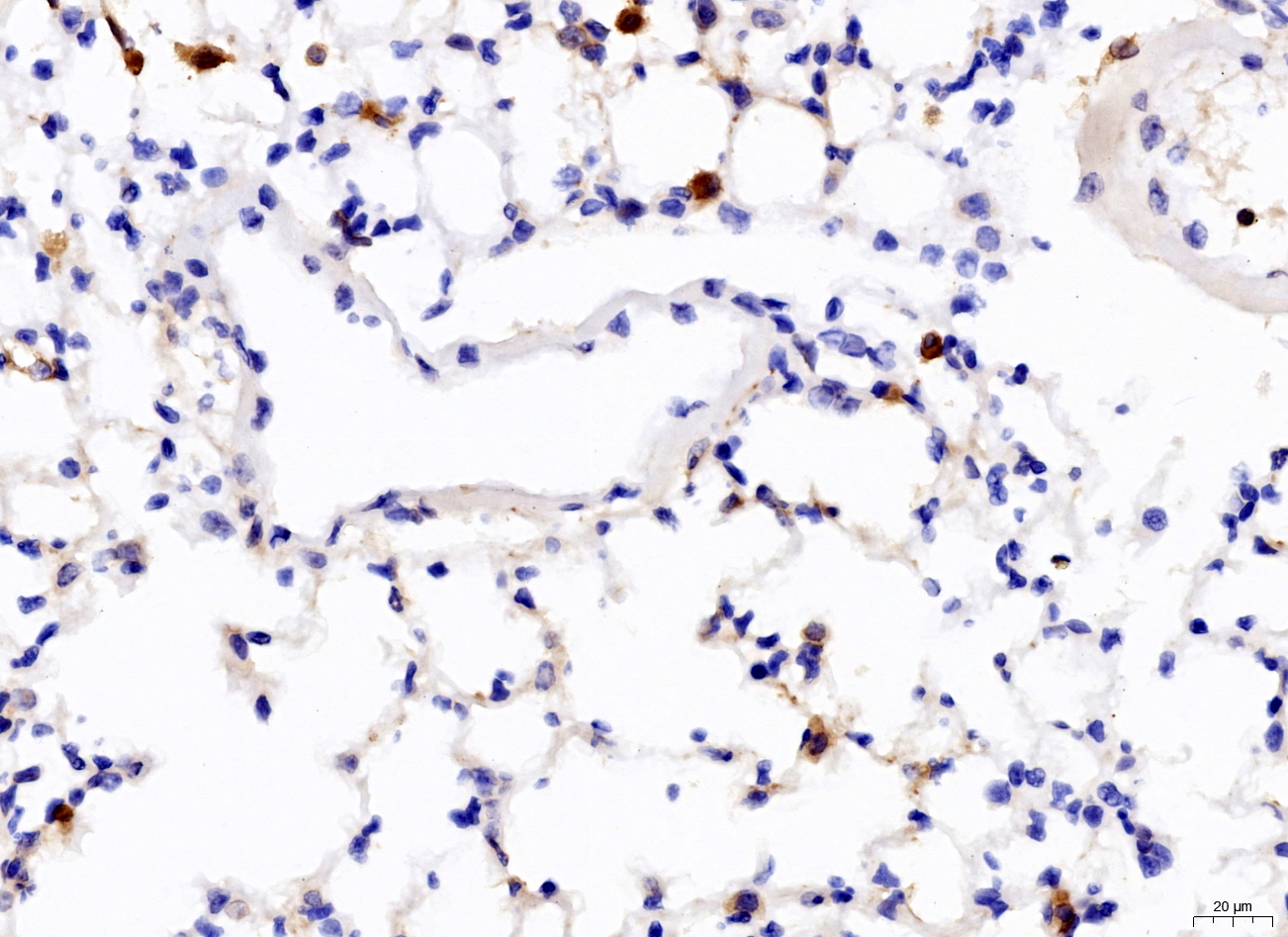

Supplement: Supplementary file 4 — Supporting File 4: advs73867‐sup‐0001‐FiguresData.zip. [file ADVS-13-e19191-s001.zip › Supporting information Figure1-10/Figure 2/Figure 2J/8 week Control/SCRS-Ferritin-8w-Control-794_40.0x-6.jpg]

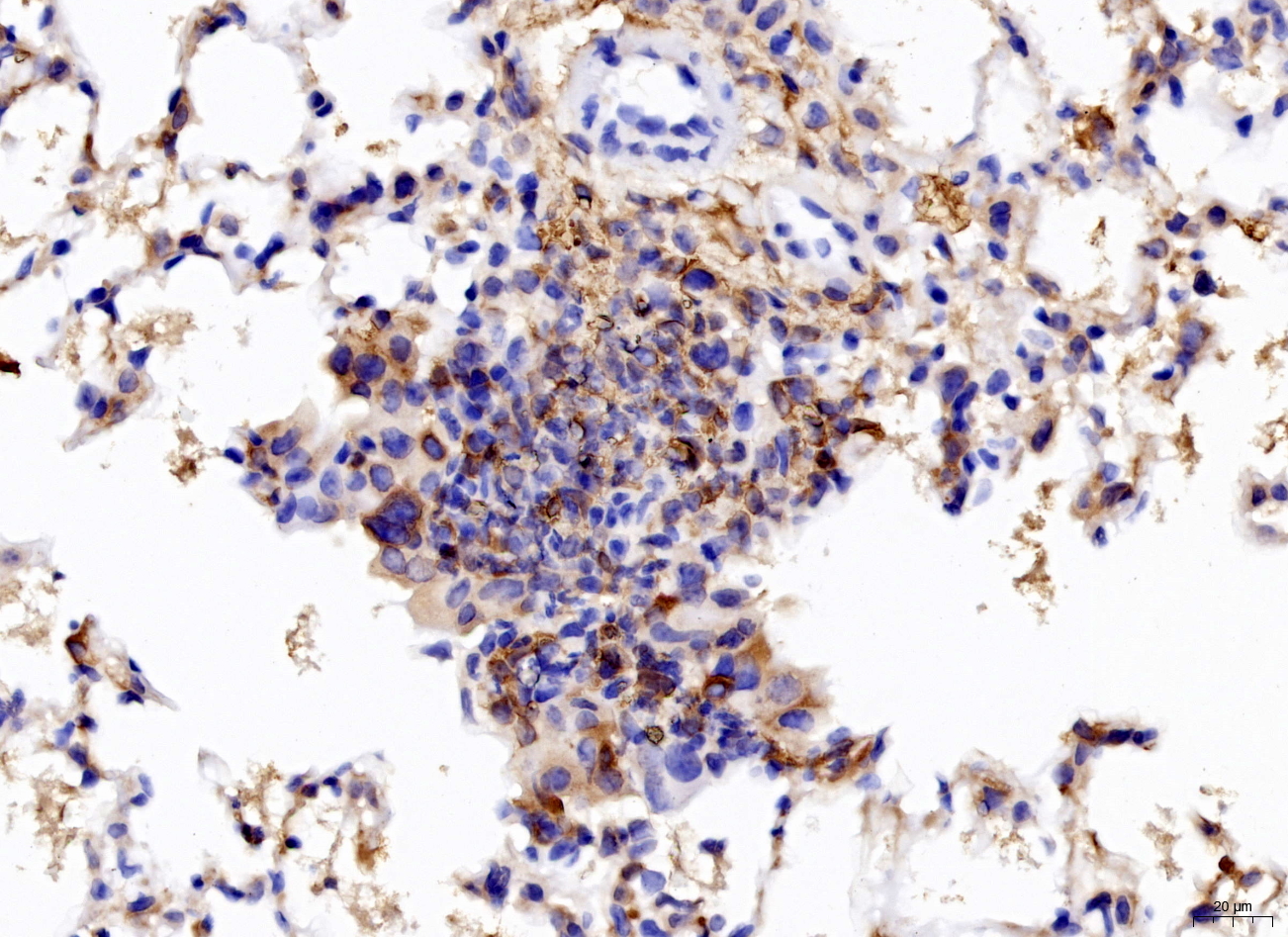

Supplement: Supplementary file 4 — Supporting File 4: advs73867‐sup‐0001‐FiguresData.zip. [file ADVS-13-e19191-s001.zip › Supporting information Figure1-10/Figure 2/Figure 2J/8 week Silica/SCRS-Ferritin-8w-Model-750_40.0x-1.jpg]

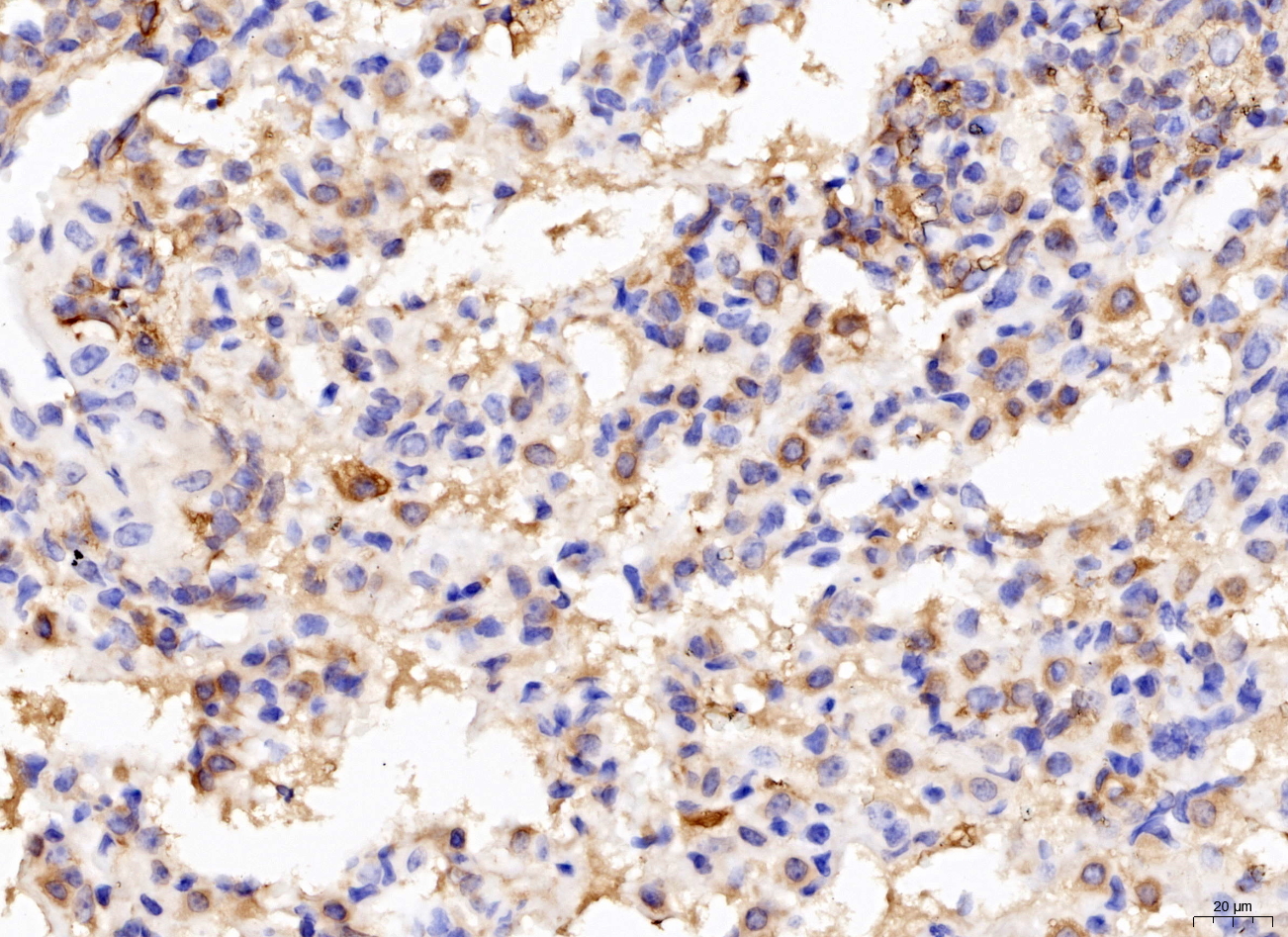

Supplement: Supplementary file 4 — Supporting File 4: advs73867‐sup‐0001‐FiguresData.zip. [file ADVS-13-e19191-s001.zip › Supporting information Figure1-10/Figure 2/Figure 2J/8 week Silica/SCRS-Ferritin-8w-Model-750_40.0x-2.jpg]

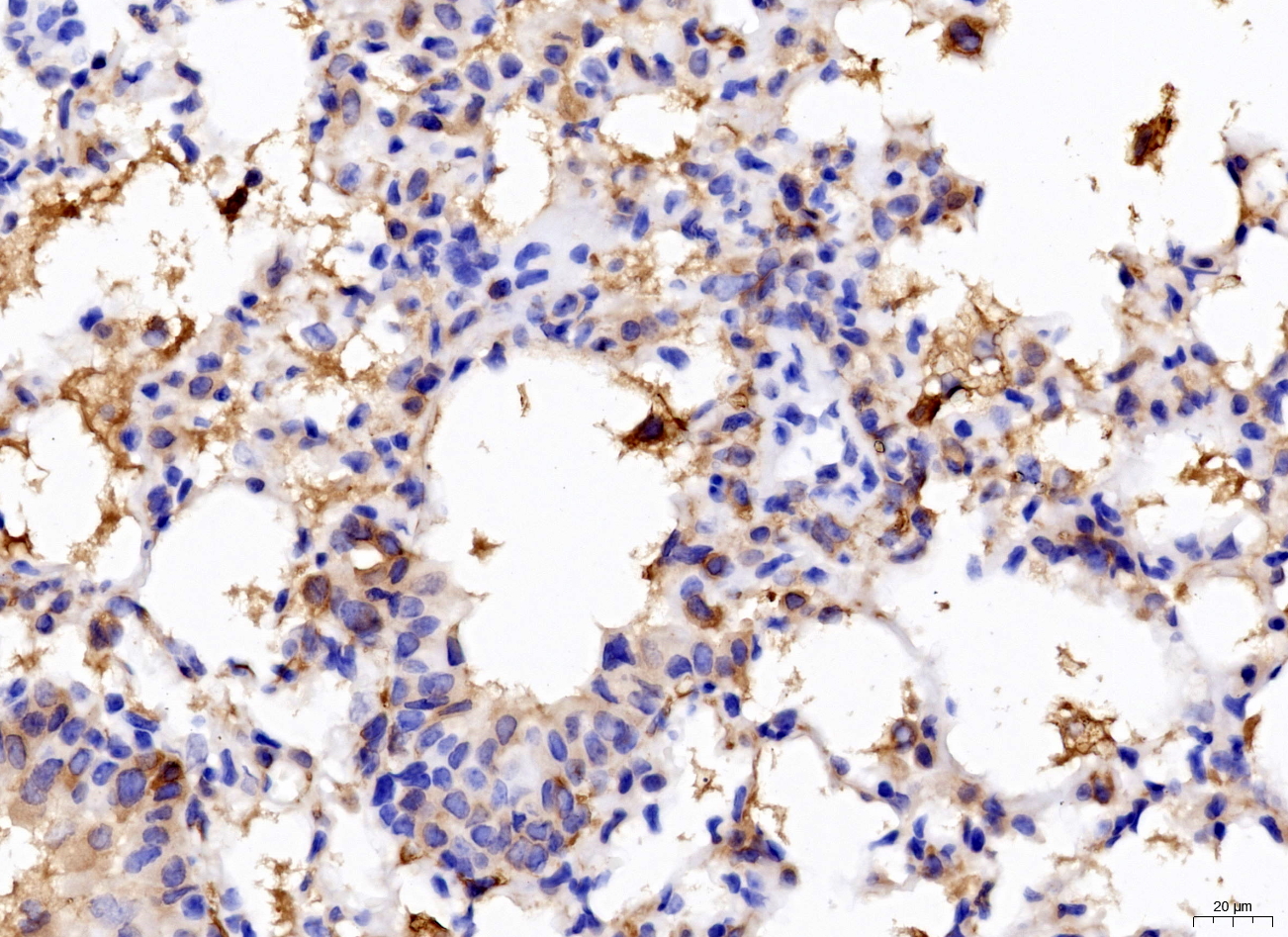

Supplement: Supplementary file 4 — Supporting File 4: advs73867‐sup‐0001‐FiguresData.zip. [file ADVS-13-e19191-s001.zip › Supporting information Figure1-10/Figure 2/Figure 2J/8 week Silica/SCRS-Ferritin-8w-Model-750_40.0x-3.jpg]

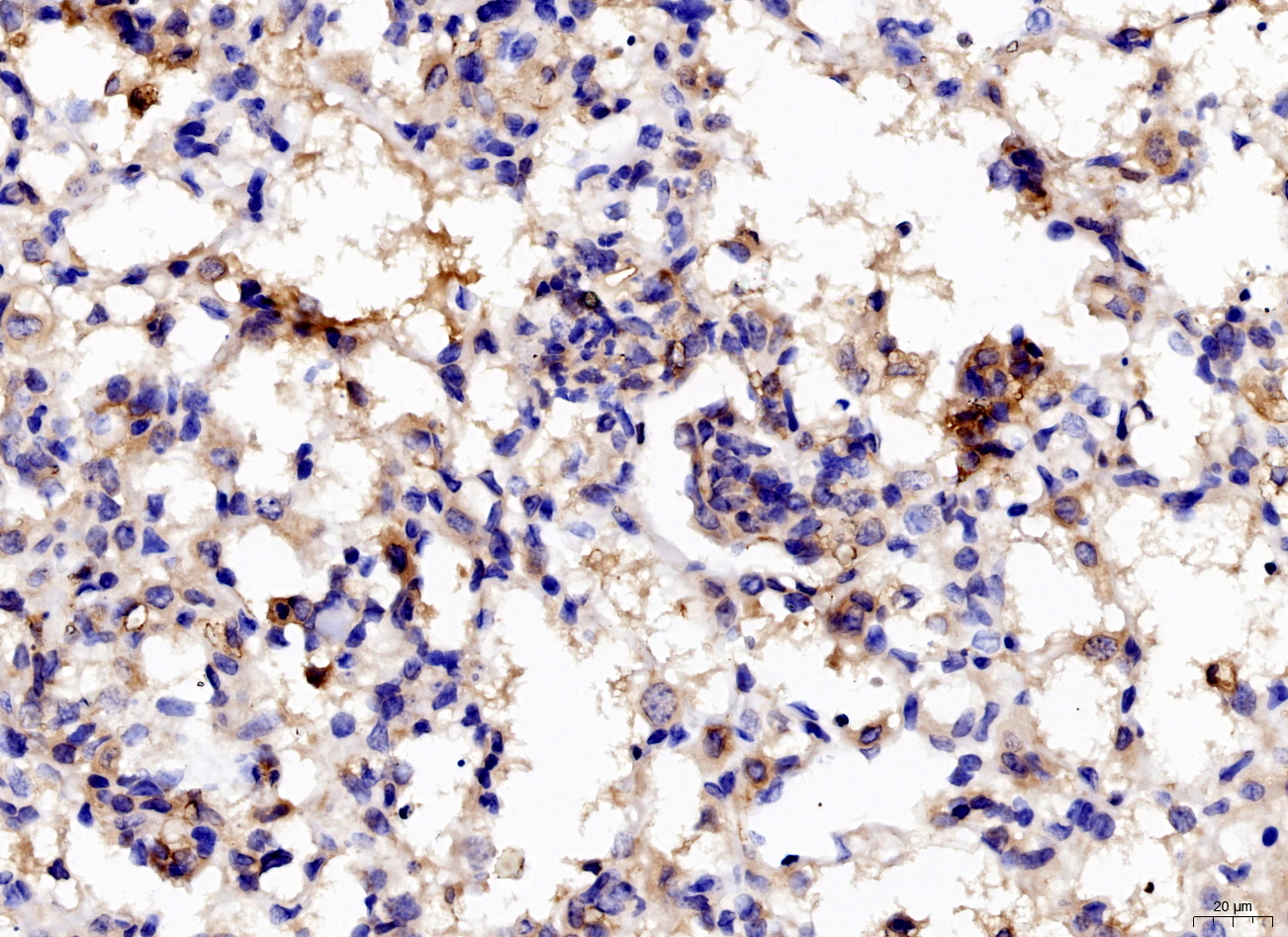

Supplement: Supplementary file 4 — Supporting File 4: advs73867‐sup‐0001‐FiguresData.zip. [file ADVS-13-e19191-s001.zip › Supporting information Figure1-10/Figure 2/Figure 2J/8 week Silica/SCRS-Ferritin-8w-Model-763_40.0x-4.jpg]

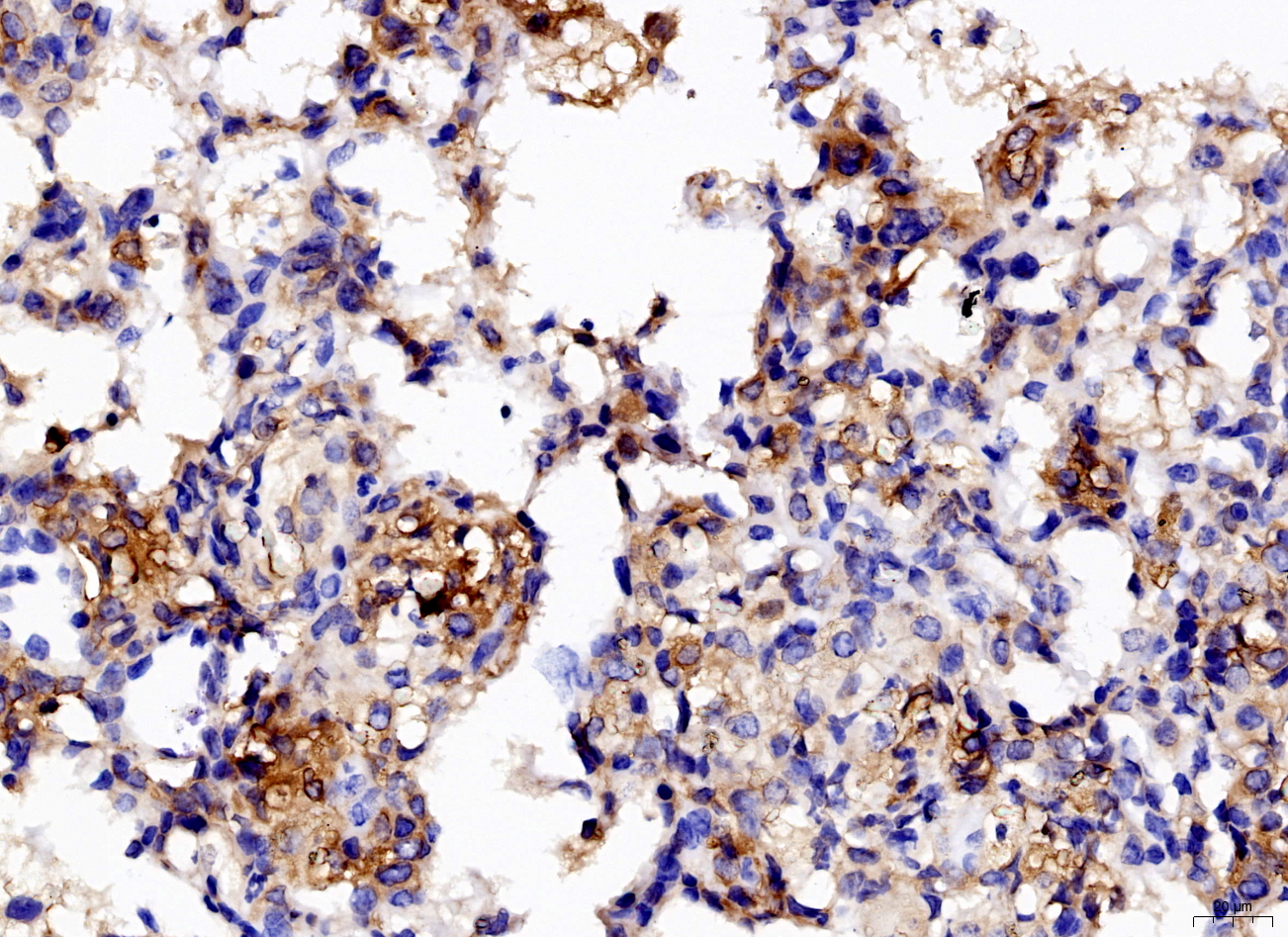

Supplement: Supplementary file 4 — Supporting File 4: advs73867‐sup‐0001‐FiguresData.zip. [file ADVS-13-e19191-s001.zip › Supporting information Figure1-10/Figure 2/Figure 2J/8 week Silica/SCRS-Ferritin-8w-Model-763_40.0x-5.jpg]

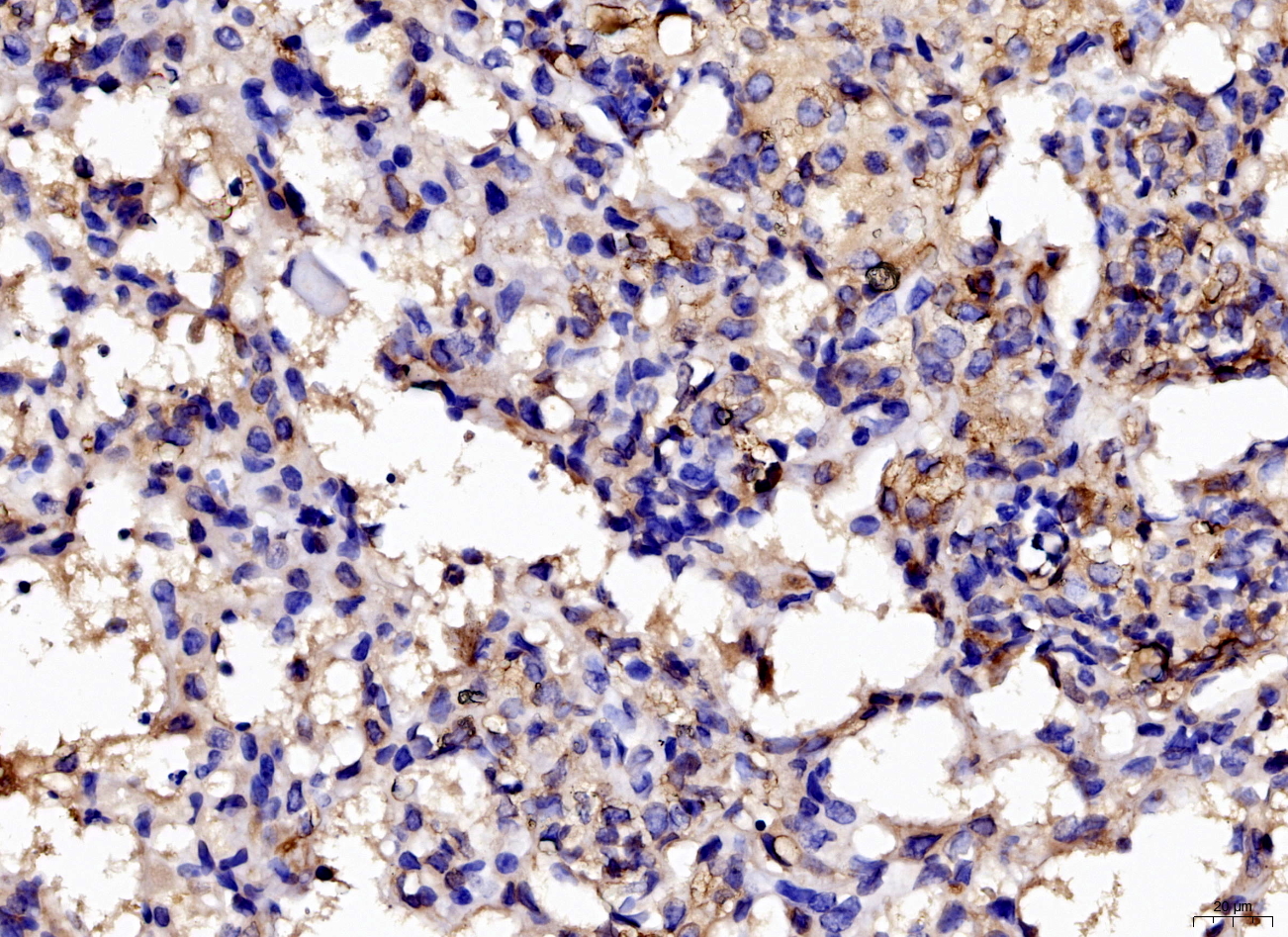

Supplement: Supplementary file 4 — Supporting File 4: advs73867‐sup‐0001‐FiguresData.zip. [file ADVS-13-e19191-s001.zip › Supporting information Figure1-10/Figure 2/Figure 2J/8 week Silica/SCRS-Ferritin-8w-Model-763_40.0x-6.jpg]

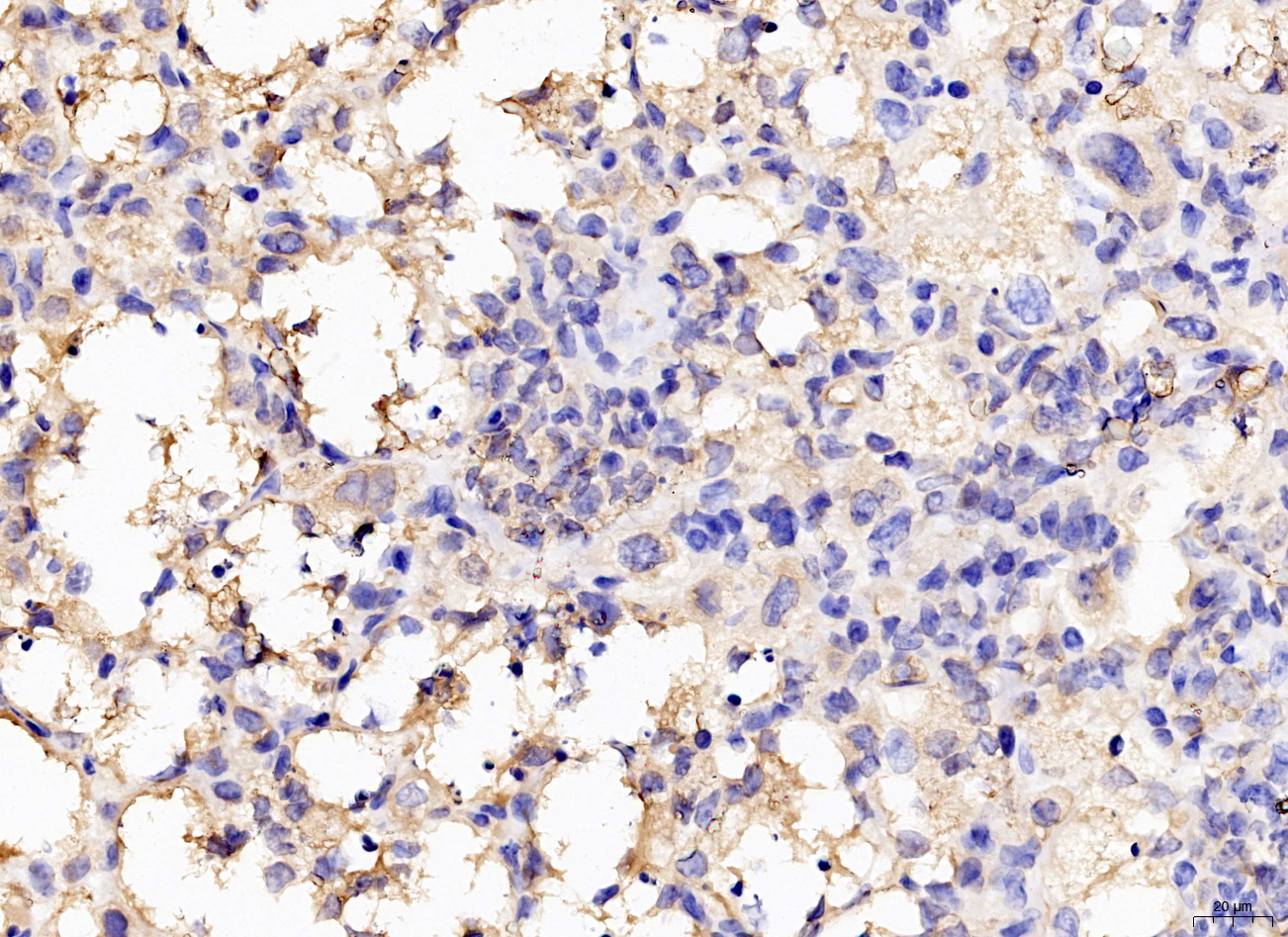

Supplement: Supplementary file 4 — Supporting File 4: advs73867‐sup‐0001‐FiguresData.zip. [file ADVS-13-e19191-s001.zip › Supporting information Figure1-10/Figure 2/Figure 2J/8 week Silica/SCRS-Ferritin-8w-Model-766_40.0x-7.jpg]

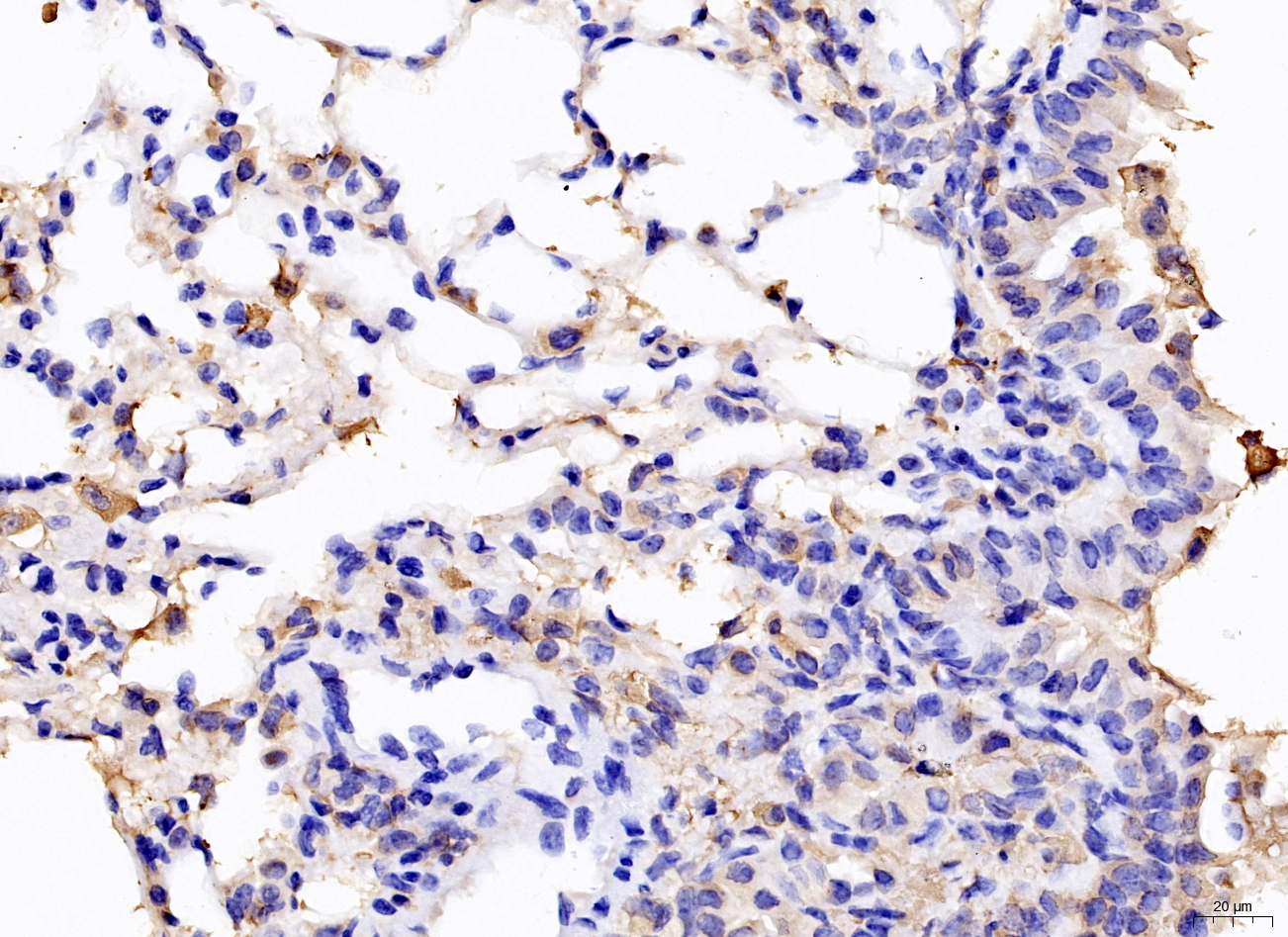

Supplement: Supplementary file 4 — Supporting File 4: advs73867‐sup‐0001‐FiguresData.zip. [file ADVS-13-e19191-s001.zip › Supporting information Figure1-10/Figure 2/Figure 2J/8 week Silica/SCRS-Ferritin-8w-Model-766_40.0x-8.jpg]

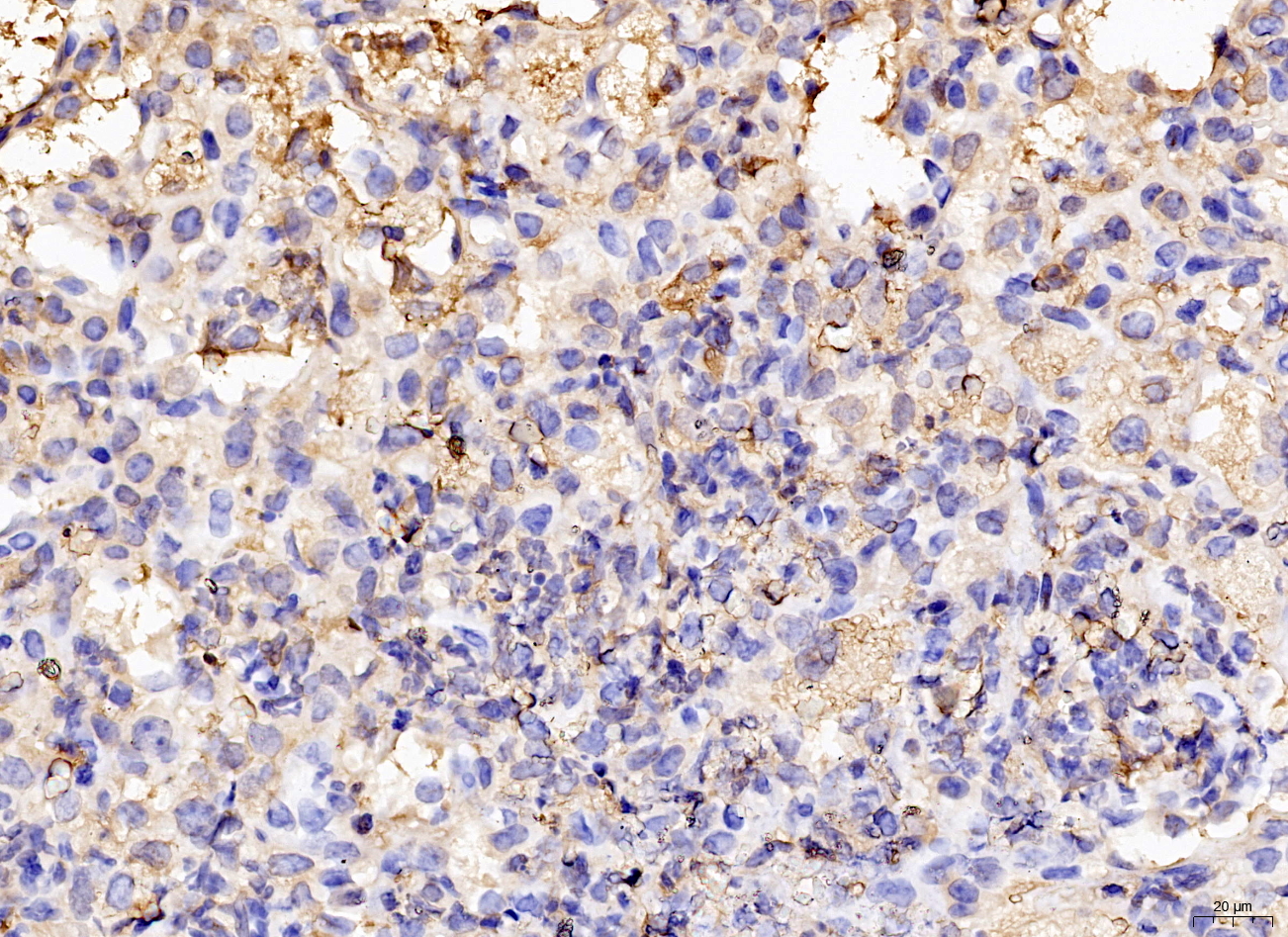

Supplement: Supplementary file 4 — Supporting File 4: advs73867‐sup‐0001‐FiguresData.zip. [file ADVS-13-e19191-s001.zip › Supporting information Figure1-10/Figure 2/Figure 2J/8 week Silica/SCRS-Ferritin-8w-Model-766_40.0x-9.jpg]

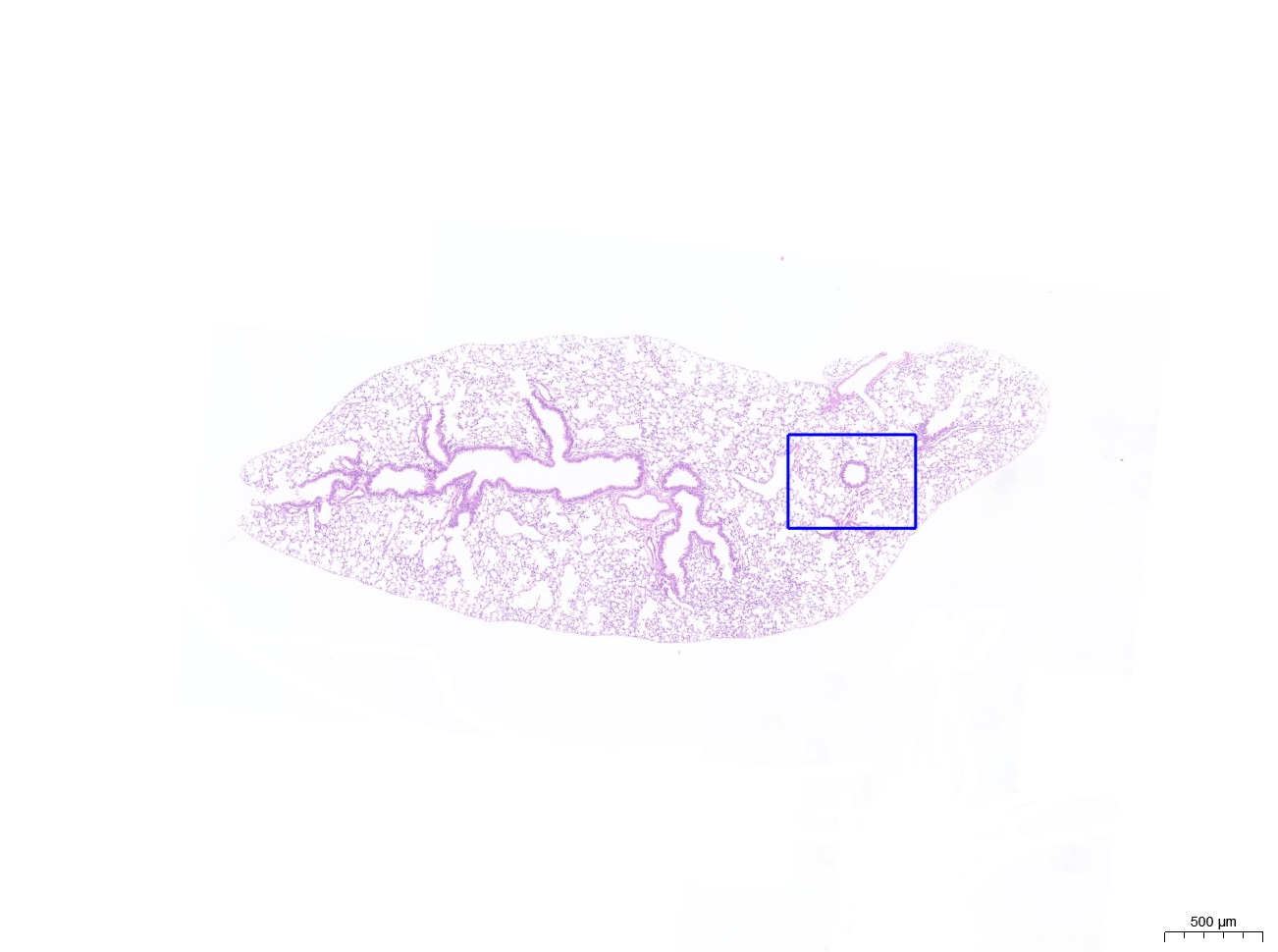

Supplement: Supplementary file 4 — Supporting File 4: advs73867‐sup‐0001‐FiguresData.zip. [file ADVS-13-e19191-s001.zip › Supporting information Figure1-10/Figure 3/Figure 3B/controli -105_2.0x.jpg]

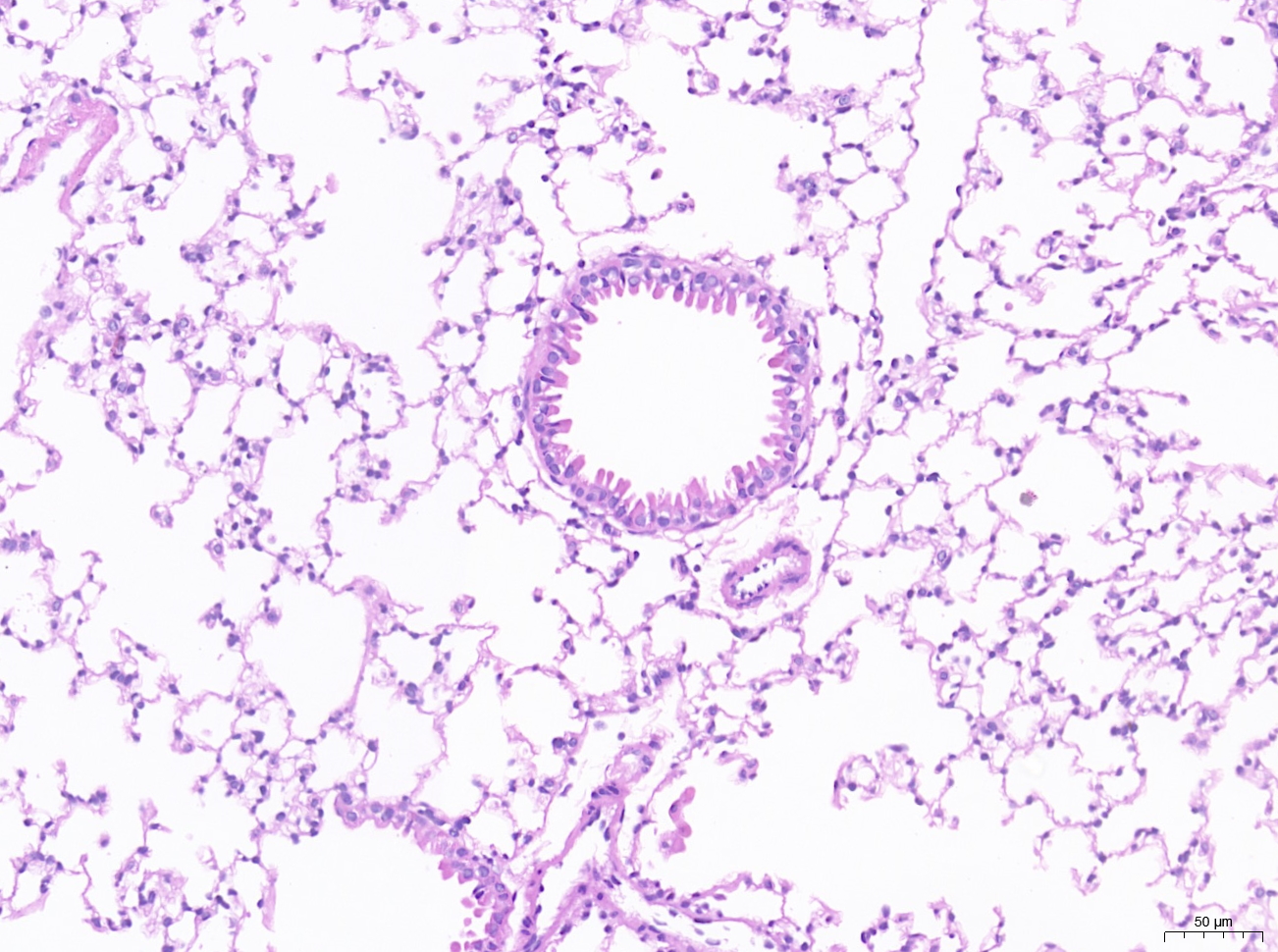

Supplement: Supplementary file 4 — Supporting File 4: advs73867‐sup‐0001‐FiguresData.zip. [file ADVS-13-e19191-s001.zip › Supporting information Figure1-10/Figure 3/Figure 3B/controli -105_20.0x.jpg]

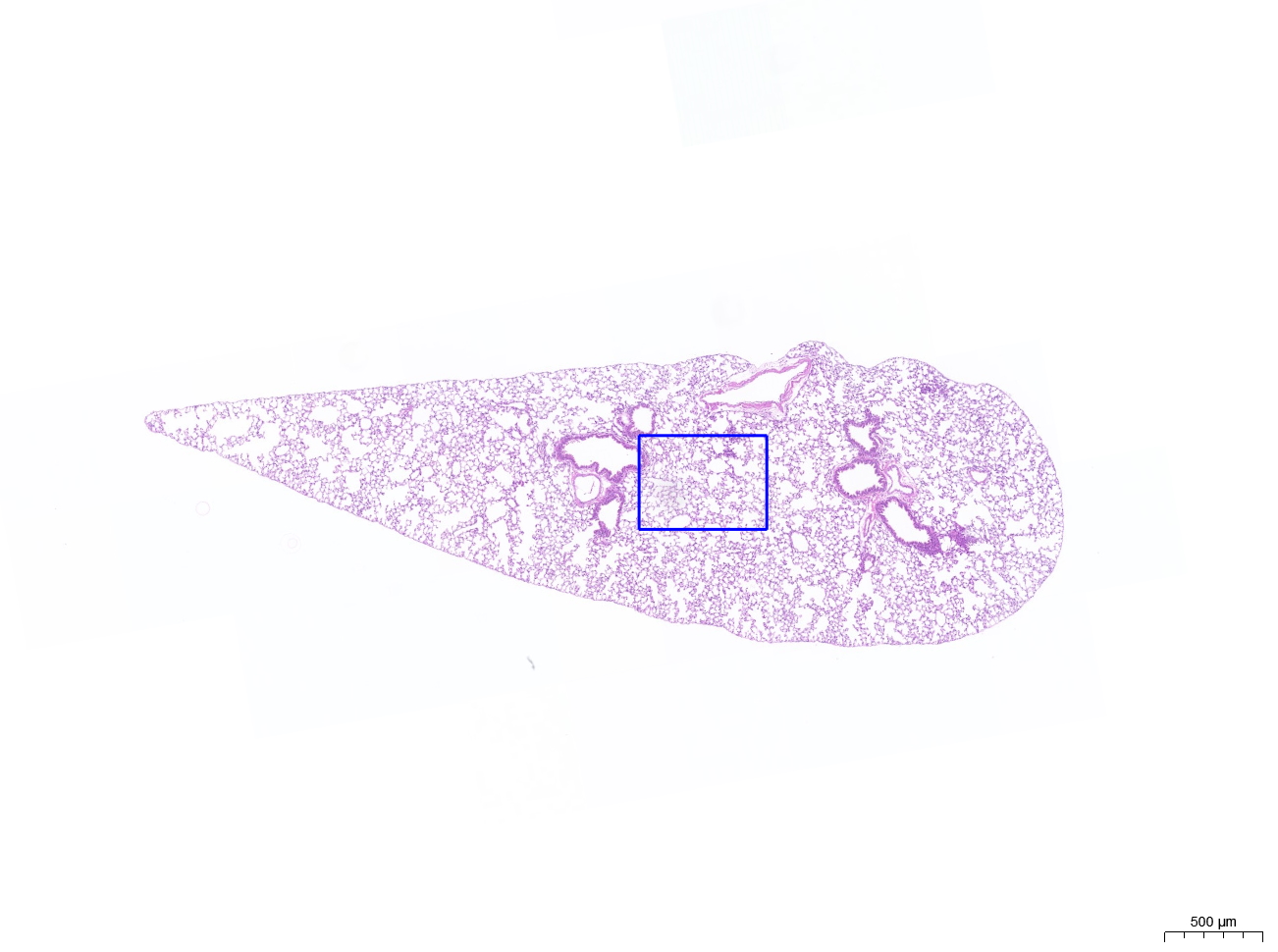

Supplement: Supplementary file 4 — Supporting File 4: advs73867‐sup‐0001‐FiguresData.zip. [file ADVS-13-e19191-s001.zip › Supporting information Figure1-10/Figure 3/Figure 3B/ferritin -122_2.0x.jpg]

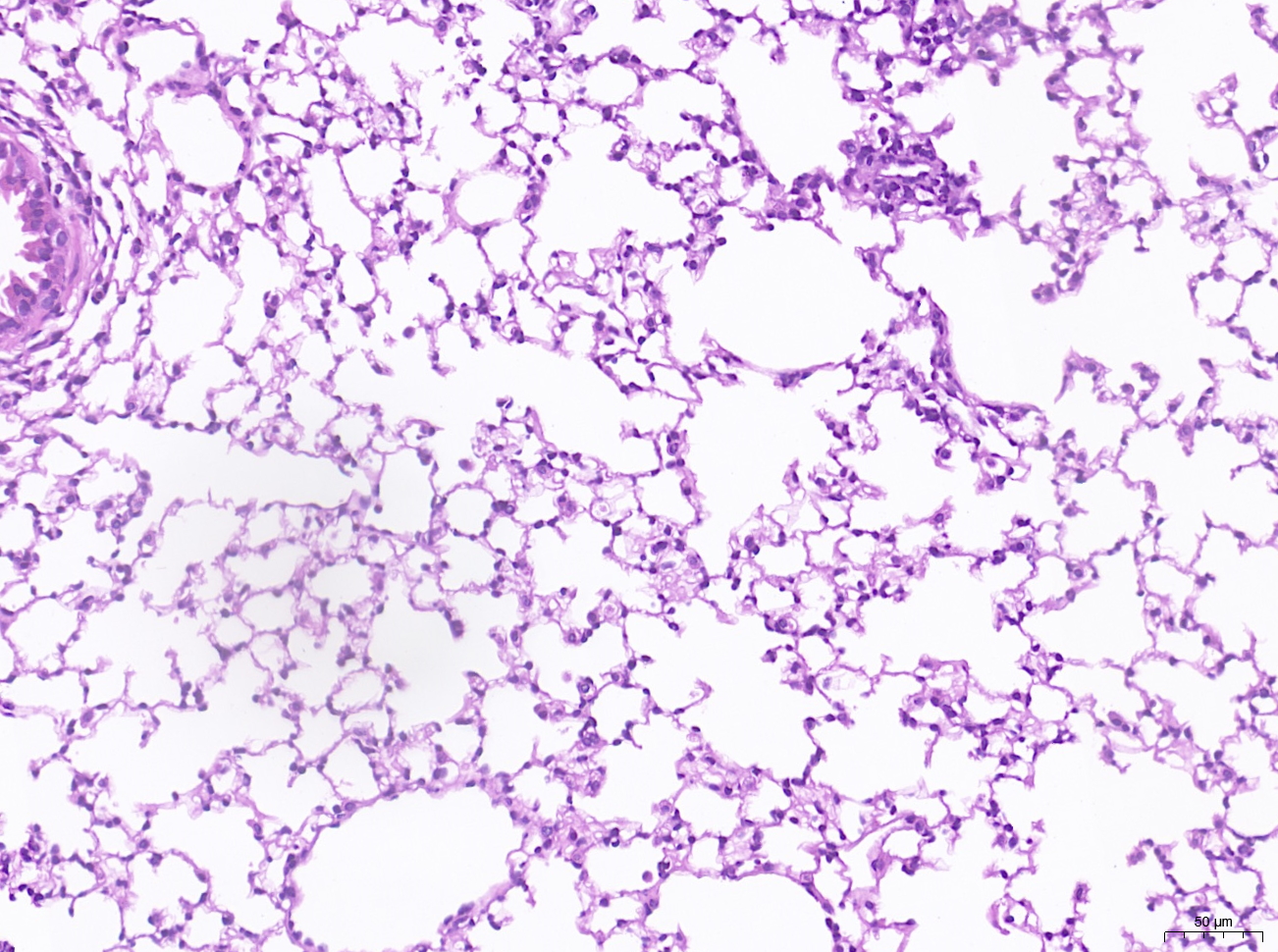

Supplement: Supplementary file 4 — Supporting File 4: advs73867‐sup‐0001‐FiguresData.zip. [file ADVS-13-e19191-s001.zip › Supporting information Figure1-10/Figure 3/Figure 3B/ferritin -122_20.0x.jpg]

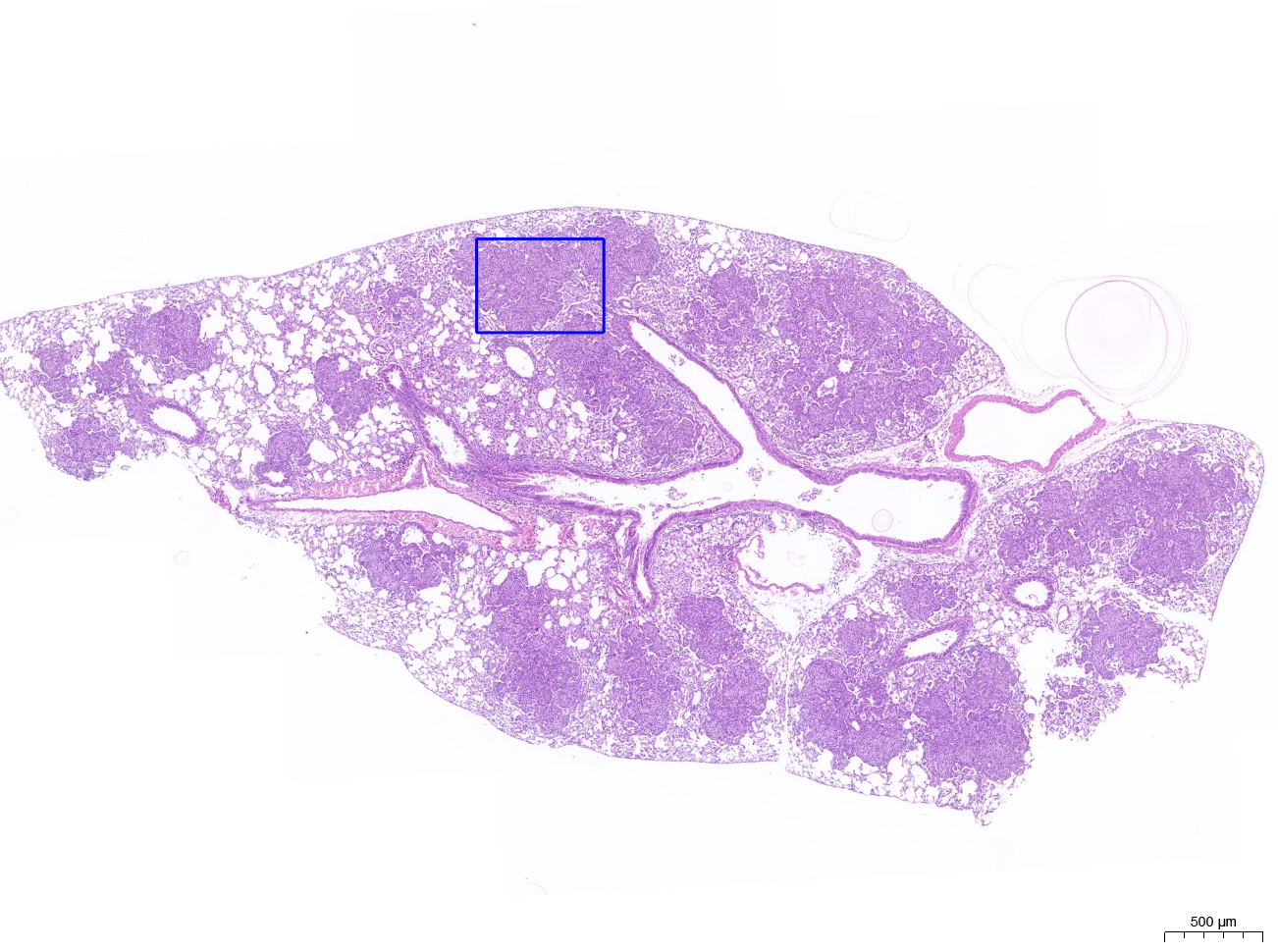

Supplement: Supplementary file 4 — Supporting File 4: advs73867‐sup‐0001‐FiguresData.zip. [file ADVS-13-e19191-s001.zip › Supporting information Figure1-10/Figure 3/Figure 3B/Silica+ferriti -120_2.0x.jpg]

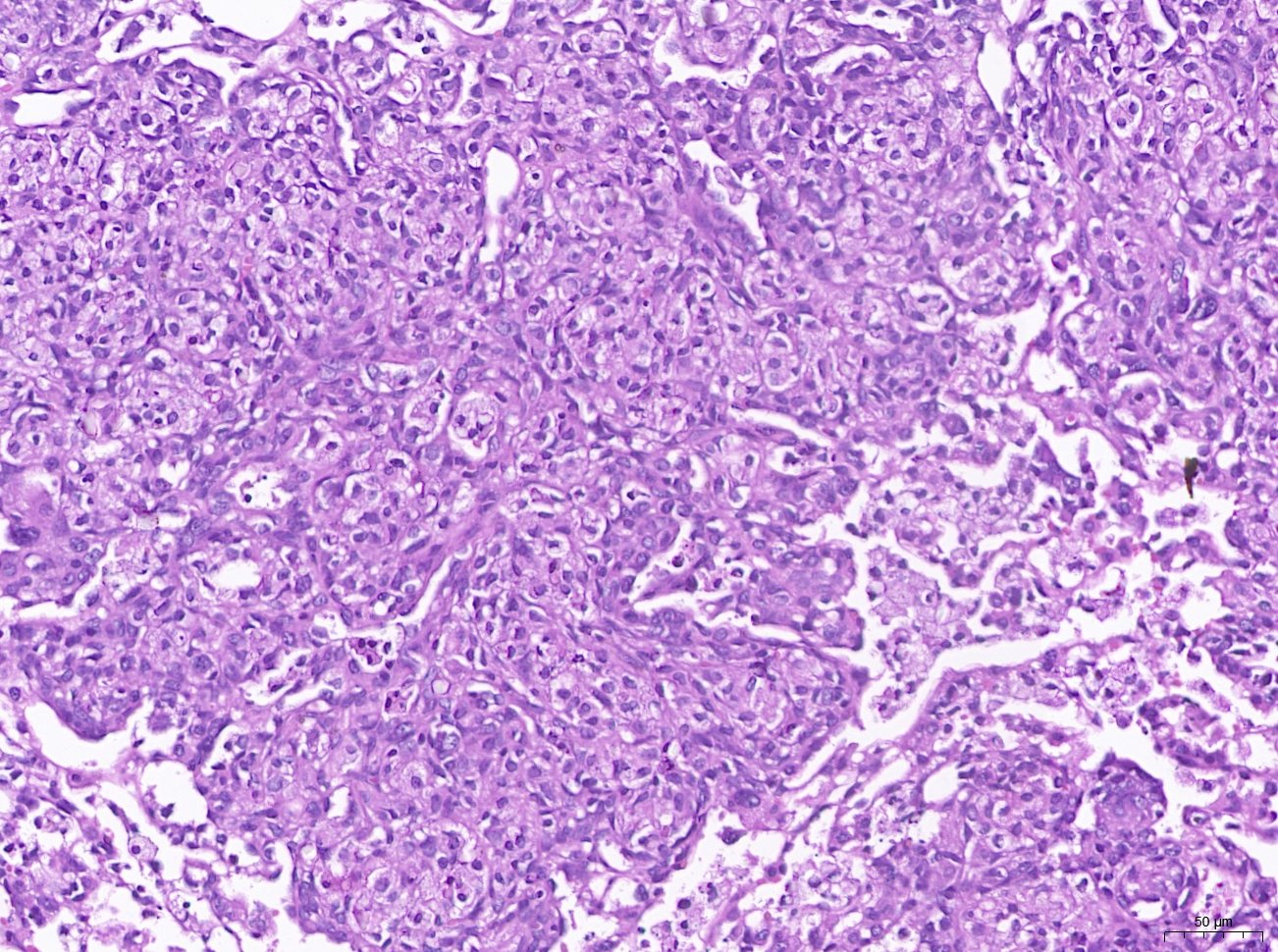

Supplement: Supplementary file 4 — Supporting File 4: advs73867‐sup‐0001‐FiguresData.zip. [file ADVS-13-e19191-s001.zip › Supporting information Figure1-10/Figure 3/Figure 3B/Silica+ferriti -120_20.0x.jpg]

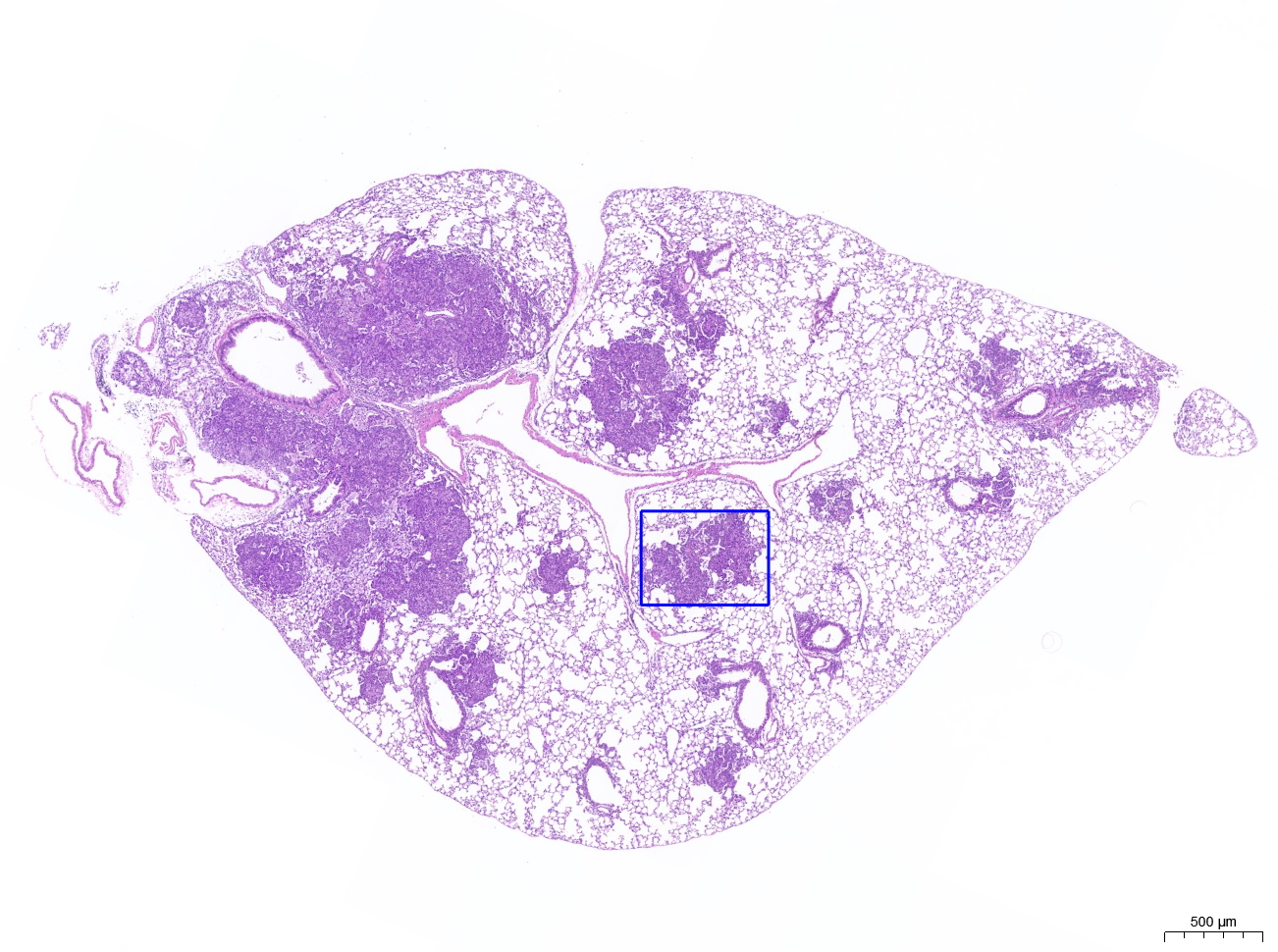

Supplement: Supplementary file 4 — Supporting File 4: advs73867‐sup‐0001‐FiguresData.zip. [file ADVS-13-e19191-s001.zip › Supporting information Figure1-10/Figure 3/Figure 3B/Silica-114_2.0x.jpg]

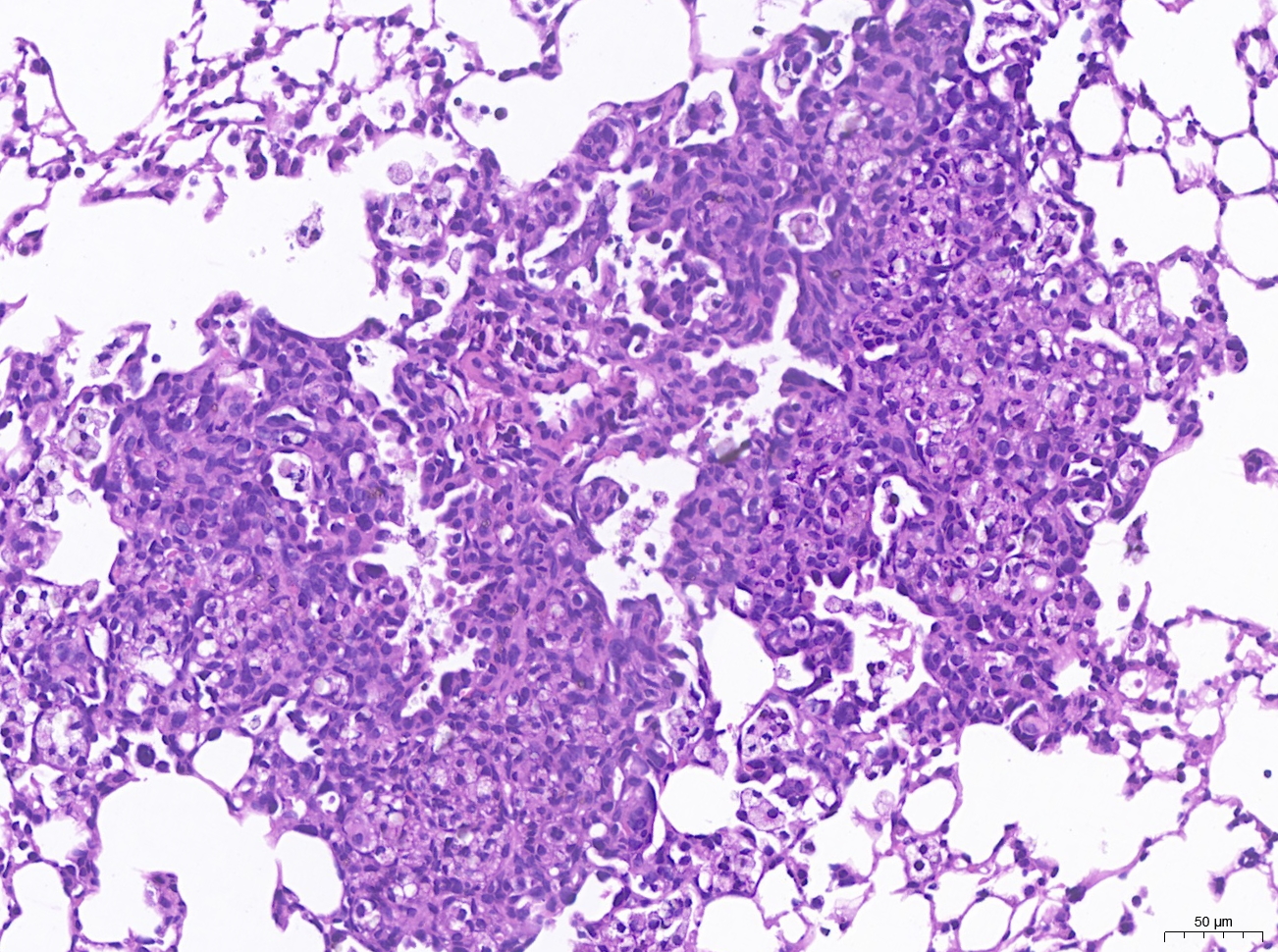

Supplement: Supplementary file 4 — Supporting File 4: advs73867‐sup‐0001‐FiguresData.zip. [file ADVS-13-e19191-s001.zip › Supporting information Figure1-10/Figure 3/Figure 3B/Silica-114_20.0x.jpg]

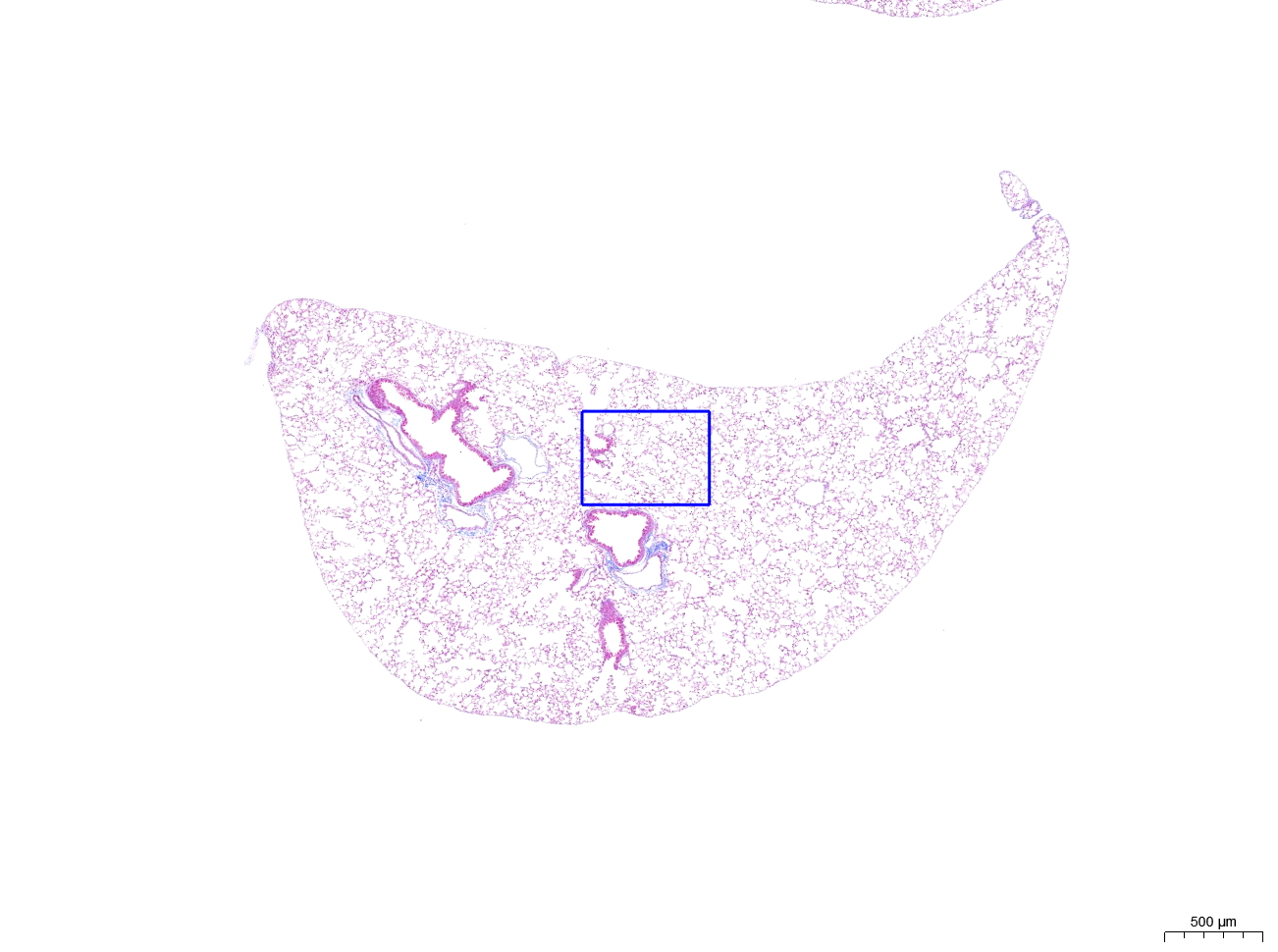

Supplement: Supplementary file 4 — Supporting File 4: advs73867‐sup‐0001‐FiguresData.zip. [file ADVS-13-e19191-s001.zip › Supporting information Figure1-10/Figure 3/Figure 3C/Control-105_2.0x.jpg]

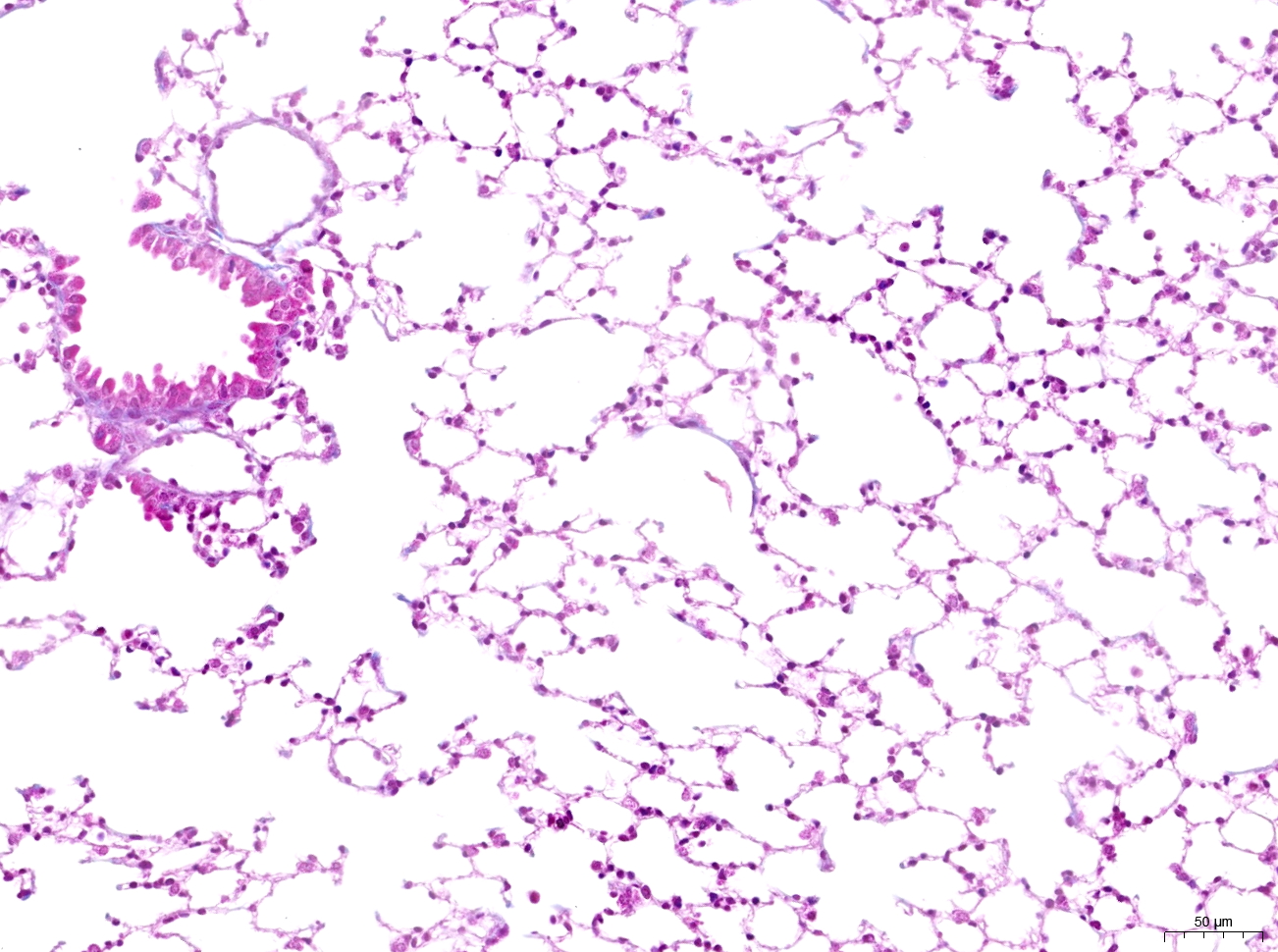

Supplement: Supplementary file 4 — Supporting File 4: advs73867‐sup‐0001‐FiguresData.zip. [file ADVS-13-e19191-s001.zip › Supporting information Figure1-10/Figure 3/Figure 3C/Control-105_20.0x.jpg]

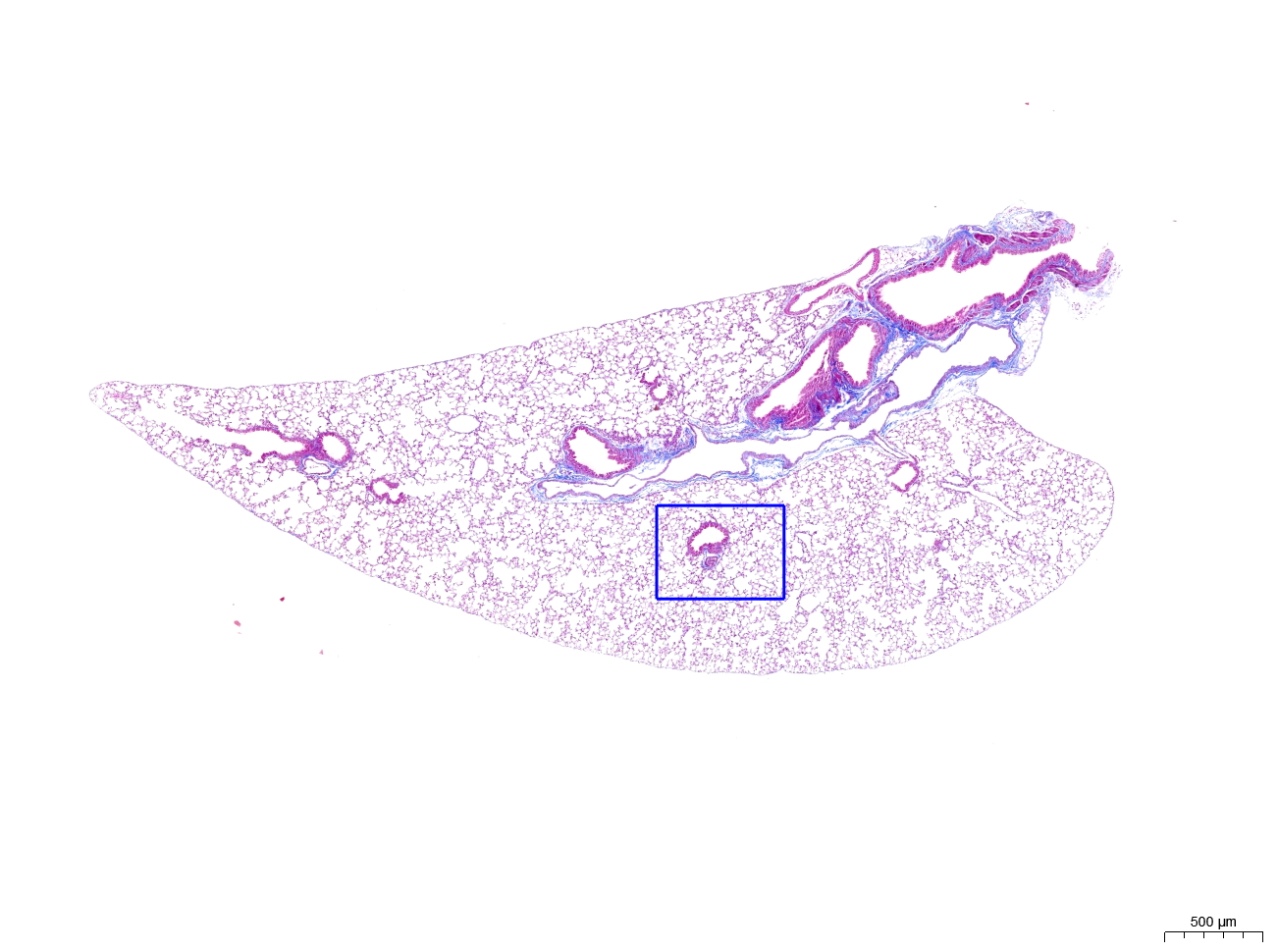

Supplement: Supplementary file 4 — Supporting File 4: advs73867‐sup‐0001‐FiguresData.zip. [file ADVS-13-e19191-s001.zip › Supporting information Figure1-10/Figure 3/Figure 3C/Ferritin-122_2.0x.jpg]

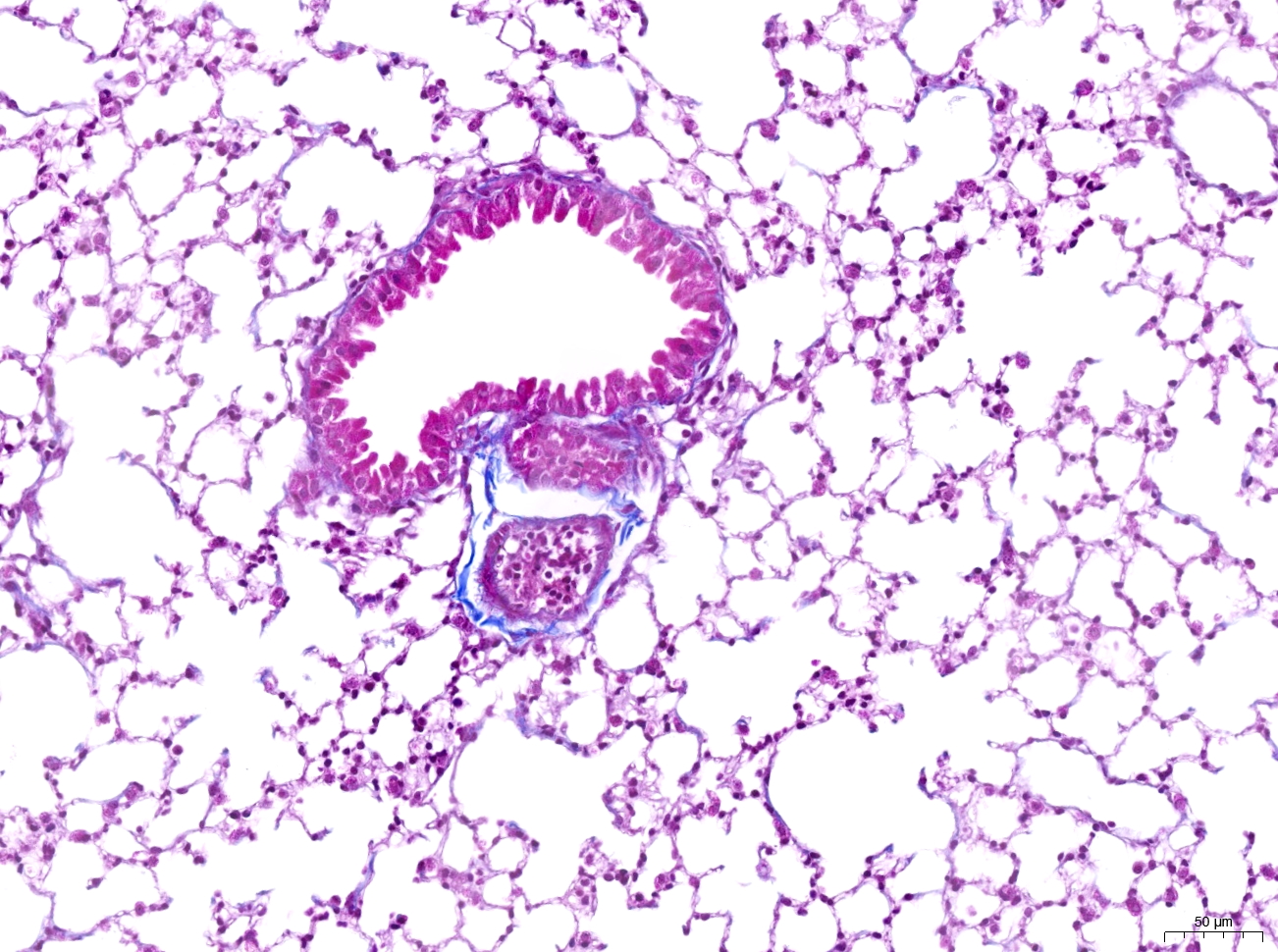

Supplement: Supplementary file 4 — Supporting File 4: advs73867‐sup‐0001‐FiguresData.zip. [file ADVS-13-e19191-s001.zip › Supporting information Figure1-10/Figure 3/Figure 3C/Ferritin-122_20.0x.jpg]

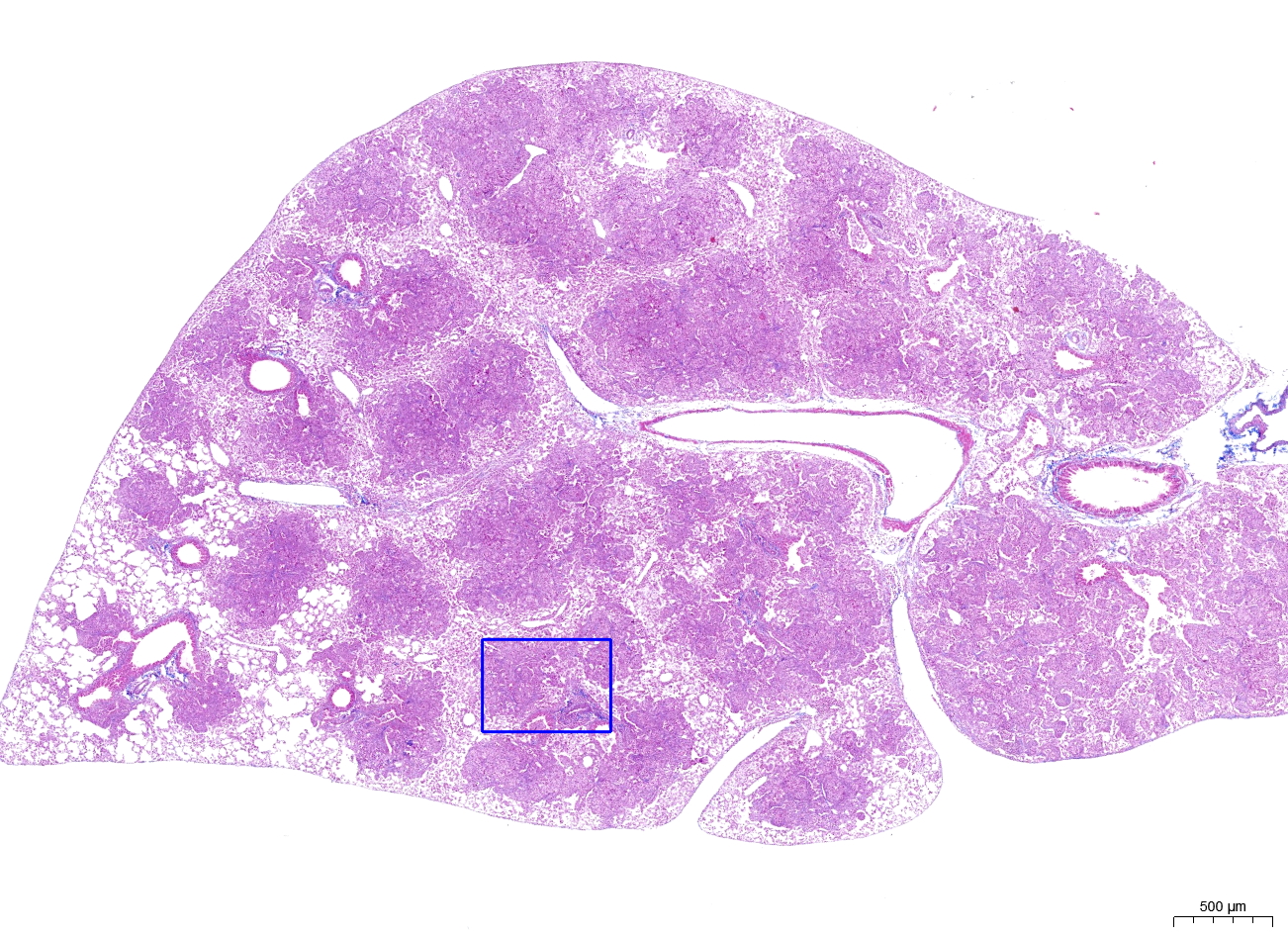

Supplement: Supplementary file 4 — Supporting File 4: advs73867‐sup‐0001‐FiguresData.zip. [file ADVS-13-e19191-s001.zip › Supporting information Figure1-10/Figure 3/Figure 3C/Silica+Ferritin-120_2.0x.jpg]

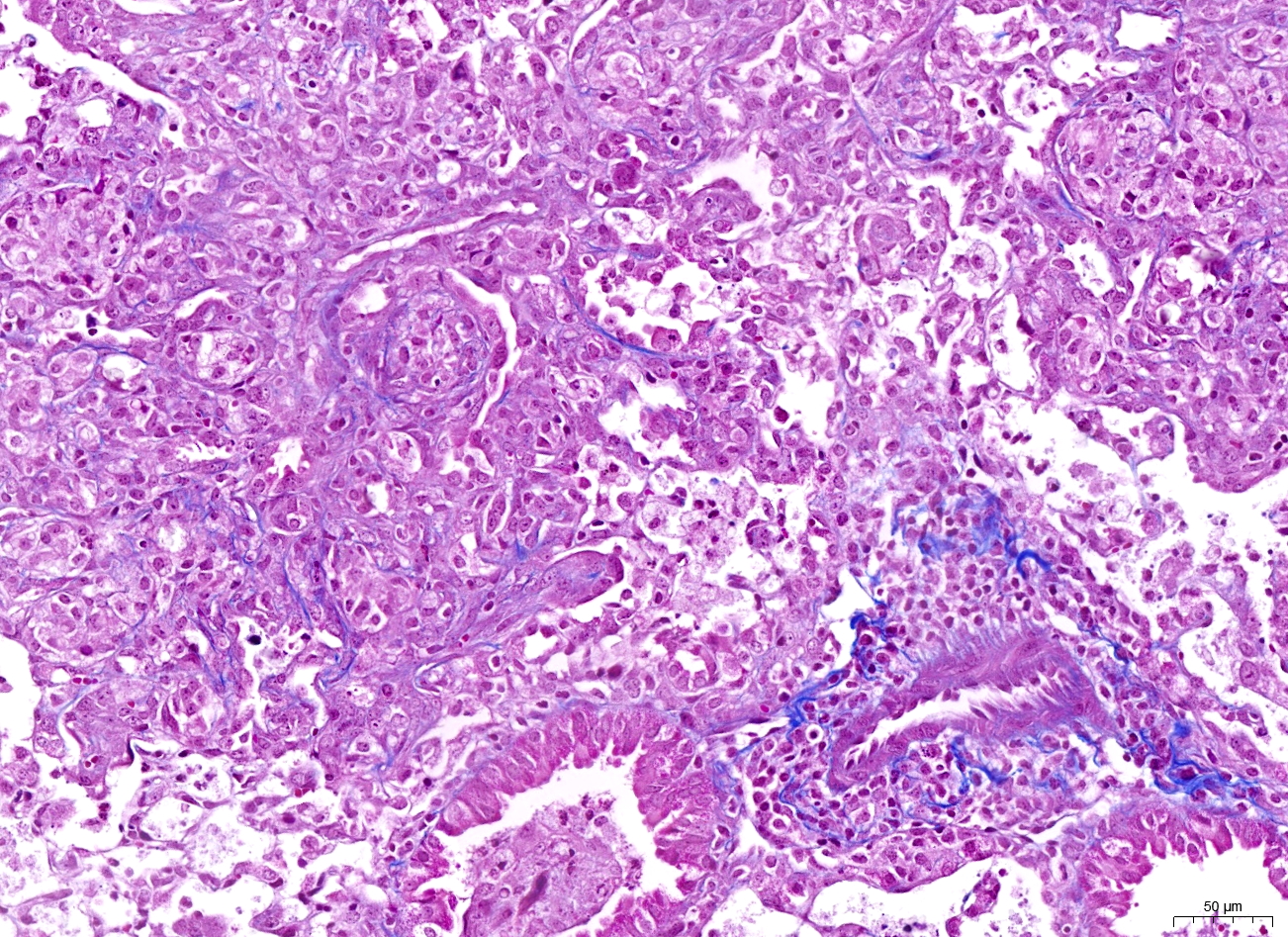

Supplement: Supplementary file 4 — Supporting File 4: advs73867‐sup‐0001‐FiguresData.zip. [file ADVS-13-e19191-s001.zip › Supporting information Figure1-10/Figure 3/Figure 3C/Silica+Ferritin-120_20.0x.jpg]

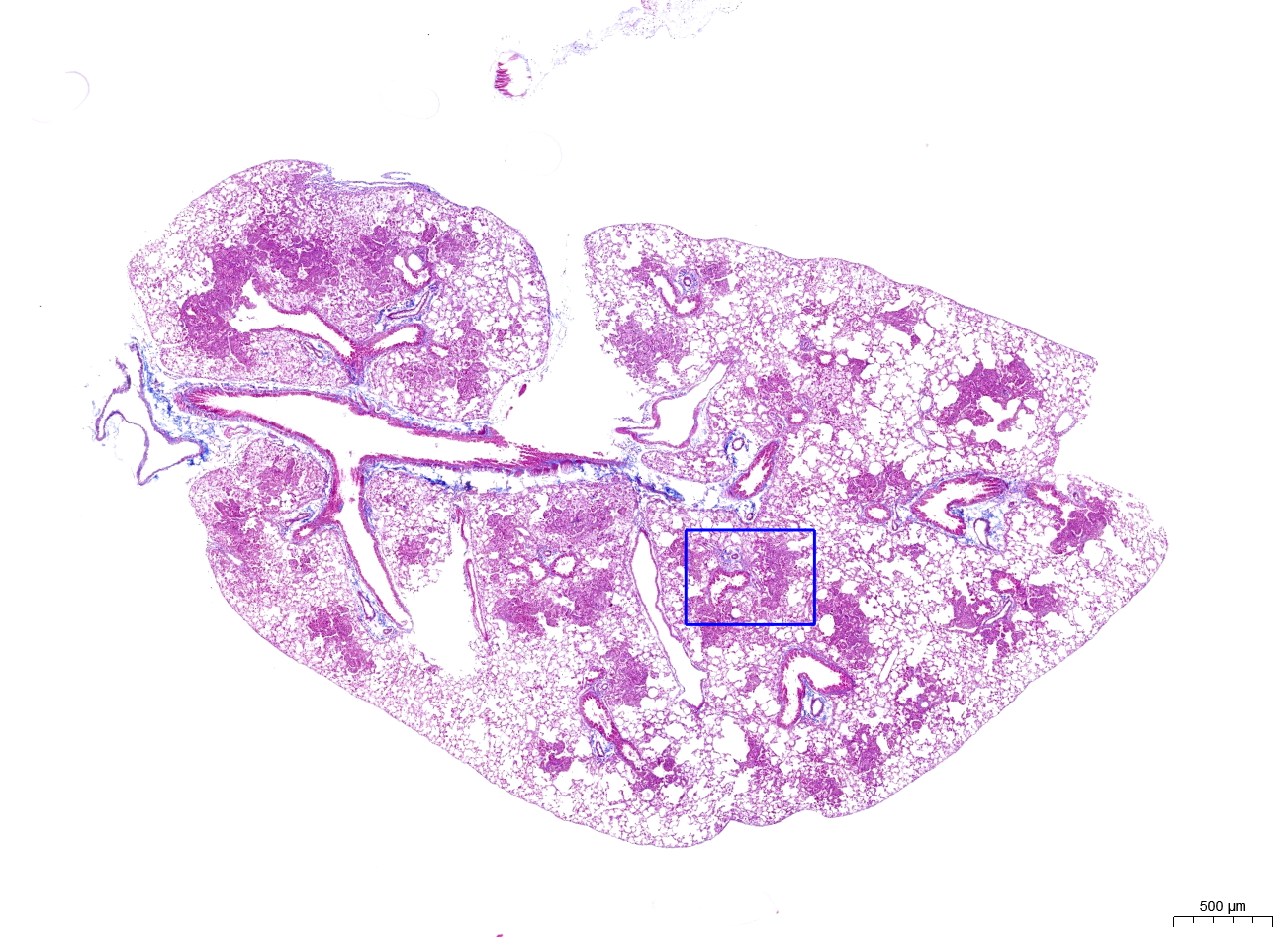

Supplement: Supplementary file 4 — Supporting File 4: advs73867‐sup‐0001‐FiguresData.zip. [file ADVS-13-e19191-s001.zip › Supporting information Figure1-10/Figure 3/Figure 3C/silica-112_2.0x.jpg]

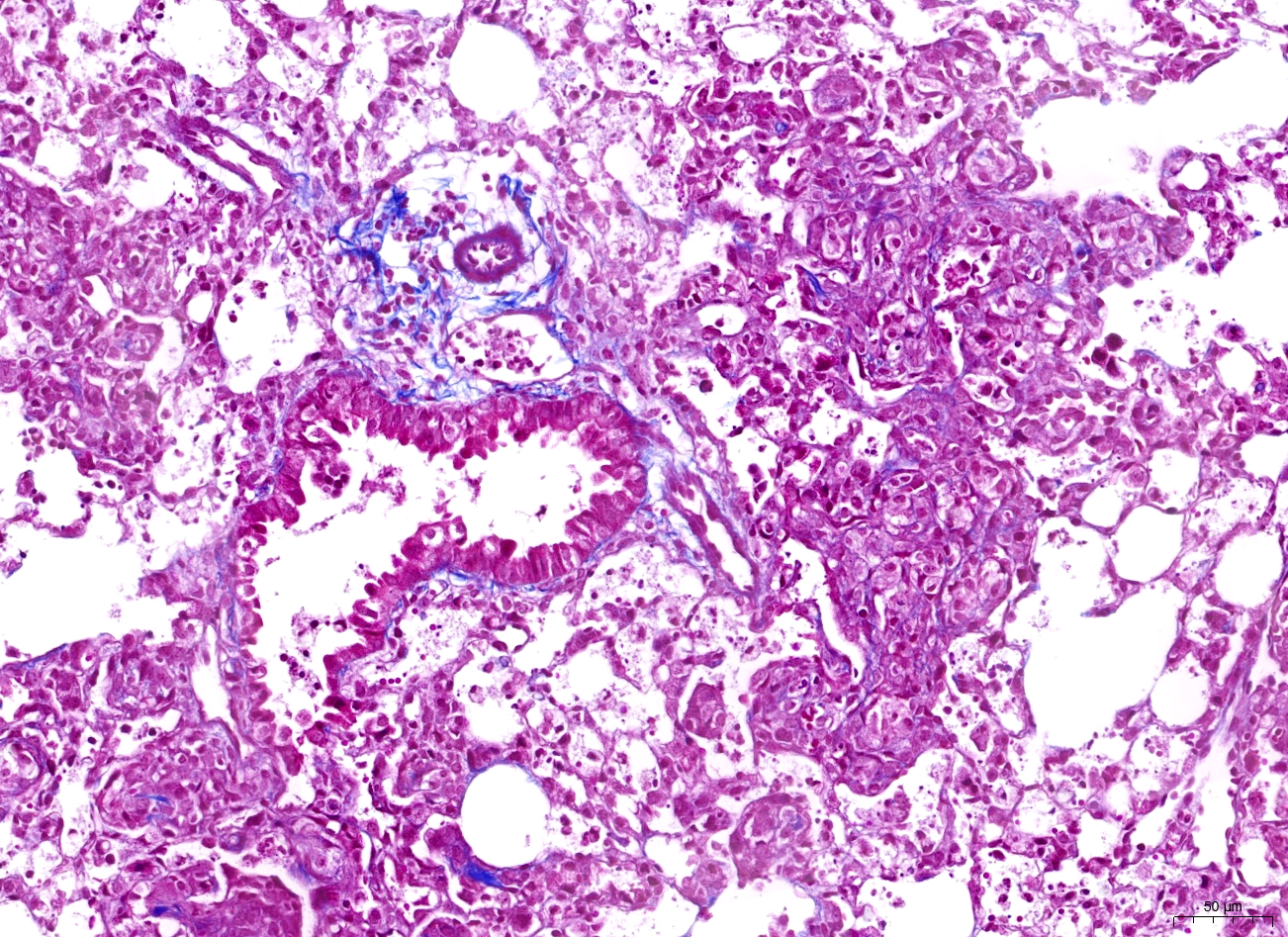

Supplement: Supplementary file 4 — Supporting File 4: advs73867‐sup‐0001‐FiguresData.zip. [file ADVS-13-e19191-s001.zip › Supporting information Figure1-10/Figure 3/Figure 3C/silica-112_20.0x.jpg]

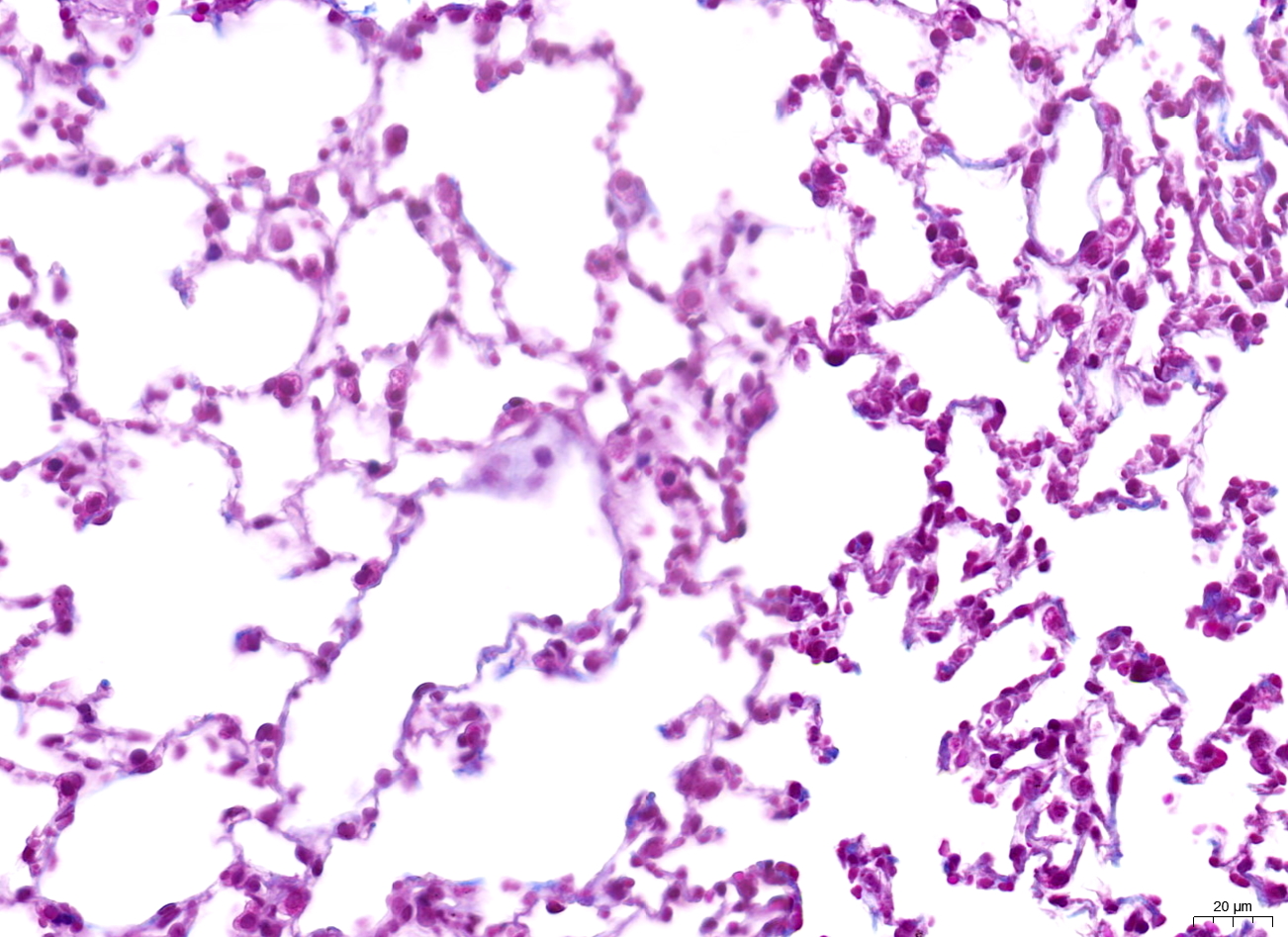

Supplement: Supplementary file 4 — Supporting File 4: advs73867‐sup‐0001‐FiguresData.zip. [file ADVS-13-e19191-s001.zip › Supporting information Figure1-10/Figure 3/Figure 3D/control/Control-101_40.0x.jpg]

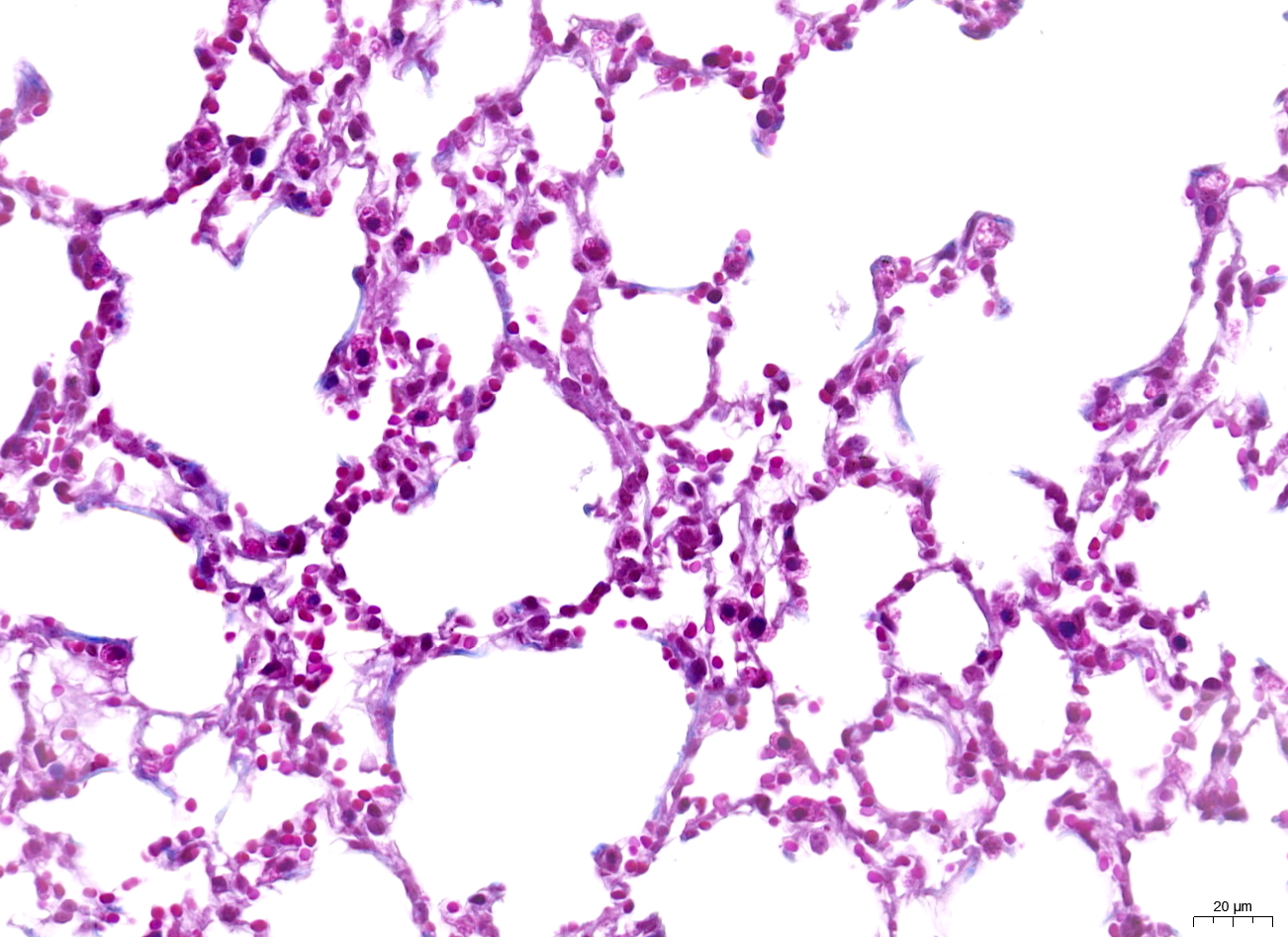

Supplement: Supplementary file 4 — Supporting File 4: advs73867‐sup‐0001‐FiguresData.zip. [file ADVS-13-e19191-s001.zip › Supporting information Figure1-10/Figure 3/Figure 3D/control/Control-101_40.0x-2.jpg]

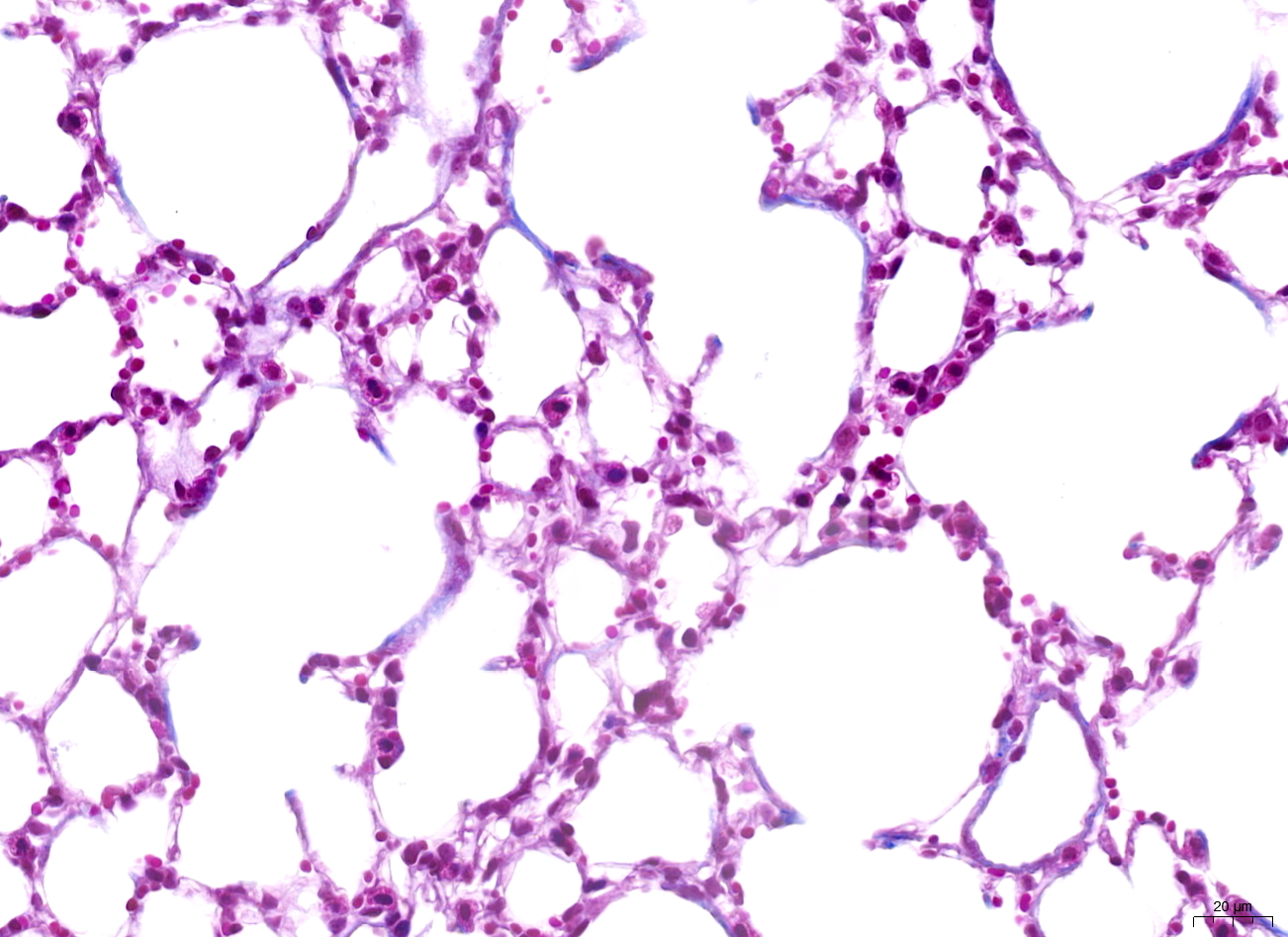

Supplement: Supplementary file 4 — Supporting File 4: advs73867‐sup‐0001‐FiguresData.zip. [file ADVS-13-e19191-s001.zip › Supporting information Figure1-10/Figure 3/Figure 3D/control/Control-101_40.0x-3.jpg]

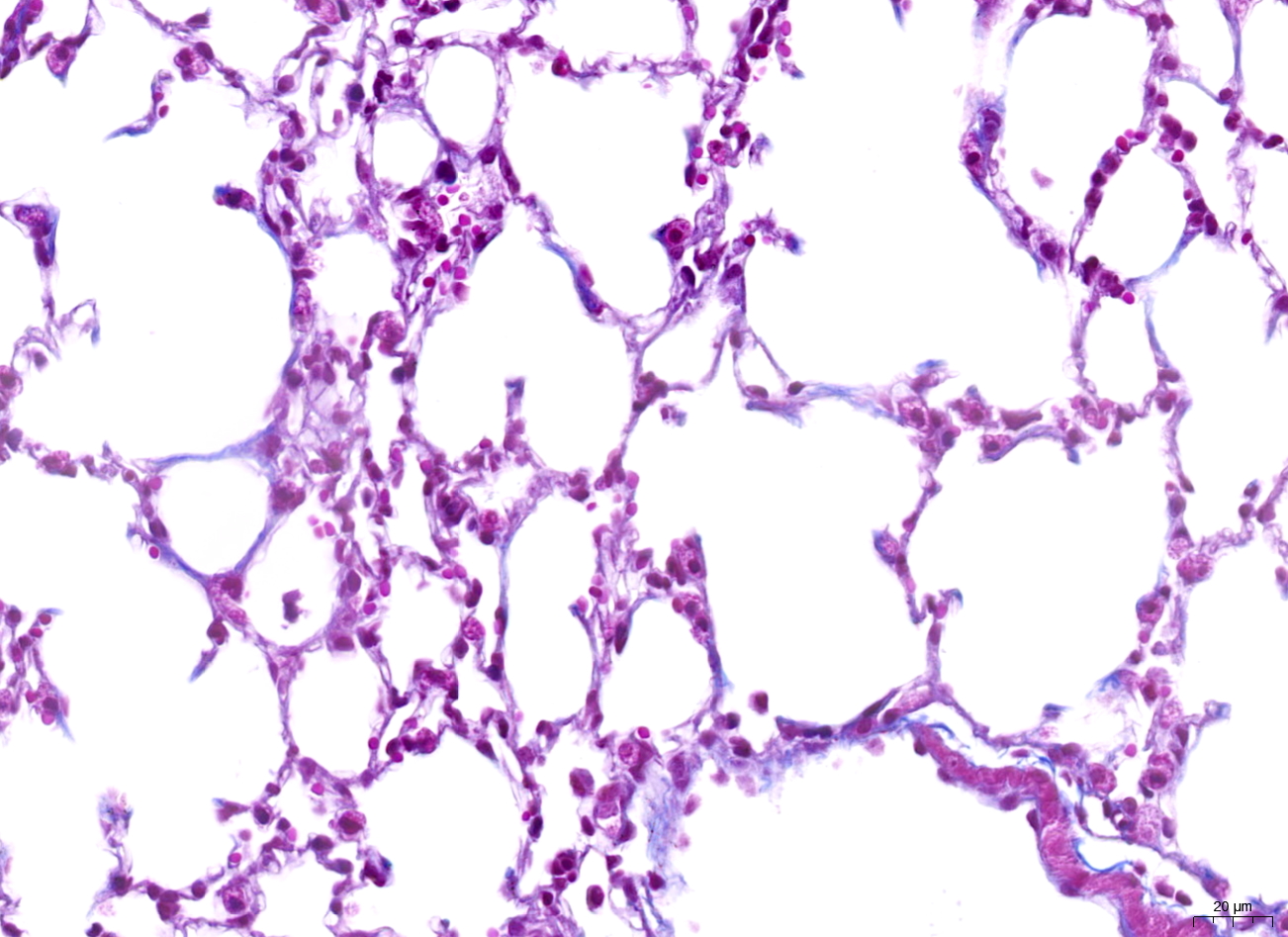

Supplement: Supplementary file 4 — Supporting File 4: advs73867‐sup‐0001‐FiguresData.zip. [file ADVS-13-e19191-s001.zip › Supporting information Figure1-10/Figure 3/Figure 3D/control/Control-104_40.0x.jpg]

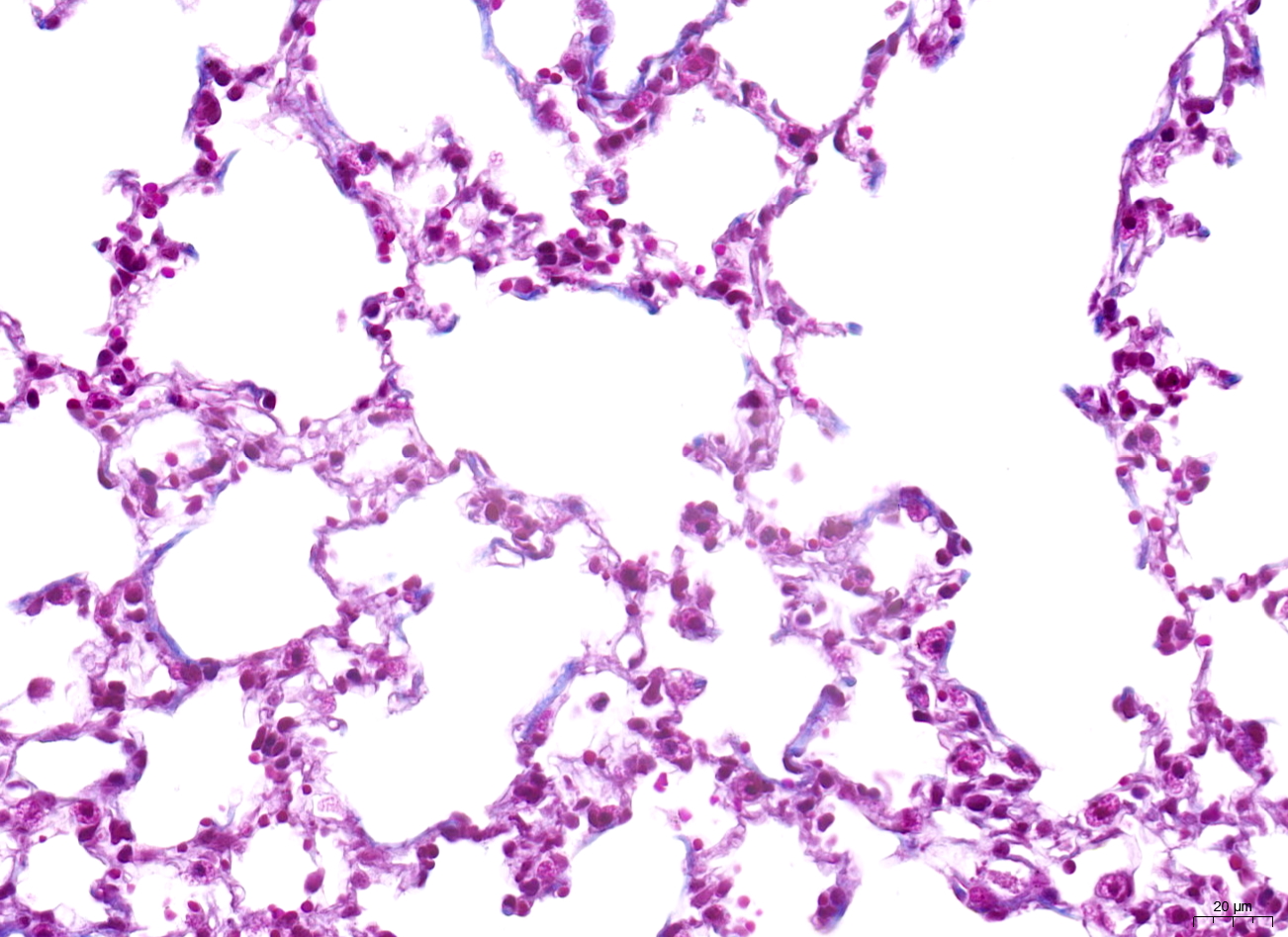

Supplement: Supplementary file 4 — Supporting File 4: advs73867‐sup‐0001‐FiguresData.zip. [file ADVS-13-e19191-s001.zip › Supporting information Figure1-10/Figure 3/Figure 3D/control/Control-104_40.0x-2.jpg]

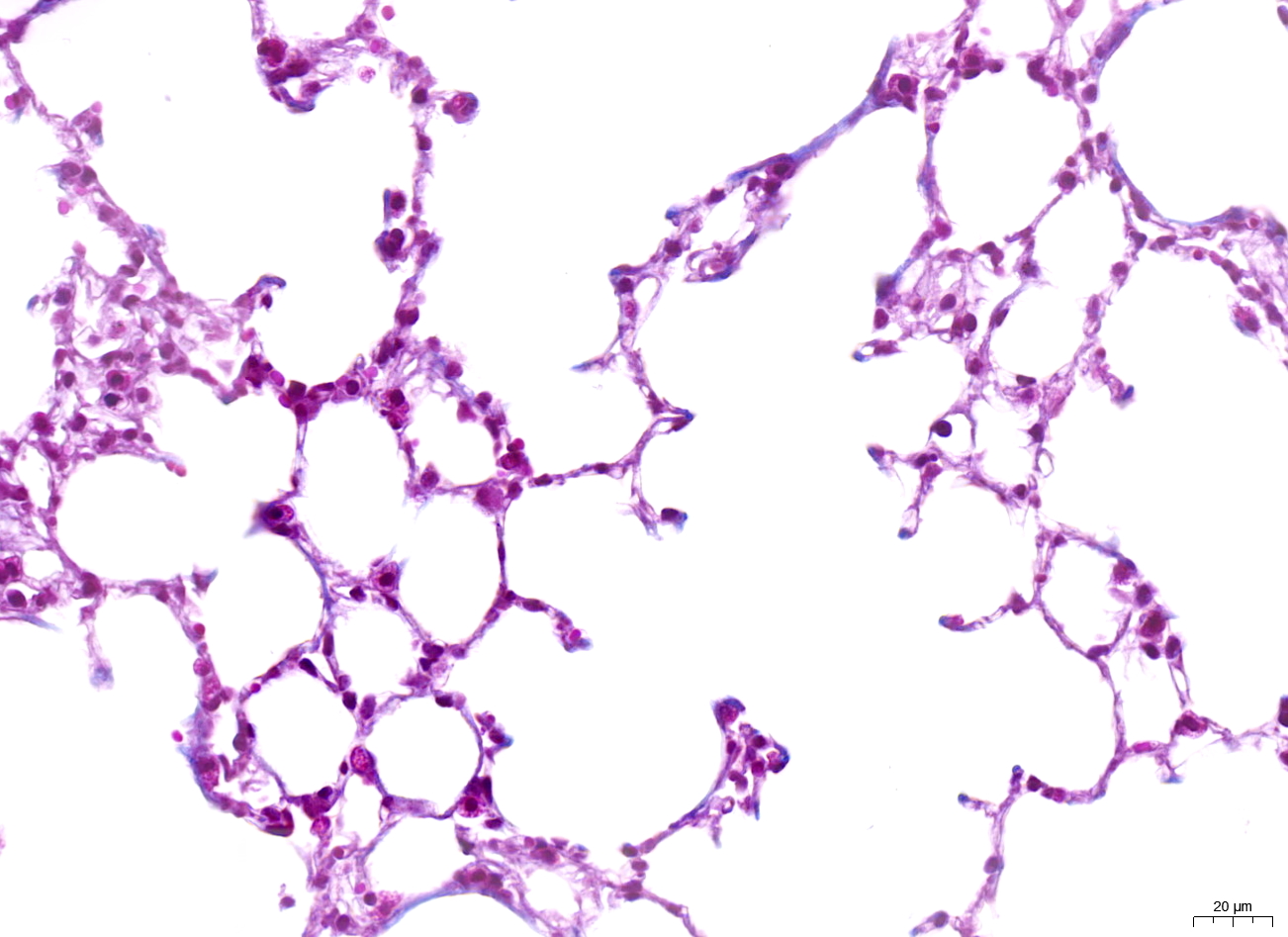

Supplement: Supplementary file 4 — Supporting File 4: advs73867‐sup‐0001‐FiguresData.zip. [file ADVS-13-e19191-s001.zip › Supporting information Figure1-10/Figure 3/Figure 3D/control/Control-104_40.0x-3.jpg]

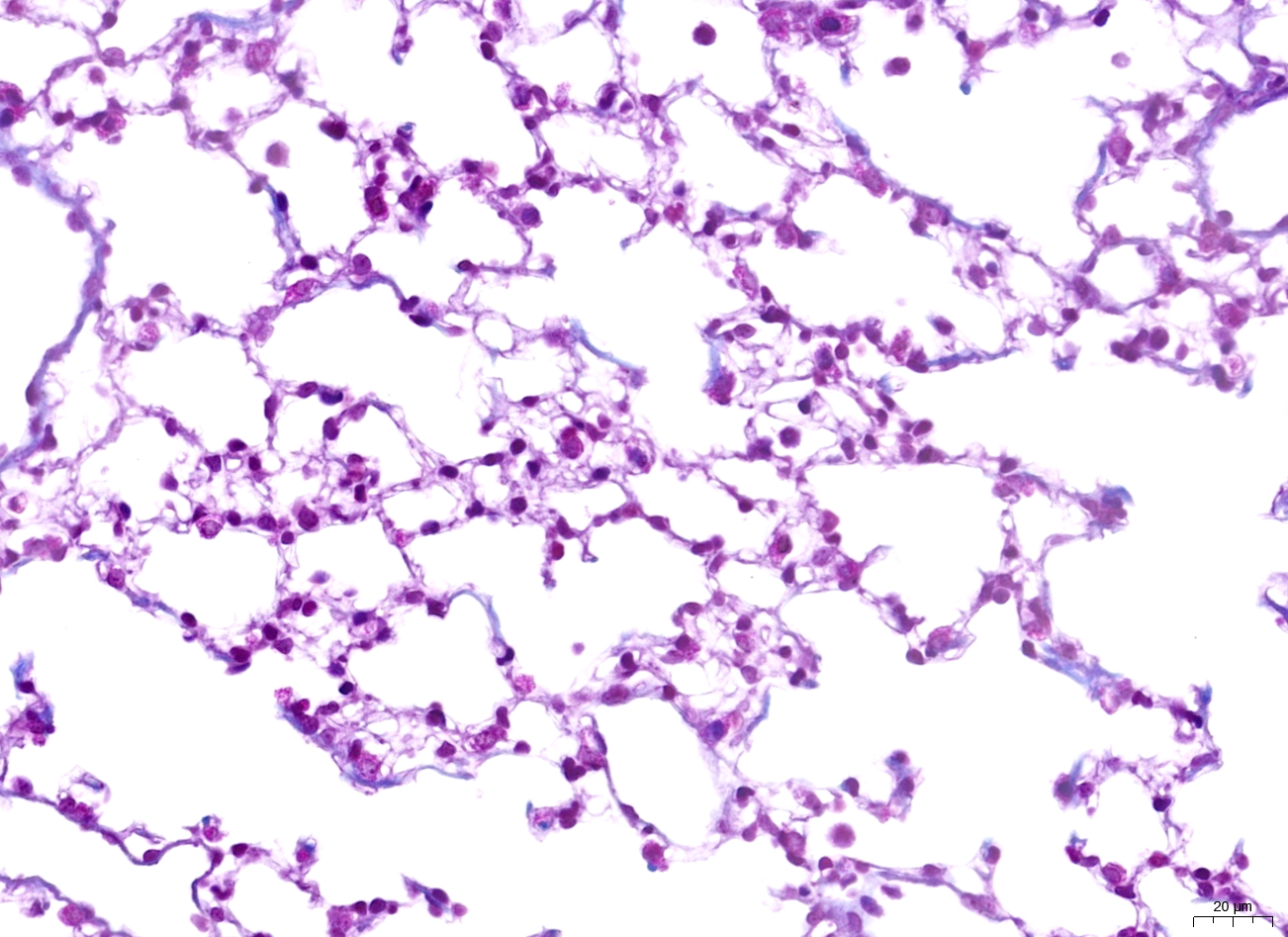

Supplement: Supplementary file 4 — Supporting File 4: advs73867‐sup‐0001‐FiguresData.zip. [file ADVS-13-e19191-s001.zip › Supporting information Figure1-10/Figure 3/Figure 3D/control/Control-105_40.0x.jpg]

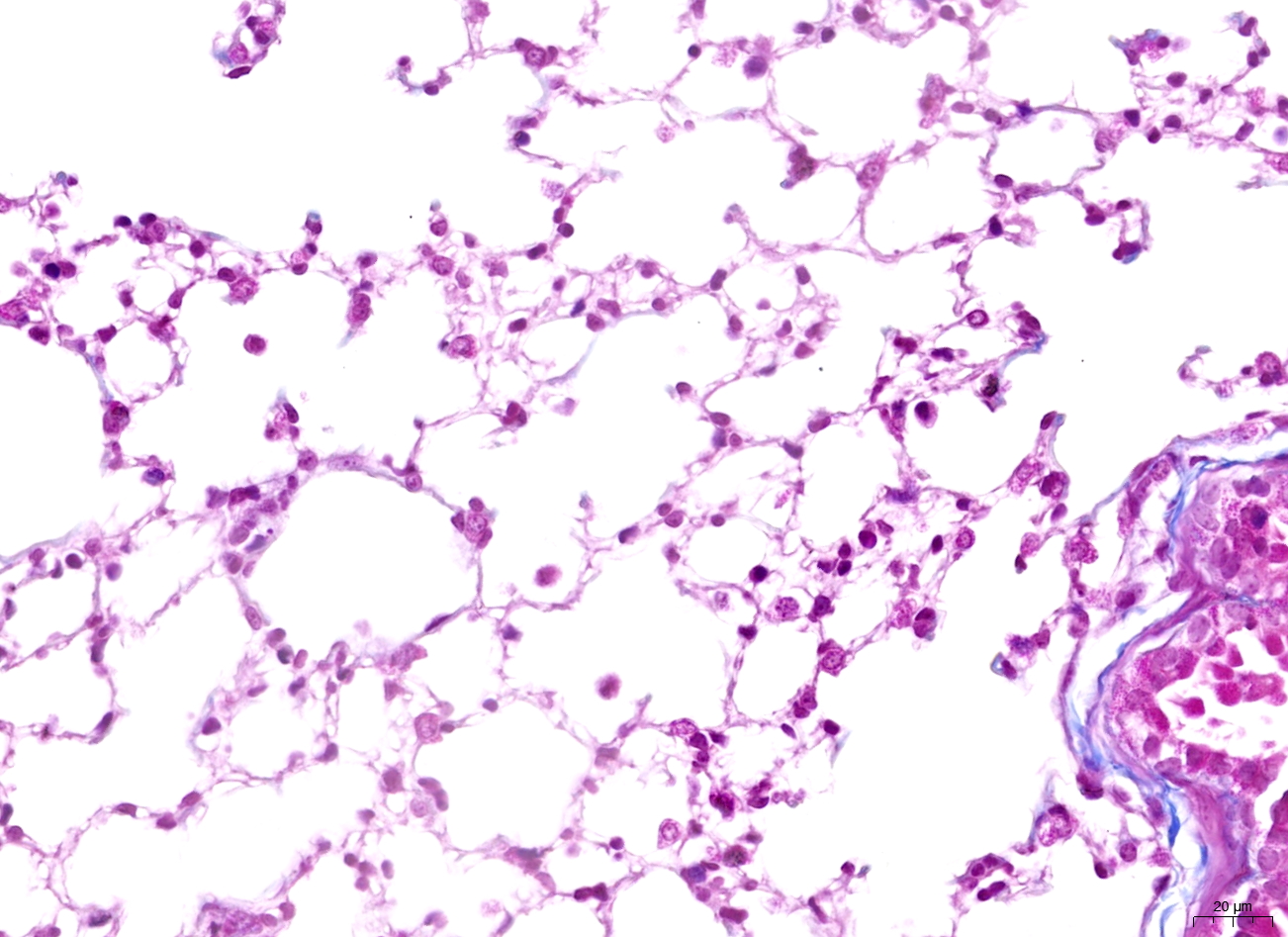

Supplement: Supplementary file 4 — Supporting File 4: advs73867‐sup‐0001‐FiguresData.zip. [file ADVS-13-e19191-s001.zip › Supporting information Figure1-10/Figure 3/Figure 3D/control/Control-105_40.0x-2.jpg]

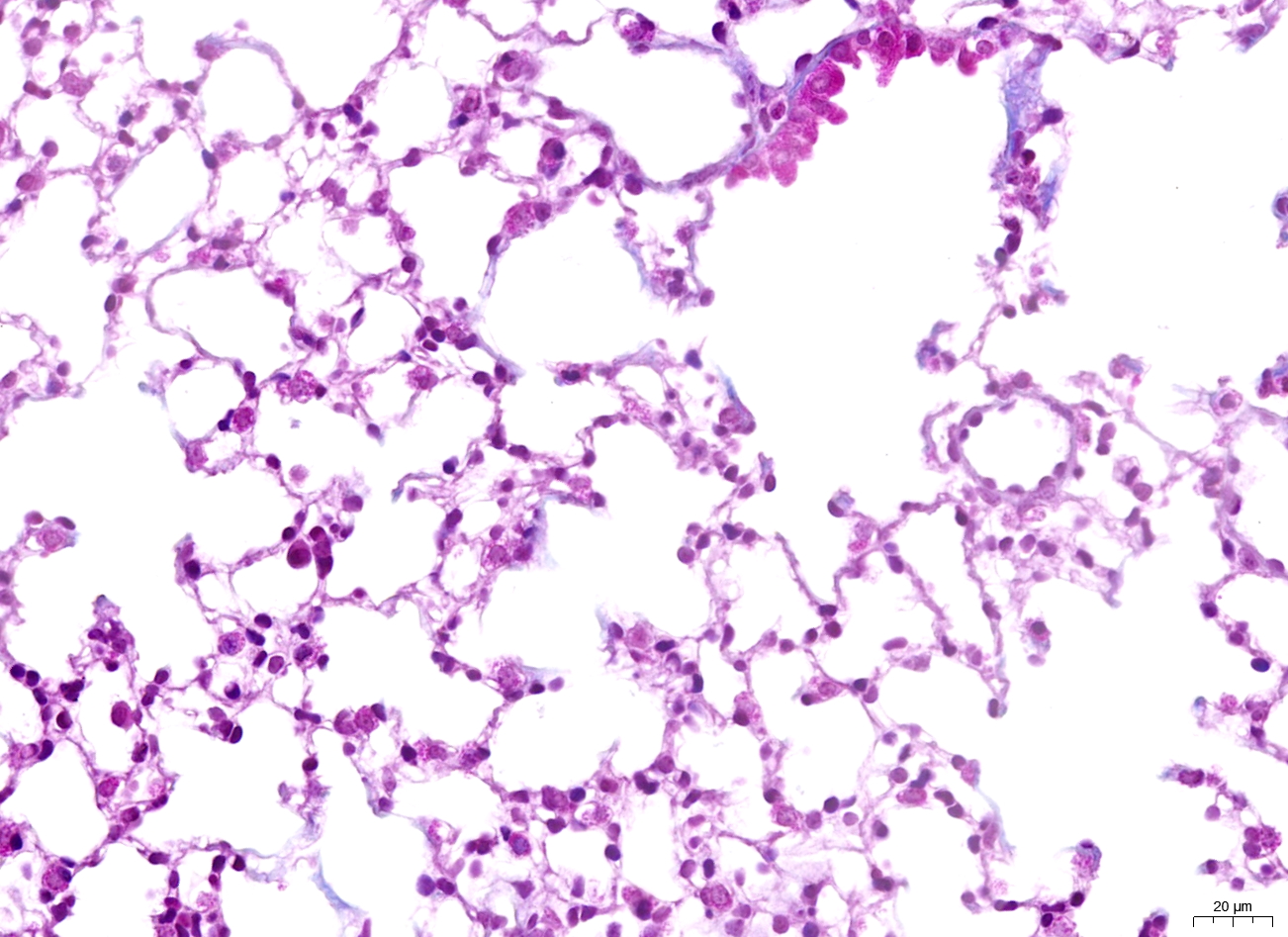

Supplement: Supplementary file 4 — Supporting File 4: advs73867‐sup‐0001‐FiguresData.zip. [file ADVS-13-e19191-s001.zip › Supporting information Figure1-10/Figure 3/Figure 3D/control/Control-105_40.0x-3.jpg]

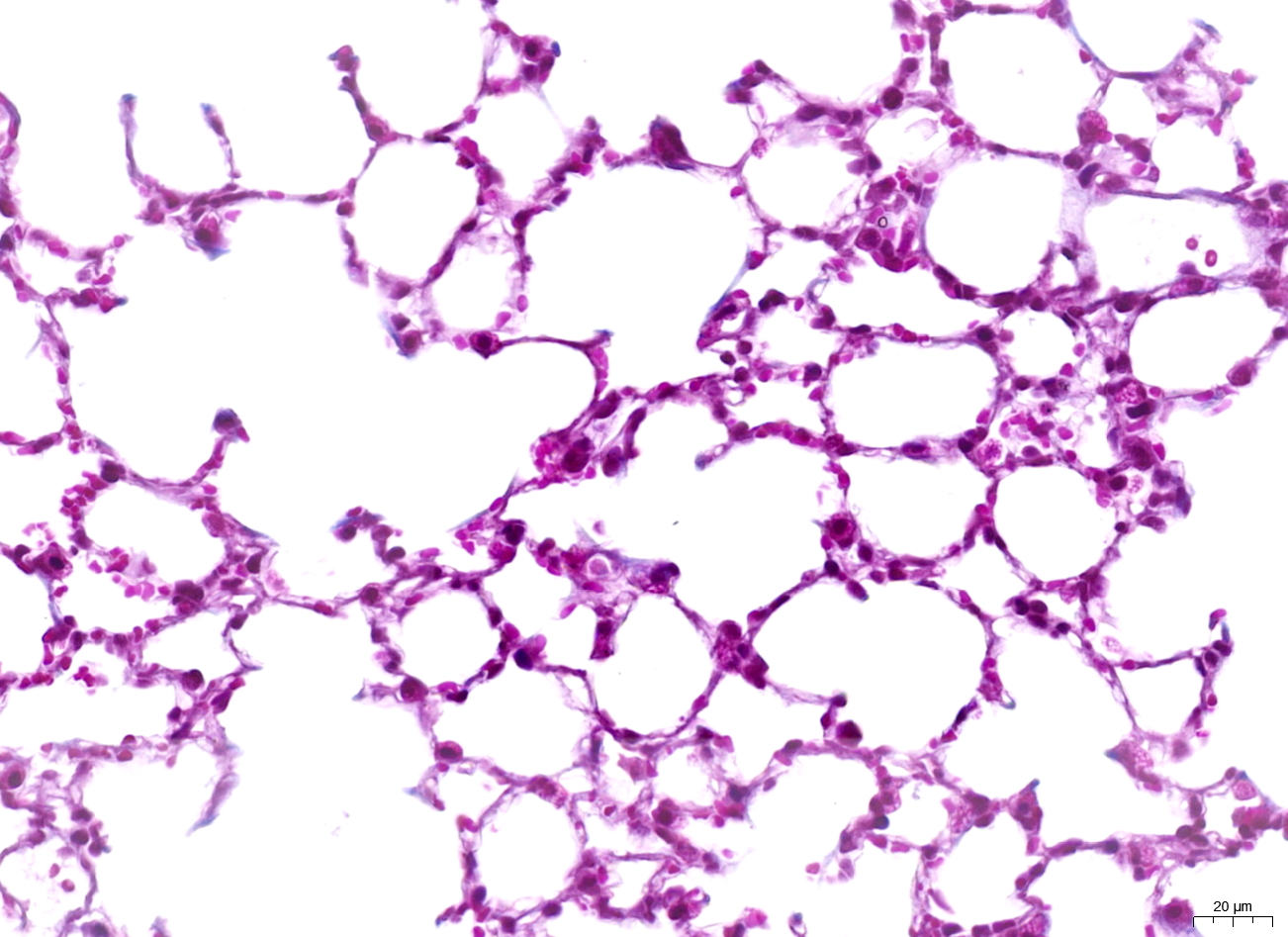

Supplement: Supplementary file 4 — Supporting File 4: advs73867‐sup‐0001‐FiguresData.zip. [file ADVS-13-e19191-s001.zip › Supporting information Figure1-10/Figure 3/Figure 3D/ferritin/Ferritin-109_40.0x.jpg]

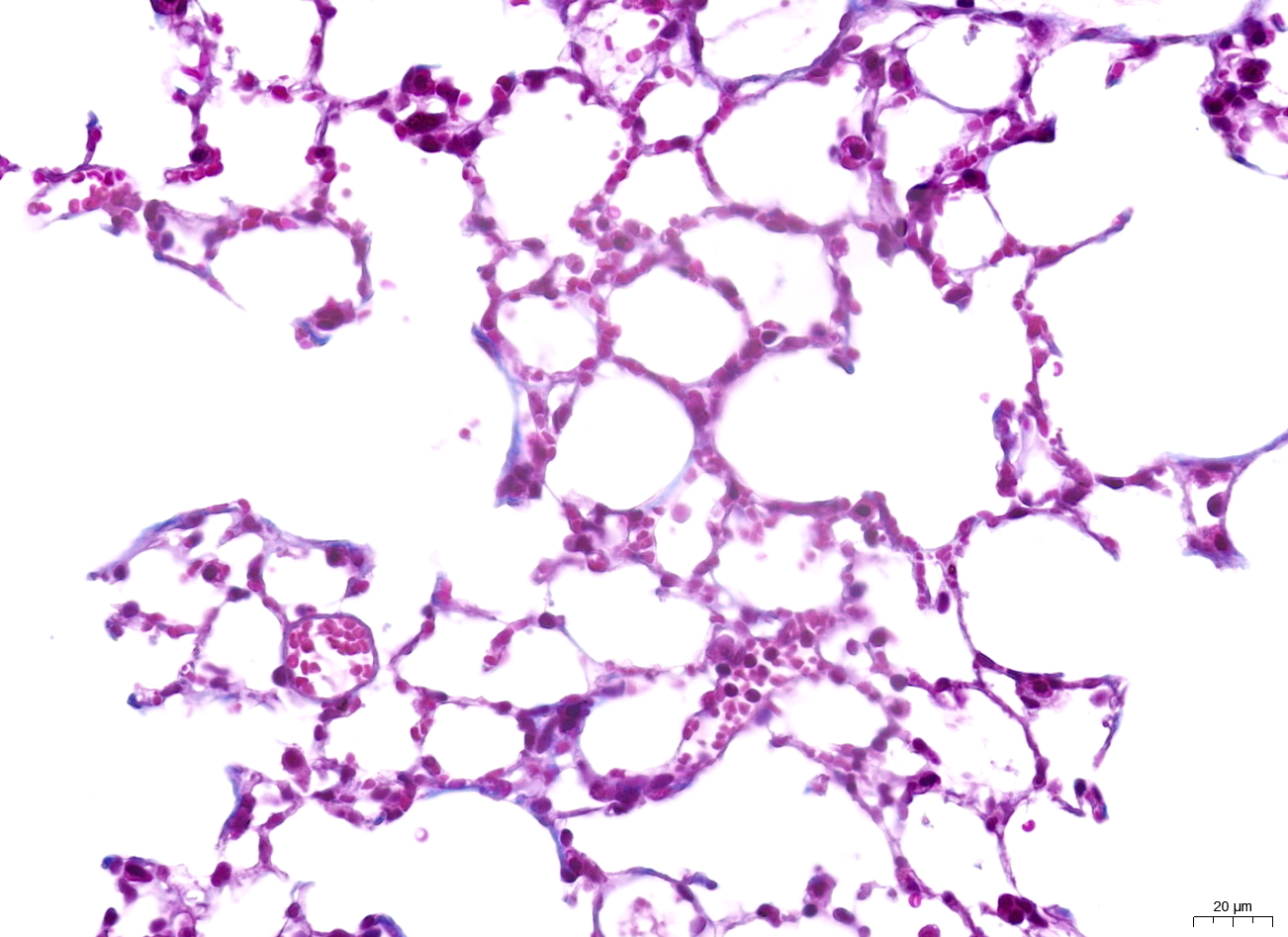

Supplement: Supplementary file 4 — Supporting File 4: advs73867‐sup‐0001‐FiguresData.zip. [file ADVS-13-e19191-s001.zip › Supporting information Figure1-10/Figure 3/Figure 3D/ferritin/Ferritin-109_40.0x-2.jpg]

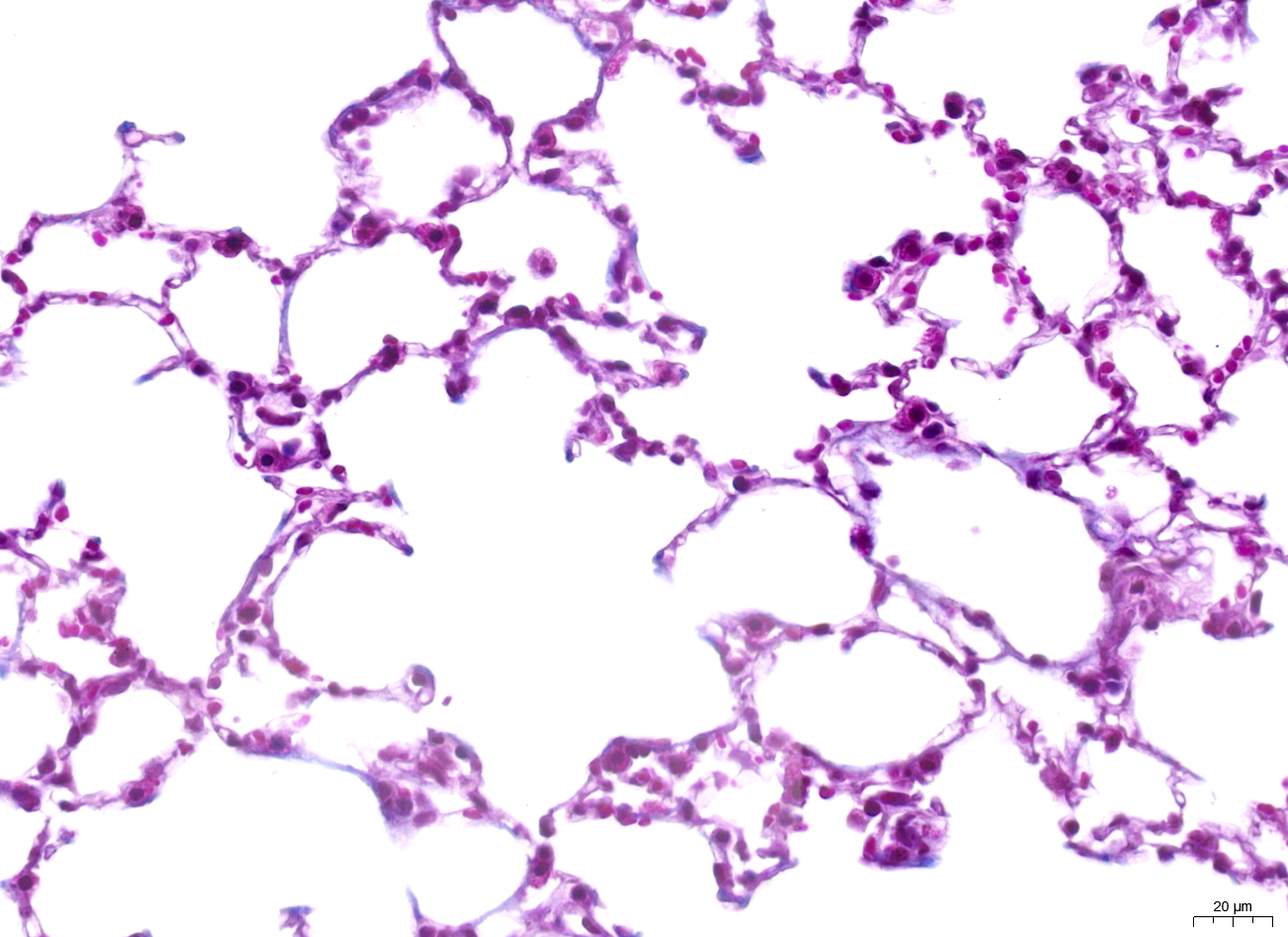

Supplement: Supplementary file 4 — Supporting File 4: advs73867‐sup‐0001‐FiguresData.zip. [file ADVS-13-e19191-s001.zip › Supporting information Figure1-10/Figure 3/Figure 3D/ferritin/Ferritin-109_40.0x-3.jpg]

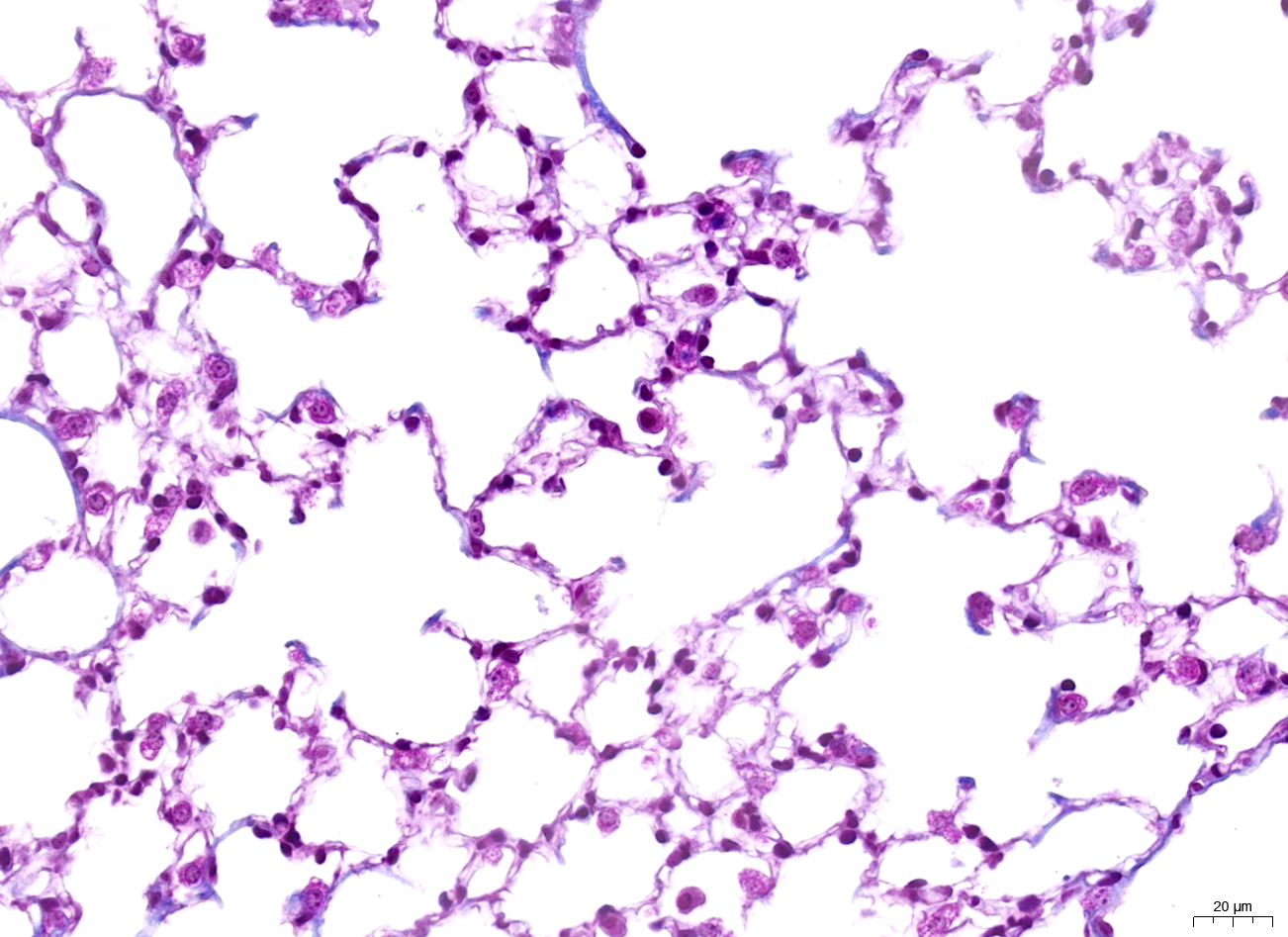

Supplement: Supplementary file 4 — Supporting File 4: advs73867‐sup‐0001‐FiguresData.zip. [file ADVS-13-e19191-s001.zip › Supporting information Figure1-10/Figure 3/Figure 3D/ferritin/Ferritin-122_40.0x.jpg]

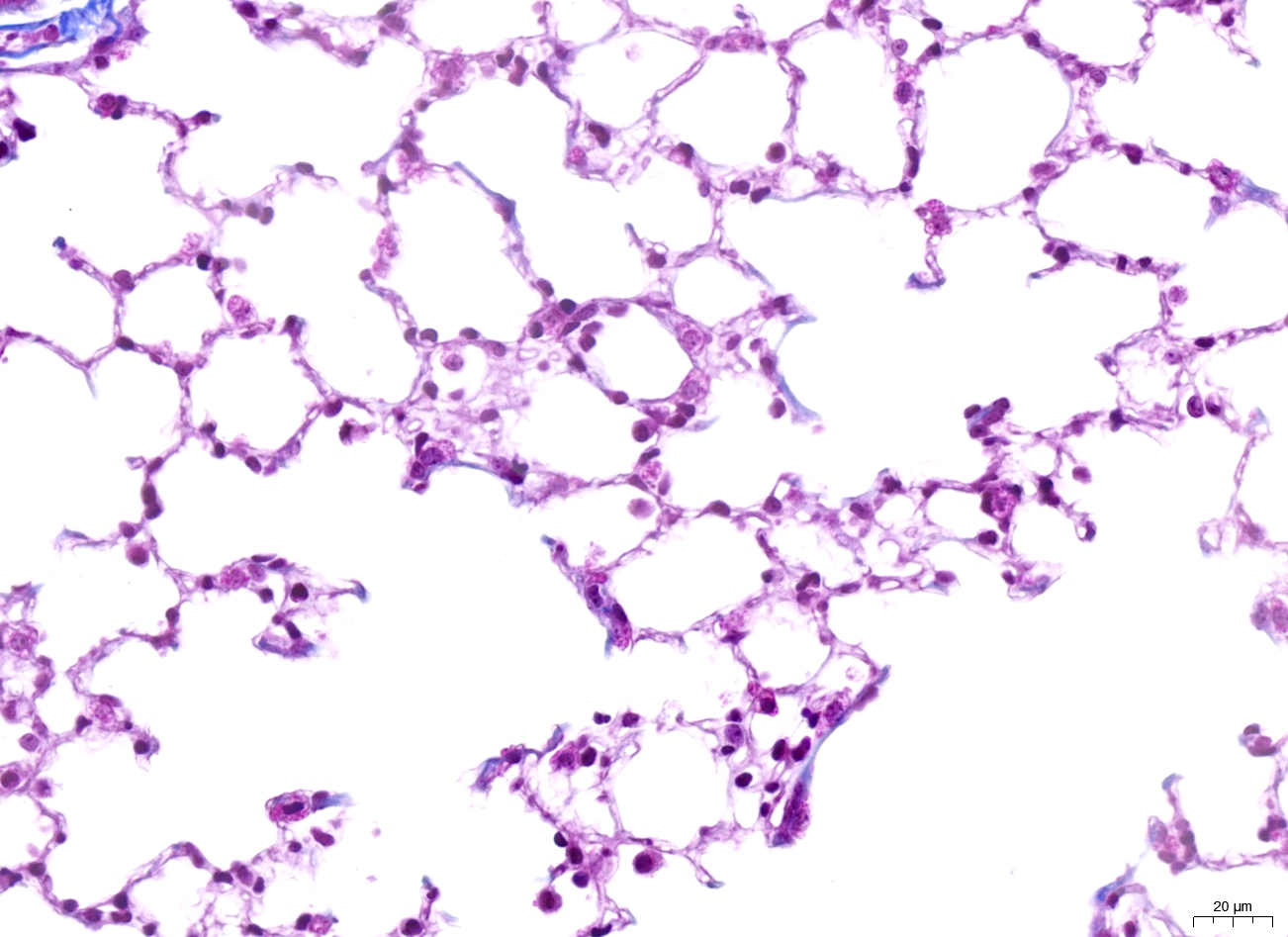

Supplement: Supplementary file 4 — Supporting File 4: advs73867‐sup‐0001‐FiguresData.zip. [file ADVS-13-e19191-s001.zip › Supporting information Figure1-10/Figure 3/Figure 3D/ferritin/Ferritin-122_40.0x-2.jpg]

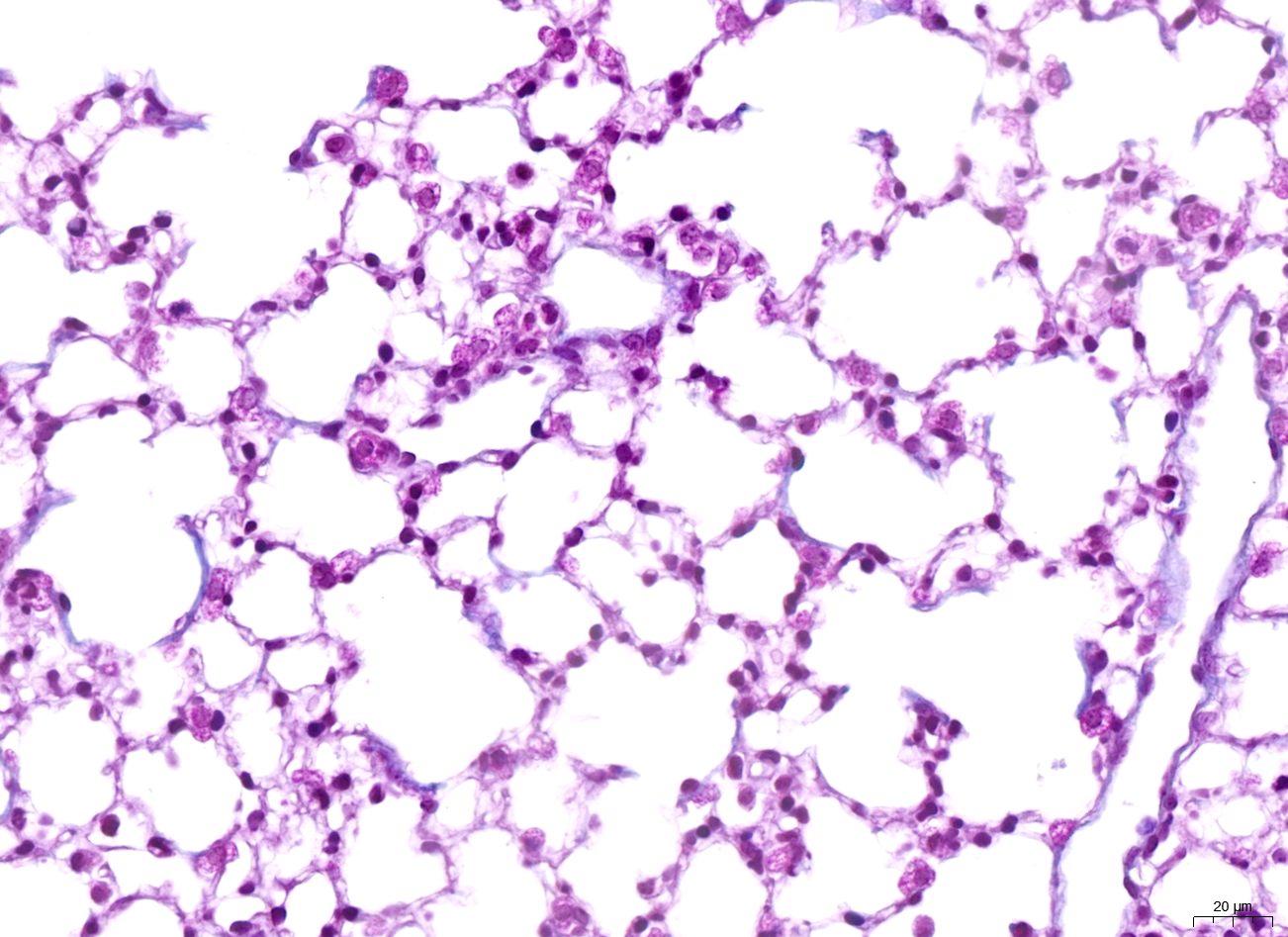

Supplement: Supplementary file 4 — Supporting File 4: advs73867‐sup‐0001‐FiguresData.zip. [file ADVS-13-e19191-s001.zip › Supporting information Figure1-10/Figure 3/Figure 3D/ferritin/Ferritin-122_40.0x-3.jpg]

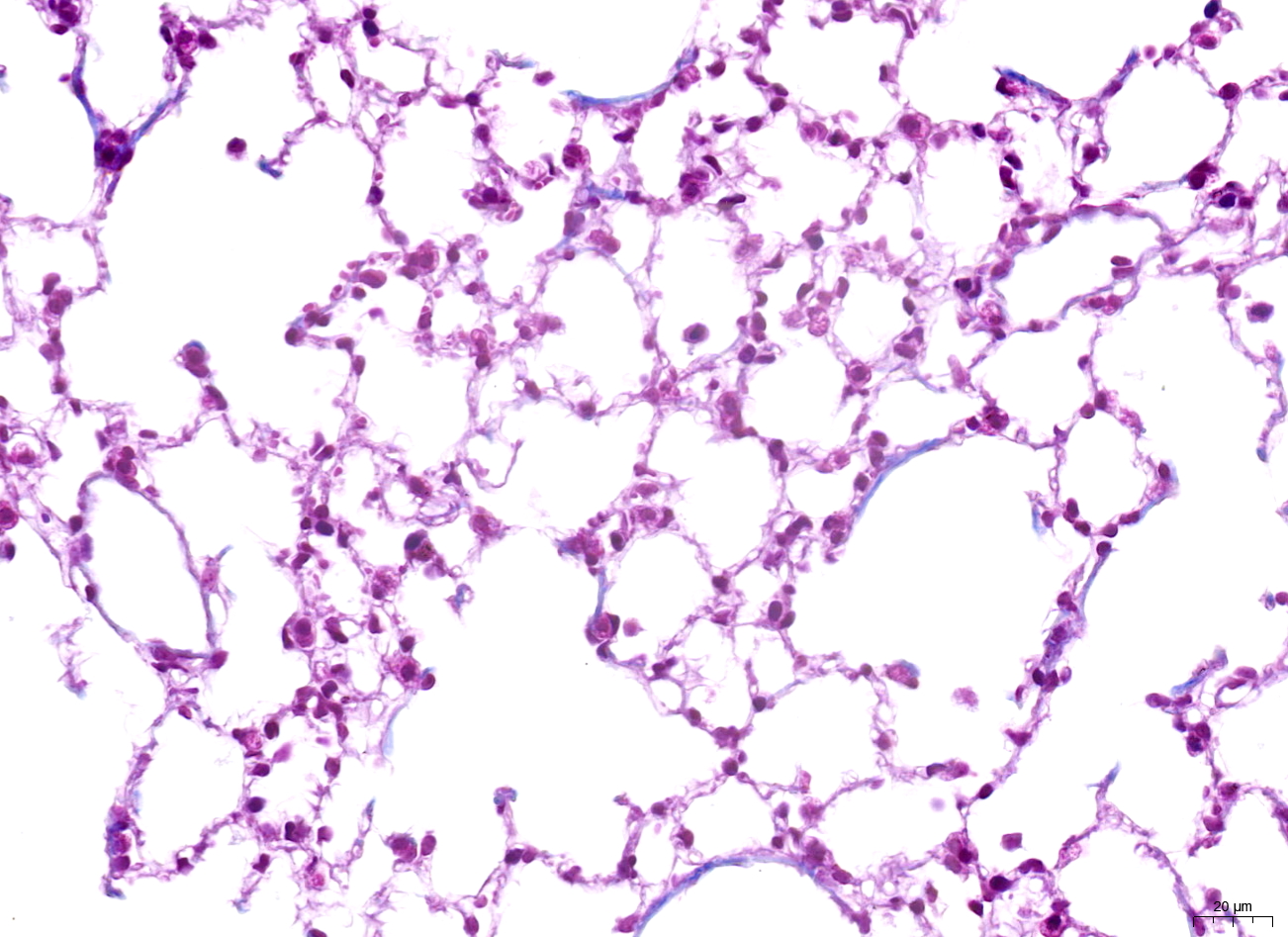

Supplement: Supplementary file 4 — Supporting File 4: advs73867‐sup‐0001‐FiguresData.zip. [file ADVS-13-e19191-s001.zip › Supporting information Figure1-10/Figure 3/Figure 3D/ferritin/Ferritin-125_40.0x.jpg]

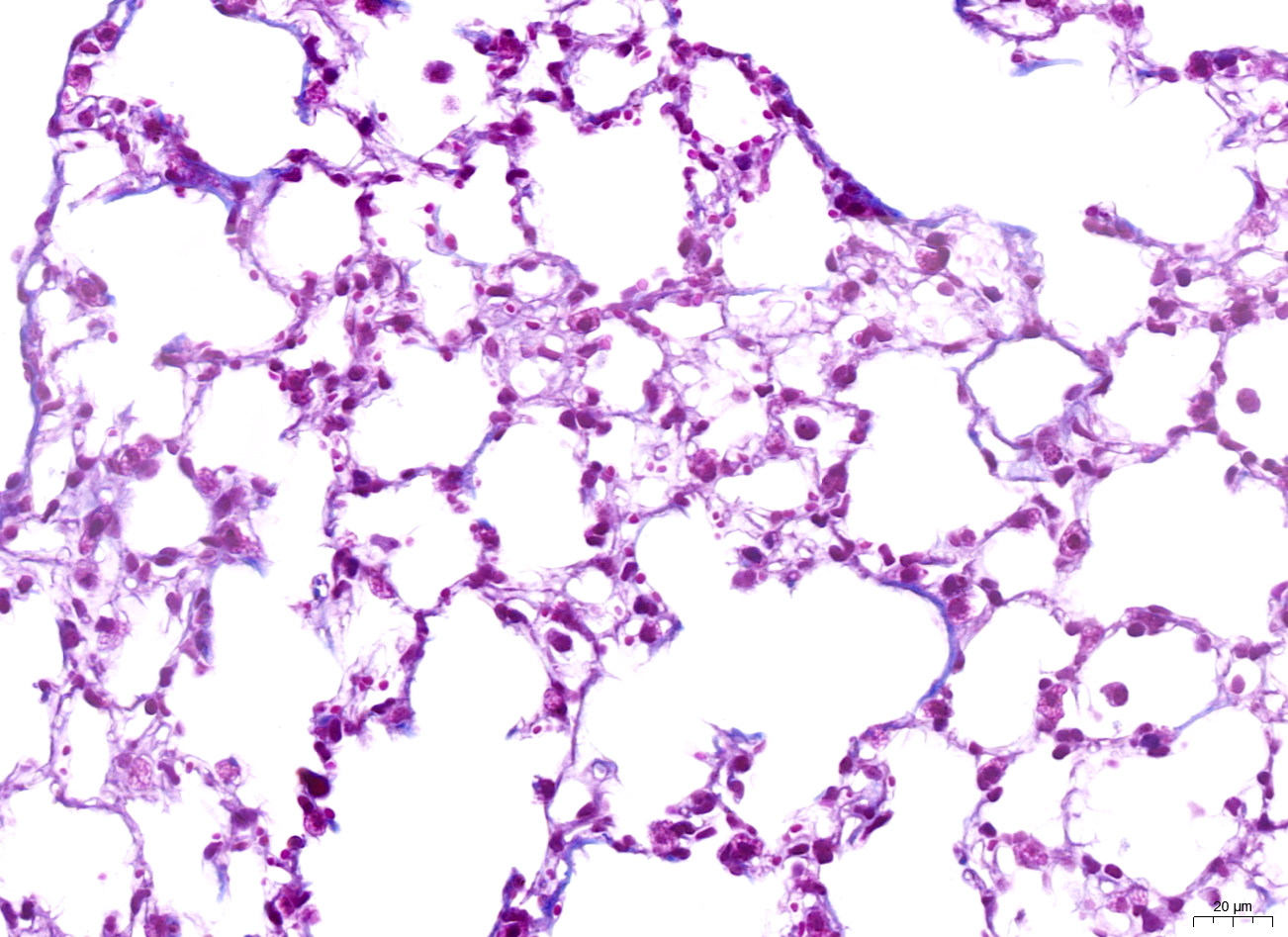

Supplement: Supplementary file 4 — Supporting File 4: advs73867‐sup‐0001‐FiguresData.zip. [file ADVS-13-e19191-s001.zip › Supporting information Figure1-10/Figure 3/Figure 3D/ferritin/Ferritin-125_40.0x-2.jpg]

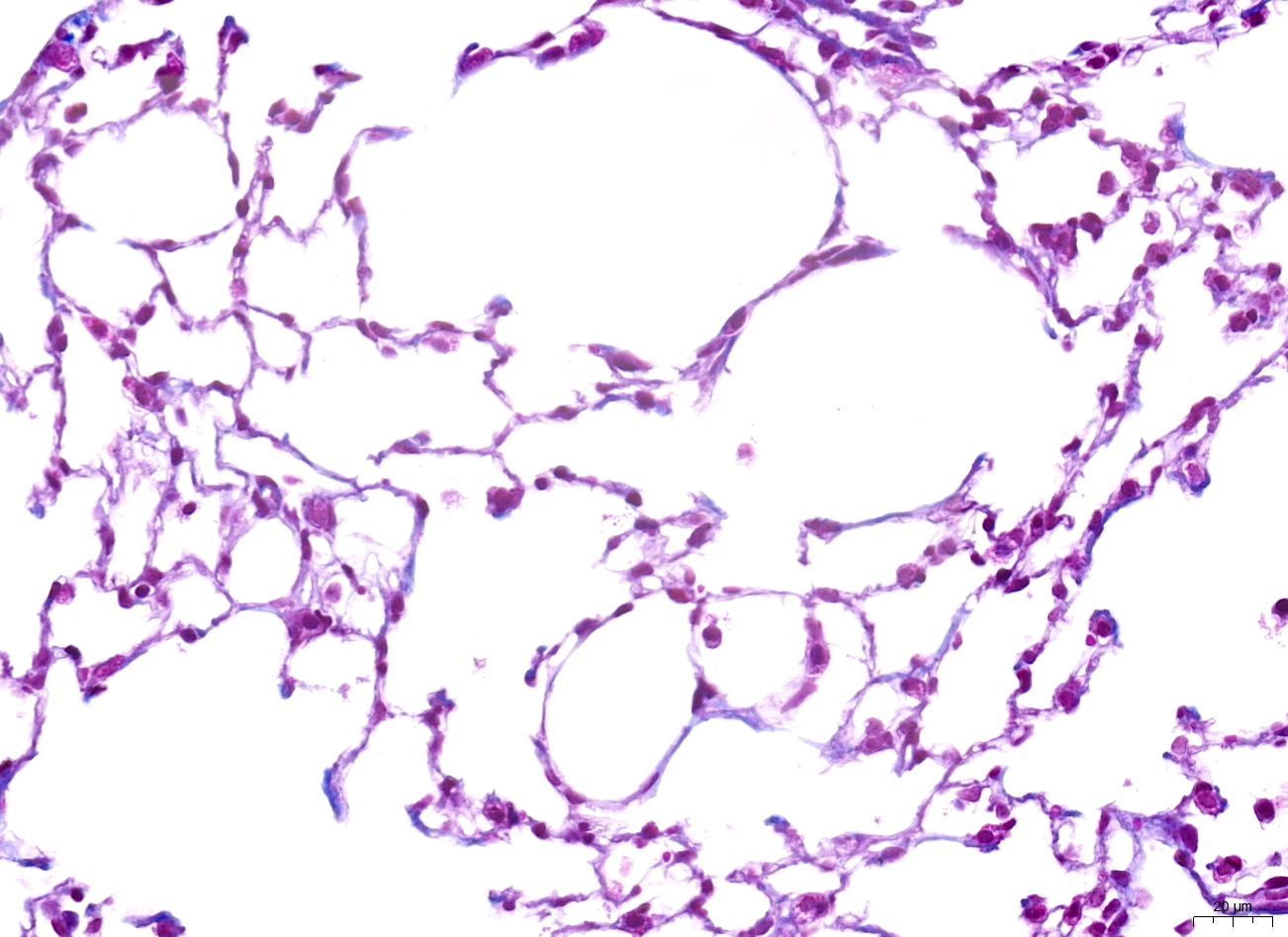

Supplement: Supplementary file 4 — Supporting File 4: advs73867‐sup‐0001‐FiguresData.zip. [file ADVS-13-e19191-s001.zip › Supporting information Figure1-10/Figure 3/Figure 3D/ferritin/Ferritin-125_40.0x-3.jpg]

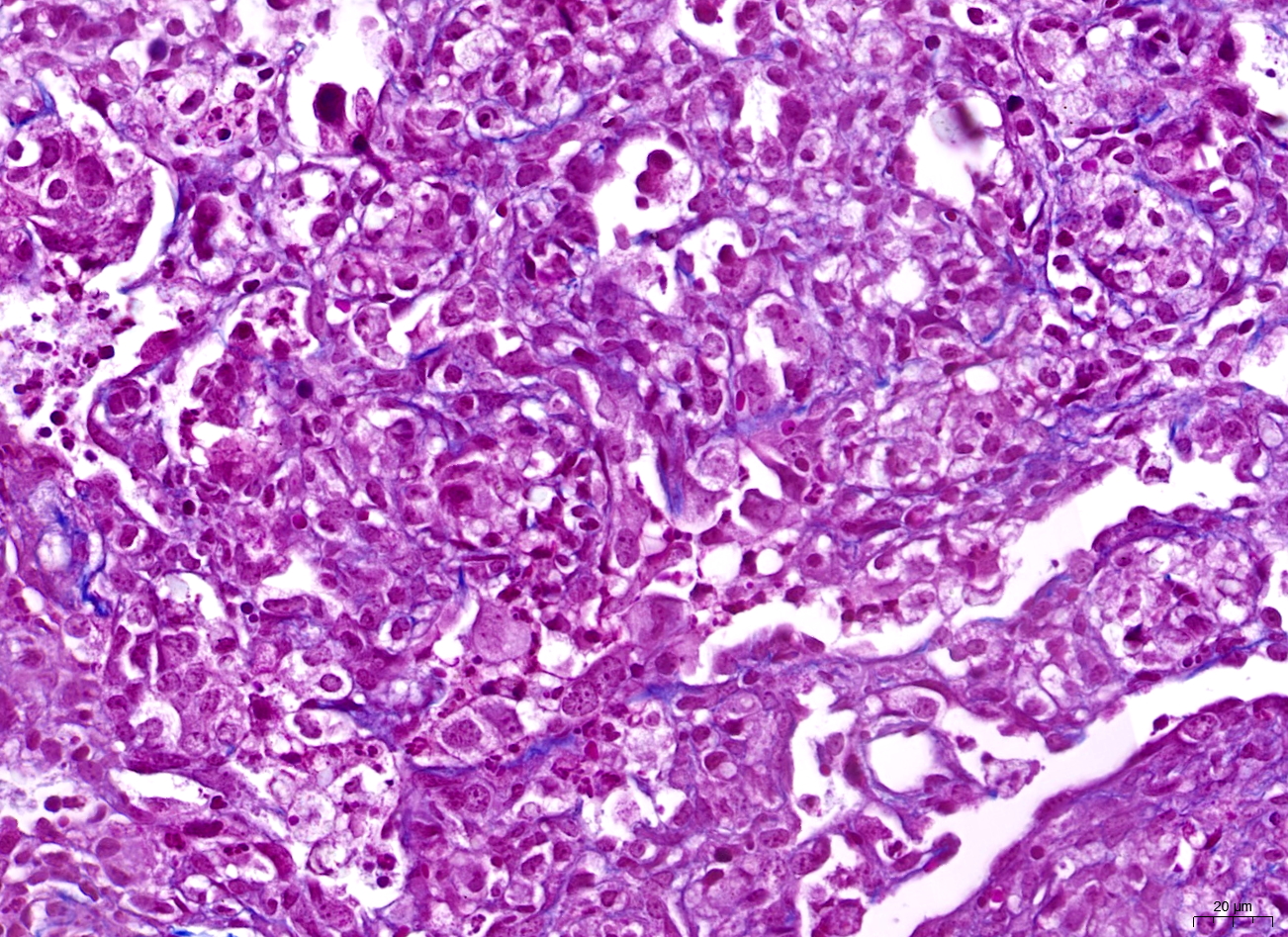

Supplement: Supplementary file 4 — Supporting File 4: advs73867‐sup‐0001‐FiguresData.zip. [file ADVS-13-e19191-s001.zip › Supporting information Figure1-10/Figure 3/Figure 3D/Silica/silica-112_40.0x.jpg]
