# Supplementary material for: Macrophage–Derived Ferritin Exacerbates Silica‐Induced Pulmonary Fibrosis via PIK3R2‐Mediated Fibroblast Differentiation
Source: Adv Sci (Weinh). 2026 Jan 21;13(17):e19191. doi: 10.1002/advs.202519191 (PMC13042690; doi:10.1002/advs.202519191)
Supplement: Supplementary file 3 — Supporting File 3: advs73867‐sup‐0003‐SupportingFiguresData.zip. [file ADVS-13-e19191-s003.zip › Supporting information Figure S1-S9/S8/Figure S8B,C.pdf]

Figure S8B,C

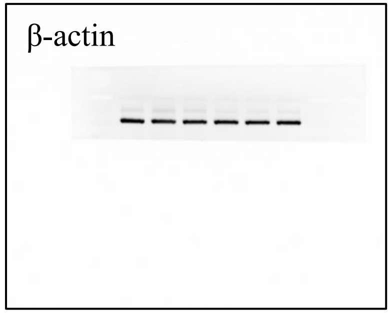

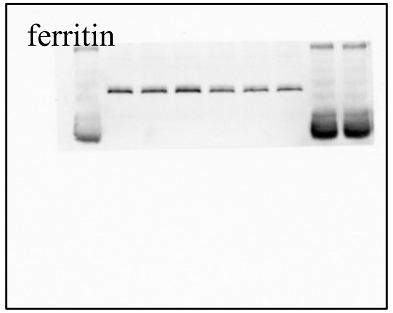

		actin	ferritin	ferritin/actin	Control mean	relative expression
AAV-C	1#	13511902	11165359	0.826335108	0.961019934	0.859852204
	2#	11738138	10863290	0.92546961	0.961019934	0.963007715
	3#	11583201	13103555	1.131255082	0.961019934	1.177140081
AAV-F	1#	12038704	8834650	0.73385391	0.961019934	0.763619863
	2#	11663848	7798528	0.668606793	0.961019934	0.695726248
	3#	12509433	8264341	0.660648728	0.961019934	0.687445395
