# Supplementary material for: Macrophage–Derived Ferritin Exacerbates Silica‐Induced Pulmonary Fibrosis via PIK3R2‐Mediated Fibroblast Differentiation
Source: Adv Sci (Weinh). 2026 Jan 21;13(17):e19191. doi: 10.1002/advs.202519191 (PMC13042690; doi:10.1002/advs.202519191)
Supplement: Supplementary file 3 — Supporting File 3: advs73867‐sup‐0003‐SupportingFiguresData.zip. [file ADVS-13-e19191-s003.zip › Supporting information Figure S1-S9/S9/Figure S9A,B, S9I.pdf]

Figure S9A,B

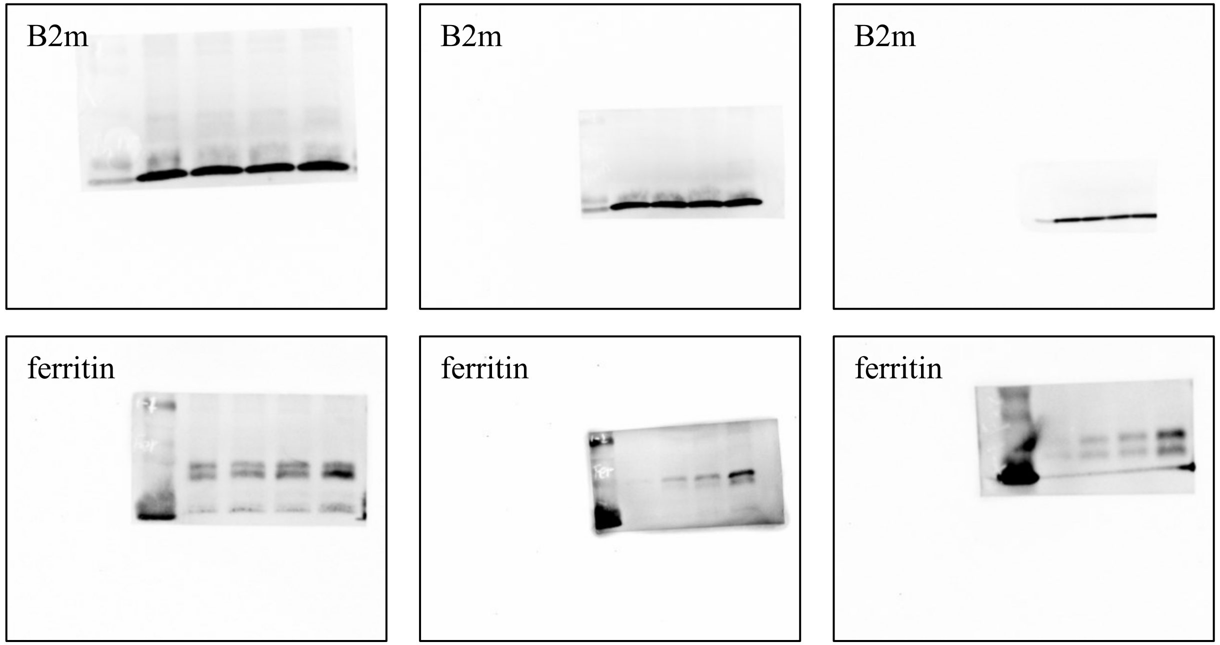

Figure S9I

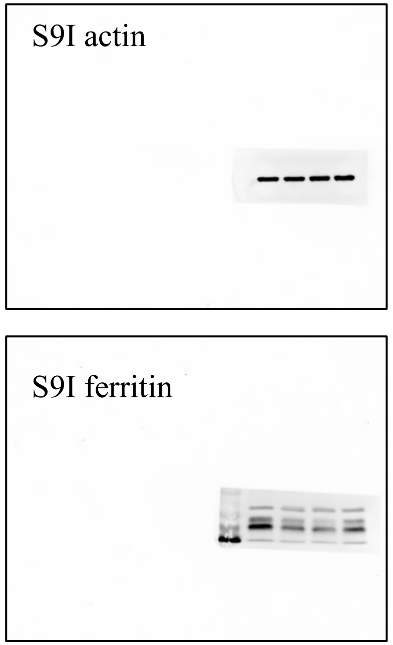

	Silica	B2m	Ferritin			Ferritin/B2m			Control mean	relative expression		
1	0	19469009	4258355	3957330	4389803	0.218725	0.203263	0.225476	0.21582143	1.013453	0.941811	1.044736
	100	18877460	4435337	4744696	5026031	0.234954	0.251342	0.266245	0.21582143	1.088651	1.164583	1.233636
	200	16995400	6432500	6755773	7123676	0.378485	0.397506	0.419153	0.21582143	1.753694	1.841828	1.94213
	400	20969249	9826900	10225166	10532664	0.468634	0.487627	0.502291	0.21582143	2.171396	2.259399	2.327345
	Silica	B2m	Ferritin			Ferritin/B2m			Control mean	relative expression		
2	0	21436873	622161	326898	375578	0.029023	0.015249	0.01752	0.02059748	1.409052	0.740349	0.850598
	100	19216869	834728	722504	886340	0.043437	0.037597	0.046123	0.02059748	2.108862	1.825339	2.239255
	200	18828647	1206164	1256834	1253775	0.06406	0.066751	0.066589	0.02059748	3.110091	3.240743	3.232856
	400	22657743	2286158	2616711	2459089	0.1009	0.115489	0.108532	0.02059748	4.898639	5.606927	5.269185
	Silica	B2m	Ferritin			Ferritin/B2m			Control mean	relative expression		
3	0	3985611	3840015	3513027	4042856	0.96347	0.881427	1.014363	0.95308666	1.010894	0.924814	1.064292
	100	3382123	5485977	5723054	6090816	1.622051	1.692148	1.800885	0.95308666	1.701893	1.77544	1.88953
	200	3116026	5674462	6049547	6471852	1.821057	1.94143	2.076957	0.95308666	1.910694	2.036992	2.17919
	400	3775912	12334023	12697043	13497354	3.266502	3.362643	3.574594	0.95308666	3.427287	3.528161	3.750545
