# Supplementary material for: Macrophage–Derived Ferritin Exacerbates Silica‐Induced Pulmonary Fibrosis via PIK3R2‐Mediated Fibroblast Differentiation
Source: Adv Sci (Weinh). 2026 Jan 21;13(17):e19191. doi: 10.1002/advs.202519191 (PMC13042690; doi:10.1002/advs.202519191)
Supplement: Supplementary file 4 — Supporting File 4: advs73867‐sup‐0001‐FiguresData.zip. [file ADVS-13-e19191-s001.zip › Supporting information Figure1-10/Figure 8/Figure 8G-J.pdf]

Figure 8G-J

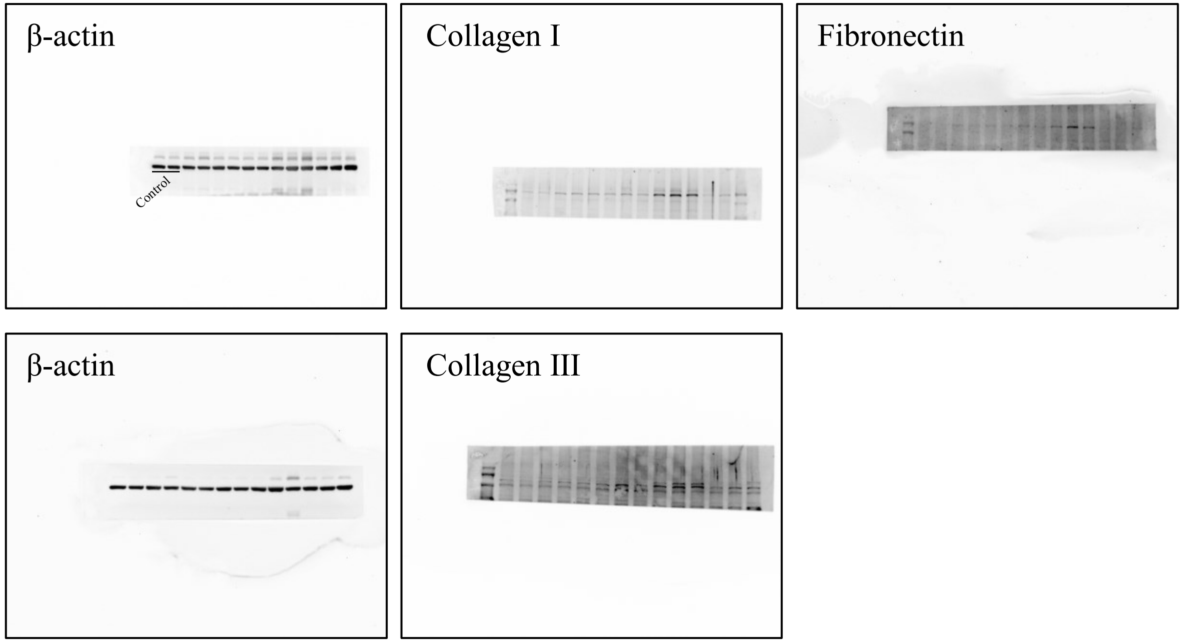

Control: A normal control that did not receive AAV virus injection

	actin	Collagen I	Collagen I/β-actin	Control mean	relative expression
Control	3810086	869335			
Control	3119130	875471			
AAV-C	3256464	1000971	0.307379722	0.314890193	0.976148922
AAV-C	3225958	1040051	0.322400664	0.314890193	1.023851078
AAV-C	2919945	1050359	0.359718762	0.314890193	1.142362544
AAV-F	3125002	1003212	0.321027635	0.314890193	1.019490736
AAV-F	3320177	1146279	0.345246353	0.314890193	1.096402367
AAV-F	3183524	1082882	0.340151983	0.314890193	1.080224123
AAV-C+Silica	2819315	1769946	0.627792921	0.314890193	1.993688388
AAV-C+Silica	2959239	2139457	0.7229754	0.314890193	2.295960358
AAV-C+Silica	3030930	1902951	0.627843929	0.314890193	1.993850374
AAV-F+Silica	3544481	1154149	0.325618617	0.314890193	1.034070365
AAV-F+Silica	4404778	1094061	0.248380509	0.314890193	0.788784517
AAV-F+Silica	5719939	1275191	0.222937867	0.314890193	0.70798606

	actin	Fibronectin	Fibronectin/β-actin	Control mean	relative expression
Control	3810086	1274952			
Control	3119130	1371457			
AAV-C	3256464	1562059	0.479679493	0.476628167	1.0064019
AAV-C	3225958	1527739	0.473576841	0.476628167	0.9935981
AAV-C	2919945	1628038	0.557557762	0.476628167	1.169796082
AAV-F	3125002	1686872	0.539798695	0.476628167	1.132536287
AAV-F	3320177	1755627	0.528775123	0.476628167	1.109408044
AAV-F	3183524	1836171	0.576773098	0.476628167	1.210111231
AAV-C+Silica	2819315	2023369	0.717681068	0.476628167	1.505746235
AAV-C+Silica	2959239	2234835	0.755205984	0.476628167	1.584476193
AAV-C+Silica	3030930	2151604	0.709882445	0.476628167	1.489384166
AAV-F+Silica	3544481	1766677	0.498430377	0.476628167	1.045742596
AAV-F+Silica	4404778	1755813	0.398615549	0.476628167	0.836323945
AAV-F+Silica	5719939	1502988	0.262762942	0.476628167	0.551295455

	actin	Collagen III	Collagen III/β-actin	Control mean	relative expression
Control	4723846	7031660			
Control	3454059	6631616			
AAV-C	3680148	6242479	1.696257596	1.74030147	0.974691814
AAV-C	3422662	6107211	1.784345343	1.74030147	1.025308186
AAV-C	3065995	5431064	1.771387103	1.74030147	1.017862212
AAV-F	3081179	5710129	1.853228586	1.74030147	1.064889399
AAV-F	4199496	5190517	1.235985699	1.74030147	0.710213558
AAV-F	3540398	5959273	1.683221208	1.74030147	0.967200935
AAV-C+Silica	4146521	9016726	2.174527996	1.74030147	1.249512245
AAV-C+Silica	4623512	9645462	2.086176482	1.74030147	1.198744309
AAV-C+Silica	4148889	9562214	2.304764962	1.74030147	1.32434811
AAV-F+Silica	4933139	7522456	1.52488223	1.74030147	0.876217286
AAV-F+Silica	5437344	6258359	1.150995596	1.74030147	0.661377133
AAV-F+Silica	4933139	7522456	1.52488223	1.74030147	0.876217286
