# Supplementary material for: Macrophage–Derived Ferritin Exacerbates Silica‐Induced Pulmonary Fibrosis via PIK3R2‐Mediated Fibroblast Differentiation
Source: Adv Sci (Weinh). 2026 Jan 21;13(17):e19191. doi: 10.1002/advs.202519191 (PMC13042690; doi:10.1002/advs.202519191)
Supplement: Supplementary file 4 — Supporting File 4: advs73867‐sup‐0001‐FiguresData.zip. [file ADVS-13-e19191-s001.zip › Supporting information Figure1-10/Figure 9/Figure 9.pdf]

Figure 9

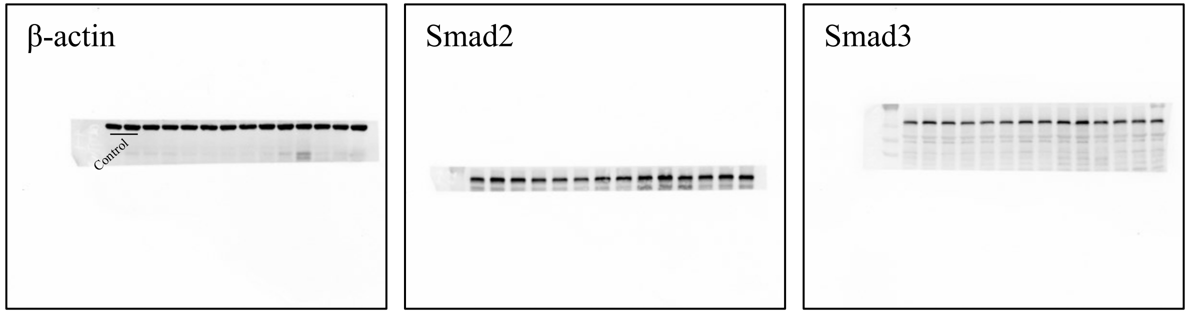

Control: A normal control that did not receive AAV virus injection

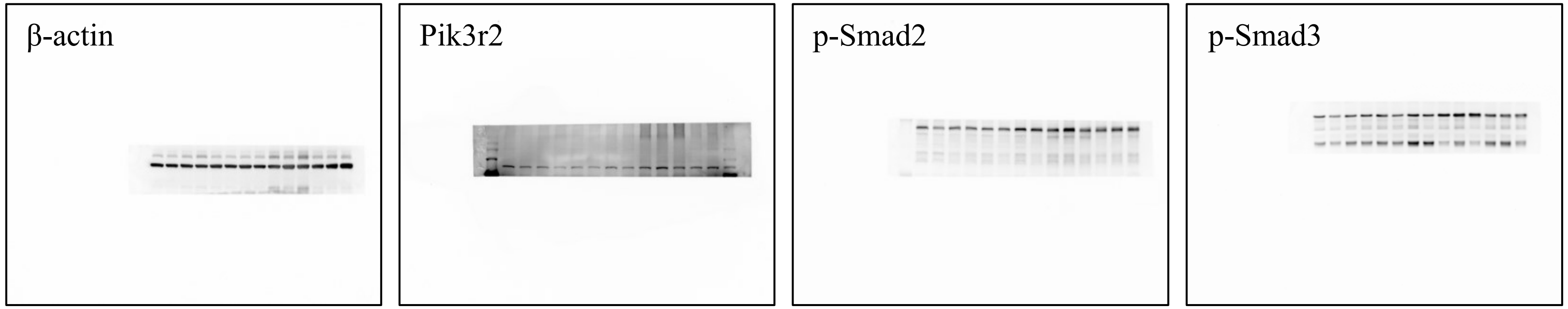

	actin	Smad2	Smad2/β-actin	Control mean	relative expression
Control	7492383	3616134			
Control	8419295	4947069			
AAV-C	6934881	4337226	0.625421835	0.608227299	1.028269918
AAV-C	5850119	3457612	0.591032764	0.608227299	0.971730082
AAV-C	6291326	3046447	0.484229716	0.608227299	0.796132821
AAV-F	6160085	2710976	0.440087434	0.608227299	0.723557516
AAV-F	5960285	3557073	0.596795791	0.608227299	0.981205203
AAV-F	5687324	3756264	0.66046246	0.608227299	1.085880987
AV-C+Silic	5961724	4728723	0.793180463	0.608227299	1.3040856
AV-C+Silic	6941707	5662767	0.815760014	0.608227299	1.341209142
AV-C+Silic	5897647	4363553	0.739880329	0.608227299	1.216453667
AV-F+Silic	6227413	3342647	0.53676334	0.608227299	0.882504518
AV-F+Silic	5806595	4123445	0.710131325	0.608227299	1.16754267
AV-F+Silic	7959071	4814871	0.604953895	0.608227299	0.994618123

	actin	Smad3	Smad3/β-actin	Control mean	relative expression
Control	7492383	2693810			
Control	8419295	3030807			
AAV-C	6934881	2797226	0.40335602	0.398441443	1.012334502
AAV-C	5850119	2302179	0.393526867	0.398441443	0.987665498
AAV-C	6291326	2203949	0.350315498	0.398441443	0.879214509
AAV-F	6160085	2442387	0.396485925	0.398441443	0.995092081
AAV-F	5960285	2911714	0.488519257	0.398441443	1.226075412
AAV-F	5687324	2450973	0.430953644	0.398441443	1.08159844
AV-C+Silic	5961724	3287715	0.551470514	0.398441443	1.384069161
AV-C+Silic	6941707	4104420	0.591269554	0.398441443	1.483955958
AV-C+Silic	5897647	2942512	0.498929827	0.398441443	1.252203644
AV-F+Silic	6227413	2855759	0.458578707	0.398441443	1.150931246
AV-F+Silic	5806595	2499950	0.430536313	0.398441443	1.080551031
AV-F+Silic	7959071	2770388	0.348079317	0.398441443	0.873602189

	actin	Pik3r2	Pik3r2/β-actin	Control mean	relative expression
Control	3810086	1595631			
Control	3119130	1447950			
AAV-C	3256464	1679944	0.515879801	0.390758121	1.32020238
AAV-C	3225958	856932	0.26563644	0.390758121	0.67979762
AAV-C	2919945	1343291	0.46003983	0.390758121	1.177300753
AAV-F	3125002	1112160	0.355890972	0.390758121	0.910770508
AAV-F	3320177	1917563	0.577548426	0.390758121	1.478020277
AAV-F	3183524	1473234	0.462768303	0.390758121	1.184283266
AV-C+Silic	2819315	2373731	0.841953099	0.390758121	2.154665646
AV-C+Silic	2959239	3116378	1.053101152	0.390758121	2.695020516
AV-C+Silic	3030930	2172989	0.716938036	0.390758121	1.834736113
AV-F+Silic	3544481	1546347	0.436268949	0.390758121	1.116468029
AV-F+Silic	4404778	1631600	0.370415944	0.390758121	0.947941768
AV-F+Silic	5719939	1083765	0.189471426	0.390758121	0.484881609

	actin	p-Smad2	p-Smad2/β-actin	Control mean	relative expression
Control	3810086	6541131			
Control	3119130	4848198			
AAV-C	3256464	6350854	1.950230065	1.957758483	0.996154573
AAV-C	3225958	6339933	1.965286901	1.957758483	1.003845427
AAV-C	2919945	6268648	2.146837697	1.957758483	1.096579438
AAV-F	3125002	6583342	2.106668092	1.957758483	1.076061276
AAV-F	3320177	8852256	2.666200025	1.957758483	1.361863605
AAV-F	3183524	8964285	2.815837104	1.957758483	1.438296464
AV-C+Silic	2819315	9701685	3.441149712	1.957758483	1.757698787
AV-C+Silic	2959239	12318851	4.162844231	1.957758483	2.126331857
AV-C+Silic	3030930	9338874	3.081190922	1.957758483	1.573836072
AV-F+Silic	3544481	8993450	2.537310822	1.957758483	1.296028516
AV-F+Silic	4404778	9507986	2.158561907	1.957758483	1.102568026
AV-F+Silic	5719939	9536278	1.667199248	1.957758483	0.851585761

	actin	p-Smad3	p-Smad3/β-actin	Control mean	relative expression
Control	3810086	3501052			
Control	3119130	2515024			
AAV-C	3256464	3822575	1.173842241	1.249515457	0.939437951
AAV-C	3225958	4275003	1.325188673	1.249515457	1.060562049
AAV-C	2919945	4626853	1.584568545	1.249515457	1.268146413
AAV-F	3125002	3159875	1.011159353	1.249515457	0.809241172
AAV-F	3320177	5026392	1.513892783	1.249515457	1.211583878
AAV-F	3183524	4258802	1.337763434	1.249515457	1.070625759
AV-C+Silic	2819315	6052792	2.146901641	1.249515457	1.718187341
AV-C+Silic	2959239	6770770	2.288010532	1.249515457	1.831118231
AV-C+Silic	3030930	7305500	2.410316306	1.249515457	1.929000792
AV-F+Silic	3544481	4691503	1.323607885	1.249515457	1.059296928
AV-F+Silic	4404778	4526418	1.027615467	1.249515457	0.822411168
AV-F+Silic	5719939	5193124	0.907898493	1.249515457	0.72660045
